# Supplementary material for: What Is the Relationship between Outdoor Time and Physical Activity, Sedentary Behaviour, and Physical Fitness in Children? A Systematic Review
Source: Int J Environ Res Public Health. 2015 Jun 8;12(6):6455–74. doi: 10.3390/ijerph120606455 (PMC4483711; doi:10.3390/ijerph120606455)
Supplement: Supplementary File 1 [file ijerph-12-06455-s001.pdf]

N2 - Aim: To explore how children's self-consciousness would change after outward bound training in order to provide a theoretical reference for effectively optimized children's self-consciousness. Methods: In June 2002, 78 children aged 8-16 years from the primary or middle schools in Xi'an city were randomized into experimental group (outward bound training group) and control group (without training group) for randomized control observation. Before outward-bound training, children in the control group were measured with the Piers-Harris children's self-concept (PHCSS). After training, children in the experimental group were measured, the experiment content was part of field programs (hiking, camping, fieldwork, outdoors survival) and ground programs (stop bridge, top tightwire, buttocking, one wood bridge, game of across river, debutton hand in hand, blind man phalanx, electrical net, etc.), and it cost 48 hours. Results: (1) Comparison of the total score between the control group and experimental group: The average total score of PHCSS in the experimental group was 61.05, increased by 2.70 as compared with that in the control group (58.70). The lowest and highest total scores in the experimental group were 11 and 1 point higher than those in the control group respectively. The total score of self-consciousness level was above 58 points in 60% and 71% of the children in the control group and experimental group respectively. (2) Comparison of the scores of the corresponding dimensions of self-consciousness between the experimental group and control group: The least scores of all six dimensions in the experimental group were increased as compared with those in the control group, which were more obvious in

the dimensions of behavior, gregariousness and anxiety (13.39+1.84, 12.10+2.92; 10.08+1.48, 9.23+2.03; 11.37+2.07, 9.75+2.49). Conclusion: Outward bound training plays an improvement role in enhancing children's self-consciousness, especially the children with low self-consciousness. Outward bound training affects the six dimensions of children's self-consciousness more or less, which is especially significantly embodied the dimensions of anxiety, gregariousness and behavior
SN - 1671-5926
AD - (Fang) Postgraduate School, Beijing Sport University, Beijing 100084, China (Luan) School of Athletic Physical Education, Beijing Sport University, Beijing 100084, China
ER -

TY - JOUR
ID - 3837
T1 - Recreational injuries in Washington State National Parks
A1 - Stephens,B.D.
A1 - Diekema,D.S.
A1 - Klein,E.J.
Y1 - 2005///
N1 - 2005575235
English
Journal: Review
KW - EMBASE
KW - Adolescent
KW - Adult
KW - age
KW - allergic reaction/ep [Epidemiology]
KW - allergic reaction/et [Etiology]
KW - altitude disease/ep [Epidemiology]
KW - altitude disease/et [Etiology]
KW - Child
KW - cold injury/ep [Epidemiology]
KW - cold injury/et [Etiology]
KW - controlled study
KW - dehydration/ep [Epidemiology]
KW - dehydration/et [Etiology]
KW - dislocation/ep [Epidemiology]
KW - dislocation/et [Etiology]
KW - Drowning/ep [Epidemiology]
KW - falling
KW - Female
KW - First Aid
KW - gender
KW - head injury/ep [Epidemiology]
KW - head injury/et [Etiology]
KW - Health Education
KW - heart infarction/ep [Epidemiology]
KW - hiking
KW - Human
KW - hypothermia/ep [Epidemiology]
KW - hypothermia/et [Etiology]
KW - injury/ep [Epidemiology]
KW - injury/et [Etiology]
KW - injury/pc [Prevention]
KW - insect sting/ep [Epidemiology]
KW - insect sting/et [Etiology]
KW - laceration/ep [Epidemiology]
KW - laceration/et [Etiology]
KW - major clinical study

KW - Male
KW - Mountaineering
KW - national park
KW - Recreation
KW - retrospective study
KW - review
KW - soft tissue injury/ep [Epidemiology]
KW - soft tissue injury/et [Etiology]
KW - sprain/ep [Epidemiology]
KW - sprain/et [Etiology]
KW - Suicide
KW - summer
KW - Swimming
KW - United States
RP - NOT IN FILE
SP - 192
EP - 197
JF - Wilderness and Environmental Medicine
VL - 16
IS - 4
CY - United States
N2 - Objectives. - The objectives of this study were to identify the number and types of recreational injuries sustained by visitors to Mount Rainier National Park and Olympic National Park in Washington State and to compare the nature of injuries sustained by children compared with adults. Methods. - We retrospectively reviewed case incident reports obtained by rangers in Mount Rainer National Park and Olympic National Park between 1997 and 2001. Data collected included victim age, gender, date of injury, activity preinjury, type of injury, and mechanism of injury. Results. - There were 535 cases of recreational wilderness injuries (including 19 total deaths), yielding a rate of 22.4 injuries per million visits. The mean age of injury victims was 34 years. Males were more likely to sustain injury than were females (59% vs 41%). Most injuries occurred during summer months between noon and 6:00 PM, and 90% occurred during daylight hours. The most common preinjury activities included hiking (55%), winter sports (15%), and mountaineering (12%), and the most common types of injuries included sprains, strains and soft tissue injuries (28%), fractures or dislocations (26%), and lacerations (15%). A total of 121 (23%) of the injuries occurred in children (<18 years of age). There were 19 deaths in the 2 national parks (18 men, 1 woman); all victims were adults. Hiking (58%) and mountaineering (26%) were the most common activities at the time of death. Mechanism of death included falls (37%), medical (eg, myocardial infarction) (21%), drowning (5%), and suicide (5%). Conclusions. - The most common type of injury was soft tissue injury, and injuries occurred most commonly while hiking, during daylight hours, and in the summer. Preinjury activities and types of injuries were different in children compared with adults. Knowledge of how and when injuries occur in national parks can assist in determining what resources are needed to help provide a safer environment for park visitors. This study may also aid prevention strategies in the national parks, guide training of rangers, aid in the preparation of first aid kits, and further the education of people who participate in wilderness activities
SN - 1080-6032
AD - (Stephens) University of Washington School of Medicine, Seattle, WA, United States (Diekema) University of Washington, Department of Pediatrics, Division of Emergency Medicine, Seattle, WA, United States (Diekema, Klein) Children's Hospital and Regional Medical Center, Seattle, WA, United States (Klein) Children's Hospital and Regional Medical Center, Mailstop B-5520, 4800 Sand Point Way NE, Seattle, WA 98105, United States
ER -

TY - JOUR
ID - 3838
T1 - Nonmedical costs associated with rotavirus disease requiring hospitalization
A1 - Lee,B.P.
A1 - Azimi,P.H.
A1 - Staat,M.A.
A1 - Louie,L.

A1 - Parada,E.
A1 - Berke,T.
A1 - Ward,R.L.
A1 - Bernstein,D.I.
A1 - Matson,D.O.
Y1 - 2005///
N1 - 2005536634
English
Journal: Article
KW - EMBASE
KW - article
KW - Child Care
KW - cost effectiveness analysis
KW - diaper
KW - Female
KW - gastroenteritis
KW - health care cost
KW - Hospitalization
KW - Human
KW - Infant
KW - major clinical study
KW - Male
KW - oral rehydration therapy
KW - pediatric hospital
KW - preschool child
KW - priority journal
KW - Rotavirus
KW - time series analysis
KW - United States
KW - virus infection/et [Etiology]
KW - Rotavirus vaccine
KW - virus vaccine
RP - NOT IN FILE
SP - 984
EP - 988
JF - Pediatric Infectious Disease Journal
JA - Pediatr Infect Dis J
VL - 24
IS - 11
CY - United States
N2 - Background: As the most common cause of severe diarrhea among children, rotavirus has a significant economic impact. Previous studies focused on the direct medical costs of rotavirus infections; however, nonmedical costs account for the majority of the financial burden from this disease. Herein, we report the results from the largest prospective study in the United States determining the nonmedical costs of severe rotavirus infections. Methods: Prospective, active, gastroenteritis case surveillance was conducted between November 1997 and December 1999 at 3 pediatric medical centers. Rotavirus infection was identified for 548 children admitted between 2 weeks and 5 years of age. Detailed information about nonmedical costs during the prehospitalization, hospitalization and posthospitalization periods was obtained through interviews. Results: The average nonmedical cost per case of rotavirus disease was $448.77, including $359.04 for missed work, $56.66 for transportation, $11.90 for oral rehydration solutions, $9.59 for diapers, $6.83 for child care changes, $3.82 for special foods and $0.93 for formula changes. More than one-half of these expenses (53%) occurred outside the hospitalization period, and 80% of the cost was attributable to missed work. Conclusions: With an estimated 50,000 hospitalizations attributable to rotavirus each year in the United States, the nonmedical costs of severe rotavirus infections may exceed $22 million annually. Previous cost effectiveness analyses of rotavirus vaccines substantially underestimated this burden, suggesting that the nonmedical costs associated with mild to moderate rotavirus disease have been similarly underestimated. These findings are needed to assess accurately the cost effectiveness of future rotavirus immunization strategies. Copyright 2005 by Lippincott Williams & Wilkins

SN - 0891-3668
AD - (Lee, Azimi, Louie) Children's Hospital and Research Center at Oakland, Oakland, CA, United States (Staat, Ward, Bernstein) Cincinnati Children's Hospital, Medical Center, Cincinnati, OH, United States (Parada, Berke, Matson) Center for Pediatric Research, Norfolk, VA, United States
ER -

TY - JOUR
ID - 3839
T1 - The role of health care providers in the prevention of overweight and type 2 diabetes in children and adolescents
A1 - Stender,S.R.S.
A1 - Burghen,G.A.
A1 - Mallare,J.T.
Y1 - 2005///
N1 - 2005521385
English
Journal: Review
KW - EMBASE
KW - Adolescent
KW - Child
KW - community care
KW - Comorbidity
KW - environmental factor
KW - epidemic
KW - Family
KW - health care planning
KW - health care system
KW - Health Promotion
KW - heredity
KW - Human
KW - Lifestyle
KW - Motivation
KW - non insulin dependent diabetes mellitus/pc [Prevention]
KW - non insulin dependent diabetes mellitus/th [Therapy]
KW - Obesity/ep [Epidemiology]
KW - Obesity/et [Etiology]
KW - obesity/pc [Prevention]
KW - obesity/si [Side Effect]
KW - Obesity/th [Therapy]
KW - physiotherapy
KW - psychosocial environment
KW - Psychotherapy
KW - Religion
KW - review
KW - Treatment Outcome
KW - anticonvulsive agent/ae [Adverse Drug Reaction]
KW - antiinflammatory agent/ae [Adverse Drug Reaction]
KW - gabapentin/ae [Adverse Drug Reaction]
KW - medroxyprogesterone acetate/ae [Adverse Drug Reaction]
KW - prednisone/ae [Adverse Drug Reaction]
KW - psychotropic agent/ae [Adverse Drug Reaction]
KW - risperidone/ae [Adverse Drug Reaction]
KW - valproic acid/ae [Adverse Drug Reaction]
RP - NOT IN FILE
SP - 240
EP - 248
JF - Diabetes Spectrum

VL - 18
IS - 4
CY - United States
N2 - Extreme measures, such as ketogenic diets and surgery, have been tried to deal quickly with obesity, but recidivism is high. There are no quick preventions or cures. The answer-permanent behavior change-is amazingly simple in description but formidable in implementation. Obesity prevention will require radical lifestyle change across the lifespan. Such change takes time, discipline, perseverance, and daily effort. And if it is not viewed as permanent, the efforts will be in vain. So what do we know, and what can we do? We know that overweight adolescents are at high risk of becoming obese adults. We know that this is a problem that may begin in utero, and insulin resistance and oxidative endothelial cell damage are basic to the disease. We know that obesity is a disease of all ages and all nationalities, not just those with thrifty genes. The morbidity and mortality associated with this pandemic and their concomitant costs are monumental, with the potential of bankrupting the health care delivery and Social Security disability systems if the situation is not curbed. Human suffering associated with obesity further feeds the epidemic through depression, resulting lack of motivation to change, increased emotional eating and social isolation, and decreased physical activity. Hopelessness prevails among health care professionals trying to care for these patients. Their lament: "Nothing works." And indeed, there is little evidence thus far for success, at least not in the long term. There is an extreme paucity of evidence-based study, but the few trials reporting success, albeit short term, are multidisciplinary, involving psychological, dietary, and exercise components. We at the University of Tennessee Health Science Center are encouraged by our experience to date using our "Guidelines for Rearing Healthy Children," and we are now collecting data as to its effectiveness, both short term and with the intention of longitudinal follow-up. The American Academy of Pediatric policy statement on prevention of childhood overweight and obesity focuses on health supervision and advocacy in bringing about desperately needed societal changes. This statement calls for individualization of the plan for each child within the broader context of societal reforms related to issues ranging from vending machines to safe playgrounds to sound public health policies. Also suggested are advocacy efforts to obtain reimbursement for delivery of preventive medical care and education and funding for further research. The power of health care professionals to impact the lives of their patients cannot be overstated. When careful compassionate words create an informed, positive environment, this voice is heard above the media din, and the self-fulfilling prophecy of hope can begin its mission. We must be optimistic enough to believe in the ability of people to change and heal. Despite occasional steps backward, meaningful and permanent lifestyle changes can be effected. If we do not believe this to be so, neither will our patients
SN - 1040-9165
AD - (Stender) Department of Pediatrics and Adolescent Medicine, Division of General Pediatrics (Burghen) Department of Pediatrics (Burghen) Division of Endocrinology and Metabolism (Mallare) Department of Pediatrics, University of Tennessee Health Science Center, Le Bonheur Children's Medical Center, Memphis, TN, United States
ER -

TY - JOUR
ID - 3840
T1 - Feasibility of establishing open psychology ward in general hospitals for patients with neurosis and mental diseases
A1 - Zou,X.-B.
A1 - Lin,J.-D.
A1 - Yang,Z.-J.
A1 - Lin,Z.-X.
A1 - Chen,G.-M.
Y1 - 2005///
N1 - 2005502144
English
Journal: Article
KW - EMBASE
KW - Adolescent
KW - Adult
KW - Aged
KW - article
KW - China

KW - controlled study
KW - depression/th [Therapy]
KW - Exercise
KW - feasibility study
KW - Female
KW - general hospital
KW - Hamilton scale
KW - hospital admission
KW - hospital department
KW - Hospitalization
KW - Human
KW - Length of Stay
KW - major clinical study
KW - Male
KW - mental disease/th [Therapy]
KW - mental health care
KW - neurosis/th [Therapy]
KW - psychiatric treatment
KW - school child
RP - NOT IN FILE
SP - 238
EP - 239
JF - Chinese Journal of Clinical Rehabilitation
VL - 9
IS - 20
CY - China
N2 - Background: At present, most general hospitals in China treat patients with neurosis and mental diseases merely by means of clinical psychological consultation, which cannot meet the requirements of social service for metal disorders. Is it feasible to establish open psychology wards? Objective: To investigate the feasibility of establishing open psychology ward in general hospitals for patients with neurosis and mental diseases. Design: Case analysis based on patients. Setting: Department of Psychology of Guangdong Medical College Affiliated Hospital. Participants: Totally 900 patients with various types of neurosis were admitted to the Department of Psychology of Guangdong Medical College Affiliated Hospital between July 2000 and March 2003. There were 688 patients with different kinds of psychoneurosis and 112 cases of mental diseases. Of them, there were 523 men and 377 women aged from 11 to 80 years with the mean age of (42+8) years and hospitalization duration of(17.0+6.5) days. Methods: Open management consisting of open ward, supporting system, humanized restriction, collective physical exercise and psychological support was adopted. Symptom checklist (SCL-90) was used by trained psychological doctors to assess the therapeutic effects in the 900 patients with neurosis and mental diseases after two-month rehabilitation. Meanwhile, 169 patients with depression were examined with Hamilton rating scale for depression(HAMD) at 2,4,8-weeks of treatment. Main outcome measures: 1 The results of HAMD in the 169 depressive patients after treatment; 2 The therapeutic effect in the 900 patients. Results: Open psychology ward could provide an environment for patients to live and make free contact with the outside world. Of the 900 patients with various neurosis and metal diseases, 531 (59.0%) had recovery to normal, 264(29.4%) had obvious improvement, 81(9.0%) had improvement, and 24 (2.6%) had no therapeutic effect, with the total effective rate of 97.4%. None of the patients had any accident. HAMD score of the 169 patients with depression was (32.3+3.6) before treatment, was obviously lower at 2,4 and 8 weeks of treatment [(17.4+4.2), (11.5+4.8) and(5.8+3.6),respectively](P < 0.01). Conclusion: The therapeutic effect of the open psychology ward in the general hospital on various neuropathies and mental diseases was 97.4% in the 900 cases, with no accident occurring during hospitalization. Depression of the 169 patients was obviously improved, indicating that it is feasible to establish open psychology ward in general hospitals
SN - 1671-5926
AD - (Zou, Lin, Yang, Lin, Chen) Department of Psychology, Hospital of Guandong Medical College, Zhanjiang 524001 Guangdong Province, China
ER -

TY - JOUR
ID - 3841

T1 - Effects of psychological behavior therapy and interests actuating in the treatment of amblyopia in children
A1 - Huang,W.-R.
A1 - Song,C.-H.
A1 - Ma,L.
Y1 - 2005///
N1 - 2005501303
Chinese
Journal: Article
KW - EMBASE
KW - Acclimatization
KW - amblyopia/di [Diagnosis]
KW - Amblyopia/th [Therapy]
KW - article
KW - Behavior Therapy
KW - Child
KW - Child Care
KW - Child Psychology
KW - clinical trial
KW - controlled clinical trial
KW - controlled study
KW - Exercise
KW - Female
KW - Human
KW - major clinical study
KW - Male
KW - Psychotherapy
KW - randomized controlled trial
KW - Reinforcement
KW - scintillation
KW - spectacles
KW - visual system examination
RP - NOT IN FILE
SP - 106
EP - 107
JF - Chinese Journal of Clinical Rehabilitation
VL - 9
IS - 28
CY - China
N2 - Aim: The reinforcement method of psychological behavior therapy combined with the interests actuating in the treatment of amblyopia of children, and to observe its effects. Methods: 1 172 amblyopic children (287 eyes) who were diagnosed in Outpatient Ophthalmology Clinic of Kaifeng Children's Hospital between January 2000 and June 2003 were selected, based on the awareness of patients and parents. They were randomly divided into two groups: Control group with 87 cases (141 eyes) and psychotherapy group with 85 cases (146 eyes). 2 The commonly used complex treatment (wearing glasses + covering + red light scintillation and meticulous exercises training, once for 15 minutes in each day) were applied in control group; The reinforcement therapy of psychotherapy and interest actuating were add to psychotherapy group on the base of the commonly used complex treatment. The different grade prizes or outside activities were awarded to children in time to irritate children's synergic motivation and enhance their acclimatization. 3 The sight changes of the children in the two groups were observed, and review and eyesight examination were conducted once every 4 months respectively and the effects were compared. Inefficiency was retrogress, immovability or increase of 0.1 of sight; Utility was increase of 0.2 or over 0.2 of sight; Significant utility was that the sight increased to > 0.9 (The effective rate=significant utility + cases of utility/the total cases of the group). 4 The difference of enumeration data was detected by chi² check. Results: 172 children patients (287 eyes) with amblyopia were all involved in the result analysis. The effective rate of intervention: It bacame higher as the prolongation of intervention time in the two groups. At the intervention for 3 months, it was similar in control group and psychotherapy group (46.1%, 49.3%, P > 0.05); But at the intervention for 6, 9 and 12 months, it was significantly higher in psychotherapy group than that in control group (71.3%, 78.8%, 83.5%; 55.3%, 59.6%, 64.5%,

chi²=7.83, 12.43, 13.56, P < 0.01). Conclusion: The reinforcement method of psychological behavior therapy which is suitable for the psychological character of children combined with the interests actuating can effectively enhance more effectively the therapeutic efficacy of the sight level of amblyopia children patients
SN - 1671-5926
AD - (Huang) Department of Ophthalmology, Kaifeng Children's Hospital, Kaifeng 475000 Henan Province, China (Song) Department of Psychology, Kaifeng Children's Hospital, Kaifeng 475000 Henan Province, China (Ma) Department of Pediatric Ophthalmology, Kaifeng Ophthalmological Hospital, Kaifeng 475000 Henan Province, China
ER -

TY - JOUR
ID - 3842
T1 - Physical activity during school recess: The liverpool sporting playgrounds project
A1 - Ridgers,N.D.
A1 - Stratton,G.
Y1 - 2005///
N1 - 2005420183
English
Journal: Article
KW - EMBASE
KW - article
KW - Child
KW - Female
KW - Heart Rate
KW - Human
KW - Male
KW - Monitoring
KW - normal human
KW - Physical Activity
KW - primary school
KW - school
KW - sex difference
KW - Telemetry
KW - Time
RP - NOT IN FILE
SP - 281
EP - 290
JF - Pediatric Exercise Science
JA - Pediatr Exerc Sci
VL - 17
IS - 3
CY - United States
N2 - Recess offers primary-school-age children the opportunity to engage in physical activity, though few studies have detailed the physical activity levels of children in this environment. The physical activity levels of 270 children ages 6-11 years from 18 schools were monitored on 1 school day using heart rate telemetry. Data revealed that boys engaged in higher levels of moderate-tovigorous and vigorous physical activity (MVPA) than did girls during recess (26 and 20 min, respectively). These results suggest that recess can make a worthwhile contribution to the recommended 60 min of MVPA per day. 2005 Human Kinetics, Inc
SN - 0899-8493
AD - (Ridgers) REACH Group, School of Physical Education, Sport and Dance, Liverpool John Moores University, Barkhill Road, Liverpool, L17 6BD, United Kingdom (Stratton) REACH Group, Research Institute for Sport and Exercise Sciences, Liverpool John Moores University, 15-21 Webster Street, Liverpool, L3 2ET, United Kingdom
ER -

TY - JOUR

ID - 3843
T1 - Thyroid autoimmunity in chronic urticaria
A1 - O'Donnell,B.F.
A1 - Francis,D.M.
A1 - Swana,G.T.
A1 - Seed,P.T.
A1 - Black,A.K.
A1 - Greaves,M.W.
Y1 - 2005///
N1 - 2005396079
English
Journal: Article
KW - EMBASE
KW - Adolescent
KW - Adult
KW - Aged
KW - article
KW - autoimmunity
KW - chronic urticaria
KW - controlled study
KW - disease association
KW - Female
KW - Human
KW - major clinical study
KW - Male
KW - priority journal
KW - school child
KW - thyroid disease
KW - microsome antibody/ec [Endogenous Compound]
KW - thyroid antibody/ec [Endogenous Compound]
KW - thyrotropin/ec [Endogenous Compound]
RP - NOT IN FILE
SP - 331
EP - 335
JF - British Journal of Dermatology
JA - Br J Dermatol
VL - 153
IS - 2
CY - United Kingdom
N2 - Background: Chronic urticaria (CU) is an autoimmune process in some patients. An association between CU and autoimmune thyroid disease has also previously been proposed. Our group has identified functionally significant histamine-releasing autoantibodies in one subset of CU patients (subset 1), predicted by positive autologous intradermal serum tests and positive histamine release from donor basophil leucocytes in vitro. Sera from a second subset of patients (subset 2), all of whom had positive autologous intradermal serum tests, failed to release histamine from donor basophils. A final disease subset (subset 3) has no identifiable skin reactivity (negative autologous serum skin test) or in vitro histamine releasing activity. Objectives: In order to examine further the possible relationships between thyroid autoimmunity, thyroid dysfunction and CU, we have examined thyroid autoantibodies and thyroid-stimulating hormone (TSH) levels (an indirect measure of thyroid dysfunction) in the three CU subsets. Patients/methods: We studied 182 patients (69% female), of whom 90 had a positive autologous intradermal serum test. Results: Eighteen skin test-positive and four skin test-negative patients had thyroid microsomal antibodies (TMA). TSH outside the normal range was found in 13 skin test-positive and one skin test-negative patient. These findings represent clustering of TMA positivity [risk ratio (RR) 4.06, 95% confidence interval (CI) 1.56-10.6] and of abnormal thyroid function (RR 15.5, CI 2.07-11.6) among the skin test-positive patients. However, in the overall study group an elevated TSH was present in seven patients (3.8%, CI 1.6-7.8) comparable to the 5% expected prevalence in the community. Thyroglobulin antibodies (TGA) were present in two of 182 patients. Conclusions: There were significant differences between skin test-positive and skin test-negative patients with regard to autoimmune thyroid disease. Evidence for

autoimmune thyroid disease and abnormal thyroid function was largely found among the skin test-positive patients, supporting the theory of an autoimmune aetiology in this group. 2005 British Association of Dermatologists
SN - 0007-0963
AD - (O'Donnell, Francis, Black, Greaves) St. John's Institute of Dermatology, St. Thomas' Hospital, Lambeth Palace Road, London SE1 7EH, United Kingdom (Swana) Department of Immunology, Guy's, King's and St. Thomas' School of Medicine (GKT), St. Thomas' Hospital, Lambeth Palace Road, London SE1 7EH, United Kingdom (Seed) Department of Obstetrics, GKT School of Medicine, King's College, London, United Kingdom (O'Donnell) Department of Dermatology, Children's University Hospital, Temple Street, Dublin 1, Ireland
ER -

TY - JOUR
ID - 3845
T1 - A randomized effectiveness trial of brief cognitive-behavioral therapy for depressed adolescents receiving antidepressant medication
A1 - Clarke,G.
A1 - DeBar,L.
A1 - Lynch,F.
A1 - Powell,J.
A1 - Gale,J.
A1 - O'Connor,E.
A1 - Ludman,E.
A1 - Bush,T.
A1 - Lin,E.H.B.
A1 - Von,Korff M.
A1 - Hertert,S.
Y1 - 2005///
N1 - 2005384988
English
Journal: Article
KW - EMBASE
KW - Adjustment
KW - Adolescent
KW - Adult
KW - article
KW - Behavior Therapy
KW - Child Psychiatry
KW - clinical trial
KW - Cognitive Therapy
KW - controlled clinical trial
KW - controlled study
KW - emotion
KW - Female
KW - follow up
KW - health maintenance organization
KW - Human
KW - major clinical study
KW - major depression/di [Diagnosis]
KW - major depression/dt [Drug Therapy]
KW - major depression/th [Therapy]
KW - Male
KW - outpatient care
KW - Patient Compliance
KW - primary medical care
KW - priority journal
KW - randomized controlled trial
KW - relapse

KW - school child
KW - Short Form 12 Mental Component Scale
KW - Short Form 36
KW - serotonin uptake inhibitor/dt [Drug Therapy]
RP - NOT IN FILE
SP - 888
EP - 898
JF - Journal of the American Academy of Child and Adolescent Psychiatry
VL - 44
IS - 9
CY - United States
N2 - Objective: To test a collaborative-care, cognitive-behavioral therapy (CBT) program adjunctive to selective serotonin reuptake inhibitor (SSRI) treatment in HMO pediatric primary care. Method: A randomized effectiveness trial comparing a treatment-as-usual (TAU) control condition consisting primarily of SSRI medication delivered outside the experimental protocol (n = 75) versus TAU SSRI plus brief CBT (n = 77). Participants were identified by a recent dispense of SSRI medication followed by telephone screening. Adolescents with a diagnosis of major depressive disorder (n = 152) were enrolled. The CBT program employed cognitive restructuring and/or behavioral activation training. Therapists consulted with prescribing pediatricians to improve medication adherence. Results: Through 1-year follow-up, the authors found CBT advantages on the Short Form-12 Mental Component Scale (p = .04), reductions in TAU outpatient visits (p = .02), and days' supply of all medications (p = .01). No effects were detected for major depressive disorder episodes; a nonsignificant trend favoring CBT was detected on the Center for Epidemiology Depression Scale (p = .07). Conclusions: The authors detected a weak CBT effect, possibly rendered less significant by the small sample and likely attenuated by the unexpected reduction in SSRI pharmacotherapy in the CBT condition. Small, incremental improvements over monotherapy, such as observed in this study, most likely represent the new norm in adolescent depression treatment research. 2005 by the American Academy of Child and Adolescent Psychiatry
SN - 0890-8567
AD - (Clarke, DeBar, Lynch, Powell, Gale, O'Connor, Hertert) Kaiser Permanente Center for Health Research, Portland, OR, United States (Ludman, Bush, Lin, Von Korff) Group Health Cooperative Center for Health Studies, Seattle, WA, United States (Clarke) Center for Health Research, 3800 North Interstate Avenue, Portland, OR 97227-1098, United States
ER -

TY - JOUR
ID - 3846
T1 - Sudden infant death syndrome in playpens
A1 - De Jonge,G.A.
A1 - Semmekrot,B.A.
Y1 - 2005///
N1 - 2005270663
Dutch
Journal: Article
KW - EMBASE
KW - age
KW - article
KW - Birth Order
KW - body posture
KW - Child Care
KW - clinical article
KW - controlled study
KW - Female
KW - Human
KW - Infant
KW - Male
KW - parental behavior
KW - preschool child

KW - Recreation
KW - risk factor
KW - sex difference
KW - Sleep
KW - Smoking
KW - sudden infant death syndrome/ep [Epidemiology]
RP - NOT IN FILE
SP - 1279
EP - 1282
JF - Nederlands Tijdschrift voor Geneeskunde
JA - Ned Tijdschr Geneeskd
VL - 149
IS - 23
CY - Netherlands
N2 - Objective. To explain the increase in the number of deaths due to sudden infant death syndrome (SIDS) that occurred while the infant was in a playpen or on a playpen mat placed outside of the pen. 13 deaths were reported in the period I September 1996-31 August 2004, in contrast to 0 deaths in the years 1984/'96. Design. Descriptive. Method. Data on the 13 infants that were reported to the National Study Group on SIDS were analysed and the presence of risk factors for SIDS was assessed. Results. Of the 13 infants, 9 were male and 4 were female. 12 were aged less than 12 months and I was aged 13 months. Known risk factors for SIDS were often present and frequently occurred in combinations: male sex (n = 9), higher birth order (n = 9), age 1-8 months (n = 12), parental smoking (n = 6), primary prone sleeping position (n = 4), secundary prone (n = 6, often the first time), face down (n = 9), no adult present (n = 13). Conclusion. There was no clear explanation for the observed increase in SIDS that occurred in a playpen or on a playpen mat placed outside of the pen
SN - 0028-2162
AD - (De Jonge) Prins Bernhardlaan 50, 2341 KL Oegstgeest, Netherlands (Semmekrot) Canisius-Wilhelmina Ziekenhuis, Afd. Kindergeneeskunde, Nijmegen, Netherlands
ER -

TY - JOUR
ID - 3847
T1 - Exposure assessment and modeling of particulate matter for asthmatic children using personal nephelometers
A1 - Wu,C.-F.
A1 - Delfino,R.J.
A1 - Floro,J.N.
A1 - Quintana,P.J.E.
A1 - Samimi,B.S.
A1 - Kleinman,M.T.
A1 - Allen,R.W.
A1 - Liu,L.-J.
Y1 - 2005///
N1 - 2005270412
English
Journal: Article
KW - EMBASE
KW - accuracy
KW - Adolescent
KW - Air Pollution
KW - ambient air
KW - article
KW - Asthma
KW - controlled study
KW - error
KW - Female
KW - Home
KW - Human

KW - Male
KW - Mathematical Computing
KW - mathematical model
KW - microenvironment
KW - multiple regression
KW - Particulate Matter
KW - Prediction
KW - priority journal
KW - school
KW - school child
RP - NOT IN FILE
SP - 3457
EP - 3469
JF - Atmospheric Environment
VL - 39
IS - 19
CY - United Kingdom
N2 - It has been shown that acute exposures to particulate matter (PM) may exacerbate asthma in children. However, most epidemiological studies have relied on time-integrated PM measurements taken at a centrally located stationary monitoring sites. In this article, we characterized children's short-term personal exposures to PM_{2.5} (PM with aerodynamic diameters <2.5 mum) and separated them into ambient and nonambient components. The personal DataRAM without a size-selective inlet was used to estimate real-time PM _{2.5} concentrations on 20 asthmatic children, inside and outside of their residences, and at a central site. The personal and indoor pDRs were operated passively, while the home outdoor and central site instruments were operated actively. The subjects received 29.2% of their exposures at school, even though they only spent 16.4% of their time there. More precise personal clouds were estimated for the home-indoor and home-outdoor microenvironments where PM concentrations were measured. The personal cloud increased with increasing activity levels and was higher during outdoor activities than during indoor activities. We built models to predict personal PM exposures based on either microenvironmental or central-site PM_{2.5} measurements, and evaluated the modeled exposures against the actual personal measurements. A multiple regression model with central site PM concentration as the main predictor had a better prediction power (R2=0.41) than a three- microenvironmental model (R2=0.11). We further constructed a source-specific exposure model utilizing the time-space-activity information and the particle infiltration efficiencies (mean=0.72+0.15) calculated from a recursive mass balance model. It was estimated that the mean hourly personal exposures resulting from ambient, indoor-generated, and personal activity PM_{2.5} were 11.1, 5.5, and 10.0 mug/m³, respectively, when the modeling error was minimized. The high PM_{2.5} exposure to personal activities reported in our study is likely due to children's more active lifestyle as compared with older adult subjects in previous studies. 2005 Elsevier Ltd. All rights reserved
SN - 1352-2310
AD - (Wu) Department of Public Health, National Taiwan University, Taipei 100, Taiwan (Republic of China) (Delfino, Floro) Epidemiology Division, Department of Medicine, University of California at Irvine, Irvine, CA 92697, United States (Quintana, Samimi) Division of Occupational and Environmental Health, San Diego State University, San Diego, CA 92182, United States (Kleinman) Department of Community and Environmental Medicine, University of California at Irvine, Irvine, CA 92697, United States (Allen, Liu) Department of Environmental and Occupational Health Sciences, University of Washington, Seattle, WA 98195, United States
ER -

TY - JOUR
ID - 3848
T1 - Development of the PEARLS model (Particulate Exposure from Ambient to Regional Lung by Subgroup) and use of Monte Carlo simulation to predict internal exposure to PM2.5 in Toronto
A1 - Gower,S.K.
A1 - McColl,S.
Y1 - 2005///
N1 - 2005216821
English
Journal: Article

KW - EMBASE
KW - Adolescent
KW - Adult
KW - Aged
KW - Air Pollution
KW - ambient air
KW - article
KW - Canada
KW - Child
KW - Female
KW - Human
KW - Infant
KW - lung alveolus
KW - Male
KW - mathematical analysis
KW - Monte Carlo Method
KW - Morbidity
KW - Mortality
KW - Particulate Matter
KW - population exposure
RP - NOT IN FILE
SP - 301
EP - 315
JF - Risk Analysis
JA - Risk Anal
VL - 25
IS - 2
CY - United States
N2 - Air pollution is a current and growing concern for Canadians, and there is evidence that ambient levels that meet current exposure standards may be associated with mortality and morbidity in Toronto, Canada. Evaluating exposure is an important step in understanding the relationship between particulate matter (PM) exposure and health outcomes. This report describes the PEARLS model (Particulate Exposure from Ambient to Regional Lung by Subgroup), which predicts exposure distributions for 11 age-gender population subgroups in Toronto to PM_{2.5} (PM with a median aerodynamic diameter of 2.5 mum or less) using Monte Carlo simulation techniques. The model uses physiological and activity pattern characteristics of each subgroup to determine region-specific lung exposure to PM_{2.5}, which is defined as the mass of PM_{2.5} deposited per unit time to each of five lung regions (two extrathoracic, bronchial, bronchiolar, and alveolar). The modeling results predict that children, toddlers, and infants have the broadest distributions of exposure, and the greatest chance of experiencing extreme exposures in the alveolar region of the lung. Importance analysis indicates that the most influential model variables are air exchange rate into indoor environments, time spent outdoors, and time spent at high activity levels. Additionally, a "critical point" was defined and introduced to the PEARLS to investigate the effects of possible threshold-pathogenic phenomena on subgroup exposure patterns. The analysis indicates that the subgroups initially predicted to be most highly exposed were likely to have the highest proportion of their population exposed above the critical point. Substantial exposures above the critical point were predicted in all subgroups for ambient concentrations of PM_{2.5} commonly observed in Toronto after continuous exposure of 24 hours or more. 2005 Society for Risk Analysis
SN - 0272-4332
AD - (Gower, McColl) University of Waterloo, Health Studies and Gerontology, Waterloo, Ont., Canada (McColl) Institute for Risk Research, Waterloo, Ont., Canada (McColl) Dep't. Health Studies and Gerontology, University of Waterloo, 200 University Ave. W., Waterloo, Ont. N2L 361, Canada
ER -

TY - JOUR
ID - 3849
T1 - Children theorize about reasons for recess engagement: Does expectancy-value theory apply?
A1 - Watkinson,E.J.

A1 - Dwyer,S.A.
A1 - Nielsen,A.B.
Y1 - 2005///
N1 - 2005183156
English
Journal: Article
KW - EMBASE
KW - Achievement
KW - article
KW - clinical article
KW - Cognitive Development
KW - Comprehension
KW - data analysis
KW - Decision Making
KW - expectancy value theory
KW - expectation
KW - experience
KW - Female
KW - Human
KW - Interview
KW - leisure
KW - Male
KW - normal human
KW - Perception
KW - Performance
KW - Physical Activity
KW - Physical Development
KW - Psychological Theory
KW - Recreation
KW - school child
KW - social evolution
KW - Social Interaction
RP - NOT IN FILE
SP - 179
EP - 197
JF - Adapted Physical Activity Quarterly
JA - Adapt Phys Act Q
VL - 22
IS - 2
CY - United States
N2 - Inclusion in activity at recess can have important implications for the health and for the physical, social, and cognitive development of children, according to play theorists (Pellegrini, 1995). This study examined whether children described their decisions (and those of fictitious others) to engage in recess activities in achievement terms consistent with expectancy-value theory (Eccles, Wigfield & Schiefele, 1998). Ten Grade 3 children with different patterns of recess engagement did confirm that attainment, interest, utility, and cost values were salient to decisions to participate. Children distinguished among value components, and confirmed that expectancies and values contributed to activity choices, providing support for the conceptualization of recess as an achievement setting in which expectancy-value theory applies. 2005 Human Kinetics Publishers, Inc
SN - 0736-5829
AD - (Watkinson, Dwyer, Nielsen) University of Alberta (Watkinson, Dwyer, Nielsen) Fac. of Phys. Educ. and Recreation, University of Alberta, Edmonton, Alta. T6G 2H9, Canada
ER -

TY - JOUR
ID - 3850
T1 - Comparison of physical activity levels between children with and without autistic spectrum disorders

A1 - Sandt,D.D.R.
A1 - Frey,G.C.
Y1 - 2005///
N1 - 2005183154
English
Journal: Article
KW - EMBASE
KW - accelerometer
KW - article
KW - Autism
KW - clinical article
KW - comparative study
KW - controlled study
KW - data analysis
KW - Demography
KW - Female
KW - Human
KW - leisure
KW - Male
KW - Physical Activity
KW - Physical Education
KW - school
KW - school child
KW - sitting
RP - NOT IN FILE
SP - 146
EP - 159
JF - Adapted Physical Activity Quarterly
JA - Adapt Phys Act Q
VL - 22
IS - 2
CY - United States
N2 - The purpose of this study was to compare daily, physical education, recess, and after school moderate to vigorous physical activity (MVPA) levels between children with and without autistic spectrum disorders (ASD). Children ages 5 to 12 years wore a uniaxial accelerometer for five days (four weekdays, one weekend day). There were no differences between children with and without ASD at any physical activity setting. Both groups were more active during recess compared to after school, and children with ASD were similarly active in recess and physical education. Although many children with ASD acquired 60 min of physical education per day, this may decrease with age as opportunities for recess and physical education are eliminated. 2005 Human Kinetics Publishers, Inc
SN - 0736-5829
AD - (Sandt) University of New Mexico (Frey) Indiana University (Sandt, Frey) Texas A and M University, College Station (Sandt) Dept. of Phys. Perf. and Development, University of New Mexico, Albuquerque, NM 87110, United States (Frey) Department of Kinesiology, Indiana University, Bloomington, IN 47405, United States
ER -

TY - JOUR
ID - 3851
T1 - The epidemiology of head injury in a hospital emergency service
A1 - Fernandez-Mayoralas,D.M.
A1 - Coll,Torres E.
A1 - Munoz,Jareno N.
A1 - San,Antonio Arce,V
A1 - Miravet,Fuster E.
A1 - Briceno,Cuadros S.
A1 - Campos-Castello,J.

Y1 - 2005///
N1 - 2005146719
Spanish
Journal: Article
KW - EMBASE
KW - Adolescent
KW - article
KW - Child
KW - computer assisted tomography
KW - emergency health service
KW - Female
KW - head injury/di [Diagnosis]
KW - head injury/dt [Drug Therapy]
KW - head injury/ep [Epidemiology]
KW - head injury/su [Surgery]
KW - Human
KW - Infant
KW - major clinical study
KW - Male
KW - Prevalence
KW - socioeconomics
KW - ibuprofen/dt [Drug Therapy]
KW - paracetamol/dt [Drug Therapy]
RP - NOT IN FILE
SP - 55
EP - 62
JF - Acta Pediatrica Espanola
VL - 63
IS - 2
CY - Spain
N2 - Objective. To determine the epidemiology of mild head injury by means of a study carried out among children treated in hospital emergency services. Material and methods. We evaluated 153 cases of pediatric head injury diagnosed in our institution. The data were analyzed using descriptive statistics. Results. The series consisted of 95 boys and 58 girls (94.1% were white). In 68.6% of cases, the mother worked outside the home. The socioeconomic conditions were poor in 6.5%. The most prevalent number of siblings per family was two. Injuries were more frequent between 11:00 and 13:00 hours (24.9%). A large majority of the injuries were produced outside the home (64.7%), with 19.6% occurring at the school playground, while 35.3% occurred at home. Frontal region injury accounted for 26.1% of the cases and 52.9% were treated by medical procedures and/or minor surgery. Conclusions. Mild head injuries were most prevalent among children under 5 years of age and occurred more frequently in boys (62.1%) than in girls; 32.7% of the children had had a previous head injury, while 19.7% had had more than one. The accidents occurred more frequently outdoors, mainly at the school playground and parks. The frontal region was that most often involved. The children received medical attention within 2 hours in 68% of cases. Neuroimaging was not necessary in our series
SN - 0001-6640
AD - (Fernandez-Mayoralas, Munoz Jareno, San Antonio Arce, Miravet Fuster, Briceno Cuadros, Campos-Castello) Servicio de Neuropediatria, Hospital Clinico San Carlos, Madrid, Spain (Coll Torres) Servicio de Medicina Preventive, Hospital Clinico San Carlos, Madrid, Spain (Fernandez-Mayoralas) C/Marques de Vallejo, 11, 28028 Madrid, Spain
ER -

TY - JOUR
ID - 3852
T1 - A long-term rehabilitation programme for children with all severities of TBI
A1 - Rehn,P.
A1 - Gotthard,M.
A1 - Hagberg,M.
A1 - Weiland,A.-L.

A1 - Zetterqvist,B.
A1 - Forssell,U.
A1 - Van't,Hooft,I
A1 - Gunnarson,E.
A1 - Ericsson,B.
Y1 - 2005///
N1 - 70008176
English
Journal: Conference Abstract
KW - EMBASE
KW - Rehabilitation
KW - traumatic brain injury
KW - Child
KW - patient
KW - school
KW - Therapy
KW - Hospital
KW - social network
KW - psychosocial care
KW - Environment
KW - outpatient
KW - cognitive defect
KW - screening
KW - childhood
KW - adolescence
KW - follow up
KW - medical specialist
KW - psychiatrist
KW - injury
KW - Consciousness
KW - hospital patient
KW - ward
KW - Day Care
KW - feedback system
KW - pediatric hospital
RP - NOT IN FILE
SP - 305
JF - Developmental neurorehabilitation
JA - Dev Neurorehabil
VL - Conference: New Frontiers in Pediatric Traumatic Brain Injury Conference San Diego, CA United States. Conference Start: 20071108 Conference End: 20071110. Conference Publication:
IS - var.pagings
N2 - Purpose: To define a comprehensive, long-term, child- and family-centred rehabilitation programme for patients following TBI. Methods: Patients are treated by three integrated multi-disciplinary teams, according to stage of recovery. The programme offers a continuous follow-up, from the acute phase until the patient reaches the age of 18. In addition to teams' own neurologists, team members may consult specialists such as neurosurgeons, ophthalmologists and psychiatrists on demand at any stage of the rehabilitation. The hospital team: In the acute phase post-injury, focus is put on informative and emotional support of the family and early multi-sensory therapy to achieve initial consciousness. Sub-acutely the rehabilitation is intensified, still located in the in-patient ward. When medical stability is reached, the patient is transferred to day-care therapy. Assessment weeks are held regularly, during which progress is evaluated by all therapists. Standardized instruments are used for evaluation. These instruments offer the teams a reliable baseline status, help form adequate therapy programmes and offer the possibility to evaluate progress over time. Feedback and recommendations are given to family and social network on a continuous basis. The school rehabilitation team: After the day therapy programme the patient is offered continued physical rehabilitation as well as cognitive and social therapy in a school-like environment, outside the hospital. The school rehabilitation team stays in close contact with patient's home school or a more appropriate school alternative. The outreach service: This team

offers an outpatient rehabilitation programme for patients following mild TBI, suffering mainly from cognitive impairments. The team provides psychological and cognitive screenings in order to give adequate advice to home and school. The team activates networks and local resources close to the patient's home. Conclusions: The rehabilitation programme at Astrid Lindgren Children's Hospital improves the patients' chances to: (1) leave the hospital at an earlier stage of recovery, (2) return to their home school and succeed academically and socially and (3) minimize secondary consequences, physical as well as social. The programme offers the patients' families continuous support throughout childhood and adolescence
SN - 1751-8423
AD - (Rehn, Gotthard, Hagberg, Weiland, Zetterqvist, Forssell, Van't Hooft, Gunnarson, Ericsson) Astrid Lindgren Children's Hospital, Karolinska University Hospital, Stockholm, Sweden
ER -

TY - JOUR
ID - 3854
T1 - Indoor air pollution and airway disease
A1 - Viegi,G.
A1 - Simoni,M.
A1 - Scognamiglio,A.
A1 - Baldacci,S.
A1 - Pistelli,F.
A1 - Carrozzi,L.
A1 - nnesi-Maesano,I.
Y1 - 2004///
N1 - 2005021771
English
Journal: Review
KW - EMBASE
KW - Acute Disease
KW - Adolescent
KW - Adult
KW - Aged
KW - air pollutant
KW - Air Pollution
KW - Asthma/ep [Epidemiology]
KW - asthma/pc [Prevention]
KW - biomass
KW - bird
KW - Bronchitis/ep [Epidemiology]
KW - bronchitis/pc [Prevention]
KW - cat
KW - Child
KW - Chronic Disease
KW - chronic obstructive lung disease/ep [Epidemiology]
KW - chronic obstructive lung disease/pc [Prevention]
KW - cleaning
KW - cockroach
KW - Cooking
KW - coughing
KW - Day Care
KW - Dermatophagoides
KW - dog
KW - domestic animal
KW - dyspnea
KW - Environmental Exposure
KW - Female
KW - fungus
KW - heating

KW - Home
KW - Human
KW - indoor air pollution
KW - insect
KW - lung function
KW - major clinical study
KW - Male
KW - motor vehicle
KW - mould
KW - Particulate Matter
KW - Pollen
KW - pollution control
KW - priority journal
KW - respiratory tract disease/ep [Epidemiology]
KW - respiratory tract disease/pc [Prevention]
KW - review
KW - rhinitis/ep [Epidemiology]
KW - rhinitis/pc [Prevention]
KW - risk factor
KW - rodent
KW - school
KW - wheezing
KW - Wood
KW - Workplace
KW - adhesive agent
KW - aldehyde
KW - aliphatic hydrocarbon
KW - allergen
KW - aromatic hydrocarbon
KW - benzene
KW - carbon monoxide
KW - formaldehyde
KW - fuel
KW - house dust allergen
KW - nitrogen dioxide
KW - Paint
KW - pollen antigen
KW - solvent
KW - styrene
KW - terpene
KW - tobacco smoke
KW - volatile organic compound
RP - NOT IN FILE
SP - 1401
EP - 1415
JF - International Journal of Tuberculosis and Lung Disease
VL - 8
IS - 12
CY - France
N2 - Scientific interest in indoor pollution has been increasing since the second half of the 1980s. Growing scientific evidence has shown that because people generally spend the majority of their time indoors, indoor pollution plays a significant role in affecting health and is thus an important health issue. Indoor environments include dwellings, workplaces, schools and day care centres, bars, discotheques and vehicles. Common indoor pollutants are environmental tobacco smoke, particulate matter, nitrogen dioxide, carbon monoxide, volatile organic compounds and biological allergens. In developing countries, relevant sources of indoor pollution include biomass and coal burning for cooking and heating. Concentrations of these pollutants can be many times higher indoors than outdoors. Indoor air pollution may increase the risk of irritation phenomena, allergic

sensitisation, acute and chronic respiratory disorders and lung function impairment. Recent conservative estimates have shown that 1.5-2 million deaths per year worldwide could be attributed to indoor air pollution. Approximately 1 million of these deaths occur in children aged under 5 years due to acute respiratory infections, and significant proportions of deaths occur due to chronic obstructive pulmonary disease and lung cancer in women. Today, indoor air pollution ranks tenth among preventable risk factors contributing to the global burden of disease. Further research is necessary to better evaluate the respiratory health effects of indoor pollution and to implement protective programmes for public health
SN - 1027-3719
AD - (Viegi, Simoni, Scognamiglio, Baldacci) Pulmon. Environ. Epidemiology Unit, CNR Institute of Clinical Physiology, Pisa, Italy (Pistelli, Carrozzi) Cardiothoracic Department, University Hospital, Pisa, Italy (Annesi-Maesano) Epidemiol. Allerg./Resp. Dis. Dept., INSERM U472, Villejuif, France (Viegi) Pulmon. Environ. Epidemiology Unit, CNR Institute of Clinical Physiology, Via Trieste 41, 56126 Pisa, Italy
ER -

TY - JOUR
ID - 3855
T1 - Experimental evaluation of the impact-absorption properties of rubber tiles for the playground
A1 - Chang,L.-T.
A1 - Tsai,K.-H.
A1 - Shiau,J.-S.
Y1 - 2004///
N1 - 2005010941
English
Journal: Article
KW - EMBASE
KW - Absorption
KW - Acceleration
KW - article
KW - Child
KW - childhood injury/pc [Prevention]
KW - falling
KW - head injury/pc [Prevention]
KW - Human
KW - materials
KW - play
KW - protective equipment
KW - Safety
KW - solid
KW - standard
KW - thickness
KW - Rubber
RP - NOT IN FILE
SP - 244
EP - 250
JF - Biomedical Engineering - Applications, Basis and Communications
VL - 16
IS - 5
CY - Taiwan (Republic of China)
N2 - Rubber tiles are popular in playgrounds as protective surfacing to reduce the incidence of head injuries caused by children falling from equipment. However, Taiwan has not yet established a test code for assessment of the shock-absorption properties of such surfacing. For this study, an experimental model was established to evaluate the behavior of various rubber tiles. A hemispherical headform was dropped from a set height to strike the center of the specimen tile. The peak acceleration and Head Injury Criterion (HIC) were measured to assess the impact absorption of and critical height for a given rubber tile. The results show that utilization of the HIC index provides a more conservative assessment of the shock absorption and, ultimately, protection from head injuries than peak acceleration. The maximum critical heights of the rubber tiles used in this study for tile thicknesses of 45, 60 and 80 mm were 1.6, 2.0 and 2.2 m, respectively. Two-part rubber tiles with a base

structure consisting of a box-like core offer superior protection from head injuries relative to analogous cylindrical, square pillar and solid structures. The maximum differences in peak-acceleration and HIC values comparing the box-like core and solid structures at a thickness of 45 mm were 21% and 44%, respectively. The results of this study suggest a minimum of rubber thickness of 60 mm, based on probable maximum fall heights of more than 1.6 m. Moreover, incorporation of an appropriate cushioning structure in the base of the rubber tile could further improve protection
SN - 1016-2372
AD - (Chang, Shiau) Dept. of Childhood Educ. and Nursery, Chia Nan Univ. of Pharm. and Science, Tainan, Taiwan (Republic of China) (Tsai) Department of Industrial Management, S. Taiwan University of Technology, Taiwan (Republic of China) (Chang) Dept. of Childhood Educ. and Nursery, Chia Nan Univ. of Pharm. and Science, 60 Erh-Jen Road, Section 1, Jen Te, 717 Tainan, Taiwan (Republic of China)
ER -

TY - JOUR
ID - 3856
T1 - Psychometric characteristics of CES-D in a sample of Mexican rural adolescents in areas with a strong migratory tradition
A1 - guilera-Guzman,R.M.
A1 - Carreno Garcia,M.S.
A1 - Juarez,Garcia F.
Y1 - 2004///
N1 - 2005001290
Spanish
Journal: Conference Paper
KW - EMBASE
KW - Adolescent
KW - Child
KW - conference paper
KW - data analysis
KW - depression
KW - Female
KW - geographic distribution
KW - Hispanic
KW - Human
KW - major clinical study
KW - Male
KW - Mexico
KW - migration
KW - psychometry
KW - rating scale
KW - rural area
KW - sampling
KW - Self Esteem
KW - symptomatology
RP - NOT IN FILE
SP - 57
EP - 66
JF - Salud Mental
VL - 27
IS - 6
CY - Mexico
N2 - Introduction. Mexico has a long migration tradition: thousands of Mexican farm laborers and, more recently, industrial and service workers, constitute an essential sector of the US economy. The effects of this geographical movement on the physical and mental health of adults who participate in this process and the changes it produces in the familial and community sphere have been widely documented. Little is known, however, about the impact of these social processes on the mental health of members of the following generations, i.e., the offspring of international migrants who remain in their communities of origin. Various

international studies report that the psychosocial malaise that this process causes in children and teenagers is reflected in behavioral problems during childhood and identity conflicts and constant mood changes during adolescence, which may or may not turn into symptoms of various psychological disorders such as depressive symptomatology. In Mexico, authors such as Lopez and Moctezuma have pointed out that there is a culture of migration in the communities of origin of these children and teenagers which makes them view migration as "something natural", since this process has been part of the family history of their inhabitants for several generations. In Mexico, the adolescence of the children of migrants takes place in social contexts where the father's physical absence as a result of international migration is expected. Studies have also shown that migrants' wives may display high rates of depressive symptomatology associated with the numerous responsibilities and new tasks they perform as a result of their husbands' absence. All these circumstances may make these teenagers more vulnerable. It is therefore important to determine whether the father's physical absence due to international migration is in any way linked to the presence of possible depressive symptomatology in these teenagers. Method: The aim of this study was to report the psychometric characteristics of a sample of rural adolescents from communities with a strong migratory tradition from the State of Zacatecas on the Radloff Center for Epidemiological Studies Depression Scale (1977) and to provide new analytical tools to determine the effects of international migration on their mental health. The CES-D was applied to a sample of 310 adolescents from primary schools, during school hours, with the consent of the school authorities. It formed part of a questionnaire students could answer on their own, including an assessment of self-esteem and suicidal ideation. Its general objective was to study psychological malaise and well-being through everyday Stressors and compensators associated with the father's physical absence due to international migration. Schools were selected through intentional, non-probabilistic sampling to ensure that semi-urban and rural schools were equally represented. Students were assured that the information provided would be confidential. The data were statistically analyzed using SPSS, version 10. Results: The age range of the sample was 11 to 14 years (mean=11.51, s=.72), 51% of the participants were male and 34.2% were the children of migrant fathers. In addition to studying, 37.7% contributed to the family income. The group mean of depressive symptomatology was 19.3 (s=7.6) and no differences were observed between males and females or between the children of migrant fathers and non-migrant fathers. The scale had a .76 Alpha Cronbach, which increases to .84 without the positive affect items that had a low correlation with the total scale. Although factor analysis yielded four factors, the configuration of the latter failed to confirm the factorial structure reported by Radloff. A total of 15.8% of the sample was located above the cut-off point (the mean plus a standard deviation =27). Being the child of an emigrant was not linked to greater depressive symptomatology, although having to work outside the home was: a higher proportion of adolescents who, in addition to studying, engaged in economic activities to contribute to the family income, had greater depressive symptomatology. At the end, an analysis was carried out of the Pearson correlation between the overall CES-D score and Coopersmith self-esteem scale, yielding a negative significant correlation ($r=-0.341$, $p< 0.01$), meaning that the higher the level of depression, the lower a person's self-esteem. Analysis of depressive symptomatology with suicidal ideation showed a positive, significant correlation through the Pearson coefficient ($r=.501$, $p<0.001$): the greater the symptomatology, the greater the presence of suicidal ideation. Discussion: Results confirm that the CES-D is a valid, reliable scale for measuring depressive symptomatology in this population. Further research, however, is required on the behavior of items concerning the positive affect factor, since its low correlation with the total scale and its lack of correlation with negative affect, psychosomatic complaints and interpersonal relations factors indicate that, as suggested by Iwata and collaborators (1998), these items are not measuring depressive symptomatology in this population. Two results call for further comment: 1. In this study, it was not the offspring of migrant fathers who obtained high scores in depressive symptomatology, but rather 2. teenagers who studied and worked who obtained scores of "possible cases" of depression. These results can be analyzed from various angles and as a consequence of the interaction of various factors: 1. Although paternal absence due to international migration has been cited as a predictive factor of emotional malaise, particularly among young teenagers, the culture of migration present in these communities may restrict its negative influence by regarding it as "normal"; 2. it is possible that certain characteristics of the mothers of these teenagers may act as a resilience factor to paternal absence and 3) regardless of whether or not teenagers lived with their fathers, there are certain structural situations that directly influence their emotional malaise, such as the conditions of everyday poverty in which they are raised and the lack of real opportunities for development (such as employment, agricultural training, and middle school education), which affect all the population sectors of these rural communities. It is essential to determine the specific importance of each of the various situations outlined here and their interaction in order to obtain the elements that will enable health professionals to deal with the emotional malaise detected in these adolescents as well as providing a satisfactory response to the structural conditions in which they are raised.

Otherwise, "going North" will continue to be their preferred life choice and Mexico will continue to lose a key element of its productive development: its youth population
SN - 0185-3325
AD - (Aguilera-Guzman) Instituto Nacional de Psiquiatria, Dir. Invest. Epidemiol./Psicosocial., Calzada Mexico-Xochimilco 101, Tlalpan, 14370, Mexico, D.F., Mexico (Aguilera-Guzman, Carreno Garcia, Juarez Garcia) Dir. Invest. Epidemiol./Psicosocial
ER -

TY - JOUR
ID - 3857
T1 - School Function Assessment: Performance of Icelandic students with special needs
A1 - Egilson,S.T.
A1 - Coster,W.J.
Y1 - 2004///
N1 - 2004518320
English
Journal: Article
KW - EMBASE
KW - Academic Achievement
KW - Adaptation
KW - article
KW - Awareness
KW - behavior disorder
KW - Child
KW - cognitive defect
KW - controlled study
KW - Cooperation
KW - disability
KW - ethnology
KW - Female
KW - functional assessment
KW - Human
KW - Iceland
KW - major clinical study
KW - Male
KW - meal
KW - medical instrumentation
KW - physical disability/co [Complication]
KW - play
KW - scoring system
KW - Social Interaction
RP - NOT IN FILE
SP - 163
EP - 170
JF - Scandinavian Journal of Occupational Therapy
VL - 11
IS - 4
CY - Norway
N2 - The purpose of this study was to determine the performance of a sample of Icelandic students, aged 6 to 12 years, with various impairments, on the School Function Assessment (SFA). The SFA was administered to 75 Icelandic students with identified special needs, 35 with physical impairments and 40 with cognitive/behavioral impairments. Both diagnostic groups demonstrated most participation in mealtime/snack time but had low scores on the school playground. Differences were found between the two study groups on all three parts of the instrument, with higher performance and participation scores for the students with cognitive/behavioral impairments. The findings raise the awareness of some of the challenges encountered by Icelandic students with special needs and indicate the importance of adapting Icelandic school settings to promote social participation of all students. 2004 Taylor & Francis

SN - 1103-8128
AD - (Egilson) University of Akureyri, Department of Health Sciences, Occupational Therapy Program, Solborg vio Nordurslod, IS-600 Akureyri, Iceland (Coster) Boston University, Sargent College, Dept. of Rehabilitation Sciences, Boston, MA, United States
ER -

TY - JOUR
ID - 3858
T1 - Measuring by hand. Arsenic picked up from the playground
A1 - Fields,S.
Y1 - 2004///
N1 - 2004447137
English
Journal: Note
KW - EMBASE
KW - carcinogenic activity
KW - Child
KW - Environmental Exposure
KW - Hand
KW - hand washing
KW - health hazard
KW - Human
KW - Inhalation
KW - leaching
KW - note
KW - priority journal
KW - Skin Absorption
KW - soil analysis
KW - statistical significance
KW - Wood
KW - arsenic/to [Drug Toxicity]
KW - arsenic derivative/to [Drug Toxicity]
KW - chromium/to [Drug Toxicity]
RP - NOT IN FILE
SP - A824
EP - A825
JF - Environmental Health Perspectives
JA - Environ Health Perspect
VL - 112
IS - 14
CY - United States
SN - 0091-6765
ER -

TY - JOUR
ID - 3859
T1 - Lung cancer epidemiology and risk factors in Asia and Africa
A1 - Lam,W.K.
A1 - White,N.W.
A1 - Chan-Yeung,M.M.
Y1 - 2004///
N1 - 2004388142
English
Journal: Review
KW - EMBASE
KW - Adolescent
KW - Adult

KW - Africa
KW - Aged
KW - Air Pollution
KW - Asia
KW - burn
KW - cancer epidemiology
KW - cancer incidence
KW - cancer prevention
KW - cancer risk
KW - cancer susceptibility
KW - China
KW - Cooking
KW - Environmental Exposure
KW - epidemic
KW - fat intake
KW - Female
KW - Food Intake
KW - food preservation
KW - Fruit
KW - genetic predisposition
KW - Government
KW - health care organization
KW - health care personnel
KW - Human
KW - industrialization
KW - lung cancer/ep [Epidemiology]
KW - lung cancer/et [Etiology]
KW - lung cancer/pc [Prevention]
KW - lung carcinogenesis
KW - major clinical study
KW - Male
KW - Microsporum canis
KW - Mycobacterium tuberculosis
KW - Occupational Exposure
KW - Prediction
KW - priority journal
KW - radiation exposure
KW - review
KW - risk factor
KW - school child
KW - Smoking
KW - Tobacco
KW - vapor
KW - vegetable
KW - Wart virus
KW - coal
KW - oil
KW - radon
KW - tobacco smoke
RP - NOT IN FILE
SP - 1045
EP - 1057
JF - International Journal of Tuberculosis and Lung Disease
VL - 8
IS - 9
CY - France

N2 - In industrialised countries, lung cancer is the most common form of cancer among males and it is growing among females. For both sexes, rates reflect smoking behaviours. The pattern appears to be different in Asia, particularly in China, where lung cancer rates in men reflect high smoking rates but high rates among non-smoking women appear to be related to other factors. The incidence of lung cancer is low in most African countries, but it is increasing. In addition to tobacco smoking, a number of etiological factors have been identified for lung cancer: indoor exposure to environmental tobacco smoke, cooking oil vapour, coal burning, or radon, outdoor air pollution and occupational exposure to asbestos and other carcinogens. Recent studies have shown that dietary factors may be important, with high consumption of vegetables and fruits being protective while preserved food and fatty food are harmful, and certain infections such as Mycobacterium tuberculosis, human papilloma virus and Microsporum canis are associated with a high risk of lung cancer. Among non-smokers, the probable role of genetic predisposition in lung cancer by increasing the individual's susceptibility to environmental carcinogens is currently being studied actively. As the single most important cause for lung cancer is tobacco smoke and, with increased sales, a major epidemic is predicted for both Asia and Africa, all health care professionals, government health authorities and national and international health organisations must join in a concerted effort against tobacco
SN - 1027-3719
AD - (Lam, Chan-Yeung) Department of Medicine, Div. of Resp. and Critical Care Med., University of Hong Kong, Hong Kong, Hong Kong (White) Department of Medicine, UCT Lung Institute, Univ. Cape Town/Groote Schuur Hosp., Cape Town, South Africa
ER -

TY - JOUR
ID - 3860
T1 - Disruptive classroom behavior in an Amish school-age child with muscular dystrophy
A1 - Stein,M.T.
A1 - Pachter,L.M.
A1 - Schwartz,L.
A1 - Taras,H.
Y1 - 2004///
N1 - 2004386664
English
Journal: Article
KW - EMBASE
KW - Academic Achievement
KW - article
KW - Attention Deficit Disorder
KW - behavior disorder
KW - Case Report
KW - Duchenne muscular dystrophy
KW - Human
KW - Male
KW - muscle weakness
KW - parental behavior
KW - priority journal
KW - Punishment
KW - Reinforcement
KW - school child
KW - teacher
RP - NOT IN FILE
SP - 280
EP - 284
JF - Journal of Developmental and Behavioral Pediatrics
VL - 25
IS - 4
CY - United States
N2 - Will is an Amish school-aged boy with Duchenne muscular dystrophy. After reading about attention-deficit hyperactivity disorder (ADHD) in the local newspaper, his mother asked Will's pediatrician, "Do you

think my son has this (condition)?" She noted that Will fidgets in different settings and has difficulty focusing on his work when in school. She also reported that his teacher in the Amish school does not want him in her classroom because he is disruptive and noncompliant. The teacher tried incentives to encourage Will to complete his schoolwork but without success. Subsequently, his teacher prevented Will from participating in recess activities; at other times, she sent him home as punishment for noncompliance in the classroom. During the past week, Will did not attend school while his teacher sent assignments home with his sister. Will's mother reported that he seemed to be happy with a normal appetite and sleep pattern. She was not aware of any behavior problems with other children. Will's father, who also attended the pediatric appointment, added that he did not feel that the teacher was patient with Will. He stated that Will has friends, but may have a difficult time keeping up with them due to his physical weakness secondary to muscular dystrophy. He also believed that due to motor limitations, writing was difficult for Will. The parents did not provide consequences for Will's insistence on not attending school nor did they positively reward him for school attendance. When at home, Will did his work and kept to himself. Will was quiet and motionless in his seat. He stated that he liked school and playing with friends and that the schoolwork was not too difficult. Copyright 2004 by Lippincott Williams & Wilkins, Inc
SN - 0196-206X
AD - (Stein) Department of Pediatrics, University of California San Diego, Children's Hospital San Diego, San Diego, CA, United States (Pachter) Dept. of Pediatrics and Anthropology, Univ. Connecticut School of Medicine, Farmington, CT, United States (Schwartz) Department of Psychology, Case Western Reserve University, Cleveland, OH, United States (Taras) Department of Pediatrics, University of California San Diego, San Diego School Districts, San Diego, CA, United States
ER -

TY - JOUR
ID - 3861
T1 - Auditory discrimination training for the treatment of tinnitus
A1 - Flor,H.
A1 - Hoffmann,D.
A1 - Struve,M.
A1 - Diesch,E.
Y1 - 2004///
N1 - 2004322542
English
Journal: Article
KW - EMBASE
KW - article
KW - auditory cortex
KW - auditory discrimination
KW - auditory system
KW - clinical article
KW - controlled study
KW - disease severity
KW - emotion
KW - Female
KW - Human
KW - Infant
KW - Male
KW - Prediction
KW - preschool child
KW - statistical significance
KW - tinnitus
KW - tonotopy
RP - NOT IN FILE
SP - 113
EP - 120
JF - Applied Psychophysiology Biofeedback
VL - 29

IS - 2
CY - United States
N2 - This paper presents a series of 12 cases of chronic tinnitus patients who participated in 4 weeks of auditory discrimination training either close to or far removed from the tinnitus frequency. The training was based on the assumption that tinnitus is related to a shift of the representation of the tinnitus frequency in auditory cortex outside of the normal tonotopic map and that training close to but not removed from the tinnitus frequency should result in a reduction in the severity of the tinnitus. Tinnitus severity was measured 4 times per day during the entire treatment and other tinnitus-related variables were assessed 1 week before and 1 month posttreatment. The comparison of the training close to as compared to remote from the tinnitus frequency did not yield a statistically significant difference. However, a post hoc analysis revealed that patients who engaged in regular training as compared to those who practiced irregularly were significantly more successful in reducing tinnitus severity independent of the trained frequencies. Treatment success was best predicted by days of training and general activity levels. The data suggest that auditory dicrimintion training shows a dose response effect irrespective of training location and that treatment success is also related to psychological variables. For more substantial changes in multiple variables an extended training period with additional consideration of emotional variables would be necessary. In addition, controls for nonspecific training effects need to be implemented
SN - 1090-0586
AD - (Flor, Hoffmann, Struve, Diesch) Dept. of Cogn. and Clin. Neurosci., University of Heidelberg, Central Institute of Mental Health, Mannheim, Germany (Flor) Dept. of Cogn. and Clin. Neurosci., University of Heidelberg, Central Institute of Mental Health, Quadrat J5, 68159 Mannheim, Germany
ER -

TY - JOUR
ID - 3862
T1 - Interinstrument consistency of the Yamax Digi-Walker pedometer in elementary school-aged children
A1 - Barfield,J.P.
A1 - Rowe,D.A.
A1 - Michael,T.J.
Y1 - 2004///
N1 - 2004296342
English
Journal: Article
KW - EMBASE
KW - article
KW - correlation coefficient
KW - cost
KW - diagnostic test
KW - diagnostic value
KW - Female
KW - Human
KW - instrument
KW - Male
KW - Measurement
KW - normal human
KW - pedometer
KW - Physical Activity
KW - Physical Education
KW - reliability
KW - school child
KW - time series analysis
RP - NOT IN FILE
SP - 109
EP - 116
JF - Measurement in Physical Education and Exercise Science
VL - 8
IS - 2
CY - United States

N2 - A pedometer is a practical, inexpensive tool used to measure physical activity. Bassett et al. (1996) found that interinstrument consistency of the Yamax Digi-Walker was higher than four other pedometers when measuring distance walked in adults. The purpose of this study was to investigate the interinstrument consistency of the Yamax pedometer in children. Seventy-one children in Grades 3 to 5 wore one Digi-Walker on each hip during school hours for 1 week. Time and counts data were recorded separately for (a) regular classroom, (b) recess, and (c) physical education class times. Interinstrument reliability for total counts was estimated using one-way analysis of variance intraclass coefficients, adjusted for a single pedometer. Intraclass correlation coefficients were .96 for the whole week, .94 for classroom time, .98 for recess time, and .92 for physical education time. For applied use and research with children, the Yamax Digi-Walker provides a reliable estimate of physical activity
SN - 1091-367X
AD - (Barfield) Department of Physical Education, Emory and Henry College, Emory, VA, United States (Rowe) Dept. of Exercise and Sport Science, East Carolina University (Michael) Dept. of Hlth., Phys. Educ./Recr., Western Michigan University (Barfield) Department of Physical Education, Emory and Henry College, P.O. Box 947, Emory, VA 24327, United States
ER -

TY - JOUR
ID - 3863
T1 - Psychosocial correlates of physical activity in children-A study of relationships when children have similar opportunities to be active
A1 - Welk,G.J.
A1 - Schaben,J.A.
Y1 - 2004///
N1 - 2004296339
English
Journal: Article
KW - EMBASE
KW - accelerometer
KW - article
KW - athlete
KW - Child Behavior
KW - Competence
KW - correlation analysis
KW - Female
KW - fitness
KW - Human
KW - human relation
KW - Male
KW - normal human
KW - Physical Activity
KW - rating scale
KW - school child
KW - Self Concept
KW - social psychology
RP - NOT IN FILE
SP - 63
EP - 81
JF - Measurement in Physical Education and Exercise Science
VL - 8
IS - 2
CY - United States
N2 - Children's daily physical activity patterns are influenced by many factors outside of their immediate control (e.g., school, parents' availability, time allowed outdoors). Because all children do not have an equal opportunity to participate in physical activity, investigating the relationship of psychosocial variables and actual voluntary activity has been problematic. Therefore, the purpose of this study was to determine the relationships among psychosocial correlates and physical activity levels when children have an equal and optimal opportunity

to be active. Participants were 25 children (ages 10-12) who participated in a summer fitness camp. The structured and repeated nature of the camp provided a unique opportunity to study variability in children's activity patterns over an extended period of time. Participants completed Harter's Perceived Athletic Competence Scale, the Children's Attraction to Physical Activity Scale, and the Children and Youth Physical Self-Perception Profile during the first 2 weeks of the camp. They wore an accelerometry-based activity monitor for 3 subsequent weeks. Physical activity was evaluated by computing T scores for their activity levels for distinct time periods and then averaging these T scores across the 3 weeks. Correlations between the activity levels and the Perceived Athletic Competence Scale were high for all 3 weeks (mean r =.72); however, correlations were consistently weak with the other correlate measures. The average pairwise correlation for the physical activity scores across the 3 weeks of the camp was high (r = .72), indicating that some children seek out ways to be active whereas others consistently choose to be less active. The consistently strong correlations with the Perceived Athletic Competence construct suggest that this may be an important mediator of children's physical activity behavior
SN - 1091-367X
AD - (Welk, Schaben) Dept. of Hlth. and Human Performance, Iowa State University, Ames, IA, United States (Welk) Dept. of Hlth. and Human Performance, 235 Forker Building, Iowa State University, Ames, IA 50011, United States
ER -

TY - JOUR
ID - 3864
T1 - Bronchial reactivity in students from moisture and mold-damaged schools: Changes in relation to changes in exposure
A1 - Immonen,J.
A1 - Taskinen,T.
A1 - Pekkanen,J.
A1 - Korppi,M.
Y1 - 2004///
N1 - 2004286895
English
Journal: Article
KW - EMBASE
KW - Adolescent
KW - article
KW - Asthma/ep [Epidemiology]
KW - bronchus reactivity
KW - controlled study
KW - Environmental Exposure
KW - Exercise Test
KW - Female
KW - Human
KW - major clinical study
KW - Male
KW - moisture
KW - mould
KW - priority journal
KW - provocation test
KW - risk factor
KW - school
KW - school child
KW - Spirometry
RP - NOT IN FILE
SP - 116
EP - 125
JF - Pediatric Asthma, Allergy and Immunology
VL - 17
IS - 2

CY - United States
N2 - There is epidemiological and preliminary clinical evidence that exposure to moisture and mold growth is associated with wheezing and asthma in children. Lung function and challenge tests may find obstructive changes in the airways before clinically evident symptoms. The aim of the study was to evaluate lung function and bronchial reactivity in school children in a follow-up setting, and relate them to changes in mold exposure over a 3-year observation period. We performed flow-volume spirometry and an exercise challenge test by outdoor free running 3 years apart to the same 139 students from moisture-problem and control schools. The subjects were classified into four exposure groups: exposure continued, eased, started, and no exposure. There were no significant differences in spirometry or in the exercise challenge test among the children in the four different exposure groups. However, there was a trend that, on average, bronchial reactivity increased when exposure started, and decreased when exposure eased. Spirometry and exercise challenge offer no benefits over symptoms in the follow-up of symptomatic children exposed to moisture and/or molds
SN - 0883-1874
AD - (Immonen, Pekkanen) National Public Health Institute, Kuopio, Finland (Taskinen) Kiuruvesi Health Care Center, Kiuruvesi, Finland (Korppi) Department of Pediatrics, Kuopio University Hospital, Kuopio, Finland (Immonen) National Public Health Institute, P.O.B. 95, FIN-70701 Kuopio, Finland
ER -

TY - JOUR
ID - 3865
T1 - Effects of a three-year intervention on children's physical activity from age 4 to 7
A1 - Saakslahti,A.
A1 - Numminen,P.
A1 - Salo,P.
A1 - Tuominen,J.
A1 - Helenius,H.
A1 - Valimaki,I.
Y1 - 2004///
N1 - 2004233911
English
Journal: Article
KW - EMBASE
KW - article
KW - Child
KW - child growth
KW - child health care
KW - child parent relation
KW - childhood
KW - controlled study
KW - Family
KW - Finland
KW - Health Promotion
KW - Human
KW - normal human
KW - Physical Activity
KW - play
RP - NOT IN FILE
SP - 167
EP - 180
JF - Pediatric Exercise Science
JA - Pediatr Exerc Sci
VL - 16
IS - 2
CY - United States
N2 - This study focused on the physical activities of 228 children over 3 years. Children were divided into control (n = 112) and intervention (n = 116) groups. Parents of intervention-group children received information and concrete suggestions on how, when, and where to encourage their child's physical activity. Children in the

intervention group spent more time playing outdoors (p =.041) than did children in the control group, and play in the high-activity category increased with age (p <.001), whereas no change occurred in the control group. Our study showed that children's physical activity could be increased via family-based intervention
SN - 0899-8493
AD - (Saakslahti, Numminen) Department of Physical Education, University of Jyvaskyla, P.O. Box 35, FIN-40014, Jyvaskylan yliopisto, Finland (Salo, Valimaki) Cardiorespiratory Research Centre, Department of Pediatrics, University of Turku, Kiinamyllynkatu 10, FIN-20520 Turku, Finland (Tuominen, Helenius) Department of Biostatistics, University of Turku, Lemminkaisenkatu 1, FIN-20520 Turku, Finland
ER -

TY - JOUR
ID - 3866
T1 - Chicken consumption is a newly identified risk factor for sporadic Salmonella enterica serotype enteritidis infections in the United States: A case-control study in FoodNet sites
A1 - Kimura,A.C.
A1 - Reddy,V.
A1 - Marcus,R.
A1 - Cieslak,P.R.
A1 - Mohle-Boetani,J.C.
A1 - Kassenborg,H.D.
A1 - Segler,S.D.
A1 - Hardnett,F.P.
A1 - Barrett,T.
A1 - Swerdlow,D.L.
Y1 - 2004///
N1 - 2004191240
English
Journal: Article
KW - EMBASE
KW - Adolescent
KW - Adult
KW - article
KW - chicken
KW - controlled study
KW - disease association
KW - Education
KW - enteritis/ep [Epidemiology]
KW - enteritis/et [Etiology]
KW - Female
KW - Food Handling
KW - Food Intake
KW - food safety
KW - Human
KW - major clinical study
KW - Male
KW - Multivariate Analysis
KW - population research
KW - priority journal
KW - prophylaxis
KW - risk factor
KW - Salmonella enterica
KW - salmonellosis/ep [Epidemiology]
KW - salmonellosis/et [Etiology]
KW - school child
KW - United States
RP - NOT IN FILE
SP - S244

EP - S252
JF - Clinical Infectious Diseases
JA - Clin Infect Dis
VL - 38
IS - SUPPL. 3
CY - United States
N2 - The sources of sporadic Salmonella enterica serotype Enteritidis (SE) infections in the United States are unclear. To determine risk factors for sporadic SE infection, we conducted a population-based case-control study in 5 Foodborne Disease Active Surveillance Network surveillance areas. During the 12-month study, 396 cases of SE infection were ascertained. Among the 182 case patients and 345 controls, SE infection was univariately associated with international travel (matched odds ratio [MOR], 61; 95% confidence interval [CI], 8-447), eating undercooked eggs (MOR, 2.2; 95% CI, 1-5), and eating chicken prepared outside of the home (MOR, 2.2; 95% CI, 1.3-3.4). Multivariate analysis revealed that eating chicken outside of the home remained the only significant risk factor for illness (MOR, 2.0; 95% CI, 1.1-3.6). Chicken consumption has not previously been identified in the United States as a risk factor for SE infection. Measures to prevent SE infections include educating consumers and food handlers about food safety and interventions to decrease contamination of eggs and poultry
SN - 1058-4838
AD - (Kimura, Reddy, Barrett, Swerdlow) Biostatist. and Info. Mgmt. Branch, Div. of Bact. and Mycotic Disease, Centers for Dis. Contr. and Prev., Atlanta, GA, United States (Marcus) Foodborne and Diarrheal Dis. Branch, Div. of Bact. and Mycotic Disease, Centers for Dis. Contr. and Prev., Atlanta, GA, United States (Cieslak) Georgia Emerging Infections Program, Atlanta, GA, United States (Mohle-Boetani) CT Emerging Infections Program, New Haven, CT, United States (Kassenborg) Health Division, Oregon Department of Human Services, Portland, OR, United States (Segler) Div. of Communicable Disease Control, CA Department of Health Services, Berkeley, CA, United States (Hardnett) Acute Disease Epidemiology Section, Minnesota Department of Health, Minneapolis, MN, United States (Hardnett) Biostatist. and Info. Mgmt. Branch, Div. of Bact. and Mycotic Diseases, Centers for Dis. Contr. and Prev., Atlanta, GA 30333, United States
ER -

TY - JOUR
ID - 3867
T1 - Physical Activity Levels of Students with Mental Retardation and Students Without Disabilities
A1 - Faison-Hodge,J.
A1 - Porretta,D.L.
Y1 - 2004///
N1 - 2004170789
English
Journal: Article
KW - EMBASE
KW - article
KW - cardiopulmonary hemodynamics
KW - comparative study
KW - controlled study
KW - disability
KW - Female
KW - fitness
KW - functional assessment
KW - Human
KW - leisure
KW - major clinical study
KW - Male
KW - mental deficiency/di [Diagnosis]
KW - Physical Activity
KW - Physical Education
KW - primary school
KW - recess
KW - school child

KW - student
KW - System for Observing Fitness Instructional Time
KW - validation process
RP - NOT IN FILE
SP - 139
EP - 152
JF - Adapted Physical Activity Quarterly
JA - Adapt Phys Act Q
VL - 21
IS - 2
CY - United States
N2 - This study compared physical education and recess physical activity levels of elementary school age students with mild mental retardation (MR) and students without disabilities who possessed either high or low cardiorespiratory fitness. For this study, the System for Observing Fitness Instructional Time (SOFIT), a measure of physical activity, was validated for students with mild MR. A significant difference for moderate to vigorous physical activity was obtained between settings. Findings suggest that students with MR and those without disabilities were more active during recess than during physical education. Students with mild MR and those with low cardiorespiratory fitness performed similarly in both the physical education and recess settings
SN - 0736-5829
AD - (Faison-Hodge) Capital University, Columbus, OH, United States (Porretta) Ohio State University, Columbus, OH, United States (Faison-Hodge) Dept. of Health and Sport Sciences, Capital University, Columbus, OH, United States (Porretta) Sch. of Phys. Activ. and Educ. Serv., Ohio State University, Columbus, OH, United States
ER -

TY - JOUR
ID - 3868
T1 - Compliance with follow-up and the informative value of diagnostic whole-body scan patients with differentiated thyroid carcinoma given recombinant human TSH
A1 - Cohen,O.
A1 - Dabhi,S.
A1 - Karasik,A.
A1 - Zwas,S.Z.
Y1 - 2004///
N1 - 2004153575
English
Journal: Article
KW - EMBASE
KW - Adolescent
KW - Adult
KW - article
KW - cancer radiotherapy
KW - Child
KW - clinical article
KW - clinical trial
KW - controlled clinical trial
KW - controlled study
KW - data analysis
KW - diagnostic value
KW - Female
KW - follow up
KW - headache/si [Side Effect]
KW - Human
KW - immunoassay
KW - Interview
KW - Male
KW - medical record

KW - Patient Compliance
KW - patient monitoring
KW - patient referral
KW - priority journal
KW - questionnaire
KW - Radiography
KW - thyroglobulin blood level
KW - thyroid carcinoma/di [Diagnosis]
KW - thyroid carcinoma/dt [Drug Therapy]
KW - thyroid carcinoma/rt [Radiotherapy]
KW - thyroid carcinoma/su [Surgery]
KW - thyroidectomy
KW - tumor differentiation
KW - whole body scintiscanning
KW - iodine 131/cb [Drug Combination]
KW - iodine 131/do [Drug Dose]
KW - iodine 131/po [Oral Drug Administration]
KW - radioactive iodine/cb [Drug Combination]
KW - radioactive iodine/do [Drug Dose]
KW - radioactive iodine/dt [Drug Therapy]
KW - radioactive iodine/po [Oral Drug Administration]
KW - recombinant thyrotropin/ae [Adverse Drug Reaction]
KW - recombinant thyrotropin/ct [Clinical Trial]
KW - recombinant thyrotropin/cb [Drug Combination]
KW - recombinant thyrotropin/do [Drug Dose]
KW - recombinant thyrotropin/im [Intramuscular Drug Administration]
KW - thyroglobulin/ec [Endogenous Compound]
KW - thyroid hormone/ec [Endogenous Compound]
RP - NOT IN FILE
SP - 285
EP - 290
JF - European Journal of Endocrinology
VL - 150
IS - 3
CY - United Kingdom
N2 - Objective: Protocols for monitoring patients with differentiated thyroid cancer (DTC) include measurement of serum Tg and, for most patients, whole-body scan (WBS) with low radioiodine activities ('diagnostic' WBS). Recently, recombinant human thyroid-stimulating hormone (rhTSH) has become available to provide the TSH stimulation necessary for these procedures, whilst avoiding thyroid hormone withdrawal and hypothyroid complications. In addition, the inclusion of diagnostic WBS in DTC follow-up has recently become controversial. We have assessed the compliance with withdrawal-aided monitoring and the informative value of diagnostic WBS in consecutive tertiary referral center patients. Design: Forty-eight patients received rhTSH (0.9 mg) in two consecutive daily injections, with radioiodine administration 24 h, diagnostic WBS 48 h, and serum Tg testing prior to and 72 h later. Methods: Compliance with withdrawal-aided monitoring was assessed with a questionnaire provided by the referring physician, patient record analysis, and patient interview. The informative value of diagnostic WBS was assessed by comparing findings against serum Tg measurements in light of physical and other radiological examinations. Results: Forty of the forty-eight patients were female, the mean age was 43.9 years and the median follow-up from diagnosis was 4.5 years (range 1-19 years). Twenty-seven (56%) patients were compliant and 12 (25%) were non-compliant; compliance was not known in nine. Of 17 patients with clinically suspicious or significant findings on any available modality, four had uptake outside the thyroid bed on WBS but stimulated Tg <2.5 ng/ml on immunometric assay, while five had a negative WBS with serum Tg >2.5 ng/ml. Conclusions: Thyroid hormone withdrawal substantially impairs, and rhTSH administration substantially promotes, compliance with DTC monitoring. rhTSH-aided WBS is informative and should be included in the follow-up of unsclected patients with DTC. 2004 Society of the European Journal of Endocrinology
SN - 0804-4643

AD - (Zwas) Nuclear Medicine Department, Chaim Sheba Medical Center, Tel Hashomer 52621, Israel (Cohen, Dabhi, Karasik) Institute of Endocrinology, Chaim Sheba Medical Center, Tel Hashomer 52621, Israel
ER -

TY - JOUR
ID - 3869
T1 - Correlates of Physical Activity in a Cypriot Sample of Sixth-Grade Children
A1 - Loucaides,C.A.
A1 - Chedzoy,S.M.
A1 - Bennett,N.
A1 - Walshe,K.
Y1 - 2004///
N1 - 2004077935
English
Journal: Article
KW - EMBASE
KW - article
KW - Exercise
KW - Female
KW - gender
KW - Human
KW - major clinical study
KW - Male
KW - Physical Activity
KW - play
KW - school child
KW - Self Concept
KW - Self Report
KW - sport
KW - summer
KW - winter
RP - NOT IN FILE
SP - 25
EP - 36
JF - Pediatric Exercise Science
JA - Pediatr Exerc Sci
VL - 16
IS - 1
CY - United States
N2 - This study examined the association between self-reported physical activity and a number of potential correlates in a sample of 256 Grade 6 children. Physical activity was assessed in both summer and winter, and children and parents completed questionnaires assessing potential correlates of physical activity. Analyses revealed that gender, time spent playing outside, self-efficacy in overcoming barriers, and number of items of exercise equipment at home were variables associated with physical activity in both seasons. School location was a variable associated with physical activity only in the summer, whereas private-lesson attendance, sports-club attendance, and best friend's physical activity were variables associated with physical activity only in winter. Variance explained in physical activity were 42% and 51% in winter and summer, respectively
SN - 0899-8493
AD - (Loucaides) Laniteio Elementary School, Limassol, Cyprus (Chedzoy, Bennett, Walshe) Sch. of Educ. and Lifelong Learning, University of Exeter, Exeter, EX1 2LU, United Kingdom
ER -

TY - JOUR
ID - 3870
T1 - The etiology, pathophysiology, and management of otitis media with effusion
A1 - Butler,C.C.
A1 - Williams,R.G.

Y1 - 2003///
N1 - 2004470941
English
Journal: Review
KW - EMBASE
KW - adenoidectomy
KW - adenotonsillectomy
KW - Audiometry
KW - bacterial virulence
KW - Breast Feeding
KW - Child
KW - clinical trial
KW - Day Care
KW - environmental factor
KW - epidemiological data
KW - family history
KW - gastroesophageal reflux
KW - hearing aid
KW - Human
KW - Immunization
KW - Infant
KW - Language Development
KW - nonhuman
KW - otoscopy
KW - pathophysiology
KW - patient referral
KW - Polymerase Chain Reaction
KW - population research
KW - practice guideline
KW - Prevalence
KW - protective equipment
KW - review
KW - risk factor
KW - screening test
KW - secretory otitis media/di [Diagnosis]
KW - secretory otitis media/dt [Drug Therapy]
KW - secretory otitis media/ep [Epidemiology]
KW - secretory otitis media/et [Etiology]
KW - secretory otitis media/pc [Prevention]
KW - secretory otitis media/su [Surgery]
KW - Smoking
KW - Swimming
KW - Treatment Planning
KW - tympanometry
KW - tympanostomy tube
KW - virus virulence
KW - amoxicillin/ct [Clinical Trial]
KW - amoxicillin/cb [Drug Combination]
KW - amoxicillin/cm [Drug Comparison]
KW - amoxicillin/dt [Drug Therapy]
KW - antibiotic agent/ct [Clinical Trial]
KW - antibiotic agent/cb [Drug Combination]
KW - antibiotic agent/cm [Drug Comparison]
KW - antibiotic agent/dt [Drug Therapy]
KW - antibiotic agent/po [Oral Drug Administration]
KW - antibiotic agent/tp [Topical Drug Administration]
KW - antihistaminic agent/dt [Drug Therapy]

KW - carbocisteine/ct [Clinical Trial]
KW - carbocisteine/dt [Drug Therapy]
KW - cytokine/ec [Endogenous Compound]
KW - decongestive agent/ct [Clinical Trial]
KW - decongestive agent/dt [Drug Therapy]
KW - decongestive agent/tp [Topical Drug Administration]
KW - ear drops/dt [Drug Therapy]
KW - ear drops/tp [Topical Drug Administration]
KW - Haemophilus vaccine/dt [Drug Therapy]
KW - influenza vaccine/ct [Clinical Trial]
KW - influenza vaccine/dt [Drug Therapy]
KW - mucin/ec [Endogenous Compound]
KW - nose drops/ct [Clinical Trial]
KW - nose drops/dt [Drug Therapy]
KW - nose drops/tp [Topical Drug Administration]
KW - Pneumococcus vaccine/ct [Clinical Trial]
KW - Pneumococcus vaccine/dt [Drug Therapy]
KW - prednisolone/ct [Clinical Trial]
KW - prednisolone/cb [Drug Combination]
KW - prednisolone/cm [Drug Comparison]
KW - prednisolone/dt [Drug Therapy]
KW - prednisolone/po [Oral Drug Administration]
KW - steroid/ct [Clinical Trial]
KW - steroid/cb [Drug Combination]
KW - steroid/cm [Drug Comparison]
KW - steroid/dt [Drug Therapy]
KW - steroid/po [Oral Drug Administration]
KW - xylometazoline/ct [Clinical Trial]
KW - xylometazoline/dt [Drug Therapy]
KW - xylometazoline/tp [Topical Drug Administration]
RP - NOT IN FILE
SP - 205
EP - 212
JF - Current Infectious Disease Reports
VL - 5
IS - 3
CY - United Kingdom
N2 - Otitis media with effusion (OME) is a common and important condition that may result in developmental delay in children, and significant health care resources are devoted to its management. Newer techniques including polymerase chain reaction are implicating organisms not previously considered important in etiology. The role of gastroesophageal reflux as a cause of OME is likely to receive greater research attention. Regarding prevention, more is being learned about potentially modifiable risk factors such as environmental smoke, care outside the home, and breast feeding. Although immunization may to play a role in the future, existing evidence suggests that the general population of children should not be immunized in order to prevent OME. Several major studies have recently added to the understanding of epidemiology and management. Large trials in the United States, the Netherlands, and the UK suggest that OME is not an appropriate condition to include in a screening program. In addition, the advantages of early treatment with ventilation tubes over watchful waiting in terms of language development tend be modest and diminish by about 18 months. Treatment with hearing aids should be further evaluated. The search for effective medical management continues, and better ways are being identified of targeting interventions to those children with OME who are most likely to benefit. Copyright 2003 by Current Science Inc
SN - 1523-3847
AD - (Butler) Department of General Practice, Univ. of Wales College of Medicine, Llanedeyrn Health Centre, Cardiff CF23 9PN, United Kingdom
ER -

TY - JOUR

ID - 3871
T1 - Connection between physical activity and smoking habits of teenagers
A1 - Nistal,Hernandez P.
A1 - Prieto Saborit,J.A.
A1 - Del Valle,Soto M.
A1 - Gonzalez,Diez,V
Y1 - 2003///
N1 - 2004181021
Spanish
Journal: Article
KW - EMBASE
KW - adolescence
KW - Adolescent
KW - Age Distribution
KW - article
KW - controlled study
KW - correlation analysis
KW - Exercise
KW - Family
KW - Female
KW - frequency analysis
KW - health statistics
KW - Health Status
KW - health survey
KW - household
KW - Human
KW - Male
KW - Physical Activity
KW - Physical Education
KW - population research
KW - school child
KW - Sex Ratio
KW - Smoking
KW - smoking habit
KW - statistical significance
KW - student
KW - Tobacco
KW - tobacco dependence/ep [Epidemiology]
KW - Training
RP - NOT IN FILE
SP - 397
EP - 403
JF - Archivos de Medicina del Deporte
VL - 20
IS - 97
CY - Spain
N2 - The aim of this research is to learn the percentage of teenagers aged amond 12 to 16, that regularly do some kind of physical activity and sedentary, to learn the percentage of smokers and to establish a connection between the smoking habit and the different types of physical exercise (individual, collective, aerobic and anaerobic), as well as other variables such as sex, age, smoking habits within the families... 367 teenagers aged among 12 to 16, belonging to the three current educative models (Public, Semi-Public and Private) have been questioned in this research. The measures and assistance to obtain the result were an epidemiologic inquiry about tobacco consumption and dependence, together with the sport activity practised by teenagers. As most interesting data we may say that the 61,9% of the inquired teenagers have never tried tobacco (65,4% of the boys and 57,6% of the girls); the 18% declared to have smoked at least once (5,6% of the boys and the 4,1% of the girls), a 4,9% consider themselves as ex-smokers; and 15,1% smoke regularly, the 6,8% only at weekends and the 8,2% everyday (7,1% of the boys and 9,4% of the girls). The average age to start smoking in this survey

has been 12, 2 years old +1, 84. 31, 5% of the students do physical activity only in their P.E. classes, whereas 68, 1% usually practice a sport outside their school (44,1% are even federacy). We find a statistical relationship between smoking and practice a sport (p<0, 01) and within those who do sport smoke less those who are federacy (p<0, 01). It is not of significance smoking with the kind of energetic system used 8aerobic, anaerobic or mixed), neither with the type of sport (individual or collective), but it is connected with the fact of sharing a house with smokers (p<0, 01). Therefore we can affirm that the students asked are active and little smoker. We can say that physical activity has a great influence on teenager's attitude towards tobacco, and to great extend it prevents from taking to smoking and even more among the federacy group. The smoking habit in the teenager family is directly connected to the acquisition of the smoking habit by the teenager
SN - 0212-8799
AD - (Nistal Hernandez, Prieto Saborit, Del Valle Soto) Escuela de Medicina Deportiva, Oviedo, Spain (Gonzalez Diez) Patronato Deportivo Munic. M. C. P., Asturias, Spain (Nistal Hernandez) Cabrales 35, 4, 33201 Gijon (Asturias), Spain
ER -

TY - JOUR
ID - 3872
T1 - Structural defects and variations in the HIV-1 nef gene from rapid, slow and non-progressor children
A1 - Casartelli,N.
A1 - Di,Matteo G.
A1 - Argentini,C.
A1 - Cancrini,C.
A1 - Bernardi,S.
A1 - Castelli,G.
A1 - Scarlatti,G.
A1 - Plebani,A.
A1 - Rossi,P.
A1 - Doria,M.
Y1 - 2003///
N1 - 2003276755
English
Journal: Article
KW - EMBASE
KW - allele
KW - Amino Acid Sequence
KW - article
KW - Child
KW - childhood disease/dt [Drug Therapy]
KW - childhood disease/et [Etiology]
KW - clinical article
KW - disease course
KW - DNA sequence
KW - gene structure
KW - genetic variability
KW - highly active antiretroviral therapy
KW - Human
KW - human cell
KW - Human immunodeficiency virus 1
KW - Human immunodeficiency virus infection/dt [Drug Therapy]
KW - Human immunodeficiency virus infection/et [Etiology]
KW - molecular cloning
KW - mononuclear cell
KW - nef gene
KW - nucleotide sequence
KW - Phylogeny
KW - Polymerase Chain Reaction
KW - priority journal

KW - virus gene
KW - virus infection/dt [Drug Therapy]
KW - virus infection/et [Etiology]
KW - antiretrovirus agent/dt [Drug Therapy]
KW - CD4 antigen
KW - Nef protein
KW - virus protein
KW - virus RNA
RP - NOT IN FILE
SP - 1291
EP - 1301
JF - AIDS
VL - 17
IS - 9
CY - United Kingdom
N2 - Objectives: Evaluation of sequence evolution as well as structural defects and mutations of the human immunodeficiency virus-type 1 (HIV-1) nef gene in relation to disease progression in infected children. Design: We examined a large number of nef alleles sequentially derived from perinatally HIV-1-infected children with different rates of disease progression: six non-progressors (NPs), four rapid progressors (RPs), and three slow progressors (SPs). Methods: Nef alleles (182 total) were isolated from patients' peripheral blood mononuclear cells (PBMCs), sequenced and analysed for their evolutionary pattern, frequency of mutations and occurrence of amino acid variations associated with different stages of disease. Results: The evolution rate of the nef gene apparently correlated with CD4+ decline in all progression groups. Evidence for rapid viral turnover and positive selection for changes were found only in two SPs and two RPs respectively. In NPs, a higher proportion of disrupted sequences and mutations at various functional motifs were observed. Furthermore, NP-derived Nef proteins were often changed at residues localized in the folded core domain at cytotoxic T lymphocytes (CTL) epitopes (E₁₀₅, K₁₀₆, E₁₁₀, Y₁₃₂, K₁₆₄, and R₂₀₀), while other residues outside the core domain are more often changed in RPs (A₄₃) and SPs (N₁₇₃ and Y₂₁₄). Conclusions: Our results suggest a link between nef gene functions and the progression rate in HIV-1-infected children. Moreover, non-progressor-associated variations in the core domain of Nef, together with the genetic analysis, suggest that nef gene evolution is shaped by an effective immune system in these patients. 2003 Lippincott Williams & Wilkins
SN - 0269-9370
AD - (Casartelli, Bernardi, Castelli, Rossi, Doria) Div. of Immunol./Infect. Disease, Children's Hosp. 'Bambino Gesu', Rome, Italy (Di Matteo, Cancrini, Rossi) Department of Pediatrics, University of Rome 'Tor Vergata', Rome, Italy (Argentini) Laboratory of Virology, Istituto Superiore di Sanita, Rome, Italy (Plebani) Unit of Immunobiology of HIV, DIBIT-San Raffaele Sci. Institute, Milan, Italy (Scarlatti) Dept. of Pediatrics G. e D. De March, University of Milan, Milan, Italy (Doria) Divisione di Immunoinfettivologia, Osp. Pediatrico Bambino Gesu, Piazza S. Onofrio 4, 00165, Rome, Italy
ER -

TY - JOUR
ID - 3873
T1 - Airway cells after swimming outdoors or in the sea in nonasthmatic athletes
A1 - Bonsignore,M.R.
A1 - Morici,G.
A1 - Riccobono,L.
A1 - Profita,M.
A1 - Bonanno,A.
A1 - Paterno,A.
A1 - Di,Giorgi R.
A1 - Chimenti,L.
A1 - Abate,P.
A1 - Mirabella,F.
A1 - Vignola,A.M.
A1 - Bonsignore,G.
Y1 - 2003///

N1 - 2003273506
English
Journal: Article
KW - EMBASE
KW - Adolescent
KW - Adult
KW - article
KW - Asthma
KW - athlete
KW - cell count
KW - cell motility
KW - controlled study
KW - endurance
KW - Human
KW - human cell
KW - human experiment
KW - hyperventilation
KW - inflammatory cell
KW - leukocyte differential count
KW - marathon runner
KW - neutrophil
KW - normal human
KW - protein expression
KW - respiratory epithelium
KW - school child
KW - sitting
KW - Spirometry
KW - sputum level
KW - Swimming
KW - Training
KW - cell adhesion molecule/ec [Endogenous Compound]
KW - Chlorine
KW - eosinophil cationic protein/ec [Endogenous Compound]
KW - L selectin/ec [Endogenous Compound]
KW - leukocyte elastase/ec [Endogenous Compound]
KW - nitric oxide/ec [Endogenous Compound]
RP - NOT IN FILE
SP - 1146
EP - 1152
JF - Medicine and Science in Sports and Exercise
VL - 35
IS - 7
CY - United States
N2 - Background: Marathon runners and elite swimmers showed increased inflammatory cells in the airways at baseline. Although airway neutrophils increase further after a marathon race, the airway response to swimming is unknown. The aim of this study was to assess the effects of swimming on airway cells. To avoid the concomitant effects of chronic exposure to chlorine, the study was conducted in seven nonasthmatic swimmers [mean age (SD): 23.3 + 7.7 yr, training: 32 + 15 km.wk⁻¹] habitually training in an outdoor pool (OP), i.e., a low-chlorine environment. Methods: Spirometry, exhaled nitric oxide (NO), induced sputum, and peripheral blood samples were obtained at baseline, after a 5-km trial in OP, and after a 5-km race in the sea (S), i.e., hypertonic airway exposure. Results: Airway neutrophil differential counts at baseline were higher in swimmers than in sedentary controls (N = 10), but cell counts, neutrophil elastase, and eosinophil cationic protein were unaffected by 5-km swimming. After swimming, L-selectin expression on airway cells decreased, suggesting exercise-induced cell mobilization into the airways and/or direct effects of hyperventilation on airway cells. After S, airway eosinophil differential counts increased slightly. Exhaled NO concentration was 19 + 6 ppb at baseline, 8 + 4 ppb after OP, and 21 + 7 ppb after S ($P < 0.005$ for OP vs baseline and S). Conclusions: In swimmers not chronically exposed to high chlorine concentrations, data obtained at baseline

suggest a direct relationship between airway neutrophilia and endurance training. The low L-selectin expression by airway cells postexercise suggests hyperventilation-induced cell recruitment or modulation of cell function. Hypertonic exposure of airways during exercise may slightly increase airway eosinophils and exhaled NO. Overall, 5-km swimming exerted smaller effects on airway cells than running a marathon
SN - 0195-9131
AD - (Bonsignore, Vignola, Bonsignore) Institute of Medicine and Pneumology, University of Palermo, Palermo, Italy (Morici) Department of Experimental Medicine, University of Palermo, Palermo, Italy (Riccobono, Profita, Bonanno, Paterno, Di Giorgi, Chimenti, Abate, Mirabella, Bonsignore) Inst. Biomed./Molec. Immunol. (IBIM), National Council of Research (CNR), Palermo, Italy (Bonsignore) IBIM-CNR, Via Ugo La Malfa 153, 90146 Palermo, Italy
ER -

TY - JOUR
ID - 3874
T1 - Assessment of sun exposure in adolescent girls using activity diaries
A1 - Sullivan,S.S.
A1 - Cobb,J.L.
A1 - Rosen,C.J.
A1 - Holick,M.F.
A1 - Chen,T.C.
A1 - Kimlin,M.G.
A1 - Parisi,A.V.
Y1 - 2003///
N1 - 2003207151
English
Journal: Article
KW - EMBASE
KW - Adolescent
KW - article
KW - bone mineralization
KW - calcium blood level
KW - Child
KW - clinical article
KW - daily life activity
KW - dosimetry
KW - Female
KW - Human
KW - nutritional requirement
KW - priority journal
KW - Risk Assessment
KW - Self Report
KW - skin flora
KW - sun exposure
KW - ultraviolet B radiation
KW - vitamin metabolism
RP - NOT IN FILE
SP - 631
EP - 644
JF - Nutrition Research
VL - 23
IS - 5
CY - United States
N2 - The objective was to test a method of assessing sun exposure for use in stratifying adolescent girls according to potential for vitamin D₃ synthesis in the skin. Thirty-five girls wore polysulphone ultraviolet (UV) dosimeters and kept diaries of their activities on August 17, 2000. Minutes spent outdoors during each hour of the day were tabulated and adjusted for the fractional strength of ultraviolet B (UVB) radiation relative to peak hour. Total adjusted minutes outdoors were compared with UV dosimeter readings. A

series of ampules containing 7-dehydrocholesterol were exposed on a flat plane to further investigate the potential for skin synthesis of previtamin D₃ with a given sun exposure. The correlation between measured UVB exposure and self-reported minutes outdoors adjusted for the time of day was r = 0.64. In vitro previtamin D₃ synthesis at midday was reduced by 50% on a cloudy day compared with a sunny day. 2003 Elsevier Inc. All rights reserved
SN - 0271-5317
AD - (Sullivan, Cobb) Department of Food Science, University of Maine, 5735 Hitchner Hall, Orono, ME 04469, United States (Rosen) St. Joseph's Hospital, Bangor, ME, United States (Holick, Chen) Boston University School of Medicine, Boston, MA, United States (Kimlin, Parisi) Ctr. for Astron./Atmosph. Research, University of Southern Queensland, Toowoomba, QLD, Australia
ER -

TY - JOUR
ID - 3875
T1 - Increasing children's school time physical activity using structured fitness breaks
A1 - Scruggs,P.W.
A1 - Beveridge,S.K.
A1 - Watson,D.L.
Y1 - 2003///
N1 - 2003200598
English
Journal: Article
KW - EMBASE
KW - Analysis of Variance
KW - article
KW - body mass
KW - controlled study
KW - Female
KW - fitness
KW - Heart Rate
KW - Human
KW - Locomotion
KW - Male
KW - normal human
KW - Physical Activity
KW - Physical Education
KW - school
KW - school child
KW - Telemetry
RP - NOT IN FILE
SP - 156
EP - 169
JF - Pediatric Exercise Science
JA - Pediatr Exerc Sci
VL - 15
IS - 2
CY - United States
N2 - Fifth-grade students' physical activity levels were examined via heart rate telemetry and pedometry during school fitness and recess breaks. Twenty-seven students with a mean age of 11.03 (+.32) years participated in morning recess (MR), lunch recess (LR), and fitness breaks (FB) for three days. Structured FB's consisted of students engaging in locomotor and nonlocomotor activities within an obstacle course framework, while recess breaks followed a traditional model. Results from repeated measures ANOVAs indicated students engaged in significantly more physical activity during FB than MR and LR. Fitness breaks provided a viable method for increasing children's school time activity levels
SN - 0899-8493
AD - (Scruggs) Department of Physical Education, Miami University, Oxford, OH, United States (Beveridge, Watson) Department of Exercise, University of Utah, Salt Lake City, UT, United States

ER -

TY - JOUR
ID - 3876
T1 - Brief probes: A method for analysing the function of disruptive behaviour in the natural environment
A1 - Aikman,G.
A1 - Garbutt,V.
A1 - Fumiss,F.
Y1 - 2003///
N1 - 2003193057
English
Journal: Article
KW - EMBASE
KW - article
KW - behavior disorder/th [Therapy]
KW - Case Report
KW - clinical feature
KW - developmental disorder/th [Therapy]
KW - disruptive behavior/th [Therapy]
KW - environmental factor
KW - escape behavior
KW - Female
KW - Human
KW - leisure
KW - priority journal
KW - school child
KW - task performance
RP - NOT IN FILE
SP - 215
EP - 220
JF - Behavioural and Cognitive Psychotherapy
VL - 31
IS - 2
CY - United Kingdom
N2 - The present study illustrates the use of brief functional analysis probe conditions to verify the results of a descriptive assessment. An initial descriptive assessment of the disruptive behaviour of an 8-year-old student with severe developmental disabilities showed that levels of disruptive behaviour (screaming and throwing equipment) were higher in some lessons than others and suggested that the behaviour might be maintained by escape from task demands. An intervention in which work demands were altemated with 5-minute periods of free activity reduced levels of screaming to under 50%, and of throwing to under 25%, of baseline levels. Brief experimental variations of demand level in some lessons confirmed that levels of disruption were generally higher under high demand conditions. We conclude that brief probes provide a method by which experimental analyses can be conducted in the client's natural environment, reducing the problem of non-occurrence of the target behaviour that can pose problems for analogue assessments and facilitating ongoing assessment during initial intervention. We note also, however, that the consequent reduction in control over establishing operations may reduce the precision of the analysis and that ethical considerations limit the range of behaviours for which the method is appropriate
SN - 1352-4658
AD - (Aikman, Garbutt) Hesley Group, Doncaster, United Kingdom (Fumiss) Hesley Group, Doncaster/University of Leicester, Leicester, United Kingdom (Fumiss) Hesley Group, Coach House, Hesley Hall, Tickhill, Doncaster DN11 9HH, United Kingdom
ER -

TY - JOUR
ID - 3877
T1 - A comparison of the traffic-related benzene, toluene, xylene, and platinum exposure of children living in Eastern and Western German states

A1 - Begerow,J.
A1 - Jermann,E.
A1 - Turfeld,M.
A1 - Sugiri,D.
A1 - Kramer,U.
A1 - Schumann,G.
A1 - Dunemann,L.
Y1 - 2003///
N1 - 2003126844
German
Journal: Article
KW - EMBASE
KW - article
KW - car
KW - catalyst
KW - Child
KW - comparative study
KW - concentration (parameters)
KW - Environmental Exposure
KW - exhaust gas
KW - Germany
KW - Human
KW - parent
KW - questionnaire
KW - Traffic
KW - urinary excretion
KW - benzene
KW - domestic chemical
KW - platinum
KW - solvent
KW - toluene
KW - xylene
RP - NOT IN FILE
SP - 33
EP - 42
JF - Umweltmedizin in Forschung und Praxis
VL - 8
IS - 1
CY - Germany
N2 - A field study was performed to assess the internal exposure of 149 five- to seven-year old children to traffic-related pollutants. The study was carried out in areas with high and low traffic density located in Western Germany (Essen/Ruhr, Borken, Northrhine-Westphalia) and Eastern Germany (Halle/Saale, Osterburg, Saxony-Anhalt). The benzene, toluene, o-,m-,p-xylene (BTX) concentrations in blood and the platinum excretion in urine served as markers for a traffic-related exposure. The parents were asked to answer a questionaire, which asked for such information as traffic density in the vicinity of the dwelling, redecoration activities inside the rooms, exposure to solvent containing household products, passive smoking, etc. The results of this study show, that emissions from traffic have a significant influence on the internal BTX burden of children. The internal benzene exposure is mainly a result of the benzene concentration in outdoor air, which as a result of the absence of indoor sources is reflected in indoor air. Regarding toluene and xylene indoor air concentrations were generally higher than those outdoors indicating that several relevant indoor sources exist. The urinary platinum (Pt) excretion of the children from both high traffic areas (Essen/Ruhr, Halle/Saale) tended to higher values when compared with the Pt excretion of those children from the low-traffic areas. However, this differences were statistically not significant. The results of this study do not definitively prove whether emissions from automobile catalysts are responsible for the slightly higher Pt exposure of these children
SN - 1430-8681
AD - (Begerow, Dunemann) Hygiene-Institut des Ruhrgebiets, Inst. Umwelthygiene/Umweltmedizin, Rotthauser Strase 19, D-45879 Gelsenkirchen, Germany (Jermann, Turfeld) Med. Institut fur Umwelthygiene,

Aufm Hennekamp 50, D-40225 Dusseldorf, Germany (Sugiri, Kramer) Inst. fur Umweltmed. Forsch. gGmbH, Auf'm Hennekamp 50, D-40225 Dusseldorf, Germany (Schumann) Hygieneinstitut Sachsen-Anhalt, Wallonerberg 2/3, D-39104 Magdeburg, Germany
ER -

TY - JOUR
ID - 3878
T1 - The active surface of suspended particles as a predictor of lung function and pulmonary symptoms in Austrian school children
A1 - Moshammer,H.
A1 - Neuberger,M.
Y1 - 2003///
N1 - 2003125753
English
Journal: Article
KW - EMBASE
KW - aerosol
KW - air monitor
KW - air monitoring
KW - article
KW - Austria
KW - clinical article
KW - coughing
KW - diffusion
KW - dyspnea
KW - electronic sensor
KW - Female
KW - Forced Expiratory Volume
KW - Human
KW - lung disease
KW - lung function
KW - Male
KW - medical examination
KW - night
KW - Particle Size
KW - pollution monitoring
KW - Prediction
KW - primary school
KW - priority journal
KW - questionnaire
KW - school child
KW - Signal Processing
KW - Spirometry
KW - surface property
KW - suspended particulate matter
KW - symptom
KW - wheezing
KW - polycyclic aromatic hydrocarbon
RP - NOT IN FILE
SP - 1737
EP - 1744
JF - Atmospheric Environment
VL - 37
IS - 13
CY - United Kingdom
N2 - At a central elementary school in the capital of Upper Austria children aged 7-10 years underwent repeated respiratory health checkups (questionnaires, diaries, spirometry). Between March and May 2001 the

daily means of the signals of a diffusion charging sensor, measuring the "active surface" of suspended particles, and a photoelectric aerosol sensor, measuring the particle-bound polycyclic aromatic hydrocarbons, were related to spirometric results of the total 164 children examined and to the daily symptom scores of a susceptible subgroup. Significant reductions of forced vital capacity (p=0.006) and forced expiratory volume in the first second (p=0.001) and significant increases of wheezing (p=0.001), shortness of breath (p=0.041), cough in the evening (p=0.031) and at night (p=0.018) were found with increase of "active surface" of suspended particles measured at the adjacent outdoor monitoring station, but not with the increase of particle-bound polycyclic aromatic hydrocarbons. Monitoring "active surface" of particles with diameters of about 10nm-1mum by means of a diffusion charging sensor might provide additional information in surveillance of particulate matter for prevention of acute effects on respiratory health. 2003 Elsevier Science Ltd. All rights reserved
SN - 1352-2310
AD - (Moshammer, Neuberger) Institute of Environmental Health, University of Vienna, Kinderspitalgasse 15, A-1095 Vienna, Austria
ER -

TY - JOUR
ID - 3879
T1 - Fostering generalization and maintenance in school settings
A1 - Scott,Yaruss J.
A1 - Reardon,N.A.
Y1 - 2003///
N1 - 2003115445
English
Journal: Article
KW - EMBASE
KW - article
KW - Comprehension
KW - daily life activity
KW - Human
KW - Interpersonal Communication
KW - language ability
KW - Learning
KW - Motivation
KW - school child
KW - school health service
KW - Speech Therapy
KW - Stuttering/th [Therapy]
KW - Treatment Planning
RP - NOT IN FILE
SP - 33
EP - 40
JF - Seminars in Speech and Language
VL - 24
IS - 1
CY - United States
N2 - One of the most challenging aspects of therapy for school-age children who stutter is generalizing the skills learned in the therapy room to other settings such as the classroom, lunchroom, playground, or home. An additional challenge is seen in maintaining gains over the long term. This article reviews common roadblocks to generalization and maintenance, including the goals of therapy, the nature of the treatment strategies that require generalization, the scheduling and implementation of generalization activities within the overall therapy process, and the child's understanding of the treatment goals. Specific strategies for overcoming these roadblocks include: desensitizing children to both stuttering and treatment strategies designed to improve fluency, using hierarchies as a way of structuring treatment and moving children toward success in their daily activities, integrating the child's real world and clinical settings, and using structured practice activities to help children solidify all of the lessons they learn in treatment. This article highlights the importance of taking a broad-based view of stuttering to help children improve their overall communication across a variety of settings and over time

SN - 0734-0478
AD - (Scott Yaruss) University of Pittsburg, Children's Hospital of Pittsburg, Stuttering Center of W. Pennsylvania, 4033 Forbes Tower, Pittsburg, PA 15260, United States
ER -

TY - JOUR
ID - 3880
T1 - Dental anomalies of the permanent lateral incisors and prevalence of hypodontia outside the cleft area in complete unilateral cleft lip and palate
A1 - Ribeiro,L.L.
A1 - Das Neves,L.T.
A1 - Costa,B.
A1 - Gomide,M.R.
Y1 - 2003///
N1 - 2003113541
English
Journal: Article
KW - EMBASE
KW - article
KW - Child
KW - cleft lip palate
KW - congenital disorder
KW - controlled study
KW - Female
KW - Human
KW - hypodontia
KW - incisor
KW - major clinical study
KW - Male
KW - panoramic radiography
KW - premolar tooth
KW - Prevalence
KW - priority journal
KW - sex difference
KW - tooth malformation
KW - tooth size
RP - NOT IN FILE
SP - 172
EP - 175
JF - Cleft Palate-Craniofacial Journal
JA - Cleft Palate Craniofac J
VL - 40
IS - 2
CY - Canada
N2 - Objective: To determine in complete unilateral cleft lip and palate (UCLP) subjects the characteristics (location, shape) of the cleft-side lateral incisor. The presence of a supernumerary tooth at the cleft side and the prevalence of hypodontia outside the cleft area were evaluated. A comparison was made of the shape of the cleft side lateral incisor to its contralateral incisor. Setting: Hospital for Rehabilitation of Craniofacial Anomalies (HRCA), Sao Paulo, Brazil. Patients: Orthopantomograms of 203 subjects with UCLP and without syndromes were chronologically selected from the HRCA data bank, within an age range of 5 to 10 years. Outcome Measure: Orthopantomograms were analyzed by the same observer according to established criteria. Results: There were no statistically significant differences between sexes for any of the criteria studied. The cleft-side lateral incisor was present in 50.2%, and it was more commonly located at the distal side (76.5%). The congenital absence of the cleft-side lateral incisor was observed in 49.8% of the sample, and its antimere was congenitally missing in 10.9%, this difference being statistically significant. The most commonly missing tooth outside the cleft area was the maxillary second premolar. Conclusions: The high prevalence of hypodontia of the

permanent lateral incisor in the cleft side showed that the cleft could play an important role in this absence. There were different patterns for the presence of the cleft-side lateral incisor
SN - 1055-6656
AD - (Ribeiro, Das Neves, Costa, Gomide) Department of Dentistry for Children, Craniofacial Anomalies Rehab. Hosp., University of Sao Paulo, Sao Paulo, Brazil (Gomide) Setor de Odontopediatria, Hosp. Reab. Anomalias Craniofaciais, Universidade de Sao Paulo, R. Silvio Marchione, 3-20, Bauru, SP, Brazil
ER -

TY - JOUR
ID - 3881
T1 - Important issues in the care and evaluation of bilingual/multilingual children
A1 - Chavda,U.
A1 - Kao,R.
A1 - Soldatou,A.
A1 - Gardner,A.
A1 - Knudson,P.
A1 - Su,H.
A1 - Schoon,Eberly S.
A1 - Van,Dyke,D.C.
Y1 - 2003///
N1 - 2003109203
English
Journal: Review
KW - EMBASE
KW - Child
KW - Child Care
KW - Education
KW - emotion
KW - environmental factor
KW - Family
KW - Human
KW - information
KW - Language Development
KW - learning disorder/di [Diagnosis]
KW - mental deficiency/di [Diagnosis]
KW - nurse
KW - pediatrician
KW - physician assistant
KW - review
KW - social worker
KW - Speech
KW - United States
RP - NOT IN FILE
SP - 8
EP - 13
JF - International Pediatrics
VL - 18
IS - 1
CY - United States
N2 - The number of children in the United States who are bilingual or multilingual is both significant and increasing. Pediatricians, physician assistants, and nurse practitioners need to understand the processes of language development in bilingual children in order to support the families of bilingual children, provide accurate educational information, and prevent potential of misdiagnoses in these children as having such conditions as mental retardation, learning disabilities, or speech-language disorders. Evaluation of a child's development begins with a careful review of home, social, educational environments, and medical history. Gathering an accurate history may require the use of resources such as an interpreter, social worker, education consultant, psychologist, or speech-language pathologist. Pediatricians, physician assistants, and nurse

practitioners can have a positive impact on the educational and heath care environment of these children by using this information, assessing the development of these children, and by sharing their knowledge with others who play a role in the lives of these children. Bilingualism/multilingualism does not lead to ongoing language delays. Bilingualism/multilingualism can play a positive role in a child's emotional development. In families that place a high value on tradition, speaking the language of parents and grandparents offers a way for the child to better understand the family's heritage even though it is not the dominant language of the child's environment outside the home. Speaking the language of a surrounding culture also provides insights into that culture. Bilingualism is an important resource; it has the potential to increase employment and offer other opportunities in the child's future especially in this rapidly globalizing world
SN - 0885-6265
AD - (Chavda, Van Dyke) Department of Pediatrics, Children's Hospital of Iowa, Ctr. for Disabil. and Development, 100 Hawkins Drive, Iowa City, IA 52242-1011, United States (Kao) Department of Emergency Medicine, University of Iowa Health Care, Iowa City, IA, United States (Soldatou) Department of Pediatrics, Children's Hospital of Iowa, Iowa City, IA, United States (Gardner) Pediatric Psychology, Ctr. for Disabil. and Development, Univ. of Iowa Hospitals and Clinics, Iowa City, IA, United States (Knudson) Dept. of Speech/Language/Audiology, Ctr. for Disabil. and Development, Univ. of Iowa Hospitals and Clinics, Iowa City, IA, United States (Su) International Programs, Center for Asian Pacific Studies, University of Iowa, Iowa City, IA, United States (Schoon Eberly) Ctr. for Disabil. and Development, Univ. of Iowa Hospitals and Clinics, Iowa City, IA, United States
ER -

TY - JOUR
ID - 3882
T1 - CD4 and Major Histocompatibility Complex Class I Downregulation by the Human Immunodeficiency Virus Type 1 Nef Protein in Pediatric AIDS Progression
A1 - Casartelli,N.
A1 - Di,Matteo G.
A1 - Potesta,M.
A1 - Rossi,P.
A1 - Doria,M.
Y1 - 2003///
N1 - 2003419479
English
Journal: Article
KW - EMBASE
KW - acquired immune deficiency syndrome/et [Etiology]
KW - Amino Acid Sequence
KW - Amino Acid Substitution
KW - antigen expression
KW - article
KW - cell surface
KW - Child
KW - clinical article
KW - controlled study
KW - disease course
KW - down regulation
KW - Human
KW - human cell
KW - Human immunodeficiency virus 1
KW - in vitro study
KW - nef gene
KW - nonhuman
KW - nucleotide sequence
KW - Pediatrics
KW - perinatal infection
KW - priority journal
KW - protein domain

KW - virus gene
KW - virus pathogenesis
KW - Virus Replication
KW - adaptor protein
KW - CD4 antigen/ec [Endogenous Compound]
KW - clathrin
KW - major histocompatibility antigen class 1/ec [Endogenous Compound]
KW - Nef protein
KW - transcription factor AP 1
RP - NOT IN FILE
SP - 11536
EP - 11545
JF - Journal of Virology
VL - 77
IS - 21
CY - United States
N2 - The human immunodeficiency virus type 1 (HIV-1) nef gene is a crucial determinant in AIDS disease progression. Although several in vitro activities have been attributed to the Nef protein, identifying the one critical for in vivo pathogenicity remains elusive. In this study, we examined a large number of nef alleles derived at various time points from 13 perinatally infected children showing different progression rates: six nonprogressors (NPs), three slow progressors (SPs), and four rapid progressors (RPs). The patient-derived nef alleles were analyzed for their steady-state expression of a Nef protein, for their relative ability to downregulate cell surface expression of CD4 and major histocompatibility complex class I (MHC-I) and for their capacity to bind the clathrin adaptor AP-1 complex. We found that NP-derived nef alleles, compared to nef alleles isolated from SPs and RPs, had reduced CD4 and MHC-I downregulation activities. In contrast, SP- and RP-derived nef alleles did not differ and efficiently downregulated both CD4 and MHC-I. AP-1 binding was a conserved function of primary nef alleles not correlated with clinical progression. Defective Nef proteins from NPs, rather than sharing common specific changes in their sequences, accumulated various amino acid substitutions, mainly located outside the conserved domains previously associated with Nef biological properties. Our data indicate that Nef-mediated downregulation of cell surface CD4 and MHC-I significantly contributes to the expression of the pathogenic potential of HIV-1
SN - 0022-538X
AD - (Casartelli, Potesta, Rossi, Doria) Div. of Immunol. and Infect. Disease, Children's Hospital Bambino Gesu, Rome, Italy (Di Matteo, Rossi) Department of Pediatrics, University of Rome Tor Vergata, Rome, Italy (Doria) Divisione di Immunoinfettivologia, Ospedale Pediatrico Bambino Gesu, Piazza S. Onofrio 4, 00165 Rome, Italy
ER -

TY - JOUR
ID - 3884
T1 - Childhood thyroid cancer in the French region of Franche-Comte: Chernobyl impact not proven
A1 - Mauny,F.
A1 - Grandmottet,M.
A1 - Marquet,G.
A1 - Floret,N.
A1 - Crenn,D.
A1 - Olivier-Koehret,M.
A1 - Viel,J.-F.
Y1 - 2002///
N1 - 2003285005
French
Journal: Article
KW - EMBASE
KW - Adolescent
KW - article
KW - cancer incidence
KW - Chernobyl accident

KW - childhood cancer/ep [Epidemiology]
KW - childhood cancer/et [Etiology]
KW - clinical article
KW - Female
KW - general practitioner
KW - Human
KW - Male
KW - nuclear accident
KW - physician
KW - Poisson Distribution
KW - Public Health
KW - school child
KW - Sex Ratio
KW - thyroid cancer/ep [Epidemiology]
KW - thyroid cancer/et [Etiology]
RP - NOT IN FILE
SP - 283
EP - 288
JF - Environnement, Risques et Sante
VL - 1
IS - 5-6
CY - France
N2 - Problem. The increased incidence of childhood thyroid cancer in areas heavily contaminated by the accident at Chernobyl is now well established. Initial studies outside the former USSR showed no evidence of changes in this incidence. France received much less fallout than did the former USSR, but deposition was highly heterogeneous in eastern France, particularly in the region of Franche-Comte. Under the auspices of the Union regionale des medecins liberaux de Franche-Comte (regional association of private practitioners), the general practitioners of this area decided to assess the health consequences in children living in Franche-Comte at the time of the accident of Chernobyl. Method. Incident cases from 1980 through 1998 among children younger than 15 years were collected from general practitioners, specialists, and hospital staff physicians in the Franche-Comte and surrounding regions. A Poisson test was used to analyse temporal heterogeneity (including a 4-year latency period) and spatial heterogeneity (between the 4 administrative areas composing the area) separately. Results. During the 19-year study period, 8 cases of childhood thyroid cancer were identified. The sex ratio was 1.67, and 7 of the cases were diagnosed in children in the 10-14 age group. The standardized incidence rate was 0.15/100,000. The incidence rate increased from 0.13/100,000 (95% CI: 0.03-0.37) in 1980-1989 to 0.26/100,000 (95% CI: 0.08-0.60) in 1990-1998. No variations were significant, however, either in space (p = 0.44) or time (p = 0.48). Conclusions. Although this study did not observe a significant increase in the incidence of childhood thyroid cancer after Chernobyl, it highlights two needs: first, for the assessment of the consequences of the accident at Chernobyl in France among a wider population, and second, for an extension of the follow-up period. This study also illustrates the role that private practice physicians can play in the field of public health
SN - 1635-0421
AD - (Mauny, Floret, Crenn, Viel) Departement de Sante Publique, Unite de Biostatistique, Faculte de Medicine et de Pharmacie, 2, place Saint-Jacques, 25030 Besancon Cedex, France (Grandmottet, Marquet, Olivier-Koehret) Union Regl. Med. Liberaux Franche-C., 28, rue de la Republique, 25000 Besancon, France
ER -

TY - JOUR
ID - 3885
T1 - A quantitative nutritional evaluation of a healthy eating intervention in primary school children in a socioeconomically disadvantaged area - A pilot study
A1 - O'Brien,N.
A1 - Roe,C.
A1 - Reeves,S.
Y1 - 2002///

N1 - 2003031673
English
Journal: Article
KW - EMBASE
KW - article
KW - caloric intake
KW - child health care
KW - child nutrition
KW - clinical article
KW - dietary intake
KW - Female
KW - Food Intake
KW - Human
KW - Male
KW - malnutrition/pc [Prevention]
KW - Nutrition
KW - obesity/pc [Prevention]
KW - priority journal
KW - school
KW - school child
KW - socioeconomics
RP - NOT IN FILE
SP - 320
EP - 328
JF - Health Education Journal
VL - 61
IS - 4
CY - United Kingdom
N2 - Objective To perform a pilot study that evaluates the nutritional and dietary habits of primary school children in a socioeconomically disadvantaged area prior to and following a healthy eating intervention programme. Design A quantitative study whereby the food intakes of 20 boys and 20 girls aged 8 to 9 years old were recorded for five days prior to, and for five days after the intervention. Discussions with the children to ascertain their attitudes towards healthy foods were carried out and parental support was encouraged. Setting A primary school in a socioeconomically disadvantaged area in the city of Limerick, Republic of Ireland. Method Food intake data was collected using food diaries and analysed using the computer package Diet 5 for Windows. Paired t-tests were performed to assess any significant changes in the dietary intakes. Results Following intervention, there were significant increases in the consumption of energy (P<0.001), protein (P<0.001), fat (P<0.01), and calcium (Palpha0.0001). Whilst most of the nutrients were consumed in appropriate amounts, the percentage energy from fat was higher than recommendations, whilst iron intakes were below recommendations. Conclusion Although, the changes in food consumption were positive, future interventions should emphasise the need to monitor fat intakes, improve iron status and encourage discussion outside the classroom environment. Similar interventions based on this pilot study could also be used with respect to other areas of health promotion, for example, activity, anti-smoking and so on
SN - 0017-8969
AD - (O'Brien) Mary Immaculate College, Limerick (Roe) St. Mary's College, Twickenham, United Kingdom (Reeves) St. Mary's College, Twickenham, Strawberry Hill, Twickenham TW1 4SX, United Kingdom
ER -

TY - JOUR
ID - 3886
T1 - Pediatric musculoskeletal trauma
A1 - Hosalkar,H.S.
A1 - Lou,J.E.
A1 - Flynn,J.M.
Y1 - 2002///

N1 - 2002412041
English
Journal: Review
KW - EMBASE
KW - Adolescent
KW - baseball
KW - Basketball
KW - bicycle
KW - bone remodeling
KW - Child
KW - Child Care
KW - childhood injury/dm [Disease Management]
KW - childhood injury/ep [Epidemiology]
KW - childhood injury/pc [Prevention]
KW - childhood injury/su [Surgery]
KW - cost benefit analysis
KW - Football
KW - Fracture Healing
KW - health care cost
KW - Human
KW - musculoskeletal injury/dm [Disease Management]
KW - musculoskeletal injury/ep [Epidemiology]
KW - musculoskeletal injury/pc [Prevention]
KW - musculoskeletal injury/su [Surgery]
KW - pediatric surgery
KW - play
KW - priority journal
KW - review
KW - sport injury/dm [Disease Management]
KW - sport injury/ep [Epidemiology]
KW - sport injury/pc [Prevention]
KW - sport injury/su [Surgery]
RP - NOT IN FILE
SP - 413
EP - 418
JF - Current Opinion in Orthopaedics
VL - 13
IS - 6
CY - United States
N2 - Current national demographic data based on the National Health Survey states that 20.6 million or approximately 25 per 100 children in the United States are injured each year. There are about 56,000 nonfatal injury episodes in children each day that require medical attention. Eight common recreational activities: baseball/softball, basketball, bicycling, football, playgrounds, roller sports, soccer, and trampolines, led to most injuries in children ages 5 to 14 years old. These injuries accounted for more than 2 million medically treated musculoskeletal injuries costing over $33 billion. Caring for children with orthopaedic injuries requires an awareness of their unique mechanism of injury, associated injuries, and healing and remodeling rates. The focus of this chapter is to review the past year's literature with a discussion of prevention, epidemiology, evaluation, treatment, and complications of pediatric orthopaedic injuries. 2002 Lippincott Williams & Wilkins, Inc
SN - 1041-9918
AD - (Hosalkar, Lou, Flynn) Div. of Orthopaedic Surgery, Children's Hospital Of Philadelphia, Wood Building, 34th St. and Civic Center Boulevard, Philadelphia, PA 19104, United States
ER -

TY - JOUR
ID - 3887
T1 - Management of status epilepticus
A1 - Misra,S.

A1 - Singh,N.N.
Y1 - 2002///
N1 - 2002365474
English
Journal: Review
KW - EMBASE
KW - Adult
KW - anticonvulsant therapy
KW - Child
KW - clinical protocol
KW - Emergency Treatment
KW - Epilepsy/di [Diagnosis]
KW - Epilepsy/dt [Drug Therapy]
KW - Epilepsy/et [Etiology]
KW - epileptic state/di [Diagnosis]
KW - epileptic state/dt [Drug Therapy]
KW - epileptic state/et [Etiology]
KW - hospital care
KW - Human
KW - pathophysiology
KW - practice guideline
KW - review
KW - seizure/dt [Drug Therapy]
KW - seizure/et [Etiology]
KW - Treatment Planning
KW - anticonvulsive agent/ad [Drug Administration]
KW - anticonvulsive agent/do [Drug Dose]
KW - anticonvulsive agent/dt [Drug Therapy]
KW - anticonvulsive agent/im [Intramuscular Drug Administration]
KW - anticonvulsive agent/iv [Intravenous Drug Administration]
KW - anticonvulsive agent/rc [Rectal Drug Administration]
KW - benzodiazepine/ad [Drug Administration]
KW - benzodiazepine/do [Drug Dose]
KW - benzodiazepine/dt [Drug Therapy]
KW - benzodiazepine/iv [Intravenous Drug Administration]
KW - benzodiazepine/rc [Rectal Drug Administration]
KW - clonazepam/ad [Drug Administration]
KW - clonazepam/dt [Drug Therapy]
KW - clonazepam/iv [Intravenous Drug Administration]
KW - diazepam/ad [Drug Administration]
KW - diazepam/do [Drug Dose]
KW - diazepam/dt [Drug Therapy]
KW - diazepam/iv [Intravenous Drug Administration]
KW - diazepam/rc [Rectal Drug Administration]
KW - fosphenytoin sodium/ad [Drug Administration]
KW - fosphenytoin sodium/do [Drug Dose]
KW - fosphenytoin sodium/dt [Drug Therapy]
KW - fosphenytoin sodium/im [Intramuscular Drug Administration]
KW - lorazepam/ad [Drug Administration]
KW - lorazepam/do [Drug Dose]
KW - lorazepam/dt [Drug Therapy]
KW - lorazepam/iv [Intravenous Drug Administration]
KW - midazolam/ad [Drug Administration]
KW - midazolam/dt [Drug Therapy]
KW - midazolam/iv [Intravenous Drug Administration]
KW - paraldehyde/ad [Drug Administration]
KW - paraldehyde/do [Drug Dose]

KW - paraldehyde/dt [Drug Therapy]
KW - paraldehyde/im [Intramuscular Drug Administration]
KW - paraldehyde/rc [Rectal Drug Administration]
KW - pentobarbital/ad [Drug Administration]
KW - pentobarbital/do [Drug Dose]
KW - pentobarbital/dt [Drug Therapy]
KW - pentobarbital/iv [Intravenous Drug Administration]
KW - phenobarbital/do [Drug Dose]
KW - phenobarbital/dt [Drug Therapy]
KW - phenytoin/do [Drug Dose]
KW - phenytoin/dt [Drug Therapy]
KW - propofol/ad [Drug Administration]
KW - propofol/do [Drug Dose]
KW - propofol/dt [Drug Therapy]
KW - propofol/iv [Intravenous Drug Administration]
KW - valproic acid/ad [Drug Administration]
KW - valproic acid/dt [Drug Therapy]
KW - valproic acid/iv [Intravenous Drug Administration]
RP - NOT IN FILE
SP - 299
EP - 303
JF - Journal of the Indian Medical Association
JA - J Indian Med Assoc
VL - 100
IS - 5
CY - India
N2 - Status epilepticus is a medical emergency, if not treated in time and effectively may cause significant mortality and morbidity. Medical therapy has been the mainstay of treatment but in refractory status surgical resection, multiple subpial transection, electroconvulsive therapy, caudate stimulation and acupuncture play important role. The present operational definition for adults and older children considers status as > 5 minutes of continuous seizure or two or more discrete seizures without regaining of full consciousness. Status epilepticus accounts for 1-8% of all hospital admissions for epilepsy. Physiological changes in generalised convulsive status epilepticus include transient or early (0-30 minutes) and late (after 30 minutes) changes. Temporal changes occur as tonic-clonic status epilepticus progresses. Management can be considered in two ways - out hospital management and inpatient management. Benzodiazepine is considered 1st line of treatment outside hospital. Emergency/inpatient management includes basic life support (0-10 minutes) and pharmacological management (10-60 minutes). Drugs used in pharmacological management are lorazepam, midazolam, propofol, phenobarbital, phenytoin, fosphenytoin, IV valproate, rectal diazepam, etc. The classical definition of refractory status epilepticus includes seizure that has not responded to sequential treatment of lorazepam, phenytoin or phenobarbitone or seizure continuing >60-90 in spite of adequate treatment
SN - 0019-5847
AD - (Misra, Singh) Department of Neurology, Institute of Medical Sciences, Banaras Hindu University, Varanasi 221005, India
ER -

TY - JOUR
ID - 3888
T1 - Risk factors: Environment, exposure to tobacco smoke
A1 - Herauld,M.C.
A1 - Herbelin-Wagner,M.L.
Y1 - 2002///
N1 - 2002290825
French
Journal: Review
KW - EMBASE
KW - allergy
KW - Asthma/et [Etiology]

KW - bronchus hyperreactivity
KW - Child
KW - Environmental Exposure
KW - environmental factor
KW - Geography
KW - Human
KW - immunoreactivity
KW - Maternal Behavior
KW - mother fetus relationship
KW - passive smoking
KW - Pollution
KW - Pregnancy
KW - prenatal exposure
KW - Prevalence
KW - protection
KW - Respiratory syncytial pneumovirus
KW - respiratory tract disease
KW - review
KW - Risk Assessment
KW - risk factor
KW - sensitization
KW - smoking and smoking related phenomena
KW - allergen
KW - tobacco smoke/to [Drug Toxicity]
RP - NOT IN FILE
SP - 377
EP - 383
JF - Archives de Pediatrie
JA - Arch Pediatr
VL - 9
IS - SUPPL. 3
CY - France
N2 - Environmental factors are usually considered as risk factors for increase of asthma prevalence. They may act isolately but are frequently associated. They act either directly by inducing asthma or more likely by increasing allergenic sensitization. Geographic situation is a well known risk factor. Important differences are noted between countries. The western lifestyle is evocated, including type of alimentation, small size of siblings, increased allergen exposure in houses. Intrauterine environment may play a role, particularly tobacco smoke during pregnancy and its respiratory effects on infant. Maternal allergenic exposure during pregancy is an important factor because of maternofetal immunologic interactions. Outdoor pollution acts by enhancing bronchial responsiveness, allergenic sensitization and worsening respiratory diseases. Its effect is probably less important in infants and small children who are living indoor most of the time. Infections seems to have a complex action. Some virus, including respiratory syncytial virus, act to induce asthma or sensitization. Other type of infections (viral ou microbial) have a protector effect. Exposure to tobacco smoke, particularly maternal smoking, is identified in all studies, as one of the most important factors to be considered in childhood asthma. Exposure to allergen increases the risk of sensitization. Its direct responsability to induce asthma is not established. Some of recent studies are suggesting the concept of a protective effect of early exposure. As far as preventive intervention is concerned, the recognition of these factors is important to limit the prevalence of childhood asthma. 2002 Editions scientifiques et medicales Elsevier SAS
SN - 0929-693X
AD - (Heraud, Herbelin-Wagner) U. Reanim./Maladies Resp. l'Enfant, Hotel-Dieu, BP 69, 63003 Clermont-Ferrand Cedex 01, France
ER -

TY - JOUR
ID - 3889
T1 - Risk and proactive factors for aggresive behavior, and its generalization in a sample of Mexican school children

A1 - Ayala,Velazquez H.
A1 - Pedroza,Cabrera F.
A1 - Morales,Chaine S.
A1 - Chaparro Caso-Lopez,A.
A1 - Barragan,Torres N.
Y1 - 2002///
N1 - 2002288498
Spanish
Journal: Review
KW - EMBASE
KW - Aggression
KW - Aggressiveness
KW - Antisocial Behavior
KW - Child Behavior
KW - child parent relation
KW - correlation analysis
KW - group psychology
KW - habit
KW - Human
KW - Mexico
KW - patient monitoring
KW - Problem Solving
KW - psychologic assessment
KW - review
KW - Risk Assessment
KW - risk factor
KW - school child
KW - social adaptation
KW - Social Interaction
KW - Social Support
RP - NOT IN FILE
SP - 27
EP - 40
JF - Salud Mental
VL - 25
IS - 3
CY - Mexico
N2 - Aggressive behavior in childhood has become a socially relevant problem demanding special attention from researchers, as a sharp increase has been reported in the aggressive and antisocial behavior of children and adolescents. Especially disturbing is the high participation of underage individuals in crimes such as robbery, drug trafficking and homicide, while numerous studies have shown that aggressive behavior evolves into more severe antisocial behaviors in adolescence and young adulthood. Research studies conducted on childhood aggressiveness has shown that these behaviors tend to become habits across time, and furthermore to progress into more complex and serious behaviors such as delinquency. For example, a high correlation has been reported between childhood aggressive behavior and antisocial behavior in adolescence and early adulthood. In an effort to prevent and stop the evolution of childhood aggressive behavior, efforts have been made to identify and describe those factors associated with the emergence of these behaviors in the developmental stages that comprise childhood and adolescence. Some researchers have coined terms such as risk and protective factors, reporting that it is highly probable that an individual might develop problem behaviors as the number of risk factors increases and exceeds the number of protective factors. It has been suggested that risk factors may be classified into four large groups: 1) the child's characteristics, 2) the parent's characteristics, 3) the contextual factors, and 4) the parent-child interaction. In a similar fashion, the protective factors that seem to ameliorate the occurrence of antisocial behaviors are classified into 5 main types: 1) parent-child support relations, 2) positive disciplinary styles, 3) monitoring and supervisory skills, 4) child centered families, and 5) parents seeking information and support. Based on the identification of risk and protective factors associated to antisocial behavior in children, other indicators that explain the progression and generalization of these behaviors across settings have been reported though they are less reliable. In particular, it has been reported that aggressive

behavior develops within the family unit, however, it has been also reported that as the child comes into contact with other settings and agents, his aggressive behavior generalizes. The generalization of aggressive behaviors in other settings seems appears to be the antecedent of its progression to antisocial behavior through developmental stages ending in adulthood. It has been reported that before this behavior progresses toward a more severe type, antisocial behavior generalizes across settings and agents. Furthermore, lack of control of the new agents promotes the increase and maintenance of antisocial behavior. Based on the results of numerous studies conducted in other countries that have identified and described those factors associated to aggressiveness in children, and the relative absence of longitudinal research studies in Mexico that might shed reliable information on this topic, it is necessary to conduct research for identifying the existing relationships between those factors described in the bibliography and the development, evolution and maintenance of antisocial behavior in Mexican children. With these considerations in mind, the purpose of the present study is to identify and describe those factors that are associated with the occurrence and maintenance of aggressive behaviors in a sample of school Mexican children, as well as to attempt to describe the process of generalization of these behaviors to other settings, specially the school environment (the classroom and the playground). A group of 345 children from seven public grammar schools in Mexico City were studied through a three year longitudinal cohort design. The sample was divided into two main groups: those whose reported level of occurrence of aggressive behaviors fell below the 25th percentile (non-aggressive) and those whose level of reported aggressive behaviors was above the 75th percentile (aggressive), based on the use of an Aggressive Behavior Checklist. The primary data for this study was gathered by using a direct observation system to record and classify parent child interactions in the home setting, teacher-child interactions in the classroom, as well as child-peer interaction in the playground. Additional data was gathered through the application of a series of psychological assessment instruments that measured childrearing processes such as: parental stress, disciplinary styles and family social environment as well as the child's anger control, peer preference, and types of friends maintained by the child. Results seem to identify those risk factors associated to the children's aggressive behavior, as well as those risk factors that are predictive of the aggressive behavior of the parents such as the use of an explosive irritable disciplinary style. Additional protective factors associated to reduced rates of aggressive behaviors by the child were also identified, as well as factors that seem to promote the generalization of childhood aggressive behaviors to other settings. In other words, the data shows that certain risk factors are related to the child's or parent's characteristics, while others are related to the type and quality of the parent-child interaction. The most important child characteristics were inadaptability, high levels of physical anger and disobedience, all of which predicted higher rates of aggressive behaviors in those children. In relation to parental characteristics, results seem to indicate that when parents perceive themselves as barely competent in childrearing, there are more probabilities of showing higher rates of aggressive behavior in their interactions with their offsprings. In a similar fashion, higher rates of aggressive behavior in the parents seem to be associated to their perception of their restricted parental role. The type of parent-child interaction which has been emphasized by other researchers, appears in the results of this study to confirm the findings that lack of childrearing skills is a risk factor significantly associated with the child's aggressive behavior. In particular, the use of unspecified instructional commands by the parents, an explosive irritable disciplinary style, as well as higher rate of their aggressive behavior, are reliable predictors of the child's aggressive behaviors. In a similar fashion, results appear to indicate that within family interactions, the parents' aggressive behavior predicts their child's aggressive behavior in concordance with Patterson's Coercion Theory, which states that coercive parent-child interactions increase the overall level of aggressive behavior in those families. Other risk factors were identified as a product of the analysis of the longitudinal data. They were those that reliably predict the parents' aggressive behavior, and which can also be classified into three general categories: child's characteristics, parental characteristics, and parent-child interaction. In terms of the child's characteristics, data shows that child's aggression and disobedience at home promote parental aggressive behavior. The parents' perception of certain child characteristics (problems in adaptability, demandness, distractibility and hyperactivity) is a risk factor that predicts parental aggressive behavior towards the child. Another risk factor were the parents vague and nonspecific instructions to the child with no opportunity for his compliance. Both risk factors reliably predict parental aggressiveness. Parental perception of his highly restricted parenting role is also reliably associated with higher levels of parental aggressive behavior. One of the identified risk factors in this study refers to parental disciplinary styles, specifically of an explosive irritable type. Results indicate that when a child is perceived by the parent as distracted, hyperactive, demanding, humorless, with adaptability problems, and non reinforcing for the parent, there is a significant increase of the probability that the parent will use an explosive irritable disciplinary style which, in turn, will be significantly associated with the child's aggressive behavior. Parental characteristics are also predictive of the parent's explosive irritable disciplinary style, in particular low childrearing competency, social isolation, reduced parental bonding family health problems,

perceived parental role restriction, depression, and low levels of spousal support. The findings also identified those factors associated with reduced levels of childhood aggression which seem to protect them against other child behavior problems. These factors include certain family characteristics and parental disciplinary styles that are reliably associated with reduced levels of childhood aggression, specifically, family environment with adequate levels of organization, goal orientation, religiousness and cohesiveness linked to a consistent and flexible disciplinary style not affected by emotional states. Appropriate levels of supervision of the child's activities outside the home setting favor the child's social and family development. The identification of these risk and protective factors points towards the relevance and need of developing specific interventions geared not only at the reduction of risk factors, but also towards the development of intervention strategies that promote the development of familial protective factors such as parent training in skills such as: childrearing, communication, supervision, social interaction, planned activities and incidental teaching. Additionally child-focused interventions in social skills, anger management and problem solving skills are also required along with setting-based intervention packages such as classroom and playground behavior management
SN - 0185-3325
AD - (Ayala Velazquez, Pedroza Cabrera, Morales Chaine, Chaparro Caso-Lopez, Barragan Torres) Facultad de Psicologia, Univ. Nacional Autonoma de Mexico, Cd. Universitaria, Av. Universidad # 3004, 04510 Mexico D.F., Mexico
ER -

TY - JOUR
ID - 3890
T1 - Expectancies, values, and perceptions of physical competence of children with and without learning disabilities
A1 - Shapiro,D.R.
A1 - Ulrich,D.A.
Y1 - 2002///
N1 - 2002264288
English
Journal: Article
KW - EMBASE
KW - Adolescent
KW - article
KW - Female
KW - Human
KW - learning disorder
KW - major clinical study
KW - Male
KW - Motor Performance
KW - Perception
KW - Physical Activity
KW - Physical Education
KW - psychometry
KW - questionnaire
KW - school child
KW - Self Concept
KW - sex difference
RP - NOT IN FILE
SP - 318
EP - 333
JF - Adapted Physical Activity Quarterly
JA - Adapt Phys Act Q
VL - 19
IS - 3
CY - United States
N2 - This study examined the relationship between components of Eccles' (Eccles et al., 1983) expectancy-value model and perceptions of physical competence of children with and without learning disabilities (LD) across three physical activity contexts (physical education class, outdoor school recess, and at home).

Participants, 60 children with and without LD between 10 and 13 years, completed the Modified Pictorial Scale of Perceived Physical Competence (Ulrich & Collier, 1990) and an expectancy-value questionnaire measuring perceived importance, usefulness, enjoyment, and gender orientation of selected motor skills. Gender differences in perceptions of physical competence were found in recess and home settings. No significant group differences were observed in perceptions of physical competence. Expectancy-value subscales contributed differently to understanding competence judgments of boys and girls across context. Results are discussed with implications for improving self-concept and expectancy-value among girls toward sport and physical activity
SN - 0736-5829
AD - (Shapiro) Georgia State University, Atlanta, GA 30303, United States (Ulrich) University of Michigan (Shapiro) Department of Kinesiology and Health, University Plaza, Georgia State University, Atlanta, GA 30303, United States (Ulrich) Division of Kinesiology, University of Michigan
ER -

TY - JOUR
ID - 3891
T1 - Acute, unintentional pediatric brodifacoum ingestions
A1 - Shepherd,G.
A1 - Klein-Schwartz,W.
A1 - Anderson,B.D.
Y1 - 2002///
N1 - 2002232701
English
Journal: Article
KW - EMBASE
KW - acute toxicity/di [Diagnosis]
KW - acute toxicity/dt [Drug Therapy]
KW - article
KW - blood clotting disorder
KW - Child
KW - Death
KW - detoxification
KW - disease severity
KW - emergency health service
KW - Home Care
KW - Human
KW - ingestion
KW - major clinical study
KW - parent counseling
KW - symptomatology
KW - Treatment Outcome
KW - activated carbon/cb [Drug Combination]
KW - activated carbon/do [Drug Dose]
KW - activated carbon/dt [Drug Therapy]
KW - brodifacoum/to [Drug Toxicity]
KW - emetic agent/dt [Drug Therapy]
KW - ipecac/dt [Drug Therapy]
KW - laxative/cb [Drug Combination]
KW - laxative/dt [Drug Therapy]
KW - phytomenadione/dt [Drug Therapy]
KW - rodenticide/to [Drug Toxicity]
KW - warfarin
RP - NOT IN FILE
SP - 174
EP - 178
JF - Pediatric Emergency Care
JA - Pediatr Emerg Care
VL - 18

IS - 3
CY - United States
N2 - Background: Brodifacoum is the major rodenticide used in the United States today. It is similar to warfarin but has more potent and prolonged effects. Large overdoses and chronic intoxication have been associated with significant coagulopathies and death. Currently, the management of acute unintentional ingestions by young children is controversial. Methods: American Association of Poison Control Centers (AAPCC) data from 1993 to 1996 were retrospectively searched for acute, unintentional brodifacoum exposures without coingestions followed-up to a known outcome in children aged 6 years and younger. The cases were analyzed by management site, symptoms, therapy, and outcome. Cases coded as having clinical or laboratory evidence of coagulopathy were further evaluated for severity. Results: Our study reviewed 10,762 cases that involved single, acute, unintentional ingestions of brodifacoum. All of the patients were followed-up to a known outcome as defined by AAPCC data collection standards. In this cohort, there were no deaths or major effects reported. Although 67 patients reported evidence of coagulopathy, no major effects or deaths were reported. Minor and moderate effects were reported in 38 and 54 children, respectively. Management occurred outside of a healthcare facility in 5404 (50.2 %) cases. Approximately half of all the children received some form of gastrointestinal decontamination. Decontamination had no effect on the distribution of outcomes. Adverse effects from decontamination therapy were reported in 42 patients. Conclusion: Acute pediatric ingestions of brodifacoum rarely caused clinical effects and were not associated with life-threatening symptoms or death in young children. It seems reasonable that acute unintentional ingestions of small quantities of brodifacoum by young children can be adequately managed with home observation and parent education
SN - 0749-5161
AD - (Shepherd, Klein-Schwartz, Anderson) Maryland Poison Center, University of Maryland, School of Pharmacy, Baltimore, MD, United States (Shepherd) North Texas Poison Center, 5201 Harry Hines Boulevard, Dallas, TX 75235, United States
ER -

TY - JOUR
ID - 3892
T1 - Comparison of P-glycoprotein expression with in vitro drug resistance profile in acute leukemias
A1 - Styczynski,J.
A1 - Wysocki,M.
A1 - Debski,R.
A1 - Kubicka,M.
A1 - Balwierz,W.
A1 - Juraszewska,E.
A1 - Moryl-Bujakowska,A.
A1 - Rokicka-Milewska,R.
A1 - Malinowska,I.
A1 - Matysiak,M.
A1 - Stanczak,E.
A1 - Balcerska,A.
A1 - Ploszynska,A.
A1 - Kowalczyk,J.R.
A1 - Stefaniak,J.
A1 - Malek,U.
A1 - Wachowiak,J.
A1 - Mazur,B.
A1 - Sonta-Jakimczyk,D.
A1 - Szczepanski,T.
A1 - Chybicka,A.
A1 - Ras,M.
Y1 - 2002///
N1 - 2002216878
Polish
Journal: Article
KW - EMBASE
KW - acute leukemia/dr [Drug Resistance]

KW - article
KW - blast cell
KW - cancer recurrence
KW - cancer resistance
KW - Child
KW - clinical article
KW - correlation analysis
KW - cytotoxicity
KW - Female
KW - Flow Cytometry
KW - Human
KW - Male
KW - protein expression
KW - 4 hydroperoxycyclophosphamide/pd [Pharmacology]
KW - 4 hydroperoxyifosfamide/pd [Pharmacology]
KW - antineoplastic agent/pd [Pharmacology]
KW - asparaginase macrogol/pd [Pharmacology]
KW - biodribine
KW - cladribine/pd [Pharmacology]
KW - cytarabine/pd [Pharmacology]
KW - daunorubicin/pd [Pharmacology]
KW - dexamethasone/pd [Pharmacology]
KW - dexaven
KW - doxorubicin/pd [Pharmacology]
KW - etoposide/pd [Pharmacology]
KW - fludarabine/pd [Pharmacology]
KW - fludarabine phosphate
KW - glycoprotein P/ec [Endogenous Compound]
KW - idarubicin/pd [Pharmacology]
KW - mercaptopurine/pd [Pharmacology]
KW - mitoxantrone/pd [Pharmacology]
KW - prednisolone/pd [Pharmacology]
KW - prednisolone sodium tetrahydrophthalate/pd [Pharmacology]
KW - teniposide/pd [Pharmacology]
KW - tioguanine/pd [Pharmacology]
KW - vincristine/pd [Pharmacology]
RP - NOT IN FILE
SP - 381
EP - 387
JF - Pediatria Polska
VL - 77
IS - 5
CY - Poland
N2 - Multidrug resistance implicates decreased sensitivity of cancer cells to various, unrelated drugs. The "classical" mechanism of resistance is the upregulation of PGP (permeability glycoprotein), product of the MDR1 gene, which plays a physiological role in ATP-dependent transport of various substances outside the cell. The scope of this study is the analysis of PGP expression in childhood acute leukemias and its correlation with in vitro drug resistance profile. Fourty-five children were enrolled in the study. PGP expression was determined as a percentage of blast cells by flow cytometry and drug resistance profile by means of the MTT assay. Cytotoxicity was measured as the concentration of drug that is lethal to 50% of cells. Median expression of PGP was: 0.24% for ALL de novo patients; 1.89% for relapsed ALL (p=0,002) and 12.60% in AML de novo (p<0.001). The highest individual PGP expression was observed in relapsed AML child (70.5%). The value of PGP expression showed a strong correlation (p<0.01) with resistance to idarubicin, mitoxantrone, etoposide, prednisolone, dexamethasone and type of leukemia. A weaker correlation (p<0.05) was observed for daunorubicin, doxorubicin, mercaptopurine, cladribine, fludarabine, 4-HOO-cyclophosphamide and 4-HOO-ifosfamide. No correlation was found between PGP expression and combined resistance profile for prednisolone, vincristine and L-asparaginase expressed by PVA score. These results might confirm that PGP

expression in childhood acute leukemias is related not only to resistance to anthracyclines, epipodophillotoxines and vinca alkaloids, but also to glucocorticoids, antimetabolites and alkylating agents. This is especially seen in AML and relapsed ALL
SN - 0031-3939
AD - (Styczynski, Wysocki, Debski, Kubicka, Balwierz, Juraszewska, Moryl-Bujakowska, Rokicka-Milewska, Malinowska, Matysiak, Stanczak, Balcerska, Ploszynska, Kowalczyk, Stefaniak, Malek, Wachowiak, Mazur, Sonta-Jakimczyk, Szczepanski, Chybicka, Ras) ul. Gersona 17-8, 85-305 Bydgoszcz, Poland
ER -

TY - JOUR
ID - 3893
T1 - Knowledge, attitude, and behavior of secondary (high) school students concerning HIV/AIDS in Enugu, Nigeria, in the year 2000
A1 - Nwokocha,A.R.C.
A1 - Nwakoby,B.A.N.
Y1 - 2002///
N1 - 2002205434
English
Journal: Article
KW - EMBASE
KW - acquired immune deficiency syndrome
KW - Adolescent
KW - article
KW - Attitude
KW - Child Behavior
KW - Curriculum
KW - Disease Transmission
KW - Education
KW - Female
KW - Human
KW - Human immunodeficiency virus infection
KW - information
KW - major clinical study
KW - Male
KW - mass medium
KW - Nigeria
KW - priority journal
KW - school
KW - school child
KW - Sexual Behavior
KW - sexual education
RP - NOT IN FILE
SP - 93
EP - 96
JF - Journal of Pediatric and Adolescent Gynecology
VL - 15
IS - 2
CY - United States
N2 - Study Objective: To investigate the knowledge, attitude, and behavior of secondary (high) school students concerning HIV/AIDS as a way of assessing the need for inclusion of reproductive health education in the school curriculum. Design: A questionnaire was constructed to expose depth of knowledge and attitude of respondents concerning HIV/AIDS. Setting: This was a workshop venue, a civic center, outside a school compound. Participants: Three hundred and sixty students were randomly selected from 5 secondary (high) schools in Enugu, Nigeria. Intervention: The questionnaire was administered just before the commencement of a workshop. Main Outcome Measure: All the students responded to the questionnaire but none responded to all the questions. Results: The students had a defective knowledge of the disease. They were aware and afraid of the disease as being deadly but not sure of the cause, nature, or modes of transmission and prevention, except

that illicit sexual activity should be avoided. Their attitude and behavior were consequently defective. Their main source of information was the media. Conclusion: There is need to provide students with correct, detailed, and broad-based information on reproductive health as part of the school curriculum to help them acquire adequate knowledge and develop appropriate attitude and behavior towards HIV/AIDS and other STDs
SN - 1083-3188
AD - (Nwokocha) Department of Pediatrics, University of Nigeria Teaching Hospital, Enugu, Nigeria (Nwakoby) Department of Community Medicine, University of Nigeria Teaching Hospital, Enugu, Nigeria
ER -

TY - JOUR
ID - 3894
T1 - Lack of somatic hypermutation of IG VH genes in lymphoid malignancies with t(2;14)(p13;q32) translocation involving the BCL11A gene
A1 - Kuppers,R.
A1 - Sonoki,T.
A1 - Satterwhite,E.
A1 - Gesk,S.
A1 - Harder,L.
A1 - Oscier,D.G.
A1 - Tucker,P.W.
A1 - Dyer,M.J.S.
A1 - Siebert,R.
Y1 - 2002///
N1 - 2002183683
English
Journal: Article
KW - EMBASE
KW - Adult
KW - article
KW - B cell lymphoma/di [Diagnosis]
KW - B lymphocyte activation
KW - Child
KW - chromosome aberration
KW - chromosome translocation 13
KW - chromosome translocation 14
KW - chromosome translocation 2
KW - chronic lymphatic leukemia/di [Diagnosis]
KW - clinical article
KW - controlled study
KW - gene amplification
KW - Gene Rearrangement
KW - gene sequence
KW - gene switching
KW - gene targeting
KW - genetic association
KW - germinal center
KW - Human
KW - human cell
KW - human tissue
KW - immune response
KW - immunocytoma/di [Diagnosis]
KW - immunoglobulin variable region
KW - lymphocyte subpopulation
KW - Lymphoma
KW - molecular cloning
KW - mutational analysis
KW - position effect

KW - priority journal
KW - proto oncogene
KW - somatic mutation
KW - T lymphocyte
KW - immunoglobulin heavy chain/ec [Endogenous Compound]
KW - oncoprotein/ec [Endogenous Compound]
RP - NOT IN FILE
SP - 937
EP - 939
JF - Leukemia
VL - 16
IS - 5
CY - United Kingdom
N2 - The t(2;14)(p13;q32.3) involving the BCL11A and IGH genes is a rare but recurrent chromosomal aberration in B-cell malignancies. Hitherto, juxtaposition of BCL11A and IGH has only been described in B-cell chronic lymphocytic leukemia (B-CLL) and immunocytoma. As subgroups of B-CLL can be distinguished by the pattern of somatic mutation of immunoglobulin variable (V) genes we investigated four lymphomas with IGH/BCL11A involvement for IGH hypermutation. Clonal V_H gene rearrangements were amplified; in all four cases, sequencing of the amplificates revealed the rearranged V_H genes to lack somatic mutations. These results suggest that t(2;14)(p13;q32.3) is associated with a subset of B-CLL/immunocytoma characterized by non-mutated IG genes deriving from pre-germinal center B cells. As the translocations in both informative cases are targeted to the switch regions of the IGG2 gene, which is mainly used in T cell-independent immune responses, these translocations presumably occurred in activated B cells in the course of T cell-independent immune responses outside the germinal center
SN - 0887-6924
AD - (Kuppers, Sonoki, Satterwhite, Gesk, Harder, Oscier, Tucker, Dyer, Siebert) University of Cologne, Department of Internal Medicine I, Institute for Genetics, Joseph-Stelzmannstr. 9, D-50931 Cologne, Germany
ER -

TY - JOUR
ID - 3895
T1 - The effects of playground markings on the energy expenditure of 5-7-year-old school children
A1 - Stratton,G.
A1 - Leonard,J.
Y1 - 2002///
N1 - 2002183423
English
Journal: Article
KW - EMBASE
KW - article
KW - body mass
KW - Child
KW - controlled study
KW - Energy Expenditure
KW - Female
KW - Heart Rate
KW - Human
KW - human experiment
KW - Male
KW - normal human
KW - Physical Activity
KW - play
KW - recumbency
KW - Running
KW - sitting
KW - Urban Population
KW - Walking

RP - NOT IN FILE
SP - 170
EP - 180
JF - Pediatric Exercise Science
JA - Pediatr Exerc Sci
VL - 14
IS - 2
CY - United States
N2 - The energy expenditure of 47 children aged 5-7 years was assessed before and after a school playground was painted with fluorescent markings. Physical activity was measured using heart rate monitors and energy expenditure calculated for 3 playtimes per child before and after the playground was painted. Total energy expenditure and the rate of energy expenditure increased significantly, as did the duration of play. The effect of painting the playground on total energy expenditure was analysed using an ANCOVA to control for play duration and body mass. Results revealed a 35% increase in total energy expenditure (P < .01) and a 6% increase in the rate of energy expenditure (P < .01). The significant interaction between time (before and after) and group (experimental and control) (P < .02) demonstrated that the intervention programme significantly increased heart rates. These results suggest that playground markings and duration of play can have a significant and positive influence on young children's energy expenditure
SN - 0899-8493
AD - (Stratton, Leonard) Ctr. Phys. Educ. Sport and Dance, I.M. Marsh Campus, Liverpool John Moores University, Liverpool, United Kingdom
ER -

TY - JOUR
ID - 3896
T1 - Institutional psychotherapy: Model of crisis management to provoke changes?
A1 - Albert,E.
A1 - Bouchaud,P.
A1 - Frottin,A.
A1 - Guberina,M.
A1 - Brunel,M.
Y1 - 2002///
N1 - 2002131963
French
Journal: Review
KW - EMBASE
KW - Child
KW - Child Psychiatry
KW - consultation
KW - crisis intervention
KW - health care personnel
KW - health care planning
KW - Human
KW - Psychotherapy
KW - review
KW - Treatment Planning
RP - NOT IN FILE
SP - 163
EP - 167
JF - Neuropsychiatrie de l'Enfance et de l'Adolescence
VL - 50
IS - 2
CY - France
N2 - The therapeutic experience inside the institution disposing of a day-care hospital and a night hospital, leads us to conclude that there is no possible therapeutic act without moments of crisis, both inside the institutional dynamic as in the child's itinerary. The institutional device proposes a complex figuration whose aim is to avoid the relation of the emprise and to favor the quest towards the incertitude: the maintenance of the

main consultant outside the institution; inscription of the children in the heterogeneous age groups; favoring fictional affiliation; children's circulation from one place to another, permitting a confrontation with reality and the process of the constructive bereavement; unequal therapeutic periods (full time or part time) that incite the therapeutic staff to a constant investment; externalized classes that help avoiding creation of the culture of exclusion. The therapeutic action includes a series of punctuations that will mark the time of the child's cure. Thus, the child itinerary is permeated with transitional periods, sources of anxiety during which the child will mobilize as soon as he encounters a therapeutic frame that favors the activity of the representation. The child will thus narcissizes himself by discovering that he can survive the changes and benefit from them. The institutional crises appears thus as a paradigm of child's cure even if the institutional actors are always taken by surprise by this phenomenon while dreaming of a solidarity therapeutic community. 2002 Editions scientifiques et mdicales Elsevier SAS
SN - 0222-9617
AD - (Albert, Bouchaud, Frottin, Guberina, Brunel) Centre De Psychotherapie Et De Reeducation, 46-48, avenue Albert Ier, 60300-Senlis, France
ER -

TY - JOUR
ID - 3897
T1 - Benefit of school-based exercise tests and educational programs to screen for undiagnosed asthma
A1 - Charton,C.
A1 - Droulers,I.
A1 - Girault,E.
A1 - Laurent,C.
A1 - Housset,B.
A1 - Delacourt,C.
Y1 - 2002///
N1 - 2002105411
French
Journal: Article
KW - EMBASE
KW - airway obstruction/di [Diagnosis]
KW - article
KW - Asthma/di [Diagnosis]
KW - Asthma/ep [Epidemiology]
KW - bronchus hyperreactivity/di [Diagnosis]
KW - Education
KW - exercise induced asthma/di [Diagnosis]
KW - exercise induced asthma/ep [Epidemiology]
KW - Exercise Test
KW - Female
KW - Health Education
KW - Human
KW - lung function test
KW - major clinical study
KW - Male
KW - Mass Screening
KW - peak expiratory flow
KW - school child
KW - scoring system
RP - NOT IN FILE
SP - 245
EP - 254
JF - Archives de Pediatrie
JA - Arch Pediatr
VL - 9
IS - 3
CY - France

N2 - Underdiagnosis of asthma is frequent in children and may be improved by the development of school-based health programs. Material and methods. - We developed an educational program in 11-year-old schoolchildren who participated in a screening test for exercise-induced asthma (EIA). All children were given an asthma questionnaire before and after two educational sessions. Results. - Mean score for asthma knowledge quiz increased from 63 to 85 % (p< 0.001). Thirty-six children (3.7 %) were initially considered as asthmatic and showed better responses than non-asthmatic children. Peak expiratory flow (PEF) was measured before and after an outside running exercise. A fall in PEF of at least 15 % was considered an abnormal result. Seventy-height children (8 %) had a decrease in PEF, including 65 children initially not recognized as asthmatic. For these latter, a medical evaluation was recommended to parents. Only 28 of these children gave informations on follow-up: 19 had lung function tests, two received a treatment without preliminary function test, and seven had neither lung function test nor treatment. Among the children who realized lung function tests, five had criteria for airway obstruction at baseline, and ten had significant bronchial hyperreactivity. Conclusions. - However, a new screening test revealed that only a small minority of children initially not recognized as asthmatic but having decreased their PEF, were consequently considered as asthmatic by their practitioner, even in case of positive lung function test. 2002 Editions scientifiques et medicales Elsevier SAS
SN - 0929-693X
AD - (Charton, Droulers, Girault, Laurent) Service de Promotion de la Sante en Faveur des Eleves, Inspection Academique du Val-de-Marne, 68, Avenue du General-de-Gaulle, 94010 Creteil, France (Housset) Service de Pneumologie, Centre Hospitalier Intercommunal, 40, Avenue de Verdun, 94000 Creteil, France (Delacourt) Service de Pediatrie, Centre Hospitalier Intercommunal, 40, Avenue de Verdun, 94000 Creteil, France
ER -

TY - JOUR
ID - 3898
T1 - Where there are no data: What has happened to life expectancy in Georgia since 1990?
A1 - Badurashvili,I.
A1 - McKee,M.
A1 - Tsuladze,G.
A1 - Mesle,F.
A1 - Vallin,J.
A1 - Shkolnikov,V.
Y1 - 2001///
N1 - 2001424791
English
Journal: Article
KW - EMBASE
KW - Adolescent
KW - Adult
KW - Aged
KW - article
KW - cancer mortality
KW - Child
KW - controlled study
KW - Death
KW - fee
KW - Female
KW - Government
KW - health survey
KW - household
KW - Human
KW - Infant
KW - Infant Mortality
KW - Life Expectancy
KW - life table
KW - Male
KW - migration
KW - Mortality

KW - parameter
KW - Population
KW - publication
KW - registration
KW - Russian Federation
KW - statistical model
KW - Statistics
KW - Time
KW - USSR
RP - NOT IN FILE
SP - 394
EP - 400
JF - Public Health
VL - 115
IS - 6
CY - United Kingdom
N2 - In recent years there has been a considerable increase in understanding of changes in mortality in Russia and some other former Soviet republics. However, the situation in the republics of the Caucasus remains poorly understood. Information on Georgia is especially fragmentary as a fifth of the country remains outside government control, there has been large scale migration since 1991, and the introduction of fees for vital registration has compromised the quality of official statistics. The aim of the study is to produce plausible estimates for life expectancy in Georgia for the period 1990-1998 and thus to assess whether Georgia has undergone changes similar to other former Soviet republics in the post-independence period. Four models were used to construct life tables. Model 1 used officially published statistics on deaths and population. Model 2 applied new estimates of population derived from household surveys to the observed deaths. Model 3 adjusted model 2 for under-registration at extremes of life, with parameter estimates derived from a survey of infant mortality and comparison of observed rates with Coale-Demeny standard life tables. Model 4 arose following inspection of death rates by cause that revealed implausible discontinuities in cancer mortality rates and involved applying the estimates of under-registration that this finding implied to model 3. The four models produce quite different estimates of life expectancy, differing by 7.8 y for men and 6.8 y for women by 1998. In any of the models, however, Georgia does not appear to have experienced the marked deterioration in life expectancy seen in Russia following the transition to independence. Importantly, Georgia had also not experienced a marked improvement in life expectancy during the 1985 Soviet anti-alcohol campaign, again unlike other Soviet republics. Official statistics substantially over-estimate life expectancy at birth in Georgia. Despite undergoing a civil war, life expectancy in Georgia has been less affected by the transition than has Russia and the overall trends in mortality since the mid 1980s suggest that this may be because alcohol has played a smaller role in these changes than it did in Russia
SN - 0033-3506
AD - (Badurashvili, Tsuladze) Georgian Centre of Population Research, Tbilisi, Georgia (McKee) European Centre On Health of Societies In Transition, London School of Hygiene and Tropical Medicine, London, United Kingdom (Mesle, Vallin) Institut National d'Etudes Demographiques, Paris, France (Shkolnikov) Max Planck Institute for Demographic Research, Rostock, Germany (McKee) European Centre On Health of Societies In Transition, London School of Hygiene and Tropical Medicine, Keppel Street, London WC1E 7HT, United Kingdom
ER -

TY - JOUR
ID - 3899
T1 - Blastomycosis: More evidence for exposure near one's domicile
A1 - Baumgardner,D.J.
A1 - Paretsky,D.P.
Y1 - 2001///
N1 - 2001404483
English
Journal: Article
KW - EMBASE
KW - article

KW - Blastomyces dermatitidis
KW - blastomycosis/et [Etiology]
KW - Case Report
KW - cat
KW - dog
KW - exposure
KW - fungus culture
KW - Human
KW - Male
KW - nonhuman
KW - school child
RP - NOT IN FILE
SP - 43
EP - 45
JF - Wisconsin Medical Journal
JA - Wis.Med J
VL - 100
IS - 7
CY - United States
N2 - Background - Our previous publications on the epidemiology of blastomycosis suggested that the etiologic organism, Blastomyces dermatitidis, may be acquired at home, however this view was challenged in an editorial. Methods - 1) Field study of 2 properties that preliminarily suggested disease acquisition in the home. Owner interviews, site visits and environmental cultures using our in-vitro technique were used. 2) An address registry of human and dog blastomycosis cases was constructed from extensions of our previously published case series. 3) Literature review. Results - 1) Blastomycosis occurred in a dog (December, 1998) and then a cat confined to its home (September, 1999), from a household in urban Manitowoc County, WI; and additionally in a houseconfined cat (July, 1998) at a home in Milwaukee, WI. Interviews implicated the basement and the attic or basement, respectively, as the most likely source of infection at these homes. Environmental cultures were negative for Blastomyces.² Of the 229 domiciles in the registry, a minimum of 27 (12%) were associated with more than one blastomycosis case, 10 sites with more than two and 7 with more than three. In 4 domiciles, repeat cases occurred in different families. Most cases were separated by 1 year or more (range: 3 weeks to 7 years).³ Recent case series reveal a minority of outdoor activities and occupations among humans with blastomycosis. The organism has been isolated from an inhabited yard and from a house being razed. Conclusions - There appears to be growing evidence that blastomycosis may be acquired at home, and that B. dermatitidis may be relatively persistent on certain properties
SN - 1098-1861
AD - (Baumgardner, Paretsky) Aurora Health Care, 2801 West KK River Pkwy, Milwaukee, WI 53215; 414.649.7909, United States
ER -

TY - JOUR
ID - 3901
T1 - Ethologic observation in day-care to a young autistic boy with tetrasomy 15q
A1 - Coq,J.M.
Y1 - 2001///
N1 - 2001344985
French
Journal: Article
KW - EMBASE
KW - article
KW - autism/et [Etiology]
KW - Case Report
KW - Child Behavior
KW - chromosomal localization
KW - chromosome 15q
KW - chromosome aberration
KW - developmental disorder/et [Etiology]

KW - Human
KW - Male
KW - preschool child
KW - Social Environment
KW - tetrasomy
RP - NOT IN FILE
SP - 337
EP - 342
JF - Neuropsychiatrie de l'Enfance et de l'Adolescence
VL - 49
IS - 5-6
CY - France
N2 - A 21 month young boy who shows autistic disorders, and who will reaveal oneself tetrasomy 15q carrier, is observed with an ethological method in the day-care he frequented during 10 months. After a first three months in which autistic behaviours and an extreme retreat, this child enrich progressively his gestual expressions'repertoire and shows less indifferent to his environment. Moreover he increases his moving area, investing gradually day-care's playgrounds and putting up with the other children and adults easier. So he reveals potential evolutions in spite of extremely serious developmental disorders. 2001 Editions scientifiques et medicales Elsevier SAS
SN - 0222-9617
AD - (Coq) Psychologue, 69, boulevard Soult, 75012 Paris, France
ER -

TY - JOUR
ID - 3902
T1 - Intensive care medicine as essential in-door and out-door competition
A1 - Ruiz,J.
A1 - Martin,M.C.
A1 - Garcia,A.
A1 - Nolla,Y.M.
Y1 - 2001///
N1 - 2001338991
Spanish
Journal: Conference Paper
KW - EMBASE
KW - Adolescent
KW - Adult
KW - Aged
KW - Child
KW - clinical practice
KW - Competition
KW - conference paper
KW - consultation
KW - critical illness
KW - Female
KW - Human
KW - intensive care
KW - major clinical study
KW - Male
KW - medical documentation
KW - patient care
KW - patient transport
KW - productivity
KW - Spain
RP - NOT IN FILE
SP - 137
EP - 144

JF - Medicina Intensiva
VL - 25
IS - 4
CY - Spain
N2 - Background. Intensive care medicine productivity has not been quantified in Spain. The aims of this study were 2-fold: a) to evaluate the productivity of staff physicians (in-door), and b) to evaluate the productivity of the intensive care unit (ICU) (out-door). Material and methods. In-door productivity was analyzed in the ICU of a center with 260 beds. To evaluate in-door productivity the daily activities performed by the staff physicians were chronologically described and analyzed: planning of daily activity, contact with external departments, consultation with patients, special techniques and inter-consulting, informing families, transfers out of the ICU, clinical documentation and clinical sessions, duty shifts and other activities. To analyze out-door productivity, a univariate identification was carried out of the number of consultations per department which were adjusted for calendar (working days of weekends and public holidays) and timetable (morning, afternoon or night nursing shifts), type of consultation, care of post-critical patients and transfers. Results. The results for in-door and out-door productivity showed that the two ICUs studied were a source of advantage, generating value among the community. The results also indicate that intensive care medicine fits within the strategic concept of essential competition. Conclusions. ICUs are ideal for meeting the social and economic needs of the community by attending critically-ill patients within a hospital
SN - 0210-5691
AD - (Ruiz, Martin, Garcia, Nolla) Servei de Medicina Intensiva, Hospital del Sagrat Cor, Viladomat, 288, 08029 Barcelona, Spain
ER -

TY - JOUR
ID - 3903
T1 - Psychomotor evaluation, therapeutic process verifications and modifications in family and social environment
A1 - Russo,R.C.
Y1 - 2001///
N1 - 2001338907
Italian
Journal: Review
KW - EMBASE
KW - Adolescent
KW - Child
KW - Child Behavior
KW - Child Development
KW - Child Psychology
KW - Family
KW - Human
KW - psychologic assessment
KW - psychomotor activity
KW - review
KW - Social Environment
RP - NOT IN FILE
SP - 137
EP - 154
JF - Imago
VL - 8
IS - 2
CY - Italy
N2 - This paper focuses the problem of psychomotor evaluation in children and adolescents in the presence of significant disorders in comparison with a norm. Knowing the child history and its events, the characteristics of parental patterns and the peculiarity of relation allows the understanding of reasons and pathogenetic dynamics which caused and support child's disorder or pathology. Theraphy must be carried out with a constant confrontation between the therapeutic process inside the setting and the child's behaviour modifications in the outside family and social environment, as well as pointing to know the way patterns react to modifications;

these factors have inevitable repercussions on setting, requiring possible adjustments. So it is indispensable to regard the evaluation as a non-static intervention to be done only before the beginning of the therapy, and as a task to be frequently accomplished in a dynamic way, with the primary purpose to take into account the child's family and social world, the area in which improvements are expected and the proper adjustments relevant to the figures the child refers to in his development
SN - 1124-3031
AD - (Russo) Centro Studi di Psicomotricita, Milano, Italy
ER -

TY - JOUR
ID - 3905
T1 - The origins of a new Trypanosoma brucei rhodesiense sleeping sickness outbreak in eastern Uganda
A1 - Fevre,E.M.
A1 - Coleman,P.G.
A1 - Odiit,M.
A1 - Magona,J.W.
A1 - Welburn,S.C.
A1 - Woolhouse,M.E.J.
Y1 - 2001///
N1 - 2001303945
English
Journal: Article
KW - EMBASE
KW - Adolescent
KW - Adult
KW - African trypanosomiasis/ep [Epidemiology]
KW - African trypanosomiasis/et [Etiology]
KW - African trypanosomiasis/pc [Prevention]
KW - Aged
KW - article
KW - case control study
KW - Child
KW - Cluster Analysis
KW - controlled study
KW - Disease Transmission
KW - epidemic
KW - Female
KW - Human
KW - Infant
KW - Infection Control
KW - major clinical study
KW - Male
KW - market
KW - parasite transmission
KW - priority journal
KW - Risk Assessment
KW - risk factor
KW - Trypanosoma brucei
KW - Trypanosoma gambiense
KW - Trypanosoma rhodesiense
KW - tsetse fly
KW - Uganda
KW - zoonosis
RP - NOT IN FILE
SP - 625
EP - 628
JF - Lancet

VL - 358
IS - 9282
CY - United Kingdom
N2 - Background: Sleeping sickness, caused by two trypanosome subspecies, Trypanosoma brucei gambiense and Trypanosoma brucei rhodesiense, is a parasitic disease transmitted by the tsetse fly in sub-Saharan Africa. We report on a recent outbreak of T b rhodesiense sleeping sickness outside the established south-east Ugandan focus, in Soroti District where the disease had previously been absent. Soroti District has been the subject of large-scale livestock restocking activities and, because domestic cattle are important reservoirs of T b rhodesiense, we investigated the role of cattle in the origins of the outbreak. Methods: We identified the origins of cattle entering the outbreak area in the 4 years preceding the outbreak. A matched case-control study was conducted to assess whether the distance of villages from the main market involved with restocking was a risk factor for sleeping sickness. We investigated the spatial clustering of sleeping sickness cases at the start of the outbreak. Findings: Over 50% (1510 of 2796) of cattle traded at the market were reported to have originated from endemic sleeping sickness areas. The case-control study revealed that distance to the cattle market was a highly significant risk factor for sleeping sickness (p<0.001) and that there was a significant clustering of cases (27 of 28) close to the market at the start of the outbreak (p<0.001). As the outbreak progressed, the average distance of cases moved away from the cattle market (0.014 km per day, 95% CI 0.008-0.020 km per day, p<0.001). Interpretations: The results are consistent with the disease being introduced by cattle infected with T b rhodesiense imported to the market from the endemic sleeping sickness focus. The subsequent spread of the disease away from the market suggests that sleeping sickness is becoming established in this new focus. Public health measures directed at controlling the infection in the animal reservoir should be considered to prevent the spread of sleeping sickness
SN - 0140-6736
AD - (Fevre, Coleman, Odiit, Welburn, Woolhouse) Centre for Tropical Veterinary Medicine, University of Edinburgh, Easter Bush, Roslin, Midlothian, EH25 9RG, United Kingdom (Odiit, Magona) Livestock Health Research Institute, PO Box 96, Tororo, Uganda
ER -

TY - JOUR
ID - 3906
T1 - Social comparisons of children with and without learning disabilities when evaluating physical competence
A1 - Shapiro,D.R.
A1 - Ulrich,D.A.
Y1 - 2001///
N1 - 2001235134
English
Journal: Article
KW - EMBASE
KW - Adolescent
KW - article
KW - clinical article
KW - Competence
KW - controlled study
KW - Evaluation
KW - Female
KW - Human
KW - learning disorder/ep [Epidemiology]
KW - Male
KW - Physical Activity
KW - Physical Education
KW - physical performance
KW - questionnaire
KW - school child
KW - social aspect
KW - statistical analysis
RP - NOT IN FILE

SP - 273
EP - 288
JF - Adapted Physical Activity Quarterly
JA - Adapt Phys Act Q
VL - 18
IS - 3
CY - United States
N2 - The purpose was to examine the reference groups used by children with and without learning disabilities (LD), ages 10-13 years, when judging perceived physical competence in three contexts (in physical education class, during outdoor school recess, and at home). Participants, 30 students with LD and 30 without LD, completed the athletic competence subscale from the Self-Perception Profile for Learning Disabled Students (SPPLD; Renick & Harter, 1988) and two social comparison questionnaires in each activity context. Differences in the percentage of students citing the various reference groups across context was not statistically significant. Observations of responses indicate participants relied primarily on classmates, self-comparison, and family members to judge their physical competence. These results suggest that, while students with and without LD tend to compare themselves with their general education classmates, their reliance on eight different social comparison groups from which to judge physical competence varies with context
SN - 0736-5829
AD - (Shapiro, Ulrich) Department of Kinesiology, Georgia State University, University Plaza, Atlanta, GA 30303, United States
ER -

TY - JOUR
ID - 3907
T1 - Dietary advice for obese children
A1 - Cassuto,D.-A.
Y1 - 2001///
N1 - 2001210342
French
Journal: Review
KW - EMBASE
KW - body growth
KW - Body Weight
KW - carbohydrate intake
KW - Child
KW - diet restriction
KW - fat intake
KW - Feeding Behavior
KW - Food Intake
KW - Human
KW - Nutrition
KW - Obesity/th [Therapy]
KW - patient counseling
KW - Physical Activity
KW - practice guideline
KW - review
RP - NOT IN FILE
SP - 135
EP - 141
JF - Cahiers de Nutrition et de Dietetique
VL - 36
IS - 2
CY - France
N2 - We hold nutritional counselling to be a prescription resulting from a customised nutritional advice. In the case of child obesity, the patient is not to be considered a miniature adult to whom one can prescribe a reduced food diet. Moreover, there are many solutions, not just one to the problem of obesity, just as there are many different cases of obesity. Therefore, different strategies are to be conducted, according to the etiological nature

of the obesity, its intensity and chronicity, depending also upon both the gender and the age of the child, the psychological problems and the family set-up, etc. We recommend that the preliminary dietary investigation be accompanied by a study on the patient's physical activities. Weekdays and week-end food behaviour during meals (at home and/or at school lunch-room) is to be observed, just as the patient's food intake outside of meals, the quantity, the family dietary behaviour such as cultural and cooking styles, etc., and the food choices of the other members are also to be taken into consideration when investigating obesity. Indeed, the prescription will be drawn up during the following clinical visits according to the above-cited specific parameters of the child and the dietary guideline. It is therefore necessary to have a perfect understanding of a child's dietary needs according to age. With additional advice on increasing the patient's physical activity and on fighting against sedentariness, the prescription will be made out based on positive instructions neither negating any food, nor listing authorised or forbidden food items. The identification of errors made in the earlier consultations will help in drawing up the prescription, so as to recommend a decrease of fat intake and an increase of carbohydrates, however checking that there be no increase in the total food energy balance. In practice, we can formulate a customised one-day dietary instruction with the help of the child: a diet that will comprise those food items that the child likes which also correspond to the desired criteria. The guidelines that concern both parents and children deserve a special attention. Snacking in-between-meals can play an important role in the total food balance. In a child, the disorders are seldom a form of bulimia, but are more often the result of snacking "in hiding", especially in the case of harsh and stringent restrictions during the meals or at afternoon snack, such that they entail a vicious circle of "restriction-desinhibition". Therefore, parents' attention ought to focus on meals taken at home, by varying the food in order to compensate the imbalanced generated by outdoor food intake. The first aim is to stabilise the weight of the obese child: to grow up without gaining weight results to be thiner. If the child cannot discipline himself/herself to the instructions, or if the weight goals are not be reached, the study of the psychological elements that determine the weight gain and that of the conflictual situations should be placed at the core of the therapy. At times, a study of the child's psychology has to be initiated and undertaken
SN - 0007-9960
AD - (Cassuto) Service de Medecine et Nutrition, Hotel-Dieu, 1, place du Parvis Notre-Dame, F 75181 Paris Cedex 04, France
ER -

TY - JOUR
ID - 3908
T1 - Canines as sentinel species for assessing chronic exposures to air pollutants: Part 1. Respiratory pathology
A1 - Calderon-Garciduenas,L.
A1 - Mora-Tiscareno,A.
A1 - Fordham,L.A.
A1 - Chung,C.J.
A1 - Garcia,R.
A1 - Osnaya,N.
A1 - Hernandez,J.
A1 - Acuna,H.
A1 - Gambling,T.M.
A1 - Villarreal-Calderon,A.
A1 - Carson,J.
A1 - Koren,H.S.
A1 - Devlin,R.B.
Y1 - 2001///
N1 - 2001198572
English
Journal: Article
KW - EMBASE
KW - air pollutant
KW - airway
KW - ambient air
KW - Animal
KW - animal experiment
KW - animal model

KW - animal tissue
KW - article
KW - bronchiole
KW - cell count
KW - cell damage
KW - cell infiltration
KW - cell proliferation
KW - Child
KW - city
KW - controlled study
KW - cytokine release
KW - dog
KW - electron microscopy
KW - endothelium cell
KW - epithelium cell
KW - Evaluation
KW - Female
KW - histopathology
KW - Human
KW - Inflammation
KW - interstitium
KW - long term exposure
KW - Lung
KW - lung alveolus cell
KW - lung alveolus cell type 1
KW - lung alveolus cell type 2
KW - lung blood vessel
KW - lung fibrosis
KW - lung lavage
KW - macrophage
KW - Male
KW - Mexico
KW - Microscopy
KW - microthrombus
KW - mononuclear cell
KW - muscle hypertrophy
KW - neutrophil
KW - nonhuman
KW - Particulate Matter
KW - respiratory tract disease/et [Etiology]
KW - smooth muscle
KW - species
KW - tissue repair
KW - urban area
KW - vegetation
KW - ozone/to [Drug Toxicity]
RP - NOT IN FILE
SP - 342
EP - 355
JF - Toxicological Sciences
VL - 61
IS - 2
CY - United States
N2 - A complex mixture of air pollutants is present in the ambient air in urban areas. People, animals, and vegetation are chronically and sequentially exposed to outdoor pollutants. The objective of this first of 2 studies is to evaluate by light and electron microscopy the lungs of Mexico City dogs and compare the results to those of 3 less polluted cities in Mexico. One hundred fifty-two clinically healthy stray mongrel dogs (91 males/61

females), including 43 dogs from 3 less polluted cities, and 109 from southwest and northeast metropolitan Mexico City (SWMMC, NEMMC) were studied. Lungs of dogs living in Mexico City and Cuernavaca exhibited patchy chronic mononuclear cell infiltrates along with macrophages loaded with particulate matter (PM) surrounding the bronchiolar walls and extending into adjacent vascular structures; bronchiolar epithelial and smooth muscle hyperplasia, peribronchiolar fibrosis, microthrombi, and capillary and venule polymorphonuclear leukocytes (PMN) margination. Ultrafine PM was seen in alveolar type I and II cells, endothelial cells, interstitial macrophages (M), and intravascular M-like cells. Bronchoalveolar lavage showed significant numbers of alveolar macrophages undergoing proliferation. Exposure to complex mixtures of pollutants-predominantly particulate matter and ozone-is causing lung structural changes induced by the sustained inflammatory process and resulting in airway and vascular remodeling and altered repair. Cytokines released from both, circulating inflammatory and resident lung cells in response to endothelial and epithelial injury may be playing a role in the pathology described here. Deep concern exists for the potential of an increasing rise in lung diseases in child populations exposed to Mexico City's environment
SN - 1096-6080
AD - (Calderon-Garciduenas) Curriculum in Toxicology, University of North Carolina, Chapel Hill, NC, United States (Calderon-Garciduenas, Mora-Tiscareno, Garcia, Osnaya, Hernandez, Acuna, Villarreal-Calderon) Instituto Nacional de Pediatria, Mexico City 14410, Mexico (Fordham, Chung) Department of Radiology, University of North Carolina, Chapel Hill, NC 27599-7310, United States (Gambling, Carson) Center for Environmental Medicine and Lung Biology, University of North Carolina, Chapel Hill, NC 27599-7310, United States (Koren, Devlin) National Health and Environmental Effects Research Laboratory, U.S. Environmental Protection Agency, Research Triangle Park, NC 27711, United States (Calderon-Garciduenas) Human Studies Facility, U.S. EPA, Research Triangle Park, NC 27711, United States
ER -

TY - JOUR
ID - 3909
T1 - Psychological job demands as a risk factor for common cold in a Dutch working population
A1 - Mohren,D.C.L.
A1 - Swaen,G.M.H.
A1 - Borm,P.J.A.
A1 - Bast,A.
A1 - Galama,J.M.D.
Y1 - 2001///
N1 - 2001134056
English
Journal: Article
KW - EMBASE
KW - Adult
KW - anamnesis
KW - article
KW - Asthma
KW - Child
KW - Child Care
KW - clinical feature
KW - Cohort Analysis
KW - common cold/ep [Epidemiology]
KW - controlled study
KW - daily life activity
KW - disease association
KW - Fatigue
KW - Female
KW - high risk population
KW - Human
KW - job analysis
KW - major clinical study
KW - Male
KW - Netherlands

KW - population research
KW - Prevalence
KW - prospective study
KW - psychological aspect
KW - Regression Analysis
KW - Risk Assessment
KW - Self Report
KW - Smoking
KW - work environment
KW - worker
RP - NOT IN FILE
SP - 21
EP - 27
JF - Journal of Psychosomatic Research
VL - 50
IS - 1
CY - United States
N2 - Objective: We investigated the effect of Psychological Job Demands (PJD) on the occurrence of the clinical symptoms of common cold. Methods: Subjects, participating in a large prospective cohort study on psychological determinants of fatigue at work, were asked to fill in a questionnaire on the occurrence of common cold during the previous four months. High PJD were considered as a potential risk factor. Other factors such as age, gender, and having young children were considered as potential confounders. Results: In logistic regression analysis, the adjusted odds ratio (OR) for having a recent cold in subjects reporting high PJD vs. those reporting low PJD was 1.20 (95% confidence interval (CI), 1.08-1.33). A higher risk emerged among those with young children (OR, 1.70; 95% CI, 1.47-1.96), those having a history of asthma (OR, 1.69; 95% CI, 1.28-2.22), or being under the age of 40 (OR, 1.28; 95% CI, 1.14-1.43) and among smokers (OR, 1.23; 95% CI, 1.09-1.38). Conclusion: The results support an association between PJD and common cold. In spite of the almost inevitable shortcoming of a large cohort study using questionnaires, this study gave us the opportunity to study the relationship between common cold and work-related factors in a nonexperimental setting with participants observed in a natural environment with all the normal everyday hassles. 2001 Elsevier Science Inc. All rights reserved
SN - 0022-3999
AD - (Mohren, Swaen) Department of Epidemiology, Maastricht University, PO Box 616, 6200 MD, Maastricht, Netherlands (Borm) Department of Fibre and Particle Toxicology, Medical Institute for Environmental Hygiene, Dusseldorf, Germany (Bast) Department of Pharmacology and Toxicology, Maastricht University, Maastricht, Netherlands (Galama) Department of Medical Virology, Institute of Medical Microbiology, University Hospital Nijmegen, Nijmegen, Netherlands
ER -

TY - JOUR
ID - 3910
T1 - Interactions in an inclusive classroom: The effects of visual status and setting
A1 - McGaha,C.G.
A1 - Farran,D.C.
Y1 - 2001///
N1 - 2001073213
English
Journal: Article
KW - EMBASE
KW - article
KW - disability
KW - handicapped child
KW - Human
KW - play
KW - preschool child
KW - school
KW - Social Interaction

KW - videorecording
KW - visual impairment
RP - NOT IN FILE
SP - 80
EP - 94
JF - Journal of Visual Impairment and Blindness
VL - 95
IS - 2
CY - United States
N2 - This study examined the effect of visual status (visually impaired or sighted) and setting (indoor or outdoor) on the social behaviors of preschool-age children in an inclusive setting. It found that regardless of their visual status, the children spent significantly more time near sighted children than near visually impaired children
SN - 0145-482X
AD - (McGaha, Farran) Dept. of Family/Consumer Sciences, Boone, NC 28608, United States
ER -

TY - JOUR
ID - 3912
T1 - Engagement in playground activities as a criterion for diagnosing developmental coordination disorder
A1 - Watkinson,E.J.
A1 - Dunn,J.C.
A1 - Cavaliere,N.
A1 - Calzonetti,K.
A1 - Wilhelm,L.
A1 - Dwyer,S.
Y1 - 2001///
N1 - 2001029942
English
Journal: Article
KW - EMBASE
KW - accuracy
KW - article
KW - daily life activity
KW - developmental disorder/cn [Congenital Disorder]
KW - developmental disorder/di [Diagnosis]
KW - Female
KW - Human
KW - major clinical study
KW - Male
KW - Motor Coordination
KW - motor dysfunction
KW - Physical Activity
KW - Physical Education
KW - play
KW - school child
KW - scoring system
KW - Self Report
KW - statistical analysis
KW - validation process
RP - NOT IN FILE
SP - 18
EP - 34
JF - Adapted Physical Activity Quarterly
JA - Adapt Phys Act Q
VL - 18
IS - 1

CY - United States
N2 - The purpose was to develop a valid protocol for use by physical educators in assessing whether children suspected of having developmental coordination disorder (DCD) meet the American Psychiatric Association (1994) diagnostic criterion of interference in activities of daily living when interference is defined as culturally subaverage engagement in activities of daily living in physical play (ADL-PP) on the playground. Participants were 136 children (75 girls, 61 boys) from Grades 1 to 4 at three elementary schools in Canada. Data were collected two ways: (a) three administrations of an ADL-PP self-report of activities done during recess and (b) observation of children's ADL-PP during two recess periods. Examination of reactivity, accuracy, content relevance, and content representativeness of the ADL-PP report form indicated protocol validity. An example illustrating the use of the ADL-PP self-report protocol to identify interference is described
SN - 0736-5829
AD - (Watkinson, Dunn, Cavaliere, Calzonetti, Wilhelm, Dwyer) Faculty of Physical Education and Recreation, University of Alberta (Calzonetti) Department of Kinesiology and Physical Education, Wilfrid Laurier University, Waterloo, Ont., Canada (Watkinson) Faculty of Physical Education and Recreation, University of Alberta, Edmonton, Alta. T6G 2H9, Canada
ER -

TY - JOUR
ID - 3913
T1 - Modelling of indoor exposure to nitrogen dioxide in the UK
A1 - Dimitroulopoulou,C.
A1 - Ashmore,M.R.
A1 - Byrne,M.A.
A1 - Kinnersley,R.P.
Y1 - 2001///
N1 - 2000363121
English
Journal: Article
KW - EMBASE
KW - Air Pollution
KW - article
KW - building
KW - computer model
KW - Computer Simulation
KW - Cooking
KW - gas transport
KW - Home Care
KW - indoor air pollution
KW - office worker
KW - population exposure
KW - priority journal
KW - school child
KW - United Kingdom
KW - Workplace
KW - nitrogen dioxide/to [Drug Toxicity]
RP - NOT IN FILE
SP - 269
EP - 279
JF - Atmospheric Environment
VL - 35
IS - 2
CY - United Kingdom
N2 - A dynamic multi-compartment computer model has been developed to describe the physical processes determining indoor pollutant concentrations as a function of outdoor concentrations, indoor emission rates and building characteristics. The model has been parameterised for typical UK homes and workplaces and linked to a time-activity model to calculate exposures for a representative homemaker, schoolchild and office worker, with respect to NO₂. The estimates of population exposures, for selected urban and rural sites, are

expressed in terms of annual means and frequency of hours in which air quality standards are exceeded. The annual mean exposures are estimated to fall within the range of 5-21ppb for homes with no source, and 21-27ppb for homes with gas cooking, varying across sites and population groups. The contribution of outdoor exposure to annual mean NO₂ exposure varied from 5 to 24%, that of indoor penetration of outdoor air from 17 to 86% and that of gas cooking from 0 to 78%. The frequency of exposure to 1h mean concentrations above 150ppb was very low, except for people cooking with gas. Copyright (C) 2000 Elsevier Science B.V
SN - 1352-2310
AD - (Dimitroulopoulou, Ashmore, Byrne, Kinnersley) T.H. Huxley School, Imperial College, London SW7 2BP, United Kingdom (Ashmore) Deptartment of Environmental Science, University of Bradford, West Yorkshire BD7 1DP, United Kingdom
ER -

TY - JOUR
ID - 3914
T1 - Sexually Transmitted Diseases in sexually abused children - Five case reports
A1 - Islam,S.
A1 - Hoque,S.
Y1 - 2000///
N1 - 2001006788
English
Journal: Article
KW - EMBASE
KW - Adolescent
KW - article
KW - Bangladesh
KW - Child
KW - Child Abuse
KW - clinical article
KW - clinical examination
KW - Disease Transmission
KW - Female
KW - genital ulcer
KW - gonococcal urethritis/di [Diagnosis]
KW - gynecologic disease
KW - Human
KW - Male
KW - reagin test
KW - Sexual Behavior
KW - sexually transmitted disease/di [Diagnosis]
KW - syphilis/di [Diagnosis]
KW - Treponema pallidum hemagglutination test
KW - urethra disease
KW - vertical transmission
RP - NOT IN FILE
SP - 20
EP - 22
JF - Bangladesh Journal of Dermatology, Venereology and Leprology
VL - 17
IS - 1
CY - Bangladesh
N2 - Sexually Transmitted Diseases (STDs) in children may be transmitted by sexual abuse, by accidental contact or perinatally. But sexual abuse is one of the most important concerns related to STDs in children. Transmission of STD pathogens occurs in 2% to 10% of abuse children and it is presumed that when penetration occurs, the risk is higher. Search in the literature of Bangladesh suggest that occurrence of STDs in children is rare. The reported cases of sexually abused children who presented to the Skin and VD outdoors of 2 Medical College hospitals in Bangladesh with Sexually Transmitted Diseases. One male of 13 years and four female

children of 5, 7, 9 and 13 years age respectively attended the Skin and VD outdoors of Sylhet MAG Osmani Medical and Bangladesh Medical College Hospitals during 1996 with the complaints of urethral / genital discharge, ulcers and multiple swellings in the genitalia along with their guardians. After proper clinical examinations, urethral / genital smear test were done where available for gonococcus, trichomonas and candida and sera were tested by RPR / TPHA for confirmation of syphilis. All the children gave the history of exposures with adult persons. Specific treatments were given and prompt improvement were noted after a week. Age range of children varies from 5 years to 13 years. Two female children and one male child were diagnosed as gonococcal urethritis and two female children were diagnosed as syphilis. It is revealed from histories that infected adults transmit these disease through sexual abuse. Children's STDs parallel those in adults. As more adults are infected in our country due to various factors, the likelihood of children contracting STD increases although few cases are included in our study. STDs in children are hard to imagine, since children are rarely sexually active, but they do occur due to sexual abuse. So, evaluation for sexual abuse should be done in all cases of child STDs. Prevention and treatment of adults are the most important steps to prevent these infections in children
SN - 1023-3903
AD - (Islam, Hoque) Dept. of Skin and VD, Sir Salimullah Medical College, Dhaka, Bangladesh
ER -

TY - JOUR
ID - 3915
T1 - Introduction of a new NHS electric-powered indoor/outdoor chair (EPIOC) service: Benefits, risks and implications for prescribers
A1 - Frank,A.O.
A1 - Ward,J.
A1 - Orwell,N.J.
A1 - McCullagh,C.
A1 - Belcher,M.
Y1 - 2000///
N1 - 2000427384
English
Journal: Article
KW - EMBASE
KW - accident
KW - Adolescent
KW - Adult
KW - Aged
KW - article
KW - caregiver
KW - Child
KW - Cohort Analysis
KW - controlled study
KW - Electricity
KW - falling
KW - Family
KW - Female
KW - hospital patient
KW - Human
KW - Interview
KW - major clinical study
KW - Male
KW - medical assessment
KW - national health service
KW - physical disability/rh [Rehabilitation]
KW - prescription
KW - Quality of Life
KW - risk benefit analysis
KW - Self Report

KW - Social Interaction
KW - wheelchair
RP - NOT IN FILE
SP - 665
EP - 673
JF - Clinical Rehabilitation
JA - Clin Rehabil
VL - 14
IS - 6
CY - United Kingdom
N2 - Objective: To determine problems associated with electric-powered indoor/outdoor wheelchairs (EPIOCs) and benefits perceived by their users. Design: Hospital-based cohort study of all patients referred over a 19-month period. Setting: Regional Wheelchair Service for North West London serving nine district wheelchairs services. Subjects: Of the 174 subjects referred, 10 withdrew their application or were found to be unsuitable at screening, 164 were assessed and 124 were prescribed chairs. One hundred and thirteen subjects were interviewed by telephone four months after provision of the chair. Main measures: Demographic and diagnostic data; current chair usage; self-reported accidents and component faults were compared with those recorded in the unit records; new activities embarked on since delivery; and benefits reported for main carers. Results: Seventeen 'mishaps' were reported by 15 users including tipping from chairs (6) and falls during transfers (3). Three probably reflected mechanical failure. Component failure was found in 39% of EPIOCs, mostly affecting control boxes (22), frames (10) and footrests (10). New activities were undertaken by 56 (50%) users, usually shopping and visits to friends/family. Eighty-five per cent of users felt felt that the chairs made life easier for their informal carers through increased independence, reducing transfer and reduced need for 'pushing'. Conclusions: The component failures and self-reported accidents (some potentially dangerous) have implications for the NHS, manufacturers, prescribers and users. EPIOCs enhance the independence of severely physically disabled individuals in the community and assist carers by lessening dependence and enhancing social interactions. This study demonstrated the need for follow-up of EPIOC users and chairs
SN - 0269-2155
AD - (Frank, Ward, Orwell, McCullagh, Belcher) Disablement Services Centre, Royal National Orthopaedic Hospital, Brockley Hill, Stanmore HA7 4LP, United Kingdom
ER -

TY - JOUR
ID - 3916
T1 - New research directions in neuroepidemiology
A1 - Cowan,L.D.
A1 - Leviton,A.
A1 - Dammann,O.
Y1 - 2000///
N1 - 2000286565
English
Journal: Review
KW - EMBASE
KW - Adult
KW - Aged
KW - Aging
KW - Alzheimer disease/ep [Epidemiology]
KW - Alzheimer disease/et [Etiology]
KW - Alzheimer disease/pc [Prevention]
KW - central nervous system
KW - Cerebral Palsy/ep [Epidemiology]
KW - cerebral palsy/et [Etiology]
KW - cerebral palsy/pc [Prevention]
KW - Child
KW - controlled study
KW - disease predisposition
KW - genetic polymorphism

KW - Human
KW - Infant
KW - Inflammation
KW - neurologic disease/ep [Epidemiology]
KW - neurologic disease/et [Etiology]
KW - neurologic disease/pc [Prevention]
KW - Newborn
KW - prematurity
KW - priority journal
KW - review
KW - risk factor
KW - stroke/ep [Epidemiology]
KW - Stroke/et [Etiology]
KW - stroke/pc [Prevention]
KW - corticosteroid
KW - cytokine
KW - thyroxine
RP - NOT IN FILE
SP - 18
EP - 23
JF - Epidemiologic Reviews
VL - 22
IS - 1
CY - United States
N2 - Many of the risk factors previously identified for disorders such as Alzheimer's disease, periventricular leukomalacia, multiple sclerosis, stroke, cerebral palsy, mental retardation, and acquired learning and attention disorders ultimately may be shown to damage the central and peripheral nervous systems through activation of inflammatory mediators. The challenge to epidemiologists in the future is to expand use of epidemiologic methods to explore how immune-mediated insults produce CNS disorders in human populations. Studies of the association of use of nonsteroidal anti-inflammatory drugs with risk of Alzheimer's disease and those of the association of immune parameters with risk of cerebral palsy are excellent examples of how epidemiology can contribute to our understanding of the causes of neurologic and/or neurodevelopmental disorders. Many of the immune parameters of interest have short half-lives and are difficult to measure outside of the laboratory setting. Questions also remain as to the proper timing of measurements in relation to the initial insult and, in some cases, which tissue is the most appropriate to sample. These measurement issues will need to be resolved before use of immune biomarkers in epidemiologic studies of the etiologies of neurologic disorders can be fully realized. Epidemiologists are most likely to help identify ways to prevent neurologic disorders if they are knowledgeable about the molecular biology of inflammation, modulators of CNS vulnerability, and genetic polymorphisms that influence both inflammation and CNS vulnerability and are prepared to become adept at biomarker epidemiology. This does not necessarily compel them to gain extensive knowledge of neurobiology. Rather, neuroepidemiology in the 21st century will require increased collaboration between epidemiologists, neurologists, and neurobiologists
SN - 0193-936X
AD - (Cowan) Department of Biostatistics and Epidemiology, College of Public Health, University of Oklahoma Health Sciences Center, Oklahoma City, OK, United States (Leviton, Dammann) Department of Neurology, Children's Hospital, Boston, MA, United States (Leviton, Dammann) Department of Neurology, Harvard Medical School, Boston, MA, United States (Cowan) College of Public Health, University of Oklahoma Health Sciences Center, PO Box 26901, Oklahoma City, OK 73190, United States
ER -

TY - JOUR
ID - 3917
T1 - A national survey of sun safety activities at U.S. zoos
A1 - Talosig,M.A.
A1 - Mayer,J.A.
A1 - Eckhardt,L.
A1 - Lewis,E.C.

A1 - Kwon,H.
A1 - Belch,G.E.
A1 - Eichenfield,L.F.
A1 - Elder,J.P.
A1 - Engelberg,M.
Y1 - 2000///
N1 - 2000283702
English
Journal: Article
KW - EMBASE
KW - Adolescent
KW - Adult
KW - Aged
KW - article
KW - cancer incidence
KW - cancer prevention
KW - Child
KW - Female
KW - health survey
KW - Human
KW - leisure
KW - major clinical study
KW - Male
KW - Protective Clothing
KW - questionnaire
KW - radiation exposure
KW - Safety
KW - skin cancer/dt [Drug Therapy]
KW - skin cancer/ep [Epidemiology]
KW - skin cancer/pc [Prevention]
KW - summer
KW - sunlight protection
KW - ultraviolet radiation
KW - United States
KW - sunscreen/dt [Drug Therapy]
RP - NOT IN FILE
SP - 331
EP - 342
JF - Journal of Community Health
JA - J Community Health
VL - 25
IS - 4
CY - United States
N2 - Skin cancer incidence in the United States has increased. Exposure to ultraviolet radiation (UVR) is a major risk factor for skin cancer. Every year, millions of children with their families visit outdoor leisure facilities such as zoos where overexposure to UVR may occur. This study was conducted to assess the proportion of U.S. zoos that provided sun safety activities during the summer of 1998 and their willingness to incorporate skin cancer prevention strategies at their facility in the future. A 56 item self-administered questionnaire was mailed to 140 zoos accredited by the American Zoo and Aquarium Association. A follow-up telephone survey was also conducted with 33 mail non-respondents. The response rate was 97% (N = 136). Results revealed that in the summer of 1998, only 5% of zoos provided sun safety recommendations to visitors and 25% of zoos provided recommendations to their employees. The recommendations made most often to visitors and employees were to use sunscreen and wear protective clothing. Eighty-five percent of zoos indicated interest in providing sun safety activities at their facilities in the future. The next step will be to design environmentally appropriate sun safety programs for zoos and to encourage zoos to implement these programs
SN - 0094-5145

AD - (Talosig, Mayer, Eckhardt, Lewis, Kwon, Elder) Division of Health Promotion at the Graduate, San Diego State University, School of Public Health, San Diego, CA, United States (Belch) Department of Marketing, San Diego State University, San Diego, CA, United States (Eichenfield) Departments of Pediatrics and Medicine, University of California, San Diego, CA, United States (Engelberg) ResearchWorks, San Diego, CA, United States
ER -

TY - JOUR
ID - 3918
T1 - Monitoring nitrogen dioxide and its effects on asthmatic patients: Two different strategies compared
A1 - Panella,M.
A1 - Tommasini,V.
A1 - Binotti,M.
A1 - Palin,L.
A1 - Bona,G.
Y1 - 2000///
N1 - 2000268744
English
Journal: Article
KW - EMBASE
KW - air monitoring
KW - article
KW - Asthma/di [Diagnosis]
KW - Asthma/ep [Epidemiology]
KW - Child
KW - clinical article
KW - controlled study
KW - environmental factor
KW - household
KW - Human
KW - human cell
KW - human tissue
KW - Italy
KW - patient education
KW - personal monitoring
KW - population risk
KW - Urban Population
KW - nitrogen dioxide
RP - NOT IN FILE
SP - 447
EP - 458
JF - Environmental Monitoring and Assessment
VL - 63
IS - 3
CY - Netherlands
N2 - Objectives: To develop a 'methodology assessment' to evaluate the strengths and the weaknesses of two different epidemiological approaches and to identify the best suited monitoring strategy to measure the effects of 'normal levels' of nitrogen dioxide exposure on the health of an urban population. Methodology: all exposures to nitrogen dioxide were determined with passive samplers, each sampler consisting of 3 measuring Palmes tubes. In the first study the nitrogen dioxide exposure was assessed in 23 school children (11 asthmatic and 12 non asthmatic). Children wore samplers for a week and parallel measurements were made in their kitchens, in bedrooms and outside their homes. The second study consisted in a case-control study where the relative risk of hospital admission was calculated considering nitrogen dioxide levels in a city of northern Italy. 110 asthmatic patients were compared to a control group of 5322 people. Results: Personal sampler measurements highlighted significant differences in exposure when nitrogen dioxide atmospheric levels were compared in asthmatic and healthy children ($p<0.05$). No other parameters were significant in the two groups. A significant action of atmospheric nitrogen dioxide on hospital admission was demonstrated ($p<0.01$). Conclusions: Although a cause-

effect relation as such cannot be identified, the studies show a relation between the exposure to nitrogen dioxide and the presence of adverse effects on people's health. However the 'disadvantage' is for subjects with asthmatic pathologies, compared to the others. To manage this problem most effectively, a combined approach with the activation of specific personal monitoring campaigns of the subjects with verified risk seems necessary. This requires 'reading' the data resulting from most extensive and up to date information systems, capable of a thorough control both of the living environment and of the clinical outcome of the whole population
SN - 0167-6369
AD - (Panella, Tommasini) Department of Public Health, Faculty of Medicine, University of Piemonte Orientale, Novara, Italy (Palin) Department of Environmental Health, Faculty of Medicine, University of Piemonte Orientale, Novara, Italy (Binotti, Bona) Department of Paediatrics, Faculty of Medicine, University of Piemonte Orientale, Novara, Italy
ER -

TY - JOUR
ID - 3919
T1 - Doping: Epidemiological data
A1 - Laure,P.
Y1 - 2000///
N1 - 2000262884
French
Journal: Review
KW - EMBASE
KW - Adolescent
KW - Adult
KW - Child
KW - Competition
KW - doping
KW - Drug Abuse
KW - drug dependence/ep [Epidemiology]
KW - drug traffic
KW - epidemiological data
KW - Female
KW - France
KW - Human
KW - Male
KW - Performance
KW - Physical Activity
KW - prescription
KW - review
KW - school child
KW - sport
KW - Sports Medicine
KW - anabolic agent/to [Drug Toxicity]
KW - central stimulant agent/to [Drug Toxicity]
KW - narcotic agent/to [Drug Toxicity]
RP - NOT IN FILE
SP - 1365
EP - 1372
JF - Presse Medicale
JA - Presse Med
VL - 29
IS - 24
CY - France
N2 - Prevalence: Whatever method is used (observation, interviews, questionnaire, laboratory tests), it is difficult to collect epidemiological data on doping. Particularly difficult problems are related to the definitions of sports players and the drugs involved as well as the often illicit nature of drug use. Results: The prevalence of doping in children and adolescents participating in sports is estimated at 3% to 5% with higher percentages in

boys, older adolescents and those playing at a competition level. Use of anabolic steroids, as early as 8 years of age, has increased since 1990, especially in girls. All studies have emphasized how easy it is for adolescents to procure any prohibited drug. In adults participating in amateur sports, the prevalence of doping would be 5% to 15%. All sports are involved with higher prevalence in men, age ranges 20-25 years and 35-39 years, and competitive level players. The main drugs used are stimulants, narcotics, corticosteroids and anabolic steroids. Combination of at least 2 drugs is frequent with an increase of mean daily dose over the last 15 years. According to users, the drugs are obtained with a medical prescription, on the underground market, or from other participants. Few data are available on doping outside sports activities. In the French department Meurthe-et- Moselle, 15% of the inhabitants use drugs to improve their occupational performance. Conclusions: Doping is more widespread than would be expected from antidoping control data. Other studies are needed to acquire more precise epidemiological data
SN - 0755-4982
ER -

TY - JOUR
ID - 3920
T1 - Measles giant-cell pneumonia in two children with acute myeloid leukemia
A1 - Sauter,S.
A1 - Niemeyer,C.
A1 - Bohm,N.
A1 - Ruschoff,J.
A1 - Brandis,M.
Y1 - 2000///
N1 - 2000261851
English
Journal: Article
KW - EMBASE
KW - acute granulocytic leukemia/di [Diagnosis]
KW - acute granulocytic leukemia/dt [Drug Therapy]
KW - acute leukemia/di [Diagnosis]
KW - acute leukemia/dt [Drug Therapy]
KW - article
KW - Case Report
KW - Child
KW - childhood leukemia/di [Diagnosis]
KW - childhood leukemia/dt [Drug Therapy]
KW - clinical feature
KW - electron microscopy
KW - Female
KW - fever/dt [Drug Therapy]
KW - giant cell
KW - Human
KW - human tissue
KW - measles/co [Complication]
KW - Measles/di [Diagnosis]
KW - measles/dt [Drug Therapy]
KW - Measles virus
KW - rash
KW - virus infection/co [Complication]
KW - virus infection/di [Diagnosis]
KW - virus infection/dt [Drug Therapy]
KW - virus infection/th [Therapy]
KW - virus pneumonia/co [Complication]
KW - virus pneumonia/di [Diagnosis]
KW - virus pneumonia/dt [Drug Therapy]
KW - virus pneumonia/th [Therapy]
KW - aciclovir/do [Drug Dose]

KW - aciclovir/dt [Drug Therapy]
KW - alpha interferon/dt [Drug Therapy]
KW - antibiotic agent/do [Drug Dose]
KW - antibiotic agent/dt [Drug Therapy]
KW - antifungal agent/dt [Drug Therapy]
KW - antineoplastic agent/cb [Drug Combination]
KW - antineoplastic agent/dt [Drug Therapy]
KW - antivirus agent/do [Drug Dose]
KW - antivirus agent/dt [Drug Therapy]
KW - cotrimoxazole/do [Drug Dose]
KW - cotrimoxazole/dt [Drug Therapy]
KW - cytarabine/cb [Drug Combination]
KW - cytarabine/dt [Drug Therapy]
KW - daunorubicin/cb [Drug Combination]
KW - daunorubicin/dt [Drug Therapy]
KW - etoposide/cb [Drug Combination]
KW - etoposide/dt [Drug Therapy]
KW - immunoglobulin/do [Drug Dose]
KW - immunoglobulin/dt [Drug Therapy]
KW - tioguanine/cb [Drug Combination]
KW - tioguanine/dt [Drug Therapy]
RP - NOT IN FILE
SP - 262
EP - 264
JF - Onkologie
VL - 23
IS - 3
CY - Germany
N2 - Background: Viral infections are a serious threat to the immunocompromised child. While effective treatment is available for varicella zoster, herpes simplex and cytomegalovirus, measles remains a major obstacle with a high mortality. Diagnosis is often hampered by atypical clinical features. Patients: We report on 2 cases of clinically unrecognized measles in children with acute myeloid leukemia (AML) and interstitial pneumonitis. Case 1 is a 3-year-old girl admitted with high fever, lymphadenopathy, hepatosplenomegaly, and leukocytosis. A diagnosis of AML was made and induction therapy initiated. Throughout treatment, the child remained febrile with temperatures above 39 degreeC. A rash was noted on both legs. Virus serology was nonspecific. Cough and tachypnea developed with interstitial infiltrations on X-ray. An open lung biopsy was diagnostic. The child died due to respiratory failure despite intensive therapy. Case 2 is a 7-year-old girl referred for treatment of AML in relapse. On admission, conjunctivitis was noted. Antibiotic therapy had been initiated for high-grade fever at an outside hospital. Reinduction chemotherapy was begun. On the 3rd hospital day a rapidly progressing pneumonitis, and on the 6th day a total-body erythema developed. The child died in spite of mechanical ventilation on day 13. On autopsy, giant-cell pneumonia was evident. Conclusion: With the currently low uptake of active immunization against measles in Germany, measles and its complications remain a continuing problem in immunocompromised children. Physicians should be aware of the atypical presenting features of measles in the oncology patient
SN - 0378-584X
AD - (Niemeyer) Universitats-Kinderklinik, Mathildenstrase 1, D-79104 Freiburg, Germany
ER -

TY - JOUR
ID - 3922
T1 - Therapeutic horse riding: What has this to do with occupational therapists?
A1 - Bracher,M.
Y1 - 2000///
N1 - 2000236216
English
Journal: Review
KW - EMBASE

KW - autism/th [Therapy]
KW - Case Report
KW - Child
KW - emotion
KW - Female
KW - horseback riding
KW - Human
KW - Male
KW - medical practice
KW - mental function
KW - occupational therapist
KW - Occupational Therapy
KW - Physical Activity
KW - Psychophysiology
KW - review
KW - spina bifida/th [Therapy]
RP - NOT IN FILE
SP - 277
EP - 282
JF - British Journal of Occupational Therapy
VL - 63
IS - 6
CY - United Kingdom
N2 - The horse has been used as a therapeutic medium since the time of the ancient Greeks and Xenophon is quoted as saying that 'the best thing for the inside of a man is the outside of the horse' (Longden 1998, p44). Man has been using horses for more than 3,500 years as draught horses or for riding; therefore, the man-horse relationship may be perceived 'as a close psychophysical attachment' (Schulz 1997, p65). The horse and its environment has been and continues to be used as a therapeutic tool for people with a wide range of needs and abilities, as is evidenced in the literature. This paper identifies some of the benefits and limitations of therapeutic horse riding and its relationship with occupational therapy, through a review of the literature and the use of case summaries from the author's personal experience
SN - 0308-0226
AD - (Bracher) Dept. Occup. Ther. and Physiother., Sch. Hlth. Professions Rehab. Sci., University of Southampton, Highfield, Southampton SO17 1BJ, United Kingdom
ER -

TY - JOUR
ID - 3923
T1 - Assessment of multipathway exposure of small children to PAH
A1 - Vyskocil,A.
A1 - Fiala,Z.
A1 - Chenier,V.
A1 - Krajak,L.
A1 - Ettlerova,E.
A1 - Bukac,J.
A1 - Viau,C.
A1 - Emminger,S.
Y1 - 2000///
N1 - 2000205630
English
Journal: Article
KW - EMBASE
KW - Air Pollution
KW - article
KW - Canada
KW - Child
KW - controlled study

KW - Environmental Exposure
KW - Female
KW - Food Contamination
KW - health hazard
KW - Human
KW - human experiment
KW - Inhalation
KW - kindergarten
KW - Male
KW - normal human
KW - priority journal
KW - Soil
KW - urinalysis
KW - 1 hydroxypyrene/to [Drug Toxicity]
KW - polycyclic aromatic hydrocarbon/to [Drug Toxicity]
KW - pyrene/to [Drug Toxicity]
RP - NOT IN FILE
SP - 111
EP - 118
JF - Environmental Toxicology and Pharmacology
VL - 8
IS - 2
CY - Netherlands
N2 - The aim of study was to assess the uptake of polycyclic aromatic hydrocarbons (PAH) by children living in a city and its effect on 1-hydroxypyrene (1-OHP) excretion. Two groups of children (n=11 and 13; 3-6 years old) were chosen: (1) a group from a kindergarten situated near a road with a high traffic density ('polluted' area); (2) a group from a kindergarten situated in a green zone ('non polluted' area). Food consumption was recorded in all children and PAH uptake from foodstuffs was estimated. Ambient air samples were collected on the playground and indoor of kindergartens during 3 days in summer 1997. Soil samples were collected on the playground. Urine samples were collected in the morning and in the evening. Mean outdoor total PAH concentration (sum of 12 individual PAH) in 'polluted' area was 12 times higher than that in 'non polluted' area (22.9 vs. 1.9 ng/m³). However, indoor concentrations were similar (3.0 vs. 2.1 ng/m³). The same trend was observed for pyrene concentrations. The contribution to the total pyrene absorbed dose from food consumption (estimated daily absorbed dose of 167 and 186 ng, respectively, in 'polluted' and 'non polluted' area) was much more important than that from inhalation (8.4 and 5.4 ng, respectively) in both areas. The estimated daily absorbed doses of pyrene from the soil were 0.061 and 0.104 ng in 'polluted' and 'non polluted' kindergarten, respectively, which correspond to 0.032 and 0.059% of the total absorbed dose. Higher urinary concentrations of 1-OHP were found in children from 'polluted' kindergarten. In conclusion, the food seems to be a main source of the total pyrene and total PAH uptake in small children, even under a relative high PAH air exposure in the city. Pyrene concentration in soil had a negligible contribution to the total pyrene absorbed dose. Usefulness of the urinary 1-OHP as an indicator of the environmental exposure to PAH needs further research. Copyright (C) 2000 Elsevier Science B.V
SN - 1382-6689
AD - (Vyskocil, Chenier, Viau) Dept. Med. Travail d'Hyg. du Milieu, Fac. Med., Univ. Montreal, C., Montreal, Que. H3C 3J7, Canada (Fiala, Ettlerova, Bukac) Dept. of Hyg. and Prev. Medicine, Charles Univ. Prague, Med. Fac. H., Hradec Kralove, Czech Republic (Krajak, Emminger) EMPLA, S.r.o., Hradec Kralove, Czech Republic
ER -

TY - JOUR
ID - 3924
T1 - Detection of gene defects in lipoprotein metabolism
A1 - Ritsch,A.
A1 - Patsch,J.R.
Y1 - 2000///
N1 - 2000182216
English
Journal: Article

KW - EMBASE
KW - article
KW - Case Report
KW - cholesterol blood level
KW - disorders of lipoprotein metabolism/cn [Congenital Disorder]
KW - disorders of lipoprotein metabolism/di [Diagnosis]
KW - disorders of lipoprotein metabolism/et [Etiology]
KW - enzyme assay
KW - enzyme deficiency/cn [Congenital Disorder]
KW - enzyme deficiency/di [Diagnosis]
KW - enzyme deficiency/et [Etiology]
KW - exon
KW - gene structure
KW - Heterozygote
KW - Human
KW - hyperlipoproteinemia type 1/cn [Congenital Disorder]
KW - hyperlipoproteinemia type 1/di [Diagnosis]
KW - hyperlipoproteinemia type 1/et [Etiology]
KW - Infant
KW - intron
KW - lipid blood level
KW - lipoprotein blood level
KW - lipoprotein metabolism
KW - nonsense mutation
KW - Polymerase Chain Reaction
KW - preschool child
KW - promoter region
KW - transcription regulation
KW - triacylglycerol blood level
KW - acid lipase/ec [Endogenous Compound]
KW - cholesterol ester transfer protein/ec [Endogenous Compound]
KW - complementary DNA
KW - high density lipoprotein cholesterol/ec [Endogenous Compound]
KW - lipoprotein/ec [Endogenous Compound]
KW - lipoprotein lipase/ec [Endogenous Compound]
KW - lysosome enzyme/ec [Endogenous Compound]
KW - triacylglycerol/ec [Endogenous Compound]
RP - NOT IN FILE
SP - 254
EP - 261
JF - LaboratoriumsMedizin
VL - 24
IS - 5
CY - Germany
N2 - Gene defects in lipoprotein metabolism usually cause conspicuous alterations in plasma lipoprotein profile and, often, more or less severe clinical problems. Increased or decreased lipid parameters therefore represent starting points for detection of these gene defects, but also provide important information to select target proteins whose activity is limited or destroyed. An increase of a single lipid parameter facilitates the search for the target protein, which can be identified afterwards by an activity assay. Employing this strategy we were able to identify lipoprotein lipase (LPL) deficiency in a 6 week old patient with grossly increased plasma triglyceride concentrations. In another instance, an increased level of HDL cholesterol in patient M.Y. led to the identification of cholesteryl ester transfer protein (CETP) deficiency. In patient M.H. displaying mixed hyperlipidemia a variety of target proteins were possible. Careful clinical examinations and performance of activity measurements of several enzymes were necessary to detect lysosomal acid Lipase (LAL) deficiency. Delineation of the molecular defect is facilitated by the knowledge of described mutations which can often be rapidly identified using PCR and subsequent restriction analysis. In this way we were able to identify a nonsense mutation in the LPL gene of patient M.K. as well as a splice site defect in the LAL gene of patient M.H. In the

case of absence of known mutations, we recommend cDNA analysis using PCR and subsequent sequence analysis. Using this technology patient M.Y. could be identified as heterozygous carrier of a nonsense mutation within the CETP gene. Defective transcription may be caused by destroyed gene structure, splice site mutations, or mutations altering expression of the corresponding gene. Amplification and sequence analysis of genomic DNA fragments allow identification of mutations within intron-exon boundaries or gene promoter regions. The gene structure can be examined via amplification employing long range PCR with subsequent restriction analysis. The identification of mutations outside the coding region of a structural gene may be complicated, since they may be able to prevent transcription although being located more than 100 kB from the corresponding gene
SN - 0342-3026
AD - (Ritsch, Patsch) Universitatsklinik Innsbruck, Innere Medizin, Anichstrasse 35, A-6020 Innsbruck, Austria
ER -

TY - JOUR
ID - 3925
T1 - Electrocorticographic factors associated with temporal lobe epileptogenicity
A1 - Weinand,M.E.
A1 - Deogaonkar,M.
A1 - Kester,M.
A1 - Ahern,G.L.
A1 - Labiner,D.M.
Y1 - 2000///
N1 - 2000180077
English
Journal: Article
KW - EMBASE
KW - Adolescent
KW - Adult
KW - article
KW - Child
KW - drug withdrawal
KW - electrocorticography
KW - epileptogenesis
KW - Female
KW - Human
KW - major clinical study
KW - Male
KW - Monitoring
KW - outcomes research
KW - priority journal
KW - seizure/et [Etiology]
KW - temporal lobe epilepsy/et [Etiology]
KW - temporal lobe epilepsy/su [Surgery]
KW - temporal lobectomy
RP - NOT IN FILE
SP - 33
EP - 39
JF - Pathophysiology
VL - 7
IS - 1
CY - Netherlands
N2 - Continuous subdural electrocorticographic (ECoG) monitoring was performed to test the hypothesis that human temporal lobe epileptogenicity, during long-term monitoring following antiepileptic drug (AED) withdrawal, regardless of the specific AED regimen, is dependent upon ECoG ictal onset and interhemispheric spread of epileptic activity. In 121 patients, ECoG parameters were analyzed for association with seizure frequency, a clinical measure of epileptogenicity. Significantly associated with increased seizure frequency

were: ictal medial temporal lobe onset, absence of ictal frontal lobe desynchronization and short interhemispheric propagation time (IHPT). Seizure frequency during long-term ECoG monitoring was not predictive of post-operative seizure outcome. It is concluded that, following AED withdrawal, regardless of the specific AED regimen, increased seizure frequency is associated with medial temporal lobe ictal onset, short IHPT and absence of frontal lobe desynchronization. The results confirm the hypothesis that human temporal lobe epileptogenicity, after withdrawal, is dependent upon ECoG ictal onset and interhemispheric spread of epileptic activity. Future development of procedures which promote ECoG factors associated with increased seizure frequency following AED withdrawal might decrease duration of invasive long-term monitoring and improve efficiency for the pre-surgical selection of temporal lobectomy candidates. Intervention producing ictal frontal lobe desynchronization and increased IHPT might inhibit temporal lobe epileptogenicity and should be evaluated for therapeutic efficacy outside of the long-term monitoring context. Copyright (C) 2000 Elsevier Science Ireland Ltd
SN - 0928-4680
AD - (Weinand, Deogaonkar) Division of Neurosurgery, Dept. Surg., P.O. B., Tucson, AZ 85724-5070, United States (Kester) Arizona Prevention Center, Univ. of Arizona College of Medicine, Tucson, AZ 85721, United States (Ahern, Labiner) Department of Neurology, Univ. of Arizona College of Medicine, Tucson, AZ 85724, United States
ER -

TY - JOUR
ID - 3926
T1 - Three consecutive (1993, 1995, 1997) surveys of food intake, nutritional attitudes and knowledge, and lifestyle in 1000 French children, aged 9-11 years
A1 - Bellisle,F.
A1 - Rolland-Cachera,M.-F.
Y1 - 2000///
N1 - 2000133951
English
Journal: Article
KW - EMBASE
KW - Anthropometry
KW - article
KW - Attitude
KW - cereal
KW - child health
KW - child nutrition
KW - dairy product
KW - Demography
KW - disease association
KW - family life
KW - Feeding Behavior
KW - Female
KW - Food Intake
KW - France
KW - Fruit
KW - fruit juice
KW - health survey
KW - high risk population
KW - Human
KW - leisure
KW - Lifestyle
KW - Male
KW - meal
KW - normal human
KW - Obesity/ep [Epidemiology]
KW - Physical Activity
KW - questionnaire

KW - school
KW - school child
KW - sitting
KW - socioeconomics
KW - sport
KW - Television
KW - fat
KW - sugar
RP - NOT IN FILE
SP - 101
EP - 111
JF - Journal of Human Nutrition and Dietetics
VL - 13
IS - 2
CY - United Kingdom
N2 - Background and Aims. The lifestyle of children in developed societies is susceptible to rapid changes and these may affect the nutritional status of children. Reduced physical activity and changes in diet have been proposed as contributing factors to the growth in childhood overweight and obesity. The aim of this study was to assess trends in the food-related behaviour and markers of activity/inactivity in French 9-11-year-old children. Methods. Three successive surveys (1993, 1995, 1997) were carried out in samples of 1000 French children, aged 9-11 years. Sociodemographic, anthropometric and food-related parameters were obtained for each child, using standardized questionnaire administered by trained interviewers. Results. Previous-day reports of food intake by the child revealed a strong persistence of the traditional French meal structure. Breakfast was eaten by 97% of children. Over the three surveys, an increasing percentage of reported breakfasts contained at least one dairy food, one cereal food, and one fruit or juice (from 11% to 17%). Almost all children had lunch, which occurred at the school cafeteria for one-third of the subjects. The afternoon snack, a traditional meal for French children, was consumed by 86-88% of the samples. Almost all children had dinner (99%), most often at home and in the company of all family members (73-87%). Lunches and dinners were composed of several courses presented in succession, as is usual in France. The foods most preferred by the children were often rich in sugar and/or fat (fried potatoes, ice cream, nut spread, chocolate, cake, etc.). The children could list 'healthy foods' competently. They also demonstrated knowledge of terms used in nutrition (e.g. calories, fats) and were aware of possible links between intake of certain substances and disease. In families of higher socio-economic strata (income, education of parents) more time was devoted to sports by the children. Over the three surveys, linear trends indicated more exercise time per week and less television viewing. A high risk of obesity was likely in very sedentary children whose proportion (33%) was stable over the 1993-1997 period. Conclusions. In general the traditional French pattern of eating persisted among these children. Although there were signs of movement towards a more complete breakfast, less television and more exercise in active children, a substantial proportion of children engaged in no sports activity outside school hours. Maintaining appropriate weight is therefore difficult in view of the plentiful supply of food available to children. Inactive children are at high risk of obesity and should be encouraged to adopt a more active lifestyle
SN - 0952-3871
AD - (Bellisle) INSERM U341 and Service de Nutrition, Hotel Dieu, 1 Place du Parvis Notre-Dame, 75181 Paris Cedex 04, France (Rolland-Cachera) XR 290 INSERM, ISTNA, CNAM, 2 rue Conte, 75003 Paris, France
ER -

TY - JOUR
ID - 3927
T1 - Postoperative nausea and vomiting after Faden operation
A1 - Welters,I.D.
A1 - Graef,M.
A1 - Menges,T.
A1 - Beikirch,C.
A1 - Kaufmann,H.
A1 - Hempelmann,G.
Y1 - 2000///

N1 - 2000107694
English
Journal: Article
KW - EMBASE
KW - antiemetic activity
KW - article
KW - Body Height
KW - Body Weight
KW - Child
KW - clinical trial
KW - controlled clinical trial
KW - controlled study
KW - double blind procedure
KW - Human
KW - major clinical study
KW - oculocardiac reflex
KW - pediatric anesthesia
KW - postoperative nausea/co [Complication]
KW - postoperative nausea/dt [Drug Therapy]
KW - postoperative nausea/pc [Prevention]
KW - premedication
KW - priority journal
KW - randomized controlled trial
KW - statistical analysis
KW - strabismus surgery
KW - suppository
KW - surgical technique
KW - vomiting/co [Complication]
KW - vomiting/dt [Drug Therapy]
KW - vomiting/pc [Prevention]
KW - dimenhydrinate/ct [Clinical Trial]
KW - dimenhydrinate/do [Drug Dose]
KW - dimenhydrinate/dt [Drug Therapy]
KW - dimenhydrinate/pr [Pharmaceutics]
KW - droperidol
KW - halothane
KW - histamine H1 receptor antagonist
KW - metoclopramide
KW - midazolam/po [Oral Drug Administration]
KW - nitrous oxide
KW - opiate
KW - Oxygen
KW - placebo
KW - thiopental/iv [Intravenous Drug Administration]
KW - vecuronium/iv [Intravenous Drug Administration]
RP - NOT IN FILE
SP - 59
EP - 63
JF - Graefe's Archive for Clinical and Experimental Ophthalmology
VL - 238
IS - 1
CY - Germany
N2 - Objective: The purpose of this study was to evaluate the antiemetic effect of prophylactic dimenhydrinate application prior to Faden operation and to compare the incidence of PONV between bimedial Faden operation (BMF) and horizontal recess-resect procedure (R and R). Methods: Ninety-nine children (4-10 years) scheduled for BMF were included in this prospective double-blind study. Midazolam (0.5 mg/kg body weight, BW) was administered orally for premedication 30 min before induction of anesthesia. Additionally, children weighing >

23 kg received either dimenhydrinate suppositories or placebo. The placebo group was compared with 148 children who underwent R and R surgery without antiemetic prophylaxis during the same period. Anesthesia was induced with thiopentone (5-10 mg/kg BW) and vecuronium (0.1 mg/kg BW) bromide and maintained with halothane (1-2 vol%) in N₂O/O₂ (65/35 vol%). Age, height, weight, and incidence of oculocardiac reflex were documented. PONV was classified into 'no vomiting', 'vomiting without therapy', and 'vomiting requiring rescue medication'. In the latter case dimenhydrinate was given again. The chi-square test was used for statistical analysis. Results: Forty-eight patients received placebo, while 51 received dimenhydrinate. No differences between any groups were observed concerning age, height, weight, and incidence of oculocardiac reflex. Compared to R and R surgery, PONV requiring rescue medication occurred significantly more frequently after BMF (45% vs 23% after R and R). PONV after BMF was significantly less severe in the dimenhydrinate group than in the placebo group. The total incidence of PONV after BMF, however, was not significantly reduced. Conclusion: The high incidence of PONV after BMF can be explained by the greater invasiveness of BMF than R and R surgery. PONV requiring antiemetic rescue medication can be reduced by preoperative administration of dimenhydrinate suppositories
SN - 0721-832X
AD - (Welters, Menges, Beikirch, Hempelmann) Department of Anaesthesiology, Operative Intensive Care, Rudolf-Buchheim-Strasse 7, D-35385 Giessen, Germany (Graef, Kaufmann) Dept. Strabismology N., University of Giessen, Giessen, Germany
ER -

TY - JOUR
ID - 3928
T1 - Late diagnosis of retinoblastoma in a developing country
A1 - Chantada,G.
A1 - Fandino,A.
A1 - Manzitti,J.
A1 - Urrutia,L.
A1 - Schvartzman,E.
Y1 - 1999///
N1 - 1999093028
English
Journal: Article
KW - EMBASE
KW - article
KW - cancer diagnosis
KW - childhood cancer/di [Diagnosis]
KW - clinical feature
KW - developing country
KW - diagnostic approach route
KW - disease severity
KW - early diagnosis
KW - Human
KW - major clinical study
KW - preschool child
KW - priority journal
KW - retinoblastoma/di [Diagnosis]
KW - school child
RP - NOT IN FILE
SP - 171
EP - 174
JF - Archives of Disease in Childhood
JA - Arch Dis Child
VL - 80
IS - 2
CY - United Kingdom
N2 - Objectives - To assess the diagnostic process of retinoblastoma in a developing country. Study design - Prospective survey of 95 consecutive parents of patients with retinoblastoma. Results - Fifty six parents

consulted initially with a paediatrician. Their children tended to be younger, with a significantly higher frequency of advanced disease. Only half of the patients who consulted with a paediatrician were appropriately referred to an ophthalmologist; the paediatrician underestimated the complaints in the remainder. Children taken to an ophthalmologist were older and had less advanced disease. In about three quarters of these children, a diagnosis of retinoblastoma was suspected by the ophthalmologist on the first visit. Parents of patients with more advanced disease consulted significantly later. Poor parental education correlated significantly with late consultation. Lack of health insurance and living outside Buenos Aires City correlated significantly with an increased risk of extraocular disease. Conclusions - Paediatricians are the first health professional seen by most children with retinoblastoma. However, the diagnosis is not readily established. There is also a delay in consultation by parents, which is significantly longer in cases with advanced extraocular disease. Socioeconomic factors and access to health care might play a role in delayed diagnosis
SN - 0003-9888
AD - (Chantada, Schvartzman) Servicio de Hemato-oncologia, Hospital JP Garrahan, Combate de los Pozos 1881 (1245), Buenos Aires, Argentina
ER -

TY - JOUR
ID - 3929
T1 - Playground safety--United States, 1998-1999
Y1 - 1999///
N1 - 10366141
English
Journal: Article
KW - EMBASE
KW - article
KW - Child
KW - Human
KW - Infant
KW - preschool child
KW - Recreation
KW - Safety
KW - United States
RP - NOT IN FILE
SP - 329
EP - 332
JF - MMWR
VL - Morbidity and mortality weekly report. 48
IS - 16
CY - United States
N2 - Each year approximately 211,000 U.S. children receive emergency department care for injuries sustained on playground equipment, making the use of this equipment the leading cause of injuries to children in school and child care environments. In response to the problem, the National Program for Playground Safety (NPPS) at the University of Northern Iowa (UNI) developed a national action plan that focuses on four areas of playground injury prevention: supervision, age-appropriateness of equipment, suitable fall surfaces, and equipment maintenance. During 1998-1999, NPPS surveyed a sample of the nation's child care, elementary school, and park playgrounds. This report summarizes the survey results, which indicate that playground injuries could be reduced by measures such as resilient surfacing below equipment, better equipment maintenance, improved supervision, and use of age-appropriate equipment
SN - 0149-2195
ER -

TY - JOUR
ID - 3930
T1 - Incidence rates of Lyme disease in Maryland: 1993 through 1996
A1 - Pena,C.A.
A1 - Strickland,G.T.
Y1 - 1999///

N1 - 10333675
English
Journal: Article
KW - EMBASE
KW - Adolescent
KW - Adult
KW - Age Distribution
KW - Aged
KW - article
KW - Child
KW - Female
KW - Human
KW - Incidence
KW - Infant
KW - Lyme Disease/ep [Epidemiology]
KW - Male
KW - Middle Aged
KW - Newborn
KW - preschool child
KW - Sex Ratio
KW - United States/ep [Epidemiology]
RP - NOT IN FILE
SP - 68
EP - 73
JF - Maryland medical journal (Baltimore, Md
VL - : 1985). 48
IS - 2
CY - United States
N2 - The incidence rate per 100,000 population by gender and age group was calculated for the 24 Maryland jurisdictions using 1,392 cases reported to the Lyme Disease Registry during 1993 to 1996. The overall incidence rate for the state was 7.0 cases per 100,000 inhabitants. Males (incidence rate = 7.7) were 1.2 times more affected than females (incidence rate = 6.2) ($p < 0.001$). The bimodal overall age distribution shows a peak in the 10 to 19 age category (incidence rate = 8.5) and in the 50 to 59 age group (incidence rate = 8.9), and in the 20 to 29 age group (incidence rate = 3.9). The incidence rate by jurisdiction ranged from 0.7 in Allegany County from Western Maryland to 88.3 in Kent County on the Upper Eastern Shore. Results from this study show that counties from the Upper Eastern Shore region had the highest specific incidence rate by age group/gender/jurisdiction while counties from Western Maryland had the lowest specific incidence rates and have not reported Lyme disease patients younger than 10 or older than 59 years of age. This difference might be explained primarily by the kind of outdoor activities in which the different age groups are involved and the relative abundance of Ixodes scapularis infected with the etiologic agent of Lyme disease, Borrelia burgdorferi, in these two regions
SN - 0886-0572
AD - (Pena, Strickland) University of Maryland School of Medicine, USA
ER -

TY - JOUR
ID - 3931
T1 - The role of cisapride in the treatment of pediatric gastroesophageal reflux
A1 - Vandenplas,Y.
A1 - Belli,D.C.
A1 - Benatar,A.
A1 - Cadranel,S.
A1 - Cucchiara,S.
A1 - Dupont,C.
A1 - Gottrand,F.
A1 - Hassall,E.
A1 - Heymans,H.S.A.

A1 - Kearns,G.
A1 - Kneepkens,C.M.F.
A1 - Koletzko,S.
A1 - Milla,P.
A1 - Polanco,I.
A1 - Staiano,A.-M.
Y1 - 1999///
N1 - 2000195531
English
Journal: Article
KW - EMBASE
KW - article
KW - Child
KW - drug contraindication
KW - drug efficacy
KW - drug metabolism
KW - drug safety
KW - drug withdrawal
KW - electrocardiogram
KW - enuresis/si [Side Effect]
KW - gastroesophageal reflux/dt [Drug Therapy]
KW - gastrointestinal toxicity/si [Side Effect]
KW - headache/si [Side Effect]
KW - Human
KW - Infant
KW - long QT syndrome/si [Side Effect]
KW - neurotoxicity/si [Side Effect]
KW - practice guideline
KW - preschool child
KW - priority journal
KW - school child
KW - skin toxicity/si [Side Effect]
KW - vertigo/si [Side Effect]
KW - cisapride/ae [Adverse Drug Reaction]
KW - cisapride/dt [Drug Therapy]
RP - NOT IN FILE
SP - 518
EP - 528
JF - Journal of Pediatric Gastroenterology and Nutrition
VL - 28
IS - 5
CY - United States
N2 - Background: Cisapride is a gastrointestinal prokinetic agent that is used worldwide in the treatment of gastrointestinal motility-related disorders in premature infants, full-term infants, and children. Efficacy data suggest that it is the most effective commercially available prokinetic drug. Methods: Because of recent concerns about safety, a critical and in- depth analysis of all reported adverse events was performed and resulted in the conclusions and recommendations that follow. Results: Cisapride should only be administered to patients in whom the use of prokinetics is justified according to current medical knowledge. If cisapride is given to pediatric patients who can be considered healthy except for their gastrointestinal motility disorder, and the maximum dose does not exceed 0.8 mg/kg per day in 3 to 4 administrations of 0.2 mg/kg (not exceeding 40 mg/d), no special safety procedures regarding potential cardiac adverse events are recommended. However, if cisapride is prescribed for patients who are known to be or are suspected of being at increased risk for drug-associated increases in QTc interval, certain precautions are advisable. Such patients include those:(1) with a previous history of cardiac dysrhythmias, (2) receiving drugs known to inhibit the metabolism of cisapride and/or adversely affect ventricular repolarisation, (3) with immaturity and/or disease causing reduced cytochrome P450 3A4 activity, or (4) with electrolyte disturbances. In such patients, ECG monitoring to quantitate the QTc interval should be used before initiation of therapy and after 3 days of treatment to ascertain

whether a cisapride-induced cardiac adverse effect is present. Conclusions: With rare exceptions, the total daily dose of cisapride should not exceed 0.8 mg/kg divided into 3 or 4 approximately equally spaced doses. If higher doses than this are given, the precautions above are advisable. In any patient in whom a prolonged QTc interval is found, the dose of cisapride should be reduced or the drug discontinued until the ECG normalizes. If the QTc interval returns to normal after withdrawal of cisapride, and the administration of cisapride is considered to be justified because of its efficacy and absence of alternative treatment options, cisapride can be restarted at half dose with control of the QTc interval. Unfortunately, at present, normal ranges of QTc interval in children are unknown. However, a critical analysis of the literature suggests that a duration of less than 450 milliseconds can be considered to be within the normal range and greater than 470 milliseconds as outside it. (C) 1999 Lippincott Williams and Wilkins, Inc
SN - 0277-2116
AD - (Vandenplas, Benatar) AZ-VUB, Laarbeeklaan 101, 1090 Brussels, Belgium (Belli) Hop. Universiatires de Geneve, Geneva, Switzerland (Cadranel) HUDE Reine Fabiola, Brussels, Belgium (Cucchiara, Staiano) Clinica Pediatrica, Naples, Italy (Dupont) Hopital Saint-Vincent-de-Paul, Paris, France (Gottrand) Hopital Jeanne de Flandre, Lille, France (Hassall) BC Children's Hospital, Vancouver, BC, Canada (Heymans) AMC, Amsterdam, Netherlands (Kearns) Pediat. Clin. Pharmacol. Exp. T., Children's Mercy Hospital, Kansas City, MO, United States (Kneepkens) AZ-VU, Amsterdam, Netherlands (Koletzko) Dr. V. Haunersches Kinerspital, Munich, Germany (Milla) Institute of Child Health, London, United Kingdom (Polanco) Hopital La Paz, Madrid, Spain
ER -

TY - JOUR
ID - 3932
T1 - Is physical activity related to body size, fundamental motor skills, and CHD risk factors in early childhood?
A1 - Saakslahti,A.
A1 - Numminen,P.
A1 - Niinikoski,H.
A1 - Rask-Nissila,L.
A1 - Viikari,J.
A1 - Tuominen,J.
A1 - Valimaki,I.
Y1 - 1999///
N1 - 1999394105
English
Journal: Article
KW - EMBASE
KW - Anthropometry
KW - article
KW - body mass
KW - Body Size
KW - Child Behavior
KW - Female
KW - Human
KW - human cell
KW - human experiment
KW - human tissue
KW - ischemic heart disease/di [Diagnosis]
KW - Male
KW - Motor Performance
KW - normal human
KW - Physical Activity
KW - preschool child
KW - risk factor
RP - NOT IN FILE
SP - 327
EP - 340

JF - Pediatric Exercise Science
JA - Pediatr Exerc Sci
VL - 11
IS - 4
CY - United States
N2 - The purpose of this study was to examine patterns of physical activity (PA) during a single weekend to ascertain possible relationships between PA and anthropometry, fundamental motor skills, and CHD risk factors among 105 normal male and female children, aged 3-4 years. The children played, when awake, on the average for 14 hr, 16 min indoors and for 5 hr, 12 min outdoors of which low activity playing accounted about 4 hr. Notable gender differences were observed in the intensity of PA but not in fundamental motor skills and CHD risk factors. The results suggest that physical activity is weakly related to fundamental motor skills and CHD risk factors at an early age. The association between PA and body size was modified by gender (p = .024): The girls who played indoors a lot were heavier than the others, and the boys who played much more outdoors were heavier in relation to other boys. The associations between PA and motor skills as well as PA and CHD risk factors were also highly gender-dependent: The boys benefited from interacting with parents, while the girls benefited from independence. The most influential factors seemed to be the amount of playing outdoors, the amount of high level play activities, as well as interaction with parents
SN - 0899-8493
AD - (Saakslahti, Numminen, Niinikoski, Rask-Nissila, Viikari, Tuominen, Valimaki) Department of Physical Education, University of Jyvaskyla, FIN-40351 Jyvaskyla, Finland
ER -

TY - JOUR
ID - 3933
T1 - Health promotion in Welsh primary schools, 1998
A1 - Mckeown,S.
A1 - Roberts,C.
A1 - Tudor-Smith,C.
A1 - Bowker,S.
Y1 - 1999///
N1 - 1999370309
English
Journal: Article
KW - EMBASE
KW - article
KW - cigarette smoking
KW - Curriculum
KW - Health Care Policy
KW - Health Education
KW - Health Promotion
KW - Human
KW - normal human
KW - nutritional health
KW - Nutritional Status
KW - population research
KW - preschool child
KW - primary school
KW - school child
KW - school health service
KW - sexual education
KW - Teaching
KW - United Kingdom
RP - NOT IN FILE
SP - 81
EP - 88
JF - International Journal of Health Promotion and Education
VL - 37

IS - 3
CY - United Kingdom
N2 - This paper presents findings from a self-completion questionnaire survey of primary schools in Wales conducted in the spring of 1998. The survey provides data on the organisation and provision of health education teaching, the implementation of health related policies, the involvement of outside agencies and professionals in the planning and delivery of health promotion, and the extent to which respondents perceive their schools to fulfil the role of a 'health promoting school'. The results from the responding 450 schools (80% response rate) show a continued commitment and expansion of health promotion activity since an earlier survey in 1993. Notable improvements include an increase in the number of schools reporting policies relating to bullying, sex education, and adult smoking on school premises. Fifteen per cent of respondents considered their school to be health promoting and it was encouraging to find that some health promoting practices were reported more often by these schools than others. However, the results also suggest that the concept of a 'health promoting' school may not yet be completely understood. It is recommended that greater attention in all schools should be given to: written health policies for both pupils and staff, in particular those regarding nutrition; the amount of in-service training devoted to health education topics; and further integration of community resources into school health promotion programmes
SN - 1463-5240
AD - (Mckeown, Roberts, Tudor-Smith, Bowker) Health Promotion Division, National Assembly for Wales, Ffynnon-Las, Ty-Glas Avenue, Llanishen, Cardiff CF14 5EZ, United Kingdom
ER -

TY - JOUR
ID - 3935
T1 - Epidemiology of blunt abdomined trauma in children: A retrospective analysis of preclinical and clinical management in 1053 traumatised children
A1 - El Nasr,M.S.
A1 - Schlickewei,W.
A1 - Haag,C.
A1 - Gotze,B.
A1 - Lascho,C.
A1 - Friedl,H.P.
Y1 - 1999///
N1 - 1999296386
German
Journal: Article
KW - EMBASE
KW - abdominal blunt trauma/ep [Epidemiology]
KW - article
KW - Child
KW - childhood injury/ep [Epidemiology]
KW - conservative treatment
KW - convalescence
KW - hospital admission
KW - Human
KW - injury scale
KW - kidney injury/ep [Epidemiology]
KW - Laparotomy
KW - liver injury/ep [Epidemiology]
KW - major clinical study
KW - Multiple Organ Failure
KW - play
KW - retrospective study
KW - spleen injury/ep [Epidemiology]
KW - sport injury
KW - traffic accident
RP - NOT IN FILE
SP - 156

EP - 160
JF - Aktuelle Traumatologie
JA - Aktuelle Traumatol
VL - 29
IS - 4
CY - Germany
N2 - In children the major cause for surgical admissions to hospital are accidents and their long term sequels. In order to get epidemiological insides on the etiology, injury patterns, required surgical interventions, duration of hospital stay, convalescence, and return times to school, we analysed 1053 cases presenting at the Department of Trauma, University Hospital Freiburg, during a period of four years. Twohunderednintysix (28.1%) admittances were due to traffic accidents, 276 (26.2%) to playground accidents, 225 (21.4%) to domestic accidents and 179 (17.0%) were associated to sport activities. Over all every 4(th) child (in case of traffic accidents every 2(nd)) suffered two or more injuries. The severity of injury was classified according to the Hannover Polytrauma Severity Score. Fractures comprising 42.8% of the treated injuries, were the main reason for surgical interventions. Sixtyseven (6.4%) of the 1053 cases represented abdominal trauma, being the 4(th) cause for treatment. Of these, 46 had blunt abdominal trauma without defined organic leasions. Fourteen had isolated organic leasions, and in 7 multiple organ injuries. The spleen was the most exposed and dangered organ and was involved in 9 cases, followed by kidney and liver trauma. In 53 cases the injuries could be treated conservatively, 14 children had to undergo laparotomy, 82.1% of the treated children recovered completely. The average time until full recovering was 85.5 days and correlated with the severity of the abdominal trauma
SN - 0044-6173
AD - (El Nasr, Schlickewei, Haag, Gotze, Lascho, Friedl) Abteilung Unfallchirurgie, Chir. Univ. Klin. Freiburg (El Nasr) Chir. Univ. Klin. Freiburg, Hugstetter Strase 55, 79106 Freiburg i. Br, Germany
ER -

TY - JOUR
ID - 3936
T1 - The evolving relationship between weight-for-height and weight-for-age in children between 3 months and 6 years old receiving preschool nutritional assistance, Sorocaba, Sao Paulo, Brazil
A1 - Correa,A.M.S.
A1 - Goncalves,N.N.D.S.
A1 - Goncalves,A.
A1 - Leite,G.P.R.
A1 - Padovani,C.R.
Y1 - 1999///
N1 - 1999272213
Portuguese
Journal: Article
KW - EMBASE
KW - age
KW - Anthropometry
KW - article
KW - Body Height
KW - Body Weight
KW - Brazil
KW - child growth
KW - Employment
KW - Female
KW - Human
KW - Infant
KW - Interview
KW - lowest income group
KW - major clinical study
KW - Male
KW - mother
KW - nutritional health
KW - Nutritional Status

KW - preschool child
RP - NOT IN FILE
SP - 26
EP - 33
JF - Revista Panamericana de Salud Publica/Pan American Journal of Public Health
VL - 6
IS - 1
CY - United States
N2 - The nutritional status of Brazilians has improved over the last three decades. Still, little is known about the role played by nutritional assistance programs that public institutions and philanthropic organizations provide for low-income preschool children, who face greater nutritional risk. Therefore, we carried out a quasi-experimental study to evaluate the nutritional impact of the municipal preschool nutritional assistance program in the city of Sorocaba, Sao Paulo, Brazil. Over the course of a year, we performed quarterly measurements of weight and height on 444 children, whose ages ranged from 3 months to 6 years. In this population, 164 children were assisted by the preschool nutritional program (intervention group), and 280 children were not (nonintervention group). The children in the nonintervention group were identified by a special census for that purpose; they did not attend the preschool but lived nearby. After obtaining informed consent and carrying out a pretest, professionals and university students interviewed the mothers of the participating children, following standard techniques and conditions. Anthropometric measurements were done by teams of two interviewers, one of whom was a permanent member of the team, using equipment calibrated by the appropriate regional technical organization. The correspondence between the first measurement and subsequent measurements was evaluated by stratifying the population into three groups by z scores for weightfor-age and for weight-for-height, using U.S. National Center for Health Statistics reference curves. The z score groupings were established at the beginning of the study for three different age groups: younger than 24 months, 24-36 months, and older than 36 months. The two study groups (intervention and nonintervention) were similar sociodemographically, with the following exceptions: maternal employment (more of the mothers of children in the intervention group had jobs outside the home); age (children in the intervention group were significantly older); nutritional status (it was worse in the intervention group); and prevalence of hospital admittance (it was higher in the intervention group). After just the first three months of the study, 32% of the children in the intervention group had moved to a higher nutritional stratum, whereas only 13% of children in the nonintervention group had shown improved nutritional status. The difference in favor of the intervention group increased with the length of the intervention and was higher among older children. These results show that the preschool nutritional assistance program helped to improve the assisted children's nutritional status
SN - 1020-4989
AD - (Correa, Goncalves, Leite) UNICAMP, Depto. de Med. Preventiva e Social, Faculdade de Ciencias Medicas, Campinas, Sao Paulo, Brazil (Goncalves, Padovani) Cd. Universitaria Dr. Zeferino Vaz, Rua Luvervi Pereira de Souza 1151, CEP 13084-031, Campinas, SP, Brazil
ER -

TY - JOUR
ID - 3937
T1 - Mite, cat, and cockroach exposure, allergen sensitisation, and asthma in children: A case-control study of three schools
A1 - Sporik,R.
A1 - Squillace,S.P.
A1 - Ingram,J.M.
A1 - Rakes,G.
A1 - Honsinger,R.W.
A1 - Platts-Mills,T.A.E.
Y1 - 1999///
N1 - 1999255582
English
Journal: Article
KW - EMBASE
KW - Adolescent
KW - article
KW - Asthma/et [Etiology]

KW - atopy/et [Etiology]
KW - bronchus hyperreactivity
KW - case control study
KW - cat
KW - cockroach
KW - controlled study
KW - Dermatophagoides pteronyssinus
KW - Housing
KW - Human
KW - major clinical study
KW - normal human
KW - prick test
KW - priority journal
KW - school child
KW - sensitization
KW - United States
KW - allergen
KW - histamine
RP - NOT IN FILE
SP - 675
EP - 680
JF - Thorax
VL - 54
IS - 8
CY - United Kingdom
N2 - Background: The amount of allergen necessary to sensitise genetically 'at risk' children is unclear. The relation between allergen exposure and asthma is also uncertain. Methods: To ensure a wide range of allergen exposures the data from case-control studies of asthma in children aged 12-14 years attending three schools in Los Alamos, New Mexico and Central Virginia were combined. Skin prick tests to indoor and outdoor allergens and bronchial hyperreactivity to histamine were assessed in children with and without symptoms of asthma. The concentration of mite, cat, and cockroach allergens in dust from the children's homes was used as a marker of exposure. Results: Three hundred and thirty two children (157 with asthmatic symptoms and 175 controls) were investigated. One hundred and eighty three were classified as atopic on the basis of allergen skin prick tests and 68 as asthmatic (symptoms plus bronchial responsiveness). The prevalence and degree of sensitisation to mite and cockroach, but not cat, was strongly associated in atopic children with increasing domestic concentrations of these allergens. Asthma was strongly associated with sensitisation to indoor allergens (p < 10⁻⁶) and weakly to outdoor allergens (p 0.026). There was an association between current asthma and the concentration of mite allergen amongst atopic children (p = 0.008) but not amongst those who were specifically mite sensitised (p = 0.16). Conclusions: The domestic reservoir concentration of mite and cockroach, but not cat, allergen was closely related to the prevalence of sensitisation in atopic children. However, the prevalence of current asthma had a limited relationship to these allergen measurements, suggesting that other factors play a major part in determining which allergic individuals develop asthma
SN - 0040-6376
AD - (Squillace, Ingram, Rakes, Platts-Mills) Asthma and Allergic Disease Center, University of Virginia, Charlottesville, VA 22908, United States (Honsinger) Los Alamos Medical Center, Los Alamos, NM 87544, United States (Sporik) Institute of Respiratory Medicine, Sydney, NSW, Australia
ER -

TY - JOUR
ID - 3938
T1 - Lung function from age 6 to 18 years (cohort study in the city of Linz on males born 1979)
A1 - Neuberger,M.
A1 - Kundi,M.
A1 - Krejci,W.
A1 - Wiesenberger,W.
Y1 - 1999///

N1 - 1999232677
German
Journal: Article
KW - EMBASE
KW - Adolescent
KW - Adult
KW - age
KW - air monitoring
KW - Air Pollution
KW - air quality
KW - ambient air
KW - article
KW - Austria
KW - Child
KW - exhaust gas
KW - Forced Expiratory Volume
KW - Human
KW - lung function
KW - major clinical study
KW - Male
KW - passive smoking
KW - peak expiratory flow
KW - suspended particulate matter
KW - nitrous oxide
KW - sulfur dioxide
RP - NOT IN FILE
SP - 324
EP - 325
JF - Atemwegs- und Lungenkrankheiten
VL - 25
IS - 6
CY - Germany
N2 - For a quarter of a century Austrian environmental protection agencies took serious efforts to improve urban air quality. During the 1980s Austria achieved the highest SO₂ reduction among the signatory states of the Helsinki Protocol. Suspended particulates were reduced simultaneously. NO₂ reduction was less, because reduction of emissions from industry, power plants and motor vehicles was counterbalanced by traffic increase. During the period of highest improvement of air quality in the 3(rd)-largest city of Austria 85% of pupils in compulsory schools (n = 15,045) have been examined by physicians and their lung function results were related to the air quality of their school district. Negative correlations of all lung function parameters were found with SO₂, especially of PEF, but also FEV₁ and FVC. Outdoor NO₂ and passive smoking both showed negative correlations with end- expiratory flow rates. A mixed longitudinal study on 3,451 children over 5 years confirmed cross-sectional results and demonstrated normalization of end-expiratory flow rates in districts where not only SO₂ and TSP but also NO₂ decreased. The cohort born 1979 had experienced the highest reduction of ambient air pollutants in elementary school and was reexamined at age 18 by the same methods. Social class and passive smoking did not show negative effects on lung function up to age 18, however, active smoking impaired growth of MEF₂₅. Higher growth of FVC was found where SO₂ decrease in winter half-years of elementary school exceeded 40 mug/m³ and higher growth of MEF₂₅ and MMEF up to age 18 was found where NO₂ decreased. Part of the improvement of MEF₂₅ could be due to the reduction of fine particulates. A matter of concern is the increase of motor traffic and Diesel engines (which destroy improvements from introduction of catalysts for cars) and continuing chronic effects of NO₂ and fine particulates on lung function growth in the area of small airways
SN - 0341-3055
AD - (Neuberger, Kundi, Krejci, Wiesenberger) Institut fur Umwelthygiene, Universitat Wien, Kinderspitalgasse 15, A - 1095 Wien, Austria
ER -

TY - JOUR
ID - 3939
T1 - Association of cutaneous malignant melanoma with intermittent exposure to ultraviolet radiation: Results of a case-control study in Ontario, Canada
A1 - Walter,S.D.
A1 - King,W.D.
A1 - Marrett,L.D.
Y1 - 1999///
N1 - 1999209001
English
Journal: Article
KW - EMBASE
KW - Adult
KW - article
KW - Canada
KW - case control study
KW - Child
KW - controlled study
KW - Female
KW - host susceptibility
KW - Human
KW - leisure
KW - long term exposure
KW - major clinical study
KW - Male
KW - malignant lentigo/ep [Epidemiology]
KW - Melanoma/ep [Epidemiology]
KW - normal human
KW - priority journal
KW - risk factor
KW - seashore
KW - skin
KW - solar radiation
KW - Sunburn/ep [Epidemiology]
KW - ultraviolet radiation
RP - NOT IN FILE
SP - 418
EP - 427
JF - International Journal of Epidemiology
JA - Int J Epidemiol
VL - 28
IS - 3
CY - United Kingdom
N2 - Background. Although solar radiation is well established as a risk factor for melanoma, it is less clear how the pattern and timing of exposure to ultraviolet (UV) radiation might be important. The particular objective of this study was to evaluate the association of melanoma risk with various measures of intermittent and chronic exposures to UV radiation, and to assess how these exposures interact with other risk factors such as skin type. Methods. Data were analysed from a large case-control study (583 cases, 608 controls) of malignant melanoma, carried out in southern Ontario, Canada. Results. Significant risk increases were identified with several measures of intermittent exposure, including beach vacations in adolescence and in the past 5 years, previous sunburn, and use of sunbeds and sunlamps. Chronic exposure, indicated by days of outdoor activity during adolescence and by occupation in recent adult life, was associated with significantly reduced risk. Subgroup analyses showed: no major risk differences by body site of melanoma: stronger association of lentigo maligna melanoma with intermittent exposure; more pronounced effects of beach vacations and sunburn in younger subjects; and consistently higher risks for intermittent exposures among subjects with skin more susceptible to burning. Conclusions. The data lend limited support to the hypothesis of increased risk associated with

intermittent UV exposure. The findings suggest that future studies should take age at diagnosis, host susceptibility and histological subtype into account
SN - 0300-5771
AD - (Walter) Dept. Clin. Epidemiol. Biostatist., McMaster University, Hamilton, Ont., Canada (King) Dept. of Comm. Hlth. and Epidemiol., Queen's University, Kingston, Ont., Canada (Marrett) Division of Preventive Oncology, Department of Public Health Sciences, University of Toronto, Toronto, Ont., Canada (Walter) Dept. Clin. Epidemiol. Biostatist., Health Sciences Centre, McMaster University, 1200 Main St West, Hamilton, Ont. L8N 3Z5, Canada
ER -

TY - JOUR
ID - 3940
T1 - Measures of effectiveness in a short-term interpersonal Cognitive Problem Solving programme
A1 - Rixon,R.
A1 - Erwin,P.G.
Y1 - 1999///
N1 - 1999118470
English
Journal: Article
KW - EMBASE
KW - article
KW - Behavior Therapy
KW - Cognition
KW - controlled study
KW - Female
KW - Human
KW - human experiment
KW - Male
KW - normal human
KW - Problem Solving
KW - school child
KW - Social Behavior
KW - social status
KW - sociology
RP - NOT IN FILE
SP - 87
EP - 93
JF - Counselling Psychology Quarterly
VL - 12
IS - 1
CY - United Kingdom
N2 - The effects of a classroom-based, short-term Interpersonal Cognitive Problem-Solving programme for 24 7- and 8-year-old children were assessed immediately after a 4-week intervention programme and again after a 4-week follow-up period. A previous study implementing such a short-term programme relied on sociometric testing to measure the social effectiveness of the training and failed to find any changes in social status. The present study incorporated playground observations and found behavioural improvements immediately after training and after the follow-up period for the experimental group. However, confirming the previous study, no significant differences in sociometric status were found. The results highlight the limitations of sociometry as a measure of the effectiveness of a programme of training in Interpersonal Cognitive Problem-solving
SN - 0951-5070
AD - (Rixon, Erwin) Metropolitan University, Manchester, United Kingdom (Erwin) Department of Psychology, Nene-University College, Northampton, Park Campus, Boughton Green Road, Northampton NN2 7AL, United Kingdom
ER -

TY - JOUR
ID - 3941

T1 - Delayed hemorrhage after nonoperative management of blunt hepatic trauma in children: A rare but significant event
A1 - Shilyansky,J.
A1 - Navarro,O.
A1 - Superina,R.A.
A1 - Babyn,P.S.
A1 - Filler,R.M.
A1 - Pearl,R.H.
Y1 - 1999///
N1 - 1999036251
English
Journal: Conference Paper
KW - EMBASE
KW - Adolescent
KW - Adult
KW - bleeding/co [Complication]
KW - Child
KW - clinical feature
KW - computer assisted tomography
KW - conference paper
KW - Human
KW - liver injury
KW - major clinical study
KW - Pain/et [Etiology]
KW - priority journal
KW - scoring system
RP - NOT IN FILE
SP - 60
EP - 64
JF - Journal of Pediatric Surgery
JA - J Pediatr Surg
VL - 34
IS - 1
CY - United States
N2 - Purpose: Nonoperative management of blunt hepatic injury (BHI) has become widely accepted in hemodynamically stable children without ongoing transfusion requirements. However, late hemorrhage, especially after discharge from the hospital can be devastating. The authors report the occurrence of serious late hemorrhage and the sentinel signs and symptoms in children at risk for this complication. Methods: Nonoperative management of hemodynamically stable children included computed tomography (CT) evaluation on admission and hospitalization with bed rest for 7 days, regardless of injury grade. Activity was restricted for 3 months after discharge. Hepatic injuries were classified according to grade, amount of hemoperitoneum, and periportal hypoattenuation. Results: Over 5 years, nonoperative management was successful in 74 of 75 children. One child returned to the hospital 3 days after discharge with recurrent hemorrhage necessitating surgical control. Review of the CT findings demonstrated that he was the only child with severe liver injury in all four classifications. A second child, initially treated at an outside hospital, presented 10 days after injury with ongoing bleeding and died despite surgical intervention. Only the two children with delayed bleeding had persistent right abdominal and shoulder discomfort in the week after BHI. Conclusions: Our findings support nonoperative management of BHI. However, late hemorrhage heralded by persistence of right abdominal and shoulder pain may occur in children with severe hepatic trauma and high injury severity scores in multiple classifications
SN - 0022-3468
AD - (Shilyansky, Navarro, Superina, Babyn, Filler, Pearl) Departments of Surgery and Radiology, Hospital for Sick Children, Toronto, Ont., Canada (Shilyansky) Department of Surgery and Pediatrics, Univ. of Chicago Children's Hospital, MC 4062, Chicago, IL 60637, United States
ER -

TY - JOUR

ID - 3942
T1 - Cardiorespiratory functional assessment in young heart transplant recipients
A1 - Calzolari,A.
A1 - Pastore,E.
A1 - Attias,L.
A1 - Cicini,P.
A1 - Giordano,U.
A1 - Parisi,F.
A1 - Ragonese,P.
A1 - Squitieri,C.
A1 - Turchetta,A.
Y1 - 1998///
N1 - 1999099213
English
Journal: Article
KW - EMBASE
KW - Adolescent
KW - article
KW - Child
KW - clinical article
KW - controlled study
KW - Exercise
KW - Exercise Test
KW - exercise tolerance
KW - Female
KW - functional assessment
KW - graft rejection
KW - heart function
KW - heart output
KW - Heart Rate
KW - heart surgery
KW - heart transplantation
KW - Human
KW - Male
KW - Oxygen Consumption
KW - parental behavior
KW - Postoperative Period
KW - questionnaire
KW - respiratory function
KW - school
KW - systolic blood pressure
KW - tachycardia/ep [Epidemiology]
KW - teacher
KW - treadmill
KW - immunosuppressive agent
KW - oxyhemoglobin/ec [Endogenous Compound]
RP - NOT IN FILE
SP - 107
EP - 111
JF - International Journal of Sports Cardiology
VL - 7
IS - 3
CY - Italy
N2 - Limited data are available on exercise capacity after surgery in young heart transplant recipients. To examine this question we assessed cardiorespiratory responses to exercise in young heart transplant recipients and healthy controls. We then sought a relation between the patients's exercise performance and three clinical post-transplant factors (time since transplantation, rejections per year and immunosuppressive drugs). Fourteen

consecutive heart transplant recipients (6 girls and 8 boys, mean age 9.4 + 0.8 years, range 5-15 years) and 14 healthy matched controls underwent a Bruce treadmill test to determine duration of test, resting and maximum heart rate, maximum systolic blood pressure, peak oxygen consumption (VO₂ peak), cardiac output and oxyhemoglobin saturation. Duration of test and heart rate increase were then compared with time since transplantation, rejections per year and immunosuppressive drugs received. Patients completed a questionnaire about their physical activity in and out of school and the reasons for eventual prohibition. Transplant recipients had tachycardia at rest (126 + 4 b/min; p < 0.001). During exercise they had significantly reduced tolerance (9.3 + 0.4 min; p < 0.001), maximum heart rate (169 + 5 b/min; p < 0.05) and cardiac output (5.65 + 0.6 l/min; p < 0.05), and a heart rate increase from rest to peak exercise lower than controls (p < 0.001), but a similar VO₂ peak. Heart rate increase correlated significantly with post-transplant time (r = 0.55; p < 0.05), rejections per year (r = -0.63; p < 0.05) and number of immunosuppressive drugs (r = -0.60; p < 0.05). Cardiorespiratory functional assessment shows that young heart transplant recipients have significantly reduced exercise capacity. Heart rate increase depends on immunosuppressive drugs, numbers of rejections and time since transplant. After surgery few of these children do physical activity outside school, possibly owing to overprotective parents and teachers and to a lack of suitable supervised facilities
SN - 0393-6066
AD - (Calzolari, Pastore, Attias, Cicini, Giordano, Parisi, Ragonese, Squitieri, Turchetta) Servizio di Medicina dello Sport, Ospedale Pediatrico Bambino Gesu, Piazza S Onofrio 4, 00165 Roma, Italy
ER -

TY - JOUR
ID - 3943
T1 - Epidemiological analyses of the relationship between environmental pollution and asthma
A1 - Duhme,H.
A1 - Weiland,S.K.
A1 - Keil,U.
Y1 - 1998///
N1 - 1999016302
English
Journal: Conference Paper
KW - EMBASE
KW - Adolescent
KW - Adult
KW - Air Pollution
KW - ambient air
KW - Asthma/ep [Epidemiology]
KW - Asthma/et [Etiology]
KW - Bronchitis/ep [Epidemiology]
KW - Bronchitis/et [Etiology]
KW - conference paper
KW - Dust
KW - hay fever/ep [Epidemiology]
KW - hay fever/et [Etiology]
KW - Human
KW - lung function
KW - occupational asthma/ep [Epidemiology]
KW - occupational asthma/et [Etiology]
KW - Particulate Matter
KW - Pollution
KW - priority journal
KW - school child
KW - allergen/to [Drug Toxicity]
KW - gasoline/to [Drug Toxicity]
KW - ozone/to [Drug Toxicity]
KW - sulfur dioxide/to [Drug Toxicity]
KW - tobacco smoke/to [Drug Toxicity]
KW - volatile organic compound/to [Drug Toxicity]

RP - NOT IN FILE
EP - 316
JF - Toxicology Letters
JA - Toxicol Lett
VL - 102-103
IS - pp 307-316
CY - Ireland
N2 - Epidemiological studies performed on children and young adults indicate that the prevalence of asthma has increased over the past decades in a number of countries. Since genetic determinants alone are unlikely to explain these findings, environmental factors are expected to have an important impact on the prevalence of this disease. Environmental pollution, among many other factors, may play a role in the initiation and manifestation of asthma. However, there is little epidemiological evidence for a causal association between the classical major outdoor air pollutants, such as SO₂ or particulate matter, and asthma. Evidence for an association between air pollution from motor vehicles and asthma is still controversial. Thus the indoor environment and other environmental determinants - including lifestyle factors - are likely to be more important as causes of asthma. Epidemiological studies are able to evaluate the effects of environmental factors on the health of human populations in their usual environments. In addition, they can measure associations between long-term environmental exposures and health outcomes. By using refined techniques for exposure and disease assessment, as well as time and cost-effective study designs, environmental epidemiology will increase the knowledge about the role of pollution as a possible cause of asthma. Copyright (C) 1998 Elsevier Science Ireland Ltd. All rights reserved
SN - 0378-4274
AD - (Duhme, Weiland, Keil) Inst. Epidemiol. und Sozialmedizin, Universitat Munster, Domagkstr. 3, D-48129 Munster, Germany
ER -

TY - JOUR
ID - 3944
T1 - A developmental and psychoeducational approach to reducing conflict and abuse in little league and youth sports: The sport psychiatrist's role
A1 - Kamm,R.L.
Y1 - 1998///
N1 - 1998330882
English
Journal: Review
KW - EMBASE
KW - Adolescent
KW - baseball
KW - Character
KW - Child
KW - Child Abuse
KW - Child Development
KW - Child Psychiatry
KW - Conflict
KW - Cooperation
KW - education program
KW - Human
KW - parent
KW - priority journal
KW - psychiatrist
KW - review
RP - NOT IN FILE
SP - 891
EP - 918
JF - Child and Adolescent Psychiatric Clinics of North America
VL - 7
IS - 4

CY - United States
N2 - To stress a point made earlier, whether backyard or Little League, the tone of the youth sports experience is greatly influenced by the player and team selection process. All possible steps should therefore be taken to ensure that the draft is held on high moral ground, and that the adult participants, even on the Major Little League level, behave cooperatively rather than competitively. If it is the community's hope that Little League will 'build character, and not characters,' it must embrace Shields and Bredemeier's work and flood the Draft Room with the four virtues of compassion, fairness, sportspersonship, and integrity. In one attempt to achieve fairness and balance, some leagues enter player evaluations into a data base and allow a properly programmed computer to project equal teams. It might be best, however, to borrow a page from backyard baseball, and make team selection a more cooperative venture. Wolff has proposed such a draft model. He recommends that each child's name be placed on a big blackboard at the beginning of the draft. All assembled give their assessment of each child's baseball ability, and a consensus skill-level number (one through five) is entered next to each player's name. If there are 72 names, and the league wishes to form six teams of 12 players each, dividing up the rated players so that the skill levels balance would assure everyone (coaches, parents, the league, and by extension, the community) that the teams were of relatively equal strength. No coach at this point would know to which team he or she was being assigned, so there would be no motive for 'stacking' a given team. Each team would be designated by a letter of the alphabet, and the six letters would be thrown into a hat. The six coaches would then blindly pick a team from the hat. If a coach desired that his or her child play on his or her team, fair adjustments (trades) could be made subject to majority agreement. The three draft models can be summarized as follows: Draft Model 1 Public tryouts Previous year rating Coach makes selections Can be very competitive, lacking in 'character' Can result in very unequal teams Draft Model 2 Player evaluations placed into a data base Properly programmed computer projects equal teams Draft Model 3 All names placed on a blackboard Relative merits of players discussed Equal team drawn up and placed into a hat Adjustments can be made for coach's child If practical, this author suggests that coaches not pick a name for the team until the first team meeting or practice, when that task can be given to the children. In a symbolic way, this returns some of the sport to them while encouraging social interaction among new teammates, and helping the coach detect who the leaders are. Names of professional teams in the major sports, especially baseball, are to be avoided, as they fuel longstanding unconscious associations and fantasies, and may subtly tilt all participants toward the professional 'win at all costs' mentality. No one draft model is perfect for every town, and even the most ethical attempt to achieve balance among teams can be severely tested by parents who request that their athlete be placed on the same team as another child for social or car pool reasons. Such requests are not inviolate, however. For example, they do not usually dictate placement in school classes, and car pools are frequently disrupted when children, in individual sports such as Tae Kwan Do, reach different ability levels, and so attend practices at different times. Baseball is no longer the national pastime and, as we approach the millennium, American children have too many other attractive, competing interests and time demands to spontaneously organize a pick-up game. One coach shared with the author that his saddest moment in CAP League came when he arrived at 6 PM at a field that had been 'reserved' for his team, and found a group of boys who were playing a pick- up game. The coach's impulse was to set his boys free so that all could mingle and organize their own game. He bemoaned the fact that he had to ask the interlopers to leave so that his team could practice. Realistically, players live further from each other in the suburbs and it is hard to get a game together. It is frequently unsafe for children 8, 9, and 10 years of age to be at a playground or sandlot unsupervised by an adult. Certainly walking home at dusk, even in pairs, after a baseball game can be dangerous in many neighborhoods. Smith and Smoll and many others have therefore concluded that the real issue is not whether adult organized youth sports should exist, but rather how we can increase the likelihood that the outcome of participation will be favorable to our children. A favorable outcome hinges on the two main variables mentioned at the beginning of this article. Psychiatrists have the expertise to affect both of these variables positively and, in so doing, move one step closer to a youth sports system that is true to Carl Stotz's original vision, a gift, not a burden, to our children
SN - 1056-4993
AD - (Kamm) 257 Monmouth Road, Oakhurst, NJ 07755, United States
ER -

TY - JOUR
ID - 3945
T1 - Effects of damp and mould in the home on respiratory health: A review of the literature
A1 - Peat,J.K.
A1 - Dickerson,J.
A1 - Li,J.

Y1 - 1998///
N1 - 1998247760
English
Journal: Review
KW - EMBASE
KW - Adult
KW - air pollutant
KW - air quality
KW - Child
KW - Dermatophagoides pteronyssinus
KW - Environmental Exposure
KW - Home
KW - Human
KW - Humidity
KW - major clinical study
KW - mould
KW - priority journal
KW - respiratory tract disease/ep [Epidemiology]
KW - respiratory tract disease/et [Etiology]
KW - respiratory tract disease/pc [Prevention]
KW - review
RP - NOT IN FILE
SP - 120
EP - 128
JF - Allergy: European Journal of Allergy and Clinical Immunology
VL - 53
IS - 2
CY - Denmark
N2 - This review examines whether there is a direct or indirect relation between damp or mould in the home and respiratory health. Home dampness is thought to have health consequences because it has the potential to increase the proliferation of house-dust mites and moulds, both of which are allergenic. The results from the many studies conducted to investigate whether damp and mould are associated with health outcomes are difficult to compare because the methods of measuring exposures and health outcomes have not been standardized. However, the studies that have been conducted in children are probably the most reliable because the confounding effects of active smoking or occupational exposures are absent, and because the presence of symptoms of cough and wheeze have been consistently investigated in many studies. The increased risk of children having these symptoms if the home has damp or mould is fairly small with an odds ratio that is generally in the range 1.5-3.5, these estimates being statistically significant when the sample size has been large enough. This range is consistent with the measured effects of other environmental exposures which are considered important to health, such as environmental tobacco smoke or outdoor air pollutants. The potential benefits of reducing mould in the home have not been investigated, and the few studies that have investigated health improvements as a result of increasing ventilation or reducing damp in order to reduce house-dust mite levels suggest that this intervention is expensive, requires a large commitment, and is unlikely to be successful in the long term. This implies that houses need to be specifically designed for primary prevention of respiratory problems associated with indoor allergen proliferation rather than using post hoc procedures to improve indoor climate and reduce allergen load as a secondary or tertiary preventive strategy
SN - 0105-4538
AD - (Peat, Li) Department of Medicine, University of Sydney, Sydney, NSW, Australia (Dickerson) Institute of Respiratory Medicine, University of Sydney, Sydney, NSW 2006, Australia (Peat) Sydney University, Dept. of Pediatrics and Child Health, New Children's Hospital, Westmead, NSW 2145, Australia
ER -

TY - JOUR
ID - 3946
T1 - Rehabilitation and Research Centre for Torture Victims, Philippines, December 1989: Refugee children from the Middle East
A1 - Montgomery,E.

Y1 - 1998///
N1 - 1998053356
English
Journal: Conference Paper
KW - EMBASE
KW - Adolescent
KW - anxiety neurosis
KW - Child
KW - conference paper
KW - Denmark
KW - emotion
KW - father
KW - Human
KW - Interview
KW - major clinical study
KW - Middle East
KW - mother
KW - parent
KW - priority journal
KW - questionnaire
KW - red cross
KW - refugee
KW - Risk Assessment
KW - symptom
KW - torture/ep [Epidemiology]
KW - torture/rh [Rehabilitation]
KW - victim
KW - violence/ep [Epidemiology]
KW - violence/rh [Rehabilitation]
KW - War
RP - NOT IN FILE
SP - I
EP - 152
JF - Scandinavian Journal of Social Medicine, Supplement
VL - 25
IS - 54
CY - Norway
N2 - Objective: To map the frequency (prevalence) of torture victims among parents in asylum seeking Middle Eastern refugee families, to map the occurrence (prevalence) of experiences of war and other forms of organised violence among the children in these families, to map the occurrence (prevalence) of emotional symptoms and behavioural problems among the children, and to identify risk indicators and modifying factors for anxiety symptoms among the children. Design: Interview with parents using a structured interview questionnaire developed for this study. Validated through a blinded semi-structured interview conducted with approximately 1/3 of the families. Auspices: The study has been carried out by the Rehabilitation and Research Centre for Torture Victims (RCT) in cooperation with the Danish Red Cross. Material: Structured interviews with parents regarding 311 children aged 3-15 from 149 families, all registered as asylum seekers from the Middle East between February 1, 1992 and April 30, 1993. The response was 90.4%. Principal variables: background (past-past) - social and demographic data; trauma complex (past) - war-related life circumstances (conditions) and experiences of war and other forms of organised violence such as loss, separation, direct exposure to violence and witnessing acts of violence (specific events and changes of life conditions); present life context (past-present) - family circumstances upon arrival in Denmark; effect (present) - the child's current psychological state. Results: 28% of the parents (44% of the fathers and 13% of the mothers) had been tortured, to the effect that 51% of the children were part of a family including a survivor of torture. The most frequent specific types of violence-related events or circumstances were 'lived in a refugee camp outside the home country' (92%), 'lived under conditions of war' (89%) and 'been on the run with parents' (89%). Twenty percent of the children had lost one parent, and another 60% had been separated from one parent for more than a month. The highest prevalence of emotional symptoms were found within the anxiety dimension, as 67% of the children were

assessed as being clinically anxious. The most important risk indicators for anxiety were 'lived in a refugee camp outside the home country', 'part of a torture surviving family', 'lack of opportunities for play with other children', 'beaten/kicked by an official', and 'loss of father'. Current parental behaviour was also an important risk indicator for anxiety, if the mother or father hit or punished the child more than was the case prior to arrival in Denmark. The most important anxiety modifying factor was arrival in Denmark in the company of both parents. Conclusions: Asylum seeking refugee children from the Middle East have had many experiences of war and other forms of organised violence. The children frequently reacted with anxiety and with other symptoms of emotional instability. Prevalent anxiety symptoms correlated both with previous living conditions and present family situation. Living under prolonged conditions influenced by war and other forms of organised violence (prevalence) were found to a higher degree to be risk indicators for anxiety than were specific events or changes of life conditions (incidence)
SN - 0301-7311
AD - (Montgomery) Rehab. Res. Ctr. Torture Victims, Copenhagen, Denmark
ER -

TY - JOUR
ID - 3947
T1 - Childhood obesity: a risk factor for traumatic injuries to anterior teeth
A1 - Petti,S.
A1 - Cairella,G.
A1 - Tarsitani,G.
Y1 - 1997///
N1 - 9558511
English
Journal: Article
KW - EMBASE
KW - Accident Proneness
KW - Age Distribution
KW - article
KW - Child
KW - Child Behavior
KW - cross-sectional study
KW - Female
KW - Human
KW - Italy/ep [Epidemiology]
KW - Lifestyle
KW - Male
KW - obesity/co [Complication]
KW - Prevalence
KW - psychological aspect
KW - questionnaire
KW - risk factor
KW - Sex Ratio
KW - tooth injury/ep [Epidemiology]
KW - tooth injury/et [Etiology]
RP - NOT IN FILE
SP - 285
EP - 288
JF - Endodontics & dental traumatology
VL - 13
IS - 6
CY - Denmark
N2 - The relationship between obesity and traumatic dental injuries was evaluated in a study population of 938 6- to 11-year-old schoolchildren from Rome, Italy, and it was suggested by the lifestyle and the physical activity of obese children, which, according to many authors, is different from the lifestyle of other children. The subjects were examined at school: presence and type of dental injury, overjet, incisor protrusion, upper lip incompetence and presence of obesity were recorded. Trauma predisposition was evaluated with a questionnaire

investigating the behaviour which may predispose to impacts. Six out of eight questions in this questionnaire concerned the children's lifestyle and the trauma predisposition score was therefore also considered an estimate of lifestyle and physical activity. The dental injury prevalence of the study population was 21.3% and obesity prevalence was 11.4%. Of the obese children 30.8% had dental injuries vs. only 20.0% of non-obese subjects (P = 0.007). Obese children had only enamel and enamel-dentine fractures and the main reported trauma cause was indoor play; conversely, lean children also had other types of injuries and the most frequently reported cause was outdoor play. The main predisposing factors significantly affecting the probability of dental injury, evaluated with multiple logistic regression, were upper lip uncoverage (OR: 1.23; P = 0.02), overjet larger than 3 mm (OR: 1.68; P = 0.001) and obesity (OR: 1.45; P = 0.01). Surprisingly, trauma predisposition was a protective factor (OR: 0.50; P = 0.00001). The results of this study were explained by the significant inverse correlation between obesity and trauma-predisposing behaviour, thus suggesting that subjects frequently playing sports and lively games were not only less obese but also more skillful and, for this reason, less prone to trauma when they fell or sustained impacts
SN - 0109-2502
AD - (Petti, Cairella, Tarsitani) G. Sanarelli Hygiene Institute, La Sapienza University, Rome, Italy
ER -

TY - JOUR
ID - 3948
T1 - A descriptive analysis of children's playground injuries in the United States 1990-4
A1 - Mack,M.G.
A1 - Hudson,S.
A1 - Thompson,D.
Y1 - 1997///
N1 - 9213154
English
Journal: Article
KW - EMBASE
KW - Adolescent
KW - Age Distribution
KW - apparatus,equipment and supplies
KW - article
KW - Child
KW - Classification
KW - Epidemiology
KW - Female
KW - Hospitalization
KW - Human
KW - Infant
KW - information processing
KW - injury/ep [Epidemiology]
KW - injury/et [Etiology]
KW - injury/pc [Prevention]
KW - injury scale
KW - Male
KW - Newborn
KW - preschool child
KW - Recreation
KW - risk factor
KW - Safety
KW - Sex Ratio
KW - Statistics
KW - Survival Rate
KW - United States/ep [Epidemiology]
RP - NOT IN FILE
SP - 100
EP - 103

JF - Injury prevention : journal of the International Society for Child and Adolescent Injury Prevention
VL - 3
IS - 2
CY - United Kingdom
N2 - OBJECTIVES: To review playground injury statistics over a five year period in order to develop an awareness of how and where children in the United States are being injured. METHODS: All data are based on the United States Consumer Product Safety Commission's National Electronic Injury Surveillance System (NEISS) for playground related injuries during 1990-4. The surveillance data includes injuries recorded in more than 90 hospital emergency departments located throughout the United States. RESULTS: Each year there are roughly 211,000 preschool or elementary school-children in the United States who receive emergency department care for injuries associated with playground equipment. On average, 17 of these cases result in death. 70% of all injuries occur on public playgrounds, with nearly one third classified as severe. Swings, climbers, and slides are the pieces of playground equipment associated with 88% of all NEISS reported injuries. Falls to the surface are responsible for 70%. CONCLUSIONS: NEISS playground injury statistics contribute to our understanding of playground injuries. By identifying where and how children are injured, suggestions can be made in an attempt to make playgrounds safer
SN - 1353-8047
AD - (Mack, Hudson, Thompson) National Program for Playground Safety, University of Northern Iowa, School of Health, Physical Education, and Leisure Services, Cedar Falls 50614-0161, USA
ER -

TY - JOUR
ID - 3949
T1 - Behaviors and blood lead levels of children in a lead-mining area and a comparison community
A1 - Murgueytio,A.M.
A1 - Evans,R.G.
A1 - Sterling,D.
A1 - Serrano,F.
A1 - Roberts,D.
Y1 - 1997///
N1 - 1998049042
English
Journal: Article
KW - EMBASE
KW - article
KW - Child
KW - Child Behavior
KW - controlled study
KW - Demography
KW - Environmental Exposure
KW - Female
KW - Health Education
KW - Home
KW - house dust
KW - Human
KW - Income
KW - Infant
KW - lead blood level
KW - Lead Poisoning/ep [Epidemiology]
KW - lead poisoning/pc [Prevention]
KW - major clinical study
KW - Male
KW - Mining
KW - personal hygiene
KW - socioeconomics
KW - soil pollution
KW - lead/to [Drug Toxicity]

KW - Paint
RP - NOT IN FILE
SP - 14
EP - 20
JF - Journal of Environmental Health
JA - J Environ Health
VL - 60
IS - 6
CY - United States
N2 - This study investigates the relationship between behavioral and other modifiable factors and blood lead in children living in a lead-mining community and in a comparison group of children. Children six to 71 months of age were selected from a community that was heavily contaminated with lead- mining waste and from a comparison community. Participants were interviewed, and venous blood was collected for lead analysis. Environmental measurements of soil, dust, and paint were made. Study results indicate that average blood lead levels and environmental measurements were significantly higher in the mining community. Factors that were related to blood lead levels included income, education, home ownership, age of home, playing in grassy areas rather than dirt, putting nonfood items in mouth, bathing and washing practices, number of hours playing outside, taking nonfood items outside, swallowing nonfood items, and putting paint chips in mouth. These factors explained more of the variation in blood lead levels in the control group than in the mining group. Lead intervention strategies that consist only of education designed to modify behavior might be less effective in high- exposure areas such as those where lead-mining and smelting operations occur. Interventions that combine education with remedial activities are more effective in prevention of lead exposure
SN - 0022-0892
AD - (Murgueytio) School of Public Health, Saint Louis University, 3663 Lindell Blvd., St. Louis, MO 63108, United States
ER -

TY - JOUR
ID - 3950
T1 - Generalization of social skills to peer interactions in a child with language delays
A1 - Johnson,C.
A1 - Golden,J.
Y1 - 1997///
N1 - 1997362832
English
Journal: Article
KW - EMBASE
KW - article
KW - Child
KW - Day Care
KW - group psychology
KW - Human
KW - language disability
KW - Male
KW - play
KW - preschool child
KW - social adaptation
KW - Social Interaction
RP - NOT IN FILE
SP - 133
EP - 147
JF - Behavioral Interventions
VL - 12
IS - 3
CY - United Kingdom
N2 - Researchers in this study have attempted to determine whether integrating generalization promoting strategies into intervention procedures would enhance the generalization and maintenance of social skills to peer

interactions in a preschool child with language delays. This intervention took place in the natural environment of the day care facility where the child was enrolled. A multiple baseline across behaviors design addressed social response behavior, approach behavior, and play organizers. The intervention utilized a prompting and social reinforcement procedure with minimal adaptations of the onging classroom activities to systematically improve the target child's peer interactions. Several generalization promoting strategies were also used. Improvements in all three types of behavior and generalization of treatment effects across persons, settings, and time were observed. These results lend support for the value of integrating several generalization promoting strategies pre-experimentally into the intervention instead of relying on post hoc evaluation of intervention
SN - 1072-0847
AD - (Johnson, Golden) East Carolina University, Greenville, United States (Golden) Department of Psychology, East Carolina University, Greenville, NC 27858, United States
ER -

TY - JOUR
ID - 3951
T1 - Collective food intoxication hospital time management
A1 - Triaureau,G.
A1 - marre-Jallet,I.
A1 - Khouri,M.
A1 - Andre,P.
A1 - Lalande,G.
Y1 - 1997///
N1 - 1997202688
French
Journal: Conference Paper
KW - EMBASE
KW - Child
KW - conference paper
KW - food poisoning/et [Etiology]
KW - gastrointestinal infection/et [Etiology]
KW - Human
KW - major clinical study
RP - NOT IN FILE
SP - 114
EP - 115
JF - Urgences Medicales
VL - 16
IS - 3
CY - France
N2 - The Mash program has been activated in Nevers Hospital after scholar food intoxication. 110 children were concerned and 30 have been hospitalized. Scholar infirmary did not evaluate the food exposure as well. The select medical center has been installed with delay in school, where a large number of victims have been examinated in second time. Emergency department was not saturated. A department with 40 beds was created with medical announcement. In France, declaration to health authorities is mandatory, epidemiological research begin as soon as possible. White Program test was a good exercise for medical personnel outside and inside the hospital
SN - 0923-2524
AD - (Triaureau, Demarre-Jallet, Khouri, Lalande) Service d'Accueil des Urgences, Centre Hospitalier, 58000 Nevers, France (Andre) Samu-Smur, Centre Hospitalier, 58000 Nevers, France
ER -

TY - JOUR
ID - 3952
T1 - Ozone personal exposures and health effects for selected groups residing in the Fraser Valley
A1 - Brauer,M.
A1 - Brook,J.R.
Y1 - 1997///

N1 - 1997182999
English
Journal: Article
KW - EMBASE
KW - Adolescent
KW - Adult
KW - Aged
KW - agricultural worker
KW - air monitoring
KW - Air Pollution
KW - air quality
KW - ambient air
KW - article
KW - Canada
KW - Environmental Exposure
KW - Female
KW - health hazard
KW - Human
KW - human experiment
KW - lung function
KW - Male
KW - normal human
KW - personal monitoring
KW - photochemistry
KW - priority journal
KW - school child
KW - summer
KW - Temperature
KW - Time
KW - Ozone
RP - NOT IN FILE
SP - 2113
EP - 2121
JF - Atmospheric Environment
VL - 31
IS - 14
CY - United Kingdom
N2 - Due to concern regarding poor ambient air quality in the Fraser Valley, a series of exposure and health effects assessments were performed to evaluate the impact of summer photochemical air pollution. In 1992 and 1993, three groups of individuals were selected for personal monitoring of ozone exposure, based on prior expectations of their activity patterns. The first group spent a majority of the work day indoors or commuting, the second group spent more time outdoors and the third group spent the entire personal monitoring period outdoors. Time-activity data were collected for the first two groups and differences in personal ozone exposures were found to be associated with the fraction of time a person spent outdoors. Similarly, differences among groups in the mean ozone exposure were associated with time spent outdoors. These results and other exposure information were used to design a study of the health impacts of summer ambient air pollution that was conducted during the time period of the Pacific '93 held campaign. Aerosol acidity levels in the Fraser Valley were observed to be very low in 1992 so the health study focused on the effects of ozone exposure. The subjects were adult farm workers (26 male, 32 female; mean age 44.4, range 10-69) who spent the entire working day outdoors (a subset of group 3 above). Lung function measurements were made twice daily on each subject, once before and once after their work shift, from 23 June-26 August 1993. Ambient O₃ concentrations were measured continuously at several nearby locations. In a regression model including individual lung function level, date, temperature and daily maximum O₃, a statistically significant (p < 0.001) negative association was observed between ozone and lung function. This association between ozone and reduced lung function was still apparent the following day, suggesting a persistent ozone effect. These results indicate that exposure to ambient O₃ concentrations below either the U.S. NAAQS (120 ppb) or

the Canadian Air Quality Objective (82 ppb) may have an adverse effect on the lung function of people engaged in outdoor work for several hours a day
SN - 1352-2310
AD - (Brauer) University of British Columbia, Dept. of Med.-Respiratory Division, Occupational Hygiene Program, 2206 East Mall, Vancouver, BC V6T 1Z3, Canada (Brook) Atmospheric Environment Service, 4905 Dufferin Street, Downsview, Ont. M3H 5T4, Canada
ER -

TY - JOUR
ID - 3953
T1 - Physical exercise, sport and television: Cross-sectional analyses among 1368 children attending compulsory school
A1 - Attina,D.A.
A1 - Catalano,G.
A1 - Surace,C.
Y1 - 1997///
N1 - 1997182720
Italian
Journal: Article
KW - EMBASE
KW - article
KW - Child
KW - controlled study
KW - Exercise
KW - Health Promotion
KW - Human
KW - Italy
KW - normal human
KW - Physical Activity
KW - school child
KW - Sports Medicine
KW - Television
RP - NOT IN FILE
SP - 9
EP - 14
JF - Medicina dello Sport
VL - 50
IS - 1
CY - Italy
N2 - A study has been conducted on a sample of 1366 children attending compulsory school in order to examine the relationship between the time spent watching television and physical activity. A distinction has been made according to the time of the day when the children watch television: before going to school, back from school throughout the afternoon, and after dinner. As far as physical activity is concerned, the weekly frequency and the duration both of the organized physical activity and of the outdoor games have been considered separatedly. The practice of organized physical activity does not have a considerable influence on television viewing unlike the physical activity carried out by children after school. In the afternoon, when children dedicate themselves to outdoor games and sport activities, the time spent watching television is inversely proportional to the physical activity practiced 3 hours per week by the youngest children and 5.9 by the grown-ups, against 5.6 and 7 hours per week spent by those children who attend primary school and by the adolescents of secondary school: those who did not practice any physical activity (group A and D), those who practiced physical exercises less than an hour a day (group B and E) and more than an hour a day (group C and F). It has been noted that, considering the groups of those children who practiced physical activity for a longer period of time, the hours spent watching television were significantly lower as far as the children attending primary school are concerned (2.55+/1.19 vs 2.39+/1.12 vs 2.16+/1.14) ($p < 0.005$ A-C, $p<0.05$ B-C) and those attending secondary schools (2.73+/1.15 vs 2.55+/1.03 vs 2.39+/1.02) ($p<0.01$ D-F, $p<0.05$ E-F). These data is mainly due to a variation in the number of hours spent watching television during the afternoon by children attending both primary (1.28+/0.87 vs 1.19+/0.86 vs 0.99+/0.81) ($p<0.001$ A-C, $p<0.05$ B-C) and secondary

schools (1.00+/0.88 vs 0,89+/0.84 vs 0.73+/0.69) (p<0.01 D-F, p<0.01 E-F). The negative effects of a prolonged time spent watching television could be contrasted promoting any kind of physical activity and of outdoor games
SN - 0025-7826
AD - (Attina, Catalano, Surace) Unita Operativa Med. dello S., Reggio Calabria, Italy (Attina) Via S. C. Diram. Costantino, 12, 89121 Reggio Calabria, Italy
ER -

TY - JOUR
ID - 3954
T1 - Heavy metal pollution in children playgrounds in Hong Kong and its health implications
A1 - Wong,J.W.C.
A1 - Mak,N.K.
Y1 - 1997///
N1 - 1997047625
English
Journal: Article
KW - EMBASE
KW - Air Pollution
KW - article
KW - Child
KW - Dust
KW - health hazard
KW - Hong Kong
KW - Human
KW - industrial area
KW - Recreation
KW - Soil
KW - Traffic
KW - urban area
KW - cadmium
KW - copper
KW - heavy metal
KW - Lead
KW - zinc
RP - NOT IN FILE
SP - 109
EP - 115
JF - Environmental Technology
VL - 18
IS - 1
CY - United Kingdom
N2 - Due to the scarcity of land in Hong Kong, most of the children playgrounds are being built on marginal lands which may be subjected to serious atmospheric emission. Hence, children playing in these playgrounds are exposed to a potential health risk. The aim of this study was to evaluate heavy metal pollution in children playgrounds using surface dusts and soils as indicators. Surface dust and soil samples were collected from seven selected playgrounds with different levels of traffic volume and urban development for the determination of total Cd, Cu, Pb and Zn concentrations. The mean Cd, Cu, Pb and Zn concentrations of surface dusts were 2.63, 201, 302 and 1517 mg kg⁻¹ respectively, whereas for soils they were 2.30, 28.4, 195 and 237 mg kg⁻¹. The results indicated elevated concentrations of Cd, Cu, Pb and Zn in both dust and soil samples especially for those samples collected from busy commercial and industrial areas i.e., Mei Foo, Tsim Sha Tsui and North Point. Surface dusts were enriched relative to soils in each metal, on average, by factors ranging from 1.1 to 7.1. The metal contents in dust and soil samples were significantly correlated with traffic volume. Significant correlations were also noted among all the metals studied indicating that Pb and Zn in the samples may be due to the same source i.e., automobile. The soil Pb and Zn contents of Mei Foo and North Point were higher than the investigation level of the Dutch Criteria for these two metals which indicated that further investigation is required to confirm their health hazard on children playing in these playgrounds

SN - 0959-3330
AD - (Wong, Mak) Department of Biology, Hong Kong Baptist University, Kowloon Tong, Hong Kong
ER -

TY - JOUR
ID - 3955
T1 - Clothing drawstring entrapment in playground slides and school buses: Contributing factors and potential interventions
A1 - Drago,D.A.
A1 - Winston,F.K.
A1 - Baker,S.P.
Y1 - 1997///
N1 - 1997026151
English
Journal: Article
KW - EMBASE
KW - Accident Prevention
KW - Adolescent
KW - article
KW - Child
KW - Child Behavior
KW - clinical article
KW - Clothing
KW - Female
KW - health hazard
KW - Human
KW - Male
KW - priority journal
KW - Risk Assessment
KW - Safety
KW - school
RP - NOT IN FILE
SP - 72
EP - 77
JF - Archives of Pediatrics and Adolescent Medicine
VL - 151
IS - 1
CY - United States
N2 - Objectives: To identify factors associated with the entrapment of children by clothing drawstrings and to explore intervention strategies that could be implemented through a voluntary standard to reduce or eliminate injuries. Design: Fifty eight cases reported to the US Consumer Product Safety Commission between January 1, 1985, and June 30, 1995, that involved the entrapment of children's clothing drawstrings in other products were reviewed. A computerized database was created for information about the victim, clothing, object in which the drawstring caught, and injury circumstances. Factors were analyzed by their contribution to preevent, event, and postevent phases, using a modified Haddon matrix. Results: Two primary and distinct hazard patterns were identified: (1) strangulation by hanging associated with the snagging of a hood or neck drawstring in a gap between segments of playground slides and (2) vehicular dragging associated with the snagging of a waist or bottom drawstring in a school bus handrail or door. Conclusions: Potential intervention strategies targeting clothing drawstrings include (1) remove drawstrings; (2) shorten drawstrings, sew them to the channel, and remove toggles and knots; and (3) design breakaway drawstrings. Based on available data, the only feasible intervention is removal of drawstrings. Improved supervision and increased awareness are in themselves infeasible interventions, but they can be interim or supplemental measures
SN - 1072-4710
AD - (Drago, Baker) Ctr. for Injury Research and Policy, Johns Hopkins University, School of Hygiene and Public Health, Baltimore, MD, United States (Winston) Children's Hospital of Philadelphia, Philadelphia, PA, United States (Baker) Johns Hopkins Ctr. Inj. Res./Plcy., 624 N Broadway, Baltimore, MD 21205-1996, United States

ER -

TY - JOUR
ID - 3956
T1 - Effects of tropospheric ozone on the respiratory tract of children
A1 - Frischer,T.
Y1 - 1996///
N1 - 1997016338
German
Journal: Article
KW - EMBASE
KW - Air Pollution
KW - article
KW - Child
KW - Human
KW - lung function
KW - neutrophil
KW - respiratory tract disease/et [Etiology]
KW - respiratory tract inflammation/et [Etiology]
KW - Ozone
RP - NOT IN FILE
SP - 185
EP - 187
JF - Padiatrie und Padologie
VL - 31
IS - 6
CY - Austria
N2 - Ozone constitutes a major outdoor air pollutant. Ambient air quality standards are frequently exceeded in large parts of Europe each summer. The pathophysiologic mechanism is related to local irritation of afferent nerve fibres in the airways as well as to inflammation of the upper and lower airways which is dominated by an influx of polymorph nuclear granulocytes. Decrements of lung function depend on the inhaled dose (concentration x time of exposure x minute ventilation) and tend to decrease after recurrent exposure. Due to physical activity pattern children are particularly susceptible to the effects of ozone. However, at current exposure levels respiratory symptoms are unlikely to occur. Data on long term effects such as morbidity or impaired growth of the respiratory tract of children are sparse. Hence, chronic adverse effects cannot be currently excluded
SN - 0030-9338
AD - (Frischer) Univ.-Kinderklinik, Wahringer-Gurtel 18, A-1090 Wien, Austria
ER -

TY - JOUR
ID - 3957
T1 - Cellular fatty acid analysis and enzyme profiles of Porphyromonas catoniae - A frequent colonizer of the oral cavity in children
A1 - Kononen,E.
A1 - Vaisanen,M.-L.
A1 - Finegold,S.M.
A1 - Heine,R.
A1 - Jousimies-Somer,H.
Y1 - 1996///
N1 - 1996380606
English
Journal: Article
KW - EMBASE
KW - abdominal abscess
KW - anaerobic bacterium
KW - article

KW - bacterium identification
KW - bacterium isolation
KW - Bacteroides
KW - Child
KW - enzyme activity
KW - gingiva
KW - Human
KW - lipid composition
KW - mouth cavity
KW - mouth flora
KW - mouth mucosa
KW - nonhuman
KW - normal human
KW - Porphyromonas
KW - priority journal
KW - alpha galactosidase/ec [Endogenous Compound]
KW - alpha glucosidase/ec [Endogenous Compound]
KW - alpha levo fucosidase/ec [Endogenous Compound]
KW - bacterial enzyme/ec [Endogenous Compound]
KW - beta galactosidase/ec [Endogenous Compound]
KW - chymotrypsin/ec [Endogenous Compound]
KW - fatty acid/ec [Endogenous Compound]
KW - n acetyl beta glucosaminidase/ec [Endogenous Compound]
KW - trypsin/ec [Endogenous Compound]
RP - NOT IN FILE
SP - 329
EP - 335
JF - Anaerobe
VL - 2
IS - 5
CY - United Kingdom
N2 - During a previous study on the Gram-negative anaerobic microflora in children we frequently isolated a non-pigmented Prevotella-like species with phenotypic characteristics not conforming to those of previously described species at the time. Among recently described Gram-negative anaerobic bacilli isolated from gingival crevices Porphyromonas catoniae (Bacteroides D26) most closely resembled our isolates biochemically which also typically produced a large amount of propionic acid. Sixty-three isolates originated from oral mucosal surfaces pooled to saliva and from the gingival crevices of 23 healthy children (mean age 32 months). In addition, one biochemically similar strain which was isolated from an abdominal abscess was included in the tests. The aim of the present study was to determine the cellular fatty acid (CFA) composition for the precise identification of our isolates, and further, to determine the enzyme profiles by API ZYM to emend the description of this species. CFA profiles agreed with the identification of Bacteroides D26 (P. catoniae), API ZYM patterns revealed positive reactions for beta-galactosidase, alpha-glucosidase (except for one isolate), N-acetyl-beta-glucosaminidase and alpha-fucosidase, and variable reactions for leucine arylamidase, trypsin, chymotrypsin and alpha-galactosidase. However, numerical analysis of the CFA data separated this species into at least three subgroups. Twenty-five oral isolates formed one subgroup which most closely resembled the type strain (ATCC 51270). The ubiquitous presence of P. catoniae in moderate numbers in the studied oral samples suggests that this anaerobic species belongs to the normal oral flora of children even at this young age; nevertheless, it may occasionally act as an opportunistic pathogen outside the oral cavity
SN - 1075-9964
AD - (Kononen, Vaisanen, Heine, Jousimies-Somer) Anaerobe Reference Laboratory, National Public Health Institute, Helsinki, Finland (Kononen) Department of Periodontology, Institute of Dentistry, University of Helsinki, Helsinki, Finland (Finegold) Wadsworth V.A. Medical Center, W. Los Angeles and UCLA Sch. of M., Los Angeles, CA, United States (Kononen) Anaerobe Reference Laboratory, National Public Health Institute, Mannerheimintie 166, FIN-00300 Helsinki, Finland
ER -

TY - JOUR

ID - 3958
T1 - An epidemiological study of influenza viruses among Chinese farm families with household ducks and pigs
A1 - Shu,L.L.
A1 - Zhou,N.N.
A1 - Sharp,G.B.
A1 - He,S.Q.
A1 - Zhang,T.J.
A1 - Zou,W.W.
A1 - Webster,R.G.
Y1 - 1996///
N1 - 1996258592
English
Journal: Article
KW - EMBASE
KW - Adult
KW - agricultural worker
KW - article
KW - Child
KW - China
KW - Chinese
KW - duck
KW - enzyme activity
KW - Family
KW - feces analysis
KW - Genotype
KW - household
KW - Human
KW - influenza/ep [Epidemiology]
KW - Influenza virus
KW - Influenza virus A/ep [Epidemiology]
KW - Interview
KW - nonhuman
KW - normal human
KW - respiratory tract infection/ep [Epidemiology]
KW - seasonal variation
KW - serum
KW - Swine
KW - throat culture
KW - viral genetics
KW - virus characterization
KW - virus isolation
KW - virus transmission
KW - sialidase/ec [Endogenous Compound]
RP - NOT IN FILE
SP - 179
EP - 188
JF - Epidemiology and Infection
VL - 117
IS - 1
CY - United Kingdom
N2 - To examine the possibility of interspecies transmission and genetic reassortment of influenza viruses on farms in Southern China, we surveyed 20 farm families living outside the city of Nanchang who raised pigs and ducks in their homes. Weekly interviews of family members and virus isolation studies of throat swabs and faecal samples, collected from September 1992 to September 1993, established the seasonal pattern of respiratory tract infections in these families and identified 11 influenza viruses (6 in humans and 5 in ducks). Most of the human isolates were type A of H3N2 subtype. Serologic studies of farm pigs indicated infection by

the same human viruses circulating in family members, but there was no evidence that either swine or avian viruses had been transmitted to pigs. Eight of 156 human serum samples inhibited the neuraminidase activity of two of the duck isolates, raising the possibility of interspecies transmission of these avian viruses. Genotype analysis of duck and human isolates provided no evidence for reassortment. Our findings support the concept that intermingling of humans, pigs and ducks on Chinese farms is favourable to the generation of new, potentially hazardous strains of influenza virus
SN - 0950-2688
AD - (Shu, Zhou, Sharp, Webster) Department of Virology, St. Jude Children's Res. Hospital, 332 North Lauderdale, Memphis, TN 38101, United States (Zhou, He, Zhang, Zou) Department of Microbiology, Jiangxi Medical College, 162 Ba Yi Road, Nanchang, Jiangxi, China (Sharp) Department of Hematology, St. Jude Children's Res. Hospital, 332 North Lauderdale, Memphis, TN 38101, United States
ER -

TY - JOUR
ID - 3959
T1 - The differences between male and female drug users: Community samples of heroin and cocaine users compared
A1 - Powis,B.
A1 - Griffiths,P.
A1 - Gossop,M.
A1 - Strang,J.
Y1 - 1996///
N1 - 1996247321
English
Journal: Article
KW - EMBASE
KW - Adult
KW - article
KW - Child
KW - criminal behavior
KW - Demography
KW - drug dependence/ep [Epidemiology]
KW - drug use
KW - Female
KW - heroin dependence/ep [Epidemiology]
KW - Human
KW - intravenous drug abuse
KW - major clinical study
KW - Male
KW - priority journal
KW - sex difference
KW - Sexual Behavior
KW - cocaine
KW - diamorphine
KW - illicit drug
RP - NOT IN FILE
SP - 529
EP - 543
JF - Substance Use and Misuse
VL - 31
IS - 5
CY - United States
N2 - Although gender-related issues are often cited as playing an important part in determining patterns of illicit drug use, little is known about the differences between male and female drug users outside treatment settings. In the present study, 558 heroin and cocaine users recruited from a range of community settings were interviewed by Privileged Access Interviewers. The women were found to be younger than the men. Differences existed in their drug use; women used smaller amounts, for a shorter duration, and were less likely to inject than

their male counterparts. No differences existed between treatment contact for the heroin users, but differences were found among the cocaine-using sample, with men being more likely to have contacted a treatment agency. Men were financing themselves through more criminal activities than women. Drug-using sexual partners were found to be an important influence over women's drug use, with most female injectors having been given their first injection by a male sexual partner. Structural differences in patterns of drug use found among female drug users and the influence of male sexual partners are likely to play an important role in determining appropriate treatment options for women drug users
SN - 1082-6084
AD - (Powis, Griffiths, Gossop, Strang) National Addiction Centre, Addiction Sciences Building, 4 Windsor Walk, Denmark Hill, London SE5 8AF, United Kingdom
ER -

TY - JOUR
ID - 3961
T1 - What can family doctors contribute to health promotion in schools?
A1 - Wiesemann,A.H.
A1 - Fay,K.
A1 - Scheidt,R.
A1 - Scheuermann,W.
Y1 - 1996///
N1 - 1996158639
German
Journal: Article
KW - EMBASE
KW - article
KW - Child
KW - general practitioner
KW - Germany
KW - Health Promotion
KW - Human
KW - school
RP - NOT IN FILE
SP - 79
EP - 89
JF - Sozial- und Praventivmedizin
JA - Soz.Praventivmed.
VL - 41
IS - 2
CY - Switzerland
N2 - Although several German schools have developed and tested concepts of a goal-directed health education, a stable and continuous implementation is missing. In this field free-practising physicians can support public health efforts. In the area Bruchsal/Ostringen it is demonstrated, that especially those primary care physicians, who work according to the 'Three-Level Concept of Free-Practising Physicians', can successfully use their geographical and social nearness to schools in their neighbourhood for a long-lasting implementation of recurring health-related activities. Within the years 1389 students in schools, further ca. 500 outside schools, were directly included in interactive learning in the framework of preventive measures, e.g. during school health days, 10 physicians (4 of them continuously) cooperated in these activities, which had been planned in close collaboration with teachers and parents, considering the different age groups. From the very beginning - although sometimes limited in scale - each activity was evaluated. Pupils in the age group 10-12 years generally showed the highest interest in health-related topics. Two years after specific school activities 28-40% of the student's family members reportedly cared more intensively for a health-related diet, 17-26% were physically more active. Promising are the results with respect to smoking: one fifth of the smoking students intended to quit as a consequence of the health activities in their schools
SN - 0303-8408
AD - (Wiesemann) Arzt fur Allgemeinmedizin, Ostringen, Germany (Fay) Rektor Grund- Hauptschule Odenheim (Scheidt, Scheuermann) Abteilung Klinische Sozialmedizin, Klin. der Univ. Heidelberg (Wiesemann) Kirchstrasse 44, D-76684 Ostringen/Odenheim, Germany

ER -

TY - JOUR
ID - 3962
T1 - Ethnic, socioeconomic, and sex differences in physical activity among adolescents
A1 - Sallis,J.F.
A1 - Zakarian,J.M.
A1 - Hovell,M.F.
A1 - Hofstetter,C.R.
Y1 - 1996///
N1 - 1996130383
English
Journal: Article
KW - EMBASE
KW - adolescence
KW - Adolescent
KW - Adult
KW - article
KW - ethnic difference
KW - Female
KW - Health Promotion
KW - Human
KW - human experiment
KW - lowest income group
KW - Male
KW - normal human
KW - Physical Activity
KW - Physical Education
KW - priority journal
KW - school child
KW - sex difference
KW - social status
KW - socioeconomics
KW - United States
RP - NOT IN FILE
SP - 125
EP - 134
JF - Journal of Clinical Epidemiology
VL - 49
IS - 2
CY - United States
N2 - Regular physical activity is recommended for health maintenance in adolescence, but basic descriptive epidemiological data are lacking for this age group. The present study examined socioeconomic status (SES), ethnic, and sex differences in physical activity in a multiethnic sample of 1871 high school students in San Diego, California. Surveys were completed in required classes by a diverse sample of students from a low income school district and by Angle students from a nearby more affluent district. Boys reported more vigorous exercise outside of school and during school physical education, as well as more participation in sports teams, but girls reported taking more activity related lessons and classes. High-SES students had more frequent physical education classes, spent more time in vigorous exercise in those classes, and participated in more activity lessons outside of school. There were few ethnic differences on summary physical activity or physical education variables. There were no SES differences, but there were ethnic differences on 5 of 22 specific activities. Demographic differences in 25 psychological, social, and environmental variables that may influence physical activity were also examined. Differences between boys and girls were found on several variables, which may explain some of the boys' higher activity levels, Ethnic and/or SES differences were found in 10 of 25 potential correlates of physical activity. These data may be used to identify specific activities that may be preferred by subgroups of adolescents and specific mediating variables that can be targeted in physical activity promotion programs for adolescents

SN - 0895-4356
AD - (Sallis, Zakarian, Hovell, Hofstetter) Depts. of Psychol. and Polit. Sci., Graduate School of Public Health, San Diego State University, San Diego, CA, United States (Sallis) Department of Psychology, San Diego State University, 6363 Alvarado Court, #103, San Diego, CA 92120, United States
ER -

TY - JOUR
ID - 3963
T1 - A test of the activity deficit hypothesis with children with movement difficulties
A1 - Bouffard,M.
A1 - Watkinson,E.J.
A1 - Thompson,L.P.
A1 - Causgrove Dunn,J.L.
A1 - Romanow,S.K.E.
Y1 - 1996///
N1 - 1996025419
English
Journal: Article
KW - EMBASE
KW - article
KW - Child
KW - clinical article
KW - controlled study
KW - Female
KW - Human
KW - hypothesis
KW - Male
KW - motor dysfunction/et [Etiology]
KW - normal human
KW - Physical Activity
KW - Physical Education
KW - play
KW - school
KW - Social Interaction
RP - NOT IN FILE
SP - 61
EP - 73
JF - Adapted Physical Activity Quarterly
JA - Adapt Phys Act Q
VL - 13
IS - 1
CY - United States
N2 - An activity deficit hypothesis was posited that children with movement difficulties are less physically active during recess than age- and gender-matched controls without movement difficulties. Criteria used in identifying children with movement difficulties were (a) a score of at least 4 on the Test of Motor Impairment, (b) regular physical education student, and (c) age 80 to 109 months. An observational study was conducted over a 2-month period in recess settings with 52 subjects. Findings revealed that during recess time, children with movement difficulties were vigorously active less often, played less often with large playground equipment, were not observable for significantly more time, and spent less time in positive social interactions with others of their own gender. Accordingly, it was concluded that the data support the activity deficit hypothesis
SN - 0736-5829
AD - (Bouffard, Watkinson, Thompson, Romanow) Fac. of Phys. Educ. and Recreation, E-407 Van Vliet Centre, University of Alberta, Edmonton, Alta. T6G 2H9, Canada (Causgrove Dunn) Faculty of Physical Activity Studies, University of Regina, Regina, Sask. S4S OA2, Canada
ER -

TY - JOUR
ID - 3964
T1 - Burkholderia (pseudomonas) cepacia epidemiology in a cystic fibrosis population: A genome finger-printing study
A1 - Cazzola,G.
A1 - Amalfitano,G.
A1 - Tonolli,E.
A1 - Perazzoli,C.
A1 - Piacentini,I.
A1 - Mastella,G.
Y1 - 1996///
N1 - 1996171892
English
Journal: Article
KW - EMBASE
KW - Adolescent
KW - Adult
KW - article
KW - bacterial colonization
KW - bacterial infection
KW - Burkholderia cepacia
KW - Child
KW - cystic fibrosis
KW - Disease Transmission
KW - Female
KW - Genome
KW - Human
KW - major clinical study
KW - Male
KW - priority journal
KW - Prognosis
RP - NOT IN FILE
SP - 554
EP - 557
JF - Acta Paediatrica, International Journal of Paediatrics
VL - 85
IS - 5
CY - Norway
N2 - In patients with cystic fibrosis, infection with Pseudomonas cepacia is associated with poor outcomes. However; the epidemiology of Burkholderia cepacia is still unclear. The aim of this study was to investigate the epidemiology of Burkholderia (Pseudomonas)) cepacia colonization among cystic fibrosis patients attending the Verona CF Centre, a large specialized unit to which patients from different parts of Italy are admitted. We used a genome finger-printing system to analyse the nucleotidic structure of B. cepacia isolates from 60 colonized cystic fibrosis patients. Forty-two different finger-printing patterns were identified. Thirty-two patients were colonized by individual B. cepacia strains (53.3%). The remaining 28 subjects were divided into 10 different subgroups, each exhibiting a distinct strain of B. cepacia (46.7%). Nevertheless, direct, person-to-person transmission was evident in only 10 cases (16.7%). The stability, up to 12 months, of the B. cepacia colonizing strain was documented in 36 individuals. Consistent with other reports, risk of B. cepacia transmission between cystic fibrosis patients through intimacy or nosocomial contact was found in our study. However, besides low contagiousness, our data suggest that the environmental reservoir of B. cepacia outside the hospital seems to play an important role in B. cepacia infection of our cystic fibrosis population
SN - 0803-5253
AD - (Cazzola, Mastella) Cystic Fibrosis Centre, Ospedale Civile Maggiore, Verona, Italy (Amalfitano, Tonolli, Perazzoli, Piacentini) Department of Microbiology, Ospedale Civile Maggiore, Verona, Italy (Cazzola) Cystic Fibrosis Centre, Ospedale Civile Maggiore, Piazzale Stefani 1, 37126 Verona, Italy
ER -

TY - JOUR
ID - 3965
T1 - Mercury exposure in a residential community--Florida, 1994
Y1 - 1994///
N1 - 7770028
English
Journal: Article
KW - EMBASE
KW - Adult
KW - article
KW - Child
KW - Environmental Exposure
KW - Environmental Monitoring
KW - Housing
KW - Human
KW - mercurialism/di [Diagnosis]
KW - mercurialism/ep [Epidemiology]
KW - school
KW - United States/ep [Epidemiology]
KW - Mercury
RP - NOT IN FILE
SP - 436
EP - 437, 443
JF - MMWR
VL - Morbidity and mortality weekly report. 44
IS - 23
CY - United States
N2 - Residential exposure to elemental mercury typically involves small amounts (e.g., the approximately 0.3 mL in a thermometer). During August 1994, five children residing in a neighborhood in Palm Beach County, Florida, found 5 pints of elemental mercury in an abandoned van. During the ensuing 25 days, the children shared and played with the mercury outdoors, inside homes, and at local schools. On August 25, 1994, a parent notified local police and fire authorities that her children had brought mercury into the home. On the same day, 50 homes were immediately vacated and an assessment of environmental and health impacts was initiated by the State of Florida Department of Environmental Protection, the Health and Rehabilitative Services of the Palm Beach County Public Health Unit, and the U.S. Environmental Protection Agency. This report summarizes the investigation of this incident.(ABSTRACT TRUNCATED AT 250 WORDS)
SN - 0149-2195
ER -

TY - JOUR
ID - 3966
T1 - Treating early mental health and post-traumatic symptoms of children in the Hanshin-Awaji earthquake
A1 - Nagao,K.
A1 - Okuyama,M.
A1 - Miyamoto,S.
A1 - Haba,T.
Y1 - 1995///
N1 - 1996008864
English
Journal: Article
KW - EMBASE
KW - article
KW - Child
KW - child health care
KW - disaster
KW - earthquake
KW - Human

KW - Japan
KW - mental health care
KW - posttraumatic stress disorder/co [Complication]
KW - posttraumatic stress disorder/di [Diagnosis]
KW - priority journal
RP - NOT IN FILE
SP - 745
EP - 754
JF - Acta Paediatrica Japonica (Overseas Edition)
VL - 37
IS - 6
CY - Japan
N2 - In this article we report on the support facilities for child mental health after the earthquake in Kobe. We believe that the most important function is the ability to cope with the confused situation on-the-spot as there were no means of transport and facilities for medical treatment had been paralyzed. Establishing contact with people outside the stricken area is of great help immediately following a disaster. After the earthquake many children were psychologically confused and people were therefore concerned with mental health care, especially for their children. However, there were only a few cases of patients seeing their doctors in medical centers. They needed information booklets or case reports to refer to. Five months after the disaster symptoms such as acute uneasiness or terror seem to be few and severely ill patients have started to recover. There are few case reports on children who have had difficulties in adapting to educational organization such as day nurseries and elementary schools. However, recently there has been an increase in the number of bone fractures possibly due to decline in resistance. Means of dealing with child mental health are now being examined because some children are still suffering post-traumatic symptoms. In this report we analyze the rapid recognition of children's mental health problems at home, in the school and in society and examine the importance of close mother-child, family and relative relationships
SN - 0374-5600
AD - (Nagao, Okuyama, Miyamoto, Haba) Sakai Child Rehabilitation Center, Department of Psychiatry, 5-1 Shiroyamadai, Sakai, Japan
ER -

TY - JOUR
ID - 3967
T1 - Development of grating acuity in children treated for unilateral or bilateral congenital cataract
A1 - Lewis,T.L.
A1 - Maurer,D.
A1 - Brent,H.P.
Y1 - 1995///
N1 - 1995266241
English
Journal: Article
KW - EMBASE
KW - article
KW - clinical protocol
KW - congenital cataract/cn [Congenital Disorder]
KW - controlled study
KW - Human
KW - Infant
KW - major clinical study
KW - optokinetic nystagmus
KW - preschool child
KW - priority journal
KW - Visual Acuity
RP - NOT IN FILE
SP - 2080
EP - 2095
JF - Investigative Ophthalmology and Visual Science

VL - 36
IS - 10
CY - United States
N2 - Purpose. To study the development of grating acuity in children treated for dense congenital unilateral or bilateral cataract and to examine how variations in treatment affect grating acuity during early childhood. Methods. The author's used optokinetic nystagmus (OKN), preferential looking (PL), or both to measure the grating acidity of children treated for congenital cataract in line eye (n = 63) or both eyes (n = 77) whenever possible from the time of treatment until 3 years of age. At each age, the authors compared patients' monocular acuity to that children with no history of eye disorders. Results. The OKN acuity of treated eyes did not improve with age and was abnormal by 12 months of age. In contrast, PL activity improved with age, and acuity of most treated eyes was not outside normal limits until 24 to 30 months of age. Nonetheless, at 12 months and at 3 years of age, PL acuity correlated significantly with age at treatment in children who had bilateral cataract. In children who had unilateral cataract, PL acuity correlated significantly with the number of hours per day the good eye had been patched since treatment. Children whose good eye was patched fewer than 3 hours per day did significantly worse than children treated at a comparable age for bilateral congenital cataract. However, children whose good eye was patched at least 3 hours per day had PL acidities similar to those of children treated at a comparable age for bilateral congenital cataract. Conclusions. Children treated for congenital cataract show deficits in grating acuity, with the deficit apparent earlier in OKN acuity than in PL acuity. At least by 1 year of age, visual development has begun to be influenced by the age at treatment and, in children treated for unilateral cataract, by patching of the good eye
SN - 0146-0404
AD - (Lewis, Maurer, Brent) Department of Psychology, McMaster University, Hamilton, Ont. L8S 4K1, Canada
ER -

TY - JOUR
ID - 3968
T1 - Correlation of exercise-induced bronchoconstriction to PC20 and maximal airway narrowing on the dose-response curve to methacholine
A1 - Lim,H.S.
A1 - Yoon,K.A.
A1 - Koh,Y.Y.
Y1 - 1995///
N1 - 1995232859
Korean
Journal: Article
KW - EMBASE
KW - article
KW - Asthma
KW - bronchospasm
KW - Child
KW - clinical trial
KW - controlled clinical trial
KW - controlled study
KW - dose response
KW - Exercise
KW - Female
KW - Human
KW - major clinical study
KW - Male
KW - provocation test
KW - methacholine
RP - NOT IN FILE
SP - 165
EP - 174
JF - Tuberculosis and Respiratory Diseases
VL - 42

IS - 2
CY - South Korea
N2 - Background: Exercise is one of the most common precipitants of acute asthma encountered in clinical practice. The development of airflow limitation that occurs several minutes after vigorous exercise, i.g. exercise-induced bronchoconstriction (EIB), has been shown to be closely correlated with the nonspecific bronchial hyperresponsiveness, which is the hallmark of bronchial asthma. All previous reports that assessed the correlation of EIB to nonspecific bronchial hyperresponsiveness have focused on airway sensitivity (PC₂₀) to inhaled bronchoconstrictor such as methacholine or histamine. However, maximal airay narrowing (MAN), reflecting the extent to which the airways can narrow, when being exposed to high dose of inhaled stimuli, has not been studied in relation to the degree of EIB. Methods: Fifty-six children with mild asthma (41 boys and 15 girls), aged 6 to 15 years (mean + SD, 9.9 + 2.5 years) completed this study. Subjects attended the laboratory on two consecutive days. Each subject performed the high-dose methacholine inhalation test at 4 p.m. on the first day. The dose-response curves were characterized by their position (PC₂₀) and MAN, which was defined as maximal response plateau (MRP: when two or three data points of the highest concentrations fell within a 5% response range) or the last of the data points (when a plateau could not be measured). On the next day, exercise challenge, free running outdoors for ten minutes, was performed at 9 a.m. FEV₁ was measured at graduated intervals, 3 to 10 minutes apart, until 60 minutes after exercise. Response (the maximal FEV₁ from the pre-exercise value) was classified arbitrarily into three groups; no response ((-) EIB: FEV₁ < 10%), equivocal response ((+)EIB: 10% < ~FEV₁ < 20%) and definite response ((+) EIB: FEV₁ > 20%). Results. 1) When geometric mean PC₂₀ of the three groups were compared, PC₂₀ of (+)EIB group was significantly lower than that of (-)EIB group. 2) There was a close correlation between PC₂₀ and the severity of EIB in the whole group (r = -0.568, p < 0.01). 3) Of the total 56 subjects, MRP could be measured in 36 subjects, and the MRP of these subjects correlated fairly with the severity of EIB (r = 0.355, p < 0.05) 4) The MAN of (+)EIB group was significantly higher than that of (-)EIB group (p < 0.01). 5) The MAN correlated well with the severity of EIB in the whole group (r = 0.546, p < 0.01). Conclusion: The degree of MAN as well as bronchial sensitivity (PC₂₀) to methacholine is correlated well with the severity of EIB. The results suggest that the two main components of airway hyperresponsiveness may be equally important determinants of exercise reactivity, although the mechanism may be different from each other. The present study also provides further evidence that EIB is a manifestation of the increased airway reactivity characteristic of bronchial asthma
SN - 0378-0066
AD - (Lim, Yoon, Koh) Department of Pediatrics, Seoul National University, College of Medicine, Seoul, South Korea
ER -

TY - JOUR
ID - 3969
T1 - Children who are shot: A 30-year experience
A1 - Laraque,D.
A1 - Barlow,B.
A1 - Durkin,M.
A1 - Howell,J.
A1 - Cladis,F.
A1 - Friedman,D.
A1 - DiScala,C.
A1 - Ivatury,R.
A1 - Stahl,W.
A1 - Hirsch,M.
A1 - Solenberger,R.
Y1 - 1995///
N1 - 1995232407
English
Journal: Conference Paper
KW - EMBASE
KW - Child
KW - childhood injury/ep [Epidemiology]

KW - childhood injury/pc [Prevention]
KW - conference paper
KW - Drug Abuse
KW - gunshot injury/ep [Epidemiology]
KW - gunshot injury/pc [Prevention]
KW - Human
KW - law
KW - major clinical study
KW - Mortality
KW - priority journal
KW - school
KW - United States
RP - NOT IN FILE
SP - 1072
EP - 1076
JF - Journal of Pediatric Surgery
JA - J Pediatr Surg
VL - 30
IS - 7
CY - United States
N2 - Three data sets describe the pattern of gunshot injuries to children from 1960 to 1993: The Harlem Hospital pediatric trauma registry (HHPTR), the northern Manhattan injury surveillance system (NMISS) a population-based study, and the National Pediatric Trauma Registry (NPTR). A small case- control study compares the characteristics of injured children with a control group. Before 1970 gunshot injuries to Harlem children were rare. In 1971 an initial rise in pediatric gunshot admissions occurred, and by 1988 pediatric gunshot injuries at Harlem Hospital had peaked at 33. Population-based data through NMISS showed that the gunshot rate for Central Harlem children 10 to 16 years of age rose from 64.6 per 100,000 in 1986 to 267.6 per 100,000 in 1987, a 400% increase. The case fatality for children admitted to Harlem Hospital (1960 to 1993) was 3%, usually because of brain injury, but the majority of deaths occurred before hospitalization. During the same period, felony drug arrests in Harlem increased by 163%. The neighboring South Bronx experienced the same increase in gunshot wound admissions and felony arrests from 1986 to 1993. The NPTR showed a similar injury pattern for other communities in the United States. In a case-control analysis, Harlem adolescents who had sustained gunshot wounds were more likely to have dropped out of school, to have lived in a household without a biological parent, to have experienced parental death, and to have known of a relative or friend who had been shot than community adolescents treated for other medical or surgical problems. Since 1990, the Harlem Injury Prevention Program formed a coalition of school and community organizations joined by the District Attorney's Office in collaboration with the Tactical Narcotic Team (to eliminate drug selling from the schools and playgrounds), to provide safe, supervised activities for children. Data from 1990 to 1992 show a moderate decline in the incidence of gunshot wounds to children. Gun control legislation in conjunction with the community violence prevention activities are needed to curb the epidemic of gunshot injuries
SN - 0022-3468
AD - (Laraque, Barlow, Durkin, Howell, Cladis, Friedman, DiScala, Ivatury, Stahl, Hirsch, Solenberger) Department of Pediatrics, Columbia University, Harlem Hospital Medical Center, 506 Lenox Ave, New York, NY 10037, United States
ER -

TY - JOUR
ID - 3970
T1 - Family history, participation in sports or tv viewing, which is the better predictor of hypercholesterolemia in children?
A1 - Gonzalez,Requejo A.
A1 - Sanchez,Bayle M.
A1 - Arnaiz,Rodriguez P.
A1 - Asensio,Anton J.
A1 - Ruiz-Jarabo,Quemada C.
A1 - Baeza,Minguez P.
A1 - Vila,Dupla S.

Y1 - 1995///
N1 - 1995201456
Spanish
Journal: Article
KW - EMBASE
KW - Adolescent
KW - article
KW - Exercise
KW - family history
KW - Human
KW - hypercholesterolemia
KW - normal human
KW - Prediction
KW - school child
KW - sport
KW - Television
KW - low density lipoprotein cholesterol/ec [Endogenous Compound]
RP - NOT IN FILE
SP - 337
EP - 340
JF - Anales Espanoles de Pediatria
VL - 42
IS - 5
CY - Spain
N2 - Recent studies have reported that excessive TV viewing is associated with an increased probability of having cholesterol values of 200 mg/dl or higher. We have studied the relationship between excessive TV viewing and high levels of total and LDL-cholesterol. In addition, we have evaluated excessive TV viewing, lack of the practice of sports outside of school, and positive family history of hypercholesterolemia, heart attack, stroke, hypertension and diabetes mellitus as tests to detect children with hypercholesterolemia. For this purpose, 2224 children and adolescents between 6 and 18 years of age were studied. We have found that when compared to children who spent less than 14 hours per week watching TV, children aged 6 to 12 years and adolescent males aged 16 to 18 who spent more than 14 hours viewing the TV showed significantly higher mean levels of total cholesterol. Adolescent males from 16 to 18 years old also showed significantly higher levels of LDL-cholesterol. Excessive TV viewing was found to be strongly associated with the non-practice of sports outside of school. The percentage of subjects with a cholesterol value of 200 mg/dl or higher was found to be significantly higher in those children who watched TV for more than 14 hours a week ($p < 0.05$), did not practice sports ($p < 0.01$) and had a positive parental history ($p < 0.001$). A positive family history was found to be the strongest predictor of hypercholesterolemia. Excessive TV viewing, as well as the non-practice of sports, although associated with an increased fisk of hypercholesterolemia, was not superior to family history as a test to detect children at risk
SN - 0302-4342
AD - (Gonzalez Requejo, Sanchez Bayle, Arnaiz Rodriguez, Asensio Anton, Ruiz-Jarabo Quemada, Baeza Minguez, Vila Dupla) Hospital del Nino Jesus, Avda. Menendez Pelayo, 65, 28009 Madrid, Spain
ER -

TY - JOUR
ID - 3971
T1 - Pharmacokinetic-based ticarcillin/clavulanic acid dose recommendations for infants and children
A1 - Reed,M.D.
A1 - Yamashita,T.S.
A1 - Blumer,J.L.
Y1 - 1995///
N1 - 1995212397
English
Journal: Review
KW - EMBASE
KW - antibacterial activity

KW - antibiotic resistance
KW - Body Weight
KW - Child
KW - dose calculation
KW - drug efficacy
KW - drug safety
KW - enzyme inhibition
KW - Human
KW - Infection/dt [Drug Therapy]
KW - intravenous drug administration
KW - kidney clearance
KW - practice guideline
KW - review
KW - clavulanic acid/ad [Drug Administration]
KW - clavulanic acid/cb [Drug Combination]
KW - clavulanic acid/do [Drug Dose]
KW - clavulanic acid/dt [Drug Therapy]
KW - clavulanic acid/pk [Pharmacokinetics]
KW - ticarcillin/ad [Drug Administration]
KW - ticarcillin/cb [Drug Combination]
KW - ticarcillin/do [Drug Dose]
KW - ticarcillin/dt [Drug Therapy]
KW - ticarcillin/pk [Pharmacokinetics]
RP - NOT IN FILE
SP - 658
EP - 665
JF - Journal of Clinical Pharmacology
VL - 35
IS - 7
CY - United States
N2 - The pharmacokinetic characteristics of ticarcillin and clavulanic acid were determined after the first dose (n = 22) and again under steady-state conditions (n = 16) in a group of infants and children. Study subjects ranged in age from 1 month to 9.3 years; all but 3 study patients were 6 months of age or older. Each patient received 50 mg of ticarcillin and 1.7 mg of clavulanic acid (30:1 ratio) per kg of body weight given intravenously every 4 hours. Elimination half-life, steady-state volume of distribution, and body clearance averaged 1.1 hours, 0.22 L/kg, and 2.7 mL/min/kg, respectively, for ticarcillin, and 0.9 hours, 0.4 L/kg, and 6.2 mL/min/kg, respectively, for clavulanic acid. A total of 71% of the ticarcillin and 50% of the clavulanic acid dose were excreted unchanged in the urine over the 4-hour sampling period. Corresponding renal clearances averaged 2.1 and 3.2 mL/min/kg for ticarcillin and clavulanic acid, respectively. No differences were observed between first dose and steady-state evaluations in the pharmacokinetic behavior of either agent. In contrast, the pharmacokinetic behavior of clavulanic acid was significantly different from that observed for ticarcillin. These pharmacokinetic data combined with known in vitro susceptibilities of important clinical pathogens support a dose of 80 mg of ticarcillin and 2.7 mg/kg clavulanic acid per kg body weight given as a fixed dose combination every 8 hours for the treatment of most systemic infections that occur outside the central nervous system
SN - 0091-2700
AD - (Reed, Yamashita, Blumer) Pedia. Pharmacol./Critical Care Div., Rainbow Babies and Childrens Hosp., 11100 Euclid Avenue, Cleveland, OH 44106-5000, United States
ER -

TY - JOUR
ID - 3972
T1 - Chronic invasive monitoring for identifying seizure foci in children
A1 - Adelson,P.D.
A1 - O'Rourke,D.K.
A1 - Leland,Albright A.
Y1 - 1995///

N1 - 1995202061
English
Journal: Review
KW - EMBASE
KW - Child
KW - computer assisted tomography
KW - electrocorticography
KW - Epilepsy/di [Diagnosis]
KW - Epilepsy/su [Surgery]
KW - Human
KW - nuclear magnetic resonance
KW - patient monitoring
KW - priority journal
KW - review
RP - NOT IN FILE
SP - 491
EP - 504
JF - Neurosurgery Clinics of North America
VL - 6
IS - 3
CY - United States
N2 - In both children and adults, it has been well established that the precise localization of a seizure focus allows for the best possible resection and outcome. Long-term invasive monitoring with DE has been the most widely used modality to evaluate patients with intractable seizures of temporal lobe origin. The study of epilepsy in children, however, requires assessment of the cause of the seizures with a resultant decision regarding the optimal modality of study for the particular problem. Complex partial seizures of medial temporal lobe origin without clear concordance, or lateralizing information are best studied using DE. With MR imaging anatomic localization, stereotactic insertion of DE can be performed accurately and relatively safely. The information obtained permits lateralization and focus localization if the lesion is within the medial structures. The ability of DE to study epilepsy outside of the medial temporal lobe, however, is suboptimal. Many centers have used surface electrodes to map temporal lobe foci successfully. SE and SGE have not been widely used in children. Subdural electrode arrays, however, are superior to DE in extratemporal epilepsy because the cortical surface contacts provide the best recordings of epileptiform activity from a wide range of extratemporal regions. The limitations of intraoperative ECoG in the awake child are well understood. It is rare that a child can undergo surgery under local anesthesia and cooperate to the extent necessary for definitive mapping of the seizure focus and eloquent areas. Thus, invasive long-term monitoring would seem ideal in the epileptic pediatric patient because it allows for a more relaxed pace in which to record events, and because of the indwelling nature of these electrodes, it is possible for the investigator to map eloquent areas of the brain before resection. This type of evaluation completely and accurately maps interictal and ictal activity, and through cortical stimulation or mapping of somatosensory areas by evoked potentials, eloquent areas of the brain, such as motor and speech regions, can be identified. This method is limited to older children because the young child often has inadequate cortical development to localize these areas definitively. One should note that in young children (under the age of 4 years), the absence of a response to standard cortical stimulation does not indicate nonfunctional cortex. Long-term intractable seizures and significant antiepileptic drug requirements may contribute to developmental and psychosocial deficiencies in the epileptic patient. Through early localization and surgical control of seizures, it is hoped that optimum development of behavior and intellect will not be impeded by repeated seizure activity or drug toxicity. Numerous reports have postulated that early surgical intervention that eliminates intractable seizures would result in an improvement in behavioral outcome. This hypothesis has been confirmed by others in patients who underwent not only resections for temporal lobe seizure foci, but also extratemporal resections. The authors, too, believe that through early evaluation, decisions regarding surgical intervention can be made for the relief of seizures. This early intervention may also alter the drug requirements and likely allow for a better developmental outcome in the pediatric epileptic population. To ensure optimal surgical resection, definitive localization through long-term invasive monitoring can be obtained in the young child with minimal morbidity. Noninvasive evaluation aids in the assessment of the proper monitoring modality for a particular patient. The authors have found these procedures to be useful and well tolerated modalities in the pediatric age group even in children as young as 1 year of age through days to weeks of recording time. Even with the

explosion of technology and functional neuroimaging, invasive monitoring can still aid in the selection of additional children as operative candidates for seizure surgery
SN - 1042-3680
AD - (Adelson, O'Rourke, Leland Albright) Department of Neurosurgery, Children's Hospital, 3705 Fifth Ave., Pittsburgh, pA 15213, United States
ER -

TY - JOUR
ID - 3973
T1 - Incidence of acute symptomatic toxoplasma retinochoroiditis in South London according to country of birth
A1 - Gilbert,R.E.
A1 - Stanford,M.R.
A1 - Jackson,H.
A1 - Holliman,R.E.
A1 - Sanders,M.D.
Y1 - 1995///
N1 - 1995126340
English
Journal: Article
KW - EMBASE
KW - Adolescent
KW - Adult
KW - Africa
KW - article
KW - Child
KW - chorioretinitis/di [Diagnosis]
KW - chorioretinitis/ep [Epidemiology]
KW - Chorioretinitis/et [Etiology]
KW - clinical article
KW - congenital toxoplasmosis/di [Diagnosis]
KW - congenital toxoplasmosis/ep [Epidemiology]
KW - congenital toxoplasmosis/et [Etiology]
KW - diagnostic value
KW - eye inflammation/co [Complication]
KW - Female
KW - Human
KW - Male
KW - prenatal screening
KW - priority journal
KW - retinitis/co [Complication]
KW - scar/co [Complication]
KW - Toxoplasmosis/di [Diagnosis]
KW - Toxoplasmosis/ep [Epidemiology]
KW - toxoplasmosis/et [Etiology]
KW - United Kingdom
KW - immunoglobulin G antibody/ec [Endogenous Compound]
RP - NOT IN FILE
SP - 1037
EP - 1022
JF - British Medical Journal
JA - Br Med J
VL - 310
IS - 6986
CY - United Kingdom
N2 - Objective - To determine the incidence of acute symptomatic toxoplasma retinochoroiditis presenting to ophthalmologists for patients born in Britain and elsewhere. Design - Population based, cross sectional study.

Setting - 11 districts in south Greater London. Subjects-All patients ophthalmologists with symptoms toxoplasma retinochoroiditis in 1992-3. Main outcome measure - Intraocular inflammation in association with a retinochoroidal scar, active adjoining retinitis, and IgG serum antibodies to toxoplasma. Results - The estimated incidence of acute symptomatic retinochoroiditis for all people born in Britain was 0.4/100 000/year and for black people born in west Africa 57/100 000/year. If a mean of two symptomatic episodes per lifetime is assumed, 100 people born in Britain may be affected each year, about a fifth of the estimated 500-600 congenitally infected people born each year. Conclusions - A substantial proportion of people with acute symptomatic toxoplasma retinochoroiditis were born outside the country, and the number born in Britain was smaller than the number previously estimated to develop retinochoroidal lesions due to congenital toxoplasmosis. These findings suggest that prenatal screening for toxoplasmosis in Britain may be of limited benefit
SN - 0959-8146
AD - (Gilbert, Stanford, Jackson, Holliman, Sanders) Dept. Epidemiology and Biostatistics, Institute of Child Health, London WC1N 1EH, United Kingdom
ER -

TY - JOUR
ID - 3974
T1 - Serum antibodies against synthetic peptides from Trypanosoma cruzi antigens in Chagas' disease patients
A1 - Vergara,U.
A1 - Lorca,M.
A1 - Engstrom,A.
A1 - Veloso,C.
Y1 - 1995///
N1 - 1995097963
English
Journal: Article
KW - EMBASE
KW - Adult
KW - Aged
KW - article
KW - Chagas disease/di [Diagnosis]
KW - Child
KW - controlled study
KW - Female
KW - Human
KW - major clinical study
KW - Male
KW - serodiagnosis
KW - Trypanosoma cruzi
KW - epitope
KW - parasite antibody/ec [Endogenous Compound]
KW - synthetic peptide
RP - NOT IN FILE
SP - 1
EP - 4
JF - Inmunologia
VL - 14
IS - 1
CY - Spain
N2 - Chagas' disease is caused by infection with the obligatory intracellular parasite Trypanosoma cruzi and affects several million people in Central and South America. The parasite can be transmitted not only by blood-sucking triatomine bugs but also by transfusion with infected blood. Therefore, serological screening of blood donors is important to prevent transmission of the parasite in endemic areas. Serological screening in blood banks can be now performed by using recombinant antigens and synthetic peptides in highly sensitive laboratory methods. In previous work we used recombinant DNA clones to study the reactivity of sera from Chagas' disease patients. We found that 51% of serum samples from symptomatic and only 25% of sera from

asymptomatic patients reacted with the SAPA clone (Shed Acute Phase Antigen), suggesting that reactivity might be related to active infection and symptoms. In this work we used sera from asymptomatic and symptomatic Chagas' disease patients to evaluate their reactivity against synthetic repeat peptides from clones 1, 2, 13, 30, 36 and SAPA. No differences in reactivity against the synthetic peptides were found when 74 symptomatic and 77 asymptomatic patients were compared. Therefore, the higher reactivity against SAPA recombinant DNA clone previously observed in symptomatic patients may be due to the recognition of epitopes located outside the repeated domain or the recognition of a repeated epitope which is not present or not represented on the SAPA repeat peptide
SN - 0213-9626
AD - (Vergara, Lorca, Engstrom, Veloso) Immunobiology Laboratory, Department of Experimental Medicine, University of Chile, Casilla 33052, Correo 33 Santiago, Chile
ER -

TY - JOUR
ID - 3975
T1 - Spontaneous generation of human CD8+ TCRalphabeta+ cells derived from precursors within the double negative compartment
A1 - Fujimiya,Y.
A1 - Nakayama,M.
A1 - Shibata,T.
A1 - Abe,J.
A1 - Shimada,M.
A1 - Kohsaka,T.
Y1 - 1995///
N1 - 1995065673
English
Journal: Article
KW - EMBASE
KW - article
KW - Flow Cytometry
KW - Human
KW - human cell
KW - human tissue
KW - Lymphocyte Activation
KW - lymphocyte proliferation
KW - preschool child
KW - thymocyte
KW - CD3 antigen/ec [Endogenous Compound]
KW - CD8 antigen/ec [Endogenous Compound]
KW - interleukin 2
KW - phorbol ester
KW - T lymphocyte receptor
RP - NOT IN FILE
SP - 33
EP - 37
JF - Immunology and Cell Biology
VL - 73
IS - 1
CY - Australia
N2 - Flow cytometric analysis demonstrated that fresh human thymocytes contain only a low level of mature CD8⁺TCRalphabeta⁺ or CD8⁺ TCR⁺ cells and that they consist of ~70% double positive (DP) and ~10% double negative (DN) cells. These unfractionated thymocytes could be selectively expanded in vitro by stimulation with 12-o-tetradecanoylphorbol 13-acetate (TPA) and PHA in the presence of IL-2. The majority of the cells expanded from unfractionated thymocytes expressed CD3, TCRalphabeta and CD8 molecules after long-term culture (18 days). When highly purified DN thymocytes were expanded over a period of 18 days in the presence of DP cells, they also co-expressed CD3, TCRalphabeta and CD8 molecules on their surface. However, when purified DN thymocytes were expanded

alone, that is, in the absence of DP cells for 18 days, they expressed CD3-associated TCR, but not CD8 or TCRalphabeta. Despite the expression of measurable levels of IL-2 alpha and beta receptors, as well as a significant level of TCRalphabeta, purified DP cells failed to proliferate. These findings provide the first evidence, in humans, that the progression of precursor cells in the DN compartment to a later stage of differentiation can be induced outside the thymus and that DP cells can affect the development of TCR expression in proliferating DN thymocytes
SN - 0818-9641
AD - (Fujimiya, Nakayama, Shibata, Abe, Shimada, Kohsaka) Division of Immunology, Research Inst. Miyagi Cancer Center, 47-1, Nodayama, Natorishi, Miyagi 981-12, Japan
ER -

TY - JOUR
ID - 3976
T1 - Clinical and neurophysiologic response of myopathy and neuropathy in long- chain L-3-hydroxyacyl-CoA dehydrogenase deficiency to oral prednisone
A1 - Tein,I.
A1 - Donner,E.J.
A1 - Hale,D.E.
A1 - Murphy,E.G.
Y1 - 1995///
N1 - 1995062630
English
Journal: Article
KW - EMBASE
KW - article
KW - Case Report
KW - cell membrane
KW - Child
KW - electromyogram
KW - electron microscopy
KW - endurance
KW - enzyme activity
KW - enzyme deficiency/di [Diagnosis]
KW - Evoked Response
KW - fast atom bombardment mass spectrometry
KW - fatty acid oxidation
KW - growth retardation/si [Side Effect]
KW - Human
KW - human cell
KW - human tissue
KW - Male
KW - mitochondrial membrane
KW - muscle biopsy
KW - muscle fiber membrane
KW - myopathy/di [Diagnosis]
KW - myopathy/dt [Drug Therapy]
KW - nerve conduction
KW - neuropathy/di [Diagnosis]
KW - neuropathy/dt [Drug Therapy]
KW - oral drug administration
KW - priority journal
KW - skin fibroblast
KW - 3 hydroxyacyl coenzyme A dehydrogenase/ec [Endogenous Compound]
KW - 3 hydroxybutyric acid/ec [Endogenous Compound]
KW - acyl coenzyme A dehydrogenase/ec [Endogenous Compound]
KW - acylcarnitine/ec [Endogenous Compound]
KW - aspartate aminotransferase/ec [Endogenous Compound]

KW - carboxylic acid/ec [Endogenous Compound]
KW - carnitine/ad [Drug Administration]
KW - carnitine/dt [Drug Therapy]
KW - carnitine palmitoyltransferase/ec [Endogenous Compound]
KW - creatine kinase/ec [Endogenous Compound]
KW - dicarboxylic acid/ec [Endogenous Compound]
KW - dystrophin/ec [Endogenous Compound]
KW - enoyl coenzyme A hydratase/ec [Endogenous Compound]
KW - fatty acid/ec [Endogenous Compound]
KW - lipid/ec [Endogenous Compound]
KW - prednisone/ae [Adverse Drug Reaction]
KW - prednisone/ad [Drug Administration]
KW - prednisone/do [Drug Dose]
KW - prednisone/dt [Drug Therapy]
KW - riboflavin/ad [Drug Administration]
KW - riboflavin/do [Drug Dose]
KW - triacylglycerol/ec [Endogenous Compound]
KW - vitamin
RP - NOT IN FILE
SP - 68
EP - 76
JF - Pediatric Neurology
JA - Pediatr Neurol
VL - 12
IS - 1
CY - United States
N2 - The purpose of this study was to evaluate the clinical and neurophysiologic responses to oral prednisone therapy in a boy with enzymatically confirmed long-chain L-3-hydroxyacyl-CoA dehydrogenase deficiency in biopsied muscle and cultured skin fibroblasts. This boy presented with progressive limb girdle myopathy, recurrent myoglobinuria, peripheral sensorimotor axonopathy, and intraventricular conduction delays. Prior to prednisone therapy, at age 8 years, he exhibited marked distal weakness greater than proximal weakness with a waddling and high-steppage gait, Gowers' maneuver (10 s to rise from the floor), fatigue after 3-20 yards of walking and the ability to climb only 2 stairs. Serum levels of creatine kinase rose from 34 to 4,124 U/L following mild exertion. Nerve conduction studies revealed progressive axonopathy with secondary demyelination. Four weeks after initiation of oral prednisone (0.75 mg/kg/day) therapy, there was approximately a 100% increase in power and endurance. He was able to walk at least 100 yards before tiring, could rise from sitting on the floor in 3-4 s, and was able to climb 20 steps in 30 s. There was concurrent improvement in nerve conduction studies. Prednisone was gradually withdrawn over the next 4 months to 0.19 mg/kg/day; lower doses of 0.08 mg/kg/ day resulted in a marked deterioration in power to the prior state. Although 0.19 mg/kg/day did not maintain the peak power achieved at 0.75 mg/kg/day, it provided adequate baseline power and endurance. It is concluded that there was a significant clinical and neurophysiologic response to prednisone at a dosage >0.16 mg/kg/day. Prednisone may stabilize muscle and neuronal plasma membranes, as well as the fatty acid oxidation enzyme complex in the mitochondrial membrane
SN - 0887-8994
AD - (Tein, Donner, Hale, Murphy) Division of Neurology, Hospital for Sick Children, 555 University Avenue, Toronto, Ont. M5G 1X8, Canada
ER -

TY - JOUR
ID - 3977
T1 - Play skills of preschool children with speech and language delays
A1 - Shepherd,J.T.
A1 - Brollier,C.B.
A1 - Dandrow,R.L.
Y1 - 1994///

N1 - 1994297650
English
Journal: Article
KW - EMBASE
KW - article
KW - Attention
KW - book
KW - clinical article
KW - controlled study
KW - Cooperation
KW - Environment
KW - Exploratory Behavior
KW - Human
KW - Imagination
KW - imitation
KW - Language
KW - language disability/th [Therapy]
KW - materials management
KW - Music
KW - Play Therapy
KW - preschool child
KW - speech disorder/th [Therapy]
RP - NOT IN FILE
SP - 1
EP - 20
JF - Physical and Occupational Therapy in Pediatrics
VL - 14
IS - 2
CY - United States
N2 - The Preschool Play Scale (PPS) was used to rate the free play behaviors of 21 children with typical language development (control group) and 20 children with speech-language delays (study group) who were three and four years of age. Prior to being scored on the PPS, each child was individually observed for 30 minutes of outdoor free play and 30 minutes of indoor free play. After the observations, additional information for five categories on the PPS was obtained from a teacher observation check list to complete the scoring process. The following general research question was addressed: Will children who have speech-language delays and children with typical language development differ in play age and dimension scores on the PPS? Statistically significant differences were found in the play age, in all dimension scores, and for 14 of the 16 category scores. The control group consistently scored approximately one year higher than the children with speech-language delays. Some practical play intervention strategies were given for children with speech and language delays
SN - 0194-2638
AD - (Shepherd, Brollier, Dandrow) Department of Occupational Therapy, Virginia Commonwealth University, MCV Station, Richmond, VA 23298-0008, United States
ER -

TY - JOUR
ID - 3978
T1 - Study of vaccinal coverage after the incorporation of a vaccination programme into a health centre's activity
A1 - Buitrago,Ramirez F.
A1 - Garcia,Molina M.
A1 - Campal,Dominguez A.
A1 - Benito,Alonso E.
A1 - Reymundo,M.
A1 - Davila,M.J.
Y1 - 1994///

N1 - 1994220226
Spanish
Journal: Article
KW - EMBASE
KW - article
KW - Child
KW - clinical trial
KW - controlled clinical trial
KW - controlled study
KW - Human
KW - major clinical study
KW - Vaccination
KW - diphtheria pertussis tetanus vaccine/ct [Clinical Trial]
KW - poliomyelitis vaccine/ct [Clinical Trial]
RP - NOT IN FILE
SP - 538
EP - 541
JF - Atencion Primaria
JA - Aten.Primaria
VL - 14
IS - 1
CY - Spain
N2 - Objective. To find the variation in the vaccinal coverage of two-year old children after incorporating the vaccination programme into the Health Centre's activities. Design. An analytical observation study of cases and check-ups. Setting. Primary Care. An urban Health Centre in Badajoz. Patients. Two random samples of 150 children. The Study Group (SG) was composed of children who began their vaccinations at the Health Centre after the incorporation of the vaccination programme. The Control Group (CG) was made up of children who were over 18 months old at the start of the vaccinal programme (October 1990) and therefore received their vaccinations outside the Health Centre. Measurements and main results. 84.8% of the CG children correctly completed the vaccination programme as against 98.7% of the SG ($p < 0.01$). 99.3% of the SG children had the triple viral vaccination as against 95.2% of the CG ($p < 0.05$). 99.3% of the SG completed three DTP-Polio vaccinations as against 93.1% CG ($p < 0.01$). And 98.7% SG had four DT-Polio as against 86.9% CG ($p < 0.01$). Conclusions. The level of vaccinal coverage of the under-two population improved after the vaccination programme was incorporated into the Health Centre's activities, data which supports the usefulness and appropriateness of such a programme
SN - 0212-6567
AD - (Buitrago Ramirez, Garcia Molina, Campal Dominguez, Benito Alonso, Reymundo, Davila) Sinforiano Madronero 19, 06011 Badajoz, Spain
ER -

TY - JOUR
ID - 3979
T1 - Measurement of skin pH
A1 - Dikstein,S.
A1 - Zlotogorski,A.
Y1 - 1994///
N1 - 1994181192
English
Journal: Conference Paper
KW - EMBASE
KW - Adolescent
KW - Adult
KW - Aging
KW - conference paper
KW - controlled study
KW - Female
KW - Human

KW - major clinical study
KW - Male
KW - Measurement
KW - school child
KW - skin ph
RP - NOT IN FILE
SP - 18
EP - 20
JF - Acta Dermato-Venereologica, Supplement
IS - 185
CY - Norway
N2 - The knowledge of the skin surface pH-acid mantle is updated. The conviction that chronic alkalinization is damaging has been strengthened. Extensive skin surface pH data on the skin of non-white people are still missing. In the case of any measurement, the need for extensive infrastructural research and evaluation by non-parametric methods is emphasized. Infrastructural studies evaluate the range and change of the measured parameters for each test method according to sex, age and body area, as well as the sensitivity of the method to pharmacological influences. A detailed knowledge of the normal-representative range would make it possible to select those individuals whose measured parameter is outside the main 95% of the population; a detailed study of such individuals would be a very effective method of understanding the basic regulatory mechanism of the measured parameter
SN - 0365-8341
ER -

TY - JOUR
ID - 3980
T1 - Physeal and periphyseal injuries of the hand: Patterns of injury and results of treatment
A1 - Fischer,M.D.
A1 - McElfresh,E.C.
Y1 - 1994///
N1 - 1994157881
English
Journal: Article
KW - EMBASE
KW - Adolescent
KW - article
KW - Child
KW - epiphysis injury/su [Surgery]
KW - Female
KW - finger fracture/su [Surgery]
KW - fracture dislocation/su [Surgery]
KW - fracture treatment
KW - hand fracture/su [Surgery]
KW - hand injury/ep [Epidemiology]
KW - hand injury/su [Surgery]
KW - Human
KW - Infant
KW - interphalangeal joint
KW - joint injury/su [Surgery]
KW - major clinical study
KW - Male
KW - metacarpophalangeal joint
RP - NOT IN FILE
SP - 287
EP - 301
JF - Hand Clinics
VL - 10
IS - 2

CY - United States
N2 - To the authors' knowledge, this is the largest study assembled of finger fractures in children. Distribution of fractures according to location within the hand (see Fig. 1), location within the phalanges themselves, and the percent of epiphyseal fractures as well as the age distribution of the patients are all similar to what has previously been presented in smaller studies. Previously unreported synchronous and double epiphyseal injuries were identified. For the most part, thanks to the remodeling capacity and rapid healing of children's bone, treatment is short and complications are few. Nevertheless, as in other locations, there is a limited capacity to remodel angular deformity and no capacity to remodel rotational deformity. The clinical information in our study conflicts somewhat with previously described anatomic information about the insertion of ligaments with respect to the growth plate. Bogumill and Hankin and Janda have suggested that the ligaments insert primarily on the epiphysis in the proximal phalanx, and on the epiphysis and metaphysis in the middle and distal phalanges. Taken by itself, this information would suggest that a Salter-Harris III mechanism would be relatively more common at the MP joint, and the Salter-Harris II and IV mechanism (where the proximal fragment includes bone both proximal and distal to the growth plate at the point where the ligament is attached) would be relatively more common at the more distal levels. In fact, the opposite was true. The Salter-Harris II mechanism made up an overwhelming majority of the percentage of injuries of the MP joint, whereas the Salter-Harris III mechanism predominated at the PIP joint and was relatively common at the DIP joint as well. This would seem to suggest that either the contribution of ligament insertion distal to the growth plate of the PIP and DIP joints is not functionally important or that other factors are involved in the injury mechanism at both levels and play a role in producing these injuries. Further study will be required, possibly involving experimental fracture production to help elucidate this issue. Finally, it is critical to recognize and properly treat the four major categories of injury that constitute a small percentage of the total but a large percentage of the complications. The condylar and subcondylar fractures must be identified by obtaining a true lateral film by whatever means necessary. If undisplaced, they need to be adequately immobilized, possibly including the entire arm of a small child, and if displaced, they almost always require internal fixation. Fracture- dislocations, which are even less common and more difficult to recognize, are usually due to a crushing or pincer-type mechanism and may present with excessive and prolonged swelling, or poor mobility. The radiograph demonstrates the presence of very subtle calcific densities that represent portions of the displaced epiphysis that are trapped either inside or outside the joint. This occurs at the PIP and DIP joint level, but it has not been reported at the MP joint level. At the MP joint, a spike of metaphyseal bone can impale flexor tendons when significant displacement occurs. Treating the fracture itself is seldom a problem. Finally, fractures in and around the growth plate of the distal phalanx on the average were responsible for the longest healing times within this series. When these injuries involve a displaced nail plate, they can usually be treated by replacement of the nail under the eponychial fold, thereby stabilizing the distal phalanx and preventing the need for a K-wire with additional complications
SN - 0749-0712
AD - (Fischer, McElfresh) Orthopaedic Consultants, P.A., 7373 France Avenue South 312, Edina, MN 55435, United States
ER -

TY - JOUR
ID - 3981
T1 - Benzene and toluene in children's bedrooms
A1 - Porstmann,F.
A1 - Boke,J.
A1 - Hartwig,S.
A1 - Kaaden,R.
A1 - Rosenlehner,R.
A1 - Schupp,A.
A1 - Stiller,T.
A1 - Wichmann,H.E.
Y1 - 1994///
N1 - 1994126617
German
Journal: Article
KW - EMBASE
KW - air quality control

KW - article
KW - Child
KW - Environmental Exposure
KW - Germany
KW - Human
KW - indoor air pollution
KW - priority journal
KW - Traffic
KW - benzene/to [Drug Toxicity]
KW - toluene/to [Drug Toxicity]
RP - NOT IN FILE
SP - 147
EP - 153
JF - Staub, Reinhaltung der Luft
VL - 54
IS - 4
CY - Germany
N2 - As part of an epidemiological study in Duisburg (FRG), exposure to benzene and toluene was investigated at 524 households in 1990. Both components were measured concurrently indoors (childs bedroom), and outdoors (window of childs bedroom) by passive sampling over 14 days. In addition, detailed information on the homes and ventilation habits was available by questionnaire. Furthermore, the seasonal variation of the components was measured at three stations in ambient air, both by passive and active sampling. For both components the average indoor concentration was 2-3 times the outdoor concentration, and seasonal differences in the absolute concentrations and the indoor/outdoors ratio could be identified. The average concentrations of benzene in the childrens bedrooms are 9,5 mug/m³, and 1.8 mug/m³ outside at the windows. The corresponding values for toluene are 19.89 mug/m³ and 4.7 mug/m³. The indoor/outdoor ratio during the winter season is 3:1 compared to 2:1 during mid-summer when windows are more frequently open. Automobile traffic has a direct impact on the concentration levels. The ambient concentrations of benzene and toluene on streets with high traffic load are twice as high as compared to courtyards. This difference is also seen in the indoor measurement, although not as pronounced. Indoor sources influencing the benzene and toluene concentrations were environmental tobacco smoke and redecoration. The use of lacquer has the strongest influence, followed by solvents and glues. Summing up, the most important contributions to indoor concentrations of benzene in childrens bedrooms as investigated in this study come from automobile traffic outdoors and redecoration indoors
SN - 0039-0771
AD - (Porstmann, Boke, Hartwig, Kaaden, Rosenlehner, Schupp, Stiller, Wichmann) Bergische Universitat GHS, FG 'Arbeitssicherheit/Umweltmedizin', Postfach 100127, D-42001 Wuppertal, Germany
ER -

TY - JOUR
ID - 3984
T1 - Bone fractures from home accidents in children
A1 - Scalamandre,A.
A1 - Lambiase,D.
A1 - Zirletta,E.
A1 - Di,Paolo A.
A1 - Nigro,G.
A1 - Frediani,T.
A1 - Fondi,G.
A1 - Taggi,F.
Y1 - 1993///
N1 - 1994248104
Italian
Journal: Article
KW - EMBASE
KW - article
KW - Child

KW - childhood injury
KW - Epidemiology
KW - fracture/ep [Epidemiology]
KW - home accident
KW - Human
KW - Prevention
RP - NOT IN FILE
SP - 39
EP - 49
JF - Aggiornamento Pediatrico
VL - 44
IS - 1-2
CY - Italy
N2 - In order to obtain epidemiological data regarding both modality and possible conditions favouring bone fractures in children, useful for preventive measures, we collected all cases of traumatic lesion in children under 12 years and 6 months of age, with the exclusion of road accidents. We interviewed 93% of the children' parents; each child was visited, during two distinct periods, in the pediatric department of a large hospital. The majority of accidents happened while children were playing games or practicing non competitive sporting activities (which are potentially not dangerous) explains the difficulty of making up preventive programs. In addition, the high number of fractures happened in presence of parents or teachers seems to put in discussion the preventive efficacy of protection-survey binomius. The frequent absence of parents or adult witnesses at the accident, especially for older children, make it necessary to favour the acquisition of self control in the school age. The variability of environmental factors is frequently dangerous for the child. In our study necessary structures of common use have contributed to determine the traumatic event, confirming the complexity of making different educative programs, aiming to reducing the prevalence of that group of domestic accidents in pediatric age
SN - 0002-0958
AD - (Scalamandre, Lambiase, Zirletta, Di Paolo, Nigro, Frediani, Fondi, Taggi) Clinica Pediatrica, Universita 'Tor Vergata', Roma, Italy
ER -

TY - JOUR
ID - 3985
T1 - Volatile organic compounds in indoor air and in expired air as markers of activities
A1 - Cailleux,A.
A1 - Turcant,A.
A1 - Premel-Cabic,A.
A1 - Allain,P.
Y1 - 1993///
N1 - 1993210085
English
Journal: Article
KW - EMBASE
KW - air analysis
KW - air quality
KW - ambient air
KW - article
KW - Child
KW - expired air
KW - gas chromatography
KW - Human
KW - human tissue
KW - Mass Spectrometry
RP - NOT IN FILE
SP - 57
EP - 59
JF - Chromatographia

VL - 37
IS - 1-2
CY - Germany
N2 - Volatile organic compounds (VOC) are present in indoor air at concentrations generally higher than in outdoor air. In this study VOC concentrations in the air of two classrooms differing in insulation, and in the expired air of children in them, were investigated. Qualitative and quantitative determinations were done by gas chromatography-mass spectrometry techniques. Differences in the concentrations of VOC in indoor air and in the expired air of the children were linked to the endogeneous or exogeneous origin of the compounds, to the activities of the children and to the quality of indoor-outdoor air exchanges
SN - 0009-5893
AD - (Cailleux, Turcant, Premel-Cabic, Allain) Laboratoire de Pharmacologie, Centre Hospitalier Universitaire, 49033 Angers Cedex 01, France
ER -

TY - JOUR
ID - 3986
T1 - The use of mobility aids by children with cerebral palsy: A pilot investigation
A1 - Munnik,M.J.
A1 - Molenbroek,J.F.M.
A1 - Marinissen,A.H.
Y1 - 1993///
N1 - 1993151395
English
Journal: Article
KW - EMBASE
KW - article
KW - Cerebral Palsy
KW - clinical article
KW - controlled study
KW - equipment
KW - Female
KW - Human
KW - Male
KW - mobilization
KW - preschool child
RP - NOT IN FILE
SP - 13
EP - 19
JF - Journal of Rehabilitation Sciences
VL - 6
IS - 1
CY - Netherlands
N2 - Children up to five years of age, who are suffering from cerebral palsy, place great demands on mobility aids. This is especially so when they cannot move themselves forward by way of walking, need support for a sitting posture, cannot use their hands easily and have limited perceptions. They often have a wheelchair to be able to sit for a long time and move around indoors. Outdoors they are transported over greater distances by their parents in a push chair. A project has been set up at the Faculty of Industrial Design Engineering of the Delft University of Technology in order to develop a mobility aid, which creates new opportunities for independent activity and experiences with mobility. This paper deals the result of the first phase of this project: a programme of requirements with respect to the seating device of the mobility aid. The seat should be provided with adjustable elements in order to meet the need for support of each individual child
SN - 0923-0211
AD - (Munnik, Molenbroek, Marinissen) Delft University of Technology, Fac of Industrial Design Engineering, Dept. of Product/Systems Ergonomics, Jaffalaan 9, 2628 BX Delft, Netherlands
ER -

TY - JOUR

ID - 3987
T1 - Protective and risk factors associated with drug use among hispanic youth
A1 - Gfroerer,J.
A1 - De La,Rosa L.
Y1 - 1993///
N1 - 1993111793
English
Journal: Article
KW - EMBASE
KW - Adolescent
KW - article
KW - Attitude
KW - city
KW - cultural factor
KW - data analysis
KW - Demography
KW - Drug Abuse
KW - ethnic group
KW - Female
KW - Health Education
KW - Human
KW - Male
KW - parent
KW - Regression Analysis
KW - risk factor
KW - school child
KW - sex difference
RP - NOT IN FILE
SP - 87
EP - 107
JF - Journal of Addictive Diseases
VL - 12
IS - 2
CY - United States
N2 - Analysis of data from a nationally representative sample of Hispanic youths age 12-17 and their parents was done to investigate the impact of a number of variables on youths' drug-using behavior. The significance of youth, household, and parental characteristics were tested using measures of youth drug use as dependent variables in regression models. Parents' attitudes and use of licit and illicit drugs were found to play an important role in their children's drug use behavior. The results also provide some support for the hypothesis that Hispanic children whose parents are more acculturated into American society are at higher risk of using drugs. Youths of Mexican origin, youths living outside large metropolitan areas, and females were found to be more likely to use drugs. The results provide supportive evidence that for drug prevention education programs to be effective with Hispanic youths, they must be family orientated
SN - 1055-0887
AD - (Gfroerer, De La Rosa) Statist Anal/Population Survey Sect, National Institute on Drug Abuse, US Dept of Health and Human Services, United States
ER -

TY - JOUR
ID - 3988
T1 - The radiological impact of agricultural activities in an area of high natural radioactivity
A1 - Amaral,E.C.S.
A1 - Rochedo,E.R.R.
A1 - Paretzke,H.G.
A1 - Franca,E.P.
Y1 - 1992///

N1 - 1993157545
English
Journal: Conference Paper
KW - EMBASE
KW - Agriculture
KW - bean
KW - carrot
KW - Case Report
KW - Cattle
KW - Child
KW - conference paper
KW - corn
KW - crop
KW - environmental radioactivity
KW - Human
KW - Inhalation
KW - natural radioactivity
KW - potato
KW - poultry
KW - Rural Population
KW - sediment
KW - Soil
KW - Swine
KW - vegetable
KW - worker
KW - radioisotope
KW - radon
KW - radon daughter
KW - Water
RP - NOT IN FILE
SP - 289
EP - 292
JF - Radiation Protection Dosimetry
JA - Radiat Prot.Dosimetry
VL - 45
IS - 1-4 SUPPL.
CY - United Kingdom
N2 - The Pocos de Caldas Plateau, Brazil, with 70 radioactive anomalies is used for cattle, pigs and poultry production and for raising crops like potatoes, corn, brown beans and carrots. Data on the radioactivity concentration of natural radionuclides in air, soil, animal products, vegetables, water, sediment, external dose rates, and outdoor and indoor radon were used together with socio-economical information to estimate the radiologiocal exposure from these agricultural activities in this high natural radioactivity area for the local rural and remote populations. For the local population agricultural products from the plateau contribute only relatively little (ca. 0.14-0.24 mSv.y⁻¹) to the high radiation exposure (ca. 6-16 mSv.y⁻¹) from external exposure and inhalation of radon and thoron daughters. However, the agricultural products carry a significant annual collective dose (ca. 14 man.Sv.y⁻¹) to remote populations
SN - 0144-8420
AD - (Amaral, Rochedo, Paretzke, Franca) Inst de Radioprotecao e Dosimetria, CNEN, Barra da Tizuca, Cx P 37750, CEP 22602, Rio de Janeiro, Brazil
ER -

TY - JOUR
ID - 3989
T1 - The expectation and risk profile of football playing children. Orthopedic aspects
A1 - Betz,M.
A1 - Klimt,F.
Y1 - 1992///

N1 - 1993031442
German
Journal: Short Survey
KW - EMBASE
KW - Adolescent
KW - age
KW - Body Weight
KW - Child
KW - Human
KW - Risk Assessment
KW - school child
KW - short survey
KW - sport
KW - sport injury/pc [Prevention]
KW - Sports Medicine
RP - NOT IN FILE
SP - 169
EP - 173
JF - Schweizerische Zeitschrift fur Sportmedizin
JA - Schweiz.Z Sportmed
VL - 40
IS - 4
CY - Switzerland
N2 - Although the rate of injuries in soccer is high, this sport puts children only at moderate risk of getting injured. In younger children this risk is very low but the number and severity of injuries increases with puberty. The most common cause for injuries is a collision with another player, the most common injury is distortion of the talocrural joint. Playing soccer indoors is more dangerous than playing outdoors. The danger of accidents and injuries can be reduced by preventive measures, such as weight categories in children leagues
SN - 0036-7885
AD - (Betz, Klimt) Philipps-Universitat, Bereich Sportmedizin, Kugelgasse 10, D-3550 Marburg/Lahn, Germany
ER -

TY - JOUR
ID - 3990
T1 - Personal exposures to acid aerosols and ammonia
A1 - Suh,H.H.
A1 - Spengler,J.D.
A1 - Koutrakis,P.
Y1 - 1992///
N1 - 1993005306
English
Journal: Article
KW - EMBASE
KW - accuracy
KW - aerosol
KW - air conditioning
KW - air pollutant
KW - Air Pollution
KW - article
KW - biological monitoring
KW - Child
KW - Circadian Rhythm
KW - clinical article
KW - comparative study
KW - data analysis
KW - drug toxicity

KW - exposure
KW - home monitoring
KW - Human
KW - Measurement
KW - pH
KW - respiratory tract disease/ep [Epidemiology]
KW - Risk Assessment
KW - statistical analysis
KW - United States
KW - ammonia/to [Drug Toxicity]
KW - sulfur oxide
RP - NOT IN FILE
SP - 2507
EP - 2517
JF - Environmental Science and Technology
VL - 26
IS - 12
CY - United States
N2 - Indoor, outdoor, and personal acid aerosol monitoring was performed for 24 children living in Uniontown, PA, during summer 1990. These measurements were used to investigate the magnitude of personal acid aerosol exposures and its relationship to indoor and outdoor concentrations. Indoor, outdoor, and personal measurements were compared to outdoor measurements collected from a centrally located, stationary ambient monitoring site. Personal exposures reached a maximum of 300 nmol m⁻³ for both sulfate and aerosol strong acidity (12-h daytime measurements). Personal exposures were lower than corresponding outdoor levels and higher than indoor levels, with differences being greater for aerosol strong acidity. Air conditioning was found to be an important predictor of indoor sulfate and aerosol strong acidity, while ammonia was found to influence indoor and personal aerosol strong acidity concentrations. Time/activity weighted models of indoor and outdoor concentrations were used to predict personal sulfate and aerosol strong acidity exposures. These models predicted personal sulfate and aerosol strong acidity exposures substantially better than outdoor concentrations alone. The aerosol acidity model, however, was unable to explain all the variability in personal exposures. Research should be conducted to determine the effects of particle loss and ammonia neutralization around the human body
SN - 0013-936X
AD - (Suh, Spengler, Koutrakis) Harvard School of Public Health, 665 Huntington Avenue, Boston, MA 02115, United States
ER -

TY - JOUR
ID - 3991
T1 - Preference for outdoor play during early adolescence
A1 - Pellegrini,A.D.
Y1 - 1992///
N1 - 1992312070
English
Journal: Article
KW - EMBASE
KW - Academic Achievement
KW - adolescence
KW - Adolescent
KW - article
KW - Behavior
KW - Female
KW - Human
KW - life
KW - Male
KW - normal human
KW - physical attractiveness

KW - play
KW - school
KW - school child
KW - sex difference
KW - Social Dominance
KW - Socialization
KW - sport
RP - NOT IN FILE
SP - 241
EP - 254
JF - Journal of Adolescence
JA - J Adolesc
VL - 15
IS - 3
CY - United Kingdom
N2 - The present study examined the extent to which boys' and girls' preference for outdoor, vigorous activity changed as they became socialized to middle school. A sample of 133 boys and girls were studied for one year: those just beginning middle school (sixth graders) and those with one year experience (seventh grade). Measures of children's preference for the playground, the duration of their stay outdoors, and behaviors while outdoors were obtained through direct observational methods. Additionally, teachers' rated children's level of achievement, physical attractiveness, and facility in games. Children also rated each other in terms of dominance status. Results indicated that boys', compared to girls', preference to play outdoors existed only in sixth grade. Factor analyses indicated that three factors described these children: Teachers' Choice, Active Female Oriented, and Outdoor Male Oriented. All three factors varied as children progressed through middle school. Results are discussed in terms of life course developmental theory
SN - 0140-1971
AD - (Pellegrini) Institute for Behavioral Research, University of Georgia, Athens, GA 30602, United States
ER -

TY - JOUR
ID - 3992
T1 - Residential exposure to chromium waste - Urine biological monitoring in conjunction with environmental exposure monitoring
A1 - Stern,A.H.
A1 - Freeman,N.C.G.
A1 - Pleban,P.
A1 - Boesch,R.R.
A1 - Wainman,T.
A1 - Howell,T.
A1 - Shupack,S.I.
A1 - Johnson,B.B.
A1 - Lioy,P.J.
Y1 - 1992///
N1 - 1992295507
English
Journal: Article
KW - EMBASE
KW - Adult
KW - article
KW - Child
KW - controlled study
KW - dust exposure
KW - Environmental Exposure
KW - Environmental Monitoring
KW - Female
KW - household
KW - Human

KW - Male
KW - normal human
KW - urinalysis
KW - waste
KW - chromium/to [Drug Toxicity]
RP - NOT IN FILE
SP - 147
EP - 162
JF - Environmental Research
JA - Environ Res
VL - 58
IS - 2
CY - United States
N2 - To determine whether a population living on or adjacent to four sites of chromate production waste was measurably exposed to environmental chromium, spot samples of urine were collected along with wipe samples of household dust and lifestyle/activity interview data. Findings were compared to those from a control population in two communities with no significant chromium use or waste sites. Urine samples were collected and analyzed, employing measures to minimize background chromium contamination. The average Cr mass in dust was 3.7 times that in control houses. The mean creatinine-corrected urine Cr (Cr/c) level of the exposed subgroup residing in households in the 75th percentile of Cr mass in wipe samples was significantly greater than that of the control population. This subgroup was primarily located at a single exposure location. Using lifestyle/activity data, significantly elevated Cr/c urine levels were identified in other exposed subgroups defined by employment location and by outside play time. These data show an association between elevated exposure to chromium in household dust and elevated urine levels of chromium, consistent with residential exposure to chromate production waste. These data also suggest an association between chromium exposure and activities outside the home which are consistent with exposure to chromate production waste
SN - 0013-9351
AD - (Stern, Freeman, Pleban, Boesch, Wainman, Howell, Shupack, Johnson, Lioy) New Jersey Envtl. Protection Dept., Trenton, NJ 08625, United States
ER -

TY - JOUR
ID - 3993
T1 - Antibiotic-resistant pneumococcal disease in South African children
A1 - Friedland,I.R.
A1 - Klugman,K.P.
Y1 - 1992///
N1 - 1992250840
English
Journal: Article
KW - EMBASE
KW - antibiotic sensitivity
KW - article
KW - bactericidal activity
KW - Child
KW - clinical feature
KW - fatality
KW - hospital infection/di [Diagnosis]
KW - hospital infection/et [Etiology]
KW - Human
KW - Incidence
KW - intravenous drug administration
KW - major clinical study
KW - meningitis/dt [Drug Therapy]
KW - meningitis/et [Etiology]
KW - Mortality
KW - oral drug administration

KW - Penicillin Resistance
KW - priority journal
KW - South Africa
KW - Streptococcus infection/di [Diagnosis]
KW - Streptococcus infection/dt [Drug Therapy]
KW - Streptococcus infection/ep [Epidemiology]
KW - Streptococcus infection/et [Etiology]
KW - Streptococcus pneumoniae
KW - ampicillin/ad [Drug Administration]
KW - ampicillin/do [Drug Dose]
KW - ampicillin/dt [Drug Therapy]
KW - cephalosporin derivative/dt [Drug Therapy]
KW - erythromycin/ad [Drug Administration]
KW - erythromycin/dt [Drug Therapy]
KW - imipenem/cb [Drug Combination]
KW - imipenem/dt [Drug Therapy]
KW - penicillin G/ad [Drug Administration]
KW - penicillin G/cr [Drug Concentration]
KW - penicillin G/do [Drug Dose]
KW - penicillin G/dt [Drug Therapy]
KW - penicillin V potassium/ad [Drug Administration]
KW - penicillin V potassium/dt [Drug Therapy]
KW - rifampicin/cb [Drug Combination]
KW - rifampicin/dt [Drug Therapy]
KW - vancomycin/dt [Drug Therapy]
RP - NOT IN FILE
SP - 920
EP - 923
JF - American Journal of Diseases of Children
JA - Am J Dis Child
VL - 146
IS - 8
CY - United States
N2 - Objective. - To determine the incidence of antibiotic-resistant pneumococcal disease and to compare the presentation and outcome of penicillin-resistant infections with penicillin-susceptible infections. Design. - Patient series. Setting. - General community hospital. Patients. - Eighty-three children with penicillin-resistant pneumococcal bacteremia or meningitis and 124 children with penicillin-susceptible pneumococcal bacteremia or meningitis. Selection Procedures. - Consecutive patients admitted between 1989 and 1991. Interventions. - None. Measurements and Results. - Forty percent of community-acquired isolates and 95% of hospital- acquired isolates were resistant to penicillin. Eighty-three (82%) of 101 penicillin-resistant infections were community acquired. Resistance to chloramphenicol, tetracycline, and erythromycin occurred in 9%, 12%, and 4% of all isolates, respectively. The proportion of penicillin-resistant pneumococci with cefotaxime minimum inhibitory concentrations greater than or equal to 0.5 mug/mL increased from 0% in a 1986 study to 21.5% in this study. The sites of infection, underlying diseases, and mortality of patients with penicillin-resistant infections outside the central nervous system did not differ significantly from those of penicillin-susceptible infections. Conclusions. - The resistance of Streptococcus pneumoniae to beta-lactam antibiotics has increased alarmingly in South Africa. Penicillin-resistant and penicillin-susceptible pneumococcal infections cause a similar spectrum of illness
SN - 0002-922X
AD - (Friedland, Klugman) Department of Pediatrics, Baragwanath Hospital, Johannesburg, South Africa
ER -

TY - JOUR
ID - 3994
T1 - Activity patterns of Californians: Use of and proximity to indoor pollutant sources
A1 - Jenkins,P.L.
A1 - Phillips,T.J.

A1 - Mulberg,E.J.
A1 - Hui,S.P.
Y1 - 1992///
N1 - 1992248544
English
Journal: Conference Paper
KW - EMBASE
KW - accuracy
KW - Adolescent
KW - Adult
KW - Aged
KW - combustion
KW - conference paper
KW - daily life activity
KW - exhaust gas
KW - exposure
KW - Female
KW - health survey
KW - Human
KW - indoor air pollution
KW - Interview
KW - major clinical study
KW - Male
KW - school child
KW - season
KW - United States
KW - Paint
KW - solvent
KW - tobacco smoke
RP - NOT IN FILE
SP - 2141
EP - 2148
JF - Atmospheric Environment - Part A General Topics
VL - Conference: 5th International Conference on Indoor Air Quality and Climate: INDOOR AIR '90 [17293]. Toronto, Ont, Can. Conference Start: 19900729 Conference End: 19900803. 26 A
IS - 12
CY - United Kingdom
N2 - The California Air Resources Board funded a statewide survey of activity patterns of Californians over 11 years of age in order to improve the accuracy of exposure assessments for air pollutants. Telephone interviews were conducted with 1762 respondents over the four seasons from fall 1987 through summer 1988. In addition to completing a 24-h recall diary of activities and locations, participants also responded to questions about their use of and proximity to potential pollutant sources. Results are presented regarding time spent by Californians in different activities and locations relevant to pollutant exposure, and their frequency of use of or proximity to pollutant sources including cigarettes, consumer products such as paints and deodorizers, combustion appliances and motor vehicles. The results show that Californians spend, on average, 87% of their time indoors, 7% in enclosed transit and 6% outdoors. At least 62% of the population over 11 years of age and 46% of nonsmokers are near others' tobacco smoke at some time during the day. Potential exposure to different pollutant sources appears to vary among different gender and age groups. For example, women are more likely to use or be near personal care products and household cleaning agents, while men are more likely to be exposed to environmental tobacco smoke, solvents and paints. Data from this study can be used to reduce significantly the uncertainty associated with risk assessments for many pollutants
SN - 0004-6981
AD - (Jenkins, Phillips, Mulberg, Hui) California Air Resources Board, Research Division, P.O. Box 2815, Sacramento, CA 95812, United States
ER -

TY - JOUR

ID - 3995
T1 - Evaluation of humoral responsiveness in children
A1 - Wheeler,J.G.
A1 - Steiner,D.
Y1 - 1992///
N1 - 1992216146
English
Journal: Review
KW - EMBASE
KW - active immunization
KW - age
KW - antibody response
KW - Human
KW - humoral immune deficiency/di [Diagnosis]
KW - humoral immunity
KW - immunocompetence
KW - immunoglobulin blood level
KW - immunoregulation
KW - Immunotherapy
KW - Infant
KW - patient care
KW - preschool child
KW - priority journal
KW - review
KW - diphtheria vaccine
KW - Haemophilus vaccine
KW - immunoglobulin G/ec [Endogenous Compound]
KW - immunoglobulin M/ec [Endogenous Compound]
KW - Pneumococcus vaccine
KW - tetanus toxoid
RP - NOT IN FILE
SP - 304
EP - 310
JF - Pediatric Infectious Disease Journal
JA - Pediatr Infect Dis J
VL - 11
IS - 4
CY - United States
N2 - The evaluation of postvaccine antibody responses can provide a reasonably convenient and useful adjunct to the evaluation of possible humoral immune dysfunction syndromes. In patients whose clinical history is suspicious for an antibody deficiency syndrome, such studies can document normal antibody responsiveness. On the other hand the wide age-dependent variation in antibody responses makes careful interpretation necessary in concluding that a patient falls outside the 'normal' pattern of antibody responsiveness. Finding a patient well below the mean values for age for multiple vaccines in concert with an appropriate clinical history would strongly suggest an abnormality of immune regulation. At this time the finding of abnormal antibody responses does not always imply that intervention with immunoglobulin therapy is indicated or that it would lead to improvement in clinical symptoms. It might help justify prophylactic antibiotic programs, however, similar to those used for recurrent otitis media. Interpretation of antibody responses in children younger than 2 years of age is probably hazardous since many patients may have relative retardation in their normal development of polysaccharide antibody responsiveness. Such patients usually develop a normal response as they grow older and do not need long term therapy. Until new technologies for measuring immune competence are developed, the above-mentioned measures of humoral immunity, when combined with clinical judgment, should be adequate to guide clinical decision-making in most cases
SN - 0891-3668
AD - (Wheeler, Steiner) Department of Pediatrics, Arkansas Children's Hospital, 800 Marshall Street, Little Rock, AR, United States
ER -

TY - JOUR
ID - 3996
T1 - Using longitudinal data to understand children's activity patterns in an exposure context: Data from the Kanawha County Health Study
A1 - Schwab,M.
A1 - McDermott,A.
A1 - Spengler,J.D.
Y1 - 1992///
N1 - 1992155189
English
Journal: Article
KW - EMBASE
KW - Air Pollution
KW - article
KW - daily life activity
KW - Environmental Exposure
KW - Female
KW - health survey
KW - Home
KW - Human
KW - Male
KW - personal monitoring
KW - school
KW - school child
KW - seasonal variation
KW - Time
RP - NOT IN FILE
SP - 173
EP - 189
JF - Environment International
JA - Environ Int
VL - 18
IS - 2
CY - United States
N2 - An important component of assessing the levels, the sources, and the health effects of children's exposure to air pollution is understanding how and where members of this sensitive population spend their time. There are, however, few data bases that allow the documentation of the day-to-day nature of children's activities. Of particular concern is whether the one-day snapshots provided by time activity diaries typically used in exposure studies represent the actual temporal and spatial extent of children's activities. As part of a community health study, longitudinal data on children's time activity patterns were recently collected. A respiratory health status and gender stratified sample of 90 children kept daily diaries over two-week periods during both the summer and the fall. This paper first presents baseline information of children's activity patterns: the sample distribution of time spent in each of five microenvironments (travel, outdoor, at school, at home, and inside other locations) and the daily temporal pattern of activities. The consistent patterns of children on school days suggest that for most days we can accurately predict children's locations by time of day. The second part of the analysis shows that there is both high child-to-child variation in the average time spent in each microenvironment, even after controlling for gender and respiratory health status, and strong temporal variability in activity patterns within a child over time, even after controlling for school days versus nonschool days
SN - 0160-4120
AD - (Schwab, McDermott, Spengler) c/o ManTech Environmental Services, 2 Triangle Drive, Research Triangle Park, NC 27709, United States
ER -

TY - JOUR
ID - 3998

T1 - Catheter ablation of left posteroseptal accessory pathways and of long RP' tachycardias with a right endocardial approach
A1 - Haissaguerre,M.
A1 - Montserrat,P.
A1 - Warin,J.F.
A1 - Donzeau,J.P.
A1 - Le Metayer Massiere,P.J.P.
Y1 - 1991///
N1 - 1991262548
English
Journal: Article
KW - EMBASE
KW - Adolescent
KW - Adult
KW - Aged
KW - article
KW - atrioventricular block/su [Surgery]
KW - atrioventricular conduction
KW - cauterization
KW - congestive cardiomyopathy/su [Surgery]
KW - coronary sinus
KW - Ebstein anomaly/su [Surgery]
KW - Electrocardiography
KW - Electrophysiology
KW - endocardium
KW - Female
KW - heart accessory conduction pathway/su [Surgery]
KW - heart atrium fibrillation/su [Surgery]
KW - heart catheter
KW - heart left bundle branch block/su [Surgery]
KW - heart preexcitation/su [Surgery]
KW - heart right atrium
KW - heart ventricle fibrillation/su [Surgery]
KW - Human
KW - hypertrophic cardiomyopathy/su [Surgery]
KW - Male
KW - priority journal
KW - school child
KW - shock/su [Surgery]
KW - syncope/su [Surgery]
KW - tachycardia/su [Surgery]
RP - NOT IN FILE
SP - 845
EP - 859
JF - European Heart Journal
VL - 12
IS - 8
CY - United Kingdom
N2 - Fifty-four patients with a posteroseptal accessory connection and symptomatic tachycardias underwent catheter ablation of the anomalous pathway. Eight had the permanent form of reciprocating tachycardia (long RP' tachycardia) and 46 had a left posteroseptal preexcitation marked by a prominent R wave in lead VI. In 14 of 19 patients, ventriculoatrial conduction time during tachycardia lengthened in conjunction with functional left bundle branch block, this behaviour was significantly different from a series of patients with right posteroseptal preexcitation in which functional left bundle branch block lengthened the ventriculoatrial time in only one of 12 patients. A quadripolar electrode catheter was left within the proximal coronary sinus in order to locate the earliest atrial or ventricular activation site. The appropriate bipole was used as the radiographic and electrophysiological reference of the insertion of the accessory pathway. A catheter was then positioned on the

septal side of the right atrium, outside the coronary sinus, so that atrial activity during reciprocating tachycardia and ventricular activity during preexcitation were synchronous with or earlier than that recorded within the proximal coronary sinus. Accessory pathway potential was not recorded in any patient. Early ventricular potential occurring -1.5+8ms relative to delta wave onset was present at that site. In 38 patients, including 5 with permanent junctional tachycardia, high current (14mA) pacing yielded direct ventricular paced QRS complexes (no delay spike-QRS) with a morphology similar to left posteroseptal maximal preexcitation. Slight movements of catheter position yielded significantly different pace-maps. One to eight 160J cathodal shocks (510+213J cumulative per patient) were delivered at this site in 61 sessions. Following fulguration, tachycardia recurred without drugs in only one patient over a follow-up period of 20+13 months. Asymptomatic intermittent preexcitation recurred in two patients. In all patients with long RP' tachycardia, the ablation procedure was successful without the need for drugs or permanent cardiac pacing. A long-term follow-up electrophysiological study in 18 patients demonstrated that conduction through the anomalous pathway was absent in 16 and deeply altered in the two patients with intermittent preexcitation; no tachycardia was inducible in any patient. In conclusion, catheter ablation of left posteroseptal accessory pathways is a feasible procedure using a right atrial approach outside the coronary sinus. This technique is also effective for the treatment of the permanent form of reciprocating tachycardia
SN - 0195-668X
AD - (Haissaguerre, Montserrat, Warin, Donzeau, Le Metayer Massiere) Ser de Cardiol et Med Interne, Hopital Saint-Andre, Bordeaux, France
ER -

TY - JOUR
ID - 3999
T1 - Reduction of bronchial hyperreactivity and exercise-induced bronchospasm in children treated for one month with a pressurized sodium cromoglycate aerosol
A1 - Calvayrac,P.
A1 - Leophonte,P.
A1 - Fuseiller,A.
A1 - Andre,C.
Y1 - 1991///
N1 - 1991167396
English
Journal: Article
KW - EMBASE
KW - aerosol
KW - article
KW - Asthma/dt [Drug Therapy]
KW - bronchospasm/dt [Drug Therapy]
KW - Child
KW - clinical article
KW - clinical trial
KW - controlled study
KW - Exercise
KW - Female
KW - Human
KW - inhalational drug administration
KW - Male
KW - priority journal
KW - cromoglycate disodium/ct [Clinical Trial]
KW - cromoglycate disodium/dt [Drug Therapy]
RP - NOT IN FILE
SP - 422
EP - 435
JF - Current Therapeutic Research - Clinical and Experimental
VL - 49
IS - 3
CY - United States

N2 - Bronchial hyperreactivity demonstrated by means of a standardized exercise test in 30 asthmatic children was studied before and after one month of treatment with a pressurized aerosol providing 5 mg of sodium cromoglycate per spray. This study was carried out as a double-blind comparative trial against placebo, outside the period of allergenic provocation. Ventilatory parameters were measured before, and then 5, 15, and 30 minutes after a period of six to nine minutes on a moving walkway. The test session included three-minute incremental increases in gradient and speed. This exercise test was performed on day 1 without prior treatment; on day 2 and day 34 it was preceded by administration of two sprays of sodium cromoglycate, 15 minutes earlier. The baseline values of ventilatory parameters on days 1, 2, and 34, like their variations following exercise on day 1, were similar in the treated and the placebo group. On day 1, five minutes after the exercise test, the fall in the forced expiratory volume in one second (FEV₁) was greater than or equal to 20% in all cases and was accompanied by a highly significant decrease in the vital capacity (VC), the midexpiratory flow between 25% and 75% of VC (MEF(25%-75%)), the flow at 25% of VC (V25), the flow at 50% of VC (V50), the flow at 75% of VC (V75), and peak expiratory flow (PEF). On day 2, five minutes after the exercise test, 66.7% of the children in the placebo group versus 20% of the children in the treated group had a fall in the FEV₁ of greater than 20% (P = 0.01). Decreases in VC, V75, and PEF were significantly greater in the placebo group than in the treated group. After one month of treatment, the active and placebo groups were statistically different for all ventilatory parameters, with the most marked variations in the placebo group; five minutes after the exercise test, 64.3% of children in the placebo group had a fall in FEV₁ of greater than 20% versus 13.3% of children in the treated group(P = 0.007); VC was not changed after exercise in the active group. Compared with day 2, five minutes after exercise, MEF(25%-75%), V25, and V50 were highly significantly improved in absolute terms in the treated group only (P < 0.001). This statistically significant improvement in airway flow rates at the bronchiolar level shows that regular use of sedium cromoglycate reduces bronchial hyperreactivity
SN - 0011-393X
AD - (Calvayrac, Leophonte, Fuseiller, Andre) Hopital Purpan, Service Pneumo-, Phtisiologie, Place du Pr. Baylac, 31059 Toulouse Cedex, France
ER -

TY - JOUR
ID - 4001
T1 - Daily activities and breathing parameters for use in respiratory tract dosimetry
A1 - Roy,M.
A1 - Courtay,C.
Y1 - 1991///
N1 - 1991084029
English
Journal: Article
KW - EMBASE
KW - Adult
KW - article
KW - breathing pattern
KW - Child
KW - controlled study
KW - dosimetry
KW - Human
KW - human experiment
KW - lung burden
KW - normal human
KW - Physical Activity
RP - NOT IN FILE
SP - 179
EP - 186
JF - Radiation Protection Dosimetry
JA - Radiat Prot.Dosimetry
VL - 35
IS - 3
CY - United Kingdom

N2 - Dosimetry of inhaled substances is based on the air volumes breathed every day by people under exposure to gases and aerosols. In order to assess modern standards for average inspired air volumes according to age and gender, information was recorded on daily activities and breathing rates both indoors and outdoors, of specific categories of the population. Economic surveys recently published provided time budgets and activities of adults, teenagers and children. The data were matched with published data on physical activities and breathing parameters in order to calculate the daily inspired volumes of air. The results were given for adults (age > 17 years), neonates, and children 1, 5, 10 and 15 years old. The values obtained are close to those published by the International Commission for Radiological Protection and the reports of the United Nations Scientific Committee on the Effects of Atomic Radiation
SN - 0144-8420
AD - (Roy, Courtay) IPSN/DPS/SEAPS, Commissariat a L'Energie Atomique, BP 6, 92265 Fontenay-aux-Roses Cedex France
ER -

TY - JOUR
ID - 4002
T1 - Anaerobic threshold alterations caused by interval training in 11-year-olds
A1 - Haffor,A.-S.
A1 - Harrison,A.C.
A1 - Kirk,P.A.C.
Y1 - 1990///
N1 - 1990254338
English
Journal: Article
KW - EMBASE
KW - anaerobic capacity
KW - article
KW - Child
KW - Exercise
KW - Female
KW - Human
KW - Male
RP - NOT IN FILE
SP - 53
EP - 56
JF - Journal of Sports Medicine and Physical Fitness
VL - 30
IS - 1
CY - Italy
N2 - The purpose of this study was to examine the effects of the interval training on metabolic parameters at maximal work and at the anaerobic threshold in 11 years olds. The subjects were five healthy male children. They trained outdoor for 50 min a day, five times a week for six weeks, using interval work at 25 and 50% above their anaerobic threshold. Before and after training program, they performed a progressive exercise test on a cycle ergometer. During the last 15 sec of each power output measurements were made of oxygen uptake (VO₂), carbon dioxide output (VCO₂), heart rate (HR), ventilation (V(E)), ventilatory equivalent for oxygen (V(E)O₂) and ventilatory equivalent for carbon dioxide V(E)CO₂). Following training, the group increased their anaerobic threshold (expressed as %VO₂max) significantly (P<0.05) during the progressive exercise test, by 22%. Also at the anaerobic threshold level, increases were observed following training in CO₂ output (VCO₂-AT) and respiratory exchange ratio (R). Oxygen uptake (VO₂, 1.min⁻¹) was increased by 19%, but the difference was not significant (P>0.05). Maximal ventilatory equivalent for (V(E)CO₂max) decreased significantly (P<0.05). Maximal heart rate was reduced significantly (P<0.05). We conclude that training led to an increase of both anaerobic and aerobic metabolism, at any submaximal work above the anaerobic threshold, for this specific age group
SN - 0022-4707
AD - (Haffor, Harrison, Kirk) Department of Pulmonary Physiology, LAC/University of Southern California Medical Center, Los Angeles, CA United States

ER -

TY - JOUR
ID - 4003
T1 - The effect of maternal work on fetal growth and duration of pregnancy: A prospective study
A1 - Launer,L.J.
A1 - Villar,J.
A1 - Kestler,E.
A1 - De,Onis M.
Y1 - 1990///
N1 - 1990136476
English
Journal: Article
KW - EMBASE
KW - article
KW - Child
KW - fetus growth
KW - fetus outcome
KW - Human
KW - major clinical study
KW - Newborn
KW - priority journal
KW - Work
RP - NOT IN FILE
SP - 62
EP - 70
JF - British Journal of Obstetrics and Gynaecology
VL - 97
IS - 1
CY - United Kingdom
N2 - The effect on birth outcome of work requiring different degrees of physical exertion was examined among 15786 pregnant women who were followed through the Guatemalan Social Security Institute's Hospital. Work inside and outside the home was ascertained through a questionnaire administered to each woman before delivery. Odds ratios were adjusted for household income, maternal height and age, and birthweight of previous infant. Women with three or more children and no household help were at increased risk for small-for-gestational-age (SGA) births compared with women with family (odds ratio (OR) 1.79; 95% confidence interval (CI) 1.31, 2.47) or hired help (OR 2.0; 95% CI 1.16 to 3.33). Compared with office work, manual work increased the risk for an SGA (OR 1.32; 95% CI 1.12 to 1.56) and SGA/preterm birth (OR 2.56; 95% CI 1.10 to 5.96). Work in a standing compared with sitting position significantly increased the risk for a preterm birth (OR 1.56; 95% CI 1.04 to 2.60). There was a significant positive trend in frequency of SGA and SGA/preterm birth with an increase in the physical demands at work, as measured by an activity score. These data suggest that interventions to reduce physical exertion among pregnant women could improve birth outcome
SN - 0306-5456
AD - (Launer, Villar, Kestler, De Onis) Prevention Research Program, Nat. Inst. of Child Health, National Institutes of Health, Bethesda, MD 20892, United States
ER -

TY - JOUR
ID - 4004
T1 - Estimating the effect of being indoors on total personal exposure to outdoor air pollution
A1 - Hayes,S.R.
Y1 - 1989///
N1 - 1990018848
English
Journal: Article
KW - EMBASE
KW - Adult

KW - agricultural worker
KW - air quality
KW - article
KW - Child
KW - Female
KW - Human
KW - indoor air pollution
KW - Male
KW - nonbiological model
KW - population exposure
KW - Work
KW - Ozone
RP - NOT IN FILE
SP - 1453
EP - 1461
JF - Journal of the Air and Waste Management Association
VL - 39
IS - 11
CY - United States
N2 - A personal air quality model (PAQM) has been developed to estimate the effect of being indoors on total personal exposure to outdoor-generated air pollution. Designed to improve air toxics risk assessment, PAQM accounts for individual hourly activity patterns, indoor-outdoor differences, physical exercise level, and geographic location for up to 56 different population groups. Unique hourly activity profiles are specified for each population group; group members are assigned each hour to one of up to 10 different indoor and outdoor microenvironments. To illustrate PAQM use, we apply it to two example cases: a long-term example representative of situations where pollutant health impact is related to integrated exposure (as in the case of potentially carcinogenic air toxics) and a short-term example representative of situations where health impact is related to acute exposure to peak concentrations (as with ozone). Case study results illustrate that personal exposure, and thus health risk, attributable to outdoor-generated air pollution is sensitive to indoor-outdoor differences and population mobility. Where health impact is related to long-term integrated exposure (e.g., air toxics), exposure and subsequent risks are likely to be lower than that estimated by previous modeling techniques which do not account for such effects
SN - 0894-0630
AD - (Hayes) Environmental Sciences, Systems Applications, Inc., 101 Lucas Valley Road, San Rafael, CA 94903 United States
ER -

TY - JOUR
ID - 4005
T1 - Health effects of ozone. A critical review
A1 - Lippman,M.
Y1 - 1989///
N1 - 1989136383
English
Journal: Article
KW - EMBASE
KW - Adult
KW - Child
KW - Forced Expiratory Volume
KW - Human
KW - human experiment
KW - lung function
KW - normal human
KW - population exposure
KW - respiratory tract disease/ep [Epidemiology]
KW - review
KW - Ozone

RP - NOT IN FILE
SP - 672
EP - 695
JF - Journal of the Air and Waste Management Association
VL - 39
IS - 5
CY - United States
N2 - Health and pollution control professionals and the general public need to develop a more complete understanding of the health effects of ozone (O₃) because: 1) we have been unable to significantly reduce ambient O₃ levels using current strategies and controls; 2) in areas occupied by more than half of the U.S. population, current peak ambient O₃ concentrations are sufficient to elicit measurable transient changes in lung function, respiratory symptoms, and airway inflammation in healthy people engaged in normal outdoor exercise and recreational activities; 3) the effects of O₃ on transient functional changes are sometimes greatly potentiated by the presence of other environmental variables; and 4) cumulative structural damage occurs in rats and monkeys exposed repetitively to O₃ at levels within currently occurring ambient peaks, and initial evidence from dosimetry models and interspecies comparisons indicate that humans are likely to be more sensitive to O₃ than rats. The extent and significance of these effects, and the multibillion dollar costs of ambient O₃ controls need to be considered in any future revisions of ambient standards and the Clean Air Act. The transient effects of O₃ are more closely related to cumulative daily exposure than to one hour peak concentrations, and future revisions of the ambient standard for O₃ should take this into account. The effects of long-term chronic exposure to O₃ remain poorly defined, but recent epidemiologic and animal inhalation studies suggest that current ambient levels are sufficient to cause premature aging of the lungs. More research is needed to determine the need for a standard with a seasonal or annual average concentration limit
SN - 0894-0630
AD - (Lippman) Institute of Environmental Medicine, New York University Medical Center, Tuxedo, NY 10016 United States
ER -

TY - JOUR
ID - 4006
T1 - Cooperation between the German Democratic Republic and the Czechoslovak Socialist Republic in the study of long-term air pollution effects in selected groups of school-age children
A1 - Pelech,L.
Y1 - 1989///
N1 - 1989060886
German
Journal: Article
KW - EMBASE
KW - Child Development
KW - child growth
KW - Human
KW - indoor air pollution
KW - normal human
KW - passive smoking
KW - Recreation
KW - school child
KW - short survey
KW - long acting drug
RP - NOT IN FILE
SP - 50
JF - Zeitschrift fur die Gesamte Hygiene und Ihre Grenzgebiete
JA - Z Gesamte Hyg
VL - 35
IS - 1
CY - Germany

N2 - This report presents key outcomes of joint growth and development studies conducted in children permanently living in areas adversely affected by pollutants in air. Further attention is focussed on the evaluation of effects of some so-called compensatory measures applied in Czechoslovakia, namely a 3-week outdoor schooling organized for these children in the relatively clean recreational areas. The beneficial health effects of year's season and specifically of the spontaneous, active and sufficiently long outdoor exercise are documented in graphs. As it is pointed out in this context, children in the place of their permanent residence tend to have relative little exercise outdoor, may run an increased risk of exposure to indoor pollutants, and may suffer from passive smoking effects if their parents cannot refrain from smoking habits at home
SN - 0049-8610
AD - (Pelech) Institut Hygieny a Epidemiologie , 100-42 Praha 10 Czechoslovakia
ER -

TY - JOUR
ID - 4008
T1 - Playground-related injuries in preschool-aged children--United States, 1983-1987
Y1 - 1988///
N1 - 3139984
English
Journal: Article
KW - EMBASE
KW - article
KW - Day Care
KW - equipment
KW - falling/pc [Prevention]
KW - Female
KW - Human
KW - Infant
KW - injury/ep [Epidemiology]
KW - injury/et [Etiology]
KW - injury/pc [Prevention]
KW - Male
KW - preschool child
KW - Recreation
KW - school
KW - sex difference
KW - United States
RP - NOT IN FILE
SP - 629
EP - 632
JF - MMWR
VL - Morbidity and mortality weekly report. 37
IS - 41
CY - United States
SN - 0149-2195
ER -

TY - JOUR
ID - 4009
T1 - Autism: A disorder of directed attention
A1 - Ornitz,E.M.
Y1 - 1988///
N1 - 1989197345
English
Journal
KW - EMBASE
KW - attention deficit disorder/cn [Congenital Disorder]
KW - autism/cn [Congenital Disorder]

KW - Brain Stem
KW - central nervous system
KW - Child
KW - congenital disorder
KW - Human
KW - Infant
KW - psychological aspect
KW - review
KW - stereotypy
RP - NOT IN FILE
SP - 309
EP - 322
JF - Brain Dysfunction
VL - 1
IS - 5-6
CY - Switzerland
N2 - Autism is a developmental disorder characterized by a triad of behavioral impairments involving (1) reciprocal social relationships, (2) communicative intent, and (3) the ability to play, to invest play with imagination, and to develop interests outside of stereotypic preoccupations. Deficiencies in these essential human characteristics occur in all autistic children, 80% of whom are mentally retarded. They can be attributed to a deficiency in the capacity to direct attention to persons, things, and activities; hence, they could reflect an impairment in sensory processing. Two additional symptom clusters are found in autism. The disturbances of sensory modulation are characterized by under- and overreactivity to sensory stimuli. The overreactivity includes distraction by background stimuli, distress from stimulation, and, paradoxically, behavior that provides repetitive sensory input. The motility disturbances are stereotypic behaviors which mediate this self-stimulation. The disturbances of sensory modulation and motility are observed clinically primarily before 6 years of age. This paper documents these sensory and motor behaviors in young autistic children by quantitative evaluation of their parents' responses on a standardized developmental inventory and demonstrates that they are essential components of the autistic syndrome. Based on their presence, a neurophysiologic model of autism as a disorder of sensory processing and directed attention is elaborated. In this model, there is dysfunction of brainstem and related diencephalic systems and the cascading impact of such dysfunction on higher neural structures which elaborate, refine and modulate the activities of the lower centers. Autism results from dysfunction of this neuroanatomically distributed sensory processing system which mediates the direction of attention
SN - 0259-1278
AD - (Ornitz) Department of Psychiatry, University of California, Los Angeles, CA 90024 United States
ER -

TY - JOUR
ID - 4011
T1 - Risk factors for infection with Echinococcus multilocularis in Alaska
A1 - Stehr-Green,J.K.
A1 - Stehr-Green,P.A.
A1 - Schantz,P.M.
A1 - Wilson,J.F.
A1 - Lanier,A.
Y1 - 1988///
N1 - 1988086122
English
Journal: Article
KW - EMBASE
KW - Adult
KW - cestode
KW - Child
KW - clinical article
KW - dog
KW - echinococcosis/di [Diagnosis]
KW - echinococcosis/ep [Epidemiology]

KW - Echinococcus multilocularis
KW - Female
KW - fox
KW - histology
KW - Human
KW - Male
KW - priority journal
KW - risk factor
KW - United States
RP - NOT IN FILE
SP - 380
EP - 385
JF - American Journal of Tropical Medicine and Hygiene
VL - 38
IS - 2
CY - United States
N2 - We studied 19 Eskimo patients with alveolar hydatid disease from the north-western coast of Alaska for risk factors for infection with Echinococcus multilocularis. Each case-patient was matched by age and sex with 2 unrelated controls who had no clinical or serologic evidence of infection with E. multilocularis and who resided in three villages endemic for alveolar hydatid disease. Behaviors thought to increase exposure to E. multilocularis and the chronologic occurence of these behaviors in the participant's life were assessed by a standardized questionnaire. Case-patients were more likely than controls to have owned dogs for their entire lives (odds ratio 6.00, P < 0.05), tethered their dogs near the house (odds ratio 8.50, P < 0.05), and lived in housed built directly on the tundra rather than on a gravel or a permanent foundation (odds ratio 11.00, P < 0.01). Case-patients were not more likely to have owned sled dogs, trapped or skinned foxes, or engaged in other outdoor activities away from home. These findings suggest that controlling the parasite in the domestic dog population, as well as controlling the dog population itself, are important aspects of preventing alveolar hydatid disease in the northwestern Native Alaskan population
SN - 0002-9637
AD - (Stehr-Green, Stehr-Green, Schantz, Wilson, Lanier) Center for Infectious Diseases, Centers for Disease Control, Atlanta, GA 30333 United States
ER -

TY - JOUR
ID - 4012
T1 - Effects of mainstreaming on motor performances of intellectually normal and trainable mentally retarded students
A1 - Beuter,A.C.
Y1 - 1983///
N1 - 1983169749
English
Journal: Article
KW - EMBASE
KW - central nervous system
KW - Human
KW - human experiment
KW - major clinical study
KW - mental deficiency
KW - musculoskeletal system
KW - normal human
KW - Physical Education
KW - Psychomotor Performance
KW - school child
KW - Therapy
RP - NOT IN FILE
SP - 48
EP - 52

JF - American Corrective Therapy Journal
VL - 37
IS - 2
CY - United States
N2 - This experiment was designed to assess the effects of integrating intellectually normal and trainable mentally retarded (TMR) children into a physical activity program. The following research hypotheses were tested: (1) TMR students integrated with intellectually normal pupils will show greater improvements in their motor performance than their nonintegrated mentally retarded peers; and (2) intellectually normal students integrated with TMR pupils will show no lesser improvements in their motor performance than their nonintegrated peers. Two age levels of TMR children were integrated with intellectually normal third and sicxsth grade children in a physical education instructional program over a period of five months. Motor performance test scores were secured at the beginning and end of the experiment. Similar data were obtained on control groups of TMR and intellectually normal children in nonintegrated instructional settings. One hundred and ten students in two experimental and four control groups completed the experiment. The intellectually normal-TMR student ratio in the integrated program was about four intellectually normal children to one TMR child. The instructional program (similar for all groups) emphasized fundamental motor patterns with attention also given to sport skills. Three motor tests, namely, the 20-yard dash, standing long jump, and softball throw were used in assessing motor performance of all children. It was found that under the conditions of this experiment the scores of the intellectually normal subjects were in no way impaired by the integrated program. Motorically, the TMR subjects participating in the integrated program showed significantly greater gains than those participating in the segregated program
SN - 0002-8088
AD - (Beuter) Dep. Phys. Educ., Univ. South. California, Los Angeles, CA 90089-0652 United States
ER -

TY - JOUR
ID - 4013
T1 - A naturalistic assessment of the motor activity of hyperactive boys: II. Stimulant drug effects
A1 - Porrino,L.J.
A1 - Rapoport,J.L.
A1 - Behar,D.
Y1 - 1983///
N1 - 1983163788
English
Journal: Article
KW - EMBASE
KW - central nervous system
KW - Child
KW - clinical article
KW - controlled study
KW - Drug Therapy
KW - Human
KW - hyperactivity
KW - Motor Activity
KW - musculoskeletal system
KW - oral drug administration
KW - psychological aspect
KW - Therapy
KW - dexamphetamine
KW - placebo
RP - NOT IN FILE
SP - 688
EP - 693
JF - Archives of General Psychiatry
JA - Arch Gen Psychiatry
VL - 40
IS - 6

CY - United States
N2 - Twenty-four-hour motor activity was assessed in a naturalistic setting in 12 hyperactive boys for four weeks (672 consecutive hours). Dextroamphetamine, 15 mg/day, or placebo was administered on alternate weeks, using a double-blind ABAB design. When the boys received dextroamphetamine, motor activity was significantly decreased for about eight hours after drug administration. This decrease was followed by a period of slight but significant increases in activity ('rebound'). Dextroamphetamine decreased activity most strikingly during structured classroom activity; during physical education, however, there was a significant drug-induced increase in motor activity
SN - 0003-990X
AD - (Porrino, Rapoport, Behar) Natl. Inst. Ment. Health, Bethesda, MD 20205 United States
ER -

TY - JOUR
ID - 4014
T1 - Bites and parasites
A1 - Honig,P.J.
Y1 - 1983///
N1 - 1983207557
English
Journal: Article
KW - EMBASE
KW - arthropod
KW - bite
KW - cestode
KW - Child
KW - Diagnosis
KW - Drug Therapy
KW - helminth
KW - helminthiasis
KW - Human
KW - insect
KW - nematode
KW - parasite
KW - scabies
KW - short survey
KW - Therapy
KW - tick bite
KW - trematode
KW - lindane
KW - tiabendazole
RP - NOT IN FILE
SP - 563
EP - 581
JF - Pediatric Clinics of North America
VL - 30
IS - 3
CY - United States
N2 - The vulnerability of children to arthropod bites, infestations, and parasites is well known. Unfortunately, the papules, urticaria, blisters, nodules, and hemorrhagic lesions produced by these conditions are frequently misdiagnosed. This difficulty in diagnosis exists because most lesions are not absolutely characteristic and physicians may not recognize the lesions caused by insects and parasites indigenous to areas outside of their practice region. The evaluation and diagnosis of any dermatologic problem requires utilization of historical clues in addition to direct observation. Information that is essential to elicit includes the seasonal incidence of the lesions, recent history of travel or migration, the playing habits of the child, exposure to pets, and the existing environment and living conditions. The grouping and distribution of lesions is also very important to consider. This article will discuss the dermatologic findings and treatment of the conditions caused by a number of the most common arthropods and helminths

SN - 0031-3955
AD - (Honig) Dermatol. Sect., Child. Hosp. Philadelphia, Philadelphia, PA 19104 United States
ER -

TY - JOUR
ID - 4015
T1 - Accidents with playground equipment. III. Accidental falls
A1 - Christensen,S.T.
A1 - Mikkelsen,P.
A1 - Reich,H.
A1 - Krebs,B.
Y1 - 1982///
N1 - 1983053532
Danish
Journal
KW - EMBASE
KW - accident
KW - age
KW - Child
KW - Epidemiology
KW - Human
KW - injury
KW - playground
RP - NOT IN FILE
SP - 3568
EP - 3571
JF - Ugeskrift for Laeger
JA - Ugeskr.Laeger
VL - 144
IS - 48
CY - Denmark
N2 - During the period 1. VII. 1979-30. VI. 1980, 466 children aged 1-14 yr were registered in the Casualty Departments of the Municipal and County Hospitals in Aarhus with injuries resulting from use of playground equipment. In 329 cases (70.2%), falls were involved. In every case, the scene of the accident was visited. The height of the fall was measured and the underlying surface described. These two parameters were compared with the age and sex of the child and the severity of the injury as classified by the Abbreviated Injury Scale (AIS). The height of fall was shown to increase with increasing age ($p < 0.001$). In the ages 0-9 yr, sex had no influence on the fall height but in the age group 10-14 yr, greater fall heights were demonstrated in boys ($p = 0.013$). The fall heights for the individual types of playground equipment deviated statistically significantly from one another, the greatest fall heights being for climbing frames and the least from swings ($p < 0.001$). The severity of the injuries sustained increased with increasing fall heights ($p < 0.001$). In the majority of cases, the underlying surfaces were unsuitable. Surfaces with sufficient shock absorbing properties were found in only 22 cases (6.7%). The problems surrounding the underlying surface are reviewed. It is considered advisable that the maximal fall height for any playground equipment and the underlying surface should be adapted to one another. It should thus be impossible for a child falling accidentally from a form of playground equipment to reach deceleration levels which exceed 50 G at the impact with the underlying surface
SN - 0041-5782
AD - (Christensen, Mikkelsen, Reich, Krebs) Ulykkes Anal. Cent. Arhus Amtskommune, 8000 Aarhus Denmark
ER -

TY - JOUR
ID - 4016
T1 - Accidents with playground equipment. I. An epidemiological description
A1 - Christensen,S.T.
A1 - Mikkelsen,P.
A1 - Reich,H.

A1 - Krebs,B.
Y1 - 1982///
N1 - 1983053530
Danish
Journal
KW - EMBASE
KW - accident
KW - Child
KW - Denmark
KW - Epidemiology
KW - geographic distribution
KW - injury
KW - play
KW - Prevention
KW - psychological aspect
KW - sex difference
KW - short survey
RP - NOT IN FILE
SP - 3561
EP - 3564
JF - Ugeskrift for Laeger
JA - Ugeskr.Laeger
VL - 144
IS - 48
CY - Denmark
N2 - In order to illustrate the frequency of accidents with playground equipment, children aged 0-14 years who sustained injuries while using such equipment during the period 1.VII.1979-30.VI.1980 were registered in the Municipal Hospital and the County Hospital in Aarhus. In addition, in-depth investigation of each accident was undertaken. A total of 466 children were registered and, as the number of children aged 0-14 years in the region served by the hospitals was 50,000, this corresponds to an incidence rate of 9.3/1000 children/year. A further 20% were treated by the general practitioners. The number of accidents was greatest in the age group 4-5 years. The sex ratio for the total material was 1.44 (boys/girls). The sex ratio was observed to increase with age. Accumulation of accidents was observed at midday and late in the afternoon. The number of accidents was greatest on week days and the number was halved at week-ends. The greatest number of accidents occurred in the months of May, August and September. One hundred and sixty-nine accidents occurred in private playgrounds, 146 in day-care institutions, 90 in school playgrounds and 55 in municipal playgrounds. Swings, climbing frames and slides were responsible for 64% of the accidents. The next most frequent were play houses and seesaws. Small children were most frequently injured on slides while older children were injured while playing on climbing frames. Girls dominated where play epuipment with a swinging motion was concerned
SN - 0041-5782
AD - (Christensen, Mikkelsen, Reich, Krebs) Ulykkes Anal. Cent. Arhus Amtskommune, 8000 Aarhus Denmark
ER -

TY - JOUR
ID - 4017
T1 - Ergonomic approach to school work: Ideas and realities
A1 - Maringue-Bardin,M.
A1 - Maringue-DeVaux,L.
Y1 - 1982///
N1 - 1982116985
French
Journal
KW - EMBASE
KW - Diagnosis
KW - ergonomics
KW - France

KW - geographic distribution
KW - Methodology
KW - normal human
KW - Prevention
KW - school
KW - school child
RP - NOT IN FILE
SP - 133
EP - 148
JF - Medecine Infantile
VL - 89
IS - 1
CY - France
N2 - The 30-year history of ergonomics as applied to adults' occupational activities is outlined. Plat applied ergonomic principles to school work and to children's activities outside school, with a view to their developing as physiologically as possible and to creating optimal conditions of perception, learning and assimilation of knowledge. Since 1976-1977, the concept of school ergonomics has been promoted officially in France by the Department of Biomedical Research at the National Institute of Pedagogic Research. This institute uses earlier work on the biological, psychological and sociological characteristics of pedology and stimulates new research. A report is presented of results so far obtained in regard to rational scientific organization of studies and anthropometric knowledge of the child. An analysis is made of the schoolchild's optimal work place, with reference to the screen terminals in computer-assisted teaching, and to optimal environmental factors
SN - 0025-6773
AD - (Maringue-Bardin, Maringue-DeVaux) Rech. Bio-Med. INRP, Serv. DP6, 63400 Chamalieres France
ER -

TY - JOUR
ID - 4018
T1 - Seven years of Day-Hospital service; a cost/benefit analysis
A1 - Lepore,L.
A1 - Paulon,G.
A1 - Tamaro,P.
Y1 - 1982///
N1 - 1982235377
Italian
Journal
KW - EMBASE
KW - Child
KW - childhood cancer
KW - cost benefit analysis
KW - economic aspect
KW - Epidemiology
KW - geographic distribution
KW - Hospital
KW - major clinical study
KW - socioeconomics
RP - NOT IN FILE
SP - 189
EP - 195
JF - Rivista Italiana di Pediatria
VL - 8
IS - 2
CY - Italy
N2 - A cost/benefit analysis of both general and oncologic Day-Hospital (D-H) activity in the Pediatric Clinic of Trieste has been performed. Three years of activity, one immediately before and two after the beginning of the service, have been considered. For each period we have evaluated: total number of D-H admissions and traditional admissions in the ward, the number of first visits and subsequent controls, and the residence of the

patients (outside or inside the province) and the average days of hospitalisation. Qualitative analysis was performed with respect to the indication for hospital admission (simple criteria for usefulness were established) and as to the outcome the problem was totally or partially solved?). The average cost of the service was calculated on the basis of laboratory and instrumental examinations concerning the general D-H, and on the basis of examinations and drugs administered to the patients in relation to the oncology D-H. The total number of hospital admissions as well as the total number of first visits did not substantially change in the three years under consideration, whereas the number of traditional admissions in the ward diminished. The average length of hospitalisation (ward + D-H) diminished from 3.6 to 2.9 days, whereas the average of days of hospitalisation in the ward increased from 3.6 to 5.4, due to the almost exclusive utilisation of the ward for severe illnesses. The decrease of the average length of hospitalisation was not due to an higher number of admissions for a single patient, since the length of stay for a single patient is still very low: 4.3 days. These data do not show hyperutilisation of D-H: 'useless' admissions passed from 23% to 4% in the general ward and from 37% to 23% in D-H. The general D-H can be considered a service with a good outcome, since 85% of the problems were solved. Expenses for the staff did not increase in the period under consideration and the cost for general D-H service per day (about 21000 liras) and for the oncologic D-H service per day (about 52000 liras) appear reasonable (the higher cost for the oncologic D-H service is mainly due to the redundance of examinations required in order to follow the multicentric protocols)
SN - 0392-5161
AD - (Lepore, Paulon, Tamaro) Clin. Ped., Ist. Inf. Burlo Garofolo, 34137 Trieste Italy
ER -

TY - JOUR
ID - 4019
T1 - Semiweekly hospitalization for very young children who are at high risk for psychosis
A1 - Rufo,M.
A1 - Dubois,Y.
A1 - Staub,D.
A1 - Soulayrol,R.
Y1 - 1981///
N1 - 1982018839
French
Journal: Article
KW - EMBASE
KW - central nervous system
KW - Hospitalization
KW - major clinical study
KW - preschool child
KW - Psychosis
KW - school child
KW - Therapy
RP - NOT IN FILE
SP - 483
EP - 488
JF - Neuropsychiatrie de l'Enfance et de l'Adolescence
VL - 29
IS - 8-9
CY - France
N2 - Child psychiatric consultations at a children's hospital have permitted earlier detection of children who show signs of a disequilibrium between their lines of development. A number of therapeutic principles were accepted: the notion that parents could no longer tolerate their children, the notion that nothing was possible without the parents' active participation in their children's therapy through weekly meetings with parents and mental health workers as specified in a preliminary 'contract' with the parents. The notion that these children were capable of rendering their parents incompetent was likewise acknowledged, and therefore care was taken to avoid making the parents feel guilty; to this effect, the staff explained their own limitations and failures with these children to their parents. Semiweekly hospitalization from Monday to Thursday appears to be the most advantageous solution in order to work intensively with the children, allow their parents time to recuperate, and ensure participation of parents at the weekly meetings. About thirty children have been treated in this fashion

over the past four years with variable success: one-third with good results, one-third with average results, one-third with poor results. At present, hospitalization for one day or for several hours at a specialized kindergarten outside of the hospital allows for treatment of a larger variety of psychiatric indications
SN - 0222-9617
AD - (Rufo, Dubois, Staub, Soulayrol) Serv. Pedopsychiat. Chateau Guis, Hop. Ste-Marguerite, 13009 Marseille France
ER -

TY - JOUR
ID - 4020
T1 - Plaster drop-out casts as a dynamic means to reduce muscle contracture. A case report
A1 - Cherry,D.B.
A1 - Weigand,G.M.
Y1 - 1981///
N1 - 1982015885
English
Journal: Article
KW - EMBASE
KW - Case Report
KW - central nervous system
KW - head injury
KW - joint
KW - joint contracture
KW - Muscle
KW - physiotherapy
KW - plaster cast
KW - school child
KW - spasticity
KW - Therapy
RP - NOT IN FILE
SP - 1601
EP - 1603
JF - Physical Therapy
JA - Phys Ther
VL - 61
IS - 11
CY - United States
N2 - Physical therapy for reducing muscle contracture, whether by passive stretch or active exercise, is often undermined by the joint position usually maintained outside the time of therapy. Any maintained joint position may be a serious problem, especially in the presence of spasticity, for example, upper extremity flexor pattern after a cerebrovascular accident or the total flexor or extensor responses of the limbs following head trauma or CNS infection. Plaster of Paris splinting that maintains the joint at the maximum comfortable range and allows further motion out of the contracted position can be effective in preventing further loss of range of motion and even in gaining additional range of motion. Such casts can be made in physical therapy with easily obtained materials. This case report illustrates this technique
SN - 0031-9023
AD - (Cherry, Weigand) Dept. Hlth Sci., Cleveland State Univ., Cleveland, OH 44115 United States
ER -

TY - JOUR
ID - 4021
T1 - Social paediatric practice. Problems and working methods
A1 - Merrick,J.
Y1 - 1981///
N1 - 1982013229
Danish
Journal

KW - EMBASE
KW - Family
KW - Infant
KW - Pediatrics
KW - preschool child
KW - school child
KW - Social Work
KW - Therapy
RP - NOT IN FILE
SP - 2466
EP - 2469
JF - Ugeskrift for Laeger
JA - Ugeskr.Laeger
VL - 143
IS - 38
CY - Denmark
N2 - The object of this investigation was to describe the clientele and working methods in a clinic for children in Copenhagen. During the period 1.1.1980-31.12.1980, the author registered consecutively all of the contacts with the children and families for whom he was responsible. This is thus registration of the work of one doctor and must be regarded as a momentary glimpse of social paediatric practice. A total of 97 children and their families were contacted (60% boys and 40% girls). The contact is described as contact at the outpatient clinic, activity outside the clinic and telephone contact. The time consumed is assessed and it is noted that social medical work is extremely time-consuming. The diagnoses and treatment emphasize the importance of prophylactic work and the requirement of close, current and frequently prolonged contact with the families
SN - 0041-5782
AD - (Merrick) Soc. Paed. Ambulat., Rigshosp., Kobenhavn Denmark
ER -

TY - JOUR
ID - 4022
T1 - Nonhandicapped adolescents teaching playground skills to their mentally retarded peers: Toward a less restrictive middle school environment
A1 - Donder,D.
A1 - Nietupski,J.
Y1 - 1981///
N1 - 1982090968
English
Journal
KW - EMBASE
KW - Adolescent
KW - Behavior
KW - Case Report
KW - central nervous system
KW - mental deficiency
KW - normal human
KW - Recreation
KW - school child
KW - Therapy
RP - NOT IN FILE
SP - 270
EP - 276
JF - Education and the Training of the Mentally Retarded
VL - 16
IS - 4
CY - United States
N2 - Nonhandicapped students served as playground tutors for three students in a class for the moderately retarded at a regular middle school. The nonhandicapped volunteers taught age-appropriate playground

recreation skills to their retarded peers in an effort to improve the playground behavior of the three retarded students and to facilitate positive interactions between retarded and nonhandicapped students. Results indicated substantial improvement in the percentage of appropriate playground behavior and in the percentage of contact between the retarded and nonhandicapped students. Follow-up probes indicated that, while the percentages of appropriate playground behavior and contact with nonhandicapped peers had decreased, they were considerably higher than during baseline
AD - (Donder, Nietupski) Dept. Spec. Educ., Univ. Illinois, Urbana, IL United States
ER -

TY - JOUR
ID - 4023
T1 - Collapsible washing lines: A strangulation hazard
A1 - Roberton,D.
A1 - Rookwood,K.
A1 - Rutter,N.
Y1 - 1981///
N1 - 1981143512
English
Journal: Article
KW - EMBASE
KW - Case Report
KW - injury
KW - preschool child
KW - strangulation
KW - Therapy
RP - NOT IN FILE
SP - 1664
JF - British Medical Journal
JA - Br Med J
VL - 282
IS - 6277
CY - United Kingdom
N2 - An 18-month-old boy was admitted to hospital after being strangled accidentally in the dependent loops of a rotary washing line. The line had been left upright but its radial arms had been folded down. A few minutes after he had gone to play outside his mother had found him kneeling by the line with three strands of cord wound tightly around his neck. He was unconscious and was not making any respiratory effort; peripheral pulses were not detectable. Initial resuscitation was performed by ambulance staff. On arrival at hospital he was breathing spontaneously and had a normal cardiac rhythm. He had widely dilated pupils, was hypertonic, and had intermittent clonic convulsions. He was given oxygen, and metabolic acidosis was corrected. Fluids were restricted and dexamethasone was given for 24 hours. He recovered rapidly and was discharged two days after admission, having suffered no permanent neurological damage. While playing with the loops of the clothes line he had presumably become entangled and had been asphyxiated during his struggles to free himself
SN - 0959-8146
AD - (Roberton, Rookwood, Rutter) Dept. Child Hlth, Med. Sch., Queen's Med. Cent., Nottingham NG7 2UH United Kingdom
ER -

TY - JOUR
ID - 4024
T1 - The validity of role play tests for assessing social skills in children
A1 - Van,Hasselt,V
A1 - Hersen,M.
A1 - Bellack,A.S.
Y1 - 1981///
N1 - 1981166129
English
Journal

KW - EMBASE
KW - major clinical study
KW - Role Playing
KW - school child
KW - social adaptation
RP - NOT IN FILE
SP - 202
EP - 216
JF - Behavior Therapy
VL - 12
IS - 2
CY - United States
N2 - A correlational study was conducted in an effort to examine the validity and reliability of role play tests for assessing social skills in children. Twenty-two male and twenty female elementary school children, ranging in age from 8-10 years, responded to role play scenarios involving social interactions. In addition to the role play procedure, a number of other assessment strategies were utilized: observations of behavior in naturalistic settings, sociometric ratings, and teachers' ratings. Results did not support the validity of the role play test. Correlations between role play performance and criterion measures were generally quite low. Test-retest reliability of the role play tests also was unacceptable. Further, results indicated that while several significant relationships were found between children's sociometric scores and teachers' ratings of interpersonal behavior, little association was found between these measures and global ratings of friendliness in role play and in vivo situations. Results are discussed in terms of the validity and utility of role play tests for children, the relationship of these data to previous research in social skills assessment, and the heuristic value of examining research concerning children's interpersonal functioning from developmental and social psychological perspectives
SN - 0005-7894
AD - (Van Hasselt, Hersen, Bellack) Dept. Psychiat., West. Psychiat. Inst. Clin., Univ. Pittsburgh Sch. Med., Pittsburgh, Pa. 15261 United States
ER -

TY - JOUR
ID - 4025
T1 - Correlations between items of the fitness-performance test of the Canadian Association for Health, Physical Education and Recreation
A1 - Massicotte,D.
A1 - Gauthier,R.
A1 - Quinney,A.
A1 - Conger,P.
Y1 - 1981///
N1 - 1981164486
French
Journal: Article
KW - EMBASE
KW - Adolescent
KW - athlete
KW - Exercise
KW - fitness
KW - human cell
KW - Methodology
KW - musculoskeletal system
KW - normal human
KW - Performance
KW - preschool child
KW - school child
KW - sport
RP - NOT IN FILE
SP - 11
EP - 15

JF - Canadian Journal of Applied Sport Sciences
VL - 6
IS - 1
CY - Canada
N2 - The Fitness-Performance Tests developed in 1965 by the Canadian Association for Health, Physical Education and Recreation have been criticized with regard to the validity of the 300 yard run as an endurance measure and with regard to the redundance of some items involving lower limb performance. The purposes of this study were to identify a more appropriate endurance performance test item and to analyze the inter-dependence of the items which utilize leg power in their performance. A sample of 9000 young Canadians aged 6 to 17 years, was randomly selected from the 10 provinces and the 2 territories. Correlation coefficients were calculated between: standing long jump, the 50 m. run, and the endurance run (800 m. for 6-9 years, 1600 m. for 10-12 years and 2400 m. for 13-17 years). Among the different age and sex groups, the correlations between the endurance run and the 50 m. run varied from 0.40 to 0.49. The correlations between standing long jump, the agility run (4 x 10 m.) and the 50 m. run were from 0.69 to 0.83 for all the boys and girls. The correlations between these three items are even lower when performances are analyzed by age and sex groups. The highest variations are mainly in the preadolescent period for both sexes
AD - (Massicotte, Gauthier, Quinney, Conger) Dept. Kinanthropol., Univ. Quebec, Montreal Canada
ER -

TY - JOUR
ID - 4026
T1 - Social play of withdrawn children. A study of the effects of teacher-mediated peer feedback
A1 - Ragland,E.U.
A1 - Kerr,M.M.
A1 - Strain,P.S.
Y1 - 1981///
N1 - 1981247749
English
Journal
KW - EMBASE
KW - behavior disorder
KW - Case Report
KW - central nervous system
KW - play
KW - school child
KW - Social Behavior
KW - Therapy
RP - NOT IN FILE
SP - 347
EP - 359
JF - Behavior Modification
JA - Behav Modif
VL - 5
IS - 3
CY - United States
N2 - The purpose of this study was to test the effectiveness of a teacher-mediated, peer feedback intervention package on the social interactions of withdrawn children. Three 10-year-old boys who were enrolled in a class for children with learning and behavior problems were the target subjects. Their class peers were eight boys who exhibited severe academic problems. During the initial baseline condition no attempt was made to influence the social behavior of the subjects in the daily 25-minute recess periods. During intervention, all target subjects were assigned the goal, 'Play more with friends during recess.' With the teacher's direction, the boys gave one another feedback as to whether the goal had been met the day before. This procedure resulted in an immediate and sustained increase in the positive social behaviors of the three target subjects. In subsequent return to baseline and intervention conditions, prior levels of subject behaviors were replicated
SN - 0145-4455
AD - (Ragland, Kerr, Strain) West. Psychiat. Inst. Clin., Pittsburgh, PA 15261 United States
ER -

TY - JOUR
ID - 4027
T1 - Severe sport-injuries in childhood
A1 - Riemer,R.
Y1 - 1981///
N1 - 1981234962
German
Journal: Article
KW - EMBASE
KW - Adolescent
KW - Bone
KW - fracture
KW - geographic distribution
KW - German Federal Republic
KW - injury
KW - joint
KW - major clinical study
KW - musculoskeletal system
KW - physiotherapy
KW - preschool child
KW - school child
KW - short survey
KW - sport injury
KW - Therapy
RP - NOT IN FILE
SP - 405
EP - 412
JF - Unfallheilkunde/Traumatology
VL - 84
IS - 10
CY - Germany
N2 - An analysis of 139 severe sport-injuries in children shows that frequently such accidents occur during unsupervised activities, outside school or club hours. Most cases involve injured limbs. Principles of fracture-treatment in children are demonstrated. Special attention is paid to lesions of the joints and the importance of active physiotherapy is stressed
AD - (Riemer) Chir. Univ. Klin., Bergmansheil, Bochum Germany
ER -

TY - JOUR
ID - 4028
T1 - Recreational activities in community health care
A1 - Shaw,I.
Y1 - 1980///
N1 - 1982021036
English
Journal
KW - EMBASE
KW - age
KW - Child
KW - disabled person
KW - Health Care
KW - industrial medicine
KW - legal aspect
KW - Prevention
KW - psychological aspect
KW - Recreation

KW - Therapy
RP - NOT IN FILE
SP - 135
EP - 141
JF - Australian Journal of Physiotherapy
VL - 26
IS - 4
CY - Australia
N2 - Recreation is a significant element of preventive health care, used by community health centres as an aid to rehabilitation, moderation of stress and maintenance of health. Although the amount of leisure available to the people is increasing, many are unable to take full advantage of it due to inadequate recreational education or other causes. Community health centres have an educative responsibility in this regard especially towards the unemployed. These centres organize therapeutic and socializing groups for patients in need. Examples are given. Recreational activities are also useful in the relief of stress, during breaks in production in work-places. The principles of safety need to be taught as an essential part of recreational education and the fitness of participants needs to be ensured by screening school children. The planning and erection of playground equipment needs supervision by competent professionals who exist in the community health centres. The conclusion recommends that safety check lists be available for all normal recreations and that everyone should receive adequate education to enable successful participation in recreational activities
SN - 0004-9514
AD - (Shaw) Caulfield Commun. Care Cent., Melbourne Australia
ER -

TY - JOUR
ID - 4029
T1 - Transfer of persistence across behaviors
A1 - Eisenberger,R.
A1 - Heerdt,W.A.
A1 - Hamdi,M.
A1 - Zimet,S.
A1 - Bruckmeir,G.
Y1 - 1979///
N1 - 528917
English
Journal: Article
KW - EMBASE
KW - Adult
KW - article
KW - Child
KW - comparative study
KW - depression
KW - Exercise
KW - Frustration
KW - habituation
KW - handwriting
KW - Human
KW - instrumental conditioning
KW - Learning
KW - learning disorder
KW - Mathematics
KW - Motivation
KW - psychological aspect
KW - Reinforcement
RP - NOT IN FILE
SP - 522
EP - 530
JF - Journal of experimental psychology

VL - Human learning and memory. 5
IS - 5
CY - United States
N2 - Two experiments tested the applicability to human beings of findings with animals that the number of performances required for the reinforcement of one behavior affects the subsequent effort expended in other instrumental behaviors. In the first experiment, adult depressed psychiatric patients worked on a sorting task for the approval of a staff psychologist. The time spent and the work completed were increased by prior approval from a ward attendant for each completion of several custodial tasks, as compared to the ward attendant's approval for each completion of a single task or a no-pretreatment control condition. In the second experiment, preadolescent learning-disabled students who were required to read and spell correctly a greater number of words per reward token later spent more time and completed more work for reward tokens in mathematics, and handwriting. Two alternative interpretations of these results are evaluated: (a) The degree of accustomed effort per reinforcer becomes a learned component of behavior, or (b) high effort increases the habituation of frustration-produced disruptive responses. The results suggest that individual differences in general persistence may arise, in part, from an accumulation of effort training in the natural environment
SN - 0096-1515
ER -

TY - JOUR
ID - 4030
T1 - Age differences in sweating during muscular exercise
A1 - Araki,T.
A1 - Toda,Y.
A1 - Matsushita,K.
A1 - Tsujino,A.
Y1 - 1979///
N1 - 1980186901
English
Journal
KW - EMBASE
KW - Adolescent
KW - age
KW - Child
KW - Exercise
KW - human cell
KW - juvenile
KW - normal human
KW - preschool child
KW - school child
KW - Sweating
RP - NOT IN FILE
SP - 239
EP - 248
JF - Japanese Journal of Physical Fitness and Sports Medicine
VL - 28
IS - 3
CY - Japan
N2 - To study the age difference in sweating during muscular exercise, two series of experiments were conducted under constant climatic conditions (29 + 1degreeC, DB, 60 + 5% RH, 0.45 + 0.05 m/sec air flow). In series A, 7 to 20 years old male subjects undertook 5-minute running or pedalling of a bicycle ergometer in various seasons. In this series of experiments, pectoral sweat volume, sweat chloride concentration, rectal and mean skin temperatures were in general determined every 5 minutes and, when necessary, the total body sweat volume was calculated from the body weight loss. In series B. the age difference in the sweating in relation to physical training was studied. Subjects, 3 to 20 years old received experimental physical training of 5-minute or 500m-running. Before and after the training, a work load or 3- or 5-minute outdoor running was assigned to them. Furthermore, before and after the training, 10 an 11 years old subjects were given a fixed mechanical work rate on a bicycle ergometer. In these experiments, most of the parameters described above were measured.

In series A, age differences in sweating during exercise were noticed to be dependent on the intensity of work load between pre- and post-adolescents. When the work load was heavy enough to cause a rapid increase in rectal temperature, the sweat volume became significantly less, the mean skin temperature was far higher, and the sweat chloride concentration was remarkably smaller in the pre-adolescent subjects than in the post-adolescent ones. In series B, an age difference in the effect of physical training was also found on sweating during exercise. The effect of physical training on sweating in the pre-adolescent individuals was generally less significant than in adults and sometimes showed a different pattern from that of adults. From these results, it can be concluded that sweating in the pre-adolescents is less adaptive, particularly to continuous severe exercise and that physical training is less effective in them than in the post-adolescents
SN - 0039-906X
AD - (Araki, Toda, Matsushita, Tsujino) Dept. Hyg., Kobe Univ. Sch. Med., Kobe Japan
ER -

TY - JOUR
ID - 4031
T1 - Telemetered heart rate as a psychophysiological correlate of mother-child interaction
A1 - Hong,K.M.
A1 - Bowden,D.M.
A1 - Kogan,K.L.
Y1 - 1979///
N1 - 1980216765
English
Journal: Article
KW - EMBASE
KW - Affect
KW - autonomic nervous system
KW - Behavior
KW - Case Report
KW - heart
KW - Heart Rate
KW - mother child relation
KW - normal human
KW - preschool child
RP - NOT IN FILE
SP - 103
EP - 118
JF - Journal of the American Academy of Child Psychiatry
VL - 18
IS - 1
CY - United States
N2 - Heart rate was telemetered from 6 preschool children during play sessions with their mothers. Their behavioural interactions was simultaneously recorded on videotape and rated on three dimensions of interaction status submission-dominance), affect (hostility-warmth), and degree of involvement; 100 specific behaviors were coded in consecutive 4 second epochs. In exploring the relationship between heart rate and the behavioral measures, we applied two kinds of analysis - state analysis and transitions analysis. The usefulness or recording heart rate in a naturalistic setting was demonstrated by replicating the finding from more rigidly defined experiments that intense looking at an object is associated with cardiac deceleration. New findings were that submissive status and warm affect of the child and dominant status and warm affect of the mother weere associated with low heart rate in the child and that the onset of smiling was associated with cardiac deceleration in most situations. The study demonstrates the feasibility and some of the potential uses of continuously telemetered heart rate for analyzing interactional and physiological variables in a naturalistic setting
SN - 0002-7138
AD - (Hong, Bowden, Kogan) Dept. Psychiat., Univ. Minnesota, Minn. United States
ER -

TY - JOUR
ID - 4032

T1 - Family origins of violent delinquents
A1 - Fagan,J.
A1 - Wexler,S.
Y1 - 1987///
N1 - 1987234708
English
Journal
KW - EMBASE
KW - Adult
KW - central nervous system
KW - Child
KW - child parent relation
KW - Crime
KW - criminology
KW - Epidemiology
KW - family study
KW - family violence
KW - forensic medicine
KW - Human
KW - Juvenile Delinquency
KW - legal aspect
KW - preliminary communication
KW - psychological aspect
KW - social aspect
KW - social psychology
KW - Violence
RP - NOT IN FILE
SP - 643
EP - 669
JF - Criminology
VL - 25
IS - 3
CY - United States
N2 - Family is central to contemporary theories of delinquent and violent behavior. Yet, the processes by which families shape violent behavior in their children are not well understood. In the past, structural views posited that a weak family exposed a child to the evils of the street. More recently, functionalists have suggested that the family plays an active role in socializing youths to violent behaviors through supervision and discipline practices and modeling and reinforcement of antisocial behaviors. Integrated theories presume that socially disorganized families weaken children's conventional bonds and attachments, leading to associations with delinquent peers and in turn antisocial behavior. However, the influence of the family as a socializing environment may shift over time, and some suggest that its influence is overshadowed during adolescence by that of other social domains - schools, neighborhoods, peers, and work. This study describes the family processes and environments of (n = 98) chronically violent delinquents. Interviews with youths and their mothers assessed family social process and environments and the social domains and institutions with which they interact. Analyses of youth reports of family environments and processes yield three family types: 'interactionist' families exhibiting a high degree of internal interaction and bonding; 'hierarchical' families characterized by parental dominance and the presence of family bond and interaction patterns; and 'antisocial' families marked by criminality and family violence. Family variables have weaker explanatory power than do other social influences on violent delinquency. The relative contributions of family supervision practices and school environment varied by crime type. Social influences outside the family appear as stronger contributors to delinquency and violence during adolescence, regardless of early childhood experiences. The results underschore the importance of integrating social policies regarding family, crime, and neighborhood
SN - 0011-1384
AD - (Fagan, Wexler) School of Social Welfare, University of California, Berkeley, CA United States
ER -

TY - JOUR

ID - 4033
T1 - Acute suppurative thyroiditis in pediatric patients
A1 - Rich,E.J.
A1 - Mendelman,P.M.
Y1 - 1987///
N1 - 1987218553
English
Journal: Review
KW - EMBASE
KW - Bacteroides
KW - Child
KW - endocrine system
KW - Etiology
KW - Human
KW - Pediatrics
KW - Peptococcus
KW - Streptococcus
KW - Therapy
KW - thyroiditis
RP - NOT IN FILE
SP - 936
EP - 940
JF - Pediatric Infectious Disease Journal
JA - Pediatr Infect Dis J
VL - 6
IS - 10
CY - United States
N2 - Acute suppurative thyroiditis is uncommon in children. It usually follows an upper respiratory tract infection and presents with fever, dysphagia, cough and a tender left anterior neck mass. Dyspnea is less common but when present it may lead to complete upper respiratory tract obstruction. The radiographic diagnosis can be confirmed by computed tomography or ultrasonography, precluding the use of thyroid scan and the unnecessary exposure to radionuclide. Thyroid function tests do not appear to be indicated (outside the neonatal period) inasmuch as nearly all patients are euthyroid. The bacteria involved include the normal oropharyngeal microflora, and broad spectrum antibiotic therapy to include anaerobic coverage should be utilized initially. Definitive management requires incisions and drainage. A left pyriform sinus fistula communicating with the perithyroid space is a common finding and probably plays a role in the pathogenesis of AST. A barium esophagogram should be performed, and if present the fistula track should be extirpated
SN - 0891-3668
AD - (Rich, Mendelman) Division of Infectious Diseases, Children's Hospital and Medical Center, Seattle, WA 98105 United States
ER -

TY - JOUR
ID - 4034
T1 - Clinical evaluation of cefuzonam in pediatrics
A1 - Haruta,T.
A1 - Yamamoto,H.
A1 - Okura,K.
Y1 - 1987///
N1 - 1987137211
Japanese
Journal: Article
KW - EMBASE
KW - adverse drug reaction
KW - alanine aminotransferase blood level
KW - aspartate aminotransferase blood level
KW - bacterial infection

KW - Child
KW - clinical article
KW - drug blood level
KW - drug efficacy
KW - Drug Therapy
KW - efficacy
KW - Human
KW - intoxication
KW - intravenous drug administration
KW - liver toxicity
KW - Pediatrics
KW - pharmacokinetics
KW - priority journal
KW - Prothrombin Time
KW - respiratory system
KW - respiratory tract infection
KW - Therapy
KW - urinary tract
KW - urinary tract infection
KW - cefuzonam
KW - l 105
KW - unclassified drug
RP - NOT IN FILE
SP - 439
EP - 447
JF - Japanese Journal of Antibiotics
VL - 40
IS - 2
CY - Japan
N2 - Cefuzonam (L-105, CZON), a new injectable cephalosporin, was used in 12 pediatric patients with infections. The 12 cases included 3 cases of tonsillitis (pathogen: Haemophilus parainfluenzae in 1 case, Haemophilus influenzae in 2 cases), 4 cases of pneumonia (Staphylococcus aureus in 1 case, pathogen unknown in 3 cases), 2 cases of nephropyelitis (Escherichia coli in 2 cases), 1 case of purulent lymphadenitis (pathogen unknown), 1 case of purulent thyroiditis (mixed infection of Streptococcus milleri, Haemophilus aphrophilus and anaerobes), and 1 case of vulvar abscess (E. coli). Dose levels of CZON were 42.9-93.3 mg/kg/day divided into 3 or 4 times and the drug was intravenously injected for 6 to 12 days. Clinical efficacies were excellent in 4 cases, good in 5 cases, and poor in 3 cases, with an efficacy rate of 75.0%. The 3 cases with poor efficacy consisted of 1 case each of pneumonia complicated with chronic granulomatosis, purulent thyroiditis associated with piriform recess fistula, and purulent lymphadenitis of the armpit after surgical operation of congenital heart disease. In the first 2 cases satisfactory efficacy was not obtained by chemotherapy alone, and complete cure was seen after surgical operation. Side effects were not observed clinically. One case each of slight prolongation of prothrombin time and transient elevations of GOT and GPT values was noted but no severe abnormalities were found in laboratory tests. The above results are reasonable from the points of view of the broad spectrum, potent antibacterial activity, and pharmacokinetics of this compound; thus, CZON appears to be a useful new antibiotic in the pediatric field. The recommended dosage for general pediatric infections is about 20 mg/kg at a time, 3 or 4 times, by which the expected efficacy may be obtainable
SN - 0368-2781
AD - (Haruta, Yamamoto, Okura) Department of Pediatrics, Kobe Central Municipal Hospital, Kobe Japan
ER -

TY - JOUR
ID - 4035
T1 - Increasing recreational behavior in mentally retarded childen
A1 - Katz,R.C.
A1 - Singh,N.N.
Y1 - 1986///

N1 - 1987050780
English
Journal: Article
KW - EMBASE
KW - Behavior
KW - central nervous system
KW - Child
KW - clinical article
KW - Human
KW - mental deficiency
KW - psychological aspect
KW - Recreation
KW - Therapy
RP - NOT IN FILE
SP - 508
EP - 519
JF - Behavior Modification
JA - Behav Modif
VL - 10
IS - 4
CY - United Kingdom
N2 - Mentally retarded children are frequently observed either to be inactive or to engage in stereotypic behaviors and isolate play. In this study, sign posting, pictorial prompts, and feedback were used to increase the recreational behaviors of 45 moderately and severely mentally retarded children. Treatment was introduced sequentially in a multiple-baseline fashion for ballplay and jungle gym activities across two school recess periods. Results showed large increases in the percentage of children participating in these activities that corresponded with the introduction of treatment. Similarly, the average amount of time the children played was roughly tripled. These gains were maintained over a 12-week follow-up period during which the pictorial prompts and feedback were gradually faded. The results demonstrate the efficacy of a simple, inexpensive, and practical way of generating higher levels of recreational play in large numbers of mentally retarded children
SN - 0145-4455
AD - (Katz, Singh) Psychology Department, University of the Pacific, Stockton, CA 95211 United States
ER -

TY - JOUR
ID - 4036
T1 - Double-blind study of the preventive action of sodium cromoglycate on the exercise induced bronchospasm in children
A1 - Viallat,J.R.
A1 - Razzouk,H.
A1 - Edmond,F.
Y1 - 1986///
N1 - 1987031291
French
Journal
KW - EMBASE
KW - Asthma
KW - bronchospasm
KW - bronchus hyperreactivity
KW - Child
KW - clinical article
KW - clinical trial
KW - controlled study
KW - drug comparison
KW - drug efficacy
KW - Drug Therapy
KW - Exercise

KW - Human
KW - Methodology
KW - oral drug administration
KW - Prevention
KW - respiratory system
KW - Therapy
KW - cromoglycate disodium
KW - placebo
RP - NOT IN FILE
SP - 121
EP - 124
JF - Revue Francaise d'Allergologie et d'Immunologie Clinique
VL - 26
IS - 3
CY - France
N2 - A double-blind study has been carried out in 40 allergic asthmatic children, average age 12 years, in order to evaluate the protective action of sodium cromoglycate, taken punctually or regularly for one month, on asthma induced exercise. RVA were measured 45 min. before an outdoors race of 8 min. then immediately, 3, 10, and 25 min. after exertion. The isolated intake of one placebo before exercise does not reduce the frequency of the crises, while cromoglycate reduces this frequency to 55 per cent (p< 0.02). After one month of treatment, only 5 per cent of the patients under active therapy had an asthma attack after exercise, versus 75 per cent of the patients under placebo (p< 0.001). Therefore Sodium cromoglycate prevents short term exercise induced asthma. Its regular administration increases its efficacy, possibly by decreasing bronchial hyperactivity
SN - 0335-7457
AD - (Viallat, Razzouk, Edmond) Service de Pneumologie, Hopital Michel-Levy, 13006 Marseille France
ER -

TY - JOUR
ID - 4037
T1 - Salmonella contamination of soil in children's play areas on Guam
A1 - Haddock,R.L.
A1 - Nocon,F.A.
Y1 - 1986///
N1 - 1987031101
English
Journal
KW - EMBASE
KW - Child
KW - dog
KW - Guam
KW - Human
KW - Hygiene
KW - playground
KW - Prevention
KW - Salmonella
KW - Soil
RP - NOT IN FILE
SP - 158
EP - 160
JF - Journal of Environmental Health
JA - J Environ Health
VL - 49
IS - 3
CY - United States
N2 - Soil samples from children's play areas on the Island of Guam were examined for the presence of Salmonella bacteria. Twenty four (30.4%) of 79 samples were positive. Test sites with high soil positivity rates tended to be in villages with higher incidence rates of human salmonellosis and higher rates of stray animals

killed on public roads. It was concluded that there was widespread contamination of Guam's environment with Salmonella bacteria and that stray dogs and other unconfined animals could contribute to the unusually high incidence of salmonellosis reported on the island
SN - 0022-0892
AD - (Haddock, Nocon) Department of Public Health and Social Services, P.O. Box 2816, Agana, Guam 96910 Guam
ER -

TY - JOUR
ID - 4038
T1 - Testing the validity of the Griffin/Keogh model for movement confidence by analyzing self-report playground involvement decisions of elementary school children
A1 - Crawford,M.E.
A1 - Griffin,N.S.
Y1 - 1986///
N1 - 1986117603
English
Journal
KW - EMBASE
KW - Diagnosis
KW - Education
KW - Human
KW - human experiment
KW - Motivation
KW - Motor Performance
KW - musculoskeletal system
KW - normal human
KW - psychological aspect
KW - school child
KW - social aspect
RP - NOT IN FILE
SP - 8
EP - 15
JF - Research Quarterly for Exercise and Sport
VL - 57
IS - 1
CY - United States
N2 - In this study, the interaction of such factors as competence, potential for enjoyment, and potential for harm as postulated in the Model for Movement Confidence was studied by analyzing responses of 250 fifth-grade children in the Omaha Public schools to a Playground Movement Confidence Inventory (PMCI). The PMCI was designed to assess movement confidence and the bases for performance decisions. Multiple discriminant analyses and analytical cross-validation were used to determine an index of discriminatory power, stability of the instrument, and a validity coefficient for scale classification power. All three model factors were found to influence the confidence of children in performing the playground tasks in the inventory. The PMCI successfully classified 85.16% of total cases representing a .5726 proportional reduction in error over chance. The factor analysis model accounted for 54% of the total variance with 90% of this variance explained by the competency subscale. The enjoyment and harm subscales combined to account for the remaining 10%. Two categories of harm variables were also depicted by the factor analysis in this study. Findings of this study represent a significant step in the validation and elaboration of the Model for Movement Confidence. Identity, separability, and measurability of the factors postulated in the model were demonstrated and the probably multivariate nature of the harm, factor identified. Specific response pattern profiles of low and high confidence groups as a priori predictions of the movement confidence model were obtained. The theoretical formulations of the model sustained as factors of enjoyment and harm as well as competence, were found to modify confidence perceptions of tasks. In this study the discriminating variables operated with a high degree of task specificity
SN - 0270-1367
AD - (Crawford, Griffin) University of Missouri, Columbia, MO United States
ER -

TY - JOUR
ID - 4039
T1 - The development of independence in chronically ill adolescents
A1 - Sinnema,G.
Y1 - 1986///
N1 - 1986250830
English
Journal
KW - EMBASE
KW - adolescence
KW - Adolescent
KW - Asthma
KW - Child
KW - Child Development
KW - Chronic Disease
KW - congenital disorder
KW - cystic fibrosis
KW - fatality
KW - heredity
KW - Human
KW - independence
KW - major clinical study
KW - respiratory system
RP - NOT IN FILE
SP - 1
EP - 14
JF - International Journal of Adolescent Medicine and Health
VL - 2
IS - 1
CY - United Kingdom
N2 - The literature often suggests that chronically ill adolescents show a delay in the development of independence, but clear-cut evidence is lacking. Cystic fibrosis (CF) is an example of a chronic illness with marked effects on growth and development. CF is a lethal genetic disease with mainly pulmonary and gastrointestinal problems; median life expectancy is about 18 years. In the authors' study 3 major psychological elements of CF are considered: its chronic nature, its fatal outcome, and the visibility of the handicap (retarded growth). In order to study the influence of these variables on adolescent independence, 3 reference groups have been chosen besides the CF-group (n = 39): adolescents with asthma (chronic, but not fatal; n = 26); healthy, but small adolescents (n = 45), and normal healthy controls (n = 70). Eight forms of independence were measured by an extensive questionnaire. Some of the main results: At younger ages (13-14) CF-adolescents show a delay in 'dating and mating', but in general they do not have less social activities outside the home than their healthy peers. At ages 15-17 CF-adolescents do have a smaller social range. Younger CF-adolescents are less independent in handling social conflicts. At ages 13-14 adolescents with CF or asthma bear less responsibility for their own body hygiene. Surprisingly, CF- and asthma-patients feel just as responsible for living according to health rules as their healthy peers. Also, there are no differences between the groups regarding the formation of ideals and the development of own ideas in politics, religion and ethics. In future research it will be necessary to avoid general labels such as 'immature' or 'dependent', and to differentiate further between different types of independence
SN - 0334-0139
AD - (Sinnema) University Children's Hospital, Het Wilhelmina Kinderziekenhuis, 3512 LK Utrecht Netherlands
ER -

TY - JOUR
ID - 4040
T1 - Children's accidents with selected types of playground equipment, registered in Odense Hospital in 1982
A1 - Hansen,A.J.

A1 - Kruse,T.
Y1 - 1985///
N1 - 1986040155
Danish
Journal
KW - EMBASE
KW - accident
KW - Adolescent
KW - Case Report
KW - Child
KW - childhood
KW - Denmark
KW - Diagnosis
KW - geographic distribution
KW - Human
KW - Infant
KW - injury
KW - playground
KW - preschool child
KW - school child
RP - NOT IN FILE
SP - 4230
EP - 4235
JF - Ugeskrift for Laeger
JA - Ugeskr.Laeger
VL - 147
IS - 51
CY - Denmark
N2 - As yet, there are no standards in Denmark for design or placing of playground equipment. An analysis of all contacts with the Casualty Department in Odense Hospital in 1982, where the injured were in the age-group 0-14 years and when the injury had occurred during play with swings, slides, seesaws, sandpits or climbing frames gave the following results: 279 children were injured and 33 of these were hospitalized; 24% of the children sustained fractures; the majority of the injured children were aged 5-9 years; the majority of the injuries occurred with swings and slides and the most serious accidents resulted from falls from climbing frames. If the distribution of accidents was correlated for the presence of the individual types of playground equipment, particular risks are found to be associated when playing with: climbing frames in schools, seesaws in public playgrounds and slides regardless of where these are. When the results are compared with a corresponding investigation carried out in Arhus in 1979-80, the pattern and the frequencies of accidents are found to be practically identical. This confirmation of the findings from Arhus should instigate efforts to establish Danish standards for playground equipment and rules for design of playgrounds including minimum norms for the shock-absorbing nature of the ground or flooring
SN - 0041-5782
AD - (Hansen, Kruse) Ulykkes Analyse Gruppen, Institut for Sundhedsokonomi og Sygdomsforebyggelse, Odense Universitet, DK-5000 Odense C Denmark
ER -

TY - JOUR
ID - 4041
T1 - Periodicity as a principle of organization in behavior
A1 - Akyurek,A.
Y1 - 1985///
N1 - 1986028210
English
Journal: Article
KW - EMBASE
KW - central nervous system
KW - Child

KW - clinical article
KW - Human
KW - infantile autism
RP - NOT IN FILE
SP - 491
EP - 506
JF - Psychological Reports
JA - Psychol Rep
VL - 57
IS - 2
CY - United States
N2 - A proposal is outlined to provide a quantitative descriptive research frame for analyzing the organization of spontaneous behavior in autistic children within and across natural settings such as home, school, daycare centre, and playground. A number of research questions are formulated and appropriate analyses are suggested. In particular the project aimed to detect periodicities in behavior, guided probably by 'clock-like' processes as have been found in the field of biological rhythms. A database has to be created by observing and notating a sufficient number of samples of behavior drawn from various settings in which the autistic children happen to live. Contrived settings may also be used
SN - 0033-2941
AD - (Akyurek) Schaperstraat 9, 9722 LN Groningen United States
ER -

TY - JOUR
ID - 4042
T1 - Social consequences of hyperactivity in a children's treatment center
A1 - Schneider,B.H.
A1 - Ledingham,J.E.
A1 - Byrne,B.M.
Y1 - 1985///
N1 - 1986057268
English
Journal: Article
KW - EMBASE
KW - Aggression
KW - behavior disorder
KW - central nervous system
KW - Child
KW - clinical article
KW - Human
KW - hyperactivity
KW - psychological aspect
RP - NOT IN FILE
SP - 429
EP - 438
JF - Journal of Pediatric Psychology
JA - J Pediatr Psychol
VL - 10
IS - 4
CY - United States
N2 - The social behavior of hyperactive and nonhyperactive conduct-disordered children at a treatment center was measured by teacher rating, direct observation of recess play, and peer rating. Hyperactive subjects were rated by their peers as more aggressive and somewhat less likable than nonhyperactives, and they were more likely to be the target of others' aggression. Though rated as less likable, hyperactives spent less time than nonhyperactives in solitary play. Hyperactives also spent less time observing the interactions of other children. Additional analyses indicated that the peer relations problems of hyperactive children in treatment cannot be attributed exclusively to their aggressiveness. These findings are discussed in terms of their implications for treatment and for the theoretical conceptualization of hyperactivity

SN - 0146-8693
AD - (Schneider, Ledingham, Byrne) Child Study Center, School of Psychology, Ottawa, Ont. K1N 6N5 Canada
ER -

TY - JOUR
ID - 4043
T1 - School-based health clinics: A new approach to preventing adolescent pregnancy?
A1 - Dryfoos,J.
Y1 - 1985///
N1 - 1985229924
English
Journal: Article
KW - EMBASE
KW - Adolescent
KW - Adolescent Pregnancy
KW - Human
KW - Methodology
KW - organization and management
KW - outpatient department
KW - Pregnancy
KW - Prevention
KW - priority journal
KW - school child
KW - school health service
KW - short survey
RP - NOT IN FILE
SP - 70
EP - 75
JF - Family Planning Perspectives
VL - 17
IS - 2
CY - United States
N2 - In an increasing number U.S. communities, comprehensive health services - including family planning services - are being offered in clinics located in or near public health schools and junior high schools. Fourteen such programs operating in 32 schools are described here; they have caseloads ranging from about 500 to around 5,000 students per year and handle up to 20,000 patient visits annually. These clinics usually are staffed by teams consisting mainly of nurse practitioners, clinic aides and part-time physicians, as well as social workers, nutritionists and other professionals. Patients at school-based clinics generally are from low-income families, a reflection of the neighborhoods in which programs are located. Most school-based clinics serve only current students, but a few also serve former students. Most of these programs were originated by an individual from outside the school system. However, the cooperation and support of principals and teachers for student health services have been vital factors in gaining approval for in-school clinics. The programs also appear to enjoy the support of their local communities. However, their long-term funding remains uncertain. So far, private foundations have helped these programs become established, but they cannot be expected to provide continuing aid. Donations from schools, health providers and other groups have played an important part in keeping the cost of school-based programs low. Public funds, particularly from state departments of maternal and child health, social services and education, are being sought in order to expand existing programs to more schools and to start new clinics in other communities. School-based programs have been credited with improving students' health, lowering their birthrates, raising their levels of contraceptive use and improving their school attendance. However, there have been few reliable studies of these programs. Improved program evaluation will be useful in helping these clinics retain their financial and community support
SN - 0014-7354
ER -

TY - JOUR
ID - 4044

T1 - Ontogenesis of taurocholate transport by rat ileal brush border membrane vesicles
A1 - Barnard,J.A.
A1 - Ghishan,F.K.
A1 - Wilson,F.A.
Y1 - 1985///
N1 - 1985111011
English
Journal: Article
KW - EMBASE
KW - age
KW - animal cell
KW - Child
KW - digestive system
KW - intestine brush border
KW - large intestine
KW - membrane vesicle
KW - nonhuman
KW - ontogeny
KW - postnatal development
KW - priority journal
KW - rat
KW - taurocholic acid
RP - NOT IN FILE
SP - 869
EP - 873
JF - Journal of Clinical Investigation
VL - 75
IS - 3
CY - United States
N2 - Developmental aspects of taurocholate transport into ileal brush border membrane vesicles were studied in 2-wk-old (suckling), 3-wk-old (weanling), and 6-wk-old (adolescent) rats. Taurocholate uptake (picomoles per milligram protein) into brush border membrane vesicles prepared from 2-wk-old rats was similar under Na⁺ and K⁺ gradient conditions (outside greater than inside). By contrast, uptake in 3- and 6-wk-old rats was significantly enhanced at 20 s, and at 1, 2, and 5 min of incubation in the presence of a Na⁺ gradient when compared with a K⁺ gradient incubation (P < 0.05). Under isotope exchange conditions, a plot of active uptake velocity versus taurocholate concentration (0.10-1.0 mM) in 2-wk-old rat membrane vesicles was linear and approached the horizontal axis, suggesting the absence of active transport. However, similar plots in 3- and 6-wk-old rats described a rectangular hyperbola, indicating a Na⁺-dependent, saturable cotransport system. Woolf-Augustinsson-Hofstee plots of the uptake velocity versus concentration data from 3- and 6-wk-old rat brush border membrane vesicles yielded V(max) values that were not significantly different, 844 and 884 pmol uptake/mg protein per 120 s, respectively. The respective K(m) values were 0.59 and 0.66 mM taurocholate. The induction of an electrochemical diffusion potential by incubating K⁺-loaded vesicles with valinomycin did not significantly enhance taurocholate uptake in 2-, 3-, or 6-wk-old rat vesicle preparations. These data indicate that taurocholate transport into rat ileal brush border membrane vesicles is mediated by an electroneural, sodium-coupled, cotransport system that is incompletely developed in the 2-wk-old suckling rat but fully developed by the time of weaning at 3 wk of age
SN - 0021-9738
AD - (Barnard, Ghishan, Wilson) Department of Pediatrics, Division of Pediatric Gastroenterology and Nutrition, Vanderbilt University Medical Center, Nashville, TN 37232 United States
ER -

TY - JOUR
ID - 4045
T1 - Respiratory and heart rate patterns in infants destined to be victims of sudden infant death syndrome: Average rates and their variability measured over 24 hours
A1 - Wilson,A.J.

A1 - Stevens,V.
A1 - Franks,C.I.
Y1 - 1985///
N1 - 1985125946
English
Journal: Article
KW - EMBASE
KW - breathing
KW - Cardiovascular System
KW - Child
KW - Diagnosis
KW - Epidemiology
KW - heart
KW - Heart Rate
KW - Holter monitoring
KW - Human
KW - Infant
KW - major clinical study
KW - Methodology
KW - Mortality
KW - priority journal
KW - sudden infant death syndrome
RP - NOT IN FILE
SP - 497
EP - 501
JF - British Medical Journal
JA - Br Med J
VL - 290
IS - 6467
CY - United Kingdom
N2 - From a prospective study in which 24 hour recordings of the electrocardiogram and respiratory activity (abdominal wall movement) were made on a population of full term infants, 22 recordings were obtained from 16 infants who later were victims of the sudden infant death syndrome. The average heart rate, average heart rate variability, average breath to breath interval, and average breath to breath interval variability over the whole of each recording for the 22 recordings were compared with those from a control group of 324 infants selected at random from the rest of the population. No significance was found in the number of recordings from those infants who suffered the sudden infant death syndrome which lay outside the 5th-95th percentile range of the control group for the four variables studied. In a group comparison no difference was found between the sudden infant death syndrome group and the controls either in terms of the respiratory variables studied or in terms of the average heart rate variability. The results did, however, suggest that there may be a group difference in terms of the average instantaneous heart rate
SN - 0959-8146
AD - (Wilson, Stevens, Franks) Department of Medical Physics and Clinical Engineering, Royal Hallamshire Hospital, Sheffield S10 2JF United Kingdom
ER -

TY - JOUR
ID - 4046
T1 - Assessment of infant-caregiver interaction using cardiac, respiratory, and behavioral monitoring: Conceptual and technical issues in a new methodology
A1 - Miller,B.D.
A1 - Hollingsworth,E.
A1 - Sander,L.W.
Y1 - 1985///
N1 - 1985160480
English
Journal: Article

KW - EMBASE
KW - Behavior
KW - breathing
KW - central nervous system
KW - Child
KW - Child Development
KW - heart
KW - Heart Rate
KW - Human
KW - human experiment
KW - psychological aspect
RP - NOT IN FILE
SP - 286
EP - 297
JF - Journal of the American Academy of Child Psychiatry
VL - 24
IS - 3
CY - United States
N2 - This paper presents a discussion of conceptual and technical issues considered critical to the understanding, design, and implementation of developmental research using behavioral and physiologic measures. Background information is presented demonstrating the effectiveness of naturalistic monitoring of infant 24-hour, sleep-awake cycles in determining state regulation, and the importance of this function as it relates to other physiologic subsystems and to infant risk. Also included is a comprehensive report, with illustrations, of a biotelemetry system for concurrent, continuous monitoring of infant behavior, heart beat, and respiration in the naturalistic setting for the purpose of assessing physiologic regulation in relation to caregiving activity
SN - 0002-7138
AD - (Miller, Hollingsworth, Sander) National Jewish Hospital/National Asthma Center, Denver, CO 80206 United States
ER -

TY - JOUR
ID - 4047
T1 - Accidents in schoolchildren
A1 - Jensen,T.T.
A1 - Uhrbrand,B.
Y1 - 1985///
N1 - 1985184184
Danish
Journal
KW - EMBASE
KW - accident
KW - Child
KW - Epidemiology
KW - Etiology
KW - Human
KW - injury
KW - school
KW - school child
RP - NOT IN FILE
SP - 2637
EP - 2639
JF - Ugeskrift for Laeger
JA - Ugeskr.Laeger
VL - 147
IS - 33
CY - Denmark

N2 - A material from a casualty department consisting of 177 accidents which occurred in children in primary schools is presented. 4.5% of the school children were injured during school hours to an extent which required medical treatment. This figure is high when the duration of the brief school day in Denmark is considered. The sites and mechanisms of the injuries are considered in relation to the various age-groups and the individual schools. The smaller children are injured most frequently by falls and while using playground equipment while the injuries in older children occurred mainly during sport and gymnastics. The majority of the injuries were slight but 6% of the cases required hospitalization. The severity and number of the injuries were relatively uniform in the various schools. In one school, however, an accumulation of accidents involving doors was observed and this was connected with the closing mechanism of the doors in that particular school
SN - 0041-5782
AD - (Jensen, Uhrbrand) Sonderborg Sygehus, Ortopaed-Kirurgisk Afdeling, DK-6400 Sonderborg Denmark
ER -

TY - JOUR
ID - 4048
T1 - Behavioral validation of the children's action tendency scale
A1 - Deluty,R.H.
Y1 - 1984///
N1 - 1985008495
English
Journal
KW - EMBASE
KW - Aggression
KW - Assertiveness
KW - Behavior
KW - central nervous system
KW - clinical article
KW - Diagnosis
KW - Human
KW - psychological aspect
KW - school child
RP - NOT IN FILE
SP - 115
EP - 130
JF - Journal of Behavioral Assessment
VL - 6
IS - 2
CY - United States
N2 - The concurrent validity of the Children's Action Tendency Scale (CATS), a self-report measure of aggressiveness, assertiveness, and submissiveness, was assessed using behavioral observations in naturalistic settings. Forty-five third- to fifth-grade children were observed over an 8-month period in a wide variety of school (and school-related) activities. Scores on the CATS subscales were found to correlate significantly with a variety of behavioral indices of aggressiveness, assertiveness, and submissiveness; these correlations tended to be higher for boys than for girls. Explanations for these sex differences and recommendations for the questionnaire's use are offered
SN - 0164-0305
AD - (Deluty) Department of Psychology, University of Maryland Baltimore County, Catonsville, MD 21228 United States
ER -

TY - JOUR
ID - 4049
T1 - Completeness of routine reporting of school-related injuries to children
A1 - Woodward,C.A.
A1 - Milner,R.
A1 - Harsanyi,Z.
Y1 - 1984///

N1 - 1985056819
English
Journal: Article
KW - EMBASE
KW - Adolescent
KW - Canada
KW - geographic distribution
KW - Human
KW - injury
KW - Monitoring
KW - organization and management
KW - playground
KW - school child
KW - short survey
RP - NOT IN FILE
SP - 454
EP - 457
JF - Canadian Journal of Public Health
JA - Can J Public Health
VL - 75
IS - 6
CY - Canada
N2 - The authors examined the amount and effects of underreporting in a passive surveillance system used by three schoolboards to monitor injuries to school children. We compared information collected through active surveillance of 50 randomly selected schools for a two month time period staggered throughout the school year with the findings of passive surveillance. This comparison showed that schools report less than 25% of injuries to children. Serious injuries are more likely to be routinely reported. It is estimated that one out of two serious injuries was reported routinely, while one in five injuries of a non-serious nature was reported. The playground is the major site of injury
SN - 0008-4263
AD - (Woodward, Milner, Harsanyi) Children's Hospital of Eastern Ontario, Ottawa, Ont. Canada
ER -

TY - JOUR
ID - 4050
T1 - Dying young in traffic
A1 - Sunderland,R.
Y1 - 1984///
N1 - 1984184409
English
Journal: Article
KW - EMBASE
KW - age
KW - Case Report
KW - Child
KW - child death
KW - economic aspect
KW - fatality
KW - geographic distribution
KW - Human
KW - Incidence
KW - injury
KW - playground
KW - social aspect
KW - socioeconomics
KW - traffic accident
RP - NOT IN FILE

SP - 754
EP - 757
JF - Archives of Disease in Childhood
JA - Arch Dis Child
VL - 59
IS - 8
CY - United Kingdom
N2 - There were 469 unnatural deaths among the Sheffield child population between 1947 and 1979, 226 of which were caused by traffic accidents. The annual incidence of these fatal traffic accidents has risen steadily since the early 1960s with boys being consistently more vulnerable. Fatal accidents were disproportionately higher among social deprived families and in poorer areas of the city. The environment rather than the child may be 'accident prone' and there is an urgent need for safer play areas in deprived areas
SN - 0003-9888
AD - (Sunderland) Children's Hospital, Birmingham B16 8ET United Kingdom
ER -

TY - JOUR
ID - 4051
T1 - I.P.A. newsletter
KW - SportDiscus
KW - *PLAYGROUNDS
KW - NEWSLETTERS
RP - NOT IN FILE
CY - Sheffield, Eng.;
PB - Intern Playground Association Resources
UR -
https://login.proxy.bib.uottawa.ca/login?url=http://search.ebscohost.com/login.aspx?direct=true&db=s3h&AN=SPHP1958&site=ehost-live
ER -

TY - JOUR
ID - 4052
T1 - 59th Annual Proceedings of the College Physical Education Association
Y1 - 1956///
KW - SportDiscus
KW - *UNIVERSITIES & colleges
KW - *PHYSICAL education & training
KW - *SPORTS
KW - MEETINGS
KW - United States
RP - NOT IN FILE
CY - Washington;
PB - College Physical Education Association
UR -
https://login.proxy.bib.uottawa.ca/login?url=http://search.ebscohost.com/login.aspx?direct=true&db=s3h&AN=SPHS-971775&site=ehost-live
ER -

TY - JOUR
ID - 4053
T1 - Recreation patterns in New Zealand
Y1 - 1971///
KW - SportDiscus
KW - *RECREATION
KW - *AQUATIC sports
KW - *AERONAUTICAL sports
KW - *MOTORSPORTS

KW - *SPORTS
KW - *STRESS (Physiology)
KW - *PARTICIPATION
KW - age
KW - AUCKLAND (N.Z.)
KW - New Zealand
KW - Child
KW - PROFESSIONAL
KW - SEX FACTOR
RP - NOT IN FILE
CY - Auckland, New Zealand;
PB - Auckland Regional Authority.
N2 - This study of areas of Auckland compares present participation patterns in sport (outdoor team and individual, water based and motor), fitness and social activities according to age and sex with what citizens 'would like to do'. Data were collected for school children between the ages of eleven and eighteen and for club officials and those responsible for administering facilities with two questionnaires. There is a need for more planning and provision of casual leisure time activities, especially in individual sports and for females
UR -
https://login.proxy.bib.uottawa.ca/login?url=http://search.ebscohost.com/login.aspx?direct=true&db=s3h&AN=SPH336247&site=ehost-live
ER -

TY - JOUR
ID - 4054
T1 - Manual on day-camps and playgrounds for the trainable retarded and some children in special education classes
Y1 - 1976///
KW - SportDiscus
KW - *DAY camps
KW - *PLAYGROUNDS
KW - *MENTAL retardation
KW - *PEOPLE with disabilities
KW - *SPECIAL education
KW - TECHNICAL manuals
KW - Child
KW - SPORTS AND RECREATION ACTIVITIES-Camping
RP - NOT IN FILE
CY - Toronto;
PB - Ontario Association for the Mentally Retarded
UR -
https://login.proxy.bib.uottawa.ca/login?url=http://search.ebscohost.com/login.aspx?direct=true&db=s3h&AN=SPH144434&site=ehost-live
ER -

TY - JOUR
ID - 4055
T1 - From five to fourteen: a report on the leisure time needs of the five to fourteen age group
Y1 - 1978///
KW - SportDiscus
KW - *LEISURE
KW - *SPORTS facilities
KW - *PLAYGROUNDS
KW - teenagers
KW - Management
KW - CITY planning
KW - INNER cities
KW - England

KW - Interests
KW - Child
KW - London
KW - MUNICIPAL
KW - Leadership
RP - NOT IN FILE
CY - ;
UR -
https://login.proxy.bib.uottawa.ca/login?url=http://search.ebscohost.com/login.aspx?direct=true&db=s3h&AN=SPH33923&site=ehost-live;http://articles.sirc.ca/search.cfm?id=33923
L2 - http://articles.sirc.ca/search.cfm?id=33923
ER -

TY - JOUR
ID - 4056
T1 - Tunnel with a jumping bird: children's concepts of the ideal playground
Y1 - 1979///
KW - SportDiscus
KW - *PLAYGROUNDS
KW - DESIGN
KW - building
RP - NOT IN FILE
CY - Wodonga;
PB - Town & Country Planning Board
UR -
https://login.proxy.bib.uottawa.ca/login?url=http://search.ebscohost.com/login.aspx?direct=true&db=s3h&AN=SPH217716&site=ehost-live
ER -

TY - JOUR
ID - 4057
T1 - Using playground equipment as the basis for activity on the days when children are not scheduled for PE
Y1 - 1985/02//
KW - SportDiscus
KW - *PLAYGROUNDS
KW - *RECREATION
KW - *STRESS (Physiology)
KW - *SAFETY
KW - PROGRAMS
KW - Elementary Schools
KW - Child
RP - NOT IN FILE
SP - 2
EP - 5
JA - Physical Education Newsletter
IS - 167
CY - ;
SN - 00318973
UR -
https://login.proxy.bib.uottawa.ca/login?url=http://search.ebscohost.com/login.aspx?direct=true&db=s3h&AN=SPH179621&site=ehost-live;http://articles.sirc.ca/search.cfm?id=179621
L2 - http://articles.sirc.ca/search.cfm?id=179621
ER -

TY - JOUR
ID - 4058
T1 - Prime Time Concern

Y1 - 1989/06//
KW - SportDiscus
KW - *OBESITY in children
KW - *JUNK food
KW - *PHYSICAL fitness
KW - TELEVISION & children
KW - TELEVISION advertising
KW - COMPULSIVE eating
RP - NOT IN FILE
SP - 119
EP - 119
JF - Journal of Sport & Exercise Psychology
JA - J Sport Exerc Psychol
VL - 11
IS - 2
N2 - The article reports on a possible relationship between excessive television watching and childhood obesity. A Nielsen study reports that children spend more than 20 hours per week watching television, which does little to burn calories compared to physical activities outdoors. In addition, children viewing TV tend to eat food high in fats, which is influenced by commercials that give children a false sense of reality. To combat this, parents should limit children's TV time and promote better eating habits
SN - 08952779
UR -
https://login.proxy.bib.uottawa.ca/login?url=http://search.ebscohost.com/login.aspx?direct=true&db=s3h&AN=20717824&site=ehost-live
ER -

TY - JOUR
ID - 4059
T1 - Measuring physical activity in fifth graders
Y1 - 1997/12//
KW - SportDiscus
KW - *OUTDOOR recreation for children
KW - FIFTH grade (Education)
RP - NOT IN FILE
SP - 435
JF - Journal of Sport & Exercise Psychology
JA - J Sport Exerc Psychol
VL - 19
IS - 4
N2 - Presents a summary of the article `Validation of Interview- and Self-Administered Physical Activity Checklists for Fifth Grade Students,' by J.F. Sallis, P.K. Strikmiller, and others, found in `Medicine and Science in Sports and Exercise,' Number 28
SN - 08952779
UR -
https://login.proxy.bib.uottawa.ca/login?url=http://search.ebscohost.com/login.aspx?direct=true&db=s3h&AN=9712131472&site=ehost-live
ER -

TY - JOUR
ID - 4060
T1 - Playground Injuries Are a Serious Problem
Y1 - 1998/09//
KW - SportDiscus
KW - *PLAYGROUNDS
KW - *CHILDREN -- Wounds & injuries
KW - SAFETY measures
KW - United States

RP - NOT IN FILE
SP - 7
JA - JOPERD: The Journal of Physical Education, Recreation & Dance
VL - 69
IS - 7
N2 - Reports that according to the National Program for Playground Safety, over 200,000 preschool and elementary-aged school children in the United States are injured by playground equipment and receive emergency hospital care. Suggestion that most injuries occur in May, June and September; Details on the injuries; How the American Association for Leisure and Recreation's Committee on Play is addressing this problem
SN - 07303084
UR -
https://login.proxy.bib.uottawa.ca/login?url=http://search.ebscohost.com/login.aspx?direct=true&db=s3h&AN=1229831&site=ehost-live
ER -

TY - JOUR
ID - 4061
T1 - New Books on Maternal--Child Health
Y1 - 1999/04//
KW - SportDiscus
KW - *BREASTFEEDING (Humans)
KW - *HEALTH
KW - MOTHER & child
KW - INFANTS
RP - NOT IN FILE
SP - 606
EP - 607
JF - American Journal of Public Health
JA - Am J Public Health
VL - 89
IS - 4
N2 - The article focuses on the book "Breast Feeding and Human Lactation," 2nd ed., by Kathleen Auerback and Jan Riordan. Infants are less likely to be breast-fed today than at any other time in history. The World Health Organization estimated in 1996 that only about one third of the babies younger than 4 months exclusively receive breast milk. The median duration of breast-feeding internationally is about 18 months. Women who are poor and live in rural settings are most likely to breast-feed. The most important obstacle is the lack of "acceptance by society of the need for a mother and child to be together and the right of the breast-feeding dyad to participate in social, civic, and commercial activities outside the home." This second edition of a text published in 1993 has expanded clinical information on the breast-feeding process and techniques and has added a new chapter on maternal nutrition; appended to most chapters are sections on clinical implications. The book is a comprehensive reference that begins by reviewing the historical, cultural, and familial context of breast-feeding
SN - 00900036
UR -
https://login.proxy.bib.uottawa.ca/login?url=http://search.ebscohost.com/login.aspx?direct=true&db=s3h&AN=1733716&site=ehost-live
ER -

TY - JOUR
ID - 4062
T1 - Keeping Kids Safe
Y1 - 2000/09//
KW - SportDiscus
KW - *SAFETY
KW - *AQUATIC sports -- Safety measures
KW - *PLAYGROUNDS

KW - *CYCLING
KW - *BASEBALL
KW - *SKATING
KW - *SOCCER
KW - Child
RP - NOT IN FILE
SP - 44
EP - 44
JA - Active Living (1206-0941)
VL - 9
IS - 4
CY - ;
N2 - This section provides parents with tips for keeping children safe while in the water, at the playground, riding a bike, playing baseball and/or soccer, skating and looking out the window
SN - 12060941
UR -
https://login.proxy.bib.uottawa.ca/login?url=http://search.ebscohost.com/login.aspx?direct=true&db=s3h&AN=SPHS-1056572&site=ehost-live;http://articles.sirc.ca/search.cfm?id=S-1056572
L2 - http://articles.sirc.ca/search.cfm?id=S-1056572
ER -

TY - JOUR
ID - 4063
T1 - Clinical update in musculoskeletal medicine. Ways to improve playground safety and accessibility
Y1 - 2000/06//
N1 - Accession Number: 2000063202. Language: English. Entry Date: 20001001. Publication Type: journal article; pictorial; tables/charts; website. Journal Subset: Allied Health; Editorial Board Reviewed; Expert Peer Reviewed; Peer Reviewed; USA. NLM UID: 8608119
KW - CINAHL
KW - Wounds and Injuries -- Prevention and Control -- In Infancy and Childhood
KW - Play and Playthings -- In Infancy and Childhood
KW - child safety
KW - Consumer Health Information
KW - Orthopedic Surgery -- Organizations
KW - Injury Pattern -- In Infancy and Childhood
KW - Information Resources
KW - Child
RP - NOT IN FILE
SP - 368
JA - Journal of Musculoskeletal Medicine
VL - 17
IS - 6
SN - 0899-2517
UR - http://search.ebscohost.com/login.aspx?direct=true&db=cin20&AN=2000063202&site=ehost-live
ER -

TY - JOUR
ID - 4064
T1 - EARLY DETECTION OF SOCIAL ANXIETY: RELIABILITY AND VALIDITY OF A TEACHER QUESTIONNAIRE FOR THE IDENTIFICATION OF SOCIAL ANXIETY IN YOUNG CHILDREN
Y1 - 2001/12//
KW - SportDiscus
KW - *ANXIETY
KW - *CHILD psychology
KW - *MENTAL health
KW - *TEACHERS
RP - NOT IN FILE

SP - 787
JA - Social Behavior & Personality: An International Journal
VL - 29
IS - 8
N2 - Socially anxious children have been described in the literature as at risk of developing increasing emotional problems and of being deprived of valid learning experiences with peers. In order to enable teachers to detect social anxiety in preschoolers the authors developed a scale. The TRSA-"junior" (Teacher Rating Scale of Social Anxiety-"junior") is a brief Likert-type questionnaire for the identification of social anxiety in 5-to 6-year-old children. Reliability (internal consistency plus test-retest correlation) collected in 2 samples was high. Convergent and divergent validity of this newly developed rating-scale were also studied. A high level of social anxiety correlated with a low level of social participation, as observed on the playground. The TRSA-"junior" score correlated with "internalizing" as measured by the Behavior Questionnaire for Toddlers and Preschoolers (BQTP) and the Child Behavior Check List (CBCL), and with the subscales "anxious-depressed", "thought problems", "social problems" and "withdrawn behavior" of the CBCL. In the second study a high score on social anxiety appeared to be related to less positive sociometeric judgments by peers. The authors conclude that the TRSA-"junior" may aid in the identification and prevention of social anxiety at an early age. In addition, the scale would appear to be a useful instrument in research, given its sound psychometric qualities. ABSTRACT FROM AUTHOR
SN - 03012212
UR -
https://login.proxy.bib.uottawa.ca/login?url=http://search.ebscohost.com/login.aspx?direct=true&db=s3h&AN=8771699&site=ehost-live
ER -

TY - JOUR
ID - 4065
T1 - Schools Becoming All Work and No Play
Y1 - 2001/11//
KW - SportDiscus
KW - *SCHOOL children
KW - *HEALTH
KW - *SCHOOL recess breaks
KW - *SCHOOLS
KW - United States
RP - NOT IN FILE
SP - 11
JA - JOPERD: The Journal of Physical Education, Recreation & Dance
VL - 72
IS - 9
N2 - Reports on the trend of United States schools rejecting scheduled recess periods in favor of additional instruction time. Concern of child development experts regarding the trend; Importance of physical play to health of students
SN - 07303084
UR -
https://login.proxy.bib.uottawa.ca/login?url=http://search.ebscohost.com/login.aspx?direct=true&db=s3h&AN=5526925&site=ehost-live
ER -

TY - JOUR
ID - 4066
T1 - NASPE TELLS PARENTS AND ELEMENTARY SCHOOL OFFICIALS "RECESS IS A MUST!"
Y1 - 2001/06//
KW - SportDiscus
KW - *PHYSICAL education & training
KW - *RECESSES
KW - Elementary Schools
KW - NATIONAL Association for Sport & Physical Education

RP - NOT IN FILE
SP - 3
EP - 3
JA - GAME (Augusta, Ga.)
CY - ;
N2 - That National Association for Sport and Physical Education (NASPE) has issues a statement indicating to parent the importance of recess in elementary school. The benefits of children having unstructured recess time for the promotion of health and physical activity are highlighted
UR -
https://login.proxy.bib.uottawa.ca/login?url=http://search.ebscohost.com/login.aspx?direct=true&db=s3h&AN=SPHS-1026331&site=ehost-live;http://articles.sirc.ca/search.cfm?id=S-1026331;http://www.gahperd.org
L2 - http://articles.sirc.ca/search.cfm?id=S-1026331
ER -

TY - JOUR
ID - 4067
T1 - Recess revival
Y1 - 2001/10//
KW - SportDiscus
KW - *GAMES
KW - *SPORTS -- Rules
KW - PROGRAMS
KW - Child
RP - NOT IN FILE
SP - 5
EP - 5
JA - Input
VL - 1
IS - 8
CY - ;
N2 - The following five games that promote physical activity are presented: Across the Great Divide, Co-op Four Square, Boom... Chick a Boom..., Ulu Maika and Hackball
SN - 14899396
UR -
https://login.proxy.bib.uottawa.ca/login?url=http://search.ebscohost.com/login.aspx?direct=true&db=s3h&AN=SPHS-796792&site=ehost-live;http://articles.sirc.ca/search.cfm?id=S-796792;http://www.mohawkc.on.ca/cira
L2 - http://articles.sirc.ca/search.cfm?id=S-796792
ER -

TY - JOUR
ID - 4068
T1 - THE HIGHER YOU CLIMB, THE HARDER YOU FALL
Y1 - 2002/04//
KW - SportDiscus
KW - *PLAYGROUNDS
KW - *EQUIPMENT & supplies
KW - *CHILD development
KW - United States
RP - NOT IN FILE
SP - 78
JA - Parks & Recreation
VL - 37
IS - 4
N2 - Talks about the research conducted in the U.S. as of April 2002, of children playing on playgrounds with equipment platforms more than ten feet high. Views of experts on playground equipment heights; Purposes of children from climbing playground equipment; Des1ription of the observation sites
SN - 00312215

UR -
https://login.proxy.bib.uottawa.ca/login?url=http://search.ebscohost.com/login.aspx?direct=true&db=s3h&AN=6725096&site=ehost-live
ER -

TY - JOUR
ID - 4070
T1 - Report #2002-9: Iowa playground injury lacked evidence of negligence
Y1 - 2002///
KW - SportDiscus
KW - *PLAYGROUNDS
KW - *WOUNDS & injuries
KW - Child Care
KW - SUPERVISION
KW - ACTIONS & defenses
KW - Iowa
KW - Child
KW - DUTY OF CARE
RP - NOT IN FILE
SP - 36
EP - 37
JA - Recreation & Parks Law Reporter
VL - 19
IS - 1
CY - ;
N2 - The Court ruled that the Defendants did not violate any standard of care and that they were in compliance with State requirements regarding the ratio of supervisors to children. Case: Brown v. United Presbyterian Home no. 1-641 / 00-1663 (Iowa App. 01/28/2002). Court of Appeals of Iowa, January 28, 2002. Attached opinion of the court has been edited and citations omitted
SN - 07435649
UR -
https://login.proxy.bib.uottawa.ca/login?url=http://search.ebscohost.com/login.aspx?direct=true&db=s3h&AN=SPHS-869026&site=ehost-live;http://articles.sirc.ca/search.cfm?id=S-869026
L2 - http://articles.sirc.ca/search.cfm?id=S-869026
ER -

TY - JOUR
ID - 4071
T1 - Fun times
Y1 - 2002/09//
KW - SportDiscus
KW - *PEOPLE with disabilities
KW - *OUTDOOR recreation
KW - *SPORTS
KW - PROGRAMS
KW - Child
KW - SPORTS AND RECREATION ACTIVITIES-General
RP - NOT IN FILE
SP - 62
EP - 62
JA - Sports 'n Spokes Magazine
VL - 28
IS - 6
CY - ;
N2 - Provides information about Turning POINT, a program that provides outdoor recreational and sport opportunities for people with physical disabilities, including children and youth
SN - 01616706

UR -
https://login.proxy.bib.uottawa.ca/login?url=http://search.ebscohost.com/login.aspx?direct=true&db=s3h&AN=SPHS-851582&site=ehost-live;http://articles.sirc.ca/search.cfm?id=S-851582;http://www.pva.org/
L2 - http://articles.sirc.ca/search.cfm?id=S-851582
ER -

TY - JOUR
ID - 4072
T1 - Suits pushing playgrounds: Citizen-Board Member Branch
Y1 - 2003/06//
KW - SportDiscus
KW - *EDUCATION
KW - Child Care
KW - PROGRAMS
KW - Legislation
KW - ADVOCACY
KW - Child
RP - NOT IN FILE
SP - 71
EP - 71
JA - Parks & Recreation
VL - 38
IS - 6
CY - ;
N2 - This article describes how corporate and business professionals are becoming increasingly visible as advocates for children's programs such as day care, after-school care and early education
SN - 00312215
UR -
https://login.proxy.bib.uottawa.ca/login?url=http://search.ebscohost.com/login.aspx?direct=true&db=s3h&AN=SPHS-943738&site=ehost-live;http://articles.sirc.ca/search.cfm?id=S-943738;http://www.nrpa.org
L2 - http://articles.sirc.ca/search.cfm?id=S-943738
ER -

TY - JOUR
ID - 4073
T1 - Physical activity tips for locked in students
Y1 - 2003/07//
KW - SportDiscus
KW - *PHYSICAL education & training
KW - *TEACHING
KW - *STRESS (Physiology)
KW - Elementary Schools
KW - Child
KW - Indoor
RP - NOT IN FILE
SP - 10
EP - 10
JA - Strategies (08924562)
VL - 16
IS - 6
CY - ;
N2 - Provides tips for teachers looking to provide physical activity opportunities for children at times when outdoor activity is not possible
SN - 08924562
UR -
https://login.proxy.bib.uottawa.ca/login?url=http://search.ebscohost.com/login.aspx?direct=true&db=s3h&AN=SPHS-893633&site=ehost-live;http://articles.sirc.ca/search.cfm?id=S-893633;http://www.aahperd.org

L2 - http://articles.sirc.ca/search.cfm?id=S-893633
ER -

TY - JOUR
ID - 4074
T1 - Feds worried about playground-cancer link
Y1 - 2003/12/14/
N1 - Accession Number: 2004097820. Language: English. Entry Date: 20040618. Revision Date: 20090313. Publication Type: journal article. Journal Subset: Biomedical; Online/Print; Public Health; USA. Special Interest: Public Health
KW - CINAHL
KW - Arsenic -- Adverse Effects -- In Infancy and Childhood
KW - Environmental Exposure -- In Infancy and Childhood
KW - Childhood Neoplasms -- Risk Factors
KW - Play and Playthings
KW - Child
KW - child safety
KW - Child,Preschool
KW - Infant
RP - NOT IN FILE
SP - 22
EP - 22
JA - Medical Letter on the CDC
SN - 1078-2907
UR - http://search.ebscohost.com/login.aspx?direct=true&db=cin20&AN=2004097820&site=ehost-live
ER -

TY - JOUR
ID - 4075
T1 - Product Roundup
Y1 - 2005/08//
KW - SportDiscus
KW - *RECREATION areas
KW - *RECREATION centers
KW - *PLAYGROUNDS
KW - *EQUIPMENT & supplies
KW - NEW product development
KW - SAFETY measures
RP - NOT IN FILE
SP - 70
EP - 184
JA - Parks & Recreation
VL - 40
IS - 8
N2 - This article presents information on several new products for use in parks and other recreation areas. New to the spraypark industry, I-Spy is Waterplay's innovative product line featuring transparent pipe. I-Spy technology heightens the play experience when water floods through the transparent piping in various directions, rates and patterns. American Locker Group Inc. introduces the new REL, keyless electronic locker system. Loose fill rubber safety surfacing is regarded as the most effective way to keep children safe on playgrounds. GroundScape Technologies' Kids is an ADA-approved loose fill rubber product that comes in a variety of colors to match any commercial playground equipment
SN - 00312215
UR -
https://login.proxy.bib.uottawa.ca/login?url=http://search.ebscohost.com/login.aspx?direct=true&db=s3h&AN=17958357&site=ehost-live
ER -

TY - JOUR
ID - 4076
T1 - TIME 2 GO
Y1 - 2005/10//
KW - SportDiscus
KW - *HIKING for children
KW - *OUTDOOR recreation for children
RP - NOT IN FILE
SP - 31
EP - 35
JA - Backpacker
VL - 33
IS - 8
N2 - Offers ideas on hiking activities and sites for children
SN - 0277867X
UR -
https://login.proxy.bib.uottawa.ca/login?url=http://search.ebscohost.com/login.aspx?direct=true&db=s3h&AN=18260114&site=ehost-live
ER -

TY - JOUR
ID - 4077
T1 - Building Community One Playground At a Time
Y1 - 2005/08//
KW - SportDiscus
KW - *PLAYGROUNDS
KW - *ATHLETIC fields
KW - PLAY environments
KW - URBAN research
KW - PUBLIC lands
KW - SOCIAL groups
KW - NEW York (N.Y.)
KW - NEW York (State)
KW - United States
RP - NOT IN FILE
SP - 18
EP - 184
JA - Parks & Recreation
VL - 40
IS - 8
N2 - This article focuses on a research by the Trust for Public Land (TPL), which reveals that three in four residents of large American cities do not have access to a nearby park, playground or open space. Children in park-poor neighborhoods play in streets, alleyways and vacant lots rather than in grassy parks, baseball diamonds or soccer fields. One of TPL's programs, Parks for People Initiative, works in cities and suburbs nationwide to build playgrounds, such as in Newark, New Jersey, and New York City. After selecting a new site and partner organization, TPL launches an extensive outreach and design process with the school and community
SN - 00312215
UR -
https://login.proxy.bib.uottawa.ca/login?url=http://search.ebscohost.com/login.aspx?direct=true&db=s3h&AN=17958040&site=ehost-live
ER -

TY - JOUR
ID - 4078
T1 - JUNIOR GOLF
Y1 - 2005/04//

KW - SportDiscus
KW - *GOLF
KW - *GOLF tees
KW - *CHILDREN
KW - Parents
RP - NOT IN FILE
SP - 86
EP - 86
JA - Golf Digest
VL - 56
IS - 4
N2 - The article presents some suggestions by "Golf Digest" teaching professional Renee Powell on how to introduce children to golf, without having to watch them being turned off from the game. On a regular course, children have been suggested to tee off from the 150-yard marker. Parents have been suggested to give their children their own par and scorecard because, they might play a hole in four shots, but it can be seven or eight for their children. Parents have also been asked to visit the course at off-peak hours to avoid slowing down experienced players, and to pick up before the score gets to double digits
SN - 0017176X
UR -
https://login.proxy.bib.uottawa.ca/login?url=http://search.ebscohost.com/login.aspx?direct=true&db=s3h&AN=16278720&site=ehost-live
ER -

TY - JOUR
ID - 4079
T1 - Physical Activity Time and Being a Good Sport
Y1 - 2006/11//
KW - SportDiscus
KW - *SCHOOL recess breaks
KW - *OUTDOOR recreation
KW - *SPORTSMANSHIP
KW - *PHYSICAL fitness for children
KW - *EXERCISE for children
KW - *PHYSICAL education teachers
KW - SOCIAL aspects
KW - PROBLEM solving in children
RP - NOT IN FILE
SP - 51
EP - 54
JA - Teaching Elementary Physical Education
VL - 17
IS - 6
N2 - The article looks at how to improve school recess time. In the article, the author, a physical education teacher, details the recess program he developed, called Physical Activity Time, which encourages children to participate in physical activity and be a good sport. The article discusses developing sportsmanship in children and teaching children how to solve problems. According to the author, by providing children with structure and clear communication of expectations, the social climate of recess at his school was greatly improved
SN - 10454853
UR -
https://login.proxy.bib.uottawa.ca/login?url=http://search.ebscohost.com/login.aspx?direct=true&db=s3h&AN=23040448&site=ehost-live
ER -

TY - JOUR
ID - 4080
T1 - Ideas to Promote Physical Activity With Your Students
Y1 - 2006/11//

KW - SportDiscus
KW - *PHYSICAL education teachers
KW - *PHYSICAL education for children
KW - *PHYSICAL fitness for children
KW - *EXERCISE for children
KW - *PEDOMETERS
KW - *PLAYGROUNDS
KW - *GAMES
KW - *HEALTH promotion
KW - NATIONAL Association for Sport & Physical Education
KW - After School Programs
RP - NOT IN FILE
SP - 74
EP - 76
JA - Teaching Elementary Physical Education
VL - 17
IS - 6
N2 - The article presents ideas that physical fitness teachers can use to promote physical activity in their students. The article examines the National Association for Sport and Physical Education's position statement on children's need for daily physical activity, which states that children should get 60 minutes or more of daily physical activity and should not have extended periods of inactivity. The article discusses running a lunchtime or after-school intramural program, developing a walking club, letting students use a pedometer for a week at a time, and teaching a unit on active playground games
SN - 10454853
UR -
https://login.proxy.bib.uottawa.ca/login?url=http://search.ebscohost.com/login.aspx?direct=true&db=s3h&AN=23040454&site=ehost-live
ER -

TY - JOUR
ID - 4082
T1 - Introduction: The Power of Physical Activity in Youth Development
Y1 - 2006/11//
KW - SportDiscus
KW - *PHYSICAL fitness for children
KW - *EXERCISE for children
KW - *CHILD development
KW - *SCHOOL recess breaks
KW - *OUTDOOR recreation
KW - SOCIAL aspects
KW - PSYCHOLOGICAL aspects
KW - School Environment
KW - EMOTIONAL trauma in children
KW - SOCIAL development
RP - NOT IN FILE
SP - 29
EP - 29
JA - Teaching Elementary Physical Education
VL - 17
IS - 6
N2 - The article introduces the special report on the use of physical activity in youth development. According to the author, physical activity is a great way to promote community, increase social development, and help children believe in themselves. The special report covers several topics, including the role of play in the development of preschoolers, recommendations for working with youth trauma survivors, strategies for working with disabled youth, social development barriers at schools, and how to improve the social environment at recess
SN - 10454853

UR -
https://login.proxy.bib.uottawa.ca/login?url=http://search.ebscohost.com/login.aspx?direct=true&db=s3h&AN=23040442&site=ehost-live
ER -

TY - JOUR
ID - 4083
T1 - Rebuilding ... One Playground at a Time
Y1 - 2006/04//
KW - SportDiscus
KW - *PLAYGROUNDS
KW - *SPORTS facilities
KW - *RECREATION
KW - ARCHITECTURE -- Conservation & restoration
KW - Photographs
KW - TSUNAMIS
KW - Sri Lanka
RP - NOT IN FILE
SP - 40
EP - 41
JA - Parks & Recreation
VL - 41
IS - 4
N2 - Several photographs relating to playground renovations being carried out in tsunami-ravaged areas in Sri Lanka are presented. One photograph shows National Recreation and Park Association member Curtis Stoddard posing with children during new playground construction. Other photograph shows Sri Lankan children posing on their newly built climber. In one photograph, locals are shown using string to test for plumb, while in other photograph a builder is shown enjoying his lunch. Another photograph shows locals passing concrete relay style from the mixing area to the equipment
SN - 00312215
UR -
https://login.proxy.bib.uottawa.ca/login?url=http://search.ebscohost.com/login.aspx?direct=true&db=s3h&AN=20459376&site=ehost-live
ER -

TY - JOUR
ID - 4084
T1 - FUN FOR ALL!
Y1 - 2006/05//
KW - SportDiscus
KW - *PLAYGROUNDS
KW - *SPORTS facilities
KW - *CHILDREN with disabilities
KW - ASSOCIATIONS,institutions,etc.
KW - ONTARIO (Calif.)
KW - California
RP - NOT IN FILE
SP - 45
EP - 45
JA - Sports 'n Spokes Magazine
VL - 32
IS - 3
N2 - The article features the playground for both able and disabled children in Ontario, California. The playground was built in just a day by KaBOOM! together with a group of Paralympic athletes and volunteers from The Home Depot and Easter Seals. This was part of the mission of KaBOOM! to build 1,000 playgrounds in 1,000 days
SN - 01616706

UR -
https://login.proxy.bib.uottawa.ca/login?url=http://search.ebscohost.com/login.aspx?direct=true&db=s3h&AN=20651646&site=ehost-live
ER -

TY - JOUR
ID - 4085
T1 - Trailblazers: Playing It Safe
Y1 - 2006/04//
KW - SportDiscus
KW - *PLAYGROUNDS
KW - *RECREATION
KW - CORPORATE presidents
KW - SAFETY measures
KW - MCDONALD'S Corp.
KW - COMMUNITY centers
RP - NOT IN FILE
SP - 74
EP - 74
JA - Parks & Recreation
VL - 41
IS - 4
N2 - The article focuses on Fran Wallach, the president of the recreation consulting company Total Recreation Management Services. She is most known for her ground-breaking work with playground safety. She has been a playground safety consultant to countless projects and organizations, including McDonald's Corp. and Discovery Zone. She had a proud moment every time one of her three children graduated from college. She thinks the future of parks and recreation is brighter than it has ever been, despite tight budgets or lack of money
SN - 00312215
UR -
https://login.proxy.bib.uottawa.ca/login?url=http://search.ebscohost.com/login.aspx?direct=true&db=s3h&AN=20459398&site=ehost-live
ER -

TY - JOUR
ID - 4086
T1 - THE QUESTIONS WITH Mike Rucker
Y1 - 2006/11/27/
KW - SportDiscus
KW - *FOOTBALL players
KW - CAROLINA Panthers (Football team)
KW - PARK rangers
RP - NOT IN FILE
SP - 35
EP - 35
JA - Sports Illustrated
VL - 105
IS - 21
N2 - This article provides an interview with Carolina Panthers football player Mike Rucker. Rucker discusses his most embarrassing moment as a football player. Rucker also notes that his favorite off-day activity is spending the day outdoors with his children, and that he would work as a park ranger if he were not a football player
SN - 0038822X
UR -
https://login.proxy.bib.uottawa.ca/login?url=http://search.ebscohost.com/login.aspx?direct=true&db=s3h&AN=23267428&site=ehost-live
ER -

TY - JOUR
ID - 4087
T1 - PEI Model of Health: Fortune Consolidated School Edible Classroom Program
Y1 - 2006/05//
KW - SportDiscus
KW - *NUTRITION
KW - *FOOD
KW - Schools
KW - PROGRAMS
KW - PRINCE Edward Island
KW - Child
RP - NOT IN FILE
SP - N12
EP - N12
JA - Active Living
VL - 15
IS - 3
CY - ;
N2 - In addition to removing less healthy options from the lunchroom, PEI's Fortune Consolidated School has arranged its recess schedule to allow kids to play before lunch and brought in a volunteer chef to cook with the kids
SN - 1188620X
UR - https://login.proxy.bib.uottawa.ca/login?url=http://search.ebscohost.com/login.aspx?direct=true&db=s3h&AN=SPHS-1029963&site=ehost-live;http://articles.sirc.ca/search.cfm?id=S-1029963
L2 - http://articles.sirc.ca/search.cfm?id=S-1029963
ER -

TY - JOUR
ID - 4088
T1 - Conducting a Successful Aussie Hoops Holiday Camp
Y1 - 2007///
KW - SportDiscus
KW - *BASKETBALL camps
KW - *OUTDOOR recreation for children
KW - *CHILDREN
KW - *SPORTS camps
KW - SOCIETIES & clubs
KW - Australia
RP - NOT IN FILE
SP - 10
EP - 11
JA - X's & O's
VL - 14
IS - 2
N2 - The article presents a guide on conducting a hoops holiday camp in Australia. A holiday camp gives children opportunity to participate in basketball activities at a local club or association over a period of time. It also provides children experiencing basketball for the first time the comfort of playing basketball activities with friends and other children their own age, fulfillment of being taught new skills by trained Coaches and recognition of attending the clinic through a camp certificate or show bag
SN - 13223747
UR - https://login.proxy.bib.uottawa.ca/login?url=http://search.ebscohost.com/login.aspx?direct=true&db=s3h&AN=26132801&site=ehost-live
ER -

TY - JOUR

ID - 4089
T1 - RECESS SKILLS FOR CHILDREN WITH SPECIAL NEEDS
Y1 - 2007/05//
KW - SportDiscus
KW - *INTRAMURAL sports
KW - *RECESSES
KW - *GAMES
KW - *PEOPLE with disabilities
KW - Child
RP - NOT IN FILE
SP - 6
EP - 7
JA - Input
CY - ;
N2 - In this article the author provides tips that can be used to include children with special needs, such as developmental disabilities, in playground fun. Highlighted are concepts that were discussed at a meeting titled, "Surviving the Playground - Recess Strategies and Learning the Rules" such as learning for one another or older siblings
SN - 14899396
UR -
https://login.proxy.bib.uottawa.ca/login?url=http://search.ebscohost.com/login.aspx?direct=true&db=s3h&AN=SPHS-1055976&site=ehost-live;http://articles.sirc.ca/search.cfm?id=S-1055976;http://www.mohawkc.on.ca/external/cira/home.html
L2 - http://articles.sirc.ca/search.cfm?id=S-1055976
ER -

TY - JOUR
ID - 4090
T1 - BUILD ITƒ_ÝTHEY WILL COME
Y1 - 2007/11//
KW - SportDiscus
KW - *SPORTS for people with disabilities
KW - *PLAYGROUNDS
KW - *HEALTH facilities
KW - BARRIER-free design
KW - Intergenerational Relations
KW - CHILDREN & adults
KW - NURSING care facilities
KW - ORLANDO (Fla.)
KW - Florida
RP - NOT IN FILE
SP - 26
EP - 26
JA - Sports 'n Spokes Magazine
VL - 33
IS - 6
N2 - This article reports on the efforts of several organizations in Orlando, Florida to build a handicap-accessible playground for both children and adults at the intergenerational care facility, Grandma's House. The playground will help the residents of Grandma's House to bridge the generation gap. The complete playground makeover was performed and completed in four days
SN - 01616706
UR -
https://login.proxy.bib.uottawa.ca/login?url=http://search.ebscohost.com/login.aspx?direct=true&db=s3h&AN=27346168&site=ehost-live
ER -

TY - JOUR

ID - 4091
T1 - Is it time to put the risk back into children's play?
Y1 - 2007/09//
KW - SportDiscus
KW - *PLAYGROUNDS
KW - *SPORTS facilities
KW - *CHILDREN -- Wounds & injuries
KW - PLAY environments
KW - Great Britain
RP - NOT IN FILE
SP - 44
EP - 45
JA - Leisure Management
VL - 27
IS - 5
N2 - The article focuses on views whether children should be given more freedom to play outside and take risks in Great Britain. There are concerns in the country's playgrounds which critics say are too safe, dull and unimaginative. This means that children are not being challenged. By cutting the element of managed risks in playgrounds, the risk for children's injuries elsewhere could be reduced
SN - 02669102
UR -
https://login.proxy.bib.uottawa.ca/login?url=http://search.ebscohost.com/login.aspx?direct=true&db=s3h&AN=26776624&site=ehost-live
ER -

TY - JOUR
ID - 4092
T1 - CONTRIBUTION OF YOUTH SPORT TO DAILY ACTIVITY
Y1 - 2007/09//
KW - SportDiscus
KW - *SPORTS -- Research
KW - *SPORTS for children
KW - *PHYSICAL education
KW - *YOUTH
KW - AMERICAN College of Sports Medicine
KW - SOCIETIES & clubs
RP - NOT IN FILE
SP - 2
EP - 2
JA - ACSM's Health & Fitness Journal
VL - 11
IS - 5
N2 - The article reports on the research examining the role of organized youth sports, physical education class and recess play in the daily physical activity levels of children by American College of Sports Medicine (ACSM). The study included 119 boys aged between 6 and 12 years from sports programs provided with moderate-to-vigorous physical activities (MVPAs) measured using accelerators. The results showed that youth sports provides an opportunity to increase total daily levels of MVPA
SN - 10915397
UR -
https://login.proxy.bib.uottawa.ca/login?url=http://search.ebscohost.com/login.aspx?direct=true&db=s3h&AN=26707212&site=ehost-live
ER -

TY - JOUR
ID - 4093
T1 - WALLACE PUBLIC SCHOOL -- WINTER ACTIVE
Y1 - 2008/01//

KW - SportDiscus
KW - *SCHOOL recess breaks
KW - *INTRAMURAL sports
KW - PUBLIC schools
KW - RURAL schools
KW - EIGHTH grade (Education)
KW - SNOW sculpture
KW - Ontario
RP - NOT IN FILE
SP - 4
EP - 5
JA - INPUT Newsletter
N2 - This article focuses on Wallace Public School, a small rural school just outside of Listowel, Ontario. There are 270 students in this Grade 8 school. It has two 20 minute breaks daily. Particular focus is given to the school's Intramural 3on3 Basketball event during winter. A variety of sculptures created by school children can be found in the snow covered school yard such as snowmen and snow forts
SN - 14899396
UR -
https://login.proxy.bib.uottawa.ca/login?url=http://search.ebscohost.com/login.aspx?direct=true&db=s3h&AN=31448014&site=ehost-live
ER -

TY - JOUR
ID - 4094
T1 - Recess for Elementary School Students A Position Paper from the National Association for Sport and Physical Education
Y1 - 2008///Spring
KW - SportDiscus
KW - *SCHOOL recess breaks
KW - *SCHOOL children
KW - *CHILD development
KW - NATIONAL Association for Sport & Physical Education
KW - SOCIAL development
RP - NOT IN FILE
SP - 10
EP - 12
JA - Pennsylvania Journal of Health, Physical Education, Recreation & Dance
VL - 78
IS - 2
N2 - The article presents a position paper from the National Association for Sport and Physical Education that all elementary school children should have a least 20 minutes of recess daily. Various government and welfare organizations recognize the importance of recess to a child's physical, social and academic development. It provides them with time to engage in physical activity to help develop their bodies, develop life skills and give them opportunities to enjoy movement. Physical activities are said to improve attention, behavior and learning
SN - 02790033
UR -
https://login.proxy.bib.uottawa.ca/login?url=http://search.ebscohost.com/login.aspx?direct=true&db=s3h&AN=33161845&site=ehost-live
ER -

TY - JOUR
ID - 4095
T1 - Learning Outside the Classroom Manifesto
Y1 - 2008///Summer
KW - SportDiscus
KW - *OUTDOOR education
KW - *OUTDOOR recreation

KW - RURAL education
KW - Environmental Education
KW - SCHOOL camps
RP - NOT IN FILE
SP - 23
EP - 23
JA - Horizons (14620677)
IS - 42
N2 - The article discusses the importance of out and about guidance for schools for all Learning Outside the Classroom (LOtC) settings, providers and venues. According to the author, this will begin later this summer and this will start out practical measures to be taken to assure the youth to experience high-quality offsite activities. Questions answerable by yes or no by the Director of Children's Services including supporting and encouraging the local authority officer responsible for LOtC are also listed
SN - 14620677
UR -
https://login.proxy.bib.uottawa.ca/login?url=http://search.ebscohost.com/login.aspx?direct=true&db=s3h&AN=33134942&site=ehost-live
ER -

TY - JOUR
ID - 4096
T1 - FIS TO LAUNCH INITIATIVE TO ATTRACT CHILDREN TO SNOWSPORTS
Y1 - 2008/01/15/
KW - SportDiscus
KW - *SKIING for children
KW - *SPORTS for children
KW - *WINTER sports
KW - INTERNATIONAL Ski Federation
RP - NOT IN FILE
SP - 9
EP - 9
JA - Ski Racing
VL - 40
IS - 5
N2 - The article reports on the plan of the International Ski Federation (FIS) to launch the Bring Kids Back to the Snow campaign to promote playing in the snow as the number 1 outdoor activity of choice for children and the youth during the winter. The campaign aims to generate passion for snow activities with the younger generations. The target market for the campaign will be countries that have interest in snow sports. FIS president Gian Franco Kasper says that the campaign is important for encouraging children and families to spend more time in the snow
SN - 00376213
UR -
https://login.proxy.bib.uottawa.ca/login?url=http://search.ebscohost.com/login.aspx?direct=true&db=s3h&AN=28686286&site=ehost-live
ER -

TY - JOUR
ID - 4097
T1 - Youths Less Active Outdoors A ú Recess Affects Student Behavior
Y1 - 2009/05//
KW - SportDiscus
KW - *OUTDOOR recreation
KW - *OUTDOOR life
KW - *OUTDOOR education
KW - *OUTDOOR recreation for children
KW - *YOUTH
KW - *MOUNTAINS -- Recreational use

KW - NATURE observation
KW - NATURE appreciation
KW - United States
RP - NOT IN FILE
SP - 9
EP - 10
JA - JOPERD: The Journal of Physical Education, Recreation & Dance
VL - 80
IS - 5
N2 - The article reports on the declined participation of the youths aged 6-12 years in nature- based outdoor activities such as mountain biking, backpacking, and trail running in the U.S. It presents the percentage evaluation of youth participants based on the 2008 Outdoor Recreation Participation Report. Also discussed is the observed behaviors of school children during daily recess period at school. A study on the issue of recess in schools set from the Early Childhood Longitudinal Study, Kindergarten Class of 1998-1999 is also presented
SN - 07303084
UR -
https://login.proxy.bib.uottawa.ca/login?url=http://search.ebscohost.com/login.aspx?direct=true&db=s3h&AN=40313868&site=ehost-live
ER -

TY - JOUR
ID - 4098
T1 - "Take the Lead!" -- A playground leadership initiative
Y1 - 2009/11/02/
KW - SportDiscus
KW - *HEALTH promotion
KW - *YOUTH
KW - *CHILDREN
KW - HEALTH programs
KW - SERVICES for
KW - Saskatchewan
RP - NOT IN FILE
SP - N7
EP - N7
JA - Active Living
N2 - The article provides information on "Take The Lead!," a health promotion program for children and the youth which is offered by the Saskatchewan Parks and Recreation Association (SPRA). The program offers several advantages including the addition of youth leadership opportunities, reduction in violent behavior in children and promotion of Healthy Active Living during free time. Information regarding the training, its venue and schedule is also provided
SN - 1188620X
UR -
https://login.proxy.bib.uottawa.ca/login?url=http://search.ebscohost.com/login.aspx?direct=true&db=s3h&AN=45461778&site=ehost-live
ER -

TY - JOUR
ID - 4099
T1 - DOES BMI IMPACT ACTIVITY LEVEL?
Y1 - 2009///Summer
KW - SportDiscus
KW - *BODY mass index
KW - *OBESITY in children
KW - *OVERWEIGHT children
KW - *PHYSICAL fitness for children
KW - *PHYSICAL education for children
KW - Hawaii

RP - NOT IN FILE
SP - 19
EP - 20
JA - Ethnicity & Disease
VL - 19
IS - 3
N2 - Research has found that obese and overweight boys and girls are prone to less physical activity. In this study, we reinvestigate the relationship between physical activity and obesity among children. We examined the differences in physical activity between overweight and normal weight children. Onehundred and twenty six Hawaii school-system children participated in this study. The children were measured for height and weight, and answered questions about their activity level. Activity level was assessed by participation in physical education, active play during recess and lunch, and other activities during the past week. Children were divided into obese, overweight and normal weight based on their age and body mass index (BMI). Ageadapted BMI was used as overweight and obesity indicator. The hypothesis for this project is that physical activity will be greater with normal weight boys and overweight girls than it is with overweight boys and normal weight girls. ABSTRACT FROM AUTHOR
SN - 1049510X
UR -
https://login.proxy.bib.uottawa.ca/login?url=http://search.ebscohost.com/login.aspx?direct=true&db=s3h&AN=42750572&site=ehost-live
ER -

TY - JOUR
ID - 4100
T1 - Panel: Trial Court Acted Prematurely in Negligence Case
Y1 - 2009/03/13/
KW - SportDiscus
KW - *OUTDOOR recreation for children
KW - JUDGMENTS
KW - ACTIONS & defenses (Law)
KW - PERSONAL injuries (Law)
KW - SCHOOL administration
KW - NEW York (State)
RP - NOT IN FILE
SP - 1
EP - 1
JA - Sports Litigation Alert
VL - 6
IS - 4
N2 - The article reports on the decision of the New York State appeals court to reverse a trial court rulings in a negligence case involving a female student who was injured while playing touch football during recess. The daughter of plaintiff Lenore Bellinger has knocked out three of her teeth and fractured a fourth after colliding with a teammate. The panel believed that a school is not responsible for every thoughtless or careless act by which one pupil may injure another
SN - 1552194X
UR -
https://login.proxy.bib.uottawa.ca/login?url=http://search.ebscohost.com/login.aspx?direct=true&db=s3h&AN=37242454&site=ehost-live
ER -

TY - JOUR
ID - 4101
T1 - Clinical highlights. Daily recess improves classroom behavior
Y1 - 2009/03//
N1 - Accession Number: 2010231970. Language: English. Entry Date: 20090403. Revision Date: 20091030. Publication Type: journal article; brief item; pictorial. Journal Subset: Core Nursing; Nursing; Peer Reviewed; USA. Special Interest: Pediatric Care. No. of Refs: 1 ref. NLM UID: 20010080R

KW - CINAHL
KW - Academic Performance -- In Infancy and Childhood
KW - Child Behavior
KW - Play and Playthings
KW - Students,Elementary
KW - Child
KW - Prospective Studies
RP - NOT IN FILE
SP - 18
EP - 18
JA - RN
VL - 72
IS - 3
SN - 0033-7021
UR - http://search.ebscohost.com/login.aspx?direct=true&db=cin20&AN=2010231970&site=ehost-live
ER -

TY - JOUR
ID - 4102
T1 - Should schools hire private companies to supervise recess?
Y1 - 2010/05//
KW - SportDiscus
KW - *SCHOOL recess breaks
KW - *PHYSICAL activity
KW - PRIVATE companies
KW - United States
RP - NOT IN FILE
SP - 9
EP - 10
JA - JOPERD: The Journal of Physical Education, Recreation & Dance
VL - 81
IS - 5
N2 - The article presents views of Americans concerning whether schools should hire private companies to supervise children during recess. Graduate student Vicki Davies comments that children would get more physical activity time if a company is hired. Student Christine Bennett expresses that the school must allow students to be themselves during recess by letting them enjoy what they are doing. Morgann Wagner remains neutral by assessing both the advantages and disadvantages of hiring companies
SN - 07303084
UR -
https://login.proxy.bib.uottawa.ca/login?url=http://search.ebscohost.com/login.aspx?direct=true&db=s3h&AN=51011896&site=ehost-live
ER -

TY - JOUR
ID - 4103
T1 - Local Knowledge
Y1 - 2010/05/03/
KW - SportDiscus
KW - *GOLF
KW - *GOLF courses
KW - MICKELSON,Phil,1970-
KW - PEBBLE Beach Golf Links (Pebble Beach,Calif.)
KW - PEBBLE Beach (Calif.)
KW - California
RP - NOT IN FILE
SP - 54
EP - 1

JA - Golf World
VL - 63
IS - 35
N2 - The article offers news briefs related to golf. Golfer Phil Mickelson is refusing requests of media for interviews as he wants to spend time with his family. He spends his time playing with his children, taking them out and his wife Amy Mickelson out on dates. Mike Davis, senior director of rules at the U.S. Golf association (USGA) has suggested having the uphill blind tee shot at par-4 eighth hole in Pebble Beach Golf Links, Pebble Beach in California by moving the fairway 25 yards
SN - 00171891
UR -
https://login.proxy.bib.uottawa.ca/login?url=http://search.ebscohost.com/login.aspx?direct=true&db=s3h&AN=51474444&site=ehost-live
ER -

TY - JOUR
ID - 4104
T1 - In-School Physical Activity for Children with Intellectual Disabilities
Y1 - 2010/09//
KW - SportDiscus
KW - *PHYSICAL education for children with mental disabilities
KW - *PEOPLE with mental disabilities
KW - *EDUCATION
KW - *PHYSICAL education for people with disabilities
KW - *CARDIOVASCULAR fitness
KW - *PHYSICAL activity
KW - United States
RP - NOT IN FILE
SP - 11
EP - 11
JA - JOPERD: The Journal of Physical Education, Recreation & Dance
VL - 81
IS - 7
N2 - The article presents a study conducted by K. H. Pitetti, M. W. Beets and C. Combs in 2009 in the U.S. on youths with intellectual disabilities (ID) who are reported to have lower levels of cardiovascular fitness and muscular strength. The researchers assessed physical activity patterns of children with ID in three school settings which include adapted physical education (APE), self-contained classrooms (CR), and inclusive recess (RE). The outcomes of the study are also presented
SN - 07303084
UR -
https://login.proxy.bib.uottawa.ca/login?url=http://search.ebscohost.com/login.aspx?direct=true&db=s3h&AN=54073796&site=ehost-live
ER -

TY - JOUR
ID - 4105
T1 - Youth Fishing Programs More Important Than Ever
Y1 - 2010/04/02/Spring
KW - SportDiscus
KW - *MASS media
KW - *TEENAGERS
KW - *CHILDREN
KW - *EDUCATION
KW - *FISHING
KW - *OUTDOOR recreation
KW - Telephone
RP - NOT IN FILE
SP - 4

EP - 4
JA - GAHPERD Journal
N2 - The article reports that a study from the Kaiser Family Foundation and Stanford University in the U.S. showed that people ages 8 to 18 years spend an average of 7 hours and 38 minutes a day using entertainment media. It notes that about half of heavy media users reported to get fair or poor grades compared to light users. Studies revealed that outdoor activities can help children increase classroom performance. The Future Fisherman Foundation is offering a youth fishing program, an outdoor fishing activity designed for youngsters
UR -
https://login.proxy.bib.uottawa.ca/login?url=http://search.ebscohost.com/login.aspx?direct=true&db=s3h&AN=51315503&site=ehost-live
ER -

TY - JOUR
ID - 4106
T1 - Physical Environment, Social Factors, and Child Characteristics Influence Activity Intensity in Preschool Settings
Y1 - 2011/10//
KW - SportDiscus
KW - *PHYSICAL fitness
KW - *GAMES
KW - *CHILD psychology
KW - CHILDREN & the environment
KW - SOCIAL factors
KW - PRESCHOOL children
KW - Child Care
KW - HUMAN ecology
KW - developmental psychology
RP - NOT IN FILE
SP - 750
EP - 750
JF - Journal of Sport & Exercise Psychology
JA - J Sport Exerc Psychol
VL - 33
IS - 5
N2 - The article cites a research study that analyzes how physical environment, social factors, and child characteristics influence preschool children's physical activity intensity. It is said that when examining predictors of physical activity intensity in young children, features of the physical environment and social factors need to be considered together. The study included 175 children from 9 preschool child-care settings. It was found that when children were outside and when more activity opportunities were present, they generally played with higher intensity
SN - 08952779
UR -
https://login.proxy.bib.uottawa.ca/login?url=http://search.ebscohost.com/login.aspx?direct=true&db=s3h&AN=66801004&site=ehost-live
ER -

TY - JOUR
ID - 4107
T1 - "Go Outside and Play"
Y1 - 2011/03//
KW - SportDiscus
KW - *OUTDOOR recreation
KW - *RECREATION
KW - *CHILDREN
KW - *CYCLING
KW - *TRIATHLON
KW - Research

KW - AMERICANS
KW - INTERNET surveys
KW - United States
RP - NOT IN FILE
SP - 16
EP - 16
JA - Parks & Recreation
VL - 46
IS - 3
N2 - The article discusses the participation of Americans in outdoor recreation as being noted in the "2010 Outdoor Recreation Participation Report." It says that the study is based on an online survey targetting over 40,000 Americans. It cites that 137.8 million Americans participated regularly in outdoor recreation in 2009 and 14,652,000 children age six to 17 involved in biking-related outings. It adds the 43.5% of 2009 triathlon participants who reported being first-time racers in the said year
SN - 00312215
UR -
https://login.proxy.bib.uottawa.ca/login?url=http://search.ebscohost.com/login.aspx?direct=true&db=s3h&AN=59442931&site=ehost-live
ER -

TY - JOUR
ID - 4108
T1 - Heart of the Nation: Basile Island 6530
Y1 - 2011/12/10/
KW - SportDiscus
KW - *PLAYGROUNDS
KW - ISLANDS -- Australia
RP - NOT IN FILE
SP - 9
EP - 9
JA - Weekend Australian (National)
N2 - The article focuses on Basile Island of the Abrolhos chain, which has a special place in the heart of Pia Boschetti as it used to be her playground every winter during her childhood
UR -
https://login.proxy.bib.uottawa.ca/login?url=http://search.ebscohost.com/login.aspx?direct=true&db=s3h&AN=71889570&site=ehost-live
ER -

TY - JOUR
ID - 4109
T1 - How to prevent childhood obesity - news from the IDEFICS study
Y1 - 2011/06/02/
N1 - Accession Number: 2011253121. Language: English. Entry Date: 20111007. Revision Date: 20131213. Publication Type: journal article; tables/charts. Supplement Title: Supplement 1. Journal Subset: Consumer Health; Continental Europe; Editorial Board Reviewed; Europe; Expert Peer Reviewed; Online/Print; Peer Reviewed. Special Interest: Nutrition; Pediatric Care. No. of Refs: 4 ref
KW - CINAHL
KW - Health Behavior -- In Infancy and Childhood
KW - Health Promotion -- Methods -- In Infancy and Childhood
KW - Pediatric Obesity -- Epidemiology
KW - Pediatric Obesity -- Prevention and Control
KW - Child
KW - Child,Preschool
KW - Diet
KW - Europe
KW - Female
KW - Male

KW - Physical Activity
KW - Sleep
RP - NOT IN FILE
SP - 2
EP - 3
JA - Food Today
N2 - Stimulating children to exercise by creating an environment with enough green space, sports facilities and playgrounds may be a simple means to combat obesity in children. At the same time parents have to make sure that their children get enough sleep during the night. These are the latest results of the European Commission-funded project IDEFICS (Identification and prevention of Dietary- and lifestyle-induced Health EFfects In Children and infantS). Ac 2011 EUFIC
UR - http://search.ebscohost.com/login.aspx?direct=true&db=cin20&AN=2011253121&site=ehost-live
ER -

TY - JOUR
ID - 4111
T1 - Measures of Play
Y1 - 2012/08//
KW - SportDiscus
KW - *CHILDREN
KW - *HEALTH
KW - *PARKS
KW - *OBESITY in children
KW - *PLAYGROUNDS
KW - *HIGH school students
KW - Statistics
KW - United States
RP - NOT IN FILE
SP - 12
EP - 12
JA - Parks & Recreation
VL - 47
IS - 8
N2 - Statistics are presented on the relation between children and parks in the U.S. including rise in childhood obesity in neighborhoods without playgrounds, proximity ratio of the number of children and parks, and percentage of physical activity pursued by high-school students
SN - 00312215
UR -
https://login.proxy.bib.uottawa.ca/login?url=http://search.ebscohost.com/login.aspx?direct=true&db=s3h&AN=79113272&site=ehost-live
ER -

TY - JOUR
ID - 4112
T1 - TIME OUTDOORS AND PHYSICAL ACTIVITY AS PREDICTORS OF INCIDENT MYOPIA IN CHILDHOOD: A PROSPECTIVE COHORT STUDY
Y1 - 2012/10//
KW - SportDiscus
KW - *MYOPIA
KW - *PHYSICAL activity
KW - *CHILDREN
KW - Nature
KW - Time
RP - NOT IN FILE
SP - 6
EP - 6
JA - SportEX Dynamics

IS - 34
N2 - The article discusses assessment of Avon Longitudinal Study of Parents and Children (ALSPAC) by noncycloplegic autorefraction between a specified age group as myopic or as emmetropic for measuring physical activity using a accelerometer producing an astounding result
SN - 17449383
UR -
https://login.proxy.bib.uottawa.ca/login?url=http://search.ebscohost.com/login.aspx?direct=true&db=s3h&AN=82052447&site=ehost-live
ER -

TY - JOUR
ID - 4113
T1 - BRIEFLY NOTED
Y1 - 2012/08//
KW - SportDiscus
KW - *LEISURE industry
KW - NEWS briefs
KW - UNITED States Tennis Association
KW - COWBOY Recreation & Nature Trail (Neb.)
KW - Nebraska
KW - Georgia
KW - United States
RP - NOT IN FILE
SP - 22
EP - 23
JA - Parks & Recreation
VL - 47
IS - 8
N2 - The article offers news briefs related to the U.S. leisure industry. The Norfolk Bridge in the Cowboy Recreation and Nature Trail of Nebraska is open for public. The United States Tennis Association (USTA) is hosting USTA Free Tennis Play Day during the National Childhood Obesity Awareness Month in September 2012. The company Little Tikes Commercial and Children's Healthcare of Atlanta Inc. have decided to build a memorial playground for children with mitochondrial disease
SN - 00312215
UR -
https://login.proxy.bib.uottawa.ca/login?url=http://search.ebscohost.com/login.aspx?direct=true&db=s3h&AN=79113545&site=ehost-live
ER -

TY - JOUR
ID - 4115
T1 - Obesity Declining Among U.S. Children
Y1 - 2013/07//
KW - SportDiscus
KW - *HEALTH promotion
KW - *OBESITY
KW - *CHILDREN
KW - Behavior Modification
KW - HEALTH behavior in children
KW - Poverty
RP - NOT IN FILE
SP - 3
EP - 4
JA - Running & FitNews
VL - 31
IS - 4

N2 - The article reports on the overall decrease of childhood obesity in the U.S. based on the new data from the U.S. Centers for Disease Control and Prevention (CDC). The CDC data provided the most detailed picture of obesity among low-income U.S. citizens, using weight and height measurements from 12 million children ages 2 to 4 years old who participate in federally funded maternal and child nutrition programs. It stresses the importance of outdoor activities for obesity prevention
SN - 08985162
UR -
https://login.proxy.bib.uottawa.ca/login?url=http://search.ebscohost.com/login.aspx?direct=true&db=s3h&AN=90294754&site=ehost-live
ER -

TY - JOUR
ID - 4117
T1 - Embrace The Pace
Y1 - 2013/08//
KW - SportDiscus
KW - *COUNTRY clubs
KW - *GOLF clubs (Associations)
KW - *GOLF courses
KW - CAPITALIZATION rate
KW - MARKET value
KW - DEALS
RP - NOT IN FILE
SP - 55
EP - 1
JA - Golf Digest
VL - 64
IS - 8
N2 - The article focuses on the capitalization of golf courses for nine holes. It states the opening of the nine-hole golf clubs at Village Greens of Woodridge Golf Course outside of Chicago, Illinois. It states that in 2013, 10 percent of the rounds of Village Greens are nine holes. Moreover, it also mentions that nine-hole deals can draw in new players, can be a means to introduce golf to children, and can help people who have less time and money to stay active in the game
SN - 0017176X
UR -
https://login.proxy.bib.uottawa.ca/login?url=http://search.ebscohost.com/login.aspx?direct=true&db=s3h&AN=90411366&site=ehost-live
ER -

TY - JOUR
ID - 4118
T1 - TOPHER GAYLORD
Y1 - 2013/01//
KW - SportDiscus
KW - *OUTDOOR games
KW - *OUTDOOR recreation
KW - CORPORATE presidents
RP - NOT IN FILE
SP - 80
EP - 80
JA - SGB
VL - 46
IS - 1
N2 - An interview with Topher Gaylord, president of Mountain Hardwear and Montrail, is presented. Gaylord shares what drives him to get involved in the outdoor activity. He cites baseball, football, and basketball as his favorite sports during his childhood days. He notes that his first job was working for a specialty windsurfing sail manufacturer

SN - 15487407
UR -
https://login.proxy.bib.uottawa.ca/login?url=http://search.ebscohost.com/login.aspx?direct=true&db=s3h&AN=85157371&site=ehost-live
ER -

TY - JOUR
ID - 4119
T1 - SERIOUS FUN
Y1 - 2013/05//
KW - SportDiscus
KW - *SPORTS participation
KW - *SPORTS -- Sociological aspects
KW - *SPORTS for children
KW - *CRICKET (Sport)
KW - *GOLF
KW - Fantasy
RP - NOT IN FILE
SP - 98
EP - 98
JA - Inside Sport
IS - 257
N2 - In this article, the author presents his views on the dreams and goals of the children to play cricket at the cricket ground of Ashes and other major tournament of sports. He mentions that participation of children in games like golf, tennis and football are taken as granted and self awareness toward athletic sports are considered as obligation than the enjoyment. He also mentions that fantasy of children toward the sports is fading day by day
SN - 10371648
UR -
https://login.proxy.bib.uottawa.ca/login?url=http://search.ebscohost.com/login.aspx?direct=true&db=s3h&AN=87427438&site=ehost-live
ER -

TY - JOUR
ID - 4120
T1 - Another Reason to Love Recess
Y1 - 2013/09//
N1 - Accession Number: 2012215522. Language: English. Entry Date: 20130823. Revision Date: 20131011. Publication Type: journal article; brief item; pictorial. Journal Subset: Consumer Health; USA. Special Interest: Consumer Health; Pediatric Care. NLM UID: 9891378
KW - CINAHL
KW - Vision -- In Infancy and Childhood
KW - Play and Playthings -- In Infancy and Childhood
KW - Myopia -- Prevention and Control -- In Infancy and Childhood
KW - Sunlight -- In Infancy and Childhood
KW - Natural Environment -- In Infancy and Childhood
KW - Child
KW - child health
RP - NOT IN FILE
SP - 70
EP - 70
JA - Parents (10836373)
VL - 88
IS - 9
SN - 1083-6373
UR - http://search.ebscohost.com/login.aspx?direct=true&db=cin20&AN=2012215522&site=ehost-live
ER -

TY - JOUR
ID - 4121
T1 - Worry in the playground
Y1 - 2013/03//
N1 - Accession Number: 2012477455. Language: English. Entry Date: 20140307. Revision Date: 20140307. Publication Type: journal article; pictorial. Journal Subset: Australia & New Zealand; Nursing; Peer Reviewed. NLM UID: 0015376
KW - CINAHL
KW - Environmental Exposure -- In Infancy and Childhood -- New South Wales
KW - Fossil Fuels -- Risk Factors -- In Infancy and Childhood
KW - Dust -- Adverse Effects
KW - Public Policy
KW - New South Wales
KW - Mining -- Adverse Effects
KW - Child
RP - NOT IN FILE
SP - 26
EP - 26
JA - Lamp
VL - 70
IS - 2
SN - 0047-3936
UR - http://search.ebscohost.com/login.aspx?direct=true&db=cin20&AN=2012477455&site=ehost-live
ER -

TY - JOUR
ID - 4122
T1 - Playing it
A1 - -Freelance journalist CARRIE MADREN writes from Northern Virginia.,Carrie
Y1 - 2012/08//
KW - SportDiscus
KW - *PLAYGROUNDS
KW - *AMUSEMENT rides
KW - *PREVENTION
KW - SAFETY measures
KW - QUALITY control inspectors
KW - OCCUPATIONAL roles
KW - JOB descriptions
KW - CHILDREN'S accidents
KW - INTELLIGENCE levels
RP - NOT IN FILE
SP - 40
EP - 45
JA - Parks & Recreation
VL - 47
IS - 8
N2 - The article focuses on the job roles of certified playground safety inspectors. It mentions that for being certified, inspectors are required to pass an examination every three years which test their awareness to latest standards for various amusement rides and parks and states that safety inspectors are required to detect hazards and prevent any accidents. Also presented is a quiz for evaluating the intelligence quotient (IQ) of safety inspectors
SN - 00312215
UR - https://login.proxy.bib.uottawa.ca/login?url=http://search.ebscohost.com/login.aspx?direct=true&db=s3h&AN=79172413&site=ehost-live
ER -

TY - JOUR
ID - 4124
T1 - Identifying and managing adverse environmental health effects: outdoor air pollution. Part 2
A1 - Abelsohn,A.
A1 - Stieb,D.
A1 - Sanborn,M.D.
A1 - Weir,E.
Y1 - 2002/04/30/
KW - SportDiscus
KW - *DISEASES
KW - *DRUGS -- Side effects
KW - *RESPIRATORY insufficiency
KW - *RESPIRATORY organs -- Obstructions
KW - *ASTHMA
KW - *EXERCISE-induced asthma
KW - *BRONCHITIS
KW - *PREVENTION
KW - AIR -- Pollution
KW - Ozone
KW - SULPHUR dioxide
KW - nitrogen dioxide
KW - Environment
KW - review
RP - NOT IN FILE
SP - 1161
EP - 1167
JA - CMAJ: Canadian Medical Association Journal
VL - 166
IS - 9
CY - ;
N2 - Air pollution contributes to preventable illness and death. Subgroups of patients who appear to be more sensitive to the effects of air pollution include young children, the elderly and people with existing chronic cardiac and respiratory disease such as chronic obstructive pulmonary disease and asthma. It is unclear whether air pollution contributes to the development of asthma, but it does trigger asthma episodes. Physicians are in a position to identify patients at particular risk of health effects from air pollution exposure and to suggest timely and appropriate actions that these patients can take to protect themselves. A simple tool that uses the CH2OPD2 mnemonic (Community, Home, Hobbies, Occupation, Personal habits, Diet and Drugs) can help physicians take patients' environmental exposure histories to assess those who may be at risk. As public health advocates, physicians contribute to the primary prevention of illness and death related to air pollution in the population. In this article we review the origins of air pollutants, the pathophysiology of health effects, the burden of illness and the clinical implications of smog exposure using the illustrative case of an adolescent patient with asthma
SN - 08203946
UR -
https://login.proxy.bib.uottawa.ca/login?url=http://search.ebscohost.com/login.aspx?direct=true&db=s3h&AN=SPHS-821769&site=ehost-live;http://articles.sirc.ca/search.cfm?id=S-821769;http://www.cma.ca/eng-index.htm
L2 - http://articles.sirc.ca/search.cfm?id=S-821769
ER -

TY - JOUR
ID - 4125
T1 - Moving Environmental Justice Indoors: Understanding Structural Influences on Residential Exposure Patterns in Low-Income Communities
A1 - Adamkiewicz,Gary
A1 - Zota,Ami
A1 - Fabian,M.
A1 - Chahine,Teresa

A1 - Julien,Rhona
A1 - Spengler,John
A1 - Levy,Jonathan,I
Y1 - 2011/12/02/
N1 - Accession Number: 2011539352. Language: English. Entry Date: 20120706. Revision Date: 20131122. Publication Type: journal article; research; tables/charts. Supplement Title: Supplement. Journal Subset: Biomedical; Double Blind Peer Reviewed; Peer Reviewed; Public Health; USA. Special Interest: Public Health. Grant Information: The project was supported by the National Institute of Environmental Health Sciences (award number R21ES017522).. NLM UID: 1254074
KW - CINAHL
KW - Environmental Health
KW - Socioeconomic Factors
KW - Race Factors
KW - Environmental Exposure
KW - Human
KW - conceptual framework
KW - United States
KW - Income
KW - Residence Characteristics
KW - Nitrogen Oxides -- Analysis
KW - Particulate Matter -- Analysis
KW - Home Safety
KW - Organic Chemicals
KW - Air Pollution
KW - Lead
KW - Allergens
KW - Pets
KW - Descriptive Statistics
KW - Simulations
KW - Ethnic Groups
KW - Poverty
KW - Funding Source
KW - Family
KW - Child
KW - Air Pollution,Indoor
RP - NOT IN FILE
SP - S238
EP - S245
JF - American Journal of Public Health
JA - Am J Public Health
N2 - Objectives. The indoor environment has not been fully incorporated into the environmental justice dialogue. To inform strategies to reduce disparities, we developed a framework to identify the individual and place-based drivers of indoor environment quality. Methods. We reviewed empirical evidence of socioeconomic disparities in indoor exposures and key determinants of these exposures for air pollutants, lead, allergens, and semivolatile organic compounds. We also used an indoor air quality model applied to multifamily housing to illustrate how nitrogen dioxide (NO2) and fine particulate matter (PM2.5) vary as a function of factors known to be influenced by socioeconomic status. Results. Indoor concentrations of multiple pollutants are elevated in low socioeconomic status households. Differences in these exposures are driven by the combined influences of indoor sources, outdoor sources, physical structures, and residential activity patterns. Simulation models confirmed indoor sources' importance in determining indoor NO2 and PM2.5 exposures and showed the influence of household-specific determinants. Conclusions. Both theoretical models and empirical evidence emphasized that disparities in indoor environmental exposure can be significant. Understanding key determinants of multiple indoor exposures can aid in developing policies to reduce these disparities
SN - 0090-0036
AD - Department of Environmental Health Harvard School of Public Health, Boston, MA
Program on Reproductive Health and the Environment, University of California, San Francisco

Department of Environmental Health Harvard School of Public Health, Boston, MA
US Environmental Protection Agency, Boston
UR - http://search.ebscohost.com/login.aspx?direct=true&db=cin20&AN=2011539352&site=ehost-live
ER -

TY - JOUR
ID - 4126
T1 - Signature works
A1 - Adams,P.H.I.L.
Y1 - 2013/10/06/
KW - SportDiscus
KW - *CHILDREN
KW - SIGNATURES (Writing)
KW - AUTOGRAPH albums
KW - LETTERS
KW - AUTOGRAPHS -- Collections
RP - NOT IN FILE
SP - 34
EP - 34
JA - Weekend Australian (National)
N2 - In this article, the author focuses on the importance of signatures made on books. He informs about the craze of autograph books among the children of primary school playground wherein they treat signatures like marbles. He further mentions that famous names on letters, documents, paintings, photographs as signed copy remains something the ebook can't replicate and also became big business and subject to fraud
UR - https://login.proxy.bib.uottawa.ca/login?url=http://search.ebscohost.com/login.aspx?direct=true&db=s3h&AN=91518289&site=ehost-live
ER -

TY - JOUR
ID - 4128
T1 - Promoting informal physical activity at school recess: a pilot study with girls and boys from 2nd and 4th grades of elementary school. (Abstract)
A1 - Afonso,B.
A1 - Botelho,G.P.
Y1 - 2003/09//
KW - SportDiscus
KW - *PHYSICAL fitness
KW - *RECESSES
KW - *CARDIOVASCULAR fitness
KW - *STRESS (Physiology)
KW - *PARTICIPATION
KW - Elementary Schools
KW - girls
KW - BOYS
KW - Comparative Studies
KW - Child
KW - SEX FACTOR
RP - NOT IN FILE
SP - 143
EP - 145
JA - Revista Portuguesa de Ciencias do Desporto
VL - 3
IS - 2
CY - ;
SN - 16450523

UR -
https://login.proxy.bib.uottawa.ca/login?url=http://search.ebscohost.com/login.aspx?direct=true&db=s3h&AN=SPHS-898017&site=ehost-live;http://articles.sirc.ca/search.cfm?id=S-898017;http://www.fcdef.up.pt
L2 - http://articles.sirc.ca/search.cfm?id=S-898017
ER -

TY - JOUR
ID - 4129
T1 - Zlatan Ibrahimovic
A1 - Agnew,Paddy
Y1 - 2011/06//
KW - SportDiscus
KW - *SOCCER players
KW - *KICKING (Football)
KW - *PROFESSIONAL sports contracts
KW - IBRAHIMOVIC,Zlatan,1981-
RP - NOT IN FILE
SP - 44
EP - 51
JA - World Soccer
VL - 51
IS - 9
N2 - A biography of Swedish forward footballer Zlatan Ibrahimovic of the Milan team is presented. Ibrahimo has played with different clubs in the 10 seasons of his professional career. As a child, he was obsessed with the game, spending hours on the playground and in the streets kicking football. He signed his first professional football contract with Malmo in 1996 and later on signed a deal with Ajax in 2001. In the early days of his career, he had problems with the Sweden national team when he broke the squad curfew during a training camp in 2006
SN - 00439037
UR -
https://login.proxy.bib.uottawa.ca/login?url=http://search.ebscohost.com/login.aspx?direct=true&db=s3h&AN=61053266&site=ehost-live
ER -

TY - JOUR
ID - 4131
T1 - Maximal oxygen uptake and daily physical activity in 7- to 12-year-old boys
A1 - Al-Hazzaa,H.M.
A1 - Sulaiman,M.A.
Y1 - 1993/11//
KW - SportDiscus
KW - *PHYSICAL fitness
KW - *AEROBIC capacity
KW - *CARDIOVASCULAR fitness
KW - *STRESS (Physiology)
KW - BOYS
KW - Calibration
KW - Child
KW - CORRELATION
RP - NOT IN FILE
SP - 357
EP - 366
JF - Pediatric Exercise Science
JA - Pediatr Exerc Sci
VL - 5
IS - 4
CY - ;

N2 - The present study examined the relationship between maximal oxygen uptake (VO2max) and daily physical activity in a group of 7- to 12-year-old boys. VO2max was assessed through the incremental treadmill test using an open circuit system. Physical activity level was obtained from heart rate telemetry outside of school time for 8 hrs during weekdays and during 40 min of physical education classes. The findings indicated that the absolute value of VO2max increased with age, while relative to body weight it remained almost the same across age, with a mean of 48.4 ml.kg-1.min-1. Moreover, heart rate telemetry showed that the boys spent a limited amount of time on activities that raise the heart rate to a level above 160 bpm (an average of 1.9 percent. In addition, VO2max was found to be significantly related to the percentage of time spent at activity levels at or above a heart rate of 140 bpm, but not with activity levels at or above a heart rate of 160 bpm
SN - 08998493
UR -
https://login.proxy.bib.uottawa.ca/login?url=http://search.ebscohost.com/login.aspx?direct=true&db=s3h&AN=SPH327708&site=ehost-live;http://articles.sirc.ca/search.cfm?id=327708;http://www.humankinetics.com/
L2 - http://articles.sirc.ca/search.cfm?id=327708
ER -

TY - JOUR
ID - 4134
T1 - Noise pollution in primary education public centres of Albacete [Spanish]
A1 - AlarcA3n,Milagros
A1 - Alcaraz,Francisco
A1 - EspA-n,Antonia
A1 - de Lerma,JosAc Luis Blanco
A1 - Saus,Ma Llanos Amo
Y1 - 2011/03//
N1 - Accession Number: 2010992028. Language: Spanish. Entry Date: 20110429. Revision Date: 20110429. Publication Type: journal article; pictorial; research; tables/charts. Journal Subset: Continental Europe; Europe; Nursing; Peer Reviewed. No. of Refs: 18 ref
KW - CINAHL
KW - Learning Environment
KW - Noise
KW - Schools,Elementary
KW - Students,Elementary
KW - Child
KW - Child,Preschool
KW - Confidence Intervals
KW - Cross Sectional Studies
KW - Data Analysis Software
KW - descriptive research
KW - Descriptive Statistics
KW - Human
KW - Nonprobability Sample
KW - Spain
RP - NOT IN FILE
SP - 68
EP - 71
JA - Metas de EnfermerA-a
VL - 14
IS - 2
N2 - Objective: to determine the ongoing noise level equivalent to the one experienced by a student in his or her daily activity in public primary schools of Albacete. Material and method: cross-sectional descriptive observational study in 11 public primary schools of Albacete. Dependent variable: ongoing noise level equal to that experienced by the student (Leq in dBA). Independent variables: school and places in the school. The measurements have been performed using type 2.226 Bruel & Kjaer sound measurers with tripod. 95% Confidence intervals were calculated. Results: the average noise level equivalent to that in the classrooms of children between the age of three and five had a mean value of 75 dBA, for ages between six and seven it was 66 dBA and for ages between 8 and 11 it was 69 dBA. The sample distribution mean (ages between 3 and 11)

was 70 dBA. The average noise level equivalent in the hallway at the end of the school day had a mean value of 71 dBA and 83 dBA in the playground during break. Conclusions: the equivalent ongoing noise levels obtained exceed the limits established by the current legislation for schools. Therefore, students are exposed to elevated noise during their school activity
SN - 1138-7262
UR - http://search.ebscohost.com/login.aspx?direct=true&db=cin20&AN=2010992028&site=ehost-live
ER -

TY - JOUR
ID - 4136
T1 - Mountain Biking Injuries in Children and Adolescents
A1 - Aleman,Kylee B.
A1 - Meyers,Michael C.
Y1 - 2010/01//
KW - SportDiscus
KW - *MOUNTAIN biking
KW - *SPORTS medicine
KW - *DISLOCATIONS
KW - *SPORTS injuries -- Risk factors
KW - *CHILDREN -- Wounds & injuries
RP - NOT IN FILE
SP - 77
EP - 90
JF - Sports Medicine
JA - Sports Med
VL - 40
IS - 1
N2 - Over the last decade, the sport of mountain biking has experienced extensive growth in youth participation. Due to the unpredictable nature of outdoor sport, a lack of rider awareness and increased participation, the number of injuries has unnecessarily increased. Many believe that the actual incidence of trauma in this sport is underestimated and is just the 'iceberg'. The most common mechanism of injury is usually attributed to downhill riding and forward falling. Although rare, this type of fall can result in serious cranial and thoraco-abdominal trauma. Head and neck trauma continue to be documented, often resulting in concussions and the possibility of permanent neurological sequelae. Upper limb injuries range from minor dermal abrasions, contusions and muscular strains to complex particular fracture dislocations. These are caused by attempting to arrest the face with an outstretched hand, leading to additional direct injury. Common overuse injuries include repeated compression from the handlebars and vibration leading to neurovascular complications in the hands. Along with reports of blunt abdominal trauma and lumbar muscle strains, lower extremity injuries may include various hip/pelvic/groin contusions, patellofemoral inflammation, and various muscle strains. The primary causes of mountain biking injuries in children and adolescents include overuse, excessive fatigue, age, level of experience, and inappropriate or improperly adjusted equipment. Additional factors contributing to trauma among this age group involve musculoskeletal immaturity, collisions and falls, excessive speed, environmental conditions, conditioning and fitness status of the rider, nonconservative behavioural patterns, and inadequate medical care. The limited available data restrict the identification and understanding of specific paediatric mountain biking injuries and injury mechanisms. Education about unnecessary risk of injury, use of protective equipment, suitable bikes and proper riding technique, coupled with attentive and proper behaviour, are encouraged to reduce unnecessary injury. This article provides information on the causation and risk factors associated with injury among young mountain bikers, and recommendations to minimize trauma and enhance optimal performance and long-term enjoyment in this outdoor sport. ABSTRACT FROM AUTHOR
SN - 01121642
UR -
https://login.proxy.bib.uottawa.ca/login?url=http://search.ebscohost.com/login.aspx?direct=true&db=s3h&AN=48169731&site=ehost-live
ER -

TY - JOUR
ID - 4137

T1 - Personal incentives for participation in summer children's camps: Investigating their relationships with satisfaction and loyalty
A1 - Alexandris,Konstantinos
A1 - Kouthouris,Charilaos
Y1 - 2005/01//
KW - SportDiscus
KW - *CAMPING
KW - *CAMPS
KW - *OUTDOOR recreation
KW - *CHILDREN
KW - Satisfaction
KW - LOYALTY
KW - Greece
RP - NOT IN FILE
SP - 39
EP - 53
JA - Managing Leisure
VL - 10
IS - 1
N2 - The objective of this study was to investigate consumer behaviour issues in a summer children's camp setting. Specifically, the study aimed to: (a) identify the personal incentives that drive children to participate in summer camps, (b) test gender and age differences in children's personal incentives, and (c) investigate which of the incentives are the most important ones in predicting children's satisfaction, loyalty, and word-of-mouth communications. The sample of the study consisted of 453 (N ?=?453) children, who participated in a camp located at the mainland of north Greece. The results indicated that: (a) six personal incentive dimensions revealed from a factor analysis of the personal incentive scale, named: Camp Experience, Socialization I (making new friends), Socialization II (meeting old friends), Independence, Activities Participation, and Parents' Decision, (b) Socialization I and Camp Experience were the two highest scored dimensions, (c) girls were more driven by Socialization II (meeting old friends) related incentives than boys, who scored higher on the Activities' Participation dimension, (d) older and more experienced campers expected more independence and social interaction than younger campers, who expected to participate in a variety of sport and cultural activities, and (e) the Camp Experience dimension was the most powerful and consistent predictor of satisfaction, loyalty and word-of-mouth communications. The managerial implications of these results are discussed. ABSTRACT FROM AUTHOR
SN - 13606719
UR -
https://login.proxy.bib.uottawa.ca/login?url=http://search.ebscohost.com/login.aspx?direct=true&db=s3h&AN=17385566&site=ehost-live
ER -

TY - JOUR
ID - 4138
T1 - Ideas: fitness dollars promote more activity
A1 - Allen,M.
Y1 - 2003/06//
KW - SportDiscus
KW - *PHYSICAL fitness
KW - *CARDIOVASCULAR fitness
KW - *STRESS (Physiology)
KW - Elementary Schools
KW - INCENTIVE MOTIVATION
KW - Child
RP - NOT IN FILE
SP - 26
EP - 26
JA - Physical Education Digest
VL - 19

IS - 4
CY - ;
N2 - Brief feature reports on a "Fitness Dollars" incentive program used in an elementary school whereby children are rewarded with extra recess time for physical activity
SN - 08432635
UR - https://login.proxy.bib.uottawa.ca/login?url=http://search.ebscohost.com/login.aspx?direct=true&db=s3h&AN=SPHS-885982&site=ehost-live;http://articles.sirc.ca/search.cfm?id=S-885982;http://www.pedigest.com/
L2 - http://articles.sirc.ca/search.cfm?id=S-885982
ER -

TY - JOUR
ID - 4139
T1 - Programa de saA §de da famA-lia: proposta para identificaA□Aœo de fatores de risco para o desenvolvimento neuropsicomotor. / Family health program: proposal for identification of risk factors for neuropsychomotor development
A1 - Amorim,Roberta C.A.
A1 - Laurentino,GlA3ria E.C.
A1 - Barros,Karla M.F.T.
A1 - Ferreira,Agelia L.P.R.
A1 - Moura Filho,Alberto G.
A1 - Raposo,Maria Cristina
Y1 - 2009/11//
KW - SportDiscus
KW - *CHILD development
KW - *DISEASES -- Risk factors
KW - *HEALTH
KW - *MOTOR ability in children
KW - FAMILIES
KW - Neuropsychology
KW - Brazil
KW - Child Development
KW - Family Health Program
KW - Motor Activity
KW - Risk Factors
KW - Atividade motora
KW - desenvolvimento infantil
KW - fatores de risco
KW - Programa de SaA §de da FamA-lia
RP - NOT IN FILE
SP - 506
EP - 513
JA - Brazilian Journal of Physical Therapy / Revista Brasileira de Fisioterapia
VL - 13
IS - 6
N2 - Objectives: To investigate the occurrence of delayed neuropsychological and motor development in a community assisted through the Family Health Program and to evaluate its association with possible biological, environmental and socioeconomic risk factors. Methods: The sample consisted of 31 five-year-old children who were evaluated using the Neurological Development Test, following guidelines proposed by Coelho (1999)*. The study was carried out at the Family Health Program (FHP) center in the Brasilit area of Recife, PE, Brazil. Data were collected using a questionnaire that sought information on the child, its mother/guardian, the family's socioeconomic status and residence. Fisher's exact test was used for the statistical analysis of the results, with a significance level of 5%. Results: Appendicular coordination was the parameter with the highest frequency of deficits (90%) and static balance was the item least compromised (12.9%). The frequency of static balance deficits was higher among children who did not attend day care centers (p=0.0163) and among those who spent less time with their mother (p=0.0278). Motor persistence deficits were statistically associated with age of entry into day care (p=0.0415) and father-child time (p=0.0436). Conclusions: The FHP was not only a valuable

setting for developing studies of this nature but also an appropriate setting for child development follow-up. ABSTRACT FROM AUTHOR
SN - 14133555
UR -
https://login.proxy.bib.uottawa.ca/login?url=http://search.ebscohost.com/login.aspx?direct=true&db=s3h&AN=47531685&site=ehost-live
ER -

TY - THES
ID - 4140
T1 - Moving children toward a better life: Hispanic mothers' vision for leisure activity
A1 - Anderson,Sue
Y1 - 2011///
N1 - Accession Number: 2012154598. Language: English. Entry Date: 20130621. Revision Date: 20131213. Publication Type: doctoral dissertation; research
KW - CINAHL
KW - Hispanics
KW - Leisure Activities
KW - Physical Activity
KW - Pediatric Obesity -- Prevention and Control
KW - Child
KW - Cultural Values
KW - Ethnographic Research
KW - Female
KW - Health Behavior
KW - Health Promotion
KW - Human
KW - Interviews
KW - Male
KW - Mothers
KW - Photography
KW - Sex Factors
KW - Theory
RP - NOT IN FILE
EP - 280
PB - University of Arizona
N2 - Background: Childhood obesity is epidemic, and Hispanic children bear a particularly high health burden because of it. Evidence suggests that obesity prevention is a better option than intervention. Because schools have decreased the amount of time spent in physical education classes, leisure time has become the time to engage in physical activity, which may prevent obesity. Social ecology theory posits that a person's interaction with the environment has a strong influence on health promoting behavior. Further, this theory suggests that cultural values also play a role. The extant literature points to the sedentary nature of Hispanic children. However, it does not provide a description of the activities that children engage in during their leisure time, or how they are influenced by their family, culture, and community's environment
Purpose: The purpose of this study was to describe and provide an understanding of how leisure time is viewed by Hispanic families, and how family and culture influences Hispanic children's participation in leisure time activity. Two broad research questions were addressed: 1) How do Hispanic families describe leisure time activities? and 2) How do culture, family and the community environment influence a Hispanic child's participation in leisure time activities?
Methods: This ethnographic study incorporated photographs taken by English-speaking Hispanic mothers of children between 6 and 10 years old (n=10) as they engaged in leisure activities for one week. The participants engaged in photo-elicited interviews, describing the children's activities and performed initial data analysis by sorting the photos in the categories of activities
Findings: Girls were more sedentary, engaging in intellectually stimulating and creative play activities inside, with family members or alone. Boys were more active, engaging in unstructured play activities outside with family members or friends. The children engaged in these activities to promote their health, prepare for their adult lives, and for fun. Cultural tensions experienced by the children's parents had a profound influence on their

leisure time choices and decisions. Activities were used to prepare the children for a better life, and to teach them to balance work and fun. Ultimately, the participants wanted their children to live a balanced life; one that is happy, healthy, and successful
Relevance: The children engaged in purposefully planned leisure activities aimed to ensure their health, happiness, and success in the U.S. The participants acknowledged that to achieve a better life, the children must become expert multicultural navigators. While many of the activities were sedentary, they had important implications for the children's future. Health care providers must consider how patients view leisure time, and enlist their ideas in order to make children's activities more physically active
SN - 978-1-267-07215-3
AV - UMI Order AAI3487690.
M1 - Ph.D.
UR - http://search.ebscohost.com/login.aspx?direct=true&db=cin20&AN=2012154598&site=ehost-live
ER -

TY - JOUR
ID - 4142
T1 - The generalization and maintenance of the peer social behaviors of young children who are deaf or hard of hearing
A1 - Antia,S.D.
A1 - Kreimeyer,K.H.
Y1 - 1997///
N1 - Accession Number: 1997010576. Language: English. Entry Date: 19970301. Revision Date: 20091218. Publication Type: journal article; research; tables/charts. Journal Subset: Allied Health; Peer Reviewed; USA. Grant Information: Grant No. H023C8008790 from the United States Department of Education. No. of Refs: 37 ref. NLM UID: 0323431
KW - CINAHL
KW - Social Skills Training -- Evaluation -- In Infancy and Childhood
KW - Hearing Disorders -- In Infancy and Childhood
KW - Social Behavior -- Evaluation -- In Infancy and Childhood
KW - Interpersonal Relations -- Evaluation -- In Infancy and Childhood
KW - Funding Source
KW - Clinical Trials
KW - Child,Preschool
KW - Child
KW - Prospective Studies
KW - Female
KW - Male
KW - Peer Group
KW - Play and Playthings
KW - videorecording
KW - analysis of covariance
KW - Descriptive Statistics
KW - Repeated Measures
KW - Observational Methods
KW - Human
RP - NOT IN FILE
SP - 59
EP - 69
JA - Language, Speech & Hearing Services in Schools
VL - 28
IS - 1
N2 - The purpose of this study was to examine the effects of a social skills and comparison intervention on peer social behaviors of 43 young children who were deaf and hard of hearing (D/HH). Both interventions were conducted in small groups consisting of children who were D/HH and hearing. Data wre obtained on 15 social behaviors in four categories: peer interaction, play, child initiations/peer responses, and peer initiations/child responses. Social behaviors were recorded during free play (a) before the intervention (b) immediately after the

intervention ceased, and (c) 4 weeks after the intervention decreased their frequency of solitary and parallel play. These changes were maintained 1 year later in an outdoor play setting
SN - 0161-1461
AD - University of Arizona, Department of Special Education and Rehabilitation, Tucson, AZ 85721
UR - http://search.ebscohost.com/login.aspx?direct=true&db=cin20&AN=1997010576&site=ehost-live
ER -

TY - JOUR
ID - 4143
T1 - ACTIVITY-BASED INTERVENTION IN MOTOR SKILL DEVELOPMENT
A1 - Apache,R.R.G.
Y1 - 2005/06/15/
KW - SportDiscus
KW - *MOTOR ability
KW - *ABILITY
KW - *PSYCHOLOGY of movement
KW - *EXERCISE
KW - *CAREER development
KW - CURRICULA (Courses of study)
RP - NOT IN FILE
SP - 1011
EP - 1020
JF - Perceptual & Motor Skills
JA - Percept.Mot.Skills
VL - 100
IS - 3
N2 - This study assessed the effectiveness of an activity-based intervention program and a direct instruction program for preschool children with disabilities. Two groups of preschool students (average age =4.1 yr.), classified as having developmental delays or at risk for such delays, were selected. They were provided 15 weeks of physical education through activity-based intervention and 15 weeks of physical education by direct instruction. Instruction was provided three times a week for 30-mm. each session. In the fall semester the morning group received physical education through activity-based intervention, while the afternoon group received physical education through direct instruction. In the spring semester delivery of instruction was reversed for each group. The curriculum and activities provided to each group were identical with only the instructional delivery format altered. Two sets of pre- and post-tests using the Test of Gross Motor Development were administered before and after each 15-wk. instructional period. Group improvement in skills was compared between instructional methods. Significant improvement in both locomotor and object control skills through the activity-based intervention was found compared to direct instruction. Activity-based intervention was shown to be easily adapted to a naturalistic educational setting befitting that of preschool education. ABSTRACT FROM AUTHOR
SN - 00315125
UR -
https://login.proxy.bib.uottawa.ca/login?url=http://search.ebscohost.com/login.aspx?direct=true&db=s3h&AN=17853269&site=ehost-live
ER -

TY - JOUR
ID - 4144
T1 - Leisure opportunities for young mothers: who cares?
A1 - Arnott,A.
Y1 - 1986/09//
KW - SportDiscus
KW - *RECREATION
KW - *HEALTH
KW - *CARDIOVASCULAR fitness
KW - Mothers
KW - Child

RP - NOT IN FILE
SP - 17
EP - 19
JA - Leisure Management
VL - 6
IS - 9
CY - ;
N2 - Discusses the responsibility of leisure providers toward disadvantaged groups, focusing on women with children under the age of five. Looks at community-based activities (the public sector), leisure provision in the private sector, and the importance of the voluntary sector in providing leisure opportunities. Reports that difficulties with transportation, time, finance, and confidence, as well as the generally unwelcoming attitude toward children in Britain, are major constraints on women's involvement in leisure activities outside the home. Shows how managers and entrepreneurs can increase leisure participation by this particular disadvantaged group by being aware of their difficulties and accommodating their needs
SN - 02669102
UR -
https://login.proxy.bib.uottawa.ca/login?url=http://search.ebscohost.com/login.aspx?direct=true&db=s3h&AN=SPH206895&site=ehost-live;http://articles.sirc.ca/search.cfm?id=206895
L2 - http://articles.sirc.ca/search.cfm?id=206895
ER -

TY - JOUR
ID - 4145
T1 - Utilization of the foot load monitor for evaluating deep plantar tissue stresses in patients with diabetes: Proof-of-concept studies
A1 - Atlas,Eran
A1 - Yizhar,Ziva
A1 - Khamis,Sam
A1 - Slomka,Noa
A1 - Hayek,Shlomo
A1 - Gefen,Amit
Y1 - 2009/04//
KW - SportDiscus
KW - *BIOLOGICAL control systems
KW - TISSUE mechanics
KW - DIABETICS
KW - CHARCOT-Marie-Tooth disease
RP - NOT IN FILE
SP - 377
EP - 382
JF - Gait & Posture
JA - Gait Posture
VL - 29
IS - 3
N2 - Abstract: The purposes of the present study were to (1) determine the internal plantar mechanical stresses in diabetic and healthy subjects during everyday activities, and (2) identify stress parameters potentially capable of distinguishing between diabetic and healthy subjects. A self-designed, portable, real-time and subject-specific foot load monitor which employs the Hertz contact theory was utilized to determine the internal dynamic plantar tissue stresses in 10 diabetic patients and 6 healthy subjects during free walking and outdoors stair climbing. Internal stress parameters and average stress-doses were evaluated, and the results obtained from the two groups were compared. Internal plantar stresses and averaged stress-doses during free walking and outdoors stairs climbing in the diabetic group were 2.5ƒ_"5.5-fold higher than in the healthy group (p <0.001; stair climbing comparisons incorporated data from five diabetic patients). The interfacial pressures measured during free walking were slightly higher (ƒ^¬1.5-fold) in the diabetic group (p <0.05), but there was no significant difference between the two groups during stairs climbing. We conclude that during walking and stair climbing, internal plantar tissue stresses are considerably higher than footƒ_"shoe interface pressures, and in diabetic patients, internal stresses substantially exceed the levels in healthy. The proposed method can be used for rating

performances or design of footwear for protecting sub-dermal plantar tissues in patients who are at risk for developing foot ulcers. It may also be helpful in providing biofeedback to neuropathic diabetic patients. Copyright &y& Elsevier
SN - 09666362
UR -
https://login.proxy.bib.uottawa.ca/login?url=http://search.ebscohost.com/login.aspx?direct=true&db=s3h&AN=36767797&site=ehost-live
ER -

TY - JOUR
ID - 4146
T1 - Esercizio fisico, sport e televisione: indagine in 1368 bambini della scuola dell'obbligo. / Physical exercise, sport and television: cross-sectional analyses among 1368 children attending compulsory school
A1 - Attina,D.A.
A1 - Catalano,G.
A1 - Surace,C.
Y1 - 1997/03//
KW - SportDiscus
KW - *SPORTS
KW - *PHYSICAL education & training
KW - *RECREATION
KW - *LIFE style
KW - sociology
KW - teenagers
KW - girls
KW - BOYS
KW - Comparative Studies
KW - Television
KW - Italy
KW - Child
KW - CORRELATION
KW - TIME FACTOR
RP - NOT IN FILE
SP - 9
EP - 14
JF - Medicina dello Sport
VL - 50
IS - 1
CY - ;
N2 - A study has been conducted on a sample of 1368 children attending compulsory school in order to examine the relationship between the time spent watching television and physical activity. A distinction has been made according to the time of the day when the children watch television: before going to school, back from school throughout the afternoon, and after dinner. As far as physical activity is concerned, the weekly frequency and the duration both of the organized physical activity and of the outdoor games have been considered separatedly. The practice of organized physical activity does not have a considerable influence on television viewing unlike the physical activity carried out by children after school. In the afternoon, when children dedicate themselves to outdoor games and sport activities, the time spent watching television is inversely proportional to the physical activity practiced 8 hours per week by the youngest children and 5.9 by the grown-ups, against 5.8 and 7 hours per week spent by those children who attend primary school and by the adolescents of secondary school: those who did not practice any physical activity (group A and D), those who practiced physical exercises less than an hour a day (group B and E) and more than an hour a day (group C and F). It has been noted that, considering the groups of those children who practiced physical activity for a longer period of time, the hours spent watching television were significantly lower as far as the children attending primary school are concerned (2.55 +/ 1.19 vs 2.39 +/ 1.12 vs 2.16 +/ 1.14) (p < 0.005 A-C, p < 0.05 B-C) and those attending secondary schools (2.73 +/ 1.15 vs 2.55 +/ 1.03 vs 2.39 +/ 1.02) (p < 0.01 D-F, p < 0.05 E-F). These data is mainly due to a variation in the number of hours spent watching television during the afternoon by children attending both primary (1.28 +/ 0.87 vs 1.19 +/ 0.86 vs 0.99 +/ 0.81) (p < 0.001 A-C, p

< 0.05 B-C) and secondary schools (1.00 +/ 0.88 vs 0,89 +/ 0.84 vs 0.73 +/ 0.69) (p < 0.01 D-F, p < 0.01 E-F). The negative effects of a prolonged time spent watching television could be contrasted promoting any kind of physical activity and of outdoor games
SN - 00257826
UR - https://login.proxy.bib.uottawa.ca/login?url=http://search.ebscohost.com/login.aspx?direct=true&db=s3h&AN=SPHS-657578&site=ehost-live;http://articles.sirc.ca/search.cfm?id=S-657578
L2 - http://articles.sirc.ca/search.cfm?id=S-657578
ER -

TY - JOUR
ID - 4147
T1 - Beating the Heat
A1 - Attwood,E.M.I.L.
Y1 - 2012/12//
KW - SportDiscus
KW - *OUTDOOR life
KW - *PLAYGROUNDS
KW - *SUNBURN
KW - *SKIN
KW - *CANCER
KW - SUN
RP - NOT IN FILE
SP - 62
EP - 63
JA - Athletic Business
VL - 36
IS - 12
N2 - The article discusses the growing clamor for more playground shade in view of the risks of staying outdoors for extended period with no sun protection. According to the article, while shades are used to keep playground equipment cool, part of the concern is the long term effect of sun exposure which with just one incident of severe sunburn in childhood doubles the risk of skin cancer later in life. As the awareness increases, shaded playground equipment has become the new norm in many states
SN - 0747315X
UR - https://login.proxy.bib.uottawa.ca/login?url=http://search.ebscohost.com/login.aspx?direct=true&db=s3h&AN=83866332&site=ehost-live
ER -

TY - JOUR
ID - 4148
T1 - How Active are Your Students?
A1 - Avery,Avery
A1 - Brandt,Janet
Y1 - 2010/09//
KW - SportDiscus
KW - *EXERCISE
KW - *STUDENTS
KW - *HEALTH
KW - *PHYSICAL activity
KW - GUIDELINES
KW - AMERICANS
KW - UNITED States.Dept.of Health & Human Services
KW - United States
RP - NOT IN FILE
SP - 34
EP - 35

JA - Strategies (08924562)
VL - 24
IS - 1
N2 - The article discusses solutions for increasing physical exercise among U.S. students. According to the 2008 Physical Activity Guidelines for Americans, the U.S. Department of Health and Human Services suggested that children and adolescents must exercise at a minimum of 1 hour every day. The U.S. National Association of Sport and Physical Education created physical fitness guidelines that consist of before-school, during-school, and after-school plans including walking or biking to and from school, and a Learnercise program during recess times
SN - 08924562
UR -
https://login.proxy.bib.uottawa.ca/login?url=http://search.ebscohost.com/login.aspx?direct=true&db=s3h&AN=54054175&site=ehost-live
ER -

TY - JOUR
ID - 4149
T1 - Implementing a Policy to Increase Physical Activity
A1 - Ayers,Suzan F.
Y1 - 2010/05//
KW - SportDiscus
KW - *PHYSICAL activity
KW - *SCHOOL children
KW - *HEALTH
KW - *PHYSICAL education
KW - NORTH Carolina.State Board of Education
KW - North Carolina
RP - NOT IN FILE
SP - 6
EP - 6
JA - JOPERD: The Journal of Physical Education, Recreation & Dance
VL - 81
IS - 5
N2 - The article focuses on the amendment of State Board of Education's Healthy Active Children Policy in North Carolina to include the requirement of 30 minutes of physical activity daily for K-8 students. The policy states that physical activity, physical education, recess, and any other type of physical activity could not be taken as a form of punishment. It also mentions a survey which reveals the effectiveness of requiring regular participation in physical activity during school days
SN - 07303084
UR -
https://login.proxy.bib.uottawa.ca/login?url=http://search.ebscohost.com/login.aspx?direct=true&db=s3h&AN=51011894&site=ehost-live
ER -

TY - JOUR
ID - 4151
T1 - Elementary school children's recess behavior. (Abstract)
A1 - Babkes,M.L.
A1 - Sinclair,C.
Y1 - 2004/03//
KW - SportDiscus
KW - *PSYCHOLOGY
KW - *PHYSICAL fitness
KW - *RECESSES
KW - *LEISURE
KW - *OBESITY
KW - *CARDIOVASCULAR fitness

KW - *STRESS (Physiology)
KW - Elementary Schools
KW - VIDEO tapes
KW - Factor Analysis
KW - Child
KW - behaviour
KW - Observation
KW - Content Analysis
RP - NOT IN FILE
SP - A
EP - a
JF - Research Quarterly for Exercise & Sport
JA - Res Q Exerc Sport
VL - 75
IS - 1 Suppl
CY - ;
SN - 02701367
UR -
https://login.proxy.bib.uottawa.ca/login?url=http://search.ebscohost.com/login.aspx?direct=true&db=s3h&AN=SPHS-951400&site=ehost-live;http://articles.sirc.ca/search.cfm?id=S-951400;http://www.aahperd.org/
L2 - http://articles.sirc.ca/search.cfm?id=S-951400
ER -

TY - JOUR
ID - 4152
T1 - Epidemiology of 10,000 High School Football Injuries: Patterns of Injury by Position Played
A1 - Badgeley,Marcus A.
A1 - McIlvain,Natalie M.
A1 - Yard,Ellen E.
A1 - Fields,Sarah K.
A1 - Comstock,R.Dawn
Y1 - 2013/02//
KW - SportDiscus
KW - *HIGH school football players
KW - *WOUNDS & injuries
KW - *FOOTBALL injuries
KW - *BRAIN -- Concussion
KW - *KNEE -- Wounds & injuries
KW - *HIGH school football
KW - Research
KW - PHYSIOLOGICAL aspects
KW - concussion
KW - knee
KW - Sports
KW - surveillance
RP - NOT IN FILE
SP - 160
EP - 169
JF - Journal of Physical Activity & Health
JA - J Phys Act Health
VL - 10
IS - 2
N2 - Background: With more than 1.1 million high school athletes playing annually during the 2005-06 to 2009-10 academic years, football is the most popular boys' sport in the United States. Methods: Using an internet-based data collection tool, RIO, certified athletic trainers (ATs) from 100 nationally representative US high schools reported athletic exposure and football injury data during the 2005-06 to 2009-10 academic years. Results: Participating ATs reported 10,100 football injuries corresponding to an estimated 2,739,187 football-

related injuries nationally. The injury rate was 4.08 per 1000 athlete-exposures (AEs) overall. Offensive lineman collectively (center, offensive guard, offensive tackle) sustained 18.3% of all injuries. Running backs (16.3%) sustained more injuries than any other position followed by linebackers (14.9%) and wide receivers (11.9%). The leading mechanism of injury was player-player contact (64.0%) followed by player-surface contact (13.4%). More specifically, injury occurred most commonly when players were being tackled (24.4%) and tackling (21.8%). Conclusions: Patterns of football injuries vary by position. Identifying such differences is important to drive development of evidence-based, targeted injury prevention efforts. ABSTRACT FROM AUTHOR
SN - 15433080
UR -
https://login.proxy.bib.uottawa.ca/login?url=http://search.ebscohost.com/login.aspx?direct=true&db=s3h&AN=85351310&site=ehost-live
ER -

TY - JOUR
ID - 4154
T1 - Children 'denied right to play': children are being prevented from playing outdoors by intolerant adults who claim they cause a noise or a nuisance, new research has revealed. Matthew Baker reports
A1 - Baker,M.
Y1 - 2003/09//
KW - SportDiscus
KW - *PLAY
KW - *OUTDOOR life
KW - *RECREATION
KW - adulthood
KW - RESTRICTIONS
KW - Child
RP - NOT IN FILE
SP - 4
EP - 4
JA - Bases World
CY - ;
N2 - Studies reveal that childhood play is much more restricted outdoors these days due to parental concerns over traffic, bullying and abduction, as well as intolerant adults who believe they are too noisy or disruptive
UR -
https://login.proxy.bib.uottawa.ca/login?url=http://search.ebscohost.com/login.aspx?direct=true&db=s3h&AN=SPHS-940782&site=ehost-live;http://articles.sirc.ca/search.cfm?id=S-940782
L2 - http://articles.sirc.ca/search.cfm?id=S-940782
ER -

TY - JOUR
ID - 4157
T1 - Uczestnictwo dzieci w rekreacji i turystyce waznym elementem ich zdrowotnej edukacji. / Children's involvement in various forms of recreation as an important element of their health education
A1 - Barlak,M.
Y1 - 1992///
KW - SportDiscus
KW - *HEALTH education
KW - *OUTDOOR recreation
KW - *HIKING
KW - kindergarten
KW - Parents
KW - Teachers
KW - Questionnaires
KW - Interviews
KW - Statistics
KW - Child

KW - Movement
RP - NOT IN FILE
SP - 11
EP - 13
JA - Kultura Fizyczna
VL - 46
IS - 5-6
CY - ;
N2 - On the basis of a previously prepared questionnaire the author carried out (1988-1989) the individual interviews with pre-school children (n=300, age: 6 years). Completing the investigation, she has collected the additional data from their parents and their nursery school teachers. The findings showed that the health-related values of various forms of recreation were appreciated by majority of children, parents and teachers. Furthermore, according to the opinion of more than half of the interviewed parents and teachers, the participation in recreation activities makes the children more resourceful in their daily life (58,8 per cent of parents and 55 per cent of teachers) and lets spend their spare time in more profitable way (50 per cent of parents and 54 per cent of teachers). Symptomatic was the fact that much fewer teachers (31 per cent) than parents (51 per cent) stressed relaxation aspects of hiking and excursions. This difference of views results from the specific approach of a nursery school towards these recreation activities: the nursery school teachers are to fulfill didactic task rather than serve purposes related to health and relaxation
SN - 01377671
UR -
https://login.proxy.bib.uottawa.ca/login?url=http://search.ebscohost.com/login.aspx?direct=true&db=s3h&AN=SPH307740&site=ehost-live
ER -

TY - JOUR
ID - 4159
T1 - Catching up with Tiny Tiger
A1 - Bartels,Casey
Y1 - 2008/08//
KW - SportDiscus
KW - *GOLFERS
KW - *GOLF for children
KW - *GOLF -- Drive
RP - NOT IN FILE
SP - 40
EP - 40
JA - Golf Magazine
VL - 50
IS - 8
N2 - The article offers information on eight-year-old golf player Reece Matthew Campbell Murphy. He is currently playing at the Saline Golf Club. The author believes that one day Murphy will shoot around 38 for nine holes. According to Murphy's father, Steven Campbell, he can drive the ball 200 yards on a downward sloping hole
SN - 10565493
UR -
https://login.proxy.bib.uottawa.ca/login?url=http://search.ebscohost.com/login.aspx?direct=true&db=s3h&AN=33335835&site=ehost-live
ER -

TY - JOUR
ID - 4160
T1 - Exercise-, nature-and socially interactive-based initiatives improve mood and self-esteem in the clinical population
A1 - Barton,Jo
A1 - Griffin,Murray
A1 - Pretty,Jules

Y1 - 2012///
KW - Reviewer Nominated
RP - NOT IN FILE
SP - 89
EP - 96
JA - Perspectives in public health
VL - 132
IS - 2
PB - Sage Publications
SN - 1757-9139
ER -

TY - JOUR
ID - 4162
T1 - An analysis of the movement patterns of disabled and nondisabled children on a playground
A1 - Basler,J.L.
Y1 - 1989///
KW - SportDiscus
KW - *PLAY
KW - *PLAYGROUNDS
KW - *PEOPLE with disabilities
KW - EXAMPLE
KW - Comparative Studies
KW - Movement
KW - NON-HANDICAPPED
KW - Child
KW - Observation
RP - NOT IN FILE
CY - Eugene, Ore.;
PB - Microform Publications, College of Human Development and Performance, University of Oregon
UR -
https://login.proxy.bib.uottawa.ca/login?url=http://search.ebscohost.com/login.aspx?direct=true&db=s3h&AN=SPH260645&site=ehost-live;http://kinpubs.uoregon.edu/
ER -

TY - JOUR
ID - 4163
T1 - Measuring collaborative consultation practices in natural environments
A1 - Basu,S.
A1 - Salisbury,C.L.
A1 - Thorkildsen,T.A.
Y1 - 2010/03//
N1 - Accession Number: 2010627818. Language: English. Entry Date: 20100604. Revision Date: 20120309. Publication Type: journal article; questionnaire/scale; research; tables/charts. Journal Subset: Allied Health; Blind Peer Reviewed; Editorial Board Reviewed; Peer Reviewed; USA. Special Interest: Pediatric Care; Quality Assurance. Instrumentation: Triadic Intervention and Evaluation Rating Scale (TIERS). No. of Refs: 48 ref. NLM UID: 8910748
KW - CINAHL
KW - Collaboration
KW - Early Intervention
KW - Instrument Construction
KW - Referral and Consultation
KW - Adult
KW - Checklists
KW - Child,Preschool
KW - Female
KW - Human

KW - Illinois
KW - Infant
KW - instrument validation
KW - internal consistency
KW - interrater reliability
KW - Intrarater Reliability
KW - Male
KW - Parent-Child Relations
KW - Professional Role
KW - Professional-Family Relations
KW - Professional-Patient Relations
KW - Quality Assessment
KW - videorecording
RP - NOT IN FILE
SP - 127
EP - 150
JF - Journal of Early Intervention
VL - 32
IS - 2
N2 - This article describes the development of the Triadic Intervention and Evaluation Rating Scale (TIERS), a 33-item instrument designed to evaluate patterns of parent, service provider, and child interactions during early intervention sessions conducted in natural environments. Twenty-eight parent-provider-child triads were videotaped in home and playgroup settings, quarterly over a 15-month period. Segments representing complete routines were selected and rated by service providers and research personnel. Scoring and scaling models were developed to evaluate the quality of triadic interactions during early intervention sessions. The TIERS (a) measured differences in providers' use of collaborative consultation strategies and parents' level of participation with service providers and their children; (b) distinguished features inherent in different sessions; (c) was easy to administer; and (d) showed appropriate psychometric properties. With additional validation efforts, this instrument may be used to evaluate the implementation of collaborative interactions between parents and early intervention service providers in natural environments
SN - 1053-8151
AD - University of Illinois-Chicago
UR - http://search.ebscohost.com/login.aspx?direct=true&db=cin20&AN=2010627818&site=ehost-live
ER -

TY - JOUR
ID - 4164
T1 - Camping with Kids
A1 - Bauch,Joel
Y1 - 1995/01//
KW - SportDiscus
KW - *CAMPING
KW - *OUTDOOR recreation for children
KW - *BACKPACKING
KW - *CANOES & canoeing
KW - *CAMPS for children
KW - DAY hiking
RP - NOT IN FILE
SP - 9
EP - 13
JA - Association of Outdoor Recreation & Education Conference Proceedings
N2 - This session will focus on preparing parents and leaders for meeting the special needs of children while engaged in outdoor activities ranging from day hikes to extended backpacking and canoeing adventures. The emphasis of this workshop is how to include children in existing programming rather than creating trips and workshops especially for that age group. This outline should be useful to outdoor programmers at non-traditional colleges, on military bases, or young professionals looking at getting jobs at summer camps and other professional outdoor agencies. ABSTRACT FROM AUTHOR

UR -
https://login.proxy.bib.uottawa.ca/login?url=http://search.ebscohost.com/login.aspx?direct=true&db=s3h&AN=32917283&site=ehost-live
ER -

TY - JOUR
ID - 4165
T1 - Correlates of physical activity: why are some people physically active and others not?
A1 - Bauman,A.E.
A1 - Reis,R.S.
A1 - Sallis,J.F.
A1 - Wells,J.C.
A1 - Loos,R.J.
A1 - Martin,B.W.
Y1 - 2012/07/21/
N1 - DA - 20120723
IS - 1474-547X (Electronic)
IS - 0140-6736 (Linking)
LA - eng
PT - Journal Article
PT - Review
SB - AIM
SB - IM
KW - Adolescent
KW - Adult
KW - Aged
KW - Child
KW - Child,Preschool
KW - Exercise
KW - Female
KW - Humans
KW - Male
KW - Middle Aged
KW - Motor Activity
KW - Risk Factors
KW - Socioeconomic Factors
KW - World Health
KW - statistics & numerical data
KW - Young Adult
KW - Reviewer Nominated
RP - NOT IN FILE
SP - 258
EP - 271
JF - Lancet
VL - 380
IS - 9838
N2 - Physical inactivity is an important contributor to non-communicable diseases in countries of high income, and increasingly so in those of low and middle income. Understanding why people are physically active or inactive contributes to evidence-based planning of public health interventions, because effective programmes will target factors known to cause inactivity. Research into correlates (factors associated with activity) or determinants (those with a causal relationship) has burgeoned in the past two decades, but has mostly focused on individual-level factors in high-income countries. It has shown that age, sex, health status, self-efficacy, and motivation are associated with physical activity. Ecological models take a broad view of health behaviour causation, with the social and physical environment included as contributors to physical inactivity, particularly those outside the health sector, such as urban planning, transportation systems, and parks and trails. New areas of determinants research have identified genetic factors contributing to the propensity to be physically active, and evolutionary factors and obesity that might predispose to inactivity, and have explored the longitudinal

tracking of physical activity throughout life. An understanding of correlates and determinants, especially in countries of low and middle income, could reduce the eff ect of future epidemics of inactivity and contribute to effective global prevention of non-communicable diseases
AD - Prevention Research Collaboration, School of Public Health, Sydney University, NSW, Australia. adrian.bauman@sydney.edu.au
UR - 22818938
ER -

TY - JOUR
ID - 4166
T1 - Physical Activity Opportunity in United States Public Elementary Schools
A1 - Beaulieu,Lisa
A1 - Butterfield,Stephen A.
A1 - Pratt,Phillip
Y1 - 2009///Fall
KW - SportDiscus
KW - *PHYSICAL education -- Study & teaching
KW - *SCHOOL children
KW - *STUDENTS
KW - *HEALTH
KW - *PHYSICAL fitness
KW - ELEMENTARY schools -- United States
KW - PUBLIC schools -- United States
KW - SCHOOL enrollment
KW - NATIONAL Center for Education Statistics
KW - United States
RP - NOT IN FILE
SP - 33
EP - 36
JA - ICHPER -- SD Journal of Research in Health, Physical Education, Recreation, Sport & Dance
VL - 4
IS - 2
N2 - Recent evidence indicates that many elementary schools have curtailed recess and Physical Education (Morrow, Jackson & Payne 1999). These finding are at variance with the goal of Healthy People 2010 to increase physical activity. The purpose of this study was to examine physical activity opportunities (PAO) in U.S. public elementary schools. Survey data obtained from the National Center for Education Statistics were analyzed using within grade (1-5 or 6) between group comparisons. Key findings included: a) PAO decreased from 222 to 204 minutes/week for grades 1 to 5; b) schools with smaller enrollments (<300 pupils) provided more PAO than schools in all larger categories (p<. 01); c) schools in rural areas provided more PAO than schools in towns, suburbs, and cities (p<. 01); d) schools with a low minority enrollment (<6%) provided more PAO than did schools with very high minority enrollment (>50%) (P<. 01); and e) schools with fewer children enrolled in free/reduced price lunch (<35%) afforded more PAO than did schools with the highest rates (>75%) (P<. 01). ABSTRACT FROM AUTHOR
SN - 19304595
UR - https://login.proxy.bib.uottawa.ca/login?url=http://search.ebscohost.com/login.aspx?direct=true&db=s3h&AN=47233649&site=ehost-live
ER -

TY - JOUR
ID - 4167
T1 - SPORTS BEAT
A1 - Bechtel,Mark
A1 - Cannella,Stephen
Y1 - 2004/08/30/
KW - SportDiscus
KW - *SPORTS

KW - *PERFORMANCE
KW - *TELEVISION broadcasting of sports
KW - CONCERTS
KW - Music
KW - VAN Halen (Performer)
KW - ORIOLE Park at Camden Yards (Baltimore,Md.)
KW - TELEVISION viewers
RP - NOT IN FILE
SP - 28
EP - 28
JA - Sports Illustrated
VL - 101
IS - 8
N2 - This article presents several sports-related news briefs. Destiny's Child may be bootylicious, but the NFL is going to great lengths to make that a mootylicious point when BeyoncAc and the gals perform at the league's kickoff concert at Gillette Stadium on Sept. 9. Among the other acts-- NFL spokesman Brian McCarthy describes them as "popular but safe choices"--singing before the Patriots-Colts game are Elton John, Toby Keith and Lenny Kravitz. In King James, a new DC Comic book, LeBron James is crowned King of Basketball after dominating an underground tournament run by a "mysterious, secret organization." The cradle may rock, but Camden Yards won't. The Orioles backed out of an agreement that would have had Van Halen playing a concert at the stadium on Sept. 2. Not ones to slink away in their spandex, Eddie and the boys filed a breach-of-contract lawsuit against the team. The suit, which asks for at least $2 million, contends that Van Halen "had to change the dates of other scheduled concerts and forgo other concert opportunities in order to accommodate the Oriole Park concert." The team won a legal battle with the Maryland Stadium Authority three years ago for the right to book bands there as a means of bolstering stadium revenue. Last week real estate agent Terrye Jackson of Springdale, Md., set a world record for the longest continuous television-watching session by taking in 50 hours and seven minutes of NBC's Games coverage. Raiders rookie defensive end Andre Sommersell has learned the value of keeping his mouth shut. The former Colorado State defensive end made it known in camp that he wasn't going to put up with any rookie initiations. Bad move. On Aug. 17, following practice, his teammates taped him to a goalpost, doused him with water and coated him with talcum powder and abandoned him
SN - 0038822X
UR -
https://login.proxy.bib.uottawa.ca/login?url=http://search.ebscohost.com/login.aspx?direct=true&db=s3h&AN=14271686&site=ehost-live
ER -

TY - JOUR
ID - 4168
T1 - Its time for creative play
A1 - Beckwith,J.
Y1 - 1982///
KW - SportDiscus
KW - *RECREATION
KW - *PLAYGROUNDS
KW - *SAFETY
KW - PROGRAMS
KW - Awareness
KW - History
KW - DESIGN
KW - North America
KW - Child
KW - equipment
RP - NOT IN FILE
CY - ;
SN - 00312215

UR -
https://login.proxy.bib.uottawa.ca/login?url=http://search.ebscohost.com/login.aspx?direct=true&db=s3h&AN=SPH120542&site=ehost-live;http://articles.sirc.ca/search.cfm?id=120542
L2 - http://articles.sirc.ca/search.cfm?id=120542
ER -

TY - JOUR
ID - 4169
T1 - I'm nice, I'm smart, I like karate: girls with physical disabilities' perceptions of physical recreation
A1 - Bedini,L.A.
A1 - Anderson,D.M.
Y1 - 2005/06//
N1 - Accession Number: 2009016201. Language: English. Entry Date: 20050930. Revision Date: 20091218. Publication Type: journal article; research; tables/charts. Journal Subset: Allied Health; Peer Reviewed; USA. Special Interest: Pediatric Care; Physical Therapy; Psychiatry/Psychology; Women's Health. No. of Refs: 63 ref. NLM UID: 0230530
KW - CINAHL
KW - Physical Activity -- Psychosocial Factors -- In Adolescence
KW - Disabled -- Psychosocial Factors -- In Adolescence
KW - Attitude to Disability -- In Adolescence
KW - Female
KW - Child
KW - adolescence
KW - adolescent health
KW - child health
KW - Pilot Studies
KW - Qualitative Studies
KW - Phenomenological Research
KW - Role Models
KW - conceptual framework
KW - Women's Health
KW - Theoretical Sample
KW - Interview Guides
KW - Semi-Structured Interview
KW - Audiorecording
KW - Reflection
KW - thematic analysis
KW - Whites
KW - Blacks
KW - Recreation
KW - freedom
KW - Empowerment
KW - Body Image
KW - Interpersonal Relations
KW - Self Concept
KW - Descriptive Statistics
KW - Human
RP - NOT IN FILE
SP - 114
EP - 130
JF - Therapeutic Recreation Journal
VL - 39
IS - 2
N2 - Healthy People 2010: Objectives for Improving Health (USDHHS, 2003) stated that more people with disabilities and women report no leisure-time physical activities than people without disabilities and men. Similarly, children with disabilities are often not included in physical education and playground activities. Therefore, girls with physical disabilities are confronted with a "double whammy" of gender and disability to

compromise their self-image. While little research exists that explores physical activity for females with disabilities specifically, it has been found that physical activity is important for their quality of life, sense of control, and autonomy. This preliminary study was conducted using a phenomenological approach to examine the perceptions of girls with physical disabilities regarding physical recreation pursuits. Results from in-depth interviews generated three themes: meanings of physical activity, ownership of disability, and poor access to role models. Recommendations for alternate research methodologies as well as future content areas are discussed
SN - 0040-5914
AD - Department of Recreation, Parks, and Tourism, University of North Carolina, Greensboro, NC
UR - http://search.ebscohost.com/login.aspx?direct=true&db=cin20&AN=2009016201&site=ehost-live
ER -

TY - JOUR
ID - 4170
T1 - Student and parent self-reported changes in physical activity behavior while wearing an unsealed pedometer
A1 - Beets,M.W.
A1 - Eilert,A.G.
A1 - Pitetti,K.H.
A1 - Foley,J.T.
Y1 - 2006/11//
N1 - Accession Number: 2009372430. Language: English. Entry Date: 20080125. Revision Date: 20091218. Publication Type: journal article; research; tables/charts. Journal Subset: Allied Health; Biomedical; Peer Reviewed; USA. Special Interest: Pediatric Care; Physical Therapy. Instrumentation: Child Parent Reactivity Questionnaire--Pedometer (CPRQ-P) (Beets et al). No. of Refs: 11 ref. NLM UID: 8909729
KW - CINAHL
KW - Accelerometry -- In Infancy and Childhood
KW - Health Behavior -- In Infancy and Childhood
KW - Parental Attitudes
KW - Physical Activity -- In Infancy and Childhood
KW - Self Report -- In Infancy and Childhood
KW - Adult
KW - Child
KW - child health
KW - Descriptive Statistics
KW - Questionnaires
KW - Human
RP - NOT IN FILE
SP - 492
EP - 499
JF - Pediatric Exercise Science
JA - Pediatr Exerc Sci
VL - 18
IS - 4
N2 - Child--parent pairs (n = 109) completed a questionnaire assessing whether changes in normal physical activity levels occurred (child) or were observed (parent) when the children wore a pedometer for 7 days. Over two-thirds of the children (78.5%) and almost half of the parents (47.3%) indicated an increase in the child's normal physical activity. Reduced television viewing (75.2%) and increased outdoor play on the weekend (35.8%) were the most frequently reported means of behavior change as reported by the children and parents, respectively. Results indicate that both children and parents perceive a reactive effect to wearing an unsealed pedometer
SN - 0899-8493
AD - Department of Public Health, Oregon State University, Corvallis, OR
UR - http://search.ebscohost.com/login.aspx?direct=true&db=cin20&AN=2009372430&site=ehost-live
ER -

TY - JOUR

ID - 4173
T1 - Association of Father Involvement and Neighborhood Quality with Kindergartners' Physical Activity: A Multilevel Structural Equation Model
A1 - Beets,Michael W.
A1 - Foley,John T.
Y1 - 2008/01//
KW - SportDiscus
KW - *CHILDREN -- Health
KW - *PHYSICAL education for children
KW - FAMILIES
KW - PARENT & child
KW - FATHER & child
KW - Neighborhoods
KW - Child
KW - Prevention Research
KW - Safety
KW - Social Support
RP - NOT IN FILE
SP - 195
EP - 203
JF - American Journal of Health Promotion
JA - Am J Health Promot
VL - 22
IS - 3
N2 - Purpose. Examine the effects of father-child involvement and neighborhood characteristics with young children's physical activity (PA) within a multilevel framework. Design. Cross-sectional analysis of the Early Childhood Longitudinal Studyƒ_"Kindergarten Cohort 1998. Setting. Nationally representative sample. Subjects. Data were available for 10,694 kindergartners (5-6 years; 5240 girls) living in 1053 neighborhoods. Measures. Parental report of child's PA level, father characteristics (e.g., time spent with child, age, education, socioeconomic status, hours worked), family time spent doing sports/activities together, and neighborhood quality (e.g., safety, presence of crime violence, garbage). Child weight status, motor skills, ethnicity, and television viewing were used as covariates. Analysis. Multilevel structural equation modeling with children nested within neighborhoods. Results. At the child level father-child time and family time doing sports together were positively associated with children's PA. At the neighborhood level parental perception of a neighborhood's safety for children to play outside fully mediated the effect of neighborhood quality on children's PA. Overall 19.1% and 7.6% of the variance in PA was explained at the child and neighborhood levels, respectively. Conclusions. Family-based interventions for PA should consider father-child time, with this contributing to a child's overall PA level. Further, neighborhood quality is an important predictor of PA only to the extent by which parents perceive it to be unsafe for their child to play outdoors. ABSTRACT FROM AUTHOR
SN - 08901171
UR - https://login.proxy.bib.uottawa.ca/login?url=http://search.ebscohost.com/login.aspx?direct=true&db=s3h&AN=29999960&site=ehost-live
ER -

TY - JOUR
ID - 4174
T1 - Defining Standards and Policies for Promoting Physical Activity in Afterschool Programs
A1 - Beets,Michael W.
A1 - Wallner,Megan
A1 - Beighle,Aaron
Y1 - 2010/08//
KW - SportDiscus
KW - *AFTER-school sports
KW - *SPORTS for children
KW - *HEALTH education

KW - After School Programs
KW - HUMAN services programs
KW - STUDENT activities
KW - child and adolescent health
KW - Health Policy
KW - Program Planning
RP - NOT IN FILE
SP - 411
EP - 417
JF - Journal of School Health
JA - J Sch Health
VL - 80
IS - 8
N2 - BACKGROUND: National guidelines exist that define ƒ_oqualityƒ__ afterschool programs (3ƒ_"6pm, ASP). No widely adopted national standards/policies exist, however, for ASP providers for the promotion of physical activity (PA). To address this gap, state-level ASP organizations have developed or adopted standards/policies related to PA. The extent to which standards and policies are uniform and disseminated is unclear. The purpose of this study was to review state-level ASP documentation to identify standards/policies for promoting PA within the ASP setting. METHODS: A systematic review of state-level ASP organizations' standards/policies was conducted to identify documentation that included language explicitly defining or related to the promotion of physical activity within ASP. State-level ASP organizations were identified from registries available from national ASP organizations. Documentation was retrieved from online sources and reviewed using a standardized content analysis protocol. RESULTS: Quality ASP definitions were retrieved from 47 states. Of these, 14 incorporated language related to the promotion of PA. This included the provision of adequate outdoor space for attendees to be active, provision of frequent PA opportunities, and promoting physical development. A total of 5 states had standards/policies that explicitly identified the amount of time for attendees to be physically active during ASP attendance, while 3 states outlined standards/policies regarding core competencies for ASP providers to promote PA. CONCLUSIONS: Overall, standards/polices related to promoting PA within ASP were largely absent. Those that do exist, however, can provide a foundation for future empirical tests. The testing of these standards/policies will assist in the development of national guidelines to promote PA within the ASP setting. ABSTRACT FROM AUTHOR
SN - 00224391
UR -
https://login.proxy.bib.uottawa.ca/login?url=http://search.ebscohost.com/login.aspx?direct=true&db=s3h&AN=51938047&site=ehost-live
ER -

TY - JOUR
ID - 4177
T1 - Using Pedometers in Elementary Physical Education
A1 - Beighle,Aaron
A1 - Morgan,Charles F.
A1 - Pangrazi,Robert P.
Y1 - 2004/01//
KW - SportDiscus
KW - *PEDOMETERS
KW - *EQUIPMENT & supplies
KW - *PHYSICAL education
KW - *PHYSICAL education for children
KW - *PHYSICAL fitness
KW - *HEALTH
KW - DISTANCES
KW - Measurement
RP - NOT IN FILE
SP - 17
EP - 18
JA - Teaching Elementary Physical Education

VL - 15
IS - 1
N2 - Discusses the use of pedometers in physical education. Effectiveness of pedometers in enhancing instruction; description of the use of pedometers in examining daily physical activity outside of physical education; Steps in examining the effectiveness of the program in promoting physical activity outside of school
SN - 10454853
UR -
https://login.proxy.bib.uottawa.ca/login?url=http://search.ebscohost.com/login.aspx?direct=true&db=s3h&AN=11901673&site=ehost-live
ER -

TY - JOUR
ID - 4179
T1 - Emerging Adulthood and Patterns of Physical Activity Among Young Australian Women
A1 - Bell,Sandra
A1 - Lee,Christina
Y1 - 2005/12//
KW - SportDiscus
KW - *EXERCISE
KW - adulthood
KW - YOUNG adults
KW - YOUNG women
KW - Motherhood
KW - Australia
KW - emerging adulthood
KW - longitudinal
KW - Physical Activity
KW - Women
RP - NOT IN FILE
SP - 227
EP - 235
JF - International Journal of Behavioral Medicine
JA - Int J Behav Med
VL - 12
IS - 4
N2 - The transition from adolescence to young adulthood is associated with a sharp decline in physical activity, particularly for women. This article explores the relations between physical activity status and change and status and change in four life domains: residential independence, employment status, relationship status, and motherhood. Two waves of survey data from a representative sample of 8,545 Australian women, aged 18-23 at Survey 1 and 22-27 at Survey 2, were analyzed. Cross-sectionally, physical inactivity was most strongly related to being a mother, married, and not being in the labor force. Longitudinally, decreases in physical activity were most strongly associated with moving into a live-in relationship, with getting married, and with becoming a mother. When considered in combination, women who were married with children and not employed outside the home were the most likely to be physically inactive. The data suggest that adoption of adult statuses, particularly traditional roles involving family relationships and motherhood, is associated with reductions in physical activity for these women, although it is possible that the effect is driven by socioeconomic factors associated with early transitions. The data suggest a need for interventions to promote continued physical activity among young women who cohabit or marry and among those not in the workforce, in addition to those supporting young mothers to be physically active. Key words: physical activity, women, emerging adulthood, longitudinal ABSTRACT FROM AUTHOR
SN - 10705503
UR -
https://login.proxy.bib.uottawa.ca/login?url=http://search.ebscohost.com/login.aspx?direct=true&db=s3h&AN=18796626&site=ehost-live
ER -

TY - JOUR

ID - 4184
T1 - Back to Nature
A1 - Benyo,Rich
Y1 - 2004/09//
KW - SportDiscus
KW - *CAMPING
KW - *OUTDOOR recreation
KW - *SCHOOL children
KW - SCHOOL camps
KW - CATHOLIC schools
KW - Pennsylvania
KW - United States
RP - NOT IN FILE
SP - 8
EP - 11
JA - Marathon & Beyond
VL - 8
IS - 5
N2 - Presents an article about the summer camp experiences of the author at the end of his second-grade at St. Joe Catholic School in East Mauch Chunk, Pennsylvania in May 1952. Description of the students during the first day of the camp; Excitement felt by the campers; Camp activities
SN - 10886672
UR -
https://login.proxy.bib.uottawa.ca/login?url=http://search.ebscohost.com/login.aspx?direct=true&db=s3h&AN=14244436&site=ehost-live
ER -

TY - JOUR
ID - 4186
T1 - Desporto aventura e auto-estima nos adolescentes, em meio escolar. / Outdoor adventure activities and self-esteem in adolescent students
A1 - Bernardo,R.P.S.
A1 - Matos,M.G.
Y1 - 2003/01//
KW - SportDiscus
KW - *PHYSICAL fitness
KW - *PSYCHOLOGY
KW - *OUTDOOR education
KW - *ADVENTURE education
KW - *CARDIOVASCULAR fitness
KW - *STRESS (Physiology)
KW - PROGRAMS
KW - teenagers
KW - Surveys
KW - Portugal
KW - EFFECTIVENESS
KW - self-esteem
KW - PHYSICAL SELF-PERCEPTION PROFILE FOR CHILDREN & YOUTH
RP - NOT IN FILE
SP - 33
EP - 46
JA - Revista Portuguesa de Ciencias do Desporto
VL - 3
IS - 1
CY - ;
N2 - The development of sport and physical activity programs may improve psychological well-being and reduce risk factors of several health problems. Self-esteem has shown to have a strong position in the

explanation of human behaviour. The purpose of this study was to evaluate the effects of an Outdoor Adventure Activities Program on the self-esteem and physical self-perceptions of Portuguese school adolescents (n = 38). A translated version of the Physical Self-Perception Profile for Children and Youth (PSPP-CY) was used to assess self-esteem and physical self-perceptions. The main conclusions of this study showed that participants in general reported higher values on their self-esteem and physical self-perceptions after the program. Further experimental programs are required to confirm the results of this study
SN - 16450523
UR -
https://login.proxy.bib.uottawa.ca/login?url=http://search.ebscohost.com/login.aspx?direct=true&db=s3h&AN=SPHS-897613&site=ehost-live;http://articles.sirc.ca/search.cfm?id=S-897613;http://www.fcdef.up.pt
L2 - http://articles.sirc.ca/search.cfm?id=S-897613
ER -

TY - JOUR
ID - 4187
T1 - The Impact of Classroom Activity Breaks on the School-Day Physical Activity of Rural Children
A1 - Bershwinger,T.E.R.E.
A1 - Brusseau,Timothy A.
Y1 - 2012/10//
KW - SportDiscus
KW - *PHYSICAL fitness for children
KW - *HEALTH
KW - *PHYSICAL activity
KW - CLASSROOM activities
KW - SCHOOL day
KW - RURAL children
KW - LIFESTYLES & health
KW - accelerometer
KW - Health
KW - pedometer
KW - Physical Education
KW - school
KW - youth
RP - NOT IN FILE
SP - 134
EP - 143
JA - International Journal of Exercise Science
VL - 5
IS - 4
N2 - Schools have been identified as a central element in a system that ensures that children participate in enough physical activity to develop healthy lifestyles. Even with this recommendation, many school-based physical activity opportunities are being eliminated/minimized (i.e. physical education (PE), recess). With children spending much of their time at school, other supplemental physical activity opportunities are essential. One such opportunity is through classroom teacher led activity breaks. Very little evidence exists identifying the impact of classroom activity breaks on the daily physical activity patterns of children. Therefore, the purpose of this paper was to determine the impact of classroom teacher led activity breaks on the step counts and moderate-to- vigorous physical activity (MVPA) of 4th grade children at school. Participants in this pilot study included a 4th grade class (N=18; aged 9.2Añ.4 years; mostly Caucasian (n=14); BMI=19.9Añ2.8) in a rural school district in the Northeast US. Children wore the NL-1000 piezoelectric pedometer for 2-weeks (a baseline week and an activity break week) during the winter of 2012. Activity breaks were created by the teacher/students to give them ownership in the program (activities included numerous classroom games and walking activities). At baseline, children averaged 4806Añ639 steps and 17.7Añ2.8 minutes of MVPA/day. During implementation of the physical activity breaks, children significantly increased both their steps (p<.01; 5651Añ627; I"=845) and MVPA (p<.01; 22.3Añ3.5; I"=4.6 minutes). Findings suggest that classroom activity breaks have the potential to increase children's daily physical activity patterns at school. Future studies are needed with a larger and more diverse population to support these findings. ABSTRACT FROM AUTHOR
SN - 1939795X

UR -
https://login.proxy.bib.uottawa.ca/login?url=http://search.ebscohost.com/login.aspx?direct=true&db=s3h&AN=87560768&site=ehost-live
ER -

TY - JOUR
ID - 4189
T1 - Elementary school children's play behaviour during school recess periods
A1 - Beth-Halachmy,S.
Y1 - 1980///
KW - SportDiscus
KW - *PLAY
KW - *OUTDOOR life
KW - *RECESSES
KW - Social Interaction
KW - Elementary Schools
KW - Child
KW - behaviour
KW - SEX FACTOR
RP - NOT IN FILE
CY - ;
UR -
https://login.proxy.bib.uottawa.ca/login?url=http://search.ebscohost.com/login.aspx?direct=true&db=s3h&AN=SPH82104&site=ehost-live;http://articles.sirc.ca/search.cfm?id=82104
L2 - http://articles.sirc.ca/search.cfm?id=82104
ER -

TY - JOUR
ID - 4190
T1 - Always Look on the Bright Side of Life: Ego-Replenishing Effects of Daylight versus Artificial Light
A1 - Beute,F.
A1 - de Kort,Y.A.W.
KW - Reviewer Nominated
RP - NOT IN FILE
ER -

TY - JOUR
ID - 4191
T1 - Vitalize me! Overcoming ego-depletion by viewing bright and sunny nature
A1 - Beute,F.
A1 - de Kort,Y.A.W.
KW - Reviewer Nominated
RP - NOT IN FILE
ER -

TY - JOUR
ID - 4192
T1 - Let the sun shine! Measuring explicit and implicit preference for environments differing in naturalness, weather type and lightness
A1 - Beute,F.
A1 - de Kort,Y.A.W.
Y1 - 2013///
KW - Reviewer Nominated
RP - NOT IN FILE
JF - Journal of Environmental Psychology
PB - Elsevier
SN - 0272-4944

ER -

TY - JOUR
ID - 4193
T1 - No artificial ingredients added: Naturalness and the replenishment of ego-depletion by bright and sunny nature
A1 - Beute,Femke
A1 - de Kort,Yvonne AW
KW - Reviewer Nominated
RP - NOT IN FILE
ER -

TY - JOUR
ID - 4194
T1 - TLC for better PE: girls and elementary physical education
A1 - Beveridge,S.
A1 - Scruggs,P.
Y1 - 2000/10//
KW - SportDiscus
KW - *PHYSICAL education & training
KW - *PARTICIPATION
KW - *RECESSES
KW - *TEACHING
KW - girls
KW - BOYS
KW - ATTITUDE (Psychology)
KW - PROGRAMS
KW - Strategy
KW - Child
KW - SEX FACTOR
RP - NOT IN FILE
SP - 22
EP - 27
JA - JOPERD: The Journal of Physical Education, Recreation & Dance
VL - 71
IS - 8
CY - ;
SN - 07303084
UR -
https://login.proxy.bib.uottawa.ca/login?url=http://search.ebscohost.com/login.aspx?direct=true&db=s3h&AN=SPHS-663410&site=ehost-live;http://articles.sirc.ca/search.cfm?id=S-663410;http://www.aahperd.org/
L2 - http://articles.sirc.ca/search.cfm?id=S-663410
ER -

TY - JOUR
ID - 4195
T1 - A Lifetime of Memories
A1 - Bhonslay,Marianne
Y1 - 2007/12//
KW - SportDiscus
KW - *SPORTING goods industry
KW - *RECREATION -- Equipment & supplies
KW - *SPORT clothes
RP - NOT IN FILE
SP - 22
EP - 25
JA - SGB

VL - 40
IS - 12
N2 - The article takes a look at the childhood of Jim Chick, owner of Chick's Sporting Goods. Chick's father founded the store and Chick would visit every Saturday and play with the fishing rods. By the time he was 12 he was an avid outdoorsman who would hunt, fish, and do archery. In 1968, at age 21, Chick bought the business. Chick states that the advent of vendor-owned retail stores is the most significant industry challenge for retailers since 1968
SN - 15487407
UR -
https://login.proxy.bib.uottawa.ca/login?url=http://search.ebscohost.com/login.aspx?direct=true&db=s3h&AN=27962062&site=ehost-live
ER -

TY - JOUR
ID - 4196
T1 - Correlates of physical activity in youth: a review of quantitative systematic reviews
A1 - Biddle,Stuart J.H.
A1 - Atkin,Andrew J.
A1 - Cavill,Nick
A1 - Foster,Charlie
Y1 - 2011/03//
KW - SportDiscus
KW - *AGING
KW - *BODY image
KW - *CINAHL (Information retrieval system)
KW - *ECOLOGY
KW - *EXERCISE
KW - *MEDICAL care
KW - *MEDICINE
KW - *PSYCHOLOGY
KW - *MEDLINE
KW - *SELF-perception
KW - *SPORTS
KW - *BODY mass index
KW - adolescence
KW - PERSONAL beauty
KW - Demography
KW - ERIC (Information retrieval system)
KW - Ethnic Groups
KW - Health Services Accessibility
KW - INFORMATION storage & retrieval systems
KW - ONLINE information services
KW - PARENT & child
KW - SEX distribution (Demography)
KW - Social Values
KW - SYSTEMATIC reviews (Medical research)
KW - Cultural Values
KW - Socioeconomic Factors
KW - correlates
KW - Physical Activity
KW - systematic review
KW - young people
RP - NOT IN FILE
SP - 25
EP - 49
JA - International Review of Sport & Exercise Psychology
VL - 4

IS - 1
N2 - To increase young people's physical activity (PA) levels it is important to understand the correlates of PA in children and adolescents. We sought to identify factors associated with children's and adolescents' PA by reviewing systematic quantitative reviews of non-intervention research. Systematic reviews examining associations between quantitatively measured variables and PA in young people (< 19 years) from 2000-2010 were identified using electronic and manual searches. Nine systematic reviews were identified. Demographic/biological correlates of PA were age and gender. Psychological correlates of PA were positive motivation, positive body image and the existence of barriers to PA. Behavioural correlates of PA were previous PA, sport participation, smoking, and sedentary behaviour. Social/cultural correlates of PA were parental influence and social support, and environmental correlates of PA were access to facilities, distance from home to school, time spent outside, and local crime. The evidence is suggestive of a number of different types of correlates of PA for children and adolescents. Beyond age and gender, though, most are likely to have only small or small-to-moderate effects in isolation and may work best in interaction with other influences. Psychologists must look to social, organisational and community-level correlates in addition to individual correlates. ABSTRACT FROM AUTHOR
SN - 1750984X
UR -
https://login.proxy.bib.uottawa.ca/login?url=http://search.ebscohost.com/login.aspx?direct=true&db=s3h&AN=60041011&site=ehost-live
ER -

TY - JOUR
ID - 4197
T1 - Managing games: rules to play by
A1 - Bigelow,B.
A1 - Moroney,T.
A1 - Hall,L.
Y1 - 2001///
KW - SportDiscus
KW - *SPORTS
KW - *COACHING (Athletics)
KW - teenagers
KW - Management
KW - Parents
KW - adulthood
KW - Child
RP - NOT IN FILE
CY - ;
N2 - Presents a discussion on how to change the administration of youth sport to make it more child friendly. Includes ideas on how adults should conduct and coach youth sport. Section headings include: Don't organize too much; School recess model; Children learn as they play; Embrace mistakes; Trust the children; Put me in, coach; Minimum meaningful minutes; Create more teams; Winning with too high a price; Defer complicated strategies; What's appropriate, what's not; Make the practice more perfect; Watch how children play; Who's winning?; Special needs; and Somebody do something
SN - 1558749276
UR -
https://login.proxy.bib.uottawa.ca/login?url=http://search.ebscohost.com/login.aspx?direct=true&db=s3h&AN=SPHS-910109&site=ehost-live;http://articles.sirc.ca/search.cfm?id=S-910109
L2 - http://articles.sirc.ca/search.cfm?id=S-910109
ER -

TY - JOUR
ID - 4198
T1 - Recreation and the law. 2nd ed
A1 - Bird,S.
A1 - Zauhar,J.
Y1 - 1997///

KW - SportDiscus
KW - *SPORTS
KW - *RECREATION
KW - law
KW - Legislation
KW - Insurance
KW - Canada
KW - LIABILITY
RP - NOT IN FILE
CY - Scarborough, Ont.;
PB - Carswell/Thomson Professional Publishing
N2 - CONTENTS: [1] The legal powers: recreation and statute law, recreation and case law, recreation and the Charter of Rights; [2] The common law system: hierarchy of federal and provincial courts, the doctrine of precedent; [3] Types/forms of law: administrative law, constitutional law, international law, criminal law, contract law, the law of torts, property law, other types/forms of law; [4] Responsibility/liability: negligence, volenti, contributory negligence and waivers, occupiers' liability, children, nuisance, recreation employers, recreation employees, volunteers, unions, users/consumers of recreation; [5] Liability case law: amusement devices, buildings, firearms and hunting - accidental discharge - mistaken identity - interfering third party (parental liability), parks and playgrounds, participants - golf - gyms and health clubs - hockey - horse-riding - skating - skiing - snowmobiling - swimming - tobogganing - miscellaneous - nuisance, railways, rescuers, school activities and sports - supervision - premises and equipment, shopping malls and stores, spectators, streets, utilities, vacant lots; [6] Image; [7] Availability of materials and working equipment: stock/inventory, equipment/tools, transported goods; [8] Inspection procedures; [9] Policies, procedures, practices; [10] Facilities: physical resources, written - visual resources; [11] Energy-saving and computerization; [12] Volunteers; [13] Recreation management: activity programming, time management, in-service training, consultative services; [14] Insurance protection: risk management and insurance protection - vehicle insurance - property insurance - theft - air and water sports - vandalism - staff - volunteers - abuse protection - weather conditions - environmental liability - special conditions protection - umbrella or excess liabilty insurance - activity equipment - fixed equipment - buildings and liability - event insurance, summary; [15] Structure: departmental managerial model, auxiliary managerial model - option A - option B - option C - option D, customized managerial model, direct action managerial model, private managerial model, project approach model, multi-dimensional management model; [16] The recreation worker: job document specifics, activity definitions; [17] Staff selection and job performance: personnel selection - application forms - equality, performance appraisals, staff discipline; [Appendix] Discussion topics: chapter discussion, group discussion items, index
SN - 0459254367
UR -
https://login.proxy.bib.uottawa.ca/login?url=http://search.ebscohost.com/login.aspx?direct=true&db=s3h&AN=SPH418619&site=ehost-live
ER -

TY - JOUR
ID - 4201
T1 - Physical activity and motor fitness of children and adolescents -- Approaches for Serious Games
A1 - Boes,Klaus
A1 - Krell,Janina
Y1 - 2010/11//
KW - SportDiscus
KW - *PHYSICAL activity
KW - *MOTOR ability in children
KW - *VIDEO games
KW - *LEISURE
KW - *SPORTS -- Physiological aspects
KW - data analysis
KW - Motor Fitness
KW - Physical Activity
KW - Serious Games

RP - NOT IN FILE
SP - 18
EP - 26
JA - International Journal of Computer Science in Sport
VL - 9
IS - 2
N2 - Physical activity is of inestimable value for health and the motoric development of young people. But in the course of late childhood and adolescence, it becomes less important compared to other leisure activities which do not contain any physical strain. The decrease of physical activity and the resulting decline of motor fitness are reflected in the outcomes of various studies. Hence, strategies are needed to maintain the children's motivation in physical activity. The enormous popularity of Serious Games could possibly be one strategy because Exergames give an access to sport or to a more active lifestyle. As an isolated movement pattern, Serious Games are no alternative or even a surrogate for real physical exercise. It is difficult to enable a realistically social interaction during virtual sport which has a positive influence on social development. Furthermore indoor video gaming is not comparable to outdoor exercises due to the lack of external influences like wind or audience, which are important contributions to the "adventure" of sport. But in combination and addition to real sport, Serious Games can contribute to the preservation and increase of motor fitness and children's pleasure in motion. ABSTRACT FROM AUTHOR
SN - 16844769
UR -
https://login.proxy.bib.uottawa.ca/login?url=http://search.ebscohost.com/login.aspx?direct=true&db=s3h&AN=57463887&site=ehost-live
ER -

TY - JOUR
ID - 4202
T1 - Socialized recess: conflict resolution model
A1 - Boland,C.J.
Y1 - 1996///Spring
KW - SportDiscus
KW - *PLAY
KW - *RECESSES
KW - Case Studies
KW - Socialization
KW - PHILADELPHIA (Pa.)
KW - Child
RP - NOT IN FILE
SP - 25
EP - 26
JA - Pennsylvania Journal of Health, Physical Education, Recreation & Dance
VL - 66
IS - 2
CY - ;
SN - 02790033
UR -
https://login.proxy.bib.uottawa.ca/login?url=http://search.ebscohost.com/login.aspx?direct=true&db=s3h&AN=SPH394101&site=ehost-live;http://articles.sirc.ca/search.cfm?id=394101
L2 - http://articles.sirc.ca/search.cfm?id=394101
ER -

TY - JOUR
ID - 4205
T1 - Shoulder Injuries Among United States High School Athletes During the 2005-2006 and 2006-2007 School Years
A1 - Bonza,John E.
A1 - Fields,Sarah K.
A1 - Yard,Ellen E.

A1 - Comstock,R.Dawn
Y1 - 2009/01//
KW - SportDiscus
KW - *SHOULDER -- Wounds & injuries
KW - *SPORTS injuries
KW - *ATHLETES
KW - *HIGH school athletes
KW - *SPORTS competitions
KW - *FRACTURES
KW - Epidemiology
KW - injury surveillance
RP - NOT IN FILE
SP - 76
EP - 83
JA - Journal of Athletic Training
VL - 44
IS - 1
N2 - Context: The shoulder is one of the most commonly injured body sites among athletes. Little previous research describes shoulder injury patterns in high school athletes. Objective: To describe and compare shoulder injury rates and patterns among high school athletes in 9 sports (football, soccer, basketball, baseball, and wrestling for boys and soccer, volleyball, basketball, and softball for girls). Design: Prospective injury surveillance study. Setting: Injury data were collected from 100 nationally representative US high schools via High School Reporting Information Online. Patients or Other Participants: Athletes from participating high schools injured while involved in a school-sanctioned practice or competition in 1 of the above sports during the 2005-2006 and 2006-2007 school years. Main Outcome Measure(s): Shoulder injury rates, diagnoses, severity, and mechanisms. Results: During the 2005-2006 and 2005-2007 school years, athletes in this study sustained 805 shoulder injuries during 3 550 141 athlete-exposures (AEs), for an injury rate of 2.27 shoulder injuries per 10 000 AEs. This corresponds to an estimated 232 258 shoulder injuries occurring nationwide during this time. Shoulder injuries were more likely to occur during competition than practice (rate ratio = 3.01, 95% confidence interval = 2.62, 3.46). Shoulder injury rates per 10 000 AEs were highest in football (5.09), wrestling (4.34), and baseball (1.90). Common shoulder injury diagnoses included sprains/ strains (39.6%), dislocations/separations (23.7%), contusions (11.5%), and fractures (6.6%). Although 44.8% of athletes sustaining a shoulder injury returned to play in less than 1 week, 22.9% were out of play for more than 3 weeks, and 6.2% of shoulder injuries required surgery. Common mechanisms of shoulder injury included player-to-player contact (57.6%) and contact with the playing surface (22.8%). Conclusions: High school shoulder injury rates and patterns varied by sport. Continued surveillance is warranted to understand trends and patterns over time and to develop and evaluate evidence-based preventive interventions. ABSTRACT FROM AUTHOR
SN - 10626050
UR -
https://login.proxy.bib.uottawa.ca/login?url=http://search.ebscohost.com/login.aspx?direct=true&db=s3h&AN=37588137&site=ehost-live
ER -

TY - JOUR
ID - 4206
T1 - Birth of a New Phenomenon
A1 - Bordeau,Topher
Y1 - 2010/12//
KW - SportDiscus
KW - *CHILDBIRTH
KW - *ROWERS
KW - *TESTOSTERONE
KW - *DIVERS
KW - Research
KW - TEST pilots
KW - ASTRONAUTS
KW - DAUGHTERS

RP - NOT IN FILE
SP - 60
EP - 60
JA - Rowing News
IS - 10
N2 - The article discusses the author's observation that rowers she knows tend to give birth to baby girls. She refers to a 2003 article from "New Scientist" about studies that found that male professional divers, test pilots and astronauts among other professions, are likely to have daughters rather than sons. One explanation for the phenomenon posited that men in these active and outdoor jobs might have higher testosterone levels
SN - 1548694X
UR -
https://login.proxy.bib.uottawa.ca/login?url=http://search.ebscohost.com/login.aspx?direct=true&db=s3h&AN=55668631&site=ehost-live
ER -

TY - JOUR
ID - 4208
T1 - The Epidemiology of US High School Basketball Injuries, 2005-2007
A1 - Borowski,Laurel A.
A1 - Yard,Ellen E.
A1 - Fields,Sarah K.
A1 - Comstock,R.Dawn
Y1 - 2008/12//
KW - SportDiscus
KW - *BASKETBALL
KW - *ACCIDENTS
KW - *EPIDEMIOLOGY
KW - *HIGH schools
KW - *SPORTS injuries
KW - *GENDER
KW - *WOUNDS & injuries
KW - *LIGAMENTS -- Wounds & injuries
KW - *ATHLETES
KW - Research
KW - United States
KW - Basketball
KW - Epidemiology
KW - gender
KW - high school
KW - injury
KW - surveillance
RP - NOT IN FILE
SP - 2328
EP - 2335
JF - American Journal of Sports Medicine
JA - Am J Sports Med
VL - 36
IS - 12
N2 - Background: With more than a million high school athletes playing during the 2006-2007 academic year, basketball is one of the most popular sports in the United States. Hypothesis: Basketball injury rates and patterns differ by gender and type of exposure. Study Design: Descriptive epidemiology study. Methods: Basketball-related injury data were collected during the 2005-2006 and 2006-2007 academic years from 100 nationally representative US high schools via Reporting Information Online. Results: High school basketball players sustained 1518 injuries during 780 651 athlete exposures for an injury rate of 1.94 per 1000 athlete exposures. The injury rate per 1000 athlete exposures was greater during competition (3.27) than during practice (1.40; rate ratio, 2.33; 95% confidence interval, 2.10-2.57) and was greater among girls (2.08) than among boys (1.83; rate ratio, 1.14; 95% confidence interval, 1.03-1.26). The ankle/foot (39.7%), knee (14.7%), head/face/neck (13.6%),

arm/hand (9.6%), and hip/thigh/upper leg (8.4%) were most commonly injured. The most frequent injury diagnoses were ligament sprains (44.0%), muscle/tendon strains (17.7%), contusions (8.6%), fractures (8.5%), and concussions (7.0%). Female basketball players sustained a greater proportion of concussions (injury proportion ratio, 2.41; 95% confidence interval, 1.49-3.91) and knee injuries (injury proportion ratio, 1.71; 95% confidence interval, 1.27-2.30), whereas boys more frequently sustained fractures (injury proportion ratio, 1.87; 95% confidence interval, 1.27-2.77) and contusions (injury proportion ratio, 1.52; 95% confidence interval, 1.00-2.31). The most common girls' injury requiring surgery was knee ligament sprains (47.9%). Conclusion: High school basketball injury patterns vary by gender and type of exposure. This study suggests several areas of emphasis for targeted injury prevention interventions. ABSTRACT FROM AUTHOR
SN - 03635465
UR -
https://login.proxy.bib.uottawa.ca/login?url=http://search.ebscohost.com/login.aspx?direct=true&db=s3h&AN=35847533&site=ehost-live
ER -

TY - JOUR
ID - 4209
T1 - Blood Lead Levels in Egyptian Children: Influence of Social and Environmental Factors
A1 - Boseila,Samia A.
A1 - Gabr,Azza A.
A1 - Hakim,Iman A.
Y1 - 2004/01//
KW - SportDiscus
KW - *ENVIRONMENTAL health
KW - *BLOOD -- Analysis
KW - LEAD poisoning in children
KW - HOUSING & health
KW - Regression Analysis
KW - Egypt
RP - NOT IN FILE
SP - 47
EP - 49
JF - American Journal of Public Health
JA - Am J Public Health
VL - 94
IS - 1
N2 - The article presents information on a study that examined the social variables predictive of higher lead levels in young Egyptian children. Children were recruited from 2 different areas in Giza, Egypt, a rural village and a lower-middle-class urban district. Houses were evaluated with respect to building materials, pavement, condition of paint, sleeping site of children and the type of floor in the house. Blood specimens were analyzed for lead by flameless atomic absorption at the Air and Industrial Hygiene Laboratory, California State Department of Health Services in Berkeley, California. Results of multiple linear regression analysis showed that blood lead levels (PbB) was significantly affected by age, traffic, mother's occupation, sleep site and street pavement. In the general population, children under the age of 5 have the highest PbB because of increased gastrointestinal absorption and exposure through behaviors such as playing outdoors and increased hand-to-mouth activity. The differences in the prevalence of elevated PbB levels between rural and urban children are likely to represent differences in housing characteristics and environmental exposure. Lead-contaminated soil and dust in high-traffic areas significantly contribute to the children's lead intake
SN - 00900036
UR -
https://login.proxy.bib.uottawa.ca/login?url=http://search.ebscohost.com/login.aspx?direct=true&db=s3h&AN=11952686&site=ehost-live
ER -

TY - JOUR
ID - 4210
T1 - A Perfect Playground--Fun for Everyone

A1 - Bossenmeyer,Melinda
A1 - Robertson,Karen
Y1 - 2002///Spring
KW - SportDiscus
KW - *PLAYGROUNDS
KW - SAFETY measures
KW - PLAY environments -- Safety measures
KW - CHILDREN'S playhouses
KW - CALIFORNIA,Southern
RP - NOT IN FILE
SP - 20
JA - Palaestra
VL - 18
IS - 2
N2 - Provides information on the Peaceful Playgrounds program designed to ensure playground safety and accessibility in South California. Background on the establishment of the program; Program guidelines; Benefits of the program to students
SN - 87565811
UR -
https://login.proxy.bib.uottawa.ca/login?url=http://search.ebscohost.com/login.aspx?direct=true&db=s3h&AN=6867599&site=ehost-live
ER -

TY - JOUR
ID - 4211
T1 - Physical activity patterns of children with movement difficulties
A1 - Bouffard,M.
A1 - Thompson,L.P.
A1 - Watkinson,E.J.
Y1 - 1992///
KW - SportDiscus
KW - *MOVEMENT disorders
KW - Child
KW - DISABILITIES-Motor impairments
RP - NOT IN FILE
CY - Gloucester, Ont.;
PB - Canadian Fitness and Lifestyle Research Institute
N2 - In this project, four interrelated studies were conducted to test the activity deficit hypothesis with children experiencing movement difficulties. This hypothesis states that children with movement difficulties are less physically active than children without movement difficulties. In two observational studies, data were collected on children with movement difficulties and children without movement difficulties during a regular physical education class and recess time at school. During regular physical education, children with movement difficulties were more often (a) not motor engaged when they are supposed to be and (b) engaged in a motor activity but in the wrong activity for the task. During recess time, it was found that children with movement difficulties were (a) less often vigorously active, (b) less often playing with large playground equipment (requiring a higher level of movement proficiency), (c) not observable for significantly more time, and (d) spent less time in positive social interactions with others of their own gender. A free leisure-time pursuit was administered during the third study. Overall, no major differences were found between children with and without movement difficulties. Finally, in the fourth study the exercise intensity level of children with movement difficulties was compared to that of children without movement difficulties during one weekend day. The intensity of physical activity was estimated by continuous monitoring of minute-by-minute heart rates using the Sport Tester PE 3000, a self-contained computerized telemetry system. No significant differences were found between both groups of children. Overall, it was found that the best setting to test the activity deficit hypothesis was a relatively free, unstructured play situation, in which the participants were free to choose participation. The setting having most of these characteristics was recess time. Accordingly, we suggest that the anecdotal evidence and current data support the activity hypothesis which is based on recent motivational theories. Children with movement difficulties have, on the average, developed a dangerous lifestyle. They have

developed a style that puts skill development, social interaction, health and fitness at risk. Implications of these results for fitness and lifestyle as well as future research needs are presented
Au moyen de quatre etudes, le but de ce projet etait de verifier si dans la vie courante l'hypothese d'hypoactivite chez les enfants maladroits est supportee. Selon cette hypothese, les enfants maladroits evitent plus souvent les situations de mouvement que leurs pairs du meme age n'ayant pas de problemes de justesse motrice. pendant deux etudes observationnelles, des enfants maladroits ainsi que des enfants n'ayant pas de problemes de justesse motrice furent observes pendant une periode d'education physique a l'ecole ainsi que pendant la recreation. Les resultats de nos observations pendant la periode d'education physique indiquerent que les enfants maladroits, comparativement aux enfants temoin, (a) accomplissaient moins souvent la tache demandee, et (b) modifiaient la tache de facon telle qu'une autre tache etait accomplie. La seconde etude indiqua que pendant la periode de recreation les enfants maladroits (a) participaient moins souvent a des activites vigoureuses, (b) jouaient moins souvent avec de grosses pieces d'equipement situe sur le terrain de jeu, (c) etaient moins souvent visibles, et (d) passaient moins de temps en interactions positives avec leurs pairs du meme sexe. Un questionnaire portant sur les activitees pratiquees durant les loisirs fut administre lors de la troisieme etude. Dans l'ensemble, ce questionnaire n'a pas revele de differences significatives entre les goupes. Finalement, la quatrieme etude compara l'intensite de la participation de ces enfants durant une journee de fin de semaine. L'intensite de l'activite fut mesuree a l'aide du Sport Tester PE 3000. Nous n'avons pas obtenu de differences significatives entre ces groupes. Dans l'ensemble, nous croyons que le meilleur environement pour tester l'hypothese d'hypoactivite etait la recreation. C'est dans ce milieu non structure que les enfants etaient libres de choisir ce qu'ils desiraient faire ainsi que l'entensite de leur participation. Consequemment, nous concluons, en accord avec de nombreuses anecdotes ainsi que de recentes theories de la motivation, que l'hypothese d'hypoactivite fut supportee dans ce projet. Les enfants maladroits ont, en general, developpe un style de vie dangereux pour l'apprentissage de gestes, l'interaction sociale, ainsi que leur sante et leur condition physique. Les implications de ces resultats pour la condition physique et le mode de vie ainsi que les recherches ulterieures sont presentes
UR -
https://login.proxy.bib.uottawa.ca/login?url=http://search.ebscohost.com/login.aspx?direct=true&db=s3h&AN=SPH327774&site=ehost-live
ER -

TY - JOUR
ID - 4217
T1 - THE CHARACTERISTICS OF SEXUAL ABUSE IN SPORT: A MULTIDIMENSIONAL SCALING ANALYSIS OF EVENTS DESCRIBED IN MEDIA REPORTS
A1 - Brackenridge,Celia H.
A1 - Bishopp,Daz
A1 - Moussalli,Sybille
A1 - Tapp,James
Y1 - 2008/12//
KW - SportDiscus
KW - *SPORTS
KW - *ATHLETES
KW - SEX crimes
KW - CRIMES against
KW - SEX offenders
KW - RAPISTS
KW - Behavioral Assessment
KW - media
KW - multidimensional scaling
KW - sex abuse
KW - sport
RP - NOT IN FILE
SP - 385
EP - 406
JA - International Journal of Sport & Exercise Psychology
VL - 6
IS - 4

N2 - Most research on sexual abuse has been conducted within family settings (Fergusson & Mullen, 1999). In recent years, following several high profile convictions and scandals, research into sexual abuse has also encompassed institutional and community settings, such as sport and the church (Gallagher, 2000; Woolfe, Jaffe, Jette, & Poisson, 2003). Research into sexual abuse in sport, for example, began with both prevalence studies (Kirby & Greaves, 1996; Leahy, Pretty, & Tenenbaum, 2002) and qualitative analyses of the processes and experiences of athlete sexual abuse (Brackenridge, 1997; Cense & Brackenridge, 2001, Toftegaard-Nielsen, 2001). From such work, descriptions of the modus operandi of abusers in sport, and the experiences and consequences for athlete victims, have been provided, informing both abuse prevention work and coach education. To date, however, no study has provided empirical support for multiple associations or identified patterns of sex offending in sport in ways that might allow comparisons with research-generated models of offending outside sport. This paper reports on an analysis of 159 cases of criminally defined sexual abuse, reported in the print media over a period of 15 years. The main aim of the study was to identify the nature of sex offending in sport, focusing on the methods and locations of offenses. The data were analysed using multidimensional scaling (MDS) as a data reduction method in order to identify the underlying themes within the abuse and explore the inter-relationships of behavior, victim, and context variables. The findings indicate that there are specific themes that can be identified within the perpetrator strategies that include "intimate," "aggressive," and "dominant" modes of interaction. The same patterns that are described here within the specific context of sport are consistent with themes that emerge from similar behavioral analyses of rapist (Bishopp, 2003; Canter & Heritage, 1990) and child molester groups (Canter, Hughes, & Kirby, 1998). These patterns show a correspondence to a broader behavioral model--the interpersonal circumplex (e.g., Leary, 1957). Implications for accreditation and continuing professional education of sport psychologists are noted. ABSTRACT FROM AUTHOR
SN - 1612197X
UR - https://login.proxy.bib.uottawa.ca/login?url=http://search.ebscohost.com/login.aspx?direct=true&db=s3h&AN=35827870&site=ehost-live
ER -

TY - JOUR
ID - 4218
T1 - Should recess be more structured and supervised in order to increase students' physical activity?
A1 - Brinkman,Bruce
A1 - Cook,Kendall L.
A1 - Kahan,David
A1 - Elliott,Gloria
A1 - Myrick,Steve
A1 - Greene,Babs
A1 - Snow,Jeffrey
A1 - Stein,Julian U.
A1 - Springs,Oliver
Y1 - 2005/11//
KW - SportDiscus
KW - *SCHOOL recess breaks
KW - *PHYSICAL education
KW - *OUTDOOR recreation
KW - *STUDENTS
KW - *EDUCATION
KW - *PLAYGROUNDS
RP - NOT IN FILE
SP - 9
EP - 49
JA - JOPERD: The Journal of Physical Education, Recreation & Dance
VL - 76
IS - 9
N2 - The article questions that should recess be more structured and supervised in order to increase students' physical activities. According to Bruce Brinkman, elementary physical education staff developer, Jordan School District, Utah some students with unstructured recess sometimes choose to do little or nothing. He believes that

structured recess is the simple answer to many elementary school problems. Their ultimate purpose as physical educators should be to guide children into being physically active for a lifetime. Unstructured free time does not guide children into anything. With structure, proper supervision, and choice, the students can practice the skills that they have learned in physical education, increase the time they spend being physically active, and have a safe and social atmosphere on the playground. Kendall L. Cook, physical education teacher at Walnut Grove Elementary School at Georgia State University, Atlanta, Georgia states that in schools today, children are constantly told what to do and when to do it. She feels that there should be more room for individuality and more time for kids to be themselves
SN - 07303084
UR -
https://login.proxy.bib.uottawa.ca/login?url=http://search.ebscohost.com/login.aspx?direct=true&db=s3h&AN=18926336&site=ehost-live
ER -

TY - JOUR
ID - 4220
T1 - How much physical activity a week to improve the health-related fitness of Polish schoolchildren? / W jakim stopniu aktywnosc fizyczna w ciagu tygodnia poprawia sprawnosc fizyczna polskich uczniow?
A1 - Bronikowski,Michal
Y1 - 2005///
KW - SportDiscus
KW - *PHYSICAL fitness
KW - *HEART beat
KW - *STRESS (Physiology)
KW - *EXERCISE -- Physiological aspects
KW - *CARDIOVASCULAR fitness
KW - *PHYSICAL education & training
KW - Calibration
KW - teenagers
KW - EDUCATION,Secondary
KW - Poland
KW - EFFECTIVENESS
KW - EVALUATION STUDY
RP - NOT IN FILE
SP - 219
EP - 223
JA - Wychowanie Fizyczne i Sport
VL - 49
IS - 3
CY - ;
N2 - Study aim: To compare the distributions of activities under two schedules of physical education (PE) classes in Poland. Material and methods: Two schedules were examined: 3 and 4 PE classes a week, lasting 45 mm each. The study was conducted in years 2002-2004 in one junior secondary school in Poznan. Heart rates of randomly selected children aged 13 years were monitored during physical education classes throughout one half-year term and the results classified into 4 exercise intensity zones. Results: The recommended volume and intensity of activities, i.e. at least 50 % of time at an intensity of at least 50 % of heart rate reserve (HRR) were recorded only for boys performing outdoor athletics and basketball. Conclusions: Like in other studies, physical education classes alone were shown to be of insufficient intensity to improve the health-directed fitness
SN - 00439630
UR -
https://login.proxy.bib.uottawa.ca/login?url=http://search.ebscohost.com/login.aspx?direct=true&db=s3h&AN=SPHS-1043640&site=ehost-live;http://articles.sirc.ca/search.cfm?id=S-1043640;http://www.awf.edu.pl
L2 - http://articles.sirc.ca/search.cfm?id=S-1043640
ER -

TY - JOUR
ID - 4222

T1 - Effects of teacher-encouraged physical activity on preschool playgrounds
A1 - Brown,W.H.
A1 - Googe,H.S.
A1 - McIver,K.L.
A1 - Rathel,J.M.
Y1 - 2009/03//
N1 - Accession Number: 2010248998. Language: English. Entry Date: 20090612. Revision Date: 20120309. Publication Type: journal article; research; tables/charts. Journal Subset: Allied Health; Blind Peer Reviewed; Editorial Board Reviewed; Peer Reviewed; USA. Special Interest: Pediatric Care. Grant Information: Partially supported by a Research and Productive Scholars Grant from the University of South Carolina, Grant H325D010063A from the Office of Special Education Programs of the U.S. Department of Education, and Grant R01 HD43125-01 from the National Institute of Child Health and Development, National Institutes of Health. No. of Refs: 58 ref. NLM UID: 8910748
KW - CINAHL
KW - Faculty-Student Relations
KW - Health Promotion -- In Infancy and Childhood
KW - Physical Activity -- In Infancy and Childhood
KW - Play and Playthings -- In Infancy and Childhood
KW - Schools,Nursery
KW - Child Day Care
KW - Child,Preschool
KW - convenience sample
KW - Dancing
KW - Female
KW - Funding Source
KW - interrater reliability
KW - Male
KW - Motivation
KW - Physical Education and Training
KW - Program Development
KW - Running
KW - Teachers
KW - Teaching Methods
KW - Walking
KW - Human
RP - NOT IN FILE
SP - 126
EP - 145
JF - Journal of Early Intervention
VL - 31
IS - 2
N2 - Increased numbers of young children who are overweight have become a significant health problem in the United States. Public health investigators have hypothesized that increased caloric intake and low levels of physical activity may be associated with the troubling trend of weight problems among children. To enhance preschool children's moderate to vigorous physical activity on playgrounds the authors develop, implement, and evaluate two practical teacher-implemented activities. Direct observations of five children in two preschool programs during teacher-implemented activities indicate increased moderate to vigorous physical activity on intervention days relative to nonintervention days (i.e., business-as-usual conditions). The results and implications for preschool practitioners are discussed
SN - 1053-8151
AD - University of South Carolina, Columbia
UR - http://search.ebscohost.com/login.aspx?direct=true&db=cin20&AN=2010248998&site=ehost-live
ER -

TY - JOUR
ID - 4223

T1 - Assessing Preschool Children's Physical Activity: The Observational System for Recording Physical Activity in Children-Preschool Version
A1 - Brown,William H.
A1 - Pfeiffer,Karin A.
A1 - McIver,Kerry L.
A1 - Dowda,Marsha
A1 - Almeida,M.Joao
A1 - Pate,Russell R.
Y1 - 2006/06//
KW - SportDiscus
KW - *PHYSICAL fitness
KW - *CARDIOVASCULAR fitness
KW - *STRESS (Physiology)
KW - INFANTS
KW - Calibration
KW - Methodology
KW - Observation
KW - Test Reliability
KW - OBSERVATIONAL SYSTEM FOR RECORDING PHYSICAL ACTIVITY IN CHILDREN-PRESCHOOL VERSION
RP - NOT IN FILE
SP - 167
EP - 176
JF - Research Quarterly for Exercise & Sport
JA - Res Q Exerc Sport
VL - 77
IS - 2
CY - ;
N2 - In this paper we present initial information concerning a new direct observation system - the Observational System for Recording Physical Activity in Children Preschool Version. The system will allow researchers to record young children's physical activity levels while also coding the topography of their physical activity, as well as detailed indoor and outdoor social and nonsocial contextual information. With respect to interobserver agreement (IOA), the kappa and category-by-category agreement mean of those obtained for the three illustrative preschools were generally above. 80. Hence, our IOA data indicated that trained observers in the three preschools frequently agreed on the eight observational categories and accompanying codes. The results for preschoolers' level of physical activity indicated they spent the majority of observational intervals in sedentary activity (i.e., more than 80 % intervals) and were observed in moderate to vigorous physical activity much less frequently (i.e., 5 % or fewer intervals). For the 15 indoor and 12 outdoor activity contexts, variability across both the activity contexts and the three preschools were evident. Nevertheless, three classroom contexts - transition, snacks, and naptime - accounted for the greatest proportion of coded activity contexts for the children. In the three preschools, 4 of 17 physical activity types - sit and squat, lie down, stand, and walk - accounted for the topography of much of children's physical activity behavior. Systematic observation of more representative preschool samples might better inform our present understanding of young children's physical activity in community preschool programs. [ABSTRACT FROM AUTHOR]
SN - 02701367
UR - https://login.proxy.bib.uottawa.ca/login?url=http://search.ebscohost.com/login.aspx?direct=true&db=s3h&AN=SPHS-1026923&site=ehost-live;http://articles.sirc.ca/search.cfm?id=S-1026923;http://www.aahperd.org/
L2 - http://articles.sirc.ca/search.cfm?id=S-1026923
ER -

TY - JOUR
ID - 4226
T1 - Fathers' child injury prevention attitudes and practices
A1 - Brussoni,M.
A1 - Olsen,L.
A1 - Sheftel,D.

A1 - George,A.
Y1 - 2010/09/02/
KW - SportDiscus
RP - NOT IN FILE
SP - A74
EP - A74
JF - Injury Prevention
JA - Inj Prev
VL - 16
N2 - Introduction Fathers have a major impact on various aspects of child health and development yet little is known about their roles in preventing injuries the most significant risk to their childrens health. While parents play a large role in mitigating childhood injury risks, particularly for younger children, the role of each parent differs substantially and requires investigation. Purpose Using a qualitative approach to develop to enhance our understanding of fathers safety-related attitudes and practices. Method Interviews were conducted with fathers of children aged 2ƒ_"7 years in British Columbia, Canada. Questions addressed fathers roles and typical activities with their children, concerns regarding child safety, safety practices, and access of safety-related resources. Grounded theory methods guided data analysis. Results A diverse sample of 32 fathers was interviewed. Central themes expressed by fathers included: exposure to risk plays an important role in childrens lives; accidents are part of life; not all injuries can be prevented; preventing serious injuries is important; injuries can represent learning experiences. Fathers focused on outdoor risk, rather than home-based risks and used supervision as an important strategy for injury prevention. Conclusion Fathers attitudes and practices are important to consider in designing prevention programs. Building on perceptions that promote injury prevention (eg, use of supervision), and considering perceptions that challenge the perceived need for prevention in programming (eg, acceptance of injuries and risk) can help ensure the relevance and success of messages. ABSTRACT FROM PUBLISHER
SN - 13538047
UR -
https://login.proxy.bib.uottawa.ca/login?url=http://search.ebscohost.com/login.aspx?direct=true&db=s3h&AN=66042547&site=ehost-live
ER -

TY - JOUR
ID - 4227
T1 - Achieving a safe ratio on the playground
A1 - Bruya,L.D.
A1 - Wood,G.
Y1 - 1998/04//
KW - SportDiscus
KW - *PLAYGROUNDS
KW - *CHILDREN -- Wounds & injuries
KW - *PREVENTION
KW - SAFETY measures
RP - NOT IN FILE
SP - 74
JA - Parks & Recreation
VL - 33
IS - 4
N2 - Focuses on how adults can supervise and encourage children to play safely. Concerns regarding the injury rates on playgrounds; Role of adults in making playgrounds safe for children; Importance of appropriate supervisor-to-child ratio in playgrounds
SN - 00312215
UR -
https://login.proxy.bib.uottawa.ca/login?url=http://search.ebscohost.com/login.aspx?direct=true&db=s3h&AN=496583&site=ehost-live
ER -

TY - JOUR

ID - 4228
T1 - Risk Factor One: Supervision on a Safe Playground
A1 - Bruya,Lawrence
A1 - Bruya,Lorna
Y1 - 2000/03//
KW - SportDiscus
KW - *PLAYGROUNDS
KW - SAFETY measures
KW - SUPERVISORS
KW - PLAY environments
RP - NOT IN FILE
SP - 20
JA - JOPERD: The Journal of Physical Education, Recreation & Dance
VL - 71
IS - 3
N2 - Discusses the elements of good supervision for the safety of children in school playgrounds. Supervisor-child ratio in playgrounds; Importance of the daily inspection of school playgrounds; Patterns of supervisor behavior that influence the safety of children; Methods used in supervisor communication systems
SN - 07303084
UR -
https://login.proxy.bib.uottawa.ca/login?url=http://search.ebscohost.com/login.aspx?direct=true&db=s3h&AN=4701251&site=ehost-live
ER -

TY - JOUR
ID - 4231
T1 - Leisure time physical activity participation among Latino visitors to outdoor recreation areas
A1 - Burk,Brooke N.
A1 - Shinew,Kimberly J.
A1 - Stodolska,Monika
Y1 - 2011/08//
KW - SportDiscus
KW - *LEISURE
KW - *PHYSICAL activity
KW - *PARTICIPATION
KW - *OUTDOOR recreation
KW - Social Support
KW - Hispanic Americans
KW - United States
KW - Attitude
KW - Attitudes
KW - constraints
KW - contraintes
KW - soutien social
RP - NOT IN FILE
SP - 325
EP - 338
JA - Leisure/Loisir: Journal of the Canadian Association for Leisure Studies
VL - 35
IS - 3
N2 - Previous research indicates that Latinos have low rates of leisure time physical activity (LTPA). As a result, Latinos suffer from higher rates of obesity and other diseases associated with this lack of activity. Thus, the primary purpose of this exploratory study was to gain a greater understanding of LTPA among Latinos. The goals of this study were threefold: (1) gain an understanding of the similarities and differences between Latino male and female LTPA levels and their levels of walking (done in a variety of settings); (2) examine the types of environments where their LTPA occurs; and (3) gain insight into Latinos' attitudes and perceptions of social support and constraints to LTPA. The study included Latinos from two suburban Chicago communities. Data

were collected at local parks and in a sports complex. The findings indicated that 39.1% of the Latinos had engaged in vigorous LTPA 2.75 days in the previous week, and men were active for longer periods of time than were women (F = 6.47; p = 0.021). Additionally, 38.6% of the Latinos had engaged in moderate LTPA an average of 2.57 days in a week. The majority of participants indicated they walked at least 10 minutes per day (77.5%), and men spent significantly more time walking than did women (F = 47.17; p = 0.001). Men were more likely to exercise in parks whereas women were more likely to exercise at home. Most Latinos indicated high levels of social support for exercise, and men found LTPA more interesting than did women (F = 4.23; p = 0.040). The top constraint for women was lack of childcare and for men it was too tired after work. Consistent with previous findings, a large proportion of respondents were totally inactive and did not participate in moderate or vigorous LTPA. Findings indicated that men and women experienced constraints differently, but that for both groups constraints led to lower LTPA. ABSTRACT FROM AUTHOR
Des recherches antAcrieures indiquent que les Latinos ont un faible taux de participation Aÿl'activitAc physique et de loisir (TPAPL). En consAcquence, les Latinos souffrent de taux AclevAcs d'obAcsitAc et autres maladies associAces Aÿce manque d'activitAc. Ainsi, le but principal de ce tte Actude exploratoire Actait d'acquAcrir une meilleure comprAchension du TPAPL chez les Latinos. Les objectifs de cette Actude Actait: (1) d'acquAcrir une comprAchension des similaritAcs et des diffAcrences masculines et fAcminines du TPAPL durant la marche (fait dans une variActAc de paramA"tres); (2) d'examiner les types d'environnements oA1 le TPAPL se produit; et (3) de mieux comprendre les attitudes Latinos et les perceptions de soutien social et les contraintes du TPAPL. L'Actude inclut des Latinos de deux banlieues AÿChicago. Les donnAces furent collectAces dans des parcs municipaux et dans un complexe sportif. Les rAcsultats indiquent que 39.1% des Latinos s'Actaient livrAcs Aÿun TPAPL vigoureux de 2.75 jours dans la semaine prAccAcdente, et que les hommes Actaient plus actifs que les femmes (F = 6.47, p = 0.021) pendant de longues pAcriodes de ce temps. De plus, 38.6% des Latinos s'Actaient livrAcs Aÿun TPAPL modAcrAc avec une moyenne de 2.57 jours durant la semaine. La majoritAc des participants ont indiquAc qu'ils marchaient au moins 10 minutes par jour (77.5%), et les hommes ont consacrAc beaucoup plus de temps dans leurs marches que les femmes (F = 47.17, p = 0.001). Les hommes Actaient plus disposAcs d'exercer dans les parcs alors que les femmes Actaient plus disposAces d'exercer Aÿla maison. La plupart des Latinos ont indiquAc des niveaux AclevAcs de soutien social pour l'exercice, et les hommes trouvent le PTAPL plus intAcressant que les femmes (F = 4.23, p = 0.040). Les plus grosses contraintes pour les femmes Latino sont le manque de garderies d'enfants et pour les hommes Latino la fatigue aprA"s l'ouvrage. ConformAcment aux rAcsultats prAccAcdents, une grande proportion de rAcpondants sont totalement inactifs et ont un TPAPL trA"s peu AclevAc. Tandis que les rAcsultats indiquent que les hommes et les femmes expAcrience diffAcrentes contraintes, les contraintes pour ces deux groupes conduits Aÿune baisse du TPAPL. ABSTRACT FROM AUTHOR
SN - 14927713
UR -
https://login.proxy.bib.uottawa.ca/login?url=http://search.ebscohost.com/login.aspx?direct=true&db=s3h&AN=67020837&site=ehost-live
ER -

TY - JOUR
ID - 4232
T1 - Exercising Their Right to Play
A1 - Burlingame,Helle
Y1 - 2007/12//
KW - SportDiscus
KW - *EXERCISE for children
KW - *CHILDREN -- Health
KW - *RECREATION
KW - *PLAYGROUNDS
KW - *WEIGHT loss
KW - Research
RP - NOT IN FILE
SP - 34
EP - 37
JA - Parks & Recreation
VL - 42
IS - 12

N2 - The article examines the importance of playground exercise and recreation for young children. According to the article, children can attain two-thirds of their recommended daily dose of exercise in only a half-an-hour of playground time. It is a unique way to keep kids trim and to focus on weight loss while emphasizing fun. Particular focus is given to an energy expenditure study conducted at the University of Florida which focused on the number of calories kids burn at the playground
SN - 00312215
UR -
https://login.proxy.bib.uottawa.ca/login?url=http://search.ebscohost.com/login.aspx?direct=true&db=s3h&AN=27901045&site=ehost-live
ER -

TY - JOUR
ID - 4233
T1 - Mountaineering of Children with regard to their Natural and Social Environment
A1 - Burnik,S.
A1 - Ko • ɨir,B.
A1 - TopiŽ_,M.Doupona
A1 - Pori,M.
Y1 - 2007/04//
KW - SportDiscus
KW - *MOUNTAINEERING
KW - CHILDREN & the environment
KW - SAFETY measures
KW - CREATIVE activities & seat work
KW - HUMAN ecology
KW - SOCIAL context
KW - Slovenia
RP - NOT IN FILE
SP - 159
EP - 165
JA - International Journal of Physical Education
VL - 44
IS - 4
N2 - The objective of our research project was to establish the influence of natural (place of residence) and social (parents, family, school) environment on the rate of mountaineering activity of a child. Our sampling group consisted of 233 subjects (age 11,4 A ñ 0,3 years), who were all in fifth grade of elementary school in Slovenia. One part of the children resided in Ljubljana and its surroundings, while the other part resided in mountainous environment. The results showed that most of the children made one to three mountaineering trips per year within their family circle, as well as within their school activity programme. Natural environment from which they originate does have an impact on the rate of their mountaineering activity as the children living close to the mountains make mountaineering trips more often within the frame of their school activities, while children living in urban environment go to the mountains oftener with their parents. Parents with their good example have a major impact on the rate of mountaineering activity of their child. Most of the children from mountaineering families like to go to the mountains. Mountaineering is more popular with the children who have mountaineering organised within the frame of their school activities. ABSTRACT FROM AUTHOR
SN - 03418685
UR -
https://login.proxy.bib.uottawa.ca/login?url=http://search.ebscohost.com/login.aspx?direct=true&db=s3h&AN=30031341&site=ehost-live
ER -

TY - JOUR
ID - 4234
T1 - MEMORY OF TEACHERS FOR PUPILS
A1 - Burtt,Harold E.
Y1 - 1942/11//
KW - SportDiscus

KW - *TEACHERS
KW - *SCHOOL children
KW - *MEMORY
KW - *PSYCHOLOGY
KW - *INTELLECT
KW - RECALL (Information retrieval)
KW - STUDY & teaching
KW - DIFFERENTIAL psychology
RP - NOT IN FILE
SP - 199
EP - 214
JA - Journal of Social Psychology
VL - 16
IS - 2
N2 - Teachers of introductory psychology courses were supplied with the names of students in their classes of several previous years and indicated with three degrees of certainty those which they could recall. They also checked from a key list reasons for recall of which they were aware and likewise added any further reasons which occurred to them. The main series involved 20 teachers and approximately seven thousand students. Under these conditions the average instructor recalled about 13 per cent of his students. For some instructors there was a negative correlation between effectiveness of recall and size of section. For other instructors no significant relation existed. Several instructors showed a significant tendency to remember better students of the opposite sex. Only one showed any possibly significant tendency to be more effective with his own sex. Several teachers showed a significant tendency to remember their good students in the sense of academic grades more readily than mediocre or poor ones. This tendency was a bit more marked with advanced than with elementary courses. There was no tendency for the poor students to leave a more lasting impression. Similarly some teachers tended to recall more readily students of higher intelligence. There was also an occasional significant tendency to remember those of lowest intelligence. Some teachers were definitely aware of the reasons for recall in most cases. For some of them the expressed reasons were mostly of one category while others scattered their reasons quite extensively. The reasons most frequently mentioned were personal appearance, conduct in class, followed by personal conferences, peculiarity of name, and contacts outside of class. Other reasons mentioned with less frequency were physical defects, peculiarity of name, location of seat in room, and presence in other classes taught by the same instructor. Occasional mention was made of grades, peculiarities of actions, voice, or handwriting. There appears to be no universal reason whereby students are remembered by their teachers. Individual differences on the part of the teachers evidently play a role, with some impressed by one aspect of a student and some with another. If one were to specify conditions under which the probability of recall by the teacher in general is enhanced, it would be something as follows: the student should be in a small class taught by an instructor of the opposite sex; should do good academic work in the class and should have high intelligence; should have something unusual about his appearance and something significant about his conduct in class; should have some personal conferences with the teacher, have a peculiar name, and should have some additional non-academic contacts outside of class. ABSTRACT FROM AUTHOR
SN - 00224545
UR -
https://login.proxy.bib.uottawa.ca/login?url=http://search.ebscohost.com/login.aspx?direct=true&db=s3h&AN=16877835&site=ehost-live
ER -

TY - JOUR
ID - 4235
T1 - Relaxing with Nature Does a Body Good
A1 - Buster,A.Natural Stress
KW - Reviewer Nominated
RP - NOT IN FILE
ER -

TY - JOUR
ID - 4236
T1 - University students learn by helping at-risk elementary school students: "Team Lincoln"

A1 - Butcher,D.A.
A1 - Hall,E.
Y1 - 1998/07//
N1 - Accession Number: 1998064718. Language: English. Entry Date: 19981001. Revision Date: 20091218. Publication Type: journal article; research; tables/charts. Journal Subset: Allied Health; Peer Reviewed; USA. No. of Refs: 24 ref. NLM UID: 101086597
KW - CINAHL
KW - School Health Services -- In Infancy and Childhood
KW - Recreation
KW - Program Evaluation -- Utah
KW - Students,College
KW - Collaboration
KW - Utah
KW - Interviews
KW - Pretest-Posttest Design
KW - Volunteer Workers
KW - Social Skills
KW - Cooperative Behavior
KW - Community-Institutional Relations
KW - Child
KW - Adult
KW - Male
KW - Female
KW - Human
RP - NOT IN FILE
SP - 191
EP - 202
JA - Social Work in Education
VL - 20
IS - 3
N2 - In the search for solutions to help at-risk youths, there has been a movement toward integration of various types of services within educational systems. Because of limited resources, it is especially imperative for institutions of higher education to develop partnerships with communities and schools. On the basis of this philosophy, the University of Utah College of Health (COH) and a Salt Lake City urban elementary school have formed a partnership to serve youths who are at risk. Team Lincoln was created to provide a program of organized recess activities for 450 at-risk children in grades 1 through 6. This article describes and evaluates the Team Lincoln school-based service learning program
SN - 0162-7961
AD - McMillan Elementary School, Murray, UT 84107
UR - http://search.ebscohost.com/login.aspx?direct=true&db=cin20&AN=1998064718&site=ehost-live
ER -

TY - JOUR
ID - 4237
T1 - Enhancing social skills through school social work interventions during recess: gender differences
A1 - Butcher,D.A.
Y1 - 1999/10//
N1 - Accession Number: 2000020992. Language: English. Entry Date: 20000301. Revision Date: 20091218. Publication Type: journal article; research; tables/charts. Journal Subset: Allied Health; Peer Reviewed; USA. No. of Refs: 42 ref. NLM UID: 101086597
KW - CINAHL
KW - Communication Skills Training -- In Infancy and Childhood
KW - Schools,Elementary
KW - Play and Playthings
KW - Social Work
KW - Observational Methods
KW - interrater reliability

KW - Descriptive Statistics
KW - Evaluation Research
KW - Repeated Measures
KW - Child
KW - Human
RP - NOT IN FILE
SP - 249
EP - 262
JA - Social Work in Education
VL - 21
IS - 4
N2 - Recess serves as a prime example of an underused social work intervention opportunity in schools. As members of the school community well know, recess is the time that both students and teachers look forward to everyday. However, the entire school climate can be negatively affected when problems occur on the playground. This article describes and evaluates a recess-based social skill intervention that targets elementary aged school children and their behaviors on the playground. Program effectiveness will be explored by examining changes in problem behaviors from before and during the program. Special attention will be paid to gender differences
SN - 0162-7961
UR - http://search.ebscohost.com/login.aspx?direct=true&db=cin20&AN=2000020992&site=ehost-live
ER -

TY - JOUR
ID - 4241
T1 - The Effect of Feedback and Information on Children's Pedometer Step Counts at School
A1 - Butcher,Zoe
A1 - Fairclough,Stuart
A1 - Stratton,Gareth
A1 - Richardson,David
Y1 - 2007/02//
KW - SportDiscus
KW - *PEDOMETERS
KW - *PHYSICAL fitness for children
KW - *CHILDREN -- Health
KW - *WALKING
KW - *SCHOOL children
KW - *HEALTH
KW - *EXERCISE for children
KW - FEEDBACK (Psychology)
KW - Motivation
KW - perceived competence
KW - Physical Activity
RP - NOT IN FILE
SP - 29
EP - 38
JF - Pediatric Exercise Science
JA - Pediatr Exerc Sci
VL - 19
IS - 1
N2 - This study examined whether feedback or feedback plus physical activity information could increase the number of pedometer steps taken during 1 school week. One hundred seventy-seven students (mean age 9.124 Añ1.11 years) in three elementary schools participated. Schools were randomly assigned to control (CON), feedback (FB), or feedback plus information (FB+I) groups. Children wore pedometers during school time for 5 consecutive weekdays. The total steps of the groups were recorded at the end of each school day, with students in the FB and FB+I groups free to view their step counts. In addition, the FB+I group received information and ideas about how they could increase their daily steps. The CON group received no step-count feedback or information. Students in the FB+I group achieved significantly more steps per minute (17.17 Añ4.87) than

those in the FB (13.77 Añ4.06, p = 0.003) and CON (12.41 Añ3.12, p = 0.0001) groups. Information, as well as step-count feedback, increased elementary students' school-based physical activity (number of steps) in the short term. A longer intervention period is necessary to assess the sustained impact of this type of approach. ABSTRACT FROM AUTHOR
SN - 08998493
UR -
https://login.proxy.bib.uottawa.ca/login?url=http://search.ebscohost.com/login.aspx?direct=true&db=s3h&AN=23758797&site=ehost-live
ER -

TY - JOUR
ID - 4242
T1 - Inventing Net/Wall Games for all Students
A1 - Butler,Joy
A1 - Hopper,Timothy
Y1 - 2011/12//
KW - SportDiscus
KW - *PLAY
KW - *CHILDREN
KW - *STUDENTS
KW - *GAMES
KW - Learning
RP - NOT IN FILE
SP - 5
EP - 9
JA - ACHPER Active & Healthy Magazine
VL - 18
IS - 3/4
N2 - The article explains the difference between spontaneous play invented by children during recess and the Inventing Games (IG) approach. It notes that the IG approach builds on children's natural instinct to play but has very clearly defined educational outcomes that allow students to cognitively learn about game constructs through a collective learning process. It describes the teaching games for understanding (TGfU) classification system of the IG approach which consists of 4 categories
SN - 13287133
UR -
https://login.proxy.bib.uottawa.ca/login?url=http://search.ebscohost.com/login.aspx?direct=true&db=s3h&AN=90666822&site=ehost-live
ER -

TY - JOUR
ID - 4243
T1 - Obesity Trends for Missouri: An Analysis of the CDC Obesity Maps
A1 - Butler,Loren L.
Y1 - 2007/07//
KW - SportDiscus
KW - *OBESITY -- Prevention
KW - *PHYSICAL education
KW - *SCHOOL recess breaks
KW - *PHYSICAL fitness for children
KW - STUDENT activities
KW - CENTERS for Disease Control & Prevention (U.S.)
KW - STATISTICAL maps
KW - Missouri
RP - NOT IN FILE
SP - 27
EP - 34
JA - Missouri Journal of Health, Physical Education, Recreation & Dance

VL - 17
N2 - The article examines obesity in the state of Missouri through the analysis of maps from the U.S. Centers for Disease Control and Prevention. According to the article, to help prevent obesity, the state should provide more physical and health education, more recess time, and more extracurricular activity opportunities. The article discusses body mass index and the increase in obesity rates in Missouri
SN - 10586288
UR -
https://login.proxy.bib.uottawa.ca/login?url=http://search.ebscohost.com/login.aspx?direct=true&db=s3h&AN=26273885&site=ehost-live
ER -

TY - JOUR
ID - 4244
T1 - A matter of child's play. In outdoor play, a child is able to control his or her world without the restriction of time, rules, or movement
A1 - Caesar,B.
Y1 - 1999/04//
KW - SportDiscus
KW - *PLAYGROUNDS
KW - *PLAY
KW - *SAFETY
KW - Child
RP - NOT IN FILE
SP - 66
EP - 69
JA - Parks & Recreation
VL - 34
IS - 4
CY - ;
SN - 00312215
UR -
https://login.proxy.bib.uottawa.ca/login?url=http://search.ebscohost.com/login.aspx?direct=true&db=s3h&AN=SPHS-43870&site=ehost-live;http://articles.sirc.ca/search.cfm?id=S-43870;http://www.nrpa.org
L2 - http://articles.sirc.ca/search.cfm?id=S-43870
ER -

TY - JOUR
ID - 4245
T1 - Families with parents and children living with HIV described strategies to meet 3 common goals for normalisation... commentary on Rehm RS, Franck LS. Long-term goals and normalization strategies of children and families affected by HIV/AIDS. ANS 2000 Sep;23:69-82
A1 - Callery,P.
Y1 - 2001/07//
N1 - Accession Number: 2001075370. Language: English. Entry Date: 20010824. Revision Date: 20130524. Publication Type: journal article; abstract; commentary. Journal Subset: Core Nursing; Europe; Nursing; Online/Print; Peer Reviewed; UK & Ireland. NLM UID: 9815947
KW - CINAHL
KW - Family Coping
KW - HIV Infections -- Psychosocial Factors -- In Infancy and Childhood
KW - HIV Infections -- Psychosocial Factors -- In Adulthood
KW - conceptual framework
KW - Ethnographic Research
KW - Semi-Structured Interview
KW - Observational Methods
KW - Urban Areas -- California
KW - California
KW - Audiorecording

KW - thematic analysis
KW - Health Status -- In Infancy and Childhood
KW - Truth Disclosure
KW - Psychological Well-Being
KW - Infant
KW - Child,Preschool
KW - Child
KW - adolescence
KW - Adult
KW - Middle Age
KW - Male
KW - Female
RP - NOT IN FILE
SP - 92
EP - 92
JA - Evidence Based Nursing
VL - 4
IS - 3
N2 - QUESTION: What are the long term goals and normalisation strategies of families with parents and children living with HIV?
Design
Interpretive study based on symbolic interaction and using ethnographic data collection methods
Setting
Urban paediatric referral centre in the US
Participants
21 participants: 9 children with HIV infection (age range 7-15 y), 6 mothers and 1 father with HIV infection (mean age 38 y), and 5 uninfected adult family member caregivers (age range 30-79 y). Participants were of diverse ethnicity
Methods
Semistructured, open ended interviews were conducted individually for children (20-45 min) and adults (60-120 min), except for 1 mother and son who requested to be interviewed together. The interview guide included questions on family composition, disease disclosure, and symptom management. Interviews were audiotaped, transcribed verbatim, and data were analysed using constant comparison. Repeat interviews with 4 key participants validated and expanded findings
Main findings
Families had 3 goals to establish normalcy and stability: staying healthy, facilitating children's participation in school and social activities, and enhancing social and emotional wellbeing of family members. Strategies for staying healthy included lifestyle decisions (eg, balanced diet and exercise) and active participation in treatment regimens. Children related taking medications to staying healthy. To reinforce the necessity of the treatment regimen, parents often took their medication on the same schedule as their children; they also tried to allow children some freedom for normal activities within the necessary regimen. Parents juggled multiple responsibilities to care for family members; some focused on guarding their own health to keep their children healthy. In facilitating children's participation in school and social activities, schools served as markers of normal childhood activity and as resources. Management of stigma was important. All families were selective in disclosing information about infection to those outside the family and controlled their children's social lives in order to preserve their ability to be treated normally. Enhancing [the] social and emotional wellbeing of family members was achieved by having extended family members provide supplemental care for children. This helped parents by reducing their stress and increasing rest time, and children valued the relationships developed with extended family members. Families cited spiritual and religious beliefs and practices as important coping strategies. Psychological counsellors provided formal emotional support, whereas other professionals, such as nurses at the HIV centre, provided informal support. Non-infected children were a source of support and concern for parents and children
Conclusion
Families with parents and children living with HIV had 3 normalisation goals to foster normal living and used strategies to minimise stigma. [Original article accession number: 2000060336 (research)]
SN - 1367-6539
AD - Senior Lecturer, University of Manchester, Manchester, UK

UR - http://search.ebscohost.com/login.aspx?direct=true&db=cin20&AN=2001075370&site=ehost-live
ER -

TY - JOUR
ID - 4246
T1 - Supporting learning opportunities in natural settings through participation-based services
A1 - Campbell,P.H.
A1 - Sawyer,L.B.
Y1 - 2007/07//
N1 - Accession Number: 2009677592. Language: English. Entry Date: 20071026. Revision Date: 20120309. Publication Type: journal article; questionnaire/scale; research; tables/charts. Journal Subset: Allied Health; Blind Peer Reviewed; Editorial Board Reviewed; Peer Reviewed; USA. Special Interest: Pediatric Care. Instrumentation: Home Visiting Observation Form (HVOF); Natural Environments Rating Scale (NERS). No. of Refs: 39 ref. NLM UID: 8910748
KW - CINAHL
KW - Early Childhood Intervention -- Evaluation
KW - Home Visits
KW - Adult
KW - Caregivers
KW - Caregivers -- Psychosocial Factors
KW - chi square test
KW - Child,Preschool
KW - convenience sample
KW - Descriptive Statistics
KW - Early Childhood Intervention -- Methods
KW - Female
KW - Infant
KW - interrater reliability
KW - Male
KW - Parental Role
KW - Professional Role
KW - Professional-Patient Relations
KW - Scales
KW - T-Tests
KW - Two-Tailed Test
KW - videorecording
KW - Human
RP - NOT IN FILE
SP - 287
EP - 305
JF - Journal of Early Intervention
VL - 29
IS - 4
N2 - Recommended early intervention practices emphasize family-centered approaches provided within natural settings through interventionist interactions with caregivers. We defined this approach as participation-based and used the Home Visiting Observation Form (HVOF) and the Natural Environments Rating Scale (NERS) to describe 50 home visits and to examine the extent to which participation-based services could be identified and distinguished from traditional services. A minority of the sample visits were described as participation-based although participation-based services were clearly distinguishable from traditionally provided services. Interventionists using participation-based approaches interacted with the caregiver-child dyad and caregivers were actively involved with their children during the session. Results from the study suggest the NERS can be used to describe characteristics of participation-based services and holds promise as a measure that might be used by interventionists, their supervisors, or by researchers to measure intervention fidelity during home visits
SN - 1053-8151
AD - 5th floor, Thomas Jefferson University, Edison, 130 S. 9th St., Philadelphia, PA 19107
UR - http://search.ebscohost.com/login.aspx?direct=true&db=cin20&AN=2009677592&site=ehost-live
ER -

TY - JOUR
ID - 4247
T1 - Little BIG TIME
A1 - Cannella,Stephen
Y1 - 2003/08/25/
KW - SportDiscus
KW - *LITTLE League World Series,Williamsport,Pa.
KW - *YOUTH league baseball
KW - *BASEBALL -- Tournaments
KW - *LITTLE League baseball -- Tournaments
KW - *LITTLE League baseball
KW - *BASEBALL for children
KW - Pennsylvania
KW - United States
RP - NOT IN FILE
SP - 28
JA - Sports Illustrated
VL - 99
IS - 7
N2 - The average 12-year-old may not realize it, but many of the trappings that make it good to be a kid are the result of Pennsylvania ingenuity, including the first bottle of soda pop, the first nickelodeon (the precursor to the modern ciniplex), and the first batch of bubble gum. The same is true of another of Pennsylvania's kid-friendly creations, the Little League World Series, which kicked off its annual 10-day run in Williamsport. In 1947 Carl Stotz convened the first Little League National Tournament, a 12-team event that invited only one squad from beyond central Pennsylvania, the Hammonton (N.J.) All-Stars. In 1955, after years of bickering with the board of directors over sponsorships and other matters, Stotz was ousted in a court battle. Stotz never attended another Little League World Series and instead organized a competing youth baseball program called Original League, which debuted in 1955 and is still active, at Memorial Park. But in recent years Little League has taken steps to mend the rift with Stotz's family and supporters. In part to stimulate the raising of $20 million for the construction of the 4,000-seat Volunteer Stadium, which opened in 2001 and enabled the tournament to expand from eight to 16 teams, Little League acted to heal old wounds by honoring Stotz with a granite memorial outside Volunteer Stadium. Little League also promotes the Original League field on the other side of the river as the birthplace of youth baseball
SN - 0038822X
UR -
https://login.proxy.bib.uottawa.ca/login?url=http://search.ebscohost.com/login.aspx?direct=true&db=s3h&AN=10613912&site=ehost-live
ER -

TY - JOUR
ID - 4249
T1 - Socioeconomic Disparities in Elementary School Practices and Children's Physical Activity During School
A1 - Carlson,Jordan A.
A1 - Mignano,Alexandra M.
A1 - Norman,Gregory J.
A1 - McKenzie,Thomas L.
A1 - Kerr,Jacqueline
A1 - Arredondo,Elva M.
A1 - Madanat,Hala
A1 - Cain,Kelli L.
A1 - Elder,John P.
A1 - Saelens,Brian E.
A1 - Sallis,James F.
Y1 - 2014/01/02/
KW - SportDiscus
KW - *SCHOOL children

KW - *PHYSICAL education
KW - *SCHOOL recess breaks
KW - *PHYSICAL activity
KW - *PHYSICAL activity -- Measurement
KW - United States
KW - Socioeconomic Factors
KW - Research
KW - Active Travel to School
KW - Health focus: fitness/physical activity
KW - Manuscript format: research
KW - Outcome measure: behavioral
KW - Prevention Research
KW - Research purpose: relationship testing
KW - Setting: school
KW - Strategy: policy
KW - Study design: nonexperimental
KW - Target population age: youth
KW - Target population circumstances: income level
RP - NOT IN FILE
SP - S47
EP - S53
JF - American Journal of Health Promotion
JA - Am J Health Promot
N2 - Purpose. To examine school socioeconomic status (SES) in relation to school physical activity-related practices and children's physical activity. Design. A cross-sectional design, was used for this study. Setting. The study was set in 97 elementary schools; (63% response rate) in two U.S. regions. Subjects. Of the children taking part in this study, 172 were aged 10.2 (standard deviation (SD) = 1.5) years; 51.7% were girls, and 69.2% were White non-Hispanic. Measures. School physical education (PE) teachers or principals responded to 15 yes/no questions on school physical activity-supportive practices. School SES (low, moderate, high) was derived from the percent of students eligible for free and reduced-price lunch. Children's moderate to vigorous physical activity (MVPA) during school was measured with accelerometers. Analysis. School level analyses involved linear and logistic regression; children's MVPA analyses used mixed effects regression. Results. Low-SES schools were less likely to have a PE teacher and had fewer physical activity- supportive PE practices than did high-SES schools (p < . 05). Practices related to active travel to school were more favorable at low-SES schools (p < .05). Children attending high-SES schools had 4.4 minutes per day more of MVPA during school than did those at low-SES schools, but this finding was not statistically significant (p = . 124). Conclusion. These findings suggest that more low- and moderate-SES elementary schools need PE teachers in order to reduce disparities in school physical activity opportunities and that PE time needs to be supplemented by classroom teachers or other staff to meet guidelines. ABSTRACT FROM AUTHOR
SN - 08901171
UR -
https://login.proxy.bib.uottawa.ca/login?url=http://search.ebscohost.com/login.aspx?direct=true&db=s3h&AN=93583961&site=ehost-live
ER -

TY - JOUR
ID - 4250
T1 - Should the ratio of supervisors to children be the same for the outdoor playground (learning environment) as for the indoor classroom?
A1 - Carpenter,Mary
A1 - Mizwicki,Michael
Y1 - 2000/04//
KW - SportDiscus
KW - *OUTDOOR recreation -- Safety measures
KW - CLASS size
RP - NOT IN FILE
SP - 10

JA - JOPERD: The Journal of Physical Education, Recreation & Dance
VL - 71
IS - 4
N2 - Presents the views of physical education teachers and teachers regarding the ratio of supervisors to children for an outdoor playground and for an indoor classroom. Safety issues with the ratio; Ability of an educator to supervise; Relation of the layout and size of outdoor play area on the ratio
SN - 07303084
UR -
https://login.proxy.bib.uottawa.ca/login?url=http://search.ebscohost.com/login.aspx?direct=true&db=s3h&AN=3086904&site=ehost-live
ER -

TY - JOUR
ID - 4253
T1 - Children's perceived competence and enjoyment in physical education and physical activity outside school. / La competence percue des eleves et le plaisir eprouve en education physique et en activite physique en dehors de l'ecole
A1 - Carroll,B.
A1 - Loumidis,J.
Y1 - 2001/02//
KW - SportDiscus
KW - *PSYCHOLOGY
KW - *PHYSICAL education & training
KW - *STRESS (Physiology)
KW - *CARDIOVASCULAR fitness
KW - *PERFORMANCE
KW - Pleasure
KW - Evaluation
KW - Multivariate Analysis
KW - girls
KW - BOYS
KW - Great Britain
KW - EXTRACURRICULAR
KW - CORRELATION
KW - univariate analysis
KW - Child
KW - EDUCATION-PHYSIQUE
KW - ELEVE
KW - ENFANT
KW - Motivation
KW - PLAISIR-DOULEUR
KW - ACTIVITE-PHYSIQUE
KW - SPORT-EXTRA-SCOLAIRE
KW - PRATIQUE-SPORTIVE
KW - SEXE
RP - NOT IN FILE
SP - 24
EP - 43
JA - European Physical Education Review
VL - 7
IS - 1
CY - ;
N2 - This study aimed to examine the relationship of children's perceived competence in physical education to their enjoyment in the subject, and how boys and girls scoring high and low in enjoyment and perceived competence differed in their levels of physical activity. Year 6 primary school children (n = 922) completed questionnaires giving their physical activity the previous week, their perceived competence in, and enjoyment of, PE. Employing correlation, multivariate and univariate analysis of variance techniques, the results indicated

moderate positive and significant relationships between enjoyment in PE and perceived competence in PE for the whole sample (r = .39, p < 0.001). Children of high perceived competence participated in significantly more physical activity (quantity and intensity) outside school than those of low perceived competence, but there was no difference in enjoyment levels and quantity of physical activity. The boys scored higher than the girls on time spent on physical activity, on perceived competence and enjoyment. Motivation theory is utilized to examine the relationship of perceived competence and enjoyment in physical education to physical activity outside school
Cette etude a vise a examiner (1) la relation entre la competence percue des eleves en education physique (EP) et le plaisir eprouve dans la discipline, (2) comment les garcons et les filles qui obtiennent des resultats plus ou moins eleves dans le plaisir eprouve ou la competence percue se sont differencies quant a leur niveau d'activite physique. 922 eleves de l'ecole primaire ages de dix ans ont renseigne des questionnaires sur leur activite physique hebdomadaire, leur competence percue et le plaisir eprouve en education physique. A l'aide de correlations, d'analyses de variance multivariee et univariee, les resultats ont indique: (1) des relations moyennement positives et significatives entre le plaisir eprouve et la competence percue en education physique pour l'ensemble de l'echantillon (r=.39, p < 0.001); (2) les enfants qui ont une competence percue elevee ont participe significativement de maniere plus importante a une activite physique en dehors de l'ecole (quantite et intensite), que ceux qui ont une competence percue plus faible; mais il n'a pas eu de difference dans les niveaux de plaisir et la quantite d'activite physique; (3) les garcons ont des resultats plus eleves que les filles dans le temps d'activite physique, la competence percue et le plaisir eprouve. La theorie de la motivation a ete utilisee pour examiner la relation entre la competence percue et le plaisir eprouve en education physique, et dans l'activite physique en dehors de l'ecole. (HERACLES) Questionnaire soumis a 922 eleves d' ecole primaire ages de 6 ans, sur leur activite physique hebdomadaire, leur perception de leurs propres competences, le plaisir eprouve en education physique. Relation entre la competence percue, le plaisir ressenti, la motivation, la participation a des activites sportives extra-scolaires, chez les garcons compares aux filles
Die Studie untersuchte (1) die Beziehung zwischen der von Kindern selbst wahrgenommenen Kompetenz im Schulsport und ihrer Freude in diesem Schulfach; (2) wie Jungen und Maedchen, die besonders viel oder wenig Spass am Sportunterricht haben und ueber eine hohe oder niedrige selbst wahrgenommene Kompetenz verfuegen, sich in Quantitaet und Intensitaet der ausserschulischen koerperlichen Aktivitaet unterscheiden. 922 Sechstklaessler gaben dazu in einer schriftlichen Befragung ihre koerperlichen Aktivitaeten in der vergangenen Woche, die persoenliche Einschaetzung selbst wahrgenommener Kompetenz und ihren Spass am Sportunterricht an. Die Ergebnisse, die unter Verwendung von Korrelationen, multivariaten und univariaten Varianzanalysetechniken erzielt wurden, zeigen, (1) dass es einen schwach positiv signifikanten Zusammenhang zwischen dem beim Sportunterricht empfundenen Spass und der selbst wahrgenommenen Kompetenz fuer die gesamte Stichprobe (r=.39, p < 0.001) gibt; (2) dass Kinder mit hoher selbst wahrgenommener Kompetenz ausserhalb der Schule signifikant staerker koerperlich aktiv (in Quantitaet und Intensitaet) sind, als jene mit einer geringeren selbst wahrgenommenen Kompetenz. Kein Unterschied liess sich feststellen in Bezug auf das Mass an Freude und die Quantitaet koerperlicher Aktivitaet; (3) dass Jungen durchschnittlich mehr Zeit mit koerperlicher Aktivitaet verbringen, hoehere Werte bezueglich selbst wahrgenommener Kompetenz und meher Spass am Sport haben als Maedchen. Die Motivationstheorie wird verwendet, um den Zusammenhang zwischen der selbst wahrgenommenen Kompetenz und dem Spass am Schulsport in Beziehung zu setzen mit der koerperlichen Aktivitaet ausserhalb der Schule
SN - 1356336X
UR -
https://login.proxy.bib.uottawa.ca/login?url=http://search.ebscohost.com/login.aspx?direct=true&db=s3h&AN=SPHS-670832&site=ehost-live;http://articles.sirc.ca/search.cfm?id=S-670832;http://www.sagepub.co.uk
L2 - http://articles.sirc.ca/search.cfm?id=S-670832
ER -

TY - JOUR
ID - 4254
T1 - What the World Could Learn From Summer Camp
A1 - Carroll,Matthew
Y1 - 2009/07//
KW - SportDiscus
KW - *CAMPS
KW - *OUTDOOR recreation
KW - CHILDREN & the environment

KW - Social Interaction
KW - NEW York (State)
RP - NOT IN FILE
SP - 58
EP - 58
JA - Camping Magazine
VL - 82
IS - 4
N2 - The article focuses on the significance of recreation for children such as camping where they can play with other children. It cites several activities during summer camp in New York where children can play outside in a safe environment the way they used to. It notes the advantages of camping wherein it allows children to spend time with their friends and develop the social skills that are important instead of sitting in front of a television set
SN - 07404131
UR -
https://login.proxy.bib.uottawa.ca/login?url=http://search.ebscohost.com/login.aspx?direct=true&db=s3h&AN=43798993&site=ehost-live
ER -

TY - JOUR
ID - 4255
T1 - The influence of free time in the access to the practice of physical and sports activities in school age children
A1 - Carvalhal,Maria Isabel MourAœo
A1 - Silva,AntA3nio JosAc
A1 - Raposo,JosAc Jacinto Vasconcelos
A1 - Louro,Hugo GonA alo Duarte
A1 - LeitAœo,LuA-s Filipe Moutinho
Y1 - 2008/03//
KW - SportDiscus
KW - *LEISURE
KW - *SCHOOL children
KW - *VIDEO games
KW - *SPORTS
KW - SCHOOL entrance age
KW - Television
KW - Motor Activity
KW - Video Games
KW - Actividad Motora
KW - Juegos de Video
KW - TelevisiA3n
KW - Atividade motora
KW - Jogos de VA-deo
KW - TelevisAœo
RP - NOT IN FILE
SP - 81
EP - 87
JA - Fitness & Performance Journal (Online Edition)
VL - 7
IS - 2
N2 - Introduction: With this study we aim to distinguish which are the free time variables associated to the adherence to physical and sports activities. Materials and Methods: A sample composed of 719 children aged between 6 and 10 years old, subject to the application of a questionnaire, was analyzed. The contingence tables and de I_ (phi) value were used to verify the association between the free time variables and the practice of physical and sports activities. Results: The results' analysis allowed us to conclude that the places to spend free time (home, street, outdoor playground and public garden) and the time spent in these places are associated to the physical and sports activities. Discussion: There is a positive association between the time that children

spend in outdoor playgrounds, street, public garden and the practice of global physical activities. In regards to the practice in clubs, we verified a negative association between the time spent in the public garden, outdoor playground and street and the adherence to the practice. The time that they spend with video games is negatively associated to the formal practice. The time that they spend watching television is positively associated to the global practice, but negatively associated to the formal practice in business days. ABSTRACT FROM AUTHOR
IntroducciA3n: Con este estudio pretendemos discriminar las variables de tiempo libre asociadas a la adhesiA3n a las actividades fA-sicas y deportivas. Materiales y MActodos: Fue analizada una muestra de 719 niA ños con edades comprendidas entre 6 y 10 aA ños, sujetas a la administraciA3n de un cuestionario. Las tablas de contingencia y el valor de (phi) habA-an sido utilizados para verificar la asociaciA3n entre variables de tiempo libre y la prA -ctica de actividades fA-sicas y deportivas. Resultados: El anA -lisis de los resultados permitiA3 concluir que los locales de tiempo libre (casa, calle, parque infantil y jardA-n pA §blico) y el tiempo dispendido en esos locales estA -n asociados a las actividades fA-sicas y deportivas. DiscusiA3n: Existe una asociaciA3n positiva entre el tiempo que los ninAæs pasan en el parque infantil, calle, jardA-n pA §blico y la prA -ctica de actividades fA-sicas globales. Relativamente a la prA -ctica en el club, se verificA3 una asociaciA3n negativa entre el tiempo planchado en el jardA-n pA §blico, parque infantil y calle y la adhesiA3n a la prA -ctica. El tiempo que pasan en juegos de vA-deo estA -asociado negativamente con la prA -ctica formal. El tiempo en que asisten televisiA3n estA -asociado positivamente con la prA -ctica global, pero negativamente con la prA -ctica formal en los dA-as A §tiles. ABSTRACT FROM AUTHOR
SN - 15199088
UR -
https://login.proxy.bib.uottawa.ca/login?url=http://search.ebscohost.com/login.aspx?direct=true&db=s3h&AN=35447237&site=ehost-live
ER -

TY - JOUR
ID - 4256
T1 - Playground activities for boys and girls: developmental and cultural trends in children's perceptions of gender differences
A1 - Carvalho,A.M.A.
A1 - Smith,P.K.
A1 - Hunter,T.
A1 - Costabile,A.
Y1 - 1990/11//
KW - SportDiscus
KW - *PLAY
KW - *GAMES
KW - *SEX role
KW - CROSS-cultural studies
KW - BOYS
KW - girls
KW - ATTITUDE (Psychology)
KW - England
KW - Italy
KW - Child
KW - STEREOTYPE
RP - NOT IN FILE
SP - 343
EP - 347
JF - Play & Culture
VL - 3
IS - 4
CY - ;
N2 - Children's own perceptions of gender appropriateness of five common playground activities, playfighting, playchasing, real fighting, football, and skipping, were assessed at different ages and in two cultures (England and Italy). Four activities showed some degree of gender stereotyping while one did not. This pattern was similar across ages (5 to 10 years) and cultures. However, there was a general trend, in both England and Italy,

for the degree of gender stereotyping to lessen with age. This trend occurred earlier in the English children, for traditionally male activities, and was more obvious in girls than boys. A cultural difference in the perception of skipping was also noted. Possible reasons for these findings are discussed
SN - 08944253
UR -
https://login.proxy.bib.uottawa.ca/login?url=http://search.ebscohost.com/login.aspx?direct=true&db=s3h&AN=SPH266637&site=ehost-live;http://articles.sirc.ca/search.cfm?id=266637
L2 - http://articles.sirc.ca/search.cfm?id=266637
ER -

TY - JOUR
ID - 4260
T1 - Students and teacher responses to a unit of student-designed games
A1 - Casey,Ashley
A1 - Hastie,Peter A.
Y1 - 2011/07//
KW - SportDiscus
KW - *EDUCATIONAL games
KW - *PHYSICAL education
KW - *TEACHERS
KW - *STUDENTS
KW - PRIMARY education
KW - Secondary Education
KW - invasion games
KW - Physical Education
KW - student-designed games
KW - teaching experiment
RP - NOT IN FILE
SP - 295
EP - 312
JA - Physical Education & Sport Pedagogy
VL - 16
IS - 3
N2 - Background: Despite the support in primary education that student-designed games enhance student contextualisation of skills and tactics, there has been little support in secondary education, nor any empirical research exploring these claims. This paper attempts to rekindle these beliefs and explores the use of student-designed games in an English secondary school. Aim: To provide a detailed account of classes of secondary students designing their own games, and to investigate their responses to the games-making process. Method: Two classes of boys (aged 14-16) at a school in England participated in the study. Each class was divided into three teacher-selected teams and attended one lesson per week, each lasting 40 minutes, for seven weeks. Lessons were adapted to give students four phases of development: a 2-week library-based planning and wiki construction phase; a 1-week outdoor modification and wiki update phase; three weeks of trials of the games with a wiki refinement phase; and a 1-week game and wiki finalization phase. In this time frame the pupils involved were challenged to design an invasion game that would be played in a subsequent unit. Data consisted of: teacher post-teaching reflections, interviews between a professor-researcher and the on-site participants, observations and analysis of wikis. Data analysis occurred on three levels. The first aspect was immediate and ongoing by the teacher-researcher to meet the 'on the spot' learning needs of his students. Secondly, the professor-researcher aided the teacher-researcher in systematic collection, organisation and analyses. Thirdly, peer debriefing occurred in which the research team analysed and critiqued the data during the collection and writing processes. Results: We found that students 'bought into' the process of games-making, were afforded an inclusive voice and worked as teams. Furthermore they engaged in immediate searches for innovation which were influenced by popular media, and shared ideas and learning with others to a degree that allowed them to exclude problematic skills. However, there was student frustration induced by watching others failing to properly play these games. Conclusions: We concluded that student-designed games 'freed' children to define competition at their own developmental level. This paper shows that by trusting pupils and supporting them to be creative in the games that they play it is possible to gain an insight into the processes by which students apply their knowledge about physical education. ABSTRACT FROM AUTHOR

SN - 17408989
UR -
https://login.proxy.bib.uottawa.ca/login?url=http://search.ebscohost.com/login.aspx?direct=true&db=s3h&AN=61459873&site=ehost-live
ER -

TY - JOUR
ID - 4261
T1 - Physical Activity During the School Day
A1 - Castelli,Darla M.
A1 - Ward,Kimberly
Y1 - 2012/08//
KW - SportDiscus
KW - *EXERCISE for children
KW - *PHYSICAL activity
KW - *STATIONARY bicycles
KW - *SCHOOL recess breaks
KW - CURRICULUM change
KW - SCHOOL employees -- Training of
KW - REWARDS & punishments in education
RP - NOT IN FILE
SP - 20
EP - 29
JA - JOPERD: The Journal of Physical Education, Recreation & Dance
N2 - The article looks at how a director of physical activity (DPA) can implement physical activities during the school day for U.S. students. According to the author, a DPA needs administrative support, justified curriculum changes, and trained staff in order to integrate activities that align with the Comprehensive School Physical Activity Program (CSPAP) initiative. Recommended activities for students include reading on a stationary bike, physical activity as an incentive, and organized recess
SN - 07303084
UR -
https://login.proxy.bib.uottawa.ca/login?url=http://search.ebscohost.com/login.aspx?direct=true&db=s3h&AN=78418607&site=ehost-live
ER -

TY - JOUR
ID - 4262
T1 - Occupational Injury Deaths of 16- and 17-Year-Olds in the United States
A1 - Castillo,Dawn N.
A1 - Landen,Deborah D.
A1 - Layne,Larry A.
Y1 - 1994/04//
KW - SportDiscus
KW - WORK-related injuries
KW - CHILD labor -- Law & legislation
KW - YOUNG workers
KW - INDUSTRIAL safety
KW - OCCUPATIONAL mortality
RP - NOT IN FILE
SP - 646
EP - 649
JF - American Journal of Public Health
JA - Am J Public Health
VL - 84
IS - 4
N2 - Data from the National Traumatic Occupational Fatalities surveillance system were used to analyze occupational injury deaths of civilian 16- and 17-year-olds during 1980 through 1989. There were 670 deaths;

the rate was 5.11 per 100,000 full-time equivalent workers. The leading causes of death were incidents involving motor vehicles and machines, electrocution, and homicide. Workers 16 and 17 years old appear to be at greater risk than adults for occupational death by electrocution, suffocation, drowning, poisoning, and natural and environmental factors. Improved enforcement of and compliance with federal child labor laws, evaluation of the appropriateness of currently permitted activities, and education are encouraged. ABSTRACT FROM AUTHOR
SN - 00900036
UR -
https://login.proxy.bib.uottawa.ca/login?url=http://search.ebscohost.com/login.aspx?direct=true&db=s3h&AN=9406010244&site=ehost-live
ER -

TY - THES
ID - 4264
T1 - Attitudes of Nurses Toward Children With Disabilities
A1 - Cervasio,Kathleen
Y1 - 2011///
N1 - Accession Number: 2012154470. Language: English. Entry Date: 20130621. Publication Type: doctoral dissertation; clinical trial; research. Instrumentation: Attitudes Towards Disabled Persons Scale (ATDP-B)
KW - CINAHL
KW - Child,Disabled
KW - Student Attitudes -- Evaluation
KW - Students,Nursing
KW - Attitude Measures
KW - Child
KW - Clinical Trials
KW - Curriculum
KW - Human
KW - Nursing Role
KW - Pretest-Posttest Design
KW - Prospective Studies
KW - Quantitative Studies
KW - Repeated Measures
KW - Scales
RP - NOT IN FILE
EP - 125
PB - Northcentral University
N2 - The attitudes of U.S. nurses toward children with disabilities have not been adequately measured over time and after an educational intervention. Disability content has not been a priority in nursing education and, if present, the focus has been on adults with disabilities. The attitudes of nurses play a significant role in the provision of quality healthcare services they provide for children with disabilities. This quantitative, experimental research measured the attitudes of graduating nursing students (N= 88) toward children with disabilities utilizing the Attitudes Towards Disabled Persons Scale (ATDP-B) before and after disability education. The control group consisted of 44 nurses while a group of 44 nurses received the treatment. The differences between the groups was measured at pretest (time 1), immediate posttest (time 2) after an educational module, and delayed posttest (time 3) one month follow up, utilizing repeated measures analysis of variance (ANOVA). Multivariate tests for within subject effect of the ATDP-B demonstrated that the dependent variable of attitudes as measured by the ATDP-B scale changes over time and after an educational module based on children with disabilities were (F= [2, 85] = 28.59, p < .01). It was discovered that the ATDP-B level changes over time, dependent on the group (F [2, 85] = 51.15, p < .01). Also, the between subjects main effect of group was significant across ATDP-B measurements (F = [1, 86] = 32.53, p <.01). The results of this research suggest that there is a significant difference in means of ATDP-B measurements between groups which indicates that the graduating nurses who received disability education performed significantly better on an assessment of attitudes toward children with disabilities than those who did not receive the education. The findings of this research should compel nursing faculty to reevaluate curriculum content, provide specific attitude measurements of nursing students at various levels of education, and develop protocols that can assist students in learning to care for children with disabilities. Future research can be designed to measure attitudes of

nurses toward children with disabilities at various levels of nursing education, in several nursing education programs within or outside the U.S, or as a comparison to other healthcare professionals
SN - 978-1-267-03755-8
AV - UMI Order AAI3486056.
M1 - Ed.D.
UR - http://search.ebscohost.com/login.aspx?direct=true&db=cin20&AN=2012154470&site=ehost-live
ER -

TY - JOUR
ID - 4265
T1 - Carolina Panthers
A1 - Chadiha,Jeffri
Y1 - 2003/09/09/
KW - SportDiscus
KW - *BACKFIELD play (Football)
KW - *RUNNING backs (Football)
KW - *FOOTBALL -- Offense
KW - CAROLINA Panthers (Football team)
KW - DAVIS,Stephen,1974-
RP - NOT IN FILE
SP - 104
EP - 107
JA - Sports Illustrated
VL - 99
IS - 8
N2 - The Carolina Panthers are predicted to finish fourth in the South Division of the National Football Conference. One week of training camp was all Stephen Davis needed to see the benefits of signing as a free-agent running back with the Panthers. Every time he looked into the crowds at Wofford College in Spartanburg, S.C., he saw his wife, Dee Dee, and their four children, who had rarely watched him practice during the seven seasons he spent with the Redskins. One morning when he trotted out of the locker room with quarterback Rodney Peete, Davis pointed to a small stadium at the end of a narrow street. That's where he played football for Spartanburg High. Ericsson Stadium in Charlotte is a mere hour and a half drive from the dream house he built in Columbia, S.C., two years ago. More important, Davis is returning to the role he enjoyed in Washington before Steve Spurrier brought his pass-happy Fun' n' Gun offense to the Redskins last season. Davis, a 6-foot, 230-pound workhorse who prefers 20 or more carries a game, averaged 17.25 an outing in '02 and gained only 820 yards. (He missed four of Washington's last nine games with a sprained right knee.) Davis won't have to worry about opportunities. The Panthers had the NFL's second-worst offense (267.5 yards a game) and ranked 25th in the league in rushing yards (99.1 per game) last season, and coach John Fox plans to rely heavily on Davis this year. Two free agents, guard Doug Brzezinski (Eagles) and wide receiver Ricky Proehl (Rams), should make immediate contributions, and first-round pick Jordan Gross will start at right tackle
SN - 0038822X
UR -
https://login.proxy.bib.uottawa.ca/login?url=http://search.ebscohost.com/login.aspx?direct=true&db=s3h&AN=10722955&site=ehost-live
ER -

TY - JOUR
ID - 4266
T1 - Just Gimme The Ball
A1 - Chadiha,Jeffri
Y1 - 2004/11/29/
KW - SportDiscus
KW - *FOOTBALL players
KW - *SPORTS teams
KW - DENVER Broncos (Football team)
KW - DENVER (Colo.)
KW - United States

RP - NOT IN FILE
SP - 58
EP - 58
JA - Sports Illustrated
VL - 101
IS - 21
N2 - The article profiles Denver Broncos football player Reuben Droughns. His three older brothers had experienced troubled childhoods, but Reuben had a different vibe. He wrote poetry and was a starter as a sophomore on the varsity football team, and when he carried the pigskin, nobody could stop him. He had a chance to be something special. And eventually he was, becoming the first member of his immediate family to attend a four-year college. (He's a semester shy of getting a degree.) When the season began the 26-year-old Droughns was the Broncos' little-known fullback, primarily a blocker who in his first four pro seasons gained all of 97 rushing yards. Now Droughns is one of the NFL's biggest surprise stories. Droughns sharpened his vision and instincts while playing fullback, leading the running backs into the holes. He's a violent runner for a man listed at 5'11", 207 pounds, a player who competes with a visible hunger. This is Droughns's time to shine. Old coaches and friends are calling to congratulate him. Fans recognize him in public. Venders peddle his number 34 jersey. But those things don't matter to him nearly as much as having the opportunity to show that he really is special
SN - 0038822X
UR -
https://login.proxy.bib.uottawa.ca/login?url=http://search.ebscohost.com/login.aspx?direct=true&db=s3h&AN=15212745&site=ehost-live
ER -

TY - JOUR
ID - 4267
T1 - Leisure and recreational places
A1 - Cheek,N.H.
A1 - Field,D.R.
A1 - Burdge,R.J.
Y1 - 1976///
KW - SportDiscus
KW - *LEISURE
KW - *RECREATION
KW - Research
KW - Methodology
KW - Experimental Design
KW - sociology
RP - NOT IN FILE
CY - Ann Arbor, Mich.;
PB - Ann Arbor Science
N2 - Chapters discuss: social groups and leisure settings, social organization of recreation places, social definition of outdoor recreation places, sociological perspectives on the zoological park, assessing participation in leisure activities, childhood socialization and adult leisure behaviour, recreation set as a concept, urban participants selected recreation patterns, recreation participation and ethnicity, the influence of uncommon leisure activities and special settings on behaviour, and the convergence of policy and social research
SN - 250400707
UR -
https://login.proxy.bib.uottawa.ca/login?url=http://search.ebscohost.com/login.aspx?direct=true&db=s3h&AN=SPH20975&site=ehost-live
ER -

TY - JOUR
ID - 4269
T1 - Education for leisure: is it our responsibility?
A1 - Cherry,C.
A1 - Woodburn,R.

Y1 - 1980///
KW - SportDiscus
KW - *EDUCATION
KW - *LEISURE
KW - Child
RP - NOT IN FILE
N2 - Discusses the need to help individuals prepare for leisure. Suggests that people are often physically, psychologically, socially, emotionally and creatively unprepared for leisure. Examines how the educational system can help people understand how free time can be effectively used to experience a state of well-being. Argues for the benefits of learning both the value of leisure and positive attitudes toward free time. Methods by which schools and government service agencies can explore the most effective means of educating for leisure are discussed
UR -
https://login.proxy.bib.uottawa.ca/login?url=http://search.ebscohost.com/login.aspx?direct=true&db=s3h&AN=SPH82096&site=ehost-live;http://articles.sirc.ca/search.cfm?id=82096
L2 - http://articles.sirc.ca/search.cfm?id=82096
ER -

TY - THES
ID - 4270
T1 - Effects of a therapeutic recreation intervention within a technology-based physical activity context on the social interaction of male youth with autism spectrum disorders
A1 - Chiang,I.
Y1 - 2003///
N1 - Accession Number: 2005093912. Language: English. Entry Date: 20050617. Revision Date: 20091218. Publication Type: doctoral dissertation; research. Special Interest: Psychiatry/Psychology
KW - CINAHL
KW - Autistic Disorder
KW - Friendship
KW - Recreational Therapy
KW - adolescence
KW - Child
KW - Loneliness
KW - Male
KW - Technology
KW - triangulation
KW - Human
RP - NOT IN FILE
EP - 179
PB - Indiana University
N2 - The purpose of this study is to examine the effect of a therapeutic recreation intervention, within a technology-based physical activity context, on the social interaction of male youth with autism spectrum disorders (ASDs). Youth with ASDs report greater feelings of loneliness and poorer quality friendships than youth without ASDs, due to deficits in social interaction inherent to the condition. Social rank theory stipulates that low social rank leads to de-escalating strategies, which include low self-esteem, avoidance and keeping a distance from peers. Youth with ASDs are typically in a position of lower social rank compared with peers. Implementing a situational reverse social rank (SRSR) to create reciprocal social interaction may decrease loneliness and increase friendship quality in this population. Six male youth ages 10-14 years with high functioning ASDs and 6 age and gender matched typically developing peers were recruited. Participants with ASDs were trained to use the Dance Dance Revolution [superscript](R) (DDR), a technology-based physical activity device, for three to six weeks. Following training, they taught a typically developing peer to play the DDR for four sessions. A combination of quantitative and qualitative methodologies was used to triangulate the effects of the intervention. Youth with ASDs reported significantly higher levels of loneliness and lower quality friendships than peers. Both groups reported significantly higher quality of friendship following the intervention. Qualitative information illustrated their desires for friendship and a cycle of losing friends. The results should prompt reconsideration of traditional peer-mediated interventions and suggest inclusion of social rank in social interventions for youth with ASDs. Traditional peer-mediated interventions may actually

jeopardize the development of friendship by increasing the gap in social rank between youth with ASD and their peers, because peers will continue to maintain a higher social status. A situational reverse social rank approach provides greater opportunities for generalization to natural settings and may improve the social status of youth with ASDs. This will help decrease levels of loneliness and increase quality of friendships in these individuals
AV - UMI Order AAI3111851.
M1 - Ph.D.
UR - http://search.ebscohost.com/login.aspx?direct=true&db=cin20&AN=2005093912&site=ehost-live
ER -

TY - JOUR
ID - 4272
T1 - coast to coast: JUNIORS TICKERS
A1 - Childress,Jason
Y1 - 2009/04//
KW - SportDiscus
KW - *VOLLEYBALL players
KW - *ATHLETES
KW - United States
RP - NOT IN FILE
SP - 40
EP - 41
JA - Volleyball (10584668)
VL - 20
IS - 4
N2 - The article profiles several volleyball players in the U.S. They include Rob Strauss, a volleyball player and a golfer who shot an all-time personal-best 72; Marty Lorenz, a middle blocker who stands 6'6" in height and has been playing volleyball for less than two years; and Patrick Schwagler, an outside hitter of Orchard Park High School who turned down offers from Ohio State University in favor of Princeton University, where he plans to study environmental engineering
SN - 10584668
UR -
https://login.proxy.bib.uottawa.ca/login?url=http://search.ebscohost.com/login.aspx?direct=true&db=s3h&AN=36800043&site=ehost-live
ER -

TY - JOUR
ID - 4273
T1 - THE METHOD TO THEIR MADNESS
A1 - Childress,Jason
Y1 - 2010/04//
KW - SportDiscus
KW - *VOLLEYBALL
KW - *SPORTS facilities
KW - *ATHLETIC clubs
KW - *OUTDOOR recreation
KW - Hawaii
RP - NOT IN FILE
SP - 38
EP - 40
JA - Volleyball (10584668)
VL - 21
IS - 4
N2 - The article explores the volleyball craze in Hawaii. It states that the sport is being played in various states but Hawaii is considered as the place that is synonymous with volleyball since the sport is played all day long, each day of the year. It traces the history of how the sport became so popular in the state that goes back to the middle of the 20th century. It mentions the Outrigger Canoe Club near the Waikiki Beach, the main venue of beach sports activity in Hawaii

SN - 10584668
UR -
https://login.proxy.bib.uottawa.ca/login?url=http://search.ebscohost.com/login.aspx?direct=true&db=s3h&AN=49179100&site=ehost-live
ER -

TY - JOUR
ID - 4274
T1 - Increasing Children's Physical Activity During School Recess Periods
A1 - Chin,John J.
A1 - Ludwig,David
Y1 - 2013/07//
KW - SportDiscus
KW - *OBESITY -- Prevention
KW - *HEALTH education
KW - *PHYSICAL activity
KW - Multivariate Analysis
KW - RESEARCH -- Finance
KW - Descriptive Statistics
KW - NEW York (State)
RP - NOT IN FILE
SP - 1229
EP - 1234
JF - American Journal of Public Health
JA - Am J Public Health
VL - 103
IS - 7
N2 - Objectives. We examined whether schools' participation in the Recess Enhancement Program (REP) in the spring of 2011 was associated with higher rates of children's vigorous physical activity. Methods. In REP, a coach guides children through age-appropriate games aimed at increasing their physical activity. During recess at 25 New York City public elementary schools (15 REP, 10 non-REP), researchers visually scanned predetermined areas (n = 1339 scans), recording the number of sedentary, walking, and very active children. Results. Multivariate statistical analysis found that participation in REP was a significant predictor (P = .027) of the rate of vigorous physical activity (percentage very active in scan area) whose least-squares means were 41% in REP schools and 27% in non-REP schools. A significantly higher rate in REP schools persisted when the coach was not in the scan area, suggesting a change in the recess culture of REP schools. Conclusions. The rate of vigorous physical activity in REP schools was 14 percentage points, or 52%, higher than the rate in non-REP schools. This low-cost intervention might be a valuable addition to the tools for combating childhood obesity and worth replicating elsewhere. ABSTRACT FROM AUTHOR
SN - 00900036
UR -
https://login.proxy.bib.uottawa.ca/login?url=http://search.ebscohost.com/login.aspx?direct=true&db=s3h&AN=87693301&site=ehost-live
ER -

TY - JOUR
ID - 4276
T1 - Certification for safety inspectors, not playgrounds
A1 - Christiansen,Monty L.
Y1 - 1994/04//
KW - SportDiscus
KW - *PLAYGROUNDS
KW - SAFETY measures
RP - NOT IN FILE
SP - 63
JA - Parks & Recreation
VL - 29

IS - 4
N2 - Discusses various aspects of playground safety. Initiatives by the US Consumer Product Safety Commission, American Society for Testing and Materials, American Association for Leisure and Recreation's Committee on Play and the National Playground Safety Institute; Why children are vulnerable to serious playground accidents; Certification for playground safety
SN - 00312215
UR -
https://login.proxy.bib.uottawa.ca/login?url=http://search.ebscohost.com/login.aspx?direct=true&db=s3h&AN=9411271029&site=ehost-live
ER -

TY - JOUR
ID - 4277
T1 - Family and Community Involvement in the Comprehensive School Physical Activity Program
A1 - Cipriani,Kristin
A1 - Richardson,Cheryl
A1 - Roberts,Georgi
Y1 - 2012/09//
KW - SportDiscus
KW - *SCHOOLS -- Exercises & recreations
KW - *PHYSICAL education
KW - *LIFESTYLES
KW - *PHYSICAL fitness
KW - COMMUNITY involvement
RP - NOT IN FILE
SP - 20
EP - 26
JA - JOPERD: The Journal of Physical Education, Recreation & Dance
VL - 83
IS - 7
N2 - The article discusses family and community involvement in the Comprehensive School Physical Activity Program. According to the authors, daily family routines establish norms, influence behavioral patterns, and determine the typical lifestyle for children. The author notes that observing the behavior and lifestyle of people in the community, children begin to familiarize themselves with and adapt to norms that exist outside of the home
SN - 07303084
UR -
https://login.proxy.bib.uottawa.ca/login?url=http://search.ebscohost.com/login.aspx?direct=true&db=s3h&AN=80825513&site=ehost-live
ER -

TY - JOUR
ID - 4278
T1 - Ageless Considerations for the Ongoing Inclusion of Play, Recess, and Physical Education
A1 - Clements,Rhonda
Y1 - 2010/08//
KW - SportDiscus
KW - *PLAY
KW - *CHILD development
KW - *PHYSICAL education
KW - STUDENT activities
KW - SOCIAL aspects
KW - DEWEY,John,1859-1952
RP - NOT IN FILE
SP - 7
EP - 11
JA - JOPERD: The Journal of Physical Education, Recreation & Dance

VL - 81
IS - 6
N2 - The author reflects on the early educators' insights regarding the value of children's play and physical activity. She discusses Swiss educational reformer Johann Pestalozzi's view on self activity or play as a tool for learning. She cites American psychologist and educator John Dewey's findings that play allows children to develop attitudes that coincide with the goals of physical education. She also underscores the need to include physical activity in educational reforms
SN - 07303084
UR -
https://login.proxy.bib.uottawa.ca/login?url=http://search.ebscohost.com/login.aspx?direct=true&db=s3h&AN=52979278&site=ehost-live
ER -

TY - JOUR
ID - 4280
T1 - All children left behind
A1 - Cohen,A.
Y1 - 2003/08//
KW - SportDiscus
KW - *PHYSICAL fitness
KW - *PLAY
KW - *RECESSES
KW - *CARDIOVASCULAR fitness
KW - Elementary Schools
KW - Activism
KW - Child
RP - NOT IN FILE
SP - 11
EP - 12
JA - Athletic Business
VL - 27
IS - 8
CY - ;
N2 - Reports on efforts by various organizations in Canada and the United States in lobbying school boards and teachers unions in an effort to re-instate recess and play time for children
SN - 0747315X
UR -
https://login.proxy.bib.uottawa.ca/login?url=http://search.ebscohost.com/login.aspx?direct=true&db=s3h&AN=SPHS-893023&site=ehost-live;http://articles.sirc.ca/search.cfm?id=S-893023;http://www.athleticbusiness.com
L2 - http://articles.sirc.ca/search.cfm?id=S-893023
ER -

TY - JOUR
ID - 4281
T1 - Don't try this at school
A1 - Cohen,A.
Y1 - 2003/08//
KW - SportDiscus
KW - *RECESSES
KW - *WOUNDS & injuries
KW - Elementary Schools
KW - ACTIONS & defenses
KW - California
KW - Child
RP - NOT IN FILE
SP - 26
EP - 26

JA - Athletic Business
VL - 27
IS - 8
CY - ;
N2 - Brief feature reports on a California lawsuit in which a school was charged with negligent supervision after an eight-year boy was injured while playing on money bars
SN - 0747315X
UR -
https://login.proxy.bib.uottawa.ca/login?url=http://search.ebscohost.com/login.aspx?direct=true&db=s3h&AN=SPHS-893036&site=ehost-live;http://articles.sirc.ca/search.cfm?id=S-893036;http://www.athleticbusiness.com
L2 - http://articles.sirc.ca/search.cfm?id=S-893036
ER -

TY - JOUR
ID - 4284
T1 - The effectiveness of aligned developmental feedback on the overhand throw in third-grade students
A1 - Cohen,Rona
A1 - Goodway,Jacqueline D.
A1 - Lidor,Ronnie
Y1 - 2012/11//
KW - SportDiscus
KW - *ACADEMIC achievement
KW - *MOTOR ability
KW - FEEDBACK (Psychology)
KW - THIRD grade (Education)
KW - information processing
KW - Learning
KW - ball velocity
KW - body components
KW - Motor Development
KW - Skill Acquisition
RP - NOT IN FILE
SP - 525
EP - 541
JA - Physical Education & Sport Pedagogy
VL - 17
IS - 5
N2 - Background: To improve student performance, teachers need to evaluate the developmental level of the child and to deliver feedback statements that correspond with the student's ability to process the information delivered. Therefore, feedback aligned with the developmental level of the child (aligned developmental feedback ƒ_" ADF) is sometimes considered to be the most appropriate type of feedback for improving student learning. It is assumed that the provision of ADF is beneficial in bringing about improvement in the learning of motor skills and subsequently in performance. However, the extent of the influence of ADF on motor performance remains unclear. Purpose: This study examined the influence of ADF on students' performance of the overhand throw for force in a naturalistic physical education setting. Participants: Ninety-seven third-grade students (39 girls, 58 boys) with a mean age of 105.4 months (SDƒ_%=ƒ_%4.76) participated in this study. All participants were selected from four intact classrooms in a mid-sized Midwestern (US) elementary school. Two of the four intact classes were randomly assigned to an ADF group (the experimental condition; Nƒ_%=ƒ_%48, 21 girls, 27 boys), and the other two intact classes were assigned to a general feedback (GF) group (the control condition; Nƒ_%=ƒ_%49, 18 girls, 31 boys). Research design and phases of the study: An experimental non-equivalent group design composed of pre-, post-, and retention phases assessed the influence of ADF on throwing performance. The study used a three-phase approach: Phase 1 involved the implementation of the control GF condition with the participants: a seven-day throwing unit using general feedback. After Phase 1 was concluded, the first part of the experimental condition was initiated (Phase 2): to train the physical education teacher (male, with seven years of teaching experience, who had no prior knowledge of developmental throwing sequences) to deliver the experimental condition (i.e. the ADF condition). Phase 2 concluded when the teacher was considered trained in the ADF condition. The final phase of the study (Phase 3) was the actual experimental

condition, consisting of a seven-day ADF instructional throwing unit. Data analysis: A chi-square analysis was used to examine the influence of ADF on the distribution of body component scores between the ADF and GF groups from the pre- to post- to retention phases as the body component data is ordinal in nature. An ANOVA with repeated measures on the highest ball velocity score was used to assess the influence of ADF on ball velocity scores. Findings: An ANOVA revealed that the ADF group had faster ball velocity than the GF group from pre- to post-intervention. Chi-square analyses revealed that the ADF group improved from pretest to posttest in the humerus and forearm components. The effects found at the end of the intervention program were maintained throughout the retention phase. Conclusions: The findings demonstrate the value of ADF in the learning process, and show how such feedback can enhance students' performance. ABSTRACT FROM AUTHOR
SN - 17408989
UR -
https://login.proxy.bib.uottawa.ca/login?url=http://search.ebscohost.com/login.aspx?direct=true&db=s3h&AN=82153945&site=ehost-live
ER -

TY - JOUR
ID - 4285
T1 - Mobilizing a low income border community to address state mandated coordinated school health
A1 - Coleman,K.J.
Y1 - 2006/01//
N1 - Accession Number: 2009115088. Language: English. Entry Date: 20060714. Revision Date: 20091218. Publication Type: journal article; research; tables/charts. Commentary: Brown KRM. Energizing a community for coordinated school health: a reaction to Coleman. AM J HEALTH EDUC 2006 Jan-Feb; 37(1): 63-4 (commentary) . Journal Subset: Blind Peer Reviewed; Editorial Board Reviewed; Expert Peer Reviewed; Health Promotion/Education; Peer Reviewed; USA. Special Interest: Pediatric Care. No. of Refs: 28 ref. NLM UID: 101090650. Email: coleman@mail.sdsu.edu
KW - CINAHL
KW - child health
KW - Health Promotion
KW - School Health Services
KW - Schools,Elementary
KW - Audiorecording
KW - Brainstorming
KW - Child
KW - Congresses and Conferences
KW - Food Services
KW - Parents
KW - Physical Education and Training
KW - School Health Nursing
KW - School Health Services -- Legislation and Jurisprudence -- Texas
KW - School Policies
KW - Teachers
KW - Texas
KW - thematic analysis
KW - Human
RP - NOT IN FILE
SP - 15
EP - 26
JA - American Journal of Health Education
VL - 37
IS - 1
N2 - This study documents the response of a low-income border community to a Texas state law requiring coordinated school health programs in every elementary school by the academic year 2006-2007. Findings are summarized from a one-day conference with 176 attendees to familiarize them with the requirements of this law and to mobilize the community to design an approach that all elementary schools in the state of Texas could use to implement coordinated school health. Four discussion groups were held during the conference to address the

following questions: 1) What are schools in the El Paso, TX border region already doing for child health?; 2) What more could be done without funding to implement coordinated health education?; and 3) What more could be done with funding to implement coordinated health education? Recommendations from these discussion groups included asking influential adults like teachers, food service personnel, and parents to model healthy behavior for children such as volunteering in the cafeteria, eating school meals, and exercising with the children during recess and physical education classes. The El Paso CATCH program models what a low-income, primarily Hispanic community can accomplish with minimal funding and resources
SN - 1932-5037
AD - Assistant Professor, Graduate School of Public Health, San Diego State University, 5500 Campanile Drive, San Diego, CA 92182-4162
UR - http://search.ebscohost.com/login.aspx?direct=true&db=cin20&AN=2009115088&site=ehost-live
ER -

TY - JOUR
ID - 4288
T1 - Barriers to outdoor play and nature connection for inner city children
A1 - Collier,Beth
Y1 - 2013///Winter
KW - SportDiscus
KW - *PHYSICAL fitness for children
KW - *YOUTH
KW - *HEALTH
KW - *PARKS
KW - *PUBLIC health
KW - NATURE appreciation
KW - CHILDREN & the environment
KW - CREATIVE ability
KW - England
RP - NOT IN FILE
SP - 10
EP - 13
JA - Horizons (14620677)
IS - 64
N2 - The author discusses the factors affecting the access of children to natural spaces including television, personal image, and poverty. She mentions the report from the Public Health England saying 70% of the young population in Great Britain are engaged in physical activities for less than an hour. She recommends exposing the kids to parks and woods to develop their enthusiasm and creativity
SN - 14620677
UR -
https://login.proxy.bib.uottawa.ca/login?url=http://search.ebscohost.com/login.aspx?direct=true&db=s3h&AN=93312885&site=ehost-live
ER -

TY - JOUR
ID - 4290
T1 - Quelles pratiques corporelles a l'ecole? Curriculum prescrit, curriculum reel et attentes des eleves. / Bodily activities at school: prescribed curriculum, real curriculum and the students' expectancies
A1 - Combaz,G.
Y1 - 1991/11//
KW - SportDiscus
KW - *PHYSICAL education & training
KW - *GYMNASTICS
KW - *SWIMMING
KW - *TRACK & field
KW - ATTITUDE (Psychology)
KW - Surveys
KW - Students

KW - teenagers
KW - Child
RP - NOT IN FILE
SP - 23
EP - 29
JA - Science et Motricite
IS - 15
CY - ;
N2 - Analysis of official documents and observation of day-to-day work reveals that the choice of activities taught during physical education clearly shows that a priority is given to some of them. Track and field athletics, and to a smaller extent, gymnastics and swimming are dominating all other activities taking place at school. As we can see from the choice of activities practised outside school, there is a considerable interest for physical activities taking place in stadiums or gymnasiums. A survey carried out on 4 985 students from high schools and secondary schools shows a strong gap between what is taught and what the students expect. It seems that activities where the follower faces uncertainties, whether from the enrivonment or because of partners or opponents, are much more attractive than individual sports, totally out of surprise. This tendency is particularly clear as far as outdoor activities are concerned ; they rarely take place at school. Moreover they vary according to social classes
L'analyse des textes officiels et l'observation des pratiques reelles revelent que le choix des contenus enseignes en education physique confere une priorite indiscutable a certaines activites. L'athletisme et, dans une moindre mesure, la gymnastique sportive et la natation occupent une place preponderante parmi l'ensemble des activites physiques pratiquees a l'ecole. A l'image de ce que l'on peut observer dans la pratique extra-scolaire, l'institution accorde une importance considerable aux pratiques corporelles qui se deroulent en milieu stable. Une enquete realisee aupres d'un echantillon de 4 985 lyceens et collegiens revele le fort decalage existant entre ce qui est reellement enseigne et les attentes des eleves en matiere de pratiques corporelles. Il apparait que les activites ou le pratiquant est soumis a une incertitude - que celle-ci provienne de l'environnement physique ou soit due a la presence de partenaires et d'adversaires - exercent beaucoup plus d'attrait que les sports individuels completement denues d'imprevu. Cette tendance est particulierement nette en ce qui concerne les activites physiques de pleine nature ; activites tres peu souvent pratiquees dans le cadre scolaire. En outre, elle se nuance quelque peu selon l'appartenance sociale des eleves
SN - 13781863
UR -
https://login.proxy.bib.uottawa.ca/login?url=http://search.ebscohost.com/login.aspx?direct=true&db=s3h&AN=SPH296100&site=ehost-live;http://articles.sirc.ca/search.cfm?id=296100
L2 - http://articles.sirc.ca/search.cfm?id=296100
ER -

TY - JOUR
ID - 4291
T1 - Preface: Texas Statewide Assessment of Youth Fitness
A1 - Cooper,Kenneth H.
A1 - Everett,Diana
A1 - Meredith,Marilu D.
A1 - Kioster,Jeff
A1 - Rathbone,Marissa
A1 - Read,Kathy
Y1 - 2010/09/02/
KW - SportDiscus
KW - *OBESITY
KW - *CHILD nutrition
KW - *PHYSICAL education
KW - *OVERWEIGHT children
KW - Research
KW - CENTERS for Disease Control & Prevention (U.S.)
KW - TELEVISION & children
KW - Texas
RP - NOT IN FILE

SP - ii
EP - iv
JF - Research Quarterly for Exercise & Sport
JA - Res Q Exerc Sport
VL - 81
N2 - The article offers information on the increasing rate of the childhood obesity in Texas. It states that national data from the Centers for Disease Control and Prevention (CDC) shows that the prevalence of obesity has increased by almost fourfold. It notes that the factors that contribute to the trends include the decline in physical education and recess requirement, decline in number of children who routinely walk and the increase in time of children watching television
SN - 02701367
UR -
https://login.proxy.bib.uottawa.ca/login?url=http://search.ebscohost.com/login.aspx?direct=true&db=s3h&AN=53842398&site=ehost-live
ER -

TY - JOUR
ID - 4296
T1 - Alice B. Beal Elementary gets in on the Movement
A1 - Coughlin,Cindy
Y1 - 2012/05//
KW - SportDiscus
KW - *TEACHERS
KW - *STUDENTS
KW - *SCHOOL children
KW - *FOOD
KW - *PHYSICAL activity
KW - Elementary Schools
KW - Educational Programs
KW - Massachusetts
RP - NOT IN FILE
SP - 6
EP - 6
JA - Strategies (08924562)
VL - 25
IS - 5
N2 - The author discusses how Alice B. Beal Elementary School in Springfield, Massachusetts has made changes to get its students active. Programs were made to allow students take activities home and get moving outside of school. The Footloose Fridays program allows students and teachers walk a set path for 15 minutes. The Fuel Up To Play 60 program encourages healthy eating and one hour of physical activity every day
SN - 08924562
UR -
https://login.proxy.bib.uottawa.ca/login?url=http://search.ebscohost.com/login.aspx?direct=true&db=s3h&AN=75498421&site=ehost-live
ER -

TY - JOUR
ID - 4300
T1 - A Review of State Regulations to Promote Physical Activity and Safety on Playgrounds in Child Care Centers and Family Child Care Homes
A1 - Cradock,Angie L.I.
A1 - O'Donnell,Emily M.
A1 - Benjamin,Sara E.
A1 - Walker,Elizabeth
A1 - Slining,Meghan
Y1 - 2010/03/02/
KW - SportDiscus

KW - *CHILDREN -- Health
KW - *PLAYGROUNDS
KW - *PHYSICAL fitness for children
KW - *HEALTH risk assessment
KW - LAW & legislation
KW - Child Welfare
KW - SAFETY measures
KW - DAY care centers -- Safety measures
KW - RISK aversion
KW - injury prevention
KW - outdoor environment
KW - Preschool
RP - NOT IN FILE
SP - S108
EP - S119
JF - Journal of Physical Activity & Health
JA - J Phys Act Health
VL - 7
N2 - Background: As interventions increasingly emphasize early child care settings, it is necessary to understand the state regulatory context that provides guidelines for outdoor physical activity and safety and sets standards for child care environments. Methods: Researchers reviewed regulations for child care facilities for 50 states, the District of Columbia and the Virgin Islands. We compared state regulations with national standards for 17 physical activity-and safety-related items for outdoor playground settings outlined in Caring for Our Children: National Health and Safety Performance Standards: Guidelines for Out-of-Home Child Care Programs (CFOC). State regulations were coded as fully, partially or not addressing the CFOC standard and state-level summary scores were calculated. Results: On average, state regulations fully addressed one-third of 17 CFOC standards in regulations for centers (34%) and family child care homes (27%). Data suggest insufficient attention to outdoor play area proximity and size, equipment height, surfacing, and inspections. Conclusions: Considerable variation exists among state regulations related to physical activity promotion and injury prevention within outdoor play areas. Many states' regulations do not comply with published national health and safety standards. Enhancing regulations is one component of a policy approach to promoting safe, physically active child care settings. ABSTRACT FROM AUTHOR
SN - 15433080
UR -
https://login.proxy.bib.uottawa.ca/login?url=http://search.ebscohost.com/login.aspx?direct=true&db=s3h&AN=49745565&site=ehost-live
ER -

TY - JOUR
ID - 4302
T1 - Developing age appropriate playground skills in children with developmental disabilities: an application of the model for movement confidence combined with precision teaching methodology
A1 - Crawford,M.E.
A1 - Livingston,C.
A1 - Swango,A.
Y1 - 2004///
N1 - Accession Number: 2009052539. Language: English. Entry Date: 20051202. Revision Date: 20091218. Publication Type: journal article; case study; CEU; exam questions; research; tables/charts. Journal Subset: Allied Health; Blind Peer Reviewed; Expert Peer Reviewed; Online/Print; Peer Reviewed; USA. Special Interest: Pediatric Care. Instrumentation: Peabody Growth and Development scale; Bayley Scale of Development. No. of Refs: 63 ref. Email: mecrawford@unmc.edu
KW - CINAHL
KW - Play and Playthings -- In Infancy and Childhood
KW - Child Development Disorders -- Rehabilitation
KW - Teaching Methods -- In Infancy and Childhood
KW - Recreational Therapy -- Methods -- In Infancy and Childhood
KW - Education,Continuing (Credit)

KW - Child
KW - child health
KW - Rehabilitation,Pediatric
KW - Natural Environment -- In Infancy and Childhood
KW - Qualitative Studies
KW - Quantitative Studies
KW - Observational Methods
KW - videorecording
KW - Checklists
KW - Confidence -- Evaluation -- In Infancy and Childhood
KW - Male
KW - Child,Preschool
KW - Clinical Assessment Tools
KW - Intelligence Tests
KW - Camps -- In Infancy and Childhood
KW - Cues -- In Infancy and Childhood
KW - Role Models -- In Infancy and Childhood
KW - post hoc analysis
KW - interrater reliability
KW - Human
RP - NOT IN FILE
SP - 38
JA - Annual in Therapeutic Recreation
VL - 13
N2 - This study reports findings on the use of precision teaching techniques and assistive behavioral shaping strategies to facilitate age appropriate play behaviors among young children with severe genetic disorders. Training occurred in a natural environment using four common playground apparatus. Qualitative and quantitative analyses of children's behaviors were utilized. Observations of playground behaviors via a videotape data base confirmed increases in competence levels. Qualitative improvement in children's confidence levels while engaging equipment was also measured utilizing a checklist derived from the Griffin and Keogh (1982) model for movement confidence. Additional samples of participant behaviors and longitudinal research efforts are needed to determine the impact of improved movement skills on the quality and quantity of peer relationships. Empirically validated playground assessments and teaching techniques are important components toward building a science of playground skill development
AD - Munroe-Meyer Institute for Genetics and Rehabilitation, University of Nebraska Medical Center, 985430 Nebraska Medical Center, Omaha, Nebraska, 68198-5430
UR - http://search.ebscohost.com/login.aspx?direct=true&db=cin20&AN=2009052539&site=ehost-live
ER -

TY - JOUR
ID - 4303
T1 - Worksite Physical Activity Policies and Environments in Relation to Employee Physical Activity
A1 - Crespo,Noe C.
A1 - Sallis,James F.
A1 - Conway,Terry L.
A1 - Saelens,Brian E.
A1 - Frank,Lawrence D.
Y1 - 2011/03//
KW - SportDiscus
KW - *PHYSICAL activity
KW - *SEDENTARY lifestyles
KW - EMPLOYEE health promotion -- Research
KW - work environment
KW - CROSS-sectional method
KW - EMPLOYEES -- Attitudes
KW - WASHINGTON (D.C.)
KW - accelerometer

KW - Health focus: physical activity
KW - Health Promotion
KW - Outcome measure: behavioral
KW - Physical Activity
KW - Prevention Research.Manuscript format: research
KW - race/ethnicity
KW - Research purpose: modeling/relationship testing
KW - Sedentary Behavior
KW - Setting: workplace
KW - Strategy: built environment and policy
KW - Study design: nonexperimental
KW - Target population circumstances: education/ income level
KW - Target population: adults
KW - Worksites
RP - NOT IN FILE
SP - 264
EP - 271
JF - American Journal of Health Promotion
JA - Am J Health Promot
VL - 25
IS - 4
N2 - Purpose. Examine associations between worksite physical activity promotion strategies and employees' physical activity and sedentary behaviors. Design. Cross-sectional. Setting. Seattleƒ_"King County, Washington and Baltimore, Marylandƒ_"Washington, D. C. regions. Subjects. Adults working outside the home (n = 1313). Mean age was 45 Añ10 years, 75.8% of participants were non-Hispanic white, 56% were male, and 51% had income ƒ% • $70,000/year. Measures. Participants reported demographic characteristics and presence/absence of nine physical activity promotion environment and policy strategies in their work environment (e.g., showers, lockers, physical activity programs). A worksite physical activity promotion index was a tally of strategies. Total sedentary and moderate-to-vigorous physical activity (MVPA) min/d were objectively assessed via 7-day accelerometry. Total job-related physical activity minutes and recreational physical activity minutes were self-reported with the International Physical Activity Questionnaire. Analysis. Mixed-effects models and generalized estimating equations evaluated the association of the worksite promotion index with physical activity and sedentary behavior, adjusting for demographics. Results. A higher worksite promotion index was significantly associated with higher total sedentary behavior(Iý= 3.97), MVPA (Iý= 1.04), recreational physical activity (Iý = 1.1 and odds ratio = 1.39; away from work and at work, respectively) and negatively with job-related physical activity (Iý= .90). Conclusions. Multiple worksite physical activity promotion strategies based on environmental supports and policies may increase recreational physical activity and should be evaluated in controlled trials. These findings are particularly important given the increasingly sedentary nature of employment. ABSTRACT FROM AUTHOR
SN - 08901171
UR -
https://login.proxy.bib.uottawa.ca/login?url=http://search.ebscohost.com/login.aspx?direct=true&db=s3h&AN=59600440&site=ehost-live
ER -

TY - JOUR
ID - 4304
T1 - The attitudes of Croatian primary healthcare workers towards injury prevention/Safety promotion for preschool children
A1 - Crnica,V.
A1 - Miskulin,M.
A1 - Srcek,I.
A1 - Mujkic,A.
Y1 - 2010/09/02/
KW - SportDiscus
RP - NOT IN FILE
SP - A95

EP - A95
JF - Injury Prevention
JA - Inj Prev
VL - 16
N2 - Objective In the period from 1995 to 2008, in Croatia, 2.553 children died because of injuries, making injury prevention efforts an important societal investment. The aim of this study was to establish the attitudes of primary healthcare workers in a sample comprised of gynaecologists and paediatricians working in the primary healthcare, family physicians and community nurses. Participants 373 primary healthcare workers (gynaecologists, paediatricians, family physicians and community nurses). Methods Anonymous self-administered questionnaire containing items on gender, age, length and type of work and the Croatian primary healthcare workers attitudes towards injury prevention/safety promotion for preschool children. Outcome Measures Attitudes towards injury prevention/safety promotion for pre-school children. Results The obtained results varied greatly for different questions. For example, almost all primary healthcare workers showed correct attitudes towards prevention of drowning during bathing, prevention of playground injuries and the prevention of traffic injuries by forbidding preschool children sitting in the front seat (366 or 98.1%) and forbidding standing between the front seats during the ride (367 or 98.4%). But only 46.4% subjects recognise provocation of vomiting after a child has swallowed a potential poison as an incorrect response. Conclusions Although a majority of primary healthcare workers demonstrated satisfactory attitudes, improvements are possible and necessary. A targeted, continuous education on the subject must be considered as one of the primary healthcare measures in the Croatian national program of healthcare measures. ABSTRACT FROM PUBLISHER
SN - 13538047
UR -
https://login.proxy.bib.uottawa.ca/login?url=http://search.ebscohost.com/login.aspx?direct=true&db=s3h&AN=66042626&site=ehost-live
ER -

TY - JOUR
ID - 4305
T1 - Fast talking
A1 - Crossman,Matt
Y1 - 2006/07/14/
KW - SportDiscus
KW - *AUTOMOBILE racing drivers
KW - *AUTOMOBILE racing
KW - NASCAR (Association)
RP - NOT IN FILE
SP - 16
EP - 20
JA - Sporting News
VL - 230
IS - 28
N2 - The article presents an interview with Darrell Waltrip and Carl Edwards, both NASCAR drivers. Waltrip recalls his childhood watching car races and his fascination on them. Edwards remembers going to school and playing at recess with cars. When asks who they want to be following them with a few laps left when they are leading a race, Edwards chooses Greg Biffle while Waltrip prefers Jeff Gordon and Tony Stewart
SN - 0038805X
UR -
https://login.proxy.bib.uottawa.ca/login?url=http://search.ebscohost.com/login.aspx?direct=true&db=s3h&AN=21461210&site=ehost-live
ER -

TY - JOUR
ID - 4307
T1 - The effects of playground markings on the physical self-perceptions of 10ƒ_"11-year-old school children
A1 - Crust,Lee
A1 - McKenna,Jim
A1 - Spence,Jon

A1 - Thomas,Catherine
A1 - Evans,Donna
A1 - Bishop,Daniel
Y1 - 2014/04//
KW - SportDiscus
KW - *PLAYGROUNDS
KW - *PHYSICAL activity
KW - *CHILD psychology
KW - *PHYSICAL education
KW - *SCHOOL children
KW - DESIGN & construction
KW - PLAY environments -- Psychological aspects
KW - SELF-perception in children
KW - Great Britain
KW - Elementary Education
KW - CY-PSPP
KW - physical self-concept
KW - self-esteem
KW - self-worth
RP - NOT IN FILE
SP - 179
EP - 190
JA - Physical Education & Sport Pedagogy
VL - 19
IS - 2
N2 - Background: Significant proportions of school children in the UK do not meet the minimum recommended daily requirements of 60-min moderate-intensity physical activity. Beyond taught classes, playtimes offer the opportunity for children to play and be physically active. Painted markings are one recent addition to school playgrounds that are inexpensive and have been shown to stimulate increased levels of physical activity among children during playtimes. Playground markings and supportive playground assistants can act as cues to increase physical activity. Purpose: While evidence exists to support the impact of playground markings on the levels of physical activity, no attempts have been made to assess the effects of playground markings on important indicators of psychological health such as self-esteem and physical self-concept. The way children view themselves from a physical perspective is likely to impact their future engagement in sport and physical activities. Given that the levels of physical activity have been found to be related to physical self-perceptions and self-esteem, and playground markings appear to facilitate increased physical activity, this study aimed to evaluate the effects of installing playground markings and training playground assistants on children's physical self-perceptions. Participants and setting: Participants were 218 primary school children (117 girls and 101 boys) aged 10 and 11 years. Informed consent was gained from all schools, and parents were given the choice to withdraw their child from the project before commencement of testing. Twelve schools received playground markings, while four further schools acted as controls. Playground assistants were trained to help facilitate use of markings by children. Playground markings consisted of painted lines and boxes. Data collection: The participants completed the Children and Youth Physical Self-Perception Profile under the supervision of trained research assistants. The behaviour of the children was also observed during the morning and lunch playtime periods prior to the installation of playground markings and again 8 months after installation. The System for Observing Children's Activity and Relationships during Play was used to assess playtime behaviour with 242 observations in total undertaken. Every sixth child to enter the playground was randomly observed for 10Aÿmin. Data analysis: Mixed -model ANOVAs were used to test for differences in physical self-perceptions of the children on the basis of intervention and over time. Percentage changes in observed physical activity and pro-social behaviours were calculated to determine differences between schools that received markings and those that did not. Findings: No significant differences were found in physical self-perceptions on the basis of intervention or over time. However, boys did report significantly higher perceptions of sport competence, physical conditioning, physical strength and physical self-worth than girls. Observed physical activity was found to increase by 7.5% in schools that received markings as opposed to a 7.7% decline in control schools. In addition, pro-social behaviours were found to increase by 6.7% following the installation of playground markings. Conclusion: These findings indicate that while playground markings can facilitate

increased physical activity during playtimes, this does not translate into enhanced self-perceptions in children when evaluated 8 months after intervention. ABSTRACT FROM PUBLISHER
SN - 17408989
UR -
https://login.proxy.bib.uottawa.ca/login?url=http://search.ebscohost.com/login.aspx?direct=true&db=s3h&AN=94773637&site=ehost-live
ER -

TY - JOUR
ID - 4308
T1 - Otrosko igrisce v Sloveniji. / Playgrounds in Slovenia
A1 - Cuk,Ivan
A1 - Bucar,Maja
A1 - Hosta,Milan
A1 - Videmsek,Mateja
A1 - Bricelj,Andraz
Y1 - 2007///
KW - SportDiscus
KW - *PLAYGROUNDS
KW - Surveys
KW - Questionnaires
KW - Management
KW - Slovenia
RP - NOT IN FILE
SP - 6
EP - 10
JA - Sport: Revija Za Teoreticna in Prakticna Vprasanja Sporta
VL - 55
IS - 1
CY - ;
N2 - Based on a survey questionnaire completed by those running the campaign Hurrah, leisure time! under the auspices of the Union for Children's and Youth Sports, the current state of 70 Slovenian playgrounds (in all regions) was analyzed in terms of their size, proprietorship, management, and playing conditions. The current state needs to be improved considerably in terms of safety, usability and comfort so as to bring the playgrounds back to life. [ABSTRACT FROM AUTHOR]
SN - 03537455
UR -
https://login.proxy.bib.uottawa.ca/login?url=http://search.ebscohost.com/login.aspx?direct=true&db=s3h&AN=SPHS-1062814&site=ehost-live;http://articles.sirc.ca/search.cfm?id=S-1062814;http://www.fsp.uni-lj.si/rsport/
L2 - http://articles.sirc.ca/search.cfm?id=S-1062814
ER -

TY - JOUR
ID - 4309
T1 - Poskodbe otrok na otroskih igriscih. / Children's injuries in playgrounds
A1 - Cuk,Ivan
A1 - Bucar,Maja
A1 - Videmsek,Mateja
A1 - Hosta,Milan
Y1 - 2007///
KW - SportDiscus
KW - *WOUNDS & injuries
KW - *PLAYGROUNDS
KW - age
KW - Child
KW - OCCURRENCE
RP - NOT IN FILE

SP - 26
EP - 28
JA - Sport: Revija Za Teoreticna in Prakticna Vprasanja Sporta
VL - 55
IS - 1
CY - ;
N2 - The authors of the article researched the available information and investigated the incidence of injuries in playgrounds, the age groups of the injured children and the types of injuries. The number of injuries in the 'developed' world is on the increase and there are many fatal outcomes as well. The current situation in Slovenia has not yet been researched as records on children's injuries are not kept separately for playgrounds. In spite of that, the average 17 fatal outcomes yearly among preschool children due to inadvertent injuries (including road accidents) and poisoning is appalling and it is therefore reasonable to consider broadening the preventive measures, including those in children's playgrounds. [ABSTRACT FROM AUTHOR]
SN - 03537455
UR - https://login.proxy.bib.uottawa.ca/login?url=http://search.ebscohost.com/login.aspx?direct=true&db=s3h&AN=SPHS-1062817&site=ehost-live;http://articles.sirc.ca/search.cfm?id=S-1062817;http://www.fsp.uni-lj.si/rsport/
L2 - http://articles.sirc.ca/search.cfm?id=S-1062817
ER -

TY - JOUR
ID - 4310
T1 - Impact of an intergenerational program on 4th graders' attitudes toward elders and school behaviors
A1 - Cummings,S.M.
A1 - Williams,M.M.
A1 - Ellis,R.A.
Y1 - 2002/11//
N1 - Accession Number: 2003107320. Language: English. Entry Date: 20030822. Revision Date: 20091218. Publication Type: journal article; research; tables/charts. Journal Subset: Allied Health; Peer Reviewed; USA. Instrumentation: Children's Attitudes Toward the Elderly (CATE) (Jantz et al). Grant Information: HCA Foundation. No. of Refs: 30 ref. NLM UID: 9890976. Email: scumming@utk.edu
KW - CINAHL
KW - Attitude to Aging -- In Infancy and Childhood
KW - Child Behavior
KW - Intergenerational Relations
KW - Aged
KW - Attitude Measures
KW - Child
KW - Descriptive Statistics
KW - Funding Source
KW - P-Value
KW - T-Tests
KW - Human
RP - NOT IN FILE
SP - 91
EP - 107
JA - Journal of Human Behavior in the Social Environment
VL - 6
IS - 3
N2 - The rapid growth of the elderly population coupled with research demonstrating the existence of ageist stereotypes even among very young children have led to the rise of intergenerational programs. Very limited research has been conducted, however, to demonstrate the effectiveness of these programs in producing attitudinal change or other beneficial outcomes for at-risk children. This study examined the impact of an intergenerational program on children's attitudes toward elders and on their school-based behaviors. Eighty-one 4th grade children enrolled in an inner-city school participated in the study. Forty-one children, with matched seniors partners, engaged in activities based on the 4th grade core curriculum within an outdoor classroom environment, while the remaining 40 children participated in usual core curriculum activities. At the conclusion

of the program those children who participated in the program had significantly more positive attitudes towards the elderly and significantly better school behavioral grades than did non-participating students. Implications and recommendations for intergenerational programming are discussed
SN - 1091-1359
AD - University of Tennessee, College of Social Work, 193 East Polk Avenue, Nashville, TN 37210
UR - http://search.ebscohost.com/login.aspx?direct=true&db=cin20&AN=2003107320&site=ehost-live
ER -

TY - JOUR
ID - 4312
T1 - Play through the eyes of children: use of cameras to study after-school use of leisure time and leisure space by pre-adolecent children. / Regard des enfants sur le jeu: utilisation d'appareils photos pour l'etude du temps et de l'espace de jeu des preadolescents apres l'ecole
A1 - Cunningham,C.
A1 - Jones,M.
Y1 - 1996///
KW - SportDiscus
KW - *PLAY
KW - *LEISURE
KW - Child
RP - NOT IN FILE
SP - 341
EP - 361
JA - Loisir et Societe
VL - 19
IS - 2
CY - ;
N2 - This paper reports on the use of cameras by children to record how they used their play environments during after-school play in an Australian provincial city. The study was one of six carried out concurrently to illuminate the complexity of children's after-school leisure and the environment in which it takes place. Twenty four children, twelve boys and twelve girls, were issued with disposable camaeras to photograph their play in after-school time and leisure space. They produced 342 usable images which were analyzed by location, setting and activities. There was no measurable gender difference in choice of play location, but place of residence strongly influenced this choice. Both boys and girls had a strong tendency to choose open and natural locations, and naturalistic settings dominated play spaces. While the expected multiplicity of activities in children's free play is demonstrated in the photographs, so is the importance of solitude and reflection. The photos also reveal a much higher incidence of mxied-gender play than is commonly reported for this age group
Le present article traite de l'utilisation d'appareils photos par les enfants d'une ville provinciale de l'Australie afin d'illustrer la maniere dont ces jeunes utilisent leur environnement lorsqu'ils jouent apres l'ecole. Il s'agit d'une de six etudes concomitantes visant a jeter un eclaireage sur la complexite du loisir des enfants et de l'environnement ou ces activites se deroulent une fois l'ecole terminee. Vingt-quatre enfants, dont douze garcons et douze filles, ont recue un appareil jetable leur permettant de photographier leurs jeux dans leur environnement de loisir. Ils ont pris 342 photos qui ont ensuite ete anlaysees en fonction du lieu, de l'environnement et des activites elles-memes. Aucune difference ne ressort entre les filles et les garcons quant au choix du lieu; qui depend en grande partie du domicile des jeunes. Tous manifestent une tendance marquee en faveur des espaces naturels comme lieurx et environnements de jeu. En outre, bien que les photos revelent, comme on s'y attendait, la grnade diversite des activites de jeu libre des enfants, elles font egalement ressortir l'importance de la solitude et de la reflexion. Elles permettent aussi de constater que les jeux regroupant les jeunes des deux sexes sont beaucoup plus frequents que ne le laissent generalement entrevoir les etudes sur ce groupe d'age
SN - 07053436
UR -
https://login.proxy.bib.uottawa.ca/login?url=http://search.ebscohost.com/login.aspx?direct=true&db=s3h&AN=SPH412858&site=ehost-live;http://articles.sirc.ca/search.cfm?id=412858
L2 - http://articles.sirc.ca/search.cfm?id=412858
ER -

TY - JOUR
ID - 4313
T1 - Small Things Make a Big Difference: Particulate Matter and Exercise
A1 - Cutrufello,Paul T.
A1 - Smoliga,James M.
A1 - Rundell,Kenneth W.
Y1 - 2012/12//
KW - SportDiscus
KW - *ASTHMA
KW - *PREVENTION
KW - *ADAPTATION (Physiology)
KW - *ANTI-inflammatory agents
KW - *ATHLETIC ability
KW - *AUTOMOBILES
KW - *BLOOD-vessels -- Dilatation
KW - *CARDIOPULMONARY system -- Physiology
KW - *CARDIOVASCULAR system -- Physiology
KW - *DIETARY supplements
KW - *ECOLOGY
KW - *ENVIRONMENTAL health
KW - *EXERCISE physiology
KW - *HEALTH
KW - *INFLAMMATION
KW - *LUNGS
KW - *MEDLINE
KW - *NITRIC oxide
KW - *PASSIVE smoking
KW - *PULMONARY artery
KW - *RESPIRATION -- Measurement
KW - *SKATING
KW - *EVIDENCE-based medicine
KW - *SPORTS events
KW - *VASOCONSTRICTION
KW - *VITAL capacity (Respiration)
KW - Risk Factors
KW - Environmental Exposure
KW - Air Pollution
KW - Antioxidants
KW - DOSE-response relationship (Biochemistry)
KW - endothelium
KW - HORMONE antagonists
KW - indoor air pollution
KW - LEUKOTRIENES
KW - METROPOLITAN areas
KW - ONLINE information services
KW - Ozone
KW - Smoke
KW - Time
KW - Transportation
KW - SYSTEMATIC reviews (Medical research)
KW - Particulate Matter
RP - NOT IN FILE
SP - 1041
EP - 1058
JF - Sports Medicine
JA - Sports Med
VL - 42

IS - 12
N2 - The increased risk of morbidity and mortality among adults and children with pre-existing cardiovascular or respiratory illness from emission-derived particulate matter (PM) is well documented. However, the detrimental effects of PM inhalation on the exercising, healthy population is still in question. This review will focus on the acute and chronic responses to PM inhalation during exercise and how PM exposure influences exercise performance. The smaller ultrafine PM (<0.01 I¬m aerodynamic diameter) appears to have the most severe health consequences compared with the larger coarse PM (2.5 <PM <10 I¬m aerodynamic diameter). While the response to PM inhalation may affect those with a pre-existing condition, the healthy population is not immune to the effects of PM inhalation, especially during exercise. This population, including the competitive athlete, is susceptible to pulmonary inflammation, decreased lung function (both acute and chronic in nature), the increased risk of asthma, vascular endothelial dysfunction, mild elevations in pulmonary artery pressure and diminished exercise performance. PM exposure is usually associated with vehicular traffic, but other sources of PM, including small engines from lawn and garden equipment, cigarette smoke, wood smoke and cooking, may also impair health and performance, The physiological effects of PM are dependent on the source of PM, various environmental factors, physical attributes and nature of exercise. There are a number of measures an athlete can take to reduce exposure to PM, as well as the deleterious effects that result from the inevitable exposure to PM. Considering the acute and chronic physiological responses to PM inhalation, individuals living and exercising in urban areas in close proximity to major roadways should consider ambient air pollution levels (in particular, PM and ozone) prior to engaging in vigorous exercise, and those exposed to PM through other sources may need to make lifestyle alterations to avoid the deleterious effects of PM inhalation. Although it is clear that PM exposure is detrimental to healthy individuals engaging in exercise, further research is necessary to better understand the role of PM on athlete health and performance, as well as measures that can attenuate the harmful effects of PM. ABSTRACT FROM AUTHOR
SN - 01121642
UR -
https://login.proxy.bib.uottawa.ca/login?url=http://search.ebscohost.com/login.aspx?direct=true&db=s3h&AN=83804907&site=ehost-live
ER -

TY - JOUR
ID - 4315
T1 - Description of the physical activity of young children using movement sensor and observation methods
A1 - Danner,F.
A1 - Noland,M.
A1 - McFadden,M.
A1 - DeWalt,K.
A1 - Kotchen,J.M.
Y1 - 1991/02//
KW - SportDiscus
KW - *STRESS (Physiology)
KW - INFANTS
KW - Calibration
KW - Methodology
KW - Movement
KW - Child
KW - Observation
RP - NOT IN FILE
SP - 11
EP - 20
JF - Pediatric Exercise Science
JA - Pediatr Exerc Sci
VL - 3
IS - 1
CY - ;
N2 - This study examined the validity of the Caltrac motion sensor for measuring physical activity in young children and described change over time in physical activity among 47 preschool children from obese and nonobese families. Children were videotaped in a controlled setting while wearing Caltrac motion sensors and

also wore Caltracs at home. Caltrac readings and an observational measure of physical activity were significantly related in both Year 1 (r=.86) and Year 2 (r=.83), and outside play was associated with higher Caltrac scores (r=.43). In addition, physical activity among young girls with obese parents decreased significantly from Year 1 to Year 2
SN - 08998493
UR -
https://login.proxy.bib.uottawa.ca/login?url=http://search.ebscohost.com/login.aspx?direct=true&db=s3h&AN=SPH271454&site=ehost-live;http://articles.sirc.ca/search.cfm?id=271454
L2 - http://articles.sirc.ca/search.cfm?id=271454
ER -

TY - JOUR
ID - 4317
T1 - Angling activity of urban youth: factors associated with fishing in a metropolitan context
A1 - Dargitz,R.E.
Y1 - 1988///
KW - SportDiscus
KW - *FISHING
KW - teenagers
KW - CITY dwellers
KW - Socialization
KW - Questionnaires
KW - RACE
KW - WATER -- Pollution
KW - SEX FACTOR
RP - NOT IN FILE
SP - 192
EP - 207
JF - Journal of Leisure Research
VL - 20
IS - 3
CY - ;
N2 - Surprisingly there apparently have been no studies which have investigated childhood experiences in angling and other types of outdoor recreation within the context of an urban setting. Such experiences obviously confront problems with access, availability, and ever increasing problems with polluted waters and contaminated fish. The concepts of race, gender, residence and angling socialization are applied to angling participation by urban youth in a mid western setting. Data from a sample of 751 teenagers representing students enrolled in science classes in 10 different schools in a midwestern city suggest that gender appears to be a more important factor in angling socialization than either race or residence. Angling socialization in turn is very important in determining the children's angling activities. Children who have anglers in their households are more likely to engage in fishing more frequently and are more likely to enjoy angling as a leisure activity than are children who do not have anglers in their households. Because males are more likely to enjoy angling than are females, teenage boys are more active within the urban context. In such a context although black teenage boys are not more likely to fish in polluted waters, they do appear to run a higher risk of consuming contaminated fish
SN - 00222216
UR -
https://login.proxy.bib.uottawa.ca/login?url=http://search.ebscohost.com/login.aspx?direct=true&db=s3h&AN=SPH231562&site=ehost-live;http://articles.sirc.ca/search.cfm?id=231562
L2 - http://articles.sirc.ca/search.cfm?id=231562
ER -

TY - JOUR
ID - 4318
T1 - Epidemiology of Severe Injuries Among United States High School Athletes
A1 - Darrow,Cory J.
A1 - Collins,Christy L.

A1 - Yard,Ellen E.
A1 - Comstock,R.Dawn
Y1 - 2009/09//
KW - SportDiscus
KW - *EPIDEMIOLOGY
KW - *PUBLIC health
KW - *SPORTS medicine
KW - *SPORTS injuries
KW - *BIOMECHANICS
KW - *WOUNDS & injuries
KW - *PREVENTION
KW - *BASKETBALL players
KW - *HEALTH
KW - *MEDICAL care
KW - *PHYSICAL education
KW - *HIGH school athletes
KW - Research
KW - high school
KW - injury
KW - severe
KW - Sports
RP - NOT IN FILE
SP - 1798
EP - 1805
JF - American Journal of Sports Medicine
JA - Am J Sports Med
VL - 37
IS - 9
N2 - Background: Over 7 million students participate in high school athletics annually. Despite numerous health benefits, high school athletes are at risk for injury. Hypothesis: Severe injury rates and patterns differ by gender and type of exposure. Study Design: Descriptive epidemiology study. Methods: Sports-related injury data were collected during the 2005-2007 academic years from 100 nationally representative United States high schools via RIO (Reporting Information Online). Severe injury was defined as any injury that resulted in the loss of more than 21 days of sports participation. Results: Participating certified athletic trainers (ATCs) reported 1378 severe injuries during 3 550 141 athlete-exposures (0.39 severe injuries per 1000 athletic exposures). Football had the highest severe injury rate (0.69), followed by wrestling (0.52), girls' basketball (0.34), and girls' soccer (0.33). The rate in all boys' sports (0.45) was higher than all girls' sports (0.26) (rate ratio [RR], 1.74; 95% confidence interval [CI], 1.54-1.98; P < .001). However, among directly comparable sports (soccer, basketball, and baseball/softball), girls sustained a higher severe injury rate (0.29) than boys (0.23) (RR, 1.28; 95% CI, 1.08-1.52; P = .006). More specifically, girls' basketball had a higher rate (0.34) than boys' basketball (0.24) (RR, 1.43; 95% CI, 1.10-1.86; P = .009). Differences between boys' and girls' soccer and baseball/softball were not statistically significant. The severe injury rate was greater in competition (0.79) than practice (0.24) (RR, 3.30; 95% CI, 2.97-3.67; P < .001). Nationally, high school athletes sustained an estimated 446 715 severe injuries from 2005-2007. The most commonly injured body sites were the knee (29.0%), ankle (12.3%), and shoulder (10.9%). The most common diagnoses were fractures (36.0%), complete ligament sprains (15.3%), and incomplete ligament sprains (14.3%). Of severe sports injuries, 0.3% resulted in medical disqualification for the athletes' career, and an additional 56.8% resulted in medical disqualification for the entire season. One in 4 (28.3%) severe injuries required surgery, with over half (53.9%) being knee surgeries. Conclusion: Severe injury rates and patterns varied by sport, gender, and type of exposure. Because severe injuries negatively affect athletes' health and often place an increased burden on the health care system, future research should focus on developing interventions to decrease the incidence and severity of sports-related injuries. ABSTRACT FROM AUTHOR
SN - 03635465
UR -
https://login.proxy.bib.uottawa.ca/login?url=http://search.ebscohost.com/login.aspx?direct=true&db=s3h&AN=47788412&site=ehost-live
ER -

TY - JOUR
ID - 4322
T1 - Metabolic cost of exercise and physical performance in children with some observations on external loading
A1 - Davies,C.T.M.
Y1 - 1980///
KW - SportDiscus
KW - *PHYSIOLOGY
KW - *OXYGEN -- Physiological transport
KW - *RUNNING
KW - *TREADMILL exercise
KW - *CARDIOVASCULAR fitness
KW - *STRESS (Physiology)
KW - Child
KW - SEX FACTOR
RP - NOT IN FILE
N2 - Metabolic cost of running for 24 boys and girls aged 11 to 13 years old was studied on a treadmill and compared to performance times during outdoor races. No significant aerobic differences were found between the two sexes. The authors found that though the children had highly developed aerobic power, their conversion of aerobic energy into mechanical work was not the most economic
SN - 03015548
UR - https://login.proxy.bib.uottawa.ca/login?url=http://search.ebscohost.com/login.aspx?direct=true&db=s3h&AN=SPH88906&site=ehost-live;http://articles.sirc.ca/search.cfm?id=88906
L2 - http://articles.sirc.ca/search.cfm?id=88906
ER -

TY - JOUR
ID - 4323
T1 - Planning an elementary Field Day
A1 - Davis,R.G.
Y1 - 2003///Fall
KW - SportDiscus
KW - *PHYSICAL education & training
KW - *TEACHING
KW - *OUTDOOR recreation
RP - NOT IN FILE
SP - 10
EP - 13
JA - Virginia Journal
VL - 25
IS - 2
CY - ;
N2 - Presents 22 activities teachers can incorporate into the Field Day program for elementary school children
SN - 07394586
UR - https://login.proxy.bib.uottawa.ca/login?url=http://search.ebscohost.com/login.aspx?direct=true&db=s3h&AN=SPHS-923370&site=ehost-live;http://articles.sirc.ca/search.cfm?id=S-923370
L2 - http://articles.sirc.ca/search.cfm?id=S-923370
ER -

TY - JOUR
ID - 4324
T1 - Stroll away fat
A1 - Davis,Valerie Kramer
Y1 - 2006/10//

KW - SportDiscus
KW - *EXERCISE
KW - *HEALTH
KW - *CARDIOVASCULAR fitness
KW - *JOINTS -- Range of motion
KW - MOTHER & child
KW - United States
RP - NOT IN FILE
SP - 53
EP - 53
JA - Health (Time Inc.)
VL - 20
IS - 8
N2 - This article reports on stroller fitness programs that are popping up across the United States. Most of the hourlong outdoor classes begin with a stroller-pushing walk or job, then progress strength moves which include modified push-ups and stretches. The programs offer cardio, strength, and flexibility training
SN - 1059938X
UR -
https://login.proxy.bib.uottawa.ca/login?url=http://search.ebscohost.com/login.aspx?direct=true&db=s3h&AN=22481808&site=ehost-live
ER -

TY - JOUR
ID - 4325
T1 - School Engagement Among Aboriginal Students in Northern Canada: Perspectives From Activity Settings Theory
A1 - Davison,Colleen M.
A1 - Hawe,P.E.N.E.
Y1 - 2012/02//
KW - SportDiscus
KW - *MOTIVATION (Psychology)
KW - *STUDENTS
KW - *HEALTH
KW - SCHOOL environment -- Evaluation
KW - ARCHIVES
KW - SCHOOL dropouts
KW - Focus Groups
KW - Indians
KW - INTERVIEWING
KW - EDUCATION theory
KW - Canada
RP - NOT IN FILE
SP - 65
EP - 74
JF - Journal of School Health
JA - J Sch Health
VL - 82
IS - 2
N2 - BACKGROUND: Educational disengagement is a public health concern among Aboriginal populations in many countries. It has been investigated previously in a variety of ways, with the conventional focus being on the children themselves. Activity settings are events and places, theorized in terms of their symbols, roles, time frame, funds, people, and physical location. According to the theory, particular behaviors and experiences are shaped by different configurations among these elements. This study explored how activity settings theory might provide new insight on school engagement. METHODS: Ethnographic study was undertaken at a grades primary to 12 school in a remote First Nations community in Canada's Northwest Territories. We collected data through interviews, focus groups, archival material, and field notes from 7 months of participant observation. An activity settings model acted as template for data collection and interpretation. RESULTS: Different aspects

of the school's physical layout, routines, procedures, transport systems, mix of people, and rules were able to be systemically assessed and classified as either creating or eroding engagement. CONCLUSION: This study applies an activity setting analysis to school engagement, thereby allowing researchers to investigate the dynamic and nested nature of context or environmental influences on engagement. It provides grounded observations that invite direct opportunities for action on dimensions that teachers and practitioners might not otherwise 'see.' ABSTRACT FROM AUTHOR
SN - 00224391
UR -
https://login.proxy.bib.uottawa.ca/login?url=http://search.ebscohost.com/login.aspx?direct=true&db=s3h&AN=70285274&site=ehost-live
ER -

TY - JOUR
ID - 4326
T1 - GLOBAL QUANTITY DIFFERENCES IN MOTOR ABILITIES OF PRE-SCHOOL BOYS. / GLOBALNE KVANTITATIVNE RAZLIKE U MOTORIŽOKIM SPOSOBNOSTIMA DJEŽOAKA PRED • ÿKOLSKOG UZRASTA
A1 - D□_ibriŽØ,D.□
A1 - HuremoviŽØ,Tarik
A1 - AhmiŽØ,Damir
Y1 - 2009/06//
KW - SportDiscus
KW - *MOTOR ability
KW - *PHYSICAL education
KW - *KINESIOLOGY
KW - Educational Programs
KW - EDUCATIONAL tests & measurements
KW - PRESCHOOL children
KW - MALES
KW - BOYS
KW - canonic discriminative analysis
KW - motor abilities
KW - djeŽ_aci
KW - kanoniŽ_ka diskriminativna analiza
KW - motoriŽ_ke sposobnosti
RP - NOT IN FILE
SP - 56
EP - 60
JA - Sport Scientific & Practical Aspects
VL - 6
IS - 1
N2 - Based on the sample of 58 male respondents (30 respondents were a part of experimental group and 28 of control group) age from five to five and a half years old, initial and final measuring was conducted with the aim to determine global quantitative changes in motor abilities between these two groups. Experimental group carried out specially designed physical education program suitable for pre-school children that lasted for three days, weekly totally 60 minutes in the Gym managed by physical education professor, while control group followed current program for pre-school education in the classroom (playground) managed by kindergarten teacher. Possible differences were determined between experimental and control group in initial and final measuring in a period of three months participation in physical education program To test motor abilities we applied 6 tests. We applied canonic discriminative analysis to determine global quantitative differences between these two groups in the area of motor abilities. Obtained results indicate there are no differences between groups in initial measuring, but in final measuring we noticed statistically significant differences regarding experimental group which means there has been some positive transformations in motor abilities of this group. Possible reason lies in program organized for experimental group as well as different approach to realization of treatment and material working conditions where the classes were carried out. ABSTRACT FROM AUTHOR
SN - 18404413

UR -
https://login.proxy.bib.uottawa.ca/login?url=http://search.ebscohost.com/login.aspx?direct=true&db=s3h&AN=44167784&site=ehost-live
ER -

TY - JOUR
ID - 4327
T1 - Environmental Correlates of Physical Activity in a Sample of Belgian Adults
A1 - De Bourdeaudhuij,Ilse
A1 - Sallis,James F.
A1 - Saelens,Brian E.
Y1 - 2003/09//
KW - SportDiscus
KW - *PHYSICAL fitness
KW - *EXERCISE
KW - *RECREATION areas
KW - *PHYSICAL fitness for youth
KW - URBAN planning
KW - Community Health Services
KW - community design
KW - Environment
KW - Physical Activity
KW - Prevention Research
KW - Walkability
RP - NOT IN FILE
SP - 83
EP - 92
JF - American Journal of Health Promotion
JA - Am J Health Promot
VL - 18
IS - 1
N2 - Purpose. This study investigated the variance in sitting, walking, and moderate and vigorous physical activity explained by neighborhood design and recreational environmental variables above and beyond the variance accounted for by individual demographic variables. Design. Cross-sectional analyses of self-reported survey data. Setting. A random sample of inhabitants of Ghent, Belgium, aged 18-65 years, was drawn. Subjects. Five hundred twenty-one adults completed questionnaires (52.1 % response rate). The average age of the sample was 41 years, and 48.2% were female. Measures. A questionnaire developed to assess neighborhood design and recreational environmental variables with a total of 81 items was administered. The environmental questionnaire showed acceptable to good reliability and acceptable validity. The previously validated International Physical Activity Questionnaire (IPAQ) was used to quantify physical activity in the past 7 days. Additional demographic information was also obtained. Results. Regression analyses showed that environmental variables were related to all types of physical activity in both sexes. However, the range of variance explained by the models including demographic and environmental variables was low, only 5 % to 13%. Minutes of walking and of moderate-intensity activity were related to quality of sidewalks and accessibility of shopping and public transportation. Vigorous physical activity was related to presence of activity supplies in the home and number of convenient activity facilities outside the home. Conclusions. Both neighborhood design and recreational environment variables had small but significant associations with multiple types of physical activity in a sample of Belgian adults. ABSTRACT FROM AUTHOR
SN - 08901171
UR -
https://login.proxy.bib.uottawa.ca/login?url=http://search.ebscohost.com/login.aspx?direct=true&db=s3h&AN=12381118&site=ehost-live
ER -

TY - JOUR
ID - 4330
T1 - Patients With Ice Hockey Injuries Presenting to US Emergency Departments, 1990--2006

A1 - Deits,Jeff
A1 - Yard,Ellen E.
A1 - Collins,Christy L.
A1 - Fields,Sarah K.
A1 - Comstock,R.Dawn
Y1 - 2010/09//
KW - SportDiscus
KW - *EPIDEMIOLOGY
KW - *HOCKEY injuries
KW - *WOUNDS & injuries
KW - AGE distribution (Demography)
KW - Analysis of Variance
KW - CHI-squared test
KW - COMPUTER software
KW - Confidence Intervals
KW - Emergency Medical Services
KW - Research
KW - LONGITUDINAL method
KW - Mathematics
KW - medical education
KW - PUBLIC health surveillance
KW - Regression Analysis
KW - SAMPLING (Statistics)
KW - SEX distribution (Demography)
KW - data analysis
KW - CONTINUING education units
KW - Severity of Illness Index
KW - Evaluation
KW - Ohio
KW - United States
KW - body checking
KW - Epidemiology
KW - face shields
KW - injury surveillance
KW - mouth guards
RP - NOT IN FILE
SP - 467
EP - 474
JA - Journal of Athletic Training
VL - 45
IS - 5
N2 - Context: Although the number of US ice hockey participants doubled from 1990 to 2006, no nationally representative studies have examined US ice hockey injuries among participants of all ages during this period. Objective: To describe patients with ice hockey injuries presenting to a representative sample of US emergency departments (EDs) from 1990 through 2006. Design: Prospective injury surveillance study. Setting: The US Consumer Product Safety Commission collects data from 100 nationally representative EDs via the National Electronic Injury Surveillance System (NEISS). Patients or Other Participants: Individuals injured while playing ice hockey and presenting to a NEISS-affiliated ED from 1990 through 2006. Main Outcome Measure(s): Incidence and patterns of ice hockey-related injuries. Results: From 1990 through 2006, 8228 patients with ice hockey-related injuries presented to NEISS-affiliated EDs, representing an estimated 302 368 ice hockey-related injuries sustained nationally during this time. Injuries occurred predominantly among males (93.5%). More than half of the injured were aged 9 to 14 years (28.9%) or 15 to 18 years (30.1%), and injury incidence in these age groups increased over the study period ($P = .009$ and $P < .001$, respectively). The most commonly injured body sites were the face (19.1%), wrist/hand/finger (14.1%), shoulder/upper arm (13.8%), and lower leg/ankle/foot (11.1%). Lacerations (27.0%), contusions/abrasions (23.6%), fractures (17.3%), and sprains/strains (16.9%) were the most common injuries. Falls (16.5%), contact with boards (13.6%), and contact with stick (13.0%) were the most common injury mechanisms. Compared with those aged 9 to 18 years, those aged 2 to 8 years and

those older than 18 years sustained larger proportions of face (injury proportion ratio [IPR] = 2.66; 95%confidence interval [CI] = 2.29, 3.08) and mouth (IPR = 4.34; 95% CI = 2.87, 6.56) injuries. Concussions were more common among those aged 2 to 18 years (9.0%) than in those who were older than 18 years (3.7%) (IPR = 2.47; 95% CI = 1.75, 3.49). Conclusions: Ice hockey injury patterns vary by age and sex. Our findings indicate that many trips to the ED might be prevented by using protective equipment appropriately.
ABSTRACT FROM AUTHOR
SN - 10626050
UR -
https://login.proxy.bib.uottawa.ca/login?url=http://search.ebscohost.com/login.aspx?direct=true&db=s3h&AN=53707203&site=ehost-live
ER -

TY - JOUR
ID - 4331
T1 - Rhinitis and its impact on sports
A1 - Delgado,L.
A1 - Moreira,A.
A1 - CapAœo-Filipe,M.
Y1 - 2006/05//
N1 - Accession Number: 2009250058. Language: English. Entry Date: 20071102. Revision Date: 20091218. Publication Type: journal article; research; systematic review; tables/charts. Journal Subset: Biomedical; Continental Europe; Editorial Board Reviewed; Europe; Expert Peer Reviewed; Online/Print; Peer Reviewed. Special Interest: Evidence-Based Practice; Patient Safety; Sports Medicine. No. of Refs: 67 ref. NLM UID: 8915137. Email: ldelgado@med.up.pt
KW - CINAHL
KW - Asthma,Exercise-Induced -- Risk Factors
KW - Rhinitis -- Therapy
KW - Administration,Intranasal -- Utilization
KW - Administration,Oral -- Utilization
KW - Administration,Topical -- Utilization
KW - adolescence
KW - Adrenal Cortex Hormones -- Therapeutic Use
KW - Adult
KW - Allergens -- Adverse Effects
KW - Asthma,Exercise-Induced -- Prevention and Control
KW - Athletes,Elite -- Evaluation
KW - causal attribution
KW - Child
KW - Cholinergic Antagonists -- Therapeutic Use
KW - Clinical Assessment Tools
KW - Confidence Intervals
KW - Conjunctivitis -- Complications
KW - Cross Sectional Studies
KW - Desensitization,Immunologic -- Methods
KW - Disease Management -- Methods
KW - Early Intervention -- Utilization
KW - Environmental Exposure -- Adverse Effects
KW - Epidemiological Research
KW - exercise physiology
KW - Histamine H1 Antagonists -- Therapeutic Use
KW - Immunotherapy -- Utilization
KW - Inflammation Mediators -- Therapeutic Use
KW - Leukotrienes -- Therapeutic Use
KW - Medical Practice,Evidence-Based
KW - MEDLINE
KW - Nasal Obstruction -- Physiopathology
KW - Nose -- Physiology

KW - Odds Ratio
KW - Ozone -- Adverse Effects
KW - Particulate Matter -- Adverse Effects
KW - Phenylephrine -- Therapeutic Use
KW - physical performance
KW - Pollen -- Adverse Effects
KW - Prospective Studies
KW - Quality of Life -- Standards
KW - Questionnaires
KW - Rhinitis -- Complications
KW - Rhinitis -- Diagnosis
KW - Running,Distance -- Adverse Effects
KW - Steroids -- Therapeutic Use
KW - Structured Questionnaires
KW - Vasoconstrictor Agents,Nasal -- Therapeutic Use
KW - Human
RP - NOT IN FILE
SP - 98
EP - 105
JA - Allergy & Clinical Immunology International
VL - 18
IS - 3
N2 - Background: This paper reviews the recent literature on rhinitis in athletes and the relationship of nasal and exercise physiology with environmental factors with a negative impact on sport performance, including the relationship between allergic rhinitis and exercise-induced asthma. The management and therapeutic plan for the elite athlete with rhinitis is also reviewed. Methods/Data base: Computer-assisted literature search on Medline for published data on rhinitis and exercise, or rhinitis and athletes. Published articles, abstracts, and conference proceedings were selected. Results: A high prevalence of rhinitis and rhinoconjunctivitis has been reported in training athletes (13.3-41%). In athletes with rhinitis, factors with a potential negative impact on sports performance include outdoor exposure to pollen and pollutants (ozone and particulate mater), or to sport-specific environmental conditions (e.g., cold dry air, indoor chlorine). Allergic rhinitis and sensitization to seasonal allergens is associated with exercise-induced bronchoconstriction in young adults and, in athletes, with decreased quality of life and athletic performance. The management of rhinitis in elite athletes includes: avoiding exposure to peak levels of allergens and pollutants, reducing symptoms and improving nasal function, and using therapies according to practice guidelines and complying with anti-doping regulation (which may require a medical notification). Conclusions: Rhinitis is a very common disease that, in athletes, can significantly impair exercise performance and increase the risk of exercise-induced symptoms. An early recognition and diagnosis are crucial for adequate management, aiming to improve nasal function and reduce the risk of asthma during exercise and competition
SN - 1097-1424
AD - Department of Immunology, Faculty of Medicine, University of Porto, Alameda Prof. HernA›ni Monteiro, 4200-319 Porto, Portugal
UR - http://search.ebscohost.com/login.aspx?direct=true&db=cin20&AN=2009250058&site=ehost-live
ER -

TY - JOUR
ID - 4332
T1 - Health-related fitness and nutritional practices: can they be enhanced in upper elementary school students?
A1 - Derri,V.
A1 - Aggeloussis,N.
A1 - Petraki,C.
Y1 - 2004///Winter
KW - SportDiscus
KW - *PHYSICAL fitness
KW - *CARDIOVASCULAR fitness
KW - *HEALTH education
KW - *NUTRITION

KW - *HEALTH promotion
KW - Elementary Schools
KW - Greece
KW - Child
RP - NOT IN FILE
SP - 35
EP - 44
JA - Physical Educator
VL - 61
IS - 1
CY - ;
N2 - The purpose of the present study was to investigate the effect of an eight-week health-related fitness and nutrition education program on fitness components and dietary habits in upper elementary school students. Forty children from the fifth and- sixth grade, 10 to 12 years of age (M = 11.2, SD = 1.1), participated in the study. The experimental group consisted of 20 children who participated in the program three days per week for one hour outside the time allotted for school physical education. The control group participated in the typical school physical education program. Health-related fitness components (cardiorespiratory endurance, abdominal strength and endurance, flexibility, and body composition) were assessed with the Prudential Fitnessgram test battery (Cooper Institute, 1992). Also, children's dietary habits were assessed based on the Health Behavior Questionnaire (HBQ) of Parcel et al. (1995) that recorded their dietary habits, after being modified to fit in the Greek diet (Bebetsos 1999). The multivariate analysis of variance for repeated measures (2x2) indicated that the group X measure interaction was significant. Children in the experimental group had better scores on cardiorespiratory endurance, on abdominal strength and endurance, and on dietary habits than those in the control group. These findings indicate that certain health-related fitness components and dietary habits as well can be improved with a three hour a week program consisting physical fitness and nutrition education activities
SN - 00318981
UR -
https://login.proxy.bib.uottawa.ca/login?url=http://search.ebscohost.com/login.aspx?direct=true&db=s3h&AN=SPHS-938100&site=ehost-live;http://articles.sirc.ca/search.cfm?id=S-938100;http://www2.truman.edu/pek
L2 - http://articles.sirc.ca/search.cfm?id=S-938100
ER -

TY - JOUR
ID - 4334
T1 - Physical Activity and Depressive Symptoms in American Adolescents
A1 - Desha,Laura N.
A1 - Ziviani,Jenny M.
A1 - Nicholson,Jan M.
A1 - Martin,Graham
A1 - Darnell,Ross E.
Y1 - 2007/08//
KW - SportDiscus
KW - *EXERCISE -- Psychological aspects
KW - *PHYSICAL fitness -- Psychological aspects
KW - *MENTAL depression
KW - *MENTAL health
KW - *HEALTH
KW - logistic regression analysis
KW - HEALTH behavior in adolescence
KW - Research
KW - DEPRESSED persons
RP - NOT IN FILE
SP - 534
EP - 543
JF - Journal of Sport & Exercise Psychology
JA - J Sport Exerc Psychol
VL - 29

IS - 4
N2 - This study employed ordinal logistic regression analyses to investigate the relationship between American adolescents' participation in physical activity and depressive symptomatology. Data were drawn from the second Child Development Supplement to the Panel Study of Income Dynamics (CDS II), which was conducted over 2002-2003. Fewer than 60% of adolescents were found to accumulate 60 min of moderate-to-vigorous physical activity (MVPA) outside of school hours on week or weekend days. Accumulated duration of MVPA was not, however, significantly associated with severity of depressive symptoms for either gender. Males who were not involved in sporting clubs or lessons were more likely than males who were highly involved to experience greater severity of depressive symptoms (OR = 3.24, CI = 1.33, 7.87). Results highlight gender variability in the psychosocial correlates of sporting participation and prompt further investigation of the relevance of current physical activity guidelines for mental health in adolescence. ABSTRACT FROM AUTHOR
SN - 08952779
UR -
https://login.proxy.bib.uottawa.ca/login?url=http://search.ebscohost.com/login.aspx?direct=true&db=s3h&AN=25930433&site=ehost-live
ER -

TY - THES
ID - 4335
T1 - Assessment of sleep and biological factors that influence sleep in young children
A1 - DiLeo,H.A.
Y1 - 2004///
N1 - Accession Number: 2009048394. Language: English. Entry Date: 20051111. Revision Date: 20091218. Publication Type: doctoral dissertation; research. Special Interest: Pediatric Care. Instrumentation: Toddler and Preschool Sleep Problems Screening Tool (TPSPST)
KW - CINAHL
KW - instrument validation
KW - Sleep -- Evaluation -- In Infancy and Childhood
KW - Biophysical Instruments
KW - Child,Preschool
KW - convenience sample
KW - Factor Analysis
KW - Infant
KW - Kruskal-Wallis Test
KW - One-Way Analysis of Variance
KW - Polysomnography
KW - reliability
KW - Research Instruments
KW - Validation Studies
KW - validity
KW - Human
RP - NOT IN FILE
EP - 165
PB - University of Texas Health Science Center at San Antonio
N2 - Background and significance. Currently there are no simple-to-use tools available to identify sleep problems in the clinical setting. Polysomnography (PSG), the "gold standard" for sleep analysis, requires an overnight stay in a sleep lab. More recently, actigraphy has been used to estimate sleep-wake patterns from activity/inactivity in naturalistic settings. The reliability and validity of these devices have not been established for all age groups or clinical populations
Purpose. The overall purpose of this study was to develop a process by which practitioners could assess for factors that influence sleep in young children. Aim 1 evaluated the reliability and validity of the Actiwatch-64RTM (Mini-Mitter, Sunriver, OR) actigraph in young children by comparing it to attended PSG. Aims 2 and 3 assessed the reliability and validity of the researcher-developed Toddler and Preschool Sleep Problems Screening Tool (TPSPST) by comparing it to the Actiwatch-64RTM
Design. An instrumentation research design was used
Methods. Subjects aged 1--5 years (n = 40) wore the Actiwatch-64RTM during a standard attended PSG. Two subjects in each age range wore two devices each to assess interdevice reliability. Next, a convenience sample

(n = 200) of parents of children 12--60 months old from day care centers completed the TPSPST. Lastly, a convenience sample of children 12--60 months old (n = 40) completed the TPSPST and wore the Actiwatch-64RTM for 72 hours
Results. All the sleep parameters significantly correlated between the Actiwatch-64RTM and PSG as well as epoch-by-epoch sleep-wake scoring for the dual watch subjects. A one-way ANOVA showed no statistical difference between the groups for the TPSPST raw scores. A Kruskal-Wallis showed no statistical difference for categories between the groups. There was a trend toward higher scores on the night-waking subscale of the TPSPST and lower sleep efficiency, although it was not statistically significant. A factor analysis was done to identify the factors that describe the association between variables measured by the TPSPST and to assess if the tool measured the dimensions that were expected
SN - 978-0-542-01361-4
AV - UMI Order AAI3166183.
M1 - Ph.D.
UR - http://search.ebscohost.com/login.aspx?direct=true&db=cin20&AN=2009048394&site=ehost-live
ER -

TY - JOUR
ID - 4337
T1 - "If only" : Outdoor and adventurous activities and generalised academic development
A1 - Dismore,Harriet
A1 - Bailey,Richard
Y1 - 2005/01//
KW - Reviewer Nominated
RP - IN FILE
SP - 9
EP - 19
JF - Journal of Adventure Education & Outdoor Learning
VL - 5
IS - 1
N2 - This paper reports on research conducted as part of a project, the aim of which was to utilise outdoor and adventurous experiences to help schools address their 'if only'factor -- that aspect of learning perceived to be key to raising standards, whether it be the acquisition of knowledge, skills or a change in attitudes to learning. The project involved nine schools and 671 children, all engaged in the "I Can~" project and the follow-up activities that took place at schools. The research was carried out with children, teachers and parents and consisted of a series of focus groups and analysis of children's work. Limitations inherent within the research opportunity prevent the confident attribution of causation. However, the study's findings do offer insight into an under-researched, yet potentially valuable educational experience. The findings suggest that outdoor and adventurous activities can impact upon children's learning in school by addressing their intellectual, affective and social development.
SN - 1472967058520
UR - http://www.tandfonline.com/doi/abs/10.1080/14729670585200561
L1 - file:///J:/_Searches/Trembly/Outside/new artilces/Dismore-2005-If only_ Outdoor a.pdf
ER -

TY - JOUR
ID - 4338
T1 - Teguvot lahaz-dam lemaamaz ezel seportaim taharutiyim. / Blood pressure response to exercise in competitive athletes
A1 - Dlin,R.
A1 - Tenenbaum,G.
A1 - Hanne,N.
Y1 - 1985///
KW - SportDiscus
KW - *ATHLETES
KW - *BLOOD pressure
KW - *STRESS (Physiology)

KW - *HEART beat
KW - *CARDIOVASCULAR fitness
KW - *HEART -- Contraction
KW - *EXERCISE -- Physiological aspects
KW - adulthood
KW - teenagers
KW - MALES
KW - Comparative Studies
KW - Israel
KW - BICYCLE ERGOMETRY
KW - NON-ATHLETE
RP - NOT IN FILE
CY - Netanya, Israel;
PB - Wingate Institute
N2 - The purpose of this study was to define the normal systolic blood pressure (SBP) response to exercise in a large group of healthy competitive athletes training in various sports. 2111 competitive athletes in the age range 14-18 years participated. An additional 550 healthy active individuals not competing in any particular sport were included for comparison. Testing was carried out on a cycle ergometer. To provide valid comparison at equivalent physiological strain, SBP was presented in relation to heart rate at any given power load. Data was presented by age group and by sport discipline. All groups fell within the 25-75 per cent range for this population. The SBP response appeared to be higher than that of non trained individuals reported in the literature. Using this data, individuals falling outside the norm can be defined and appropriate measures taken
UR -
https://login.proxy.bib.uottawa.ca/login?url=http://search.ebscohost.com/login.aspx?direct=true&db=s3h&AN=SPH191747&site=ehost-live
ER -

TY - JOUR
ID - 4339
T1 - Are Victorian primary schools SunSmart? The ongoing development of the SunSmart Schools Program
A1 - Dobbinson,S.J.
A1 - Peipers,A.M.
A1 - Borland,R.
A1 - Nolan,K.M.
Y1 - 2000/04//
N1 - Accession Number: 2003054009. Language: English. Entry Date: 20030411. Revision Date: 20091218. Publication Type: journal article; research; tables/charts. Journal Subset: Australia & New Zealand; Blind Peer Reviewed; Editorial Board Reviewed; Health Promotion/Education; Peer Reviewed. Grant Information: VicHealth. No. of Refs: 22 ref. NLM UID: 9710936. Email: sdobbins@accv.org.au
KW - CINAHL
KW - School Policies -- Victoria
KW - Sunburn -- Prevention and Control -- In Infancy and Childhood
KW - Sunlight -- Prevention and Control -- In Infancy and Childhood
KW - chi square test
KW - Child
KW - Descriptive Statistics
KW - Mantel-Haenszel Test
KW - Questionnaires
KW - random sample
KW - Survey Research
KW - Victoria
KW - Funding Source
KW - Human
RP - NOT IN FILE
SP - 43
EP - 50
JF - Health Promotion Journal of Australia

JA - HEALTH PROMOT J AUST
VL - 10
IS - 1
N2 - ISSUE ADDRESSED:
Reducing children's exposure to ultraviolet (UV) radiation is a major focus for public health efforts to prevent skin cancer in Australia. Schools have a role to play in reducing children's exposure to UV radiation. In 1993 a SunSmart Schools Accreditation program was developed to encourage and support primary schools in Victoria to introduce sun protection policies. A SunSmart School sign provided recognition for schools that met accreditation standards as well as promoting the program to other schools
METHODS:
In 1997 the prevalence of sun protection policies and practices of a representative sample of Victorian primary schools was assessed. Principals from 259 schools reported on the sun protection policies and practices at their schools. At the time of this study 38% of schools had SunSmart accreditation
RESULTS AND CONCLUSIONS:
Results suggest that the majority of Victorian primary schools have addressed strategies such as hat wearing, sunscreen provision and curriculum-based sun protection education. However, shade provision and the scheduling of outdoor activities to avoid peak UV periods remain areas needing further improvement. SunSmart accreditation was more strongly associated with better sun protection in some strategy areas than policies that weren't accredited. Nevertheless non-accredited policies appear to have benefits over the lack of a sun protection policy. Moreover there was an association between sun protection practices in schools and whether these were specifically addressed in the policies
SO WHAT?
Providing support for schools to introduce policies and setting standards for sun protection are likely to improve sun protection practices in schools
SN - 1036-1073
AD - Centre for Behavioural Research in Cancer, Cancer Control Research Institute, Anti-Cancer Council of Victoria, 1 Rathdowne St, Carlton South 3053, Australia
UR - http://search.ebscohost.com/login.aspx?direct=true&db=cin20&AN=2003054009&site=ehost-live
ER -

TY - JOUR
ID - 4340
T1 - Advocacy Update: Returning to Nature
A1 - Dolesh,Richard J.
Y1 - 2007/07//
KW - SportDiscus
KW - *OUTDOOR recreation for children
KW - *OUTDOOR recreation
KW - *CHILDREN
KW - *CONDUCT of life
KW - GOVERNMENT policy
KW - MASS media & children
KW - NATIONAL Recreation & Park Association
KW - UNITED States.Congress
KW - United States
RP - NOT IN FILE
SP - 16
EP - 19
JA - Parks & Recreation
VL - 42
IS - 7
N2 - The article discusses the efforts of the National Recreation and Parks Association (NRPA) and other agencies to promote outdoor activities for children. The article discusses how U.S. children are spending less time outdoors and more time watching television, playing video games, or on the Internet. The U.S. Congress may hold hearings to address the disconnect between children and nature
SN - 00312215

UR -
https://login.proxy.bib.uottawa.ca/login?url=http://search.ebscohost.com/login.aspx?direct=true&db=s3h&AN=25788019&site=ehost-live
ER -

TY - JOUR
ID - 4342
T1 - The Impact of the Balanced School Day on Student Physical Activity and Nutrition
A1 - Dorman,Sandra
A1 - Gauthier,Alain
A1 - Thirkill,Laura
Y1 - 2013///Winter
KW - SportDiscus
KW - *SCHOOL children
KW - *FOOD
KW - *PHYSICAL activity
KW - *SCHOOL attendance
KW - SCHOOL day
KW - Ontario
RP - NOT IN FILE
SP - 6
EP - 10
JA - Physical & Health Education Journal
VL - 78
IS - 4
N2 - Over the past decade, school boards across Ontario have adopted the 'balanced school day'; wherein classroom time is divided into three blocks of 100 minutes separated by two 40 minute nutrition/activity breaks. The traditional schedule consists of a morning and afternoon recess of 15 minutes and one longer lunch break. Educators advocate that the balanced school day improves classroom learning; however, little evidence exists to support this. Given that classroom scheduling impacts time allocated for eating and play, scheduling is also an important component to children's overall health. To date, no systematic evaluation of the related health effects of scheduling on student nutrition and exercise has been done. The purpose of this article is to review the current literature in relation these questions and to reflect upon potential implications of this altered schedule.
ABSTRACT FROM AUTHOR
Au fil de la derniA"re dAccennie, les conseils scolaires de l'Ontario ont commencAc Aÿinstaurer le principe de la A®journAce d'Accole AcquilibrAce A¯. Il s'agit d'une journAce d'Accole oA1 les heures de classe sont rAcparties en trois blocs de 100 minutes avec deux pauses de nutrition/activitAc intercalAces de 40 minutes, alors que l'horaire traditionnel prAcvoit deux pAcriodes de rAccrAcation de I5 minutes, l'une en avant-midi et l'autre en aprA"s-midi, avec une pause plus longue pour le repas du midi. MalgrAc la pAcnurie de donnAces probantes Aÿcet effet, les enseignantes et enseignants sont d'avis que la journAce d'Accole AcquilibrAce favorise l'apprentissage des AclA"ves. Puisque l'horaire scolaire a des incidences sur l'alimentation et le jeu, l'organisation de l'horaire de l'Accole constitue une composante clAc de la santAc globale de l'enfant. Aucune Acvaluation systAcmatique n'a ActAc menAce jusqu'ici pour dActerminer les effets de l'horaire sur deux composantes de la santAc des AclA"ves, soit la nutrition et l'exercice. Cet article examine la documentation actuelle sur le sujet et rAcflAcchit aux rAcpercussions possibles de l'horaire modifiAc. ABSTRACT FROM AUTHOR
SN - 14980940
UR -
https://login.proxy.bib.uottawa.ca/login?url=http://search.ebscohost.com/login.aspx?direct=true&db=s3h&AN=92533393&site=ehost-live
ER -

TY - JOUR
ID - 4343
T1 - Urban ecology and urban ecosystems: understanding the links to human health and well-being
A1 - Douglas,Ian
Y1 - 2012///

KW - Reviewer Nominated
RP - NOT IN FILE
JA - Current Opinion in Environmental Sustainability
PB - Elsevier
SN - 1877-3435
ER -

TY - JOUR
ID - 4344
T1 - PROPS, PALS, RAP, TSM Active Playgrounds
A1 - Doyle,Pat
Y1 - 2007/03//
KW - SportDiscus
KW - *PLAYGROUNDS
KW - *INTRAMURAL sports
KW - PROGRAMS
KW - Child
RP - NOT IN FILE
SP - 4
EP - 5
JA - Input
CY - ;
N2 - The author provides the reader with tips for incorporating games into playground time. She recommends providing resources and support but let students teach other students. A list of successful prorgams is provided
SN - 14899396
UR -
https://login.proxy.bib.uottawa.ca/login?url=http://search.ebscohost.com/login.aspx?direct=true&db=s3h&AN=SPHS-1049282&site=ehost-live;http://articles.sirc.ca/search.cfm?id=S-1049282;http://www.mohawkc.on.ca/external/cira/home.html
L2 - http://articles.sirc.ca/search.cfm?id=S-1049282
ER -

TY - JOUR
ID - 4345
T1 - PIRMOK • ýFIZINIO AKTYVUMO PRIKLAUSOMYBŽ- NUO SOCIALINI • ý-EKONOMINI • ý VEIKSNI • ý. / PHYSICAL ACTIVITY OF FIRST GRADE PUPILS IN RELATION TO SOCIAL ECONOMIC FACTORS
A1 - Dregval,Liudmila
A1 - MalinauskaitŽ-,Vaida
Y1 - 2008/11//
KW - SportDiscus
KW - *EDUCATIONAL attainment
KW - *EDUCATION
KW - *SPORTS
KW - *PHYSICAL fitness
KW - *OUTDOOR games
KW - Socioeconomic Factors
KW - Parents
KW - DANCE parties
KW - parents' education
KW - Physical Activity
KW - social economic factors
KW - fizinis aktyvumas
KW - socialiniai-ekonominiai veiksniai
KW - tŽ-v • 3 i • -silavinimas
RP - NOT IN FILE
SP - 29

EP - 36
JA - Education.Physical Training.Sport
IS - 71
N2 - Physical activity of first grade pupils in relation to parents' social economic position is analysed in this article. The study was carried out in randomly selected Siauliai region schools in 2008. Questionnaires were distributed among 630 randomly selected first grade pupils and filled-in by 515 parents (response rate 81.8%). Physical activity of pupils was assessed according to parents' education, income and living place. It was established that sport or dancing clubs were attended by 39.6% of children. The highest number of pupils attending the clubs was among those whose parents (mother or farther) had the university education (52.8 and 59.5%, respectively). In families where parents had the vocational or the lower educational level this number was lower. More first grade pupils in cities attended sport or dancing clubs (45.5%) than in villages (35.4%, $p < 0.05$). More than half of children go to school (58.8%) and come from it (72.2%) on foot, 1.2% ride a bicycle. One third of children were brought to school by their parents in a car but only one fifth of them was brought back. The relation between the number of children brought to school by car and parents' income was observed: the highest number of children was in the families where parents' income was the highest (31.1% in families with low income and 46.3% with high income). One third of pupils (36.9%) spent their time playing out-of-doors for 3 or more hours on workdays and majority of them (87.1%) spent the same time at weekends. The study showed that first grade pupils spent much time passively playing computer games or watching TV. Mostly children used a computer for one hour per day (33.9% on workdays and 28.5% at weekends), mostly watched television for 2 hours on workdays and for 3 hours or more at weekends (45.1 and 41.4%, respectively). Conclusions: 1) Physical activity of first grade pupils is insufficient. The majority of them do not attend sport or dancing clubs, children spend much time passively watching TV or playing on a computer. 2) There are relations between social economic factors and children's physical activity. Most pupils attending sport or dancing clubs and playing on a computer were in families where parents had the highest educational level as well as in the families with the highest income. 3) First grade pupils from villages participate in sports and play computer games less than their peers in cities, they spend more time playing out-of-door. ABSTRACT FROM AUTHOR
SN - 13925644
UR -
https://login.proxy.bib.uottawa.ca/login?url=http://search.ebscohost.com/login.aspx?direct=true&db=s3h&AN=36124679&site=ehost-live
ER -

TY - JOUR
ID - 4347
T1 - THE IMPACT OF A SCHOOL-BASED ACTIVE VIDEO GAME PLAY INTERVENTION ON CHILDREN'S PHYSICAL ACTIVITY DURING RECESS
A1 - Duncan,Michael J.
A1 - Staples,Victoria
Y1 - 2010/06//
KW - SportDiscus
KW - *PHYSICAL fitness for children
KW - *VIDEO games & children
KW - *VIDEO games -- Physiological aspects
KW - *OUTDOOR recreation for children
KW - *PHYSICAL activity
KW - *PEDOMETERS
KW - *HEART rate monitoring
KW - Research
KW - exergaming
KW - heart rate monitoring
KW - pedometer
KW - recess
KW - steps
RP - NOT IN FILE
SP - 95
EP - 99

JA - Human Movement
VL - 11
IS - 1
N2 - Purpose. To assess physical activity levels during active video game play over time and compare this to 'free play' associated with recess activity in a sample of British primary school children over a 6-week period. Basic procedures. Thirty children (ages 10-11, 12 boys, 18 girls) from central England were randomly selected to participate in a 6 week, recess based, active video gaming intervention (n = 15) or act as controls (n = 15). Repeated measures analysis of covariance (controlling for body fatness) was used to examine any differences in physical activity, determined by pedometry and heart rate monitoring over time and between intervention and control groups. Main Findings. Children in the intervention accumulated significantly greater steps/day than the control group during the first week of the intervention. This pattern was reversed at the mid and end points of the intervention (p = .03). Irrespective of time point, children engaging in active video game play spent a lesser percentage of time engaged in MVPA than the controls undertaking 'traditional' recess activity (p = .0001). Conclusions. Active video game play does not appear to be a sustainable means to enhance children's physical activity. Although physical activity (steps/min) was greater on initial presentation of active video games compared to 'traditional' recess activity, this appears to be an acute effect. ABSTRACT FROM AUTHOR
SN - 17323991
UR -
https://login.proxy.bib.uottawa.ca/login?url=http://search.ebscohost.com/login.aspx?direct=true&db=s3h&AN=51924656&site=ehost-live
ER -

TY - JOUR
ID - 4348
T1 - PHYSICAL ACTIVITY LEVELS DURING A 6-WEEK, SCHOOL-BASED, ACTIVE VIDEOGAMING INTERVENTION USING THE GAMERCIZE POWER STEPPER IN BRITISH CHILDREN
A1 - Duncan,Michael J.
A1 - Birch,Samantha
A1 - Woodfield,Lorayne
A1 - Hankey,Joanne
Y1 - 2011/07//
KW - SportDiscus
KW - *PHYSICAL fitness
KW - *VIDEO games -- Physiological aspects
KW - *SCHOOL children
KW - *HEALTH
KW - *HEART rate monitoring
KW - Great Britain
KW - exergaming
KW - heart rate monitoring
KW - pedometer
KW - recess
KW - steps
RP - NOT IN FILE
SP - 81
EP - 87
JF - Medicina Sportiva
VL - 15
IS - 2
N2 - The article discusses a study which examined the levels of physical activity during active videogame play and compared it to free play in a sample of primary school children in Great Britain. The differences in physical activity were determined using pedometry and heart rate monitoring. Findings revealed that greater steps per minute were accumulated by children in the intervention group during the first week of research. In addition, a lesser percentage of time was spent by children engaging in active video game play
SN - 14290022

UR -
https://login.proxy.bib.uottawa.ca/login?url=http://search.ebscohost.com/login.aspx?direct=true&db=s3h&AN=62642102&site=ehost-live
ER -

TY - JOUR
ID - 4351
T1 - PLAYGROUND INSPECTION--IT'S YOUR CHOICE
A1 - Eager,David
Y1 - 2007/03//
KW - SportDiscus
KW - *PLAYGROUNDS
KW - *SPORTS facilities
KW - *GROUNDS maintenance
KW - INSPECTION & review
KW - SAFETY measures
KW - PLAY environments
RP - NOT IN FILE
SP - 5
EP - 6
JA - Australasian Parks & Leisure
VL - 10
IS - 1
N2 - The article deals with the levels of inspection recommended in the Australian Standards for play spaces and playground equipment. These standards offer specific requirements for playground undersurfacing and can be utilized to risk assess the safety of children's play spaces and environment. The levels of inspection include Routine Inspection, Operational Inspection and Annual Inspection. In many cases experienced council grounds maintenance staff can conduct routine inspections
SN - 14465604
UR -
https://login.proxy.bib.uottawa.ca/login?url=http://search.ebscohost.com/login.aspx?direct=true&db=s3h&AN=25122024&site=ehost-live
ER -

TY - JOUR
ID - 4353
T1 - The Active Living Research 2013 Conference: Achieving Change Across Sectors: Integrating Research, Policy, and Practice
A1 - Economos,Christina D.
A1 - Sallis,James F.
A1 - Keith,NiCole R.
A1 - Newkirk,Jimmy
Y1 - 2014/01/02/
KW - SportDiscus
KW - *PHYSICAL activity
KW - *OBESITY in children
KW - *PREVENTION
KW - MEDICAL policy -- Congresses
KW - MEDICAL research -- Methodology
RP - NOT IN FILE
SP - S1
EP - S4
JF - American Journal of Health Promotion
JA - Am J Health Promot
N2 - The authors describe the Active Living Research 2013 Conference: Achieving Change Across Sectors: Integrating Research, Policy, and Practice event that was held in San Diego, California from February 26 to 28,

2013. Topics include the Instant Recess routine of Dr. Toni Yancey, the collection of evidence to inform practice, initiatives to prevent childhood obesity, and musical chairs
SN - 08901171
UR -
https://login.proxy.bib.uottawa.ca/login?url=http://search.ebscohost.com/login.aspx?direct=true&db=s3h&AN=93573183&site=ehost-live
ER -

TY - JOUR
ID - 4354
T1 - Sharing spaces: school playground design and children's well-being
A1 - Edwards,K.
Y1 - 2006/03//
N1 - Accession Number: 2009182987. Language: English. Entry Date: 20060609. Revision Date: 20091218. Publication Type: journal article; research; tables/charts. Journal Subset: Biomedical; Europe; Health Promotion/Education; Online/Print; Peer Reviewed; UK & Ireland. Special Interest: Psychiatry/Psychology. No. of Refs: 11 ref. NLM UID: 101252769. Email: Kate.edwards@northstaffs.nhs.uk
KW - CINAHL
KW - Equipment Design
KW - Planning Techniques
KW - Play and Playthings
KW - Schools,Elementary -- Administration
KW - Child
KW - Environment
KW - Program Development
KW - Public Spaces
KW - Questionnaires
KW - Human
RP - NOT IN FILE
SP - 18
EP - 23
JA - Journal of Public Mental Health
VL - 5
IS - 1
N2 - The shared space of school grounds can provide a rich educational resource in addition to a safe environment for play, enjoyment and relaxation. The project described in this paper developed guidance for schools on grounds development and also provided direct support to three schools to enable them to improve their grounds and to work towards Eco Schools and Health Promoting Schools accreditation. The evaluation demonstrated a relationship between improved grounds and a reduction in negative playground incidents, improved co-operation at break times, children having more pride in their school, children feeling happier at break time, increased sun awareness and the use of school grounds as a teaching resource. The project also achieved some influence at a more strategic level
SN - 1746-5729
AD - Programme Leader, Mental Health, Directorate of Health Promotion, Herbert Minton Building, 79 London Road, Stoke-on-Trent ST4 7PZ
UR - http://search.ebscohost.com/login.aspx?direct=true&db=cin20&AN=2009182987&site=ehost-live
ER -

TY - JOUR
ID - 4357
T1 - Variability in the measurement of uptime in children: a preliminary study
A1 - Eldridge,Beverly
A1 - Mccoy,A.
A1 - Galea,M.
A1 - Wolfe,R.
A1 - Graham,H.K.
Y1 - 2003/08//

KW - SportDiscus
KW - *CHILDREN -- Health
KW - BIPEDALISM
KW - MELBOURNE (Vic.)
KW - Victoria
RP - NOT IN FILE
SP - 499
JF - Clinical Rehabilitation
JA - Clin Rehabil
VL - 17
IS - 5
N2 - 'Uptime' can be defined as time spent in the upright position. The opposite of uptime is 'downtime', which is a measure of time spent in the recumbent position. The concept and usage of these terms have been common parlance in research into chronic pain for over 20 years. Measures of uptime have been used to evaluate changes in a person's functional performance. Uptime provides new information about a patient's response to rehabilitation that complements other outcome measures currently in use. In patients with chronic pain it has been shown that as uptime increases in symptoms of pain and an improvement in functional capacity. However, no research to date has reported the measurement of uptime in children. This could be a useful outcome measure when the focus of rehabilitation was on enhancing a child's ability to stand, walk or move around in the upright position. An accurate way to measure uptime is by direct observation. However, this is not practical for long periods of time, or large numbers of subjects. 'Uptimers' are battery-operated devices that record uptime by the activation of a mercury tilt switch. These devices are attached to the outside the thigh, and when they are seated or reclining the device records downtime. The reliability and validity of uptimers in adults have been demonstrated by comparing uptime as recorded by an uptimer with recorded by an independent observer. ABSTRACT FROM AUTHOR
SN - 02692155
UR - https://login.proxy.bib.uottawa.ca/login?url=http://search.ebscohost.com/login.aspx?direct=true&db=s3h&AN=10331555&site=ehost-live
ER -

TY - JOUR
ID - 4358
T1 - A COMPARATIVE STUDY REGARDING THE IMPACT OF PHYSICAL EXERCISES OVER THE SOMATIC INDICATORS FOR CHILDREN LIVING IN URBAN AND RURAL AREAS (10-11 YEARS)
A1 - Elena,L.U.P.U.
Y1 - 2012/09/02/
KW - SportDiscus
KW - *CHILDREN
KW - *GROWTH
KW - *SCHOOL children
KW - *HEALTH
KW - *PHYSICAL fitness for children
KW - *PHYSICAL education
KW - *SEDENTARY lifestyles
KW - STUDY & teaching
KW - Motion
KW - Physical Education
KW - pupils
KW - rural
KW - Sedentary Lifestyle
KW - somatic indices
KW - Urban
RP - NOT IN FILE
SP - 422
EP - 426
JA - Ovidius University Annals, Series Physical Education & Sport/Science, Movement & Health

VL - 12
IS - 2
N2 - Problem Statement: Once a child starts its school experience, the learning process is triggered, as it is considered that it has to develop on the basis of a healthy organism, due to the fact that the intellectual effort requires a greater physical resistance. Furthermore, knowing children's anatomic and physiologic particularities is a necessary condition for a good proceeding of the Physical Education lesson in school, because it enables the members of the didactic staff to understand the importance of physical education for primary school pupils, and the necessity of these lessons to be realised in an organised and systematic manner. Somatic parameters are a barometer for the didactic staff, and a comparative presentation of these parameters could represent a starting signal for the didactic process. Purpose of Study: This research started from the hypothesis according to which there are significant differences in the somatic development of children in urban areas, as compared to those living in rural areas, and we believe that these differences occurred due to the lack of movement. The present research was undertaken on a group of 20 subjects: 10 subjects living and learning in urban areas, and other 10 in rural areas. Research Methods: For our research we used the bibliographic study method, observation method, enquiry method (discussion, conversation, Lexical association - La, etc.), pedagogical experiment method, statistical-mathematical method, and graphical method. Findings: The purpose of this research was to observe if significant differences occur in the somatic development for children from urban areas, which have only two Physical Education lessons are more sedentary, as compared with children from rural areas, who, in addition to the two lessons of Physical Education they have in the weekly syllabus, spend about 2-3 hours a day outdoors playing ball, running or playing several specific countryside childhood games (catch, hide and seek, etc). Conclusions: Therefore, Physical Education lesson combined with leisure activities has a positive effect on children's growth and development, an aspect which should be taken into consideration, and we should offer more outdoor time for children living and studying in urban areas. ABSTRACT FROM AUTHOR
SN - 12247359
UR -
https://login.proxy.bib.uottawa.ca/login?url=http://search.ebscohost.com/login.aspx?direct=true&db=s3h&AN=85380166&site=ehost-live
ER -

TY - JOUR
ID - 4359
T1 - The Values of the Traditional Camp Experience
A1 - Elkind,David
Y1 - 2007/01//
KW - SportDiscus
KW - *CHILD development
KW - *CAMPS
KW - *CHILDREN
KW - *CONDUCT of life
KW - *PLAY
KW - ELKIND,David,1930-
KW - PSYCHOLOGICAL aspects
RP - NOT IN FILE
SP - 1
EP - 4
JA - Camping Magazine
VL - 80
IS - 1
N2 - The article offers opinion on the importance of play and outdoor activity for cognitive and emotional development in children from psychologist David Elkind. A discussion of the lessons that children can learn and the insights they can gain from attending summer camps is presented. A decrease which has been seen in the amount of time U.S. children spend playing outdoors is discussed
SN - 07404131
UR -
https://login.proxy.bib.uottawa.ca/login?url=http://search.ebscohost.com/login.aspx?direct=true&db=s3h&AN=24105147&site=ehost-live
ER -

TY - JOUR
ID - 4360
T1 - Recess Physical Activity Packs in Elementary Schools: A Qualitative Investigation
A1 - Elliott,Steven
A1 - Combs,Sue
A1 - Boyce,Robert
Y1 - 2011///Fall
KW - SportDiscus
KW - *PHYSICAL education for children
KW - *PHYSICAL education
KW - *CHILDREN
KW - *PHYSICAL activity
KW - *HEALTH behavior
KW - *SCHOOL recess breaks
KW - *OUTDOOR recreation for children
KW - Research
RP - NOT IN FILE
SP - 150
EP - 162
JA - Physical Educator
VL - 68
IS - 3
N2 - To supplement the present weekly allotment of 30 minutes of physical education, a school district in southeastern North Carolina identified recess time as part of the state mandated (HSP-S-000) 150 minutes of physical activity (PA) per week and have purchased fitness equipment (recess packs) for the children to use. Twelve participants were selected from four elementary schools that had recently received the recess packs equipment and training. The participants were; (a) four principals, (b) four teachers, and (c) four students. They were selected by a purposeful sampling technique. Qualitative data analysis procedures were used to explore participants 'views on how the recess packs had impacted PA levels during recess. Several themes emerged during the interviews including: (a) gender differences in the types of activities performed during recess before and after the introduction of the recess packs, (b) how the type and the developmentally appropriateness of activities had been affected by the recess packs, (c) how the recess packs initiative had changed the teacher 's role during recess, (d) the importance of addressing nutritional concerns in addition to encouraging more physical activity during recess, and (e) the training that principals and teachers had received. Overall, the study demonstrates the PA benefits in providing equipment during school recess periods. Also, it stresses the importance of training principals and teachers to effectively plan and deliver developmentally appropriate activities resulting in more intense bouts of PA. ABSTRACT FROM AUTHOR
SN - 00318981
UR -
https://login.proxy.bib.uottawa.ca/login?url=http://search.ebscohost.com/login.aspx?direct=true&db=s3h&AN=67018187&site=ehost-live
ER -

TY - JOUR
ID - 4361
T1 - PLANINARSKI IZLETI KAO VANNASTAVNA AKTIVNOST U OSNOVNIM •ŠKOLAMA. / MOUNTAINEERING TRIPS AS AN OUTDOOR ACTIVITY IN PRIMARY SCHOOLS
A1 - Elvir,KazazoviŽØ
A1 - Elvira,BeganoviŽØ
A1 - ŽOaplar,Alan
Y1 - 2010/06//
KW - SportDiscus
KW - *MOUNTAINEERING
KW - *STUDENTS -- Recreation
KW - *OUTDOOR recreation for children
KW - *HIKING for children

KW - *PHYSICAL education for children
KW - and hiking trips for children
KW - organization
KW - preparation
KW - izleti i planinarenje djece
KW - organizacija
KW - priprema
RP - NOT IN FILE
SP - 33
EP - 36
JA - Sportekspert
VL - 3
IS - 1
N2 - Mountaineering trips represent a widespread form of outdoor activities. The main goal of mountaineering trips is to educate and teach students how to accept mountaineering as a way of living. In this paper we presented a way of planning and organizing trips that can help teachers in an efficient way while they implement and realize the extracurricular activities within the school subject of Physical and Health Training. We presented a preparation for a trip (the equipment for a daily hiking and dayslong hiking), days-long trips and staying overnight in the mountains, including exercise and stay in the mountain. Mountaineering trips are interdisciplinary activities that are realized in different circumstances. It is very important to respect the sequence of planned activities in the process of organizing the trip, especially when we work with children who possess a lower level of anthropological potential. ABSTRACT FROM AUTHOR
SN - 18403638
UR -
https://login.proxy.bib.uottawa.ca/login?url=http://search.ebscohost.com/login.aspx?direct=true&db=s3h&AN=55435449&site=ehost-live
ER -

TY - JOUR
ID - 4362
T1 - AHL teams continue to give back
A1 - Eminian,D.A.V.E.
Y1 - 2009/01/26/
KW - SportDiscus
KW - *HOCKEY teams
KW - *HOCKEY players
KW - Community Services
KW - AMERICAN Hockey League
RP - NOT IN FILE
SP - 38
EP - 38
JA - Hockey News
VL - 62
IS - 15
N2 - The article offers information on the community service of American Hockey League (AHL) players. Rookie defenseman Mike Moore, for one, visited sick children in a hospital. He even shoveled gravel into potholes and shopped for sketch pads and crayons at Toys R Us. All these were done by Moore on behalf of his team, the Worcester Sharks. Meanwhile, the Grand Rapids Griffins hosted a 24-hour marathon skate where coaches and players join the public on an outdoor rink as part of a Winterfest celebration
SN - 00183016
UR -
https://login.proxy.bib.uottawa.ca/login?url=http://search.ebscohost.com/login.aspx?direct=true&db=s3h&AN=36382118&site=ehost-live
ER -

TY - JOUR
ID - 4363

T1 - OUTDOOR ACTIVITY IN WINTER SPORT - ALPINE SKIING
A1 - Ene-Voiculescu,Carmen
A1 - Ene-Voiculescu,Virgil
Y1 - 2009/09//
KW - SportDiscus
KW - *WINTER sports
KW - *DOWNHILL skiing
KW - *TEENAGERS
KW - *OUTDOOR life
KW - *LEISURE
KW - *GENDER
KW - LONGITUDINAL method
KW - CORRELATION (Statistics)
KW - Analysis of Variance
KW - adolescence
KW - alpine-skiing
KW - outdoor activity
RP - NOT IN FILE
SP - 109
EP - 111
JA - Ovidius University Annals, Series Physical Education & Sport/Science, Movement & Health
VL - 9
IS - 2
N2 - The aim of this study was to examine whether early and sustained organized youth sport during childhood and adolescence predicts frequency of leisure-time alpine-skiing at age 27. A 5-year longitudinal study of 180 adolescents was conducted to practice alpine-skiing after faculty. The correlation coefficients tended to be higher among males and decreased over time both in females and males. The age at becoming a member in organized sport and duration of participant in organized outdoor activity in winter during adolescence predicted 9 % of the variance of young adult alpine-skiing. The correlations were higher in males than in females, but these differences were not significant. Organized youth sports during childhood and adolescence was positively related to frequency of leisure-time alpine-skiing in young adulthood. ABSTRACT FROM AUTHOR
SN - 12247359
UR -
https://login.proxy.bib.uottawa.ca/login?url=http://search.ebscohost.com/login.aspx?direct=true&db=s3h&AN=53995229&site=ehost-live
ER -

TY - JOUR
ID - 4364
T1 - Cultural and gender effects on Israeli children's preferences for activities
A1 - Engel-Yeger,Batya
A1 - Jarus,Tal
Y1 - 2008/06//
KW - SportDiscus
KW - *CHILDREN
KW - *GENDER
KW - *OCCUPATIONAL therapists
KW - Culture
KW - SOCIAL skills in children
KW - OCCUPATIONAL therapy for children
KW - Israel
KW - CHILDREN
KW - Participation
KW - Enfants
RP - NOT IN FILE
SP - 139
EP - 148

JA - Canadian Journal of Occupational Therapy
VL - 75
IS - 3
N2 - Background. Knowledge about factors that affect participation, as preference of activities, has major intervention implications. Purpose. To evaluate culture and gender effects on Israeli Jewish and Druze children's preference of activities performed outside mandated schooL This study used the "Preference Assessment of Children" (PAC) (King et al., 2004). Findings. On most scales, the Jewish children showed significantly less interest in activities than the Druze children. Among the Jews, girls showed higher preference in most PAC scales than boys while among the Druze girls showed higher preference than the boys only in social skills. Implications. Culture and gender may influence children's preference of activity. More studies should elaborate the knowledge about individuals' preferred activities; understanding the factors that affect these preferences may enhance occupational therapy evaluation and intervention processes. Canadian occupational therapists, as health care professionals in a multicultural society, must develop cultural competency and explore people's experience as cultural beings. ABSTRACT FROM AUTHOR
Description. Les connaissances sur les facteurs qui influent sur la participation, comme les prAcfAcrences en matiA"re d'activitAcs, ont des consAcquences majeures sur l'intervention. But. A%valuer les effets de la culture et du sexe sur les prAcfAcrences d'enfants juifs et druzes israAcliens face Aÿdes activitAcs rAcalisAces Aÿ l'extAcrieur d'une Accole mandatAce. Cette Actude a ActAc effectuAce Aÿl'aide de la A® Preference Assessment of Children A¯(PAC) (King et al., 2004). RAcsultats. A la plupart des Acchelles, les enfants juifs ont manifestAc moins d'intAcrA ¦t pour les activitAcs que les enfants druzes. Parmi les enfants juifs, les filles ont montrAc une plus grande prAcfAcrence que les garA ons dans la plupart des Acchelles de la PAC, alors que chez les enfants druzes, les filles ont montrAc une plus grande prAcfAcrence que tes garA ons seulement pour les habiletAcs sociales. ConsAcquences. La culture et le sexe peuvent influencer les prAcfAcrences des enfants face AÿdiffAcrentes activitAcs. La plupart des Actudes devraient chercher Aÿproduire des connaissances sur les activitAcs prAcfAcrAces par les enfants; une meilleure comprAchension des facteurs qui influent sur ces prAcfAcrences pourrait permettre d'amAcliorer les processus d'Acvaluation et d'intervention en ergothAcrapie. Les ergothAcrapeutes canadiens, de mA ¦me que les professionnels de la santAc Acvoluant dans une sociActAc multiculturelle doivent acquAcrir des compActences culturelles et examiner l'expAcrience des gens en tant qu'A ¦tres culturels. ABSTRACT FROM AUTHOR
SN - 00084174
UR -
https://login.proxy.bib.uottawa.ca/login?url=http://search.ebscohost.com/login.aspx?direct=true&db=s3h&AN=43100015&site=ehost-live
ER -

TY - JOUR
ID - 4365
T1 - Measuring Physical Activity of Fifth-Grade Students Using Pedometers. (Abstract)
A1 - Engstrom,Lorri Francis
A1 - Francis,Richard W.
Y1 - 2007/02//
KW - SportDiscus
KW - *PHYSICAL fitness
KW - *CARDIOVASCULAR fitness
KW - *STRESS (Physiology)
KW - *PHYSICAL education & training
KW - *RECESSES
KW - Pedometers
KW - Elementary Schools
KW - Students
KW - Child
KW - SEX FACTOR
KW - Step
RP - NOT IN FILE
SP - A
EP - a
JF - Research Quarterly for Exercise & Sport

JA - Res Q Exerc Sport
VL - 78
IS - 1
CY - ;
SN - 02701367
UR -
https://login.proxy.bib.uottawa.ca/login?url=http://search.ebscohost.com/login.aspx?direct=true&db=s3h&AN=SPHS-1048680&site=ehost-live;http://articles.sirc.ca/search.cfm?id=S-1048680;http://www.aahperd.org/
L2 - http://articles.sirc.ca/search.cfm?id=S-1048680
ER -

TY - JOUR
ID - 4366
T1 - Metodika uŽ_enja ko • -arkarskih iger na en ko • -. / Methodology for learning basketball games with one basket
A1 - ErŽ_ulj,Frane
Y1 - 2013/12//
KW - SportDiscus
KW - *BASKETBALL -- Competitions
KW - *BASKETBALL instruction
KW - *PHYSICAL education
KW - *PHYSICAL training & conditioning
KW - *BASKETBALL -- Rules
KW - Research
KW - Learning
KW - Basketball
KW - Physical Education
KW - Training
KW - • -portna vzgoja
KW - ko • -arka
KW - trening
KW - uŽ_enje
RP - NOT IN FILE
SP - 13
EP - 20
JA - Sport: Revija Za Teoreticna in Prakticna Vprasanja Sporta
VL - 61
IS - 3/4
N2 - Games with one basket are basic basketball games where players learn about the essential elements of the basketball game (5:5 with two baskets) as well as systematically build up and broaden their knowledge. These games are simpler because a smaller number of players is involved; however, they are suitable for strengthening and maintaining many motor and functional abilities and they also impact significantly on the development of man's psycho-social dimensions. One can say that games with one basket positively affect the development of one's personality and also have an important educational function. Owing to the above and the fact that many times in sports halls and also on outdoor basketball courts it is not possible to play basketball with two baskets (smaller sports halls, only one-half of the court can be used, too many children), games with one basket also represent important contents of physical education classes. The article presents the methodology for learning basketball games with one basket, some other games with adjusted rules as well as types of competitions in games with one basket. ABSTRACT FROM AUTHOR
SN - 03537455
UR -
https://login.proxy.bib.uottawa.ca/login?url=http://search.ebscohost.com/login.aspx?direct=true&db=s3h&AN=93342666&site=ehost-live
ER -

TY - JOUR
ID - 4367

T1 - An Injury and Illness Surveillance Program for Children and Staff: Improving the Safety of Youth Settings
A1 - Erceg,Linda E.
A1 - Garst,Barry A.
A1 - Powell,Gwynn M.
A1 - Yard,Ellen E.
Y1 - 2009///Winter
KW - SportDiscus
KW - *RECREATION
KW - *CHILDREN -- Wounds & injuries
KW - *DISEASE management
KW - *YOUTH
KW - *WOUNDS & injuries
KW - *OUTDOOR recreation
KW - *CAMPING
KW - Research
KW - United States
KW - camp
KW - illness
KW - injury
KW - Risk Management
KW - surveillance
KW - youth programs
RP - NOT IN FILE
SP - 121
EP - 132
JA - Journal of Park & Recreation Administration
VL - 27
IS - 4
N2 - The prevention of injuries to children and staff is of critical importance to recreation providers. Although program providers often document participant illnesses and injuries, the power of intentionally monitoring and sharing this information has not often been tapped in the recreation field. This article demonstrates how an injury and illness surveillance program was used to reveal patterns of injuries and illnesses within day and resident camps. These patterns suggested that children are less likely to be injured in day and resident camp settings when compared with participation in community-based sports, and, additionally, illustrated an organizing framework for injury and illness prevention that may to useful to other youth program providers. Primary injury topics appeared to be supervision patterns, falling on uneven terrain, and head injuries. Preventative strategies suggest analysis of levels of supervision, footwear policies, and activity-specific head protection. Two significant clusters of illness were identified. One included throat, nose, lung, ear, and eye maladies, while the second was comprised of upper and lower gastrointestinal illnesses, those often associated with what one eats or is exposed to (e.g., Norwalk virus). Proactive approaches to illness management center on maintaining resilience so people are less susceptible to pathogens and implementing practices that minimize the potential that someone will get ill. The innovation of sharing of data in a systematic way proved helpful in understanding illness and injury. The systematic investigation of when, where, and how injuries and illnesses occurred brought to light trends that yielded specific, practical strategies that administrators can implement to improve safety for staff and participants. This new approach to understanding what is happening in youth programs allows for targeted intervention based on real data rather than perceptions of trends. As program administrators work to improve health and safety, understanding the trends related to illness, injury, and proactive management can make a difference in safety improvements. ABSTRACT FROM PUBLISHER
SN - 07351968
UR -
https://login.proxy.bib.uottawa.ca/login?url=http://search.ebscohost.com/login.aspx?direct=true&db=s3h&AN=45463299&site=ehost-live
ER -

TY - JOUR
ID - 4369
T1 - Examination of physical activity during a physical activity intervention and recess. (Abstract)

A1 - Ernst,M.
Y1 - 2003/03//
KW - SportDiscus
KW - *PHYSICAL fitness
KW - *CARDIOVASCULAR fitness
KW - *STRESS (Physiology)
KW - *OBESITY
KW - *PREVENTION
KW - *RECESSES
KW - PROGRAMS
KW - Schools
KW - Elementary Schools
KW - FIELD experiments
KW - Child
KW - EFFECTIVENESS
RP - NOT IN FILE
SP - A
EP - 43
JF - Research Quarterly for Exercise & Sport
JA - Res Q Exerc Sport
VL - 74
IS - 1 Suppl
CY - ;
SN - 02701367
UR -
https://login.proxy.bib.uottawa.ca/login?url=http://search.ebscohost.com/login.aspx?direct=true&db=s3h&AN=SPHS-883362&site=ehost-live;http://articles.sirc.ca/search.cfm?id=S-883362;http://www.aahperd.org/
L2 - http://articles.sirc.ca/search.cfm?id=S-883362
ER -

TY - JOUR
ID - 4374
T1 - PLAY AREA AND PHYSICAL ACTIVITY IN RECESS IN PRIMARY SCHOOLS. / IGRALI •ÿTE I TJELESNA AKTIVNOST ZA VRIJEME •ÿKOLSKOG ODMORA U OSNOVNIM • ÿKOLAMA
A1 - Escalante,Yolanda
A1 - Backx,Karianne
A1 - Saavedra,Jose M.
A1 - GarcA-a-Hermoso,Antonio
A1 - DomA-nguez,Ana M.
Y1 - 2012/12//
KW - SportDiscus
KW - *PHYSICAL activity -- Measurement
KW - *EXERCISE for children
KW - *CHILDREN -- Health
KW - *PHYSICAL fitness for children
KW - *SEDENTARY lifestyles
KW - *YOUTH -- Recreation
KW - Research
KW - HEALTH behavior in children
KW - childhood
KW - Exercise
KW - Health
KW - Sedentary Lifestyle
KW - djetinjstvo
KW - sedentarni stil • _ivota
KW - tjelovje • _ba
KW - zdravlje

RP - NOT IN FILE
SP - 123
EP - 129
JF - Kinesiology
VL - 44
IS - 2
N2 - The aim of the present study was to describe the daily physical activity (PA) during recess of primary-school children and its relationship with the play area and their age. 738 children (8.5A ñ1.7 years, range six to eleven years) participated in the study. The playground recess PA of each child was measured using accelerometry. An ANOVA was used to determine the differences in PA by play area (large >15 mA ý/child and small area <8 mA ý/child) in each age group. In general, the children in larger play areas were more active than the children in small play areas (effect size = .36). This difference was larger in nine-year (effect size = .81), ten-year (effect size = .60) and eleven-year old children (effect size = .55). It seems necessary to carry out strategies that provide a greater opportunity for PA in small playgrounds with a high density of children. ABSTRACT FROM AUTHOR
SN - 13311441
UR -
https://login.proxy.bib.uottawa.ca/login?url=http://search.ebscohost.com/login.aspx?direct=true&db=s3h&AN=85708707&site=ehost-live
ER -

TY - JOUR
ID - 4376
T1 - PROMOTING PLAY
A1 - Espinoza,Mariana
Y1 - 2005/10//
KW - SportDiscus
KW - *SPECIAL events
KW - *RECREATION
KW - *PLAYGROUNDS
KW - *RECREATION centers
KW - *WALKING (Sports)
KW - Texas
RP - NOT IN FILE
SP - 72
EP - 75
JA - Parks & Recreation
VL - 40
IS - 10
N2 - This article presents information on the Play Day, a widespread fitness event organized by the Austin Parks and Recreation Department (APARD) where more than 700 children joined the mayor of Austin in a one-mile walk along Austin's hike and bike trail. This special event took place on June 8, 2005 in Texas. Campers from 30 playground sites and 16 recreation centers participated in this opening ceremony as part of the 2005 Play Day Initiative, created by the APARD and supported by two local programs--Walk Texas and Steps to a Healthier Austin
SN - 00312215
UR -
https://login.proxy.bib.uottawa.ca/login?url=http://search.ebscohost.com/login.aspx?direct=true&db=s3h&AN=18581659&site=ehost-live
ER -

TY - JOUR
ID - 4378
T1 - Operationalising the principles of playground design: a case study
A1 - Evans,J.
A1 - Payne,P.
Y1 - 1983///

KW - SportDiscus
KW - *PLAYGROUNDS
KW - Elementary Schools
KW - Surveys
KW - age
KW - planning
KW - equipment
KW - USE STUDY
KW - Child
KW - TIME FACTOR
KW - SEX FACTOR
RP - NOT IN FILE
CY - ;
N2 - The purpose of this study was to evaluate the existing use and condition of an elementary school playground. Children were asked to give suggestions on how to improve the school playground equipment. Other agencies were surveyed in order to gather information on structure, design, cost, location and maintenance of the playground
UR -
https://login.proxy.bib.uottawa.ca/login?url=http://search.ebscohost.com/login.aspx?direct=true&db=s3h&AN=SPH132637&site=ehost-live;http://articles.sirc.ca/search.cfm?id=132637
L2 - http://articles.sirc.ca/search.cfm?id=132637
ER -

TY - JOUR
ID - 4379
T1 - The teacher role in playground supervision
A1 - Evans,J.
Y1 - 1990/08//
KW - SportDiscus
KW - *PLAYGROUNDS
KW - *RECESSES
KW - CROSS-cultural studies
KW - Roles
KW - Teachers
KW - SUPERVISION
KW - Schools
RP - NOT IN FILE
SP - 219
EP - 234
JF - Play & Culture
VL - 3
IS - 3
CY - ;
N2 - Of the many factors that come to influence elementary school children's play, the role of the supervising adult has been infrequently studied. This paper commences with some reflections from an earlier study of children's play and the role taken by the teachers on duty in a midwestern American school. It then describes the findings of a broader study, this time located in Australian primary schools, which had as its focus an investigation of the attitudes and role of teachers on playground supervision or 'yard duty' as it is called in Australia. The study found that the teachers saw yard duty in much the same way as their American counterparts. That is, as a necessary but genereally unwelcome task. They saw themselves as fulfilling multiple roles including that of police officer, nurse, lawyer, psychologist, and social worker. The number and nature of the interactions between teachers and children varied with age and gender. This study concludes that the teacher role is an important one, and that there is a sound argument for not only more research in the area, but more attention to the education of teachers for their role as supervisor
SN - 08944253

UR -
https://login.proxy.bib.uottawa.ca/login?url=http://search.ebscohost.com/login.aspx?direct=true&db=s3h&AN=SPH282865&site=ehost-live;http://articles.sirc.ca/search.cfm?id=282865
L2 - http://articles.sirc.ca/search.cfm?id=282865
ER -

TY - JOUR
ID - 4380
T1 - Surplus energy theory: an enduring but inadequate justification for school breaktime
A1 - Evans,J.
A1 - Pellegrini,A.
Y1 - 1997/11//
KW - SportDiscus
KW - *RECESSES
KW - *PLAY
KW - *PLAYGROUNDS
KW - Schools
KW - Child
RP - NOT IN FILE
SP - 229
EP - 236
JA - Educational Review
VL - 49
IS - 3
CY - ;
SN - 00131911
UR -
https://login.proxy.bib.uottawa.ca/login?url=http://search.ebscohost.com/login.aspx?direct=true&db=s3h&AN=SPH463552&site=ehost-live
ER -

TY - JOUR
ID - 4381
T1 - Making progress? Sport policy, women and innovation in physical education
A1 - Evans,J.
A1 - Davies,B.
A1 - Penney,D.
Y1 - 1997///
KW - SportDiscus
KW - *CARDIOVASCULAR fitness
KW - *PHYSICAL education & training
KW - *PARTICIPATION
KW - *SPORTS
KW - GOVERNMENT policy
KW - girls
KW - Women
KW - EQUITY
KW - Elementary Schools
KW - EDUCATION,Secondary
KW - Great Britain
KW - Policy
RP - NOT IN FILE
SP - 39
EP - 50
JA - European Journal of Physical Education
VL - 2
IS - 1

N2 - In recent years, a variety of policy documents issuing from major providers of sport in the UK and elsewhere (for example, Sports Council UK, 1994 a,b; Sports Council for Wales, 1995a; Sports Council for Northern Ireland, 1996) have highlighted, amongst many other things, the significance of in-school and extra-school Physical Education (PE) in any endeavour to promote equity and greater participation of girls and women at all levels in school and community physical activity and sport. With reference mainly to the most recent of these policy texts, the Sports Council for Northern Ireland (SCNI) Women in Sport Policy Framework, published in 1996, and calling on research carried out by the authors of this paper on PE in primary and secondary schools in England and Wales, this paper adds further weight to the claim expressed in these texts that PE in the primary sector in particular, is a critical site of influence upon children's thinking about themselves and others, both in physical activity and sport. Lesson time PE and more informal playground activity are in dire need of attention. And unless women, the majority of teachers in primary schools in England and Wales, are positioned as leading agents in curriculum innovation and change, progress towards greater equity in PE and sport is unlikely to be achieved. Finally the paper considers whether the arrival of a National Curriculum PE (NCPE) will help promote the interests of girls and women in PE and sport in both primary and secondary schools and prepare them for their futures in leisure and paid work
SN - 13627120
UR -
https://login.proxy.bib.uottawa.ca/login?url=http://search.ebscohost.com/login.aspx?direct=true&db=s3h&AN=SPH462188&site=ehost-live;http://articles.sirc.ca/search.cfm?id=462188
L2 - http://articles.sirc.ca/search.cfm?id=462188
ER -

TY - JOUR
ID - 4382
T1 - Changes to (primary) school recess and their effect on children's physical activity: an Australian perspective
A1 - Evans,J.
Y1 - 2003/05//
KW - SportDiscus
KW - *PHYSICAL fitness
KW - *RECESSES
KW - *CARDIOVASCULAR fitness
KW - *STRESS (Physiology)
KW - *PHYSICAL education & training
KW - primary school
RP - NOT IN FILE
SP - 53
EP - 62
JA - Journal of Physical Education New Zealand
VL - 36
IS - 1
CY - ;
N2 - Headlines greet us on almost a daily basis lamenting the declining health of Australian children. They are said to be inactive, unfit, overweight and lacking in fundamental motor skills. It is a disturbing picture. Calls have gone out to parents to encourage their children to be more active and for schools to counter these problems by allocating more time to sport and physical education however, in both instances, there are particular problems to be addressed. One aspect of schooling that is rarely considered in discussions about how to increase children's activity levels is recess. This article examines the problems facing physical education and offers some suggestions as to how schools might preserve and promote physical activity during recess breaks
SN - 11725958
UR -
https://login.proxy.bib.uottawa.ca/login?url=http://search.ebscohost.com/login.aspx?direct=true&db=s3h&AN=SPHS-887704&site=ehost-live;http://articles.sirc.ca/search.cfm?id=S-887704
L2 - http://articles.sirc.ca/search.cfm?id=S-887704
ER -

TY - JOUR

ID - 4383
T1 - THE PLAYGROUND WORKOUT
A1 - Even-Esh,Z.A.C.H.
Y1 - 2011/08//
KW - SportDiscus
KW - *PHYSICAL fitness
KW - *EXERCISE
KW - *PHYSICAL training & conditioning
KW - *PLAYGROUNDS
KW - OPEN spaces
RP - NOT IN FILE
SP - 87
EP - 98
JA - Men's Fitness
VL - 27
IS - 6
N2 - The article offers how one can do workouts in local parks this summer away from a dark gym. All one really need is something to push on or pull on and a little open space one can move the body The program makes use of wide open spaces, plus the equipment that's generally found around parks and children's playgrounds. One can do pull-ups and dips and also do fun body-weight exercises. The article also gives day-wise exercise schedules
SN - 15412776
UR -
https://login.proxy.bib.uottawa.ca/login?url=http://search.ebscohost.com/login.aspx?direct=true&db=s3h&AN=63308395&site=ehost-live
ER -

TY - JOUR
ID - 4385
T1 - Early-Life Outdoor Experiences and an Individual's Environmental Attitudes
A1 - Ewert,A.L.A.N.
A1 - Place,G.R.E.G.
A1 - Sibthorp,J.I.M.
Y1 - 2005/05//
KW - SportDiscus
KW - *COLLEGE students
KW - *OUTDOOR life
KW - *CHILDREN
KW - *OUTDOOR recreation
KW - Life Change Events
KW - Regression Analysis
KW - Research
KW - early-life outdoor experience
KW - Environmental Attitudes
KW - New Environmental Paradigm (NEP)
RP - NOT IN FILE
SP - 225
EP - 239
JF - Leisure Sciences
VL - 27
IS - 3
N2 - This study investigated the effects of early-life experiences on an individual's environmental beliefs. Data from a survey of 533 university undergraduate students from 20 areas of academic study were analyzed using sequential regression to determine the degree to which current environmental beliefs could be explained by early childhood experiences. Results showed that four of the seven independent variables (appreciative outdoor activities, consumptive outdoor activities, media exposure, and witnessing negative environmental events) explained 14% of the variance in the eco-centric/anthropocentric beliefs. Three of the independent variables

(early-life participation in mechanized outdoor activities, education, and involvement with organizations) were not significant predictors of eco-centric/anthropocentric beliefs. Implications for research and practice were discussed. ABSTRACT FROM AUTHOR
SN - 01490400
UR -
https://login.proxy.bib.uottawa.ca/login?url=http://search.ebscohost.com/login.aspx?direct=true&db=s3h&AN=16968009&site=ehost-live
ER -

TY - JOUR
ID - 4386
T1 - A Review of Physical Activity Levels During Elementary School Physical Education
A1 - Fairclough,Stuart J.
A1 - Stratton,Gareth
Y1 - 2006/04//
KW - SportDiscus
KW - *PHYSICAL education
KW - *SCHOOLS -- Exercises & recreations
KW - *EXERCISE
KW - *PHYSICAL education in literature
KW - *STUDENTS
KW - *SCHOOL children
KW - Elementary Education
KW - Great Britain
KW - United States
RP - NOT IN FILE
SP - 239
EP - 257
JF - Journal of Teaching in Physical Education
VL - 25
IS - 2
N2 - The article focuses on the importance of physical education in schools. In the United States and Great Britain, guides are published that emphasize on the roles of the physical education and suggest that school children should work out at least for 60 minutes per day to maintain their physical, physiological and psychological developments. The article also tries to correlate physical activity with the educational progress. During elementary education, children should be involved in physical education so that they remain active in it through out their educational time span. The article reviews literature of physical education and tries to define terms and concepts of physical activities which could be taught during physical education sessions. The article also provides the assessment modes to measure physical activities of students
SN - 02735024
UR -
https://login.proxy.bib.uottawa.ca/login?url=http://search.ebscohost.com/login.aspx?direct=true&db=s3h&AN=21368892&site=ehost-live
ER -

TY - JOUR
ID - 4391
T1 - Kvinner fritid - finns det utrymme foer motion? / Women's leisure time - is there room for physical activity?
A1 - Fasting,K.
Y1 - 1988///
KW - SportDiscus
KW - *LEISURE
KW - *RECREATION
KW - *SPORTS
KW - *STRESS (Physiology)
KW - WOMEN -- Sociological aspects

KW - Women
RP - NOT IN FILE
CY - ;
N2 - The first part of the paper presented contains definition of leisure time and the critique of these definitions from a woman's perspective. The second part contains results from different studies that have taken place during the last years. These studies show that women have less time for their own disposal. They are also less involved in organized activities outside the home compared to men. A large study from Sheffield in England showed that gender had great significance for the leisure behaviour. Particularly in milieus where homework and taking care of the children first of all were done by women. Existing norms about what is suitable behaviour for women seemed to be a significant barrier. The next part of the article discusses what conclusions can be drawn in relation to the Nordic countries. The question asked is if it is reasonable to suggest that women should prefer physical exercise before other leisure time activities? In this connection it focuses on the difference between the male and the female culture. The last part of the article contains results from a Norwegian study which covered married 30 year old women with children from 2 different parts of the city of Oslo, Norway. This study shows that women participate in traditional sport disciplines which can be done individually, independent of other people and independent of special sport facilities. The conclusion is that this is in many ways a logical consequence of a mother's everyday life
UR -
https://login.proxy.bib.uottawa.ca/login?url=http://search.ebscohost.com/login.aspx?direct=true&db=s3h&AN=SPH241634&site=ehost-live;http://articles.sirc.ca/search.cfm?id=241634
L2 - http://articles.sirc.ca/search.cfm?id=241634
ER -

TY - JOUR
ID - 4392
T1 - A Well Baby Clinic in Indianapolis
A1 - Fee,Elizabeth
A1 - Brown,Theodore M.
A1 - Beatty,Roxanne L.
Y1 - 2003/02//
KW - SportDiscus
KW - *MEDICAL centers
KW - *HEALTH promotion
KW - INFANT health services
KW - INDIANAPOLIS (Ind.)
KW - Indiana
RP - NOT IN FILE
SP - 271
EP - 271
JF - American Journal of Public Health
JA - Am J Public Health
VL - 93
IS - 2
N2 - The article presents background information on the baby clinic of the Herman C. Morgan Health Center in Indianapolis, Indiana. The health center's baby clinic was opened in 1947. The center was helped established by two physicians, Walter H. Maddux and Harvey N. Middleton. The health center was created as part of the Flanner House, which promotes the social, moral and physical welfare of African American community. Flanner House's programs included a day nursery, children's playground and employment center. The program established at the center was the only health facility in the city providing a public health program and preventive medicine
SN - 00900036
UR -
https://login.proxy.bib.uottawa.ca/login?url=http://search.ebscohost.com/login.aspx?direct=true&db=s3h&AN=9036707&site=ehost-live
ER -

TY - JOUR

ID - 4394
T1 - Leg strength is related to endurance run performance in children and adolescents with mental retardation
A1 - Fernhall,B.
A1 - Pitetti,K.H.
Y1 - 2000/08//
N1 - Accession Number: 2000065807. Language: English. Entry Date: 20001001. Revision Date: 20131213. Publication Type: journal article; research; tables/charts. Journal Subset: Allied Health; Biomedical; Peer Reviewed; USA. No. of Refs: 20 ref. NLM UID: 8909729
KW - CINAHL
KW - Leg -- In Adolescence
KW - Muscle Strength -- In Adolescence
KW - Physical Endurance -- In Adolescence
KW - Running -- In Adolescence
KW - Intellectual Disability -- In Adolescence
KW - Child
KW - adolescence
KW - Male
KW - Female
KW - Oxygen Consumption -- Evaluation
KW - Isokinetic Exercises
KW - Exercise Test
KW - Body Weights and Measures
KW - Descriptive Statistics
KW - Treadmills
KW - Dynamometry
KW - Step-Wise Multiple Regression
KW - Torque -- Evaluation
KW - Flexion -- Evaluation
KW - Human
RP - NOT IN FILE
SP - 324
EP - 333
JF - Pediatric Exercise Science
JA - Pediatr Exerc Sci
VL - 12
IS - 3
N2 - This study evaluated the relationship between leg strength and endurance run performance, independent of aerobic capacity (VO2peak), body size, and gender, in children and adolescents with mild or moderate mental retardation. Twenty-six individuals (15 boys and 11 girls) volunteered and underwent tests of VO2peak, isokinetic leg strength, and endurance run performance (600-yard run/walk and 20-m shuttle run). Results showed that leg strength was significantly related to both types of run performance; however, when controlling for VO2peak, body size, and gender, leg strength was a more significant contributor to the 600-yard run/walk than to 20-m shuttle run performance. Gender did not influence these relationships. These data suggest that leg strength has a significant influence on endurance run performance in children and adolescents with mild or moderate mental retardation
SN - 0899-8493
AD - Exercise Science Programs, George Washington University Medical Center, Washington, DC 20052
UR - http://search.ebscohost.com/login.aspx?direct=true&db=cin20&AN=2000065807&site=ehost-live
ER -

TY - JOUR
ID - 4396
T1 - Dostopnost slovenskih □-olskih telovadnic za gibalno ovirane uŽ_ence. / Accessibility of Slovene school sport halls for pupils with physical disabilities
A1 - FilipŽ_iŽ_,Tja • a
A1 - VardiŽ_,Katarina
A1 - PodpeŽ_an,Katja

A1 - Verhovnik,Maja
A1 - Rebek,Helena
A1 - Stojko,Monika
A1 - Jurak,Gregor
Y1 - 2012/06//
KW - SportDiscus
KW - *CHILDREN with disabilities
KW - *SPORTS facilities
KW - School Facilities
KW - PRIMARY schools
KW - Secondary Education
KW - Slovenia
KW - accessibility
KW - architectonic barriers
KW - pupils with physical impairments
KW - arhitektonske ovire
KW - dostopnost
KW - gibalno ovirani uŽ_enci
RP - NOT IN FILE
SP - 203
EP - 208
JA - Sport: Revija Za Teoreticna in Prakticna Vprasanja Sporta
VL - 60
IS - 1/2
N2 - All public buildings should be accessible to all. Accessibility should be universal. In present research 107 primary and secondary schools from 5 Slovene regions were involved. We found out 53% of schools don't have suitable access to enter the school, 55% don't have suitable access in a school sport hall, a restrooms are accessible inappropriately still (60%). No school has suitable access to outside playgrounds. Largest barriers are a stairs, too narrow or rotating door and narrow corridors. Results demand suitable treatment and deliberation now and of course at that time, when possible modernizations and renovations of our schools and school gymnasia would take place. ABSTRACT FROM AUTHOR
SN - 03537455
UR -
https://login.proxy.bib.uottawa.ca/login?url=http://search.ebscohost.com/login.aspx?direct=true&db=s3h&AN=77392866&site=ehost-live
ER -

TY - JOUR
ID - 4398
T1 - The school sport co-ordinator programme: changing the role of the physical education teacher?
A1 - Flintoff,A.
Y1 - 2003/10//
KW - SportDiscus
KW - *SPORTS
KW - *PHYSICAL education & training
KW - *TEACHING
KW - Schools
KW - PROGRAMS
KW - Management
KW - POLITICAL science
KW - Teachers
KW - Great Britain
KW - Policy
RP - NOT IN FILE
SP - 231
EP - 250
JA - Sport, Education & Society

VL - 8
IS - 2
CY - ;
N2 - Over the last decade or so, young people have increasingly become a focus of UK sport policy. Fuelled in part by concerns such as the increasing levels of childhood inactivity and obesity, and the lack of international success in sport, a plethora of policy initiatives aimed at young people have been developed. In April 2000, the government published its sport strategy document, A Sporting Future for All, pulling together all the threads of recent policies, and in it, restating its commitment to youth sport, sport in education, excellence and sport in the community. One such policy initiative, the School Sport Co-ordinator programme, is the focus of this paper. The School Sport Co-ordinator programme, currently being introduced into schools in England, is an initiative that involves two government departments (sport and education) and a number of other agencies, reflecting the government's current agenda to ensure 'joined up policy' thinking. It aims to develop opportunities for youth sport through co-ordinated links between PE and sport in schools, both within and outside of the formal curriculum, with those in local community sports settings. The essence of the School Sport Co-ordinator programme is to free up nominated teachers in schools from teaching to allow them time for development activities, specifically to encourage schools and community sports providers to work in partnership. This paper draws on data from an ongoing research project examining the implementation of one School Sport Co-ordinator partnership, Northbridge'. Drawing on in-depth interviews, it explores the perceptions of the newly established School Sport Co-ordinators of their changing role. The paper highlights some of the initial tensions and challenges for them in their task of working across different educational and sporting contexts
SN - 13573322
UR - https://login.proxy.bib.uottawa.ca/login?url=http://search.ebscohost.com/login.aspx?direct=true&db=s3h&AN=SPHS-912723&site=ehost-live;http://articles.sirc.ca/search.cfm?id=S-912723
L2 - http://articles.sirc.ca/search.cfm?id=S-912723
ER -

TY - JOUR
ID - 4400
T1 - An international investigation on the validity of the CSAPPA [Children's Self-perceptions of Adequacy in and Predilection for Physical Activity] scale in screening for developmental coordination disorder
A1 - Flouris,A.D.
Y1 - 2004///
KW - SportDiscus
KW - *MOVEMENT disorders
KW - *DIAGNOSIS
KW - PERCEPTUAL-motor processes
KW - Child Development
KW - COORDINATION
KW - MOTOR DISORDER
RP - NOT IN FILE
CY - Eugene, OR;
PB - Kinesiology Publications, University of Oregon
N2 - The main objective of the present investigation was to continue the research initiated by Hay and colleagues (2004) in examining the efficacy of the Children's Self-Perceptions of Adequacy in and Predilection for Physical Activity (CSAPPA) scale as a proxy for the short form .of the Bruininks-Oseretsky Test of Motor Proficiency (BOTMP-SF) in screening for Developmental Coordination Disorder (DCD) in children. To better appreciate DCD knowledge outside Canada, the measurements of this investigation were expanded in Greece. A translated Greek CSAPPA scale and the BOTMP-SF were administered for the first time in Greek children. A second objective was to investigate the relationship between DCD and various risk factors of coronary artery disease (CAD) in Canadian and Greek children. A sample of 591 (Ms=322; Fs=269) Canadian and 392 (Ms=211; Fs=181) Greek children, aged 9 to 13 years, consented to the BOTMP-SF, CSAPPA Scale, participating in physical activity questionnaire, Leger 20-meter Multistage Shuttle Run test, and body fat using bioelectric impedance. Prevalence of DCD in Canada and Greece was 8% and 19%, respectively. Significant agreement (p<0.001) was confirmed when comparing the CSAPPA scale to the BOTMP-SF test in both countries. Canadian children revealed significantly lower percent body fat, CSAPPA scores, and participation in physical activity, as well as higher aerobic fitness levels and BOTMP-SF compared to their Greek peers.

Clumsiness was associated with increased percent body fat and low aerobic fitness values. Physical activity was a significant mediator in the clumsiness-aerobic fitness relationship. It is concluded that the CSAPPA scale is an accurate, practical, and inexpensive screening tool for DCD, and that motoric competence is associated with aerobic fitness through physical activity participation
UR -
https://login.proxy.bib.uottawa.ca/login?url=http://search.ebscohost.com/login.aspx?direct=true&db=s3h&AN=SPHS-994339&site=ehost-live;http://kinpubs.uoregon.edu/
ER -

TY - JOUR
ID - 4401
T1 - Fun for the Whole Family
A1 - Flynn,Jennifer
Y1 - 2013/10//
KW - SportDiscus
KW - *PHYSICAL activity
KW - *HEALTH
KW - *LIFESTYLES
KW - *ROPE skipping
KW - *CHILDREN -- Health
KW - FAMILIES
KW - COST effectiveness
KW - PHYSIOLOGICAL aspects
RP - NOT IN FILE
SP - 4
EP - 4
JA - ACSM Fit Society Page
VL - 15
IS - 3
N2 - The article focuses on the engagement of the physical activity as a way for helping the family in adopting the healthy lifestyles and spending more time together. It says that one of the most cost-effective strategies for physical activity is the outdoor activities. It adds that bone-strengthening benefits for both the parents and children can be offered by jumping rope. The likelihood of the children in adopting a healthy lifestyle can be influenced by a physically active family environment
UR -
https://login.proxy.bib.uottawa.ca/login?url=http://search.ebscohost.com/login.aspx?direct=true&db=s3h&AN=91743834&site=ehost-live
ER -

TY - JOUR
ID - 4403
T1 - Exploring the physical activity levels of students with mental retardation and students without disabilities in both school and after-school environments
A1 - Foley,J.T.
Y1 - 2005///
KW - SportDiscus
KW - *MENTAL retardation
KW - *ACCELEROMETERS
KW - *CARDIOVASCULAR fitness
KW - PERCEPTUAL-motor processes
KW - TESTING
KW - Elementary Schools
KW - Child
KW - DISABILITIES-Mental retardation - research
RP - NOT IN FILE
CY - Eugene, OR;
PB - Kinesiology Publications, University of Oregon

N2 - The focus of this research was to gain an understanding of physical activity in elementary school-aged children with mental retardation (MR). The primary purpose of the first study was to investigate physical activity levels of children with and without MR in both school and out of school environments. The secondary purpose of the first study was to determine if there is a relationship between physical activity and motor skills in children with MR. Physical activity levels of 9 children with MR and 37 without were recorded over 7 days using an accelerometer. Motor skills were assessed using the Movement Assessment Battery for Children. Physical activity levels for children with MR were significantly lower during the school day. Children without MR were 53% more active during recess and 133% more active during physical education than children with MR. The disparity in physical activity continued outside of school where children without MR were 52% more active after school and 33% more active on the weekend than children with MR. There were no significant correlations between physical activity times and motor skills in either group. These findings indicate that interventions to increase physical activity in individuals with MR should to begin in the elementary school years. The primary purpose of the second study was to investigate if children with MR spend more time watching TV or on the computer than children without disabilities. The secondary purpose of study was to determine if total screen time correlates with physical activity levels. Parents of the participants recorded TV time and computer time over four days. Physical activity levels of the children were recorded with accelerometers. There was no difference in TV time or computer time in children with MR and children without MR. The correlation between after school physical activity and screen time was moderate in children with MR (r=.69 and low for children without MR (r=-.32). The results of this research suggest that there might be different patterns in the development of sedentary behaviors between children with and without MR, indicating that unique interventions may be needed for individuals with MR
UR -
https://login.proxy.bib.uottawa.ca/login?url=http://search.ebscohost.com/login.aspx?direct=true&db=s3h&AN=SPHS-1005677&site=ehost-live;http://kinpubs.uoregon.edu/
ER -

TY - JOUR
ID - 4404
T1 - Blood Pressure During Leg Extension in Children
A1 - Forberg,S.I.
A1 - StA,ren,A,
A1 - Fredriksen,P.M.
Y1 - 2012/10//
KW - SportDiscus
KW - *BLOOD pressure
KW - *EXERCISE
KW - *JOINTS -- Range of motion
KW - *LEG
KW - *MUSCLE strength
KW - analysis of covariance
KW - Analysis of Variance
KW - Statistics
KW - T-test (Statistics)
KW - data analysis
KW - REPEATED measures design
KW - DATA analysis -- Software
KW - Descriptive Statistics
KW - adolescents
KW - knee extension
KW - strength testing
RP - NOT IN FILE
SP - 802
EP - 806
JF - International Journal of Sports Medicine
JA - Int J Sports Med
VL - 33
IS - 10

N2 - This study investigated blood pressure responses (BP) during bilateral leg extension in healthy children at different numbers of repetitions. BP was measured non-invasively during 1 repetition maximum (1RM), 5 repetitions at 85-90 % of 1RM (5 R), 30 repetitions at 35-40 % of 1RM (30 R) and during 5 s of maximal isometric work (5SM). It is important to investigate if maximal strength testing in healthy children provokes an unfavourable rise in BP, in order to be able to do further research on hypertensive risk groups of children. 39 (18 male and 21 female) healthy children (10.5 Añ2.0 years old) participated in this study. Peak BP values and peak HR increased significantly with increasing number of repetitions. Peak BP increased from 1RM (144 Añ16/110 Añ19 mmHg) to 30R (170 Añ26/123 Añ21 mmHg) and peak HR increased from 1RM (122 Añ10 bpm) to 30 R (147 Añ14 bpm). 30R also gave significantly higher BP and HR than 5SM (156 Añ24/120 Añ23 mmHg and 131 Añ16 bpm, respectively). The boys had significantly higher diastolic BP during 1RM than the girls and significantly higher BP during 5SM. In conclusion, maximal strength testing induced lower increases in BP than submaximal strength testing with more repetitions, and may be performed in healthy children within safe BP limits (< 225 mmHg in systolic BP). ABSTRACT FROM AUTHOR
SN - 01724622
UR -
https://login.proxy.bib.uottawa.ca/login?url=http://search.ebscohost.com/login.aspx?direct=true&db=s3h&AN=80236932&site=ehost-live
ER -

TY - JOUR
ID - 4406
T1 - Pilot Study of Novel Test Instrumentation to Evaluate Therapeutic Horseback Riding
A1 - Fox,V.Marie
A1 - Lawlor,Valorie A.
A1 - Luttges,Marvin W.
Y1 - 1984/01//
KW - SportDiscus
KW - *OUTDOOR recreation for children with disabilities
KW - *HORSEMANSHIP
KW - *HAND
KW - *HIP joint
KW - *KNEE
KW - *ANKLE
KW - PHYSICAL therapist & patient
KW - SELF-reliance in children
RP - NOT IN FILE
SP - 30
EP - 36
JF - Adapted Physical Activity Quarterly
JA - Adapt Phys Act Q
VL - 1
IS - 1
N2 - A novel test instrument was designed to objectively quantify the progress of persons who participated in therapeutic horseback riding programs. Nineteen handicapped children, ages 7 to 14 years, with heterogeneous impairments were evaluated before and after riding. For measures of sitting balance and coordination, and hand, hip, knee, and ankle strength, marked improvements were noted for most children. Clinical impressions of therapists and parents suggested concomitant progress in characteristics such as self-confidence and interaction with others. Results from this pilot study appeared to support the use of the apparatus in that field setting. ABSTRACT FROM AUTHOR
SN - 07365829
UR -
https://login.proxy.bib.uottawa.ca/login?url=http://search.ebscohost.com/login.aspx?direct=true&db=s3h&AN=20846419&site=ehost-live
ER -

TY - JOUR
ID - 4407

T1 - Spielraum anders begreifen und gestalten. / Conceiving and organizing playing spaces in a different way
A1 - Franke,A.
A1 - Fischer,K.
Y1 - 2001/09//
KW - SportDiscus
KW - *RECREATION
KW - *PLAY
KW - *OUTDOOR life
KW - *PLAYGROUNDS
KW - INFANTS
KW - Child Development
KW - DESIGN
KW - building
KW - Child
KW - Case Report
RP - NOT IN FILE
SP - 108
EP - 118
JA - Motorik
VL - 24
IS - 3
CY - ;
N2 - Only a few decades ago children had the possibility to spend their whole leisure time in the open air. In many places they found natural playing spaces where they could move freely about and which they could perceive with all their senses. They had even the opportunity to modify and design their environment in a variety of ways. This article deals with the importance of spaces for moving about and gathering experiences for the development of children and presents project ideas for changing the outside area of a nursery school to a natural playing space
Il y a peu de decennies, les enfants avaient la possibilite de passer tout leur temps libre a l'exterieur. A maintes places ils trouvaient des espaces de jeu naturels, dans lesquels ils pouvaient se mouvoir librement et qu'ils pouvaient percevoir avec tous les sens. De cette facon ils avaient aussi la possibilite de changer et de creer leur environnement. La contribution traite de l'importance de l'espace de jeu de mouvement et d'exploration pour le developpement de l'enfant et presente des idees de projet concernant la transformation de l'exterieur d'une classe prescolaire en un espace de jeu naturel
Noch vor wenigen Jahrzehnten hatten Kinder die Moeglichkeit, ihre gesamte Freizeit im Freien zu verbringen. Vielerorts fanden sie naturbelassene Spielraeume vor, in denen sie sich frei bewegen und die sie mit allen Sinnen wahrnehmen konnten. So hatten sie auch die Moeglichkeit, ihre Umgebung zu veraendern und zu gestalten. Der Beitrag thematisiert die Bedeutung des Bewegungs- und Erfahrungsspielraumes fuer die kindliche Entwicklung und stellt Projektideen zur Umgestaltung eines Kindergartenaussengelaendes zu einem naturnahen Spielraum vor
SN - 01705792
UR -
https://login.proxy.bib.uottawa.ca/login?url=http://search.ebscohost.com/login.aspx?direct=true&db=s3h&AN=SPHS-793786&site=ehost-live;http://articles.sirc.ca/search.cfm?id=S-793786
L2 - http://articles.sirc.ca/search.cfm?id=S-793786
ER -

TY - JOUR
ID - 4408
T1 - My garden is an expression of me: Exploring householders' relationships with their gardens
A1 - Freeman,Claire
A1 - Dickinson,Katharine JM
A1 - Porter,Stefan
A1 - van Heezik,Yolanda
Y1 - 2012///
KW - Reviewer Nominated
RP - NOT IN FILE

SP - 135
EP - 143
JF - Journal of Environmental Psychology
VL - 32
IS - 2
PB - Elsevier
SN - 0272-4944
ER -

TY - JOUR
ID - 4409
T1 - Assessing the Social and Physical Contexts of Children's Leisure-Time Physical Activity: An Ecological Momentary Assessment Study
A1 - Fridlund Dunton,Genevieve
A1 - Kawabata,Keito
A1 - Intille,Stephen
A1 - Wolch,Jennifer
A1 - Pentz,Mary Ann
Y1 - 2012/01//
KW - SportDiscus
KW - *LEISURE
KW - *PHYSICAL activity
KW - *PHYSICAL fitness
KW - FAMILIES
KW - CHINO (Calif.)
KW - California
KW - behavioral
KW - Built Environment
KW - descriptive
KW - education/income level
KW - Exercise
KW - Family
KW - fitness/physical activity
KW - geographic location
KW - Health focus
KW - local community
KW - modeling/relationship testing
KW - nonexperimental
KW - Outcome measure
KW - Physical Activity
KW - Prevention Research.Manuscript format
KW - race/ethnicity
KW - Repeated Measures
KW - Research
KW - Research purpose
KW - Setting
KW - Strategy
KW - study design
KW - Target population age
KW - Target population circumstances
KW - youth
RP - NOT IN FILE
SP - 135
EP - 142
JF - American Journal of Health Promotion
JA - Am J Health Promot
VL - 26

IS - 3
N2 - Purpose. To use Ecological Momentary Assessment with mobile phones to describe where and with whom children's leisure-time physical activity occurs. Design. Repeated assessments across 4 days (Fridayƒ_"Monday) during nonschool time (20 total). Setting. Chino, California, and surrounding communities. Subjects. Primarily low to middle income children (N = 121; aged 9-13 years; x = 11.0 years, SD = 1.2 years; 52% male, 38% Hispanic/Latino). Measures. Electronic surveys measured current activity (e.g., active play /sports/exercise, watching TV/movies), social company (e.g., family, friends, alone), physical location (e.g., home, outdoors, school), and other perceived contextual features (e.g., safety, traffic, vegetation, distance from home). Analysis. Multilevel linear and multinomial logistic regression. Results. Most of children's physical activity occurred outdoors (away from home) (42%), followed by at home (indoors) (30%), front/backyard (at home) (8%), someone else's house (8%), at a gym/recreation center (3%), and other locations (9%). Children's physical activity took place most often with multiple categories ofpeople together (e.g., friends and family) (39%), followed by family members only (32%), alone (15%), and with friends only (13%). Age, weight status, income, and racial/ ethnic differences in physical activity contexts were observed. Conclusions. The most frequently reported contexts for children's leisure time physical activity were outdoors and with family members and friends together. ABSTRACT FROM AUTHOR
SN - 08901171
UR -
https://login.proxy.bib.uottawa.ca/login?url=http://search.ebscohost.com/login.aspx?direct=true&db=s3h&AN=70286282&site=ehost-live
ER -

TY - JOUR
ID - 4410
T1 - DOES HEIGHT MATTER?
A1 - Frost,Joe
A1 - Sutterby,John
A1 - Therrell,Jim
A1 - Brown,Pei San
A1 - Thornton,Candra
Y1 - 2002/05//
KW - SportDiscus
KW - *PLAYGROUNDS
KW - *EQUIPMENT & supplies
KW - *FALLS (Accidents)
KW - SAFETY measures
KW - United States
RP - NOT IN FILE
SP - 74
JA - Parks & Recreation
VL - 37
IS - 5
N2 - Discusses the risks of the designed fall height of several playground equipment in the U.S. Benefits of climbing for children; Details of the national guidelines and standards for heights and surfacing in the U.S.; Recommended fall height of climbing equipment and slides in the country; Factors which affect the potential injury resulting from falls; Discussion on playground safety
SN - 00312215
UR -
https://login.proxy.bib.uottawa.ca/login?url=http://search.ebscohost.com/login.aspx?direct=true&db=s3h&AN=6554089&site=ehost-live
ER -

TY - JOUR
ID - 4411
T1 - Les organisations de jeunesse en Alsace concordataire, 1918-1939: pour une histoire des sociabilites. / Youth organisations under the Concordat in Alsace, 1918-1939: for a history of sociabilities
A1 - Fuchs,J.

Y1 - 2003///Hiver
KW - SportDiscus
KW - *SPORTS
KW - teenagers
KW - FEDERATIONS
KW - History
KW - France
KW - Child
KW - ALSACE
KW - 1920D
KW - 1930D
KW - 1918
KW - 1919
RP - NOT IN FILE
SP - 27
EP - 42
JA - STAPS: Revue des Sciences et Techniques des Activites Physiques et Sportives
VL - 24
IS - 60
CY - ;
N2 - The following paper deals with the youth organisations in Alsace and covers the inter-war years (1918-1939). This period mark indeed a significant change in the educational practices, now characterized by an expansion and renewal of the organisations being in charge of the youth outside school time. The study is based on the ideas of the people in charge and on the sociability practices offered to the young Alsatians in these movements. In this frame, the valorization of nature as the privileged environment for socialization appears to be a transversal process. This trend is backed up by the use of physical exercises (walk and collective sports game for example) that become major teaching aids. The sources used in this paper are original and rarely capitalized on in the STAPS field (archives from the Strasbourg's Bishopric, the Alsace-Lorraine's Directory of the Augsbourg's Confession, internal archives of organisations, former members' accounts etc.). These sources help to pay attention to a whole range of physical practices that have an educational ambition. As a region shaped by a unique history and particular social links, the example of Alsace allows us to reach an understanding of the objectives and means of mobilization of the youth at the beginning of the twentieth century
L'etude proposee prend pour objet les organisations de jeunesse en Alsace. La periode consideree est celle de l'entre-deux-guerres (1918-1939). Moment de mutations importantes par rapport aux pratiques educatives de l'avant-guerre, elle se caracterise par un essor et un renouvellement des structures de la prise en charge des jeunes hors du temps scolaire. L'objectif de la recherche est d'approcher les conceptions des responsables et les pratiques de sociabilite proposees aux jeunes Alsaciens dans ces groupements. La valorisation de la nature en tant qu'espace privilegie de socialisation emerge dans ce cadre comme une tendance transversale. Ce processus s'accompagne de l'utilisation d'exercices physiques (marche et jeux sportifs collectifs notamment) comme supports pedagogiques prioritaires. L'etude entreprise s'appuie sur des sources originales et peu exploitees dans le champ des STAPS (archives de l'Eveche de Strasbourg, du Directoire de l'Eglise de la Confession d'Augsbourg d'Alsace-Lorraine, archives internes des organisations, temoignages d'anciens membres ...); celles-ci incitent a prendre en compte un ensemble de pratiques corporelles qui ont une ambition educative. Terrain singulier d'histoire et de liens sociaux, l'Alsace constitue ici un exemple particulierement riche des enjeux et moyens de la mobilisation de la jeunesse au debut du XXe siecle
Lo studio proposto ha per oggetto le organizzazioni della gioventu in Alsazia. Il periodo considerato e quello tra le due guerre (1918-1939). Momento di trasformazioni importanti in rapporto alle pratiche educative del secondo anteguerra, esso si caratterizza per uno stimolo ed un rinnovamento delle strutture della presa in carico dei giovani al di fuori del tempo scolastico. L'obiettivo della ricerca e di avvicinarsi alle concezioni dei responsabili a delle pratiche di sociabilita proposte ai giovani alsaziani in questi raggruppamenti. La valorizzazione della natura, in quanto spazio privilegiato di socializzazione, emerge in questo quadro come una tendenza trasversale. Questo processo e accompagnato dall'utilizzazione di esercizi fisici (in particolare la marcia ed i giochi sportivi collettivi) come supporti pedagogici prioritari. Lo studio intrapreso si basa su fonti originali a poco sfruttate nel campo degli STAPS (archivi del Vescovo di Strasburgo, del Curia della Chiesa di Augsbourg dell'Alsazia Lorena, archivi interni delle organizzazioni, testimonianze di vecchi membri, ecc.); questo ultimi invitano a prendere in considerazione un insieme di pratiche corporee the hanno un'ambizione educativa. Terreno singolare di storia a di legami sociali, l'Alsazia costituisce qui un esempio particolarmente

ricco delle poste in gioco a dei mezzi di mobilitazione della gioventu all'inizio del XX secolo
El estudio tiene por objetivo analizar las organizaciones de los jovenes de la Alsace en el periodo considerado entre las dos guerras (1919-1939). Momentos de cambios importantes en relacion a las practicas educativas antes de la guerra, esto se caracteriza por un desarrollo y una renovacion de las estructuras que tienen a cargo a los jovenes despues de las horas escolares. El objetivo de la investigacion es de aproximar las concepciones de los responsables y las practicas de sociabilidad propuesta a los jovenes Alsacianos. La valorizacion material que emerge como proceso socializador es una tendencia transversal. Este proceso es acompanado de ejercicios fisicos (caminatas, juegos deportivos colectivos) como soportes pedagogicos prioritarios. El estudio emprendido se apoya en fuentes originales y poco explotadas en el campo de las STAPS (archivos del arzobispado de Estrasbourgo, del directorio de la iglesia de la Confesion de Ausburgo de Alsace Lorrain, archivos internos de las organizaciones, testimonios de miembros antiguos), ellos invitan a tomar en cuenta el conjunto de practicas corporales que tienen una ambicion educativa
Die vorliegende Studie befasst sich mit Jugendorganisationen im Elsass zwischen den Weltkriegen (1918-1939). Als eine Periode wichtiger Mutationen hinsichtlich erzieherischer Praktiken vor dem Krieg ist diese Zeit gekennzeichnet durch ein Aufkommen und eine Erneuerung von ausserschulischen Strukturen der Jugendbetreuung. Ziel der Untersuchung ist es, die Konzeptionen der Verantwortlichen und die in diesen Strukturen den jungen Elsaessern gebotenen Soziabilitaetspraktiken in Beziehung zu setzen. Bei diesem Prozess dienen die Leibesuebungen (besonders Maersche und Mannschaftsspiele) als vorwiegendes paedagogisches Mittel. Die Studie stuetzt sich auf wenig ausgewertete Originalquellen im Bereich der Sportwissenschaft (Archive des Bistums Strassburg, der Kirchenverwaltung der Augsburger Konfession von Elsass-Lothringen, interne Archive der Organisationen, Augenzeugenberichte...), die auf koerperliche Praktiken mit erzieherischen Ambitionen verweisen. In seiner historischen und sozialen Einzigartigkeit zeigt sich das Elsass hier als ausserordentlich reiches Beispiel, bei dem es um die Mobilisierung der Jugend Anfang des 20. Jahrhunderts ging
SN - 0247106X
UR - https://login.proxy.bib.uottawa.ca/login?url=http://search.ebscohost.com/login.aspx?direct=true&db=s3h&AN=SPHS-879614&site=ehost-live;http://articles.sirc.ca/search.cfm?id=S-879614
L2 - http://articles.sirc.ca/search.cfm?id=S-879614
ER -

TY - JOUR
ID - 4416
T1 - Hot topics in pediatric environmental health
A1 - Galvez,M.P.
A1 - Graber,N.M.
A1 - Sheffield,P.E.
A1 - Forman,J.A.
A1 - Balk,S.J.
Y1 - 2009/07//
N1 - Accession Number: 2010352914. Language: English. Entry Date: 20090828. Revision Date: 20090828. Publication Type: journal article; pictorial; tables/charts. Journal Subset: Biomedical; Blind Peer Reviewed; Editorial Board Reviewed; Expert Peer Reviewed; Online/Print; Peer Reviewed; USA. Special Interest: Pediatric Care. No. of Refs: 42 ref. NLM UID: 8702030
KW - CINAHL
KW - Air Pollutants,Environmental
KW - Environmental Exposure -- In Infancy and Childhood
KW - Home Environment
KW - Natural Environment
KW - Air Pollutants
KW - Bottle Feeding -- Adverse Effects
KW - Child
KW - Counseling
KW - Fish -- Adverse Effects
KW - Food Contamination
KW - Lead -- Adverse Effects
KW - Mercury Poisoning

KW - Mercury -- Legislation and Jurisprudence
KW - Play and Playthings -- Adverse Effects
KW - Product Labeling
KW - United States
RP - NOT IN FILE
SP - 34
JA - Contemporary Pediatrics
VL - 26
IS - 7
N2 - The dangers of phthalates, mercury, bisphenol A, and lead are examined, giving practitioners the background to pass on wise safety tips to their patients and parents
SN - 8750-0507
AD - Assistant Professor, Department of Community and Preventive Medicine, Mount Sinai School of Medicine, New York, NY
UR - http://search.ebscohost.com/login.aspx?direct=true&db=cin20&AN=2010352914&site=ehost-live
ER -

TY - JOUR
ID - 4417
T1 - The Social Role of Urbanization and the Children's Playgrounds
A1 - Gantu,M.
Y1 - 1973///
KW - SportDiscus
KW - *PLAY
KW - *SPORTS facilities
KW - *SPORTS
KW - *RECREATION
KW - SOCIALISM
KW - Time Management
KW - Urbanization
KW - Child
RP - NOT IN FILE
SP - 10
JA - Unpublished Paper
SN - 01959131
UR -
https://login.proxy.bib.uottawa.ca/login?url=http://search.ebscohost.com/login.aspx?direct=true&db=s3h&AN=SPH337746&site=ehost-live;http://articles.sirc.ca/search.cfm?id=337746
L2 - http://articles.sirc.ca/search.cfm?id=337746
ER -

TY - JOUR
ID - 4418
T1 - The effects of a hockey-specific training program on performance of Bantam players
A1 - Geer,N.
A1 - Serfass,R.
A1 - Picconatto,W.
A1 - Blatherwick,J.
Y1 - 1992/03//
KW - SportDiscus
KW - *HOCKEY
KW - *TRAINING
KW - *ACHIEVEMENT
KW - BANTAMS
KW - Comparative Studies
KW - Child
KW - SPECIFICITY OF TRAINING

KW - VERTICAL JUMP
KW - Acceleration
RP - NOT IN FILE
SP - 65
EP - 69
JA - Canadian Journal of Sport Sciences
VL - 17
IS - 1
CY - ;
N2 - Few studies have attempted to identify the effects of training on performance measures related to ice hockey. The present study was designed to examine the effects of a 7-week hockey-specific training program on the on- and off-ice test performance scores of 14- and 15-year-old (Bantam) hockey players. Pre- and post-training tests of percent fat (ultrasound), center of gravity location, 40-yard dash, vertical jump, and on-ice test of top speed, acceleration, and cornering ability were completed on 28 male subjects (16 in a training group, 12 in a control group of summer league participants). The training group showed significant improvements (p is less than .01) in percent fat, top speed, acceleration, and cornering test performance whereas only percent fat was significantly improved for the control group. The results suggest that performance on tests related to ice hockey can be improved by training specifically for hockey but that performance is not affected by summer league play alone
Peu d'etudes se sont arretees a identifier les effects de l'entrainement sur des mesures de performance au hockey sur glace. La presente etude porte sur les effets d'un programme d'entrainement de sept semaines specifique au hockey sur des mesures de performance sur glace et hors glace chez des jeunes de 14 et 15 ans (Bantam). Les mesures suivantes sont enregistrees avant et apres ces sept semaines d'entrainement: le pourcentage de graisse par ultrasonographie, la localisation du centre de gravite, le temps de performance sur 40 verges, la hauteur de saut vertical, la vitesse maximale sur glace, l'acceleration en patins, et l'habilete dans les coins de patinoire. Vingt-huit sujets d'un camp d'ete, soit 16 dans le groupe experimental et 12 dans le groupe temoin, participent a l'etude. Le groupe experimental ameliore significativement son pourcentage de graisse, sa vitesse maximale, son acceleration, et sa performance dans les coins de patinoire; le groupe temoin n'ameliore que son pourcentage de graisse. Les resultats suggerent qu'on peut ameliorer la performance a des tests relies au hockey sur glace mais qu'un camp d'ete seul ne suffit pas
SN - 08331235
UR -
https://login.proxy.bib.uottawa.ca/login?url=http://search.ebscohost.com/login.aspx?direct=true&db=s3h&AN=SPH295499&site=ehost-live;http://articles.sirc.ca/search.cfm?id=295499
L2 - http://articles.sirc.ca/search.cfm?id=295499
ER -

TY - JOUR
ID - 4419
T1 - Paternally grateful
A1 - Gemmell,Nikki
Y1 - 2012/09/09/
KW - SportDiscus
KW - *PLAYGROUNDS
KW - Fathers
KW - MEMORY (Philosophy)
KW - TENDERNESS (Psychology)
KW - Laughter
KW - SUSPICION
RP - NOT IN FILE
SP - 10
EP - 10
JA - Weekend Australian (National)
N2 - The article presents the author's reflection on the memories of her father that involve tenderness, cherishing and a fierce love of a child. The author asserts that such masculine tenderness towards a child, does not diminish his father's masculinity. She cites seeing young boys in a school playground, who were delightedly

and confidently passing around a baby and competing to get the child laughing. On the other hand, she laments the paranoid suspicion of the male in public, with kids
UR -
https://login.proxy.bib.uottawa.ca/login?url=http://search.ebscohost.com/login.aspx?direct=true&db=s3h&AN=79967055&site=ehost-live
ER -

TY - JOUR
ID - 4420
T1 - Effect of Competitive Situations on Motor Activity and Aspirations of Children Participating in Sport Classes
A1 - Geron,E.
Y1 - 1981///
KW - SportDiscus
KW - *ACHIEVEMENT motivation
KW - *ATHLETES
KW - *SPORTS
KW - AMBITION
KW - Israel
KW - Child
KW - Competition
KW - NON-ATHLETE
KW - FAILURE
RP - NOT IN FILE
SP - 12
JA - Unpublished Paper
N2 - Examines the effect of participation in competitive sport outside and inside the school on children's achievement motivation as measured by the motoric motivation test in 3 realms of behavior - motor activity, level of motivation and stability. Subjects were students chosen for sports classes, candidates for sports classes and ordinary students. Students in sports classes differed from other students by attaining a greater level of effort, but were more threatened by failure and it is suggested that this was a result of previous sport experience. After one year in the sport class, a favorable effect was found. Concludes that the organization of children's competitive sport in school can help with the negative effects in the competitive experience of child athletes
SN - 01959131
UR -
https://login.proxy.bib.uottawa.ca/login?url=http://search.ebscohost.com/login.aspx?direct=true&db=s3h&AN=SPH338402&site=ehost-live;http://articles.sirc.ca/search.cfm?id=338402
L2 - http://articles.sirc.ca/search.cfm?id=338402
ER -

TY - BOOK
ID - 4421
T1 - Nothing ventured : balancing risks and benefits in the outdoors
A1 - Gill,Tim.,English Outdoor Council.
Y1 - 2010///
KW - Reviewer Nominated
RP - NOT IN FILE
CY - [England]
PB - English Outdoor Council
SN - 9780956663603 0956663605
ER -

TY - JOUR
ID - 4422
T1 - The great outdoors: how a green exercise environment can benefit all
A1 - Gladwell,Valerie F.
A1 - Brown,Daniel K.

A1 - Wood,Carly
A1 - Sandercock,Gavin R.
A1 - Barton,Jo L.
Y1 - 2013///
KW - Reviewer Nominated
RP - NOT IN FILE
SP - 1
EP - 7
JA - Extreme Physiology & Medicine
VL - 2
IS - 1
PB - BioMed Central
SN - 2046-7648
ER -

TY - JOUR
ID - 4423
T1 - THE RELATION OF CERTAIN UNSETTLED HOME CONDITIONS TO THE ACADEMIC SUCCESS OF HIGH SCHOOL PUPILS
A1 - gnes Curtis,Erta
A1 - Nemzek,Claude L.
Y1 - 1938/11//
KW - SportDiscus
KW - *HIGH schools
KW - *SOCIAL psychology
KW - Family Conflict
KW - HOME & school
KW - Secondary Education
KW - CHRONOLOGY
RP - NOT IN FILE
SP - 419
EP - 435
JA - Journal of Social Psychology
VL - 9
IS - 4
N2 - This study has attempted to answer the question: What is the relation of unsettled or broken home conditions to the academic success of high school pupils ? The following six factors were considered broken home conditions: loss of father by death, loss of father by divorce or separation, unemployment of father, loss of mother by death, loss of mother by divorce or separation, or employment of mother outside the home. Fifty pupils were located for each of these six broken home conditions. They were paired with pupils from normal homes on the bases of intelligence, chronological age, grade in school, sex, and nationality. An honor point average based upon teachers' marks was computed for each of the 600 pupils and used as the measure of academic success. The data indicate that the school achievement of pupils from broken homes is inferior to that of pupils from normal homes. This is true for seven comparisons; four of the comparisons reveal differences which are distinctly significant statistically; two differences approach statistical significance. Experimental and control pupils were then compared on the following factors: amount of absence, amount of tardiness, occupation of the father, number of brothers, number of sisters, number of only children, language spoken in the home, amount of outside employment, amount of outside work at home, participation in club activities and athletics, number of consultations with a doctor, amount of absence due to illness, amount of home study, study conditions at home, and type of high school curriculum. These comparisons revealed slight differences, if any, and failed to indicate any causal relation to the differential achievement of the experimental and control pupils. ABSTRACT FROM AUTHOR
SN - 00224545
UR - https://login.proxy.bib.uottawa.ca/login?url=http://search.ebscohost.com/login.aspx?direct=true&db=s3h&AN=16584253&site=ehost-live
ER -

TY - JOUR
ID - 4424
T1 - What protects youngsters from violence victimisation?
A1 - Gofin,R.
A1 - Avitzour,M.
Y1 - 2010/09/02/
KW - SportDiscus
RP - NOT IN FILE
SP - A87
EP - A88
JF - Injury Prevention
JA - Inj Prev
VL - 16
N2 - Background Violence, in its varied presentation forms is common among youth. Risk factors have been identified in diverse populations for different types of violence. In this study we assessed victimisation for specific types of violence common among young people, and determined what the personal, familial and school protecting factors are. Methods Students (12ƒ_"14 years old) in 35 randomly selected junior high schools were from the Jerusalem Hebrew (secular and religious) and Arab educational system (n=2610). They answered an anonymous questionnaire, addressing personal, family, and school characteristics, unintentional injuries and violence. Victimisation included experiencing at least one of the following: was bullied, mocked, boycotted, was stolen or was taken personal belongings by force. Results Only 24.2% did not experience violence in the last term. In a multivariate analysis independent protective factors from victimisation were: type of school, being older, female (OR 1.76, 95% CI 1.40 to 2.21), being participated in activities (OR 4.33, 95% CI 2.82 to 6.63), having two or more close friends (OR 1.82, 95% CI 1.07 to 3.10), having good relations among students (OR 2.73, 95% CI 1.04 to 7.15) and having a high family support (OR 1.53, 95% CI 1.19 to 2.00). Hours of television viewing, participating in sports activities, membership in a youth movement or the reported family socioeconomic status were not significant protective factors. Conclusions Few children are not victims of violence in school. Protective factors for violence are positive personal, family and school relations. Interventions addressing these positive factors may help to reduce violence in young people and their natural environment. ABSTRACT FROM PUBLISHER
SN - 13538047
UR -
https://login.proxy.bib.uottawa.ca/login?url=http://search.ebscohost.com/login.aspx?direct=true&db=s3h&AN=66042598&site=ehost-live
ER -

TY - JOUR
ID - 4425
T1 - Seasonal effect on exercise challenge tests for the diagnosis of exercise-induced bronchoconstriction
A1 - Goldberg,S.
A1 - Mimouni,F.
A1 - Joseph,L.
A1 - Izbicki,G.
A1 - Picard,E.
Y1 - 2012/09//
N1 - DA - 20121002
IS - 1539-6304 (Electronic)
IS - 1088-5412 (Linking)
LA - eng
PT - Evaluation Studies
PT - Journal Article
SB - IM
KW - Adolescent
KW - Asthma,Exercise-Induced
KW - Diagnosis
KW - Exercise Test

KW - Methods
KW - Female
KW - Humans
KW - Male
KW - Seasons
KW - Sensitivity and Specificity
KW - Young Adult
KW - Reviewer Nominated
RP - NOT IN FILE
SP - 416
EP - 420
JF - Allergy & Asthma Proceedings
JA - Allergy Asthma Proc
VL - 33
IS - 5
N2 - Exercise challenge test (ECT) may help in the diagnosis of asthma. Asthma is season dependent (relative summer nadir).This study was designed to prospectively show that ECT sensitivity decreases when performed in summer and/or out of personal asthma season. We performed two ECTs in 49 patients with suspected asthma, at baseline and at least 6 months later. Test positivity was defined as a >/=10% drop of forced expiratory volume in 1 second after exercise. Patients were defined as having exercise-induced bronchoconstriction (EIB) if at least one of the two tests was positive. We analyzed the impact of season and of patient-reported asthma season on ECT sensitivity. We performed 21 ECTs in the summer and 77 in other seasons. Summer tests were positive in 14.3% of patients versus 33.8% in other seasons (p = 0.11). In patients with confirmed EIB, the sensitivity (95% CI) of a summer test was 0.25 (0.007-0.57), compared with 0.76 (0.58-0.89) in other seasons. Tests performed out of personal asthma season were positive in 10.0% of tests compared with 37.1% of tests during personal asthma season (p = 0.007). In patients with confirmed EIB, the sensitivity (95% CI) of a test performed out of asthma season was 0.27 (0.07-0.61), compared with 0.74 (0.55-0.87) during season. The sensitivity of ECT performed in the summer or outside of the personal asthma season is about one-third of ECT performed not in the summer or during personal asthma season. We suggest performing ECTs during personal asthma season and not in the summer if there is no asthma season known
AD - Pediatric Pulmonology, Shaare Zedek Medical Center, Hebrew University Medical School, Jerusalem, Israel. sgoldberg@szmc.org.il
UR - 23026183
ER -

TY - JOUR
ID - 4430
T1 - A PLAY GROUND FOR THE AGES
A1 - Greco,JoAnn
Y1 - 2006/08//
KW - SportDiscus
KW - *PLAYGROUNDS
KW - *PSYCHOLOGY
KW - *CHILDREN -- Wounds & injuries
KW - *PREVENTION
KW - *CHILDREN
KW - DESIGN & construction
KW - SAFETY measures
KW - AGE groups
KW - SOCIAL skills in children
KW - Socialization
RP - NOT IN FILE
SP - 52
EP - 55
JA - Parks & Recreation
VL - 41
IS - 8

N2 - The article focuses on the design of playgrounds. Playground design is becoming more targeted to appeal to specific age groups. Playground designers now consider the ways children of different ages play before construction. Safety now plays more of a role in construction than innovation within the design. The article notes that playgrounds still need to challenge and entertain children
SN - 00312215
UR -
https://login.proxy.bib.uottawa.ca/login?url=http://search.ebscohost.com/login.aspx?direct=true&db=s3h&AN=21994830&site=ehost-live
ER -

TY - JOUR
ID - 4431
T1 - "D-feat D Flu?" Vitamin D and Pediatric Influenza
A1 - Greenfield,Russell H.
Y1 - 2010/05//
KW - SportDiscus
KW - *INFLUENZA
KW - *ASTHMA in children
KW - *PLACEBOS (Medicine)
KW - *VITAMIN D
KW - *CHILDREN -- Diseases
KW - *CHILD development
RP - NOT IN FILE
SP - 52
EP - 53
JA - Alternative Medicine Alert
VL - 13
IS - 5
N2 - THE AUTHORS OF THIS JAPANESE MULTICENTER (12 HOSPITALS and 8 outside physicians), randomized, double-blind, placebo-controlled, parallel-group trial investigated whether vitamin D supplements might impact the incidence of influenza A in school children during winter and early spring. Children aged 6-15 years (mean age 10.2 years, with 65% having started nursery school or kindergarten at 3 years or older) were enrolled from November to early December in 2008 and then asked to begin taking their study drugs within the first 2 weeks of December. Subjects were randomized to receive daily divided doses of either 1,200 IU of vitamin D3 or placebo from December 2008 through March 2009. Parents were provided with eight numbered bottles, each containing 90 tablets of either 200 IU vitamin D or placebo identical in appearance. The children were to take 3 tablets twice daily. Pre-study questionnaires collected demographic data and medical history, while the post-study questionnaire focused on answers to questions about compliance with study protocol, outdoor activities, specific food intake, and illnesses that may have occurred. Daily logs were also completed. Compliance was measured in part by direct viewing of the supplement bottles on clinic visits. Primary outcome of interest was the incidence of influenza A, diagnosed by outpatient rapid influenza antigen testing using a nasopharyngeal swab specimen. A total of 430 schoolchildren/parent pairs were randomized but there was significant study attrition, with 50 and 46 children being lost to follow-up in the active and placebo groups, respectively (intention-to-treat analysis was used). Compliance with the study medication was very high (96%). Influenza A occurred in 18/167 (10.8%) of children in the vitamin D group compared with 31/167 (18.6%) in the placebo group (relative risk [RR] = 0.58), with peak incidence occurring from middle to late January. Between days 31 and 60, influenza A occurred significantly less often in the vitamin D group than in the placebo group (5.4% vs. 13.2%; RR = 0.41), but there was no difference in the development of influenza A between the two groups early or late in the trial. In subgroup analysis it was shown that a reduction in occurrence in influenza A was more prominent in children who previously had not been taking vitamin D and in those who had started nursery school after age 3 years (starting nursery school before age 3 years may increase the chances of exposure to influenza and of obtaining immunity). In children with a previous history of asthma, exacerbations occurred in 2 children taking vitamin D3 vs. 12 children in the placebo group (RR =0.17). The incidence of influenza B and antigen-testing negative flu-like illness was not different between the two groups. No serious adverse effects were identified, including urinary stones. The authors concluded that vitamin D3 supplementation during the winter and early spring may have a preventive effect against the development of childhood influenza A infection. ABSTRACT FROM AUTHOR

SN - 1096942X
UR -
https://login.proxy.bib.uottawa.ca/login?url=http://search.ebscohost.com/login.aspx?direct=true&db=s3h&AN=50544267&site=ehost-live
ER -

TY - JOUR
ID - 4433
T1 - The What and the Why of Camp Releases
A1 - Gregg,Charles R.
A1 - Hansen-Stamp,Catherine
Y1 - 2007/01//
KW - SportDiscus
KW - *CAMPS
KW - *OUTDOOR recreation
KW - *CAMPING
KW - *CHILDREN -- Health
KW - SAFETY measures
KW - Management
KW - MORAL & ethical aspects
RP - NOT IN FILE
SP - 1
EP - 4
JA - Camping Magazine
VL - 80
IS - 1
N2 - The article focuses on the importance of negligence release forms for summer camps. Release forms are something that most if not all summer campers must sign which release the camp from all responsibility from injuries or traumas related to activities occurring while camping. The traditional responsibilities of camp owners and employees regarding child safety and welfare are explained
SN - 07404131
UR -
https://login.proxy.bib.uottawa.ca/login?url=http://search.ebscohost.com/login.aspx?direct=true&db=s3h&AN=24105158&site=ehost-live
ER -

TY - JOUR
ID - 4434
T1 - The development of KidTest 2002 update: a talent identification inventory for predicting success in sports for children
A1 - Grice,T.
Y1 - 2003///
KW - SportDiscus
KW - *SPORTS
KW - Ability
KW - TESTING
KW - Child
KW - TALENT IDENTIFICATION
KW - instrumentation
KW - PERFORMANCE PREDICTION
RP - NOT IN FILE
SP - 229
EP - 247
JA - Applied Research in Coaching & Athletics Annual
VL - 18
CY - ;

N2 - Historically, very few of the great and talented athletes from the United States are deliberately selected through a structured, scientific system. Most American athletes begin participation in sports because of a role model, either a parent, a coach or a professional athlete. Continued participation seems to depend upon several factors; including, enjoyment of the sport, successful participation and/or peer pressure to name a few. The development of a talent identification process would allow children to experience greater enjoyment and reduce the possibility of selecting a sport in which the child had a very limited potential for success. There are numerous opportunities for children in organized sports, but they would probably enjoy these attempts at structured activity much more if they are successful and are having fun. Therefore, the development of a process to predict success for children in specific sports would seem to be an attractive alternative to selection and participation on a random basis. The purpose of this study was to update KidTest, a talent identification test inventory which provides a subjective evaluation of the potential talent that a child may exhibit for success in specific sports. A total of 173 (75 boys, 98 girls) were randomly selected from grades 6, 7 and 8 from three different schools. Data was collected during physical education classes at A.E. Phillips Laboratory School, Mann Middle School and Vernon Intermediate School. This study replicates an earlier study completed in 1999 on kindergarten through fifth grade students. Nine (9) physical fitness components were administered and the data was collected to develop a working model for identifying talent in specific sports. Using a three-digit composite, norms were established for student subjects, sixth through eighth grade, based on performance on various tests in aerobic power, motor skills and coordination. A predicted Max VO2 score was established by the Houston Non-exercise Test. Percent body fat was done as a means of cross-validation. Results indicated relatively high validity across the three grade levels. An aerobic run was performed with all three-grade levels doing a one-mile walk/run. The motor ability tests consisted of a forty-yard dash measuring speed, a fifteen-yard shuttle run for agility and the standing long jump testing leg power. Hand-eye-foot coordination was measured by a catching test along with throwing and kicking tests for accuracy and distance along. Each of the three composite scores was averaged on a scale from one to five. A legend was developed utilizing the mean and standard deviation to cross reference numerous sports activities with a corresponding three-digit composite score for each sport
UR -
https://login.proxy.bib.uottawa.ca/login?url=http://search.ebscohost.com/login.aspx?direct=true&db=s3h&AN=SPHS-904793&site=ehost-live;http://articles.sirc.ca/search.cfm?id=S-904793
L2 - http://articles.sirc.ca/search.cfm?id=S-904793
ER -

TY - JOUR
ID - 4436
T1 - Interaction between physical environment, social environment, and child characteristics in determining physical activity at child care
A1 - Gubbels,J.S.
A1 - Kremers,S.P.
A1 - Van Kann,D.H.
A1 - Stafleu,A.
A1 - Candel,M.J.
A1 - Dagnelie,P.C.
A1 - Thijs,C.
A1 - de Vries,N.K.
Y1 - 2011/01//
N1 - DA - 20110208
IS - 1930-7810 (Electronic)
IS - 0278-6133 (Linking)
LA - eng
PT - Journal Article
SB - IM
KW - PubMed
KW - Reviewer Nominated
KW - Child Day Care Centers
KW - Child,Preschool
KW - Environment
KW - Female

KW - Humans
KW - Linear Models
KW - Male
KW - Motor Activity
KW - Netherlands
KW - Observation
KW - Social Environment
RP - NOT IN FILE
SP - 84
EP - 90
JF - Health Psychology
JA - Health Psychol
VL - 30
IS - 1
N2 - OBJECTIVE: To investigate the association between the child-care environment and physical activity of 2- and 3-year-olds. Based on an ecological view of environmental influences on health behavior, we hypothesized that the social and physical environment, as well as child characteristics (age and gender), would show independent and interactive effects on children's physical activity intensity. DESIGN: Observations of physical activity intensity were performed among children (N = 175) at 9 Dutch child-care centers. Aspects of the child-care environment were assessed using the validated Environment and Policy Assessment and Observation (EPAO) Instrument. Multilevel linear regression analyses examined the association of environment and child characteristics with children's activity intensity. Moderation was tested by including interaction terms in the analyses, with subsequent post hoc analyses for significant interaction terms. MAIN OUTCOME MEASURE: Observed child physical activity intensity, measured with the Observational System for Recording Physical Activity in Children-Preschool Version. RESULTS: A large proportion of the observed activities were classified as sedentary, while far fewer observations were classified as moderate or vigorous. Activity opportunities in the physical environment (assessed using EPAO) and prompts by staff and peers were significantly and positively related to physical activity intensity, while group size was negatively related to activity intensity. The influence of the physical environment was moderated by social environment (peer group size), while the social environment in turn interacted with child characteristics (age and gender) in determining activity intensity. CONCLUSION: Our findings are in line with the ecological perspective regarding environmental influences on behavior, and stress the importance of incorporating the child-care environment in efforts to prevent childhood overweight and obesity
AD - NUTRIM School for Nutrition, Toxicology and Metabolism, Maastricht University Medical Centre, Department of Health Promotion, Maastricht, the Netherlands. jessica.gubbels@maastrichtuniversity.nl
UR - PM:21133542
ER -

TY - JOUR
ID - 4437
T1 - Normal and abnormal visual field maps in albinos. Central effects of non-matching maps
A1 - Guillery,R.W.
Y1 - 1990/09//
N1 - DA - 19910314
IS - 0167-6784 (Print)
IS - 0167-6784 (Linking)
LA - eng
PT - Journal Article
PT - Review
SB - IM
KW - Albinism
KW - physiopathology
KW - Animals
KW - Auditory Pathways
KW - Geniculate Bodies
KW - Humans
KW - Optic Chiasm

KW - Vision Disorders
KW - visual cortex
KW - Visual Fields
KW - Reviewer Nominated
RP - NOT IN FILE
SP - 177
EP - 183
JA - Ophthalmic Paediatr Genet.
VL - 11
IS - 3
N2 - The abnormal chiasmatic crossing characteristic of all albino mammals brings two discordant representations of the visual field to the central visual relays. The representation from the nasal retina is normal whereas the one from the temporal retina is disrupted, a part representing the contralateral visual field as is normal and a part coming from the ipsilateral visual field as a mirror image of a part of the normal representation. Experiments that were designed to define the rules on the basis of which the abnormal representations can be established in the lateral geniculate nucleus and visual cortex are described. These experiments are related to more recent studies of albino cats, and the observations of the visual pathways are related speculatively to abnormalities seen in the auditory pathways of albinos. The possibility is raised that the auditory abnormalities are secondary to the visual abnormalities, produced by a failure of the two systems to establish normally matching maps of sensory space
AD - Department of Human Anatomy, Oxford
UR - PM:2280975
ER -

TY - JOUR
ID - 4438
T1 - How School Time Physical Activity Is the "Big One" for Daily Activity Among Schoolchildren: A Semi-Experimental Approach
A1 - Guinhouya,Benjamin C.
A1 - Lemdani,Mohamed
A1 - ApActAc,GAcoffroy K.
A1 - Durocher,Alain
A1 - Vilhelm,Christian
A1 - Hubert,HervAc
Y1 - 2009/07//
KW - SportDiscus
KW - *SCHOOL sports
KW - *PHYSICAL fitness -- Research
KW - *HEALTH behavior
KW - *HEALTH self-care
KW - *STUDENTS
KW - *HEALTH
KW - Age Factors
KW - Accelerometry
KW - behavioral science
KW - biostatistics
KW - experimental research
KW - overweight/obesity
KW - Pediatrics
RP - NOT IN FILE
SP - 510
EP - 519
JF - Journal of Physical Activity & Health
JA - J Phys Act Health
VL - 6
IS - 4

N2 - Background: This study was designed to model the relationship between an ActiGraphbased "in-school" physical activity (PA) and the daily one among children and to quantify how school can contribute to the daily PA recommendations. Method: Fifty boys and 43 girls (aged 8 to 11 years) wore ActiGraph for 2 schooldays of no structured PA. The daily moderate-to-vigorous PA (MVPAd) was regressed on the school time MVPA (MVPAs). Then, a ROC analysis was computed to define the required MVPAs. Results: Children spent 57% of their awaking time at school. School time PA opportunities (ie, recesses: ƒ^¬18% of a child's awaking time) accounted for >70% of the MVPAd among children. Then, MVPAd (Y) could be predicted from MVPAs (X) using the equation: Y = 2.06 X0.88; RAý= .889, P < .0001. Although, this model was sex-specifically determined, cross-validations showed valid estimates of MVPAd. Finally, with a sensitivity of 100% and a specificity of 90%, MVPAs, a 34 min.d-1 was required to prompt the daily recommendation. Conclusions: The current study shows the contribution of MVPA at school to recommended activity levels and suggests the value of activity performed during recesses. It also calls for encouraging both home- and community-based interventions, predominantly directed toward girls. ABSTRACT FROM AUTHOR
SN - 15433080
UR -
https://login.proxy.bib.uottawa.ca/login?url=http://search.ebscohost.com/login.aspx?direct=true&db=s3h&AN=43799558&site=ehost-live
ER -

TY - JOUR
ID - 4439
T1 - The recess period: a key moment of prepubescent children's daily physical activity?
A1 - Guinhouya,C.B.
A1 - Hubert,H.
A1 - Dupont,G.
A1 - Durocher,A.
Y1 - 2005/08/01/
N1 - Accession Number: 2009056442. Language: English. Entry Date: 20051209. Revision Date: 20110513. Publication Type: journal article; research; tables/charts. Journal Subset: Blind Peer Reviewed; Editorial Board Reviewed; Expert Peer Reviewed; Health Promotion/Education; Online; Peer Reviewed; USA. No. of Refs: 21 ref. NLM UID: 101083500
KW - CINAHL
KW - Physical Activity
KW - Physical Education and Training
KW - Schools
KW - Accelerometers
KW - Child
KW - Descriptive Statistics
KW - Female
KW - Male
KW - Pearson's Correlation Coefficient
KW - Rural Areas
KW - Time Factors
KW - Human
RP - NOT IN FILE
SP - 1
EP - 9
JA - International Electronic Journal of Health Education
VL - 8
N2 - This study was designed to analyze the significance of the recess period and the effect of its duration on children's daily physical activity. Thirteen pupils attending school in a rural area were monitored with accelerometers during the study weeks. The intervention consisted of modifying regularly scheduled recess period for a month. Data from two school days during the third week were recorded for analyses. Analyses indicated that, according to the recommended 2 OE 15 min recess schedule, children spent on average from 16.9 +/- 5.7 min to 22.8 +/- 3.7 min each day in moderate-to-vigorous physical activity (MVPA) at recess; that is, 16.3% to 20.0% of their daily MVPA (comprised between 103.4 +/- 32.5 min and 113.9 +/- 32.5 min). This level of activity increased with the increased recess duration. A 1.2% increase of the time spent at recess

resulted in a 6.6% increase of their activity (average 23% to 26.5%). It appears that a more appropriate reorganization of school schedule, including recess period and proper physical education classes, may be beneficial for school children to experience adequate amount of physical activity
SN - 1529-1944
AD - University of Lille 2, 42, rue Ambroise ParA", F-59120 Loos
UR - http://search.ebscohost.com/login.aspx?direct=true&db=cin20&AN=2009056442&site=ehost-live
ER -

TY - JOUR
ID - 4441
T1 - Comparison of perceived support for physical activity and physical activity related practices of children and young adolescents in Hong Kong and Australia
A1 - Ha,Amy
A1 - Abbott,Rebecca
A1 - Macdonald,Doune
A1 - Pang,Bonnie
Y1 - 2009/06//
KW - SportDiscus
KW - *PHYSICAL activity
KW - *CHILDREN -- Health
KW - *TEENAGERS -- Health
KW - Comparative Studies
KW - Social Support
KW - HONG Kong (China)
KW - China
KW - Australia
KW - CHILDREN
KW - Hong Kong
KW - Parents
KW - Physical Activity
RP - NOT IN FILE
SP - 155
EP - 173
JA - European Physical Education Review
VL - 15
IS - 2
N2 - This study compared the self-reported physical activity, perceived social support for, and perceived barriers to, physical activity of primary and secondary school children from Hong Kong and Australia. Hong Kong boys and girls reported spending significantly less time, outside of school hours, on physical activity than their Australian contemporaries (p < .05). There was significantly higher perceived support for activity from Australian parents (p < .001), teachers (p < .001) and peers (p < .001) compared to their Hong Kong counterparts. Parental support significantly correlated with self-reported activity in Australia, but not in Hong Kong. These data add support to the notion that strategies to increase physical activity need to embed family and school. ABSTRACT FROM AUTHOR
SN - 1356336X
UR -
https://login.proxy.bib.uottawa.ca/login?url=http://search.ebscohost.com/login.aspx?direct=true&db=s3h&AN=51363765&site=ehost-live
ER -

TY - JOUR
ID - 4443
T1 - Promoting Physical Activity Through Physical Education: Increasing Parental Involvement
A1 - Hager,Lisa
A1 - Beighle,Aaron
Y1 - 2006/01//
KW - SportDiscus

KW - *PHYSICAL education
KW - *PHYSICAL education teachers
KW - *LIFESTYLES
KW - *PHYSICAL fitness
KW - *STUDENTS
KW - *CHARITIES
KW - *SPECIAL events
KW - PARENT-teacher relationships
KW - PARENT & child
KW - CALENDARS
RP - NOT IN FILE
SP - 28
EP - 31
JA - Teaching Elementary Physical Education
VL - 17
IS - 1
N2 - The article provides information on strategies and ideas for physical education teachers to get parents and other significant adults involved in helping to get children physically active as a lifestyle habit. Physical activity calendars may be used, which are easy to create and may provide students with active habits outside physical education. It also serves as a medium for communicating with parents about physical education, physical activity and health. Designing charity events around physical activity is also effective not only in providing an active lifestyle but in raising money for physical education as well. Special events called Fun Days could be established with parent participation to create anticipation at the start of the school year or late in the year to celebrate the end of the great school year that passed
SN - 10454853
UR -
https://login.proxy.bib.uottawa.ca/login?url=http://search.ebscohost.com/login.aspx?direct=true&db=s3h&AN=19505980&site=ehost-live
ER -

TY - JOUR
ID - 4444
T1 - Economic, environmental, and health/well-being benefits associated with green industry products and services: A review
A1 - Hall,Charles R.
A1 - Dickson,Madeline W.
Y1 - 2011///
KW - Reviewer Nominated
RP - NOT IN FILE
SP - 96
JA - Journal of Environmental Horticulture
VL - 29
IS - 2
SN - 0738-2898
ER -

TY - JOUR
ID - 4445
T1 - Tell Your Kids to Take a Hike
A1 - Halvorson,Ryan
Y1 - 2011/06//
KW - SportDiscus
KW - *RECREATION
KW - *WALKING
KW - FAMILIES
KW - Nature
KW - CONTINUING education units

RP - NOT IN FILE
SP - 17
EP - 17
JA - IDEA Fitness Journal
VL - 8
IS - 6
N2 - In this article, outdoor enthusiast Jeff Alt offers tips on the ways to get children and toddlers excited about hiking as a method to combine family time and physical activity
SN - 1548419X
UR -
https://login.proxy.bib.uottawa.ca/login?url=http://search.ebscohost.com/login.aspx?direct=true&db=s3h&AN=61079159&site=ehost-live
ER -

TY - JOUR
ID - 4448
T1 - Rephrase the Question
A1 - Hannan,Maureen
Y1 - 2011/09//
KW - SportDiscus
KW - *PARKS
KW - *RECREATION
KW - *PLAYGROUNDS
KW - INDIANA University
KW - MAINTENANCE & repair
KW - United States
RP - NOT IN FILE
SP - 25
EP - 26
JA - Parks & Recreation
VL - 46
IS - 9
N2 - The article offers news briefs related to parks and recreation of the U.S. as of September 2011. A research conducted by Gary T. Green, associate professor at University of Georgia's WarneU School of Forestry and Natural Resources, concludes that children spend more time outside than thought of. Virginia resident Nicholas Anoia has provided 500 hour services to the city's parks organizations. An Indiana University study reveals that playground surfaces need maintenance after 12 months
SN - 00312215
UR -
https://login.proxy.bib.uottawa.ca/login?url=http://search.ebscohost.com/login.aspx?direct=true&db=s3h&AN=66213174&site=ehost-live
ER -

TY - JOUR
ID - 4449
T1 - Shelter from the Sun
A1 - Hannan,Maureen
Y1 - 2012/04//
KW - SportDiscus
KW - *CHILDREN
KW - *SKIN diseases
KW - *PREVENTION
KW - PLAY environments
KW - DERMATOLOGISTS
KW - SERVICES for
KW - United States
RP - NOT IN FILE

SP - 14
EP - 14
JA - Parks & Recreation
VL - 47
IS - 4
N2 - The article focuses on the efforts of Elizabeth Martin, youth education chair for the American Academy of Dermatology (AAD), and her peers at AAD to volunteer their time to equip schools, parks, and childcare facilities with high-quality shade structures for their outdoor play areas in the U.S. They offer organizations serving children a way to build sun safety into their infrastructure. She outlines the myths about sun safety practices such as the expenses of installing sun shade structures
SN - 00312215
UR -
https://login.proxy.bib.uottawa.ca/login?url=http://search.ebscohost.com/login.aspx?direct=true&db=s3h&AN=74286611&site=ehost-live
ER -

TY - JOUR
ID - 4450
T1 - PHYSICAL EDUCATION IN SCHOOLS: A GLOBAL PERSPECTIVE. / TJELESNI ODGOJ U
• ỹKOLAMA: GLOBALNI PREGLED STANJA
A1 - Hardman,Ken
Y1 - 2008/06//
KW - SportDiscus
KW - *PHYSICAL fitness for children
KW - *HEALTH
KW - *PHYSICAL education
KW - *PHYSICAL education teachers -- Training of
KW - *TEACHERS
KW - *OBESITY -- Prevention
KW - CHILDREN & adults
KW - Research
KW - TRAINING of
KW - TEACHING aids & devices
KW - Facilities
KW - fitness standards
KW - Funding
KW - Obesity
KW - PE curriculum
KW - PE provision
KW - PE teacher education
KW - political agenda
KW - subject status
KW - teaching material
KW - time allocation
RP - NOT IN FILE
SP - 5
EP - 28
JF - Kinesiology
VL - 40
IS - 1
N2 - This article draws from the Second Worldwide Survey of the situation of physical education (PE) in schools. The Survey was undertaken as a contribution to the UN dedicated 2005 Year of Sport and PE and in response to inter-governmental agencies' calls for regular monitoring of developments in school PE in the form of a 'reality check'. The overall purpose of the Survey was to assess the worldwide situation of school PE as well as developments since the Physical Education World Summit held in November 1999 in Berlin for which a multi-method/pluralistic approach was adopted with analysis of a range of sources comprising globally and regionally as well as on-line disseminated questionnaires, national surveys, continental regional and national

PE-related projects, case studies and a comprehensive literature review. The pluralistic methods facilitated data collection on national level policies and practice-related issues in school PE, the PE curriculum, resources (human and material), the PE environment (school subject and PE teacher status; and pathway links to PE activity in out-of-school settings) and 'Best Practice' exemplars. The data generated provide an indication of patterns and trends in school PE in countries and regions across the world. The 'reality check' indicates that positive developments and policy rhetoric are juxtaposed with adverse practice shortcomings. Thus, the overall scenario is one of 'mixed messages' with evidence that national and/or regional governments have committed themselves through legislation to making provision for PE but some have been either slow or reticent in translating this into action through actual implementation and assurance of quality of delivery. Essentially, the situation especially in economically under-developed and developing regions has changed little since the 1999 Berlin Physical Education Summit. Continuing concerns embrace: insufficient curriculum time allocation, perceived inferior subject status, insufficient competent qualified and/or inadequately trained teachers (particularly in primary schools), inadequate provision of facilities and equipment and teaching materials frequently associated with under-funding, large class sizes and funding cuts and, in some countries, inadequate provision or awareness of pathway links to wider community programmes and facilities outside of schools. More generally, there is disquiet over the falling fitness standards of young people, rising levels of obesity amongst children of school age and high youth dropout rates from physical/sporting activity engagement. Whilst some improvements in inclusion (related to gender and disability) policy and practice can be identified since the Berlin Physical Education Summit, barriers to equal provision and access opportunities for all still remain. However, current intergovernmental initiatives (European Parliament's 2007 Resolution on the Role of Sport in Education and UNESCO advocacy action) place PE on the political agenda. With such inter-governmental commitments to policy principles and action advocacy, a secure and sustainable future for PE appears to be realizable. ABSTRACT FROM AUTHOR
SN - 13311441
UR -
https://login.proxy.bib.uottawa.ca/login?url=http://search.ebscohost.com/login.aspx?direct=true&db=s3h&AN=33115394&site=ehost-live
ER -

TY - THES
ID - 4451
T1 - An investigation of the influence of vicarious experience on perceived self-efficacy
A1 - Harrison,M.B.
Y1 - 2004///
N1 - Accession Number: 2009014466. Language: English. Entry Date: 20050909. Revision Date: 20091218. Publication Type: doctoral dissertation; research. Special Interest: Pediatric Care
KW - CINAHL
KW - rock climbing
KW - Self-Efficacy
KW - adolescence
KW - Child
KW - Experimental Studies
KW - Female
KW - Male
KW - Multivariate Analysis of Covariance
KW - Questionnaires
KW - Wilderness Experience
KW - Human
RP - NOT IN FILE
EP - 61
PB - Clemson University
N2 - In the climate of unpredictable availability of health care in the United States, a prevalent discussion is the prevention of disease and illness. Recreation participation is one route to maintaining physical and mental well-being. Perceived self-efficacy is an indicator of maintenance, effort, and performance of various behaviors, including recreational activities. Recreational and therapeutic recreational programming can be structured to enhance participants' efficacy regarding specific recreation activities. This can be done by addressing the four sources of efficacy information (verbal persuasion, vicarious experience, affective state, mastery experiences)

that participants' use to make judgments regarding their ability to perform a particular recreation activity
The purpose of this study was to evaluate the effectiveness of one of the sources of efficacy information, vicarious experience via modeling, in enhancing efficacy beliefs of at-risk youth who are participating in a wilderness therapy activity. This study also investigated the influence of activity specific efficacy perceptions on both self-regulatory self-efficacy and perceived performance in the specific activity
Three groups of youth at-risk participated in three separate rock climbing sessions at the Clemson University Outdoor Laboratory's climbing tower. The participants (N = 38) in this study, both male and female and ranging in age from 6--18, were drawn from a year-round private school for at-risk youth. Upon arrival, each group took a rock climbing and self-regulatory self-efficacy questionnaire. This questionnaire included a previous rock climbing experience measure. Then, prior to rock climbing, one of the groups observed a youth model the rock climbing activity, one of the groups observed an adult model the rock climbing activity, and one of the groups did not observe a model. Directly after this each group took another rock climbing and self-regulatory self-efficacy questionnaire. This questionnaire also included a perceived 'similarity to model' measure. After each participant performed the activity of rock climbing, he or she took a third rock climbing and self-regulatory self-efficacy questionnaire. This questionnaire additionally included a perceived performance measure
Multivariate analysis of covariance indicated that groups who observed a model had significantly higher rock climbing self-efficacy and self-regulatory self-efficacy than the group that did not observe a model. There was no significant finding with regard to influence of previous rock climbing experience, model type, or perceived similarity to model. Significant relationships were found between self-regulatory self-efficacy, rock climbing self-efficacy, and perceived performance
Because the participants did not perceive there to be a difference between the models, these results do not provide us with information regarding the influence of perceptions of similarity to the model. These results do show that groups observing a model demonstrate rock climbing during ground school are significantly more likely to show increase in both rock climbing and self-regulatory self-efficacy than groups who do not observe a demonstration. In light of this research it is apparent that providing a model is an effective tool that can be used to assist participants in achieving the positive outcomes of a rock climbing experience, and potentially those of other outdoor and indoor educational pursuits. Further research regarding self-efficacy may indicate that, when teaching or promoting recreation activities, using a self-efficacy based curriculum provides individuals with the best possible chance of becoming life-long recreators
SN - 978-0-496-06594-3
AV - UMI Order AAI3146221.
M1 - Ph.D.
UR - http://search.ebscohost.com/login.aspx?direct=true&db=cin20&AN=2009014466&site=ehost-live
ER -

TY - CHAP
ID - 4452
T1 - Health benefits of nature experience: Psychological, social and cultural processes
A1 - Hartig,Terry
A1 - van den Berg,Agnes E.
A1 - Hagerhall,Caroline M.
A1 - Tomalak,Marek
A1 - Bauer,Nicole
A1 - Hansmann,Ralf
A1 - Ojala,Ann
A1 - Syngollitou,Efi
A1 - Carrus,Giuseppe
A1 - van Herzele,Ann
Y1 - 2011///
KW - Reviewer Nominated
RP - NOT IN FILE
SP - 127
EP - 168
T2 - Forests, trees and human health
PB - Springer
SN - 9048198054
ER -

TY - JOUR
ID - 4453
T1 - Elementary School Students' Level Of Physical Activity During Recess
A1 - Hasken,Julie
A1 - Temme,Megan
Y1 - 2010/07//
KW - SportDiscus
KW - *SCHOOL children
KW - *PHYSICAL fitness
KW - *PHYSICAL activity
KW - *PHYSICAL education
KW - *SCHOOL recess breaks
KW - *LEISURE
RP - NOT IN FILE
SP - 20
EP - 28
JA - Missouri Journal of Health, Physical Education, Recreation & Dance
VL - 20
N2 - National guidelines recommend accumulation of at least one hour of moderate to vigorous physical activity each day for students. Since school recess provides scheduled periods of time to accumulate some of these much-needed minutes of moderate to vigorous physical activity, the purpose of this study was to determine the proportion of elementary students in a rural, northeast Missouri school district who were physically active at the intensity level recommended by the national guidelines during their recess periods. A modified version of the System for Observing Play and Leisure Activity in Youth (SOPLAY) was used to collect observational data on the number of students, their gender, and their physical activity intensity levels in defined areas of the playground during recess. Overall, the majority of students seemed to meet the guidelines for moderate to vigorous physical activity levels during recess, but the addition of more open play space and more game equipment may help move many from the moderate to more highly active levels. It is recommended that school districts increase the amount of recess time as their school schedules permit to help students meet national guidelines for accumulated physical activity time. ABSTRACT FROM AUTHOR
SN - 10586288
UR -
https://login.proxy.bib.uottawa.ca/login?url=http://search.ebscohost.com/login.aspx?direct=true&db=s3h&AN=88871983&site=ehost-live
ER -

TY - JOUR
ID - 4454
T1 - Family socioeconomic status and various types of fall injuries among young people
A1 - Hasselberg,M.
Y1 - 2010/09/02/
KW - SportDiscus
RP - NOT IN FILE
SP - A209
EP - A210
JF - Injury Prevention
JA - Inj Prev
VL - 16
N2 - Problem and Objective Studies focusing on the socioeconomic distribution of fall injuries among children are few and show mixed results. Previous studies are mainly area-based and no studies have investigated the relationship between family socioeconomic position and subgroups of fall injuries. The current study examines whether socioeconomic position affect the risk of having fall related injuries of different types. Method This population-based cohort study links Swedish national register data for a cohort of 1 544 047 individuals born between 1977 and 1991. Individual records from the 1985 census were linked to the National Hospital Discharge Register for the period 1998ƒ_"2004. Fall injuries requiring hospitalisation were recorded (n=38 529). Logistic regressions were used to measure the effect (odd ratios) of socio-economic position on eight

subgroups of fall injuries (based on external codes, ICD-10). Results An analysis of all types of fall aggregated (W00-W19) showed no significant differences between young people from different socioeconomic groups. However, analyses of subgroups of falls showed that children of manual workers had significant higher odds of having injuries related to falls from playground equipment (OR 1.35 (1.21 to 1.50), stairs (OR 1.48, CI 1.30 to 1.69), trees (1.36 (1.17 to 1.58) and falls from buildings (OR 1.38 CI 1.18 to 1.60). On the other hand, children in lower socioeconomic groups had significantly lower odds for sport related falls compared to children of parents who are intermediate- and high level salaried employees. Conclusion This study indicates that when falls are split into subgroups the effect of low socioeconomic position can be both protective and aggravating. ABSTRACT FROM PUBLISHER
SN - 13538047
UR -
https://login.proxy.bib.uottawa.ca/login?url=http://search.ebscohost.com/login.aspx?direct=true&db=s3h&AN=66043075&site=ehost-live
ER -

TY - JOUR
ID - 4455
T1 - Sports participation and health during periods of educational transition: a study of 30ƒ_"35-year-olds in north-west England
A1 - Haycock,David
A1 - Smith,Andy
Y1 - 2014/02//
KW - SportDiscus
KW - *SPORTS participation
KW - *HEALTH
KW - *LEISURE
KW - *HEALTH behavior
KW - Research
KW - adulthood
KW - SOCIAL classes -- Research
KW - SOCIAL aspects
KW - Adultsƒ_T Health
KW - Education
KW - Friendship
KW - leisure
KW - Life-transitions
KW - sport
KW - youth
RP - NOT IN FILE
SP - 168
EP - 185
JA - Sport, Education & Society
VL - 19
IS - 2
N2 - In view of the persistence of health inequalities and declines in leisure-sport participation over the life course, several quantitative investigations have explored the links between participation and other leisure activities, which have their foundations in childhood and youth, as a means of understanding adultsƒ_T health behaviours. This paper presents new qualitative evidence to examine the largely under-explored relationship between leisure-sport participation and health within the context of educational transitions among a sample of 30ƒ_"35-year-olds in north-west England. Drawing on semi-structured interviews conducted with 19 participants between July and August 2009, the paper illuminates the differential impact that gender and social class had on sports participation, and other health-related behaviours (e.g. drinking and smoking), among different groups of adults during the inherently transitional life-stage of youth. The findings suggest that while significant, the length of time spent in education and the differential educational experiences recalled by adults cannot adequately explain the observed differences in health and leisure-sport participation. The major sources of difference, while associated with educational transitions, appeared to lie instead in the broader inequalities that characterized adultsƒ_T lives and it is argued that simply enhancing leisure-sport participation and

individual lifestyle change, as a means of health promotion, is a futile endeavour that does little to tackle the socio-economic structural determinants of health. We conclude by suggesting that until this is recognized by government and those both inside and outside of the health and sport policy communities, stubborn differences in leisure-sport participation rates and health inequalitiesƒ_"that have their foundations in childhood and youth, but extend over the life courseƒ_"are likely to remain intact, and the unequal lives people currently lead are likely to become even more unequal in the future. ABSTRACT FROM PUBLISHER
SN - 13573322
UR -
https://login.proxy.bib.uottawa.ca/login?url=http://search.ebscohost.com/login.aspx?direct=true&db=s3h&AN=94003687&site=ehost-live
ER -

TY - JOUR
ID - 4456
T1 - How does the concept of Physical Literacy relate to what is and what could be the practice of Physical Education?
A1 - Haydn-Davies,Dominic
Y1 - 2005///Autumn
KW - SportDiscus
KW - *PHYSICAL education & training
KW - *PERFORMANCE
KW - *TEACHING
KW - GOVERNMENT policy
KW - PROGRAMS
KW - Research
KW - POLITICAL science
KW - Schools
KW - Great Britain
KW - LIFETIME SPORT
KW - PERFORMANCE PREDICTION
RP - NOT IN FILE
SP - 45
EP - 48
JA - British Journal of Teaching Physical Education
VL - 36
IS - 3
CY - ;
N2 - This paper investigates the impact of a Physical Education (PE) approach based on the concept of Physical Literacy (PL). Using Whiteheads (2001) definition of PL, the paper looks at how this philosophical viewpoint can be used in a practical PE setting. The Qualifications and Curriculum Authority (QCA, 2002) identification of high quality PE outcomes corresponds, in part, with Whitehead's definition of PL. This study concludes that for PE to be considered 'high quality', it should actively aim to prepare children to participate in lifelong physical activity (LLPA) by focusing on skills and attitudes to be used outside limited curriculum time. Further considerations relate to teacher education with a focus on generic skills and strategies fundamental to learning in PE, rather than activity specific content. [ABSTRACT FROM AUTHOR]
SN - 14724375
UR -
https://login.proxy.bib.uottawa.ca/login?url=http://search.ebscohost.com/login.aspx?direct=true&db=s3h&AN=SPHS-1025392&site=ehost-live;http://articles.sirc.ca/search.cfm?id=S-1025392;http://www.pea.uk.com
L2 - http://articles.sirc.ca/search.cfm?id=S-1025392
ER -

TY - JOUR
ID - 4457
T1 - Playing It Too Safe?
A1 - Hayward,Philip
Y1 - 2012/08//

KW - SportDiscus
KW - *PLAYGROUNDS
KW - *CHILD development
KW - SAFETY measures
RP - NOT IN FILE
SP - 6
EP - 6
JA - Parks & Recreation
VL - 47
IS - 8
N2 - An introduction is presented in which the editor discusses an article within the issue related to the adverse impact of advances in playground design and safety on the emotional development of children
SN - 00312215
UR - https://login.proxy.bib.uottawa.ca/login?url=http://search.ebscohost.com/login.aspx?direct=true&db=s3h&AN=79113126&site=ehost-live
ER -

TY - JOUR
ID - 4459
T1 - Screen-related sedentary behaviours of school-aged children: Principalsƒ_T and teachersƒ_T perspectives
A1 - He,Meizi
A1 - PichAc,Leonard
A1 - Beynon,Charlene
A1 - Kurtz,Joanne
A1 - Harris,Stewart
Y1 - 2011/03//
KW - SportDiscus
KW - *OBESITY -- Prevention
KW - *HEALTH education
KW - *PHYSICAL education
KW - *TEACHERS
KW - *SEDENTARY lifestyles
KW - *CHILDREN
KW - INTERVIEWING
KW - RESEARCH -- Methodology
KW - RESEARCH -- Finance
KW - School Administrators
KW - Attitudes
KW - Television
KW - USER interfaces (Computer systems)
KW - Qualitative Research
KW - HUMAN services programs
KW - DATA analysis -- Software
KW - Canada
KW - CHILDREN
KW - Physical Activity
KW - school personnel
KW - sedentary behaviour
RP - NOT IN FILE
SP - 32
EP - 38
JF - Health Education Journal
VL - 70
IS - 1
N2 - Objective: To solicit school principalsƒ_T and teachersƒ_T perspectives on childrenƒ_Ts screen-related sedentary behaviour and to identify possible solutions to reduce sedentary behaviours among school-aged

children.Method: In-person interviews using a semi-structured interview guide were conducted with school principals and grades five and six classroom teachers in 14 randomly selected elementary schools in London and Middlesex County, Ontario. Fourteen principals and 39 classroom teachers participated in the study. Inductive content analysis was performed independently by two researchers.Results: Both principals and teachers were very concerned about childrenƒ_Ts excessive screen viewing activities, but they did not perceive that they could play a key role in reducing these behaviours. Key barriers were identified to reducing screen-related sedentary behaviour and to childrenƒ_Ts active living both at and away from school. They included competing demands from other subjects, limited gym resources/space within the school, a lack of control over the home environment, and a perception that parents were poor role models. Notwithstanding the above barriers, principals and teachers still recommended increasing childrenƒ_Ts daily physical activity both within and outside of school hours. Furthermore, they stressed the need for parents to play a key role in reducing their childrenƒ_Ts screen-related sedentary behaviours and increasing their level of physical activity.Conclusion: School principals and teachers were very concerned about excessive screen-related behaviour among school-aged children when away from school and suggested that interventions should emphasize increasing daily physical education, promoting recreational sports at or away from school, and engaging parents in regulating screen time at home. ABSTRACT FROM PUBLISHER
SN - 00178969
UR -
https://login.proxy.bib.uottawa.ca/login?url=http://search.ebscohost.com/login.aspx?direct=true&db=s3h&AN=66966300&site=ehost-live
ER -

TY - JOUR
ID - 4460
T1 - Parents and Toddler Outdoor Play
A1 - Healey,Ian
Y1 - 2013///Winter
KW - SportDiscus
KW - CHILDREN & the environment
KW - PARENT & child
RP - NOT IN FILE
SP - 30
EP - 33
JA - Horizons (14620677)
IS - 64
N2 - The author reflects on the significance of the parent and toddler group Forest Tots established by Rachel Healey, and ponders on the influence of the natural environment exposure to the adults and their children
SN - 14620677
UR -
https://login.proxy.bib.uottawa.ca/login?url=http://search.ebscohost.com/login.aspx?direct=true&db=s3h&AN=93312892&site=ehost-live
ER -

TY - JOUR
ID - 4463
T1 - Philosopher in the Playground: Notes on the Meaning of Sport
A1 - Heinegg,P.
Y1 - 1985///
KW - SportDiscus
KW - *SPORTS
KW - *SPORTS spectators
KW - Theory
KW - Philosophy
KW - COLLECTIVE BEHAVIOUR
KW - PROFESSIONAL
RP - NOT IN FILE

N2 - Argues that there must be a philosophical explanation for the current mass fascination with spectator sports, although this interest may be written off as organized childishness or a quest for large amounts of money. Compares athletes to artists, concluding that sport is essentially a mode of escape from reality. Offers support for this conclusion from a philosophical point of view
UR -
https://login.proxy.bib.uottawa.ca/login?url=http://search.ebscohost.com/login.aspx?direct=true&db=s3h&AN=SPH335304&site=ehost-live;http://articles.sirc.ca/search.cfm?id=335304
L2 - http://articles.sirc.ca/search.cfm?id=335304
ER -

TY - JOUR
ID - 4465
T1 - Response to Letter to the Editor
A1 - Hemond,Harry
A1 - Solo-Gabriele,Helena
Y1 - 2005/02//
KW - SportDiscus
KW - LETTERS to the editor
KW - arsenic
RP - NOT IN FILE
SP - 7
EP - 11
JA - Risk Analysis: An International Journal
VL - 25
IS - 1
N2 - Presents a response by Harry Hemond and Helena Solo-Gabriele to a letter to the editor about their article "Children's Exposure to Arsenic From CCA-Treated Wooden Decks and Playground Structures," in the 2004 issue
SN - 02724332
UR -
https://login.proxy.bib.uottawa.ca/login?url=http://search.ebscohost.com/login.aspx?direct=true&db=s3h&AN=16454749&site=ehost-live
ER -

TY - JOUR
ID - 4466
T1 - Observing Outcomes in Youth Development: An Analysis of Mixed Methods
A1 - Henderson,Karla A.
A1 - Powell,Gwynn M.
A1 - Scanlin,Margery M.
Y1 - 2005///Winter
KW - SportDiscus
KW - *CAMPS
KW - *OUTDOOR recreation
KW - *TEENAGERS
KW - *CAMP sites,facilities,etc.
KW - *CAMPING
KW - YOUTH development
KW - camps
KW - mixed methods
KW - positive youth development
KW - qualitative
KW - Quantitative
RP - NOT IN FILE
SP - 58
EP - 77
JA - Journal of Park & Recreation Administration

VL - 23
IS - 4
N2 - Organizations such as park and recreation departments, not-for-profit youth groups, churches, and resident and day camps offer opportunities to promote positive youth development through structured recreation activities. Researchers involved with organized camp programs have documented some values of camp experiences relative to growth and development, yet more is to be learned about how camp structures and settings influence positive youth development and how the outcomes of youth development can be measured. The purpose of this analysis was to explore indicators of youth development outcomes through a comparison of two forms of data collected at six camps. The results of comparing quantitative and qualitative data sources to determine if indicators of positive youth development can be observed in a short period of time in the recreational and educational setting of summer camp is described. Quantitative data came from a study that sampled families representing American Camp Association (ACA) accredited camps from across the United States. The data included pre- and post-questionnaires given to campers to measure domains such as positive identity, social skills, positive values, and thinking and physical skills. The instrument used was called the Camper Growth Index-Children (CGI-C). Qualitative data came from on-site observations in six camps that had participated in the quantitative study the prior year. A guided outline was used as the basis for field observations and informal interviews. The observation rankings/ratings and the quantitative results were compared. Two of the three camps showing statistically positive developmental change in campers also were ranked higher in using the focused qualitative observation. Two of the three camps showing no statistically significant change were also identified as ranking lower through the observation process. Observing opportunities for adventure and exploration activities as well as leadership and independence displayed by campers were easiest to observe. Observing indicators for developmental outcomes, including environmental awareness, positive identity, and positive values, were more difficult. Although consistent agreement did not exist in this comparison of quantitative and qualitative data, the two approaches provided some convergence and complementary data. This study provided an opportunity to explore the measurement of camp experiences from an external view along with an internal self-report approach. We examined the micro data obtained from the individual campers in relation to the social environmental macro structure of the camp. Implications exist for triangulating data and validating methods in other recreation organizations to better understand how and why youth development programs in recreation and camp settings work. Given the complexity of the desired outcomes and the individual nature of growth and development, mixed methods and systematic multimeasure approaches offer information for supporting youth development. ABSTRACT FROM AUTHOR
SN - 07351968
UR -
https://login.proxy.bib.uottawa.ca/login?url=http://search.ebscohost.com/login.aspx?direct=true&db=s3h&AN=19626748&site=ehost-live
ER -

TY - THES
ID - 4467
T1 - The influence of elementary-school programs on childhood obesity
A1 - Henry,L.L.
Y1 - 2006///
N1 - Accession Number: 2009711527. Language: English. Entry Date: 20071123. Revision Date: 20131213. Publication Type: doctoral dissertation; research. Special Interest: Pediatric Care
KW - CINAHL
KW - Schools,Elementary
KW - Students,Elementary
KW - Pediatric Obesity -- Epidemiology
KW - Academic Performance
KW - Child
KW - Epidemiological Research
KW - Ethnic Groups
KW - Food Services
KW - Learning
KW - Obesity -- Complications
KW - Physical Activity
KW - Physical Education and Training

KW - school health education
KW - United States
KW - Human
RP - NOT IN FILE
EP - 148
PB - George Mason University
N2 - This study describes the dramatic rise in childhood obesity within the United States and explores the influence of elementary-school programs on the number of overweight children or on those at risk for overweight status. The relationship between the prevalence of overweight status in children at the elementary-school level and the amount of time these students spend in physical activity and education related to a healthy lifestyle during the school day, available playground equipment, and school lunches is explored. This study also examines the relationship between overweight status in kindergarten and third-grade students and their academic achievement. The findings indicate that school lunches, the ethnic makeup of schools, and the percentage of students participating in the free or reduced lunch program are all associated with incidence rates of overweight children within these age-groups. The study also found that school playgrounds and nutrition-and-health instruction are often inadequate. An association was also found between overweight status in elementary-school students and low performance on the standardized test determining the readiness to learn and acquired learning for kindergarten and third-grade children
SN - 0-542-55599-9
AV - UMI Order AAI3206043.
M1 - Ph.D.
UR - http://search.ebscohost.com/login.aspx?direct=true&db=cin20&AN=2009711527&site=ehost-live
ER -

TY - JOUR
ID - 4468
T1 - Outdoor play spaces in Canada: the safety dance of standards as policy
A1 - Herrington,S.
A1 - Nicholls,J.
Y1 - 2007/02//
N1 - Accession Number: 2009524804. Language: English. Entry Date: 20071102. Publication Type: journal article; pictorial; review. Journal Subset: Biomedical; Blind Peer Reviewed; Europe; Online/Print; Peer Reviewed; UK & Ireland. Special Interest: Pediatric Care. No. of Refs: 18 ref. Email: susan.herrington@ubc.ca
KW - CINAHL
KW - Architecture
KW - Child Day Care
KW - Child Safety -- Standards
KW - Play and Playthings -- Standards
KW - Public Policy
KW - Canada
KW - Child
KW - Child Development
KW - Child,Preschool
KW - Construction Materials
KW - Equipment Design
KW - Equipment Safety
KW - Government Agencies
KW - Infant
RP - NOT IN FILE
SP - 128
EP - 138
JA - Critical Social Policy
VL - 27
IS - 1
N2 - Over the past decade the outdoor play spaces designed for children in Canada have been largely shaped by fear and profit, rather than by what we know about children's play and development. Since the early 1980s the Canadian Standards Association (CSA) has played an increasingly important role in this transformation as their

technical standards for children's outdoor play spaces have been gradually adapted as policy by local and regional agencies. While the CSA has historically regulated industrial and commercial projects that enable international trade and harmonization with countries such as the United States, their extension of influence to early childhood is problematic; particularly when applied to childcare centres. The following describes some of the issues that arise from the use of safety standards as policy, and the problems these standards cause when applied to childcare centres
SN - 0261-0183
AD - University of British Columbia, 2357 Main Mall Vancouver, British Columbia V6T 1Z4, Canada
UR - http://search.ebscohost.com/login.aspx?direct=true&db=cin20&AN=2009524804&site=ehost-live
ER -

TY - JOUR
ID - 4469
T1 - No play, no gain, school officials fear
A1 - Higgins,Lori
Y1 - 2008///Fall
KW - SportDiscus
KW - *SCHOOL recess breaks
KW - *SCHOOLS
KW - United States
RP - NOT IN FILE
SP - 36
EP - 36
JA - MAHPERD Journal
N2 - The article focuses on the issue on recess gap in various schools in the U.S. According to Patte Barth, director of the Center for Public Education in Alexandria, Virginia, children who attend the highest poverty schools are the least likely to get recess at all. To solve this problem, Center officials addressed the issue in a conference call with the National School Boards Association (NSBA)
UR -
https://login.proxy.bib.uottawa.ca/login?url=http://search.ebscohost.com/login.aspx?direct=true&db=s3h&AN=35588683&site=ehost-live
ER -

TY - JOUR
ID - 4470
T1 - Normal QT Response During Exercise Testing and Hyperventilation in Children
A1 - Hill,James
A1 - O'Riordan,Mary Ann
A1 - Bansal,Manish
A1 - Fiutem,Justin
A1 - Zahka,Kenneth
Y1 - 2012/02//
KW - SportDiscus
KW - *PHYSICAL activity
KW - *HYPERVENTILATION
KW - *HEART beat
KW - *HEART diseases
KW - *PEDIATRICS
KW - *CHILDREN -- Diseases
KW - Databases
KW - Exercise
KW - hyperventilation
KW - Pediatric
KW - QT
RP - NOT IN FILE
SP - 65
EP - 75

JA - Journal of Exercise Physiology Online
VL - 15
IS - 1
N2 - Our goal was to describe normal heart rate (HR), QT interval, and Bazett's-corrected QT (QTc) values during exercise testing in children, and to test our hypothesis that hyperventilation is associated with QTc prolongation in children. This study was a retrospective review of 200 consecutive normal exercise tests in 108 males and 92 females with low likelihood of Long QT Syndromes (LQTS) and no evidence of cardiac disease, with mean age 14.7 Añ4.0 yrs. The QT interval and RR interval were measured throughout exercise testing and hyperventilation. The QTc values were calculated using Bazett's formula. A database of HR, QT, and QTc values is presented for standing baseline and hyperventilation, and throughout exercise and recovery. Heart rate and stage of testing had independent effects on repolarization throughout exercise and recovery, while age and sex did not. We constructed reference tables of mean QT and QTc values during exercise and recovery, referenced by HR and stage. With hyperventilation, mean HR increased by 22.7 Añ 12.8 beatsƒ<.min-1, QT shortened by 26.3 Añ21.6 ms, and mean QTc lengthened by 30.3 Añ25.4 ms 1/2 to 442 Añ26 ms1/2 (P<0.001). There were no significant sex, age, or HR effects on the magnitude of QTc prolongation with hyperventilation. This study provides the largest data set for repolarization behavior during exercise testing in children without evidence of heart disease. This is also the first time that the QTc has been shown to prolong with hyperventilation. It is unknown whether this is specifically related to hyperventilation or simply explained by a limitation in Bazett's formula. It highlights exercising caution when using Bazett's formula outside the narrow reliable HR range, especially in children and even more so during exercise. Database of normal population data is perhaps more reliable in those situations, although further validation must be done to confirm its utility. ABSTRACT FROM AUTHOR
SN - 10979751
UR - https://login.proxy.bib.uottawa.ca/login?url=http://search.ebscohost.com/login.aspx?direct=true&db=s3h&AN=74536774&site=ehost-live
ER -

TY - JOUR
ID - 4472
T1 - Free-Play Behaviors and Participation in Structured, Competitive Sports: A Study of the Relationship
A1 - Holt-Hale,S.A.
Y1 - 1984///
KW - SportDiscus
KW - *PLAY
KW - *SPORTS
KW - age
KW - teenagers
KW - Child
KW - Personality
RP - NOT IN FILE
SP - 22
JA - Unpublished Paper
N2 - Provided information on the free-play behaviors of children ages 6-8 and defined the relationship between participation in competitive sports and free-play behaviors. Focused on a comparison of children's behaviors in structured, competitive sports and those not in sports in relation to: social grouping, classification of play, and characteristics of playfulness. Observations of the subjects' (n=84) play behaviors were recorded during scheduled recess periods. An instrument was designed in order to code the behavior. Concludes that the free play behaviors of children in structured, competitive sports differ from those of children not in sport. Children in sport participated more in group activities, were more often involved in playing-at-a-game, and displayed fewer total expressions of playfulness
SN - 01959131
UR - https://login.proxy.bib.uottawa.ca/login?url=http://search.ebscohost.com/login.aspx?direct=true&db=s3h&AN=SPH338720&site=ehost-live;http://articles.sirc.ca/search.cfm?id=338720
L2 - http://articles.sirc.ca/search.cfm?id=338720
ER -

TY - JOUR
ID - 4473
T1 - Prevalence of low physical activity level among preschool children. / PrevalA ¦ncia de baixo nivel de atividade fA-sica em crianA   as prAc-escolares
A1 - Honda Barros,Simone Storino
A1 - da Silva Lopes,Adair
A1 - Gomes de Barros,Mauro Virgilio
Y1 - 2012/07//
KW - SportDiscus
KW - *EPIDEMIOLOGY
KW - *PHYSICAL activity
KW - *CHILDREN
KW - Confidence Intervals
KW - INTERVIEWING
KW - SAMPLING (Statistics)
KW - Statistics
KW - logistic regression analysis
KW - data analysis
KW - CROSS-sectional method
KW - RESEARCH methodology evaluation
KW - DATA analysis -- Software
KW - Descriptive Statistics
KW - Brazil
KW - Epidemiology
KW - Motor Activity
KW - Preschool
KW - Atividade motora
KW - Epidemiologia
KW - PrAc-escolar
RP - NOT IN FILE
SP - 390
EP - 400
JA - Brazilian Journal of Kineanthropometry & Human Performance
VL - 14
IS - 4
N2 - Physical activity (PA) in children has a decisive role in motor development and prevention of childhood obesity. The available evidence suggests that there is high prevalence of low levels of PA in children, but little is known about the level of PA in preschool children. The objective of this study was to identify the prevalence and the factors associated with low levels of PA in preschool children. This was a cross-sectional study performed in private schools in the municipality of Olinda (state of Pernambuco), with data collection through parent's face-to-face interviews. The study included 265 children (54.3% girls) with mean age of 4.9 years (SD=0.8). Children who did not perform at least 60 minutes/day of outdoors physical activities were considered exposed to low levels of PA. Data analysis was performed by logistic regression considering low level of PA as the outcome. The results showed that 65.3% (95%CI: 9.4-70.8) of children were classified as exposed to 'low level of PA'. Analysis showed that higher parental education (OR=2.41; 95%CI: 1.13-5.10), lack of space for playing at home (OR=2.36; 95%CI: 1.17- 4.78), and attending school in the afternoon (OR=2.92, 95%CI 1.55-5.49) or full-time (OR=57.1, 95%CI 6.57-496.2) were associated with low levels of PA. Preschoolers from families with higher number of children had lower likelihood of low level of PA (OR=0.49; 95%CI 0.26-0.93). It can be concluded that the proportion of children exposed to low levels of PA is high compared to the results of similar studies and that parental and environmental factors are associated with physical activity level in preschool-aged children. ABSTRACT FROM AUTHOR
SN - 14158426
UR - https://login.proxy.bib.uottawa.ca/login?url=http://search.ebscohost.com/login.aspx?direct=true&db=s3h&AN=91924872&site=ehost-live
ER -

TY - CHAP
ID - 4474
T1 - Facilitating Change Through Leisure: The Leisure and Well-Being Model of Therapeutic Recreation Practice
A1 - Hood,Colleen D.
A1 - Carruthers,Cynthia P.
Y1 - 2013///
KW - Reviewer Nominated
RP - NOT IN FILE
SP - 121
EP - 140
T2 - Positive Leisure Science
PB - Springer
SN - 9400750579
ER -

TY - JOUR
ID - 4475
T1 - Bringing Family Back to the Park
A1 - Hornig,Eric F.
Y1 - 2005/07//
KW - SportDiscus
KW - *FAMILY recreation
KW - *EXERCISE
KW - *PHYSICAL fitness
KW - *RECREATION
KW - FAMILIES
KW - United States
RP - NOT IN FILE
SP - 46
EP - 50
JA - Parks & Recreation
VL - 40
IS - 7
N2 - The article focuses on family recreation. One of the key elements required for family development is simply time spent together. It is no secret that an activity promotes physical and mental health. Recent trends toward reducing obesity in children in the U.S. and even legislative efforts to promote physical activity such as the Get Outdoors Act, clearly indicate that recreation improves individual health which is the basic platform for improved family health. Communication is the key to many different forms of success, and relationships that help each other communicate more effectively are essential to family function. Communication and interaction between siblings and parents are no exception. Recreation environments provide a neutral, safe and fun arena for these interactions to occur. Family recreation, in general, is families participating in leisure activities together. To be successful, families must implement the physical, social and emotional elements into their lives. Physical elements are the most commonly recognized forms of recreation involving gross and fine motor skills which need development and conditioning at all ages
SN - 00312215
UR - https://login.proxy.bib.uottawa.ca/login?url=http://search.ebscohost.com/login.aspx?direct=true&db=s3h&AN=17651571&site=ehost-live
ER -

TY - JOUR
ID - 4476
T1 - The effects of the environment on physical activity patterns of children with mental retardation. / Effets de l ' environnement sur l ' activite physique pratiquee par des enfants deficients mentaux
A1 - Horvat,M.

A1 - Franklin,C.
Y1 - 2001/06//
KW - SportDiscus
KW - *MENTAL retardation
KW - *PLAY
KW - *CARDIOVASCULAR fitness
KW - *PARTICIPATION
KW - *RECESSES
KW - EXAMPLE
KW - Evaluation
KW - Schools
KW - Child
KW - PHYSICAL FITNESS-General
KW - ACTIVITE-PHYSIQUE
KW - PRATIQUE-PHYSIQUE
KW - HANDICAPE
KW - ENFANT
KW - DEFICIENT-MENTAL
RP - NOT IN FILE
SP - 189
EP - 198
JF - Research Quarterly for Exercise & Sport
JA - Res Q Exerc Sport
VL - 72
IS - 2
CY - ;
N2 - La pratique d ' une activite physique pour des enfants deficients mentaux varie selon leur lieu de scolarisation et les methodes pedagogiques employees
SN - 02701367
UR - https://login.proxy.bib.uottawa.ca/login?url=http://search.ebscohost.com/login.aspx?direct=true&db=s3h&AN=SPHS-780376&site=ehost-live;http://articles.sirc.ca/search.cfm?id=S-780376;http://www.aahperd.org/
L2 - http://articles.sirc.ca/search.cfm?id=S-780376
ER -

TY - JOUR
ID - 4478
T1 - Meaning in Nature: Meaning in Life as a Mediator of the Relationship Between Nature Connectedness and Well-Being
A1 - Howell,Andrew J.
A1 - Passmore,Holli Anne
A1 - Buro,Karen
KW - Reviewer Nominated
RP - NOT IN FILE
SP - 1
EP - 16
JA - Journal of Happiness Studies
PB - Springer
SN - 1389-4978
ER -

TY - JOUR
ID - 4479
T1 - Nature connectedness: Associations with well-being and mindfulness
A1 - Howell,Andrew J.
A1 - Dopko,Raelyne L.
A1 - Passmore,Holli Anne

A1 - Buro,Karen
Y1 - 2011///
KW - Reviewer Nominated
RP - NOT IN FILE
SP - 166
EP - 171
JF - Personality and Individual Differences
VL - 51
IS - 2
PB - Elsevier
SN - 0191-8869
ER -

TY - CHAP
ID - 4480
T1 - The Nature of Happiness: Nature Affiliation and Mental Well-Being
A1 - Howell,Andrew J.
A1 - Passmore,Holli Anne
Y1 - 2013///
KW - Reviewer Nominated
RP - NOT IN FILE
SP - 231
EP - 257
T2 - Mental Well-Being
PB - Springer
SN - 940075194X
ER -

TY - JOUR
ID - 4487
T1 - Play it safe: steps to keeping playground surfacing safe for our children. Findings from several studies help to mold a standard for safe playground surfaces. Does your neighborhood playground measure up?
A1 - Hudson,S.D.
A1 - Mack,M.G.
A1 - Thompson,D.
Y1 - 2000/04//
KW - SportDiscus
KW - *SAFETY
KW - *PLAYGROUNDS
KW - *DISEASES -- Causes & theories of causation
KW - *WOUNDS & injuries
KW - standards
KW - SURFACES
RP - NOT IN FILE
SP - 78
EP - 86
JA - Parks & Recreation
VL - 35
IS - 4
CY - ;
SN - 00312215
UR -
https://login.proxy.bib.uottawa.ca/login?url=http://search.ebscohost.com/login.aspx?direct=true&db=s3h&AN=SPHS-168406&site=ehost-live;http://articles.sirc.ca/search.cfm?id=S-168406;http://www.nrpa.org
L2 - http://articles.sirc.ca/search.cfm?id=S-168406
ER -

TY - JOUR
ID - 4488
T1 - Signs of the times. The proper use of signs is one way to control public playgrounds and demonstrate a commitment to a safe play environment for all children
A1 - Hudson,S.D.
A1 - Thompson,D.
Y1 - 2002/04//
KW - SportDiscus
KW - *PLAYGROUNDS
KW - *SAFETY
KW - *WOUNDS & injuries
KW - *PREVENTION
KW - SIGNS & symbols
RP - NOT IN FILE
SP - 60
EP - 65
JA - Parks & Recreation
VL - 37
IS - 4
CY - ;
N2 - Playground injuries occur when children use equipment that's inappropriate for their development or when they lack proper supervision. Signs may help to raise awareness of safe playground practices
SN - 00312215
UR -
https://login.proxy.bib.uottawa.ca/login?url=http://search.ebscohost.com/login.aspx?direct=true&db=s3h&AN=SPHS-820968&site=ehost-live;http://articles.sirc.ca/search.cfm?id=S-820968;http://www.nrpa.org
L2 - http://articles.sirc.ca/search.cfm?id=S-820968
ER -

TY - JOUR
ID - 4489
T1 - Are we safe yet? A twenty-five year look at playground safety
A1 - Hudson,Susan D.
A1 - Thompson,Donna
Y1 - 1997/10//
KW - SportDiscus
KW - *PLAYGROUNDS
KW - SAFETY measures
RP - NOT IN FILE
SP - 32
JA - JOPERD: The Journal of Physical Education, Recreation & Dance
VL - 68
IS - 8
N2 - Questions the safety of playground equipment while highlighting the number of injuries children sustain from them. Reference to the creation of a `sand box' at the Boston Parameter Church; Number of playgrounds that can be found in the United States; Reference to the United States Consumer Product Safety Commission; Estimated number of hospital emergency treatment for injuries related to playground equipment; Information on playground safety issues
SN - 07303084
UR -
https://login.proxy.bib.uottawa.ca/login?url=http://search.ebscohost.com/login.aspx?direct=true&db=s3h&AN=9711234981&site=ehost-live
ER -

TY - JOUR
ID - 4490
T1 - SIGNS OF THE TIMES

A1 - Hudson,Susan D.
A1 - Thompson,Donna
Y1 - 2002/04//
KW - SportDiscus
KW - *PLAYGROUNDS
KW - SAFETY measures
KW - 3M Co.
KW - SIGNS & signboards
KW - United States
RP - NOT IN FILE
SP - 60
JA - Parks & Recreation
VL - 37
IS - 4
N2 - Talks about the collaboration of the National Program for Playground Safety, 3M Corp, Nexcare and First Aid products, to develop playground signs to address playground safety issues in the U.S. Percentage of public playgrounds which had any type of signage concerning equipment use; Percentage of the time that children were on play on structures when no adults were present; Description of the signages
SN - 00312215
UR - https://login.proxy.bib.uottawa.ca/login?url=http://search.ebscohost.com/login.aspx?direct=true&db=s3h&AN=6725065&site=ehost-live
ER -

TY - JOUR
ID - 4491
T1 - How Safe Are Our Playgrounds?
A1 - Hudson,Susan D.
A1 - Thompson,Donna
A1 - Olsen,Heather
Y1 - 2004/04//
KW - SportDiscus
KW - *PLAYGROUNDS
KW - *EQUIPMENT & supplies
KW - *RECREATION areas
KW - PLAY environments
KW - SAFETY measures
KW - ASSOCIATIONS,institutions,etc.
KW - CHILDREN & the environment
RP - NOT IN FILE
SP - 53
EP - 59
JA - Parks & Recreation
VL - 39
IS - 4
N2 - Discusses issues concerning safety in the play environments. Areas of safety in the play environment identified by the National Program for Playground Safety; Crucial element to having a safe play environment for children; Factors that determine proper surfacing under and around playground equipment
SN - 00312215
UR - https://login.proxy.bib.uottawa.ca/login?url=http://search.ebscohost.com/login.aspx?direct=true&db=s3h&AN=12860742&site=ehost-live
ER -

TY - JOUR
ID - 4492
T1 - Building Your Field of Dreams

A1 - Hudson,Susan D.
Y1 - 2006/01//
KW - SportDiscus
KW - *SCHOOL children
KW - *RECREATION areas
KW - *CHILD development
KW - PLAY environments
KW - School Environment
KW - SCHOOL boards
KW - BUILDINGS -- Repair & reconstruction
KW - SCHOOL administration
KW - United States
RP - NOT IN FILE
SP - 4
EP - 10
JA - JOPERD: The Journal of Physical Education, Recreation & Dance
VL - 77
IS - 1
N2 - The article discusses the importance of proper outdoor play environment for school children. The author feels that the professionals of the American Alliance for Health, Physical Education, Recreation and Dance (AAHPERD) have failed to impress upon other educators and the general public the importance of creating meaningful play environments for school children. The professionals in these fields are least bothered and have allowed people, who have little knowledge about the importance of play in children's lives, plan the environments where children spend up to one-sixth of their time in school. While school boards allocate millions of dollars to build the inside infrastructure, less is done for outside environments. The results are that outside play environments do little to promote the physical, emotional, social, or intellectual skills and abilities of children. The author feels that the AAHPERD professionals need to get involved in the planning process of schools for promoting the well-being of children. This involves seeking ways to get on the planning committee for any new school or the renovation of older schools
SN - 07303084
UR -
https://login.proxy.bib.uottawa.ca/login?url=http://search.ebscohost.com/login.aspx?direct=true&db=s3h&AN=19510587&site=ehost-live
ER -

TY - JOUR
ID - 4493
T1 - Reported health, physical activity and body image in 11 to 17 year old rural teenagers
A1 - Huk-Wieliczuk,E.
Y1 - 2001///
KW - SportDiscus
KW - *PHYSICAL fitness
KW - *BODY image
KW - *STRESS (Physiology)
KW - *CARDIOVASCULAR fitness
KW - *HEALTH education
KW - teenagers
KW - Rural Population
KW - Poland
RP - NOT IN FILE
SP - 24
EP - 34
JA - Acta Kinesiologiae Universitatis Tartuensis
VL - 6
CY - ;
N2 - In a group of 11-, 13-, 15-, and 17-years-old rural pupils (N = 780), the perception of their health, body image and physical activity in leisure time was studied. The survey was based on the Health Behavior in

School-Aged Children (HBSC) a WHO Cross-National Study. The majority (84.7 %) of subjects considered themselves as 'healthy', or 'very healthy'; chronic diseases were found in 11 % of rural adolescents. Many adolescents have common ailments and emotional states. The most frequent were nervousness (39 %), irritability or bad mood (29 %) and headache (18.8 %). The majority of rural youths studied (77 %) reported their fitness as 'very good' or 'good'. Every fourth boy and every second girl do not intense exercise enough, and exercise too seldom every week outside school hours. Due to the discontent with their appearance, 4.7 % of boys and 14.8 % of girls used slimming diets, and another 9.7 % of girls and 26 % of boys thought they ought to go on a diet to lose weight. The results of this study can be useful for planning health care and health education for rural adolescents
SN - 14069822
UR -
https://login.proxy.bib.uottawa.ca/login?url=http://search.ebscohost.com/login.aspx?direct=true&db=s3h&AN=SPHS-803389&site=ehost-live;http://articles.sirc.ca/search.cfm?id=S-803389
L2 - http://articles.sirc.ca/search.cfm?id=S-803389
ER -

TY - JOUR
ID - 4494
T1 - Identification of a common language describing paediatric physiotherapy practice for children with additional support needs, to support communication with those outside the physiotherapy profession
A1 - Hunter,Cathleen
A1 - Maciver,Donald
A1 - Howden,Stella
A1 - Forsyth,Kirsty
A1 - Adamson,Amanda
A1 - Bremner,Lynne
Y1 - 2013/03//
KW - SportDiscus
KW - *DEVELOPMENTAL disabilities
KW - *EXERCISE
KW - *MUSCLE strength
KW - *PEDIATRICS
KW - *PHYSICAL therapy
KW - *PEOPLE with disabilities
KW - Treatment
KW - Communication
KW - Focus Groups
KW - Interprofessional Relations
KW - INTERVIEWING
KW - RESEARCH -- Methodology
KW - RESEARCH -- Finance
KW - SOUND recordings
KW - Qualitative Research
KW - JUDGMENT sampling
KW - thematic analysis
RP - NOT IN FILE
SP - 84
EP - 91
JA - Physiotherapy
VL - 99
IS - 1
N2 - Abstract: Children with additional support needs (ASNs) often require physiotherapy intervention to help maximise their participation within the primary school setting. The aim of this research was to investigate paediatric physiotherapy practice in supporting primary school aged children with ASNs, in order to identify a language to describe this, which could be used to support communication with teachers, parents and others outside the profession. Design: Using a qualitative research multiple methods design, 2 focus groups and 5 structured interviews were held to investigate physiotherapy practice for this group. Participants: Senior

paediatric physiotherapists (n =13) from a range of specialities, with experience of supporting primary school aged children with ASNs. Analysis: Focus groups and interviews were digitally recorded, transcribed verbatim and analysed to establish links and patterns: followed by a cyclical process of respondent validation, and expert review. Results: Eight targets for physiotherapy intervention and twelve technique headings were synthesised from the data. The language used for labelling and description of these was aimed to be easily understood by colleagues outside the profession. Conclusions: The findings clearly identified the role of the paediatric physiotherapist as being to support primary school aged children with ASNs to acquire aspects of postural control, mobility and cardio-respiratory function. By grouping the data into eight areas of challenge as the focus of intervention, and twelve commonly used techniques, the researchers generated a language which can be used by paediatric physiotherapists to support communication with teachers, parents and others outside the profession, when describing their intent and interactions regarding these children. Copyright &y& Elsevier
SN - 00319406
UR -
https://login.proxy.bib.uottawa.ca/login?url=http://search.ebscohost.com/login.aspx?direct=true&db=s3h&AN=85154427&site=ehost-live
ER -

TY - JOUR
ID - 4495
T1 - Scooter Board Games and Activities
A1 - Hutchinson,Don
Y1 - 2007///Winter
KW - SportDiscus
KW - *BALL games
KW - *PHYSICAL education
KW - *PHYSICAL education for children
KW - *PHYSICAL fitness
KW - *SCHOOLS -- Exercises & recreations
KW - *GAMES
KW - *GAMES -- Rules
KW - RIDING toys
RP - NOT IN FILE
SP - 34
EP - 34
JA - Physical & Health Education Journal
VL - 73
IS - 4
N2 - This article describes the game scooter board basketball that is played using scooter boards. The author notes that games played on scooter boards are excellent group activities for any age or ability. For scooter board basketball, the author gives information on how to play discussing the rules and equipment needed. The game should include two baskets and three foam balls and enough scooters and pinnies to have two teams. Each player is not permitted to move while in possession of a ball but may move anywhere in the playing field at any other time. Shots must be made from outside a designated crease area. The ball may not be grabbed from an opponent's hand. One point is awarded for each shot that lands in the basket. Safety considerations and game modifications are also included
SN - 14980940
UR -
https://login.proxy.bib.uottawa.ca/login?url=http://search.ebscohost.com/login.aspx?direct=true&db=s3h&AN=31204535&site=ehost-live
ER -

TY - JOUR
ID - 4498
T1 - Frameworks of education: perspectives of Southeast Asian parents and Head Start staff
A1 - Hwa-Froelich,D.A.
A1 - Westby,C.E.
Y1 - 2003/10//

N1 - Accession Number: 2004042568. Language: English. Entry Date: 20040312. Revision Date: 20091218. Publication Type: journal article; questionnaire/scale; research; tables/charts. Journal Subset: Allied Health; Peer Reviewed; USA. Special Interest: Pediatric Care; Speech-Language Pathology/Audiology. Instrumentation: Interview Guide for Southeast Asian Parents; Interview Guide for Head Start Staff. Grant Information: Department of Health and Human Services (DHHS), Administration for Children and Families (ACF), and the Administration on Children, Youth, and Families (ACYF), Grant #90YD006101. No. of Refs: 81 ref. NLM UID: 0323431. Email: hwafroda@slu.edu
KW - CINAHL
KW - Asians
KW - Cultural Diversity
KW - Education -- In Infancy and Childhood
KW - Parents
KW - Project Head Start
KW - Adult
KW - Asia,Southeastern -- Ethnology
KW - Audiorecording
KW - Child
KW - Child,Disabled
KW - Discipline
KW - Early Childhood Intervention
KW - Ethnographic Research
KW - Funding Source
KW - Goals and Objectives
KW - Interview Guides
KW - Parent-Child Relations
KW - Parents of Disabled Children
KW - participant observation
KW - Semi-Structured Interview
KW - Teachers
KW - triangulation
KW - videorecording
KW - Human
RP - NOT IN FILE
SP - 299
EP - 319
JA - Language, Speech & Hearing Services in Schools
VL - 34
IS - 4
N2 - Purpose: Interviews with Southeast Asian families and Head Start staff, participant observation of family and staff conferences, observations of teacher-child and parent-child interactions, and a review of all written Head Start information given to parents were used to gain information regarding how Southeast Asian parents, children, and Head Start staff make sense of early childhood education, their roles in child learning, and the identification of disabilities or learning problems. Methods: Nine Southeast Asian families and 10 children were participants. One Vietnamese and 3 EuroAmerican Head Start staff members participated in a series of two or three semistructured individual interviews. Additionally, 3 families were observed during scheduled conferences with Head Start staff. Each child was observed at Head Start during meals, center time, and outdoor play time. All literature routinely given to parents was analyzed. Ethnomethodology (the study of how participants make sense of their world) was used in collection and analysis of the data. The constructs of organizational culture (Schein, 1987, 1992), independence/interdependence, and power/distance relationships were used to help to make sense of the data themes. Clinical Implications: Both families and staff were unaware of differences in their beliefs and values in the areas of education, parenting, child learning, and disability, which led to confusion and misunderstanding. All early childhood staff need to be aware of their underlying assumptions and how these may affect their interactions with children and families who may have different assumptions and expectations
SN - 0161-1461
AD - Saint Louis University, 12 McGannon Hall, St Louis, MO 63108-3412
UR - http://search.ebscohost.com/login.aspx?direct=true&db=cin20&AN=2004042568&site=ehost-live

ER -

TY - JOUR
ID - 4499
T1 - National recess survey: parental and child attitudes toward recess. (Abstract)
A1 - Hynes-Dusel,J.M.
Y1 - 2003/03//
KW - SportDiscus
KW - *RECESSES
KW - *PLAY
KW - *CARDIOVASCULAR fitness
KW - *STRESS (Physiology)
KW - Elementary Schools
KW - Parents
KW - ATTITUDE (Psychology)
KW - Interviews
KW - Child
RP - NOT IN FILE
SP - A
EP - 22
JF - Research Quarterly for Exercise & Sport
JA - Res Q Exerc Sport
VL - 74
IS - 1 Suppl
CY - ;
SN - 02701367
UR -
https://login.proxy.bib.uottawa.ca/login?url=http://search.ebscohost.com/login.aspx?direct=true&db=s3h&AN=SPHS-883145&site=ehost-live;http://articles.sirc.ca/search.cfm?id=S-883145;http://www.aahperd.org/
L2 - http://articles.sirc.ca/search.cfm?id=S-883145
ER -

TY - JOUR
ID - 4500
T1 - Systematic Review of Recess Interventions to Increase Physical Activity
A1 - Ickes,Melinda J.
A1 - Erwin,Heather
A1 - Beighle,Aaron
Y1 - 2013/08//
KW - SportDiscus
KW - *SCHOOL recess breaks
KW - *PHYSICAL activity
KW - *PLAYGROUNDS
KW - SYSTEMATIC reviews (Medical research)
KW - COST effectiveness
KW - Socioeconomic Factors
KW - Learning
KW - CHILDREN
KW - playground
KW - playtime
KW - program
KW - study
RP - NOT IN FILE
SP - 910
EP - 926
JF - Journal of Physical Activity & Health
JA - J Phys Act Health

VL - 10
IS - 6
N2 - Background: With the rapid increase in obesity rates among youth, efforts to increase physical activity (PA) have become a priority. School-based strategies for PA promotion must be cost-effective, unobtrusive, and linked to improved academic performance. Efforts to maximize recess PA are advocated because of both health and academic benefits. The purpose of this manuscript was to review recess interventions aimed to improve PA among youth, and make recommendations to develop related best practices. Methods: An extensive literature search was conducted to include all primary research articles evaluating any recess intervention with PA as an outcome. Results: The included 13 interventions represented both settings within the U.S and internationally, among preschools and elementary/primary schools. A variety of strategies were used within the design and implementation of each of the interventions including: added equipment/materials, markings, zones, teacher involvement, active video games, activity of the week, and activity cards. Of the included studies, 95% demonstrated positive outcomes as a result of the recess intervention. Conclusions: A number of simple, low-cost strategies can be implemented to maximize the amount of recess time students are allotted. Long-term follow-up studies are warranted for each of the recess strategies identified to be effective. ABSTRACT FROM AUTHOR
SN - 15433080
UR -
https://login.proxy.bib.uottawa.ca/login?url=http://search.ebscohost.com/login.aspx?direct=true&db=s3h&AN=90061418&site=ehost-live
ER -

TY - JOUR
ID - 4501
T1 - Dancing in the Park
A1 - Ilchena,Bev
Y1 - 2008///Spring
KW - SportDiscus
KW - *FOLK dancing
KW - *DANCE -- Study & teaching
KW - *DANCE
KW - *SCHOOLS -- Exercises & recreations
KW - *STUDENTS -- Recreation
KW - *SCHOOL children
KW - *PHYSICAL education for children
KW - Elementary Schools
KW - Canada
RP - NOT IN FILE
SP - 14
EP - 15
JA - Physical & Health Education Journal
VL - 74
IS - 1
N2 - The article presents information on an outdoor folk dance festival hosted by River East Transcona School Division in Canada, and presents step-by-step instructions for several dance forms. It mentions that the school division organizes this festival as part of its schedule of divisional events for elementary school students. It reports that the festival takes place over two days in May and involves students from grade one to grade three from 27 elementary schools in the division. It presents step-by-step instructions for the Hustle, Savila se Bela Loza, and the Tarantella dance forms
SN - 14980940
UR -
https://login.proxy.bib.uottawa.ca/login?url=http://search.ebscohost.com/login.aspx?direct=true&db=s3h&AN=31846463&site=ehost-live
ER -

TY - JOUR
ID - 4502

T1 - INVESTIGATION OF RELATIONS BETWEEN SCHOOL ACHIEVEMENTS AND MOTIVATION OF 6TH-GRADE STUDENTS FOR INVOLVEMENT IN PHYSICAL EDUCATION CLASSES
A1 - IliŽØ,Jelena
A1 - Vi□-njiŽØ,Dragoljub
A1 - MartinoviŽØ,Dragan
A1 - Markovi • «̧ • «ivorad
Y1 - 2010/11//
KW - SportDiscus
KW - *ACADEMIC achievement
KW - *PHYSICAL education for children
KW - MOTIVATION (Psychology) in children
KW - Elementary Schools
KW - SIXTH grade (Education)
KW - GENDER differences in education
KW - Regression Analysis
KW - Achievement
KW - Motivation
KW - Physical Education
RP - NOT IN FILE
SP - 34
EP - 41
JA - Proceedings of the Faculty of Physical Education, University of Banja Luka
IS - 2
N2 - Relations between school achievements and motivation for involvement in PE classes were investigated in a sample of 185 6th graders of both genders in elementary school. The independent variables included: the general success of the previous grade; PE grade; students' opinions about the sufficiency of knowledge acquired in physical education classes and students' engagement in sport. The scale for measurement of motivation consisted of 29 items obtained by adaptation of the Scale for measurement of the motives of sports achievement. The data were analyzed by multiple regression analysis and IØA ý test. It was established that there is a greater motivation for engagement in sport of male students compared to females. The following assumptions were not confirmed: that males will have higher PE grade compared to females; that the success of male students was negatively associated with sport commitment; that general success is negatively related to students' engagement in physical education and that PE grade is positively related to motivation for engagement in classes. However, in contrast to the results obtained in the sample of 7th graders, it was confirmed that the PE grade was associated with engagement in sport. Additionally, it was shown that students' general success and PE grade are not statistically significant predictors of student motivation for active participation and engagement in physical education classes, and that at this age, gender, students' engagement in some sport outside regular curricular activities and their opinion on the amount of knowledge acquired in PE classes are significant predictors. ABSTRACT FROM AUTHOR
SN - 9789993838135
UR - https://login.proxy.bib.uottawa.ca/login?url=http://search.ebscohost.com/login.aspx?direct=true&db=s3h&AN=77466325&site=ehost-live
ER -

TY - JOUR
ID - 4505
T1 - Epidemiology of Knee Injuries Among Boys and Girls in US High School Athletics
A1 - Ingram,Jay G.
A1 - Fields,Sarah K.
A1 - Yard,Ellen E.
A1 - Comstock,R.Dawn
Y1 - 2008/06//
KW - SportDiscus
KW - *KNEE -- Wounds & injuries
KW - *HIGH school athletes
KW - *SPORTS injuries

KW - *KNEE -- Surgery
KW - *SPORTS medicine
KW - United States
KW - Epidemiology
KW - high school
KW - injury
KW - knee
KW - Sports
KW - surveillance
RP - NOT IN FILE
SP - 1116
EP - 1122
JF - American Journal of Sports Medicine
JA - Am J Sports Med
VL - 36
IS - 6
N2 - Background: The knee joint is the second most commonly injured body site and the leading cause of high school sports-related surgeries. Knee injuries are among the most economically costly sports injuries and may require subsequent surgery or extensive and expensive rehabilitation. Purpose: To report the incidence, risk, and severity of high school knee injuries across sports, genders, and type of exposure. Study Design: Descriptive epidemiology study. Methods: During the 2005-2006 and 2006-2007 school years, 100 US high schools were randomly selected for a nationally representative sample. Certified athletic trainers tracked injuries using an online injury surveillance system, High School RIOƒ,›, in 9 high school sports. Results: There were 1383 knee injuries reported during 3 551 131 athlete exposures for a rate of 3.89 knee injuries per 10 000 athlete exposures. Although boys had a higher overall rate of knee injury (rate ratio, 1.38; confidence interval, 1.22-1.55), girls were twice as likely to sustain knee injuries requiring surgery (major knee injuries) than were boys (injury proportion ratio, 1.98; confidence interval, 1.45-2.70) and twice as likely to incur noncontact major knee injuries (injury proportion ratio, 1.98; confidence interval, 1.23-3.19) as were boys. Although illegal play was identified as a contributing factor in only 5.7% of all knee injuries, 20% of knee injuries resulting from illegal play required surgery. Conclusion: Knee injury rates and patterns varied by sport, gender, and type of exposure. Identified gender differences included differences in injury rates, injury severity, and basic injury mechanism. Further surveillance is crucial for the development of targeted, evidence-based injury prevention strategies to reduce the morbidity and economic impact of knee surgeries. ABSTRACT FROM AUTHOR
SN - 03635465
UR -
https://login.proxy.bib.uottawa.ca/login?url=http://search.ebscohost.com/login.aspx?direct=true&db=s3h&AN=32582228&site=ehost-live
ER -

TY - JOUR
ID - 4507
T1 - Privacy behaviour of preschool children: mechanisms and functions in the day-care environment
A1 - Jacobs,E.
Y1 - 1980///
KW - SportDiscus
KW - *PSYCHOLOGY
KW - *PLAY
KW - INFANTS
KW - Child Care
KW - Indoor
KW - behaviour
RP - NOT IN FILE
CY - ;
UR -
https://login.proxy.bib.uottawa.ca/login?url=http://search.ebscohost.com/login.aspx?direct=true&db=s3h&AN=SPH82103&site=ehost-live;http://articles.sirc.ca/search.cfm?id=82103
L2 - http://articles.sirc.ca/search.cfm?id=82103

ER -

TY - JOUR
ID - 4509
T1 - Translating the PLAYgrounds program into practice: A process evaluation using the RE-AIM framework
A1 - Janssen,Mirka
A1 - Toussaint,Huub M.
A1 - van Mechelen,Willem
A1 - Verhagen,Evert A.L.M.
Y1 - 2013/05//
KW - SportDiscus
KW - *PRACTICE (Sports)
KW - *PLAYGROUNDS
KW - *PHYSICAL activity -- Measurement
KW - CURRICULUM frameworks
KW - ACQUISITION of data
KW - Questionnaires
RP - NOT IN FILE
SP - 211
EP - 216
JF - Journal of Science & Medicine in Sport
JA - J Sci Med Sport
VL - 16
IS - 3
N2 - Abstract: Objectives: To present the results of the process evaluation of the PLAYgrounds program, using the RE-AIM framework. Design: This study provides information regarding Reach, Adoption, Implementation and Maintenance. Methods: The PLAYgrounds program promotes increasing levels of physical activity in 6ƒ_"12 years old children and was evaluated using the RE-AIM framework in 4 intervention schools. Data collection consisted of a physical activity questionnaire with children (n =765, Reach), SOPLAY observations (Implementation and Maintenance), questionnaires on the satisfaction of the implemented elements with teachers (n =59) and children (n =730, Implementation) and interviews for increased depth of information. In addition a simple counting of participating schools, describing of non-participating reasons and characteristics of the schools were documented (Adoption). Results: Reach of the target population (i.e. inactive children) was 60.7% (n =464) and the target population was representative for populations in low-SES neighbourhoods. The PLAYgrounds program was adopted by 4 schools (80%), at which 5 (from 7) program elements were successfully implemented. At 18 months follow-up, 3 of those 5 elements were completely maintained. Conclusions: Adoption, Implementation, and Maintenance proved to be very high. Most likely due to the PLAYgrounds program being a complete intervention package that included financial, material, and staff support. Therefore, it is recommended to retain this high level of support when introducing the PLAYgrounds (or any other intervention) program in schools. In the future it would be recommended to evaluate the PLAYgrounds program on maintenance in schools where the key-person is employed at the school and funding is not available. Copyright &y& Elsevier
SN - 14402440
UR -
https://login.proxy.bib.uottawa.ca/login?url=http://search.ebscohost.com/login.aspx?direct=true&db=s3h&AN=89077197&site=ehost-live
ER -

TY - JOUR
ID - 4510
T1 - Childhood participation in after-school activities: what is to be expected?
A1 - Jarus,T.
A1 - Anaby,D.
A1 - Bart,O.
A1 - Engel-Yeger,B.
A1 - Law,M.
Y1 - 2010/08/15/

N1 - Accession Number: 2010762319. Language: English. Entry Date: 20100924. Revision Date: 20121109. Publication Type: journal article; research; tables/charts. Journal Subset: Allied Health; Double Blind Peer Reviewed; Europe; Expert Peer Reviewed; Peer Reviewed; UK & Ireland. Special Interest: Occupational Therapy; Pediatric Care. Instrumentation: Children's Assessment of Participation and Enjoyment (CAPE) (King et al) [Hebrew]. No. of Refs: 29 ref. NLM UID: 7708186
KW - CINAHL
KW - Leisure Activities -- In Infancy and Childhood
KW - Leisure Activities -- In Adolescence
KW - Recreation -- In Infancy and Childhood
KW - Recreation -- In Adolescence
KW - Social Isolation -- In Infancy and Childhood
KW - Social Isolation -- In Adolescence
KW - Child Development
KW - Adolescent Development
KW - Human
KW - Research Instruments
KW - Two-Way Analysis of Variance
KW - convenience sample
KW - Task Performance and Analysis
KW - Descriptive Statistics
KW - post hoc analysis
KW - effect size
KW - Sex Factors
KW - Age Factors
KW - Physical Activity -- In Infancy and Childhood
KW - Physical Activity -- In Adolescence
KW - Skill Acquisition -- In Infancy and Childhood
KW - Skill Acquisition -- In Adolescence
KW - Child
KW - adolescence
KW - Scales
RP - NOT IN FILE
SP - 344
EP - 350
JF - British Journal of Occupational Therapy
VL - 73
IS - 8
N2 - Participation is recognised as a key to health and wellbeing and is considered to be a vital part of the development of children and youth. The purpose of this study was to examine the participation patterns of children and adolescents in their time outside formal school. More specifically, the influence of age and gender on after-school participation patterns was explored. Method: Three hundred and thirty-two children and adolescents (5 to 18 years old) were divided into five age groups and completed the Children's Assessment of Participation and Enjoyment (CAPE). To test the interaction effect of gender and age on participation measures, ANOVAs were conducted. Results: The results indicated differences with moderate to high effect size in participation patterns across age, where a decline in participation was detected as children made the transition to adolescence. Differences with negligible effect size in participation were found between boys and girls in some of the activity types. No interaction effect between age and gender was observed. Conclusion: Participation patterns change with age, particularly as children make the transition to adolescence; however, this change does not depend on gender. Intervention strategies might take into account these inherent changes. Future studies might consider additional factors (for example, environment) while examining participation throughout age
SN - 0308-0226
AD - University of British Columbia, CanDo Research Center, Vancouver, British Columbia, Canada
UR - http://search.ebscohost.com/login.aspx?direct=true&db=cin20&AN=2010762319&site=ehost-live
ER -

TY - JOUR
ID - 4513

T1 - A Probabilistic Arsenic Exposure Assessment for Children Who Contact Chromated Copper Arsenate (CCA)-Treated Playsets and Decks, Part 2: Sensitivity and Uncertainty Analyses
A1 - Jianping,Xue
A1 - Zartarian,Valerie G.
A1 - A-zkaynak,HalA¯k
A1 - Dang,Winston
A1 - Glen,Graham
A1 - Smith,Luther
A1 - Stallings,Casson
Y1 - 2006/04//
KW - SportDiscus
KW - *TOXICOLOGY
KW - arsenic
KW - PEDIATRIC toxicology
KW - PARIS green
KW - RISK exposure
KW - Uncertainty
KW - CCA
KW - children's arsenic exposure
KW - probabilistic exposure model
KW - sensitivity analyses
KW - uncertainty analyses
RP - NOT IN FILE
SP - 533
EP - 541
JA - Risk Analysis: An International Journal
VL - 26
IS - 2
N2 - A probabilistic model (SHEDS-Wood) was developed to examine children's exposure and dose to chromated copper arsenate (CCA)-treated wood, as described in Part 1 of this two-part article. This Part 2 article discusses sensitivity and uncertainty analyses conducted to assess the key model inputs and areas of needed research for children's exposure to CCA-treated playsets and decks. The following types of analyses were conducted: (1) sensitivity analyses using a percentile scaling approach and multiple stepwise regression; and (2) uncertainty analyses using the bootstrap and two-stage Monte Carlo techniques. The five most important variables, based on both sensitivity and uncertainty analyses, were: wood surface residue-to-skin transfer efficiency; wood surface residue levels; fraction of hand surface area mouthed per mouthing event; average fraction of nonresidential outdoor time a child plays on/around CCA-treated public playsets; and frequency of hand washing. In general, there was a factor of 8 for the 5th and 95th percentiles and a factor of 4 for the 50th percentile in the uncertainty of predicted population dose estimates due to parameter uncertainty. Data were available for most of the key model inputs identified with sensitivity and uncertainty analyses; however, there were few or no data for some key inputs. To evaluate and improve the accuracy of model results, future measurement studies should obtain longitudinal time-activity diary information on children, spatial and temporal measurements of residue and soil concentrations on or near CCA-treated playsets and decks, and key exposure factors. Future studies should also address other sources of uncertainty in addition to parameter uncertainty, such as scenario and model uncertainty. ABSTRACT FROM AUTHOR
SN - 02724332
UR -
https://login.proxy.bib.uottawa.ca/login?url=http://search.ebscohost.com/login.aspx?direct=true&db=s3h&AN=20251731&site=ehost-live
ER -

TY - JOUR
ID - 4514
T1 - A Meta-Analysis of Children's Hand-to-Mouth Frequency Data for Estimating Nondietary Ingestion Exposure
A1 - Jianping,Xue
A1 - Zartarian,Valerie

A1 - Moya,Jacqueline
A1 - Freeman,Natalie
A1 - Beamer,Paloma
A1 - Black,Kathy
A1 - Tulve,Nicolle
A1 - Shalat,Stuart
Y1 - 2007/04//
KW - SportDiscus
KW - *CHILDREN
KW - *HAND
KW - *MOUTH
KW - *CHEMICALS
KW - ingestion
KW - META-analysis
KW - Children's exposure
KW - hand-to-mouth frequency
KW - nondietary ingestion
RP - NOT IN FILE
SP - 411
EP - 420
JA - Risk Analysis: An International Journal
VL - 27
IS - 2
N2 - Because of their mouthing behaviors, children have a higher potential for exposure to available chemicals through the nondietary ingestion route; thus, frequency of hand-to-mouth activity is an important variable for exposure assessments. Such data are limited and difficult to collect. Few published studies report such information, and the studies that have been conducted used different data collection approaches (e.g., videography versus real-time observation), data analysis and reporting methods, ages of children, locations, and even definitions of ƒ_omouthing.ƒ__ For this article, hand-to-mouth frequency data were gathered from 9 available studies representing 429 subjects and more than 2,000 hours of behavior observation. A meta-analysis was conducted to study differences in hand-to-mouth frequency based on study, age group, gender, and location (indoor vs. outdoor), to fit variability and uncertainty distributions that can be used in probabilistic exposure assessments, and to identify any data gaps. Results of this analysis indicate that age and location are important for hand-tomouth frequency, but study and gender are not. As age increases, both indoor and outdoor hand-to-mouth frequencies decrease. Hand-to-mouth behavior is significantly greater indoors than outdoors. For both indoor and outdoor hand-to-mouth frequencies, interpersonal, and intra-personal variability are ƒ^¬60% and ƒ^¬30%, respectively. The variance difference among different studies is much bigger than its mean, indicating that different studies with different methodologies have similar central values. Weibull distributions best fit the observed data for the different variables considered and are presented in this article by study, age group, and location. Average indoor hand-to-mouth behavior ranged from 6.7 to 28.0 contacts/hour, with the lowest value corresponding to the 6 to <11 year olds and the highest value corresponding to the 3 to <6 month olds. Average outdoor hand-to-mouth frequency ranged from 2.9 to 14.5 contacts/hour, with the lowest value corresponding to the 6 to <11 year olds and the highest value corresponding to the 6 to <12 month olds. The analysis highlights the need for additional hand-to-mouth data for the <3 months, 3 to <6 months, and 3 to <6 year age groups using standardized collection and analysis because of lack of data or high uncertainty in available data. This is the first publication to report Weibull distributions as the best fitting distribution for hand-to-mouth frequency; using the best fitting exposure factor distribution will help improve estimates of exposure. The analyses also represent a first comprehensive effort to fit hand-to-mouth frequency variability and uncertainty distributions by indoor/outdoor location and by age groups, using the new standard set of age groups recommended by the U.S. Environmental Protection Agency for assessing childhood exposures. Thus, the data presented in this article can be used to update the U.S. EPA's Child-Specific Exposure Factors Handbook and to improve estimates of nondietary ingestion in probabilistic exposure modeling. ABSTRACT FROM AUTHOR
SN - 02724332
UR -
https://login.proxy.bib.uottawa.ca/login?url=http://search.ebscohost.com/login.aspx?direct=true&db=s3h&AN=25065584&site=ehost-live
ER -

TY - JOUR
ID - 4515
T1 - Sociological research on social position, leisure time and sports life of Chinese and Japanese women
A1 - Jin,Y.Q.
A1 - Liu,Y.
A1 - Yang,Z.W.
A1 - Liu,Z.J.
A1 - Yao,S.Q.
A1 - Guo,D.G.
Y1 - 2002///
KW - SportDiscus
KW - *LEISURE
KW - *SPORTS
KW - *LIFE style
KW - WOMEN -- Sociological aspects
KW - Women
KW - SOCIAL factors
KW - sociology
KW - Comparative Studies
KW - China
KW - Japan
RP - NOT IN FILE
SP - 154
EP - 156
JA - Journal of Beijing University of Physical Education
VL - 25
IS - 2
CY - ;
N2 - Since the reform and the opening to the outside world, the Chinese women's social position has been raised remarkably. Numerous women have been entitled to participate in the deliberation and administration of state affairs and enjoy equal pay for equal work, which shows the advantages of the socialist system. These are superior to those of the Japanese women of the same era. In respect of sports life, the proportions of the Chinese and Japanese women' participation in sport are lower than those of the men's. The rate of the Japanese women's participation is higher than that of the Chinese women's. Therefore, to encourage and support the vast women to participate in physical activities is of much significance in their physical and mental health as well as in the education of their children. And it is also the important contents of their healthy life style. In physical education reform and development, more attention should be paid to the work of women's sport
SN - 10073612
UR -
https://login.proxy.bib.uottawa.ca/login?url=http://search.ebscohost.com/login.aspx?direct=true&db=s3h&AN=SPHS-840732&site=ehost-live
ER -

TY - JOUR
ID - 4516
T1 - Skoleidrett som tilbudsundervisning. / School athletics as elective instruction
A1 - Johannessen,T.
A1 - Stensaasen,S.
Y1 - 1971///
KW - SportDiscus
KW - *SPORTS
KW - *PHYSICAL education & training
KW - *PARTICIPATION
KW - sociology
KW - ATTITUDE (Psychology)
KW - Elementary Schools

KW - Norway
KW - Child
KW - EXTRACURRICULAR
RP - NOT IN FILE
CY - Oslo, Norway;
PB - Norges Idrettshogskole
N2 - Report on the second year of a 4-year experiment in voluntary after school physical education. Teachers and specialized instructors gave 259 pupils in 3 elementary grades one hour of instruction daily in one or more elective physical activities. Information was collected on pupil's attitudes toward sport and a number of other variables. Provisional results indicate that voluntary after school programs may be useful supplements to in school activities and that participation of outside instructors is successful
UR -
https://login.proxy.bib.uottawa.ca/login?url=http://search.ebscohost.com/login.aspx?direct=true&db=s3h&AN=SPH336439&site=ehost-live
ER -

TY - JOUR
ID - 4519
T1 - Possible influences of urban environments on eating and physical activity behaviors
A1 - Johns,D.P.
A1 - Ha,A.S.C.
A1 - Chan,D.W.K.
Y1 - 2001///Fall
KW - SportDiscus
KW - *PHYSICAL fitness
KW - *STRESS (Physiology)
KW - *CARDIOVASCULAR fitness
KW - *NUTRITION
KW - *LIFE style
KW - BOYS
KW - girls
KW - HONG Kong (China)
KW - Observation
KW - Environment
KW - urban area
KW - Child
RP - NOT IN FILE
SP - 40
EP - 45
JA - Journal of the International Council for Health, Physical Education, Recreation, Sport & Dance
VL - 37
IS - 4
CY - ;
N2 - This article reports findings of an exploratory research project to examine specific aspects of lifestyles of children who lived in an extremely dense urban areas in Hong Kong. The Behavior Eating and Activity for Children's Health Evaluation System was used to characterize physical and social environments, physical activity, ingestion of food, and interactions relating to food intake and levels of physical activity of 40 children (N = 40; M = 15; F = 25; CA 6-8 years). Data were derived from momentary sampling frequencies. Means, standard deviations, and chi square statistics were utilized. Findings showed children might experience constrained lifestyles, possibly influenced by adults and social conditions of their environments. They did not participate at levels of physical activity of sufficient intensity to have any effects on fitness and health. Moreover, school recesses were associated with snacking, rather than participating in vigorous activities
Cet article fait etat des resultats d'une recherche exploratoire ayant pour but d'examiner certains aspects particuliers de styles de vie d'enfants vivant dans des milieux urbains a tres haute densite. Une categorisation d'environnements physiques et sociaux; d'activites physiques; de consommation de nourriture et d'interactions existant entre la consommation de nourriture et la pratique d'activites physiques a ete fait aupres de 40 enfants (H = 15, F = 25; CA 6-8 ans) a partir du "Behavior Eating and Activity for Children's Health Evaluation

System". Les donnees ont ete traitees a partir de moyennes, ecarts types et khi carre. Les resultats demontrent que les enfants sont possiblement exposes a des styles de vie contraignants, probablement influences par les adultes, et conditions sociales inherentes a leur environnement. Selon les resultats les enfants ne pratiquent pas d'activites physiques a une intensite suffisante pour que ces dernieres aient un effet sur leur niveau de conditionnement physique et leur sante. De plus les recreations a l'ecole sont pour la plupart associees a des collations, au lieu d'activites physiques vigoureuses
Der Artikel berichtet ueber Ergebnisse eines Forschungsprojektes, das spezifische Aspekte der Lebensgewohnheiten von Kindern in Staedten mit sehr hoher Bevoelkerungsdichte untersucht hat. Das "Behavior Eating und Activity for Children's Health Evaluation System" Instrument wurde verwendet, um die physische und soziale Lebensumwelt der Kinder, Art und Umfang ihrer koerperlichen Betaetigung, Ernaehrung sowie mit der Ernaehrung in Zusammenhang stehende Interaktionen von 40 Kindern (M = 15, W = 25; CA 6-8 Jahre) zu erfassen. Daten wurden mit Hilfe des "momentary sampling frequencies" Verfahrens erhoben. Mittelwerte, Standardabweichungen und Chi Square Statistiken wurden errechnet. Die Ergebnisse zeigten, dass Kinder, moeglicherweise beeinflusst durch Erwachsene sowie die sozialen Bedingungen ihrer Lebensumwelten, in ihren Lebensgewohnheiten eingeschraenkt sein koennten. Die Kinder waren nicht in ausreichendem Mass koerperlich aktiv, dass es sich positiv auf ihre Fitness und Gesundheit ausgewirkt haette. Ausserdem wurden Schulpausen eher zum Essen als zur koerperlichen Betaetigung genutzt
SN - 10912193
UR -
https://login.proxy.bib.uottawa.ca/login?url=http://search.ebscohost.com/login.aspx?direct=true&db=s3h&AN=SPHS-798669&site=ehost-live;http://articles.sirc.ca/search.cfm?id=S-798669;http://www.aahperd.org
L2 - http://articles.sirc.ca/search.cfm?id=S-798669
ER -

TY - JOUR
ID - 4520
T1 - Gender and Race in Beliefs about Outdoor Air Pollution
A1 - Johnson,Branden B.
Y1 - 2002/08//
KW - SportDiscus
KW - RISK communication
KW - Air Pollution
KW - ENVIRONMENTAL risk assessment
KW - Risk Assessment
KW - HOUSEHOLD surveys
KW - DEMOGRAPHIC surveys
KW - gender
KW - RACE
KW - Risk Perception
RP - NOT IN FILE
SP - 725
EP - 738
JA - Risk Analysis: An International Journal
VL - 22
IS - 4
N2 - Universal need for, or reactions to, risk communications should not be assumed; potential differences across demographic groups in environmental risk beliefs, attitudes, and behaviors could affect risk levels or opportunities for risk reduction. This article reports relevant findings from a survey experiment involving 1,100 potential jurors in Philadelphia concerning public responses to outdoor air pollution and air quality information. Flynn et al. (1994) and Finucane et al. (2000) found significant differences in risk ratings for multiple hazards, and in generic risk beliefs, between white men (or a subset) and all others (white women, nonwhite men, and nonwhite women). This study examined whether white men had significantly different responses to air pollution and air pollution information. An opportunity sample of volunteers from those awaiting potential jury duty in city courts (matching census estimates for white versus nonwhite proportions, but more female than the cityƒ_Ts adult population and more likely to have children) filled out questionnaires distributed quasiƒ_"randomly. On most measures there were no statistically significant differences among white men (N = 192), white women (N = 269), nonwhite men (N = 165), and nonwhite women (N = 272). Nonwhites overall

(particularly women) reported more concern about and sensitivity to air pollution than whites, and were more concerned by (even overly sensitive to) air pollution information provided as part of the experiment. Nonwhites also were more likely (withinƒ_"gender comparisons) to report being active outdoors for at least four hours a day, a measure of potential exposure to air pollution, and to report intentions to reduce such outdoor activity after reading air pollution information. Differences between men and women were less frequent than between whites and nonwhites; the most distinctive group was nonwhite women, followed by white men. Flynn et al. (1994) and Finucane et al. (2000) found a far larger proportion of significant differences, with white men as most distinctive, probably due to use of different measures, study design, and population samples. However, all three studies broadly confirm the existence of gender and race interactions in risk beliefs and attitudes (particularly for white men and nonwhite women) that deserve more attention from researchers. ABSTRACT FROM AUTHOR
SN - 02724332
UR -
https://login.proxy.bib.uottawa.ca/login?url=http://search.ebscohost.com/login.aspx?direct=true&db=s3h&AN=7203579&site=ehost-live
ER -

TY - JOUR
ID - 4522
T1 - Weight training injury trends: a 20-year survey
A1 - Jones,C.S.
A1 - Christensen,C.
A1 - Young,M.
Y1 - 2000/07//
N1 - Accession Number: 2000065903. Language: English. Entry Date: 20001001. Revision Date: 20091218. Publication Type: journal article; CEU; research; tables/charts. Journal Subset: Allied Health; Peer Reviewed; USA. No. of Refs: 22 ref. NLM UID: 0427461. Email: ches@comp.uark.edu
KW - CINAHL
KW - Weight Lifting
KW - Athletic Injuries -- Trends
KW - Education,Continuing (Credit)
KW - Retrospective Design
KW - Emergency Service
KW - Record Review
KW - Athletic Injuries -- Epidemiology
KW - probability sample
KW - Child,Preschool
KW - Child
KW - adolescence
KW - Adult
KW - Middle Age
KW - Aged
KW - Male
KW - Female
KW - Injury Pattern
KW - Confidence Intervals
KW - Descriptive Statistics
KW - Data Analysis Software
KW - Age Factors
KW - Sex Factors
KW - Severity of Injury
KW - Odds Ratio
KW - Weight Lifting -- Mortality
KW - Human
RP - NOT IN FILE
SP - 61
JF - Physician & Sportsmedicine

JA - Phys Sportsmed.
VL - 28
IS - 7
N2 - Damage from long-term, cumulative effects of sun exposure, while slow to develop, can become life-threatening. The cutaneous effects of ultraviolet light include sunburn, photosensitivity, immunosuppression, premature aging, and several types of skin cancer. Clinicians need to know the characteristic appearance of premalignant lesions, basal cell carcinoma, squamous cell carcinoma, and malignant melanoma. Also, patients who are active outdoors need education about sun protection, including avoidance at peak hours, clothing, sunglasses, and sunscreens. Care for sunburn may involve cool compresses, corticosteroid cream, oral antihistamines, and anti-inflamatory drugs
SN - 0091-3847
AD - University of Arkansas, Dept of Health Science, Kinesiology, Recreation, and Dance, 308 HPER Bldg, Fayetteville, AR 72701
UR - http://search.ebscohost.com/login.aspx?direct=true&db=cin20&AN=2000065903&site=ehost-live
ER -

TY - JOUR
ID - 4523
T1 - A phase 2 autologous cellular therapy trial in patients with acute, complete spinal cord injury: pragmatics, recruitment, and demographics
A1 - Jones,L.A.T.
A1 - Lammertse,D.P.
A1 - Charlifue,S.B.
A1 - Kirshblum,S.C.
A1 - Apple,D.F.
A1 - Ragnarsson,K.T.
A1 - Poonian,D.
A1 - Betz,R.R.
A1 - Knoller,N.
A1 - Heary,R.F.
A1 - Choudhri,T.F.
A1 - Jenkins,A.L.
A1 - Falci,S.P.
A1 - Snyder,D.A.
Y1 - 2010/11//
KW - SportDiscus
KW - *SPINAL cord -- Wounds & injuries
KW - *SPINAL cord
KW - *SURGERY
KW - *MEDICAL records
KW - Stem Cells -- Transplantation
KW - Analysis of Variance
KW - Clinical Trials
KW - MEDICAL cooperation
KW - MEDICAL referral
KW - Research
KW - SAMPLING (Statistics)
KW - Statistics
KW - data analysis
KW - RESEARCH bias
KW - RESEARCH subjects (Persons)
KW - Patient Selection
KW - Israel
KW - United States
RP - NOT IN FILE
SP - 798
EP - 807

JF - Spinal Cord
VL - 48
IS - 11
N2 - Study design:Post hoc analysis from a randomized controlled cellular therapy trial in acute, complete spinal cord injury (SCI).Objectives:Description and quantitative review of study logistics, referral patterns, current practice patterns and subject demographics.Setting:Subjects were recruited to one of six international study centers.Methods:Data are presented from 1816 patients pre-screened, 75 participants screened and 50 randomized.Results:Of the 1816 patients pre-screened, 53.7% did not meet initial study criteria, primarily due to an injury outside the time window (14 days) or failure to meet neurological criteria (complete SCI between C5 motor/C4 sensory and T11). MRIs were obtained on 339 patients; 51.0% were ineligible based on imaging criteria. Of the 75 participants enrolled, 25 failed screening (SF), leaving 50 randomized. The primary reason for SF was based on the neurological exam (51.9%), followed by failure to meet MRI criteria (22.2%). Of the 50 randomized subjects, there were no significant differences in demographics in the active versus control arms. In those participants for whom data was available, 93.8% (45 of 48) of randomized participants received steroids before study entry, whereas 94.0% (47 of 50) had spine surgery before study enrollment.Conclusion:The 'funnel effect' (large numbers of potentially eligible participants with a small number enrolled) impacts all trials, but was particularly challenging in this trial due to eligibility criteria and logistics. Data collected may provide information on current practice patterns and the issues encountered and addressed may facilitate design of future trials. ABSTRACT FROM AUTHOR
SN - 13624393
UR -
https://login.proxy.bib.uottawa.ca/login?url=http://search.ebscohost.com/login.aspx?direct=true&db=s3h&AN=54960791&site=ehost-live
ER -

TY - JOUR
ID - 4525
T1 - A Cognitive Approach to Elementary School Recess
A1 - Jones,Rose B.
Y1 - 2005/03//
KW - SportDiscus
KW - *SCHOOL recess breaks
KW - *PHYSICAL education
KW - *SCHOOL children
KW - *PLAY
KW - COGNITIVE learning
KW - CLASSROOM learning centers
RP - NOT IN FILE
SP - 33
EP - 34
JA - Teaching Elementary Physical Education
VL - 16
IS - 2
N2 - The article lays emphasis on need to have a cognitive recess curriculum that strengthens the need for physical education and benefits the learning process throughout the school. A cognitive recess curriculum offers four learning centers, with an additional free play area. The learning centers consist of a math center, science center, language development center, and music or art center. Classroom teachers would provide prepared descriptions of several cognitive games like playing school, math bingo, etc. that children would likely enjoy playing during their recess period. Also included are early versions or variations that can still be played today to improve communication and interaction
SN - 10454853
UR -
https://login.proxy.bib.uottawa.ca/login?url=http://search.ebscohost.com/login.aspx?direct=true&db=s3h&AN=16330814&site=ehost-live
ER -

TY - JOUR

ID - 4526
T1 - Players truly appreciate bringing their kids to work
A1 - Jones,Todd
Y1 - 2005/06/24/
KW - SportDiscus
KW - *ATHLETES
KW - *BASEBALL
KW - *CLUBHOUSES
KW - *STADIUMS
KW - *SPORTS
KW - FATHER & child
RP - NOT IN FILE
SP - 41
EP - 166
JA - Sporting News
VL - 229
IS - 25
N2 - The article presents the author's views related to children and of baseball athletes. According to the author, team Florida Marlins manager Jack McKeon knows how important family is to the players, and he graciously allows the kids be a part of clubhouse life. The players, in turn, know how much of a privilege that is. The author's 10-year-old son's day at the yard begins around 2 o'clock. He dresses in his own locker at Dolphins Stadium, then joins about a half-dozen other kids at the indoor batting cage to plot the day's activities. If the athletes win, their children are paraded into the clubhouse afterward to join their fathers
SN - 0038805X
UR -
https://login.proxy.bib.uottawa.ca/login?url=http://search.ebscohost.com/login.aspx?direct=true&db=s3h&AN=17354333&site=ehost-live
ER -

TY - JOUR
ID - 4527
T1 - A Multisport Epidemiologic Comparison of Anterior Cruciate Ligament Injuries in High School Athletics
A1 - Joseph,Allan M.
A1 - Collins,Christy L.
A1 - Henke,Natalie M.
A1 - Yard,Ellen E.
A1 - Fields,Sarah K.
A1 - Comstock,R.Dawn
Y1 - 2013/11//
KW - SportDiscus
KW - *ANTERIOR cruciate ligament -- Surgery
KW - *ANTERIOR cruciate ligament -- Wounds & injuries
KW - *ATHLETIC trainers
KW - *EPIDEMIOLOGY
KW - *FOOTBALL injuries
KW - *HIGH school athletes
KW - *INTERNET
KW - *SOCCER injuries
KW - *SPORTS
KW - *SPORTS injuries
KW - *TEENAGERS -- Health
KW - *SPORTS events
KW - *PHYSICAL training & conditioning
KW - PUBLIC health surveillance -- Methodology
KW - Confidence Intervals
KW - Research
KW - Mathematics

KW - RESEARCH -- Methodology
KW - SEX distribution (Demography)
KW - data analysis
KW - CONTINUING education units
KW - NATIONAL Athletic Trainers Association
KW - DATA analysis -- Software
KW - Descriptive Statistics
KW - adolescence
KW - United States
KW - knee
KW - Sports
KW - surveillance
RP - NOT IN FILE
SP - 810
EP - 817
JA - Journal of Athletic Training
VL - 48
IS - 6
N2 - Background: The knee joint is the second most commonly injured body site after the ankle and the leading cause of sport-related surgeries. Knee injuries, especially of the anterior cruciate ligament (ACL), are among the most economically costly sport injuries, frequently requiring expensive surgery and rehabilitation. Objective: To investigate the epidemiology of ACL injuries among high school athletes by sport and sex. Design: Descriptive epidemiology study. Main Outcome Measure(s): Using an Internet-based data-collection tool, Reporting Information Online (RIO), certified athletic trainers from 100 nationally representative US high schools reported athlete-exposure and injury data for athletes from 9 sports during the 2007/08-2011/12 academic years. The outcome of interest in this study was ACL injuries. Results: During the study period, 617 ACL injuries were reported during 9452180 athlete exposures (AEs), for an injury rate of 6.5 per 100000 AEs. Nationally, in the 9 sports studied, an estimated 215628 ACL injuries occurred during the study period. The injury rate was higher in competition (17.6) than practice (2.4; rate ratio [RR] = 7.3, 95% confidence interval [CI] = 6.08, 8.68). Girls' soccer had the highest injury rate (12.2) followed by boys' football (11.1), with boys' basketball (2.3) and boys' baseball (0.7) having the lowest rates. In sex-comparable sports, girls had a higher rate (8.9) than boys (2.6; RR = 3.4, 95% CI = 2.64, 4.47). Overall, 76.6% of ACL injuries resulted in surgery. The most common mechanisms of injury were player-to-player contact (42.8%) and no contact (37.9%). Conclusions: Anterior cruciate ligament injury rates vary by sport, sex, and type of exposure. Recognizing such differences is important when evaluating the effectiveness of evidence-based, targeted prevention efforts. ABSTRACT FROM AUTHOR
SN - 10626050
UR -
https://login.proxy.bib.uottawa.ca/login?url=http://search.ebscohost.com/login.aspx?direct=true&db=s3h&AN=92723890&site=ehost-live
ER -

TY - JOUR
ID - 4528
T1 - Can 5-6 year old children orientate themselves in a cave?
A1 - Jovignot,F.
Y1 - 1995///Autumn
KW - SportDiscus
KW - *ORIENTEERING
KW - *SPELEOLOGY
KW - Ability
KW - Cognition
KW - Child
KW - SPATIAL ORIENTATION
RP - NOT IN FILE
SP - 64
EP - 75
JA - Scientific Journal of Orienteering

VL - 11
IS - 2
CY - ;
N2 - We propose 3 experimental approaches to test the capacities of 5-6 year-old children in orientation with a map. The first 2 are essential preconditions in order to put the third to the test. We first submitted the children to an electronic maze test. This showed a very significant difference between the performance of the 5 year old children compared with the 6 year olds. We then trained them to read a series of simple maps ranging from their classroom to a playground. The children succeeded in using these maps, to find landmarks, but they were unable to orientate themselves with a more complex street map. Only the older children took part in an orienteering race, the results of which did not correlate with those of the maze test. Finally, we asked every child to use a map, in order to find their way back on a caving trip, previously prepared. It turned out that they were unable to do that, probably for some because the emotion triggered by this first speleological experience causes a regression of their cognitive capacities. For the others, we think that this task was too complex, we would have needed time and a fitting-in the situation in order to help them to be successful
SN - 10120602
UR -
https://login.proxy.bib.uottawa.ca/login?url=http://search.ebscohost.com/login.aspx?direct=true&db=s3h&AN=SPH389348&site=ehost-live;http://articles.sirc.ca/search.cfm?id=389348
L2 - http://articles.sirc.ca/search.cfm?id=389348
ER -

TY - JOUR
ID - 4530
T1 - Spending of summer holidays of Slovenian secondary school children. / Traveni letnich prazdnin slovinskymi stredoskolaky
A1 - Jurak,G.
A1 - Kovac,M.
A1 - Strel,J.
Y1 - 2002///
KW - SportDiscus
KW - *PHYSICAL fitness
KW - *LIFE style
KW - *CARDIOVASCULAR fitness
KW - *STRESS (Physiology)
KW - *SPORTS
KW - *PARTICIPATION
KW - EDUCATION,Secondary
KW - teenagers
KW - Students
KW - summer
KW - VACATIONS
KW - Surveys
KW - Slovenia
RP - NOT IN FILE
SP - 51
EP - 66
JA - Acta Universitatis Carolinae Kinanthropologica
VL - 38
IS - 1
CY - ;
N2 - The main objective of our research was to analyse ways in which Slovenian secondary school children spend their summer holidays. We included 2,369 young people attending secondary schools in our research. For the analysis a special questionnaire has been used which did tested individuals at home answer. The collected data has been analysed with the calculation of Pearson's Chi2 coefficient, Spearman's coefficient of the correlation range and the discriminate analysis. The analysis has shown that the rhythm of young people's daily activity during the summer vacation is worrying. Roughly one fifth of secondary school children quite often go to sleep after 1 o' clock in the morning, and they start their day rather late. Young people spend too much time

lying in bed or in front of TV screens, and they are insufficiently physically as well as mentally active. We have also found out that only 22.1 % of secondary school youth practices sport every day during their summer vacation, and as much as 13.6 % never practice sport at all. Girls and boys differ in participation (p < .000), and their involvement in summer sports programmes is also different (p < .000). The analysis of the sports activity frequency of secondary school youth, implementing discriminate analysis, has shown that their participation is mostly influenced by their former experiences with sport, acquired at home (activity of their parents), and at school (opinion, self-esteem and their mark from physical education at school). The same characteristics also mostly differentiate young people among themselves during summer holidays when being involved in the sports programmes. The overview of sports activity participation indicates that boys prefer team sports (football, basketball), and girls more tend to select individual sports activities (athletics, aerobics, trekking) and other activities requiring less physical efforts (cycling, roller-skating, volleyball, badminton). Secondary school children also wish to participate in open area sports activities, quite often linked with other activities, where they do not express any reservations against sports exercises organised by schools during holidays, as long as they are organised in a way acceptable for them. According to the obtained issues we would like to suggest introducing a national project on putting lights to outdoor sports facilities and sports areas which would so enable secondary school children to organise sports activities by themselves with help of their mentors
SN - 03230511
UR -
https://login.proxy.bib.uottawa.ca/login?url=http://search.ebscohost.com/login.aspx?direct=true&db=s3h&AN=SPHS-930268&site=ehost-live;http://articles.sirc.ca/search.cfm?id=S-930268
L2 - http://articles.sirc.ca/search.cfm?id=S-930268
ER -

TY - JOUR
ID - 4531
T1 - Variations in Observed Park Physical Activity Intensity Level by Gender, Race, and Age: Individual and Joint Effects
A1 - Kaczynski,Andrew T.
A1 - Stanis,Sonja A.W.
A1 - Hastmann,Tanis J.
A1 - Besenyi,Gina M.
Y1 - 2011/08/02/
KW - SportDiscus
KW - *PHYSICAL activity
KW - *OUTDOOR recreation
KW - *GENDER
KW - *PARKS
KW - Research
KW - Regression Analysis
KW - AGE distribution (Demography)
KW - Demographic Characteristics
KW - PUBLIC use
KW - Built Environment
KW - environmental justice
KW - Observation
KW - parks
RP - NOT IN FILE
SP - S151
EP - S160
JF - Journal of Physical Activity & Health
JA - J Phys Act Health
N2 - Background: Parks are important settings for physical activity (PA), but few studies have documented the actual behaviors of park users. The purpose of this study was to examine the individual and joint effects of various park user demographic characteristics on observed PA intensity levels. Methods: Four parks were observed using the System for Observing Play and Recreation in Communities. Observers recorded the age group, gender, race, and intensity level of all park users in 83 activity areas over two weekends at each park. Logistic regression examined whether male/White, female/White, and male/non-White users were more likely

than female/non-White users to be observed engaging in moderate-to-vigorous PA (MVPA) rather than sedentary activity across 4 age groups. Results: In total, 8612 users were observed during the study. In the child age group, male/White users were significantly more likely to be observed in MVPA than female/non-White users. For teens, female/White and male/White users were less likely to engage in MVPA. For both adults and seniors, female/White and male/White users were more likely to be observed in MVPA. Conclusion: Observations revealed significant differences in intensity levels across gender, age, and race groups. Future interventions should emphasize park design that promotes increased MVPA among diverse groups.
ABSTRACT FROM AUTHOR
SN - 15433080
UR -
https://login.proxy.bib.uottawa.ca/login?url=http://search.ebscohost.com/login.aspx?direct=true&db=s3h&AN=66699162&site=ehost-live
ER -

TY - JOUR
ID - 4532
T1 - As a flower needs sunshine - the origins of organized children's recreation in Philadelphia, 1886-1911
A1 - Kadzielski,M.A.
Y1 - 1977///Summer
KW - SportDiscus
KW - *RECREATION
KW - *PLAYGROUNDS
KW - *CHARITIES
KW - History
KW - CITY planning
KW - Management
KW - POLITICAL science
KW - Urbanization
KW - PHILADELPHIA (Pa.)
KW - Child
RP - NOT IN FILE
SP - 169
EP - 188
JA - Journal of Sport History
VL - 4
IS - 2
CY - ;
N2 - Chronological description of the development of organized children's recreation in Philadelphia which relates it to the economic, social and political climate of the period. Analyzes the rationale advanced by proponents of organized play and points out it was considered necessity for the development of spiritually and physically healthy citizens
SN - 00941700
UR -
https://login.proxy.bib.uottawa.ca/login?url=http://search.ebscohost.com/login.aspx?direct=true&db=s3h&AN=SPH28919&site=ehost-live;http://articles.sirc.ca/search.cfm?id=28919
L2 - http://articles.sirc.ca/search.cfm?id=28919
ER -

TY - JOUR
ID - 4533
T1 - Recess, Extracurricular Activities, and Active Classrooms
A1 - Kahan,David
Y1 - 2008/02//
KW - SportDiscus
KW - *PHYSICAL education
KW - *PHYSICAL education for children
KW - *OUTDOOR recreation

KW - *ACTIVITY programs in education
KW - *SCHOOL children
KW - *HEALTH
KW - Elementary Education
KW - CLASSROOM activities
KW - TEACHER-student relationships
KW - United States
RP - NOT IN FILE
SP - 26
EP - 39
JA - JOPERD: The Journal of Physical Education, Recreation & Dance
VL - 79
IS - 2
N2 - The article discusses the importance of increasing the physical activity opportunities for elementary school students through recess, extracurricular activities, and active classroom in the U.S. In the research on physical activity (PA) and recess, the authors recommended 40% of playtime to be spent in moderate-to-vigorous physical activity (MVPA), however, they found that recess activity accounted for one-third of the 60 minutes per day of the MVPA recommendation. It also found that maximal school PA can be achieved in extracurricular programs when it is taught by a well-trained staff, and strong and unwavering administrative support. Moreover, it recommended that more attention should be given to classroom setting as a supplement to regular physical education class
SN - 07303084
UR -
https://login.proxy.bib.uottawa.ca/login?url=http://search.ebscohost.com/login.aspx?direct=true&db=s3h&AN=29990361&site=ehost-live
ER -

TY - JOUR
ID - 4535
T1 - Development of junior high school students' fundamental movement skills and physical activity in a naturalistic physical education setting
A1 - Kalaja,Sami Pekka
A1 - Jaakkola,Timo Tapio
A1 - Liukkonen,Jarmo Olavi
A1 - Digelidis,Nikolaos
Y1 - 2012/09//
KW - SportDiscus
KW - *PHYSICAL activity
KW - *PHYSICAL education
KW - *MUSCULOSKELETAL system
KW - Junior High School Students
KW - Educational Programs
KW - Finland
KW - Intervention
KW - movement skills
KW - Physical Activity
KW - school physical education
RP - NOT IN FILE
SP - 411
EP - 428
JA - Physical Education & Sport Pedagogy
VL - 17
IS - 4
N2 - Background: There is evidence showing that fundamental movement skills and physical activity are related with each other. The ability to perform a variety of fundamental movement skills increases the likelihood of children participating in different physical activities throughout their lives. However, no fundamental movement skill interventions implemented with junior high school students have yet been reported. Purpose: To

investigate the changes in students' locomotor, manipulative, and balance skills and their level of self-reported physical activity during the specific intervention program aiming to increase students' fundamental movement skills in Finnish junior high school physical education. Participants and setting: 446 Finnish Grade 7 students (ƒ^¬13 years old) from Central Finland. Research design: A quasi-experimental intervention study with pre-, middle-, post-, and retention tests was used. The experimental group consisted of 199 students taught by 4 teachers and the control group included 247 students taught by 6 teachers. The intervention consisted of 33 sessions, 25 minutes of each, and included training of fundamental movement skills within naturalistic physical education classes during one academic year. Data collection: There were four waves of measurement for fundamental movement skill tests and self-reports of physical activity. Data analysis: Repeated measures MANOVAs were conducted to analyze interactions between condition (experimental/control) and time (four measurement points). In cases with an interaction between condition and time, follow-up post hoc tests were performed to examine which group means differed from each other. Findings: Results indicated significant conditionƒ_%A-ƒ_%time interaction in static balance, dynamic balance, balance skills sum score, movement skills sum score, and self-reported physical activity. The experimental group demonstrated more positive development of these variables compared to the control group. Conclusions: This study revealed that it is possible to develop junior high school students' fundamental skills through physical education. These changes seem to be more obvious when focusing on students' balance skills. Although further longitudinal investigation is needed, the fundamental movement skill intervention seemed also to prevent the typical decline in physical activity within junior high school students. ABSTRACT FROM AUTHOR
SN - 17408989
UR -
https://login.proxy.bib.uottawa.ca/login?url=http://search.ebscohost.com/login.aspx?direct=true&db=s3h&AN=79343335&site=ehost-live
ER -

TY - JOUR
ID - 4536
T1 - Risk Factor Four: Equipment and Surfacing Maintenance on Safe Playgrounds
A1 - Kalinowski,Lyn Burdick
A1 - Bowler,Thomas
Y1 - 2000/04//
KW - SportDiscus
KW - *PLAYGROUNDS
KW - *EQUIPMENT & supplies
KW - MAINTENANCE & repair
KW - PLAY environments -- Safety measures
RP - NOT IN FILE
SP - 20
JA - JOPERD: The Journal of Physical Education, Recreation & Dance
VL - 71
IS - 4
N2 - Part II. Provides information for the development of a systematic inspection and maintenance program for playground equipment. Historical perspective on playground equipment; Safety of children on playground equipment; Development of a maintenance action plan
SN - 07303084
UR -
https://login.proxy.bib.uottawa.ca/login?url=http://search.ebscohost.com/login.aspx?direct=true&db=s3h&AN=3086907&site=ehost-live
ER -

TY - JOUR
ID - 4537
T1 - The architecture of children's relationships with nature: a phenomenographic investigation seen through drawings and written narratives of elementary students
A1 - Kalvaitis,Darius
A1 - Monhardt,Rebecca M.
Y1 - 2012/04//

N1 - Accession Number: 73326396; Kalvaitis, Darius 1; Email Address: dariusk@hawaii.edu; Monhardt, Rebecca M. 2; Affiliations: 1 : Education Department, University of Hawaiâ€™i Hilo, USA; 2 : Education Department, Loras College, USA; Source Info: Apr2012, Vol. 18 Issue 2, p209; Thesaurus Term: NATURE; Thesaurus Term: ENVIRONMENTAL education; Subject Term: CHILDREN; Subject Term: PHENOMENOGRAPHY; Subject Term: CONTENT analysis (Communication); Author-Supplied Keyword: children; Author-Supplied Keyword: constructivism; Author-Supplied Keyword: developmental; Author-Supplied Keyword: drawings; Author-Supplied Keyword: nature; Author-Supplied Keyword: phenomenography; Author-Supplied Keyword: relationship; Number of Pages: 19p; Document Type: Article
KW - Nature
KW - Environmental Education
KW - CHILDREN
KW - PHENOMENOGRAPHY
KW - CONTENT analysis (Communication)
KW - Constructivism
KW - Developmental
KW - drawings
KW - relationship
KW - Reviewer Nominated
RP - NOT IN FILE
SP - 209
EP - 227
JF - Environmental Education Research
VL - 18
IS - 2
N2 - How do young children portray and describe their relationships with nature? In what ways do young children's relationships with nature vary by grade level? These two research questions guided this phenomenographic study, which investigated developmental differences in children's relationships with nature. A total of 176 children, aged 6-1, from ten classrooms in grades 1â€“5 participated and were asked to â€˜draw pictures of themselves outside doing something and write about their picture and their relationship with natureâ€™. The draw and write methodology was employed within a constructivist framework. Drawings were analyzed using binary quantitative visual content analysis procedures and were further statistically investigated using SAS/STAT. Written narratives were analyzed with NVIVO 8 to further triangulate findings. Results indicated that at all grade levels, children demonstrated a positive relationship with nature. Variations were found between grade level groups with increasing or decreasing trends in the types of activities, settings, and people portrayed. Younger children included more family, friends, insects, and animals, while older children included more natural areas, chores, and hiking. The implications of considering the range of childrenâ€™s relationships with nature for environmental education are explored. [ABSTRACT FROM AUTHOR]
Copyright of Environmental Education Research is the property of Routledge and its content may not be copied or emailed to multiple sites or posted to a listserv without the copyright holder's express written permission. However, users may print, download, or email articles for individual use. This abstract may be abridged. No warranty is given about the accuracy of the copy. Users should refer to the original published version of the material for the full abstract. (Copyright applies to all Abstracts.)
SN - 13504622
M3 - Article
UR - http://search.ebscohost.com/login.aspx?direct=true&db=8gh&AN=73326396&site=ehost-live
ER -

TY - JOUR
ID - 4539
T1 - A critical study of sports talent selection and promotion of sports participation, at young age
A1 - Kansal,Devinder K.
Y1 - 2010/09/02/
KW - SportDiscus
RP - NOT IN FILE
SP - i65
EP - i66
JF - British Journal of Sports Medicine

JA - BJSM online
VL - 44
N2 - It has been a long drawn effort of the author to draw attention of the authorities to the importance and emphasis needed to be given to the question of most appropriate talent selection at young age for prospective excellence in sports. Unfortunately non-existence of graduation degree in sports coaching in India and wide spread unawareness, among departments and institutions of higher education in physical education and sports to the fields of physical growth, development, physique and physiological variations prevalent in India, children are left to select mostly sports unsuitable to their adult body potentials. The present study has been based on survey and critical analysis and new interpretations of existing research data on Olympic Champions (Carter 1984; Hirata 1979; Tanner 1964). In general, coaches and sports federations in India have been found to lack not only in the application of existing scientific principles but also in developing or maintaining any library or any literature on talent selection, or on measurement and evaluation even at any offices of national and state level sports associations/federations. The study has presented the scientific results on talent selection in a suitable form to make it easy to be used by the sports trainers and sports counsellors. Critical points of failure in the talent selection at young age have been dealt with scientifically along with a mapping of the long term plans and easy solutions for removing many hurdles currently in implementation of scientific preparation of Indian teams for national and international participation. Findings have also been reported to improve the number of sports participants in the country through proper talent selection at young age. The present population of sportspersons in India is extremely less, being less than 1% of the students up to middle schools and less than 10% of the students in colleges and universities. It has been proposed for better sports talent grooming that all high schools should be given sports infrastructure for training and participation in atleast one outdoor sport and one indoor sport/fitness activity. Each school must be accordingly required to participate in inter scholastic competitions in atleast these two sports or one sports and one fitness event. Prediction of adult physique at young age and scientific selection of most suitable sports has been presented in the present paper, to be presented in the Commonwealth Games 2010 Conference. It has also been explained that there is an urgent need to start an international CAKE (ie, Centre of Application of Knowledge Existing in the field of Sports research literature for preparing key guidelines for talent selection at young age). Sports participation will be increased many fold if children are guided properly to select a right sport as per their adult physiques and sports potentials. ABSTRACT FROM PUBLISHER
SN - 03063674
UR - https://login.proxy.bib.uottawa.ca/login?url=http://search.ebscohost.com/login.aspx?direct=true&db=s3h&AN=66325209&site=ehost-live
ER -

TY - JOUR
ID - 4540
T1 - Managing students with ADHD in out-of-class settings
A1 - Kapalka,G.M.
Y1 - 2008/03//
N1 - Accession Number: 2009923414. Language: English. Entry Date: 20080808. Revision Date: 20091218. Publication Type: journal article; research; tables/charts. Journal Subset: Biomedical; Blind Peer Reviewed; Europe; Peer Reviewed; UK & Ireland. Special Interest: Pediatric Care; Psychiatry/Psychology. Instrumentation: School Situations Questionnaire (SSQ) (Barkley). No. of Refs: 19 ref. NLM UID: 9616901
KW - CINAHL
KW - Attention Deficit Hyperactivity Disorder
KW - Behavior Modification -- In Infancy and Childhood
KW - Child Behavior Disorders -- Therapy
KW - Teachers
KW - Analysis of Variance
KW - Behavior Rating Scales
KW - Child
KW - Descriptive Statistics
KW - Exploratory Research
KW - Male
KW - New Jersey
KW - Pretest-Posttest Design

KW - Questionnaires
KW - Repeated Measures
KW - Rural Areas
KW - Human
RP - NOT IN FILE
SP - 21
EP - 30
JF - Emotional & Behavioural Difficulties
VL - 13
IS - 1
N2 - Many students with Attention-Deficit Hyperactivity Disorder (ADHD) present with behavioral problems that are particularly evident in out-of-class settings (in the lunch room, on the playground, during field trips and special assemblies, etc). Barkley's (1997) technique has been known to help parents handle ADHD children's behaviors in out-of-home situations, and so its effectiveness to reduce problems in out-of-class settings was investigated with 65 teachers of male students previously diagnosed with ADHD. ANOVA revealed that Barkley's technique was effective in reducing the students' behavioral problems in out-of-class settings. This technique is easy to administer and school psychologists will likely find it useful in assisting teachers to handle ADHD students' behavioral problems
SN - 1363-2752
AD - Department of Psychological Counseling, Monmouth University, West Long Branch, USA
UR - http://search.ebscohost.com/login.aspx?direct=true&db=cin20&AN=2009923414&site=ehost-live
ER -

TY - JOUR
ID - 4541
T1 - For the Kid in You
A1 - Kaspriske,Ron
Y1 - 2002/07//
KW - SportDiscus
KW - *GOLF
KW - *SWIMMING pools
KW - *PARKS
KW - United States
RP - NOT IN FILE
SP - 68
JA - Golf Digest
VL - 53
IS - 7
N2 - Features destinations for family golf trips in the U.S. Expenses for constructing the pool at Westin Innisbrook Resort in Florida; Measurement of the indoor and outdoor water park at Grand Geneva Resort & Spa in Wisconsin; Availability of a children's activity center at Kiawah Island Resort in South Carolina
SN - 0017176X
UR -
https://login.proxy.bib.uottawa.ca/login?url=http://search.ebscohost.com/login.aspx?direct=true&db=s3h&AN=6953253&site=ehost-live
ER -

TY - JOUR
ID - 4542
T1 - Educational intervention on water intake improves hydration status and enhances exercise performance in athletic youth
A1 - Kavouras,S.A.
A1 - Arnaoutis,G.
A1 - Makrillos,M.
A1 - Garagouni,C.
A1 - Nikolaou,E.
A1 - Chira,O.

A1 - Ellinikaki,E.
A1 - Sidossis,L.S.
Y1 - 2012/10//
KW - SportDiscus
KW - *DEHYDRATION (Physiology) -- Prevention
KW - *BASKETBALL
KW - *DRINKING (Physiology)
KW - *EXERCISE tests
KW - *HEAT
KW - *URINE
KW - *VOLLEYBALL
KW - *WATER
KW - *BODY movement
KW - OUTCOME assessment (Medical care) -- Methodology
KW - Analysis of Variance
KW - Comparative Studies
KW - Drinking Behavior
KW - Specific Gravity
KW - PROBABILITY theory
KW - Questionnaires
KW - RESEARCH -- Finance
KW - FIELD work (Research)
KW - DATA analysis -- Software
KW - adolescence
KW - CHILDREN
KW - dehydration
KW - environmental symptoms questionnaire
KW - fluid ingestion
KW - hydration state
KW - sport camp
KW - urine specific gravity
RP - NOT IN FILE
SP - 684
EP - 689
JF - Scandinavian Journal of Medicine & Science in Sports
JA - Scand J Med Sci Sports
VL - 22
IS - 5
N2 - We aimed to evaluate whether an intervention program emphasizing in increased fluid intake can improve exercise performance in children exercising in the heat. Ninety-two young athletes participated in the study (age: 13.8 Añ0.4 years, weight: 54.9 Añ1.5 kg). Thirty-one (boys: 13, girls: 18) children served as the control group (CON) and 61 (boys: 30, girls: 31) as the intervention (INT). Volunteers had free access to fluids. Hydration was assessed on the basis of first morning urine. A series of field tests were used to evaluate exercise performance. All tests occurred outdoors in the morning (mean ambient temperature=28 AøC). After baseline testing, INT attended a lecture on hydration, and urine color charts were mounted in all bathrooms. Additionally, water accessibility was facilitated in training, dining and resting areas. Hydration status was improved significantly in the INT [USG: pre=1.031 Añ0.09, post=1.023 Añ0.012, P<0.05; urine osmolality (mOsm/kg water): pre=941 Añ30, post=782 Añ34, P<0.05], while no statistically significant changes were found in the CON [USG: pre=1.033 Añ0.011, post=1.032 Añ0.013, P>0.05; urine osmolality (mOsm/kg water) 970 Añ38 vs 961 Añ38, P>0.05]. Performance in an endurance run was improved significantly only in INT (time for 600 m: pre=189 Añ5 s, post=167 Añ4 s, P<0.05). Improving hydration status by ad libitum consumption of water can enhance performance in young children exercising in the heat. ABSTRACT FROM AUTHOR
SN - 09057188
UR - https://login.proxy.bib.uottawa.ca/login?url=http://search.ebscohost.com/login.aspx?direct=true&db=s3h&AN=79956018&site=ehost-live

ER -

TY - JOUR
ID - 4546
T1 - Faces in the Crowd
A1 - Kennedy,Kostya
A1 - Bechtel,Mark
Y1 - 2003/11/24/
KW - SportDiscus
KW - *CHILD athletes
KW - *HIGH school athletes
KW - *SPORTS
KW - *SPORTS tournaments
KW - United States
RP - NOT IN FILE
SP - 35
EP - 35
JA - Sports Illustrated
VL - 99
IS - 20
N2 - This article focuses on the accomplishments of elementary and high school athletes in the United States. Javarris, a sophomore running back at Immokalee High, rushed for 1,151 yards on 175 carries and scored 14 touchdowns this season to lead the Indians to the state Class 2A playoffs. Huffman, a senior midfielder at Connecticut College, was named a National Field Hockey Coaches Association All-America for the second time. Davis, a fourth-grader at Center Hill Elementary, won gold medals in forms and sparring at the 2003 Junior Olympics & Masters Karate Championships in Myrtle Beach, S.C. It was his 28th gold medal in forms or sparring competitions this year. Adam, a senior at Quincy Senior High, won the Class AA state high school golf championship at The Den at Fox Creek in Bloomington. Bill, 17, a junior at the Oregon Episcopal School, won the men's sabre gold medal in the junior division (under-20) at the 2003 Junior & Cadet Pan American Fencing Championships in Guadeloupe
SN - 0038822X
UR -
https://login.proxy.bib.uottawa.ca/login?url=http://search.ebscohost.com/login.aspx?direct=true&db=s3h&AN=11452818&site=ehost-live
ER -

TY - JOUR
ID - 4547
T1 - WHO'S Hot/WHO'S Not
A1 - Kennedy,Kostya
Y1 - 2004/12/06/
KW - SportDiscus
KW - *SPORTS
KW - *FOOTBALL
KW - LISTS
KW - SCHOTTENHEIMER,Marty,1943-
RP - NOT IN FILE
SP - 41
EP - 41
JA - Sports Illustrated
VL - 101
IS - 22
N2 - This article presents a list of sports players, teams, and entities which are designated as being either hot or not hot. When does a two-game average of 125 passing yards make you hot in these gunslingin' times? When those yards lead to back-to-back wins for a Jets QB who was abruptly released by the Cowboys in August and who rebounded after being knocked ga-ga and missing three series on Sunday. More fun-on-a-bun: Bills coach Mike Mularkey had his team eat cheeseburgers last Saturday to change its luck out West. Marty Schottenheimer

is the odds-on choice for coach of the year after his Chargers beat his former team, the Chiefs, 34-31 on Sunday. Son Brian Schottenheimer(San Diego's QB coach) missed the game but still helped deliver a winner: His wife, Gemmi, had the couple's first child on Saturday
SN - 0038822X
UR -
https://login.proxy.bib.uottawa.ca/login?url=http://search.ebscohost.com/login.aspx?direct=true&db=s3h&AN=15236860&site=ehost-live
ER -

TY - JOUR
ID - 4548
T1 - Sports Beat
A1 - Kennedy,Kostya
A1 - Bechtel,Mark
Y1 - 2004/03/08/
KW - SportDiscus
KW - *SPORTS
KW - *OLYMPIC Games (28th : 2004 : Athens,Greece)
KW - JOLIE,Angelina,1975-
KW - TORCHES
KW - CUBAN,Mark,1958-
KW - WOODS,Tiger,1975-
KW - TELEVISION advertising
KW - MCGRADY,Tracy,1979-
RP - NOT IN FILE
SP - 24
EP - 24
JA - Sports Illustrated
VL - 100
IS - 10
N2 - This article presents several news briefs related to sports. Now that Billy Bob Thornton is out of the picture, Angelina Jolie is carrying a torch for someone else -- Juan Antonio Samaranch. Saying she would "consider it an honor to run with the Olympic torch as a gesture of hope for refugees and to express my active support for sport and world peace," the Oscar-winning actress will carry the flame for a few hundred yards in Athens on Aug. 12, the day before the Opening Ceremonies; In the March 9 "Queer Eye for the Straight Guy" the Fab Five will make over a guy who already has a wardrobe filled with sequined outfits: three-time national pairs figure skating champion John Zimmerman. The 30-year-old has been getting less than a perfect 6.0 from his new wife, Silvia Fontana, a former Italian women's figure-skating champion; For a man who's about to host a reality show, Mark Cuban is on a pretty unreal run. "In the span of seven months I will have enjoyed the birth of my first child, played against the Globetrotters, been KO'd in the World Wrestling Entertainment (WWE) and played in a baseball game with batting lessons from Steve Garvey," says the Mavericks' owner; Although he's never golfed with the Dalai Lama, Tiger Woods came close to reaching total consciousness by playing the role of Caddyshack's Carl Spackler in an American Express commercial that debuted Sunday; Skater Nancy Kerrigan recently recorded "Simply the Best" on a Tina Turner tribute album; Gatorade is planning to introduce an "ESPN-flavored" drink; Tracy McGrady, Orlando Magic guard, after being fined $10,000 for kicking the ball into the stands twice during a game: "That's a lot of money. I'll have to get me another autograph-signing session to pay that."
SN - 0038822X
UR -
https://login.proxy.bib.uottawa.ca/login?url=http://search.ebscohost.com/login.aspx?direct=true&db=s3h&AN=12416896&site=ehost-live
ER -

TY - JOUR
ID - 4549
T1 - Playgrounds
A1 - Kennedy,Mike

Y1 - 2006/06//
KW - SportDiscus
KW - *PLAYGROUNDS
KW - *EQUIPMENT & supplies
KW - *PREVENTION
KW - Risk Assessment
KW - SAFETY measures
KW - CHILDREN'S accidents
KW - United States
RP - NOT IN FILE
SP - 16
EP - 16
JA - American School & University
VL - 78
IS - 11
N2 - This article reports on the results of the risk factor survey of school, child-care and park playgrounds in the U.S., which was conducted by the National Program for Playground Safety. The report card determined that several areas of school playground safety warranted a failing grade because too few school playgrounds had supervision rules posted, playgrounds were lacking separate areas for different age groups and signage identifying appropriate ages and too much equipment was not free of splinters
SN - 00030945
UR - https://login.proxy.bib.uottawa.ca/login?url=http://search.ebscohost.com/login.aspx?direct=true&db=s3h&AN=21458732&site=ehost-live
ER -

TY - JOUR
ID - 4551
T1 - Research trends. Outdoor spider mites as asthma allergens: important in farming and nonfarming communities
A1 - Kim,Y.
A1 - Kim,Y.
Y1 - 2005/09//
N1 - Accession Number: 2009168343. Language: English. Entry Date: 20060526. Revision Date: 20090313. Publication Type: journal article; pictorial; review; tables/charts. Journal Subset: Biomedical; Continental Europe; Editorial Board Reviewed; Europe; Expert Peer Reviewed; Online/Print; Peer Reviewed. No. of Refs: 24 ref. NLM UID: 8915137. Email: youyoung@plaza.snu.ac.kr
KW - CINAHL
KW - Allergens
KW - Asthma -- Etiology
KW - Farmworkers
KW - Mites -- Classification
KW - Occupational Diseases -- Etiology
KW - adolescence
KW - Adult
KW - Aged
KW - Asthma -- Epidemiology
KW - Child
KW - Fruit
KW - Middle Age
KW - Mites -- Immunology
KW - Occupational Diseases -- Epidemiology
KW - Occupational Exposure
KW - Rural Areas
RP - NOT IN FILE
SP - 193
EP - 197

JA - Allergy & Clinical Immunology International
VL - 17
IS - 5
N2 - Backgound: Asthma induced by house dust mites is a mayor health problem worldwide. Spider mites are outdoor phytophagous mites and they have the severest economic effect in the agricultural sector; they also have a worldwide distribution. Although predatory mites attack spider mites, the current predator complex does not afford adequate control, particularly when spray organic phosphate or sulfur spray programs upset the natural balance. Environmental changes may have played a key role in the newly discovered development of spider mite-induced allergic diseases, especially asthma
Methods/Data base: A review of the literature
Results: Epidemiological studies have demonstrated that spider mites such as citrus red mite (CRM), European red mite (ERM), and two-spotted spider mite (TSM) are important allergens in the development of work-related asthma in fruit- cultivating farmers. Moreover, outdoor spider mites are not only common sensitizing allergens, but are also associated with the prevalence of asthma among children and nonfarming adults living in rural areas
Conclusion: in conclusion, these findings provide a rationale for including outdoor spider mites in tests conducted to identify causative allergens in patients with asthma and other allergic diseases
SN - 1097-1424
AD - Dept of Internal Medicine, Seoul National University College of Medicine, Youngondong 28, Chongnogu, Seoul 110-744, Korea
UR - http://search.ebscohost.com/login.aspx?direct=true&db=cin20&AN=2009168343&site=ehost-live
ER -

TY - JOUR
ID - 4553
T1 - Class of the Underclass
A1 - King,Peter
Y1 - 2004/09/13/
KW - SportDiscus
KW - *ROOKIE football players
KW - *FOOTBALL players
RP - NOT IN FILE
SP - 40
EP - 40
JA - Sports Illustrated
VL - 101
IS - 10
N2 - The article looks at five football rookies who weren't first-round picks, but will still have impact. So which rookies chosen after the first round will rise quickly to the top? Last year angular wideout Anquan Boldin, who was taken 54th by the Cardinals, ranked third in the league with 101 catches. Picked 101st by the Texans, rugged running back Domanick Davis finished 15th in rushing yardage with 1,031. Here are five first-year players with a chance to have a similar impact. Chris Snee: G, Giants (second round, 34th pick). One NFL East scout says he is already the best offensive lineman on the team. The 6'3", 314-pound Snee--who married New York coach Tom Coughlin's daughter in July after the two had had a child while at Boston College--earned the starting right guard spot early in camp with his strong run blocking and skill at pulling. Dontarrious Thomas: OLB, Vikings (second, 48th). He'll start on the weak side of the defense behind end Kenechi Udeze, the first-round choice out of USC. Thomas had only 5 1/2 sacks in four years at Auburn, but Minnesota believes he's strong enough at 241 pounds to bust up pass blocking and get to quarterbacks. Keary Colbert: WR, Panthers (second, 62nd). He'll battle veteran Ricky Proehl for playing time as a third receiver. Darnell Dockett: DE, Cardinals (third, 64th). At 301 pounds he was chosen as a tackle, but because of his ability to pressure the quarterback and injuries on the line, Arizona shifted him to end midwaythrough the preseason. Bobby McCray: DE, Jaguars (seventh, 249th). The 6'6" 245-pounder from Florida was so impressive in training camp that Jacksonville cut former AllPro Hugh Douglas to ensure McCray playing time
SN - 0038822X
UR -
https://login.proxy.bib.uottawa.ca/login?url=http://search.ebscohost.com/login.aspx?direct=true&db=s3h&AN=14363851&site=ehost-live
ER -

TY - JOUR
ID - 4557
T1 - A Case of a Buccal Abscess through Trauma Caused by Soccer
A1 - Kondo,Keisuke
A1 - Maruoka,Yasubumi
A1 - Matsui,Shohei
A1 - Kobayashi,Marina
A1 - Aoyama,Keita
A1 - Ito,Yuki
A1 - Masuda,Issei
A1 - Myers,Mie
A1 - Horie,Norio
A1 - Shimoyama,Tetsuo
A1 - Sano,Haruo
Y1 - 2012/08//
KW - SportDiscus
KW - *MOUTH
KW - *WOUNDS & injuries
KW - *SOCCER injuries
KW - *DENTISTRY
KW - *SPORTS
KW - abscess
KW - Child
KW - contact sports
KW - oral infection
KW - oral trauma
KW - Soccer
RP - NOT IN FILE
SP - 20
EP - 22
JA - Japanese Journal of Sports Dentistry
VL - 16
IS - 1
N2 - Recently, more people are playing sports to improve their health, while children also actively take part in sports activities and other events. Here we report on the case of a patient who developed a buccal abscess through dental trauma caused by soccer. The patient's right upper lip was lacerated by a kick to his face after he fell over during a game of soccer. Two days after the injury, he visited our office because of swelling from the upper lip to the buccal region. We administered antiphlogistic treatment, and after 10 days he showed a healing tendency. Trauma in the outdoors caused by specific bacteria has the potential to be become severe, so a rapid response is necessary. ABSTRACT FROM AUTHOR
SN - 1344140X
UR -
https://login.proxy.bib.uottawa.ca/login?url=http://search.ebscohost.com/login.aspx?direct=true&db=s3h&AN=82609195&site=ehost-live
ER -

TY - JOUR
ID - 4558
T1 - The Association Between the Type, Context, and Levels of Physical Activity Amongst Adolescents
A1 - Koorts,Harriet
A1 - Mattocks,Calum
A1 - Ness,Andy R.
A1 - Deere,Kevin
A1 - Blair,Steven N.
A1 - Pate,Russell R.
A1 - Riddoch,Chris

Y1 - 2011/11//
KW - SportDiscus
KW - *PHYSICAL activity
KW - TEENAGERS with disabilities
KW - HEALTH behavior in adolescence
KW - CROSS-sectional method
KW - LONGITUDINAL method
KW - Accelerometry
KW - Epidemiology
KW - Longitudinal Studies
KW - Questionnaires
KW - school and nonschool
RP - NOT IN FILE
SP - 1057
EP - 1065
JF - Journal of Physical Activity & Health
JA - J Phys Act Health
VL - 8
IS - 8
N2 - Background: Little is known about how the type and context of physical activity behaviors varies among adolescents with differing activity levels. The aim of this study was to assess differences in the type and context of physical activity behaviors in adolescents by level of objectively measured physical activity. Methods: Cross-sectional analysis of 2728 adolescents (1299 males, 1429 females) participating in the Avon Longitudinal Study of Parents and Children (ALSPAC). The mean (SD) age was 13.8 (+0.1) years. Physical activity was measured using an Actigraph over 7 days. Adolescents were categorized into tertiles of activity (less, moderately, highly active) using counts/mm and min/d of moderate-to-vigorous activity (MVPA). Activity type was reported using the Previous Day Physical Activity Recall (PDPAR). Differences in the type and context of activity by activity level were analyzed using Chi squared. Results: Highly active boys reported more job, outside, and sports activities on school days (P < .05), and more sports activities on nonschool days (P < .05). Highly active girls reported more outside activities on school days (P < .05). Conclusions: Identifying the type and context of physical activity behaviors associated with more active adolescents, can help inform policy and physical activity interventions aimed at increasing activity levels in adolescents. ABSTRACT FROM AUTHOR
SN - 15433080
UR -
https://login.proxy.bib.uottawa.ca/login?url=http://search.ebscohost.com/login.aspx?direct=true&db=s3h&AN=70465929&site=ehost-live
ER -

TY - JOUR
ID - 4559
T1 - Predictors of back pain in a general population cohort... including commentary by Gerr F and Zwerling C
A1 - Kopec,J.A.
A1 - Sayre,E.C.
A1 - Esdaile,J.M.
Y1 - 2004/01/01/
N1 - Accession Number: 2004132493. Language: English. Entry Date: 20040806. Revision Date: 20100122. Publication Type: journal article; commentary; research; tables/charts. Journal Subset: Allied Health; Biomedical; Peer Reviewed; USA. Special Interest: Pain and Pain Management; Physical Therapy. Grant Information: Arthritis Society of Canada. No. of Refs: 44 ref. NLM UID: 7610646. Email: jkopec@bc.arthritis.ca
KW - CINAHL
KW - Back Pain
KW - chronic pain
KW - Forecasting
KW - Models,Theoretical
KW - Male
KW - Female

KW - Prospective Studies
KW - Epidemiological Research
KW - Step-Wise Multiple Regression
KW - Logistic Regression
KW - Back Pain -- Epidemiology
KW - Sex Factors
KW - statistical significance
KW - Age Factors
KW - Middle Age
KW - Adult
KW - Body Weights and Measures
KW - Health Status -- Evaluation
KW - Self Report
KW - Physical Activity -- Evaluation
KW - Horticulture
KW - Stress
KW - Arthritis
KW - Back Pain -- Psychosocial Factors
KW - Critical Incident Stress -- In Infancy and Childhood
KW - Critical Incident Stress -- In Adolescence
KW - Child
KW - adolescence
KW - Canada
KW - probability sample
KW - ROC Curve
KW - Data Analysis Software
KW - Aged
KW - Odds Ratio
KW - Confidence Intervals
KW - Body Mass Index -- Evaluation
KW - Descriptive Statistics
KW - Funding Source
KW - Human
RP - NOT IN FILE
SP - 70
EP - 78
JF - Spine
VL - 29
IS - 1
N2 - STUDY DESIGN: The study used longitudinal data from the first and second cycles (1994-1995 and 1996-1997) of the Canadian National Population Health Survey. OBJECTIVE: Our objective was to derive prediction models for back pain in the general male and female household populations. SUMMARY OF BACKGROUND DATA: Little is known about the predictors of back pain in the general population. Most previous studies focused on specific occupational groups and used a cross-sectional or case-control design. METHODS: The study cohort consisted of all respondents aged 18+ years who reported no back problems in the 1994-1995 National Population Health Survey cycle (N = 11,063). Potential predictors of chronic back pain were classified into nine groups and entered into stepwise logistic regression models. Bootstrap methods were used to derive the final models and assess their predictive power. RESULTS: The overall incidence of back pain was 44.7 per 1,000 person-years and was higher in women (47.0 per 1,000 person-years) compared with men (42.2 per 1,000 person-years). In men, significant predictors of back pain were age (peak effect in 45-64 years), height, self-rated health, usual pattern of activity (especially heavy work), yard work or gardening (negative association), and general chronic stress. In women, significant factors were self-reported restrictions in activity, being diagnosed with arthritis, personal stress, and history of psychological trauma in childhood or adolescence. CONCLUSIONS: Overall health and psychosocial factors are important predictors of back pain in both men and women. Other risk factors differ between the two sexes
SN - 0362-2436
AD - Arthritis Research Centre of Canada, 895 West 10th Avenue, Vancouver, BC V5Z 1L7, Canada

UR - http://search.ebscohost.com/login.aspx?direct=true&db=cin20&AN=2004132493&site=ehost-live
ER -

TY - JOUR
ID - 4560
T1 - Costs of health care for unintentional injury in Stavanger, Norway
A1 - Kopjar,B.
Y1 - 1997/09//
N1 - Accession Number: 1998015657. Language: English. Entry Date: 19980301. Revision Date: 20091218. Publication Type: journal article; research; statistics; tables/charts. Journal Subset: Biomedical; Europe; Peer Reviewed; Public Health; UK & Ireland. Grant Information: Grant from the Norwegian Research Council. No. of Refs: 29 ref. NLM UID: 9204966
KW - CINAHL
KW - Accidents -- Economics -- Norway
KW - Health Services -- Utilization
KW - Norway
KW - Prospective Studies
KW - Retrospective Design
KW - Record Review
KW - Kruskal-Wallis Test
KW - Analysis of Variance
KW - Regression
KW - Age Factors
KW - Hospitalization -- Economics -- Norway
KW - Infant
KW - Child,Preschool
KW - Child
KW - adolescence
KW - Adult
KW - Middle Age
KW - Aged
KW - Male
KW - Female
KW - Funding Source
KW - Human
RP - NOT IN FILE
SP - 321
EP - 327
JF - European Journal of Public Health
JA - Eur J Public Health
VL - 7
IS - 3
N2 - The objective was to analyse the utilization of health services and the costs of treatment of unintentional injuries by type of injuries according to the common Nordic classification of injuries. From prospective registration of all injuries seen at the only hospital and emergency clinic in Stavanger, Norway, we selected at random a stratified sample of 2,819 cases from 7,019 unintentional injuries that occurred in 1992 among the residents of Stavanger. From medical records, we obtained information about the utilization of health services and estimated medical costs during the first year after injury. Of the patients 70% required only a single out-patient visit, while an additional 20% completed their treatment within the first 30 days after injury. Fifty per cent of the total costs were due to injuries among persons aged 65 years and older. Home and leisure-time injuries accounted for 75% of the total costs. The cost per injury was NOK 3,807 (US$ 614), NOK 24,831 per hospitalized patient and NOK 1,011 per non-hospitalized patient. The per-injury cost among people aged 65 years and older was NOK 15,428, compared with NOK 2,158 among people aged 0-64 years, the difference primarily due to the increase in the average severity of injury. The per-injury costs were ranked (in descending order): nursing home, undefined, street, home, other home and leisure, traffic, occupational, free nature, sports, school and day care centre/playground injuries. If health care costs are to be reduced by injury prevention,

priority should be given to injuries occurring at home and during leisure time and to injuries among elderly people
SN - 1101-1262
AD - National Institute of Public Health, Department of Population Health Sciences, Geitmyrsveien 75, 0462 Oslo, Norway
UR - http://search.ebscohost.com/login.aspx?direct=true&db=cin20&AN=1998015657&site=ehost-live
ER -

TY - JOUR
ID - 4561
T1 - The Silver Drawing Test of Cognition and Emotion: standardization in Russia
A1 - Kopytin,A.
Y1 - 2002/05//
N1 - Accession Number: 2002077266. Language: English. Entry Date: 20020726. Revision Date: 20091218. Publication Type: journal article; research; tables/charts. Journal Subset: Allied Health; Double Blind Peer Reviewed; Editorial Board Reviewed; Expert Peer Reviewed; Peer Reviewed; USA. No. of Refs: 7 ref. NLM UID: 0237447
KW - CINAHL
KW - Art Therapy
KW - instrument validation
KW - Validation Studies
KW - Child
KW - adolescence
KW - Adult
KW - Middle Age
KW - Female
KW - Male
KW - Emotions
KW - Russia
KW - Estonia
KW - United States
KW - Age Factors
KW - Sex Factors
KW - Self Concept
KW - Human
RP - NOT IN FILE
SP - 223
EP - 237
JA - American Journal of Art Therapy
VL - 40
IS - 4
N2 - Until recently care settings for those with Alzheimer's disease have not focused on the outside environment and the need to encourage older adults to spend time outside in fresh air and sunshine. Safe, secure outside spaces encourage walking and other forms of exercise and, in addition, offer beneficial exposure to increased natural light levels. Gardens and other outdoor settings provide many opportunities for socialization, sensory stimulation, fun, and meaningful activities. Designing the outside environment has been an overlooked opportunity to create meaningful places that are rich in association, encourage good health and exercise, and are responsive to the magic of the changing seasons
SN - 0007-4764
UR - http://search.ebscohost.com/login.aspx?direct=true&db=cin20&AN=2002077266&site=ehost-live
ER -

TY - JOUR
ID - 4563
T1 - Children at play: behavior of children at recess
A1 - Kraft,R.E.
Y1 - 1989/04//

KW - SportDiscus
KW - *PHYSICAL education & training
KW - *CARDIOVASCULAR fitness
KW - *STRESS (Physiology)
KW - Elementary Schools
KW - Child
KW - behaviour
RP - NOT IN FILE
SP - 21
EP - 24
JA - JOPERD: The Journal of Physical Education, Recreation & Dance
VL - 60
IS - 4
CY - ;
N2 - Questions the extent to which school recess periods are contributing to the fitness needs of children. Observes the extent of activity and types of behaviour engaged in by children (n=369) during unstructured play time at five elementary schools. Classifies the observed behaviours as either active (vigorous activity, moderate activity, or minimal activity) or passive (social interaction, independent behaviour, and waiting). Results indicate that the children are involved in some degree of physical play about 59% of the time. However, only 21% of recess time (i.e. about six minutes) is devoted to vigorous physical activity. Racial differences are minimal. Points out evidence which shows that the average elementary school child spends only two to three minutes in moderate to vigorous exercise during a physical education class. Suggests that the activity patterns during recess and physical education classes are the same, and that physical education programs do not provide the appropriate activities to adequately promote fitness, which could be carried over to recess
SN - 07303084
UR -
https://login.proxy.bib.uottawa.ca/login?url=http://search.ebscohost.com/login.aspx?direct=true&db=s3h&AN=SPH233599&site=ehost-live;http://articles.sirc.ca/search.cfm?id=233599
L2 - http://articles.sirc.ca/search.cfm?id=233599
ER -

TY - JOUR
ID - 4565
T1 - Rural Elementary School Strategies to Promote Outdoor Physical Activity
A1 - Krawiec,Kelly
A1 - King,Kristi McClurg
Y1 - 2013///Spring
KW - SportDiscus
KW - *PHYSICAL activity
KW - *OUTDOOR recreation
KW - *HEALTH promotion
KW - RURAL schools
KW - Elementary Schools
RP - NOT IN FILE
SP - 46
EP - 46
JA - KAHPERD Journal
VL - 50
IS - 2
N2 - The purpose of this abstract is to present a preliminary grant proposal outline for a rural, low-income elementary school that was the recipient of a Carol M. White Physical Education Program (PEP) award. Since the mission of the elementary school is to provide high quality educational and service programs that support, empower, and build capacity in school learning communities, the school must ensure that its students achieve their highest academic, social, and emotional potential. Based on two years of physical activity (pedometry) and cardiovascular fitness (PACER test) improvements seen due to the implementation of a variety of health and fitness interventions, a new intervention to ensure the sustainability of health progress is being proposed. The mission of the "Outdoor Walking Trail" intervention is to provide the elementary school community with an

outdoor walking trail that allows families a safe place to play outside while developing an appreciation of a healthy, outdoor physically active lifestyle. The goals of the intervention are to increase children's outdoor physical activity frequency and duration, increase children's physical activity self-efficacy, and to promote the use of the safe, outdoor walking trail for children and their families. The intervention is framed within the Social Cognitive Theory and measurement of the effectiveness of the intervention will include subjective measures of physical activity knowledge and self-efficacy and perceived access to physical activity opportunities (via surveys) and objective measures of physical activity participation (via pedometry). The intervention grant proposal is being developed by students enrolled in the HSS 434: Public Health Education Methods course within the Public Health Education and Community Health Program at University of Louisville and will be offered to the elementary school by the students and faculty mentor as part of their university/community engagement partnership. ABSTRACT FROM AUTHOR
SN - 10712577
UR -
https://login.proxy.bib.uottawa.ca/login?url=http://search.ebscohost.com/login.aspx?direct=true&db=s3h&AN=88160581&site=ehost-live
ER -

TY - JOUR
ID - 4566
T1 - School's out: so who watches the kids?
A1 - Kreisher,K.
Y1 - 2001/07//
N1 - Accession Number: 2004189844. Language: English. Entry Date: 20041126. Publication Type: journal article; pictorial. Journal Subset: Allied Health; USA. No. of Refs: 2 ref. NLM UID: 9301128
KW - CINAHL
KW - Child Day Care
KW - Child Welfare
KW - adolescence
KW - California
KW - Child
KW - Child Day Care -- Economics
KW - Community Programs
KW - District of Columbia
KW - Massachusetts
KW - Parents
RP - NOT IN FILE
SP - 24
JA - Children's Voice
VL - 10
IS - 4
N2 - Many of us fondly remember long, steamy summer days spent exploring the neighborhood, playing with friends, or spending time at the local swimming pool. Tim Esons, Director of School-Age Programs at South Shore Day Care Services, Weymouth, Massachusetts, shares similar memories. "During the summers, we spent our free time in the neighborhood with other kids and parents; but now, because parents are working or neighborhoods aren't safe. we are their neighborhood, he says, referring to the eight-week summer program offered by South Shore
The face of summer has changed across the country as the number of single-parent and dual-income families has increased. In 1996, 77% of married mothers with school-age children worked outside the home, compared with 40% in 1960. Mothers today are also more likely to work full time
An April 2000 report from the U.S. Departments of Education and Justice revealed that in 69% of all married-couple families with children ages 6-17, both parents work outside the home. The custodial parent works in 71% of single-mother families and 85% of single-father families. "It's hard to create a neighborhood now when no one is home in the afternoon," Esons says
SN - 1057-736X
UR - http://search.ebscohost.com/login.aspx?direct=true&db=cin20&AN=2004189844&site=ehost-live
ER -

TY - JOUR
ID - 4571
T1 - The effects of the Norsk Funktion-walking orthosis on the walking ability of children with cerebal palsy and severe gait impairment
A1 - Kuenzle,C.
A1 - Brunner,R.
Y1 - 2009/07//
N1 - Accession Number: 2010374210. Language: English. Entry Date: 20090911. Revision Date: 20091218. Publication Type: journal article; pictorial; research; tables/charts. Journal Subset: Allied Health; Double Blind Peer Reviewed; Editorial Board Reviewed; Expert Peer Reviewed; Peer Reviewed; USA. Special Interest: Pediatric Care; Physical Therapy. Instrumentation: Gross Motor Function Classification System (GMFCS); Functional Independence Measure for Children (WeeFIM). Grant Information: This study was supported by eo-Funktion, Switzerland, in planning and carrying it out and by Eling D. de Bruin, PhD, Institute of Human Movement Sciences, Swiss Federal Institute of Technology, Zurich.. No. of Refs: 15 ref. NLM UID: 8903233. Email: christoph.kuenzle@kispisg.ch
KW - CINAHL
KW - Cerebral Palsy -- Complications
KW - Cerebral Palsy -- Physiopathology
KW - Orthoses
KW - Walking
KW - adolescence
KW - Balance,Postural
KW - chi square test
KW - Child
KW - Child,Preschool
KW - Clinical Assessment Tools
KW - Data Analysis Software
KW - Female
KW - Funding Source
KW - Male
KW - Nonexperimental Studies
KW - outcome assessment
KW - Prospective Studies
KW - Questionnaires
KW - Torso
KW - Two-Way Analysis of Variance
KW - Wilcoxon Rank Sum Test
KW - Human
RP - NOT IN FILE
SP - 138
EP - 144
JA - Journal of Prosthetics & Orthotics (JPO)
VL - 21
IS - 3
N2 - The purpose of this study was 1) to test whether an orthosis, which provides postural stability of the trunk and guides the leg movements during walking (Norsk Funktion-walker orthosis [NFWO]), would enable nonambulant children with cerebral palsy with poor or no leg coordination and with little or no trunk control to walk on their own, 2) to investigate if there is an increase in motor function and activity while using a NFWO, and 3) defining requirements for a successful provision. Ninety-three children (39 girls, 54 boys; mean age 7.6 years; 67 with bilateral spastic, 10 with dyskinetic, 10 with mixed, 6 with ataxic cerebral palsy; Gross Motor Function classification System level 4: 45, level 5: 48) were provided with a NFWO. The following assessments were carried out immediately before and 3 months after using the NFWO: WeeFIM walking score; independence rating by parents or caregivers; aims or expectations of parents or caregivers; at a mean interval of 265 days after provision: mean daily walking distance (meters). With the NFWO, 78 children (84%) became ambulatory, 10 children (11%) used it exclusively as a dynamic standing frame only, and 5 children (5%) returned the NFWO. The mean daily walking distance was 99 m (2-463 m). The mean WeeFIM walking score of 1.99 (SD 0.83) without the NFWO increased to 4.42 (SD 1.00) with the NFWO indoors and to 3.71 (SD 1.24)

(p < 0.001) outdoors. Independence rating by parents or caregivers reflected a highly significant increase in independent mobility with the NFWO compared with locomotion without ambulatory aides (p < 0.001) and of bilateral hand function (p < 0.001). No significant increase in the mobility was found when comparing former mobility aides (wheelchair, tricycle) with the NFWO. The ability to cross obstacles did neither significantly increase with the NFWO. Successful indication for a NFWO depends on 1) the child's motivation to walk and the support of the environment to achieve independent walking mobility through the use of this assistive tool, 2) the ability for selective reciprocal leg movements, and 3) no flexion contractures of hips and knees above 20[degrees] and a foot dorsiflexion of at least neutral-0[degrees]. The NFWO proved to be a useful ambulatory aid in the children with cerebral palsy with severe gait impairment to increase independent mobility
SN - 1040-8800
AD - Department of Rehabilitation and Development, East Swiss Children's Hospital, Claudiusstr. 6, CH-9006 Saint Gallen, Switzerland
UR - http://search.ebscohost.com/login.aspx?direct=true&db=cin20&AN=2010374210&site=ehost-live
ER -

TY - JOUR
ID - 4573
T1 - A Survey of Maxillofacial and Oral Injuries of Elementary School Pupils and Junior High School Students in Iwate Prefecture in One Year from April 2003 to March 2004
A1 - Kumagai,Keiji
A1 - Hirai,Tohei
A1 - Kobayashi,Takuzoh
A1 - Yamada,Masao
A1 - Maekawa,Hiroshi
A1 - Suzuki,Takuya
A1 - Suzuki,Shunichi
Y1 - 2006/03//
KW - SportDiscus
KW - *CHILDREN -- Health
KW - *PHYSICAL education -- Safety measures
KW - *STUDENTS
KW - *MOUTH protectors
KW - *ATHLETICS -- Equipment & supplies
KW - Research
KW - Elementary Education
KW - elementary school pupils
KW - Iwate prefecture
KW - Junior High School Students
KW - maxillofacial and oral injuries
KW - questionnaire
KW - sports dentistry
RP - NOT IN FILE
SP - 13
EP - 19
JA - Japanese Journal of Sports Dentistry
VL - 9
IS - 1
N2 - The Iwate Dental Association has made various efforts to establish how to maintain oral health care for children. This article reports an investigation into the conditions of maxillofacial and oral injuries in order to keep children safe when playing sports at elementary and junior high schools in Iwate Prefecture from 2003 to 2004. The results were as follows?? 1. In the one year which we examined, 746 cases of injury occurred at 62 elementary schools out of a total of 90, and 187 cases of injury at 47 junior high schools out of a total of 72. Boys sustained injuries more frequently than girls both at elementary schools and at junior high schools. 2. First?? t year pupils sustained injuries most frequently, and the risk frequencies of injury lowered with the progress in school year for elementary school pupils. For junior high school students of each school year, injuries occurred at similar frequencies. 3. The incidence of injury was high in June at elementary schools and in May at junior high schools. The period of time during the day when injuries happened most frequently was

12.00 to 14.00 at elementary schools and 16.00 to 18.00 at junior high schools. The places where injuries happened frequently were the school playground, gymnasium and classroom for elementary school pupils and the school playground and gymnasium for junior high school students. Injuries in elementary school pupils happened frequently at recess, and those in junior high school students happened frequently during extracurricular activities. 4. Frequently sustained injuries were face contusion, lip lesion, tooth fracture, tooth luxation and tooth displacement both for elementary school pupils and for junior high school students??for junior high school students, serious injuries such as brain concussion, cervical vertebrae injury, and mandibular fracture also occurred. When injured, junior high school students were more likely than elementary school pupils to go to hospital. ABSTRACT FROM AUTHOR
SN - 1344140X
UR -
https://login.proxy.bib.uottawa.ca/login?url=http://search.ebscohost.com/login.aspx?direct=true&db=s3h&AN=25847234&site=ehost-live
ER -

TY - JOUR
ID - 4576
T1 - Games on a Polynesian Outlier Island: A Case Study of the Implications of Cultural Change
A1 - Kuschel,R.
Y1 - 1975///
KW - SportDiscus
KW - *GAMES
KW - *LEISURE
KW - *LIFE style
KW - *PARTICIPATION
KW - RELIGIONS
KW - SOCIAL history
KW - Child
RP - NOT IN FILE
SP - 25
EP - 66
JA - Journal of the Polynesian Society
VL - 84
IS - 1
N2 - Presents a description of games and play activities which were prevalent among children and adults on Bellana, an island in the British Solomon Islands, before contact with the outside world in 1938. Data were collected through interviews and participant observation. Points to radical increase in population and change in residential form, plus the judgement of others, especially missionaries, of the Bellonese culture as inferior, as determinants of the gradual submergence of this part (i.e. games) of the Bellonese culture. Changes in game forms have also affected traditional patterns of settling political disputes. Net result has been a serious break in the continuity of the cultural tradition
SN - 00324000
UR -
https://login.proxy.bib.uottawa.ca/login?url=http://search.ebscohost.com/login.aspx?direct=true&db=s3h&AN=SPH332005&site=ehost-live;http://articles.sirc.ca/search.cfm?id=332005
L2 - http://articles.sirc.ca/search.cfm?id=332005
ER -

TY - JOUR
ID - 4577
T1 - Play it safe
A1 - Kutska,Ken
Y1 - 1996/07//
KW - SportDiscus
KW - *PLAYGROUNDS
KW - *CHILDREN -- Health
KW - SAFETY measures

KW - United States
RP - NOT IN FILE
SP - 56
JA - American Fitness
VL - 14
IS - 4
N2 - Offers advice for parents on how to keep children safe on playgrounds. Checking playgrounds for potential hazards; Playground safety tips from the National Recreation and Park Association and its National Playground Safety Institute; Estimated number of children who are injured on playgrounds each year in the United States
SN - 08935238
UR -
https://login.proxy.bib.uottawa.ca/login?url=http://search.ebscohost.com/login.aspx?direct=true&db=s3h&AN=9608010474&site=ehost-live
ER -

TY - JOUR
ID - 4578
T1 - Feeling Tired?: How Sharing Positive Experiences Can Boost Vitality
A1 - Lambert,Nathaniel
A1 - Gwinn,A.Marlea
A1 - Stillman,Tyler F.
A1 - Fincham,Frank D.
Y1 - 2011///
KW - Reviewer Nominated
RP - NOT IN FILE
JA - International Journal of Wellbeing
VL - 1
IS - 3
SN - 1179-8602
ER -

TY - JOUR
ID - 4580
T1 - The Central Harlem Playground Injury Prevention Project: A Model for Change
A1 - Laraque,Danielle
A1 - Barlow,Barbara
A1 - Davidson,Leslie
A1 - Welborn,Charles
Y1 - 1994/10//
KW - SportDiscus
KW - *CHILDREN -- Wounds & injuries
KW - *PREVENTION
KW - *PLAYGROUNDS
KW - *HOSPITALS
KW - CHILDREN'S accidents
KW - SAFETY measures
KW - HARLEM (New York,N.Y.)
KW - NEW York (N.Y.)
RP - NOT IN FILE
SP - 1691
EP - 1691
JF - American Journal of Public Health
JA - Am J Public Health
VL - 84
IS - 10

N2 - The article provides information on the Harlem Hospital Playground Injury Prevention Program (HHPIPP), a project established by the Departments of Pediatric Surgery and Pediatrics at Harlem Hospital in New York City in 1988. The objectives of the program are to decrease the number of injuries to children in playgrounds in Central Harlem and to provide safe playgrounds. The article first explores the prevalence of playground injuries in the city predominantly of African American residents. The program's planning process, which is a multifaceted approach, is then discussed. The article also details how the HHPIPP teamed up with parents in the community to form the Safe Kids/Healthy Neighborhood Coalition
SN - 00900036
UR -
https://login.proxy.bib.uottawa.ca/login?url=http://search.ebscohost.com/login.aspx?direct=true&db=s3h&AN=9411030313&site=ehost-live
ER -

TY - JOUR
ID - 4581
T1 - Effects of an Environmental Education Program on the Environmental Orientations of Children from Different Gender, Age, and Ethnic Groups
A1 - Larson,Lincoln R.
A1 - Castleberry,Steven B.
A1 - Green,Gary T.
Y1 - 2010///Fall
KW - SportDiscus
KW - *OUTDOOR education
KW - *CHILDREN
KW - *EDUCATION
KW - Environmental Education
KW - Georgia
KW - United States
KW - CHILDREN
KW - eco-affinity
KW - eco-awareness
KW - environmental orientations
KW - ethnicity
RP - NOT IN FILE
SP - 95
EP - 113
JA - Journal of Park & Recreation Administration
VL - 28
IS - 3
N2 - Concerns regarding the profound physical, social, and psychological problems associated with childhood nature deprivation have catalyzed extensive growth in nature-based education programs. Recent environmental education (EE) initiatives and legislative measures, such as the proposed No Child Left Inside Act, have stressed the importance of EE in the formal education sector. However, out-of-school EE programs remain an appealing alternative to in-class science education. Non-formal programs provide children with a unique opportunity to experience substantial outdoor immersion and often influence affective development. These benefits suggest that an expansion of the depth and scope of non-formal outdoor programs is necessary to achieve the long-term goal of an environmentally literate population. Efforts to develop strategies for implementing and evaluating affective changes associated with EE programs could place a greater emphasis on the environmental orientations of children from different backgrounds. This exploratory study employed a new instrument, the Children's Environmental Perceptions Scale, to investigate baseline differences in the environmental orientations of 133 six to thirteen-year-old children from different gender, age, and racial/ethnic groups in Athens-Clarke County, Georgia. This study used a mixed-method, pre-test, post-test approach to examine the effects of a one-week EE summer program sponsored by the State Botanical Garden of Georgia on children's eco-affinity, eco-awareness, and environmental knowledge. Responses of EE program participants were compared to a control group of students enrolled in local after-school programs. Brief personal interviews and open-ended evaluations supplemented quantitative survey data. Results did not reveal gender differences in baseline environmental orientations. However, eco-affinity levels were significantly lower in participants age ten or older. African

American children also displayed significantly lower eco-awareness and environmental knowledge scores than white children prior to the EE program. Interviews detailing children's unique interactions with nature yielded several possible explanations for these discrepancies. The EE program produced significantly higher adjusted mean eco-affinity and environmental knowledge post-test scores than the control group, regardless of gender, age, or race/ethnicity. The summer camp's influence on eco-affinity, an affective component of environmental orientations that is difficult to alter, was especially encouraging. Fun and exciting concepts expressed through physical activity appeared to be a key component of effective EE programming. Overall, results suggested that non-formal EE programs in public parks and other recreation areas provide an ideal forum for stimulating positive environmental orientations in a diverse group of children and may generate future support for outreach efforts in underserved communities. ABSTRACT FROM AUTHOR
SN - 07351968
UR -
https://login.proxy.bib.uottawa.ca/login?url=http://search.ebscohost.com/login.aspx?direct=true&db=s3h&AN=53159442&site=ehost-live
ER -

TY - JOUR
ID - 4582
T1 - Children's Time Outdoors: Results and Implications of the National Kids Survey
A1 - Larson,Lincoln R.
A1 - Green,Gary T.
A1 - Cordell,H.K.
Y1 - 2011///Summer
KW - SportDiscus
KW - *OUTDOOR recreation for children
KW - *LEISURE
KW - *PARKS
KW - *CYCLING
KW - *JOGGING
KW - *INTERNET games
KW - TELEPHONE surveys
KW - CHILDREN
KW - Leisure Time
KW - National Kids Survey
KW - nature-deficit disorder
KW - OUTDOOR recreation
KW - Technology
RP - NOT IN FILE
SP - 1
EP - 20
JA - Journal of Park & Recreation Administration
VL - 29
IS - 2
N2 - EXECUTIVE SUMMARY: A growing body of literature suggests that children today are spending less time outdoors than their predecessors. This assertion, however, is confounded by the absence of a baseline for detecting trends in children's activities and time spent outdoors. The U.S.D.A. Forest Service initiated the National Kids Survey to address this problem. This general population random digit dialing telephone survey reached 1,450 U.S. households with children from 2007-2009. A proxy household member (e.g. parent or guardian) age 20 or older spoke for children between the ages 6 and 15. Teens between ages 16 and 19 were interviewed directly. Participants were asked about a variety of topics including time children spend outdoors, common outdoor activities, and reasons for not spending time outdoors. Data showed that, in general, most children (> 62.5%) spent at least two hours outdoors daily. Results also indicated that children spent either more time (39.5%) or about the same amount of time (44.8%) outdoors this year as they did last year. Males, younger children, and Hispanics spent more time outside than other demographic groups. Playing or simply hanging out was the most common outdoor activity (84.0% of respondents). Other common activities included biking, jogging, or running (79.9%) and using electronic media outdoors (65.3%). Children participated in outdoor nature-based activities less frequently than many alternatives. Interest in other activities such as listening to

music, art, or reading (57.0%), watching TV, DVDs, or playing video games (48.1%), and using electronic media including internet and texting (47.8%) were the most common reasons for not spending time outside. African American and Hispanic respondents cited more reasons for not going outside than other racial/ethnic groups. Comparisons using contingency coefficients showed that children's outdoor time on weekdays, weekend days, and time spent outdoors relative to last year were strongly correlated with the amount of time their parents/guardians were spending outdoors. Results suggest that, contrary to popular beliefs, many children today are spending a substantial amount of time outdoors. However, the nature of children's outdoor time may be changing. For example, playing or hanging out, physical activities, and technology-centered activities are more popular than nature-based activities. Electronic media consumption and parental involvement in outdoor recreation activities seem to be important factors influencing children's time outdoors. Future research efforts should continue to monitor these trends and measure the frequency and type of children's outdoor activities across diverse recreation settings. To remain relevant in the lives of American youth, park and recreation professionals could use instruments such as the National Kids Survey to adapt current services and develop innovative outdoor recreation opportunities that appeal to multiple audiences. ABSTRACT FROM AUTHOR
SN - 07351968
UR -
https://login.proxy.bib.uottawa.ca/login?url=http://search.ebscohost.com/login.aspx?direct=true&db=s3h&AN=66248864&site=ehost-live
ER -

TY - JOUR
ID - 4584
T1 - Step Off the Treadmill
A1 - Lave,Tamara Rice
Y1 - 2009/06//
KW - SportDiscus
KW - *RUNNING
KW - *TREADMILLS (Exercise equipment)
KW - *RUNNERS (Sports)
KW - *PHYSICAL fitness centers
KW - Mothers
RP - NOT IN FILE
SP - 20
EP - 20
JA - Running Times
IS - 367
N2 - The author discusses the differences between running outdoors and running on a treadmill. She understands that people who work long hours prefer the treadmill to allow them to remain in a place that is safe and convenient. The treadmill also lets mothers work out while they keep an eye on their children. Speaking on the virtues of the treadmill, the author's coach Kevin McCarey states that aging runners need to keep their cardiovascular system and may need to achieve that in the gym
SN - 01472968
UR -
https://login.proxy.bib.uottawa.ca/login?url=http://search.ebscohost.com/login.aspx?direct=true&db=s3h&AN=40536916&site=ehost-live
ER -

TY - JOUR
ID - 4585
T1 - So Good, Too Soon?
A1 - Layden,Tim
Y1 - 2003/12/08/
KW - SportDiscus
KW - *FOOTBALL players
KW - *HEISMAN Trophy
KW - *SPORTS
KW - CHILDREN of cancer patients

KW - UNIVERSITY of Pittsburgh
KW - Pennsylvania
KW - United States
RP - NOT IN FILE
SP - 63
EP - 66
JA - Sports Illustrated
VL - 99
IS - 22
N2 - A year ago, as a freshman wideout at Pittsburgh, Larry Fitzgerald caught 69 passes for 1,005 yards and 12 touchdowns and was named All-Big East. He was a very good player, but well short of dominant. A year later he is one of the best college players in the country, a transcendent receiver who demands that defenses rewrite their game plans, fans remain anchored to their seats and Heisman voters write his name somewhere on their ballots. This ascent began in a most unlikely manner, with a heartbreaking telephone call. Early last April, Fitzgerald's mother, Carol, 47, lay in a Minneapolis hospital, racked with a cancer that had taken root in one of her breasts six years earlier, spread to her lungs and now reached her brain.Last Saturday night, as wintry winds sliced through Pittsburgh's Heinz Field, Fitzgerald's brilliant season ended with a thud in a 28-14 loss to Miami that cost the Panthers (8-4) a piece of the Big East championship and their first BCS bowl berth. In a demonstration of why it's so difficult for a receiver to control a game (and win the Heisman), Miami sacked Pitt senior quarterback Rod Rutherford nine times and draped two defenders on Fitzgerald all night, a cornerback in press man-to-man coverage and a safety sitting behind him. In the near future, Fitzgerald will decide whether to join former Ohio State running back Maurice Clarett in challenging the NFL's rule prohibiting college players from entering the draft until three years after graduating from high school
SN - 0038822X
UR -
https://login.proxy.bib.uottawa.ca/login?url=http://search.ebscohost.com/login.aspx?direct=true&db=s3h&AN=11598494&site=ehost-live
ER -

TY - JOUR
ID - 4586
T1 - Get Out and Play!
A1 - Layden,Tim
Y1 - 2004/11/15/
KW - SportDiscus
KW - *OBESITY in children
KW - *CHILD nutrition
KW - *EXERCISE
KW - *PHYSICAL fitness
KW - *SCHOOL children
KW - NUTRITION disorders in children
RP - NOT IN FILE
SP - 80
EP - 93
JA - Sports Illustrated
VL - 101
IS - 19
N2 - The article discusses the efforts of health experts and gym teachers to introduce children to the benefits of exercise, in light of the alarming rate of overweight school-age children. The kids got up long before sunrise and went to school early because some grown-ups had offered them a chance to play. The grown-ups rose in darkness because they had an opportunity to use sports and games to fight, in a small way, the obesity that is rampant among young Americans. The kids and the grown-ups arrived at Parker Memorial, a public school for third- and fourth-graders in Tolland, Conn., just as the early autumn fog was lifting off the nearby green hills. The students tossed their backpacks and jackets onto a table and stormed into the small gymnasium. The adults tightened the laces on their sneakers and cued up a boom box. This sweet convergence of problem and solution was born last winter when Jaci VanHeest, an associate professor of kinesiology at the University of Connecticut's Neag School of Education, was referred to Parker Memorial by representatives of a state health

district that had received a small federal grant. The most delicate task was inviting students who were overweight or at risk of becoming so. On the first day of Paw Pals, in March 2004, skeptical parents stood outside peering through the open gym doors. Children lined up against the wall, appearing terrified of the unfamiliar adults in their school. VanHeest asked them what they wanted to play. "Superman tag," said one pupil, breaking the silence. So they played Superman tag. For one hour every morning before school, through the end of May, the children played games that they themselves suggested. And something extraordinary happened: They discovered that they loved exercising. No statistics were kept, but parents reported to VanHeest that their children were losing weight. Teachers said the kids were more alert in the classroom
SN - 0038822X
UR -
https://login.proxy.bib.uottawa.ca/login?url=http://search.ebscohost.com/login.aspx?direct=true&db=s3h&AN=15037301&site=ehost-live
ER -

TY - JOUR
ID - 4588
T1 - Health Values from Ecosystems
A1 - Lead,Coordinating
A1 - Pretty,Jules N.
A1 - Barton,Jo
A1 - Colbeck,Ian
A1 - Hine,Rachel
A1 - Mourato,Susana
A1 - MacKerron,George
A1 - Wood,Carly
KW - Reviewer Nominated
RP - NOT IN FILE
ER -

TY - JOUR
ID - 4589
T1 - Early Intensive Postural and Movement Training Advances Head Control in Very Young Infants
A1 - Lee,Hui Min
A1 - Galloway,James Cole
Y1 - 2012/07//
KW - SportDiscus
KW - *CAREGIVERS
KW - *HEAD
KW - *KINEMATICS
KW - *NEUROPHYSIOLOGY
KW - *POSTURE
KW - *NEUROMUSCULAR system
KW - *MOTION capture (Medicine)
KW - INFANT psychology
KW - INFANTS -- Development
KW - LONGITUDINAL method
KW - OUTCOME assessment (Medical care)
KW - PHYSICAL therapy for children
KW - PROBABILITY theory
KW - Statistics
KW - Time
KW - U-statistics
KW - Early Intervention (Education)
KW - Randomized Controlled Trials
KW - INTER-observer reliability
KW - BLIND experiment
KW - DATA analysis -- Software

KW - DIARY (Literary form)
RP - NOT IN FILE
SP - 935
EP - 947
JF - Physical Therapy
JA - Phys Ther
VL - 92
IS - 7
N2 - Background. Daily experiences are thought to play an important role in motor development during infancy. There are limited studies on the effect of postural and movement experiences on head control. Objective. The purpose of this study was to quantify the effects of postural and movement experiences on head control through a comprehensive set of measurements beginning when infants were 1 month old. Design. This was a prospective, longitudinal, 2-cohort study. Methods. Twenty-two full-term infants who were healthy were randomly assigned to either a training group or a control group. Infants were observed every other week from 1 to 4 months of age. Head control was assessed using a standardized developmental assessment tool, the Test of Infant Motor Performance (TIMP), as well as behavioral coding and kinematics of infants' head postures and movements in a supported sitting position. Caregivers performed at least 20 minutes of daily postural and movement activities (training group), or social interaction (control group) for 4 weeks. Results. The training group had higher TIMP scores on head control-related items during the training period and after training stopped compared with the control group. Starting from the during training phase, the training group infants had their heads in a vertical and midline position longer compared with the control group infants. After training stopped, the training group infants actively moved their heads forward more often and for larger distances. Limitations. The experiences outside daily training were not monitored, and the results may be specific to the experimental setup for infants with typical development. Conclusions. Young infants are able to take advantage of postural and movement experiences to rapidly advance their head control as early as 4 to 6 weeks of postnatal life. Infant positioning, caregiver handling, and caregiver-infant interactions were likely contributing factors. This database of comprehensive measures may be useful in future trials focused on head control in infants with special needs. ABSTRACT FROM AUTHOR
SN - 00319023
UR -
https://login.proxy.bib.uottawa.ca/login?url=http://search.ebscohost.com/login.aspx?direct=true&db=s3h&AN=77911650&site=ehost-live
ER -

TY - JOUR
ID - 4591
T1 - Generalization of tactics in tag rugby from practice to games in middle school physical education
A1 - Lee,Myung Ah
A1 - Ward,Phillip
Y1 - 2009/04//
KW - SportDiscus
KW - *EDUCATION
KW - *PHYSICAL education
KW - *EXERCISE -- Research
KW - *RUGBY football for children
KW - *COACHING (Athletics)
KW - CLASSES (Groups of students)
KW - Research
KW - Middle School Education
KW - generalization
KW - tactics
KW - tag rugby
RP - NOT IN FILE
SP - 189
EP - 207
JA - Physical Education & Sport Pedagogy
VL - 14

IS - 2
N2 - Background: Many of the issues relating to game performance of students found in the physical education literature can be considered a failure of generalization from practices to games, and from games to games. However, no study in secondary physical education has examined generalization effects as a result of effective game pedagogy in the context of teaching games. Purpose: The purpose of this study was: (a) to examine the effects of technique-focused and tactic-focused instructional conditions on the learning of a tactic by students aged 12-14 years in physical education lessons participating in a tag rugby unit, and (b) to assess the extent to which the performance of this tactic generalized from instructional games to match games. Participants and setting: This study was conducted at an urban middle school (students between 12 and 14 years of age) in the Midwest of the USA. This study was conducted during a 20-day tag rugby instructional unit. Students in three classes participated in the study. Four students from each class were selected to be observed. Intervention: The tactic-focused instructional condition served as an intervention, while technique-focused instruction was used as a baseline. Research design: A multiple baseline design across two classes, with a third class serving as a control, was used to assess the performance of the participants. Data collection: The dependent variable was 'supporting movement', which occurred during instructional games and match games. Data were coded for each target student during instructional games and match games using event-recording procedures from observation of a digital-video of the lessons. Coding occurred for each episode for each target student. Each episode ended with any incidence of a pass, tag, score, a ball carrier's error, or if the ball went outside the field of play. When the 'supporting movements' met the decided criteria they were coded as correct. Data analysis: Each player's total number of correct 'supporting movements' was divided by the number of opportunities for supporting movement and then multiplied by 100 to calculate the percentage of correct supporting movements for each target student. Findings: Low-skilled, female and male students, and average-skilled female students improved their percentage of correct supporting movements after the tactic-focused instruction was implemented. Generalization from instructional games to match games occurred for all students except average-skilled males. Conclusions: Students who typically remain unaffected by instructional interventions improved their performance of supporting behavior during tactic-focused instruction. Furthermore, those students were able to apply a tactic from instructional settings to match games. This study presents an alternative analysis of the conceptualization of sports and tactics. ABSTRACT FROM AUTHOR
SN - 17408989
UR -
https://login.proxy.bib.uottawa.ca/login?url=http://search.ebscohost.com/login.aspx?direct=true&db=s3h&AN=37604138&site=ehost-live
ER -

TY - JOUR
ID - 4592
T1 - To carry or to pull: a study to investigate the transport of a junior's golf bag
A1 - Leigh,R.J.
A1 - Young,D.B.
Y1 - 2007/12//
N1 - Accession Number: 2009766993. Language: English. Entry Date: 20080307. Revision Date: 20091218. Publication Type: journal article; research; tables/charts. Journal Subset: Alternative/Complementary Therapies; Continental Europe; Double Blind Peer Reviewed; Europe; Online/Print; Peer Reviewed. Special Interest: Chiropractic Care; Pediatric Care. No. of Refs: 36 ref. NLM UID: 101182363
KW - CINAHL
KW - Golf -- In Infancy and Childhood
KW - Sports Equipment and Supplies -- In Infancy and Childhood
KW - Weight-Bearing
KW - adolescence
KW - Body Weight
KW - chi square test
KW - Child
KW - Consumer Attitudes
KW - Descriptive Statistics
KW - England
KW - Female
KW - Male

KW - Pain -- Classification
KW - Pain -- Epidemiology
KW - Parent-Child Relations
KW - Questionnaires
KW - Human
RP - NOT IN FILE
SP - 198
EP - 204
JA - Clinical Chiropractic
VL - 10
IS - 4
N2 - Excessive load carrying by children and its resultant problems is not a new issue. Given the popularity of golf amongst youngsters, this study set out to examine the proportion of junior golfers who carried a bag; the weights junior golfers were carrying around the course in their bags; what type of bag and how they carried it; whether and where they experienced any pain or discomfort; and a number of other related factors. Seventy-five junior golfers responded to a questionnaire examining their carrying habits. The weight they carried was calculated using a balance and they self-reported any incidence of pain or discomfort felt and its location. Results showed that a significant proportion of juniors carried a bag, rather than pulled a trolley, that they carried an average of 18.7% of bodyweight in a bag, which falls outside acceptable recommendations, and 51% of bag carriers experienced some measure of pain or discomfort whilst carrying. The location of the discomfort was consistent with the manner in which the bag was carried. It is recommended that parents and professionals could take a greater interest in the problem and be more pro-active in encouraging youngsters to adopt alternative methods of club transport
SN - 1479-2354
AD - The University of Chchester, College Lane, Chichester PO19 6PE, United Kingdom
UR - http://search.ebscohost.com/login.aspx?direct=true&db=cin20&AN=2009766993&site=ehost-live
ER -

TY - JOUR
ID - 4593
T1 - PHYSICAL FITNESS AND THE PHYSICAL ACTIVITY STATUS OF 15-YEAR-OLD ADOLESCENTS IN A SEMI-URBAN COMMUNITY
A1 - Lennox,Anita
A1 - Pienaar,Anita E.
A1 - Wilders,Cilas
Y1 - 2008/03//
KW - SportDiscus
KW - *SPORTS -- Psychological aspects
KW - *SPORTS medicine
KW - *HEALTH promotion
KW - *SPORTS sciences
KW - *PHYSICAL fitness
KW - APPLIED psychology
KW - adolescents
KW - Low socio-economic environment
KW - Physical Activity
KW - Physical Fitness
RP - NOT IN FILE
SP - 59
EP - 73
JA - South African Journal for Research in Sport, Physical Education & Recreation (SAJR SPER)
VL - 30
IS - 1
N2 - Continuous research with regards to physical activity and physical fitness patterns of children is essential for the development and implementation of health promotion programmes. This study aimed to determine the physical fitness (PF) and physical activity (PA) status of 15-year-old adolescent learners from a low socio-economic, semi-urban community in the North-West province of South Africa, and the relationships between

PF, PA and distances the children walked to school. Grade 8 learners of two schools were selected for the study: School 1 (N=252), 116 boys; 136 girls, School 2 (N=66), 21 boys; 45 girls. The testing protocol included fitness tests for aerobic endurance, flexibility and body composition and the PDPAR questionnaire to determine PA levels. The results indicated that the boys and girls in School 1 and boys in School 2 were moderately active, while the girls in School 2 showed a significant lower PA level. Longer commuting distances and higher mean physical fitness values were found in School 1, while more hours of watching television were found among boys and girls ($p<0.05$) in School 2. Poor strength levels, falling outside the healthy fitness zone, showed negative relationships with aerobic fitness and flexibility. Television viewing time and commuting distances to school appeared to have a moderate influence on the moderate to low PA levels of the total group, and physical activity showed a relationship with higher fitness values. It is recommended that activity intervention strategies must aim to empower adolescents with knowledge and skills to enable them to improve their PA levels and strength. ABSTRACT FROM AUTHOR
SN - 03799069
UR -
https://login.proxy.bib.uottawa.ca/login?url=http://search.ebscohost.com/login.aspx?direct=true&db=s3h&AN=32480443&site=ehost-live
ER -

TY - JOUR
ID - 4596
T1 - Sex differences in complexity of children's play and games
A1 - Lever,J.
Y1 - 1978///
KW - SportDiscus
KW - *PLAY
KW - *GAMES
KW - *SEX role
KW - sociology
KW - BOYS
KW - girls
KW - Learning
KW - SEX FACTOR
KW - Child
RP - NOT IN FILE
CY - ;
N2 - Examines play and games as situations in which important informal learning occurs. Attention is given to the social skills that emerge as a consequence of a particular play style. Observations of children at play during recess, gym, after school and competitive games indicated very distinct play patterns for boys and girls. Attributes such as role differentiation, interdependence between players, size of the play group, explicitness of goals, number of rules and team formation, suggest that boys' play is more complex than girls' play. Sources and consequences of these sex differences are explored
UR -
https://login.proxy.bib.uottawa.ca/login?url=http://search.ebscohost.com/login.aspx?direct=true&db=s3h&AN=SPH42763&site=ehost-live;http://articles.sirc.ca/search.cfm?id=42763
L2 - http://articles.sirc.ca/search.cfm?id=42763
ER -

TY - JOUR
ID - 4601
T1 - THE EFFECTS OF PARENTAL MONITORING AND LEISURE BOREDOM ON ADOLESCENTS' INTERNET ADDICTION
A1 - Lin,Chien Hsin
A1 - Lin,Shong Lin
A1 - Wu,Chin Pi
Y1 - 2009///Winter
KW - SportDiscus
KW - *TEENAGERS

KW - *LEISURE
KW - *INTERNET
KW - *SELF-control
KW - *MOTIVATION (Psychology)
KW - Internet Addiction
KW - PARENT & child
KW - Boredom
KW - Taiwan
RP - NOT IN FILE
SP - 993
EP - 1004
JF - Adolescence
VL - 44
IS - 176
N2 - This study explored the effects of parental monitoring, leisure boredom, and leisure activity on Internet addiction. The sample was 1,289 adolescents from eleven senior high schools in Taiwan. Participants were asked about their perception of being monitored by their parents, leisure boredom, leisure activities, and Internet addiction behavior. Results showed that leisure boredom and involvement in Internet and social activities increase the probability of Internet addiction; however, family and outdoor activities along with participative and supportive parental monitoring decrease these tendencies. Overall evidence suggests that parental monitoring is a major inhibitor of Internet addiction. Thus, adolescents should be supervised in their daily routines and encouraged to participate in family and outdoor activities. In addition, adolescents should develop a positive attitude toward leisure and the skills to prevent overdependence on online relationships with the assistance of parents. These findings suggest the preventive strategies regarding Internet addiction. ABSTRACT FROM AUTHOR
SN - 00018449
UR -
https://login.proxy.bib.uottawa.ca/login?url=http://search.ebscohost.com/login.aspx?direct=true&db=s3h&AN=47716281&site=ehost-live
ER -

TY - JOUR
ID - 4602
T1 - Sport and activity participation of Hong Kong school children and youth -- part 1
A1 - Lindner,K.J.
Y1 - 1998/05//
N1 - Accession Number: 1999048143. Language: English. Entry Date: 19990701. Revision Date: 20091218. Publication Type: journal article; research; tables/charts. Journal Subset: Allied Health; Asia; Double Blind Peer Reviewed; Peer Reviewed. Grant Information: Funded by a grant from the Hong Kong Sports Development Board. No. of Refs: 18 ref
KW - CINAHL
KW - Sports -- In Infancy and Childhood -- Hong Kong
KW - Sports -- In Adolescence -- Hong Kong
KW - Physical Activity -- In Infancy and Childhood -- Hong Kong
KW - Physical Activity -- In Adolescence -- Hong Kong
KW - Funding Source
KW - Questionnaires
KW - Hong Kong
KW - Sex Factors
KW - Socioeconomic Factors
KW - Age Factors
KW - Students,Elementary -- Hong Kong
KW - Students,Middle School -- Hong Kong
KW - Students,High School -- Hong Kong
KW - random sample
KW - Survey Research
KW - Data Analysis Software

KW - Descriptive Statistics
KW - chi square test
KW - One-Way Analysis of Variance
KW - Multivariate Analysis of Variance
KW - Two-Way Analysis of Variance
KW - Physical Education and Training
KW - Child
KW - adolescence
KW - Adult
KW - Male
KW - Female
KW - Human
RP - NOT IN FILE
SP - 16
EP - 28
JA - Hong Kong Journal of Sports Medicine & Sports Science
VL - 6
N2 - A random sample of 4,690 school children and youth from Primary grade 5 to Secondary grade 7 completed a questionnaire inquiring about the nature and extent of their sport and physical activity participation during the previous school year and their wishes for sport participation in the future. In terms of frequency of participation, the boys were fairly close to figures from western countries and showed little decline over age levels, whereas the girls had quite low participation frequencies and a sharp decline with age. Ninety percent of the school children and youth had engaged in at least one sport or activity in the past year. However, extent of participation based on frequency, duration and number of months per year was lower than western norms for the boys, and the girls were far below western girls in participation extent. There were some significant differences in participation patterns among the various districts of the Territory. The data showed that an overwhelming proportion of sport participation takes place in informal free-play settings as less than 3% of the sample indicated that they belonged to a sport club or similar activity organisation, and two-thirds of all participation occurred in a setting other than the school, a club, or in the form of formal lessons. There were substantial age and regional differences in the popularity of sports. More than 25% of the respondents had dropped out of an activity in which they had been involved the previous year. Hong Kong youngsters, if given a free choice, would give preference to familiar sports and physical activities, such as basketball, badminton, soccer (boys), and swimming (girls), but also show a remarkable interest in ice skating (girls). Dislikes for sport were different for males and females, the former avoiding dance, the latter mainly soccer and combative sports. Recommendations emanating from this study included (1) targeting of the female population of children and youth in Hong Kong for sports participation promotion; (2) prioritization of encouragement and facilitation of sport participation for youngsters in particularly the Kowloon area, (3) finding ways to improve quality of sport performance in informal playground participation which is the prevalent mode of sport involvement for Hong Kong children and youth, and (4) finding ways to change the image of sport and physical activity and to make them more attractive to encourage entry into sport and diminish withdrawal
SN - 1027-7323
AD - Physical Education and Sports Science Unit, University of Hong Kong
UR - http://search.ebscohost.com/login.aspx?direct=true&db=cin20&AN=1999048143&site=ehost-live
ER -

TY - JOUR
ID - 4603
T1 - Temporal variation in childhood injury from common recreational activities
A1 - Loder,Randall T.
A1 - Abrams,Sam
Y1 - 2010/07//
KW - SportDiscus
KW - *CHILDREN -- Wounds & injuries
KW - *PREVENTION
KW - *RECREATION
KW - *SNOWBOARDING injuries
KW - *SPORTS injuries

KW - Emergency Medical Services
RP - NOT IN FILE
SP - 887
EP - 899
JF - Injury
VL - 41
IS - 7
N2 - Abstract: Background/purpose: To investigate the month and day of the week of injury in common childhood activities using the National Electronic Injury Surveillance System database. Methods: All emergency department visits 2002ƒ_"2006 from bicycles/tricycles, scooters, playground equipment, swimming/water activities, skiing/snowboarding, trampolines, and skating were analysed. The NEISS weighted and stratified data set was analysed using SUDAAN software. Weekday and month of injury, gender, race, anatomical location of the injury, geographical location of injury, and disposition were tabulated. Simple variation by month or weekday was analysed using cosinor analysis; combined variation for both month and weekday was analysed by topographical analysis. Results: There were an estimated 4.61 million emergency department visits for injuries from these activities in children in the United States. The average age was 9.5 years; there were 1.65 million girls (35.9%) and 2.97 boys (64.1%). Cosinor analysis demonstrated significant single peaks for month of injury for snow activities (January 27), trampolines (June 10), scooters (June 24), cycling (July 6), and water (July 12) activities. Double cosinor peaks were noted for skating (April 13 and September 12) and playground (April 22 and September 21) activities. Cosinor analyses demonstrated that the peak week days of injury were Monday for trampoline and snow activities, Saturday/Sunday for skating activities, Sunday for cycling, and Wednesday for playground equipment. There was no peak injury day for scooter or water activities. Topographical representation of paediatric injuries demonstrated that injuries from slides most frequently occurred Aprilƒ_"May on Wednesdays and Saturdays, and those on swings Aprilƒ_"May all days except Tuesday. Monkeybar injuries were bimodal, with the spring peak on Wednesdayƒ_"Thursday in April/May and the fall peak Tuesdayƒ_"Friday in September. Rollerblade injuries occurred Saturdayƒ_"Sunday from March/April; rollerskates on Saturdayƒ_"Sunday in Januaryƒ_"April, and skateboards Saturdayƒ_"Monday in Augustƒ_"October and Sundays in April. Conclusions: These findings can be used to further guide childhood injury prevention programmes/campaigns and especially track improvements after targeted prevention programmes. Public parks and schools should check/correct the status of playground landing surfaces just before the bimodal peaks. Education campaigns reinforcing the need for bicycle helmets could be concentrated immediately before the increase in cycling activity ƒ_" March on weekends and April/May for weekdays. Copyright &y& Elsevier
SN - 00201383
UR -
https://login.proxy.bib.uottawa.ca/login?url=http://search.ebscohost.com/login.aspx?direct=true&db=s3h&AN=51807721&site=ehost-live
ER -

TY - JOUR
ID - 4605
T1 - Vis Medicatrix naturae: does nature" minister to the mind
A1 - Logan,Alan C.
A1 - Selhub,Eva M.
Y1 - 2012///
KW - Reviewer Nominated
RP - NOT IN FILE
SP - 11
JA - Biopsychosoc Med
VL - 6
IS - 1
ER -

TY - JOUR
ID - 4606
T1 - AN ANALYSIS OF SCHOOL PHYSICAL ACTIVITY IN ADOLESCENT GIRLS. / ANALA_ZA
•ŠKOLNA_ POHYBOVA% AKTIVITY ADOLESCENTNA_CH DA_VEK

A1 - LokvencovA ¸Petra NovA ¡kovA -
A1 - Skalik,Krzysztof
A1 - FrA mel,Karel
A1 - GA3rna- • _ukasik,Krystyna
Y1 - 2011/06//
KW - SportDiscus
KW - *PHYSICAL activity
KW - *HEALTH
KW - *PHYSICAL education
KW - TEENAGE girls
KW - SCHOOL involvement
KW - girls
KW - monitoring of physical activity
KW - Physical Education
KW - Secondary school
KW - dA-vky
KW - monitoring pohybovAc aktivity
KW - st • TednA- • ¡kola
KW - tŽ>lesnA- vA«chova
RP - NOT IN FILE
SP - 65
EP - 70
JA - Acta Universitatis Palackianae Olomucensis.Gymnica
VL - 41
IS - 2
N2 - It has been well evidenced that physical activity (PA) has a positive eff ect on human health. It is very likely that a physically active child will grow up to be a physically active adult. Growing older, there is evidence of decreasing PA in children and youth. In the period of adolescence, girls tend to be less physically active than boys and therefore they are understood to be a risk group in relation to PA. OBJECTIVE: The aim of the study was to characterize school related weekly PA in adolescent girls and to identify the infl uence of participation in physical education lessons (PE) on the structure of weekly PA. METHODS: The study was carried out using the ActiGraph monitor and record sheets in a period of five school days and two weekend days. RESULTS: The results have shown that girls participating in two or more PE lessons show higher overall PA performed at school and also spend more time performing intensive PA at school than girls participating only in one or no PE lessons during the week. At school, the girls spent on average 10 minutes performing moderate PA (3.1ƒ_"6 MET) and 0.6 minutes intensive PA (> 6 MET). The girls spent most of the time at school in being physically inactive, on average 125 minutes. When we compare these results with health recommendations (seven times a week 60 minutes of moderate PA and 3 times a week 20 minutes of intensive PA), we can argue that PA in adolescent girls really needs to be examined in research. Moreover, the results show that school and PA performed there (physical education lessons) still plays its irreplaceable role in the education system regarding the total level of PA and healthy lifestyle in adolescent girls. CONCLUSIONS: The increase of total PA in adolescent girls can be secured by the increase in school related PA (more PE lessons, introducing at least one physically active recess for 30 minutes during a school day, enhancing active transport to and from school) and by the increase of girlsE¬ participation in organized PA outside of school and PA at weekends. ABSTRACT FROM AUTHOR
SN - 12121185
UR -
https://login.proxy.bib.uottawa.ca/login?url=http://search.ebscohost.com/login.aspx?direct=true&db=s3h&AN=71816472&site=ehost-live
ER -

TY - JOUR
ID - 4607
T1 - 2001 North American Society for Sport History: proceeding & newsletter
A1 - Lomax,M.E.
A1 - Smith,R.
Y1 - 2001///

KW - SportDiscus
KW - *SPORTS
KW - History
KW - MEETINGS
KW - North America
KW - Canada
KW - United States
RP - NOT IN FILE
CY - Iowa City, Iowa;
PB - North American Society for Sport History
SN - 00936235
UR -
https://login.proxy.bib.uottawa.ca/login?url=http://search.ebscohost.com/login.aspx?direct=true&db=s3h&AN=SPHS-818771&site=ehost-live
ER -

TY - JOUR
ID - 4608
T1 - Physical activity levels in normal weight and overweight Portuguese children: an intervention study during an elementary school recess
A1 - Lopes,L.
A1 - Lopes,V.
A1 - Pereira,B.
Y1 - 2009///
N1 - Accession Number: 2010458468. Language: English. Entry Date: 20091204. Revision Date: 20110513. Publication Type: journal article; equations & formulas; research; tables/charts. Journal Subset: Blind Peer Reviewed; Editorial Board Reviewed; Expert Peer Reviewed; Health Promotion/Education; Online; Peer Reviewed; USA. Special Interest: Pediatric Care. Grant Information: FCT grant 430808/2008. No. of Refs: 39 ref. NLM UID: 101083500. Email: luis.iec.um@hotmail.com
KW - CINAHL
KW - Body Weight
KW - Physical Activity
KW - Play and Playthings
KW - Students,Elementary -- Portugal
KW - Accelerometry
KW - Age Factors
KW - Analysis of Variance
KW - Body Mass Index
KW - Child
KW - control group
KW - Correlational Studies
KW - exercise intensity
KW - Experimental Studies
KW - Female
KW - Funding Source
KW - Male
KW - Obesity
KW - Portugal
KW - Repeated Measures
KW - Sex Factors
KW - Time Factors
KW - Human
RP - NOT IN FILE
SP - 175
EP - 184
JA - International Electronic Journal of Health Education
VL - 12

N2 - This study aimed to analyze the effects of an intervention strategy during the school recess on physical activity (PA) levels, by gender, age and body mass index (BMI). The sample comprises 158 Portuguese children aged 6 to 12 years. Weight and height were objectively measured. PA was assessed by accelerometry during the recess in pre-intervention and post-intervention periods. Introduction of extra outdoor play equipment was used as an intervention strategy. Significant interaction effects were found for the following areas: percentage of time spent in PA (F(1,150)=70.157;p<0.001), intervention X age group (F(1,150)= 24.416;p<0.001) and gender X age group (F(1,150)=6.919;p=0.009); the time spent in Moderate PA for intervention X gender (F(1,150)=15.290;p<0.001) and BMI X gender (F(1,150)=6.411;p=0.012); the time spent in Vigorous and Very Vigorous PA (F(1,150)=54.790;p=0.001), intervention X age group (F(1,150)=9.490; p=0.002), intervention X gender (F(1,150)=14.161;p<0.001) and BMI X gender (F(1,150)=5.049;p=0.026). It appears that successful recess interventions to improve PA for children in elementary scholars are possible by providing relatively inexpensive play equipment
SN - 1529-1944
AD - Department of Expressions and Physical Education at the Institute of Child Studies, University of Minho, Campus de Gualtar, 4710-057 Braga, Portugal
UR - http://search.ebscohost.com/login.aspx?direct=true&db=cin20&AN=2010458468&site=ehost-live
ER -

TY - JOUR
ID - 4609
T1 - Physical activity patterns during school recess: a study in children 6 to 10 years old
A1 - Lopes,V.
A1 - Vasques,C.M.S.
A1 - Pereira,M.B.F.
A1 - Maia,J.A.R.
A1 - Malina,R.M.
Y1 - 2006/11//
N1 - Accession Number: 2009584842. Language: English. Entry Date: 20070713. Revision Date: 20110513. Publication Type: journal article; research; tables/charts. Journal Subset: Blind Peer Reviewed; Editorial Board Reviewed; Expert Peer Reviewed; Health Promotion/Education; Online; Peer Reviewed; USA. No. of Refs: 29 ref. NLM UID: 101083500. Email: vplopes@ipb.pt
KW - CINAHL
KW - Physical Activity
KW - Students,Elementary
KW - Age Factors
KW - Analysis of Variance
KW - Child
KW - Descriptive Statistics
KW - Female
KW - Male
KW - Portugal
KW - random sample
KW - Repeated Measures
KW - Schools,Elementary
KW - Sex Factors
KW - Human
RP - NOT IN FILE
SP - 192
EP - 201
JA - International Electronic Journal of Health Education
VL - 9
N2 - The aims of this study were to characterize the spontaneous physical activity of children during school recess, and to estimate variation in physical activity associated with gender and age. A MTI actigraph (Model 7164) was used with a sample of 140 boys and 131 girls, 6 to 10 years of age. MTI counts were converted to METs using a regression equation developed for children. The number and average duration of periods of activity by intensity were calculated for each child during a 30 minute recess: rest or mild physical activity (</=2.9 METs), moderate physical activity (3.0-5.9 METs,), vigorous physical activity (6.0-8.9 METs) and very

vigorous physical activity (>/= 9.0 METs). Boys and girls spent about 50% of the recess in physical activity. Physical activity was characteristically done in very short bursts with intervals of rest or mild physical activity. The number of episodes of physical activity of all intensity levels was lower in older children while the inverse occurred at rest or mild physical activity. Boys engaged in higher intensity activity than girls and in general spent more recess time in physical activity
SN - 1529-1944
AD - Polytechnic Institute of BraganA   a, School of Education, Apartado 1101, Quinta de Santa ApolA3nia, BraganA   a, 5301-856, Portugal
UR - http://search.ebscohost.com/login.aspx?direct=true&db=cin20&AN=2009584842&site=ehost-live
ER -

TY - JOUR
ID - 4612
T1 - Correlates of Pedometer-Assessed Physical Activity in Cypriot Elementary School Children
A1 - Loucaides,Constantinos A.
A1 - Jago,Russell
Y1 - 2006/07//
KW - SportDiscus
KW - *PEDOMETERS
KW - *PHYSICAL fitness
KW - *EXERCISE
KW - CYPRIOT students
KW - Cyprus
KW - BMI
KW - preadolescents
KW - steps/day
RP - NOT IN FILE
SP - 267
EP - 276
JF - Journal of Physical Activity & Health
JA - J Phys Act Health
VL - 3
IS - 3
N2 - Background: The purpose of this study was to examine the association between pedometer-assessed physical activity and a number of individual, social, and environmental correlates among Cypriot elementary school children. Methods: School children in grades 5 and 6 (N = 104) and their parents (N = 70) wore pedometers for five consecutive weekdays and completed questionnaires assessing potential correlates of steps/d. Results: A hierarchical regression analysis indicated that gender, weekly frequency of sports club attendance, and hours playing outside accounted for 32% of the variance in steps/d. In addition, children with a body-mass index (BMI) above the 85th percentile (based on age and gender) scored signify scantly lower steps/d than children with a BMI below the 85th percentile. Conclusions: This study suggested that correlates of steps/d in children are similar to the findings of other studies using different measures of physical activity behavior. ABSTRACT FROM AUTHOR
SN - 15433080
UR -
https://login.proxy.bib.uottawa.ca/login?url=http://search.ebscohost.com/login.aspx?direct=true&db=s3h&AN=21984216&site=ehost-live
ER -

TY - JOUR
ID - 4615
T1 - Playground Exploration: An Opportunity for Incidental Learning of Mechanical Principles
A1 - Lyons,Brian
Y1 - 2005/03//
KW - SportDiscus
KW - *PLAYGROUNDS
KW - *CHILDREN

KW - *INERTIA (Mechanics)
KW - *ACCELERATION (Mechanics)
KW - Mechanics
KW - Learning
RP - NOT IN FILE
SP - 21
EP - 24
JA - Teaching Elementary Physical Education
VL - 16
IS - 2
N2 - The article focuses on the incidental learning of mechanical principles that occurs on playgrounds. Many children experience principles of mechanics long before their first formal exposure in a science class. On the playground, they experience translatory and rotary motion, forces such as gravity, and attempts to overcome gravity with muscular effort. These efforts result in work and the expenditure of energy. Playing on swings, merry-go-rounds, slides, and teeter-totters helps children understand concepts illustrated by Newton's laws of motion. Unknowingly, they learn about inertia, acceleration, ground reaction forces, and leverage
SN - 10454853
UR -
https://login.proxy.bib.uottawa.ca/login?url=http://search.ebscohost.com/login.aspx?direct=true&db=s3h&AN=16330765&site=ehost-live
ER -

TY - JOUR
ID - 4617
T1 - Playground safety: using research to guide community policy
A1 - Mack,M.G.
A1 - Hudson,S.D.
A1 - Thompson,D.
Y1 - 1999/11//
N1 - Accession Number: 2000038627. Language: English. Entry Date: 20000601. Publication Type: journal article; review. Journal Subset: Health Promotion/Education; Peer Reviewed; USA. Grant Information: Supported by grant number U17/CCU 712119-02 from the Centers for Disease Control and Prevention (CDC). No. of Refs: 40 ref. NLM UID: 9102137
KW - CINAHL
KW - Wounds and Injuries -- Prevention and Control -- In Infancy and Childhood
KW - Play and Playthings
KW - child safety
KW - Funding Source
KW - Epidemiological Research
KW - Wounds and Injuries -- Epidemiology -- In Infancy and Childhood
KW - Risk Factors
KW - Child Day Care
KW - Schools,Elementary
KW - Infant
KW - Child,Preschool
KW - Child
RP - NOT IN FILE
SP - 352
JA - Journal of Health Education
VL - 30
IS - 6
N2 - Each year over 200,000 children in the United States require emergency medical treatment for playground-related injuries, costing an estimated $1.2 billion. Thus, the issue of playground safety is a topic of concern for many health care professionals. However, without a reasonable understanding of the scope and magnitude of the problem, any attempts to develop an injury prevention program will be limited. Therefore, this article reviews the data-based playground research so that suggestions can be made concerning the development of a comprehensive playground injury prevention program

SN - 1055-6699
UR - http://search.ebscohost.com/login.aspx?direct=true&db=cin20&AN=2000038627&site=ehost-live
ER -

TY - JOUR
ID - 4618
T1 - Happiness is greater in natural environments
A1 - MacKerron,George
A1 - Mourato,Susana
Y1 - 2013///
KW - Reviewer Nominated
RP - NOT IN FILE
JA - Global Environmental Change
PB - Elsevier
SN - 0959-3780
ER -

TY - JOUR
ID - 4621
T1 - Padrao de actividade fisica: estudo em criancas de ambos os sexos do 4.ano de escolaridade. / Physical activity patterns: a study in primary school children of both gender
A1 - Magalhaes,L.
A1 - Maia,J.
A1 - Silva,R.
A1 - Seabra,A.
Y1 - 2002/07//
KW - SportDiscus
KW - *PHYSICAL fitness
KW - *CARDIOVASCULAR fitness
KW - *STRESS (Physiology)
KW - *PARTICIPATION
KW - *ACCELEROMETERS
KW - EXAMPLE
KW - Questionnaires
KW - Portugal
KW - Child
KW - primary school
KW - SEX FACTOR
RP - NOT IN FILE
SP - 47
EP - 57
JA - Revista Portuguesa de Ciencias do Desporto
VL - 2
IS - 5
CY - ;
N2 - The present study is aimed to perceive the different standards of physical activity in leisure time and the pattern of physical activity (regarding its manner, intensity, duration and frequency) of children within school context. A group of 120 children of both sexes, around the age of ten, attending Year 4 was selected amongst two Primary Schools in Vila Nova de Gaia. To assess the physical activity within their free time it was used Godin and Shephard's (21) questionnaire. Forty-nine children were asked to wear a portable accelerometer (Tritrac-R3D) for five days in order to assess their physical activity at school. The statistical procedures used were an average descriptive gauge, a standard deviation, unconditional and comparative occurrence; the independent measurement t-test and the Mann-Whitney non-parametric test. The results and conclusions obtained through this study suggest that: (1) boys show a greater rate of activity during free time in comparison to girls, specially in what concerns to high vigour physical activity; (2) children clearly manifest a pattern of physical activity which is fortuitous and it varies in its intensity and duration; (3) during school time children have physical activities of low intensity with less than 30 minutes per day of it being moderate/vigorous; (4) in

the playground boys are significantly more physically active than girls (p < 0.05); (5) even in planned PE sessions the standard of physical activity is of low intensity thus demonstrating children spend less than 50 % of lessons performing moderate/vigorous exercises; (6) a change of behaviour is neither reflected in planned PE sessions nor in playground activity regardless of the energy outlay
SN - 16450523
UR -
https://login.proxy.bib.uottawa.ca/login?url=http://search.ebscohost.com/login.aspx?direct=true&db=s3h&AN=SPHS-880916&site=ehost-live;http://articles.sirc.ca/search.cfm?id=S-880916;http://www.fcdef.up.pt
L2 - http://articles.sirc.ca/search.cfm?id=S-880916
ER -

TY - JOUR
ID - 4625
T1 - Girls versus boys: it's time to leave behind the childish playground behaviour
A1 - Mahoney,P.
Y1 - 2003/02//
KW - SportDiscus
KW - *GOLF for women
KW - *GOLF
KW - Women
KW - MEMBERSHIP
KW - SEX discrimination
KW - Great Britain
KW - AUGUSTA NATIONAL GOLF CLUB
KW - GENDER EQUITY
RP - NOT IN FILE
SP - 17
EP - 17
JA - Golf World (00171883)
VL - 44
IS - 2
CY - ;
N2 - Commentary on the debate concerning Augusta National Golf Club disallowing women as members crossing over into the United Kingdom
SN - 00171883
UR -
https://login.proxy.bib.uottawa.ca/login?url=http://search.ebscohost.com/login.aspx?direct=true&db=s3h&AN=SPHS-900751&site=ehost-live;http://articles.sirc.ca/search.cfm?id=S-900751;http://www.emapmagazines.co.uk
L2 - http://articles.sirc.ca/search.cfm?id=S-900751
ER -

TY - JOUR
ID - 4626
T1 - The difficult concussion patient: what is the best approach to investigation and management of persistent (>10 days) postconcussive symptoms?
A1 - Makdissi,Michael
A1 - Cantu,Robert C.
A1 - Johnston,Karen M.
A1 - McCrory,Paul
A1 - Meeuwisse,Willem H.
Y1 - 2013/04//
KW - SportDiscus
KW - *BRAIN -- Concussion
KW - *BRAIN -- Wounds & injuries
KW - *SYMPTOMS
KW - *SPORTS injuries
KW - *SPORTS medicine

RP - NOT IN FILE
SP - 1
EP - 7
JF - British Journal of Sports Medicine
JA - BJSM online
VL - 47
IS - 5
N2 - Background Concussion in sport typically recovers clinically within 10 days of injury. In some cases, however, symptoms may be prolonged or complications may develop. The objectives of the current paper are to review the literature regarding the difficult concussion and to provide recommendations for an approach to the investigation and management of patients with persistent symptoms. Methods A qualitative review of the literature on concussion in sport was conducted with a focus on prolonged recovery, long-term complications and management including investigation and treatment strategies. MEDLINE and Sports Discus databases were reviewed. Results Persistent symptoms (>10 days) are generally reported in 10-15% of concussions. This figure may be higher in certain sports (eg, ice hockey) and populations (eg, children). In general, symptoms are not specific to concussion and it is important to consider and manage coexistent pathologies. Investigations may include formal neuropsychological testing and conventional neuroimaging to exclude structural pathology. Currently, there is insufficient evidence to recommend routine clinical use of advanced neuroimaging techniques or genetics markers. Preliminary studies demonstrate the potential benefit of subsymptom threshold activity as part of a comprehensive rehabilitation programme. Limited research is available on pharmacological interventions. Conclusions Cases of concussion in sport where clinical recovery falls outside the expected window (ie, 10 days) should be managed in a multidisciplinary manner by healthcare providers with experience in sports-related concussion. Important components of management, after the initial period of physical and cognitive rest, include associated therapies such as cognitive, vestibular, physical and psychological therapy, assessment for other causes of prolonged symptoms and consideration of a graded exercise programme at a level that does not exacerbate symptoms. ABSTRACT FROM AUTHOR
SN - 03063674
UR -
https://login.proxy.bib.uottawa.ca/login?url=http://search.ebscohost.com/login.aspx?direct=true&db=s3h&AN=86209672&site=ehost-live
ER -

TY - JOUR
ID - 4628
T1 - Alcohol intoxication in adolescents... including commentary by Werner M
A1 - Manzoor,A.
A1 - bu-Arafeh,I.A.
A1 - Cole,G.F.
Y1 - 1995/03//
N1 - Accession Number: 1999078467. Language: English. Entry Date: 19991201. Revision Date: 20091218. Publication Type: journal article; commentary; research; tables/charts. Journal Subset: Biomedical; Europe; Peer Reviewed; UK & Ireland. No. of Refs: 13 ref. NLM UID: 9508883
KW - CINAHL
KW - Alcoholic Intoxication -- In Adolescence
KW - adolescence
KW - United Kingdom
KW - Retrospective Design
KW - Record Review
KW - Child
KW - Female
KW - Male
KW - Recovery -- In Infancy and Childhood
KW - Alcoholic Intoxication -- Complications -- In Adolescence
KW - Alcoholic Intoxication -- Blood -- In Adolescence
KW - Human
RP - NOT IN FILE
SP - 99

EP - 104
JF - Ambulatory Child Health
VL - 1
IS - 2
N2 - The case notes of all children, under the age of 16 years, who were admitted with alcohol intoxication to hospitals in the City of Aberdeen, during the 5-year period 1988-1992 were studied retrospectively. The circumstances in which the children took alcohol were noted and our hospitals' admission policy on children with alcohol intoxication was reviewed. A total of 154 children aged between 11 and 16 years (mean 14.4, SID 2.8) were admitted. The male to female ratio was 1:1. Most children consumed alcohol either at their own homes or at outdoors venues and about half of them drank in the company of their peers. 15% had a history of previous alcohol ingestion, drug poisoning or overdose, glue sniffing or attending hospital casualty department for accidental injuries. Ten children presented with coma and 2 children had associated physical injuries. 1 child required intensive care therapy due to multiple injuries. 142 children (93%) were admitted for overnight observation and in the majority of children no active treatment was required. None of the children in this age group had problems with hypoglycaemia or hypothermia and all children made a complete recovery. This study confirms that alcohol intoxication is common in children and complete recovery is always expected unless there were associated coma or other injuries at presentation. We conclude that children between 11 and 16 years of age with uncomplicated course of alcohol intoxication may be looked after at home safely under the supervision of the primary health services. Health education about the dangers of alcohol need to be addressed from an early age if reduction in teenage drinking is to be achieved
SN - 1355-5626
AD - Senior Registrar in Paediatrics, Royal Devon and Exeter Hospital, Exeter, UK
UR - http://search.ebscohost.com/login.aspx?direct=true&db=cin20&AN=1999078467&site=ehost-live
ER -

TY - JOUR
ID - 4629
T1 - THE TOURISM ASPECT OF PHYSICAL ACTIVITY IN PRO HEALTHFUL BEHAVIORS OF SCHOOL YOUTH
A1 - Marcinkowska,Bo • ¬ena Ewa
A1 - Szczepanowska,Ewa
Y1 - 2007/11/02/
KW - SportDiscus
KW - *LEISURE
KW - *SCHOOL children
KW - *TEENAGERS -- Health
KW - *PHYSICAL fitness
KW - *TOURISM
KW - healthy lifestyle
KW - Leisure Time
KW - Physical Activity
KW - sport
KW - tourism
RP - NOT IN FILE
SP - S57
EP - S69
JF - Medicina Sportiva
VL - 11
IS - 4
N2 - The article discusses an evaluation on the preferences of spending leisure time among primary pupils and gymnasium pupils concerning physical activity with elements of tourism. It mentions that physical activity as practicing tourism can be treated as acting upon preservation of health. It mentions that outdoor physical activity can be treated as acting upon preservation of health
SN - 14290022
UR -
https://login.proxy.bib.uottawa.ca/login?url=http://search.ebscohost.com/login.aspx?direct=true&db=s3h&AN=34309627&site=ehost-live

ER -

TY - JOUR
ID - 4630
T1 - Outdoor Leisure Pursuits: Opportunities and Constraints
A1 - Marek,J.
A1 - Bennett,R.
A1 - Kjode,A.
Y1 - 1984///
KW - SportDiscus
KW - *LEISURE
KW - *OUTDOOR recreation
KW - *RECREATION
KW - *PARTICIPATION
KW - Socioeconomic Factors
KW - Demography
KW - Norway
KW - NON-ATHLETE
RP - NOT IN FILE
N2 - Presents findings from an interview survey study carried out in three separate districts in Norway on the types and frequencies of participation in leisure pursuits, and geographical patterns of the most popular activities. Relationships between predictor variables, participation frequencies, and satisfaction are examined in order to ascertain how to increase the level of engagement in outdoor leisure activities by passive groups. Results indicate that there is not a need to create new norms or expectations, but only to maintain the existing outdoor leisure cultures. Identifies old people, housewives with small children and those with physical handicaps as having lower levels of engagement in outdoor leisure activities
UR - https://login.proxy.bib.uottawa.ca/login?url=http://search.ebscohost.com/login.aspx?direct=true&db=s3h&AN=SPH335235&site=ehost-live;http://articles.sirc.ca/search.cfm?id=335235
L2 - http://articles.sirc.ca/search.cfm?id=335235
ER -

TY - JOUR
ID - 4631
T1 - ASCERTAINING STUDY ON THE MANIFESTATION LEVEL OF THE ABILITY TO BALANCE AT CHILDREN OF 4-5 YEARS RURAL AREAS
A1 - Marin,F.I.N.I.
Y1 - 2012/09/02/
KW - SportDiscus
KW - *EQUILIBRIUM (Physiology)
KW - *CHILD development
KW - *CHILDREN -- Health
KW - CHILD development centers
KW - TEST interpretation
KW - TEST theory
KW - CHILDREN
KW - equilibrium capacity
KW - rural area
RP - NOT IN FILE
SP - 411
EP - 416
JA - Ovidius University Annals, Series Physical Education & Sport/Science, Movement & Health
VL - 12
IS - 2
N2 - Purpose: This article is an accessible material, well-structured and easy to use by educators, teachers of physical education and sports, coaches in physical education classes, namely during the performance sport activities. Prepared in accordance with current modern bibliographical needs, this article aims to support the

theoretical and practical- methodological knowledge in whose possession to be able to respond to social and motor orders related with the skill development, namely with the balance in children of preschool age. Methods: Research methods used in this research are part of private methods and of those processed, analysis and interpretation of data collected by the study. Measurements and records method consisted of applying and recording the `Flamingo" and Matorin test results. Results: Knowledge level of manifestation of the equilibrium capacity (coordination) in pre-school children in independent motor activity, by use of specific means to physical education and sport, is subject to the manifestation of motor skills, functional and psychological characteristics of each individual. Independent physical education activities will ensure the continuity of motor preparation, since at this age, by establishing a proper proportion between the individual physical condition and learning motor skills specific to physical education and sport, but also a gradual scaling of the physical exercise. Conclusions: Programming the physical education and sports activities shall be in conformity with the geographical environment, the material conditions of the school curriculum and with the analytical curriculum of the field. At the preparation and organization of physical education classes and sports, should be taken into account the number of children, the ratio number of girls and boys, health, level of physical development, psychological differences, the place that the physical education and sport occupies at the group's daily schedule, the conditions in which it is carried on (in room or outdoor), the provision of school material, etc.. The use of the most efficient means and methods specific to physical education and sports can provide an improvement in health, harmonious physical development, an individual optimal physical condition, along with training and development of mental skills necessary to integrate children into society. ABSTRACT FROM AUTHOR
SN - 12247359
UR -
https://login.proxy.bib.uottawa.ca/login?url=http://search.ebscohost.com/login.aspx?direct=true&db=s3h&AN=85380164&site=ehost-live
ER -

TY - JOUR
ID - 4634
T1 - PREDICTORS OF PHYSICAL ACTIVITY AMONG CZECH AND AMERICAN CHILDREN WITH HEARING IMPAIRMENT
A1 - Martin,Jeffrey J.
A1 - Shapiro,Deborah R.
A1 - Prokesova,Eva
Y1 - 2013/09//
KW - SportDiscus
KW - *HEALTH
KW - *PHYSICAL fitness
KW - *PHYSICAL activity
KW - *CHILDREN with disabilities
KW - HEARING impaired children
KW - Czech Republic
KW - adapted physical activity
KW - environmental influences
KW - Social Cognitive Theory
RP - NOT IN FILE
SP - 38
EP - 47
JA - European Journal of Adapted Physical Activity
VL - 6
IS - 2
N2 - Research is scarce in relation to using social-cognitive theory inclusive of social-cognitive, affective and environmental constructs to predict physical activity (PA) and fitness with hearing impaired (HI) children. Hence, the purpose of our investigation was to evaluate the ability of social cognitive variables (e.g., self-efficacy and social support), environmental (i.e., time outside) and affective constructs (e.g., physical activity enjoyment) to predict PA. Children from the Czech Republic and the USA (N = 64, M age = 14.1) with hearing impairments completed questionnaires assessing predictor variables and PA. Using multiple regression analyses we accounted for 29% of the variance in PA. Based on standardized beta-weights, the best predictors of PA were gender, country, and a block of social-cognitive constructs. Compared to males and females from the

Czech Republic and to females in the USA, American males receiving social support from their friends and who enjoyed physical education were the children most likely to be physically active. Future research examining environmental influences more fully (e.g., school settings, after school programs) both within the USA and cross-culturally and adult influences beyond parents (e.g., teachers, coaches) are encouraged. ABSTRACT FROM AUTHOR
SN - 18033857
UR -
https://login.proxy.bib.uottawa.ca/login?url=http://search.ebscohost.com/login.aspx?direct=true&db=s3h&AN=91728835&site=ehost-live
ER -

TY - JOUR
ID - 4635
T1 - EDITORIAL
A1 - Mason,Elspeth
Y1 - 2012/09//
KW - SportDiscus
KW - *SPORTS
KW - *OUTDOOR recreation
KW - Parents
KW - GRANDPARENTS
KW - EDUCATORS
RP - NOT IN FILE
SP - 1
EP - 1
JA - Horizons (14620677)
IS - 59
N2 - The author reflects on the rationale of active participation in sport and outdoors which is fundamentally a value-based attitude absorbed from strong influences. He indicated that family not school or clubs facilitates the strongest , most sustained outdoor experiences that children can ever get. He noted that parents and grandparents send children to schools with outdoor ethos and provide outdoor opportunities. A noted challenge to outdoor educators is how to reach less advantaged families
SN - 14620677
UR -
https://login.proxy.bib.uottawa.ca/login?url=http://search.ebscohost.com/login.aspx?direct=true&db=s3h&AN=79895257&site=ehost-live
ER -

TY - JOUR
ID - 4636
T1 - Learning in the outdoor environment: a missed opportunity?
A1 - Maynard,Trisha
A1 - Waters,Jane
Y1 - 2007/10//
KW - Reviewer Nominated
RP - IN FILE
SP - 255
EP - 265
JF - Early Years
VL - 27
IS - 3
N2 - In 2001, the Welsh Assembly Government announced proposals for a Foundation Phase for children aged three to seven years. The Foundation Phase framework promotes a play-based approach to children's learning in both indoor and outdoor contexts and places children's wellbeing and their personal and social development at its core. While the framework is currently being piloted across Wales, full implementation will not take place until 2010; for many schools, then, this is a time of transition. This paper discusses the findings of a research project that aimed to

document the current use of the outdoors by a group of early years teachers working in South
Wales. Drawing on data from interviews and observations conducted in four schools, it is
suggested that the teachers missed many of the opportunities afforded by the outdoor environment
to enhance children's learning. The paper considers the reasons why this might be the case and
comments on the distinction, apparent in two schools, between what was seen as 'normal' and
'special' outdoor activity.
Keywords: Outdoor play; Foundation
UR - http://www.tandfonline.com/doi/abs/10.1080/09575140701594400
L1 - file:///J:/_Searches/Trembly/Outside/new artilces/Maynard-2007-Learning in the outd.pdf
ER -

TY - JOUR
ID - 4637
T1 - Moving outdoors: further explorations of ƒ_~child-initiatedƒ_T learning in the outdoor environment
A1 - Maynard,Trisha
A1 - Waters,Jane
A1 - Clement,Jennifer
Y1 - 2013/06//
KW - Reviewer Nominated
RP - IN FILE
SP - 282
EP - 299
JF - Education 3-13
VL - 41
IS - 3
N2 - This article reports on a study in which eight Foundation Phase teachers were
supported in exploring Reggio-inspired projects in the outdoor environments of
their settings. The study found that the teachers did adopt more child-initiated/
centred approaches although it is maintained that in part this was related to the
outdoor context in which the explorations took place. However, supporting such
approaches was not easy for the teachers while many did not make regular use of
their outdoor spaces. It appeared that 'real work' was seen to take place within
classrooms and was focused on the learning of subject content.
UR - http://www.tandfonline.com/doi/abs/10.1080/03004279.2011.578750
L1 - file:///J:/_Searches/Trembly/Outside/new artilces/Maynard-2011-Moving outdoors_ fur.pdf
ER -

TY - JOUR
ID - 4638
T1 - Recreational pursuits of mentally-handicapped adults
A1 - McConkey,R.
A1 - Walsh,J.
A1 - Mulcahy,M.
Y1 - 1981///
KW - SportDiscus
KW - *MENTAL retardation
KW - *RECREATION
KW - adulthood
KW - SPORTS AND RECREATION ACTIVITIES-General
RP - NOT IN FILE
N2 - Investigated how mentally handicapped adults spend their leisure time. Parents or care-givers of mentally handicapped adults, living at home, were interviewed as to how their son or daughter spent their leisure time. Findings showed that these handicapped adults lead a sheltered existence within the community. Activities were passive and solitary in nature. Nearly half of the sample did not take part in any activity outside of the family, only one third took part in any community activity and only one fifth had non-handicapped friends. Parents seemed willing for this leisure pattern to continue and few had suggestions for different activities. Parents

believed their children were not interested in other activities and felt recreational activities should be well supervised
UR -
https://login.proxy.bib.uottawa.ca/login?url=http://search.ebscohost.com/login.aspx?direct=true&db=s3h&AN=SPH117365&site=ehost-live;http://articles.sirc.ca/search.cfm?id=117365
L2 - http://articles.sirc.ca/search.cfm?id=117365
ER -

TY - JOUR
ID - 4639
T1 - Agricultural injury risk among rural California public high school students: prospective results
A1 - McCurdy,S.A.
A1 - Kwan,J.A.
Y1 - 2012/07//
N1 - DA - 20120607
IS - 1097-0274 (Electronic)
IS - 0271-3586 (Linking)
LA - eng
PT - Journal Article
PT - Research Support, U.S. Gov't, P.H.S
SB - IM
KW - Accidents,Occupational
KW - statistics & numerical data
KW - Adolescent
KW - Agriculture
KW - California
KW - Epidemiology
KW - Confidence Intervals
KW - Cross-Sectional Studies
KW - Female
KW - Health Knowledge,Attitudes,Practice
KW - Humans
KW - Incidence
KW - Logistic Models
KW - Male
KW - Occupational Diseases
KW - Occupational Exposure
KW - adverse effects
KW - Odds Ratio
KW - Prospective Studies
KW - Questionnaires
KW - Risk Assessment
KW - Risk-Taking
KW - Rural Population
KW - Schools
KW - Sex Factors
KW - Reviewer Nominated
RP - NOT IN FILE
SP - 631
EP - 642
JF - American Journal of Industrial Medicine
JA - Am J Ind Med
VL - 55
IS - 7
N2 - OBJECTIVES: To characterize prospective agricultural injury experience among rural California Central Valley public high school students enrolled in agricultural sciences curriculum. METHODS: The University of California, Davis Youth Agricultural Injury Study (UCD-YAIS) examined prospective farm-work injury among

students from 10 California Central Valley public high schools. RESULTS: Of eligible subjects, 882 (62.5%) completed at least one annual follow-up survey. Of these, 489 reported farm work in the previous year, including 40 (8.2%) with at least one farm work-related injury. Fractures were the most common injury, especially among girls. Girls were more likely to suffer animal-related injury and boys injury from motor vehicles, machinery, or tool use. Prospective injury risk was strongly associated with prior-year farm injury (OR 8.53; 95% CI 4.02, 18.1) and farm work hours. After adjustment for farm work hours, grade level, and sex, risk was significantly associated with machinery operation, applying chemicals, number of hazardous tasks performed, riding motorcycles or mopeds, riding in back of an uncovered pick-up truck, and smoking. Risky attitude toward farm safety was associated prospectively with injury in stepwise fashion. CONCLUSIONS: Adolescents are at risk for serious farm-work injuries. Although limitations on hazardous tasks and farm work hours are likely to be the most efficacious means for reducing injury, education will play an important role. Education should include inculcating safety-related attitudes and habits and focus on hazardous tasks, such as those involving animals (for girls) and motor vehicles and machinery (for boys), especially among youth with prior farm injury
AD - Department of Public Health Sciences, University of California, Davis School of Medicine, Davis, CA, USA. samccurdy@ucdavis.edu
UR - 22069123
ER -

TY - JOUR
ID - 4640
T1 - Challenge/initiative recreation programs as a treatment for low self-concept children
A1 - McDonald,R.G.
Y1 - 1989///
KW - SportDiscus
KW - *PSYCHOLOGY
KW - *RECREATION
KW - *BALL games
KW - *ADVENTURE education
KW - *THERAPEUTICS
KW - Child Abuse
KW - Child Development
KW - Comparative Studies
KW - Child
KW - SELF-CONCEPT
KW - Aggression
KW - COOPERATIVE GAME
RP - NOT IN FILE
SP - 242
EP - 253
JF - Journal of Leisure Research
VL - 21
IS - 3
CY - ;
N2 - An investigation was initiated to determine if challenge/initiative games, conducted in an existing recreation setting for one hour daily, could be an effective treatment to enhance the self-concept of abused children living in a residential care facility. Over 28 consecutive days, a treatment group of 18 abused children received one hour of challenge/initiative games ("New," cooperative, or adventure games inclusive of a debriefing segment). A control group of 20 abused children participated for one hour daily in a traditional recreational (playground, diversionary, or competitive) games program. The results indicated that challenge/initiative games significantly enhanced four out of six components of self-concept in abused children, as measured by the Piers-Harris children's Self Concept Scale. Based on the results of this study, recreation professionals should consider incorporating challenge/initiative games as a part of their structured programs.(JLR)
SN - 00222216

UR -
https://login.proxy.bib.uottawa.ca/login?url=http://search.ebscohost.com/login.aspx?direct=true&db=s3h&AN=SPH248056&site=ehost-live;http://articles.sirc.ca/search.cfm?id=248056
L2 - http://articles.sirc.ca/search.cfm?id=248056
ER -

TY - JOUR
ID - 4642
T1 - Think Outside the Sandbox!
A1 - McGonigle,Hope
A1 - Bowman-Kruhm,Mary
Y1 - 2011/11//
N1 - Accession Number: 67676323; McGonigle, Hope; Bowman-Kruhm, Mary; Source Info: Nov/Dec2011, Issue 142, p14; Thesaurus Term: HEALTH; Thesaurus Term: AUTISM; Subject Term: CHILDREN; Subject Term: MOTOR ability in children; Subject Term: ATTENTION-deficit hyperactivity disorder; Subject Term: STRESS in children; Subject Term: COGNITIVE development; Subject Term: SOCIAL development; Subject: UNITED States; Number of Pages: 4p; Illustrations: 1 Color Photograph; Document Type: Article
KW - Health
KW - Autism
KW - CHILDREN
KW - MOTOR ability in children
KW - ATTENTION-deficit hyperactivity disorder
KW - STRESS in children
KW - Cognitive Development
KW - SOCIAL development
KW - United States
KW - Reviewer Nominated
RP - NOT IN FILE
SP - 14
EP - 17
JA - Natural Life
IS - 142
N2 - The article discusses the essence of creating a family-friendly backyard to help raise physically fit children in the U.S. It states that playing outdoors provide young children the opportunities for extensive gross-motor movement as well as developing their social, physical and cognitive abilities. Moreover, playing outside can reduce stress as well as the risk of autism and attention-deficit hyperactivity disorder in children
SN - 07018002
M3 - Article
UR - http://search.ebscohost.com/login.aspx?direct=true&db=8gh&AN=67676323&site=ehost-live
ER -

TY - JOUR
ID - 4643
T1 - Socio-economic background of white male athletes from four selected sports at the Pennsylvania State University
A1 - McIntyre,T.D.
Y1 - 1959///
KW - SportDiscus
KW - *SPORTS
KW - *UNIVERSITIES & colleges
KW - *BASKETBALL
KW - *FOOTBALL
KW - *WRESTLING
KW - *GYMNASTICS
KW - Socioeconomic Factors
KW - CAUCASIAN race
KW - MALES

KW - Pennsylvania
RP - NOT IN FILE
CY - s.l.
PB - s.n.
N2 - Determines and compares the socio-economic background parental occupations, incomes, type of education) of white male athletes participating in four sports (football, basketball, wrestling, gymnastics) at the Pennsylvania State University. Hypotheses were 1) differences in socio-economic levels are characteristic of persons engaging in various types of sports, and 2) individuals who participate in contact or combative type sports, such as football and wrestling, are more likely to have been brought up in a lower social setting than those athletes who participate primarily in non-combative sports. Data partially supported hypothesis (1) while no support was found for hypothesis (2). Football players were characterized by socio-economic factors which are not typical of other athletes: formal schooling of parents, a higher proportion of mothers working outside the home, and a larger number of children in the family
UR -
https://login.proxy.bib.uottawa.ca/login?url=http://search.ebscohost.com/login.aspx?direct=true&db=s3h&AN=SPH80246&site=ehost-live
ER -

TY - JOUR
ID - 4644
T1 - Physical activity levels and prompts in young children at recess, a two year study of a bi ethnic sample (Niveau d'activite et rapidite chez les jeunes enfants en vacances, une etude sur deux ans d'un echantillon bi ethnique)
A1 - Mckenzie,T.
A1 - Sallis,J.
A1 - Elder,J.
A1 - Berry,C.
A1 - Hoy,P.
A1 - Nader,P.
Y1 - 1997///
KW - SportDiscus
KW - ACTIVITE-PHYSIQUE
KW - VITESSE
KW - ENFANT
KW - RACE
KW - MILIEU-SOCIO-CULTUREL
KW - STATUT-SOCIAL
KW - SANTE
KW - REFLEXE
KW - TEMPS-DE-REACTION
RP - NOT IN FILE
UR -
https://login.proxy.bib.uottawa.ca/login?url=http://search.ebscohost.com/login.aspx?direct=true&db=s3h&AN=SPHS-630837&site=ehost-live
ER -

TY - JOUR
ID - 4648
T1 - Neighborhood Income Matters: Disparities in Community Recreation Facilities, Amenities, and Programs
A1 - McKenzie,Thomas L.
A1 - Moody,Jamie S.
A1 - Carlson,Jordan A.
A1 - Lopez,Nanette V.
A1 - Elder,John P.
Y1 - 2013/12//
KW - SportDiscus
KW - Built Environment

KW - CHILDREN
KW - Facilities
KW - Health
KW - Physical Activity
KW - recreation centers
KW - Socioeconomic Status
RP - NOT IN FILE
SP - 12
EP - 22
JA - Journal of Park & Recreation Administration
VL - 31
IS - 4
N2 - Physical activity is important for children's development and their current and future health; national recommendations are for them to engage in at least 60 minutes of moderate to vigorous physical activity daily. Most of children's physical activity occurs outside of school hours; thus, access to and the quality of community recreation facilities and programming are particularly relevant. Researchers have identified strong links among socioeconomic disparities, physical inactivity, and poor health, but a limited number of studies have examined how access to community recreation facilities and physical activity programming are affected by local socioeconomic conditions. In many low-income communities, park and recreation facilities may be the only place for children to be physically active outside of school; thus, it is important to understand the connection between community environmental characteristics and child use of facilities. We were interested in determining whether the characteristics of community recreation center environments would be associated with neighborhood income and children's use of the centers. To do this we designed a study to identify whether neighborhood income disparities were associated with recreation center environmental characteristics and whether those characteristics were associated with young children's use of the center. We believed that findings to these questions could inform policy decisions within recreation centers and recreation departments to improve equity, facility use, and levels of physical activity. Thirty community recreation centers and 541 nearby families with children aged 5-8 years in five cities in Southern California participated in the study. To generate data we used multiple research instrumentation including (a) a structured physical activity survey of program offerings and barriers to children's participation in physical activity at the center [completed by recreation center supervisors], (b) direct observation of the presence and condition of recreation center facilities and amenities by trained assessors, and (c) a parent questionnaire on child use of the center. Results indicated that the condition of the community center facilities and amenities, but not their number, was positively related to neighborhood income ($p < .05$). As well, the number of cost-free, but not total, youth physical activity programs was inversely associated with neighborhood income ($p < .05$). Parent's report of their children using centers was positively associated with the number of amenities observed there ($p < .05$). The results suggest that policy makers and community recreation center staff should consider both neighborhood economic issues and environmental characteristics in their efforts to promote children's physical activity at recreation centers. ABSTRACT FROM AUTHOR
SN - 07351968
UR - https://login.proxy.bib.uottawa.ca/login?url=http://search.ebscohost.com/login.aspx?direct=true&db=s3h&AN=94949247&site=ehost-live
ER -

TY - JOUR
ID - 4650
T1 - Time-motion analysis of playground activity of primary school children in Northern Ireland. (Abstract) / Analyse des activites pratiquees dans les cours d ' ecoles primaires en Irlande du Nord.) (Resume
A1 - McLaughlin,E.
A1 - O'Donoghue,P.G.
Y1 - 2002/01//
KW - SportDiscus
KW - *PHYSICAL fitness
KW - *PLAYGROUNDS
KW - *STRESS (Physiology)
KW - *CARDIOVASCULAR fitness

KW - *PARTICIPATION
KW - sociology
KW - Northern Ireland
KW - Child
KW - primary school
KW - TIME FACTOR
KW - COURSE
KW - ACTIVITE-PHYSIQUE
KW - ENFANT
KW - ECOLE
KW - IRLANDE-DU-NORD
KW - INTENSITE-DE-L-EFFORT
KW - MARCHE
RP - NOT IN FILE
SP - 24
EP - 24
JF - Journal of Sports Sciences
JA - J Sports Sci
VL - 20
IS - 1
CY - ;
SN - 02640414
UR -
https://login.proxy.bib.uottawa.ca/login?url=http://search.ebscohost.com/login.aspx?direct=true&db=s3h&AN=SPHS-848783&site=ehost-live;http://articles.sirc.ca/search.cfm?id=S-848783;http://www.tandf.co.uk/journals
L2 - http://articles.sirc.ca/search.cfm?id=S-848783
ER -

TY - JOUR
ID - 4651
T1 - Taking Them to Task
A1 - McLeod,Malkia
Y1 - 2006/04//
KW - SportDiscus
KW - *PLAYGROUNDS
KW - *SPORTS facilities
KW - SAFETY measures
KW - ARCHITECTURE -- Conservation & restoration
KW - TASK forces
KW - Elementary Schools
KW - BALTIMORE (Md.)
KW - Maryland
RP - NOT IN FILE
SP - 34
EP - 39
JA - Parks & Recreation
VL - 41
IS - 4
N2 - The article reports on the playground renovations being carried out by the Playground Task Force comprising various representative organizations from Baltimore, Maryland, to provide safe playgrounds to children in the state. The task force recommended the development of a city-wide playground network. Elementary schools would have playground areas, which included structures and spaces that encouraged interaction with nature. The second network of playgrounds would be established in parks to provide clusters of outdoor. The task force was committed to competing for grants and raising public and private funds from city, state, local businesses and non-profit foundations
SN - 00312215

UR -
https://login.proxy.bib.uottawa.ca/login?url=http://search.ebscohost.com/login.aspx?direct=true&db=s3h&AN=20459374&site=ehost-live
ER -

TY - JOUR
ID - 4652
T1 - The Questions with Randy McMichael
A1 - McMichael,Randy
A1 - Kennedy,Kostya
Y1 - 2004/10/18/
KW - SportDiscus
KW - *FOOTBALL players
KW - *TIGHT ends (Football)
KW - MIAMI Dolphins (Football team)
KW - Interviews
RP - NOT IN FILE
SP - 39
EP - 39
JA - Sports Illustrated
VL - 101
IS - 15
N2 - This article presents an interview with football player Randy McMichael, tight end with the Miami Dolphins. What was your welcome-to-the-NFL moment? When I got that phone call on draft day in '02, it was real exciting, but it didn't really hit me until I got to the Fort Lauderdale airport. Favorite off-day activity? I buy new clothes for me and my two kids, and I try to keep everybody clean. If I weren't in the NFL, I'd be... A kindergarten teacher. You've got to mold children when they're young, because when they get to high school, they get a little out of control. McMichael, the Dolphins' top pass catcher, had four receptions for 62 yards versus the Pats. Week 6 has been good for McMichael: In '02 he had a team-high five catches
SN - 0038822X
UR -
https://login.proxy.bib.uottawa.ca/login?url=http://search.ebscohost.com/login.aspx?direct=true&db=s3h&AN=14704417&site=ehost-live
ER -

TY - JOUR
ID - 4653
T1 - Poster Abstracts from the AAAP 20th Annual Meeting and Symposium
A1 - McNicholas,Laura F.
Y1 - 2010/07//
KW - SportDiscus
KW - *DRUG abuse
KW - *OPIOIDS
KW - ABSTRACTS
KW - BUPRENORPHINE -- Abstracts
KW - MEDICAL appointments & schedules -- Abstracts
KW - HEALTH planning -- Abstracts
RP - NOT IN FILE
SP - 368
EP - 381
JA - American Journal on Addictions
VL - 19
IS - 4
N2 - We are very pleased that The American Journal on Addictions (AJA) has committed to continuing to publish all abstracts from poster presentations made at the 2009 annual meeting of the American Academy of Addiction Psychiatry (AAAP). Scientific presentations made at the meeting play a vital and important role in the content of the meeting, and AAAP has been committed to fostering this aspect of the program. Prior to the

2005 meeting, AJA published a small number of abstracts based upon ratings made by members of the Research Committee as a means for acknowledging the work presented. After discussion, the Board of Directors recommended publishing all abstracts from poster presentations, and recent changes in the Journal and consensus by the Board have resulted in this new initiative. By publishing these abstracts, AAAP completes what may be viewed as a three-step process. The first step is the submission of abstracts for a poster presentation at the meeting; these abstracts undergo peer review and critique by the Program Committee, and those that do not fulfill a minimal standard of quality are not accepted. The second step is then the actual presentation at the meeting. This new, final step is the publication of the abstract in a citable journal that is well established and respected in the field of addictions. Our hope is that the opportunity to have an abstract published in the Journal will capture the attention of, and be attractive to, researchers in the field. The AAAP annual meeting provides a unique opportunity for researchers and clinicians to interact, and the AAAP and AJA are taking this step in an effort to reach out to members of the research community who may not traditionally attend and present at the meeting. Other steps taken by AAAP, such as major presentations at the meeting centered on NIDA's Clinical Trials Network (CTN), also seek to capitalize upon this unique opportunity for the research and treatment communities to interact and learn from each other. It is the intent of AAAP and AJA that publication of all poster abstracts will occur following annual meetings into the future. We hope you will consider submitting a presentation for the 2010 annual meeting. The process is simple, the meeting is collegial and enjoyable, and there is now the additional bonus of a citable reference for your work. Finally, congratulations to the authors of these abstracts. Poster 1: Buprenorphine-Medication Assisted Treatment: A Retrospective Analysis of Health Plan Data Charles Ruetsch, PhD, Health Analytics LLC, Columbia, Maryland; J Tkacz Background/Objective: Buprenorphine-medication assisted therapy (B-MAT) is an effective treatment for opioid dependence (OD), but its cost benefit is unclear. This study estimated cost benefit of B-MAT from the payor perspective. Methods: A pre-post case matched time series analysis of 2 groups (1. B-MAT fill and, 2. no B-MAT fills) were analyzed over time. Data were claims (Q4 2005 to Q3 2008) from a large MCO of members with a diagnosis of OD. A within subjects Poisson model was used to regress patient characteristics onto six service utilization count measures and a within subjects MANCOVA was used to analyze service cost measures. Results: After controlling for demographics, Medicaid status, and health status, Group 1 had more Rx fills (5.21) and physician and outpatient visits (.88), but fewer hospital days (1.46) and ER visits (.22; p's < .05). Group 1 had higher Rx ($1,208) and physician and outpatient costs ($160), but lower inpatient hospital costs ($1,986; p's < .05). Overall, Group 1 had lower total healthcare costs ($11,200, p < .001). Utilization of costly services and costs decreased over time and the interaction terms indicate that B-MAT patients drive most of the decreases. Conclusions: Though B-MAT patients incur higher pharmacy and office visit costs, they use fewer expensive health care services in other areas, resulting in an overall positive cost-benefit conclusion. Further, the longer that patients are on B-MAT the lower their cost is overall compared to non-B-MAT patients. Source of Funding: Reckitt Benckiser Pharmaceuticals. Poster 2: Compliance with Buprenorphine- Medication Assisted Treatment Decreases Drug Use among Opioid Addicts Joseph Tkacz, MS, Health Analytics LLC, Columbia, Maryland; C Ruetsch Background/Objective: Among those who are opioid dependent (OD), there is also a risk for poly-substance abuse. Follow-up substance abuse was examined on a national sample of OD patients during treatment with buprenorphine-medication assisted therapy (B-MAT). Methods: Subjects (N = 1,426) were new B-MAT patients who were randomly assigned to an intervention arm (B-MAT + telephonic patient support medication compliance program) or control arm (B-MAT alone) as part of a larger RCT. Measures included the Addiction Severity Index collected at baseline, 1, 2, 3, and 6 months. Difference in use of a variety of substances at 6 months was compared between subjects who were compliant (C) with B-MAT and subjects who were noncompliant (NC). Compliance was defined as taking medication on at least 22 of the previous 28 days. Chi-square tests of equality of proportions were used to compare groups. Results: Compared to C subjects, NC subjects were significantly more likely to use: alcohol (44% vs. 33%), heroin (11% vs. 3%), methadone (5% vs. 1%), other opiates (22% vs. 7%), cocaine (6% vs. 2%), and cannabis (23% vs. 15%); p's < .05. Conclusions: Results indicate that, in addition to less opioid use, C Ss used additional licit and illicit substances less often than did NC Ss. These results are particularly important given the number of OD patients who abuse multiple substances, and the high costs associated with drug dependence (eg, treatment, prison, crime, etc.). Future studies should compare the effectiveness of other treatment modalities on subsequent drug use patterns in opioid-dependent patients. Source of Funding: Reckitt Benckiser Pharmaceuticals. Poster 3: Predictors of Buprenorphine-Medication Assisted Treatment Compliance among Opioid-Dependent Patients: Results at Month Six Charles Ruetsch, PhD, Health Analytics LLC, Columbia, Maryland; J Tkacz Background/Objective: Compliance with buprenorphine medication assisted therapy (B-MAT) drives early treatment outcomes among opioid-dependent (OD) patients. This study examines the relationship between patient behavior, participation in a compliance program, and 3-month treatment compliance. Methods: Treatment (B-MAT) seeking patients (N

= 1,426), were randomly assigned to an intervention group (B-MAT plus patient support program) or control group (B-MAT as usual). Measures included the Addiction Severity Index and Treatment Services Review collected at baseline, 1, 2, 3, and 6 months. Chi-square tests of equality of proportions were used to compare compliance between groups at each measurement period. Month six compliance was regressed onto group, demographic indicators, and baseline indicators. Subjects who were assigned to the intervention, but did not choose to participate, were excluded from analyses. Results: Subjects assigned to the intervention were significantly more likely to be compliant with B-MAT at all 4 follow-up measurement periods (months 1, 2, 3, and 6; p's < .001). Baseline heroin and barbiturate use, the number of days receiving treatment for psychological problems, and the number of days experiencing medical problems were significant inverse predictors of B-MAT compliance. Older subjects who were paid for more days of work were more likely to be compliant with B-MAT. Conclusions: Baseline drug use, medical and psychiatric issues, and a variety of demographic indicators were all predictive of B-MAT compliance at month 6. Future research should focus on developing methods that allow or facilitate application of compliance programs, such as Here-ToHelpƒ,›, among these more vulnerable populations. Source of Funding: Reckitt Benckiser Pharmaceuticals. Poster 4: Differences in Opioid Dependence Severity by Health Insurance Ameliorated by Month Six of Buprenorphine Treatment Charles Ruetsch, PhD, Health Analytics LLC, Columbia, Maryland; J Tkacz Background/Objective: Few studies have examined the effect of patient insurance coverage on outcomes among opioid-dependent (OD) patients in buprenorphine-medication assisted therapy (B-MAT). Vital to payors, both commercial and government, the purpose of this study was to understand differences in treatment outcomes between commercially insured B-MAT patients and those with public sector coverage (ie, Medicare or Medicaid) or no insurance coverage. Methods: Subjects (N = 1,426) were new B-MAT patients who were randomly assigned to an intervention arm (B-MAT + telephonic patient support medication compliance program) or control arm (B-MAT alone) as part of a larger RCT. Measures included the Addiction Severity Index collected at baseline, 1, 2, 3, and 6 months. Results: At baseline, subjects with Medicaid/Medicare or no coverage (n = 606) had significantly higher legal, psychiatric, and employment composite scores, indicative of greater problem severity in these areas, compared to the commercially insured (p's < .05). The commercially insured group had a significantly higher alcohol composite score (p < .05). By month 6, there were no longer any differences between the two groups on the legal and psychiatric composite scores, yet the differences on employment and alcohol composites remained. Conclusions: B-MAT appears to have a positive impact on many areas of the OD patient's life. Future research is needed to elucidate why only certain differences between insurance groups disappeared after 6 months of treatment while others remained, and to investigate whether the sequela of other types of substance abuse (eg, alcohol) differ based on one's health insurance type. Source of Funding: Reckitt Benckiser Pharmaceuticals. Poster 5: Cytochrome P450 Abnormalities in Patients Who Require High-Dose Opioids Forest Tennant, MD, Dr PH, Veract Intractable Pain Clinic, West Covina, California Background: For unclear reasons, some chronic pain patients require very high-dose opioid therapy to adequately control pain. Recently, it has become possible to test for some genetic cytochrome P450 enzyme abnormalities. Methods: Fifteen (15) chronic pain patients who required 100 mg or more of morphine equivalence a day to control pain were tested for one or both the cytochrome P450, 2C9 or 2D6, genotypes. Their opioid serum concentrations were determined approximately 1 hour after their usual opioid dosage. Results: One of 11 (9.0%) patients tested for genotype 2D6 was abnormal. This patient is of Asian extraction. Serum concentrations of oxycodone and its metabolite, oxymorphone, were undetectable 1 hour after dosing. Two (2) of 8 (25%) patients tested for 2C9 genotype were abnormal, and both had very high serum concentrations of fentanyl (8.1 and 16.4 mg/ml) 1 hour after dosing. All three patients with cytochrome P450 genotype abnormalities stated that opioids provided pain relief for a very short time, usually only 1 to 2 hours, after administration. Conclusions: This preliminary investigation shows that genetic, cytochrome P450 abnormalities exist, in at least some high-dose opioid patients, even though only two of many genotypes were tested here. Cytochrome P450 abnormalities may require high serum opioid concentrations to stimulate ('force') enzyme activity and/or the abnormality may result in ultrafast metabolism. High-dose opioid patients are often stigmatized as abusers or addicts, and this preliminary study compels further study and possibly a new clinical perception of these patients. Source of Funding: None. Poster 6: Outcomes of Chronic Pain Patients Treated with Opioids for Over 10 Years Forest Tennant, MD, Dr PH, Veract Intractable Pain Clinic, West Covina, California Background: Recent epidemiologic studies indicate that about 10 million Americans now take opioid drugs for pain control. This relatively recent and dramatic occurrence has had little outcome study. The author recently reported 24 Southern California chronic pain patients who were treated with opioids over 10 years and who had positive social, physical, and functional results. Methods: Physicians who have long-term opioid-treated pain patients were recruited by advertising in a trade journal for physicians. Once identified, physicians completed a questionnaire for each 10-year patient. Information consisted of opioid use, dosage, underlying cause of pain, length of opioid therapy, stability of dosage, complications, and functional

abilities. Results: To date 50 total patients from six geographic sites have been identified. The majority of patients have remained on a stable opioid dosage for long periods and maintain good function in activities of daily living including dressing, reading, work, care for family, attendance at social functions, driving a car, and ambulation without assistance. Conclusions: This survey study leaves out patients who may have experienced severe side effects or found opioids to be an impairment to normal activities of daily living and discontinued their use. Although the percentage of patients who fall in the 'poor outcome' category is unknown, it is clear that long-term opioid use is associated with a positive outcome in some chronic pain patients. Given the findings here, there is no obvious reason to discourage opioid use or encourage pain patients to cease opioids. Source of Funding: None. Poster 7: Implementation of an Electronic Information System to Enhance Practice at an Opioid Treatment Program Steven Kritz, MD, Addiction Research and Treatment Corporation, Brooklyn, New York; LS Brown, Jr, M Chu, R Zavala Background: Adoption of electronic health systems (EHS) is commonly discussed in healthcare reform, especially in addiction treatment. We report the design and pre-implementation quality and patient and staff satisfaction results of an integrated EHS in an outpatient opioid agonist treatment program, consisting of seven clinics and providing primary medical and HIV-related care for approximately 3,000 predominantly minority adults in New York City. Methods: Besides quality and satisfaction, three other aims (productivity, financial performance, and risk management) were in the parent study, using a pre-post, prospective comparative design. Quality measures included: (1) viral load assessment in hepatitis C virus (HCV) infected patients, and (2) timely assessment of patients' medical and addiction status at various intervals. Using an anonymous survey, patient and staff satisfaction were assessed. Associations were assessed with staff and patient variables. Results: HCV viral load was appropriately performed in 92% of cases; annual medical assessments were timely for 83% of cases; annual addiction assessments were timely at 30 days, 90 days, and annually for 81%, 46%, and 70% of cases, respectively. Seventy-four percent of patients were satisfied/very satisfied with their care, while 33% of clinicians and managers were satisfied/very satisfied with the pre-EHS system for providing care. Conclusions: These results provide insight into primary care and addiction treatment delivery in one large program and provide the basis of assessing the impact of an integrated EHS, further informing healthcare reform discussions. Source of Funding: This R01 study is funded by the National Institute on Drug Abuse: DA022030. Poster 8: Design of a Novel Relapse Prevention Videogame as an Adjunct to Treatment for Alcoholic Patients Marcia L Verduin, MD, University of Central Florida, Orlando, Florida and Orlando VA Medical Center, Orlando, Florida; S LaRowe, C Bowers, R Joyce, P Smith, H My- rick, J Salazar, B Stabile Background: Relapse prevention (RP) therapy is well supported in the treatment of alcoholism. However, current delivery of RP therapy provides little opportunity to practice skills in a safe, realistic environment. Innovative approaches are needed to develop alternative settings for RP skill practice and maintenance. Methods: Addiction specialists collaborating with serious games programmers developed an interactive videogame grounded in RP theory for alcoholics to play as an adjunct to standard treatment. The game is currently being evaluated in a randomized prospective clinical trial. Results: The game concept for 'Guardian Angel' is based on lifestyle management-type games. The group developed four minigames housed within an overarching core game. In the core game, patients go about their daily lives while managing emotions, cravings, finances, and relationships. During each 'day' of the game, patients must play one of the minigames-Route Planner (navigating home while avoiding potential triggers), Clean House (ridding their home of hidden alcohol and other triggers), Drink Refusal (practicing drink refusal skills), and Balance Out ('therapy' sessions to reinforce key RP skills). Feedback from focus groups has been uniformly positive. A laptop will be provided for attendees to play the game during the poster session. Conclusions: An interactive videogame based on RP theory may provide a motivating, safe, realistic environment for alcoholics to practice their RP skills, in addition to standard treatment. Preliminary focus groups indicate that patients find the videogame acceptable and engaging. A clinical trial is underway to determine the efficacy of the game as an adjunct to treatment. Source of Funding: Robert Wood Johnson Foundation. Poster 9: Cross-Cultural Adaptations and Physician Training Integrating Alcohol Interventions into Routine Tuberculosis Care in Tomsk, Russia Hilary Smith Connery, MD, PhD, McLean Hospital, Belmont, Massachusetts and Harvard Medical School, Boston, Massachusetts; V Livchits, A Shields, L McGrady, N Patrick, CS Lastimoso, A Grishkina, JE Hart, SS Shin, SF Greenfield Background: IMPACT (Integrated Management of Physician-delivered Alcohol Care for Tuberculosis patients) is a randomized controlled trial to assess the effectiveness of alcohol interventions as an integrated component of TB care provided to patients with co-occurring TB and alcohol use disorders (AUD) in Tomsk, Russia. We discuss cross-cultural adaptations and physician training to provide an alcohol brief counseling intervention (BCI) and medical management with naltrexone (NTX). Methods: Alcohol interventions were delivered monthly by TB physicians as part of a 6-month directly observed therapy, short-course ('DOTS') protocol for TB treatment. Patients with TB meeting CIDI-SAM criteria for AUD provided informed consent for random assignment to receive BCI, NTX, BCI + NTX, or TAU (treatment as usual) adjunct to DOTS. Eighteen TB

physicians were certified to perform all adjunct interventions through a series of trainings by a US-Russian multidisciplinary team using in-person and teleconferenced didactics, role-plays, feedback, and mock interviews. AUD interventions were audiotaped, translated, and reviewed weekly by a bilingual United States-Russia fidelity team consisting of expert AUD supervisors and trained bilingual coders and Russian supervisors. TB physicians received ongoing corrective feedback and training for adherence to each intervention. Results: Financial incentives and personalized training positively affected physician attitudes toward AUD intervention delivery and adherence to protocol. Cross-cultural challenges include linguistic adaptations, altering cultural norms of physician-patient dialogue, and limited referral support services for AUDs. Conclusions: Frequent dialogue and performance feedback with TB specialists by a multidisciplinary United States-Russian AUD training team resulted in cross-culturally acceptable and feasible AUD interventions integrated into TB care. Source of Funding: Supported in part by a grant from the National Institute on Alcohol Abuse and Alcoholism R01AA016318 (SS) and from the National Institute on Drug Abuse K24DA019855 (SFG). Poster 10: Ethics and Addiction: Moving Beyond Mistrust in Individual and Collective Care Relationship Aymeric Reyre, MD, Avicenne Hospital, Paris, France and WHO Collaboratve Center for Ethics, Paris, France; O TaA_eb, JF Rouchon, G Broder, E Hirsch, MR Moro, T Baubet Background: Data from the literature as well as the daily care of addict patients stress the importance of pejorative representations they are carrying. These representations lead to a climate of mistrust responsible for major difficulties in establishing an authentic care relationship. Methods: Our phenomenological analysis of mistrust is based on Georges Devereux's complementarist epistemology. It allows us to link anthropological, philosophical, and clinical viewpoints and opens tracks for the medical person's ethical positioning. Results: The pragmatic approach based on the analysis of interactions in the economic field and on game theory, calls to bet on trust to maximize systemic efficiency. On the other hand, a philosophy of action lead to Michel Foucault's ethics of the 'Care of the Self': the medical staff, in an environment of mistrust, must develop a thinking and an aesthetic of its action. Finally, Paul Ricoeur and Hannah Arendt's moral philosophy calls for an ethics of mutual recognition and leads to rethinking the role of the promise in the care relationship. Conclusions: It is possible to remove the care relationship from the stranglehold of mistrust: (1) by taking the risk of trust, (2) by reflecting on our action in the field of care and exposing this action to the opinions of the others, (3) by using promise wisely, without limitation or excess, (4) by forming 'islets of trust' in our care institutions, spaces free from the need to promise, which could guarantee their democratic nature. Source of Funding: None. Poster 12: Maternal Cigarette Smoking and Substance Use Disorders during Pregnancy and Offspring Bipolar Disorder Timothy E Wilens, MD, Massachusetts General Hospital, Boston, Massachusetts; M Martelon, J Wozniak, M Monuteaux, M Evans, T Parcell, J Biederman Objectives: Recent work has suggested a positive relationship between prenatal exposure to cigarettes and substances (alcohol or illicit drugs) and offspring psychiatric disorders. No study to date, however, has primarily examined these exposures with offspring bipolar disorder (BPD). We looked to replicate previous work from our group that reported a positive association between maternal use of cigarettes during pregnancy and offspring BPD using a separate sample. Methods: As part of two family studies of BPD, 322 families, including 225 BPD subjects (mean age AñSD: 11.60 Añ3.50) and 97 controls (mean age AñSD: 13.70 Añ2.10), were comprehensively assessed with a structured psychiatric diagnostic interview for psychopathology and substance use. Maternal use of nicotine and substances during pregnancy were captured using two methods derived from structured psychiatric interviews. Results: BPD offspring were more likely to be exposed to prenatal maternal smoking than control offspring (odds ratio (95% CI) = 2.48 (1.15, 5.32)). However, this association lost significance when adjusted for comorbidity with conduct and attention deficit hyperactivity disorders (odds ratio (95% CI) = 1.17 (.47, 2.94)). There was no association between substance use disorders (SUD; including drug or alcohol abuse or dependence) during pregnancy and offspring BPD. There were notable differences in the clinical characteristics of youth with BPD exposed to prenatal maternal smoking and alcohol use disorders. Conclusions: We partially replicated previous findings showing that comorbid BPD is associated with maternal use of nicotine during pregnancy. Our results warrant further replication in larger, less comorbid, prospective samples. Source of Funding: NIH R01 DA12945 (TW), K24 DA016264 (TW), and NIH R01 MH066237 (JW). Poster 13: Does Conduct Disorder Mediate the Development of Substance Use Disorders in Adolescents with Bipolar Disorder? A Case-Control Family Study Timothy E Wilens, MD, Massachusetts General Hospital, Boston, Massachusetts; MK Martelon, MJP Kruesi, T Parcell, D Westerberg, M Schillinger, M Gignac, J Biederman Background: Recent work has highlighted important relationships among conduct disorder (CD), substance use disorders (SUD), and bipolar disorder (BPD) in youth. However because BPD and CD are frequently comorbid in the young, the impact of CD in mediating SUD in BPD youth remains unclear. Methods: A total of 105 adolescents with DSM-IV bipolar disorder (mean AñSD = 13.6 Añ2.50 years) and 98 controls (13.7 Añ2.10 years) were comprehensively assessed with a structured psychiatric diagnostic interview for psychopathology and SUD. The study was conducted from January 2000 through December 2004. Results:

Among bipolar disorder youth, those with CD were more likely to report cigarette smoking and/or SUD than youth without CD. However, CD preceding SUD or cigarette smoking did not significantly increase the subsequent risk of SUD or cigarette smoking. Adolescents with bipolar disorder and CD were significantly more likely to manifest a combined alcohol plus drug use disorder compared to subjects with bipolar disorder without CD (X2= 11.99, p < .001). Conclusions: While bipolar disorder is a risk factor for SUD and cigarette smoking in a sample of adolescents with BPD, comorbidity with CD in adolescents, comorbidity with preexisting CD does not increase the risk for SUD. Further follow-up of this sample through the full risk of SUD into adulthood is necessary to confirm these findings. Source of Funding: National Institutes of Health grants RO1 DA12945 and K24 DA016264. Poster 14: Open Trial of Sustained Release Bupropion in Adults with ADHD plus Substance Use Disorders Timothy E Wilens, MD, Massachusetts General Hospital, Boston, Massachusetts; JB Prince, J Waxmonsky, R Doyle, T Spencer, MK Martelon, M Evans Objective: To evaluate the effectiveness and tolerability of bupropion in adults with Attention-Deficit/ Hyperactivity Disorder (ADHD) and comorbid active Substance Use Disorders (SUD). Methods: This was a 6-week open trial of sustained-release (SR) bupropion in adults aged 18 to 55 years diagnosed with both ADHD and SUD. Bupropion-SR was initiated at 100 mg SR and increased weekly to a target dose of 200 mg SR twice daily. Subjects were assessed on multiple outcomes including ADHD, SUD, and adverse effects. All analyses were intent to treat, with last observation carried forward. Results: Thirty-two subjects were treated with bupropion, with 19 subjects completing the entire protocol (59%). At end point there were clinically significant reductions in the ADHD RS (34.1 Añ8.2 to 19.4 Añ 11.4, ƒ^'43%, t = 6.49, p < 0.0001) and the Clinical Global Impression (CGI) of ADHD severity (baseline = 5.0, endpoint = 3.8, ƒ^'24%, t = 6.16, p < 0.0001). In contrast, there were clinically negligible effects on the self-report of substance use (p's > 0.05) and on the overall CGI of SUD severity (ƒ^'23%, t = 4.95, p < 0.0001). Conclusions: Results from this open trial suggest that in adults with ADHD and SUD, treatment with bupropion-SR is associated with clinically significant reductions in ADHD, but not SUD. Source of Funding: NIH K24DA016264, Departmental funds for clinical trial. Poster 15: Addiction Psychiatry Service: A Specialty Addiction Consultation-Liaison Service in an Academic Medical Center Joji Suzuki, MD, Brigham and Women's Hospital/Harvard Medical School, Boston, Massachusetts; J Kauffman, M Renaud, S Brenner, E Margetson, J Peteet, D Curtiss, A Fife Background: Substance use disorders contribute significantly to the morbidity and mortality of hospitalized medical patients. To better manage medical/surgical patients with substance use disorders in the general hospital setting, a specialized multidisciplinary addiction consultation-liaison team (Addiction Psychiatry Service) has been providing expert diagnostic, management, interventional, and referral services. Methods: Consultations received by the Addiction Psychiatry Service during the period of January 2008 through December 2008 were reviewed for demographic and clinical characteristics. The history, development, composition of the service, as well as the integration with the existing psychiatric consultation-liaison service were also examined. Results: A total of 996 patients were referred to Addiction Psychiatry Service in the year 2008, with the following demographic and clinical characteristics: mean age 51.6 (range 17-87), male 63.9%, ethnicity (White 47.7%, African-American 31.8%, Latino 20.5%), consulting service (medicine 56.8%, surgery 17.8%, ICU 15.1%, OB/GYN 6.2%, ED 2.1%, neurology 2.1%), reason for consultation (withdrawal management 61.3%, counseling/referral to treatment 44.9%, delirium 11.4%, pain vs. addiction 10.2%, transfer to detoxification treatment 5.1%), substances used (alcohol 59.1%, opioids 26.1%, cocaine 22.1%, cannabis 7.6%, benzodiazepines 3.6%, amphetamines .4%, barbiturates .4%), and referrals accepted by patient (outpatient individual 37.1%, intensive outpatient 23.6%, methadone maintenance 11.2%, outpatient dual diagnosis 7.9%, inpatient detoxification 5.6%, residential and partial hospital 3.4%). Conclusions: Hospitalized patients in an academic medical setting have high rates of substance use disorders. A multidisciplinary addiction consultation-liaison service may be a useful model for supporting the medical/surgical staff to better care for patients with substance use disorders. Source of Funding: None. Poster 16: Ethnographic Survey of Hispanic Adolescent Heroin Users in Dallas County Carlos F Tirado, MD, MPH, University of Texas Southwestern Medical Center, Dallas, Texas; C Coton, KR Williams, JC Maxwell, B Adinoff Background: Since 2005, Dallas County has seen an unprecedented rise in the rate of intranasal and intravenous heroin use among Hispanic children and adolescents. There are documented cases of children as young as nine seeking opioid detoxification and there have been at least 26 heroin-related deaths in children under age 18 since 2005. Statewide, Hispanics represent the largest proportion of heroin users entering treatment under age 25. Methods: Seventy-two (N = 72) consecutive English speaking/reading respondents of Mexican and Mexican-American origin between age 13 and 18 with a primary diagnosis of heroin dependence were recruited from three participating drug rehab programs over a 7-month period. Subjects participated in a 90-minute self-report and structured interview session to obtain data on intranasal heroin use patterns, psychiatric comorbidity, high-risk sexual behaviors, and cultural and familial factors related to level of acculturation and intergenerational drug and alcohol use. Results: Sixty percent of respondents were female.

Roughly one-third of respondents were born in Mexico versus 85% of parents reported as Mexican born. Average age at interview was 15.7 years with average age of first use of heroin 13.5 years and age of initiation of daily use 14.1 years. One in six respondents had used heroin IV and one in four reported at least one certain heroin-related overdose. Nearly half (46%) of respondents reported they first tried heroin on school grounds. A third of respondents reported greater than four lifetime sexual partners. Fifty-one percent reported sometimes or rarely/never using a condom. Seventy-four percent of respondents reported at least one prior 30+ day residential treatment episode. One in five reported prior psychiatric treatment for depression. The most common secondary drugs of abuse were marijuana (46%), cocaine (22%), and alcohol (21%). One in six reported some form of physical, sexual, or psychological trauma. Forty-percent reported having used alcohol to the point of intoxication with a first or second degree relative and 46% reported using drugs to get high with a first- or second-degree relative. Preliminary hypothesis testing revealed no statistically significant relationship between level of acculturation and IV drug use or frequency of treatment entry. Conclusions: There are clear and concerning trends signaling the disproportionate and increasing use of intranasal heroin among young Hispanics in North Texas. Adolescent Hispanics who are in treatment centers have well-established heroin dependence and problem use of several other drugs. The use of heroin in area schools is troubling, but provides opportunity for assertive preventive measures. The presence of a psychiatri
SN - 10550496
UR -
https://login.proxy.bib.uottawa.ca/login?url=http://search.ebscohost.com/login.aspx?direct=true&db=s3h&AN=64503074&site=ehost-live
ER -

TY - JOUR
ID - 4654
T1 - Treatment of Epileptic Encephalopathies
A1 - McTague,Amy
A1 - Cross,J.Helen
Y1 - 2013/03//
KW - SportDiscus
KW - *BRAIN damage
KW - *NEUROLOGY
KW - *PATHOLOGY
KW - *SPASMS
KW - *CENTRAL nervous system depressants
KW - Treatment
KW - DEVELOPMENTAL delay
RP - NOT IN FILE
SP - 175
EP - 184
JA - CNS Drugs
VL - 27
IS - 3
N2 - Epileptic encephalopathy is defined as a condition where the epileptic activity itself may contribute to the severe neurological and cognitive impairment seen, over and above that which would be expected from the underlying pathology alone. The epilepsy syndromes at high risk of this are a disparate group of conditions characterized by epileptic seizures that are difficult to treat and developmental delay. In this review, we discuss the ongoing debate regarding the significance of inter-ictal discharges and the impact of the seizures themselves on the cognitive delay or regression that is a common feature of these syndromes. The syndromes also differ in many ways and we provide a summary of the key features of the earlyonset epileptic encephalopathies including Ohtahara and West syndromes in addition to later childhood-onset syndromes such as Lennox Gastaut and Doose syndromes. An understanding of the various severe epilepsy syndromes is vital to understanding the rationale for treatment. For example, the resolution of hypsarrhythmia in West syndrome is associated with an improvement in cognitive outcome and drives treatment choice, but the same cannot be applied to frequent inter-ictal discharges in Lennox Gastaut syndrome. We discuss the evidence base for treatment where it is available and describe current practice where it is not. For example, in West syndrome there is some evidence for preference of hormonal treatments over vigabatrin, although the choice and duration of hormonal treatment remains unclear. We describe the use of conventional and newer anti-epileptic medications in the various

syndromes and discuss which medications should be avoided. Older possibly forgotten treatments such as sulthiame and potassium bromide also have a role in the severe epilepsies of childhood. We discuss hormonal treatment with particular focus on the treatment of West syndrome, continuous spike wave in slow wave sleep (CSWS)/electrical status epilepticus in slow wave sleep (ESES) and Landau Kleffner syndrome. The role of the ketogenic diet has in recent years come to the fore of the management of these severe epilepsies and we describe successful use in myoclonic astatic epilepsy, Lennox Gastaut syndrome and Dravet syndrome. It is important that resective epilepsy surgery is not ignored in the management of these children, particularly those with hemipathology who may present with ESES and respond well to hemispheric disconnection. Adjunctive and symptomatic surgical treatments such as vagal nerve stimulation and corpus callosotomy may improve seizure burden. Finally, it is vital that the identification and treatment of developmental, behavioural and psychiatric co-morbidities are not neglected and that a rational, holistic approach is taken to the management of epileptic encephalopathies. ABSTRACT FROM AUTHOR
SN - 11727047
UR -
https://login.proxy.bib.uottawa.ca/login?url=http://search.ebscohost.com/login.aspx?direct=true&db=s3h&AN=86219304&site=ehost-live
ER -

TY - JOUR
ID - 4656
T1 - Walk to school - does it make a difference in children's physical activity levels?
A1 - Michaud-Tomson,L.
A1 - Davidson,M.
A1 - Cuddihy,T.F.
Y1 - 2003///
KW - SportDiscus
KW - *PHYSICAL fitness
KW - *CARDIOVASCULAR fitness
KW - *WALKING
KW - Calibration
KW - Transportation
KW - Australia
KW - Child
KW - primary school
RP - NOT IN FILE
SP - 16
EP - 24
JA - ACHPER Healthy Lifestyles Journal
VL - 50
IS - 3-4
CY - ;
N2 - School and community based physical activity programs aim to increase the involvement of children through providing more comprehensive and extended access to opportunities to be active. This research project was initially devised to compare physical activity levels and practices of children at an 'Active Australia' school and at a school utilizing a school based Health and Physical Education program. The framework involved primary school children in grades 4-7 (girls, n=259; boys, n=232) wearing a pedometer and completing a physical activity survey each day for four days. The results of a One-way ANOVA indicated the daily step means of two schools differed significantly [F(1,489(=7.36, p=.007]. Irrespective of school, boys' means were significantly higher than girls [F (1,489)=53.04, p<.0001]. Sport was the most frequent outside of school activity cited. A significant difference for boys [F(3,284)=8.81, p<.0001] and girls [F(3,318)=13.92, p=<.0001] respectively was found for mode of transport to school. Children who walked to school averaged significantly more steps per day than those who travelled by bus or car. Chi-square indicated that those who walked to school were also significantly more likely to play sport. The Effect Size for walking versus all other forms of transport to school was 12.3, which is very large
SN - 13210394

UR -
https://login.proxy.bib.uottawa.ca/login?url=http://search.ebscohost.com/login.aspx?direct=true&db=s3h&AN=SPHS-928817&site=ehost-live
ER -

TY - JOUR
ID - 4659
T1 - PHYSICAL ACTIVITY IN PRE-SCHOOL CHILDREN FROM THE ASPECT OF HEALTH CRITERIA. / POHYBOVA_ AKTIVITA U P□~ED□ÿKOLNA_CH DŽ"TA_ Z HLEDISKA ZDRAVOTNA_CH KRITA%RIA_
A1 - MiklA -nkovA -,Ludmila
A1 - Elfmark,Milan
A1 - Sigmund,Erik
A1 - MitA -• -,Josef
A1 - FrA   mel,Karel
Y1 - 2009/03//
KW - SportDiscus
KW - *PHYSICAL activity
KW - *ENERGY metabolism
KW - *HEALTH behavior
KW - *CHILDREN -- Health
KW - PRESCHOOL children
KW - California
KW - environmental stimulation
KW - health recommendations
KW - Physical Activity
KW - pre-school age
KW - p • Ted • -kolni vŽ>k
KW - pohybova aktivita
KW - stimulace prost • Tedim
KW - zdravotni doporuŽ_eni
RP - NOT IN FILE
SP - 39
EP - 47
JA - Acta Universitatis Palackianae Olomucensis.Gymnica
VL - 39
IS - 1
N2 - The main aim of this study was to assess potential diff erences between groups of pre-school children meeting and not meeting health recommendations for physical activity (PA) from the aspect of so called indicators of physical activity (active energy expenditure, number of steps). The number of steps was measured by Yamax Digi-Walker SW-200 pedometers (Yamax Corporation, Tokyo, Japan) and the active energy expenditure was measured by Caltrac (Muscle Dynamics Fitness Network, Torrance, California) accelerometers. Evaluation of PA from the aspect of so called health criteria was realized following the criteria of FrA   mel, Novosad and Svozil (1999), who recommend values of so called health criteria of PA. Meeting these criteria, it is possible to suppose that PA positively aff ects the healthy development of an individual. The sample consisted of 200 pre-school children. Forty nine of these children (30 boys and 19 girls) were labeled as group A (children who did not meet the recommended values at least in one of the health criteria of PA). Group B (children who met the recommended values at least in one of the mentioned criteria of PA) consisted of 151 children (74 boys and 77 girls). The Mann-Whitney U test was used to test the statistical signifi cance of possible diff erences between groups A and B and also between boys and girls within these groups as well. In all monitored parts of a week, as well as in both monitored parts of a day, a signifi cant diff erence (p < .001) was found between groups A and B in both indicators of PA. Regarding these variables, the smallest diff erences (p < .02) were observed within the time spent at school. Our results confi rm that a stay at kindergarten belongs among the relevant parts of a day from the aspect of children's physical activity in the sense of a very "problematic" part of a day, when the values of active energy expenditure and steps are decreased almost by 50% even in children physically active outside the school. On the other hand, a kindergarten is a proper place

for children with hypokinetic behavior, who can be activated by an adequate incidence of school physical regimen. ABSTRACT FROM AUTHOR
SN - 12121185
UR -
https://login.proxy.bib.uottawa.ca/login?url=http://search.ebscohost.com/login.aspx?direct=true&db=s3h&AN=48188346&site=ehost-live
ER -

TY - JOUR
ID - 4661
T1 - The importance of recess
A1 - Miller,M.C.
Y1 - 2009/08//
N1 - Accession Number: 2010359400. Language: English. Entry Date: 20090911. Revision Date: 20090911. Publication Type: journal article. Journal Subset: Consumer Health; Editorial Board Reviewed; Expert Peer Reviewed; Peer Reviewed; USA. Special Interest: Consumer Health; Pediatric Care; Psychiatry/Psychology. NLM UID: 9417017
KW - CINAHL
KW - Child Behavior
KW - Child Development
KW - Play and Playthings -- Psychosocial Factors -- In Infancy and Childhood
KW - Child
RP - NOT IN FILE
SP - 8
EP - 8
JA - Harvard Mental Health Letter
VL - 26
IS - 2
SN - 1057-5022
UR - http://search.ebscohost.com/login.aspx?direct=true&db=cin20&AN=2010359400&site=ehost-live
ER -

TY - RPRT
ID - 4662
T1 - Feel Better Outside, Feel Better Inside: Ecotherapy for mental wellbeing, resilience and recovery
A1 - Mind
Y1 - 2013///
KW - Reviewer Nominated
RP - IN FILE
CY - London
PB - Mind
N2 - Ecotherapy is an intervention
that improves mental and physical
health and wellbeing by supporting
people to be active outdoors:
doing gardening, food growing or
environmental conservation work.

This report shows how ecotherapy is being used to
help people maintain good mental wellbeing - and how
the same intervention is increasingly being used as
treatment to help people recover from a range of mental
health problems.
SN - 978-1-906759-80-3
L1 - http://www.mind.org.uk/ecotherapy
ER -

TY - JOUR
ID - 4663
T1 - Zaleznosc pomiedzy intensywnoscia a kosztem energetycznym pracy. / The relation between intensity and energetical cost of work
A1 - Mirus,M.
Y1 - 1997///
KW - SportDiscus
KW - *ENERGY metabolism
KW - *PHYSIOLOGY
KW - *STRESS (Physiology)
KW - *EXERCISE -- Physiological aspects
KW - *OXYGEN -- Physiological transport
KW - *ENERGY expenditure
KW - *ATHLETES
KW - *SWIMMING
KW - BOYS
KW - Recovery
KW - BICYCLE ERGOMETRY
KW - CADENCE
KW - CORRELATION
KW - Child
RP - NOT IN FILE
SP - 101
EP - 109
JA - Wychowanie Fizyczne i Sport
VL - 41
IS - 4
CY - ;
N2 - The objective of the dissertation was to differentiate the influence of strength changes and, separately, of the motorial frequency on the energetical cost of physical work. The examinations were carried out on the group of twenty one 12-14 year old boys who had practiced swimming for 3-5 years. They underwent a 6 min effort test on a Jaeger bicycle cycloergometer. In all the tests, during a rest, work and restitution, the following factor was measured of oxygen inhaled (VO2). In all cases examined in my work differences essential for statistics characterize the changes of energetical cost more significantly during the work with a small or even without outside resistance, than during efforts with greater outside resistance
SN - 00439630
UR - https://login.proxy.bib.uottawa.ca/login?url=http://search.ebscohost.com/login.aspx?direct=true&db=s3h&AN=SPHS-670159&site=ehost-live;http://articles.sirc.ca/search.cfm?id=S-670159
L2 - http://articles.sirc.ca/search.cfm?id=S-670159
ER -

TY - JOUR
ID - 4664
T1 - Is physical activity in natural environments better for mental health than physical activity in other environments?
A1 - Mitchell,Richard
Y1 - 2012///
KW - Reviewer Nominated
RP - NOT IN FILE
JF - Social Science & Medicine
JA - Soc Sci Med
PB - Elsevier
SN - 0277-9536
ER -

TY - JOUR

ID - 4665
T1 - Selecting safer playground equipment
A1 - Mittelstaedt,Arthur
Y1 - 1997/11//
KW - SportDiscus
KW - *PLAYGROUNDS
KW - SAFETY measures
RP - NOT IN FILE
SP - 25
JA - Grounds Maintenance
VL - 32
IS - 11
N2 - Focuses on installing safe playground equipment. Accessibility to both parents and children; Correct positioning of the units such as the apparatus, equipment or composite units; Identification of the zone area for children's play; Play-surfaces safety standards; Play-equipment safety standards
SN - 00174688
UR -
https://login.proxy.bib.uottawa.ca/login?url=http://search.ebscohost.com/login.aspx?direct=true&db=s3h&AN=9712053433&site=ehost-live
ER -

TY - JOUR
ID - 4666
T1 - Unsafe to Play? Neighborhood Disorder and Lack of Safety Predict Reduced Physical Activity Among Urban Children and Adolescents
A1 - Molnar,Beth E.
A1 - Gortmaker,Steven L.
A1 - Bull,Fiona C.
A1 - Buka,Stephen L.
Y1 - 2004/05//
KW - SportDiscus
KW - *HEALTH
KW - *PHYSICAL fitness
KW - *CARDIOVASCULAR system -- Diseases
KW - *BODY weight
KW - *OUTDOOR recreation
KW - CITY children
KW - URBAN youth
KW - LONGITUDINAL method
RP - NOT IN FILE
SP - 378
EP - 386
JF - American Journal of Health Promotion
JA - Am J Health Promot
VL - 18
IS - 5
N2 - Purpose. Lack of physical activity is associated with increased risk of overweight and cardiovascular disease, conditions associated with lower socioeconomic status (SES). Associations between activity levels of urban youth and limited access to safe recreation areas in their neighborhoods of residence were investigated. Design. Analyses of data front the Project on Human Development in Chicago Neighborhoods, a multilevel longitudinal study of families and communities, are reported. Setting. Chicago, Illinois. Subjects. Individual-level data were obtained front 1378 youth 11 to 16 years old and caregivers living in 80 neighborhood clusters. Neighborhood-level data were collected front 8782 community residents and videotapes of 15,141 block faces. Measures. Parental estimates of hours youth spent in recreational programming were used to estimate physical activity. A scale of residents' assessment of neighborhood safety for children's play was created; disorder measures came front videotaped observations. Results. Physical activity averaged 2.7 hours/week (SD = 5.0), varying significantly across neighborhoods. Using hierarchical linear regression, SES, age, and male gender, but

not body mass index, were independently associated with physical activity. Lower neighborhood safety and social disorder were significantly associated with less activity, controlling for demographics. Conclusions. One mechanism for reduced physical activity among youth may be the influence of unsafe neighborhoods. Neighborhood interventions to increase safety and reduce disorder may be efficacious in increasing physical activity, thereby reducing risk of overweight and cardiovascular disease. ABSTRACT FROM AUTHOR
SN - 08901171
UR -
https://login.proxy.bib.uottawa.ca/login?url=http://search.ebscohost.com/login.aspx?direct=true&db=s3h&AN=13264823&site=ehost-live
ER -

TY - JOUR
ID - 4667
T1 - Design pattern for children's environments: synopsis of a two-year research and design project
A1 - Moore,G.T.
A1 - Cohen,U.
A1 - McGinty,T.
Y1 - 1979///
KW - SportDiscus
KW - *PLAYGROUNDS
KW - *SPORTS facilities
KW - DESIGN
KW - standards
KW - Child Care
KW - Child
KW - Policy
RP - NOT IN FILE
CY - Arlington, Va.;
PB - ERIC
UR -
https://login.proxy.bib.uottawa.ca/login?url=http://search.ebscohost.com/login.aspx?direct=true&db=s3h&AN=SPH83760&site=ehost-live
ER -

TY - JOUR
ID - 4668
T1 - Italian physical culture demonstration: a report of the visit, training and accomplishments of the forty Italian students who were guests of Bernarr Macfadden during a stay of six months in the United States studying his methods of physical culture
A1 - Morgan,T.B.
A1 - DeVitalis,C.M.
Y1 - 1998///
KW - SportDiscus
KW - *PHYSICAL education & training
KW - *CARDIOVASCULAR fitness
KW - *TRAINING
KW - *BACK
KW - *STRESS (Physiology)
KW - *DIET
KW - *NUTRITION
KW - MALES
KW - Case Studies
KW - strength
RP - NOT IN FILE
CY - New York;
PB - Macfadden Book Co.

N2 - The authors describe the events that lead to forty Italian physical culture student instructors being brought to the United States for a six-month study program under physical culturist, Bernarr Macfadden. In 1930 Macfadden went to Europe to study health conditions as a member of US President Hoover's Committee on Child Health and Welfare. In Italy he met with Signor Renato Ricci, Italian Under-Secretary of State for Physical Education who described and showed Macfadden physical training facilities, classes, and programs. He also met with Benito Mussolini, and after many conferences with Ricci and others, he invited Mussolini to send 40 students to the United States to work with him and learn Macfadden's physical fitness program. Other chapters describe the introduction of the students to New York City, and the various cultural activities shown to them upon their arrival. The focus of the physical training program included exercises which strengthened the spine and muscles, the participation in sports of basketball, baseball, tennis, wrestling, boxing and track, and a diet of "eating only a sufficient amount of good food to nourish the body and no more." During the experiment, the diet included some fruit and meat, with emphasis on freshly ground wheat, rye and corn. The latter portion of the book has text and photographs of the exercises used and the forty individual records of each man's physique measurements at the beginning and the end of the training program. Also included in the individual records is the outcome of tests at the beginning and end of the program including, two hand press, back lift, arm pull ups, abdominal lift, deep knee bend, 100 yard dash, one mile run
UR -
https://login.proxy.bib.uottawa.ca/login?url=http://search.ebscohost.com/login.aspx?direct=true&db=s3h&AN=SPHS-662519&site=ehost-live
ER -

TY - JOUR
ID - 4669
T1 - Measured effects on children in the primary grades from use of selected playground equipment
A1 - Morris,M.R.
Y1 - 1955///
KW - SportDiscus
KW - *PLAYGROUNDS
KW - *SPORTS facilities
KW - *GAMES
KW - *PLAY
KW - Child
KW - equipment
RP - NOT IN FILE
CY - Eugene, Ore.;
PB - Univ. of Oregon
UR -
https://login.proxy.bib.uottawa.ca/login?url=http://search.ebscohost.com/login.aspx?direct=true&db=s3h&AN=SPH4370&site=ehost-live;http://kinpubs.uoregon.edu/
ER -

TY - JOUR
ID - 4670
T1 - The integration of anatomy and physiology into fifth grade physical education
A1 - Morrison,C.J.
Y1 - 1998///
KW - SportDiscus
KW - *ANATOMY
KW - *TEACHING
KW - *PHYSICAL education & training
KW - Elementary Schools
KW - PROGRAMS
KW - Methodology
KW - physiology
KW - Child
RP - NOT IN FILE
CY - Eugene, OR;

PB - Microform Publications, University of Oregon
N2 - The purpose of this study was to measure the effectiveness of integrated anatomy and physiology lessons on fifth grade students taught in their physical education class. Knowledge base, attitudes, and participation level changes were the targeted areas during the study. This quasi-experimental study looked at the changes that took place in the control group and the experimental group over the eight week study. The evaluations of this project were obtained through analyzing the test scores of the control and experimental groups; a pre-test and a post-test were administered. There was an attitude survey to identify changes in attitudes before and after the lessons were taught. Other evaluations came from teacher observations, recorded in journal entries, and from looking at student journal entries throughout the unit. The results showed a dramatic increase in the content knowledge of the experimental group. The control group showed little improvement; very few made dramatic increases in scores. Another very noticeable difference was in the attitude and behavior of the two classes. The control group, who were exposed only to the standard physical education curriculum, were very negative about the test and general information, even when being covered in the class. They could not connect the information with activities, because there was never enough taught to them. The experimental group were eager to take the post-test and had confidence in what they were being tested on. They referred to the information and used the terms in general conversation outside of the classes where the material was covered. The things they were learning had a purpose. The learning became part of their physical education class and connections between the material and physical activities were made by many students
UR -
https://login.proxy.bib.uottawa.ca/login?url=http://search.ebscohost.com/login.aspx?direct=true&db=s3h&AN=SPHS-791506&site=ehost-live;http://kinpubs.uoregon.edu/
ER -

TY - JOUR
ID - 4672
T1 - Psychological determinants of risk taking by children: an integrative model and implications for interventions
A1 - Morrongiello,B.A.
A1 - Lasenby-Lessard,J.
Y1 - 2007/02//
N1 - DA - 20070213
IS - 1353-8047 (Print)
IS - 1353-8047 (Linking)
LA - eng
PT - Journal Article
PT - Research Support, Non-U.S. Gov't
SB - IM
KW - Accident Prevention
KW - Methods
KW - Age Factors
KW - Child
KW - Child Behavior
KW - Psychology
KW - Family
KW - Female
KW - Humans
KW - Male
KW - Models,Psychological
KW - Motivation
KW - Parenting
KW - Play and Playthings
KW - Risk-Taking
KW - Sex Factors
KW - Social Behavior
KW - Reviewer Nominated
RP - NOT IN FILE
SP - 20

EP - 25
JF - Injury Prevention
JA - Inj Prev
VL - 13
IS - 1
N2 - OBJECTIVES: To draw on empirical findings of the psychological factors that cause elementary-school children to engage in risky play behaviors that can lead to injury, with the aim of developing an integrative model that can support intervention-program planning. METHODS: An extensive review of literature on this topic was conducted, determinants of risk taking for which there was empirical support were identified, and results were synthesized to create an integrative model of children's risk taking. RESULTS: Research on risk taking in children is limited, but the findings support the importance of examining child, family and socio-environmental factors to understand children's risk-taking behaviors. CONCLUSIONS: Development of a model outlining the determinants of risk behaviors can provide a foundation for initiatives that aim to reduce such behaviors and prevent childhood injuries
AD - Psychology Department, University of Guelph, Guelph, Ontario, Canada. bmorrong@uoguelph.ca
UR - 17296684
ER -

TY - JOUR
ID - 4674
T1 - Interval training: the effects of a 12-week programme on elite, pre-pubertal male soccer players. / Interval training: les effets d' un programme d' une duree de 12 semaines chez des joueurs d' elite de football, d' age pre-pubertaire
A1 - Mosher,R.E.
A1 - Rhodes,E.C.
A1 - Wenger,H.A.
A1 - Filsinger,B.
Y1 - 1985/03//
KW - SportDiscus
KW - *PHYSIOLOGY
KW - *SOCCER
KW - *CARDIOVASCULAR fitness
KW - *ANAEROBIC capacity
KW - *ATHLETES
KW - TESTING
KW - BOYS
KW - INTERVAL TRAINING
KW - Child
KW - INTERVAL-TRAINING
KW - EFFET-DE-L-ENTRAINEMENT
KW - athlete
KW - ENFANT
KW - CAPACITE-PHYSIQUE
KW - AEROBIE
KW - ANAEROBIE
KW - Football
RP - NOT IN FILE
SP - 5
EP - 9
JF - Journal of Sports Medicine & Physical Fitness
JA - J Sports Med Phys Fitness
VL - 25
IS - 1/2
CY - ;
N2 - The purpose of this study was to determine the effects of a 12-week interval fitness training program on pre-pubertal, elite-level male soccer players. Thirteen 10 and 11 year old soccer players served as the experimental group while 11 age-matched players from a team in the same league acted as controls. All subjects

were tested prior to and following a 12-week training programme. The tests included: an anaerobic speed test (SAT) in which subjects performed a treadmill run at 7 m.p.h. and an 18 percent grade, until volitional fatigue; 7 repeats of 40 yds. every 20 seconds, and a timed 1-mile endurance run. The 12 week anaerobic training programme for the experimental group consisted of 4 different high speed activities performed over 15-20 minutes 3 times weekly at a work: rest ratio of between 1:2 and 1:3. Analysis of variance for a 2 by 2 (Exp-Count, Pre-Post) factorial design revealed a significant Groups by Pre-Post interaction on all variables except the 40 yard speed measure. It was concluded that the 12-week interval training programme significantly increased the anaerobic and aerobic fitness of pre-pubertal, elite-level male soccer players
(HERACLES) Etude des effets d' un programme d' entrainement d' interval training, axe sur le developpement du metabolisme anaerobie lactique et alactique, sur les capacites aerobie et anaerobie d' enfants ages de 10 a 11 ans, joueurs de football de competition; les tests anaerobie consistent en une epreuve de vitesse sur tapis roulant (7 mph) avec pente de 18 % et une course de vitesse de 40 yards; le test aerobie en course d' endurance de 1 mile
SN - 00224707
UR -
https://login.proxy.bib.uottawa.ca/login?url=http://search.ebscohost.com/login.aspx?direct=true&db=s3h&AN=SPH172443&site=ehost-live;http://articles.sirc.ca/search.cfm?id=172443
L2 - http://articles.sirc.ca/search.cfm?id=172443
ER -

TY - JOUR
ID - 4675
T1 - Ideas: activity boxes encourage an active recess
A1 - Moss,D.
Y1 - 2002/06//
KW - SportDiscus
KW - *PLAY
KW - *RECESSES
KW - Child
KW - equipment
RP - NOT IN FILE
SP - 26
EP - 26
JA - Physical Education Digest
VL - 18
IS - 4
CY - ;
N2 - Advocates introduction of activity box to recess. Suggests appropriate equipment for inclusion
SN - 08432635
UR -
https://login.proxy.bib.uottawa.ca/login?url=http://search.ebscohost.com/login.aspx?direct=true&db=s3h&AN=SPHS-828790&site=ehost-live
ER -

TY - JOUR
ID - 4676
T1 - Ideas: teach elementary students games for recess
A1 - Moss,D.
Y1 - 2003/03//
KW - SportDiscus
KW - *PHYSICAL education & training
KW - *GAMES
KW - *RECESSES
KW - Elementary Schools
RP - NOT IN FILE
SP - 23
EP - 23

JA - Physical Education Digest
VL - 19
IS - 3
CY - ;
N2 - Suggests games for the physical education classroom that children can play during recess
SN - 08432635
UR -
https://login.proxy.bib.uottawa.ca/login?url=http://search.ebscohost.com/login.aspx?direct=true&db=s3h&AN=SPHS-881909&site=ehost-live;http://articles.sirc.ca/search.cfm?id=S-881909;http://www.pedigest.com/
L2 - http://articles.sirc.ca/search.cfm?id=S-881909
ER -

TY - JOUR
ID - 4677
T1 - Gender differences in physical activity during recess in Portuguese primary schools
A1 - Mota,J.
A1 - Stratton,G.
Y1 - 2003/09//
KW - SportDiscus
KW - *PHYSICAL fitness
KW - *RECESSES
KW - *CARDIOVASCULAR fitness
KW - *STRESS (Physiology)
KW - *PLAY
KW - Elementary Schools
KW - Comparative Studies
KW - Portugal
KW - Child
KW - SEX FACTOR
KW - primary school
RP - NOT IN FILE
SP - 150
EP - 150
JA - Revista Portuguesa de Ciencias do Desporto
VL - 3
IS - 2
CY - ;
SN - 16450523
UR -
https://login.proxy.bib.uottawa.ca/login?url=http://search.ebscohost.com/login.aspx?direct=true&db=s3h&AN=SPHS-898025&site=ehost-live;http://articles.sirc.ca/search.cfm?id=S-898025;http://www.fcdef.up.pt
L2 - http://articles.sirc.ca/search.cfm?id=S-898025
ER -

TY - JOUR
ID - 4681
T1 - Utjecaj karakteristicnih obiljezja socijalnog statusa na bavljenje kinezioloskim aktivnostima. / The effect of the characteristic traits of the social status on taking up kinesiologic activities
A1 - Mrakovic,M.
A1 - Metikos,D.
A1 - Katic,R.
Y1 - 1988///
KW - SportDiscus
KW - *KINESIOLOGY
KW - *PARTICIPATION
KW - WOMEN -- Sociological aspects
KW - Women

KW - Students
KW - Research
KW - Questionnaires
KW - SOCIAL factors
RP - NOT IN FILE
SP - 73
EP - 78
JA - Kineziologija
VL - 20
IS - 1
CY - ;
N2 - The investigation was performed in order to establish the effect of the social status on taking up kinesiologic activities. The data obtained in the questionnaire for assessment of the social status and scales for establishing the intensity of kinesiologic activity were processed by means of the factor and regression analysis. The results show: (1) there are relatively low but statistically significant relations between the social status and taking up kinesiologic activities. (2) the greatest positive effect on taking up kinesiologic activities is the dimension named as the socio-political status, to a certain extent also the so called life style, political orientation and the basic residential status. The negative effect, although statistically smaller is the educational status of the mother and the basic economic status. Besides the statistical reasons there are some objective ones of the obtained results. A greater participation of women in sports is related with the status of the parents in society and at work, but also with the nature of influence of the social environment on the individual, particularly in childhood, as well as with the political orientation and a higher standard of living, while the educational status of the mother is on the margins of positive social power, at least in the case of women who participated in this investigation. A few hypotheses have been postulated for these results that should be checked since they differ from some investigations carried out so far
SN - 03511057
UR -
https://login.proxy.bib.uottawa.ca/login?url=http://search.ebscohost.com/login.aspx?direct=true&db=s3h&AN=SPH357236&site=ehost-live;http://articles.sirc.ca/search.cfm?id=357236
L2 - http://articles.sirc.ca/search.cfm?id=357236
ER -

TY - JOUR
ID - 4682
T1 - How informed are the parents about the burden of injuries?
A1 - Mujkic,A.
A1 - Miskulin,M.
A1 - Crnica,V.
A1 - Kovacic,L.
Y1 - 2010/09/02/
KW - SportDiscus
RP - NOT IN FILE
SP - A119
EP - A119
JF - Injury Prevention
JA - Inj Prev
VL - 16
N2 - Objective Determining the knowledge and attitudes towards injury prevention/safety promotion (IPSP) for pre-school children among Croatian parents of newborn babies. Participants 351 parents (285 mothers, 66 fathers). Methods Anonymous self-administered questionnaire containing demographic items and items on knowledge and attitudes regarding IPSP for pre-school children. Main Outcome Measures Knowledge and attitudes towards IPSP for pre-school children. Results The answers the parents gave varied greatly for the 40 questions asked. For example: almost all parents showed correct attitudes and knowledge regarding leaving an infant unsupervised in water (99.3% mothers, 100% fathers answered correctly), having pre-school child sitting at the front seat if held by an adult (99.3% mothers, 100% fathers answered correctly) and playground safety (98.2% mothers, 100% fathers answered correctly). An adequate number (73.9% mothers, 76,9% fathers) chose the correct answer on the question related to the storage of chemicals and a similar number (77.5% mothers,

65.6% fathers) said they indeed did store them in the proper manner. It was shocking to see how low they scored on some very important questions: 35.9% mothers and 23.1% fathers recognise child-walkers as dangerous and only 12.7 fathers and 20.0% mothers knew that the safest position for sleeping for an infant is on the back. Conclusions Parents play a crucial role in injury prevention/safety promotion as models for their children, their educators and persons in the position to intervene within environment making it safer. Therein lays the importance of their sufficient knowledge and positive attitudes. Our results show a need for further public health education of the parents. ABSTRACT FROM PUBLISHER
SN - 13538047
UR -
https://login.proxy.bib.uottawa.ca/login?url=http://search.ebscohost.com/login.aspx?direct=true&db=s3h&AN=66042721&site=ehost-live
ER -

TY - JOUR
ID - 4683
T1 - Validation of the Caltrac movement sensor using direct observation in young children
A1 - Mukeshi,M.
A1 - Gutin,B.
A1 - Anderson,W.
A1 - Zybert,P.
A1 - Basch,C.
Y1 - 1990/08//
KW - SportDiscus
KW - *ENERGY metabolism
KW - *ENERGY expenditure
KW - *STRESS (Physiology)
KW - *CARDIOVASCULAR fitness
KW - INFANTS
KW - TESTING
KW - Test Reliability
RP - NOT IN FILE
SP - 249
EP - 254
JF - Pediatric Exercise Science
JA - Pediatr Exerc Sci
VL - 2
IS - 3
CY - ;
N2 - The validity of the Caltrac movement sensor for use with preschool children was assessed. Caltrac-derived values for energy expenditure were compared with those derived via laborious coding of direct observation that involved classification of the child's videotaped activity every other 5 seconds for an hour in the day-care center or on the playground. Both Caltrac and direct observation values were expressed in kilocalories. The subjects were 20 children with a mean age of 35 months. The correlation coefficient for the total of indoor and outdoor activity was r=.62 (p is less than .01). The separate correlations for indoor and outdoor activity were r=.56 (p is less than .05) and r=.48 (p is less than .05), respectively. However, when the children's weight, height, age, and sex were factored our of both the Caltrac and direct observation scores, the correlations fell to r=.25 (n.s.), r=47 (p is less than .05), and r=.16 (n.s.) for the total, indoor and outdoor activity, respectively. Thus the Caltrac seemed to record indoor activity (mainly walking) more accurately than it recorded the more varied playground movements, casting doubt on its value as a means of measuring physical activity in children 2-3 years of age
SN - 08998493
UR -
https://login.proxy.bib.uottawa.ca/login?url=http://search.ebscohost.com/login.aspx?direct=true&db=s3h&AN=SPH272237&site=ehost-live;http://articles.sirc.ca/search.cfm?id=272237
L2 - http://articles.sirc.ca/search.cfm?id=272237
ER -

TY - JOUR

ID - 4685
T1 - Children's perceived physical competence at different categories of physical activity. / Perception par les enfants de leurs capacites physiques en fonction de differents types d'activite physique
A1 - Mullan,E.
A1 - Albinson,J.
A1 - Markland,D.
Y1 - 1997/08//
KW - SportDiscus
KW - *PSYCHOLOGY
KW - *STRESS (Physiology)
KW - *SPORTS
KW - SELF-perception
KW - Ability
KW - Child
KW - PROGRESSION
KW - ACTIVITE-PHYSIQUE
KW - ENFANT
KW - APTITUDE-PHYSIQUE
KW - CAPACITE-PHYSIQUE
KW - AUTO-EVALUATION
KW - Evaluation
KW - questionnaire
KW - CONDITION-PHYSIQUE
KW - Motivation
RP - NOT IN FILE
SP - 237
EP - 242
JF - Pediatric Exercise Science
JA - Pediatr Exerc Sci
VL - 9
IS - 3
CY - ;
N2 - This study explored whether children differentiate between their physical capabilities at play activities, informal recreational activities, and formal competitive activities. Harter's (11) six-item Athletic Competence subscale from the Self-Perception Profile for Children was administered to 578 children and adolescents (ages 7-15 years). The items were modified to refer to three different categories of physical activity instead of sport or outdoor games as used in the original subscale. Repeated measures ANOVA revealed that children differentiated between the three categories of physical activity, and that the competitive sport category was their area of lowest perceived competence. Males had higher levels of perceived competence than females in each category
SN - 08998493
UR -
https://login.proxy.bib.uottawa.ca/login?url=http://search.ebscohost.com/login.aspx?direct=true&db=s3h&AN=SPH457301&site=ehost-live;http://articles.sirc.ca/search.cfm?id=457301;http://www.humankinetics.com/
L2 - http://articles.sirc.ca/search.cfm?id=457301
ER -

TY - JOUR
ID - 4686
T1 - End of the Line?
A1 - Munson,Lester
A1 - Chadiha,Jeffri
A1 - Kennedy,Kostya
A1 - Bechtel,Mark
Y1 - 2003/07/07/
KW - SportDiscus
KW - *WOMEN

KW - TAMPA Bay Buccaneers (Football team)
KW - family violence
KW - WIFE abuse
KW - MARITAL violence
KW - CRIMES against
KW - TAMPA Bay (Fla.)
KW - Florida
KW - United States
RP - NOT IN FILE
SP - 22
JA - Sports Illustrated
VL - 99
IS - 1
N2 - It's not just baseball players who know that the third strike is a bitter pill. Michael Pittman, 27, the Buccaneers' running back you last saw celebrating his 124-yard performance in the Super Bowl, may have a long wait before he plays his next down. Pittman was arrested on May 31 outside his Phoenix home and charged with two counts of felony assault on his wife, Melissa, who on Mother's Day had given birth to their second child, a daughter. According to police reports Pittman and Melissa, who have been married for four years, began arguing after Melissa found phone numbers belonging to other women on Pittman's cellphone bill. Police say the argument unfolded outside the house, where Pittman berated Melissa with profanities and then rammed his Hummer into the passenger side of Melissa's Mercedes as she tried to drive away
SN - 0038822X
UR -
https://login.proxy.bib.uottawa.ca/login?url=http://search.ebscohost.com/login.aspx?direct=true&db=s3h&AN=10171149&site=ehost-live
ER -

TY - JOUR
ID - 4689
T1 - Asociaciones entre el IMC, la realizaciA3n de actividad fA-sica y la calidad de vida en adolescentes. / Relationships among BMI, physical activity practice and quality of life in teenagers
A1 - Muros Molina,JosAc JoaquA-n
A1 - Castillo,Antonio Som
A1 - GarcA-a de la Serrana,Herminia LA3pez
A1 - DA-az,Mikel Zabala
Y1 - 2009/11//
KW - SportDiscus
KW - *QUALITY of life
KW - *TEENAGERS -- Health
KW - *BODY mass index
KW - *PHYSICAL activity
KW - *WELL-being
KW - *PHYSICAL fitness
KW - *NUTRITION disorders
KW - Research
KW - Age Factors
KW - GRANADA (Spain)
KW - Spain
KW - Health
KW - Obesity
KW - Physical Activity
KW - Quality of Life
KW - youth
KW - actividad fA-sica
KW - calidad de vida
KW - jA3venes
KW - obesidad

KW - salud
RP - NOT IN FILE
SP - 159
EP - 165
JA - Cultura, Ciencia y Deporte
VL - 4
IS - 12
N2 - The aim of this study was to analyze the quality of life related to health (QLRH) and physical activity (PA) patterns of adolescents with overweight or obesity and adolescents with normal weight. A total of 106 subjects (12.06 Â±0.9 years of age) from a school in the city of Granada (Spain) participated in the study. The Kid-Kindl version of the Kindl questionnaire (adapted and validated to Spanish) was used to measure QLRH by means of the dimensions of physical well-being, emotional well-being, self-esteem, family, friends, and school. Results demonstrate that the percentage of active boys (those that practice 2 or more hours of moderate to vigorous PA outside of school) is significantly greater than the percentage of active girls. When comparing the PA done by subjects with normal weight and those with obesity/overweight, it is observed that subjects with normal weight are significantly more active. For the points obtained in the questionnaire about quality of life, it is shown that subjects with normal weight obtain significantly better scores in general. This difference is also observed in all other dimensions of the questionnaire, except in the "Family" dimension where there are no differences; however, statistically significant differences are seen only in the "Self-esteem", and "School" dimensions. Given these results, it is necessary to develop intervention plans to generate healthy habits in children and adolescents, so they can maintain them in the future, and in this way reverse the tendency toward being obese adults. ABSTRACT FROM AUTHOR
El estudio pretende analizar la calidad de vida relacionada con la salud (CVRS) y la realizaciÃ3n de actividad fÃ-sica entre adolescentes con sobrepeso u obesidad y adolescentes con normopeso. Participaron en el estudio un total de 106 alumnos (12,06 Â±0,9 aÃ±os) de un centro educativo ubicado en Granada capital. Como herramienta de recogida de datos se utilizÃ3 el cuestionario Kindl en su versiÃ3n Kid-Kindl (adaptado y validado al espaÃ±ol) para medir la CVRS a travÃ©s de las dimensiones de bienestar fÃ-sico, bienestar emocional, autoestima, familia, amigos y colegio. Los resultados muestran cÃ3mo el porcentaje de sujetos masculinos activos (realizan 2 o mÃ¡s horas de actividad fÃ-sica extraescolar de intensidad moderada o vigorosa) es bastante superior al de alumnas activas, siendo estas diferencias estadÃ-sticamente significativas. Al comparar la actividad fÃ-sica realizada por alumnos con normopeso y sobrepeso/ obesidad, observamos cÃ3mo los alumnos con normopeso son mÃ¡s activos, siendo estas diferencias estadÃ-sticamente significativas. En cuanto a las puntuaciones obtenidas en el cuestionario de calidad de vida, se puede comprobar que los alumnos con normopeso obtienen mejores puntuaciones en la puntuaciÃ3n global del cuestionario, siendo esta diferencia estadÃ-sticamente significativa. Lo mismo ocurre en todas las dimensiones del cuestionario, exceptuando la dimensiÃ3n "Familia", donde no existen diferencias, aunque la significatividad estadÃ-stica sÃ3lo se da en las dimensiones de "Autoestima" y "Colegio". A la vista de los resultados se hace imprescindible crear planes de intervenciÃ3n para generar hÃ¡bitos de vida saludables en niÃ±os, para que Ã©stos los puedan mantener en un futuro, y podamos invertir la tendencia de ser adultos obesos. ABSTRACT FROM AUTHOR
SN - 16965043
UR -
https://login.proxy.bib.uottawa.ca/login?url=http://search.ebscohost.com/login.aspx?direct=true&db=s3h&AN=48176968&site=ehost-live
ER -

TY - JOUR
ID - 4690
T1 - Spittin' venom
A1 - Murphy,Austin
Y1 - 1996/01/29/
KW - SportDiscus
RP - NOT IN FILE
SP - 174
JA - Sports Illustrated
VL - 84
IS - 4

N2 - Profiles Greg Lloyd, the five-time Pro Bowl outside linebacker of the Pittsburgh Steelers. His charitable contributions; Lloyd as one of the most feared players in the National Football League; The fine levied against him for a hit against Green Bay Packers' quarterback Brett Favre; His humble childhood years spent with his aunt, Bertha Mae Rumph, and nine other kids in a two-bedroom apartment after his mother abandoned him at age 2
SN - 0038822X
UR -
https://login.proxy.bib.uottawa.ca/login?url=http://search.ebscohost.com/login.aspx?direct=true&db=s3h&AN=9601317854&site=ehost-live
ER -

TY - JOUR
ID - 4691
T1 - Unintentional childhood injury burden, risks, and outcomes at the paediatric emergency unit in Kampala
A1 - Mutto,M.
A1 - Lawoko,S.
A1 - Nansamba,C.
A1 - Ovuga,E.
A1 - SvanstrA m,L.
Y1 - 2010/09/02/
KW - SportDiscus
RP - NOT IN FILE
SP - A34
EP - A34
JF - Injury Prevention
JA - Inj Prev
VL - 16
N2 - Abstract Unintentional Childhood Injury burden, outcomes and risks are assessed in Kampala. Proportional incidence rates, percentages and injury odds are computed; risk trends are constructed. Five hundred and fifty-six childhood injuries were recorded between January and May 2008. Male: female ratio was 15:10. Parents transported most patients to hospital using mini-buses, private cars and motorcycles. Median injury location was 5km. Homes, roads, schools were lead locations. Play and daily living activities are commonest injury time activities. Falls, burns and traffic constitute 70.5% of injuries. Burns, open wounds, fractures are commonest. Motorcycles, buses and passenger cars caused most crashes. Play grounds, furniture, stairs and trees were commonest fall sources. Liquids, fires and hot objects cause most burn. 43.8% of cases were admitted. 30% were not disabled; 10%, disabled; 1%, fatal. Injury odds varied with age, place and cause. We failed to capture poisoning and drowning. Local paediatric injury priorities should include home, road and school safety. ABSTRACT FROM PUBLISHER
SN - 13538047
UR -
https://login.proxy.bib.uottawa.ca/login?url=http://search.ebscohost.com/login.aspx?direct=true&db=s3h&AN=66042384&site=ehost-live
ER -

TY - JOUR
ID - 4692
T1 - Playground interactions for preschool-age children with special needs
A1 - Nabors,L.
A1 - Badawi,M.
Y1 - 1997/09//
N1 - Accession Number: 1998043620. Language: English. Entry Date: 19980701. Revision Date: 20091218. Publication Type: journal article; research; tables/charts. Journal Subset: Allied Health; Double Blind Peer Reviewed; Editorial Board Reviewed; Expert Peer Reviewed; Peer Reviewed; USA. Instrumentation: ABILITIES Index. Grant Information: Department of Education. No. of Refs: 28 ref. NLM UID: 8109120
KW - CINAHL
KW - Play and Playthings -- Evaluation
KW - Special Populations -- In Infancy and Childhood

KW - Interpersonal Relations -- Evaluation -- In Infancy and Childhood
KW - Funding Source
KW - Multivariate Analysis of Variance
KW - Sex Factors
KW - Age Factors
KW - Ethnic Groups
KW - Cross Sectional Studies
KW - Aptitude Tests
KW - Research Instruments
KW - Kappa Statistic
KW - Repeated Measures
KW - Child,Preschool
KW - Adult
KW - Middle Age
KW - Male
KW - Female
KW - Human
RP - NOT IN FILE
SP - 21
EP - 31
JF - Physical & Occupational Therapy in Pediatrics
JA - Phys Occup Ther Pediatr
VL - 17
IS - 3
N2 - Forty-five typically-developing children and 19 children with special needs, ages 3 to 5 years, were observed interacting on the playground. Observers recorded three types of play engaged in by the children. These included: (1) playing alone, (2) playing with a teacher, or (3) playing cooperatively with peer(s). Scan-sampling was the observational technique used by the observers. This technique required a snapshot observation of a child at play for 10 seconds. Results indicated that children with special needs engaged in less cooperative play and were observed playing alone or with a teacher more often than their typically-developing peers. Furthermore, children with special needs often arrived on the playground later than their typically-developing classmates; consequently, they had fewer opportunities to be involved in play with peers. Field notes made by observers indicated that in some instances teachers used effective strategies to facilitate the inclusion of children with special needs in cooperative interactions. Results and observations are discussed and recommendations for practice and future research are provided
SN - 0194-2638
AD - University of Maryland School of Medicine, University of Maryland at Baltimore, Dept of Pediatrics, Division of Behavioral and Developmental Pediatrics, 630 W Fayette St, Room 5-697, Baltimore, MD 21201-1585
UR - http://search.ebscohost.com/login.aspx?direct=true&db=cin20&AN=1998043620&site=ehost-live
ER -

TY - JOUR
ID - 4693
T1 - Physical activity patterns among 10-13-year old students engaged in team pentathlon with regards to age, sex, and season
A1 - Nadeau,L.
A1 - Martel,D.
A1 - Gagnon,J.
A1 - Michaud,V.
A1 - Godbout,P.
Y1 - 2013/02//
KW - SportDiscus
KW - *PHYSICAL activity
KW - *PENTATHLON
KW - *STUDENTS
KW - *PHYSICAL education

KW - CORRECTION factors (Mathematics)
KW - Elementary Schools
KW - SECONDARY schools
RP - NOT IN FILE
SP - 26
EP - 37
JA - International Journal of Physical Education
VL - 50
IS - 2
N2 - Background: Team pentathlon (TP) is designed to increase physical activity (PA) among children. Every day for eight weeks students register each PA episode completed outside of physical education classes. Depending upon the type and intensity of the activity, a correction factor is applied to the duration of each episode; a total of 60 corrected minutes represents one pentathlon hour (PH). The study intended to characterize elementary and secondary school students' patterns of PA in relation with grade level, sex and season. Methods: The study considered a total of 1496 students from grade five and six and secondary first students of both sexes who had completed TP either during fall or winter. The students' PA pattern considered weekly number of PA episodes, average duration of episodes, uncorrected weekly PA volume, average correction factor, and average number of PHs per week. Results: Activities selected by boys were significantly more intense than those selected by girls and their overall average number of PHs per week was slightly higher. Winter PA lasted longer than during fall but was less intense, resulting in a significant but meaningless difference between winter and fall in terms of weekly PHs. Overall, grade five students were more active than older students both in terms of uncorrected and corrected PA volume per week. Conclusion: Results of the study suggest that sex and season differences usually reported in the literature are not present when students engage in TP. ABSTRACT FROM AUTHOR
SN - 03418685
UR -
https://login.proxy.bib.uottawa.ca/login?url=http://search.ebscohost.com/login.aspx?direct=true&db=s3h&AN=89707327&site=ehost-live
ER -

TY - JOUR
ID - 4694
T1 - Ethnic and gender trends for cardiovascular risk behaviors in Anglo and Mexican American children, ages four to seven
A1 - Nader,P.R.
A1 - Sallis,J.F.
A1 - Broyles,S.L.
A1 - McKenzie,T.L.
A1 - Berry,C.C.
A1 - Davis,T.B.
A1 - Zive,M.M.
A1 - Elder,J.P.
A1 - Frank-Spohrer,G.C.
Y1 - 1995/03//
KW - SportDiscus
RP - NOT IN FILE
SP - S27
EP - s35
JA - Journal of Health Education
VL - 26
IS - 2
CY - ;
N2 - Gender and ethnic trends were examined in 351 Mexican American and Anglo American children between the ages of four and seven. Four physiologic variables, seven observed and reported variables related to diet, and eight observed and reported variables related to physical activity were periodically assessed. Children were observed at home at meal times and at school (or a day care center) at lunch and recess. A linear regression model was used. Of the four physiologic variables, two variables (height and total skinfolds) showed significant

ethnic differences, with Mexican American children showing greater skinfolds over time and the tendency to be shorter than Anglo American children. Of the variables related to diet, five of the seven variables showed gender or ethnic differences; percent of calories from fat and food preparation behaviors tended to place Mexican American children at greater risk. When examining physical activity, four of the eight variables showed gender and/or ethnic differences in trend. As children age, they get less active at home and more active and are prompted more often to be active. They also participated in more organized physical activities as they get older
SN - 10556699
UR -
https://login.proxy.bib.uottawa.ca/login?url=http://search.ebscohost.com/login.aspx?direct=true&db=s3h&AN=SPH386944&site=ehost-live;http://articles.sirc.ca/search.cfm?id=386944
L2 - http://articles.sirc.ca/search.cfm?id=386944
ER -

TY - JOUR
ID - 4696
T1 - A report of the civic activities in a children's ward [Japanese]
A1 - Naka,M.
Y1 - 2007///
N1 - Accession Number: 2009581538. Language: Japanese. Entry Date: 20070720. Publication Type: journal article; pictorial. Journal Subset: Asia; Double Blind Peer Reviewed; Nursing; Online/Print; Peer Reviewed. Special Interest: Pediatric Care. No. of Refs: 5 ref. NLM UID: 9213446
KW - CINAHL
KW - Pediatric Care
KW - Play Therapy
KW - Child
KW - Child,Preschool
KW - Hospital Units
KW - Infant
KW - Japan
RP - NOT IN FILE
SP - 60
EP - 67
JA - Bulletin of St.Luke's College of Nursing
IS - 33
N2 - These days, civic participation has been popularized in various fields of public service. It is well known that civic participation improves the quality of public service. In the area of medical service, civic participation has not been popular compared with other fields. For the staff of medical facilities, especially in hospitals, it is very important to protect the safety of patients. As a result they are not receptive to civic assistance from outside their realm. We can find some civic activities in children's ward in a few facilities offering children amusement and mental support. In this paper I would like to report one of such activities in a children's ward. It could be considered a model case of civic participation in medical service. The substance of this report is comprised of the "life story" of a woman who has managed it
SN - 0289-2863
UR - http://search.ebscohost.com/login.aspx?direct=true&db=cin20&AN=2009581538&site=ehost-live
ER -

TY - JOUR
ID - 4698
T1 - Dramatic play in early childhood
A1 - Needles,D.J.
Y1 - 1980///
KW - SportDiscus
KW - *PLAY
KW - Child
RP - NOT IN FILE
CY - ;

N2 - Discusses how small children learn through play. Proposes that play teaches them essential social and physical skills. Suggests that children who do not have a chance to develop their skills will be distinctly handicapped in approaching the world around them. Examines how children need a large amount of time exploring their physical world in order to enhance muscle development and make sense of the universe. Play is the way children communicate their ideas to themselves and is therefore of tremendous importance toward their overall intellectual development
UR - https://login.proxy.bib.uottawa.ca/login?url=http://search.ebscohost.com/login.aspx?direct=true&db=s3h&AN=SPH82113&site=ehost-live;http://articles.sirc.ca/search.cfm?id=82113
L2 - http://articles.sirc.ca/search.cfm?id=82113
ER -

TY - JOUR
ID - 4699
T1 - Ankle Injuries Among United States High School Sports Athletes, 2005-2006
A1 - Nelson,Alex J.
A1 - Collins,Christy L.
A1 - Yard,Ellen E.
A1 - Fields,Sarah K.
A1 - Comstock,R.Dawn
Y1 - 2007/07//
KW - SportDiscus
KW - *SPORTS injuries
KW - *HIGH school athletes
KW - *WOUNDS & injuries
KW - *ANKLE -- Wounds & injuries
KW - *PHYSICAL fitness -- Research
KW - *BASKETBALL for women
KW - *BASKETBALL injuries
KW - *FOOTBALL injuries
KW - Research
KW - PHYSIOLOGICAL aspects
KW - injury epidemiology
KW - injury surveillance
KW - lower extremity injuries
RP - NOT IN FILE
SP - 381
EP - 387
JA - Journal of Athletic Training
VL - 42
IS - 3
N2 - Context: Ankle injuries are the most common sport-related injuries. To date, no studies have been published that use national data to present a cross-sport, cross-sex analysis of ankle injuries among US high school athletes. Objective: To investigate the incidence rates of ankle injuries by sex, type of exposure, and sport. Design: Descriptive epidemiologic study. Setting: One hundred US high schools. Patients or Other Participants: United States high school athletes. Main Outcome Measure(s): We reviewed ankle injury data collected over the 2005-2006 school year from a nationally representative sample obtained by High School RIO, an injury surveillance system. Specific sports studied were boys' football, boys' and girls' soccer, girls' volleyball, boys' and girls' basketball, boys' wrestling, boys' baseball, and girls' softball. Results: An estimated 326 396 ankle injuries occurred nationally in 2005-2006, yielding an injury rate of 5.23 ankle injuries per 10 000 athlete-exposures. Ankle injuries occurred at a significantly higher rate during competition (9.35 per 10 000 athlete-exposures) than during practice (3.63) (risk ratio = 2.58; 95% confidence interval = 2.26, 2.94; P < .001). Boys' basketball had the highest rate of ankle injury (7.74 per 10 000 athlete-exposures), followed by girls' basketball (6.93) and boys' football (6.52). In all sports except girls' volleyball, rates of ankle injury were higher in competition than in practice. Overall, most ankle injuries were diagnosed as ligament sprains with incomplete tears (83.4%). Ankle injuries most commonly caused athletes to miss less than 7 days of activity (51.7%), followed by 7 to 21 days of activity loss (33.9%) and more than 22 days of activity loss (10.5%). Conclusions:

Sports that combine jumping in close proximity to other players and swift changes of direction while running are most often associated with ankle injuries. Future research on ankle injuries is needed to drive the development and implementation of more effective preventive interventions. ABSTRACT FROM AUTHOR
SN - 10626050
UR -
https://login.proxy.bib.uottawa.ca/login?url=http://search.ebscohost.com/login.aspx?direct=true&db=s3h&AN=27075304&site=ehost-live
ER -

TY - JOUR
ID - 4700
T1 - Mountain Biking-Related Injuries Treated in Emergency Departments in the United States, 1994-2007
A1 - Nelson,Nicolas G.
A1 - McKenzie,Lara B.
Y1 - 2011/02/01/
KW - SportDiscus
KW - *CYCLING accidents
KW - *BRAIN -- Wounds & injuries
KW - *EPIDEMIOLOGY
KW - *HOSPITAL care
KW - *WOUNDS & injuries
KW - *MEDICAL records
KW - Emergency Medical Services -- United States
KW - AGE distribution (Demography)
KW - Analysis of Variance
KW - COMPUTER software
KW - Confidence Intervals
KW - Research
KW - RESEARCH -- Methodology
KW - product safety
KW - PUBLIC health surveillance
KW - SEX distribution (Demography)
KW - Statistics
KW - data analysis
KW - Retrospective Studies
KW - Severity of Illness Index
KW - Evaluation
KW - United States
KW - emergency department
KW - injury
KW - mountain bike
KW - National Electronic Injury Surveillance System
KW - outdoor
RP - NOT IN FILE
SP - 404
EP - 409
JF - American Journal of Sports Medicine
JA - Am J Sports Med
VL - 39
IS - 2
N2 - Background: Injury research on mountain biking has been mostly limited to examining professional riders and off-road biking. Mountain bikes represent the largest segment of bike sales in the United States. Recreational mountain bike use is popular and understudied.Purpose: To describe the scope, distribution, and trends of mountain bikeƒ_"related injuries treated in US emergency departments.Study Design: Descriptive epidemiologic study.Methods: A retrospective analysis was conducted with data from the National Electronic Injury Surveillance System of the US Consumer Product Safety Commission for patients aged ƒ% • 8 years from 1994 through 2007. Sample weights provided by the system were used to calculate national estimates of

mountain bikeƒ_"related injuries based on 4624 cases. Bivariate comparisons between categorical variables were assessed with injury proportion ratios and 95% confidence intervals.Results: Nationwide, an estimated 217 433 patients were treated for mountain bikeƒ_"related injuries in US emergency departments from 1994 to 2007, an average of 15 531 injuries per year. The annual number of injuries decreased 56%, from a high of 23 177 in 1995 to 10 267 in 2007 (P < .001). The most common injuries were upper extremity fractures (10.6%) and shoulder fractures (8.3%). Patients aged 14 to 19 years sustained a greater proportion of traumatic brain injuries (8.4%) than did patients aged 8 to 13 years and ƒ% • 20 years combined (4.3%). A greater proportion of female riders (6.1%) than male riders (4.5%) were hospitalized.Conclusion: Mountain bikeƒ_"related injuries decreased from 1994 to 2007. Upper extremity fractures were the most common injury. Girls and women may be more likely than boys and men to sustain more severe injuries requiring hospitalization. Despite the decline over the past decade, more can be done to improve safety and reduce injuries in this popular recreational activity. ABSTRACT FROM AUTHOR
SN - 03635465
UR -
https://login.proxy.bib.uottawa.ca/login?url=http://search.ebscohost.com/login.aspx?direct=true&db=s3h&AN=58766298&site=ehost-live
ER -

TY - JOUR
ID - 4701
T1 - Play it safe
A1 - Neporent,Liz
Y1 - 1995/04//
KW - SportDiscus
KW - *EXERCISE -- Equipment & supplies
KW - *OUTDOOR recreation -- Safety measures
KW - SAFETY measures
RP - NOT IN FILE
SP - 62
JA - Shape
VL - 14
IS - 8
N2 - Offers advice on preventing exercise-equipment-related injuries and accidents. Statistics on exercise-equipment-related accidents in the United States in 1993; Reading instruction manuals of exercise machines; Keeping children away from potentially dangerous equipment; Investigating the safety record of health clubs and gyms; Safety precautions to take when exercising outdoors
SN - 07445121
UR -
https://login.proxy.bib.uottawa.ca/login?url=http://search.ebscohost.com/login.aspx?direct=true&db=s3h&AN=9503235104&site=ehost-live
ER -

TY - JOUR
ID - 4705
T1 - Ausserschulische Sportliche Betaetigung Von Kindern Und Schuelern. / Sports Activities of Children and Pupils Outside of School
A1 - Niedermann,E.
Y1 - 1980///
KW - SportDiscus
KW - *PHYSICAL education & training
KW - *LEISURE
KW - *SPORTS
KW - *PARTICIPATION
KW - teenagers
KW - ATTITUDE (Psychology)
KW - Family
KW - Child

KW - EXTRACURRICULAR
RP - NOT IN FILE
SP - 171
EP - 175
JA - Leibesubungen - Leibeserziehung
VL - 34
IS - 8
N2 - The preparation of children and adolescents for physical activities during leisure time is considered to be one of the main goals of physical education in schools. Collects data through interviews with students (n=1058) and parents (n=281). Data are listed in statistical summaries but not analyzed. Collected data concerning leisure activities of children and adolescents, membership in sport clubs, physical activities of parents, and students' evaluation of physical education in school
UR -
https://login.proxy.bib.uottawa.ca/login?url=http://search.ebscohost.com/login.aspx?direct=true&db=s3h&AN=SPH332753&site=ehost-live;http://articles.sirc.ca/search.cfm?id=332753
L2 - http://articles.sirc.ca/search.cfm?id=332753
ER -

TY - JOUR
ID - 4706
T1 - Physical activity at recess; who is doing what? (Abstract)
A1 - Nielsen,A.B.
A1 - Wakinson,J.
A1 - Causgrove-Dunn,J.
A1 - Cavaliere,N.
Y1 - 2003/03//
KW - SportDiscus
KW - *PHYSICAL fitness
KW - *CARDIOVASCULAR fitness
KW - *STRESS (Physiology)
KW - *RECESSES
KW - Schools
KW - age
KW - EXAMPLE
KW - Comparative Studies
KW - Elementary Schools
KW - Child
KW - SEX FACTOR
KW - behaviour
RP - NOT IN FILE
SP - A
EP - 52
JF - Research Quarterly for Exercise & Sport
JA - Res Q Exerc Sport
VL - 74
IS - 1 Suppl
CY - ;
SN - 02701367
UR -
https://login.proxy.bib.uottawa.ca/login?url=http://search.ebscohost.com/login.aspx?direct=true&db=s3h&AN=SPHS-883418&site=ehost-live;http://articles.sirc.ca/search.cfm?id=S-883418;http://www.aahperd.org/
L2 - http://articles.sirc.ca/search.cfm?id=S-883418
ER -

TY - JOUR
ID - 4710
T1 - Chapter 19: CHILDREN AND YOUTH

A1 - Nieman,David C.
Y1 - 1998/01//
KW - SportDiscus
KW - *CHILDREN -- Health
KW - *TEENAGERS -- Health
KW - *PHYSICAL fitness
KW - *HEALTH promotion
KW - CENTERS for Disease Control & Prevention (U.S.)
KW - United States
RP - NOT IN FILE
SP - 265
EP - 276
JA - Exercise-Health Connection
N2 - Chapter 19 of the book "Exercise-Health Connection" is presented. It discusses concerns regarding the physical fitness and activity issues related to children and youth in the U.S. Enumerated are the guidelines released by the Centers for Disease Control and Prevention (CDC) in 199 urging schools to help young people to get active and stay active such as providing recess activities such as jumping rope and promoting physical activity through a comprehensive school health program with links to the community
SN - 9780880115841
UR -
https://login.proxy.bib.uottawa.ca/login?url=http://search.ebscohost.com/login.aspx?direct=true&db=s3h&AN=27975590&site=ehost-live
ER -

TY - JOUR
ID - 4711
T1 - The parental evaluative tendency for the operational model of private camps in the Greek region
A1 - Nikitaras,N.
A1 - Dallas,G.
A1 - Patsantaras,N.
A1 - Diafas,V.
A1 - Diamanti,V.
Y1 - 2009/07//
KW - SportDiscus
KW - *PHYSICAL education
KW - VACATION schools
KW - SCHOOL camps
KW - Educational Programs
KW - Greece
KW - Diet
KW - Socialization
RP - NOT IN FILE
SP - 13
EP - 20
JA - International Journal of Fitness
VL - 5
IS - 2
N2 - Aim of this study is the investigation of significance of the functional parameters that Greek Parents take into consideration for the qualitative' camps evaluation in our country. The sample constituted 9026 parents which had sent their children in various camps in Southern Greece, during one of the three camping periods, and who have filled in a questionnaire of closed questions considering functional subjects of the camp, i.e. facilities, level of education, specialization of executives (group leaders, etc), program of activities, etc. Results showed that the basic camp's evaluation factors are in order of evaluation, executives, program of activities of the camps, and are followed by, infrastructures - facilities while in smaller percentage diet and the natural environment of camps are evaluated. Moreover according to parents' opinions on the way of compensation that should be differentiated depending on the level of services given. The majority of the respondents would like their children to be involved mainly with sport environmental activities, while with regard to executives' level of

education priority is given in the graduates or students of higher education institutions and more concrete in professors or students of Physical Education. Parents prefer the energetic entertainment and involvement (active participation in the activities) of their children, which is realised in an optional and not in an obligatory base. This fact dictates the need for capable executives to correspond in the necessary process of encouragement and incentive of the campers. ABSTRACT FROM AUTHOR
SN - 09732152
UR -
https://login.proxy.bib.uottawa.ca/login?url=http://search.ebscohost.com/login.aspx?direct=true&db=s3h&AN=44137709&site=ehost-live
ER -

TY - JOUR
ID - 4712
T1 - Design of safe consumer product based on children behaviour model constructed from behaviour observation: case study of playground equipment
A1 - Nishida,Y.
A1 - Motomura,Y.
A1 - Kitamura,K.
A1 - Yamanaka,T.
Y1 - 2010/09/02/
KW - SportDiscus
RP - NOT IN FILE
SP - A219
EP - A220
JF - Injury Prevention
JA - Inj Prev
VL - 16
N2 - This study presents behaviour-model-based product design as a new approach to safe product design for children. Ubiquitous sensing technology and statistical modelling technology allows us to quantitatively observe and record children behaviour and thus acquire children behaviour model from the large-scale sensory data. This study proposes a system for modelling children behaviour and designing consumer product based on the developed behaviour model. The proposed system consists of a wearable sensor for spatially and temporally measuring children behaviour in an everyday setting together with Bayesian network modelling technology to acquire a children behaviour model. We consider this system from both the theoretical and practical viewpoints. The theoretical framework describes a behavioural model in terms of spatial statistics. The practical aspect of this paper is concerned with a case study in which the proposed system is used to create a new type of playground equipment that is safer for children, in order to demonstrate the practical effectiveness of the system. In this case study, we conducted in situ measurement of 47 children playing with equipment by a wireless wearable location ƒ_" electromyography sensor that the authors developed in cooperation with a kindergarten. A model on children's climbing behaviour was created from the measured data. By cooperating with a playground equipment maker, a new climbing wall type of equipment was developed and the developed equipment was installed at a kindergarten. This study also evaluates the performance of the model statistically by observing children playing with the developed equipment using cameras for over 1 year. ABSTRACT FROM PUBLISHER
SN - 13538047
UR -
https://login.proxy.bib.uottawa.ca/login?url=http://search.ebscohost.com/login.aspx?direct=true&db=s3h&AN=66043114&site=ehost-live
ER -

TY - JOUR
ID - 4713
T1 - The Influence of Context: A Naturalistic Study of Ugandan Children's Doings in Outdoor Spaces
A1 - Njelesani,Janet
A1 - Sedgwick,Amy
A1 - Davis,Jane
A1 - Polatajko,Helene

Y1 - 2011/09//
N1 - Accession Number: 2011247094. Language: English. Entry Date: 20110916. Revision Date: 20120907. Publication Type: journal article; research; tables/charts. Journal Subset: Allied Health; Europe; Peer Reviewed; UK & Ireland. Special Interest: Occupational Therapy; Pediatric Care. No. of Refs: 31 ref. NLM UID: 9433361
KW - CINAHL
KW - Occupation (Human) -- Evaluation -- In Infancy and Childhood
KW - Occupation (Human) -- Evaluation -- Uganda
KW - Pediatric Occupational Therapy
KW - Human
KW - Naturalistic Inquiry
KW - Cross Sectional Studies
KW - thematic analysis
KW - Constant Comparative Method
KW - Uganda
KW - Purposive Sample
KW - Male
KW - Female
KW - Child,Preschool
KW - Child
KW - Field Notes
KW - Observational Methods
KW - Play and Playthings
KW - Work
RP - NOT IN FILE
SP - 124
EP - 132
JF - Occupational Therapy International
VL - 18
IS - 3
N2 - The purpose of this study was to explore children's everyday outdoor occupations in context. A naturalistic observational approach was employed to record the observed outdoor occupations of children in Mbarara, Uganda. Thematic analysis, through pattern formation and constant comparative analysis, was used to uncover categories and themes in the data. Patterns emerged from the data revealing two overarching categories containing three themes: 1) types of occupations: play, work and nothing in particular and 2) characteristics of occupational engagement: being in peer groups, having fun and using materials in the environment. The themes reveal that children's occupations are both similar and different across minority and majority world cultures and that children's occupations are contextually situated and flexible in nature. A limitation of the study was that the observational approach made identification of children's ages subjective. Subsequent studies should explore children's experiences of occupational engagement using interviews and focus groups. Copyright Ac 2010 John Wiley & Sons, Ltd
SN - 0966-7903
AD - Graduate Department of Rehabilitation Science, Faculty of Medicine, University of Toronto, Canada
Department of Occupational Science and Occupational Therapy, Faculty of Medicine, University of Toronto, Canada
Graduate Department of Rehabilitation Science, Faculty of Medicine, University of Toronto, Canada
UR - http://search.ebscohost.com/login.aspx?direct=true&db=cin20&AN=2011247094&site=ehost-live
ER -

TY - JOUR
ID - 4714
T1 - The effect of school uniform on incidental physical activity among 10-year-old children
A1 - Norrish,Hannah
A1 - Farringdon,Fiona
A1 - Bulsara,Max
A1 - Hands,Beth
Y1 - 2012/04//
KW - SportDiscus

KW - *SPORTS uniforms
KW - *PHYSICAL activity
KW - *PEDOMETERS
KW - *SCHOOL recess breaks
KW - SCHOOL uniforms
KW - SCHOOL children's clothing
KW - CROSSOVER trials
KW - CHILDREN
KW - gender difference
KW - incidental breaks
KW - pedometer
KW - Physical Activity
KW - school uniform
RP - NOT IN FILE
SP - 51
EP - 63
JA - Asia-Pacific Journal of Health, Sport & Physical Education
VL - 3
IS - 1
N2 - The school setting provides a unique opportunity to promote physical activity in children by ensuring adequate time, appropriate facilities and education guidance is offered. However school uniform design could also limit physical activity. A repeated measures crossover design was used to compare school recess and lunchtime physical activity over four weeks in 64 primary school children (M = 10.48 yrs) when wearing winter uniform or sports uniform. Pedometers recorded step counts during each school recess and lunch break. Perception of the level of intensity of physical activity was also measured using a self-report log book. Mixed model analyses found that girls, but not boys, were significantly more active at recess (p = .03), lunch (p = .04) and overall (p = .006) when wearing their sports uniform compared to their winter uniform. School uniform did not impact the boy's physical activity levels. Perceived intensity of physical activity increased slightly among both girls and boys. A physically restrictive school uniform has the potential to inhibit physical activity among primary-school-aged girls. ABSTRACT FROM AUTHOR
SN - 18377122
UR -
https://login.proxy.bib.uottawa.ca/login?url=http://search.ebscohost.com/login.aspx?direct=true&db=s3h&AN=77321953&site=ehost-live
ER -

TY - JOUR
ID - 4715
T1 - Evaluation of activ8: the effectiveness of a joint dietetic and physiotherapy weight management group intervention in children and adolescents
A1 - Norton,D.
A1 - Samani-Radia,D.
A1 - Van Tonder,A.
Y1 - 2011/06//
KW - SportDiscus
KW - *OBESITY
KW - *ANTHROPOMETRY
KW - *BODY composition
KW - *CHILDREN -- Health
KW - *CHILD nutrition
KW - *EXERCISE
KW - *GAMES
KW - *NUTRITION
KW - *TEENAGERS -- Health
KW - *WEIGHT loss
KW - *YOUTH -- Nutrition
KW - *BODY mass index

KW - *TREATMENT effectiveness
KW - *CHILDREN
KW - Treatment
KW - Analysis of Variance
KW - Clinical Trials
KW - COMPUTER software
KW - Confidence Intervals
KW - STUDY & teaching
KW - OUTCOME assessment (Medical care)
KW - data analysis
KW - PRE-tests & post-tests
KW - Great Britain
RP - NOT IN FILE
SP - 297
EP - 297
JF - Journal of Human Nutrition & Dietetics
JA - J Hum Nutr Diet
VL - 24
IS - 3
N2 - With the prevalence of overweight and obesity in the under 18 s on the rise, there are increasing numbers of weight management programmes offering treatment within and outside of the NHS, however only a few are formally evaluated (Aicken et al., 2008). Activ8 is a 6-week community based group intervention targeting overweight and obese children aged between 5 and 18 years. The course consists of weekly 1-h sessions that combine game based physical activities and nutritional education sessions. The aim of this study was to evaluate the effect of the Activ8 intervention on anthropometry and body composition. Routinely collected data was pooled from all children attending Activ8 during 2009. Information recorded included gender, date of birth and measurements for weight, height and body fat percentage (%BF), measured using bioelectrical impedance analysis. Patients with only a single measurement, missing birth date and attending siblings under the 91st percentile for BMI for age were excluded. The prevalence of co-morbidities such as type 2 diabetes and sleep apnoea were not assessed. Statistical analysis was carried out using SPSS comparing anthropometric and body compositional variables before and after attendance and examining the effect of age and gender on outcomes. Ethical approval was received from the Research Ethics Review Panel of the London Metropolitan University prior to the commencement of data collection. In 2009, 15 courses were organised. Of the 133 children starting, 70 completed the course. All but two of the participants were clinically obese at the start of the course (mean BMI centile = 99.69 (0.60). Mean age was 10.62 (2.97) years. Girls represented 52.9% of the sample, whilst 86.7% were from ethnic minority backgrounds. The results indicated that at the end of the intervention average absolute BMI decreased by ƒ^'0.29 kg m (SD = 0.49, P = 0.000, CI = 95%), which remained significant when converted to z-scores and percentiles. Whilst %BF decreased on average by ƒ^'0.79%, this did not reach statistical significance (SD = 2.43, P = 0.080, CI = 95%). Reduction in z-BMI, but not absolute BMI or BMI centile, was significantly greater (P = 0.046) in boys compared with girls. Some differences in outcomes stratified by age were also observed with the younger age groups achieving significantly greater reductions in absolute BMI (P = 0.003), z-BMI (P = 0.000) and BMI centile (P = 0.009). No significant differences were observed according to age group or gender in the reduction of %BF. The results are in line with the findings of other, similar interventions (Oude Luttikhuis et al., 2009). In line with NICE guidelines, the results indicate that Activ8 appears to be successful in achieving a decrease in adiposity as indicated by BMI, at least in the short term. Whether these results are sustained should be examined in a longer-term follow up study. It is recommended that the reasons behind the relatively high drop out and low uptake rates should be investigated through the adoption of a robust evaluation process. This audit of Activ8 has shown that it compares favourably to similar interventions, however further studies are required to evaluate long term efficacy, to eliminate the possible confounding effect of factors such as ethnicity and co morbidities and to strengthen the quality of the evidence by the use of randomisation and a control group. Aicken, C., Arai, L. & Roberts, H. (2008) Schemes to Promote Healthy Weight Amongst Obese and Overweight Children in England. London: Evidence for Policy and Practice Information Centre, Social Science Research Unit, Institute of Education, University of London. Luttikhuis, H.O. et al. (2009) Interventions for Treating Obesity in Children. Cochrane Database Syst. Rev. , 1-57. ABSTRACT FROM AUTHOR
SN - 09523871

UR -
https://login.proxy.bib.uottawa.ca/login?url=http://search.ebscohost.com/login.aspx?direct=true&db=s3h&AN=60457968&site=ehost-live
ER -

TY - JOUR
ID - 4717
T1 - Physical Activity Patterns Measured by Accelerometry in 6- to 10-yr-Old Children
A1 - Nyberg,Gisela A.
A1 - Nordenfelt,Anja M.
A1 - Ekelund,Ulf
A1 - Marcus,Claude
Y1 - 2009/10//
KW - SportDiscus
KW - *SEX differences (Biology)
KW - *EXERCISE -- Research
KW - *CHILDREN
KW - *PHYSICAL activity
KW - *ACTIVITIES of daily living training
KW - *PHYSICAL fitness
KW - *ACCELEROMETERS
KW - Research
KW - data analysis
KW - ACTIWATCH
KW - MOTION SENSORS
KW - PHYSICAL ACTIVITY LEVELS
KW - school
RP - NOT IN FILE
SP - 1842
EP - 1848
JF - Medicine & Science in Sports & Exercise
JA - Med Sci Sports Exerc
VL - 41
IS - 10
N2 - The article focuses on one study which examines the differences in patterns of objectively measured physical activity (PA) when done weekdays and weekend days and whether age and gender play integral roles in the existence of these differences. A number of children were asked to participate in a randomized school-based obesity prevention program or Stockholm Obesity Prevention Project. They were asked to spend a great deal of time outdoors or in a stimulating environment where their PA will be measured using the accelerometer. Collected data were then analyzed using Statistica. After data analysis, it was revealed that it is at the age of six when chlidren's PA starts to decline. It added that PA levels are disproportionally low in both girls and boys during weekends
SN - 01959131
UR -
https://login.proxy.bib.uottawa.ca/login?url=http://search.ebscohost.com/login.aspx?direct=true&db=s3h&AN=44494667&site=ehost-live
ER -

TY - JOUR
ID - 4718
T1 - Facilitators and Barriers to Unstructured Free Play in Rural Communities in Northern Alberta
A1 - Nykiforuk,Candace
A1 - Vallianatos,Helen
Y1 - 2012/09//
KW - SportDiscus
KW - *OBESITY
KW - *PLAY

KW - *CHILDREN
KW - Surveys
KW - Canada
RP - NOT IN FILE
SP - 1
EP - 2
JA - Research Update
VL - 19
IS - 3
N2 - The article offers information on a survey report stating that 2012 Active Healthy Kids Canada Report Card states that children are not spending time in active free play and this could lead to obesity. Many strategies were undertaken to encourage free play. It mentions that although some barriers limit unstructured free play, families in rural communities in Northern Alberta spend lots of time playing both indoors and outdoors. The participants should use the already exiting facilities
SN - 19208502
UR -
https://login.proxy.bib.uottawa.ca/login?url=http://search.ebscohost.com/login.aspx?direct=true&db=s3h&AN=80164015&site=ehost-live
ER -

TY - THES
ID - 4719
T1 - Family experiences with long-term childhood technology dependence: an interpretive interactionist approach
A1 - O'Brien,M.E.
Y1 - 1996///
N1 - Accession Number: 1999070499. Language: English. Entry Date: 19991001. Revision Date: 20091218. Publication Type: doctoral dissertation; research
KW - CINAHL
KW - Child,Medically Fragile
KW - Life Experiences -- Evaluation
KW - Perception -- Evaluation
KW - Family
KW - Long Term Care -- Psychosocial Factors
KW - conceptual framework
KW - Naturalistic Inquiry
KW - descriptive research
KW - Observational Methods
KW - Unstructured Interview
KW - Purposive Sample
KW - Child,Preschool
KW - Child
KW - Human
RP - NOT IN FILE
EP - 358
PB - UNIVERSITY OF TEXAS AT AUSTIN
N2 - Increased numbers of children who are technology-dependent are now being cared for at home by parents and other caregivers. However, there is inadequate understanding of the daily lives of families who care for technology-dependent children at home and how these families manage over time. The purpose of this descriptive, naturalistic study was to explore the meaning of and factors involved in the long-term home care of a child who is technology-dependent from the family's point of view using an interpretive interactionist approach (Denzin, 1989). The sensitizing theoretical framework for the study was Kazak's (1986, 1989, 1992) systems and social-ecological model. The purposive sample consisted of 15 families which included at least one child aged 3-12 years who: (a) had been technology-dependent and living at home for at least one year, and (b) was medically stable at the time of the study. Family experiences with childhood technology dependence were explored using unstructured parental interviews and home observations of family members engaged in usual routines and activities of daily living. Demographic data were also obtained. Four themes emerged from the

interpretive interactionist data analysis: (a) managing daily life with technology, (b) negotiating with outside entities, (c) maintaining a functioning family, and (d) making sense of life. The potential for frequent and unexpected change, unpredictability, and limited parental control inherent in each of these major thematic areas contributed to families' perceptions of the fragility and instability of family life with technology. The contextualization of the phenomenon of family experience with long-term childhood technology dependence revealed that families felt as if they were "living in a house of cards." Major strategies used to increase stability were vigilance, advocacy, and reframing. Based on the results of the study, recommendations for nursing are made
AV - UMI Order PUZ9633249.
M1 - PH.D.
UR - http://search.ebscohost.com/login.aspx?direct=true&db=cin20&AN=1999070499&site=ehost-live
ER -

TY - JOUR
ID - 4721
T1 - One Family's Approach
A1 - O'Malley,Sean
Y1 - 2009/05//
KW - SportDiscus
KW - *CHILDREN
KW - *LIFESTYLES
KW - *WHITEWATER kayaking
KW - *VOYAGES & travels
KW - *PLAY
KW - Learning
RP - NOT IN FILE
SP - 14
EP - 19
JA - American Whitewater
VL - 49
IS - 3
N2 - The article discusses the approach in immersing children in an active lifestyle such as whitewater kayaking. Some of the methods in engaging children in active lifestyle are providing early exposures to the outdoors at a young age, keeping the trips short, and making play as the main goal with learning as the natural side effect. According to the article, natural active lifestyle will raise fearless, bold children
SN - 03007626
UR -
https://login.proxy.bib.uottawa.ca/login?url=http://search.ebscohost.com/login.aspx?direct=true&db=s3h&AN=39553399&site=ehost-live
ER -

TY - JOUR
ID - 4724
T1 - Environmental determinants of physical activity in children: A systematic review
A1 - Oliveira,A.F.
A1 - Moreira,C.
A1 - Abreu,S.
A1 - Mota,J.
A1 - Santos,R.
Y1 - 2014/06//
KW - SportDiscus
KW - *PHYSICAL activity
KW - *PHYSICAL fitness for children
KW - *HEALTH risk assessment
KW - *CHILDREN -- Health
KW - *ENVIRONMENTAL health
KW - SYSTEMATIC reviews (Medical research)

KW - CHILDREN
KW - Environment
KW - Physical Activity
RP - NOT IN FILE
SP - 254
EP - 261
JA - Archives of Exercise in Health & Disease
VL - 4
IS - 2
N2 - The lack of physical activity (PA) in children is an important health risk factor. Previous studies have shown that some environmental features may be associated with PA levels. Objectives: Find and comprehend which environmental features were mostly positively associated with children's physical activity. Methods: Twenty-eight studies were systematically reviewed. This research was used to assess the association between environmental features and physical activity among children (ages 3 to 12) who fulfilled the selection criteria. Results: Results across the various studies showed that facilities and parks/playgrounds are mostly positively associated with children's PA. Sidewalks and bike lanes were positively associated to PA in all studies. Safety, traffic and weather showed inconsistent associations with children's PA. Conclusions: Beginning to understand which environmental features contribute more to PA in children can lead to increased levels of PA. ABSTRACT FROM AUTHOR
SN - 16471652
UR -
https://login.proxy.bib.uottawa.ca/login?url=http://search.ebscohost.com/login.aspx?direct=true&db=s3h&AN=94750345&site=ehost-live
ER -

TY - JOUR
ID - 4725
T1 - Strategies for playground injury prevention: an overview of a playground project
A1 - Olsen,H.
A1 - Hudson,S.D.
A1 - Thompson,D.
Y1 - 2010/05//
N1 - Accession Number: 2010666574. Language: English. Entry Date: 20100730. Revision Date: 20100730. Publication Type: journal article; research; tables/charts. Journal Subset: Blind Peer Reviewed; Editorial Board Reviewed; Expert Peer Reviewed; Health Promotion/Education; Peer Reviewed; USA. Special Interest: Pediatric Care. Grant Information: Iowa State Legislature.. No. of Refs: 29 ref. NLM UID: 101090650
KW - CINAHL
KW - Child Safety -- Standards
KW - Play and Playthings
KW - Risk Management
KW - Schools
KW - Wounds and Injuries -- Prevention and Control
KW - Child
KW - Descriptive Statistics
KW - Evaluation Research
KW - Funding Source
KW - Health Educators
KW - Human
KW - Iowa
KW - Materials Testing
KW - Pretest-Posttest Design
KW - Program Evaluation
KW - random sample
RP - NOT IN FILE
SP - 187
EP - 192
JA - American Journal of Health Education

VL - 41
IS - 3
N2 - Preventing injuries to children, especially debilitating and life threatening, requires an awareness of where these types of injuries occur during the school days. This review examines falls from playground equipment, events that have been identified as the leading causes of nonfatal unintentional injuries for children. Thus, the issue of playground safety is a topic of concern for health educators. School health educators play an essential role in developing safe and healthy outdoor play environments for children. This paper highlights the importance of injury prevention awareness and outlines different strategies that health educators can take for preventing playground injuries. In addition, this paper examines a project that was conducted in the state of Iowa in relation to what effects playground surfacing materials and staff training may have on injury prevention on school playgrounds. The results of the project concluded that with the addition of proper surfacing material and staff training, playground injuries could be reduced. Health educators need to investigate the types of playground injuries in current programs and develop a strategy to keep children healthy and active
SN - 1932-5037
AD - Assistant Professor, School of HPELS, University of Northern Iowa, Cedar Falls, IA
UR - http://search.ebscohost.com/login.aspx?direct=true&db=cin20&AN=2010666574&site=ehost-live
ER -

TY - JOUR
ID - 4731
T1 - Otroska igrisca z vidika zakonodaje. / Children's playgrounds from the legislative perspective
A1 - Pajek,Maja Bucar
A1 - Cuk,Ivan
A1 - Videmsek,Mateja
A1 - Hosta,Milan
Y1 - 2007///
KW - SportDiscus
KW - *PLAYGROUNDS
KW - Legislation
KW - standards
KW - Slovenia
KW - Policy
RP - NOT IN FILE
SP - 11
EP - 16
JA - Sport: Revija Za Teoreticna in Prakticna Vprasanja Sporta
VL - 55
IS - 1
CY - ;
N2 - Modern lifestyles are characterized by excessive sitting. People who are aware of this problem try to engage in various recreational activities in their leisure time so as to diminish the negative impact of their sedentary lifestyle. All well-informed parents want their children to spend quality leisure time. This is where the first problem occurs: older children are usually well taken care of since sports activities in urban centres are relatively readily available. The youngest children are not so fortunate: sport activities are scarce, playgrounds are uncared for, the green areas of parks are in a state of disrepair, and similar. Hence, our research focused on the Slovenian legislation governing children's playgrounds. [ABSTRACT FROM AUTHOR]
SN - 03537455
UR -
https://login.proxy.bib.uottawa.ca/login?url=http://search.ebscohost.com/login.aspx?direct=true&db=s3h&AN=SPHS-1062815&site=ehost-live;http://articles.sirc.ca/search.cfm?id=S-1062815;http://www.fsp.uni-lj.si/rsport/
L2 - http://articles.sirc.ca/search.cfm?id=S-1062815
ER -

TY - JOUR
ID - 4733
T1 - Beyond the Gym: Increasing Outside of School Physical Activity through Physical Education
A1 - Palmer,Stephen

A1 - Bycura,Dierdra
Y1 - 2014/01//
KW - SportDiscus
KW - *HEALTH promotion
KW - *SELF-evaluation
KW - *ABILITY
KW - *CHILDREN -- Health
KW - *CYCLING
KW - *ECOLOGY
KW - *HEALTH behavior
KW - *PERCEPTION
KW - *PHYSICAL education
KW - *SAFETY
KW - *TEENAGERS -- Health
KW - *PHYSICAL activity
KW - Methodology
KW - Teaching Methods
KW - PARENTS -- Attitudes
RP - NOT IN FILE
SP - 28
EP - 35
JA - JOPERD: The Journal of Physical Education, Recreation & Dance
VL - 85
IS - 1
N2 - The purpose of physical education is to guide youngsters to become and remain physically active for life. Research on correlates and determinants of physical activity has shown the importance of developing intrinsic motivation in students so that they will choose to be physically active in their leisure time. When the physical education curriculum is considered a physical activity intervention, learning outcomes, instructional tasks, and activities then target known correlates of physical activity. This article presents teaching strategies and curriculum outcomes that specifically target six correlates of physical activity among children and adolescents. By directing efforts toward known correlates of physical activity during physical education, teachers can help youth become more physically active outside of school. ABSTRACT FROM PUBLISHER
SN - 07303084
UR -
https://login.proxy.bib.uottawa.ca/login?url=http://search.ebscohost.com/login.aspx?direct=true&db=s3h&AN=93350830&site=ehost-live
ER -

TY - JOUR
ID - 4734
T1 - School time physical activity of students with and without autism spectrum disorders during PE and recess
A1 - Pan,C.
Y1 - 2008/10//
N1 - Accession Number: 2010058786. Language: English. Entry Date: 20090206. Revision Date: 20091218. Publication Type: journal article; research; tables/charts. Journal Subset: Allied Health; Peer Reviewed; USA. Special Interest: Pediatric Care; Physical Therapy; Psychiatry/Psychology. No. of Refs: 40 ref. NLM UID: 8701671. Email: chpan@nknucc.nknu.edu.tw
KW - CINAHL
KW - Autistic Disorder -- In Infancy and Childhood
KW - Learning Environment -- In Infancy and Childhood
KW - Physical Activity -- In Infancy and Childhood
KW - Physical Education and Training -- In Infancy and Childhood
KW - Play and Playthings -- In Infancy and Childhood
KW - Accelerometry -- In Infancy and Childhood
KW - Child
KW - Child,Disabled
KW - Comparative Studies

KW - Confidence Intervals
KW - convenience sample
KW - Data Analysis Software
KW - Descriptive Statistics
KW - effect size
KW - Intraclass Correlation Coefficient
KW - Power Analysis
KW - T-Tests
KW - Taiwan
KW - Time Factors -- In Infancy and Childhood
KW - Two-Way Analysis of Variance
KW - Human
RP - NOT IN FILE
SP - 308
EP - 321
JF - Adapted Physical Activity Quarterly
JA - Adapt Phys Act Q
VL - 25
IS - 4
N2 - This study compared moderate-to-vigorous physical activity (MVPA) of students with autism spectrum disorders (ASD) and students without disabilities during inclusive physical education and recess. Students (7-12 years) wore a uniaxial accelerometer in school for 5 consecutive school days. Results indicated a significant difference between settings, F(1,46) = 15.94, p < .01, partial eta2 = 0.26, observed power = 0.97. Students with and without ASD spent a higher proportion of time in MVPA during physical education than during recess, relative to the amount of time spent in those settings. In addition, structured physical education offers opportunities to increase students' MVPA engagement
SN - 0736-5829
AD - Department of Physical Education, National Kaohsiung Normal University, Taiwan
UR - http://search.ebscohost.com/login.aspx?direct=true&db=cin20&AN=2010058786&site=ehost-live
ER -

TY - JOUR
ID - 4735
T1 - Efekty lekcji wychowania fizycznego w terenie i w sali. / Effects of outdoor and indoor physical education lessons
A1 - Panczyk,W.
Y1 - 1998///
KW - SportDiscus
KW - *OUTDOOR life
KW - *PHYSICAL education & training
KW - *HEART beat
KW - Elementary Schools
KW - PROGRAMS
KW - teenagers
KW - Calibration
KW - Comparative Studies
KW - Indoor
KW - Child
KW - COOPER'S 12 MINUTE RUN TEST
RP - NOT IN FILE
SP - 59
EP - 67
JA - Wychowanie Fizyczne i Sport
VL - 42
IS - 4
CY - ;

N2 - The aim of the work was to compare effects of outdoor and indoor physical education lessons with the main focus on the health aspects. Indoor activities are usually lower in intensity because of the less space available. Also, physical exercises performed mostly in a gym lead to certain limitation of children's skills to adaptation to external (environmental and climatic) conditions. That is why outdoor lessons seem to be more effective. During one- year experiment, conducted on a school-children sample (n = 227, aged 10-14 years), indoor and outdoor lessons were compared, using heart-rate (HR) monitors , movement counters and personal opinions of the participants about the intensity of the workout. Also out-of-school physical activity (leisure time), endurance level and number of classes skipped due to health problems were compared. Outdoor lessons proved to be better in every compared aspect. The HR during outdoor lessons averaged around 159,1 beats/min., and only 142,1 during indoor activities. Over 50 % of the outdoor class time was conducted in high HR (HR160), while only 26,4 % duration of the indoor lessons demanded that intensity. All these results were supported by the high percentage of the HR-max used and personal opinion of the participants about the intensity of the workout, and also by almost 20 % greater number of movements during outdoor lessons. Percentage representation of the endurance improvement in outdoor groups was 11 % and in indoor ones only 1 %. During the period of this experiment, amount of leisure time devoted to extracurricular recreation activities in outdoor groups increased almost twice as much as before the experimentation, and number of absences decreased considerably
SN - 00439630
UR -
https://login.proxy.bib.uottawa.ca/login?url=http://search.ebscohost.com/login.aspx?direct=true&db=s3h&AN=SPHS-778486&site=ehost-live;http://articles.sirc.ca/search.cfm?id=S-778486
L2 - http://articles.sirc.ca/search.cfm?id=S-778486
ER -

TY - JOUR
ID - 4736
T1 - Time for a Play Day
A1 - Parker,David B.
Y1 - 2004/12//
KW - SportDiscus
KW - *PLAY
KW - *CHILD development
KW - *PARKS
KW - *PLAYGROUNDS
KW - PLAY environments
KW - SAFETY measures
KW - United States
RP - NOT IN FILE
SP - 60
EP - 63
JA - Parks & Recreation
VL - 39
IS - 12
N2 - Discusses reasons behind the need for children to play. Definition of play; Importance of play environment to the health and wellness development of children; Factors that led to the transformation of school yards and parks according to the article "The Dulling of American Playgrounds"; Background on the certification program of the National Playground Safety Institute
SN - 00312215
UR -
https://login.proxy.bib.uottawa.ca/login?url=http://search.ebscohost.com/login.aspx?direct=true&db=s3h&AN=15414683&site=ehost-live
ER -

TY - JOUR
ID - 4738
T1 - Observing Children's Playground Activity Levels at 13 Illawarra Primary Schools Using CAST2
A1 - Parrish,Anne Maree

A1 - Iverson,Don
A1 - Russell,Ken
A1 - Yeatman,Heather
Y1 - 2009/09/02/
KW - SportDiscus
KW - *PHYSICAL fitness -- Research
KW - *PHYSICAL fitness for children
KW - *CHILDREN -- Health
KW - *PLAYGROUNDS
KW - *OBESITY in children
KW - *OBESITY
KW - Research
KW - Risk Factors
KW - PUBLIC health surveillance
KW - Australia
KW - children's activity
KW - observational instrument
KW - Physical Activity
KW - scanning tool
KW - Socioeconomic Status
RP - NOT IN FILE
SP - S89
EP - S96
JF - Journal of Physical Activity & Health
JA - J Phys Act Health
VL - 6
N2 - Background: Declining levels of children's physical activity may contribute to Australia's increasing childhood obesity epidemic. School recess is an underutilized opportunity to increase children's physical activity. Methods: Thirteen regional Australian public primary schools participated in the study (2946 children). The Children's Activity Scanning Tool 2 (CAST2) collected observational playground physical activity data. The research also addressed: length of break, socioeconomic status (SES), gender, number of scanning days, and instrument calibration. Results: The proportions of Moderate or Vigorous Physically Activity (MVPA) children at the observed schools ranged from 0.4 to 0.7. The odds ratio of boys being MVPA relative to girls ranged from 0.8581 to 2.137. There were significant differences between the mean proportions of 3 days of activity (range P = .001 to P = .015) and no association between SES school groupings (deviance ratio: 0.48; P = .503). Interrater reliability for instrument calibration using Spearman correlations coefficients ranged from r = .71 to r = .99. Conclusions: There were significant differences between proportions of MVPA children at the 13 schools and between male and female populations. There was no association between playground physical activity and SES. The monitoring period for CAST2 should be at least 3 days. Interrater reliability indicates that correlations between observers were consistently high. ABSTRACT FROM AUTHOR
SN - 15433080
UR -
https://login.proxy.bib.uottawa.ca/login?url=http://search.ebscohost.com/login.aspx?direct=true&db=s3h&AN=48049908&site=ehost-live
ER -

TY - JOUR
ID - 4740
T1 - Psychosocial barriers to playground activity levels
A1 - Parrish,Anne Maree
A1 - Iverson,Don
A1 - Russell,Ken
A1 - Yeatman,Heather
Y1 - 2012/04//
N1 - Accession Number: 2012132824. Language: English. Entry Date: 20130614. Revision Date: 20130614. Publication Type: journal article; pictorial; research; tables/charts. Journal Subset: Europe; Nursing; Peer

Reviewed; UK & Ireland. Special Interest: Pediatric Care. Instrumentation: Children's Activity Scanning Tool (CAST2). NLM UID: 101297722
KW - CINAHL
KW - Physical Activity -- In Infancy and Childhood
KW - Play and Playthings -- In Infancy and Childhood
KW - Students,Elementary
KW - Child Behavior
KW - Self Report
KW - Social Learning Theory
KW - Surveys
KW - School Administrators
KW - Teachers
KW - Schools,Elementary
KW - New South Wales
KW - Bullying
KW - Uniforms
KW - Weather
KW - Wounds and Injuries
KW - Questionnaires
KW - Shyness
KW - Self-Efficacy
KW - Descriptive Statistics
KW - Summated Rating Scaling
KW - Scales
KW - Data Analysis Software
KW - Student Attitudes
KW - Faculty Attitudes
KW - Observational Methods
KW - Male
KW - Female
KW - Job Experience
KW - Child
KW - T-Tests
KW - Spearman's Rank Correlation Coefficient
KW - Task Performance and Analysis
KW - Mann-Whitney U Test
KW - Comparative Studies
KW - Learning Environment
KW - Games
KW - Time Factors
KW - Human
RP - NOT IN FILE
SP - 131
EP - 137
JA - British Journal of School Nursing
VL - 7
IS - 3
SN - 1752-2803
AD - School of Health Sciences, University of Wollongong, NSW, Australia
Faculty of Health and Behavioural Sciences, University of Wollongong, NSW, Australia
School of Computing and Maths, Charles Sturt University, Australia
UR - http://search.ebscohost.com/login.aspx?direct=true&db=cin20&AN=2012132824&site=ehost-live
ER -

TY - JOUR
ID - 4741
T1 - The Effect of School Recess Interventions on Physical Activity

A1 - Parrish,Anne Maree
A1 - Okely,Anthony
A1 - Stanley,Rebecca
A1 - Ridgers,Nicola
Y1 - 2013/04//
KW - SportDiscus
KW - *CHILDREN -- Health
KW - *HEALTH promotion
KW - *MEDICAL care
KW - *MEDLINE
KW - *PLAY
KW - *RECREATION
KW - *SPORTS
KW - *TEENAGERS -- Health
KW - *EVIDENCE-based medicine
KW - *TREATMENT effectiveness
KW - *PHYSICAL activity
KW - RESEARCH methodology evaluation
KW - Experimental Design
KW - INFORMATION storage & retrieval systems
KW - ONLINE information services
KW - OUTCOME assessment (Medical care)
KW - RESEARCH -- Finance
KW - SCALES (Weighing instruments)
KW - School Environment
KW - SYSTEMATIC reviews (Medical research)
KW - Descriptive Statistics
KW - Evaluation
KW - Australia
RP - NOT IN FILE
SP - 287
EP - 299
JF - Sports Medicine
JA - Sports Med
VL - 43
IS - 4
N2 - Background: The benefits of physical activity to maintain optimal health and well-being in children and adolescents are undisputed. The school environment offers opportunities for children to be physically active. Objective: The aim of this review is to systematically examine the effects of recess-based interventions on the physical activity (PA) levels of school-aged children and adolescents. Data Sources: A systematic literature search was conducted to identify papers reporting interventions to promote PA during school recess and/or lunchtime periods. The search was conducted in six databases (PubMed, SPORTDiscusƒ,›, Web of Science, Proquest, Cochrane and Scopus) for papers published between January 2000 and April 2011. Study Selection: Articles were included in the review if (i) they reported the findings of an intervention targeting PA levels of children and/or adolescents during school recess and/or lunchtime; (ii) have a measure of PA as an outcome variable; (iii) participants were aged between 5 and 18 years; and (iv) were published in English. Methods: Two authors independently searched the literature using the same search strategies to identify papers reporting interventions that promote PA during school recess and lunchtime periods. Methodological quality was assessed using an adapted eight item assessment scale. The effects of the interventions were assessed with a rating system used in a recent review of interventions in youth. Results: The search originally retrieved 2,265 articles. Nine published peer-reviewed journal articles met the inclusion criteria for this review. Eight studies used randomized controlled trials and one was a controlled trial. Three studies demonstrated high methodological quality (33%). None of the studies adequately reported the randomization procedure or used power calculations. Few studies reported potential confounders and three studies had less than a 6 week follow-up. Five studies demonstrated a positive intervention effect on children's PA levels, with four reporting statistically significant increases and two reporting significant decreases in recess PA. The summary of the levels of evidence for intervention effects found inconclusive results for all intervention types, though promising strategies that require

further investigation were identified. Limitations: Whilst every effort was made to ensure that this review was as encompassing as possible, it may be limited by its search terms especially if there were studies with unclear titles or abstracts. In addition, only manuscripts published in English were considered, eliminating any possible studies published in other languages. Conclusions: All of the studies used an objective measure to assess PA outcomes, although several criteria were consistently absent from the studies. The levels of evidence were not sufficient to establish conclusive intervention effects on children's recess PA. This could be due to the small number of published studies. There is a need for higher-quality intervention research to strengthen published findings to inform recess PA interventions. Intervention research is needed in adolescents due to the absence of school recess intervention research in this population. ABSTRACT FROM AUTHOR
SN - 01121642
UR -
https://login.proxy.bib.uottawa.ca/login?url=http://search.ebscohost.com/login.aspx?direct=true&db=s3h&AN=86303776&site=ehost-live
ER -

TY - JOUR
ID - 4743
T1 - Physical Activity in Preschool Children With the Transition to Outdoors
A1 - Pate,Russell R.
A1 - Dowda,Marsha
A1 - Brown,William H.
A1 - Mitchell,Jonathan
A1 - Addy,Cheryl
Y1 - 2013/02//
KW - SportDiscus
KW - *OUTDOOR recreation for children
KW - *PHYSICAL activity
KW - *EXERCISE for children
KW - Research
KW - PRESCHOOL children -- Research
KW - Child Care
KW - early childhood
KW - young children
RP - NOT IN FILE
SP - 170
EP - 175
JF - Journal of Physical Activity & Health
JA - J Phys Act Health
VL - 10
IS - 2
N2 - Background: It is known that children are more physically active outdoors than indoors. However, few previous studies have observed the time course for physical activity as young children transition from indoor to outdoor activities. Methods: Participants were 3- to 5-year-old children enrolled in the Children's Activity and Movement in Preschool Study (CHAMPS). Trained observers used the Observational System for Recording Physical Activity in Children-Preschool Version (OSRAC-P) to record children's physical activity levels over 20 minutes in outdoor settings. The 20-minute outdoor observational period began immediately following the transition from indoors to outdoors. Results: Children's activity levels were moderately high at the time of transition and declined over the 20-minute observation period. Different patterns, however, were observed for boys and girls. Overall, boys were more active than girls. Boys' activity levels declined in a linear fashion over the 20-minute period, while girls' activity levels increased slightly, decreased, and then increased slightly again. Conclusions: These data indicate that physical activity levels decline with increased duration of outdoor play. The frequency and duration of outdoor play should be investigated for the purpose of optimizing physical activity levels.. ABSTRACT FROM AUTHOR
SN - 15433080
UR -
https://login.proxy.bib.uottawa.ca/login?url=http://search.ebscohost.com/login.aspx?direct=true&db=s3h&AN=85351311&site=ehost-live

ER -

TY - JOUR
ID - 4744
T1 - ATTITUDES ABOUT WOMEN EXECUTIVES IN GOVERNMENT POSITIONS
A1 - Patrick,Catharine
Y1 - 1944/02//
KW - SportDiscus
KW - *WOMEN
KW - *CHARITIES
KW - FEMININE identity
KW - WOMEN executives
KW - HOUSEWIVES
KW - PUBLIC welfare
RP - NOT IN FILE
SP - 3
EP - 34
JA - Journal of Social Psychology
VL - 19
IS - 1
N2 - A certain stereotyped picture of the feminine office-holder is shared alike by the housewives, the male executives, and even the women executives, which many times conflicts with what they know of themselves and their own personal experience. According to this general opinion, the feminine -executive most often attains her office through influential friends. She goes into politics, because she wants to earn a living and likes that sort of career. She has many firm convictions of good government and advocates equality of laws. The woman office-holder is conscientious about her work. She may admit her mistakes and the truth of opponents' arguments, and is able to see the humor in unpleasant details. She has about the same number of outside interests as men. The female executive is often termed conservative, and is not considered to have much general interest in politics that do not concern her personally. She places more emphasis on matters of social welfare than on those of financial and international importance. Her efficiency is affected by worry over social relations. The care of the home is a big handicap to her profession and, conversely, the profession interferes greatly with the home and marriage. The woman executive has difficulty deciding on committees and methods of procedure for meetings; and in persuading others to accept her point of view. She makes judgments in government matters quickly and occasionally wishes to change decisions after making them. She is not easily approached by employees, although she is apt to criticize them for errors. Inconsistency in statements and actions and the tendency to sit back and let the men lead the discussions are often characteristics. She is jealous of her fellow workers and they of her. She acts from intuition and permits feelings of irritation and depression to affect her work. She gives excessive attention to details, which annoy her when they go wrong. She is inhibited in her work by fear of criticism and is too much influenced by what other people say. In comparison with this attitude, it is interesting to note the actual characteristics of the woman executive, as determined from the self-ratings and facts about the lives of 120 of them. She is well educated and has had 10 years or more experience in holding executive offices. Her activities may have been along party lines and her career may have been hindered by prejudice against women, although not necessarily so. She has received the cooperation and support of feminine organizations both before and after obtaining office. The chief factors contributing to her success were excessive work and the ability to speak in public, rather than such factors as influential friends. She is motivated to seek office by the wish to carry out the ideals of good government and the desire for that sort of career. She is liberal, with many firm convictions of good government and much interest in general politics. She may or may not advocate equal laws for men and women. She puts as much emphasis on financial and international matters as on those of social welfare. She has probably been married sometime in her life and is frequently married at the present time. If she is married, the years of matrimony and number of children in the family average about the same as those of the non-professional woman of the same age and educational background. These facts, as well as her own assertions, indicate that her profession has not interfered with her marriage or management of her home. Nor has the care of the home or worry over social matters interfered with her profession as re-elections indicate. Of the present married women legislators, 46 per cent have been re-elected, which is about the same proportion as that of the total group of feminine legislators. The feminine executive's husband does not support her, although he may or may not contribute to some extent. She is apt to maintain her children and also other relatives, either partly or entirely. During the year she has missed less than a week's work on account of illness. In regard to her

executive ability, she does not have difficulty deciding on committees and plans, persuading others, or enforcing orders on men below her in rank. She makes judgments in government matters quickly. She mav or mav not be ambitious and is apt to give excessive attention to details. While the woman executive occasionally may wish to change decisions, she is on the whole consistent in statements and actions. She is approachable to her employees and does not criticize them. She may or may not sit back and let the men carry on discussions at meetings. The feminine office-holder does not experience jealousy or feel it towards her fellow workers. She is not inhibited in her profession by fear of criticism, and does not let feelings of irritation or depression affect her efficiency. She is not apt to be frequently annoyed by details which go wrong. The woman executive is somewhat influenced by others' viewpoints, but does not have difficulty resisting majority opinion in committee meetings. She does not often act from intuition and frequently sees humor in unpleasant details. She is conscientious about her work, and admits her own mistakes and the truth of opponents' arguments. She has about the same number of outside interests as the male office-holder. A marked discrepancy exists between the stereotyped opinion of the woman executive (which is even held by women office-holders themselves) and the personal characteristics manifested by this group. The percentages of re-election, which are similar to those of men executives, are evidence of the efficiency of the woman office-holder. The 1937 survey of the National League of Women Voters (5) showed that 44 per cent of the feminine legislators were serving re-election terms. Similarly, 39 per cent of the feminine legislators of the present study have previously held the same office. Of several states representing different sections of the country, the present Legislative Directory of Vermont (8) shows that 27 per cent of the feminine members of the House of Representatives have held seats there before; 50 per cent of those in Maryland (4) in 1939 have done the same. The record for Nebraska (7) over a period of 74 years from 1866-1940 shows that 33 per cent of the women members of the legislature in that period have been re-elected. These figures are similar to those for the men; 33 per cent of the male legislators of the present study are serving re-election terms. For the state of Vermont, the Legislative Directory (8) indicates that 49 per cent of the present male members of the Senate and 31 per cent of the House of Representatives have held the same office previously. In Maryland (4), 28 per cent of the male members of the Senate and 27 per cent of the House of Representatives were serving re-election terms in 1939. The record of Nebraska (7) over a period of 74 years shows that 28 per cent of the male members of the legislature served more than one term. The record for both men and women is higher in the present U. S. Congress (1). All of the present feminine members have held office previously. Two-thirds of the male members of the Senate and 85 per cent of the House have held office formerly. In both the state legislatures and the U. S. Congress, the percentages of re-election are similar for men and women. As large a proportion of the women are re-elected as the men, even though the former have to face prejudices which the latter do not. If re-election can be taken as an adequate criterion of efficiency, then we may conclude that, on the whole, women office-holders are as efficient as the men. The disparity between stereotyped opinions of the lack of efficiency of the woman executive and her actual ability become apparent. Such attitudes are partly developed by men to maintain the present system of employment, from which they benefit, and by the home-makers, because they profit from that economic system. The strength of these opinions is indicated by the fact that the women executives continue to hold similar ideas, although they are contrary to their own personal experience. Such opinions have hindered many women from ever obtaining office, but once they have obtained them, they have proved as efficient as the men, as re-election indicates. If an educational program is to be started to remove the discrepancy between the general opinion of the woman executive and her actual qualifications, it should be directed not only toward men and non-professional women, but also toward the women executives themselves. They also reflect the general attitudes about women office-holders, which do not accord with the facts of their own lives. ABSTRACT FROM AUTHOR
SN - 00224545
UR -
https://login.proxy.bib.uottawa.ca/login?url=http://search.ebscohost.com/login.aspx?direct=true&db=s3h&AN=16878956&site=ehost-live
ER -

TY - JOUR
ID - 4745
T1 - URBAN OUTDOOR RECREATION: CHILDREN'S PLAYGROUNDS IN WARSAW
A1 - Pawlikowska-Piechotka,A.N.N.A.
Y1 - 2010/12/15/
KW - SportDiscus
KW - *SPORTS
KW - *PLAYGROUNDS

KW - *SPORTS facilities
KW - PLAY environments
KW - WARSAW (Poland)
KW - Poland
KW - active recreation
KW - children's playgrounds
KW - space for all
RP - NOT IN FILE
SP - 375
EP - 384
JA - Studies in Physical Culture & Tourism
VL - 17
IS - 4
N2 - Over the last century a visible change has taken place in the relationship between children and their living environment in Warsaw. In many Polish cities increased road traffic, pollution and crime, fragmentation of urban fabric, and reduction of public green areas have resulted in the creation of a 'children-unfriendly' environment. The present paper is based on a research project carried out in the Institute of Tourism and Recreation of the JA3zef Pi • ,sudski University of Physical Education in Warsaw between 2008 and 2010 regarding children's playgrounds in Warsaw. The project consisted of case studies on specific themes such as playground facilities, safety, pressures for change, popularity of play sites, and proposed renewal strategies. The authors studied diverse preferences of children and their caregivers as well as their expectations of outdoor recreation infrastructure and management improvements. The emphasis was also placed on disabled children and constraints on the use of playgrounds by these groups. A good playground means safety, fun, delight and excitement. However, there are still many problems connected with small, local playgrounds with poor facilities, situated mostly on communal or co-operative housing estates. Built from cheap and flimsy materials, many playgrounds are not safe for children as they often do not meet the most fundamental safety requirements. Moreover, in any playground design it is important to realize that children's needs are likely to change over time, but due to rather tough budgets of communal or co-operative housing, refurbishments may be too expensive. There is no doubt that the openness of outdoor recreation areas, following the 'space for all' principle, with unlimited public use in mind (for the disabled and the able bodied alike), is a crucial condition of an integrated healthy environment. ABSTRACT FROM AUTHOR
SN - 08671079
UR -
https://login.proxy.bib.uottawa.ca/login?url=http://search.ebscohost.com/login.aspx?direct=true&db=s3h&AN=57738300&site=ehost-live
ER -

TY - JOUR
ID - 4746
T1 - Practical solutions concerning an intervention study in nursing -- as an example on research of children's postoperative pain relief [Finnish]
A1 - PA lkki,T.
A1 - PietilA ,A.
A1 - VehvilA inen-Julkunen,K.
A1 - Kiviluoma,K.
Y1 - 2004/07//
N1 - Accession Number: 2005036841. Language: Finnish. Entry Date: 20050218. Publication Type: journal article; tables/charts. Journal Subset: Continental Europe; Double Blind Peer Reviewed; Editorial Board Reviewed; Europe; Nursing; Peer Reviewed. Special Interest: Evidence-Based Practice. No. of Refs: 44 ref. NLM UID: 9104138
KW - CINAHL
KW - Experimental Studies
KW - Nursing Interventions
KW - Nursing Staff,Hospital
KW - pediatric surgery
KW - Postoperative Pain -- Nursing -- In Infancy and Childhood
KW - Research,Nursing

KW - Child
KW - Data Collection
KW - Nursing Outcomes
KW - Nursing Practice,Evidence-Based
KW - validity
RP - NOT IN FILE
SP - 146
EP - 157
JA - Hoitotiede
VL - 16
IS - 4
N2 - The purpose of this article was to describe the phases of planning of an intervention study in nursing. The phases were selecting an appropriate intervention, developing and testing the intervention, planning the study design and educating the nursing staff involved in the study. As an example, a research project concerning children's postoperative pain relief is described
Intervention research was a method for investigating the effectiveness of a nursing action in a natural setting. In the process of planning, it was important to choose and develop an intervention that was effective, innovative, practical enough to be implemented in the patient's care and ethically correct. The study requires an experimental or quasi-experimental study design, which was still rarely used in nursing research. One of the most significant phases in an intervention study was to educate the interveners, as the nursing staffs were responsible for collecting the data in practice
When assessing validity, it was important to ensure that the outcome was produced by the treatment rather than by extraneous factors. The outcome was influenced by the patient, the intervener and characteristics of the setting
Evidence-based nursing requires that intervention studies are taken into more active use in nursing science. This kind of research was time-consuming and requires the researcher to collaborate with representatives of different professions and disciplines
SN - 0786-5686
UR - http://search.ebscohost.com/login.aspx?direct=true&db=cin20&AN=2005036841&site=ehost-live
ER -

TY - JOUR
ID - 4748
T1 - Air pollutants, exercise, and risk of developing asthma in children
A1 - Peden,D.B.
Y1 - 2003/01//
KW - SportDiscus
KW - *DISEASES
KW - *EXERCISE-induced asthma
KW - *DISEASES -- Causes & theories of causation
KW - *OUTDOOR life
KW - *STRESS (Physiology)
KW - *GROUP games
KW - teenagers
KW - AIR -- Pollution
KW - Ozone
KW - Risk
KW - Questionnaires
KW - Child
KW - FOLLOW-UP STUDY
RP - NOT IN FILE
SP - 62
EP - 63
JF - Clinical Journal of Sport Medicine
JA - Clin J Sport Med
VL - 13
IS - 1

CY - ;
N2 - Commentary on Asthma in exercising children exposed to ozone: a cohort study by McConnell, R., Berhane, K., Gilliland, F., London, S.J., Islam, T., Gauderman, W.J., Avol, E., Margolis, H.G., Peters, J.M. The aim of this study is to investigate the relation between the first time diagnosis of asthma and team sports in children exposed to air pollutants. Results indicate that there is a relationship between a new diagnoses of asthma in children and heavy exercise in communities with high concentrations of ozone which may lead to the conclusion that outdoor exercise in an area of air pollution could contribute to the development of asthma in children
SN - 1050642X
UR -
https://login.proxy.bib.uottawa.ca/login?url=http://search.ebscohost.com/login.aspx?direct=true&db=s3h&AN=SPHS-864619&site=ehost-live;http://articles.sirc.ca/search.cfm?id=S-864619
L2 - http://articles.sirc.ca/search.cfm?id=S-864619
ER -

TY - JOUR
ID - 4749
T1 - So What About Recess, Really?
A1 - Pellegrini,A.D.
Y1 - 1989///
KW - SportDiscus
KW - *STRESS (Physiology)
KW - *PLAY
KW - Elementary Schools
KW - Aggression
KW - Child
RP - NOT IN FILE
SP - 354
EP - 356
JF - Play & Culture
VL - 2
IS - 4
N2 - Outlines the extent to which research addresses the issue of whether or not elementary schoolchildren need recess. Examines the anti-recess argument: 1) the current climate in schools stresses academic achievement, and recess is viewed as taking away from academic instructional time, and 2) recess results in aggression and fights, particularly for boys. Presents counterarguments and makes a case for recess. Concludes that children are better and happier students and citizens as a result of recess
SN - 08944253
UR -
https://login.proxy.bib.uottawa.ca/login?url=http://search.ebscohost.com/login.aspx?direct=true&db=s3h&AN=SPH333573&site=ehost-live;http://articles.sirc.ca/search.cfm?id=333573
L2 - http://articles.sirc.ca/search.cfm?id=333573
ER -

TY - JOUR
ID - 4750
T1 - Growth status and physical fitness of primary school children in an urban and a rural community in Oaxaca, southern Mexico
A1 - Pena Reyes,M.E.
Y1 - 2002///
KW - SportDiscus
KW - *CARDIOVASCULAR fitness
KW - Child Development
KW - Schools
KW - Mexico
KW - Growth
KW - Child

KW - primary school
KW - Anthropometry
RP - NOT IN FILE
CY - Eugene, OR;
PB - Microform Publications, University of Oregon
N2 - This study considered the growth status and physical fitness of primary school children in a rural and an urban community in the Valley of Oaxaca, southern Mexico, in 1999-2000. The prevalence of growth stunting, wasting, and overweight and obesity were also considered. The sample included 700 school children 6 to 13 years of age, 361 from the rural community (177 males, 184 females) and 339 from the urban community (173 males, 166 females). Anthropometric dimensions included body size, segment lengths, skeletal breadths, limb circumferences and skinfolds. The body mass index (BMI), leg length, arm and calf muscle circumferences, sum of skinfolds, and a trunk/extremity skinfold ratio were derived. Physical fitness items included motor- and health-related tests: right and left grip strength (muscular strength), 35-yard dash (32.3 meters, speed), standing long jump (explosive power), sit- and-reach (flexibility), timed sit-ups (30 seconds, abdominal strength and endurance), and a distance run (8 minutes in grades 1-3 and 12 minutes in grades 4-6, cardiovascular endurance). Height, weight, sitting height, estimated leg length and skeletal breadths on the trunk were significantly larger in urban than in rural school children. The BMI, sitting height/stature ratio, skeletal breadths on the extremities, limb and estimated limb muscle circumferences, subcutaneous fatness and relative fat distribution did not consistently differ by age group and sex between urban and rural children. Urban children tended to perform better in the sit and reach, sit-ups and standing long jump, but results were not consistently significant. Rural children 6-9 years tended to perform better in the dash. There were no urban-rural differences in grip strength, but strength per unit body mass was greater in rural children. Rural children in grades 1-3 performed better in the distance run, but results in older children were not consistent. The prevalence of stunting was about twice as great in rural than in urban children, but the prevalence of wasting was very low in both communities. The prevalence of overweight was significantly greater in urban than in rural children, but the prevalence of obesity was low. Rural children, boys more than girls, reported more frequent daily participation in household-related activities classified as moderate and moderate-to vigorous in intensity compared to urban children. Urban boys reported more household-related activities of light or very light intensity. Urban and rural girls did not differ in reported frequencies of daily household activities classified of light and very light intensity. Urban children reported more diversity in diet, specifically at breakfast and dinner, than rural children. Animal protein represented in meats and meat derivatives were rather limited in the diets of rural children. The results of the comparisons of growth status, physical fitness, daily activities and diet of urban and rural primary school children reflect, to a large extent, the contrast of living conditions and access to resources in the urban and rural communities
UR -
https://login.proxy.bib.uottawa.ca/login?url=http://search.ebscohost.com/login.aspx?direct=true&db=s3h&AN=SPHS-872043&site=ehost-live;http://kinpubs.uoregon.edu/
ER -

TY - JOUR
ID - 4752
T1 - PADRAO DE ACTIVIDADE FISICA DE ESCOLARES. / PATTERN OF PHYSICAL ACTIVITY OF SCHOLARLY
A1 - Pereira da Silva,Simonete
A1 - Magalhaes,Maria
A1 - Garganta,Rui Manuel
A1 - Teixeira e Seabra,Andre
A1 - Bustamante,Alcibiades
A1 - Maia,Jose Antonio
Y1 - 2006/06//
KW - SportDiscus
KW - *PHYSICAL fitness
KW - *CARDIOVASCULAR fitness
KW - *STRESS (Physiology)
KW - *ACCELEROMETERS
KW - Elementary Schools
KW - Child

RP - NOT IN FILE
SP - 19
EP - 26
JA - Revista brasileira de cineantropometria & desempenho humano (Florianopolis)
VL - 8
IS - 2
CY - ;
N2 - The main purpose of the present study was to assess usual physical activity (PA) patterns of school children. The study was carried out with 49 boys and girls (mean age of 10 years) in 4th grade of two schools in Vila Nova de Gaia/Portugal. Children wore a portable accelerometer device (Tritrac-R3D) on the waist for 5 days. Statistical procedures included descriptives (means, standard deviation, frequency) and independent t-test. The main results were as follows: (1) during the school period, PA level was low, with children not doing the daily minimum of 30 minutes of moderate to vigorous PA; (2) boys showed a higher pattern of moderate to vigorous PA than girls during recess period; (3) children, even in organized physical education (PE) classes, demonstrated a low PA pattern, with less of 50 % of the total PE class time in moderate at vigorous PA; (4) there was no difference in energy expenditure when comparing PE class and school recess. [ABSTRACT FROM AUTHOR]
SN - 14158426
UR - https://login.proxy.bib.uottawa.ca/login?url=http://search.ebscohost.com/login.aspx?direct=true&db=s3h&AN=SPHS-1037620&site=ehost-live;http://articles.sirc.ca/search.cfm?id=S-1037620;http://www.cds.ufsc.br/
L2 - http://articles.sirc.ca/search.cfm?id=S-1037620
ER -

TY - JOUR
ID - 4753
T1 - Children's playground accessibility in the North of Portugal
A1 - Pereira,B.
Y1 - 2004///
KW - SportDiscus
KW - *LEISURE
KW - *PLAY
KW - *PLAYGROUNDS
KW - *PARTICIPATION
KW - Portugal
KW - Child
KW - DELIVERY SYSTEM
RP - NOT IN FILE
SP - 38
EP - 45
JA - World Leisure & Recreation
VL - 46
IS - 1
CY - ;
N2 - With the growing significance of sedentary activities, such as television-watching, playgrounds remain important leisure facilities for children, since physical play activities have an important role in supporting children's motor, social and emotional development. When children have the opportunity to play in a playground they are promoting healthy lifestyles. In Portugal children are required to attend school until the age of 16 years and are provided with education, which is regulated by national government standards, but in regard to leisure opportunities, there is considerable inequality of provision from locality to locality. Seeking to promote the principles of play and health in the open air, a project was developed to discover the pattern of provision of children's playgrounds in different regions of the North of Portugal and to examine their physical characteristics. A questionnaire was developed and sent to Town Halls, seeking information on playground provision in each district and on the characteristics of the equipment provided in the playgrounds. This study includes four sub-regions - Ave, Cavado, Minho-Lima, Alto Tras-os-Montes - that include 38 districts. The north region of Portugal covers 21.194 km2 and is occupied by more than 3.600.000 inhabitants. The region has one of European youngest populations. It was found that almost half of the districts surveyed provided less than

5 playgrounds in their area. Children in general therefore have few opportunities to play in playgrounds because of lack of provision and have very few opportunities to choose the activities they like to engage in because of the lack of attractive equipment. Playgrounds are located mostly in areas without heavy traffic and the majority of playgrounds have minimal equipment - typically four or less items. The paper discusses the implications of these findings for policy-makers in the region
SN - 10261133
UR -
https://login.proxy.bib.uottawa.ca/login?url=http://search.ebscohost.com/login.aspx?direct=true&db=s3h&AN=SPHS-939626&site=ehost-live;http://articles.sirc.ca/search.cfm?id=S-939626;http://www.worldleisure.org
L2 - http://articles.sirc.ca/search.cfm?id=S-939626
ER -

TY - JOUR
ID - 4754
T1 - ELIMINATE PLAYGROUNDS? YOU MUST BE NUTS!
A1 - Peterson,James A.
Y1 - 2002/04//
KW - SportDiscus
KW - *PLAYGROUNDS
KW - *CHILD development
KW - SAFETY measures
KW - United States
RP - NOT IN FILE
SP - 92
JA - Parks & Recreation
VL - 37
IS - 4
N2 - Talks about playground safety for children in the U.S. Benefits of playgrounds to a child's development; Identification of playground risk hazards; Role of the National Playground Safety Institute
SN - 00312215
UR -
https://login.proxy.bib.uottawa.ca/login?url=http://search.ebscohost.com/login.aspx?direct=true&db=s3h&AN=6725122&site=ehost-live
ER -

TY - JOUR
ID - 4756
T1 - The Nature of Caregiver Supervision of Young Children in Public Pools
A1 - Petrass,Lauren A.
A1 - Blitvich,Jennifer D.
Y1 - 2012/02//
KW - SportDiscus
KW - *SWIMMING pools
KW - *DROWNING
KW - CHI-squared test
KW - CHILDREN'S accidents
KW - SUPERVISION
RP - NOT IN FILE
SP - 11
EP - 23
JA - International Journal of Aquatic Research & Education
VL - 6
IS - 1
N2 - This study examined the level of caregiver supervision when children were engaged in active play at public pools. A six-hour unobtrusive observation period was conducted at six different indoor aquatic venues with caregivers accompanying children aged < 10 years eligible to be monitored. Child behavior, corresponding caregiver supervision, and the willingness of parents to intervene when children exhibited unsafe behaviors all

were considered. Environmental factors and pool conditions were also recorded. Chi-square tests illustrated that increased supervision was associated with decreased incidents and lower risk behaviors. Supervision was significantly affected by child and caregiver age, number of children for whom caregivers were responsible, and child swimming ability. The nature of caregiver supervision in aquatic settings requires further investigation to enable the development of effective programs to address poor supervision practices. ABSTRACT FROM AUTHOR
SN - 19329997
UR -
https://login.proxy.bib.uottawa.ca/login?url=http://search.ebscohost.com/login.aspx?direct=true&db=s3h&AN=71702025&site=ehost-live
ER -

TY - JOUR
ID - 4757
T1 - Unobtrusive Observation of Caregiver-Child Pairs at Public Pools and Playgrounds: Implications for Child Unintentional Injury Risk
A1 - Petrass,Lauren A.
A1 - Blitvich,Jennifer D.
Y1 - 2013/08//
KW - SportDiscus
KW - *PLAYGROUNDS
KW - *AQUATIC exercises
KW - UNOBTRUSIVE measures
KW - CAREGIVER-child relationships
KW - SUPERVISION
KW - caregiver supervision
KW - drowning
KW - parent child aquatics
KW - playground
RP - NOT IN FILE
SP - 204
EP - 213
JA - International Journal of Aquatic Research & Education
VL - 7
IS - 3
N2 - This study aimed to determine and compare the nature of supervision children received in two settings that have different levels of environmental risk, an aquatic setting (public pools) and a nonaquatic setting (playgrounds). An observational design was implemented to examine caregiver and child behaviors at six indoor public pools and four playgrounds. Chi-square tests of homogeneity were conducted to determine associations between caregiver and child variables. Preschool children received significantly higher levels of supervision than school-aged children in both settings. For school-aged children the level of supervision varied between settings, with children significantly more likely to be unsupervised in public pools and poorly supervised on playgrounds. Reasons for the lack of supervision in aquatic settings remain unclear, particularly as this setting was considered to present higher environmental risk, because drowning rates are high for young children. Because evidence indicates inadequate supervision is common in aquatic settings, further investigations are required to identify ways to promote closer supervision practices and determine caregiver perceptions regarding their responsibilities when supervising young children in aquatic settings. ABSTRACT FROM AUTHOR
SN - 19329997
UR -
https://login.proxy.bib.uottawa.ca/login?url=http://search.ebscohost.com/login.aspx?direct=true&db=s3h&AN=89639150&site=ehost-live
ER -

TY - THES
ID - 4758
T1 - Exploration of agency in preschool children with disabilities
A1 - Pfeifer,T.A.

Y1 - 1999///
N1 - Accession Number: 2005060060. Language: English. Entry Date: 20050408. Revision Date: 20091218. Publication Type: doctoral dissertation; research
KW - CINAHL
KW - Adaptation,Psychological -- In Infancy and Childhood
KW - Child,Disabled
KW - Child,Preschool
KW - Environment
KW - Ethnographic Research
KW - Observational Methods
KW - secondary analysis
KW - Human
RP - NOT IN FILE
EP - 158
PB - Texas Woman's University
N2 - The purpose of this line of research was to describe how agency is manifested in preschool children with disabilities, and to analyze how manifestations of agency are influenced by natural environmental contexts of home, therapy settings, and peer settings. An additional purpose was to describe an eleven week parent-oriented intervention based on the construct of agency and document changes in parents and children that occurred during the course of this intervention
Three related studies addressed these issues. Three studies were conducted using naturalistic methods of field observation and ethnography. The first study included six children with disabilities and their families. The participants were observed a minimum of five times and no more than nine times in home, therapy settings, and peer settings. The data from Study One were used to analyze children with disabilities in terms of occupational roles and as active participants in different environments. Study Two was a secondary analysis of the data gathered in Study One. The data were examined with a focus on the environmental settings. The researcher analyzed themes of agency or active participation across settings. Additionally, the data were used to examine aspects or qualities of different settings that enhance agency. These qualities may be in the social, cultural, and physical environment that each setting offers. Study Three examined an ongoing parent-oriented treatment setting that utilized the construct of agency and the environment as treatment modalities for children with disabilities. The data analysis revealed aspects of treatment, such as parent education, complexity of play objects, and environmental elements such as peer interaction and classroom routines, that help produce change and lasting adaptation in children with disabilities
The potential significance of these studies is twofold. The first is the addition of basic knowledge to the field of occupational therapy about agency, a theoretical construct. The information gathered from this research will add to the knowledge base of the profession by moving the construct of agency from the philosophical to the practical. The practical knowledge is expected to include ways of observing agency as it is manifested in different roles, activities, and environments of children with disabilities. The second area of potential significance is learning to view therapy with children differently and children with disabilities as action agents. It is hoped that the results of these studies will help practitioners view children with disabilities in ecologically valid domains. The results indicate that children with disabilities have a variety of behaviors that indicate their active involvement with the environment that is not reflected in standard assessments. These may be related to functional activities required in the roles and in the environments in which they participate
SN - 978-0-599-33392-5
AV - UMI Order AAI9932900.
M1 - Ph.D.
UR - http://search.ebscohost.com/login.aspx?direct=true&db=cin20&AN=2005060060&site=ehost-live
ER -

TY - JOUR
ID - 4760
T1 - Appropriation of the environment, motor experiences and sporting activities of girls and women. / Prise de possession de l'environnement, experience motrice et activites sportives des filles et des femmes
A1 - Pfister,G.
Y1 - 1993///
KW - SportDiscus
KW - *STRESS (Physiology)

KW - *CARDIOVASCULAR fitness
KW - *PARTICIPATION
KW - Women
KW - girls
KW - Sociocultural Factors
KW - Environment
KW - SEXE-FEMININ
KW - ACTIVITE-SPORTIVE
KW - MOTRICITE
KW - PRATIQUE-SPORTIVE
KW - SOCIOLOGIE
RP - NOT IN FILE
SP - 159
EP - 173
JA - International Review for the Sociology of Sport
VL - 28
IS - 2/3
CY - ;
N2 - In the western world women, if they are interested in sport at all, are mostly engaged in physical activities which allow an aesthetic presentation and/or a modelling of the body. They are relatively seldom active in types of sport in which the body is employed as an instrument in trails of strength with others or with the environment. At the same time women do not claim much space in many respects - their presence does not fill a room, they do not prowl the streets, they seldom appropriate and use the natural environment for sport. The differences between men and women in sport orientation and the use of physical space begins in childhood. The results of the research can be summed up as follows: 1. The space used by boys was much larger than the space used by girls. 2. Girls are underrepresented on public sporting grounds. 3. Boys and girls differ in the way they use play grounds and other areas appropriated for free movement. 4. Boys' activities tend to contain sporting elements while girls' leisure time activities are more sedentary. The causes for these different play and motor preferences lay in different areas: the family, peer-groups, sporting facilities and so on. Here are the possible sites for change
Les femmes qui vivent en Occident, si elles sont generalement interessees par le sport en general, s'orientent dans la plupart des cas vers des activites sportives qui permettent une presentation esthetique et/ou un modelage du corps. Elles se consacrent relativement peu a des activites sportives dans le cadre desquelles le corps est utilise en tant qu'instrument dans des epreuves de force necessitant un affrontement avec d'autres personnes ou avec l'environnement. En meme temps, les femmes ne revendiquent pas enormement d'espace, et ce, a bien des egards - elles ne remplissent pas les salles, n'envahissent pas les rues, ne s'approprient et n'utilisent que rarement leur environnement naturel pour exercer leur sport. Les differences existant entre les hommes et les femmes, en matiere d'orientation sportive et d'utilisation de l'espace physique s'observent des l'enfance. Les conclusions de l'etude realisee en ce sens peuvent etre resumees de la maniere suivante: 1. L'espace utilise par les garcons est beaucoup plus important que l'espace utilise par les filles. 2. Les filles sont sous-representees sur les terrains de sport publics. 3. On constate de nettes differences dans la maniere dont les garcons et les filles utilisent les terrains de sport et les autres espaces permettant une certaine liberte de mouvement. 4. Les activites pratiquees par les garcons affichent une reelle tendance a comporter des elements sportifs, alors que les activites de loisir preferees des filles affichent un caractere plus sedentaire. Les raisons de ces differences entre les preferences des filles et des garcons en matiere de jeu et de mouvement sont de divers ordres: la famille, le groupe des semblables, l'infrastructure sportive, etc. Ce sont la les domaines possibles d'intervention. (HERACLES)
Differences existant entre les hommes et les femmes en matiere d ' orientation sportive et d ' utilisation de l ' espace physique
SN - 10126902
UR -
https://login.proxy.bib.uottawa.ca/login?url=http://search.ebscohost.com/login.aspx?direct=true&db=s3h&AN=SPH324811&site=ehost-live;http://articles.sirc.ca/search.cfm?id=324811
L2 - http://articles.sirc.ca/search.cfm?id=324811
ER -

TY - JOUR
ID - 4761

T1 - LIFELONG COMPETENCY: MODEL OF MOTOR DEVELOPMENT. / MODEL MOTORIŽONEGA RAZVOJA ZA VSE • «IVLJENJSKO KOMPETENCO
A1 - Pi • ‹ot,Rado
Y1 - 2012/08//
KW - SportDiscus
KW - *MOTOR ability
KW - CONCEPTUAL structures (Information theory)
KW - DEVELOPMENTAL psychobiology
KW - HUMAN life cycle
KW - MATHEMATICAL models
KW - Theory
KW - CHILDREN
KW - Motor Development
KW - motoriŽ_ni razvoj
KW - otroci
RP - NOT IN FILE
SP - 35
EP - 46
JA - Kinesiologia Slovenica
VL - 18
IS - 3
N2 - Changes, slowly but persistently occurring in human development, adjustments that are required during the life and work of the modern man, which nature cannot follow at the pace, and the decrease of basic human functional competences are some of the undamental reasons, due to which profound attention should be dedicated to the sustainability of complete human development. The discussion on sustainable development and ecological awareness should not gnore the efforts to create an environment promoting health maintenance. History shows that human ntervention in the natural environment enabled man, motivated by the hunger for development and progress, which considerably simplified his daily life, however, concomitantly made him, neglect the very thing that throughout the millennia not only preserved, but also enabled him to evolve as a species -- that is physical activity. Human development that reflects in changes in humans that occur over time, are usually presented and described as a complex system of constant, reciprocal exchanges in basic domains: the physical domain (body size, body proportions, brain development, motor development, etc.); the cognitive domain (thought processes and ntellectual abilities including attention, memory, problem solving, creativity, academic and everyday knowledge, etc.) and the social/emotional domain (self-knowledge, self-esteem, expression of emotions, temperament, interpersonal skills, etc.). Motor development is a process that in the individual's life, through different life periods enables the transition to a higher level of motor competences -- the development of motor abilities and acquiring more demanding and upgraded motor skills in constant interaction and correlation with the environment. Among these competencies motor skill is the basis or human motor capital, which in the complete unctioning of humans and in the process of preserving and ensuring health presents the fundamental lever and one of the needed skills that significantly contribute to the individual's quality of life and development through all life periods. Consequently, the period that is devoted today to monitoring and studying motor development of the individual, by considering the role of sustainability and ecological perspective, can be named competence- oriented period, an approach that from the start and continuingly by following the set goals of the problem discussion defines motor competencies, the lifelong competency model of motor development. Among three periods of the presented model; i.e. the acquisition of motor competence, the utilization of motor competences and the decline of motor competences; the period of childhood is of substantial importance in the scope of lifelong functional competences as motor competences. Inadequately acquired fundamental motor patterns negatively affect the process of upgrading motor stereotypes, leading to the lack of motor competences, and consequently also result in inadequate and irregular physical/sports activity in adulthood. On the basis of different research findings, the importance of fundamental motor patterns as the foundation for lifelong functional competences will be presented. ABSTRACT FROM AUTHOR
SN - 13182269
UR - https://login.proxy.bib.uottawa.ca/login?url=http://search.ebscohost.com/login.aspx?direct=true&db=s3h&AN=89167858&site=ehost-live
ER -

TY - JOUR
ID - 4762
T1 - FOLK PLAYS AND GAMES IN SOUTHERN PODLASIE: TRADITION WORTH RECALLING
A1 - Piech,Krzysztof
Y1 - 2003/06//
KW - SportDiscus
KW - *LEISURE
KW - *SPORTS
KW - *GAMES
KW - Sociocultural Factors
KW - Poland
KW - TRADITION
RP - NOT IN FILE
SP - 115
EP - 115
JA - Studies in Physical Culture & Tourism
VL - 10
IS - 1
CY - ;
N2 - Podlasie is a historical region situated along the middle stretch of the Bug River and the upper Narew River in Eastern Poland. Throughout the centuries it underwent various alterations often changing borders and the national status. The former Podlasie was the land of primeval forests, woods and marshlands cut by the rivers Biebrza, Narew, Bug and Krzna. For a long time the immemorial Podlasie was an object of contentions and fights, and that is why over the centuries it went under Polish, Russian and Lithuanian rule respectively. On numerous occasions throughout its existence it was also burnt and devastated by the Tartars, the Swedes, and the Teutonic Knights. Apart from the mentioned incursions the peoples of Podlasie successfully resented the marching of troops during the wars. Podlasie is a historical region situated along the Bug River and the upper Narew River in eastern Poland. The ethnic and religious diversity of southern Podlasie has vastly contributed to the mingling of cultures and the introduction or formation of various customs and traditions in the area. The latter also include plays and games which along with the settlement of different ethnic groups have enriched the lives of inhabitants of the region. Traditional folk plays and games are disappearing in southern Podlasie. Old-time pastimes traditionally passed on from generation to generation have been replaced with modem sports already known in other regions, or by passive forms of leisure such as computer games or television. The plays and games described in the article were collected from habitants of numerous towns and villages of southern Podlasie. They are divided into games played on village paths, playgrounds and in households as well as holiday games and winter games. They all bear witness to the richness of life of children and youngsters in the area in the past. [ABSTRACT FROM AUTHOR]
SN - 08671079
UR - https://login.proxy.bib.uottawa.ca/login?url=http://search.ebscohost.com/login.aspx?direct=true&db=s3h&AN=SPHS-975403&site=ehost-live;http://articles.sirc.ca/search.cfm?id=S-975403
L2 - http://articles.sirc.ca/search.cfm?id=S-975403
ER -

TY - JOUR
ID - 4765
T1 - Impact of early life outdoor experiences on an individual's environmental attitudes
A1 - Place,G.S.
Y1 - 2000///
KW - SportDiscus
KW - *PSYCHOLOGY
KW - *OUTDOOR life
KW - *OUTDOOR education
KW - *OUTDOOR recreation
KW - Environmental Education
KW - ATTITUDE (Psychology)

KW - experience
KW - Child
RP - NOT IN FILE
CY - Eugene, OR;
PB - Microform Publications, University of Oregon
N2 - The problem of the study was to determine the differences in early-life outdoor experiences (individually, with family, environmental education, etc.) between individuals who have adopted an anthropocentric view of the environment and individuals who have adopted a bio-centric or eco-centric view of the environment. To test this difference, three phases of the study were performed. The subjects for the first phase were five historical figures (John Muir, Aldo Leopold, Rachel Carson, Theodore Roosevelt, and Henry David Thoreau) determined by the use of a Delphi study (using various Indiana University faculty) as America's greatest conservationists/environmentalists. The second phase involved ten modern day individuals (four men and six women) who were identified by various Monroe County agencies as being actively involved in the environmental movement. The early life of each historical figure was examined, and the modern day individuals were interviewed to determine which factors (using constant comparison) were consistent in causing them to become active in the conservation movement. Those influential factors, consistent in causing the historical figures and modern day conservationists to become actively involved in the environmental movement, were appreciative early-life outdoor experiences (alone and with friends), reading, and family (influence and vacations). The third phase involved a quantitative survey of 583 (resulting in 537 usable surveys) Indiana University students from a variety of major areas of study. A Discriminant Analysis was performed on the independent (17 variables based on previous research) and dependent variables (individuals were classified as eco-centric or anthropocentric). The results showed that the 17 independent variables explain 7.62% (Rc=276, r2 =.762) of the group variance. In addition, the confusion matrix for classification procedure shows that 75.6% of the respondents were correctly classified as either eco-centric or anthropocentric individuals. Those independent variables that are the most significant in discriminating between eco-centric and anthropocentric individuals include appreciative early-life outdoor experiences (alone and with friends), family early-life outdoor experiences, media (television and reading), and negative experiences. This study provides evidence that encouraging and providing opportunities for interaction with natural environments during an individual's early-life is important in developing environmental attitudes
UR -
https://login.proxy.bib.uottawa.ca/login?url=http://search.ebscohost.com/login.aspx?direct=true&db=s3h&AN=SPHS-812485&site=ehost-live;http://kinpubs.uoregon.edu/
ER -

TY - JOUR
ID - 4766
T1 - Integrated Well-being: Positive Psychology and the Natural World
A1 - Pluta,Aislinn
Y1 - 2012///
KW - Reviewer Nominated
RP - NOT IN FILE
ER -

TY - JOUR
ID - 4768
T1 - The relationship between aerobic and anaerobic exercise capacities in pre-pubertal children
A1 - Prasad,N.
Y1 - 1996///
KW - SportDiscus
KW - *AEROBIC metabolism
KW - *ANAEROBIC metabolism
KW - *CARDIOVASCULAR fitness
KW - Comparative Studies
KW - Energy Metabolism
KW - WINGATE ANAEROBIC TEST
KW - BICYCLE ERGOMETRY
KW - Child

RP - NOT IN FILE
CY - Eugene, Ore.;
PB - Microform Publications, Int'l Inst for Sport & Human Performance, University of Oregon
N2 - It is well established in athletic adult individuals that specialization to an aerobic or anaerobic phenotype can occur. Less is known about this specialization in children. While childhood participation in organized sports is increasing, still very little is known about the physiological potential of young athletes. For example, the development of the aerobic and anaerobic energy systems in relation to each other in children is not well known. Many children are being put on training programs before puberty without any knowledge as to whether or not this can influence specialization to an aerobic or anaerobic phenotype. Early authors basing their results on twin studies, suggested a strong genetic component to aerobic or anaerobic phenotypes (Klissouras et al., 1973). This was not confirmed by muscle biopsy studies in children (Bell et al., 1980) and in fact Bouchard et al. (1992) have proposed a strong environmental or training influence to athletic performance. The purpose of this study was to look at the question of metabolic specialization in pre pubertal children before they had any influence of growth or maturation or training effects. The hypothesis was that if specialization exists, then those children with the better anaerobic capacity would have the lower aerobic capacity and vice versa. An attempt was made to screen out the better sprint and endurance performers with field tests, as it was felt that if specialization was occurring it would most likely be present in this group of individuals. A total of 42 pre-pubertal children from one school completed the study. Mean age was 9.31 years (range 8-11 years). All children initially completed field tests of 50 yard run and 1600 yard run to determine the best sprint and endurance performers. The sprint group (S) performed the 50 yard run under 8.50 seconds, while the endurance group (E) ran 1600 yards under 8 minutes. A questionnaire was filled out to exclude subjects who were involved with a track club or regular training program. On a separate day laboratory tests and anthropometric measures were performed. The laboratory tests performed in random order consisted of a Wingate protocol for anaerobic parameters on one day and a Quinton cycle ergometer for aerobic parameters on another day. Statistical analysis consisted of a zero order correlational analysis for the dependent variables of age, sex, height, weight, sum of skinfolds, sprint run time, endurance run time, anaerobic capacity, peak and mean anaerobic power, and aerobic capacity. In addition a Hotelling's T2 test was performed to determine significant differences between the sprint and endurance groups. The results showed no significant differences between sprint or endurance groups with respect to anthropometric or laboratory measures. There was a trend for the sprint group to have higher peak anaerobic power 9.43 plus/minus 0.87 W/kg (S) versus 8.67 plus/minus 1.25 W/kg (E). However, they also showed a trend towards higher aerobic power 52.03 plus/minus 7.97 ml/kg/min (S) versus 47.73 plus/minus 9.25 ml/kg/min (E). This suggests no specialization. In addition the correlational analysis showed high positive correlations between mean anaerobic power and VO2max (r is 0.88) and peak anaerobic power and VO2max (r is 0.82) suggesting that those children who do best aerobically also do best anaerobically in the pre-pubertal age group. The evidence provided from this study suggests that pre-pubertal children are metabolic non-specialists. Therefore rigorous training programs trying to implement aerobic or anaerobic specialization in this age group are likely not beneficial. The specialization seen in adolescents and adults may be secondary to growth or maturational changes after puberty and this would most likely be the better stage to begin regular training programs in children
UR - https://login.proxy.bib.uottawa.ca/login?url=http://search.ebscohost.com/login.aspx?direct=true&db=s3h&AN=SPH411028&site=ehost-live;http://kinpubs.uoregon.edu/
ER -

TY - JOUR
ID - 4770
T1 - Attitudes of key persons to accessibility problems in playgrounds for children with restricted mobility: a study in a medium-sized municipality in northern Sweden
A1 - Prellwitz,M.
A1 - Tamm,M.
Y1 - 1999/12/31/
N1 - Accession Number: 2000052541. Language: English. Entry Date: 20000801. Revision Date: 20091218. Publication Type: journal article; research; tables/charts. Journal Subset: Allied Health; Continental Europe; Double Blind Peer Reviewed; Europe; Peer Reviewed. Grant Information: "Save the Children", Sweden. No. of Refs: 27 ref. NLM UID: 9502210
KW - CINAHL
KW - Child,Disabled

KW - Attitude to Disability
KW - Architectural Accessibility
KW - Play and Playthings
KW - Funding Source
KW - Environment
KW - Government Agencies
KW - Parents
KW - Semi-Structured Interview
KW - Content Analysis
KW - Attitude Measures
KW - Child
KW - Sweden
KW - Human
RP - NOT IN FILE
SP - 166
EP - 173
JF - Scandinavian Journal of Occupational Therapy
VL - 6
IS - 4
N2 - Playgrounds are an important outdoor environment for children. Yet few playgrounds are designed to be accessible to children with restricted mobility. In this study the child with restricted mobility is defined as one who is unable to move around without the aid of a wheelchair, walking-frame, walking-stick or other walking device. The purpose of the study was to explore the attitudes to accessibility problems in playgrounds among two groups of key persons: "creators" and "users of playgrounds" in a medium-sized municipality in northern Sweden. Eleven key persons (5 "creators of playgrounds" and 6 "users of playgrounds") were interviewed in a semi-structured interview. The interviews were analysed according to content analysis and could be coded under different themes. The results showed that those who created playgrounds had (i) a fragmented organization, (ii) insufficient knowledge of disabilities, (iii) poor economy, and (iv) attitudes as an obstacle. Interviews with the users of the playgrounds were coded under two themes (i) the playground is not for me (i.e. for children with restricted mobility) and (ii) assistance is a precondition for accessibility. The results were discussed in the light of how the inaccessibility of play environments can affect the development of children with restricted mobility, and affect their possibilities of a life on a par with that of other children
SN - 1103-8128
AD - Boden University College of Health Sciences, Boden, Sweden. E-mail: maria.prellwitz@hv.luth.se
UR - http://search.ebscohost.com/login.aspx?direct=true&db=cin20&AN=2000052541&site=ehost-live
ER -

TY - JOUR
ID - 4771
T1 - Sports days
A1 - Price,L.
Y1 - 2002///Spring
KW - SportDiscus
KW - *PHYSICAL education & training
KW - *TEACHING
KW - *OUTDOOR life
KW - spring
KW - summer
KW - primary school
RP - NOT IN FILE
SP - 16
EP - 17
JA - British Journal of Teaching Physical Education
VL - 33
IS - 1
CY - ;

N2 - Examines outdoor sports days as a beneficial physical education activity for primary school children during the months of June and July. Includes sample sports day timetable
SN - 14724375
UR -
https://login.proxy.bib.uottawa.ca/login?url=http://search.ebscohost.com/login.aspx?direct=true&db=s3h&AN=SPHS-819410&site=ehost-live;http://articles.sirc.ca/search.cfm?id=S-819410;http://www.pea.uk.com
L2 - http://articles.sirc.ca/search.cfm?id=S-819410
ER -

TY - JOUR
ID - 4772
T1 - Divisions of household work, routines, and child care occupations in families
A1 - Primeau,L.
Y1 - 2000/04//
N1 - Accession Number: 2000044389. Language: English. Entry Date: 20000701. Revision Date: 20091218. Publication Type: journal article; research. Journal Subset: Allied Health; Australia & New Zealand; Double Blind Peer Reviewed; Peer Reviewed. Instrumentation: Household Work Questionnaire. Grant Information: California Foundation for Occupational Therapy. No. of Refs: 40 ref. NLM UID: 100963710. Email: laprimea@utmb.edu
KW - CINAHL
KW - Home Maintenance
KW - Child Care
KW - Family Role
KW - Paternal Role
KW - Maternal Role
KW - Research Instruments
KW - Human Activities
KW - Gender Identity
KW - Qualitative Studies
KW - Interviews
KW - grounded theory
KW - Questionnaires
KW - participant observation
KW - triangulation
KW - Child,Preschool
KW - Child
KW - Adult
KW - Female
KW - Male
KW - Funding Source
KW - Human
RP - NOT IN FILE
SP - 19
EP - 28
JF - Journal of Occupational Science
VL - 7
IS - 1
N2 - To meet their family's needs, parents must create and maintain daily routines that are sustainable, meaningful, provide opportunities to shape their children's development, and meet the often conflicting and competing needs of individual family members. As the number of women participating in paid work outside of the home continues to increase, parents are faced with the dilemma of how to create and maintain routines that facilitate their accomplishment of the work required to meet their family's needs for both economic support and physical and psychological nurturance of their children. This article tells the stories of three families with traditional and non-traditional divisions of household work in order to demonstrate various methods that they used to divide the work within their family. These stories are followed by a discussion of differences in routines and patterns of engagement in child care occupations found in families with non-traditional and traditional divisions of household work. This article contributes to a growing body of knowledge in occupational science

that captures the complexity of occupation through its description, exploration of its meaning, and portrayal of its storied nature in people's lives
SN - 1442-7591
AD - University of Texas Medical Branch at Galveston, 301 University Blvd, Galveston, Texas 77555-1028
UR - http://search.ebscohost.com/login.aspx?direct=true&db=cin20&AN=2000044389&site=ehost-live
ER -

TY - JOUR
ID - 4773
T1 - An evaluation of the Local Exercise Action Pilots and impact on moderate physical activity
A1 - Pringle,A.
A1 - Gilson,N.
A1 - McKenna,J.
A1 - Cooke,C.
Y1 - 2009/09//
N1 - Accession Number: 2010441251. Language: English. Entry Date: 20091204. Revision Date: 20091218. Publication Type: journal article; research; tables/charts. Journal Subset: Blind Peer Reviewed; Editorial Board Reviewed; Europe; Expert Peer Reviewed; Health Promotion/Education; Peer Reviewed; UK & Ireland. Instrumentation: International Physical Activity Questionnaire (IPAQ) [short version]. Grant Information: Funding received from the Department of Health, Natural England and Sport England. No. of Refs: 18 ref. NLM UID: 0374646. Email: a.pringle@leedsmet.ac.uk
KW - CINAHL
KW - Community Programs
KW - Health Promotion
KW - Physical Activity
KW - adolescence
KW - Adult
KW - Aged
KW - Child
KW - Descriptive Statistics
KW - Diaries
KW - Energy Metabolism
KW - England
KW - Exercise
KW - Female
KW - Funding Source
KW - Male
KW - Middle Age
KW - Motivational Interviewing
KW - Paired T-Tests
KW - Prospective Studies
KW - Questionnaires
KW - Self Report
KW - Human
RP - NOT IN FILE
SP - 179
EP - 185
JF - Health Education Journal
VL - 68
IS - 3
N2 - Objective: Identifying community physical activity interventions that facilitate increases in moderate physical activity (MPA) is important in meeting targets set in government health policy. This study evaluated community interventions that aimed to increase levels of MPA. Intervention themes included exercise referral, classes and groups, peermentoring, motivational interviewing, campaigns and directories, outdoor activity and training physical activity leaders. Design: Longitudinal, pre-intervention and intervention assessments occurred between 2004 and 2006. Setting: Recruitment of 10,433 people from ten sites located in areas of high health need across England. Method: Demographic data were collected by questionnaire, with seven-day, population-

specific self-reports assessing pre-intervention and intervention MPA. Using guidelines for young people and adults, MPA MET-minutes/week were used to assign participants to four MPA categories (sedentary, lightly, moderately and highly active) at pre-intervention and intervention phases. Cross-tabulation established movement between three change categories (reversal, stability and progression). Results: From nine sites, 5324 people engaged in the evaluation, and a sub-sample of 998 provided demographic and 1022 self-report baseline and intervention data (i.e. completers) for a typical week's physical activity participation. Of completers who were sedentary or lightly active at pre-intervention, 58.5 per cent achieved recommended physical activity guidelines. 80.9 per cent who were sedentary at pre-intervention reported as lightly, moderately or highly active at intervention. Conclusions Completers increased overall activity levels, including those sedentary and lightly active at pre-intervention who increased levels to recommended guidelines
SN - 0017-8969
AD - Carnegie Research Institute, Leeds Metropolitan University, Leeds, UK
UR - http://search.ebscohost.com/login.aspx?direct=true&db=cin20&AN=2010441251&site=ehost-live
ER -

TY - JOUR
ID - 4774
T1 - Hammer Park Study
A1 - Prosenc,M.
Y1 - 1971///
KW - SportDiscus
KW - *SPORTS facilities
KW - *LEISURE
KW - *PARKS
KW - *PARTICIPATION
KW - planning
KW - Socioeconomic Factors
KW - Demography
KW - Work
KW - GERMANY (West)
KW - WEEKEND
RP - NOT IN FILE
SP - 117
EP - 120
JA - Society & Leisure
VL - 3
IS - 1
N2 - Contains the results of a poll in a section of Hamburg, German Federal Republic and in a recreation park in the center of the section (N=500). Results were: (1) a recreational park with different equipment for children, young people and grownups has its own leisure function and is not a nature replacement, (2) the positive R between activity in the job and activity during leisure time was confirmed. (3) re the setting up and equipment of leisure parks, the preference scale concerning the wishes of weekend tourism is supported, game and recreation possibilities, quiet places, playgrounds, a restaurant, a cafeteria. (4) Age, sex, marital situation, status, leisure activity and awareness of free time are the variables which strongly influence free time behaviour and park visiting
SN - 07053436
UR -
https://login.proxy.bib.uottawa.ca/login?url=http://search.ebscohost.com/login.aspx?direct=true&db=s3h&AN=SPH332106&site=ehost-live;http://articles.sirc.ca/search.cfm?id=332106
L2 - http://articles.sirc.ca/search.cfm?id=332106
ER -

TY - JOUR
ID - 4775
T1 - Health and well-being naturally: 'contact with nature' in health promotion for targeted individuals, communities and populations
A1 - Pryor,A.FAU

A1 - Townsend,M.FAU
A1 - Maller,C.FAU
A1 - Field,K.
KW - Reviewer Nominated
RP - NOT IN FILE
IS - 1036-1073 (Print)
N2 - ISSUE ADDRESSED: The increase in mental health disorders worldwide makes it important to recognise health promotion interventions that are effective, accessible and affordable. Although natural spaces are coming to be recognised as health-promoting settings for general populations, little is understood about the use of nature-contact in treatment and care for individuals experiencing ill-health. METHODS: This paper provides a summary of key research findings and presents a case study examining the self-reported health and well-being benefits of nature contact for a small clinical sample. The 'Spectrum of Interventions for Mental Health Problems and Mental Disorders' provides a conceptual framework for ordering current and future information relating to nature-based interventions. RESULTS: Evidence demonstrates that separately, physical activity, social connection, and contact with nature enhance human health and well-being. The case example illustrates how 'active', 'social' and 'adventurous' contact with nature may be combined within a treatment intervention to protect and enhance the health of individuals experiencing chronic mental, emotional and physical health difficulties. CONCLUSIONS: 'Contact with nature' constitutes a health promotion strategy with potential application in prevention, early intervention, treatment and care. Recommendations include further research to investigate the benefits of nature contact within existing interventions, and the impacts of 'active' and 'social' nature contact within tailored interventions for targeted individuals and communities
AD - School of Health and Social Development, Deakin University, Victoria. anita.pryor@deakin.edu.au FAU - Pryor, Anita
ER -

TY - CHAP
ID - 4778
T1 - Culture, Environmental Psychology, and Well-Being: An Emergent Theoretical Framework
A1 - Rainisio,Nicola
A1 - Inghilleri,Paolo
Y1 - 2013///
KW - Reviewer Nominated
RP - NOT IN FILE
SP - 103
EP - 116
T2 - Well-Being and Cultures
PB - Springer
SN - 9400746105
ER -

TY - JOUR
ID - 4783
T1 - Activating Places for Physical Activity: When "Honey Go Outside and Play" Isn't Enough
A1 - Randolph,Mikaela J.
A1 - Keith Benjamin,Randall
Y1 - 2014/01/02/
KW - SportDiscus
KW - *HEALTH promotion
KW - *SCHOOL children
KW - *OBESITY in children
KW - *PREVENTION
KW - *WALKING
KW - *CYCLING
KW - SAFETY measures
KW - SERVICES for
KW - Built Environment
RP - NOT IN FILE

SP - S119
EP - S121
JF - American Journal of Health Promotion
JA - Am J Health Promot
N2 - The author discuss the Safe Routes to School National Partnership of the Voices for Healthy Kids initiative that aims to eliminate barriers to walking and biking to school for children by improving community design and safety measures for students as of 2014. Topics include childhood obesity prevention, health promotion initiatives for physical activity, shared use agreements, and built environments
SN - 08901171
UR -
https://login.proxy.bib.uottawa.ca/login?url=http://search.ebscohost.com/login.aspx?direct=true&db=s3h&AN=93593064&site=ehost-live
ER -

TY - JOUR
ID - 4784
T1 - Physical activity, fitness and somatic characteristic of prepubertal girls
A1 - Raudsepp,L.
A1 - Jurimae,T.
Y1 - 1996///
KW - SportDiscus
KW - *CARDIOVASCULAR fitness
KW - *STRESS (Physiology)
KW - *RUNNING
KW - *SOMATOTYPES
KW - *EUROFIT
KW - *PHYSICAL fitness
KW - *SPEED
KW - *JOINTS -- Range of motion
KW - *MOTOR ability
KW - *STATURE
KW - *BODY weight
KW - TESTING
KW - Skinfold Thickness
KW - girls
KW - Calibration
KW - Evaluation
KW - Research
KW - Movement
KW - strength
KW - Adipose Tissue
KW - CORRELATION
KW - VARIANCE
KW - Child
KW - Growth
RP - NOT IN FILE
SP - 55
EP - 60
JA - Biology of Sport
VL - 13
IS - 1
CY - ;
N2 - The purpose of this study was to examine the relationship between physical activity, physical fitness and somatic characteristics in 27 prepubertal girls aged 9 years. Somatic measurements included body height, body mass and 5 skinfolds. Physical activity was recorded using the parental 7-day recall. Physical fitness was evaluated using the EUROFIT test battery. The sum of 5 skinfolds was negatively correlated with endurance shuttle run and bent arm hang. Physical fitness tests - where functional strength, flexibility, speed of limb

movement or agility are required - were not significantly correlated with somatic features and physical activity scores. The results of stepwise multiple correlation revealed that somatic characteristics accounted for 32 per cent of variance, and in combination with moderate-to-vigorous physical activity - for 41 per cent of variance in endurance shuttle run. These findings indicate that in pubertal girls the body mass, body fatness and moderate-to-vigorous physical activity were significantly, negatively correlated with those physical fitness tests where the body mass was moved or projected. In summary, the results of present investigation strongly support the theoretical standpoints of motor development according to which the fitness level of a growing child is influenced by biological and environmental factors. However, the magnitude and nature of associations between fitness, biological variables and environmental factors are not so clear
SN - 0860021X
UR -
https://login.proxy.bib.uottawa.ca/login?url=http://search.ebscohost.com/login.aspx?direct=true&db=s3h&AN=SPH405397&site=ehost-live;http://articles.sirc.ca/search.cfm?id=405397
L2 - http://articles.sirc.ca/search.cfm?id=405397
ER -

TY - JOUR
ID - 4788
T1 - An integrative program in sport sciences and physical activity for gifted and talented children
A1 - Raviv,S.
A1 - Lavyan,Z.
Y1 - 2000///Winter
KW - SportDiscus
KW - *SPORTS sciences
KW - *PHYSICAL education & training
KW - PROGRAMS
KW - Israel
KW - GIFTED CHILD
RP - NOT IN FILE
SP - 48
EP - 53
JA - Journal of the International Council for Health, Physical Education, Recreation, Sport & Dance
VL - 36
IS - 2
CY - ;
N2 - This project has two main aims - (1) expose intellectually gifted and talented students to scientific aspects of physical education and sport to connect theory and practice in physical education activity; and (2) offer an interesting and enjoyable multi-disciplinary enrichment program outside of regular learning hours in a unique domain not included in school curricula to help change student attitudes toward physical education as a subject matter, and part of the daily routine. Participating in the project were 15 boys (N = 7) and girls (N = 8) (CA = 10-13) from eighth grade classes in Israel. The program consisted of 24 double lessons applied in three laboratories of sport and physical education sciences - psychomotor, biomechanics, and physiology laboratories; eight double lessons were held in each of the three laboratories. Every theme or study unit taught in this program included three components - theoretical subject, laboratory experiences, and applications during movement and physical activity. The project was evaluated by three means - a group task to examine academic achievement, a feed back questionnaire, and a post-program gathering of students and parents. From knowledge students demonstrated, as well as from their written evaluations, their positive attitudes towards physical education, their perseverance in studies, and their performances of optimal tasks, it was concluded the project attained its aims
Ce projet avait deux objectifs principaux: (1) exposer les etudiants intellectuellement surdoues et talentueux a l'aspect scientifique de l'education physique et du sport afin de permettre un lien entre la theorie et la pratique au niveau d'activites d'education physique, et (2) offrir un programme extracurriculaire multidisciplinaire et enrichi qui se voulait a la fois interessant et plaisant. Ce programme etait centre sur un domaine unique ne faisant pas partie du programme d'etude afin de permettre a chaque participant de modifier son attitude envers l'education physique autant en tant que matiere academique que d'activite journaliere. Au total 15 eleves de huitieme annee en Israel prirent part a l'etude, 7 garcons et 8 filles, ages entre 10-13. Le programme consistait en 24 lecons doubles, dans trois laboratoires de sciences du sport et d'education physique, appliques aux domaines de la

psychomotricite, la biomecanique et la physiologie. Huit lecons doubles eurent lieu dans chacun de ces laboratoires. Chaque theme, ou unite d'apprentissage, enseigne dans le cadre de ce programme comprenait trois composantes; les sujets theoriques, les experiences de laboratoire et l'application durant l'execution de mouvements et activites physiques. Le projet a ete evalue a partir de trois moyens: un projet de groupe afin d'evaluer le cheminement academique, un questionnaire de feedback et une rencontre post programme avec les parents et les eleves. A partir des connaissances acquises par les eleves, leurs evaluations ecrites, leurs attitudes positives envers l'education physique, leur perseverance dans leurs etudes et leur performance optimale des taches, il semble que les objectifs du programme ont ete rencontres
SN - 10912193
UR -
https://login.proxy.bib.uottawa.ca/login?url=http://search.ebscohost.com/login.aspx?direct=true&db=s3h&AN=SPHS-164573&site=ehost-live;http://articles.sirc.ca/search.cfm?id=S-164573;http://www.aahperd.org
L2 - http://articles.sirc.ca/search.cfm?id=S-164573
ER -

TY - JOUR
ID - 4789
T1 - An Epidemiologic Comparison of High School Sports Injuries Sustained in Practice and Competition
A1 - Rechel,Julie A.
A1 - Yard,Ellen E.
A1 - Comstock,R.Dawn
Y1 - 2008/03//
KW - SportDiscus
KW - *SPORTS
KW - *HIGH school students
KW - *SPORTS injuries
KW - *SCHOOL sports
KW - *WOUNDS & injuries
KW - *ATHLETIC trainers
KW - *ACCIDENTS
KW - SCHOOL year
KW - COMPETITIONS
KW - injury rates
KW - injury surveillance
RP - NOT IN FILE
SP - 197
EP - 204
JA - Journal of Athletic Training
VL - 43
IS - 2
N2 - Context: More than 7 million US high school students play sports. Objective: To compare practice and competition injury rates and patterns in 5 boys' sports (football, soccer, basketball, wrestling, and baseball) and 4 girls' sports (soccer, volleyball, basketball, and softball) during the 2005-2006 school year. Design: Prospective injury surveillance study. Setting: Injury data were collected from 100 nationally representative United States high schools via High School RIO (Reporting Information Online). Patients or Other Participants: Athletes from participating high schools injured while participating in a school-sanctioned practice or competition in one of the above sports. Main Outcome Measure(s): Practice and competition injury rates, body site, diagnosis, and severity. Results: High school athletes participating in these 9 sports at participating schools sustained 4350 injuries during the 2005-2006 school year, which corresponds to an estimated 1 442 533 injuries nationally. The rate of injury per 1000 athlete-exposures was higher in competition (4.63) than in practice (1.69) (rate ratio [RR] = 2.73, 95% confidence interval [Cl] = 2.58, 2.90). Of all sports, football had the highest competition (12.09) and practice (2.54) injury rates per 1000 athlete-exposures. Compared with injuries sustained during practice, higher proportions of competition injuries were head/face/neck injuries (proportion ratio [PR] = 1.61, 95% Cl = 1.34, 1.94), particularly in boys' soccer (PR = 7.74, 95% Cl = 2.53, 23.65) and girls' basketball (PR = 6.03, 95% Cl = 2.39, 15.22). Competition injuries were more likely to be concussions (PR = 2.02, 95% Cl = 1.56, 2.62), especially in boys' soccer (PR = 6.94, 95% Cl = 2.01, 23.95) and girls' basketball (PR = 5.83, 95% Cl = 2.06, 16.49). Higher proportions of competition injuries caused the athlete to miss more

than 3 weeks of play (PR = 1.28, 95% Cl = 1.08, 1.52), particularly in baseball (PR = 3.47, 95% Cl = 1.48, 8.11) and volleyball (PR = 2.88, 95% CI = 1.01, 8.24). Conclusions: Rates and patterns of high school sport injuries differed between practice and competition. Providing athletic trainers with this information is a crucial step in developing the targeted, evidence-based interventions required to effectively reduce injury rates among the millions of high school student-athletes. ABSTRACT FROM AUTHOR
SN - 10626050
UR -
https://login.proxy.bib.uottawa.ca/login?url=http://search.ebscohost.com/login.aspx?direct=true&db=s3h&AN=31719636&site=ehost-live
ER -

TY - JOUR
ID - 4790
T1 - A Methodological Model for Collecting High-Quality Data on Physical Activity in Developing Settings--The Experience of the 1993 Pelotas (Brazil) Birth Cohort Study
A1 - Reichert,Felipe Fossati
A1 - Menezes,Ana Maria Batista
A1 - Wells,Jonathan Charles Kingdom
A1 - Ekelund,Ulf
A1 - Rodrigues,Fabiane Machado
A1 - Hallal,Pedro Curi
Y1 - 2009/05//
KW - SportDiscus
KW - *HEALTH surveys
KW - *PUBLIC health
KW - Surveys
KW - PUBLIC health surveillance
KW - Brazil
KW - Accelerometry
KW - Adolescent
KW - Body Composition
KW - Measurement
KW - MOTION SENSORS
KW - physical activity assessment
RP - NOT IN FILE
SP - 360
EP - 366
JF - Journal of Physical Activity & Health
JA - J Phys Act Health
VL - 6
IS - 3
N2 - Background: Prospective studies on physical activity (PA), diet, and body composition in adolescents are lacking, particularly outside high-income countries. Goals: To describe the methods used to assess these variables in the 1993 Pelotas (Brazil) Birth Cohort and to discuss the fieldwork challenges faced and alternatives to overcome them. Methods: In 2006-07 a subsample of the 1993 Pelotas cohort was revisited. PA was estimated using questionnaires, a combined heart-rate and motion sensor (Acti-Heart), and the ActiGraph GT1M accelerometer. Diet was investigated by questionnaire. Total body water was determined by stable isotopes. Thirty individuals had their total energy expenditure assessed by doubly labeled water. All data were collected at participants' home. Results: The logistics of the fieldwork and the difficulties in undertaking the study and alternatives to overcome them are presented. Preliminary analyses show that 511 individuals were traced (response rate = 90.0%). Compliance of both adolescents and their families for the motion sensors and body-composition measurements was excellent. Conclusions: The authors conclude that it is feasible to carry out high-quality studies on PA in developing countries. They hope the article will be useful to other researchers interested in carrying out similar studies. ABSTRACT FROM AUTHOR
SN - 15433080

UR -
https://login.proxy.bib.uottawa.ca/login?url=http://search.ebscohost.com/login.aspx?direct=true&db=s3h&AN=41893124&site=ehost-live
ER -

TY - JOUR
ID - 4791
T1 - The importance of the International Tennis Federation's junior boys' circuit in the development of professional tennis players
A1 - Reid,Machar
A1 - Crespo,Miguel
A1 - Santilli,Luca
A1 - Miley,Dave
A1 - Dimmock,James
Y1 - 2007/04//
KW - SportDiscus
KW - *CHILD athletes
KW - *TENNIS
KW - *TENNIS instruction
KW - *TENNIS players
KW - *ATHLETES
KW - Competition
KW - juniors
KW - tennis
RP - NOT IN FILE
SP - 667
EP - 672
JF - Journal of Sports Sciences
JA - J Sports Sci
VL - 25
IS - 6
N2 - The relationship between junior boys' tennis success, as measured by a top 20 International Tennis Federation's Junior Circuit (ITFJC) ranking, and subsequent ranking accomplishments in professional men's tennis is discussed. The names, countries, and birthdates of all players to achieve a top 20 ITFJC boys' year-end ranking from 1992 to 1998 were recorded. The progress of these players through the professional ranks was then tracked to the end of 2004. Results indicate that 91% of top 20-ranked boys achieved a professional men's ranking, while a stepwise regression analysis revealed junior ranking (JR) to be a predictor of future, professional ranking (I ý= 0.232, r2 = 0.054, p < 0.05). A regression equation [predicted professional rank = 78.17 + 6.31*(JR)] accounted for a significant amount of variance in professional ranking. For male players, therefore, the achievement of a top 20 junior ranking appears to be a reasonable yardstick for future, professional success. The type of surface upon which junior players develop their games was also shown to influence professional ranking highs, with play on clay courts or a combination of clay and hard courts helping to produce higher, professionally ranked players than hard court play alone (pƒ%□ 0.01). ABSTRACT FROM AUTHOR
SN - 02640414
UR -
https://login.proxy.bib.uottawa.ca/login?url=http://search.ebscohost.com/login.aspx?direct=true&db=s3h&AN=24436489&site=ehost-live
ER -

TY - JOUR
ID - 4792
T1 - The weak shall inherit the gym: dodgeball is one of the few times in life when you get to let out your aggressions, no questions asked
A1 - Reilly,R.
Y1 - 2001/05/14/
KW - SportDiscus

KW - *PLAYGROUNDS
KW - *BALL games
KW - *SAFETY
KW - *DODGE ball
KW - Child
RP - NOT IN FILE
SP - 96
EP - 96
JA - Sports Illustrated
VL - 94
IS - 20
CY - ;
N2 - Schools have decided to ban that childhood playground game of dodgeball because of the implications of hurting weaker students
SN - 0038822X
UR -
https://login.proxy.bib.uottawa.ca/login?url=http://search.ebscohost.com/login.aspx?direct=true&db=s3h&AN=SPHS-794393&site=ehost-live;http://articles.sirc.ca/search.cfm?id=S-794393
L2 - http://articles.sirc.ca/search.cfm?id=S-794393
ER -

TY - JOUR
ID - 4793
T1 - The Fat of the Land
A1 - Reilly,Rick
Y1 - 2003/09/22/
KW - SportDiscus
KW - *OVERWEIGHT children
KW - *OBESITY in children
KW - *CHILDREN
KW - *PHYSICAL education
KW - *SPORTS -- Sociological aspects
KW - *PHYSICAL fitness
KW - *ATHLETICS
KW - United States
KW - SCHOOL administration
KW - School Administrators
KW - JAMES,LeBron,1984-
RP - NOT IN FILE
SP - 84
EP - 84
JA - Sports Illustrated
VL - 99
IS - 11
N2 - Overweight children are increasing in the United States. Is your Little Leaguer so fat his blood type is Chee-tos? Americans have the fattest kids on earth. Over the last 20 years the number of overweight children in this country has doubled. Soon, if that trend continues, one of every three kids will be obese. We only have each other to blame. It's you, Mr. Dad, pumping your bike madly while you let your triple-chinned five-year-old lie in the back of his little vinyl bike caboose. It's you, Mrs. Elementary School P.E. Instructor, letting policy wonks talk you into replacing sports that actually make a kid sweat -- dodgeball, kickball, tag -- with "activities" like competitive cup-stacking. It's you, Mr. School Board Member, cutting gym classes to supposedly focus on "literacy." Or reducing gym to one or two times a week. It's you, Mrs. U.S. Senator, spending hundreds of billions of dollars to check grandmothers for shoe bombs while letting funding for schools shrink to the size of Uday Hussein's heart. According to the Centers for Disease Control, one in three kids born in 2000 will contract type 2 diabetes -- and potentially the heart disease, blindness, asthma, sleep apnea, gall bladder disease and depression that may come with it -- because they are obese. At least there's one person who wants to do something about it, and you won't believe who it is -- LeBron James. The Cleveland Cavaliers rookie is fast-

breaking a campaign, sponsored by Nike, to get kids off the PlayStation and back on the playground. He's visiting 47 schools in seven cities and donating sports equipment, getting courts and school yards resurfaced, and paying for instructors who think kids ought to play something other than Capture the Flab. The average kid spends 5 1/2 hours per day in front of a TV, a video-game monitor or a computer
SN - 0038822X
UR -
https://login.proxy.bib.uottawa.ca/login?url=http://search.ebscohost.com/login.aspx?direct=true&db=s3h&AN=10854706&site=ehost-live
ER -

TY - JOUR
ID - 4794
T1 - ALL DAY ALL THE WAY
A1 - Reiter,Ben
Y1 - 2012/12/24/
KW - SportDiscus
KW - *RUNNING backs (Football)
KW - *ANTERIOR cruciate ligament -- Wounds & injuries
KW - *MEDICAL rehabilitation
KW - *MUSCLE strength
KW - *ATHLETIC ability
KW - *FOOTBALL players
KW - *PSYCHOLOGY
KW - PETERSON,Adrian Lewis,1985-
KW - MINNESOTA Vikings (Football team)
KW - NATIONAL Football League
RP - NOT IN FILE
SP - 42
EP - 47
JA - Sports Illustrated
VL - 117
IS - 25
N2 - The article focuses on football player Adrian Peterson, a running back for the Minnesota Vikings team of the National Football League (NFL). An autograph Peterson signed for a child shortly after suffering a torn anterior cruciate ligament in a game on December 24, 2011 is used to illustrate his determination to recover from the injury. Peterson's rapid recovery from the injury in time to play the entire 2012 season is considered, as are his very high levels of muscle strength and physical fitness. Peterson's performance in the 2012 season, in which he rushed for over 1800 yards in the Vikings' first 14 games, is discussed
SN - 0038822X
UR -
https://login.proxy.bib.uottawa.ca/login?url=http://search.ebscohost.com/login.aspx?direct=true&db=s3h&AN=84620402&site=ehost-live
ER -

TY - JOUR
ID - 4797
T1 - The European Youth Heart Studyƒ_"Cardiovascular Disease Risk Factors in Children: Rationale, Aims, Study Design, and Validation of Methods
A1 - Riddoch,Chris
A1 - Edwards,Dawn
A1 - Page,Angie
A1 - Froberg,Karsten
A1 - Anderssen,Sigmund A.
A1 - Wedderkopp,Niels
A1 - Brage,S.A
A1 - Cooper,Ashley R.
A1 - Sardinha,Luis B.

A1 - Harro,Maarike
A1 - Klasson-HeggebA,,Lena
A1 - van Mechelen,Willem
A1 - Boreham,Colin
A1 - Ekelund,Ulf
A1 - Andersen,Lars Bo
Y1 - 2005/01//
KW - SportDiscus
KW - *CARDIOVASCULAR system -- Diseases
KW - *DISEASES -- Risk factors
KW - *CHILDREN -- Diseases
KW - DIRECT marketing
KW - medical research
KW - Europe
KW - Diet
KW - fitness
KW - Lifestyle
KW - Methods
KW - Physical Activity
KW - Psychosocial Factors
KW - validity
RP - NOT IN FILE
SP - 115
JF - Journal of Physical Activity & Health
JA - J Phys Act Health
VL - 2
IS - 1
N2 - Background: The aim of the European Youth Heart Study (EYHS) is to establish the nature, strength, and interactions between personal, environmental, and lifestyle influences on cardiovascular disease (CVD) risk factors in European children. Methods: The EYHS is an international study measuring CVD risk factors, and their associated influences, in children. Relationships between these independent factors and risk of disease will inform the design of CVD interventions in children. A minimum of 1000 boys and girls ages 9 and 15 y were recruited from four European countriesƒ_"Denmark, Estonia, Norway, and Portugal. Variables measured included physical, biochemical, lifestyle, psychosocial, and sociodemographic data. Results: Of the 5664 children invited to participate, 4169 (74%) accepted. Response rates for most individual tests were moderate to high. All test protocols were well received by the children. Conclusions: EYHS protocols are valid, reliable, acceptable to children, and feasible for use in large, field-based studies. ABSTRACT FROM AUTHOR
SN - 15433080
UR -
https://login.proxy.bib.uottawa.ca/login?url=http://search.ebscohost.com/login.aspx?direct=true&db=s3h&AN=15961252&site=ehost-live
ER -

TY - JOUR
ID - 4799
T1 - 12-MONTH EFFECTS OF A PLAYGROUND INTERVENTION ON CHILDREN'S RECESS PHYSICAL ACTIVITY LEVELS
A1 - Ridgers,N.D.
A1 - Stratton,G.
A1 - Fairclough,S.J.
A1 - Richardson,D.J.
Y1 - 2007/12/02/
KW - SportDiscus
KW - *PHYSICAL fitness for children
KW - ABSTRACTS
RP - NOT IN FILE
SP - 159

EP - 160
JA - Acta Kinesiologiae Universitatis Tartuensis
VL - 12
N2 - An abstract of the article "12-Month Effects of a Playground Intervention on Children's Recess Physical Activity Levels," by N. D. Ridgers, G. Stratton, S. J. Fairclough, and D. J. Richardson is presented
SN - 14069822
UR -
https://login.proxy.bib.uottawa.ca/login?url=http://search.ebscohost.com/login.aspx?direct=true&db=s3h&AN=31674091&site=ehost-live
ER -

TY - JOUR
ID - 4800
T1 - CONTRIBUTION OF RECESS TO HABITUAL PHYSICAL ACTIVITY LEVELS IN BOYS AND GIRLS: THE A-CLASS PROJECT
A1 - Ridgers,N.D.
A1 - Stratton,G.
Y1 - 2007/12/02/
KW - SportDiscus
KW - *MOTOR ability in children
KW - ABSTRACTS
RP - NOT IN FILE
SP - 158
EP - 159
JA - Acta Kinesiologiae Universitatis Tartuensis
VL - 12
N2 - An abstract of the study "Contribution of Recess to Habitual Physical Activity Levels in Boys and Girls: The A-Class Project," by N. D. Ridgers and G. Stratton is presented
SN - 14069822
UR -
https://login.proxy.bib.uottawa.ca/login?url=http://search.ebscohost.com/login.aspx?direct=true&db=s3h&AN=31674090&site=ehost-live
ER -

TY - JOUR
ID - 4807
T1 - Assessing free-living physical activity using accelerometry: Practical issues for researchers and practitioners
A1 - Ridgers,Nicola D.
A1 - Fairclough,Stuart
Y1 - 2011/05//
KW - SportDiscus
KW - *EXERCISE
KW - *DECISION making
KW - *LIFESTYLES
KW - Evaluation
KW - Accelerometry
KW - Great Britain
KW - Accelerometers
KW - Measurement
KW - objective techniques
RP - NOT IN FILE
SP - 205
EP - 213
JA - European Journal of Sport Science
VL - 11
IS - 3

N2 - Physical activity is an integral component of a healthy lifestyle, with relationships documented between physical activity, chronic diseases, and disease risk factors. There is increasing concern that many people are not sufficiently active to benefit their health. Consequently, there is a need to determine the prevalence of physical activity engagement, identify active and inactive segments of the population, and evaluate the effectiveness of interventions. The aim of the present study was to identify and explain a number of methodological and decision-making processes associated with accelerometry, which is the most commonly used objective measure of physical activity in child and adult research. Specifically, this review addresses: (a) pre-data collection decisions, (b) data collection procedures, (c) processing of accelerometer data, and (d) outcome variables in relation to the research questions posed. An appraisal of the literature is provided to help researchers and practitioners begin field-based research, with recommendations offered for best practice. In addition, issues that require further investigation are identified and discussed to inform researchers and practitioners of the surrounding debates. Overall, the review is intended as a starting point for field-based physical activity research using accelerometers and as an introduction to key issues that should be considered and are likely to be encountered at this time. ABSTRACT FROM AUTHOR
SN - 17461391
UR -
https://login.proxy.bib.uottawa.ca/login?url=http://search.ebscohost.com/login.aspx?direct=true&db=s3h&AN=60507909&site=ehost-live
ER -

TY - JOUR
ID - 4810
T1 - Liverpool Sporting Playgrounds Project
A1 - Ridges,N.D.
A1 - Stratton,G.
A1 - Curley,J.
A1 - White,G.
Y1 - 2005/10//
N1 - Accession Number: 2009129278. Language: English. Entry Date: 20060324. Revision Date: 20091218. Publication Type: journal article; research; tables/charts. Journal Subset: Biomedical; Editorial Board Reviewed; Europe; Expert Peer Reviewed; Peer Reviewed; UK & Ireland. Special Interest: Pediatric Care. Grant Information: Sport England and Liverpool Department for Lifelong Learning. No. of Refs: 14 ref. NLM UID: 8805809. Email: N.Ridges@ljmi.ac.uk
KW - CINAHL
KW - Health Promotion
KW - Physical Activity -- In Infancy and Childhood
KW - Play and Playthings -- In Infancy and Childhood
KW - Schools,Elementary
KW - Child
KW - Child,Preschool
KW - England
KW - Evaluation Research
KW - Female
KW - Funding Source
KW - Games
KW - Male
KW - Semi-Structured Interview
KW - Social Behavior
KW - Sports
KW - Student Attitudes
KW - Human
RP - NOT IN FILE
SP - 51
EP - 53
JA - Education & Health
VL - 23
IS - 4

N2 - The purpose of this article is to provide an overview of the initial findings from the project to date and discuss baseline measures of physical activity, gender issues and school approaches to developing health promoting playgrounds
SN - 0265-1602
AD - Research Assistant, REACH (Research into Exercise and Children's Health) Group, Research Institute for Sport and Exercise Sciences, Liverpool John Moores University, Liverpool
UR - http://search.ebscohost.com/login.aspx?direct=true&db=cin20&AN=2009129278&site=ehost-live
ER -

TY - JOUR
ID - 4811
T1 - Preparing for Camp Fairs: Strategies to Help Students
A1 - Riley,Kevin Walsh
A1 - Mitchell,Melanie
Y1 - 2005/08//
KW - SportDiscus
KW - *CAMPS
KW - *STUDENTS
KW - *COLLEGE students
KW - *OUTDOOR recreation
KW - *PERSONALITY
KW - Employment
KW - United States
RP - NOT IN FILE
SP - 128
EP - 134
JA - Schole: A Journal of Leisure Studies & Recreation Education
VL - 20
N2 - The article discusses summer camp employment for college students in the U.S. Summer camp employment provides excellent pre-service opportunities for college students who are majoring in physical education and recreation management. Through these experiences they can develop and enhance their teaching, leadership, and communication skills that will be beneficial in future employment. Many camp directors have expressed that a camp employee should possess certain personality traits in order to be effective. An effective employee is a hard worker, possesses the hard skills related to specific camps, has a good work ethic, is a role model, and has prior related work experience. Another important personality essential for the camp employee is to be child-centered and compassionate of children attending the camp. According to the American Camping Association, there are more than 10,000 day and resident camps in the United States where more than half a million people work as counselors, activity leaders, and program directors
SN - 1937156X
UR -
https://login.proxy.bib.uottawa.ca/login?url=http://search.ebscohost.com/login.aspx?direct=true&db=s3h&AN=19144201&site=ehost-live
ER -

TY - JOUR
ID - 4812
T1 - Forest school -- How creative processes impact on emotional wellbeing
A1 - Riley,Mark
Y1 - 2012/09//
KW - SportDiscus
KW - *CHILDREN
KW - *OUTDOOR education
KW - OUTDOOR learning laboratories
KW - OPEN-air schools
KW - Learning
RP - NOT IN FILE
SP - 16

EP - 19
JA - Horizons (14620677)
IS - 59
N2 - The article examines the activities in a Forest School which is a play-based, child initiate learning taking place over time in which children explore and experiment at their own pace with support of practitioners. It is suggested that whether Forest School or Outdoor Classroom, children need to be provided with a stimulating environment supporting creative play and learning. A Forest School site should reportedly be sufficiently different to other outdoor spaces like a local woodland
SN - 14620677
UR -
https://login.proxy.bib.uottawa.ca/login?url=http://search.ebscohost.com/login.aspx?direct=true&db=s3h&AN=79895265&site=ehost-live
ER -

TY - JOUR
ID - 4813
T1 - Recess Activities of the Week: Are They Beneficial and for Whom?
A1 - Rist,Bethany
Y1 - 2012/03//
KW - SportDiscus
KW - *SCHOOL recess breaks
KW - *PHYSICAL activity
RP - NOT IN FILE
SP - 53
EP - 56
JA - JOPERD: The Journal of Physical Education, Recreation & Dance
VL - 83
IS - 3
N2 - An abstract of the article "Differences in Children's Recess Physical Activity: Recess Activity of the Week Intervention" by M. Stellino, C. Sinclair, J. Partridge, and K. King in the periodical Journal of School Health, which discusses impacts of recess activities of the week (RAWs) on student physical activity (PA), is presented
SN - 07303084
UR -
https://login.proxy.bib.uottawa.ca/login?url=http://search.ebscohost.com/login.aspx?direct=true&db=s3h&AN=73756437&site=ehost-live
ER -

TY - JOUR
ID - 4815
T1 - Effect of Self-Determination Theory-based Strategies for Staging Recreation Encounters on Intrinsic Motivation of Youth Residential Campers
A1 - Roark,Mark F.
A1 - Ellis,Gary D.
Y1 - 2009///Winter
KW - SportDiscus
KW - *YOUTH
KW - *PSYCHOLOGY
KW - *INTRINSIC motivation
KW - *TEENAGERS
KW - *RECREATION
KW - *OUTDOOR recreation
KW - *CAMPING
KW - AUTONOMY in youth
KW - DEVELOPMENTAL psychology -- Research
KW - autonomy support
KW - camp
KW - developmental outcomes

KW - Intrinsic Motivation
KW - Self-Determination Theory
KW - youth
RP - NOT IN FILE
SP - 1
EP - 16
JA - Journal of Park & Recreation Administration
VL - 27
IS - 4
N2 - Parents and caregivers generally believe that the camp experiences they purchase for their children will not only yield transient pleasure and lasting memories of fun encounters, but significant developmental outcomes as well. Camp professionals serving over 11 million youths each year embrace such outcomes and commonly advance mission statements that point to the development of friendship skills, self-confidence, competence, self-reliance, independence, citizenship, and many other developmental outcomes. Empirical evidence of how these outcomes might be facilitated through specific recreation leadership strategies (e.g., activity staging) is lacking. The Self-Determination Theory (SDT) would suggest that youth leaders (e.g., camp counselors, recreation activity specialists) could attend to competence, relatedness, and autonomy in staging experiences for the outcome of intrinsic motivation. Such evidence is essential if immediate conscious experiences of campers are to be optimized and stable outcomes to campers are to be expected. Thus, this study examined the effect of three strategies for staging a recreation encounter on intrinsic motivation of youth attending a residential summer camp. Youth participated in a storytelling activity on three occasions, with each occasion staged differently by leaders. One of the staging strategies included a technique intended to elicit high degrees of autonomy, along with competence. A second strategy was designed to evoke autonomy and relatedness, and a third condition represented a traditional model (i.e., competent tutor) for staging encounters in organized recreation settings. A significant effect was found for the contrast of the combined autonomy conditions (representing autonomy support) vs. the competent tutor condition. This finding is particularly important to camp practitioners. This applied test of Self-Determination Theory-based conditions presents evidence to practitioners that providing recreation encounters that foster the three basic psychological needs of competence, relatedness, and autonomy are more likely to increase intrinsic motivation compared to a traditional method (i.e., competent tutor). Accordingly, staff of youth serving programs might implement strategies derived from Self-Determination Theory in the stream of daily recreation activities to facilitate camper acquisition of intrinsic motivation. For instance, the detailed scripts in this study are a model of how to stage recreation encounters (i.e., storytelling activity) to increase intrinsic motivation of campers. Specifically during the storytelling activity, the autonomy-supportive leaders 1) paused to allow time for responses, 2) provided specific, meaningful feedback to encourage effort, and 3) verbally acknowledged campers' perspectives. ABSTRACT FROM PUBLISHER
SN - 07351968
UR -
https://login.proxy.bib.uottawa.ca/login?url=http://search.ebscohost.com/login.aspx?direct=true&db=s3h&AN=45463292&site=ehost-live
ER -

TY - JOUR
ID - 4816
T1 - Looking Up
A1 - Roberts,Rachel
Y1 - 2007/10//
KW - SportDiscus
KW - *PLAYGROUNDS
KW - *EQUIPMENT & supplies
KW - *RECREATION areas
KW - *YOUTH -- Recreation
KW - SAFETY measures
RP - NOT IN FILE
SP - 46
EP - 51
JA - Parks & Recreation

VL - 42
IS - 10
N2 - The article focuses on how playground designers and manufacturers are taking into account younger children when updating and creating climbing features of playgrounds and recreational areas. Particular focus is given to recreational boulder equipment, with safety features and advice discussed. Veterans Park in Lehigh Acres, Florida is highlighted in the article, with commentary from Colleen Gierut, the recreation supervisor. Comments from playground inspector Dave Parker are included as well
SN - 00312215
UR -
https://login.proxy.bib.uottawa.ca/login?url=http://search.ebscohost.com/login.aspx?direct=true&db=s3h&AN=26993079&site=ehost-live
ER -

TY - JOUR
ID - 4819
T1 - Developmental changes in object control skills as a result of motor intervention in disadvantaged preschoolers
A1 - Robinson,Leah E.
A1 - Goodway,Jacqueline D.
A1 - Dunn,Robin
A1 - Johnson,Erica
A1 - Devins,Lori
Y1 - 2007/07/02/
KW - SportDiscus
KW - *HEALTH
KW - *MOTOR ability in children
KW - *BIOLOGICAL control systems
KW - *LOCOMOTION
KW - *PHYSIOLOGY
KW - *MOTOR ability
KW - POOR children
KW - REGULATION
KW - AFRICAN American children
KW - Research
KW - Socioeconomic Factors
RP - NOT IN FILE
SP - S42
EP - S43
JF - Journal of Sport & Exercise Psychology
JA - J Sport Exerc Psychol
VL - 29
N2 - Children from poor, urban, and disadvantaged environments often demonstrate poor motor skill competence in both object control (OC) and locomotor skills (Goodway & Rudisill, 1996; Hamilton et al., 1999). This study examined the motor skill competence of disadvantaged, African American preschoolers. Further, it investigated the effects of a 9-week OC intervention on OC competence. Two instructional approaches were compared: low autonomy (LA) consisting of direct-instruction and a mastery motivational climate (MMC). Participants were randomly assigned to a LA (n = 36), MMC (n = 38), or comparison (no motor intervention) group (n = 39). The LA and MMC groups participated in 18 30-min OC sessions and the comparison group participated in the same amount of unstructured recess. All children were pre-and posttested on the OC subscale of the TGMD-2 (Ulrich, 2001). Preintervention OC competence was low based on percentile ranks (LA = 23%, MMC = 20%, comparison = 20%). A 3 (Group) x 2 (Sex) ANOVA on pre-OC scores revealed no significant group differences (p = .90) or Group x Sex interaction (p = .60). However, a significant sex main effect was found with boys having greater OC competence than girls, F(1, 107) = 29.34, p < .001. A 3 (Group) x 2 Time x 2 (Sex) ANOVA with repeated measures revealed a significant Group x Time interaction, F(2, 107) = 210.97, p < .001, indicating the OC intervention was effective. Post hoc Tukey HSD analysis reported the LA (p ƒ%□ = < .001) and MMC (p ƒ%□ = < .001) groups had significantly better OC competence than the Comparison group from pre- to postintervention. There was no difference between the LA

and MMC groups (p = .88). A nonsignificant Group x Time x Sex interaction (p = .94) showed that there were no sex differences within groups from pre- to postintervention. Clearly, disadvantaged preschoolers had low OC competence and needed motor intervention. When instruction was provided by either LA or MMC, preschool children improved their OC skills. ABSTRACT FROM AUTHOR
SN - 08952779
UR -
https://login.proxy.bib.uottawa.ca/login?url=http://search.ebscohost.com/login.aspx?direct=true&db=s3h&AN=25216201&site=ehost-live
ER -

TY - JOUR
ID - 4823
T1 - Educational objectives of physical activity
A1 - Rogers,F.R.
Y1 - 1929///
KW - SportDiscus
KW - *PHYSICAL education & training
KW - *GOAL (Psychology)
KW - Schools
KW - Philosophy
RP - NOT IN FILE
CY - New York;
PB - A.S. Barnes
N2 - Outlines the theoretical, social and educational considerations and goals of physical activity for school-age children. States that activity is the real objective of life including organic vigor and vitality. Suggests how to measure and assess these goals in the three areas of physical conditioning, physical fitness, and athletic ability. The implications of these goals are important for every teacher as each is a physical educator. Suggests also the optimum classroom environment, importance of recess, the importance of the diversity of the program, and the increasing differentiation of activities between boys and girls as they mature
UR -
https://login.proxy.bib.uottawa.ca/login?url=http://search.ebscohost.com/login.aspx?direct=true&db=s3h&AN=SPHS-170811&site=ehost-live
ER -

TY - JOUR
ID - 4824
T1 - Happiness and Children's Health: An Investigation of Art, Entertainment, and Recreation
A1 - Rogers,Mary A.M.
A1 - Zaragoza-Lao,Emily
Y1 - 2003/02//
KW - SportDiscus
KW - *CHILDREN -- Health
KW - *AMUSEMENTS
KW - *OUTDOOR recreation for children
KW - *CHILDREN
KW - *HOSPITAL care
KW - HAPPINESS in children
KW - Ohio
RP - NOT IN FILE
SP - 288
EP - 289
JF - American Journal of Public Health
JA - Am J Public Health
VL - 93
IS - 2
N2 - The article discusses a study on the relationship between happiness and children's health in Ohio. Health care providers have recognized the importance of amusement activities for ill or hospitalized children. The study

assesses whether counties with more art, entertainment and recreational activities have healthier children. The results showed that 62% of the children were in excellent health, 23.34% were in very good health, while only 3.81% were in fair or poor health. It was also noted that the population-adjusted dollar volume received for arts, recreation and entertainment increased with higher health status. It was concluded that the communities that offer amusements are more likely to have healthier children
SN - 00900036
UR -
https://login.proxy.bib.uottawa.ca/login?url=http://search.ebscohost.com/login.aspx?direct=true&db=s3h&AN=9036725&site=ehost-live
ER -

TY - JOUR
ID - 4825
T1 - Perceived competence, discrepancy scores, and global self-worth
A1 - Rose,E.
A1 - Larkin,D.
Y1 - 2002/04//
N1 - Accession Number: 2002130826. Language: English. Entry Date: 20021004. Revision Date: 20091218. Publication Type: journal article; research; tables/charts. Journal Subset: Allied Health; Peer Reviewed; USA. Instrumentation: Self-Perception Profile for Children (SPPC) (Harter); Importance Rating Scale (IRS); McCarron Assessment of Neuromuscular Coordination (MAND). No. of Refs: 40 ref. NLM UID: 8701671. Email: e.rose@cowan.edu.au
KW - CINAHL
KW - Self Concept -- In Infancy and Childhood
KW - Motor Skills -- In Infancy and Childhood
KW - Child
KW - Male
KW - Female
KW - Motor Skills -- Classification -- In Infancy and Childhood
KW - Regression
KW - Body Image -- In Infancy and Childhood
KW - Interpersonal Relations -- In Infancy and Childhood
KW - Psychological Tests
KW - Australia
KW - Clinical Assessment Tools
KW - Reliability and Validity
KW - Sports -- In Infancy and Childhood
KW - internal consistency
KW - Test-Retest Reliability
KW - Multivariate Analysis of Variance
KW - Analysis of Variance
KW - Data Analysis Software
KW - Descriptive Statistics
KW - multiple regression
KW - Step-Wise Multiple Regression
KW - Human
RP - NOT IN FILE
SP - 127
EP - 140
JF - Adapted Physical Activity Quarterly
JA - Adapt Phys Act Q
VL - 19
IS - 2
N2 - Athletic ability is highly valued by a majority of children and youth. Comparison processes in the athletic domain occur in physical education and organized sport as well as in the unstructured contexts of play and games. Children with movement difficulties are more likely to face failure or exclusion in sport and on the playground. Yet we know little of the processes underlying their self-esteem. These children struggle in physical

education, and on sports days are likely to experience humiliation before their peers, teachers, and parents. Therefore, it seems likely that the social, psychological, and physical experiences of these children impact on the relative contribution of domain-specific perceptions of competence and importance to global self-worth (GSW). In this study, we employed Harter's Importance Rating Scale (IRS) and Self-Perception Profile for Children (SPPC) to examine importance ratings, discrepancy scores, and domain-specific perceptions of competence as predictors of GSW. Children (N = 130, 62 boys and 68 girls) aged 8-12 years were categorized into high (HMC; n = 62) and low motor coordination (LMC; n = 68) groups according to their scores on a motor proficiency battery. Regression analyses using domain-specific perceptions of competence, importance, and discrepancy scores confirmed that self-perception ratings were the best predictors of GSW. For both groups, perceptions of physical appearance, social acceptance, and behavioral conduct contributed significantly to prediction of GSW. By contrast, perceived athletic competence increased prediction of GSW for the HMC group but not the LMC group
SN - 0736-5829
AD - School of Biomedical and Sports Science, Edith Cowan University, Mount Lawley, 6050 Western Australia
UR - http://search.ebscohost.com/login.aspx?direct=true&db=cin20&AN=2002130826&site=ehost-live
ER -

TY - JOUR
ID - 4826
T1 - Incorporating the Outdoors in Physical Education
A1 - Rose,Tessie
Y1 - 2001/08//
KW - SportDiscus
KW - *PHYSICAL education -- Study & teaching
KW - *OUTDOOR recreation for children
KW - SCHOOL field trips
RP - NOT IN FILE
SP - 17
JA - JOPERD: The Journal of Physical Education, Recreation & Dance
VL - 72
IS - 6
N2 - Suggests the incorporation of outdoor recreation activities in teaching physical education. List of suggested outdoor activities; Safety measures in conducting field trips; Ways to raise funds for outdoor activities; Financial aid given by non-profit organizations and local colleges; Management and scheduling of field trips
SN - 07303084
UR -
https://login.proxy.bib.uottawa.ca/login?url=http://search.ebscohost.com/login.aspx?direct=true&db=s3h&AN=4990974&site=ehost-live
ER -

TY - JOUR
ID - 4827
T1 - After physical education... physical activity outside of school physical education programs
A1 - Ross,J.
A1 - Dotson,C.O.
A1 - Gilbert,G.G.
A1 - Katz,S.J.
Y1 - 1985/01//
KW - SportDiscus
KW - *PHYSICAL fitness
KW - *CARDIOVASCULAR fitness
KW - PROGRAMS
KW - adulthood
RP - NOT IN FILE
SP - 77

EP - 81
JA - JOPERD: The Journal of Physical Education, Recreation & Dance
VL - 56
IS - 1
CY - ;
N2 - Presents the findings from the National Children and Youth Fitness Study which examined the extra curricular physical activity involvement of children in grades 5 through 12. Discusses weekly time spent in physical activity, seasonality in weekly physical activity time, participation in community organizations, and predominant activities. Stresses the importance of monitoring trends and patterns of participation in physical activity sources other than school physical education
SN - 07303084
UR -
https://login.proxy.bib.uottawa.ca/login?url=http://search.ebscohost.com/login.aspx?direct=true&db=s3h&AN=SPH185224&site=ehost-live;http://articles.sirc.ca/search.cfm?id=185224
L2 - http://articles.sirc.ca/search.cfm?id=185224
ER -

TY - JOUR
ID - 4828
T1 - Comparison of Physical Activity Levels Between Children With and Without Autistic Spectrum Disorders
A1 - Rosser Sandt,Dawn D.
A1 - Frey,Georgia C.
Y1 - 2005/04//
KW - SportDiscus
KW - *LEISURE
KW - *STRESS (Physiology)
KW - *PLAY
KW - *PHYSICAL education & training
KW - *RECESSES
KW - *PARTICIPATION
KW - Comparative Studies
KW - Elementary Schools
KW - Autism
KW - Child
KW - SPORTS AND RECREATION ACTIVITIES-Adapted physical education
RP - NOT IN FILE
SP - 146
EP - 159
JF - Adapted Physical Activity Quarterly
JA - Adapt Phys Act Q
VL - 22
IS - 2
CY - ;
N2 - The purpose of this study was to compare daily, physical education, recess, and after school moderate to vigorous physical activity (MVPA) levels between children with and without autistic spectrum disorders (ASD). Children ages 5 to 12 years wore a uniaxial accelerometer for five days (four weekdays, one weekend day). There were no differences between children with and without ASD at any physical activity setting. Both groups were more active during recess compared to after school, and children with ASD were similarly active in recess and physical education. Although many children with ASD acquired 60 min of physical education per day, this may decrease with age as opportunities for recess and physical education are eliminated. [ABSTRACT FROM AUTHOR]
SN - 07365829
UR -
https://login.proxy.bib.uottawa.ca/login?url=http://search.ebscohost.com/login.aspx?direct=true&db=s3h&AN=SPHS-984513&site=ehost-live;http://articles.sirc.ca/search.cfm?id=S-984513;http://www.humankinetics.com/
L2 - http://articles.sirc.ca/search.cfm?id=S-984513
ER -

TY - JOUR
ID - 4832
T1 - Duration, frequency, and types of children's activities: Potential of a classification procedure
A1 - Ruch,Nicole
A1 - Melzer,Katarina
A1 - MA der,Urs
Y1 - 2013/12//
KW - SportDiscus
KW - *ANTHROPOMETRY
KW - *CHILDREN -- Health
KW - *JUMPING
KW - *PHYSICAL education
KW - *PLAY
KW - *RUNNING
KW - *WALKING
KW - *SPORTS participation
KW - *BODY mass index
KW - *PHYSICAL activity
KW - *CHILDREN
KW - Algorithms
KW - Analysis of Variance
KW - Confidence Intervals
KW - Elementary Schools
KW - Factor Analysis
KW - Statistics
KW - Time
KW - data analysis
KW - Accelerometry
KW - REPEATED measures design
KW - DATA analysis -- Software
KW - DIARY (Literary form)
KW - Descriptive Statistics
KW - Switzerland
RP - NOT IN FILE
SP - 85
EP - 94
JA - Journal of Exercise Science & Fitness
VL - 11
IS - 2
N2 - This study investigated the potential of a classification procedure to determine type, duration, and frequency of children's physical activity (PA) during 7 days based on accelerometer data. Hip and wrist accelerometer data (1-second epoch) were collected over a week in 41 children (age: 10.7 Añ0.9 years). The classification procedure was used to assign each second into one of the following four categories: stationary activities, walking, running, and jumping. A diary was used to assess the simultaneous activity setting. Children spent 75.5% of the time (600.9 Añ80.1 minutes/day) on stationary activities, 15.6% (124.6 Añ33.6 minutes/day) on walking, 2% (16.1 Añ8.6 minutes/day) on running, and 1% (4.7 Añ5.2 minutes/day) on jumping. The median duration of stationary activities, walking, running, and jumping was 4, 2, 1, and 1 seconds, respectively. The largest proportion of running and jumping occurred during outdoor sport training (10.7%), physical education classes (6.7%), and vigorous outdoor activity (6.1%). The classification procedure used in this study shows the potential for analyzing children's PA in free- living conditions. The study results revealed that children's PA is characterized by very short activity bouts and that providing the possibility for children to participate in structured or unstructured outdoor activities might increase their PA. Therefore, the classification procedure enhanced the analysis of the transitory nature of children's PA and the understanding of their PA behavior during different activity settings. ABSTRACT FROM AUTHOR
SN - 1728869X

UR -
https://login.proxy.bib.uottawa.ca/login?url=http://search.ebscohost.com/login.aspx?direct=true&db=s3h&AN=94059148&site=ehost-live
ER -

TY - JOUR
ID - 4834
T1 - Garden-Based Learning: An Experience With â€œAt Riskâ€• Secondary Education Students
A1 - Ruiz-Gallardo,Jos+ ªReyes
A1 - Verde,Alonso
A1 - Vald+ ªs,Arturo
Y1 - 2013///
KW - Reviewer Nominated
RP - NOT IN FILE
SP - 252
EP - 270
JF - The Journal of Environmental Education
VL - 44
IS - 4
PB - Taylor & Francis
SN - 0095-8964
ER -

TY - JOUR
ID - 4836
T1 - Dumbest Sports Moments
A1 - Rushin,Steve
Y1 - 2004/09/27/
KW - SportDiscus
KW - *SPORTS -- History
KW - *SPORTS records
KW - *SPORTS journalism
KW - *MASS media & sports
KW - *SPORTSCASTERS
KW - *TELEVISION broadcasting of sports
KW - *ATHLETES
KW - *SPORTS personnel
KW - *COACHES (Athletics)
KW - *ATHLETICS
KW - *SPORTS
KW - *SPORTS teams
RP - NOT IN FILE
SP - 173
EP - 174
JA - Sports Illustrated
VL - 101
IS - 12
N2 - The article presents descriptions of some of the worst moments in the history of sports according to Sports Illustrated magazine. In the third-worst trade in history, behind beads-for-Manhattan and Ruth-for-cash, the Super Bowl--contending Minnesota Vikings exchange five players and eight draft choices for one man with two bad wheels and a penchant for speaking in the third person. Herschel Walker, newly acquired from the Dallas Cowboys, runs 47 yards on his first play as a Viking. Another bad moment was when the night before Super Bowl XXXIII, Atlanta Falcons free safety Eugene Robinson leaves his wife and two children in a Miami hotel and propositions an undercover cop posing as a prostitute. He does so 100 yards from a police sub-station. With 5,000 sportswriters in town. Hours after accepting the Bart Starr Award from Athletes in Action for exhibiting "high moral character." Another bad moment in sports occurred when by holding a 10-Cent Beer Night, the Cleveland Indians give new meaning to bad hops. Some 25,000 fans come and--to be fair--they don't all streak

across the diamond or sprint into centerfield to moon the bleacherites. But many do. With the game tied 5-5 and the Indians at bat in the ninth, the bases weren't loaded but the crowd was. Umpire Nestor Chylak and Cleveland reliever Tom Hilgendorf are both hit over the head with chairs by fans. When order proves impossible to restore, the Indians, having forfeited their dignity, also forfeit the game
SN - 0038822X
UR -
https://login.proxy.bib.uottawa.ca/login?url=http://search.ebscohost.com/login.aspx?direct=true&db=s3h&AN=14532510&site=ehost-live
ER -

TY - JOUR
ID - 4837
T1 - Children and Dangerous Sport and Recreation
A1 - Russell,J.S.
Y1 - 2007/10//
KW - SportDiscus
KW - *SPORTS -- Safety measures
KW - *RECREATION
KW - *CHILD development
KW - *PREVENTION
KW - SAFETY measures
KW - Child Welfare
KW - CHILDREN'S accidents
KW - RISK society
KW - SAFETY regulations
RP - NOT IN FILE
SP - 176
EP - 193
JA - Journal of the Philosophy of Sport
VL - 34
IS - 2
N2 - The article examines the issue of how much focus should be placed on the elimination of physical risk or danger from a child's environment, particularly in the area of sports or physical recreation are concerned. It is suggested in the article that the complete elimination of risk and danger from a child's environment could be a bad thing. The article explores whether physical risk or danger contributes positively in any way to a child's development or life in general. The article also discusses how many U.S. schools have eliminated such games as touch football and tag from their playgrounds and physical education programs
SN - 00948705
UR -
https://login.proxy.bib.uottawa.ca/login?url=http://search.ebscohost.com/login.aspx?direct=true&db=s3h&AN=26651031&site=ehost-live
ER -

TY - JOUR
ID - 4838
T1 - Sun safety: new wrinkles on a burning subject
A1 - Rutsch,L.
A1 - Baird,S.B.
Y1 - 2006/06//
N1 - Accession Number: 2009268464. Language: English. Entry Date: 20061006. Revision Date: 20130322. Publication Type: journal article; anecdote; pictorial. Journal Subset: Double Blind Peer Reviewed; Expert Peer Reviewed; Nursing; Peer Reviewed; USA
KW - CINAHL
KW - Camp Nursing
KW - Health Education -- Methods -- In Infancy and Childhood
KW - Sunburn -- Prevention and Control -- In Infancy and Childhood
KW - Teaching Materials -- Utilization

KW - Child
KW - child safety
KW - Environmental Exposure
KW - Information Resources
KW - Nurse Attitudes
KW - Nursing Role
KW - Skin Neoplasms -- Prevention and Control
KW - Staff Development
KW - Sunscreening Agents
KW - Ultraviolet Rays
RP - NOT IN FILE
SP - 11
EP - 13
JA - CompassPoint
VL - 16
IS - 2
N2 - Promoting healthy camp communities is an inherent part of the camp nurse's role. Sunburn, as a totally preventable injury, is a constant threat in a camp life where outdoor activities are occurring throughout the day. The nurse can use a variety of approaches to teach staff and campers sun safety. Sun Wise, a free teaching kit from the Environmental Protection Agency, provides ideas, activities, and supplies to use with K-8 aged campers
AD - Director, SunWide Program, US Environmental Protection Agency
UR - http://search.ebscohost.com/login.aspx?direct=true&db=cin20&AN=2009268464&site=ehost-live
ER -

TY - JOUR
ID - 4839
T1 - Vitalizing effects of being outdoors and in nature
A1 - Ryan,Richard M.
A1 - Weinstein,Netta
A1 - Bernstein,Jessey
A1 - Brown,Kirk Warren
A1 - Mistretta,Louis
A1 - Gagne,Marylene
Y1 - 2010///
KW - Reviewer Nominated
RP - NOT IN FILE
SP - 159
EP - 168
JF - Journal of Environmental Psychology
VL - 30
IS - 2
PB - Elsevier
SN - 0272-4944
ER -

TY - JOUR
ID - 4840
T1 - Acoustics in physical education settings: the learning roadblock
A1 - Ryan,Stu
A1 - Mendel,Lisa Lucks
Y1 - 2010/01//
KW - SportDiscus
KW - *MUSIC in physical education
KW - *SCHOOL children
KW - SOUND -- Psychological aspects
KW - AMERICAN Speech-Language-Hearing Association

KW - AMERICAN National Standards Institute
KW - ACADEMIC improvement
KW - GUIDELINES
KW - DECIBELS
KW - Florida
KW - Acoustics
KW - Noise
KW - Physical Education
KW - Sound
RP - NOT IN FILE
SP - 71
EP - 83
JA - Physical Education & Sport Pedagogy
VL - 15
IS - 1
N2 - Background: The audibility of teachers and peers is an essential factor in determining the academic performance of school children. However, acoustic conditions in most classrooms are less than optimal and have been viewed as 'hostile listening environments' that undermine the learning of children in school. While research has shown that typical classrooms may be acoustically challenging, the acoustic conditions in physical education settings are without a doubt more challenging than in the typical classroom. To date, researchers in physical education have sparingly focused on the way acoustics affect physical education settings. Aims: The focus of this research effort was to measure noise levels in elementary-, middle- and high-school physical education settings and compare them to the American Speech-Language-Hearing Association (ASHA) guidelines (30 dBA) and American National Standards Institute (ANSI) standards (40 dBA). Documentation of these ambient noise levels may give greater understanding to the effect of background noise on student learning in physical education. Methods: Acoustic environments were measured in indoor and outdoor settings in 12 elementary, 4 middle, and 6 high schools in northwest Florida. Nine outdoor, seven gymnasium, and six covered area settings were evaluated. Ambient noise levels were recorded for each unoccupied physical education setting. A sound decibel digital meter which met American National Standard Specification for Sound Level Meters set on the 'A'-weighted scale and on slow response was used to record the noise levels for each setting. Two-tailed t-tests were used for comparison of the overall mean noise levels in outdoor, gymnasium, and covered physical education settings to the standards established by ANSI and ASHA. Results: The findings showed that all of the settings were significantly ($p < .05$) higher than the established standards and only 1 of the 22 physical education settings studied had background noise levels at or below 40 dBA. These results also indicate that the physical education settings investigated exhibit ambient noise levels at 10-15 dB higher than recommended levels. Conclusions: In light of the acoustics standards and prior research, these results are discouraging if not alarming. These results also suggest that the background noise in physical education settings is likely detrimental to student learning. These findings indicate an immediate need for administrators to support future research and determine the most appropriate, cost-effective procedures to reduce noise in existing physical education settings. Strategies for reducing high noise levels in physical education settings include sound amplification devices, acoustical modifications, and future construction strategies. ABSTRACT FROM AUTHOR
SN - 17408989
UR -
https://login.proxy.bib.uottawa.ca/login?url=http://search.ebscohost.com/login.aspx?direct=true&db=s3h&AN=46777265&site=ehost-live
ER -

TY - JOUR
ID - 4841
T1 - A Weekend on the Banadad Trail
A1 - Rykken,Anne
Y1 - 2011/11//
KW - SportDiscus
KW - *SKIS & skiing
KW - FIRST person narrative
KW - Minnesota

RP - NOT IN FILE
SP - 69
EP - 70
JA - Cross Country Skier
VL - 31
IS - 1
N2 - A personal narrative is presented which explores the author's experience of cross country skiing with a group of children on the Banadad Ski Trail along the Gunflint Trail in Grand Marais, Minnesota, which included a two night stay in a yurt, playing outside, and hiking and skiing on frozen Bedew Lake
SN - 02789213
UR - https://login.proxy.bib.uottawa.ca/login?url=http://search.ebscohost.com/login.aspx?direct=true&db=s3h&AN=69668600&site=ehost-live
ER -

TY - JOUR
ID - 4842
T1 - Are younger children lacking exercise?
A1 - S.,Matsuoka
A1 - K.,Mori
A1 - T.,Yamaue
A1 - M.,Takamatsu
A1 - T.,Oonishi
Y1 - 2008/08//
KW - SportDiscus
KW - *EXERCISE for children
KW - *LIFESTYLES
KW - *CHILDREN -- Health
KW - CHILDREN & the environment
KW - PARENTAL influences
KW - CHILDREN
KW - Exercise
KW - Life Style
KW - Parents
KW - sport
RP - NOT IN FILE
SP - 369
EP - 374
JA - Japanese Journal of Clinical Sports Medicine
VL - 16
IS - 3
N2 - In order to resolve the theme "Are younger children lacking exercise?", the relationships among the quantity of exercise, lifestyle, home environment and parents' thoughts were studied in 204 preschoolers aged 4-5 years old. Lifestyle and parents' thoughts affected the activity and quantity of exercise of kids. Early rising, the presence of siblings, attending kindergarten and the mother not working allowed younger children to exercise more than late risers, those without siblings, holidays, and with both parents working. Moreover, parents' opinion on sport affected the quantity of the child's exercise. In order to increase the amount of exercise in younger children, it is advisable for parents to wake up earlier and play with their kids outside. ABSTRACT FROM AUTHOR
SN - 13464159
UR - https://login.proxy.bib.uottawa.ca/login?url=http://search.ebscohost.com/login.aspx?direct=true&db=s3h&AN=35010687&site=ehost-live
ER -

TY - JOUR
ID - 4849

T1 - Measuring Physical Environments of Parks and Playgrounds: EAPRS Instrument Development and Inter-Rater Reliability
A1 - Saelens,Brian E.
A1 - Frank,Lawrence D.
A1 - Auffrey,Christopher
A1 - Whitaker,Robert C.
A1 - Burdette,Hillary L.
A1 - Colabianchi,Natalie
Y1 - 2006/01/02/
KW - SportDiscus
KW - *PARKS
KW - *PLAYGROUNDS
KW - *OUTDOOR recreation
KW - *PHYSICAL fitness
KW - *EXERCISE
KW - *PUBLIC health
KW - Active Living
KW - activity
KW - recreation facilities
KW - trails
RP - NOT IN FILE
SP - S190
EP - S207
JF - Journal of Physical Activity & Health
JA - J Phys Act Health
VL - 3
N2 - Background: Reliable and comprehensive measurement of physical activity settings is needed to examine environment-behavior relations. Methods: Surveyed park professionals (n = 34) and users (n = 29) identified park and playground elements (e.g., trail) and qualities (e.g., condition). Responses guided observational instrument development for environmental assessment of public recreation spaces (EAPRS). Item inter-rater reliability was evaluated following observations in 92 parks and playgrounds. Instrument revision and further reliability testing were conducted with observations in 21 parks and 20 playgrounds. Results: EAPRS evaluates traillpath, specific use (e.g., picnic), water-related, amenity (e.g., benches), and play elements, and their qualities. Most EAPRS items had good-excellent reliability, particularly presence/number items. Reliability improved from the original (n = 1088 items) to revised (n = 646 items) instrument for condition, coverage/shade, and openness/visibility items. Reliability was especially good for play features, but cleanliness items were generally unreliable. Conclusions: The EAPRS instrument provides comprehensive assessment of parks' and playgrounds' physical environment, with generally high reliability. ABSTRACT FROM AUTHOR
SN - 15433080
UR - https://login.proxy.bib.uottawa.ca/login?url=http://search.ebscohost.com/login.aspx?direct=true&db=s3h&AN=20971316&site=ehost-live
ER -

TY - JOUR
ID - 4851
T1 - The dark and bright sides of self-efficacy in predicting learning, innovative and risky performances
A1 - Salanova,M.
A1 - Lorente,L.
A1 - Martinez,I.M.
Y1 - 2012/11//
N1 - DA - 20121119
IS - 1138-7416 (Print)
IS - 1138-7416 (Linking)
LA - eng
PT - Comparative Study
PT - Journal Article

PT - Research Support, Non-U.S. Gov't
SB - IM
KW - Achievement
KW - Adolescent
KW - Adult
KW - Creativity
KW - Female
KW - Forecasting
KW - Humans
KW - Learning
KW - Longitudinal Studies
KW - Male
KW - Middle Aged
KW - Psychological Tests
KW - Risk-Taking
KW - Self Efficacy
KW - Students
KW - Psychology
KW - Young Adult
KW - Reviewer Nominated
RP - NOT IN FILE
SP - 1123
EP - 1132
JA - Span.J Psychol
VL - 15
IS - 3
N2 - The objective of this study is to analyze the different role that efficacy beliefs play in the prediction of learning, innovative and risky performances. We hypothesize that high levels of efficacy beliefs in learning and innovative performances have positive consequences (i.e., better academic and innovative performance, respectively), whereas in risky performances they have negative consequences (i.e., less safety performance). To achieve this objective, three studies were conducted, 1) a two-wave longitudinal field study among 527 undergraduate students (learning setting), 2) a three-wave longitudinal lab study among 165 participants performing innovative group tasks (innovative setting), and 3) a field study among 228 construction workers (risky setting). As expected, high levels of efficacy beliefs have positive or negative consequences on performance depending on the specific settings. Unexpectedly, however, we found no time x self-efficacy interaction effect over time in learning and innovative settings. Theoretical and practical implications within the social cognitive theory of A. Bandura framework are discussed
AD - Department of Social Psychology, Universitat Jaume I. Av. Sos Baynat, s/n. 12071. Castellon, Spain. Marisa.Salanova@psi.uji.es
UR - 23156920
ER -

TY - JOUR
ID - 4856
T1 - Insights about young children working with a partner: "initiators, responders, collaborators and the non-compliant"
A1 - Sanders,S.W.
Y1 - 2001///Spring
KW - SportDiscus
KW - *PHYSICAL education & training
KW - *PARTICIPATION
KW - Cooperation
KW - Child Development
KW - Video Recording
KW - kindergarten
KW - BOYS
KW - girls

KW - motor skill
KW - Observation
KW - primary school
KW - Child
RP - NOT IN FILE
SP - 58
EP - 66
JA - Physical Educator
VL - 58
IS - 2
CY - ;
N2 - This study identified and described participation roles kindergarten and second grade children took on during partner tasks in physical education. Over a period of one year four participation roles were identified: (1) initiating partner, (2) responding partner, (3) collaborating partner, and (4) non-compliant partner. Data suggested a shift from kindergarten to second grade in roles students assumed during partner activities and that this change may be related to egocentrism as a developmental characteristic of young. A class of 24 kindergarten children eagerly wait at the door of the gymnasium to enter and begin 30-minutes of physical education. As they enter, the teacher instructs them to find and sit on one of the two-dozen (four-inch diameter) colored dots which have been painted on the gym floor. The dots are a variety of different colors, spaced six feet apart, and are used by the teacher to assist in spacing the young children evenly on one half of the gym. The 24 dots have been painted on the floor in a rectangular pattern. As the teacher views her class she sees four lines of children with equal space between each line. She asks the children in the two middle lines to turn and face the children in the outside lines. Children have been asked to turn and face a classmate before. The children's voices can now be heard, whispering, and they appear ready to burst into movement at any time. Children do not know if they will be throwing, catching, kicking or striking, but they do know that the classmate they are facing is to be their partner. On the other end of the gym two classes of second grade students, taught by a different teacher, are sitting on 48 colored dots, involved in a similar routine
SN - 00318981
UR -
https://login.proxy.bib.uottawa.ca/login?url=http://search.ebscohost.com/login.aspx?direct=true&db=s3h&AN=SPHS-815033&site=ehost-live;http://articles.sirc.ca/search.cfm?id=S-815033;http://www2.truman.edu/pek
L2 - http://articles.sirc.ca/search.cfm?id=S-815033
ER -

TY - JOUR
ID - 4860
T1 - THE EFFECTS OF CONCURRENT RESISTANCE AND ENDURANCE TRAINING FOLLOW A DETRAINING PERIOD IN ELEMENTARY SCHOOL STUDENTS
A1 - Santos,Albano P.
A1 - Marinho,Daniel A.
A1 - Costa,Aldo M.
A1 - Izquierdo,M.I.K.E.
A1 - Marques,Ma_Rio C.
Y1 - 2012/06//
KW - SportDiscus
KW - *ADAPTATION (Physiology)
KW - *AEROBIC exercises
KW - *BODY composition
KW - *CARDIOPULMONARY system
KW - *CHILDREN -- Health
KW - *EXERCISE
KW - *EXERCISE physiology
KW - *EXERCISE tests
KW - *BIOELECTRIC impedance
KW - *JUMPING
KW - *MUSCLE strength
KW - *MUSCLE strength -- Testing

KW - *RUNNING
KW - *TEENAGERS -- Health
KW - *CARDIOVASCULAR fitness
KW - *THROWING (Sports)
KW - *OXYGEN consumption (Physiology)
KW - *EXERCISE intensity
KW - adolescence
KW - Analysis of Variance
KW - Clinical Trials
KW - LONGITUDINAL method
KW - SAMPLING (Statistics)
KW - SCALES (Weighing instruments)
KW - PRE-tests & post-tests
KW - REPEATED measures design
KW - Descriptive Statistics
KW - Portugal
KW - cardiorespiratory
KW - experimental
KW - program recess
KW - schooling pupils
KW - strength
KW - youth
RP - NOT IN FILE
SP - 1708
EP - 1716
JA - Journal of Strength & Conditioning Research (Lippincott Williams & Wilkins)
VL - 26
IS - 6
N2 - The article reports on research conducted to compare an eight-week training period of resistance training alone (GR), or combined resistance and endurance training (GCCM), followed by 12 weeks of detraining (DT) and to determine the effect each had on body composition, strength, and VO2max adaptations in 42 adolescent school boys. Researchers found significant loss was observed after a DT period in GR but not in GCCM and that training program effects persist at the end of the DT period
SN - 10648011
UR -
https://login.proxy.bib.uottawa.ca/login?url=http://search.ebscohost.com/login.aspx?direct=true&db=s3h&AN=76403657&site=ehost-live
ER -

TY - JOUR
ID - 4861
T1 - Gender differences in physical activity during recess time. (Abstract)
A1 - Santos,P.
A1 - Silva,P.
A1 - Guerra,S.
A1 - Ribeiro,J.C.
A1 - Oliveira,J.
A1 - Duarte,J.A.R.
A1 - Mota,J.
Y1 - 2003/09//
KW - SportDiscus
KW - *PHYSICAL fitness
KW - *RECESSES
KW - *CARDIOVASCULAR fitness
KW - *STRESS (Physiology)
KW - *PARTICIPATION
KW - *EXERCISE -- Physiological aspects

KW - Elementary Schools
KW - BOYS
KW - girls
KW - Comparative Studies
KW - Child
KW - SEX FACTOR
RP - NOT IN FILE
SP - 150
EP - 151
JA - Revista Portuguesa de Ciencias do Desporto
VL - 3
IS - 2
CY - ;
SN - 16450523
UR -
https://login.proxy.bib.uottawa.ca/login?url=http://search.ebscohost.com/login.aspx?direct=true&db=s3h&AN=SPHS-898033&site=ehost-live;http://articles.sirc.ca/search.cfm?id=S-898033;http://www.fcdef.up.pt
L2 - http://articles.sirc.ca/search.cfm?id=S-898033
ER -

TY - JOUR
ID - 4862
T1 - Gender differences in physical activity during fifth-grade physical education and recess periods
A1 - Sarkin,J.A.
A1 - McKenzie,T.L.
A1 - Sallis,J.F.
Y1 - 1997/10//
KW - SportDiscus
KW - *PHYSICAL education & training
KW - *CARDIOVASCULAR fitness
KW - Elementary Schools
KW - SEX FACTOR
RP - NOT IN FILE
SP - 99
EP - 106
JF - Journal of Teaching in Physical Education
VL - 17
IS - 1
CY - ;
N2 - Boys are typically more physically active than girls, but schools have the potential to promote health-related physical activity for all children. This study investigated gender differences in children's physical activity levels during physical education (PE) classes and unstructured recess periods. The activity levels of 91 fifth-grade children were assessed by accelerometer (Caltrac) on 3 days. Results indicated that children's activity levels were low to moderately stable from day to day in both environments. Boys and girls had similar activity levels during PE, t(89) = 1.49, p = .140, but boys were significantly more active than girls during recess, t(89) = 3.27, p = .002. Girls were significantly more active during PE than they were at recess, t(51) = 2.08, p = .043. the results suggest that structured PE classes may provide similar amounts of physical activity for both genders
SN - 02735024
UR -
https://login.proxy.bib.uottawa.ca/login?url=http://search.ebscohost.com/login.aspx?direct=true&db=s3h&AN=SPH437314&site=ehost-live;http://articles.sirc.ca/search.cfm?id=437314;http://www.humankinetics.com/
L2 - http://articles.sirc.ca/search.cfm?id=437314
ER -

TY - JOUR
ID - 4864

T1 - The Quality of Time in Nature: How Fascination Explains and Enhances the Relationship Between Nature Experiences and Daily Affect
A1 - Sato,Izuru
A1 - Conner,Tamlin S.
Y1 - 2013///
KW - Reviewer Nominated
RP - NOT IN FILE
JA - Ecopsychology
PB - Mary Ann Liebert, Inc. 140 Huguenot Street, 3rd Floor New Rochelle, NY 10801 USA
SN - 1942-9347
ER -

TY - JOUR
ID - 4865
T1 - Promotion of school recess physical activity among elementary school children: A literature review
A1 - Sato,Mai
A1 - Ishii,Kaori
A1 - Shibata,Ai
A1 - Oka,Koichiro
Y1 - 2012/04//
KW - SportDiscus
KW - *PHYSICAL activity
KW - *SCHOOL recess breaks
KW - *SCHOOL children
KW - *PLAYGROUNDS
KW - School Environment
KW - Japan
KW - CHILDREN
KW - Environment
KW - Exercise
KW - Health
RP - NOT IN FILE
SP - 157
EP - 167
JA - Japanese Journal of Physical Fitness & Sports Medicine
VL - 61
IS - 2
SN - 0039906X
UR -
https://login.proxy.bib.uottawa.ca/login?url=http://search.ebscohost.com/login.aspx?direct=true&db=s3h&AN=77466204&site=ehost-live
ER -

TY - JOUR
ID - 4872
T1 - Getting Kids Outside to Appreciate Nature
A1 - Scheer,Roddy
A1 - Moss,Doug
Y1 - 2013/04/14/
N1 - Accession Number: 87098431; Scheer, Roddy; Moss, Doug; Source Info: 4/14/2013, p3; Thesaurus Term: NATURE; Subject Term: CHILDREN -- United States; Subject Term: TELEVISION monitors; Subject Term: DEPRESSION in children; Subject Term: CREATIVE ability in children; Subject Term: AGGRESSION (Psychology) in children; Number of Pages: 1p; Document Type: Article; Full Text Word Count: 611
KW - Nature
KW - CHILDREN -- United States
KW - TELEVISION monitors
KW - DEPRESSION in children

KW - CREATIVE ability in children
KW - AGGRESSION (Psychology) in children
KW - Reviewer Nominated
RP - NOT IN FILE
SP - 3
EP - 3
JA - E THIS WEEK
N2 - The article discusses a report which revealed that kids who stay inside the house too much may suffer "nature deficit disorder" which can add to a range of behavioral problems like attention disorders, depression and declining creativity. Another report showed that U.S. children spend more than 53 hours a week engaged with entertainment media, only about six percent of the children play. Researchers also found that kids who play outside are less aggressive, in better shape and more creative
M3 - Article
UR - http://search.ebscohost.com/login.aspx?direct=true&db=8gh&AN=87098431&site=ehost-live
ER -

TY - JOUR
ID - 4873
T1 - Campers' Affinity for Nature in Environmental Education Programs
A1 - Schmillen,Justin
A1 - Jacobs,Jeff
A1 - Roark,Mark F.
Y1 - 2011/07//
KW - SportDiscus
KW - *OUTDOOR education
KW - *SEDENTARY lifestyles
KW - Nature
KW - SOCIAL development
KW - Environmental Education
RP - NOT IN FILE
SP - 8
EP - 11
JA - Camping Magazine
VL - 84
IS - 4
N2 - The article presents a study exploring the affinity among children towards nature as it not only develop environmental values and attitude but also foster positive social development. However, it highlights the advancing technological culture as one of the factors that separate children from nature as it introduced them the sedentary lifestyle that are spent indoors. Researchers have depicted a decline by 50 percent from 1997 to 2003 on time spent in outdoor activities
SN - 07404131
UR -
https://login.proxy.bib.uottawa.ca/login?url=http://search.ebscohost.com/login.aspx?direct=true&db=s3h&AN=65958741&site=ehost-live
ER -

TY - JOUR
ID - 4875
T1 - Throwing and catching in children with developmental coordination disorder (DCD)
A1 - Schott,Nadja M.
A1 - Rhode,Robert
Y1 - 2007/07/02/
KW - SportDiscus
KW - *MOTOR ability in children
KW - *DEVELOPMENTAL disabilities
KW - *THROWING (Sports)
KW - *PHYSICAL education for children

KW - TESTING
KW - SOCIAL aspects
KW - MOVEMENT disorders in children
KW - Social Interaction
RP - NOT IN FILE
SP - S125
EP - S126
JF - Journal of Sport & Exercise Psychology
JA - J Sport Exerc Psychol
VL - 29
N2 - Background and purpose: Skills that are important for both social interaction on the playground and performance in physical education are ball handling skills because they are the cornerstones of many games. Unlike motor skills that develop naturally with general movement experiences, even children without disabilities need to be taught and to practice ball handling skills to become proficient. The purpose of this study was to compare ball skill performance of children with and without DCD in a one-to-one testing situation. Subjects: We recruited a sample of 116 children, 51 girls and 65 boys, aged between 7 and 11 years (M = 8.82 years, SD = 0.80). In total, 30 (25.9%) met criteria for DCD, and 86 (74.1%) did not. Method: Motor ability was assessed using the Movement Assessment Battery (M-ABC). Each participant was required to complete 10 overhand throws and to catch a ball adjusted to their hand size with two hands 10 times. Video analysis was used to determine the developmental level of throwing and catching (Roberton & Konczak, 2001). Results: A MANOVA with the factors DCD-group and age band on throwing (foot action, trunk action, backswing, humerus action, forearm action) and catching (arm action, hand action, body action) revealed a significant main effect of DCD for the developmental sequences of throwing (p < .05) as well as for catching (p < .01). Furthermore, children without DCD caught more balls than did the DCD children, F(3, 99) = 10.13, p < .001, I úA ý= .235, and their throwing accuracy was significantly superior to that of the DCD children, F(3, 101) = 3.59, p = .016, I úA ý= .096. Conclusions: The findings from this study clearly showed that children with DCD are at a disadvantage in controlling a ball. Without a tailored intervention, these children will not be able to participate properly in physical activities. ABSTRACT FROM AUTHOR
SN - 08952779
UR -
https://login.proxy.bib.uottawa.ca/login?url=http://search.ebscohost.com/login.aspx?direct=true&db=s3h&AN=25216340&site=ehost-live
ER -

TY - JOUR
ID - 4876
T1 - Intramural Programs: A Recess Renovation
A1 - Schroeder,Cynthia
A1 - Cluphf,David
Y1 - 2007///Spring
KW - SportDiscus
KW - *PHYSICAL education
KW - *INTRAMURAL sports
KW - *STUDENTS -- Recreation
KW - COLLINSVILLE Intermediate School (Collinsville,Ill.)
KW - GOVERNMENT policy
KW - COLLINSVILLE (Ill.)
KW - Illinois
RP - NOT IN FILE
SP - 52
EP - 55
JA - Illinois Journal for Health, Physical Education, Recreation & Dance
VL - 59
N2 - The article discusses how Illinois schools are dealing with government guidelines for children's physical education (PE) and how intramural sports programs fit in. Many schools get waivers that release students to take other classes besides PE at the risk of their overall daily fitness activity. The Collinsville Intermediate School

(CIS) in Collinsville, Illinois instituted an intramural (IM) sports program to provide children with athletic opportunities
SN - 10622764
UR -
https://login.proxy.bib.uottawa.ca/login?url=http://search.ebscohost.com/login.aspx?direct=true&db=s3h&AN=26493039&site=ehost-live
ER -

TY - JOUR
ID - 4880
T1 - Young people's health as a challenge for physical education in schools in the twenty-first century: the case of Flanders (Belgium)
A1 - Seghers,Jan
A1 - de Martelaer,Kristine
A1 - Cardon,Greet
Y1 - 2009/10//
KW - SportDiscus
KW - *YOUTH
KW - *HEALTH
KW - *OVERWEIGHT teenagers
KW - *PHYSICAL education
KW - *PHYSICAL activity
KW - *HEALTH behavior
KW - OBESITY in adolescence
KW - School Environment
KW - CURRICULA (Courses of study)
KW - FLANDERS
KW - physical activity promotion
KW - Public Health
KW - quality physical education
KW - young people
RP - NOT IN FILE
SP - 407
EP - 420
JA - Physical Education & Sport Pedagogy
VL - 14
IS - 4
N2 - Background: A lack of physical activity (PA) and the occurrence of overweight and obesity among school-aged youth have become a major societal problem. Arising from the general concern about the future generations' health, schools have been found to be potentially important settings to promote positive health behaviour, since all pupils can be reached spending large amounts of time in the school environment. Moreover, it is beyond doubt that physical education (PE) has a key role to play. As in other European countries, the Flemish PE goals identify the importance of PA in the development and maintenance of good health. However, the discrepancy between the important role attributed to PE with regard to the development of a healthy and physically active lifestyle and the currently observed increasing sedentary and unhealthy behaviour of children and youth, is one of the reasons to question the efficiency of the current PE curricula. Aims: Within the framework of the Flemish education system, this paper aims to document on the pathways by which PE and schools can contribute to establish lifetime adherence to sport and physical activity in order to enhance and maintain young people's health later in life. From a public health perspective, this is one of the main challenges for PE in the forthcoming decades. Methods: The current PE curriculum requirements and implementation in Flemish schools are described as a starting point. Then, evidence-based recommendations are formulated in order to optimise the content and delivery of the PE curriculum towards its health-related goals. Conclusions: School PE has been recognised as the foundation base of the PA participation pyramid. Nevertheless, there is a need to move beyond the PE and school curriculum. Especially, schools, parents and the community should work together to encourage children and youth to be physically active. As a consequence, the PE teachers' role should not be restricted to the education of pupils during PE lessons, but should be expanded to include guiding youths in the process of becoming physically active both inside and outside school and for life. PE teachers have

to accept this responsibility and this effort has to be recognised by the school board. ABSTRACT FROM AUTHOR
SN - 17408989
UR -
https://login.proxy.bib.uottawa.ca/login?url=http://search.ebscohost.com/login.aspx?direct=true&db=s3h&AN=45048538&site=ehost-live
ER -

TY - JOUR
ID - 4881
T1 - Use of recess-based activities as a method to increase adolescents' time spent in vigorous activity... CSM 2013
A1 - Sen,A.
A1 - Caballero,D.
A1 - Fentnor,L.
A1 - Donovan,S.
Y1 - 2012/12//
N1 - Accession Number: 2011796616. Language: English. Entry Date: 20130208. Revision Date: 20130208. Publication Type: journal article; abstract; research. Journal Subset: Allied Health; Double Blind Peer Reviewed; Editorial Board Reviewed; Expert Peer Reviewed; Peer Reviewed; USA. Special Interest: Pediatric Care; Physical Therapy. Instrumentation: System of Observind Play and leisure Activity in children (SOPLAY). NLM UID: 101155626
KW - CINAHL
KW - Physical Activity -- In Infancy and Childhood
KW - Students,Elementary
KW - Accelerometry -- Equipment and Supplies
KW - Child
KW - convenience sample
KW - Female
KW - Human
KW - Male
KW - Observational Methods
KW - Scales
KW - Sex Factors
KW - Virginia
RP - NOT IN FILE
SP - 42
EP - 42
JA - Cardiopulmonary Physical Therapy Journal
VL - 23
IS - 4
SN - 1541-7891
UR - http://search.ebscohost.com/login.aspx?direct=true&db=cin20&AN=2011796616&site=ehost-live
ER -

TY - JOUR
ID - 4882
T1 - Recent Change in the Annual Pattern of Sexually Transmitted Diseases in the United States
A1 - Shah,Ami P.
A1 - Smolensky,Michael H.
A1 - Burau,Keith D.
A1 - Cech,Irina M.
A1 - Lai,Dejian
Y1 - 2007/09//
KW - SportDiscus
KW - *SEXUALLY transmitted diseases
KW - *DISEASES

KW - Databases
KW - Seasonal Variations
KW - CHLAMYDIA
KW - GONORRHEA
KW - syphilis
KW - United States
KW - Chlamydia,Gonorrhea
KW - seasonal variation
KW - Sexually Transmitted Diseases
RP - NOT IN FILE
SP - 947
EP - 960
JA - Chronobiology International: The Journal of Biological & Medical Rhythm Research
VL - 24
IS - 5
N2 - This study analyzed the 1999 to 2003 database of the Center for Disease Control and Prevention (CDC) for seasonal and longer-term time trends in the sexually transmitted diseases (STDs) of chlamydia, gonorrhea, and syphilis in the United States. Linear regression was used to ascertain time trends, and a linear mixed auto-regression model was applied to determine the statistical significance of the major peaks relative to the annualized time series mean. A statistically significant increasing trend during the 5 yr span was documented only in the incidence of chlamydia. No clear annual periodicity was detected in any of the STDs; instead, significant three-month cycles were documented in all the STDs, with prominent peaks evident in March, May, August, and November. The March and May peaks could be associated with the sexual activities of young adults during spring break, which for different colleges and universities, commences as early as mid- to late-February and concludes as late as early- to mid-April, when huge numbers of sexually active youth congregate at beach resort settings. We propose the August peak is representative of summer sexual activity, in particular, of youths during school recess when adult supervision is poor. Finally, the autumn peak seems to be an expression of an endogenous annual rhythm in human reproductive biology, exemplified by elevated levels of testosterone in young males and sexual activity at this time of the year. ABSTRACT FROM AUTHOR
SN - 07420528
UR -
https://login.proxy.bib.uottawa.ca/login?url=http://search.ebscohost.com/login.aspx?direct=true&db=s3h&AN=27442300&site=ehost-live
ER -

TY - JOUR
ID - 4883
T1 - Understanding Constraints Younger Youth Face in Engaging as Volunteers
A1 - Shannon,Charlene S.
A1 - Robertson,Brenda J.
A1 - Morrison,Kate S.
A1 - Werner,Tara L.
Y1 - 2009///Winter
KW - SportDiscus
KW - *TEENAGERS
KW - *RECREATION
KW - *OUTDOOR recreation
KW - *INTRINSIC motivation
KW - YOUNG volunteers
KW - VOLUNTEER service
KW - ATLANTIC Provinces
KW - constraints
KW - volunteering
KW - youth volunteers
RP - NOT IN FILE
SP - 17
EP - 37

JA - Journal of Park & Recreation Administration
VL - 27
IS - 4
N2 - Many nonprofit organizations needing volunteers struggle to find individuals who can provide the hours of support their programs, services, and events need (Urban Institute, 2004). Researchers have recognized the contribution of youth volunteers and have examined various aspects of youth volunteer experiences to better understand them (e.g., Hall, McKeown, & Roberts, 2001). Much of the research has focus on those 13 and older, however, youth between the ages of 8 and 12 (younger youth) have been overlooked. Research shows that younger youth do volunteer (Grimm, Dietz, Spring, Arey, & Foster-Bey, 2005), but little is known about the experiences they have. The purpose of this study was to explore constraints younger youth face and ways in which these constraints are negotiated. Using semi-structured, face-to-face interviews, data were collected from 73 youth volunteers (31 males; 42 females) ages 8 to 12 and 7 Executive Directors from Boys and Girls Clubs (BGC) in Atlantic Canada. The findings indicated that attitudes of adults in the community toward younger youth can limit the volunteer opportunities that are welcoming for this age group, affect younger youths' perception of their abilities, and influence their enjoyment of the volunteer experience. The important roles facilitators played in younger youths' access to and enjoyment of volunteer experiences was evident. Youth leaders can help younger youth overcome constraints by ensuring that youth are matched with volunteer tasks that are appropriate for their skill set, are adequately prepared for the tasks and situations that may arise while performing their tasks, participate in tasks with friends, and have chances to discuss their volunteer experiences. Parents can also support involvement through preparing their child for, and debriefing volunteer experiences. Forming partnerships with organizations that need volunteers may be helpful in lessening some of the challenges youth leaders encounter when trying to facilitate volunteering experiences for younger youth. Efforts to educate the public about the abilities of younger youth to contribute to their community may be important in shifting attitudes and increasing the welcoming opportunities available. ABSTRACT FROM PUBLISHER
SN - 07351968
UR -
https://login.proxy.bib.uottawa.ca/login?url=http://search.ebscohost.com/login.aspx?direct=true&db=s3h&AN=45463293&site=ehost-live
ER -

TY - JOUR
ID - 4894
T1 - How physically active are students during recess periods?
A1 - Sherman,Nestor W.
A1 - Silverman,Stephen
Y1 - 1998/03//
KW - SportDiscus
KW - *SCHOOL recess breaks
KW - *EXERCISE
RP - NOT IN FILE
SP - 11
JA - JOPERD: The Journal of Physical Education, Recreation & Dance
VL - 69
IS - 3
N2 - Presents a study aimed at determining the level of physical activity of school children during the recess period. Views of school administrators on the recess period; Details on the subjects of the study; Results of the study; Recommendations made by the researchers conducting the study
SN - 07303084
UR -
https://login.proxy.bib.uottawa.ca/login?url=http://search.ebscohost.com/login.aspx?direct=true&db=s3h&AN=351669&site=ehost-live
ER -

TY - JOUR
ID - 4896
T1 - The Potential for Brain Injury on Selected Surfaces Used by Cheerleaders
A1 - Shields,Brenda J.

A1 - Smith,Gary A.
Y1 - 2009/11//
KW - SportDiscus
KW - *CHEERLEADING
KW - *BRAIN -- Wounds & injuries
KW - *GYMNASIUMS
KW - *SPORTS facilities
KW - *FALLS (Accidents)
KW - SOIL moisture
KW - critical height
KW - Head Injury Criterion
KW - surface impact attenuation
KW - Triax
RP - NOT IN FILE
SP - 595
EP - 602
JA - Journal of Athletic Training
VL - 44
IS - 6
N2 - Context: Although playground surfaces have been investigated for fall impact attenuation, the surfaces that cheerleaders use have received little attention. Objective: To determine (1) the critical height for selected surfaces used by cheerleaders at or below which a serious head impact injury from a fall is unlikely to occur, (2) the critical heights for non-impact-absorbing surfaces for comparison purposes, and (3) the effect of soil moisture and grass height on gmax (which is defined as the multiple of g [acceleration due to gravity at the earth's surface at sea level: ie, 32.2 feetA ús-1A ús-1] that represents the maximum deceleration experienced during an impact) and the Head Injury Criterion (HIC) at the critical height for a dry grass surface. Design: Observational study. Settings: A local cheerleading gym, indoor locations within the authors' institution, and various outdoor locations. Main Outcome Measure(s): gmax, HIC, and critical height. Results: Critical heights for the surfaces tested ranged from 0.5 ft (0.15 m) for concrete and vinyl tile installed over concrete to more than 11 ft (3.35 m) for a spring floor. Increases in grass height and soil moisture resulted in an increase in the critical height for grass surfaces. Only spring floors and 4-in (0.10-m)-thick landing mats placed on traditional foam floors had critical heights greater than 10.5 ft (3.20 m), thus providing enough impact-absorbing capacity for performance of 2-level stunts. Conclusions: The potential for serious head impact injuries can be minimized by increasing the shock-absorbing capacity of the surface, decreasing the height from which the person falls, or both. Cheerleaders and cheerleading coaches should use the critical heights reported in this study to compare the relative impact-absorbing capacities of the various surfaces tested, with critical height as an indicator of the impact-absorption capacity of the surface. The findings of this study can be used to select the most appropriate surface for the type of maneuver to be performed, based on the maximum height expected to be achieved by the cheerleader(s) during execution of the maneuver. Cheerleaders should not perform maneuvers at heights that exceed the critical height for the surface on which they are performing. ABSTRACT FROM AUTHOR
SN - 10626050
UR -
https://login.proxy.bib.uottawa.ca/login?url=http://search.ebscohost.com/login.aspx?direct=true&db=s3h&AN=47096238&site=ehost-live
ER -

TY - JOUR
ID - 4897
T1 - A Study of Physical Contact in Passing-Under Movements --Children with Developmental Disorders Have More Collisions--
A1 - Shimatani,K.O.J.I.
A1 - Tanaka,Y.O.S.H.
A1 - Kanai,S.H.U.S.
A1 - Otsuka,A.K.I.R.
A1 - Oki,S.A.D.A.
A1 - Sekiya,H.I.R.O.
Y1 - 2008/12//

KW - SportDiscus
KW - *CHILDREN
KW - *DISEASES
KW - *HUMAN growth
KW - *HUMAN locomotion
KW - Physical Contact
KW - developmental disorders
KW - passing-under movements
RP - NOT IN FILE
SP - 721
EP - 725
JA - Rigakuryoho Kagaku
VL - 23
IS - 6
N2 - [Purpose] In this study, we examined physical contact with obstacles by children with and without developmental disorders (DD). [Subjests] Participants were nine children with DD and nine normal children. [Methods] All participants went through a course with seven pieces of playground equipment and six bars at different heights under three conditions: the first trial was with no instruction, the second trial was with an instruction to avoid collision, and the third trial was with an instruction to avoid collision and go through as fast as possible. [Results] The children with DD had more physical contact with the bars than normal children, indicating that attention deficit was not the cause of the increased contact in the children with DD. They also showed a significantly higher number of waist-contacts when compared to the normal children. This suggests that the children with DD had more contact when concurrent visual-feedback could not be used during avoidance movements. [Conclusion] It is possible that incorrect estimation based on body-characteristic information is the cause of the increased contact in the children with DD. ABSTRACT FROM AUTHOR
SN - 13411667
UR -
https://login.proxy.bib.uottawa.ca/login?url=http://search.ebscohost.com/login.aspx?direct=true&db=s3h&AN=37211758&site=ehost-live
ER -

TY - JOUR
ID - 4898
T1 - Virtual competition : video games, children and sport
A1 - Sibillin,A.
Y1 - 2005///
KW - SportDiscus
KW - *PHYSICAL fitness
KW - *PARTICIPATION
KW - *STRESS (Physiology)
KW - *CARDIOVASCULAR fitness
KW - Child
KW - VIDEO GAME
RP - NOT IN FILE
SP - 36
EP - 37
JA - Sports Coach
VL - 28
IS - 1
CY - ;
N2 - Kids are getting fatter and lazier. Why? They spend too much time in front of the television, playing video games. Solution? Turn off the television and send them outside to play. [ABSTRACT FROM AUTHOR]
SN - 03145468
UR -
https://login.proxy.bib.uottawa.ca/login?url=http://search.ebscohost.com/login.aspx?direct=true&db=s3h&AN=SPHS-994902&site=ehost-live;http://articles.sirc.ca/search.cfm?id=S-994902
L2 - http://articles.sirc.ca/search.cfm?id=S-994902

ER -

TY - JOUR
ID - 4901
T1 - Relating Physical Education and Activity Levels to Academic Achievement in Children
A1 - Siegel,Donald
Y1 - 2007/01//
KW - SportDiscus
KW - *ACADEMIC achievement
KW - *SPORTS participation
KW - *PHYSICAL education
KW - *CHILD development
KW - *ATHLETICS
KW - *SPORTS
KW - *SCHOOLS -- Exercises & recreations
KW - *PHYSICAL fitness
KW - STUDENT organizations & activities
RP - NOT IN FILE
SP - 10
EP - 10
JA - JOPERD: The Journal of Physical Education, Recreation & Dance
VL - 78
IS - 1
N2 - The article presents a study showing the positive relationship between academic achievement and physical activity or participation in sports conducted by Coe, Pivarnik, Womack, Reeves, and Malina. The group reveals that the students involved in regular physical activity outside of school have higher academic achievement scores. This result implies that sports participation meets threshold level for physical activity that is essential to produce potentially desirable effects. Physical education classes need to offer greater number of healthy activities because the students are found to be inactive in physical activities in school
SN - 07303084
UR -
https://login.proxy.bib.uottawa.ca/login?url=http://search.ebscohost.com/login.aspx?direct=true&db=s3h&AN=23727850&site=ehost-live
ER -

TY - JOUR
ID - 4902
T1 - How Do Youth Sports Affect a Child's Daily Activity Level?
A1 - Siegel,Donald
Y1 - 2008/01//
KW - SportDiscus
KW - *PHYSICAL education
KW - *SPORTS instruction
KW - *SPORTS for children
KW - *PHYSICAL education for children
KW - *SPORTS
KW - *SPORTS -- Physiological aspects
KW - *PHYSICAL fitness
KW - *SPORTS sciences
KW - *SOCCER for children
KW - *BASKETBALL for children
KW - *FOOTBALL for children
KW - Evaluation
RP - NOT IN FILE
SP - 9
EP - 9
JA - JOPERD: The Journal of Physical Education, Recreation & Dance

VL - 79
IS - 1
N2 - The article presents information on the study done to determine the total moderate-to-vigorous physical activity (MVPA) that can be attributed to sports involvement in the youth. The article also assesses the contributions to MVPA from recess and physical education. The method of research is also disclosed along with the results. It is revealed that the researchers selected and observed a group of boys between the ages of six and 12 who participated in soccer, basketball, and football. It is pointed out that there is a need to consider the unique characteristics of the youth in regard to sports, physical education, and recess. The limitations of the study are also acknowledged by its proponents along with suggestions for future studies
SN - 07303084
UR -
https://login.proxy.bib.uottawa.ca/login?url=http://search.ebscohost.com/login.aspx?direct=true&db=s3h&AN=28452656&site=ehost-live
ER -

TY - JOUR
ID - 4903
T1 - Efekt pohybovAcho programu ve • -kolnA-m prost • TedA- na zmA-rnŽ>nA- vA«skytu dŽ>tskAc obezity: vA «sledky 4letAc longitudinA -lnA- studie. / The effect of physical activity program at school on reducing childhood obesity: A results of four-year longitudinal study
A1 - SigmundovA -,Dagmar
A1 - Sigmund,Erik
Y1 - 2012/08//
KW - SportDiscus
KW - *PHYSICAL activity
KW - *OBESITY in children
KW - *BODY weight
KW - *PEDOMETERS
KW - *SCHOOL recess breaks
KW - *OBESITY -- Prevention
KW - LONGITUDINAL method
KW - • -kolnA- dru • _ina
KW - • -kolnA- p • TestA -vky
KW - after-school nursery
KW - kroky
KW - Obesity
KW - obezita
KW - pedometer Yamax
KW - pedometr Yamax
KW - school recess periods
KW - steps
RP - NOT IN FILE
SP - 129
EP - 141
JA - Medicina Sportiva Bohemica et Slovaca
VL - 21
IS - 3
N2 - An apparent increase of childhood obesity along with observed low physical activity (PA), initiates searching for effective strategies and verification of programs enhancing an increase of PA in children and reduction of their excessive weight. School environment offers apt opportunities to carry out physical and nutrition intervention programs because children spend a significant part of the day at school. The main goal was to identify the effect of a two-year longitudinal school-based PA intervention program aimed at the reduction of obesity in children after two years since the program was finished. 84 girls and 92 boys aged 10-11 participated in the study who participated in the survey in 2006-2008 as 6-9 year-olds and were included in the intervention group (43 girls and 45 boys) and the control group (41 girls and 47 boys). PA was monitored using Yamax pedometer and obesity was estimated using the percentile graph of Body Mass Index of World Health Organization (WHO) for girls and boys aged 5-19. At the beginning of the intervention program, there were no

significant differences found between the proportion of obese girls and boys in the intervention and the control groups - (girls 6.9%x 7.3 %, boys 11.1 % x 6.4 %, intervention x control groups resp.). At the end of the intervention program, there was no occurrence of obesity in the intervention group whereas there were 21.9 % girls and 23.4 % boys obese in the control group. After two years since the intervention program finished, there was a significantly lower (p = 0.0124) occurrence of obesity (girls 2.3 %, boys 6.7 %) and higher (p < 0.0001) number of school steps (girls 3099, boys 3368) in the intervention group than in the control group (girls 17.1 %, boys 23.4 %; girls 1751, boys 1799). A long-term increase of PA performed at school environment can significantly enhance the prevention of childhood obesity. ABSTRACT FROM AUTHOR
SN - 12105481
UR -
https://login.proxy.bib.uottawa.ca/login?url=http://search.ebscohost.com/login.aspx?direct=true&db=s3h&AN=84298830&site=ehost-live
ER -

TY - JOUR
ID - 4904
T1 - THE IMPACT OF PHYSICAL ACTIVITY AND SPORTS PRACTICE IN SOME HEALTH RELATED PARAMETERS IN SCHOOL AGE STUDENTS FROM VILA REAL
A1 - Silva,AntA3nio JosAc
A1 - MourAœo-Carvalhal,Maria Isabel
A1 - de Castro Coelho,Eduarda Maria Rocha Teles
A1 - Alves LibA3rio,Magda Fernanda Requeijo
Y1 - 2008/05//
KW - SportDiscus
KW - *OBESITY
KW - *DRINKING of alcoholic beverages
KW - *SPORTS
KW - *PHYSICAL fitness
KW - *PHYSICAL education
KW - Drug Utilization
KW - Health Status Indicators
KW - Motor Activity
KW - Obesity
KW - Actividad Motora
KW - Indicadores de Salud
KW - obesidad
KW - Atividade motora
KW - Indicadores BA sicos de SaA §de
KW - Obesidade
RP - NOT IN FILE
SP - 151
EP - 157
JA - Fitness & Performance Journal (Online Edition)
VL - 7
IS - 3
N2 - Introduction: This study had two main purposes: i) to identify the incidence rate of physical activity practice and some other health related parameters (alcohol ingestion; obesity tobacco and drugs) in school age students from Via Real; ii) to verify the impact of physical activity and sports practice in these health related parameters. Materials and Methods: A sample of 376 school age students with age ranged from 16 to 20 years was used. To evaluate the physical activity practice and alcohol, tobacco and drugs abuse the Health Behavior in School-Aged Children inquiry was used. For the Quetlet Index the height and weight of each one of the students was evaluated. The cut of points proposed by Cole (2000) to determine overweight and obesity was used. The X2 was used in the comparison between the categorical variables and the Mann-Whitney test in the ordinal variables. Results: The all evaluated sample had practice in physical education discipline, and 34.6% of student had also extra school physical activity (clubs).The incidence rate of overweight and obesity in the sample was 11.2% and 2.1% respectively. In what concerns the illicit abuse ingestion 34.8% on inquired students had normal alcohol consumption, 20% tobacco consumption and 22% drugs consumption. Discussion: The results

pointed showed also significant statistical differences comparing the groups with and without extra physical activity outside the school yard. In beer consumption (p=0.004). These results are in accordance with previous results Matos (2003) which refer that young child who does not practice physical activity have higher alcohol consumption values. In what refers to the other variables in spite of non statistical significant, it was observed for more obesity, more alcohol and tobacco consumption in non active students. These results agreed with the specific consulted literature. ABSTRACT FROM AUTHOR
IntroducciA n: Este estudio tiene dos objetivos: i) verificar la incidencia de la prA¡ctica de actividad fA-sica y de algunos indicadores de salud (obesidad, consumo de alcohol, tabaco y drogas) en alumnos del 2Aø y 3Aø Ciclo de la EnseAñanza BA¡sica y Secundaria de Vila Real; ii) verificar el impacto de la prA¡ctica de actividad fA-sica y deportiva en estos indicadores de salud. Materiales y MActodos: La muestra fue constituida por 376 jA venes con edades comprendidas entre los 16 y los 20 aAños. Para evaluar la prA¡ctica de actividad fA-sica y lo uso de sustancias ilA-citas fue utilizado el cuestionario "Comportamiento y Salud en JA venes en Edad Escolar", adaptado del estudio europeo Health Behavior in School-Aged Children. Para el cA¡lculo del A_ndice de Masa Corporal, fue recogido el peso y la altura, siendo utilizados los valores de corte propuestos por Cole (2000) para definir sobrecarga u obesidad. En la comparaciA n de grupos se usA³ a X2 en las variables categA ricas y el test de Mann-Whitney en las ordinais. Resultados: La totalidad de la muestra participaba de las clases de educaciA n fA-sica escolar y 36,4% de los jA venes practicaban deporte extraescolar. La incidencia de sobrecarga ponderal y obesidad fue de 11,2% y 2,1%, respectivamente. Cuanto al consumo de sustancias ilA-citas, 34,8% de los inquiridos consumA-an alcohol, 20% de los alumnos fumaban y 22% afirmaron ya tener experto drogas. DiscusiA n: Los resultados demostraron haber diferencias significativas cuando se comparA³ jA venes que practicaban educaciA n fA-sica escolar y otra modalidad deportiva extraescolar en el consumo de cerveza (p=0.004), presentando, los primeros, consumos superiores. Estos resultados estA¡n de acuerdo con Matos (2003), indicando que los jA venes que no practican actividad fA-sica afirman, mA¡s frecuentemente, consumir alcohol. Relativamente a las otras variables dependientes, a pesar de las diferencias no sean estadA-sticamente significativas, se verifica una tendencia para los jA venes que sA lo practican educaciA n fA-sica escolar sean mA¡s obesos, consuman mA¡s vino y bebidas blancas. Estos resultados estA¡n en consonancia con la literatura de la especialidad. ABSTRACT FROM AUTHOR
SN - 15199088
UR -
https://login.proxy.bib.uottawa.ca/login?url=http://search.ebscohost.com/login.aspx?direct=true&db=s3h&AN=35447246&site=ehost-live
ER -

TY - JOUR
ID - 4906
T1 - Some Thoughts on Getting Youngsters Out of the Classroom
A1 - Simpson,Richard
Y1 - 2009/09//
KW - SportDiscus
KW - *OUTDOOR education
KW - FIELDWORK (Educational method)
KW - LISTER School (Herford,Germany)
KW - Educational Technology
KW - POCKET computers
KW - HERFORD (Germany)
KW - Germany
RP - NOT IN FILE
SP - 16
EP - 17
JA - Horizons (14620677)
IS - 47
N2 - The author describes a field study day that he conducted from Lister School in Herford, Germany. He worked with Advanced Skills Teacher (AST) Keith Bull who was investigating the use of Personal Digital Assistants (PDAs) with children from primary schools. The objectives of the activity include exploring the use of PDAs as useful communication tools and enabling children to acquire real experiences and learning about the world. The planning and preparation effort for the study is also cited
SN - 14620677

UR -
https://login.proxy.bib.uottawa.ca/login?url=http://search.ebscohost.com/login.aspx?direct=true&db=s3h&AN=44299795&site=ehost-live
ER -

TY - JOUR
ID - 4907
T1 - Recess Activities of the Week (RAW): Promoting Free Time Physical Activity to Combat Childhood Obesity
A1 - Sinclair,Christina D.
A1 - Stellino,Megan Babkes
A1 - Partridge,Julie A.
Y1 - 2008/05//
KW - SportDiscus
KW - *PHYSICAL fitness
KW - *OBESITY in children
KW - *PREVENTION
KW - *TEACHERS
KW - CENTERS for Disease Control & Prevention (U.S.)
KW - United States
RP - NOT IN FILE
SP - 21
EP - 24
JA - Strategies (08924562)
VL - 21
IS - 5
N2 - The article discusses free time physical activity to fight obesity in children. The Recess Activity of the Week (RAW) program is presented as an effective way to motivate children to join physical activities. Sample activities were developed to help teachers develop their own programs in school. The U.S. Centers for Disease Control and Prevention states that 18.8 percent of children between 6-11 years old are overweight
SN - 08924562
UR -
https://login.proxy.bib.uottawa.ca/login?url=http://search.ebscohost.com/login.aspx?direct=true&db=s3h&AN=32172297&site=ehost-live
ER -

TY - JOUR
ID - 4908
T1 - Hostile Press Measure of Fear of Failure and Its Relation to Child-Rearing Attitudes and Behavior Problems
A1 - Singh,Satvir
Y1 - 1992/06//
KW - SportDiscus
KW - *ATTITUDE (Psychology)
KW - *SOCIAL psychology
KW - PARENT & child
KW - Child Rearing
KW - INTERPERSONAL conflict
KW - Behavioral Assessment
RP - NOT IN FILE
SP - 397
EP - 399
JA - Journal of Social Psychology
VL - 132
IS - 3
N2 - This article assesses the usefulness of the Parent Attitude Research Instrument (PARI) as a means of distinguishing the maternal child-rearing attitudes in groups with high and low levels of fear of failure (FF). In

an achievement-oriented culture, a negative approach to achievement could lead to poor adjustment. The purpose of the study conducted by the author was to assess the usefulness of the PARI as a means of distinguishing the maternal child-rearing attitudes in groups with high and low levels of FF. The study showed that mothers of the highest FF scorers tended to show more marital conflict and more irritability than mothers of the lowest FF scorers. They believed more in avoidance of communication and suppression of sexuality. They exhibited more inconsideration of the husband, showed more ascendency, and fostered more dependency. On the other hand, mothers of the lowest FF scorers appeared to foster more martyrdom, show more interest in excluding outside influence, and were very permissive in approving the child's activity
SN - 00224545
UR -
https://login.proxy.bib.uottawa.ca/login?url=http://search.ebscohost.com/login.aspx?direct=true&db=s3h&AN=9209070092&site=ehost-live
ER -

TY - JOUR
ID - 4909
T1 - Camps and Tramps: Civilization, Culture and the Use of Leisure in Early Twentieth-Century Outdoor Adventure Series Books for Girls and Boys
A1 - Singleton,Ellen
Y1 - 2005///
KW - SportDiscus
KW - *OUTDOOR recreation
KW - *CAMPING
KW - *HIKING
KW - *POPULAR culture
KW - SOCIAL history
KW - CHILDREN'S literature
KW - FICTION
RP - NOT IN FILE
SP - 49
EP - 49
JA - Leisure/Loisir: Journal of the Canadian Association for Leisure Studies
VL - 29
IS - 1
CY - ;
N2 - Series books, although "by no means literary masterpieces" (Inness, 1997, p. 96), serve as windows onto the popular culture of the time. Vintage juvenile fiction that focuses on the "new" leisure activities of camping and hiking provides valuable insights into the construction of race, class, gender and sexuality at the turn of the century. Not just representations of the modern historical period, these books provide insights into the "ideological density" of the era and as such broaden and strengthen our understanding about the role of leisure in our lives. The focus of this paper is on the "ideological density" of both series and single-issue books for boys and girls featuring the leisure activities of camping and hiking. [ABSTRACT FROM AUTHOR]
"En aucun cas" se trouve dans les livres de series "un chef-d'oeuvre litteraire" (Inness, 1997, P. 96), mais ils permettent d'obtenir de l'information aupres de la culture pop de cette periode. La fiction juvenile typique qui se fixe aupres des "nouveaux" activites loisirs de camping et d'excursion offre des apercus precieux dans la construction de race, classe, sexe et sexualite a la tournure du siecle. Non seulement est-ce que ces livres offrent une representation de la periode historique moderne, mais aussi des apercus dans la "densite ideologique" de cette epoque et comme tel, elargissent et renforcent notre comprehension du role des loisirs dans nos vies. Cet article se fixe sur la "densite ideologique" des livres de series et de simple edition pour les garcons et filles qui incluent les activites loisirs de camping et d'excursion. [ABSTRACT FROM AUTOR]
SN - 14927713
UR -
https://login.proxy.bib.uottawa.ca/login?url=http://search.ebscohost.com/login.aspx?direct=true&db=s3h&AN=SPHS-975514&site=ehost-live;http://articles.sirc.ca/search.cfm?id=S-975514
L2 - http://articles.sirc.ca/search.cfm?id=S-975514
ER -

TY - JOUR
ID - 4915
T1 - Physical activity levels of preadolescent children in England
A1 - Sleap,M.
A1 - Warburton,P.
Y1 - 1994///Summer
KW - SportDiscus
KW - *PHYSICAL fitness
KW - *PHYSICAL education & training
KW - *STRESS (Physiology)
KW - Surveys
KW - BOYS
KW - girls
KW - age
KW - Schools
KW - England
KW - Child
KW - SEX FACTOR
KW - EXTRACURRICULAR
RP - NOT IN FILE
SP - 2
EP - 6
JA - Research Supplement: British Journal of Physical Education
IS - 14
CY - ;
N2 - Continuous observation of 34 girls and 30 boys aged between 5 and 10 years was undertaken as part of an ongoing investigation of children's activity levels. Observations were carried out during school break times, lunch times and physical education lessons and during free time outside of school. Children engaged in moderate to vigorous physical activity (MVPA) for 28 percent of total time. Twenty four children (37.5 percent) recorded at least one sustained 20-min period of MVPA and 31 children (48 percent) recorded at least one 15-min period of sustained MVPA. Sixty four physical education lessons were observed. During these lessons 28 children (44 percent) participated in MVPA for a sustained 5-min period and 10 children (16 percent) engaged in MVPA for a sustained 10-min period. There is evidence that some preadolescents are engaging in sustained MVPA. Whether the findings could be considered satisfactory or otherwise, in terms of heart health, depends on the length of sustained activity deemed appropriate for preadolescents. There are also many young children who take part in minimal physical activity and therefore widespread efforts to promote physical activity among children need to be maintained
UR -
https://login.proxy.bib.uottawa.ca/login?url=http://search.ebscohost.com/login.aspx?direct=true&db=s3h&AN=SPH405743&site=ehost-live;http://articles.sirc.ca/search.cfm?id=405743
L2 - http://articles.sirc.ca/search.cfm?id=405743
ER -

TY - JOUR
ID - 4917
T1 - Multiple determinants of parenting: predicting individual differences in maternal parenting behavior with toddlers
A1 - Smith,C.L.
Y1 - 2010/01//
N1 - Accession Number: 2010551598. Language: English. Entry Date: 20100402. Revision Date: 20120309. Publication Type: journal article; research; tables/charts. Journal Subset: Biomedical; Blind Peer Reviewed; Editorial Board Reviewed; Expert Peer Reviewed; Online/Print; Peer Reviewed; USA. Special Interest: Pediatric Care. Instrumentation: Big Five Personality Inventory; Social Support Measure; Parenting Stress Index-Short Form; Early Parenting Coding System (EPCS). Grant Information: Virginia Tech ASPIRES (A Support Program for Innovative Research Strategies) Award. No. of Refs: 30 ref. NLM UID: 101093743
KW - CINAHL
KW - Maternal Behavior

KW - Mother-Child Relations
KW - Mothers -- Psychosocial Factors
KW - Parenting
KW - Child,Preschool
KW - Clinical Assessment Tools
KW - Coding
KW - Coefficient Alpha
KW - Descriptive Statistics
KW - Female
KW - Funding Source
KW - Human
KW - Male
KW - Personality
KW - Questionnaires
KW - Regression
KW - Scales
RP - NOT IN FILE
SP - 1
EP - 17
JA - Parenting: Science & Practice
VL - 10
IS - 1
N2 - Objective. A comprehensive understanding of individual differences in maternal parenting behaviors cannot be realized without consideration of multiple determinants of parenting within the same study. The goal of the present study was to examine the association of 3 categories of determinants of parenting (i.e., parent personality, contextual sources of stress and support, and child effects) to individual differences in maternal parenting behaviors. Design. Supportive and controlling parenting and child social responsiveness were observed during 2 free-play sessions when toddlers (N = 140) were 30 to 36 months old. Mothers completed questionnaires measuring personality and contextual sources of stress and support. Results. More maternal supportive behaviors were predicted by higher levels of child social responsiveness. More maternal control was associated with lower socioeconomic status, more work outside of the home, more extraversion, and more neuroticism. Conclusions. Because the determinants of parenting that predicted maternal controlling behaviors were different from those that predicted supportive parenting behaviors, examining how multiple determinants of parenting relate to different aspects of parenting behavior is necessary to more fully understand individual difference in parenting behaviors
SN - 1529-5192
AD - Department of Human Development, Virginia Polytechnic Institute and State University, Department of Human Development, Blacksburg, VA, USA
UR - http://search.ebscohost.com/login.aspx?direct=true&db=cin20&AN=2010551598&site=ehost-live
ER -

TY - JOUR
ID - 4918
T1 - Barriers to Accessing Safe Motherhood & Reproductive Health Services: The Situation of Women With Disabilities in Lusaka, Zambia
A1 - Smith,E.
A1 - Murray,S.F.
A1 - Yousafzai,A.K.
A1 - Kasonka,L.
Y1 - 2004/01/21/
KW - SportDiscus
KW - *WOMEN'S health services
KW - *WOMEN with disabilities
KW - *MEDICAL care
KW - LUSAKA (Zambia)
KW - Zambia
RP - NOT IN FILE

SP - 121
EP - 127
JF - Disability & Rehabilitation
JA - Disabil Rehabil
VL - 26
IS - 2
N2 - Purpose : To ascertain how well health services in Lusaka, Zambia currently meet the safe motherhood and reproductive health care needs of women who have physical impairment leading to disability. Methods : A qualitative study was conducted in Lusaka, Zambia. In-depth tape-recorded interviews were conducted with 24 purposively selected women with disabilities and with 25 safe motherhood/reproductive public sector health service providers. Qualitative analysis was conducted using NVivo software. Results : Women with disabilities encounter various social, attitudinal and physical barriers to accessing safe motherhood and reproductive health (RH) services in this particular setting. The strong desire for children and affection can increase vulnerability to sexual exploitation. At the same time, a generalized assumption among reproductive health service providers that women with disabilities will not be sexually active, and not require RH services, leads to increased vulnerability to sexually transmitted infection including HIV. Once pregnant, traditional beliefs about transmission of disabilities can create barriers to integration in ante-natal clinics. Nurse-midwives' fear of delivery complications in women with physical impairments can also result in routine over-referral to a tertiary maternity facility which is outside the locality and harder for women with mobility limitations to get to. Conclusion : Greater understanding of the influences underpinning societal attitudes towards sexuality and disability in this setting, and more extensive communication between health care staff and women with disabilities would facilitate positive action towards improving safe motherhood and reproductive health services for women with disabilities. ABSTRACT FROM AUTHOR
SN - 09638288
UR -
https://login.proxy.bib.uottawa.ca/login?url=http://search.ebscohost.com/login.aspx?direct=true&db=s3h&AN=11650392&site=ehost-live
ER -

TY - JOUR
ID - 4919
T1 - Assessment of Physical Activity Levels of 3rd and 4th Grade Children Using Pedometers during Physical Education Class
A1 - Smith,John
A1 - Nichols,David
A1 - Biggerstaff,Kyle
A1 - DiMarco,Nancy
Y1 - 2009///Spring
KW - SportDiscus
KW - *CHILDREN -- Health
KW - *PHYSICAL education
KW - *PHYSICAL fitness testing
KW - *PEDOMETERS
KW - *PHYSICAL activity
KW - Research
KW - Analysis of Variance
RP - NOT IN FILE
SP - 73
EP - 79
JA - ICHPER -- SD Journal of Research in Health, Physical Education, Recreation, Sport & Dance
VL - 4
IS - 1
N2 - The article reports on research which was conducted to to determine the amount of physical activity in which children engage during physical education classes and factors that mediate their participation. Third and 4th grade students wore pedometers during each 30-minute physical education class for one school year. Researchers found that third and fourth grade boys took significantly more steps than third and fourth grade girls, and those involved in youth sports accumulated more steps during a 30-minute physical education class

compared to those who were not involved in youth sports. They also found that steps accumulated during classes held outside were significantly greater compared to those accumulated during classes held inside, and steps accumulated on free (recess) days were significantly greater compared to those accumulated on structured days. They concluded that their results highlight the need for recognition of the contexts of gender and youth sport participation when planning and implementing an appropriate physical education experience for children
SN - 19304595
UR -
https://login.proxy.bib.uottawa.ca/login?url=http://search.ebscohost.com/login.aspx?direct=true&db=s3h&AN=45123835&site=ehost-live
ER -

TY - JOUR
ID - 4920
T1 - Pulled in All the Right Directions at Camp
A1 - Smith,Peg
Y1 - 2005/09//
KW - SportDiscus
KW - *CHILDREN
KW - *AMUSEMENTS
KW - *OUTDOOR recreation
KW - *OUTDOOR life
KW - MUIR,John,1838-1914
RP - NOT IN FILE
SP - 1
JA - Camping Magazine
VL - 78
IS - 5
N2 - Reveals that over the last twenty years there has been a 25 percent decline in the time children spend playing and a 50 percent decline in time spent in unstructured outdoor activities, according to William Doherty, a University of Minnesota researcher. Findings from independently conducted research from the American Camp Association and supported by Lilly Endowment Inc.; Remarks from naturalist John Muir about outdoor life
SN - 07404131
UR -
https://login.proxy.bib.uottawa.ca/login?url=http://search.ebscohost.com/login.aspx?direct=true&db=s3h&AN=19960699&site=ehost-live
ER -

TY - JOUR
ID - 4921
T1 - CHAPTER 8: Development of Recreation and Leisure Through Art Interventions
A1 - Smitheman-Brown,Valerie
Y1 - 2003/01//
KW - SportDiscus
KW - *RECREATION
KW - *LEISURE
KW - *CHILDREN with disabilities
KW - *PLAY
KW - Art
KW - COGNITION in children
KW - Drawing
KW - United States
RP - NOT IN FILE
SP - 61
EP - 69
JA - Implementing Recreation & Leisure Opportunities for Infants & Toddlers with Disabilities

N2 - Presents a chapter of "Implementing Recreation and Leisure Opportunities for Infants and Toddlers With Disabilities" on the development of recreation and leisure through art interventions in the U.S. Assumption of cognitive development theorist Jean Piaget on a link between the development of the rational thinking of a child with the outside world; Inclusion of solitary and associative plays among the types of play; Provision of an alternative method of communication and self-expression to children through the encouragement of creative art and drawing
SN - 9781571673848
UR -
https://login.proxy.bib.uottawa.ca/login?url=http://search.ebscohost.com/login.aspx?direct=true&db=s3h&AN=20083869&site=ehost-live
ER -

TY - JOUR
ID - 4922
T1 - Daytime light exposure and feelings of vitality: Results of a field study during regular weekdays
A1 - Smolders,K.C.H.J.
A1 - de Kort,Y.A.W.
A1 - van den Berg,S.M.
Y1 - 2013///
KW - Reviewer Nominated
RP - NOT IN FILE
JF - Journal of Environmental Psychology
PB - Elsevier
SN - 0272-4944
ER -

TY - JOUR
ID - 4923
T1 - Leadership research in youth sports
A1 - Smoll,F.L.
A1 - Smith,R.E.
Y1 - 1984///
KW - SportDiscus
KW - *SPORTS
KW - *COACHING (Athletics)
KW - teenagers
KW - Research
KW - EXAMPLE
KW - Evaluation
KW - Child
KW - Leadership
KW - COACHING BEHAVIOUR ASSESSMENT SYSTEM
KW - COACH EFFECTIVENESS TRAINING
KW - Certification
RP - NOT IN FILE
CY - ;
N2 - Presents a theoretical model and rationale for studying leadership behaviors in youth sports. Considers the basic components of a mediational model of coach-player interactions and examines some preliminary research which emphasizes a behavioral assessment approach to measuring coach behavior in naturalistic settings. Reviews empirically derived coaching guidelines and makes recommendations for their implementation. Describes the development and testing of a psychologically oriented training program for coaches
SN - 0931250595
UR -
https://login.proxy.bib.uottawa.ca/login?url=http://search.ebscohost.com/login.aspx?direct=true&db=s3h&AN=SPH154105&site=ehost-live;http://articles.sirc.ca/search.cfm?id=154105
L2 - http://articles.sirc.ca/search.cfm?id=154105
ER -

TY - JOUR
ID - 4925
T1 - Early Life Experiences and Adult Sports Participation
A1 - Sofranko,Andrew J.
A1 - Nolan,Michael F.
Y1 - 2009/03//
KW - SportDiscus
KW - *SPORTS participation
KW - *CHILDREN
KW - *HUNTING
KW - *FISHING
KW - adulthood
KW - Pennsylvania
KW - CHILDHOOD PARTICIPATION
KW - CHILDHOOD RESIDENCE
KW - INTERVENING VARIABLE
KW - OUTDOOR recreation
KW - Participation
KW - Socialization
RP - NOT IN FILE
SP - 425
EP - 437
JF - Journal of Leisure Research
VL - 41
IS - 3
N2 - This paper examines the influence of some childhood background factors on adult levels of participation in hunting and fishing activities. The study, based on data collected from hunters and fishermen in Pennsylvania, offers some insights into the effect early experiences with two types of outdoor recreational activities have on participation in adult life. The data show that residence in youth and source of introduction to hunting and fishing, although significantly related to extent of participation as a youth, are not related to higher levels of adult participation. However, for hunters, and to a limited extent for fishermen, frequency of participation as a youth is positively related to current level of participation. It is suggested that intervening factors occurring in the time interval between youth and adulthood serve to reduce or eliminate the influence of childhood residence and source of introduction, and that at different points of the life and work cycles certain variables recede and others emerge as influences on participation. Work-related influences on level of adult participation are presented as one such "intervening" variable. ABSTRACT FROM AUTHOR
SN - 00222216
UR -
https://login.proxy.bib.uottawa.ca/login?url=http://search.ebscohost.com/login.aspx?direct=true&db=s3h&AN=44089954&site=ehost-live
ER -

TY - JOUR
ID - 4926
T1 - Play patterns and exposure to risks: indications for childhood injuries. (Poster Session)
A1 - Soori,H.
Y1 - 2002///
KW - SportDiscus
KW - *PLAY
KW - *WOUNDS & injuries
KW - *OUTDOOR life
KW - Risk
KW - Parents
KW - Perception
KW - Questionnaires
KW - Case Studies

KW - Iran
KW - Child
RP - NOT IN FILE
CY - ;
UR -
https://login.proxy.bib.uottawa.ca/login?url=http://search.ebscohost.com/login.aspx?direct=true&db=s3h&AN=SPHS-849754&site=ehost-live;http://articles.sirc.ca/search.cfm?id=S-849754;http://www.dundee.ac.uk/iea/
L2 - http://articles.sirc.ca/search.cfm?id=S-849754
ER -

TY - JOUR
ID - 4927
T1 - Realizacja zajec wychowania fizycznego w klasach nauczania poczatkowego szkol podstawowych na przykladzie wojewodztwa wloclawskiego. / Realisation of physical education in the youngest children of primary schools at the Wloclawek province
A1 - Sosnowska,S.
A1 - Kostka,T.
Y1 - 2002/02//
KW - SportDiscus
KW - *PHYSICAL education & training
KW - *SPORTS facilities
KW - *GYMNASIUMS
KW - *PLAYGROUNDS
KW - Elementary Schools
KW - Questionnaires
KW - Evaluation
KW - Statistics
KW - Poland
KW - Child
RP - NOT IN FILE
SP - 69
EP - 74
JA - Medycyna Sportowa
VL - 18
IS - 2(127)
CY - ;
N2 - Conditions and realisation of physical education in youngest children of primary schools at the Wloclawek province (202 schools) were analysed. Only 19,31% of schools had an appropriate gymnasium, sports area and recreation area. 72 schools (35,64%) had a full-size gymnasium, 69,31% of schools had a full-size sports area, and 57,43% of schools had a full-size recreation area of surface at least 450 m2. Overall weekly physical exercises organised by schools were 3 hours or more in 99.5% of schools, 4 hours at least in 73.77% of schools, and 5 hours at least in 18.32% of schools. The majority of schools realised program of 4-5 hours or more per week (55,45%). Equipment with recreation area was a significant determinant of number of hours of physical exercises provided by schools (chi2=6,76; p=0,03), while equipment with gymnasium (chi2=2,31; p=0,52) or sports area (chi2=1,33; p=0,51) were not
SN - 1232406X
UR -
https://login.proxy.bib.uottawa.ca/login?url=http://search.ebscohost.com/login.aspx?direct=true&db=s3h&AN=SPHS-877181&site=ehost-live;http://www.medsport.pl
ER -

TY - JOUR
ID - 4928
T1 - SAFETY Sans SACRIFICES
A1 - Spencer,Anne Marie
Y1 - 2008/08//
KW - SportDiscus

KW - *PLAYGROUNDS
KW - *LIFE skills
KW - DESIGN & construction
KW - PLAY environments -- Safety measures
KW - RISK-taking (Psychology) in children
KW - SELF-confidence in children
KW - SELF-esteem in children
RP - NOT IN FILE
SP - 54
EP - 57
JA - Parks & Recreation
VL - 43
IS - 8
N2 - The article discusses the balance between safety and enjoyability in playground design. The need for a certain amount of risk in children's play is discussed, citing children's disinterest in parks that are designed too conservatively. Benefits of healthy risk-taking are discussed, including self-confidence, prevention of fragility, and healthy development of life skills such as self-esteem
SN - 00312215
UR -
https://login.proxy.bib.uottawa.ca/login?url=http://search.ebscohost.com/login.aspx?direct=true&db=s3h&AN=33653696&site=ehost-live
ER -

TY - JOUR
ID - 4929
T1 - Managing children's interactions with cultural heritage places
A1 - Spennemann,D.H.R.
A1 - Taffe,R.
Y1 - 1998///Summer
KW - SportDiscus
KW - tourism
KW - HISTORIC sites
KW - Learning
KW - Pleasure
KW - HERITAGE
KW - Needs
KW - Child
RP - NOT IN FILE
SP - 73
EP - 87
JA - Journal of Park & Recreation Administration
VL - 16
IS - 2
CY - ;
N2 - The impact of children on cultural heritage places is a phenomenon dreaded by parks managers. Children's means of exploring and comprehending the world around them is much more direct and less abstract than that of adults. By understanding children's learning behavior, the processes leading to a detrimental impact cannot only be avoided, but moreover be turned into a positive experience for children and parents alike. Parks management can be informed by experiences garnered in the fields of museum interpretation and education in zoological and marine parks. The mere provision of testual and pictorial information is limiting the learning experience of children, as well as that of many adults. Showcased specimens of artifacts are illustrative to an adult audience; they are "remote" for children. Management needs to provide for controlled environments in which children can satisfy their needs of examining artifacts and sensitive items on their terms through touch tables and other interactive displays, forcing the adoption of different postures, etc. If well done, this satisfies children's need for physical exploration and reduces impact in the open space setting. For geographically remote parks, there is a need to provide an outlet for children to "let off steam" after a long trip in the car. The establishment of an adventure playground would be desirable, preferably designed with thematic reference to the specific park

visited. Park infrastructure must address children's learning and energy dissipation needs through the provision of interpretation centers and (outside) activity areas appropriate to children of various ages. This needs to be coupled with information and guidelines for parents visiting parks, including strategies to control and channel children's actions and energy through (i) careful planning of the trip and the approaches to the park, (ii) planning of activities at arrival, and (iii) guidelines for appropriate parental control during the visit. Parents will have a better visitor experience if their children are engaged in a captivating learning experience. Such children will be less demanding of parental control and their parents will have more time to follow their own interests. It follows that such parents will also be more responsive to issues of nature and cultural conservation
SN - 07351968
UR -
https://login.proxy.bib.uottawa.ca/login?url=http://search.ebscohost.com/login.aspx?direct=true&db=s3h&AN=SPH480912&site=ehost-live;http://articles.sirc.ca/search.cfm?id=480912
L2 - http://articles.sirc.ca/search.cfm?id=480912
ER -

TY - JOUR
ID - 4935
T1 - Voices in the playground: A qualitative exploration of the barriers and facilitators of lunchtime play
A1 - Stanley,Rebecca M.
A1 - Boshoff,Kobie
A1 - Dollman,James
Y1 - 2012/01//
KW - SportDiscus
KW - *PLAYGROUNDS
KW - *GAMES
KW - *CHILDREN
KW - Voice
KW - Qualitative Research
KW - INTERVAL measurement
KW - FACILITATED learning
KW - Attitudes
RP - NOT IN FILE
SP - 44
EP - 51
JF - Journal of Science & Medicine in Sport
JA - J Sci Med Sport
VL - 15
IS - 1
N2 - Abstract: Objectives: To explore children"s perceptions of the factors influencing their engagement in physical activity during the ƒ_ocriticalƒ__ lunchtime period, using a socialƒ_"ecological framework. Design: This study was an in-depth descriptive qualitative design. Methods: Fifty-four South Australian children aged 10ƒ_"13 years participated in same-gender focus groups. Transcripts, field notes and activity documents were analysed using content analysis. Using an inductive thematic approach, data were coded and categorised into perceived barriers and facilitators according to a socialƒ_"ecological model. Results: Children identified a range of environmental, social and intrapersonal barriers and facilitators. Bullying/teasing, the school uniform and school rules were exposed as explicit barriers to lunchtime play. Other important barriers included lack of access to, and poor suitability of, space, lack of access to programs/facilities and equipment, and lack of peer and teacher support. Perceived facilitators of lunchtime physical activity centred on access to equipment, enjoyment, motivation to improve skills, and peer support and acceptance. The freedom to make up or modify rules for games was also perceived to be a facilitator of lunchtime play. Conclusions: Communicating with children has been an effective approach in uncovering perceived barriers and facilitators to lunchtime play that may not have been previously considered in the quantitative correlate literature. Lunchtime interventions targeting children"s physical activity should focus on addressing the barriers perceived to be important to lunchtime play. Copyright &y& Elsevier
SN - 14402440

UR -
https://login.proxy.bib.uottawa.ca/login?url=http://search.ebscohost.com/login.aspx?direct=true&db=s3h&AN=70044396&site=ehost-live
ER -

TY - JOUR
ID - 4937
T1 - Family adherence as a predictor of child outcome in an intervention for pediatric obesity: different outcomes for self-report and objective measures
A1 - Steele,M.M.
A1 - Steele,R.G.
A1 - Hunter,H.L.
Y1 - 2009/01//
N1 - Accession Number: 2010171502. Language: English. Entry Date: 20090724. Revision Date: 20131213. Publication Type: journal article; research; tables/charts. Journal Subset: Biomedical; Blind Peer Reviewed; Editorial Board Reviewed; Expert Peer Reviewed; Online/Print; Peer Reviewed; USA. Special Interest: Nutrition; Pediatric Care. No. of Refs: 25 ref. NLM UID: 8101257
KW - CINAHL
KW - Family Centered Care
KW - Outcomes (Health Care)
KW - Patient Compliance
KW - Self Report
KW - Pediatric Obesity -- Therapy
KW - Behavioral Changes
KW - Body Mass Index
KW - Child
KW - Comparative Studies
KW - Correlational Studies
KW - Descriptive Statistics
KW - Diet
KW - Exercise
KW - Female
KW - Male
KW - multiple regression
KW - nutrition education
KW - Human
RP - NOT IN FILE
SP - 64
EP - 75
JA - Children's Health Care
VL - 38
IS - 1
N2 - This study examined the relations between specific adherence variables and child outcomes among a sample of 60 families participating in a family-based pediatric obesity program. Adherence was measured using both objective and self-report methods. Family adherence to the treatment protocol significantly predicted child outcomes. However, parent self-report measures of adherence were better predictors of child outcome than objective measures. The importance of including parent self-report measures of adherence for behaviors outside of the treatment setting was demonstrated in this study. Pediatric obesity treatment should emphasize the importance of modifying parent behaviors as a means for improving child outcomes
SN - 0273-9615
AD - Department of Psychology, Washington State University, Pullman, WA
UR - http://search.ebscohost.com/login.aspx?direct=true&db=cin20&AN=2010171502&site=ehost-live
ER -

TY - JOUR
ID - 4938
T1 - SOFT MARKET

A1 - Steinbach,P.A.U.L.
Y1 - 2009/05//
KW - SportDiscus
KW - *PLAYGROUNDS
KW - *PARKS
KW - *CHILDREN -- Wounds & injuries
KW - *PREVENTION
KW - SAFETY measures
KW - SURFACES (Technology)
KW - DETROIT (Mich.).Dept.of Recreation
KW - DETROIT (Mich.)
KW - Michigan
RP - NOT IN FILE
SP - 36
EP - 42
JA - Athletic Business
VL - 33
IS - 5
N2 - The article focuses on safety surfacing used in playgrounds in Detroit, Michigan. Tim Karl, chief of landscape architecture, claims that the city has continued to renovate its parks. The director and managers of the Detroit Recreation Department, also claim that the safety surfacing in their parks were better than American Society for Testing and Materials (ASTM) requirements. It emphasizes the importance of safety surfacing to the prevention of injuries in playgrounds
SN - 0747315X
UR -
https://login.proxy.bib.uottawa.ca/login?url=http://search.ebscohost.com/login.aspx?direct=true&db=s3h&AN=41236279&site=ehost-live
ER -

TY - JOUR
ID - 4940
T1 - Intrinsically Motivated, Free-Time Physical Activity
A1 - Stellino,Megan Babkes
A1 - Sinclair,Christina D.
Y1 - 2008/04//
KW - SportDiscus
KW - *PHYSICAL fitness
KW - *SCHOOL children
KW - *SCHOOL recess breaks
KW - *OUTDOOR recreation
KW - *PHYSICAL education
KW - *OUTDOOR games
KW - Elementary Schools
KW - MALES -- Behavior
KW - Elementary Education
RP - NOT IN FILE
SP - 37
EP - 40
JA - JOPERD: The Journal of Physical Education, Recreation & Dance
VL - 79
IS - 4
N2 - The article focuses on the lack of physical activity among children during recess. According to the findings from a research conducted by M. L. Babkes and C. D. Sinclair, elementary school children appear to rarely reach very active intensity levels during recess while fifth-grade children took the most steps and were most physically active than any other children in elementary. The research also reveals that male children were more physically active in terms of duration, intensity and frequency during recess as compared to female counterparts

SN - 07303084
UR -
https://login.proxy.bib.uottawa.ca/login?url=http://search.ebscohost.com/login.aspx?direct=true&db=s3h&AN=31800699&site=ehost-live
ER -

TY - JOUR
ID - 4942
T1 - Leave No Child Inside: Taking Physical Education Out of the Gym
A1 - Stenger-Ramsey,Tammie L.
A1 - Curl,Lora
Y1 - 2010///Spring
KW - SportDiscus
KW - *ATTENTION-deficit hyperactivity disorder
KW - *MENTAL depression
KW - *OBESITY in children
KW - *PHYSICAL education
KW - Nature
KW - LOUV,Richard,1949-
RP - NOT IN FILE
SP - 15
EP - 17
JA - Kentucky Newsletter for Health, Physical Education, Recreation & Dance
VL - 47
IS - 1
N2 - As our society becomes more technologically advanced, people are spending more time indoors in artificial environments and less time in natural settings. Richard Louv coined the phrase "Nature Deficit Disorder" to describe some of the social, psychological, and ecological struggles that have arisen as members of society become less in tune with the natural world. Physical Education teachers are in a unique position to get children outside into natural environments while still meeting necessary educational requirements. This article will also address a case study of a child with ADHD. Nature Deficit Disorder (NDD) is a term developed by Richard Louv, author of "Last Child in the Woods: Saving our children from Nature Deficit Disorder," which refers to the recent trend of children spending less time outdoors in natural environments. Louv suggested that the detachment from nature has contributed to a wide range of behavioral problems including depression, childhood obesity and an increase in the symptoms of Attention Deficit Hyperactivity Disorder (ADHD). His research has shown that children who get regular, daily doses of green spaces tend to perform better in school, are more physically active, and are more content (Louv, 2005). Physical Education by its nature gets children active and helps them burn excess energy. When Physical Education moves outside, children benefit even more. ABSTRACT FROM AUTHOR
UR -
https://login.proxy.bib.uottawa.ca/login?url=http://search.ebscohost.com/login.aspx?direct=true&db=s3h&AN=53011931&site=ehost-live
ER -

TY - JOUR
ID - 4943
T1 - Outdoor
A1 - Stidder,Gary
Y1 - 2007/09//
KW - SportDiscus
KW - *OUTDOOR recreation for children
KW - *PHYSICAL education
KW - *ACTIVITY programs in education
KW - Secondary Education
KW - STUDENT activities
KW - CURRICULA (Courses of study)
RP - NOT IN FILE

SP - 34
EP - 40
JA - Physical Education Matters
VL - 2
IS - 3
N2 - This article discusses a study which examined the extent to which secondary schools include the teaching of on-site outdoor and adventurous activities (OAA) within the physical education curriculum. The study also described several difficulties and challenges faced by school staff with regards to the inclusion of OAA in schools. Several factors that have contributed and restricted school staff to offer OAA in schools include cost, expertise, facilities, time and training
SN - 17510988
UR -
https://login.proxy.bib.uottawa.ca/login?url=http://search.ebscohost.com/login.aspx?direct=true&db=s3h&AN=27467554&site=ehost-live
ER -

TY - JOUR
ID - 4944
T1 - School-based Physical Activity Programs
A1 - Stiehl,Jim
A1 - Galvan,Christine
Y1 - 2005/11//
KW - SportDiscus
KW - *YOUTH
KW - After School Programs
KW - CURRICULA (Courses of study)
KW - Middle School Education
KW - Middle Schools
KW - YOUTH development
RP - NOT IN FILE
SP - 25
EP - 31
JA - JOPERD: The Journal of Physical Education, Recreation & Dance
VL - 76
IS - 9
N2 - The article highlights on an after-school program in a middle school and youth development strategies that can also be embedded in the physical education curriculum and can serve a wide age range. It typifies a number of programs that use physical activity to tap children's developmental potential Project Effort and Project Lead. Today's youths face many obstacles as they move toward adulthood. Regardless of their life circumstances, all young people need ample choices for supervised growth!promoting activities both inside and outside of school. The goal is for youths to become productively independent and to be able to survive and thrive in a society that needs their contribution. Many youths, however, are learning negative, habit-forming, life-altering behaviors that will inhibit them from finding success in the adult society and that will lead to a pattern of personal failure throughout life. The after-school leadership program is designed to provide positive growth experiences to middle school youngsters. Participants in this program include students who are recommended by school staff because they are at or below the academic average of the school. Physical activities, especially adventure activities, constitute the primary means for achieving program goals
SN - 07303084
UR -
https://login.proxy.bib.uottawa.ca/login?url=http://search.ebscohost.com/login.aspx?direct=true&db=s3h&AN=18926339&site=ehost-live
ER -

TY - JOUR
ID - 4945
T1 - Recreation Participation Patterns and Physical Activity among Latino Visitors to Three Urban Outdoor Recreation Environments

A1 - Stodolska,Monika
A1 - Shinew,Kimberly J.
A1 - Li,Monica Z.
Y1 - 2010///Summer
KW - SportDiscus
KW - *OUTDOOR recreation
KW - *OUTDOOR life
KW - COMMUNITY centers
KW - CITIES & towns
KW - ECOLOGICAL modernization
KW - ENVIRONMENTAL sociology
KW - Latinos
KW - natural environments
KW - parks
KW - Physical Activity
KW - Recreation participation
RP - NOT IN FILE
SP - 36
EP - 56
JA - Journal of Park & Recreation Administration
VL - 28
IS - 2
N2 - The goals of this study were to compare visitation patterns and recreation participation among Latino men and women at three different urban outdoor recreation environments and to examine the types of physical activities that took place at each of the three environments. Ecological model was employed as a theoretical framework in this study. Data were collected with the use of questionnaire surveys distributed to 917 visitors at 1) two community parks in Elgin, Illinois, 2) a section of the Lincoln Park in Chicago that contains an extensive trail system, and 3) an outdoor sports complex in Aurora, Illinois. The results showed significant variations in the recreation use of these three environments and, in particular, in their suitability to foster physically active leisure among Latino men and women. Walking was more common at the community parks and trails, whereas soccer was more popular at the sports complex. Trails were also conducive to jogging/running and bicycling. Latino users of all environments showed significant gender variations in recreation participation, with women more likely to be involved in walking and all passive activities other than barbecuing/picnicking, and men more likely to be involved in all active pastimes (other than walking). The implications of the study's findings are explored. Park managers are encouraged to consider the family-focused nature of the Latino culture when programming and/or planning for this particular subgroup of the population. Providing programs and services that allow Latino parents to be physically active with their children should be encouraged. Examples include family field trips, family soccer games, dances, and mother-children walks. Informational campaigns that clearly explain the health benefits associated with physical activity (for both adults and children) are also encouraged. In summary, organizing family-oriented events that promote various physical activities in parks, trails, and sports complexes may be an effective strategy for improving the activity levels of Latino men, women, and children. ABSTRACT FROM PUBLISHER
SN - 07351968
UR -
https://login.proxy.bib.uottawa.ca/login?url=http://search.ebscohost.com/login.aspx?direct=true&db=s3h&AN=51445216&site=ehost-live
ER -

TY - JOUR
ID - 4946
T1 - International Play Association, USA
A1 - Stork,Steve
Y1 - 2005/03//
KW - SportDiscus
KW - *PLAY
KW - *SCHOOL recess breaks
KW - ASSOCIATIONS,institutions,etc.

KW - UNITED Nations
KW - CHILDREN'S rights
KW - WEBSITES
KW - United States
RP - NOT IN FILE
SP - 35
EP - 35
JA - Teaching Elementary Physical Education
VL - 16
IS - 2
N2 - The article focuses on International Play Association that aims to protect, preserve, and promote play as a fundamental right for all humans. Legislative and advocacy activities of IPA/USA are based on the United Nations' Declaration of the Rights of the Child. IPA/USA rationalizes the importance and relevance of play by noting that children are the foundation of the world's future. Play helps children develop physically, mentally, emotionally, and socially. IPA/USA is a strong advocate for recess. For more information about membership and the many facets of IPA/USA, one can see their web page at http://www.ipausa.org/
SN - 10454853
UR -
https://login.proxy.bib.uottawa.ca/login?url=http://search.ebscohost.com/login.aspx?direct=true&db=s3h&AN=16330818&site=ehost-live
ER -

TY - JOUR
ID - 4949
T1 - Chapter 13: Promoting children's physical activity in primary school: an intervention study using playground markings
A1 - Stratton,Gareth
Y1 - 2002/03/14/
KW - SportDiscus
KW - *PHYSICAL fitness
KW - *SCHOOL children
KW - *PLAYGROUNDS
KW - *SPORTS facilities
KW - *HEART beat
KW - Research
KW - CHILDREN
KW - Exercise
KW - Heart Rate
KW - playground markings
KW - School playtime
RP - NOT IN FILE
SP - 159
EP - 167
JA - Advances in Sport, Leisure & Ergonomics
N2 - The physical activity levels of 47, 5- to 7-year-old children were assessed before and after a school playground was painted with fluorescent markings. Children's physical activity was measured using heart rate telemetry during three playtimes before and after the markings were laid down. Children in the experimental and control groups spent 27 and 29 min, respectively, in moderate to vigorous physical activity (MVPA) before the intervention, increasing to 45 and 36 min, respectively, during the intervention period. MVPA, vigorous physical activity (VPA) and mean heart rate remained relatively stable in the control group compared to respective increases of 10 and 5% of playtime and 6 beats min' in the experimental group during the intervention period. The ANCOVA analysis revealed significant interactions and main effects for the intervention for MVPA, VPA and mean heart rate. Conversely there were no main effect differences between groups. These results suggest that while playground markings had a significant and positive influence on children's physical activity, factors other than playground markings may also influence children's physically active play.
ABSTRACT FROM AUTHOR
SN - 9780415271257

UR -
https://login.proxy.bib.uottawa.ca/login?url=http://search.ebscohost.com/login.aspx?direct=true&db=s3h&AN=16735405&site=ehost-live
ER -

TY - JOUR
ID - 4954
T1 - Physical exercise through mountain hiking in highâ€ • risk suicide patients. A randomized crossover trial
A1 - Sturm,J.
A1 - Pl+Ýderl,M.
A1 - Fartacek,C.
A1 - Kralovec,K.
A1 - Neunh+¤userer,D.
A1 - Niederseer,D.
A1 - Hitzl,W.
A1 - Niebauer,J.
A1 - Schiepek,G.
A1 - Fartacek,R.
Y1 - 2012///
KW - Reviewer Nominated
RP - NOT IN FILE
SP - 467
EP - 475
JA - Acta Psychiatrica Scandinavica
VL - 126
IS - 6
PB - Wiley Online Library
SN - 1600-0447
ER -

TY - JOUR
ID - 4955
T1 - It's Miller Time
A1 - Sturtz,R.A.C.H.
Y1 - 2014/01//
KW - SportDiscus
KW - *VOLLEYBALL players
KW - MILLER,Bode,1977-
KW - BROTHERS
KW - love
RP - NOT IN FILE
SP - 62
EP - 67
JA - Outside
VL - 39
IS - 1
N2 - The article focuses the life of Bode Miller, a skier and his wife Morgan Beck, a volleyball player, who faced rough time in the year 2013. It informs that Miller lost his brother on April 7, 2013 which is one of the saddest parts of his life. It further informs that Miller and Beck also fought a battle for the custody of a child from his ex-girlfriend in 2013. It also mentions that despite of these odd things both of them love each other very much and understands each others feelings
SN - 02781433
UR -
https://login.proxy.bib.uottawa.ca/login?url=http://search.ebscohost.com/login.aspx?direct=true&db=s3h&AN=93329270&site=ehost-live
ER -

TY - JOUR
ID - 4956
T1 - The perceived impact of outdoor residential camp experience on the spirituality of youth
A1 - Sweatman,M.M.
A1 - Heintzman,P.
Y1 - 2004///
KW - SportDiscus
KW - *CAMPING
KW - *WILDERNESS survival
KW - teenagers
KW - Spirituality
KW - Ontario
RP - NOT IN FILE
SP - 23
EP - 31
JA - World Leisure & Recreation
VL - 46
IS - 1
CY - ;
N2 - The purpose of this study was to investigate the perceived impact of outdoor residential camp experience on the spirituality of youth. This research focused on 11 participants in month long leadership programs at two YMCA residential camps in Ontario, Canada. After conducting an in-depth interview at the beginning and end of each of the participant's leadership experience, it was found that the participants did feel camp had an affect on their spirituality. The camp setting played a role by providing a natural setting and an opportunity for the participants to get away from their everyday lives in an urban environment. In this environment, experiencing structured and unstructured time alone influenced their spirituality, by providing them with opportunities to reflect on their lives. Social experiences also had an impact on the participants' spirituality by allowing them to develop relationships through formal and informal activities. Finally, the majority of the participants expressed that the camp experience provided them with positive feelings that they associated with their spirituality. This study found that the four themes of the camp setting, alone time, social experiences, and positive feelings played an interrelated role in the participant's camp experience and spirituality
SN - 10261133
UR -
https://login.proxy.bib.uottawa.ca/login?url=http://search.ebscohost.com/login.aspx?direct=true&db=s3h&AN=SPHS-939624&site=ehost-live;http://articles.sirc.ca/search.cfm?id=S-939624;http://www.worldleisure.org
L2 - http://articles.sirc.ca/search.cfm?id=S-939624
ER -

TY - JOUR
ID - 4957
T1 - Patterns of Recurrent Injuries Among US High School Athletes, 2005-2008
A1 - Swenson,David M.
A1 - Yard,Ellen E.
A1 - Fields,Sarah K.
A1 - Comstock,R.Dawn
Y1 - 2009/08//
KW - SportDiscus
KW - *SPORTS injuries
KW - *ATHLETICS
KW - *ANATOMY
KW - *PHYSICAL education
KW - *PUBLIC health
KW - *STIFLE joint
KW - *MEDICAL personnel
KW - Allied Health Personnel
KW - PARAMEDICAL education
KW - Epidemiology

KW - high school
KW - injury
KW - recurrent injury
KW - Reinjury
KW - Sports
KW - surveillance
RP - NOT IN FILE
SP - 1586
EP - 1593
JF - American Journal of Sports Medicine
JA - Am J Sports Med
VL - 37
IS - 8
N2 - Background: High school sports participants sustain millions of injuries annually; many are recurrent injuries that can be more severe than new injuries. Hypothesis: Recurrent injury patterns differ from new injury patterns by sport and gender. Study Design: Descriptive epidemiology study. Methods: High school sports injury data for the 2005 through 2008 academic years were collected via High School Reporting Information Online (RIO) from a nationally representative sample of 100 US high schools. Results: From 2005 through 2008, certified athletic trainers reported 13 755 injuries during 5 627 921 athlete exposures (24.4 injuries per 10 000 athlete exposures). Recurrent injuries accounted for 10.5% of all injuries. Football players had the highest rate of recurrent injury (4.36 per 10 000 athlete exposures). Girls had higher rates of recurrent injuries than boys in soccer (injury rate ratio = 1.39; 95% confidence interval, 1.07-1.82). Recurrent injuries most often involved the ankle (28.3%), knee (16.8%), head/ face (12.1%), and shoulder (12.0%), and were most often ligament sprains (incomplete tears) (34.9%), muscle strains (incomplete tears) (13.3%), and concussions (11.6%). A greater proportion of recurrent injuries than new injuries resulted in the student choosing to end participation (recurrent = 2.4%, new = 0.7%). Recurrent shoulder injuries were more likely to require surgery than new shoulder injuries (injury proportion ratio = 4.51; 95% confidence interval, 2.82-7.20). Conclusion: Recurrent injury rates and patterns differed by sport. Because recurrent injuries can have severe consequences on an athlete's health and future sports participation, injury prevention must be a priority. Knowledge of injury patterns can drive targeted preventive efforts. ABSTRACT FROM AUTHOR
SN - 03635465
UR -
https://login.proxy.bib.uottawa.ca/login?url=http://search.ebscohost.com/login.aspx?direct=true&db=s3h&AN=44185099&site=ehost-live
ER -

TY - JOUR
ID - 4958
T1 - A Weighty Issue for Minorities
A1 - T.L.
Y1 - 2004/11/15/
KW - SportDiscus
KW - *OBESITY in children
KW - *OVERWEIGHT children
KW - *CHILDREN -- Health
KW - CHILDREN of minorities
KW - MEXICAN American children
KW - AFRICAN American children
RP - NOT IN FILE
SP - 88
EP - 88
JA - Sports Illustrated
VL - 101
IS - 19
N2 - The article discusses how obesity is even more pronounced among black and Mexican children than among whites. Obesity among all children in the U.S. has reached alarming levels, but according to a Centers for Disease Control and Prevention study (chart, below) the problem is considerably worse for minority

populations than for Caucasians. "If you think about all the environmental risk factors that contribute to this childhood obesity, many are worse in minority and low-income communities," says Shiriki Kumanyika, associate dean for health promotion and disease prevention at the University of Pennsylvania School of Medicine, who has studied obesity in minority populations for more than two decades. "You often have poorer availability of healthy food. Cultural and regional food preferences also may be contributors. Schools in lower-income communities often do not have adequate outside play areas, and there are built-in environmental issues like safety and street lighting [that inhibit exercise]. Two other factors: First, obesity rates are also higher in minority adults than in Caucasians, and the children of obese adults are more likely to be overweight than the children of normal-weight adults. Second, Kumanyika says, "a high rate of obesity is a longstanding situation in several minority populations: Mexicans, Native Americans and adult black women." But among young African-Americans, she says, "it's a more recent trend."
SN - 0038822X
UR -
https://login.proxy.bib.uottawa.ca/login?url=http://search.ebscohost.com/login.aspx?direct=true&db=s3h&AN=15037304&site=ehost-live
ER -

TY - JOUR
ID - 4959
T1 - Why Our Kids Are Overweight
A1 - T.L.
Y1 - 2004/11/15/
KW - SportDiscus
KW - *OBESITY in children
KW - *OVERWEIGHT children
KW - *EXERCISE
KW - *CHILDREN -- Health
KW - NUTRITION disorders in children
KW - United States
RP - NOT IN FILE
SP - 84
EP - 84
JA - Sports Illustrated
VL - 101
IS - 19
N2 - The article discusses reasons why children in the United States are heavier than ever before, as of 2004. Geography. In the latter half of the 20th century Americans increasingly moved from city neighborhoods to suburbs. Kids stopped walking and riding their bicycles and got into cars. Routine forms of exercise were lost. Fear. Children who were inclined to leave their homes to play were discouraged by parents who were fearful of abduction. Hardworking Parents. The increase in the number of two-income households has left kids stranded in after-school and extended day-care programs. Before, children were outdoors burning calories. The Soccer Myth. Organized youth teams have exploded--particularly in soccer--but youngsters who join them play only a couple of days a week and get a fraction of the exercise they need, often standing around awaiting their turns in static drills. Emphasis on Test Scores. Cash strapped schools, frightened by No Child Left Behind mandates, have shifted resources and class time from phys ed to prepping students for standardized tests. Fast Food. Mom's baked potato has been replaced by a supersized order of fries cooked in beef fat. Soft-drink machines have been lodged in high school hallways. Technology. First came television, followed by cable and digital and hi-def. Video games evolved from Pong to Tomb Raider. Finally came the Internet and the IM monster. Children sit and click. A Family Trait. The children of overweight people are often overweight themselves, and more than 64% of U.S. adults are overweight
SN - 0038822X
UR -
https://login.proxy.bib.uottawa.ca/login?url=http://search.ebscohost.com/login.aspx?direct=true&db=s3h&AN=15037303&site=ehost-live
ER -

TY - JOUR

ID - 4960
T1 - Developing fundamental movement skills in outdoor settings : three case studies of children playing
A1 - Taggart,A.
A1 - Keegan,L.
Y1 - 1997///
KW - SportDiscus
KW - *PLAY
KW - Case Studies
KW - Child
KW - motor skill
RP - NOT IN FILE
SP - 11
EP - 17
JA - ACHPER Healthy Lifestyles Journal
VL - 44
IS - 4
CY - ;
N2 - Recently research has shown that many children are not developing competence in fundamental movement skills. For example, it has been suggested that Victorian children in Grade 2 have major developmental deficits in throwing, catching, striking and kicking. It is clear that early schooling experiences must provide equitable and outcome producing play experiences for children if they are to develop fundamental movement skills that form that basis of future participation in physical activity. Three comparative case studies examined the opportunities that pre-primary children (5 year-olds) have to engage in fundamental movement skills in an outdoor play settings. Direct observation of individuals and small groups allowed for the analysis of the fundamental movement skills performed by children. Results indicated that many children are not engaging in the manipulative skills of throwing, catching, hitting and kicking while running, jumping and climbing were the predominant skills performed. Further findings indicate the variability in the practice of fundamental movement skills at the different centres relate to time allocation, teacher/adult presence in the play setting, playground design and equipment usage/availability
SN - 13210394
UR -
https://login.proxy.bib.uottawa.ca/login?url=http://search.ebscohost.com/login.aspx?direct=true&db=s3h&AN=SPH455193&site=ehost-live;http://articles.sirc.ca/search.cfm?id=455193
L2 - http://articles.sirc.ca/search.cfm?id=455193
ER -

TY - JOUR
ID - 4961
T1 - The impact of preschoolers' gender and sensory preferences on physical activity levels during play
A1 - Taleb Ismael,Noor
A1 - Mische Lawson,Lisa
Y1 - 2012/09/15/
N1 - Accession Number: 2011684145. Language: English. Entry Date: 20120928. Revision Date: 20130927. Publication Type: journal article; research; tables/charts. Journal Subset: Allied Health; Double Blind Peer Reviewed; Europe; Expert Peer Reviewed; Peer Reviewed; UK & Ireland. Special Interest: Occupational Therapy; Pediatric Care. Instrumentation: Sensory Profile (Dunn); Body Position Scale. No. of Refs: 38 ref. NLM UID: 7708186
KW - CINAHL
KW - Physical Activity -- In Infancy and Childhood
KW - Play and Playthings
KW - sensation
KW - Movement -- In Infancy and Childhood
KW - Mental Processes -- In Infancy and Childhood
KW - Clinical Assessment Tools
KW - Sex Factors
KW - Retrospective Design
KW - Nonparticipant Observation

KW - Questionnaires
KW - Summated Rating Scaling
KW - T-Tests
KW - One-Way Analysis of Variance
KW - P-Value
KW - hypothesis
KW - Child,Preschool
KW - Child
KW - Human
RP - NOT IN FILE
SP - 429
EP - 435
JF - British Journal of Occupational Therapy
VL - 75
IS - 9
N2 - Introduction: This study examined the differences between boys and girls in relation to activity levels during play and sensory preferences for activity level, body position and movement. A review of the literature revealed no evidence to explain if boys and girls prefer different body positions while playing or if body position might be used to measure their activity levels. Method: This retrospective study used data from demographic forms, children's Sensory Profiles and observations of children's play in classroom settings. The sample consisted of 53 typically developing children between ages 3-5 years from a suburban preschool in Kansas in the United States. Results: Analysis of data from play observations revealed no significant differences between boys and girls in relation to body position mean (p = 0.33), body position standard deviation (p = 0.19) and total number of change position times (p = 0.28). Analysis of data from children's Sensory Profiles showed no significant differences (p = 0.97) between boys' and girls' seeking preferences reflected by activity level, body position and movement items in the Sensory Profile. Conclusion: Future research should investigate children's play in outdoor playgrounds and implement more rigorous methods in rating activity levels
SN - 0308-0226
AD - Lecturer, Department of Rehabilitation Sciences, Faculty of Applied Medical Sciences, Jordan University of Science and Technology, Irbid, Jordan
Assistant Professor, Occupational Therapy Education, University of Kansas Medical Centre, Kansas City, Kansas, USA
UR - http://search.ebscohost.com/login.aspx?direct=true&db=cin20&AN=2011684145&site=ehost-live
ER -

TY - JOUR
ID - 4965
T1 - EFFECTS OF A SHORT-TERM COORDINATION EXERCISE PROGRAM DURING SCHOOL RECESS: AGILITY OF SEVEN- TO EIGHT-YEAR-OLD ELEMENTARY SCHOOL CHILDREN
A1 - Tatsuo,Y.A.S.U.
A1 - Haruo,N.O.G.A.
Y1 - 2013/04//
KW - SportDiscus
KW - *SCHOOL children
KW - *HEALTH promotion
KW - *EXERCISE
KW - *EXERCISE tests
KW - *MOTOR ability
KW - *STUDENTS
KW - *HEALTH
KW - Elementary Education
KW - Methodology
KW - Analysis of Variance
KW - CORRELATION (Statistics)
KW - Elementary Schools
KW - Statistics
KW - data analysis

KW - HUMAN services programs -- Evaluation
KW - DATA analysis -- Software
KW - Descriptive Statistics
KW - Japan
RP - NOT IN FILE
SP - 598
EP - 610
JF - Perceptual & Motor Skills
JA - Percept.Mot.Skills
VL - 116
IS - 2
N2 - This study tested the effect of a short-term coordination program focused on improving the agility of school children ages 7 to 8 years. Healthy Japanese children were placed in an experimental group of 26 children (10 girls, 16 boys) and a control group of 20 children (9 girls, 11 boys). The experimental group participated in 10 coordination program sessions during recesses; each session was performed 1 to 3 times per week, for 26 days. No differences were observed between the groups in repeated side-step scores prior to the program, although an interaction was observed after the completion of the treatment. The short-term coordination program was effective in increasing the agility of elementary school children aged 7 to 8 years. ABSTRACT FROM AUTHOR
SN - 00315125
UR - https://login.proxy.bib.uottawa.ca/login?url=http://search.ebscohost.com/login.aspx?direct=true&db=s3h&AN=91821357&site=ehost-live
ER -

TY - JOUR
ID - 4966
T1 - Physical activity behavior and related characteristics of highly active eighth-grade girls
A1 - Taverno Ross,S.E.
A1 - Dowda,M.
A1 - Beets,M.W.
A1 - Pate,R.R.
Y1 - 2013/06//
N1 - DA - 20130524
IS - 1879-1972 (Electronic)
IS - 1054-139X (Linking)
LA - eng
PT - Comparative Study
PT - Journal Article
PT - Research Support, N.I.H., Extramural
SB - IM
KW - Accelerometry
KW - Adolescent
KW - Adolescent Psychology
KW - Body Mass Index
KW - Cohort Studies
KW - Exercise
KW - Psychology
KW - Female
KW - Health Surveys
KW - Humans
KW - Leisure Activities
KW - Motivation
KW - Motor Activity
KW - Self Efficacy
KW - Social Environment
KW - Social Support

KW - Socioeconomic Factors
KW - United States
KW - Reviewer Nominated
RP - NOT IN FILE
SP - 745
EP - 751
JF - Journal of Adolescent Health
JA - J Adolesc Health
VL - 52
IS - 6
N2 - PURPOSE: Although girls are generally less physically active than boys, some girls regularly engage in high levels of physical activity (PA); however, very little is known about these girls and how they differ from those who are less physically active. This study examined the PA behavior and related characteristics of highly active adolescent girls and compared them with those who are less active. METHODS: Data from 1,866 eighth-grade girls from six field centers across the United States participating in the Trial of Activity for Adolescent Girls (TAAG) were included in the present analysis. Mixed-model analysis of variance examined differences in sociodemographic, anthropometric, psychosocial, and physical activity (accelerometry and self-report) variables between high- and low-active girls; effect sizes were calculated for the differences. RESULTS: High-active girls were taller, had lower body mass indices and body fat, and were less sedentary. High-active girls scored higher on self-efficacy, enjoyment of PA, self-management strategies, outcome-expectancy value, and support from family and friends than low-active girls. Low-active girls participated in more leisure time and educational sedentary activities than high-active girls. High-active girls participated in more PA classes/lessons outside of school, team sports, and individual sports. They were also more likely to participate in sports in an organized setting in the community or at school than low-active girls. CONCLUSIONS: Health promotion efforts should focus on decreasing the amount of time girls spend in sedentary activities and replacing that time with organized PA opportunities; such efforts should seek to minimize perceived barriers and increase self-efficacy and support for PA
AD - Department of Exercise Science, University of South Carolina, Columbia, SC 29208, USA. sross@mailbox.sc.edu
UR - 23384978
ER -

TY - JOUR
ID - 4967
T1 - Playgrounds: Managing your risk
A1 - Teague,Travis L.
Y1 - 1996/04//
KW - SportDiscus
KW - *PLAYGROUNDS
KW - *CHILDREN
KW - SAFETY measures
KW - United States
RP - NOT IN FILE
SP - 54
JA - Parks & Recreation
VL - 31
IS - 4
N2 - Reports on the management of playgrounds in the United States and the prevention of injuries to children through improved design and maintenance of the facilities. Americans' understanding of the legal system; Development and implementation of risk management programs; Areas where risks can be reduced; What risk management programs involved; Evaluation of risks
SN - 00312215
UR -
https://login.proxy.bib.uottawa.ca/login?url=http://search.ebscohost.com/login.aspx?direct=true&db=s3h&AN=9605202438&site=ehost-live
ER -

TY - JOUR
ID - 4968
T1 - The Relationship between Physical Activity, Fitness and Educational Achievement of Rural South African Children
A1 - Themane,M.J.
A1 - Koppes,L.L.J.
A1 - Kemper,H.C.G.
A1 - Monyeki,K.D.
A1 - Twisk,J.W.R.
Y1 - 2006/04//
KW - SportDiscus
KW - *PHYSICAL fitness
KW - *EXERCISE
KW - *ACADEMIC achievement
KW - *SCHOOL children
KW - South Africa
RP - NOT IN FILE
SP - 48
EP - 54
JA - Journal of Physical Education & Recreation (10287418)
VL - 12
IS - 1
N2 - The purpose of this study was to investigate the relationships of physical activity and physical fitness with educational achievement in rural South African school children aged 7 to 14 years (n= 212). All boys and girls underwent the following physical fitness tests: standing long jump, bent arm hang, sit ups, 10 x 5m shuttle run, 50 meter run, 1,600 meter run, flamingo balance, sit and reach, and plate tapping. All tests were performed in accordance with the European Tests of Physical Fitness (EUROFIT) and American Alliance for Health, Physical Education, Recreation and Dance (AAHPERD) protocols. A questionnaire was used to gather physical activity information concerning indoor and outdoor household activities, games and sports performed during the week days and weekend days. Educational achievement was assessed by means of the Educational Achievement Tests in Mathematics and English designed by South African Human Sciences Research Council. In contrast to what has been found in developed countries, the results of the present study did not show strong evidence for the hypothesis that physical activity and physical fitness are positively associated with educational achievement. Explanations for this finding may be that: first, the children in this population are confronted by a number of educational problems that may have confounded the relationship. Second, because all these children have high levels of physical activity it may be difficult to measure its influence on educational achievement, and third, the cross-sectional nature of this study. ABSTRACT FROM AUTHOR
SN - 10287418
UR -
https://login.proxy.bib.uottawa.ca/login?url=http://search.ebscohost.com/login.aspx?direct=true&db=s3h&AN=22451453&site=ehost-live
ER -

TY - JOUR
ID - 4970
T1 - Outdoor play: combating sedentary lifestyles
A1 - Thigpen,B.
Y1 - 2007/09//
N1 - Accession Number: 2009686474. Language: English. Entry Date: 20080222. Revision Date: 20131213. Publication Type: journal article; pictorial. Journal Subset: Biomedical; USA. Special Interest: Pediatric Care. No. of Refs: 10 ref. NLM UID: 9879031
KW - CINAHL
KW - Child Day Care
KW - Environment -- In Infancy and Childhood
KW - Life Style,Sedentary -- Prevention and Control -- In Infancy and Childhood
KW - Physical Activity -- In Infancy and Childhood
KW - Play and Playthings -- In Infancy and Childhood

KW - Child
KW - Child,Preschool
KW - Infant
KW - Information Resources
KW - World Wide Web
KW - Pediatric Obesity -- Prevention and Control
RP - NOT IN FILE
SP - 19
EP - 23
JA - Zero to Three
VL - 28
IS - 1
N2 - Increasingly sedentary lifestyles are contributing to overweight and other health concerns as children spend less and less time outside engaged in active play. Outdoor play provides important opportunities to explore the natural world, interact with peers, engage in vigorous physical activity, and learn about our environment. However, out-door environments often fall short of providing infants and toddlers with comfortable, engaging, and challenging activities needed for optimal physical development. This article provides suggestions for creating satisfying and developmentally appropriate outdoor play spaces for infants and toddlers
SN - 0736-8038
AD - Early Education and Management Specialist, Training and Technical Assistance Services, Western Kentucky University Research Foundation
UR - http://search.ebscohost.com/login.aspx?direct=true&db=cin20&AN=2009686474&site=ehost-live
ER -

TY - JOUR
ID - 4971
T1 - A developmental analysis of gender differences in health related physical fitness
A1 - Thomas,J.R.
A1 - Nelson,J.K.
A1 - Church,G.
Y1 - 1991/02//
KW - SportDiscus
KW - *PHYSICAL fitness
KW - *CARDIOVASCULAR fitness
KW - Surveys
KW - BOYS
KW - girls
KW - teenagers
KW - Puberty
KW - SEX FACTOR
KW - Child
KW - Growth
KW - Anthropometry
RP - NOT IN FILE
SP - 28
EP - 42
JF - Pediatric Exercise Science
JA - Pediatr Exerc Sci
VL - 3
IS - 1
CY - ;
N2 - Data for the analysis were the health related fitness scores, anthropometric measures, and physical activity information from the National Children and Youth Fitness Study. The subjects were 6,800 boys and 6,523 girls, ages 6 through 18. Multiple regression produced linear composites that were used as covariates to evaluate physical and environmental characteristics that relate to gender differences. The distance runs, chin-ups, and sit-ups displayed similar patterns in gender differences across age. Before puberty the important covariates are

mainly physical, namely skinfolds. Following puberty the major factors that reduce gender differences are skinfolds and the amount of exercise done outside of school time
SN - 08998493
UR -
https://login.proxy.bib.uottawa.ca/login?url=http://search.ebscohost.com/login.aspx?direct=true&db=s3h&AN=SPH271457&site=ehost-live;http://articles.sirc.ca/search.cfm?id=271457
L2 - http://articles.sirc.ca/search.cfm?id=271457
ER -

TY - JOUR
ID - 4973
T1 - Does participating in physical activity in outdoor natural environments have a greater effect on physical and mental wellbeing than physical activity indoors? A systematic review
A1 - Thompson Coon,J.
A1 - Boddy,K.
A1 - Stein,K.
A1 - Whear,R.
A1 - Barton,J.
A1 - Depledge,Michael H.
Y1 - 2011///
KW - Reviewer Nominated
RP - NOT IN FILE
SP - 1761
EP - 1772
JF - Environmental Science & Technology
JA - Environ Sci Technol
VL - 45
IS - 5
PB - ACS Publications
SN - 0013-936X
ER -

TY - THES
ID - 4974
T1 - Usual mobility methods of children with cerebral palsy: a comparison across home, school, and outdoors/community settings
A1 - Tieman,B.L.
Y1 - 2002///
N1 - Accession Number: 2004032465. Language: English. Entry Date: 20040213. Revision Date: 20091218. Publication Type: doctoral dissertation; research. Instrumentation: Gross Motor Function Classification System (GANEFS); Gross Motor Function Measure
KW - CINAHL
KW - Physical Mobility -- In Infancy and Childhood
KW - Cerebral Palsy -- In Infancy and Childhood
KW - Child,Disabled
KW - Home Environment
KW - Learning Environment
KW - Social Environment
KW - Activities of Daily Living
KW - Age Factors
KW - Child
KW - adolescence
KW - Prospective Studies
KW - Comparative Studies
KW - random sample
KW - Questionnaires
KW - Parents

KW - Clinical Assessment Tools
KW - functional assessment
KW - Psychomotor Performance
KW - Human
RP - NOT IN FILE
EP - 129
PB - MCP Hahnemann University
N2 - The purpose of this dissertation was to examine usual mobility methods of children with cerebral palsy (CP) in the home, school, and outdoors/community settings. Mobility is needed in activities of daily living and limitations in mobility may impact participation in society. Even though interventions are often provided in natural environments, knowledge of the impact of environmental setting on mobility is limited
The subjects were 307 children with CP, ages 6-14 years who participated in a longitudinal study, randomly selected from 19 centers, and stratified by age and Gross Motor Function Classification System (GANEFS) level. Measures included the GANEFS, parent-completed questionnaire on children's usual mobility methods, and the Gross Motor Function Measure
The first study indicated differences in gross motor capability and parent report of performance of mobility methods in children with CP. Among children with CP who have similar capability, there were differences in performance across settings. The second study demonstrated among children within the same GANEFS level, the variability of mobility methods across settings. Children performed higher-ranked mobility methods at home (requiring the most gross motor control), lower-ranked mobility methods at school, and the lowest-ranked mobility methods (requiring the least gross motor control) in the outdoors/community. The third study provides evidence for nonlinear changes in mobility over time in children with CP. During the first 1 1/2ƒ_"2 1/2 years, mobility methods increased to higher-ranked methods, while in the second 1 1/2ƒ_"2 years, mobility methods were unchanged or decreased to lower-ranked methods. The changes in mobility methods over time did not differ across settings
The findings indicate that environmental setting has an impact on the usual mobility methods of children with CP. The examination of children with CP should include capability and/or performance measures, depending on the purposes of examination. Performance should be measured specific to settings where children function, including the contextual features and personal factors that may influence mobility. Intervention should address the factors contributing to mobility, such as environmental adaptations and child-related factors. Screening of children with CP should occur at regular intervals so that interventions can address the changes in mobility
SN - 978-0-493-54687-2
AV - UMI Order AAI3041292.
M1 - Ph.D.
UR - http://search.ebscohost.com/login.aspx?direct=true&db=cin20&AN=2004032465&site=ehost-live
ER -

TY - JOUR
ID - 4976
T1 - SWIMMING TRAINING STUDENTS FUTURE TEACHERS AND EDUCATORS TO RECREATIONAL ACTIVITIES VALID EXECUTION
A1 - TosiE%c,Sladjana
A1 - MilanoviE%c,Sandra
A1 - IgnjatoviE%c,Aleksandar
Y1 - 2012/12//
KW - SportDiscus
KW - *SWIMMING -- Training
KW - *RECREATION
KW - *PHYSICAL education
KW - *UNDERGRADUATES
KW - *SWIMMERS
KW - *PHYSICAL education teachers
KW - CURRICULA (Courses of study)
RP - NOT IN FILE
SP - 246
EP - 255
JA - Research in Kinesiology

VL - 40
IS - 2
N2 - In the elective course of Basic games and swimming it has been realized a seven days practical training program as a part of undergraduate studies for the following courses: classroom teacher, the preschool teacher and educator in children's homes. The purpose of this course is to enable students to track, Organize, -and continuously upgrade the teaching process in the field of outdoor activities and swimming techniques, as well as the elementary games on the water. With a good organization, under the existing conditions (indoor Olympic swimming pool), the methodological procedures that have so far proved as most effective in a very short time, non-swimmer students have mastered the basic elements of swimming. In the beginning they were practicing on the ground through a game and then through specific movements in the water. The participant s sample consisted of students of the Faculty of Pedagogy in Jagodina. A total of 116 students followed the curriculum, 44 of who were non-swimmers, 14 were swimmers and 58 were only swimmer beginners. After having the results at the final measuring, after carrying out the plan and program, we were been able to determine that a large number of students were able to learn the techniques properly and that the number of floaters decreased significantly, while the number of swimmers increased. ABSTRACT FROM AUTHOR
SN - 18577679
UR -
https://login.proxy.bib.uottawa.ca/login?url=http://search.ebscohost.com/login.aspx?direct=true&db=s3h&AN=85771356&site=ehost-live
ER -

TY - JOUR
ID - 4977
T1 - The determination of a management model for sport at the Witbank campus of Technikon Pretoria
A1 - Tossel,G.L.F.
Y1 - 2004///
KW - SportDiscus
KW - *COLLEGE sports -- Management
KW - *UNIVERSITIES & colleges
KW - *SPORTS
KW - South Africa
KW - MANAGEMENT SYSTEM
RP - NOT IN FILE
CY - Eugene, OR;
PB - Kinesiology Publications, University of Oregon
N2 - In March of 1995, the Ministry of Sport presented the Government's first attempt at a new sport policy by submitting the 'White Paper on Sport' to the nation. The White Paper, aptly titled 'Getting the Nation to Play' represented the Government's broad vision of how South African sport could be developed. Though touching on all aspects of sport, it emphasizes that the focus is to be at the bottom end of the sport development continuum(South Africa, Ministry of Sport, 1995:87). This is crucial because the goal is to reverse the low levels of participation among the Black population, especially those in the rural areas. The changes that have taken place in the political arena in South Africa since 1990, have not only impacted on the political, social and economic spheres of South Africa but have also permanently changed the face of South African sport inside and outside the country. South Africans as citizens of the global sports village however now have to deal with tremendous challenges facing the administrators, managers, coaches and participants. One of these challenges is to improve the participation levels of the youth in South Africa. The expression a child in sport is a child out of courthas special relevance in this regard. The purpose of this research is inter alia to: shortly discuss the inception and development of Technikon Pretoria with specific relevance to the Witbank Campus; describe the management of sport in South Africa; document the practical execution of a needs assessment for the management and development of sport at the Witbank Campus of Technikon Pretoria; and process the raw data into understandable and interpreted units. These units will then be used as a basis from which mechanisms will follow to ensure that the management and development of sport at the Witbank Campus of Technikon Pretoria be done in a holistic manner but at the same time with practical application. The main aim is to improve the participation levels in sport of all students at the Witbank Campus of Technikon Pretoria
UR -
https://login.proxy.bib.uottawa.ca/login?url=http://search.ebscohost.com/login.aspx?direct=true&db=s3h&AN=SPHS-1005642&site=ehost-live;http://kinpubs.uoregon.edu/

ER -

TY - JOUR
ID - 4978
T1 - Selected socio-psychological factors as related to the childhood games of successful women
A1 - Toyama,J.S.
Y1 - 1979///
KW - SportDiscus
KW - *PLAY
KW - *GAMES
KW - *SEX role
KW - girls
KW - Child Rearing
KW - behaviour
KW - Child
RP - NOT IN FILE
CY - ;
N2 - Investigates the types of games played by successful women outside of school during the ages of 6-12 years. Hypothesis that successful women might not necessarily conform to the game involvement for women in general was supported. Investigates differences between academic and administrative women in terms of factors which would have the greatest predictive power as related to success in each role. Questionnaires were received from 194 full-time professional academic and non-academic women. Found that the success of female administrators was related to number of team games, but not to the number of male games
UR -
https://login.proxy.bib.uottawa.ca/login?url=http://search.ebscohost.com/login.aspx?direct=true&db=s3h&AN=SPH67642&site=ehost-live;http://articles.sirc.ca/search.cfm?id=67642
L2 - http://articles.sirc.ca/search.cfm?id=67642
ER -

TY - JOUR
ID - 4979
T1 - Factors Associated with Physical Inactivity in Adolescents in Ho Chi Minh City, Vietnam
A1 - Trang,Nguyen H.H.D.
A1 - Hong,Tang K.
A1 - Dibley,Michael J.
A1 - Sibbritt,David W.
Y1 - 2009/07//
KW - SportDiscus
KW - *PHYSICAL fitness for children
KW - *TEENAGERS
KW - *PHYSIOLOGY
KW - *EXERCISE
KW - Junior High School Students
KW - Surveys
KW - CROSS-sectional method
KW - logistic regression analysis
KW - HO Chi Minh City (Vietnam)
KW - Vietnam
KW - Environment
KW - High School Students
KW - Physical Activity
KW - Risk Factors
KW - Southeast Asia
RP - NOT IN FILE
SP - 1374
EP - 1383
JF - Medicine & Science in Sports & Exercise

JA - Med Sci Sports Exerc
VL - 41
IS - 7
N2 - The article discusses a study which examines the risk factors for physical inactivity among adolescents in Ho Chi Minh City (HCMC), Vietnam. Using multistage cluster sampling, a cross-sectional survey was performed among 2684 junior high school students in 2004. Hierarchical multivariate logistic regression was used to identify potential risk factors associated with physical inactivity. The study has found that overweight status, passive transportation, absence of recess exercises, and availability of a nearby game shop were among the factors associated with physical inactivity among adolescents. Findings indicate that physical activity among adolescent is associated with household, school, and community factors
SN - 01959131
UR -
https://login.proxy.bib.uottawa.ca/login?url=http://search.ebscohost.com/login.aspx?direct=true&db=s3h&AN=44288617&site=ehost-live
ER -

TY - RPRT
ID - 4980
T1 - A Healthy Dose of Green:A prescription for a healthy population
A1 - Trees Ontario
Y1 - 2012///
KW - Reviewer Nominated
RP - IN FILE
VL - www.treesontario.ca
CY - Toronto
PB - Trees Ontario
N2 - (we have the PDF of the full report)
 Executive Summary
Our health and well-being are intricately interconnected with the health of our natural environment. Trees and forests are integral components of healthy ecosystems that support healthy human populations.
Trees help to reduce smog and pollution in our cities by filtering out many airborne pollutants that have negative impacts on our health, such as carbon dioxide, carbon monoxide, lead, nitrogen dioxide, ozone, sulphur dioxide and particulates. These pollutants have been linked to heart disease, respiratory illnesses, diabetes and cancer.
Forests and green spaces have also been linked to a significant decline in stress, improved rehabilitation, faster hospital recovery rates, and a decrease in the severity of symptoms in attention deficit disorders.
Experts have determined that a minimum 30 per cent forest cover is required to maintain a healthy, sustainable ecosystem. Currently, forest cover is as low as five per cent in some regions of Ontario's settled landscape, compromising the health of our ecosystems and their inhabitants.
The decline in forest cover is evident in both rural and urban landscapes in Ontario. In the settled regions, rural forests have been diminished as a result of increased population density, agricultural land uses and a rapid growth in residential and commercial development. Trees and forests in urban areas face their share of challenges including pollution, climate change, introduced pests, competing demand for root space, disease and old age. Ontario now has severely fragmented forests in both rural and urban areas, threatening species migration, wildlife habitats, water cycles and climate regulation.
Efforts to enrich our
ER -

TY - JOUR
ID - 4981
T1 - Incidental movement, lifestyle-embedded activity and sleep: new frontiers in physical activity assessment
A1 - Tremblay,Mark S.
A1 - Esliger,Dale W.
A1 - Tremblay,Angelo
A1 - Colley,Rachel
Y1 - 2007/12/03/

KW - SportDiscus
KW - *PHYSICAL activity
KW - *SLEEP
KW - *LIFESTYLES
KW - *CALORIC expenditure
KW - *HUMAN mechanics
KW - *PUBLIC health
KW - Canada
KW - Energy Expenditure
KW - lifestyle assessment
KW - NEAT
KW - non-exercise activity thermogenesis
KW - physical inactivity
KW - sedentary behaviour
KW - Acvaluation du mode de vie
KW - comportement sAcdentaire
KW - dAcpense d'Acnergie
KW - inactivitAc physique
KW - thermogenA"se d'origine autre que l'activitAc physique
RP - NOT IN FILE
SP - S208
EP - S217
JA - Applied Physiology, Nutrition & Metabolism
VL - 32
N2 - Canadian public health messages relating to physical activity have historically focused on the prescription of purposeful exercise, most often assessing leisure-time physical activity (LTPA). Although LTPA contributes to total energy expenditure (TEE), a large part of the day remains neglected unless one also considers the energy expended outside of purposeful exercise. This paper reviews the potential impact of incidental (non-exercise or non-purposeful) physical activity and lifestyle-embedded activities (chores and incidental walking) upon TEE and indicators of health. Given that incidental movement occurs sporadically throughout the day, this form of energy expenditure is perhaps most vulnerable to increasingly ubiquitous mechanization and automation. The paper also explores the relationship of physical inactivity, including sleep, to physical activity, TEE, and health outcomes. Suggestions are provided for a more comprehensive physical activity recommendation that includes all components of TEE. Objective physical activity monitors with time stamps are considered as a better means to capture and examine human movements over the entire day. ABSTRACT FROM AUTHOR
Les messages de santAc publique en matiA"re dƒ_TactivitAc physique au Canada ont mis traditionnellement lƒ_Taccent sur la prescription dƒ_Texercices physiques utiles, principalement pratiquAcs durant les pAcriodes de loisir (LTPA). MA ¦me si les LTPA contribuent Aÿla dAcpense AcnergActique totale (TEE), une grande partie des activitAcs faites durant la journAce sont nAcgligAces Aÿmo ins de porter une attention Aÿ lƒ_TAcnergie dAcpensAce en plus des activitAcs utiles. Cet article analyse lƒ_Timpact des activitAcs physiques dites accessoires (ne constituant pas de lƒ_Texercice physique en soi ou nƒ_TActant pas utiles) et des activitAcs physiques intAcgrAces dans le mode de vie (les tA›ches journaliA"res et la marche concomitante) sur la TEE et les indicateurs de santAc. Comme les mouvements accessoires sƒ_Taccomplissent de faA□on intermittente dans le quotidien, cette forme de dAcpense dƒ_TAcnergie devrait diminuer face Aÿ la gAcnAcralisation de la mAccanisation et de lƒ_Tautomatisation des tA›ches. Cet article analyse aussi la relation entre lƒ_TinactivitAc physique, y compris le sommeil, et lƒ_TactivitAc physique, la TEE et les rAcpercussions sur la santAc. On y trouve Acgalement des propositions visant Aÿ la prescription dƒ_TactivitAc physique plus dActaillAce incluant toutes les composantes de la TEE. Le monitorage objectif des activitAcs physiques selon les pAcriodes de la journAce constitue le meilleur moyen dƒ_TAcvaluer et dƒ_Tanalyser les mouvements humains durant toute la journAce. ABSTRACT FROM AUTHOR
SN - 17155312
UR -
https://login.proxy.bib.uottawa.ca/login?url=http://search.ebscohost.com/login.aspx?direct=true&db=s3h&AN=45548566&site=ehost-live
ER -

TY - JOUR

ID - 4984
T1 - Physical Abilities and Mobility Scale: Reliability and Validity in Children Receiving Inpatient Rehabilitation for Acquired Brain Injury
A1 - Trovato,Melissa K.
A1 - Bradley,Elena
A1 - Slomine,Beth S.
A1 - Salorio,Cynthia F.
A1 - Christensen,James R.
A1 - Suskauer,Stacy J.
Y1 - 2013/07//
KW - SportDiscus
KW - *MOTOR ability
KW - *CHILDREN
KW - *HOSPITALS
KW - *ACTIVITIES of daily living
KW - *BODY movement
KW - *BRAIN -- Wounds & injuries
KW - *PATIENTS
KW - Evaluation
KW - OUTCOME assessment (Medical care) -- Methodology
KW - PHYSICAL therapy for children -- Methodology
KW - CORRELATION (Statistics)
KW - Disability Evaluation
KW - Factor Analysis
KW - HOSPITAL utilization -- Length of stay
KW - RESEARCH -- Methodology
KW - Psychometrics
KW - Rehabilitation Centers
KW - Research -- Evaluation
KW - RESEARCH -- Finance
KW - Statistics
KW - T-test (Statistics)
KW - INTER-observer reliability
KW - MULTITRAIT multimethod techniques
KW - RESEARCH methodology evaluation
KW - acquired brain injury (ABI)
KW - developmental functional quotient (DFQ)
KW - Gross Motor Function Measure (GMFM)
KW - intraclass correlation coefficient (ICC)
KW - Pediatric Evaluation and Disability Inventory (PEDI)
KW - Physical Abilities and Mobility Scale (PAMS)
KW - traumatic brain injury (TBI)
RP - NOT IN FILE
SP - 1335
EP - 1341
JF - Archives of Physical Medicine & Rehabilitation
JA - Arch Phys Med Rehabil
VL - 94
IS - 7
N2 - Abstract: Objective: To investigate the psychometric properties of the Physical Abilities and Mobility Scale (PAMS) in children receiving inpatient rehabilitation for acquired brain injury (ABI). Design: Admission and discharge PAMS item and total scores were evaluated. The WeeFIM was used as the criterion standard. A case study was used to illustrate the complementary nature of the PAMS and WeeFIM. Setting: A single, free-standing, academically affiliated pediatric rehabilitation hospital. Participants: Children (N=107) aged 2 through 18 years receiving inpatient rehabilitation for ABI between March 2009 and March 2012. Forty-two additional children treated during this time were excluded because of missing PAMS data. Interventions: Not applicable. Main Outcome Measures: Internal consistency was evaluated using Cronbach alpha. Interrater reliability was

evaluated through overall agreement, Pearson correlations, and intraclass correlations. Construct validity was examined through exploratory factor analysis. Criterion validity was explored through correlations of PAMS overall and factor scores with WeeFIM total and subscale scores. Sensitivity to recovery was examined using paired t tests, examining differences between admission and discharge scores for each item and for the total score. Results: Internal consistency and interrater reliability were high. Factor analysis revealed 2 factors: lower-level skills and higher-level mobility skills. Correlations with the WeeFIM ranged from moderate to very strong; total PAMS score most strongly correlated with the WeeFIM mobility subscore. Total PAMS score and each item score significantly increased between admission and discharge. Conclusions: The PAMS is a reliable and valid measure of progress during inpatient rehabilitation for children with ABI. By capturing fine-grain progress toward both lower-level and higher-level mobility skills, the PAMS complements the WeeFIM in assessing functional gains during the rehabilitation stay. Copyright &y& Elsevier
SN - 00039993
UR -
https://login.proxy.bib.uottawa.ca/login?url=http://search.ebscohost.com/login.aspx?direct=true&db=s3h&AN=89338298&site=ehost-live
ER -

TY - JOUR
ID - 4985
T1 - A report on selected dietary characteristics in children 9-11 years of age
A1 - Tsagdi,C.
A1 - Laparidis,K.
A1 - Michalopoulou,M.
A1 - Taxildaris,K.
A1 - Trigonis,I.
Y1 - 2000///
KW - SportDiscus
KW - *NUTRITION
KW - *FOOD -- Caloric content
KW - *BODY weight
KW - *STATURE
KW - *OBESITY
KW - Skinfold Thickness
KW - girls
KW - BOYS
KW - Greece
KW - Adipose Tissue
KW - SEX FACTOR
KW - EVALUATION STUDY
KW - PREPUBESCENT
KW - Child
RP - NOT IN FILE
SP - 76
EP - 84
JA - Exercise & Society Journal of Sport Science
IS - 26
CY - ;
N2 - The purpose of this study was to report the eating behavior inside and outside the school environment, of the prepubescent children. Also, to discover any relation between their physical characteristics, and their every day eating behavior. 60 Greek children (M = 10.5 years old, SD = 1.5 years), participated in this research. The sample consisted of 31 boys and 29 girls from the 5th and 6th grade of the 2nd Primary Public School of Komotini. Children's weight and height were recorded. A skinfold caliper was used for the assessment of the percentage of the body fat, according to the equation of Slaughter et al. (1988). For the evaluation of children's eating behaviors in every day life, special cards adopted from Baranowski et al. (1986) were used for a one-week period. The special food cards were then evaluated with Diet food analysis software (Strength & Conditioning, 1997). The results have shown that: food intake effects the concentration of all food ingredients, like calories, proteins, carbohydrates, fats etc. According the body fat results of the subjects, boys belong to

obese group and girls tend to obesity. Results also revealed that the Physical characteristics like weight, height, % of body fat and obesity could be predicted by the estimation of the rest Physical characteristics and by selected nutrients
SN - 1105655X
UR - https://login.proxy.bib.uottawa.ca/login?url=http://search.ebscohost.com/login.aspx?direct=true&db=s3h&AN=SPHS-794095&site=ehost-live;http://articles.sirc.ca/search.cfm?id=S-794095
L2 - http://articles.sirc.ca/search.cfm?id=S-794095
ER -

TY - JOUR
ID - 4986
T1 - Assessing physical activity & physical characteristics of prepubescent children in Greece
A1 - Tsagdi,C.
A1 - Taxildaris,K.
A1 - Laparidis,K.
A1 - Michalopoulou,M.
Y1 - 2002///Spring
KW - SportDiscus
KW - *PHYSICAL fitness
KW - *STRESS (Physiology)
KW - *CARDIOVASCULAR fitness
KW - *BODY weight
KW - *STATURE
KW - Questionnaires
KW - Television
KW - Parents
KW - BOYS
KW - girls
KW - Greece
KW - Adipose Tissue
KW - CORRELATION
KW - Child
RP - NOT IN FILE
SP - 75
EP - 82
JA - Physical Educator
VL - 59
IS - 2
CY - ;
N2 - Despite the great importance given by the government to improve the conditions of children's living through fitness and activities, there aren't many researches attempting to record prepubescent physical activity as a means of improving health. It is unlikely that children's amount of range physical activity, of which develops in their social environment, has never been studied in Greece. In an attempt to overcome this, the purposes of this research were to record physical activity inside and outside the school environment, and discover a possible relationship between the motor activity of children and their physical characteristics. Sixty Greek children (10 years of age, SD 0.5 years) participated in this research. The sample consisted of 31 boys and 29 girls from 5th and 6th grade of Primary Public School. Physical activity was recorded with the use of a questionnaire (Sallis, et al., 1993), and a questionnaire for the assessment of parents' opinion about physical activity of their children was also distributed (Rose et al., 1987). Children's weight and height were recorded and a skin fold caliper was used for the assessment of the percentage of body fat, according to the equation of Slaughter, et al., 1988. The statistical analysis of the data included linear correlation, stepwise regression analysis, frequencies and ANOVA. Results showed: Children's physical activity was not correlated with the physical characteristics. Physical characteristics like weight, height, percent body fat and obesity could be anticipated by the values of one or more physical characteristics and, there was a significant difference between parent's estimates and children's personal opinion
SN - 00318981

UR -
https://login.proxy.bib.uottawa.ca/login?url=http://search.ebscohost.com/login.aspx?direct=true&db=s3h&AN=SPHS-847505&site=ehost-live;http://articles.sirc.ca/search.cfm?id=S-847505;http://www2.truman.edu/pek
L2 - http://articles.sirc.ca/search.cfm?id=S-847505
ER -

TY - JOUR
ID - 4987
T1 - Is the Incidence in Volleyball Injuries Age Related? A Prospective Study in Greek Male Volleyball Players
A1 - Tsigganos,George
A1 - Beneka,Anastasia
A1 - Malliou,Paraskevi
A1 - Gioftsidou,Asimenia
A1 - Zetou,Helen
A1 - Godolias,George
Y1 - 2007/08//
KW - SportDiscus
KW - *VOLLEYBALL
KW - *ATHLETES
KW - *WOUNDS & injuries
KW - *TRAINING
KW - *DIAGNOSIS
KW - MALES
KW - age
KW - teenagers
KW - YOUNG adults
KW - Competition
KW - Child
KW - prospective study
RP - NOT IN FILE
JA - Physical Training
CY - ;
N2 - Objective: Record the injury incidence in all age categories in male volleyball players and clarify the role of age in injury occurrence. Design and Setting: The injury incidence rate, severity, diagnosis and the anatomical location of the injuries, which occurred during practice and competition in all the championship period, were recorded prospectively during the period 2005-2006. Participants: A total of 407 Greek male volleyball players participating in all Volleyball Championships in Greece according the Greek Volleyball Federation have been observed on weekly basis for the period 2005-06. Main Outcome Measurements: The injury incidence rate recorded as number of injured players and also number of injuries per player/year (total exposure time). Results: Injury occurrence is age related and junior volleyball players have lower injury rate than the senior ones. Acute injuries were significantly more in comparison to overuse syndrome and the ankle was the most common injured anatomical location. Finally, outside hitters, universals and setters presented different injury rate than the other players and the "incorrect sprawls" & "step on others' foot" were the most common injury factors. Conclusions: Findings in injury occurrence rate must be interpreted in relation to the total exposure time in order to have more realistic conclusions. [ABSTRACT FROM AUTHOR]
SN - 14921685
UR -
https://login.proxy.bib.uottawa.ca/login?url=http://search.ebscohost.com/login.aspx?direct=true&db=s3h&AN=SPHS-1064645&site=ehost-live;http://articles.sirc.ca/search.cfm?id=S-1064645;http://www.ejmas.com/
L2 - http://articles.sirc.ca/search.cfm?id=S-1064645
ER -

TY - JOUR
ID - 4992
T1 - Withholding Recess From Elementary School Students: Policies Matter
A1 - Turnerx,L.I.N.D.

A1 - Chriqui,Jamie F.
A1 - Chaloupka,Frank J.
Y1 - 2013/08//
KW - SportDiscus
KW - *ACADEMIC achievement
KW - *CHILDREN
KW - *CONDUCT of life
KW - *EPIDEMIOLOGY
KW - *STUDENTS
KW - *HEALTH
KW - *PHYSICAL activity
KW - CHI-squared test
KW - Confidence Intervals
KW - Multivariate Analysis
KW - Questionnaires
KW - School Administrators
KW - SCHOOL administration
KW - logistic regression analysis
KW - data analysis
KW - CROSS-sectional method
KW - DATA analysis -- Software
KW - law
KW - Obesity
KW - obesity.
KW - Physical Activity
KW - school district
RP - NOT IN FILE
SP - 533
EP - 541
JF - Journal of School Health
JA - J Sch Health
VL - 83
IS - 8
N2 - ABSTRACT BACKGROUND Recess is a key aspect of a healthy elementary school environment and helps to keep students physically active during the school day. Although national organizations recommend that students not be withheld from recess, this practice occurs in schools. This study examined whether district policies were associated with school practices regarding the withholding of recess for behavior management or academic reasons, as well as the use of physical activity (eg, running laps) for behavior management. METHODS Data were gathered from administrators at US public elementary schools by mail-back surveys conducted during the spring of the 2008-2009, 2009-2010, and 2010-2011 school years. Corresponding school district policies were gathered and coded. Each year's sample was nationally representative and data were analyzed as a stacked cross-sectional sample. A total of 1,919 surveys were received (61.2% response rate). RESULTS Averaged over the 3 years, students were not withheld from recess for poor behavior or academic reasons at 28.3% and 26.7% of elementary schools, respectively. Strong district policy was associated with an increased odds of not withholding students from recess for poor behavior (adjusted odds ratio [OR] = 2.27, p < .001) or completing schoolwork (adjusted OR = 1.99, p < .001). Withholding recess was less common in the South versus the West, Midwest, and Northeast. Use of physical activity for behavior management was rare and not associated with district policy. CONCLUSIONS Strengthening district policies may be an effective strategy for changing school practices pertaining to withholding of recess, and to ensure that all students have opportunities to be physically active during the school day. ABSTRACT FROM AUTHOR
SN - 00224391
UR -
https://login.proxy.bib.uottawa.ca/login?url=http://search.ebscohost.com/login.aspx?direct=true&db=s3h&AN=88931859&site=ehost-live
ER -

TY - JOUR

ID - 4993
T1 - Reading, writing, recess: is jump-rope the answer to the obesity epidemic?
A1 - Tyre,P.
Y1 - 2003/11/03/
N1 - Accession Number: 2004036653. Language: English. Entry Date: 20040220. Publication Type: journal article; pictorial. Journal Subset: Consumer Health; USA. Special Interest: Consumer Health. NLM UID: 9877127
KW - CINAHL
KW - child health
KW - Obesity -- Prevention and Control
KW - Physical Activity -- In Infancy and Childhood
KW - School Health
KW - Child
KW - Schools,Elementary
KW - United States
RP - NOT IN FILE
SP - 66
EP - 66
JA - Newsweek
VL - 142
IS - 18
SN - 0028-9604
UR - http://search.ebscohost.com/login.aspx?direct=true&db=cin20&AN=2004036653&site=ehost-live
ER -

TY - JOUR
ID - 4994
T1 - Physical recreation participation of females and males during the adult life cycle
A1 - Unkel,M.B.
Y1 - 1981///
KW - SportDiscus
KW - *SPORTS
KW - *RECREATION
KW - *PARTICIPATION
KW - adulthood
KW - age
KW - SEX FACTOR
KW - life cycle
RP - NOT IN FILE
CY - ;
N2 - This study examined changes occurring in the physical activity pattern of college-educated men and women during the adult life cycle. Participation was measured both in terms of the variety of activities and the number of participation days across a spectrum of 30 activities, and for three categories of activities within that spectrum (individual/dual activities, outdoor activities, and team sports). The results indicate lower participation of females than males in team sports and outdoor activities, but not in individual/dual sports. A decline in participation was noted with increasing age and this effect differed between males and females. Marital status and presence of children did not differentially affect participation of males and females. Participation was found to decrease faster with age for single persons as opposed to married persons or those with children. Implications for physical education curriculum planning are discussed
SN - 01490400
UR -
https://login.proxy.bib.uottawa.ca/login?url=http://search.ebscohost.com/login.aspx?direct=true&db=s3h&AN=SPH95856&site=ehost-live;http://articles.sirc.ca/search.cfm?id=95856
L2 - http://articles.sirc.ca/search.cfm?id=95856
ER -

TY - JOUR

ID - 4995
T1 - Validity of Accelerometry for Monitoring Real-World Arm Activity in Patients With Subacute Stroke: Evidence From the Extremity Constraint-Induced Therapy Evaluation Trial
A1 - Uswatte,Gitendra
A1 - Giuliani,Carol
A1 - Winstein,Carolee
A1 - Zeringue,Angelique
A1 - Hobbs,Laura
A1 - Wolf,Steven L.
Y1 - 2006/10//
KW - SportDiscus
KW - *ARM
KW - *CEREBROVASCULAR disease
KW - *REHABILITATION
KW - Movement
KW - CONSTRAINT-INDUCED MOVEMENT THERAPY
KW - Treatment Outcome
RP - NOT IN FILE
SP - 1340
EP - 1345
JF - Archives of Physical Medicine & Rehabilitation
JA - Arch Phys Med Rehabil
VL - 87
IS - 10
CY - ;
N2 - Objective: To examine the psychometric properties of an objective method for assessing real-world arm activity in a large sample with subacute stroke. Design: Validation study. Setting: Community. Participants: Persons 3 to 9 months poststroke (N = 169) with mild to moderate motor impairment of their hemiparetic arm enrolled in a multisite, randomized clinical trial of constraint-induced movement therapy. Interventions: Not applicable. Main Outcome Measures: Participants wore an accelerometer on each arm outside the laboratory for 3 days before and after treatment or an equivalent no-treatment period. They also completed the Actual Amount of Use Test (AAUT), which is an observational measure of spontaneous more-impaired arm use, and the Motor Activity Log (MAL), which is an interview assessing more-impaired arm use in daily life. Results: Low-pass-filtered accelerometer recordings were reliable (r range, > .8) and stable (P range, > .48). Their validity was also supported. Correlations calculated across all participants at baseline between the ratio of more-impaired to less-impaired arm accelerometer recordings and AAUT and MAL scores were .60 and .52, respectively. Conclusions: Accelerometry provides an objective, real-world index of more-impaired arm activity with good psychometric properties. [ABSTRACT FROM AUTHOR]
SN - 00039993
UR -
https://login.proxy.bib.uottawa.ca/login?url=http://search.ebscohost.com/login.aspx?direct=true&db=s3h&AN=SPHS-1037896&site=ehost-live;http://articles.sirc.ca/search.cfm?id=S-1037896
L2 - http://articles.sirc.ca/search.cfm?id=S-1037896
ER -

TY - JOUR
ID - 4996
T1 - Kids Need "Off-leash" Areas Too!
A1 - Vallentyne,Jane
Y1 - 2008///Spring
KW - SportDiscus
KW - *CHILDREN
KW - *PLAY
KW - *RECREATION
KW - *AMUSEMENTS
KW - *OUTDOOR recreation
RP - NOT IN FILE

SP - 21
EP - 21
JA - Fitness Informer
N2 - This article discusses a need by children to enjoy the joys of playing outdoors. According to the article, the growing trend of shifting away from outdoor play can be attributed to societal trends, including urbanization, changing family structures and technology. Several ways parents can enhance the play experiences of their children include making time for play, providing play materials and offering a variety of play experiences
UR -
https://login.proxy.bib.uottawa.ca/login?url=http://search.ebscohost.com/login.aspx?direct=true&db=s3h&AN=32068010&site=ehost-live
ER -

TY - JOUR
ID - 4997
T1 - AMBASSADOR SCHWARZER
A1 - Van Aken,M.A.R.K.
Y1 - 2009/09//
KW - SportDiscus
KW - *SOCCER goalkeepers
KW - *SOCCER
KW - AUTHORS
KW - CHILDREN'S books
KW - Australia
RP - NOT IN FILE
SP - 34
EP - 35
JA - Soccer International
VL - 17
IS - 8
N2 - The article focuses on Fulham goalkeeper Mark Schwarzer and his career outside the playing field as the author of the children's book series titled "Megs." The book which is co-authored by Schwarzer's business partner Neil Montagnana is about a fictitious character, Megs Morrison, who loves football and struggles to garner the recognition the game deserves. The author of the article presents his day with Schwarzer and Morrisson as they promote "Megs" on screen and in schools across Australia
SN - 13217763
UR -
https://login.proxy.bib.uottawa.ca/login?url=http://search.ebscohost.com/login.aspx?direct=true&db=s3h&AN=44233718&site=ehost-live
ER -

TY - JOUR
ID - 4998
T1 - Effects of the redevelopment of an elementary school playground
A1 - van Andel,J.
Y1 - 1985/09//
KW - SportDiscus
KW - *PLAYGROUNDS
KW - *PLAY
KW - *PARTICIPATION
KW - Elementary Schools
KW - DESIGN
KW - MODIFICATIONS
KW - age
KW - Child
KW - behaviour
KW - SEX FACTOR
KW - Observation

RP - NOT IN FILE
SP - 307
EP - 320
JF - Leisure Studies
VL - 4
IS - 3
CY - ;
N2 - The effects of the redevelopment of an elementary school playground were evaluated by comparing the situation before and after physical transformation. Observations produced data on the actual use during play time. Interviews were held with all the pupils on their perception and evaluation of the change. A group of adult experts judged the environment before and after the change on aspects like complexity, 'manipulability' and 'affordance' for different activities. An interview with the designer of the plan gave information about his intentions and expectations, especially regarding behavioural aspects. Data from these different sources are presented and related to each other
SN - 02614367
UR -
https://login.proxy.bib.uottawa.ca/login?url=http://search.ebscohost.com/login.aspx?direct=true&db=s3h&AN=SPH176143&site=ehost-live;http://articles.sirc.ca/search.cfm?id=176143
L2 - http://articles.sirc.ca/search.cfm?id=176143
ER -

TY - JOUR
ID - 4999
T1 - What makes nature restorative?
A1 - van der Ha,Bianca
Y1 - 2011///
KW - Reviewer Nominated
RP - NOT IN FILE
ER -

TY - JOUR
ID - 5000
T1 - BEWARE: NATURE AHEAD!
A1 - Van Duyne,Gloria
Y1 - 2011/12//
N1 - Accession Number: 71663243; Van Duyne, Gloria; Source Info: Dec2011, Vol. 66 Issue 3, p20; Thesaurus Term: OUTDOOR recreation; Thesaurus Term: NATURE; Subject Term: HIKING; Subject Term: STRESS management; Subject Term: AGGRESSION (Psychology); Number of Pages: 4p; Document Type: Article; Full Text Word Count: 1323
KW - OUTDOOR recreation
KW - Nature
KW - hiking
KW - STRESS management
KW - AGGRESSION (Psychology)
KW - Reviewer Nominated
RP - NOT IN FILE
SP - 20
EP - 23
JA - New York State Conservationist
VL - 66
IS - 3
N2 - The author discusses the benefits of enjoying nature. She relates his experience of hiking in the hills and exploring the nearby woods, creeks and fields when she was a child. She cites studies and articles about the benefits of playing outside, including stress reduction, reduced aggression, and better concentration
SN - 21571082
M3 - Article
UR - http://search.ebscohost.com/login.aspx?direct=true&db=8gh&AN=71663243&site=ehost-live

ER -

TY - JOUR
ID - 5004
T1 - Mountain Bike Parks for Kids of All Ages
A1 - Vander Pol,Justin
Y1 - 2007/08//
KW - SportDiscus
KW - *PARKS
KW - *MOUNTAIN biking
KW - *MOUNTAIN bikes
KW - *PLAYGROUNDS
KW - United States
RP - NOT IN FILE
SP - 1
EP - 9
JA - Bicycle Paper
VL - 36
IS - 6
N2 - The article deals with the construction of mountain bike parks for children in the U.S. The parks provide endless hours of entertainment in a controlled area. They are designed to help children of all ages progress in their abilities. The parks are all located in populated areas within major residential areas that offer easy access without the need to travel long distances to search for distant trails
SN - 07428308
UR -
https://login.proxy.bib.uottawa.ca/login?url=http://search.ebscohost.com/login.aspx?direct=true&db=s3h&AN=26504515&site=ehost-live
ER -

TY - JOUR
ID - 5011
T1 - Tractor Pull
A1 - Verdi,Bob
Y1 - 2004/07/16/
KW - SportDiscus
KW - *GOLF
KW - *OUTDOOR recreation
KW - *GOLFERS
KW - JOHN Deere tractors
KW - REAL property
KW - PICNICS
RP - NOT IN FILE
SP - 18
EP - 20
JA - Golf World
VL - 58
IS - 4
N2 - The article focuses on "Can You Dig It?" party before the John Deere Classic. This is no ordinary tractor pull that one can see on cable TV at weird hours. Last year's debut of "Can You Dig It?" was well-attended. This year, the number of players, wives, children, friends and caddies doubled, although there is no such thing as overflow on this massive stretch of real estate. It's turned into a company picnic, with golfers interacting with each other and its staff. The John Deere test site is basically an outdoor office that serves as a summer venue for customers and dealers
SN - 00171891
UR -
https://login.proxy.bib.uottawa.ca/login?url=http://search.ebscohost.com/login.aspx?direct=true&db=s3h&AN=14937822&site=ehost-live

ER -

TY - JOUR
ID - 5012
T1 - Girl's book of physical fitness. Completely rewritten and updated
A1 - Vermes,J.C.
Y1 - 1972///
KW - SportDiscus
KW - *CARDIOVASCULAR fitness
KW - *SPORTS
KW - *STRESS (Physiology)
KW - *RECREATION
KW - *BODY weight
KW - *HEALTH
KW - *HYGIENE
KW - *DIET
KW - teenagers
KW - girls
KW - CHILDREN'S literature
KW - Competition
RP - NOT IN FILE
CY - New York;
PB - Association Press
SN - 0809618516
UR -
https://login.proxy.bib.uottawa.ca/login?url=http://search.ebscohost.com/login.aspx?direct=true&db=s3h&AN=SPH22315&site=ehost-live
ER -

TY - JOUR
ID - 5013
T1 - HOJA IN TEK NA SMUŽOEH ZA MLAJ • E OTROKE. / CROSS -COUNTRY WALKING AND SKIING FOR SMALL CHILDREN
A1 - Videm • ek,Mateja
A1 - Pi • ot,Rado
A1 - Flisek,Mojmir
A1 - Pustovrh,Janez
Y1 - 2010/12//
KW - SportDiscus
KW - *CROSS-country skiing
KW - *INDIVIDUAL sports
KW - *WINTER sports
KW - *SPORTS for children
KW - *RECREATION
KW - CHILDREN
KW - cross-country walking and skiing
KW - Learning
KW - Parents
KW - Teachers
KW - hoja in tek na smuŽ_eh
KW - otroci
KW - star • i
KW - uŽ_enje
KW - uŽ_itelji
RP - NOT IN FILE
SP - 126
EP - 129

JA - Sport: Revija Za Teoreticna in Prakticna Vprasanja Sporta
VL - 58
IS - 3/4
N2 - Cross-country skiing has become an increasingly popular outdoor sport activity of adults in winter time. Due to the many positive effects of this type of sport on a human being it is only reasonable that children should also become familiar with it. Teaching of this sport must first and foremost follow the golden rule: children must find cross-country walking and skiing fun! It is only in this way that a seed of desire and need for a pleasant and healthy winter recreational activity will be planted in children, so that it will continue developing also at an older age. ABSTRACT FROM AUTHOR
SN - 03537455
UR -
https://login.proxy.bib.uottawa.ca/login?url=http://search.ebscohost.com/login.aspx?direct=true&db=s3h&AN=57337616&site=ehost-live
ER -

TY - JOUR
ID - 5014
T1 - Pomen otroskega igrisca za otrokov razvoj. / The playground's role in a child's development
A1 - Videmsek,Mateja
A1 - Hosta,Milan
A1 - Bucar,Maja
A1 - Cuk,Ivan
Y1 - 2007///
KW - SportDiscus
KW - *PLAY
KW - *PLAYGROUNDS
KW - Child Development
KW - Child
RP - NOT IN FILE
SP - 3
EP - 5
JA - Sport: Revija Za Teoreticna in Prakticna Vprasanja Sporta
VL - 55
IS - 1
CY - ;
N2 - In the modern civilized world children's play has become increasingly static, resulting in a higher number of contemporary diseases. Given that the need for movement and playing are a child's basic needs, children should be offered opportunities for active and healthy leisure time. One such possibility is the children's playground which has to be expertly designed in terms of safety, usability and comfort. A quality playground must consist of play structures that meet all applicable standards, be properly maintained and interesting and appealing to children. Well-designed and adequately equipped children's playgrounds near kindergartens, schools and in residential areas may play an important role in the physical, motor, cognitive, emotional and social development of children. [ABSTRACT FROM AUTHOR]
SN - 03537455
UR -
https://login.proxy.bib.uottawa.ca/login?url=http://search.ebscohost.com/login.aspx?direct=true&db=s3h&AN=SPHS-1062813&site=ehost-live;http://articles.sirc.ca/search.cfm?id=S-1062813;http://www.fsp.uni-lj.si/rsport/
L2 - http://articles.sirc.ca/search.cfm?id=S-1062813
ER -

TY - JOUR
ID - 5015
T1 - Advocacy Update: Reducing Obesity Through Recreation
A1 - Vinluan,Monica Hobbs
Y1 - 2005/10//
KW - SportDiscus
KW - *OBESITY

KW - *PARKS
KW - *PHYSICAL fitness
KW - COMMUNITY centers
KW - POLICY sciences
KW - United States
RP - NOT IN FILE
SP - 16
EP - 18
JA - Parks & Recreation
VL - 40
IS - 10
N2 - The article focuses on the need of increased federal funding to parks and recreation in order to support efforts to reduce health problems. Public parks and recreation facilities offer low-cost opportunities to all Americans of every age, ability and income level to increase their daily amount of physical activity and thus prevent chronic health conditions. Also, most physical activity among children occurs outside of the school setting. Community sports and recreation programs can complement the efforts of schools by providing children opportunities to engage in the types and levels of physical activity that may not be offered in Federal policymakers have just begun to develop initiatives to combat obesity and to promote the general population's health and well-being
SN - 00312215
UR - https://login.proxy.bib.uottawa.ca/login?url=http://search.ebscohost.com/login.aspx?direct=true&db=s3h&AN=18581601&site=ehost-live
ER -

TY - JOUR
ID - 5016
T1 - Advocacy Update: Promoting a Summer of PLAY
A1 - Vinluan,Monica Hobbs
A1 - Adamson,Katie Clarke
A1 - Burgeson,Charlene
Y1 - 2007/08//
KW - SportDiscus
KW - *EXERCISE for children
KW - *CHILDREN -- Health
KW - *OUTDOOR recreation for children
KW - *YOUNG Men's Christian associations
KW - NATIONAL Recreation & Park Association
KW - United States
RP - NOT IN FILE
SP - 16
EP - 19
JA - Parks & Recreation
VL - 42
IS - 8
N2 - This article looks at the exercise needs of America's youth and how best to make sure every child has an opportunity for at least one hour of exercise per day. A partnership between the Young Men's Christian Association (YMCA) and the National Recreation and Parks Association (NRPA) has created a program called Partnership for PLAY Every Day which has been instituted in Boulder, Colorado and Fort Worth, Texas
SN - 00312215
UR - https://login.proxy.bib.uottawa.ca/login?url=http://search.ebscohost.com/login.aspx?direct=true&db=s3h&AN=26159040&site=ehost-live
ER -

TY - JOUR
ID - 5017

T1 - Fitness education for children: a team approach
A1 - Virgilio,S.J.
Y1 - 1997///
KW - SportDiscus
KW - *PHYSICAL fitness
KW - *CARDIOVASCULAR fitness
KW - *TEACHING
KW - *PHYSICAL education & training
KW - PROGRAMS
KW - Child
RP - NOT IN FILE
CY - Champaign, Ill.;
PB - Human Kinetics Publishers
N2 - CONTENTS: [1] A new perspective in elementary physical education: how physical activity affects health and mortality, healthy people 2000, Surgeon General's report on physical activity and health, components of health-related physical fitness, sport-related physical fitness, what is fitness education?, summary; [2] A team approach to fitness education: mixed messages, a multidisciplinary team approach, health assessment, authentic assessment, summary; [3] Behavioral change and motivational strategies: rainbow to youth fitness and active lifestyles, what motivates children to move?, types of motivation, motivational strategies, summary; [4] Teaching strategies: traditional models of teaching, a humanistic approach to teaching fitness, the spectrum of teaching styles, class structure, summary; [5] Principles of health-related physical fitness: core principles of health-related physical fitness, stages of a physical activity session, components of health-related physical fitness, summary; [6] Fitness education for children with physical disabilities: individualized education plan, guidelines for inclusion, obesity, asthma, spinal cord impairments, mental retardation, summary; [7] Getting parents and your community involved: establishing a plan of action, communication, parent education, parent participation during school hours, home-based activities, community involvement, summary; [8] Planning for fitness: sample yearly plan for developmental level III, sample lesson plans, fitness education lessons, fitness integration lessons, summary; [9] Teaching health-related physical fitness concepts: strategies for teaching fitness concepts, scope and sequence, developmental level I fitness concepts: fitness is fun, developmental level II fitness concepts: the best I can be, developmental level III fitness concepts: let's get heart smart, summary; [10] Collaborating with the classroom teacher: collaborating with the classroom teacher, communication strategies, thematic units, an approach to integrated learning, cardiovascular health: a thematic unit, health-related physical fitness classroom activities, developmental level I classroom activities, developmental level II classroom activities, developmental level III classroom activities, summary; [11] Developmental exercises: cardiorespiratory endurance, muscular strength and muscular endurance, flexibility, exercises to avoid, summary; [12] Active games: characteristics of developmental games, promoting physical activity through active games, developmental level I games, developmental level II games, developmental level III games, summary; [13] Dance and rhythmic activities: safety precautions, planning developmentally appropriate activities, developmental level I dance and rhythmic activities, developmental level II dance and rhythmic activities, developmental level III dance and rhythmic activities, summary; [14] School-wide events: fitness field day, fit for life family night, geography run, school health fair, early bird stretch and afternoon perk-up, jump rope for heart, hoops for heart, and step for heart, ACES: all children exercising simultaneously, recess workouts, fitness clubs, principal walks, holiday classics, summary; [Appendix A] : sample personal fitness education portfolio; [Appendix B] Additional resources
SN - 0873227239
UR -
https://login.proxy.bib.uottawa.ca/login?url=http://search.ebscohost.com/login.aspx?direct=true&db=s3h&AN=SPH412021&site=ehost-live
ER -

TY - JOUR
ID - 5018
T1 - Zur Problematik der Korrespondenz des kindgemaessen Geraetturnens in der Schule und des freien Spiels an Pausenhofgeraeten
A1 - Voss-Achim,H.
Y1 - 1976///
KW - SportDiscus

KW - *PHYSICAL education & training
KW - *GYMNASTICS
KW - *PLAY
KW - *RECESSES
KW - equipment
KW - Child
RP - NOT IN FILE
CY - ;
UR -
https://login.proxy.bib.uottawa.ca/login?url=http://search.ebscohost.com/login.aspx?direct=true&db=s3h&AN=SPH131045&site=ehost-live;http://articles.sirc.ca/search.cfm?id=131045
L2 - http://articles.sirc.ca/search.cfm?id=131045
ER -

TY - JOUR
ID - 5020
T1 - Study of free-play patterns of elementary-school age children in playground equipment areas
A1 - Wade,G.R.
Y1 - 1968///
KW - SportDiscus
KW - *PLAY
KW - *PLAYGROUNDS
KW - *GAMES
KW - Schools
KW - Elementary Schools
KW - Child
KW - equipment
RP - NOT IN FILE
CY - Eugene, Ore.;
PB - Univ. of Oregon
UR -
https://login.proxy.bib.uottawa.ca/login?url=http://search.ebscohost.com/login.aspx?direct=true&db=s3h&AN=SPH15212&site=ehost-live;http://kinpubs.uoregon.edu/
ER -

TY - JOUR
ID - 5021
T1 - WHAT IS THE MINIMUM STANDARD OF CARE THAT THE PLAYGROUND OWNER MUST PROVIDE?
A1 - Wadell,Keith A.
Y1 - 2001/04//
KW - SportDiscus
KW - *PLAYGROUNDS
KW - *RECREATION leaders
KW - *ACCIDENT prevention
KW - SAFETY measures
KW - United States
RP - NOT IN FILE
SP - 80
JA - Parks & Recreation
VL - 36
IS - 4
N2 - Discusses the safety standards that the law requires from playground owners in the United States. Importance of the standards for the safety of children; Adequate and proper environment; Systematic ongoing inspection program; Types of equipment which can be used in playgrounds
SN - 00312215

UR -
https://login.proxy.bib.uottawa.ca/login?url=http://search.ebscohost.com/login.aspx?direct=true&db=s3h&AN=4333837&site=ehost-live
ER -

TY - JOUR
ID - 5022
T1 - Take it outside: occupational therapy's role in the making the most of recess
A1 - Waite,Andrew
Y1 - 2013/03/25/
N1 - Accession Number: 2012075334. Language: English. Entry Date: 20130524. Revision Date: 20130524. Publication Type: journal article; pictorial. Journal Subset: Allied Health; USA. Special Interest: Occupational Therapy; Pediatric Care. No. of Refs: 9 ref. NLM UID: 9602488
KW - CINAHL
KW - Learning Environment -- In Infancy and Childhood
KW - Pediatric Occupational Therapy -- Methods
KW - Play and Playthings -- In Infancy and Childhood
KW - Child
KW - Child Development
KW - child health
KW - Child,Preschool
KW - Friendship
KW - Health Promotion
KW - Information Resources
KW - Professional Role
RP - NOT IN FILE
SP - 7
EP - 11
JA - OT Practice
VL - 18
IS - 5
SN - 1084-4902
UR - http://search.ebscohost.com/login.aspx?direct=true&db=cin20&AN=2012075334&site=ehost-live
ER -

TY - JOUR
ID - 5023
T1 - Participation in sport and active leisure by New Zealand children and adolescents
A1 - Walker,S.
A1 - Ross,J.
A1 - Gray,A.
Y1 - 1999///Winter
KW - SportDiscus
KW - *PHYSICAL fitness
KW - *PARTICIPATION
KW - *SPORTS
KW - *CARDIOVASCULAR fitness
KW - *LEISURE
KW - teenagers
KW - New Zealand
KW - Child
RP - NOT IN FILE
SP - 4
EP - 8
JA - Journal of Physical Education New Zealand
VL - 32
IS - 1

CY - ;
N2 - The amount of sport and physically active leisure undertaken by 1518, five to seventeen year-old New Zealanders was surveyed. The percentage of participants who met the recommended physical activity guideline that they undertake at least thirty minutes of moderate intensity physical activity on most days of the week was determined. Sixty nine percent of the children and adolescents met this guideline; this percentage is comparable to other international studies. Children and adolescents undertook, on average, 6.2 hours per week of sport and active leisure with boys undertaking significantly (p < .01) more hours of activity compared with girls. Similarly, significantly more Maori (75 % - p < .05) and young people from a European background (69 % - p < .10) achieved the recommended guidelines compared with young people of other cultures (58 %). Physical activity declined with increasing age, with the most dramatic decrease in activity seen in the 16-17 year-old age group. Seventy three percent of those surveyed participated in sport or active leisure at school (during school hours), with 24 % taking part in activities organised by the school outside normal school hours. Thirty four percent of young people took part in sports and activities organised by non-school based clubs, with significantly more boys involved in club sports and activities than girls. The most popular activities undertaken with clubs with swimming and ball sports like soccer, netball and rugby. Sixty five percent of young people took part in sports and activities with family and/or friends. Informal activities like swimming, cycling and walking were among the top five activities for both boys and girls. A number of policies and programmes are in place aimed at activating the approximately 30 % of young people who are insufficiently active
SN - 11725958
UR -
https://login.proxy.bib.uottawa.ca/login?url=http://search.ebscohost.com/login.aspx?direct=true&db=s3h&AN=SPHS-62402&site=ehost-live;http://articles.sirc.ca/search.cfm?id=S-62402
L2 - http://articles.sirc.ca/search.cfm?id=S-62402
ER -

TY - JOUR
ID - 5024
T1 - Developmental activities that lead to dropout and investment in sport
A1 - Wall,Michael
A1 - CA't,Jean
Y1 - 2007/02//
KW - SportDiscus
KW - *SPORTS participation
KW - *HOCKEY players
KW - *SPORTS for children
KW - *ATHLETES
KW - *MOTIVATION (Psychology)
KW - *SPORTS -- Psychological aspects
KW - *LONG-term athlete development
KW - *INTRINSIC motivation
KW - TRAINING of
KW - Research
KW - Surveys
RP - NOT IN FILE
SP - 77
EP - 87
JA - Physical Education & Sport Pedagogy
VL - 12
IS - 1
N2 - Background: Studies suggest that expert performance in sport is the result of long-term engagement in a highly specialized form of training termed deliberate practice. The relationship between accumulated deliberate practice and performance predicts that those who begin deliberate practice at a young age accumulate more practice hours over time and would, therefore, have a significant performance advantage. However, qualitative studies have shown that a large amount of sport-specific practice at a young age may lead to negative consequences, such as dropout, and is not necessarily the only path to expert performance in sport. Studies have yet to investigate the activity context, such as the amount of early sport participation, deliberate play and deliberate practice within which dropout occurs. Purpose: To determine whether the nature and amount of

childhood-organized sport, deliberate play and deliberate practice participation influence athletes' subsequent decisions to drop out or invest in organized sport. It was hypothesized that young athletes who drop out will have sampled fewer sports, spent less time in deliberate play activities and spent more time in deliberate practice activities during childhood sport involvement. Participants: The parents of eight current, high-level, male, minor ice hockey players formed an active group. The parents of four high-level, male, minor ice hockey players who had recently withdrawn from competitive hockey formed a dropout group. Data collection: Parents completed a structured retrospective survey designed to assess their sons' involvement in organized sport, deliberate play and deliberate practice activities from ages 6 to 13. Data analysis: A complete data-set was available for ages 6 through 13, resulting in a longitudinal data-set spanning eight years. This eight-year range was divided into three levels of development corresponding to the players' progress through the youth ice hockey system. Level one encompassed ages 6ƒ_"9, level two included ages 10ƒ_"11 and level three covered ages 12ƒ_"13. Descriptive statistics were used to report the ages at which the active and dropout players first engaged in select hockey activities. ANOVA with repeated measures across the three levels of development was used to compare the number of sports the active and dropout players were involved in outside of hockey, the number of hours spent in these sports, and involvement in various hockey-related activities. Findings: Results indicated that both the active and dropout players enjoyed a diverse and playful introduction to sport. Furthermore, the active and dropout players invested similar amounts of time in organized hockey games, organized hockey practices, specialized hockey training activities (e.g. hockey camps) and hockey play. However, analysis revealed that the dropout players began off-ice training at a younger age and invested significantly more hours/year in off-ice training at ages 12ƒ_"13, indicating that engaging in off-ice training activities at a younger age may have negative implications for long-term ice hockey participation. Conclusion: These results are consistent with previous research that has found that early diversification does not hinder sport-specific skill development and it may, in fact, be preferable to early specialization. The active and dropout players differed in one important aspect of deliberate practice: off-ice training activities. The dropout players began off-ice training at a younger age, and participated in more off-ice training at ages 12 and 13 than their active counterparts. This indicates a form of early specialization and supports the postulate that early involvement in practice activities that are not enjoyable may ultimately undermine the intrinsic motivation to continue in sport. Youth sport programs should not focus on developing athletic fitness through intense and routine training, but rather on sport-specific practice... ABSTRACT FROM AUTHOR
SN - 17408989
UR -
https://login.proxy.bib.uottawa.ca/login?url=http://search.ebscohost.com/login.aspx?direct=true&db=s3h&AN=24078640&site=ehost-live
ER -

TY - JOUR
ID - 5025
T1 - An update on the play ground safety movement
A1 - Wallach,Frances
Y1 - 1996/04//
KW - SportDiscus
KW - *PLAYGROUNDS
KW - SAFETY measures
KW - U.S.Consumer Product Safety Commission
KW - United States
RP - NOT IN FILE
SP - 46
JA - Parks & Recreation
VL - 31
IS - 4
N2 - Focuses on the efforts made by owner/operators of public-use playgrounds to make them safer for children, since the United States Consumer Product Safety Commission (CPSC) issued `Handbook for Public Playground Safety'. Number of years it took for the guidelines to be felt by municipalities; CPSC accident statistics; Establishment of the National Playground Safety Institute
SN - 00312215

UR -
https://login.proxy.bib.uottawa.ca/login?url=http://search.ebscohost.com/login.aspx?direct=true&db=s3h&AN=9605202436&site=ehost-live
ER -

TY - JOUR
ID - 5026
T1 - Safe Playgrounds
A1 - Wallach,Frances
Y1 - 2001/04//
KW - SportDiscus
KW - *PLAYGROUNDS
KW - *RECREATION -- Equipment & supplies
KW - *CHILDREN
KW - SAFETY measures
KW - Attitudes
KW - PLAY environments
KW - United States
RP - NOT IN FILE
SP - 140
JA - Parks & Recreation
VL - 36
IS - 4
N2 - Comments on playground safety in the United States. Guidelines from various agencies; Behavior of children when playing; Discussions on several types of equipment
SN - 00312215
UR -
https://login.proxy.bib.uottawa.ca/login?url=http://search.ebscohost.com/login.aspx?direct=true&db=s3h&AN=4333846&site=ehost-live
ER -

TY - JOUR
ID - 5027
T1 - THE QUEST FOR PLAYGROUND SAFETY
A1 - Wallach,Frances
Y1 - 2002/05//
KW - SportDiscus
KW - *PLAYGROUNDS
KW - *RECREATION
KW - *CHILDREN
KW - SAFETY measures
KW - SEPTEMBER 11 Terrorist Attacks,2001
KW - United States
RP - NOT IN FILE
SP - 59
JA - Parks & Recreation
VL - 37
IS - 5
N2 - Introduces a series of articles that focus on playground safety in the U.S. Factors associated with playground injuries; Impact of the September 11, 2001 terrorist attacks in the U.S. on children; Significance of the availability of recreation places and playgrounds for children with traumatic experiences
SN - 00312215
UR -
https://login.proxy.bib.uottawa.ca/login?url=http://search.ebscohost.com/login.aspx?direct=true&db=s3h&AN=6554061&site=ehost-live
ER -

TY - JOUR
ID - 5029
T1 - TEACHING K-12 STUDENTS TO COMBAT OBESITY
A1 - Wallhead,Tristan
Y1 - 2007/10//
KW - SportDiscus
KW - *PHYSICAL education
KW - *OBESITY in children
KW - *OBESITY -- Prevention
KW - *OVERWEIGHT children
KW - *PHYSICAL education teachers
KW - *EXERCISE
KW - *STUDENTS
KW - *HEALTH
KW - *EDUCATION
KW - United States
RP - NOT IN FILE
SP - 26
EP - 28
JA - JOPERD: The Journal of Physical Education, Recreation & Dance
VL - 78
IS - 8
N2 - The article provides information on how physical education teachers can make physical activities enjoyable for students to help them combat obesity in the U.S. The author inferred that the instructor can accomplish this through two mechanisms such as the effective use of lesson time to promote more moderate-to-vigorous physical activity and making the physical education experience motivating for overweight students to choose to be active outside of lesson time. Furthermore, information on how teachers can convince overweight students to participate in exercises after class is also provided
SN - 07303084
UR -
https://login.proxy.bib.uottawa.ca/login?url=http://search.ebscohost.com/login.aspx?direct=true&db=s3h&AN=27031218&site=ehost-live
ER -

TY - JOUR
ID - 5030
T1 - In-school physical activity patterns of primary school learners from disadvantaged schools in South Africa
A1 - Walter,C.M.
Y1 - 2011/12/02/
KW - SportDiscus
KW - *SCHOOL children
KW - *RECREATION
KW - *PHYSICAL education
KW - *PHYSICAL activity
KW - *SEDENTARY lifestyles
KW - Administration
KW - South Africa
KW - Health
KW - Physical Activity
KW - SCHOOL children
RP - NOT IN FILE
SP - 779
EP - 788
JA - African Journal for Physical, Health Education, Recreation & Dance
N2 - Guidelines for the maintenance of health and wellbeing in children recommend at least 60 minutes of moderate-to-vigorous physical activity (MVPA) daily. In South Africa, community sport and recreation facilities and opportunities are meagre in areas previously disadvantaged by apartheid, so schools should be

primary sites for enabling children to meet these recommendations. However, physical education has been marginalized in the curriculum and extra-curricular sports provision has declined. The purpose of this study was to investigate children's school day physical activity patterns in relation to meeting daily recommended guidelines. A total of 112 children (boys n=53; girls n=59), from Grades 3 to 6 (aged 8 to 12 years) from 3 disadvantaged schools participated in the study. Physical activity, representing the whole school day and recess, was measured over 5 days using ActiGraphaccelerometers. Qualitative interviews were conducted on a subsample of 20 learners. The results indicated that children accumulated 35 minutes of in-school MVPA, contributing 58% of the 60 minutes of MVPA a day recommendation. Recess accounted for 37% of in-school MVPA. Boys participated in significantly more in-school MVPA than girls (boys=38.47 Añ16.10 mm, girls=32.59 Añ14.03 mm). Almost 90% of Grade 6 children failed to accumulate 30 minutes of MVPA. Low levels of in-school MVPA among girls and the marked decline at Grade 6 are disturbing. Schools should be pivotal in the provision of physical activity to combat the health risks associated with increasingly sedentary lifestyles. Schools need to increase PA through the provision of quality physical education and find cost effective ways of promoting school-day PA. ABSTRACT FROM AUTHOR
SN - 11174315
UR -
https://login.proxy.bib.uottawa.ca/login?url=http://search.ebscohost.com/login.aspx?direct=true&db=s3h&AN=71912922&site=ehost-live
ER -

TY - JOUR
ID - 5041
T1 - A Property Not A PLAYGROUND
A1 - Weaver,Tracy
Y1 - 2010/06//
KW - SportDiscus
KW - *HORSE farms
KW - *CHILDREN
KW - SAFETY measures
KW - ASPHYXIA in children
KW - HAZARDOUS substances -- Safety measures
KW - Water Supply
RP - NOT IN FILE
SP - 33
EP - 36
JA - Hoofbeats: An Australian Riding, Training & Horse Care Magazine
VL - 32
IS - 1
N2 - The article pinpoints potential hazards for small children on a horse property in order to boost safety. It mentions that any property needs safe storage of chemicals and poisons. It cites that pelleted feeds and powdered supplements can pose as choking hazards. It suggests that the placement of water sources on a livestock property should be considered carefully because children are at risk of drowning
SN - 08118698
UR -
https://login.proxy.bib.uottawa.ca/login?url=http://search.ebscohost.com/login.aspx?direct=true&db=s3h&AN=51365246&site=ehost-live
ER -

TY - JOUR
ID - 5042
T1 - The Role of Biographical Characteristics in Preservice Classroom Teachers' School Physical Activity Promotion Attitudes
A1 - Webster,Collin
A1 - Monsma,Eva
A1 - Erwin,Heather
Y1 - 2010/10//
KW - SportDiscus

KW - *PHYSICAL fitness
KW - *HEALTH education
KW - *HEALTH promotion
KW - *STUDENTS
KW - *HEALTH
KW - STUDENT teachers -- Training of
KW - Educational Programs
KW - Health Promotion
KW - Pediatrics
KW - Physical Activity
KW - Physical Education
KW - Psychology
KW - Teaching
RP - NOT IN FILE
SP - 358
EP - 377
JF - Journal of Teaching in Physical Education
VL - 29
IS - 4
N2 - Recommendations for increasing children's daily physical activity (PA) call on classroom teachers to assume an activist role at school. This study examined relationships among preservice classroom teachers' (PCT; n = 247) biographical characteristics, perceptions and attitudes regarding school PA promotion (SPAP). Results indicated participants who completed SPAP-related college coursework and had PA-related teaching/coaching experiences reported higher SPAP competence. Significant relationships were found among BMI, personal PA competence and SPAP competence in the contexts of PE and extracurricular settings. Personal PA competence and SPAP competence at recess and in the classroom predicted 19% of the variance in SPAP attitudes. Experiences in PA settings and preservice training may have important implications for the overall success of efforts to enhance school PA promotion. ABSTRACT FROM AUTHOR
SN - 02735024
UR -
https://login.proxy.bib.uottawa.ca/login?url=http://search.ebscohost.com/login.aspx?direct=true&db=s3h&AN=55424656&site=ehost-live
ER -

TY - JOUR
ID - 5044
T1 - Last Child in the Woods, First Book in the Field
A1 - Weilbacher,Mike
Y1 - 2009///Winter
N1 - Accession Number: 47733157; Weilbacher, Mike 1; Affiliations: 1 : Executive director of the Lower Merion Conservancy outside Philadelphia, Pennsylvania; Source Info: Winter2010, Issue 87, p3; Thesaurus Term: ENVIRONMENTAL education; Subject Term: BOOKS -- Reviews; Subject Term: NONFICTION; Number of Pages: 6p; Illustrations: 1 Color Photograph, 6 Black and White Photographs; Document Type: Book Review
KW - Environmental Education
KW - BOOKS -- Reviews
KW - NONFICTION
KW - LOUV,Richard,1949-
KW - LAST Child in the Woods (Book)
KW - Reviewer Nominated
RP - NOT IN FILE
SP - 3
EP - 8
JA - Green Teacher
IS - 87
N2 - The article reviews the book "Last Child in the Woods," by Richard Louv
SN - 11921285

M3 - Book Review
UR - http://search.ebscohost.com/login.aspx?direct=true&db=8gh&AN=47733157&site=ehost-live
ER -

TY - JOUR
ID - 5045
T1 - 2007 C. H. McCloy Lecture: "Field of Dreams:" Sport as a Context for Youth Development
A1 - Weiss,Maureen R.
Y1 - 2008/12//
KW - SportDiscus
KW - *SPORTS -- Social aspects
KW - *SPORTS for children -- Coaching
KW - *CHILD development
KW - *PHYSICAL education
KW - *MOTOR ability in children
KW - *SPORTS -- Sociological aspects
KW - YOUTH development
KW - DEVELOPMENTAL psychobiology
KW - United States
KW - Moral Development
KW - Motivation
KW - self-perceptions
KW - social influence
RP - NOT IN FILE
SP - 434
EP - 449
JF - Research Quarterly for Exercise & Sport
JA - Res Q Exerc Sport
VL - 79
IS - 4
N2 - The article focuses on the significance of sports for youth development in the U.S. It says that physical activity contexts primarily include organized sport, school physical education, recreational activities, motor skill development programs, dance, recess, and active transport. It notes of these contexts, organized sport is particularly salient for children and adolescents. Despite its potential to promote social, psychological, and physical development and health-related outcomes, it stresses that the youth sport context is often neglected in the physical activity literature. The author also shares his most important experience as a youth sports coach for a parks and recreation department during his undergraduate and graduate years in California
SN - 02701367
UR -
https://login.proxy.bib.uottawa.ca/login?url=http://search.ebscohost.com/login.aspx?direct=true&db=s3h&AN=35946804&site=ehost-live
ER -

TY - JOUR
ID - 5046
T1 - Rebuilding Hope in Haiti, One Playground at a Time
A1 - Weitzel,Greg A.
Y1 - 2013/03//
KW - SportDiscus
KW - *PLAYGROUNDS
KW - DESIGN & construction
KW - Nonprofit Organizations
KW - Natural Disasters
KW - Homeless Persons
KW - HAITI
RP - NOT IN FILE
SP - 12

EP - 14
JA - Parks & Recreation
VL - 48
IS - 3
N2 - In this article, the author discusses how he helps to secure and rebuild a playground in Carrefour, Haitti while working with a team of health professionals for providing temporary home to injured and homeless Haitians affected by earthquake. He informs about the nonprofit organization Kids Around the World (KIDS) which provide shelter by building playgrounds and deliver food to children in communities affected by poverty and natural disasters
SN - 00312215
UR - https://login.proxy.bib.uottawa.ca/login?url=http://search.ebscohost.com/login.aspx?direct=true&db=s3h&AN=87279517&site=ehost-live
ER -

TY - JOUR
ID - 5049
T1 - Development and Evaluation of a Program to Promote Sportsmanship in Youth Sports
A1 - Wells,Mary Sara
A1 - Ellis,Gary D.
A1 - Paisley,Karen P.
A1 - rthur-Banning,Skye G.
Y1 - 2005///Spring
KW - SportDiscus
KW - *SPORTSMANSHIP
KW - *YOUTH
KW - *PHYSICAL fitness
KW - *BASKETBALL
KW - *ATHLETES
KW - program design
KW - program outcomes
KW - youth sports
RP - NOT IN FILE
SP - 1
EP - 17
JA - Journal of Park & Recreation Administration
VL - 23
IS - 1
N2 - Poor sportsmanship is a serious and growing problem in youth sports. Unpleasant experiences that follow from incidents involving poor sportsmanship can lead children to limit their physical activity, thereby increasing their risk of developing health problems such as obesity and limiting the quality of their lives. In contrast, creating sport experiences that promote sportsmanship can heighten the fun in the activity and encourage children to adopt physical activity as part of their lifestyles. The "Play Hard, Play Fair, Play Fun" (PHPFPF) youth basketball program incorporated elements of prosocial behavior theory to shift the focus from competition to an atmosphere of sportsmanship. Specific techniques were implemented to facilitate sportsmanship behavior among participants and spectators in a youth basketball program, to enhance feelings of cooperation, and to help form relationships among participants on competing teams. Examples of these techniques included pre-game introductions among the players and referees, a signed petition on a large poster board in support of sportsmanship posted prominently outside the gym, large banners and posters promoting sportsmanship on display in the gym, awards to players for good sportsmanship, re-setting the score to zero when the score discrepancy between the two teams became too large, a post-game social event for the players and coaches, and a league website featuring photographs of each team's weekly sportsmanship award winner. Evaluation data showed that such techniques led to positive feelings from the parents about their children's sportsmanship attitudes and provided insight into relationships among fun, age, game outcome, and similarity of ability levels of opposing teams. Overall, participants reported higher levels of fun when the game was close (as opposed to a "blow-out"). The negative impact of a blow-out game on fun levels of participants was most severe for the oldest participants who, statistically, are those on the cusp of dropping-out. This suggests that making efforts to

promote sportsmanship and to balance the skill and ability levels of teams may be critical components to fun and, thus, continued participation. More generally, results demonstrate the effectiveness of the PHPFPF program in enhancing fun and sportsmanship in youth sports. ABSTRACT FROM AUTHOR
SN - 07351968
UR -
https://login.proxy.bib.uottawa.ca/login?url=http://search.ebscohost.com/login.aspx?direct=true&db=s3h&AN=19626722&site=ehost-live
ER -

TY - JOUR
ID - 5051
T1 - A Construcao do Genero no Espaco Escolar. / The gender's construction in the school place
A1 - Wenetz,Ileana
A1 - Stigger,Marco Paulo
Y1 - 2006/01//
KW - SportDiscus
KW - *RECESSES
KW - *PLAY
KW - *SEX role
KW - Schools
KW - Culture
KW - BOYS
KW - girls
KW - Gender Identity
KW - ethnology
KW - Diaries
KW - Methodology
KW - Interviews
KW - Theory
KW - Observation
KW - FOUCAULT,M.
RP - NOT IN FILE
SP - 59
EP - 80
JA - Movimento (Porto Alegre)
VL - 12
IS - 1
CY - ;
N2 - We look for, from the lines of the Cultural and Gender Studies that approach the post-structuralism of Foulcault, to understand how the gender and the sexuality establish different ways of being boys/girls inside the break time play. Considering that these actions take place through relations of power, we look for sought to map and identify them through an ethnographically inspired methodology. We focused on groups of 2nd and 3rd grades. We argue that, in the space of break time a non-official and non-intentional learning takes place, from which or through which the children learn how to be boys and girls. [ABSTRACT FROM AUTHOR] Procuramos, a partir de las vertientes de los Estudios Culturales y de Genero que se aproximan del pos-estructuralismo de Foucault, entender como el genero instituye modos diferenciados de ser nino/a en el recreo escolar. Considerando que ese es un proceso permiado de relaciones de poder, procuramos mapearlas e identificarlas a travez de una metodologia de inspiracion etnografica. (observacion participante y entrevistas) realizada, durante un ano, en una escuela publica de Porto Alegre, RS. Focalizamos un segundo y un tercer grado. Argumentamos que, a traves de los juegos, sucede un aprendizaje no-oficial y no-intencional, a partir del cual los/las ninos/ as apre(e)enden determinadas formas de femenilidad e de masculinidad. [ABSTRACT FROM AUTHOR]
SN - 0104754X
UR -
https://login.proxy.bib.uottawa.ca/login?url=http://search.ebscohost.com/login.aspx?direct=true&db=s3h&AN=SPHS-1068204&site=ehost-live;http://articles.sirc.ca/search.cfm?id=S-1068204;http://www.ufrgs.br/universidadeviva/

L2 - http://articles.sirc.ca/search.cfm?id=S-1068204
ER -

TY - JOUR
ID - 5052
T1 - OUTSIDE LOOKING IN
A1 - Wertheim,L.Jon
Y1 - 2004/11/29/
KW - SportDiscus
KW - *TENNIS players
KW - *TENNIS
KW - *ATHLETES
KW - DEBT
KW - United States
RP - NOT IN FILE
SP - 64
EP - 77
JA - Sports Illustrated
VL - 101
IS - 21
N2 - This article profiles former tennis player, Roscoe Tanner. Rock bottom? That's tough to pinpoint, he says, sighing. So many lows to choose from. Rock bottom might have come when he walked into that Stygian cell in a German prison and realized that for the length of his stay he would be defecating in front of his cellmate. "Man," he says, "that was humiliating." The time he was arrested in front of all those fans at a senior tennis event, that was pretty embarrassing, too. And when his father, once his most ardent supporter, turned uncommunicative, that one really stung. No, wait, I've got it, he says. Here's the lowest moment of his odyssey: Immediately after he finished serving four months in a maximum security county jail in Florida on a grand theft charge, he was transferred to a New Jersey jail where he would serve five months for willfully withholding child support. For two weeks he sat on a bus that zigzagged along the Eastern Seaboard picking up other criminals who were being reassigned from one jail to another. At night the convicts either slept in their seats or, if they were lucky, bunked down at a county jail. They were allotted three meals a day, but there was a catch: Their hands never came out of restraints. Ever try eating McDonald's with your wrists locked at your waist? It's not real pleasant. This is what it came to recently for Tanner. The tennis star who once shared drinks with Prince Rainier on the French Riviera and dined with the Reagans in the White House was sitting on a bus near an exit ramp, positioning his chin at just the right angle so he could eat a Big Mac. It's hard to imagine a less likely candidate to make an absolute mess of things. Tennis players just don't fall into the abyss--especially not a player born to the manor, with a Stanford diploma, a thick Rolodex of connections and, if that weren't endowment enough, movie-star looks and boundless reserves of charm
SN - 0038822X
UR -
https://login.proxy.bib.uottawa.ca/login?url=http://search.ebscohost.com/login.aspx?direct=true&db=s3h&AN=15212747&site=ehost-live
ER -

TY - JOUR
ID - 5053
T1 - Survey of preferences of pediatric physical therapists for assessment and treatment of balance dysfunction in children
A1 - Westcott,S.L.
A1 - Murray,K.H.
A1 - Pence,K.
Y1 - 1998/06//
N1 - Accession Number: 1998063955. Language: English. Entry Date: 19981001. Revision Date: 20091218. Publication Type: journal article; questionnaire/scale; research; tables/charts. Journal Subset: Allied Health; Peer Reviewed; USA. No. of Refs: 28 ref. NLM UID: 8912748
KW - CINAHL
KW - Physical Therapy Assessment -- Methods

KW - Pediatric Physical Therapy -- Methods
KW - Balance,Postural -- Evaluation -- In Infancy and Childhood
KW - Child
KW - Surveys
KW - Descriptive Statistics
KW - chi square test
KW - Vignettes
KW - Questionnaires
KW - Human
RP - NOT IN FILE
SP - 48
EP - 61
JF - Pediatric Physical Therapy
JA - PEDIATR
VL - 10
IS - 2
N2 - The purpose of this study was to determine preferences for assessment and treatment strategies used by pediatric physical therapists (PTs) for balance dysfunction in children. Sixty-seven percent (566 of 843) of a random sample of PTs, stratified by region of the United States, who belonged to the Section on Pediatrics of the American Physical Therapy Association in 1994, and who had agreed to receive outside mailings, returned a mail survey. The respondents were either currently practicing physical therapy or had practiced within the last five years at the time of the survey. In the questionnaire, two case studies were presented, a child with mild balance dysfunction and a child with moderate balance dysfunction. Therapists rated from listed choices: 1) assessments and treatment techniques they would use for these two children, 2) frequency of treatment, and 3) philosophy of therapy. Respondents indicated that they would: 1) most often use nonstandardized balance assessments, 2) use a combination of functional and more traditional treatment activities, 3) adjust frequency of treatment based on treatment setting and the child's severity of dysfunction, and 4) most often identify with the neurodevelopmental (NDT) philosophy of treatment. No relationships were found between geographical region and treatment philosophy. A significant relationship was found between treatment philosophy and years of pediatric PT experience. PTs with more experience indicated a preference for the NDT philosophy as compared with a motor learning philosophy chi square = 15.4, $p < 0.05$). These results can be used to understand pediatric PTs' current practice related to assessment and treatment of balance dysfunction in children and to design studies that compare the effectiveness of traditional assessment and treatment strategies for balance deficits with newly proposed strategies
SN - 0898-5669
AD - Associate Professor, Allegheny University of the Health Sciences, Department of Physical Therapy, MS 502, Broad and Vine, Philadelphia, PA 19102-1192. E-mail address: westcotts@auhs.edu
UR - http://search.ebscohost.com/login.aspx?direct=true&db=cin20&AN=1998063955&site=ehost-live
ER -

TY - JOUR
ID - 5054
T1 - William Buckingham Curtis: The Founding Father of American Amateur Athletics, 1837-1900
A1 - Wettan,Richard G.
A1 - Willis,Joe D.
Y1 - 1977///Winter
KW - SportDiscus
KW - *ATHLETES
KW - *AMATEUR sports
KW - *ROWING
KW - *SPORTS
KW - *ATHLETICS
KW - *PHYSICAL fitness
KW - *PHYSICAL education
KW - *OUTDOOR recreation
RP - NOT IN FILE
SP - 28

EP - 37
JA - Quest (00336297)
VL - 27
N2 - The article discusses the life of William Buckingham Curtis as an athlete, sports official, sports editor, organizer and administrator. William Buckingham Curtis is regarded as the father of American Amateur Athletics. His childhood was flawed by poor health and his parents did not vastly support him in his athletic inclination. He became sports and managing editor of "Spirit of the Times." He actively joins sports competition and he excelled in rowing, wherein he won a three mile single scull match in New York
SN - 00336297
UR -
https://login.proxy.bib.uottawa.ca/login?url=http://search.ebscohost.com/login.aspx?direct=true&db=s3h&AN=20079872&site=ehost-live
ER -

TY - JOUR
ID - 5055
T1 - Some observations of interest in locomotor and gross motor activities in the nursery school
A1 - Wetton,P.
Y1 - 1983///
KW - SportDiscus
KW - *PLAY
KW - *LOCOMOTION
KW - *TEACHING
KW - PERCEPTUAL-motor processes
KW - INFANTS
KW - Nursery Schools
KW - BOYS
KW - girls
KW - Learning
KW - SEX FACTOR
KW - Observation
KW - motor skill
RP - NOT IN FILE
CY - ;
N2 - Regardless of the nature of the environment the 4 year old children observed displayed a need to move. Wandering was an important activity for these children. The boys displayed a higher incidence of chasing and other vigorous activities that the girls. More teacher direction is seen to be needed to incorporate other skills being taught at this level
SN - 01407708
UR -
https://login.proxy.bib.uottawa.ca/login?url=http://search.ebscohost.com/login.aspx?direct=true&db=s3h&AN=SPH145905&site=ehost-live;http://articles.sirc.ca/search.cfm?id=145905
L2 - http://articles.sirc.ca/search.cfm?id=145905
ER -

TY - JOUR
ID - 5056
T1 - Father's Day Festivities
A1 - Wheaton,Mike
Y1 - 2008/09//
KW - SportDiscus
KW - *OUTDOOR recreation for children with disabilities
KW - *OUTDOOR recreation
KW - *PEOPLE with disabilities
KW - United States
KW - FATHER'S Day
KW - Colorado

RP - NOT IN FILE
SP - 50
EP - 50
JA - PN
VL - 62
IS - 9
N2 - The article reports on the three outdoor recreational activities that the disabled Coloradans look forward to in celebrating the Father's Day event every the 14th day of June. They include an annual picnic and family fishing day at Swift Ponds in Windsor, Colorado, a fishing day activity for children with disabilities sponsored by the Wild Turkey Federation's and Wheelin' Sportsman's Mount Evans chapter, and an annual "Family shoot" in Aurora sponsored by the Sunrise Disabled Hunters Program
SN - 00311766
UR -
https://login.proxy.bib.uottawa.ca/login?url=http://search.ebscohost.com/login.aspx?direct=true&db=s3h&AN=33590400&site=ehost-live
ER -

TY - JOUR
ID - 5058
T1 - Feelings of restoration from recent nature visits
A1 - White,Mathew P.
A1 - Pahl,Sabine
A1 - Ashbullby,Katherine
A1 - Herbert,Stephen
A1 - Depledge,Michael H.
Y1 - 2013///
KW - Reviewer Nominated
RP - NOT IN FILE
JF - Journal of Environmental Psychology
PB - Elsevier
SN - 0272-4944
ER -

TY - JOUR
ID - 5059
T1 - Young Children's Relationship with Nature: Its Importance to Children's Development & the Earth's Future
A1 - White,Randy
Y1 - 2006///Fall
KW - SportDiscus
KW - *OUTDOOR education
KW - *PLAYGROUNDS
KW - Child Development
KW - Child
RP - NOT IN FILE
SP - 12
EP - 18
JA - Taproot (Cortland, N.Y.)
VL - 16
IS - 2
CY - ;
N2 - The importance of nature in child development is highlighted in this article. A brief history of children and nature and how the lives of children have changes over time is provided. The role of the naturalization of playgrounds in the development of empathy and feeling comfortable in nature is described
SN - 10655204
UR -
https://login.proxy.bib.uottawa.ca/login?url=http://search.ebscohost.com/login.aspx?direct=true&db=s3h&AN=

SPHS-1046499&site=ehost-live;http://articles.sirc.ca/search.cfm?id=S-1046499;http://www.outdooredcoalition.org
L2 - http://articles.sirc.ca/search.cfm?id=S-1046499
ER -

TY - JOUR
ID - 5063
T1 - Spring Into Safety
A1 - Wilkinson,R.I.
Y1 - 2001/04//
KW - SportDiscus
KW - *OUTDOOR recreation
KW - *PLAYGROUNDS
KW - SAFETY measures
KW - NATIONAL Recreation & Park Association
RP - NOT IN FILE
SP - 2
JA - Parks & Recreation
VL - 36
IS - 4
N2 - Deals with the activities of the National Playground Safety Institute. Expectations regarding the recreation activities of children during the spring season; Certification for playground inspectors; Role of the National Recreation and Park Association
SN - 00312215
UR - https://login.proxy.bib.uottawa.ca/login?url=http://search.ebscohost.com/login.aspx?direct=true&db=s3h&AN=4333633&site=ehost-live
ER -

TY - JOUR
ID - 5064
T1 - Sport is Part of our Free Time
A1 - Wille,U.
Y1 - 1981///
KW - SportDiscus
KW - *SPORTS facilities
KW - *CARDIOVASCULAR fitness
KW - *PHYSICAL education & training
KW - *SPORTS
KW - *PARTICIPATION
KW - teenagers
KW - Culture
KW - SOCIAL policy
KW - GERMANY (East)
KW - Child
RP - NOT IN FILE
SP - 18
EP - 23
JA - Sports in the GDR
VL - 3
N2 - Examines physical activity and sport for children in the German Democratic Republic. Discusses constitutional commitments, facilities, school sport involvement, school sports clubs and the DTSB, a program for children outside of school. Analyzes the different competition schemes available for children, suggesting that 95% of schoolchildren are involved. Traces the development of the Spartakiad Movement, held at local and national levels since 1965. States that even holidays include planning for physical activity

UR -
https://login.proxy.bib.uottawa.ca/login?url=http://search.ebscohost.com/login.aspx?direct=true&db=s3h&AN=SPH332801&site=ehost-live;http://articles.sirc.ca/search.cfm?id=332801
L2 - http://articles.sirc.ca/search.cfm?id=332801
ER -

TY - JOUR
ID - 5066
T1 - Changing Times for Outdoor Learning in Wales: Outdoor Learning Cards, Adventurous Activities and Physical Education
A1 - Williams,Andy
A1 - Wainwright,Nadia
Y1 - 2011///Winter
KW - SportDiscus
KW - *OUTDOOR education
KW - *PHYSICAL education -- Study & teaching
KW - *SCHOOL children
KW - Elementary School Teachers
KW - Questionnaires
KW - INTERVIEWING
KW - Wales
RP - NOT IN FILE
SP - 30
EP - 33
JA - Horizons (14620677)
IS - 56
N2 - The article reports on the result of a small-scale study regarding the impact of Outdoor Learning Cards training courses on the learning and teaching of adventurous activities as part of Physical Education among 8-15 year old students in Wales. The study uses data generated from a questionnaire and semi-structured interviews of 90 teachers. The study reveals that the Outdoor Learning Cards have positive impact on learning and teaching in Wales
SN - 14620677
UR -
https://login.proxy.bib.uottawa.ca/login?url=http://search.ebscohost.com/login.aspx?direct=true&db=s3h&AN=70377908&site=ehost-live
ER -

TY - JOUR
ID - 5067
T1 - Arkansas and Mississippi superintendents' attitudes toward K-6 physical education in the public school
A1 - Williams,L.G.
Y1 - 1998///
KW - SportDiscus
KW - *PHYSICAL education & training
KW - *TRAINING
KW - *MANAGERS of sports teams
KW - *EDUCATION
KW - Schools
KW - Elementary Schools
KW - ARKANSAS
KW - Mississippi
KW - rural
RP - NOT IN FILE
CY - Eugene, Or.;
PB - Microform Publications, University of Oregon
N2 - One objective of Healthy People 2000 is to increase physical education class time by at least 50% to allow students to spend more time in physical activity. In addition, the Council on Physical Education for Children

(1992) indicated that children should be provided recurrent and meaningful age-appropriate practice times to develop understanding of movement concepts. This study consisted of 97 Arkansas superintendents and 91 Mississippi superintendents who volunteered to participated and were given the Superintendents' Attitudes Toward Physical Education questionnaire (SATPE) which asked about their attitudes toward K-6 physical education in the public school. Both Arkansas and Mississippi are considered to be rural states and were found to be similar in size according to the mean number of students enrolled in each school and a compatible match was assumed. This allowed for a comparison between Arkansas, which has state mandates requiring elementary physical education, and Mississippi which has no state mandates for the offering of elementary physical education programs. A Pearson's correlation, t-test, regression, and factoral analysis were performed on the data. The factor structure of 28 questionnaire items yielded five factor components. These factor components were named implementation of programs, barriers to implementation, requirements for implementation, benefits of physical education programs, and physical education curriculum concerns. The results indicated that a greater number of Arkansas superintendents held bachelor's degrees in physical education and a greater number of Mississippi superintendents held bachelor's degrees in social studies. Although a greater number of Arkansas superintendents held degrees in physical education, it was revealed that they are less likely to support any aspect of elementary physical education. In addition, the longer Arkansas superintendents were in education the less likely they were to support K-6 physical education. On the other hand, Mississippi superintendents were more likely to support elementary physical education programs the longer they were in education, even though most did not have a physical education background. Mississippi superintendents were also more likely to purchase equipment, books, and workbooks for schools, teachers, and students. Mississippi superintendents were also interested in paying a certified physical education specialist from outside the district to train classroom teachers in physical education curriculum. The SATPE, Appendixes, and references are included
UR -
https://login.proxy.bib.uottawa.ca/login?url=http://search.ebscohost.com/login.aspx?direct=true&db=s3h&AN=SPHS-170994&site=ehost-live;http://kinpubs.uoregon.edu/
ER -

TY - JOUR
ID - 5070
T1 - Encounters with Nature as a Path of Self-Realisation: A Meaning-Making Framework
A1 - Wilson,Michael
Y1 - 2011///
KW - Reviewer Nominated
RP - NOT IN FILE
SP - 11
EP - 29
JA - Journal of Transpersonal Research
VL - 3
IS - 1
SN - 1989-6077
ER -

TY - JOUR
ID - 5071
T1 - Diet Quality and Weight Change among Overweight and Obese Postpartum Women Enrolled in a Behavioral Intervention Program
A1 - Wiltheiss,Gina A.
A1 - Lovelady,Cheryl A.
A1 - West,Deborah G.
A1 - Brouwer,Rebecca J.N.
A1 - Krause,Katrina M.
A1 - A~stbye,Truls
Y1 - 2013/01//
KW - SportDiscus
KW - *PUERPERIUM
KW - *WEIGHT gain
KW - *WEIGHT loss

KW - *LACTATION
KW - *PHYSICAL activity
KW - *MOTIVATIONAL interviewing
KW - *OBESITY -- Prevention
KW - *OBESITY
KW - *BREASTFEEDING (Humans)
KW - *DIET
KW - *EXERCISE
KW - *FOOD habits
KW - *PSYCHOLOGICAL tests
KW - *REDUCING diets
KW - Risk Factors
KW - Analysis of Variance
KW - Multivariate Analysis
KW - Treatment
KW - Behavior Modification
KW - CHI-squared test
KW - Clinical Trials
KW - CORRELATION (Statistics)
KW - Income
KW - ingestion
KW - Mothers
KW - patient education
KW - PROBABILITY theory
KW - Questionnaires
KW - RESEARCH -- Finance
KW - Statistics
KW - data analysis
KW - MULTIPLE regression analysis
KW - Edinburgh Postnatal Depression Scale
KW - FOOD diaries
KW - HUMAN services programs -- Evaluation
KW - Descriptive Statistics
KW - North Carolina
RP - NOT IN FILE
SP - 54
EP - 62
JF - Journal of the Academy of Nutrition & Dietetics
JA - J Acad Nutr Diet
VL - 113
IS - 1
N2 - Background: Postpartum weight retention is a risk factor for long-term weight gain. Encouraging new mothers to consume a healthy diet may result in weight loss. Objective: To assess predictors of diet quality during the early postpartum period; to determine whether diet quality, energy intake, and lactation status predicted weight change from 5 to 15 months postpartum; and to determine whether an intervention improved diet quality, reduced energy intake, and achieved greater weight loss compared with usual care. Design: Randomized clinical trial (KAN-DO: Kids and Adults Nowƒ_"Defeat Obesity), a family- and home-based, 10-month, behavioral intervention to prevent childhood obesity, with secondary aims to improve diet and physical activity habits of mothers to promote postpartum weight loss. Participants: Overweight/obese, postpartum women (n=400), recruited from 14 counties in the Piedmont region of North Carolina. Intervention: Eight education kits, each mailed monthly; motivational counseling; and one group class. Methods: Anthropometric measurements and 24-hour dietary recalls collected at baseline (approximately 5 months postpartum) and follow-up (approximately 10 months later). Diet quality was determined using the Healthy Eating Index-2005 (HEI-2005). Statistical analyses: Descriptive statistics, IØ2, analysis of variance, bi- and multivariate analyses were used. Results: At baseline, mothers consumed a low-quality diet (HEI-2005 score=64.4Ąñ11.4). Breastfeeding and income were positive, significant predictors of diet quality, whereas body mass index was a negative predictor. Diet quality did not predict weight change. However, total energy intake, not working

outside of the home, and breastfeeding duration/intensity were negative predictors of weight loss. There were no significant differences in changes in diet quality, decreases in energy intake, or weight loss between the intervention (2.3Añ5.4 kg) and control (1.5Añ4.7 kg) arms. Conclusions: The family-based intervention did not promote postpartum weight loss. Reducing energy intake, rather than improving diet quality, should be the focus of weight-loss interventions for overweight/obese postpartum women. Copyright &y& Elsevier
SN - 22122672
UR -
https://login.proxy.bib.uottawa.ca/login?url=http://search.ebscohost.com/login.aspx?direct=true&db=s3h&AN=84478389&site=ehost-live
ER -

TY - JOUR
ID - 5072
T1 - Mothers meeting
A1 - Wimbush,E.
Y1 - 1988///
KW - SportDiscus
KW - *LEISURE
KW - WOMEN -- Sociological aspects
KW - Women
KW - Mothers
KW - Scotland
RP - NOT IN FILE
CY - ;
N2 - Explores the significance of leisure in the lives of women with young children in Edinburgh, Scotland. States that across all ethnic groups, it is the mother, rather than the father, who makes extensive changes to her life when children are born. Reports that many mothers with young children have no energy left at the end of a day for activities outside the home, and that many women rely on contacts with other mothers during the day for socializing and support. Identifies exit routes' which provide a break from children and domestic routines a day off to shop, a night out with girlfriends, a part-time job - and addresses the constraints to enjoying these activities. Focuses on the role that other women play in providing support. Concludes that for mothers, time off from work goes hand in hand with the struggle for improved childcare provisions and renegotiating the division of this labour
UR -
https://login.proxy.bib.uottawa.ca/login?url=http://search.ebscohost.com/login.aspx?direct=true&db=s3h&AN=SPH230623&site=ehost-live;http://articles.sirc.ca/search.cfm?id=230623
L2 - http://articles.sirc.ca/search.cfm?id=230623
ER -

TY - JOUR
ID - 5073
T1 - Raising Children Who Love to Play Outside: A Field-Tested Model
A1 - Wirth,Susan
Y1 - 2009///
N1 - Accession Number: 54435403; Wirth, Susan; Email Address: swirth@arbordayfarm.org; Source Info: 2009 Annual Meeting, p1; Subject Term: CHILDREN -- Conduct of life; Subject Term: ROLES (Social aspects); Subject Term: CHILDREN -- Attitudes; Subject Term: ETIQUETTE for children & teenagers; Subject Term: SOCIAL adjustment; Number of Pages: 0p; Document Type: Article
KW - CHILDREN -- Conduct of life
KW - ROLES (Social aspects)
KW - CHILDREN -- Attitudes
KW - ETIQUETTE for children & teenagers
KW - Social Adjustment
KW - Reviewer Nominated
RP - NOT IN FILE
SP - 1
JA - Conference Papers -- North American Association of Environmental Education

N2 - Gain research-based, field-tested strategies for engaging families with young children in activities that strengthen nature connections based on an effective early childhood center model. Take home hands-on activities and adaptable materials to organize a Nature Families' Club in your community. ..PAT.-Unpublished Manuscript [ABSTRACT FROM AUTHOR]
Copyright of Conference Papers -- North American Association of Environmental Education is the property of North American Association of Environmental Education and its content may not be copied or emailed to multiple sites or posted to a listserv without the copyright holder's express written permission. However, users may print, download, or email articles for individual use. This abstract may be abridged. No warranty is given about the accuracy of the copy. Users should refer to the original published version of the material for the full abstract. (Copyright applies to all Abstracts.)
M3 - Article
UR - http://search.ebscohost.com/login.aspx?direct=true&db=8gh&AN=54435403&site=ehost-live
ER -

TY - JOUR
ID - 5074
T1 - Tryb zycia uczniow-sportowcow w ocenie higienicznej. / Way of life of sports students in hygenic (sic) appraisal
A1 - Woitas-Slubowska,D.
Y1 - 1993///
KW - SportDiscus
KW - *LIFE style
KW - *NUTRITION
KW - *FOOD -- Caloric content
KW - *LEISURE
KW - *OUTDOOR life
KW - *STRESS (Physiology)
KW - *ATHLETES
KW - sociology
KW - Sleep
KW - Comparative Studies
KW - teenagers
KW - BOYS
KW - girls
KW - Poland
KW - NON-ATHLETE
KW - Child
RP - NOT IN FILE
SP - 53
EP - 62
JA - Wychowanie Fizyczne i Sport
VL - 37
IS - 1
CY - ;
N2 - Good sports results are determined by many factors of which most essential is perfect health. In order to enjoy it, it is necessary that, among others, the way of life should meet hygiene requirements. The questionnaire investigation covered 142 elementary and secondary school students practising sport, and a control group of 257 subjects. Investigated were items selected from a day routine: sleep, outdoor stay, number of meals, time of school and homework as well as after-class activities. In groups under investigation there was discerned an incorrect way of life manifesting in: sleep and movement deficiency and too small a number of meals. In the elementary school group those shortcomings were more frequent among sports students than in the control group. This dependence has not been noticed in the investigated group of secondary school students. The conclusion points at organizational possibilities to improve the way of life of children and the youth practising sport
SN - 00439630

UR -
https://login.proxy.bib.uottawa.ca/login?url=http://search.ebscohost.com/login.aspx?direct=true&db=s3h&AN=SPHS-668813&site=ehost-live;http://articles.sirc.ca/search.cfm?id=S-668813
L2 - http://articles.sirc.ca/search.cfm?id=S-668813
ER -

TY - JOUR
ID - 5075
T1 - Experiencing connection with nature: The matrix of psychological well-being, mindfulness, and outdoor recreation
A1 - Wolsko,Christopher
A1 - Lindberg,Kreg
Y1 - 2013///
KW - Reviewer Nominated
RP - NOT IN FILE
SP - 80
EP - 91
JA - Ecopsychology
VL - 5
IS - 2
PB - Mary Ann Liebert, Inc. 140 Huguenot Street, 3rd Floor New Rochelle, NY 10801 USA
N2 - Ecopsychological theory and practice underscore the vital importance of realizing the interdependence between human beings andEcopsychological theory and practice underscore the vital importance of realizing the interdependence between human beings and the diverse living universes that they inhabit. This article focuses on the mental health implications of the shift toward a more ecologically rooted identity by examining relationships between psychological well-being and the personal experience of connection with nature. Three separate surveys conducted with undergraduate and community samples assessed relationships between the Connectedness to Nature Scale (CNS), psychological well-being, mindfulness, and outdoor recreation. Higher CNS scores were consistently associated with greater trait mindfulness, more participation in appreciative outdoor activities, and higher scores on multiple measures of both hedonic and eudaimonic aspects of psychological well-being. Discussion focuses on informing optimal strategies for nature-based interventions. the diverse living universes that they inhabit. This article focuses on the mental health implications of the shift toward a more ecologically rooted identity by examining relationships between psychological well-being and the personal experience of connection with nature. Three separate surveys conducted with undergraduate and community samples assessed relationships between the Connectedness to Nature Scale (CNS), psychological well-being, mindfulness, and outdoor recreation. Higher CNS scores were consistently associated with greater trait mindfulness, more participation in appreciative outdoor activities, and higher scores on multiple measures of both hedonic and eudaimonic aspects of psychological well-being. Discussion focuses on informing optimal strategies for nature- based interventions.
SN - 1942-9347
ER -

TY - JOUR
ID - 5077
T1 - To see the world in a grain of sand..
A1 - Wright,David R.
Y1 - 2009/09//
N1 - Accession Number: 50613670; Wright, David R.; Source Info: Autumn2009, Vol. 92, p10; Thesaurus Term: ENVIRONMENTAL education; Thesaurus Term: ENVIRONMENTAL literacy; Subject Term: LEARNING by discovery; Subject Term: CURRICULA (Courses of study) -- Great Britain; Subject Term: OBSERVATION (Psychology); Subject Term: THEORY of knowledge; Subject Term: COMPREHENSION (Theory of knowledge); Subject Term: SCHOOL children; Subject: GREAT Britain; Number of Pages: 4p; Illustrations: 8 Black and White Photographs; Document Type: Article
KW - Environmental Education
KW - ENVIRONMENTAL literacy
KW - LEARNING by discovery
KW - CURRICULA (Courses of study) -- Great Britain

KW - OBSERVATION (Psychology)
KW - THEORY of knowledge
KW - COMPREHENSION (Theory of knowledge)
KW - SCHOOL children
KW - Great Britain
KW - Reviewer Nominated
RP - NOT IN FILE
SP - 10
EP - 13
JA - Environmental Education
VL - 92
N2 - The article focuses on lessons that can be learned in an ordinary street. The author states that the process of learning real history and geography may happen somewhere else aside from the classrooms. Learning environmental education outside the classroom is marked with a real and comprehensible experience and is applicable to all ages. Students who are four to five years old or 16-years-old have the chance to acquire knowledge and understanding of the world around them while walking in an ordinary street
SN - 03098451
M3 - Article
UR - http://search.ebscohost.com/login.aspx?direct=true&db=8gh&AN=50613670&site=ehost-live
ER -

TY - JOUR
ID - 5078
T1 - Improving healthy eating and physical activity at school
A1 - Wundersitz,Simon
Y1 - 2013/08//
KW - SportDiscus
KW - *FOOD habits
KW - *SCHOOLS
KW - *SCHOOL children -- Nutrition
KW - *HEALTH promotion
KW - Australia
KW - LUNCHBOXES
RP - NOT IN FILE
SP - 17
EP - 18
JA - ACHPER Active & Healthy Magazine
VL - 20
IS - 2
N2 - The article discusses the eat well be active - Primary School Project (EWBA) of the Immanuel Lutheran School Gawler in Gawler, South Australia. The school started the EWBA in 2009 with the objective of gathering data on what students had their lunch boxes. The results showed that many had healthy lunches but ate a lot of processed food for recess. Focus was given on what students ate at recess. Friendly Food Friday was created where students can purchase healthy homemade recess foods
SN - 13287133
UR -
https://login.proxy.bib.uottawa.ca/login?url=http://search.ebscohost.com/login.aspx?direct=true&db=s3h&AN=89777940&site=ehost-live
ER -

TY - JOUR
ID - 5079
T1 - Screen time and physical activity of young children reflects their mother's behaviours: findings from the Healthy Beginnings Trial
A1 - Xu,Huilan
A1 - Wen,Li
Y1 - 2012/08//

N1 - Accession Number: 2011627045. Language: English. Entry Date: 20120824. Revision Date: 20140221. Publication Type: journal article; letter; research. Journal Subset: Australia & New Zealand; Biomedical; Editorial Board Reviewed; Expert Peer Reviewed; Peer Reviewed; Public Health. Special Interest: Pediatric Care; Public Health. Grant Information: Australian National Health and Medical Research Council (ID number: 393112).. NLM UID: 9611095
KW - CINAHL
KW - Physical Activity -- In Infancy and Childhood
KW - Television
KW - Maternal Behavior
KW - Human
KW - Funding Source
KW - Child,Preschool
KW - Cross Sectional Studies -- New South Wales
KW - New South Wales
KW - secondary analysis
KW - Natural Environment
KW - Linear Regression
KW - Guideline Adherence
KW - Confidence Intervals
RP - NOT IN FILE
SP - 396
EP - 396
JF - Australian & New Zealand Journal of Public Health
JA - Aust N Z J Public Health
VL - 36
IS - 4
SN - 1326-0200
AD - Health Promotion Service, South Western Sydney and Sydney Local Health Districts, NSW Health
UR - http://search.ebscohost.com/login.aspx?direct=true&db=cin20&AN=2011627045&site=ehost-live
ER -

TY - JOUR
ID - 5080
T1 - A study on the influence of "children's playground" style of hospitalization environment on their rehabilitation of disease [Chinese]
A1 - Yang,Q.
A1 - Chen,X.
A1 - Chen,L.
Y1 - 2002/06//
N1 - Accession Number: 2002150104. Language: Chinese. Entry Date: 20021101. Revision Date: 20091218. Publication Type: journal article; research; tables/charts. Journal Subset: Asia; Double Blind Peer Reviewed; Editorial Board Reviewed; Expert Peer Reviewed; Nursing; Peer Reviewed. No. of Refs: 3 ref
KW - CINAHL
KW - Child,Hospitalized -- Psychosocial Factors
KW - Health Facility Environment
KW - Play and Playthings
KW - Rehabilitation,Pediatric
KW - T-Tests
KW - Child
KW - Length of Stay
KW - Child Psychology
KW - Human
RP - NOT IN FILE
SP - 356
EP - 357
JA - Chinese Nursing Research
VL - 16

IS - 6
N2 - To probe into the rehabilitation effects of"children's playground" style hospitalization environment acted on, 163 cases of hospitalized children were investigated on their psychological and behavioral problems and medical indexes changes of these children before and after the hospitalization environmental changes were compared with each other. Results: after the hospitalization environment being changed, among the investigated 12 items of psychological and behavioral questions, the fractional value rate of often happening was 2.3% . The fractional value rate of at times happening accounted for 12.5%. The fractional value rate of not happening accounted for 85.2%. The numbers of admissions increased by 318.4%. The rate of satisfaction in patients increased 4% The average hospitalization day of patients were 2.82 days shortened. And the average hospitalization expenses of patients were 45.84 Yuan decreased. Suggested that "children's playground" style of hospitalization environment may help the pediatric patients to recover from their illness. This abstract was translated into English by the publisher or author
SN - 1009-6493
AD - Gongli Hospital of Pudong New District of Shanghai City, Shanghai 200135 China
UR - http://search.ebscohost.com/login.aspx?direct=true&db=cin20&AN=2002150104&site=ehost-live
ER -

TY - JOUR
ID - 5081
T1 - The Epidemiology of United States High School Soccer Injuries, 2005ƒ_"2007
A1 - Yard,Ellen E.
A1 - Shcroeder,Matthew J.
A1 - Fields,Sarah K.
A1 - Collins,Christy L.
A1 - Comstock,R.Dawn
Y1 - 2008/10//
KW - SportDiscus
KW - *EPIDEMIOLOGY
KW - *SOCCER injuries
KW - *SPORTS injuries
KW - *SCHOOL sports
KW - *BRUISES
KW - *LIGAMENTS -- Wounds & injuries
KW - *MUSCLES -- Wounds & injuries
KW - *ATHLETIC trainers
KW - Confidence Intervals
KW - United States
KW - Epidemiology
KW - gender
KW - high school
KW - injury
KW - Soccer
KW - surveillance
RP - NOT IN FILE
SP - 1930
EP - 1937
JF - American Journal of Sports Medicine
JA - Am J Sports Med
VL - 36
IS - 10
N2 - Background: United States high school soccer participation increased 5 fold over the last 30 years. With increased participation comes increased injury incidence. Hypothesis: High school soccer injury patterns will vary by gender and type of exposure. Study Design: Descriptive epidemiologic study. Methods: Soccer-related injury data were collected over the 2005-2006 and 2006-2007 school years from 100 nationally representative United States high schools via Reporting Information Online (RIO, an Internet-based sports-related injury surveillance system). Results: Participating certified athletic trainers reported 1524 soccer injuries during 637 446 athlete exposures (AEs), for an injury rate of 2.39 per 1000 AEs, corresponding to a nationally estimated

807 492 soccer-related injuries during the 2005-2006 and 2006-2007 seasons. The injury rate per 1000 AEs was greater during competition (4.77) than practice (1.37) (rate ratio [RR] = 3.49; 95% confidence interval [CI], 3.15-3.87). Overall, the most frequent diagnoses were incomplete ligament sprains (26.8%), incomplete muscle strains (17.9%), contusions (13.8%), and concussions (10.8%). The most commonly injured body sites were the ankle (23.4%), knee (18.7%), head/face (13.7%), and thigh/upper leg (13.1%). Similar proportions of boys (57.9%) and girls (53.9%) returned to activity in <1 week. During competition, girls sustained complete knee ligament sprains requiring surgery at a rate of 26.4 per 100 000 AEs, higher than the rate among boys during competition (1.98 per 100 000 AEs) (RR = 13.3; 95% CI, 3.15-56.35) and among girls during practice (2.34 per 100 000 AEs) (RR = 11.3; 95% CI, 4.31-29.58). Player-to-player contact was more common among competition injuries (injury proportion ratio [IPR] = 2.42; 95% CI, 2.01-2.92), while noncontact mechanisms were more common among practice injuries (IPR = 2.39; 95% CI, 1.90-3.01). Conclusions: High school soccer injury patterns vary by gender and type of exposure. Identifying such differences in injury patterns is the important first step in the development of evidence-based, targeted injury prevention efforts. ABSTRACT FROM AUTHOR
SN - 03635465
UR -
https://login.proxy.bib.uottawa.ca/login?url=http://search.ebscohost.com/login.aspx?direct=true&db=s3h&AN=34477913&site=ehost-live
ER -

TY - JOUR
ID - 5082
T1 - Effects of Field Location, Time in Competition, and Phase of Play on Injury Severity in High School Football
A1 - Yard,Ellen E.
A1 - Comstock,R.Dawn
Y1 - 2009/01//
KW - SportDiscus
KW - *HIGH school athletes
KW - *HIGH school football players
KW - *FOOTBALL players
KW - *FOOTBALL injuries
KW - *SPORTS competitions
KW - United States
KW - competition intensity
KW - concussion
KW - Epidemiology
KW - kickoff
KW - punt
RP - NOT IN FILE
SP - 35
EP - 49
JF - Research in Sports Medicine
JA - RES SPORTS MED
VL - 17
IS - 1
N2 - This study evaluated the effects of competitive intensity, represented by the variables time in competition, phase of play, and field location, on injury severity in U.S. high school football. The injury rate was higher in competition than practice (RR = 4.75, 95% CI: 4.34-5.20). Mild and moderate injuries were frequently lower leg/foot/ankle sprains/strains and concussions. Severe injuries were frequently knee Sprains/strains and arm fractures. Severe injuries composed a greater proportion of injuries sustained during the beginning and middle of competition compared with injuries sustained during the end/overtime (IPR = 1.83, 95% CI: 1.25-2.69). Compared with injuries sustained during general play, a greater proportion of kickoff/punt injuries were severe (IPR = 1.69, 95% CI: 1.07-2.68) or were concussions (IPR = 1.86, 95% CI: 1.05-3.30). Identifying factors contributing to severe injury is a crucial first step toward developing targeted evidence-based interventions aimed at reducing the incidence of severe injuries among the millions of high school football players. ABSTRACT FROM AUTHOR

SN - 15438627
UR -
https://login.proxy.bib.uottawa.ca/login?url=http://search.ebscohost.com/login.aspx?direct=true&db=s3h&AN=36819594&site=ehost-live
ER -

TY - JOUR
ID - 5083
T1 - Injuries Sustained by Pediatric Ice Hockey, Lacrosse, and Field Hockey Athletes Presenting to United States Emergency Departments, 1990-2003
A1 - Yard,Ellen Elizabeth
A1 - Comstock,R.Dawn
Y1 - 2006/10//
KW - SportDiscus
KW - *SPORTS injuries
KW - *HOCKEY
KW - *LACROSSE
KW - *FIELD hockey
KW - *HIGH school athletes
KW - *WOUNDS & injuries
KW - *BRAIN -- Concussion
KW - Athletic Injuries
KW - Epidemiology
KW - protective equipment
RP - NOT IN FILE
SP - 441
EP - 449
JA - Journal of Athletic Training
VL - 41
IS - 4
N2 - Context: Ice hockey, lacrosse, and field hockey are increasingly popular sports among US youth athletes, but no authors to date have compared injuries in male and female pediatric (ages 2 through 18 years) participants. Objective: To compare patterns of injury among pediatric ice hockey, lacrosse, and field hockey players. Design: A descriptive analysis of all pediatric (ages 2 through 18 years) ice hockey, lacrosse, and field hockey injuries captured by the US Consumer Product Safety Commission's National Electronic Injury Surveillance System. Setting: US Consumer Product Safety Commission's National Electronic Injury Surveillance System. Patients or Other Participants: Children with ice hockey, lacrosse, or field hockey injuries presenting to emergency departments participating in the National Electronic Injury Surveillance System. Main Outcome Measure(s): We reviewed all ice hockey, lacrosse, and field hockey injuries captured by the National Electronic Injury Surveillance System and categorized them by sex, age, injury site, and injury diagnosis. Results: An estimated 321 237 pediatric participants in ice hockey, lacrosse, and field hockey presented to US emergency departments from 1990 through 2003. The injured were primarily male (74.4%) and aged 10 through 18 years (95.4%). Ice hockey accounted for more injuries (53.6%) than lacrosse (26.5%) or field hockey (19.9%). Children aged 2 through 9 years sustained twice the proportion of head and face injuries (53.1%) as children aged 10 through 18 years (23.2%) (incidence rate ratio [IRR] = 2.25, 95% confidence interval [CI] = 1.94 to 2.62). Males incurred a higher proportion of shoulder and upper arm injuries (14.1%) than females (3.1%) (IRR = 4.51, 95% CI = 3.07 to 6.62). The proportion of concussion was higher in ice hockey players (3.9%) than in field hockey players (1.4%) (IRR = 2.75, 95% CI = 1.17 to 6.46). Females in lacrosse had twice the proportion of facial injuries (20.9%) as males (10.5%) (IRR = 1.95, 95% CI = 1.46 to 2.60). In all sports, the ball or puck caused a greater proportion of face injuries in females than in males (IRR = 2.48, 95% CI = 2.03 to 3.05). Facial injuries from falls occurred in higher proportions in ice hockey players (10.6%) than in lacrosse (2.4%) (IRR = 4.32, 95% CI = 1.53 to 12.18) and field hockey (0.4%) players (IRR = 28.38, 95% CI = 6.71 to 120.01). Conclusions: Pediatric ice hockey, lacrosse, and field hockey injuries differed by age and sport and, within each sport, by sex. An understanding of sport-specific patterns of injury should assist coaches and certified athletic trainers in developing targeted preventive interventions. ABSTRACT FROM AUTHOR
SN - 10626050

UR -
https://login.proxy.bib.uottawa.ca/login?url=http://search.ebscohost.com/login.aspx?direct=true&db=s3h&AN=23643164&site=ehost-live
ER -

TY - JOUR
ID - 5084
T1 - Effects of the Coordination Exercise Program on School Children's Agility: Short-Time Program during School Recess
A1 - Yasumitsu,Tatsuo
A1 - Nogawa,Haruo
Y1 - 2011///Fall
KW - SportDiscus
KW - *PHYSICAL training & conditioning
KW - *PHYSICAL fitness
KW - *SCHOOL children
KW - *PHYSIOLOGY
KW - *EXERCISE physiology
KW - *SCHOOL recess breaks
KW - Elementary Schools
KW - Elementary School Students
KW - fitness
RP - NOT IN FILE
SP - 10
EP - 13
JA - ICHPER -- SD Journal of Research in Health, Physical Education, Recreation, Sport & Dance
VL - 6
IS - 2
N2 - This study examined the impact of a short-time coordination program conducted during recess periods on improving agility in elementary school students. The subjects consisted of 62 third grade students, who were randomly assigned to an experimental group (n = 31) and a control group (n = 31). The experimental group completed a coordination program intended to improve agility during 20-minute recesses, three times a week for four weeks, whereas the control group participated in usual activities such as mini soccer games and tag games during the recess. Repetitive side steps, an indicator of agility, were measured for both groups at the beginning and end of the intervention period. While the test scores indicated no difference between the two groups in the pretest, the posttest indicated a significant difference in the test scores in favor of the experimental group, t (60) = 2.27 and p < .027. The results indicated that the short-time coordination program can be effective in improving agility in elementary school students. ABSTRACT FROM AUTHOR
SN - 19304595
UR -
https://login.proxy.bib.uottawa.ca/login?url=http://search.ebscohost.com/login.aspx?direct=true&db=s3h&AN=69880361&site=ehost-live
ER -

TY - JOUR
ID - 5087
T1 - Reexamining the significance of childhood recreation patterns on adult leisure behavior
A1 - Yoesting,D.R.
A1 - Christensen,J.E.
Y1 - 1978///
KW - SportDiscus
KW - *RECREATION
KW - *LEISURE
KW - *PARTICIPATION
KW - sociology
KW - adulthood
KW - Socialization

KW - Interests
KW - life cycle
KW - Child
RP - NOT IN FILE
CY - ;
N2 - Examines the impact of childhood participation levels in outdoor recreation on the activity level of that individual as an adult. A stratified random sample of adult residents in Iowa is utilized with forty-five outdoor recreation activities used to determine the carryover of participation from childhood to adulthood. Results indicate a direct effect of the childhood level of participation on adult level of participation. Implications of education for leisure and aspects of socialization are discussed
SN - 01490400
UR -
https://login.proxy.bib.uottawa.ca/login?url=http://search.ebscohost.com/login.aspx?direct=true&db=s3h&AN=SPH41608&site=ehost-live;http://articles.sirc.ca/search.cfm?id=41608
L2 - http://articles.sirc.ca/search.cfm?id=41608
ER -

TY - JOUR
ID - 5089
T1 - Young adolescents' leisure patterns. / Modeles de loisir chez les jeunes adolescents
A1 - Zeijl,E.
A1 - Du Bois-Reymond,M.
A1 - Te Poel,Y.
Y1 - 2001///
KW - SportDiscus
KW - *LEISURE
KW - *SPORTS
KW - *PARTICIPATION
KW - sociology
KW - teenagers
KW - age
KW - Netherlands
KW - Interests
KW - Child
RP - NOT IN FILE
SP - 379
EP - 402
JA - Loisir et Societe
VL - 24
IS - 2
CY - ;
N2 - Until recently, studies into juveniles' leisure activities concentrated mainly on adolescents. In this study, the leisure activities of both pre-adolescents and adolescents are studied. Departing from sociological debates on the busyness of contemporary young adolescents' leisure diaries and of an advanced onset of the transition from childhood into the youth phase, a questionnaire was designed for 10 to 15 year-olds. 927 Dutch youngsters were studied. By means of CATPCA, it was found that 10-12 year-old girls mainly engage in reading and in creative activities, whereas the boys spend most time playing outside. Fourteen and fifteen year-olds, on the other hand, showed a clear interest in youth-4cultural activities, which did not support theoretical notions about the advancement of adolescence at the expense of childhood. Questions on organized activities showed that the market of leisure opportunities is not equally accessible to everyone: higher class kids are in an advantageous position compared to lower class kids, which affects the degree to which these subgroups have the opportunity to develop important leisure capital through informal learning
Jusqu'a recemment, l'etude des activites de loisir chez les jeunes etait centree principalement sur les adolescents. Dans la presente etude, nous examinons les activites de loisir tant chez les preadolescents que chez les adolescents. A partir de debats sociologiques sur l'importance qu'occupent les loisirs dans l'emploi du temps des jeunes adolescents d'aujourd'hui et sur la precocite de la transition de l'enfance a l'adolescence, un questionnaire a ete elabore a l'intention des jeunes de 10 a 15 ans. Ainsi, 927 jeunes Hollandais ont fait l'objet de cette etude.

Une analyse CATPCA (analyse categorique des composantes principales) a revele que les fillettes de 10 a 12 ans s'adonnaient principalement a la lecture et aux activites creatrices dans leurs moments de loisir, tandis que les garcons du meme age passaient le plus gros de leur temps a jouer dehors. Toutefois, chez les 14 et 15 ans, on a decele un interet marque pour les activites culturelles pour les jeunes, ce qui allait a l'encontre de la theorie voulant que l'adolescence precoce s'installe au detriment de l'enfance. Les questions portant sur les activites organisees ont permis de constater que le marche des loisirs n'etait pas egalement accessible a tous. En effet, les enfants des classes superieures sont avantages, compares a ceux des classes moins bien nanties, ce qui a un effet sur la mesure dans laquelle ces sous-groupes ont la chance d'accumuler un capital-loisir significatif par le biais d'un apprentissage non structure
Hasta hace recientemente el estudio de las actividades de ocio en los jovenes estaba enfocado principalmente sobre los adolescente. En el presente estudio, examinamos las actividades de ocio tanto en los preadolescentes que en los adolescentes. A partir de debates sociologicos sobre la importancia que ocupan las diversiones en el empleo de tiempo de los jovenes adolescentes de hoy en dia y sobre la precocidad de la transicion de la infancia a la adolescencia, un cuestionario dirigido a jovenes de 10 a 15 anos fue elaborado. Asi, 927 jovenes holandeses formaron parte de este estudio. Un analisis CATPCA (analisis de categorias de componentes principales no lineal) revelo que las ninos de 10 a 12 anos se consagran principalmente a la lectura y a las actividades creativas en sus momentos de ocio, mientras que los ninos de la misma edad pasan la mayor parte del tiempo a jugar fuera. Sin embargo, en los jovenes de 14 y 15 anos, se descubrio un interes destacado por las actividades culturales para los jovenes, lo que va en contra de la teoria que preconiza que la adolescencia precoz se instala en detrimento de la ninez. Las preguntas referentes a las actividades organizadas, permitieron de constatar que el mercado de las diversiones no es igualmente accesible a todos. En efecto, los ninos de clases sociales altas son favorecidos, comparados a los de las clases menos pudientes, lo que tiene un efecto en la medida que estos subgroups denen la suerte de acumular un capital-ocio significativo por medio de un aprendizaje no estructurado
SN - 07053436
UR -
https://login.proxy.bib.uottawa.ca/login?url=http://search.ebscohost.com/login.aspx?direct=true&db=s3h&AN=SPHS-821889&site=ehost-live;http://articles.sirc.ca/search.cfm?id=S-821889;http://www.uquebec.ca/puq
L2 - http://articles.sirc.ca/search.cfm?id=S-821889
ER -

TY - JOUR
ID - 5091
T1 - The Boomerang Bunch: a school-based multifamily group approach for students and their families recovering from parental separation and divorce
A1 - Ziffer,J.M.
A1 - Crawford,E.
A1 - Penney-Wietor,J.
Y1 - 2007/06//
N1 - Accession Number: 2009584051. Language: English. Entry Date: 20070720. Revision Date: 20080418. Publication Type: journal article; CEU; exam questions. Journal Subset: Biomedical; Blind Peer Reviewed; Online/Print; Peer Reviewed; USA. Special Interest: Pediatric Care; Psychiatry/Psychology. No. of Refs: 19 ref. NLM UID: 9882671
KW - CINAHL
KW - Counseling -- In Infancy and Childhood
KW - Divorce -- Psychosocial Factors
KW - Family Therapy
KW - Psychotherapy,Group
KW - School Health Services -- In Infancy and Childhood
KW - Support,Psychosocial -- In Infancy and Childhood
KW - Adult
KW - Child
KW - Child,Preschool
KW - Education,Continuing (Credit)
KW - Female
KW - Group Processes
KW - Male
KW - Parents

KW - Theory
RP - NOT IN FILE
SP - 154
JA - Journal for Specialists in Group Work
VL - 32
IS - 2
N2 - Because the community lacked counseling services for whole families experiencing separation and divorce, The Boomerang Bunch was conceived, designed and implemented by six school counselors to identify and therapeutically address changes impacting the participant families as they attempted to "bounce back" from separation and divorce. Building on the strengths and resilience of these families, school counselors met with students and their families at the community public school outside of school hours for 8 weeks. The rationale, group process, techniques, therapeutic activities, and outcome are outlined providing school counselors with the specifics to replicate The Boomerang Bunch in their school setting
SN - 0193-3922
AD - Rollins College
UR - http://search.ebscohost.com/login.aspx?direct=true&db=cin20&AN=2009584051&site=ehost-live
ER -

TY - JOUR
ID - 5092
T1 - Strategies for conducting physical activity research with young children with special needs
A1 - Zittel,L.
A1 - Macfarlane,P.
Y1 - 1995///Fall
KW - SportDiscus
KW - *PEOPLE with disabilities
KW - Methodology
KW - Research
KW - Child
KW - Needs
KW - BEST PRACTICE
KW - SPORTS AND RECREATION ACTIVITIES-General
RP - NOT IN FILE
SP - 111
EP - 125
JA - Brazilian International Journal of Adapted Physical Education Research - BIJAPER
VL - 2
IS - 1
CY - ;
N2 - Conducting physical activity research with young children with special needs presents unique challenges that researchers working with other groups may not encounter. Children with special needs may include children with diagnosed disabilities and/or children experiencing developmental delays. When possible, research should be conducted in the natural play or school environment so the young child's typical behavior is viewed, however, equipment constraints may necessitate that a clinical or laboratory setting be used to collect accurate data. Best practice in early childhood research will require consideration of the following: subject recruitment, researcher experience, data collection environments, assistants in data collection, child interaction patterns and task persistence, and trials to be analyzed. While these topics may relate to collecting research data with children of all abilities, this paper will emphasize specific concerns related to young children with special needs
SN - 10870237
UR -
https://login.proxy.bib.uottawa.ca/login?url=http://search.ebscohost.com/login.aspx?direct=true&db=s3h&AN=SPH415631&site=ehost-live;http://articles.sirc.ca/search.cfm?id=415631
L2 - http://articles.sirc.ca/search.cfm?id=415631
ER -

TY - JOUR
ID - 5094

T1 - Cystic fibrosis and school: the teachers' viewpoint... including commentary by Pachter LM
A1 - Zoritch,B.
A1 - Eiser,C.
A1 - Hiller,E.J.
A1 - Taylor,C.
Y1 - 1996/07//
N1 - Accession Number: 1999058878. Language: English. Entry Date: 19990901. Revision Date: 20091218. Publication Type: journal article; commentary; research; tables/charts. Journal Subset: Biomedical; Europe; Peer Reviewed; UK & Ireland. Grant Information: Cystic Fibrosis Trust and the Cancer Research Campaign, London. No. of Refs: 22 ref. NLM UID: 9508883
KW - CINAHL
KW - Teachers
KW - cystic fibrosis
KW - Health Knowledge -- Evaluation
KW - Faculty Attitudes
KW - Social Adjustment -- In Infancy and Childhood
KW - Academic Performance -- In Infancy and Childhood
KW - Funding Source
KW - Absenteeism -- In Infancy and Childhood
KW - Coping -- In Infancy and Childhood
KW - Health Status -- In Infancy and Childhood
KW - Descriptive Statistics
KW - United Kingdom
KW - adolescence
KW - Questionnaires
KW - Child
KW - Mann-Whitney U Test
KW - Spearman's Rank Correlation Coefficient
KW - Human
RP - NOT IN FILE
SP - 302
EP - 310
JF - Ambulatory Child Health
VL - 1
IS - 4
N2 - Background: School experiences of children with cystic fibrosis (CF) have not been documented in great detail before. The survival and the quality of life of children with CIF have considerably improved in the last decade. Whether CF affects their school performance and what information about CIF is available to their teachers is unknown. Method: A questionnaire was designed in two parts: 1) to ascertain academic progress, practical difficulties, attendance, coping at school and restrictions imposed: 2) to identify teachers' perceived level of information and their actual knowledge of CF. Subjects were 41 children--21 boys and 20 girls with a mean age of 11.5 years. Mann-Whitney tests were used to look at association between variables. Correlation was estimated using Spearman rank correlation test on SPSSPC+ package, Results: The majority of children were making good progress at school, regardless of clinical status, A minority (22%) had sports restrictions, most commonly in outdoor games, The median number of days absent from school in the previous year was 1 (range 0-90). 48% of children had special arrangements organized in the school, usually for medication and physiotherapy. 68% of the teachers felt they were not well informed about CF and this was confirmed by the knowledge questionnaire. Conclusion: Overall, children with CF appear well adjusted to school life. General information on CF should be provided to teachers with more specific and sensitive information given for selected individuals with parental consent. Implication for practice: School health services should have better liaison with the teams caring for children with CF Although well adjusted at school in general, some children with CF are restricted from certain activities and have difficulties with homework and continuous assessment related to their time consuming treatment. In addition, they might benefit from increased use of ambulatory treatment of chest infections, which includes not only IV therapy but also chest physiotherapy, both of which can be given in the school setting to minimize school absence
SN - 1355-5626
UR - http://search.ebscohost.com/login.aspx?direct=true&db=cin20&AN=1999058878&site=ehost-live

ER -

TY - CONF
ID - 5107
T1 - Use of Green Spaces and Behavioral Indicators of Neurodevelopment in Schoolchildren
A1 - Elmira,Amoly
A1 - Payam,Dadvand
A1 - Joan,Forns,
A1 - Monica,Lopez
A1 - Marta,Cirach
A1 - Mar,+ • lvarez Pedrerol
A1 - Mark J,Nieuwenhuijsen
A1 - Jordi,Sunyer,
Y1 - 2013///
KW - Reviewer Nominated
RP - NOT IN FILE
JA - Environment and Health: Bridging North, South, East and West.Basel Switzerland, August 19-23
N2 - Abstract Number : 4185 | ID : O-4-09-04 Use of Green Spaces and Behavioral Indicators of Neurodevelopment in Schoolchildren. Exposure: green spaces Background: Contact to green spaces has been associated with improved mental health and well-being; however, available evidence on the impact of green spaces on child behavioral development is scarce. Aim: To study the association between use of green spaces and behavioral indicators of neurodevelopment in primary school children. Methods: This study was based on a sample of 2407 schoolchildren (6-8 years) from 39 schools across Barcelona (2012) under the framework of BREATHE study. Parents were asked to fill a questionnaire including sociodemographic and household characteristics and childâ€™s frequency of green space use. Annual total time spent in green spaces was abstracted by multiplying the number of days and hours per day that children spent in green spaces during school days, weekends, and holidays. To evaluate the behavioral patterns we utilized Strengths and Difficulties Questionnaire (SDQ) filled by parents. We applied linear regression models to estimate the association between quartiles of the annual total time spent in green spaces and SDQ total and subscale scores, adjusted for parental education, occupation, marital status, and smoking, breastfeeding, childâ€™s sex, ethnicity, school level, and preterm birth. Results: Comparing the fourth quartile with the first quartile of annual total time spent in green spaces, we found a 0.9 (95% CI: 1.5, 0.3), 0.3 (95% CI: 0.5, 0.1), and 0.4 (95% CI: 0.5, 0.2) reduction in SDQ total, emotion, and peer scores respectively. For these associations we observed dose-response relationship across quartiles of annual time spent in green spaces. For hyperactivity, there was a dose-response relationship; however, the association for the fourth quartile was not statistically significant. For prosocial and conduct subscales the results were not conclusive. Conclusion: We found a moderate beneficial impact of contact to green spaces on behavioral development in schoolchildren, though adjustment for social context has not yet been completed
UR - http://ehp.niehs.nih.gov/ehbasel13/o-4-09-04/
ER -

TY - CONF
ID - 5110
T1 - Buffering Effect of Residential Surrounding Greenness and Roadside Trees on the Association between Residential Proximity to Roads and Term Low Birth Weight
A1 - Payam,Dadvand
A1 - Bart,Ostro,
A1 - Margarita,Triguero-Mas
A1 - Xavier,Basaga+Ýa,
A1 - Ant+Ýnia,Valent+ -n,
A1 - David,Mart+ -nez
A1 - Michael,Jerrett
A1 - ; Mark J,Nieuwenhuijsen
Y1 - 2013///
KW - Reviewer Nominated
RP - NOT IN FILE
JA - Environment and Health: Bridging North, South, East and West.Basel Switzerland, August 19-23

N2 - Abstract Number : 3819 | ID : O-4-09-02 Buffering Effect of Residential Surrounding Greenness and Roadside Trees on the Association between Residential Proximity to Roads and Term Low Birth Weight. Exposure: green spaces BACKGROUND: Maternal residential proximity to major roads has been associated to a range of adverse pregnancy outcomes; however, there is no available report on buffering effect of residential surrounding greenness or roadside trees on such an association. AIMS: To investigate the buffering effect of residential surrounding greenness and roadside trees on the association between maternal residential proximity to major roads and term low birth weight (LBW). METHODS: This study was based on 6487 singleton term births occurring in Barcelona (2001-05). We utilized satellite-derived Vegetation Continuous Fields (VCF) and Normalized Difference Vegetation Index (NDVI) to respectively abstract the averages of percent tree cover in a 30m buffer around major roads and NDVI in a 500m buffer around each home. We generated an indicator with three categories: 1) those women residing further than 200m of a major road (reference category), 2) those residing within 200m of greenest major roads (i.e. third tertileof roadside tree percentages) and 3) those residing within 200m from a less green major road (i.e. the first and second tertiles). We developed similar indicator for residential surrounding greenness based on NDVI tertiles. RESULTS: Compared to the reference category, there was a non-significant increase in the adjusted risk of term LBW for those women living within 200m of greenest major roads (OR=1.32 (0.85, 2.06)) or having highest surrounding greenness (OR=1.31 (0.81, 2.11)). This association became stronger and attained statistical significance for those living within 200m of major roads with lower roadside tree coverage (OR=1.52 (1.07, 2.16)) or homes with less surrounding greenness (OR=1.51 (1.07, 2.12)). Stratified analyses according to tertiles of roadside tree percentage or NDVI showed similar patterns. CONCLUSION: Roadside trees and residential surrounding greenness could buffer the association between residential proximity to major roads and term LBW
UR - http://ehp.niehs.nih.gov/ehbasel13/o-4-09-02/
ER -

TY - CONF
ID - 5111
T1 - Association between city parks and risk of hypertension amongst reproductive age women
A1 - Regina,Grazuleviciene,Vytauto Didziojo Universitetas,Lithuania; Asta,Daneleviciute,Vytauto Didziojo Universitetas,Lithuania; Audrius,Dedele,Vytauto Didziojo Universitetas,Lithuania; Jone,Vencloviene
Y1 - 2013///
KW - Reviewer Nominated
RP - NOT IN FILE
JA - Environment and Health: Bridging North, South, East and West.Basel Switzerland, August 19-23
N2 - Abstract Number : 3710 | ID : P-2-17-05 Association between city parks and risk of hypertension amongst reproductive age women. Cardiology Clinic, Lithuanian University of Health Sciences, Lithuania; Tomas, Grazulevicius, Vytauto Didziojo Universitetas, Lithuania; Sandra, Andrusaityte, Vytauto Didziojo Universitetas, Lithuania Exposure: green spaces Background There is some evidence that exposure to green environments is associated with health benefits, including lower blood pressure, although little research has been reported using exposure measures at the personal level. Aims We examined the association between city parks and hypertension amongst reproductive aged women. Methods This study included 3,416 women 20-45 yearâ€™s old Kaunas city residents. We used a geographic information system (GIS) to assess the ambient NO2 exposure and surrounding greenness in buffers of 300 m, 700 m and 1000 m around each women place of residence. Green space exposure effects on doctor-diagnosed hypertension were estimated by multiple logistic regressions with adjustments for potential confounders and NO2 exposure. Results An increase in the distance from city parks across 20-45 years old women was associated with an increase in odds ratio (OR) for high normal blood pressure group and an exposure-response relation was indicated (OR=1.10, 95% CI 0.80â€“1.50 and OR=2,01, 95% CI 1.31â€“3,11, moderate vs. lowest and highest vs. lowest exposure, respectively). The effect was less pronounced amongst women of hypertension group (OR=1.22, 95% CI 0.80â€“1.86, highest vs. lowest exposure). Conclusions The present study findings suggest a beneficial effect of exposure to green spaces on hypertension risk amongst 20-45 years old women. Neighbourhood greenness may have an impact in prevention hypertension and reducing related morbidity
UR - http://ehp.niehs.nih.gov/ehbasel13/p-2-17-05/
ER -

TY - CONF
ID - 5112
T1 - The influence of urban green space on mental health in Auckland, New Zealand

A1 - Simon,Kingham
A1 - Daniel,Nutsford
A1 - Amber,Pearson
Y1 - 2013///
KW - Reviewer Nominated
RP - NOT IN FILE
JA - Environment and Health: Bridging North, South, East and West.Basel Switzerland, August 19-23
N2 - Abstract Number : 3511 | ID : O-4-09-03 The influence of urban green space on mental health in Auckland, New Zealand. Exposure: green spaces BackgroundAs urbanization is escalating globally, urban features which improve or hinder physical and, more recently, mental health are of growing importance. One area of investigation entails the health benefits of urban green spaces. This study examined the relationship between six different measures of green space access and anxiety/mood disorders in Auckland, the largest and fastest- growing city in New Zealand. MethodsAnxiety/mood disorder treatment counts by three age groups were aggregated to 3149 small area units in Auckland. Six measures of green space access were derived using GIS techniques involving total green spaces and useable green spaces. We fitted negative binomial regression models to test the relationship between access to green space and area-level anxiety/mood disorder, adjusted for age and area-level deprivation. ResultsAnxiety/mood disorders were associated with three green space measures. Access to total and useable green space within 300 meters did not exhibit significant associations, however access measures within 3km and distance to nearest useable green space both indicated a protective effect of increased access on decreased anxiety/mood disorders. ConclusionThis study found that proximity to useable green space and the proportion of green space within the larger neighbourhood was positively associated with decreased anxiety/mood disorders in an urban environment. This suggests the benefits of green space on mental health may relate both to the ability to participate in activities in parks near to the home and to visualization of green space in the neighbourhood environment
UR - http://ehp.niehs.nih.gov/ehbasel13/o-4-09-03/
ER -

TY - CONF
ID - 5113
T1 - Contribution of the physical environment to socioeconomic gradients in walking in the Whitehall II study
A1 - Triantafyllos,Pliakas
A1 - Cathryn,Tonne,
A1 - Paul,Wilkinson
Y1 - 2013///
KW - Reviewer Nominated
RP - NOT IN FILE
JA - Environment and Health: Bridging North, South, East and West.Basel Switzerland, August 19-23
N2 - Abstract Number : 3444 | ID : P-2-17-10 Contribution of the physical environment to socioeconomic gradients in walking in the Whitehall II study. Exposure: green spaces Background: Socioeconomic gradients in walking are well documented, but evidence on the extent to which these may be attributed to the physical environment (PE) is limited. Aims: To examine the contribution of objective measures of PE to socioeconomic gradients in minutes walked per week in participants living in London, UK. Methods: Analysis included 3363 participants (50-74 years) from the Whitehall II study (2002-4) who provided data on self-reported walking, socio-demographic characteristics, and whose postcode of residence could be linked to objective measures of PE. Individual-level socioeconomic position (SEP) was measured as most recent employment grade (six groups). Area-level SEP included quintiles of the income score from the Index of Multiple Deprivation. Attributes of PE included crime, land use, air pollution, road safety, road density, green space, access to services, access to public transport, and population density. Data on walking was log transformed prior to regression modelling. Results: In models adjusted for age, sex, ethnicity and labour market status, average minutes walked were 15.1% (95% CI: 4%-27.5%, p[trend]<0.001) higher in the lowest employment grade compared to the highest. Similarly, adjusted average minutes walked were 12% (95% CI: 2.5%-22.1%, p[trend]=0.041) higher in the lowest quintile of area-level SEP compared to the highest. Objective measures of PE attenuated to some extent the gradient for individual-level SEP (13.8%; 95% CI: 2.6%-26.0%, p[trend]<0.001 for lowest vs highest employment grade) and substantially reduced the area-level SEP gradient in walking (4.6%; 95% CI: -5.9%-16.3%, p[trend]=0.691 for lowest vs highest quintile). Of PE variables, only road safety was found to be predictive of walking. Conclusions : The contribution of PE on individual-level SEP gradients in walking appears to have a small effect in this relatively affluent population of older adults living in

greater London. Whether these results are generalizable to the general UK population and outer-London areas requires further research
UR - http://ehp.niehs.nih.gov/ehbasel13/p-2-17-10/
ER -

TY - CONF
ID - 5114
T1 - Green space and spatial analysis of social inequalities in infant mortality in France
A1 - Wahida,Kihal,
A1 - Cindy,Padilla
A1 - Beno+®t,Lallou+ª,
A1 - Marcello,Gelormini
A1 - Denis,Zmirou-Navier
A1 - S+ªverine,Deguen
Y1 - 2013///
KW - Reviewer Nominated
RP - NOT IN FILE
JA - Environment and Health: Bridging North, South, East and West.Basel Switzerland, August 19-23
N2 - Abstract Number : 3103 | ID : P-2-17-17 Green space and spatial analysis of social inequalities in infant mortality in France. Exposure: green spaces Background- Few studies considered environmental benefits to explain social health inequalities. Natural environments that contribute to good health might have an effect on socioeconomic health inequalities. In developed countries, the evidence that green spaces have health benefits for urban population is now strong. Recent studies suggest such positive effects on pregnancy outcomes. Aims- To investigate the relationships between green spaces and the spatial distribution of infant mortality taking into account neighborhood deprivation levels. Methods- The study took place in the Lyon metropolitan area, France (1340 155 in 2009). All infant deaths cases aged <1 yearthat occurred between 2000 and 2009 were geocoded at the census block level (n= 715). Each census block was assigned greenness and socioeconomic deprivation levels. Using a spatialâ€“scan statistic, we examined whether there were significant clusters of high risk of infant mortality according to these neighborhood characteristics. Results- Our results highlight that infant mortality is not randomly spatially distributed, with clusters of high riskin the southwest of the Lyon metropolitan area (53 census blocks; RR:1.70; p<0.003). After adjustment on greenness levels and socioeconomic deprivation, this cluster disappears, suggesting that these factors explain in great part the spatial distribution of infant mortality. No interaction was found between the two factors. Conclusions - We propose 3 hypotheticals not exclusive pathways by which green space may have a beneficial effect on adverse pregnancy outcomes: (i) a psychological pathway, (ii) a physiological disruption process and (iii) an environmental pathway. This work adds some evidence to the link between access to green space and pregnancy outcomes but requires further research for confirmation
UR - http://ehp.niehs.nih.gov/ehbasel13/p-2-17-17/
ER -

Additional File 4: Summary of Study by Study Results

Table S2. Associations between outdoor time and habitual physical activity.

First Author, Year	Study Results
Baranowski, 1993	(+) A model including gender, month and outdoor/indoor location accounted for 75% of the variance in preschoolers' observed physical activity.<br>F (11, 624) = 1.94, $p < 0.03$
Boldemann, 2006	(+) Children who spent more than 45.88 % (the median outdoor time for all children in the study) of their time outdoors achieved more steps/min than children who spent less time outdoors (20.2 *vs.* 19.2 steps/min).<br>*Outdoor stay above vs. below median value*<br>*Boys and girls*:<br>20.2 steps/min; CI: 19.2–21.2 vs.19.2 steps/min; CI: 19.2–20.2<br>*Boys:*<br>21.2 steps/min; CI: 19.8–22.7 *vs.* 20.6 steps/min; CI: 19.1–22.0<br>*Girls*:<br>18.7 steps/min; CI: 17.4–19.9 vs.17.4 steps/min; CI: 16.2–18.7
Burdette, 2005	(+) Parent reported outdoor time checklist and recall of a typical day in the last month were significantly positively correlated with VPM.<br>*Parent reported outdoor play time assessed by:*<br>*Checklist:*<br>$r = 0.33$, $p = 0.001$<br>*Recall:*<br>$r = 0.20$, $p = 0.003$[h]

Table S2. *Cont.*

First Author, Year	Study Results
Cleland, 2008	(+)At T1, each additional h outdoors on weekdays in cooler months associated with an extra 11.6 min/week (1.6%) of MVPA for older (10-12 y/o) boys and an extra 17.0 min/week (2.8%) for older girls. At T2 (3 y follow-up), each additional h outdoors on weekdays in warmer and cooler months was associated with an extra 12.7 min/week (2.5%) and 13.6 min/week (2.6%) of MVPA for older boys. At T1, each additional h outdoors on weekends was associated with an additional 6.1 min/week (2.4%) and 4.7 min/week (2.2%) of MVPA for older boys and older girls in warmer months, and an additional 9.4 min/week (3.7%) and 9.5 min/week (4.4%) of MVPA for older boys and older girls in cooler months. Each additional h outdoors on weekends during warmer months at T1 was prospectively associated with an additional 4.5 min/week (1.7%) of MVPA at T2 for older boys. At T1, each additional h outdoors on weekends during cooler months associated with an additional 9.4 and 9.5 min/week (3.7% and 4.4%) for older boys and girls. Each additional h outdoors on weekends during cooler months at T1 was prospectively associated with an additional 4.8 min/week (1.9%) of MVPA at T2 for older boys. Cross-sectional associations at T2 showed outdoors time was associated with 4.5 additional min/week (1.7%) during warmer months and 4.8 additional min/week (1.9%) during cooler months for older boys. Linear regressions adjusted for maternal education and SEs adjusted for school attended at T1.<br>(≠) On weekends at T1 there were no cross-sectional associations between outdoor time and MVPA during warmer months, for younger children (5–6 y/o) during cooler months, or prospectively; At T2 outdoor time was not related to MVPA during warmer or cooler months for younger boys, younger girls, or older girls. On weekends there were no relationships between outdoor time and MVPA for younger boys or girls; no prospective relationships for older girls or younger children; At T2 there were no cross-sectional associations for older girls or younger children.<br>Weekdays<br>*T1 cooler months, older boys*: b = 11.6; CI: 4.9–18.3<br>*T1 cooler months, older girls:* b = 17.0; CI: 3.3–30.7<br>*T2 warmer months, older boys:* b = 12.7: CI: 0.7–24.7<br>*T2 cooler months, older boys:* b = 13.6; CI: 2.5–24.6<br>Weekends<br>*T1 cooler months, older boys*: b = 9.4; CI: 3.1–15.7<br>*T1 cooler months, older girls*: b = 9.5; CI: 4.3–14.8<br>*T1 warmer months, older boys:* b = 6.1; CI: 1.5–10.7<br>*T1 warmer months, older girls:* b = 4.7; CI: 0.7–8.7<br>*Prospective, warmer months, older boys:* b = 4.5; CI: 0.6–8.4<br>*Prospective, cooler months, older boys:* b = 4.8; CI: 0.8–8.9<br>*T2 warmer months, older boys:* b = 4.5; CI: 0.6–8.4<br>*T2 cooler months, older boys:* b = 4.8; CI: 0.8–8.9<br>P’s < 0.05
Conrad, 2013	(+) There was a positive association between PA status and outdoor time (p < 0.001). The association remained significant (p < 0.001) when age and gender were included as a covariate – values were not reported.<br>Low PA status:<br>$\bar{x}_{\text{outdoor time}}$ = 3:32 hours/day; CI: 3:24–3:40<br>Medium PA status:<br>$\bar{x}_{\text{outdoor time}}$ = 3:43 h/day; CI: 3:36–3:49<br>High PA status:<br>$\bar{x}_{\text{outdoor time}}$ = 3:53 h/day; CI: 3:45–4:01<br>$p < 0.001$

Table S2. *Cont.*

First Author, Year	Study Results
Danner, 1991	(+) There was a significant positive relationship between "accelerometer readings" and parent-reported outdoor time (for each of 4h, mothers indicated if their child spent the majority of the hour indoors = 0 or outdoors = 1). R = 0.43, $p = 0.002$
Dolinksy, 2011	(+) Children who spent more time outdoors had 33.8% more min/day MVPA ($p = 0.001$) than children who spent less time outdoors (adjusted for group and h monitoring). $\bar{x}_{\text{outdoor time}} = 2.0$ (± 1.6) h/day $\bar{x}_{\text{MVPA}} = 14.9$ (9.5) min/day % difference = 33.8; CI: 18.7–50.7
Jones, 2009	(+) Children (total sample, boys, girls) who spent an above average amount of time outside the home had higher $\bar{x}$ min/day spent in bouts of MVPA ($p = 0.002$) Total sample: Above average outdoor time, $\bar{x}_{\text{MVPA}} = 45.1$ (38.8) min/day vs. Below average outdoor time, $\bar{x}_{\text{MVPA}} = 27.7$ (38.3) min/day $p = 0.002$ Boys: Above average outdoor time, $\bar{x}_{\text{MVPA}} = 57.1$ (39.9) min/day vs. below average outdoor time, $\bar{x}_{\text{MVPA}} = 47.6$ (51.1) min/day $p < 0.05$ Girls: above average outdoor time, $\bar{x}_{\text{MVPA}} = 28.8$ (31.3) min/day *vs.* below average outdoor time, $\bar{x}_{\text{MVPA}} = 17.0$ (24.4) min/day $p < 0.05$
Klesges, 1990	(+) A model that include environment (% of time the child spent outdoors vs. indoors) and also sex, relative weight, family risk of obesity, familial interaction, parental encouragements to be active, parental discouragements to be active, and environment x family risk interaction accounted for 25% of the variance in physical activity. Standardized B = 0.34, $p = 0.000$
Ludvigsson, 2007	(+) Playing outdoors was associated with a higher degree of physical exercise. $R^2 = 0.082$, $p < 0.05$
Loucaides, 2006	(+) Hours per day playing outside was significantly correlated with steps/day. Outside time was significant predictor of steps/day in a model that also included gender (entered at step one), self-efficacy (step two), social variables (step three) and hours playing outside and sports club attendance at (step four). Sports club attendance and time playing outside contributed 7% unique variance in steps/day [$F(2, 93) = 6.10$, $p < 0.01$] when entered at step 4. r = 0.40 p <0 .001. Standardized B = 0.261, $p < 0.05$
McKenzie, 1992	(+) Percent time outdoors was significantly and positively correlated with estimated caloric expenditure at home (r = 0.65) and at recess (r = 0.29) (p value not reported). r = 0.65
McKenzie, 2008	(+) Indoor time was negatively correlated with % time children spent in MVPA, and EER (estimated energy expenditure rate), respectively. Media viewing, PA motivated (% time PA was being prompted), other adult present, sibling present, other children present, and number of children living in household, and being indoors significantly predicted % time in MVPA (square root) in a regression model ($R^2 = .599$) . Being indoors independently predicted reduced MVPA % in the model. r = −0.73. r = −0.74, p's < 0.01. B = −0.341, SE = 0.040, t = −8.47, $p < 0.01$

Table S2. *Cont.*

First Author, Year	Study Results
Ortlieb, 2013	(+) During summer parents reported their children engaged in x̅ = 12.7 h (± 11.0) LPA; 8.2 h (± 6.1 MPA); 5.5 h (± 4.5) VPA per week. During winter parents reported their children engaged in 9.5 h (± 8.9) LPA, 5.2 h (± 4.3) MPA, and 5.0 h (± 3.3) VPA per week. An average amount of outdoor time in the summer (3–5 h/week) is associated with accumulating 5-9 h/week and > 9 h/week of MPA. A high amount of outdoor time in the summer (>5 h/week) is associated with accumulating 5–9 h/week and >9 h/week of MPA. An average and high amount of outdoor time in the summer are both associated with accumulating 4–6 h/week and >6 h/week of VPA, respectively. An average (2–3 h/week) and high (>3 h/week) amount of outdoor time in winter are associated with accumulating 3–6 h/week and >6 h/week of MPA, respectively. An average and high amount of outdoor time in winter are associated with accumulating 3–4 h/week and >4 h/week of VPA, respectively.<br>*MPA in summer*<br>*Average outdoor time*<br>*5-9 h/week of MPA,* OR = 2.38; CI: 1.18–3.14<br>*>9 h/week of MPA,* OR = 5.75; CI: 3.89–8.49<br>*High outdoor time*<br>*5-9 h/week of MPA,* OR = 3.27; CI: 2.26–4.74<br>*>9 h/week of MPA, OR* = 12.41; CI: 7.92–19.43<br>*VPA in summer*<br>*Average outdoor time*<br>*4-6 h/week of VPA,* OR = 1.72; CI: 1.29–2.28<br>*>6 h/week of VPA,* OR = 3.71; CI: 2.50–5.52<br>*High outdoor time*<br>*4-6 h/week of VPA,* OR = 2.21; CI: 1.55–3.17<br>*Winter*<br>*>6 h/week of VPA,* OR = 7.55; CI: 4.81–11.84<br>*Average outdoor time*<br>*3-6 h/week of MPA,* OR = 1.70; CI: 1.39–2.06<br>*>6 h/week of MPA,* OR = 4.33; CI: 3.35–5.59<br>*High outdoor time*<br>*3-6 h/week of MPA,* OR = 2.50; CI: 1.61–3.90<br>*>6 h/week of MPA,* OR = 9.39; CI: 5.92–14.90<br>*Winter*<br>*Average outdoor time*<br>*3-4 h/week of VPA,* OR = 1.31; CI: 1.06–1.60<br>*>4 h/week of VPA,* OR = 2.06; CI: 1.64–2.58<br>*3-4 h/week of VPA,* OR = 1.96; CI: 1.29–2.98<br>*>4 h/week of VPA,* OR = 4.35; CI: 2.87–6.60
Sallis, 1993	(+) Percentage of observed intervals spent outdoors (15.5% (±15.5) was correlated with observed PA (0.060 (±0.008) kcal/kg/min) kcal/kg/min).<br>r = 0.74, p < 0.001
Wickel, 2013	(+) The percentage of MVPA was low inside the home (3.3%), inside another home (8.4%) and inside at school (10%), compared with outside a home (70%), within the neighborhood (84.1%), and outside during an after-school program (72.9%). Time outside at home (3-6PM) increased the odds of exceeding M MVPA levels 2.56 times. Time outside at another home increased the odds of exceeding M MVPA levels 2.42 times.<br>β = 0.94, p < 0.001, OR = 2.56; CI: 2.06–3.17<br>β = 0.88, p < 0.001, OR = 2.42; CI: 1.87–3.13

Notes: (+), positive relationship; (≠), no relationship; x̅, mean; CI, 95% confidence interval; h, hour; h/week, hours per week; GPS, Global Positioning System; M, Median; MPA, moderate physical activity; MVPA, moderate-to-vigorous physical activity; OR, odds ratio; PA, physical activity; SE, standard error; T1, time 1; T2, time 2; VPA, vigorous physical activity; VPM, vector per minute.

Table S3. Associations between outdoor time and acute physical activity.

First Author, Year	Study Results
Cooper, 2010	(+) Children recorded $\bar{x}$ = 41.7(±46.1) min outdoor time after school each day. PA was more than 2.5 times higher outdoors than indoors. This difference was consistent in each h of measurement from 3:30pm until 8:30 pm. Each min outdoors was associated with an increase of 1018.1 (CI: 875.4-1160.7) CPM in the unadjusted model, and 1000.6 (CI: 850.0–1151.2) in a model adjusted for gender, age, BMI SD scores, IMD, daylight, pubertal status.<br>$\bar{x}_{outdoors}$ = 1345.8 ± 907.3 CPM vs. $\bar{x}_{indoors}$ = 508.9 ±282.9 CPM<br>F = 783.2, $p < 0.001$<br>β = 1018.1 (unadjusted model)<br>CI: 875.4–1160.7<br>t = 14.8, $p < 0.001$, $R^2 = 0.137$<br>B = 1000.6 (adjusted model)<br>CI: 850.0–1151.2<br>t = 13.78, $p < 0.001$, $R^2 = 0.144$
Dunton, 2011	(+) Total steps were significantly greater when children were outdoors at home (PMM = 448.58 steps) and outdoors away from home (PMM = 462.79 steps) than in any other location. The likelihood of attaining at least 5 min of MVPA during the 30 min before the EMA survey was significantly greater for entries occurring outdoors (23%) compared with at home indoors (5%), at someone else's house (9%), or in other locations (8%), all p's < 0.05.<br>*Step counts*<br>*Outdoors not at home:* PMM (SE) = 462.79 steps (42.22)<br>*Outdoors in yard:* PMM (SE) = 448.58 steps (80.83)<br>*At other house:* PMM (SE) = 274.88 steps (49.29)<br>*indoors at home:* PMM (SE) = 210.61 steps (12.50)<br>*Other locations:* PMM (SE) = 354.16 steps (25.94)<br>P's < 0.05<br>*5 minute MVPA bouts*<br>*Outdoors*: PMM (SE) = 0.23 (0.04)<br>*Indoors at home*: PMM (SE) = 0.05 (0.01)<br>*Someone else's house*: PMM (SE) = 0.09 (0.04)<br>*Other locations*: PMM (SE) = 0.08 (0.02)<br>P's < 0.05
Klinker, 2014	(+) Boys had 7.7% more of their daily MVPA outdoors than girls (adjusted). Boys had 24.3 more minutes of MVPA outdoors than girls did (adjusted). During the course of the whole day, a 1 hour increase in outdoor time was associated with an increase of $\bar{x}$ = 9.8 min MVPA (adjusted model controlling for age, BMI, number of valid days, and time in domain. School was included as fixed effect to account for clustering of students within school).<br>Daily median (IQR) proportion of MVPA spent outdoors<br>Children = 70.0 (63.5-76.5)<br>Boys = 73.8 (68.6–78.4)<br>Girls = 65.3 (55.9–71.4)<br>Adjusted difference = 7.7%, p = 0.000<br>Daily outdoor median (IQR) min of MVPA<br>Children = 50.1 (36.5–65.5)<br>Boys = 61.8 (41.0–76.0)<br>Girls = 42.3 (25.3–52.3)<br>Adjusted difference = 24.3 min, p = 0.000<br>Coefficient = 9.8, p < 0.001; CI: 6.9–12.8

Table S3. *Cont.*

First Author, Year	Study Results
Raustorp, 2012	(+) CPM of total activity were significantly higher for outdoor than indoor activity (1098 vs. 493 CPM, $P < 0.001$) in both boys (1134 vs. 530 CPM, $P < 0.001$) and girls (1064 vs. 459 CPM, $P < 0.001$). A greater percentage of MVPA and light PA occurred outdoors than indoors for the total sample, for boys, and for girls.<br>*Overall total CPM*<br>*Total sample*<br>Outdoor = 1098 CPM vs. Indoor = 493 CPM; $p < 0.001$<br>*Boys*<br>Outdoor =1134 CPM vs. Indoor = 530 CPM; $p < 0.001$<br>*Girls*<br>Outdoor 1064 CPM vs. Indoor = 459 CPM; $p < 0.001$<br>~25% of outdoor (65 min), 10% of indoor (75 min) time spent non-sedentary<br>Overall MVPA<br>*Total sample*<br>Outdoor % = 7.3 ± 5.7 vs. Indoor % = 2.3 ± 1.7; $p < 0.001$<br>Boys<br>7.5% *vs.* 2.0%; $p < 0.001$<br>Girls<br>7.0% *vs.* 2.7%; $p < 0.001$<br>Light PA total sample<br>Outdoor % = 17.6 ± 6.1 vs. Indoor % = 8.5 ± 2.6
Smith, 2009	(+) A one-way within-subjects ANOVA indicated a significant effect of inside vs. outside classes on average steps per class. Average steps/class during classes held outside ($\bar{x}$ = 1,874, *SD* = 220) were significantly greater than average steps/class during classes held inside ($\bar{x}$ =1,393, *SD* = 197).<br>Pillai's Trace = 0.96, $F(2, 136) = 1718.41$, $p < 0.001$, partial $\eta^2 = 0.96$.<br>$t(135) = 58.76$, $p < 0.001$, $\eta^2 = 0.96$
Skala, 2012	(+) Children spent 5.36% more time standing, 4.42% more time walking, and 5.29% more time in MVPA in outdoor PE classes compared with indoor classes. MVPA was significantly associated with outdoor class vs. indoor class location.<br>*PE class % time standing*<br>Outdoor = 41.40 ±13.98; Indoor = 36.04 ± 12.06; $\bar{x}$ difference = -5.36, $p < 0.05$<br>*PE class % time walking*<br>outdoor = 26.60 ± 14.23; indoor = 22.18 ± 9.73; $\bar{x}$ difference = -4.42, $p < 0.05$<br>*PE class % time MVPA*<br>outdoor = 41.42 ± 16.75; indoor = 36.13 ± 11.93; $\bar{x}$ difference = −5.29, $p = 0.037$<br>Unstandardized β = 5.794, SE = 2.817, p = 0.041, CI: 0.233–11.354

Table S3. *Cont.*

First Author, Year	Study Results
Vanderloo, 2013	(+) Preschoolers had higher average min/h MVPA outdoors than indoors. Preschools had higher average total min/h outdoors than indoors. Boys participated in more min/h MVPA outdoors vs. indoors. Both boys and girls displayed a statistically significant increase in TPA during outdoor playtime compared with indoor play time.<br>(≠) There was not a significant difference for the % of class time that was very active outdoors ($\bar{x}$ = 14.83, SD = 8.04) vs. indoors ($\bar{x}$ = 13.96, SD =7.46). There was not a significant $\bar{x}$ difference in minutes of PA for standing, walking, being very active, or engaging in MVPA during an indoor vs. outdoor lesson.<br>*Average min/h of MVPA*<br>Indoor $\bar{x}$ = 0.54 (0.59)<br>Outdoor $\bar{x}$ = 5.03(4.92)<br>$z = -4.41$, $p < 0.017$<br>*Average min/h of TPA*<br>Indoor $\bar{x}$ = 14.42 (6.78)<br>Outdoor $\bar{x}$ = 31.68 (10.83)<br>$z = -4.70$, $p <.017$<br>*Boys MVPA*<br>indoor $\bar{x}$ = 0.63 (0.63)<br>outdoor $\bar{x}$ = 6.42 (4.97)<br>$z = 3.62$, $p < .0083$<br>*Boys' TPA*<br>indoor $\bar{x}$ = 16.91 (7.22)<br>outdoor $\bar{x}$ = 35.71 (7.65)<br>boys, $z = 3.57$, $p < 0.0083$<br>*Girls TPA*<br>indoor $\bar{x}$ = 11.40 (4.92)<br>outdoor $\bar{x}$ = 26.79 (12.32)<br>$z = 3.12$, $p < 0.0083$[l]
Wheeler, 2010	(+) Compared with indoors, boys and girls had a greater odds of an epoch of PA being MVPA outdoors in greenspace, outdoors not in greenspace, and outdoors out of the neighbourhood area. Relative odds of an epoch exceeding MVPA threshold remained significantly higher outdoors than indoors after adjusting for confounders (BMI, age, pubertal stage, deprivation index, season, minutes of daylight).<br>(≠) Preschool girls' MVPA not significantly different indoors from outdoors.<br>Boys<br>*in green space:* OR = 6.02; CI: 5.32–6.81, p < 0.01<br>*not in green space:* OR = 4.28; CI: 3.87–4.73, p < 0.01<br>*Outdoors out of area:* OR = 1.84; CI: 1.54–2.19, p < 0.01<br>Girls<br>*in green space*: OR = 5.22; CI: 4.61–5.91, $p < 0.01$<br>*Not in green space:* OR = 4.76; CI: 4.31–5.26, $p < 0.01$<br>*Outdoors out of area:* OR = 1.92; CI: 1.63–2.27, $p < 0.01$<br>Boys<br>*Green space:* OR = 5.77; CI: 5.08–6.54, $p < 0.01$<br>*Not green space:* OR = 4.22; CI: 3.81–4.66, $p < 0.01$<br>*Outdoors out of area:* OR = 1.81; CI: 1.52–2.15, $p < 0.01$<br>Girls<br>*Green space*: OR = 5.12; CI: 4.52–5.79, $p < 0.01$<br>*Not green space*: OR = 4.75; CI: 4.29–5.24 $p < 0.01$<br>*Outdoors out of area:* OR = 1.93; CI: 1.63–2.29, $p < 0.01$[m]

Notes: (+), positive relationship; (≠), no relationship; ANOVA, analysis of variance; BMI, body mass index; CI, 95% confidence interval; CPM, counts per minute; EMA, electronic momentary assessment; h, hour; IMD, index of multiple deprivation; IQR, inter quartile range; $\bar{x}$, mean; min, minute; MVPA, moderate-to-vigorous physical activity; OR, odds ratio; PA, physical activity; PE, physical education; PMM, predicted marginal mean; SD, standard deviation; SE, standard error; TPA, total physical activity.

Table S4. Associations between outdoor time and habitual sedentary behaviour.

First Author, Year	Study Results
Dolinksky, 2011	(+) Children's mean sedentary time was 6.1 (1.3) h/day. Children who spent 1 h ≤ outdoors had 4.4% less sedentary time than children who spent less than 1 h/day outdoors. (Adjusted for group and h of monitoring).
	Boys and girls
	% difference = −4.4; CI: −7.3–−1.4, p = 0.005

Notes: (+), positive relationship; CI, confidence interval; h, hour.

Table S5. Associations between outdoor time and acute sedentary behaviour.

First Author, Year	Study Results
Raustorp, 2012	(+) Significantly more sedentary behavior was accumulated indoors than outdoors for the total sample, for boys and for girls.
	Total sample
	89.1% *vs.* 75.2%, p < 0.001
	boys
	89.2% *vs.* 73.8%, p < 0.001
	girls
	89.1% *vs.* 76.8%, p < 0.001
Skala, 2012	(+) Children spent significantly greater portion of indoor PE classes being sedentary than outdoor PE classes.<br>16.76 ± 14.83 *vs.* 26.81 ± 13.52, $\bar{x}$ difference = 10.05, p < 0.001
Vanderloo, 2013	(+) Preschoolers engaged in fewer min/h of sedentary activity outdoors vs. indoors. Boys had significantly higher sedentary levels indoors vs. outdoors.
	Boys and girls
	Outdoors, $\bar{x}$ = 25.33 ±10.78 min/h *vs. indoors,* $\bar{x}$ = 50.21 ± 19.98 min/h
	z = –4.25, p < 0.017
	Boys
	Outdoors $\bar{x}$ = 23.11 ± 8.39 min/h *vs. Indoors* $\bar{x}$ = 53.14 ± 22.84
	z = 3.57, p < 0.0083

Notes: (+), positive relationship; (≠), no relationship; CI, 95% confidence interval; $\bar{x}$, mean; min/h, minutes per hour; PE, physical education; SE, standard error.

Table S6. Associations between outdoor time and motor skills.

First Author, Year	Study Results
Saakslahti, 1999	(+) More time playing outdoors was correlated with faster completion of 10 m run.
	(≠) Total time playing outdoors was not related to the following motor skills: walking, standing broad jump, agility, throwing at a target from 2 or 3 m, throwing-catching combination, clapping, galloping, somersault, kicking a ball.
	10 m run time
	Outdoor playing time
	r = −0.29, p = 0.42

Notes: (+), positive relationship; (≠), no relationship; m, metre.

Table S7. Associations between outdoor time and cardiovascular fitness.

First Author, Year	Study Results
Gopinath, 2011	(+) Children in the highest tertile of outdoor activity had a significantly lower diastolic and systolic BP than children in the lowest tertile of outdoor activity. Indoor activities were associated with an increased likelihood of having an elevated BP.
	highest vs. lowest tertile of outdoor PA (≥0.57 hours/day vs. ≤0.14 hours/day) diastolic BP $\bar{x}$ = 61.5 (59.5–63.5) vs. $\bar{x}$ = 63.0 (60.8–65.2); *p* = 0.01
	highest vs. lowest tertile of indoor PA (≥0.18 h/day vs. ≤0.06 h/day) systolic BP $\bar{x}$ = 98.9 (97.7–100.1) vs. $\bar{x}$ = 101.2 (99.6–102.8); *p* = 0.03
	indoor physical activity and elevated BP OR = 0.50; CI: 0.27–0.92; *p* = 0.03
Saakslahti, 1999	(-)Systolic BP was positively correlated with playing outdoors.
	(≠) Systolic BP was not correlated with playing indoors. Diastolic BP was not correlated with playing indoors or outdoors.

Notes: (+), positive relationship; (≠), no relationship; (−), negative relationship; BP, blood pressure, CI, Confidence Interval; h/day, hours per day; $\bar{x}$, mean; OR, odds ratio; PA, physical activity.

© 2015 by the authors; licensee MDPI, Basel, Switzerland. This article is an open access article distributed under the terms and conditions of the Creative Commons Attribution license (http://creativecommons.org/licenses/by/4.0/).
